# Proteolytic Potential of the MSC Exosome Proteome: Implications for an Exosome-Mediated Delivery of Therapeutic Proteasome

**DOI:** 10.1155/2012/971907

**Published:** 2012-07-18

**Authors:** Ruenn Chai Lai, Soon Sim Tan, Bao Ju Teh, Siu Kwan Sze, Fatih Arslan, Dominique P. de Kleijn, Andre Choo, Sai Kiang Lim

**Affiliations:** ^1^Institute of Medical Biology, A∗STAR, 8A Biomedical Grove, No. 06-06 Immunos, Singapore 138648; ^2^School of Biological Sciences, Nanyang Technological University, 60 Nanyang Drive, Singapore 637551; ^3^Laboratory of Experimental Cardiology, University Medical Center Utrecht, Heidelberglaan 100, 3584 CX Utrecht, The Netherlands; ^4^The Netherlands Heart Institute, Catharijnesingel 52, 3511 GC Utrecht, The Netherlands; ^5^Cardiovascular Research Institute, and YLL School of Medicine, NUS, Singapore 119074; ^6^Department of Surgery, YLL School of Medicine, NUS, Singapore 119074; ^7^Bioprocessing Technology Institute, A∗STAR, Singapore 138671

## Abstract

Mesenchymal stem cells (MSCs) are used in many of the current stem cell-based clinical trials and their therapeutic efficacy has increasingly been attributed to secretion of paracrine factors. We have previously demonstrated that a therapeutic constituent of this secretion is exosome, a secreted bilipid membrane vesicle of *~*50–100 nm with a complex cargo that is readily internalized by H9C2 cardiomyocytes. It reduces infarct size in a mouse model of myocardial ischemia/reperfusion (MI/R) injury. We postulate that this therapeutic efficacy is derived from the synergy of a select permutation of individual exosome components. To identify protein candidates in this permutation, the proteome was profiled and here we identified 20S proteasome as a protein candidate. Mass spectrometry analysis detected all seven **α** and seven **β** chains of the 20S proteasome, and also the three beta subunits of “immunoproteasome” with a very high confidence level. We demonstrated that a functional proteasome copurified with MSC exosomes with a density of 1.10–1.18 g/mL, and its presence correlated with a modest but significant reduction in oligomerized protein in a mouse model of myocardial infarction. Circulating proteasomes in human blood also copurified with exosomes. Therefore, 20S proteasome is a candidate exosome protein that could synergize with other constituents to ameliorate tissue damage.

## 1. Introduction

Mesenchymal stem cells (MSCs) are multipotent fibroblast-like cells that reside in many adult tissues such as bone marrow adipose tissue [1, 2], liver [3], muscle connective tissue [4], amniotic fluid [5], placenta [6, 7], umbilical cord blood [1], and dental pulp [8, 9]. Although their differentiation potentials are primarily osteogenesis, chondrogenesis, and adipogenesis, MSCs have been reported to have the potential to differentiate into an amazing array of cell types that include nearly every major cell types in the adult body [10, 11]. MSCs are currently the most evaluated experimental stem cells with more than 100 clinical trials in 2010 to test their efficacy in treating a myriad of diseases such as cardiovascular diseases (e.g., acute myocardial infarction, endstage ischemic heart disease, or prevention of vascular restenosis), osteogenesis imperfecta (OI) or brittle bone disease, amyotrophic lateral sclerosis (ALS), lysosomal storage diseases (e.g., Hurler syndrome), steroid refractory graft versus host disease (GVHD), periodontitis and bone fractures [12].

The use of MSCs as therapeutics was initially predicated on the hypothesis that transplanted MSCs home and engraft in injured tissues, and then differentiate into cells to replace damaged cells. However, it has been estimated that <1% of transplanted cells actually reached the target tissue with most of the cells being trapped in the liver, spleen, and lung [13], and reported evidence for differentiation of transplanted MSCs at the site of injury often cannot eliminate the possibility of cell fusion [14–16]. It has also been increasingly observed that the therapeutic efficacy of MSC therapy is not dependent on the engraftment of MSC at the site of injury or differentiation capability of the transplanted MSC [17–20], essentially eliminating the need for MSCs to be in the vicinity of their target tissue or to differentiate to exert a therapeutic effect. To reconcile this discrepancy between the therapeutic efficacy of MSC and the lack of MSC engraftment or differentiation at the site of injury, it was proposed that MSCs exert their therapeutic effects through secreted trophic mediators. The general acceptance of this proposal is reflected in the MSC clinical trials of 2010 in which 65 of the 101 clinical trials were rationalized on the trophic secretion of MSCs while only 36 were based on the differentiation potential of MSCs [21]. This paradigm shift in the therapeutic mechanism of MSC from one based on cell engraftment, differentiation and replacement to one based on secretion and paracrine signaling could potentially engender the development of biologic instead of cell-based therapeutics.

In 2008, our group demonstrated that intravenous administration of a single bolus of culture medium conditioned by human embryonic stem cell-derived MSCs (hESC-MSCs) reduced relative infarct size in a pig and mouse model of ischemia/reperfusion injury [22]. By molecular weight fractionation of this conditioned medium (CM), we demonstrated that the active component had a presumptive size of 50–200 nm [22]. Using size exclusion high performance liquid chromatography (HPLC), we purified a population of homogenously sized particles that have the biophysical parameters of exosomes, namely, a hydrodynamic radius of 55–65 nm and a flotation density in sucrose of 1.10–1.18 g/mL. We subsequently demonstrated that this exosome population alone could reduce infarct size by ~40% in a mouse model of myocardial ischemia/reperfusion injury and therefore was the therapeutic agent in the secretion of mesenchymal stem cells [23]. Therapeutic exosomes are also found to be secreted by primary MSC cultures [24] and myc-immortalized hESC-MSCs [25]. The exosomes have exosome-associated proteins such as the tetraspanin proteins, CD9 and CD81, Alix, Tsg101, and RNA that consists primarily of short RNAs of less than 300 nt. Some of these RNAs are microRNAs that are predominantly premicroRNAs [26].

As cells for example, H9C2 cardiomyocytes [26] readily internalized MSC exosomes, possibly by endocytosis, the exosome cargo of protein and RNA could be delivered across plasma membranes into cells. This diverse exosome cargo could, in principle provide a molecular basis for the therapeutic efficacy of MSC secretion in treating a diverse range of diseases (e.g., acute myocardial infarction, endstage ischemic heart disease, or prevention of vascular restenosis), osteogenesis imperfecta (OI) or brittle bone disease, amyotrophic lateral sclerosis (ALS), lysosomal storage diseases (e.g., Hurler syndrome), steroid refractory graft versus host disease (GVHD), periodontitis and bone fractures [12]. The cargo complexity of exosome could also provide a rationale for the efficacy of exosomes in treating complex tissue injuries such as myocardial ischemia/reperfusion injury that involves multiple tissues and targets.

We believe that the individual components in exosome cargo are not equally or sufficiently efficacious in ameliorating tissue injury. We hypothesize that the therapeutic efficacy of MSC exosome against a specific injury is derived from the synergy of a select permutation of individual exosome components. To identify optimal permutation of biochemical activities for effective amelioration of myocardial ischemia/reperfusion injury and other injury, we are now systematically analyzing each of the candidate biochemical activities in MSC exosomes to first assess each activity and its potential to ameliorate injury.

In this paper, we focused on the proteome of exosomes to identify candidate proteins or protein complexes that could drive their efficacy against diverse disease targets, the proteome of these purified exosomes was profiled here by mass spectrometry and antibody array, and found to contain 857 unique gene products (http://www.exocarta.org/). These proteins were distributed over a wide array of biochemical and cellular processes such as communication, structure and mechanics, inflammation, exosome biogenesis, tissue repair and regeneration and metabolism (Lai et al., submitted) consistent with the reported proteome complexity of MSC exosomes.

A predominant feature of MSC exosome proteome was the presence of all seven *α* and seven *β* chains of the 20S proteasome, and the three beta subunits of “immunoproteasome” which were identified with very high confidence level by mass spectrometry. These observations were indicative of fully assembled and possibly functional 20S proteasome and immunoproteasome which constitute the catalytic core of the 26S proteasome. The presence of 20S proteasome in exosomes also provides a mechanism for cellular extrusion of the relatively large intact 20S proteasomes as extracellular, circulating proteasome [27–31]. Circulating proteasomes are functionally active and could degrade small peptides. They have been shown to correlate with disease state or progression demonstrating that extracellular proteasomes have important physiological and pathological functions [27–31]. It has been postulated that these extracellular proteasomes are important in degrading soluble peptides in extracellular fluids and modulating potentially pathogenic protein aggregation such as Alzheimer's amyloid plaques [32]. A key to understanding the functions of these extracellular proteasomes will be to determine how and when cells extrude proteasome. As the level of circulating proteasome does not correlate with lactate dehydrogenase it is unlikely that extracellular proteasomes are products of cell lysis [33].

In this paper, we demonstrated that MSC exosomes contained functional 20S proteasomes and its presence correlated with a modest but significant reduction in oligomerized protein in a mouse model of myocardial infarction. Therefore, 20S proteasome is a candidate exosome protein that could synergize with other constituents to ameliorate tissue damage.

## 2. Materials and Methods

### 2.1. Preparation of Exosomes

Exosomes were purified from culture medium conditioned by huES9.E1, human ESC-derived mesenchymal stem cells [34] by HPLC as previously described [24, 35]. Briefly, CM collected from MSCs culture was concentrated 50x by tangential flow filtration (TFF) using a membrane with a 100 kDa MWCO (Sartorius, Goettingen, Germany). CM was fractionated by high performance liquid chromatography (HPLC) (TSK Guard column SWXL, 6 × 40 mm and TSK gel G4000 SWXL, 7.8 × 300 mm, Tosoh Corp., Tokyo, Japan). Exosomes were collected from the first peak of the elution and concentrated using 100 kDa MWCO filter (Sartorius). Exosomes were filtered with a 0.22 *μ*m filter and stored in −20°C freezer until use.

### 2.2. LC MS/MS

Three independent preparations (biological replicates) of HPLC-purified exosomes were analyzed by LC-MS/MS over nine months using three different methods of tryptic digestion. In each preparation, about 2 mL proteins of dialyzed exosomes were used. In the first preparation, the sample was in-solution trypsin digested, that is, reduced, alkylated and tryptic digested as described [36]. After desalting using Sep-Pak C-18 SPE cartridge (Waters, Milford, MA, USA), the tryptic digest was directly analyzed with LC-MS/MS twice (two injections) without further fractionation. In the second preparation, the sample was separated by SDS-PAGE and the gel lane was cut into 8 slices for in gel digestion. Tryptic peptides from each gel slice were analyzed by LC-MS/MS. In the third preparation, the sample was tryptic digested and desalted. To reduce sample complexity, offline peptide fractionation was carried out with a HPLC system (Shimadzu, Japan) through a Polysulfoethyl SCX column (200 mm × 4.6 mm) (PolyLC, USA). Mobile phase A (5 mM KH4PO4 + 30% acetonitrile) and mobile phase B (5 mM KH4PO4 + 30% acetonitrile + 350 mM KCl) at 1 mL/min. Eight fractions were collected, desalted, and dried with a vacuum centrifuge. All three independent replicates were analyzed by a LC-MS/MS system including a Shimadzu micro HPLC system coupled online to a LTQ-FT Ultra linear ion trap mass spectrometer (Thermo Electron, Bremem, Germany) fitted with a nanospray source. Injected peptides were trapped and desalted in a Zorvax 300SB-C18 enrichment column (5 mm × 03 mm, Agilent Technologies, Germany) and eluted into a nano-bored C18 packed column (75 *μ*m × 100 Å, Michrom Bioresources, Auburn, CA USA). A 90 minute gradient at a constant flow rate of 20 *μ*L/min with a splitter to an effective flow rate of 300 nl/min was used to elute the peptides into the mass spectrometer. The LTQ was operated in a data-dependent mode by performing MS/MS scans for 8 of the most intense peaks from each MS scan in the FTMS. For each experiment, all MS/MS (dta) spectra of each replicate were combined into a single mascot generic file by a home-written program. Protein identification was achieved by searching the combined data against the IPI human protein database (version 3.34; 69,164 sequences, 29,064,825 residues) via an in-house Mascot server (Version 2.2.04, Matrix Science, UK). The search parameters were a maximum of 2 missed cleavages using trypsin; fixed modification was carbamidomethylation of cysteine and variable modification was oxidation of methionine. The mass tolerances were set to 10 ppm and 0.8 Da for peptide precursor and fragment ions, respectively. Protein identification was accepted as true positive if two different peptides were found to have scores greater than the homology scores.

### 2.3. Antibody Array

500 *μ*L of non-conditioned media and exosomes were assayed for the presence of cytokines andother proteins using RayBio Biotin Label-based Human Antibody Array I according to manufacturer's instructions (RayBio, Norcross, GA). The cytokines and other proteins were considered to be present in the exosomes if the signal intensity was 2 fold higher (*P* < 0.05) than that in non-conditioned medium.

### 2.4. Western Blot Hybridization

3 *μ*g of conditioned medium or exosomes were separated on 4–12% SDS-polyacrylamide gels and electroblotted onto a nitrocellulose membrane. The membrane was transferred to the membrane holder of SNAP i.d. system (Millipore, Billerica, MA), blocked and incubated with 1 : 200 diluted mouse anti-20S proteasome *α*1–7. The blot was then incubated with a 1 : 1250 diluted horseradish peroxidase-coupled goat anti-mouse IgG. All antibodies were obtained from Santa Cruz Biotechnology, Santa Cruz, CA. The blot was then incubated with HRP-enhanced chemiluminescent substrate (Thermo Fisher Scientific Inc., Waltham, MA, USA) and then exposed to X-ray film.

### 2.5. 20S Proteasome Enzymatic Assay

The proteasome activity was measured using a 20S proteasome activity assay kit (Millipore) based on detection of the fluorophore 7-Amino-4 methylcoumarin (AMC) after cleavage from the labeled substrate LLVY-AMC by 20S proteasome in the presence or absence of lactacystin, a specific 20S proteasome inhibitor. Briefly, 4 *μ*g of exosome was incubated with a reaction buffer containing LLVY-AMC in the presence or absence of 25 *μ*M lactacystin. The samples and AMC standards were incubated at 37°C and fluorescence intensity at Ex/Em = 380/460 nm was monitored for 2 hours.

### 2.6. Animals and Experiments

Male C57Bl6/J (10–12 wks, 25–30 g) mice were obtained from Jackson Laboratory (Bar Harbor, USA). Mice received standard diet and water ad libitum. Myocardial infarction was induced between 8 am and 1 pm by left coronary artery ligation, just below the left atrial appendage. All animal experiments are performed in accordance with the national guidelines on animal care and with prior approval by the Animal Experimentation Committee of Utrecht University, The Netherlands.

### 2.7. Myocardial Infarction *In Vivo*

Mice were anesthetized with a mixture of Fentanyl (Jansen-Cilag) 0.05 mg/kg, Dormicum (Roche) 5 mg/kg and medetomidine 0.5 mg/kg through an intraperitoneal injection. Core body temperature was maintained around 37°C during surgery by continuous monitoring with a rectal thermometer connected to an automatic heating blanket. Mice were intubated and ventilated (Harvard Apparatus Inc.) with 100% oxygen. The left coronary artery (LCA) was ligated for 30 minutes using an 8-0 Ethilon (Ethicon) with a section of polyethylene-10 tubing placed over the LCA. Ischemia was confirmed by bleaching of the myocardium and ventricular tachyarrhythmia. Five minutes before reperfusion, mice were intravenously infused with 200 *μ*L saline-diluted exosome containing 0.4 *μ*g protein via the tail vein. Control animals were infused with 200 *μ*L saline. Reperfusion was initiated by releasing the ligature and removing the polyethylene-10 tubing. Reperfusion of the endangered myocardium was characterized by typical hyperemia in the first few minutes. The chest wall was closed and the animals received subcutaneously atipamezole (Antisedan, Pfizer) 2.5 mg/kg, flumazenil (Anexate, Roche) 0.5 mg/kg and buprenorphine (Temgesic, Schering-Plough) 0.1 mg/kg. At 15 min, 1 hour, 24 hours, or 3 days after reperfusion (*n* = 4/group/time point), animals were sacrificed and the area at risk was excised for protein extraction. LCA ligation in mice results in anterior wall ischemia, while the septal wall is perfused by a septal coronary artery that originates separately from the sinus valsalva. The entire anterior wall of the left ventricle below the level of ligation was excised for area at risk protein extraction.

### 2.8. Antioligomer Dot Plot

Protein was extracted from area at risk of 30 min LCA ligated mouse heart treated with exosome or saline after 15 minutes, 1 hour, 1 day, and 3 days reperfusion using a cell extraction buffer from Biovision, Mountain View, CA, USA according to manufacturer's instruction. Four animals were used at each time point in the exosome or saline treatment arm. One *μ*g of each sample was applied on a nitrocellulose membrane, air dried, stained with Ponceau S, quantified using Image Lab (Bio-rab Laboratories) and destained. The membrane was then blocked with 10% non-fat dry milk TBST solution, incubated with a 1 : 1000 diluted rabbit anti-oligomer antibody (Invitrogen, Carlsbad, CA) for 2 hours and washed 3 times with TBST. This was followed by one hour incubation with a 1 : 2000 diluted horseradish peroxidase-coupled donkey anti-rabbit IgG (Santa Cruz Biotechnology, Santa Cruz, CA, USA). 3 washes with TBST and incubation with HRP-enhanced chemiluminescent substrate (Thermo Fisher Scientific Inc., Waltham, MA USA). The signal was scanned using ChemiDoc System (Bio-rab Laboratories, Philadelphia, PA) and quantified using Image Lab (Bio-rab Laboratories). The signal was normalized against the intensity of the Ponceu S stain.

### 2.9. Sucrose Density Gradient Equilibrium Centrifugation

To generate the sucrose density gradient for centrifugation, 14 sucrose solutions with concentrations from 22.8 to 60% were prepared and layered sequentially in an ultracentrifuge tube (Beckman Coulter Inc., CA, USA) starting with the most concentrated solution. Exosome or human plasma was loaded on top before ultracentrifugation for 16.5 h at 200 000 g, 4°C in a SW60Ti rotor (Beckman Coulter Inc.). After centrifugation, 13 fractions of 330 *μ*L each were collected sequentially starting from the top of the gradient. The densities of each fraction were determined by weighing a fixed volume. For pretreatment with detergent-based lysis buffer (Cell Extraction Buffer, Biovision), exosome was incubated with an equal volume of the lysis buffer containing protease inhibitors (Halt Protease Inhibitor Cocktail, Thermo Fisher Scientific) for 30 min on ice before loading on the sucrose density gradient. For subsequent western blot hybridization, 20 *μ*L samples from each fraction were used. The mouse anti-CD63, -CD9, -CD81, and -CD59 antibodies, were obtained from Santa Cruz Biotechnology, Santa Cruz, CA, USA (1 : 60 dilution).

### 2.10. Liquid Phase Isoelectric Focusing

Liquid phase isoelectric focusing was performed using MicroRotofor, Liquid-Phase IEF Cell, from Bio-Rad (Bio-Rad Laboratories). 80 *μ*g exosome was added to 3 mL of 0.5% Bio-Lyte 3/10 Ampholyte (Bio-Rad Laboratories, diluted with PBS). The sample was loaded into the focusing chamber with 0.1 M phosphoric acid on anode and 0.1 M Sodium Hydroxide on cathode. Isoelectric focusing was performed for 215 min at 1 W constant power. The samples were then collected using vacuum suction into 10 fractions. The pH of each fraction was determined using pH indicator paper (Macherey-Nagel, Bethlehem, PA, USA) before concentrating to 25 *μ*L using 100 kDa MWCO filter (Millipore).

### 2.11. Cholera Toxin B Precipitation

50 *μ*L of each human plasma fraction from sucrose gradient density equilibrium ultracentrifugation as described above was incubated with 0.005 *μ*g biotinylated-cholera toxin B conjugated to streptavidin-conjugated magnetic beads (Invitrogen Corporation) for 30 min with gentle shaking followed by 30 min incubation The supernatant was then discarded and the magnetic beads were washed thrice with 0.1% BSA/PBS before incubating with 50 *μ*L of detergent-based lysis buffer (Cell Extraction Buffer, Biovision) on ice for 10 min. The lysate was then collected and biotinylated by incubating with 4 *μ*L 2.5 mM EZ-Link Sulfo-NHS-LC-LC-Biotin (Thermo Fisher Scientific) for 30 min with gentle shaking. The remaining reactive biotin was then quenched by adding 100 mM glycine buffer. The biotinylated lysate was then incubated with 5 *μ*L anti-CD81 or -20S *α*1–7 conjugated magnetic beads (Invitrogen Corporation) for 30 min with gentle shaking. The supernatant was then discarded and the magnetic beads were washed thrice with 0.1% BSA/PBS before incubating with 50 *μ*L of 1 : 5000 diluted HRP-conjugated streptavidin (Biolegend, San Diego, CA, USA) for 15 min at room temperature with gentle shaking. Finally the supernatant was discarded and the magnetic beads were washed thrice with 0.1% BSA/PBS. The amount of bound HRP was determined using Amplex UltraRed Reagent (Invitrogen Corporation) according to manufacturer's instruction.

## 3. Results

### 3.1. Proteomic Profiling of Exosome

**Figure 1:**
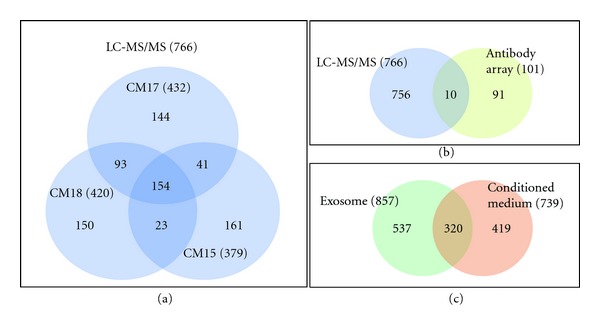
Exosome proteins. (a) Venn diagram of number of proteins detected by LC-MS/MS in 3 independent batches of exosomes, CM15, CM17, and CM18. There are 379, 432, and 420 proteins detected in CM15, CM17 and CM18, respectively. The combined protein number is 766. (b) Venn diagram shows the overlap of proteins detected by LC-MS/MS and antibody array. There are 101 proteins detected by antibody array in CM15. 10 of the 101 proteins detected are overlapping with proteins detected by LC-MS/MS. (c) Intersection of the 739 proteins previously identified in MSC conditioned medium versus the 857 proteins identified in purified exosomes.

Proteomic profiling using mass spectrometry and antibody array approaches were performed as previously described [24, 35, 37] on three or one independent preparations of HPLC-purified exosomes, respectively. Mass spectrometric analysis of the three exosome preparations identified 379, 432, and 420 proteins, respectively (please refer to Supplementary File 1 for the LC MS/MS raw data). Of these, 154 (~20%) were present in all three preparations, 157 (~20%) were present in two while 455 (~59%) were present in only one (Figure 1(a)). These analyses were subsequently validated by western blot hybridization or biochemical assays using either the three exosome preparations used in the mass spectrometric analysis or other independently prepared exosomes. Several proteins determined by mass spectrometry to be present in two or one of the three preparations were found to be present in these or other subsequent preparations by either western blot hybridization or biochemical assays. For example, PFKFB3 and PGK were detected in only one and two of the three batches, respectively. However, when tested by western blot hybridization or biochemical assays, both proteins were found to be present in two or more of the three preparations and also subsequent exosome preparations (data not shown). This indicated that the three independent exosome preparations analyzed by LC-MS/MS within nine months interval using three different sample preparation methods were complementary to each other in identifying different sets of low abundant proteins or our selection criteria were too stringent. To ensure a more comprehensive coverage of the exosome proteome, we combined the 3 sets of mass spectrometry data. The analysis of the exosome proteome by antibody array identified 101 proteins of which 10 were also detected by mass spectrometry analysis (Figure 1(b)). By combining the results from mass spectrometry and antibody array, we determined that the exosome proteome has a total of 857 proteins (Table 1) and this dataset has been deposited at http://www.exocarta.org/. In Table 1, the regular font symbols represent the proteins identified by LC MS/MS; the underline symbols represent the proteins identified by antibody arrays; the bold and underline symbols represent the proteins identified by both LC MS/MS and antibody arrays; and lastly the grey shaded symbols represent the proteins that were found to be present in at least 50% of exosomes characterized [38]. Of these 857 proteins, 320 were found in the 739 proteins previously identified in the unfractionated conditioned medium [24, 35] (Figure 1(c)). The remaining 537 proteins in the exosome were detected only when HPLC-purified exosomes were used. Since exosomes constituted about 10% of the total proteins in the conditioned medium [35], we postulated that many of the exosome proteins were masked by the more abundant nonexosome proteins in the conditioned medium.

Based on 15 proteomic analyses carried out on exosomes purified from cultured cells and from biological fluids by different groups, Thery et al. had observed that a set of about 17 proteins, namely glyceraldehyde 3-phosphate dehydrogenase (GAPDH), pyruvate kinase (PK), eukaryotic translation elongation factor 1A1 (EEF1A1), milk fat globule EGF factor 8 protein (MFGE8), tetraspanins, 14-3-3 proteins, G*α* proteins, clathrin, Alix (PDCD6IP), MHC class1, annexins (ANX), Rab proteins, ezrin(VIL2), radixin(RDX) and moesin (MSN)(ERM), actin, tubulin, HSP70, and HSP90 were found to be present in at least 50% of the exosomes that were characterized [38]. Not unexpectedly, most of these proteins were also found in the proteome of the HPLC-purified MSC exosomes (Table 1). Also consistent with the endosomal origin of exosomes, we detected the presence of endosome-associated proteins such as Alix (PDCD6IP) and Rab (Table 1).

To better understand the biological significance of the proteins in the exosomes, we had previously performed functional clustering of the 857 proteins into biological processes using PANTHER (protein analysis through evolutionary relationships) analytical software [39, 40] (Lai et al.; submitted) The observed frequency of genes from the exosome proteome in each biological process was compared with the reference frequency of 25431 *Homo sapiens *gene list in the NCBI database for that biological process. The 857 gene products could be clustered into 32 biological processes that were over-represented (*P* < 0.001) and 3 that were under-represented (*P* < 0.001) (Lai et al.; submitted). Many of these biological processes are consistent with activities associated with exosome biology for example, communication, cellular motility, inflammation, and exosome biogenesis. One predominant biological process that could not be definitively associated with exosome biology is proteolysis (*P* = 3.76 × 10^−10^) and of the unique genes clustered in this process, ~22% (17 out of 78) encode for peptides in the 20S proteasome or immunoproteasome. To ensure the robustness of this observation, examination of the 3 sets of mass spectrometry datasets revealed that with only one exception of PSMB5 in the first exosome preparation, all the 14 peptides of 20S proteasome were detected with very high confidence in all three exosome preparations as demonstrated by the Mascot search results, number of identified peptides with score higher than homology or identity scores, as well as the MS/MS spectra and fragment ions assignment of unique identified peptides (Supplementary data 1–7).

### 3.2. Presence of 20S Proteasome in MSC Exosome

**Figure 2:**
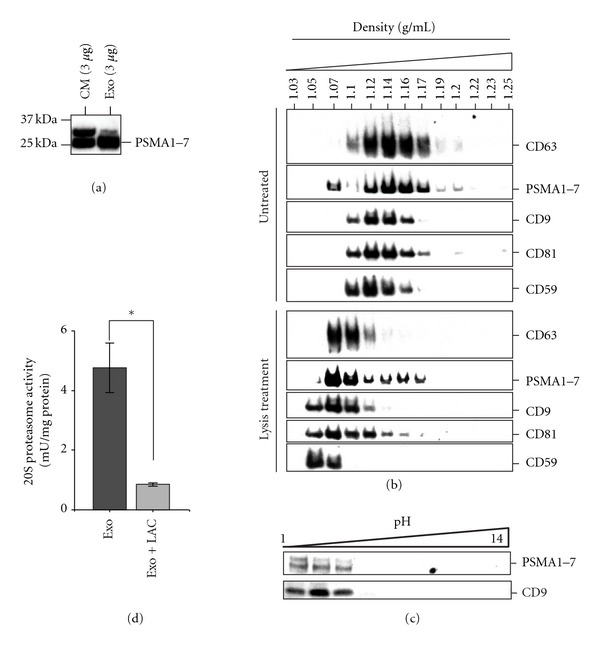
20S proteasome in MSC exosome. (a) Western blot analysis of MSC conditioned medium (CM) and exosome (Exo) using an antibody specific for PMSA 1–7 peptides. (b) Protein analysis of exosome fractionated on a sucrose gradient density. Exosome or exosome pretreated with lysis buffer was loaded on a sucrose density gradient prepared by layering 14 sucrose solutions of concentrations from 22.8 to 60% (w/v) in a SW60Ti centrifuge tube and then ultracentrifuged for 16.5 h at 200 000 g, 4°C, in a SW60Ti rotor. The gradients were removed from the top and the density of each fraction was calculated by weighing a fixed volume of each fraction. The fractions were analyzed by western blot analysis for CD9, PSMA1–7, CD81, CD59, and CD63 in exosome (upper panel) and pretreated exosomes (lower panel). (c) pI of 20S proteasome and CD9 in exosome. Exosome sample was separated by liquid-phase isoelectric focusing into 10 fractions from pH1–14. Each fraction was then concentrated and analyzed by western blot analysis for CD9, PSMA1–7. (d) Proteasome activity in MSC exosome was determined using a commercially available proteasome activity assay kit as described in the Materials and Methods section. Proteasome activity was measured by the rate of degradation of a fluorogenic peptide in the absence or presence of lactacystin, a proteasome inhibitor. One unit (U) enzyme activity is defined as the activity to generate 1 *μ*mole product per minute at 37°C. Each bar represents mean ± SEM of 2 independent assays with each assay performed in triplicate. *P = 0.00023.

Mass spectrometry analysis of MSC exosomes not only detected the presence of all seven *α*- (PSMA1–7) and all seven *β*-subunits (PSMB1–7) of the 20S core particle with very high confidence (See Supplementary Material available online at doi:10.1155/2012/971907 for the representative spectra, number of peptide detected, and the peptide score of them), but also the three beta subunits of “immunoproteasome,” PSMB8 (*β*5i or LMP7), PSMB9 (*β*1i or LMP2), PSMB10 (*β*2i or LMP10) gene product [41]. To form the 26S proteasome, the 20S core particle must be complemented by other peptides. Although some of these peptides such as PSMC5, PSMD6, PSMD7, PSMD11, and PSMD14 were present, they were not sufficient to form the 26S proteasome. The presence of some of the 20S proteasome peptides was further confirmed by western blot hybridization (Figure 2(a)). As exosomes are known to have a typical density range of 1.10 to 1.18 gmL^−1^ that could be resolved on sucrose gradients [42], we checked whether these 20S proteasome subunits have the typical exosome flotation density and postulated that the flotation densities of the 20S proteasome subunits would be different before and after release from such vesicles by a detergent-based buffer. Fractionation of exosomes or exosomes pretreated with a detergent-based lysis buffer on a sucrose density gradient by equilibrium ultracentrifugation revealed that 20S proteasome subunits had a similar flotation density as that of exosome-associated proteins, namely, CD9, CD81, CD59, and CD63. All of them had an exosomes-like flotation density of 1.10–1.18 g/mL (Figure 2(b)). Pretreatment with a detergent-based cell lysis buffer decreased the apparent flotation densities of these proteins. Liquid phase isoelectric focusing of exosomes further revealed that 20S proteasome had a similar protein isoelectric point (pI) as that of exosome-associated CD9 (Figure 2(c)). Together the exosome-like flotation density and pI of the 20S proteasome suggested that the 20S proteasome was associated with exosomes and not a contaminant of exosome preparation. 

### 3.3. Enzymatic Activity of 20S Proteasome in MSC Exosome

The presence of all seven *α*- and all seven *β*-subunits of the 20S core particle indicates that MSC exosomes contain intact 20S proteasome complexes and therefore 20S proteasome enzymatic activity. To test if exosomes contain functional 20S proteasome, purified MSC exosomes were incubated with a fluorophore-labeled peptide, LLVY-AMC (a substrate for the chymotrypsin-like activity; AMC, 7-amido 4-methylcoumarin) [43], that upon cleavage by 20S proteasome released fluorescent AMC. The fluorescence produced by the released AMC would be directly proportional to the proteasome activity. Based on this assay, the 20S proteasome enzymatic activity in MSC exosomes was determined to be 5.00 *μ*U/*μ*g protein where one unit (U) enzyme activity is defined as the activity to generate 1 *μ*mole product per minute at 37°C. This degradation was inhibited by lactacystin, a proteasome-specific inhibitor (Figure 2(d)).

### 3.4. Exosome Proteasome Reduced Misfolded Proteins *In Vivo*

To determine if exosome proteasome can reduce misfolded or aberrant proteins *in vivo* in a disease model and possibly contribute to a therapeutic outcome, we measured the accumulation of misfolded proteins in heart tissue of a mouse model of myocardial ischemia/reperfusion injury with and without exosome treatment.

This model is most appropriate for our aim as accumulation of misfolded proteins has been recognized as an important contributor to tissue damage during myocardial ischemia/reperfusion injury [44–48] (reviewed [49]) and we have previously shown that exosome treatment resulted in extensive cardioprotection [23]. Furthermore, in our hands, the area at risk in this mouse model is also highly reproducible [23, 24, 50–52].

To determine if exosome could reduce the amount of misfolded proteins, we used a conformation-dependent antibody [53] to quantify the amount of misfolded proteins in the area at risk of ischemic mouse heart after 15 min, one hour, one day and three days reperfusion. The level of denatured or misfolded protein was assayed using an antibody against oligomers. Denatured or partially unfolded proteins tend to self-associate to form high molecular aggregates such as oligomers and these oligomers have a common structure that is independent of amino acid sequence [54]. An antibody known as A11 that was raised against synthetic A*β* oligomers was found to specifically recognize this common structure present in misfolded protein oligomers but did not recognize monomers or mature fibers of proteins or peptides [54]. It will react with soluble A*β*40 oligomers and does not react with soluble low molecular weight A*β*40 or A*β*40 fibrils.

**Figure 3:**
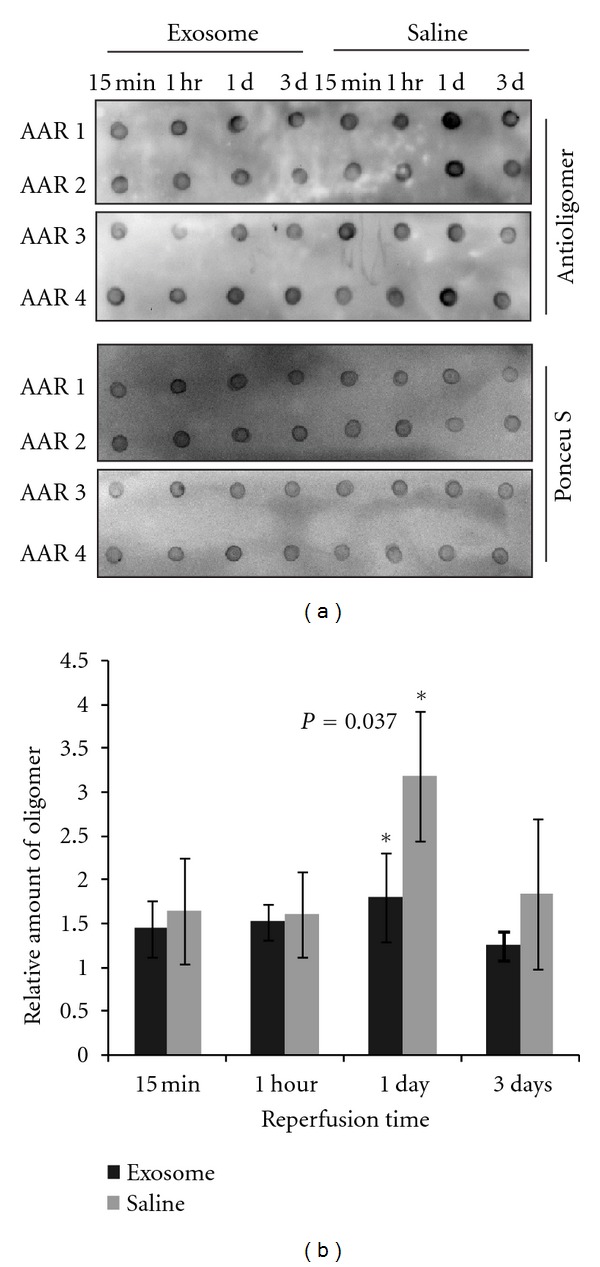
Exosome reduced oxidized protein *in vivo*. Myocardial ischemia/reperfusion injury was induced by ligation of the left coronary artery (LCA) for 30 min and subsequent reperfusion by releasing the ligation. Five minutes before reperfusion, mice (*n* = 4/group/time point) were intravenously infused with exosome or saline. At 15 min, 1 hour, 1 day, or 3 days after reperfusion, the animal was scarified and the area at risk (AAR) was excised and extracted for protein. One *μ*g of each sample was applied onto a nitrocellulose membrane, stained with Ponceau S, destained and probed with rabbit antioligomer antibody and a HRP reporting system as described in material and method. The intensity of the antibody binding was quantified using Image Lab and normalized against the intensity of Ponceau S staining. (a) Representative dot plots. (b) Each bar represented mean ± SEM of 4 animal.

Using this antibody, we found that those mice treated with exosome had a modest but significantly lower level of misfolded proteins (*P* = 0.037) (*n* = 4 per time point) 1 day after reperfusion compared with those saline treated controls (*n* = 4 per time point) (Figure 3). This demonstrated that MSC exosome has the potential to reduce misfolded proteins *in vivo* and may contribute to the exosome-mediated cardioprotection.

### 3.5. Association of Plasma 20S Proteasome with Exosome

**Figure 4:**
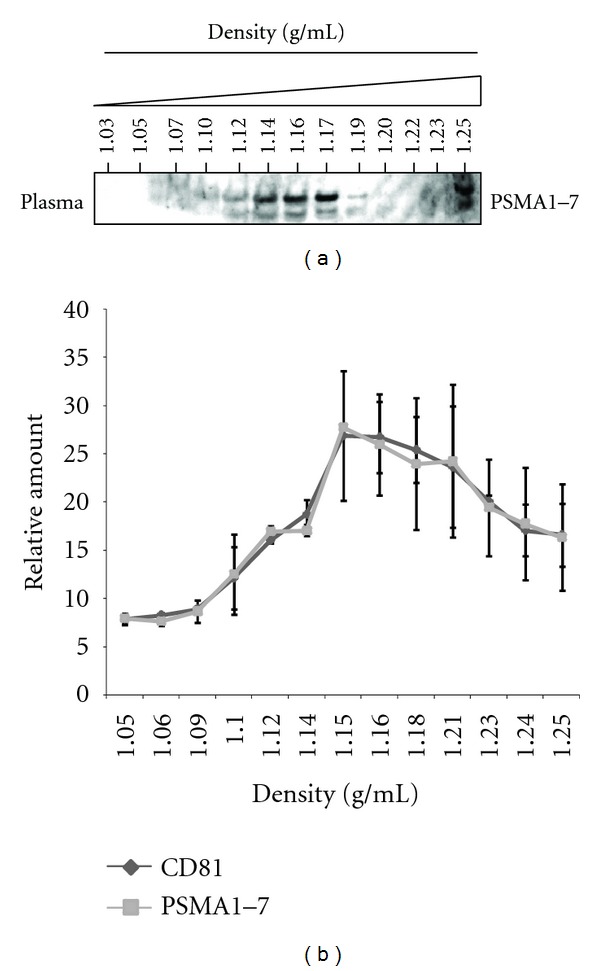
Human plasma contained exosome-associated 20S proteasome. (a) Human plasma was loaded on a sucrose density gradient prepared by layering 14 sucrose solutions of concentrations from 22.8 to 60% (w/v) in a SW60Ti centrifuge tube and then ultracentrifuged for 16.5 h at 200 000 g, 4°C, in a SW60Ti rotor. 330 *μ*L fractions were removed sequentially from the top and the density of each fraction was calculated by weighing a fixed volume of each fraction. The fractions were analyzed by Western blot analysis for PSMA1–7. (b) Relative amount of CD81 and PSMA1–7 in plasma exosome. Exosomes which are known to have membrane enriched in GM1 gangliosides were isolated from the sucrose density gradient by the high affinity of GM1 gangliosides for cholera toxin B (CTB) chains conjugated to magnetic beads. The CTB extract was assayed by ELISA for the amount of PSMA1–7 and CD81. Each point represents mean ± SEM (*n* = 2).

Although our experimental observations strongly supported the presence of functional 20S proteasome in MSC exosomes, the association of 20S proteasome with exosomes could be a culture artifact contaminant. To test this possibility, we determined if circulating 20S proteasome in human plasma is associated with exosomes. 20S proteasomes are widely reported to be present in plasma [27–31] and we confirmed this by immunoblotting plasma for the presence of proteasome subunits (Figure 4(a)). Fractionation of plasma on a sucrose density gradient revealed that the plasma 20S proteasome fractionated at the flotation density of exosome that is, 1.10–1.18 g/mL (Figure 4(a)). CD81, a tetraspanin protein commonly found on exosome membrane also floated at this density. Each of the fractions was then extracted with cholera toxin B chain (CTB) which binds GM1 ganglioside, a glycolipid found to be enriched in the membrane of exosomes [55] and also MSC exosomes (unpublished data). CTB together with cholera toxin A chain forms the cholera toxin. Cholera toxin enters the cytosol by binding GM1 ganglioside through CTB [56]. This cellular entry of cholera toxin is a well characterized highly specific receptor mediated pathway [57]. The CTB extract was then assayed by ELISA for 20S proteasome subunits and CD81. 20S proteasome subunits and CD81 were both found to be present in the CTB extraction (Figure 4(b)). These observations demonstrated that plasma 20S proteasome colocalized with CD81 in GM1 ganglioside-enriched complex that floats at 1.10–1.18 g/mL and that at least some of the circulating proteasomes in the plasma are associated with exosomes. The presence of 20S proteasome in MSC exosomes and plasma exosomes together implicated exosome as a normal physiological conduit for cellular extrusion of 20S proteasome.

## 4. Discussion

In this paper, we investigated the proteome of MSC exosome to identify candidate proteins or protein complexes that could contribute to their therapeutic efficacy in ameliorating myocardial ischemia/reperfusion injury or other pathological conditions. We hypothesize that this therapeutic efficacy against a complex injury is likely to be derived from the synergy of a select permutation of individual exosome components rather than a single component. To elucidate this permutation which may include proteins, RNA and other molecules, we focused first on the proteins by elucidating the exosome proteome to identify candidate proteins or protein complexes that have the biochemical potential to elicit a therapeutic response.

The proteome of 3 independently prepared, HPLC-purified ESC-derived MSC exosome using mass spectrometry and cytokine array were analyzed and 857 proteins were identified. We have previously reported that 739 proteins were profiled in hESC-MSC conditioned medium using LTQ-FT mass spectrometer. These proteins included many proteins commonly found in other exosomes. The proteome of MSC exosomes contained a diverse array of proteins. Clustering of these proteins according to their functions suggested that the exosome has the potential to drive many biological processes (Lai et al., submitted). This is consistent with the reported efficacy of MSCs in treating a myriad of diseases such as cardiovascular diseases (e.g., acute myocardial infarction, endstage ischemic heart disease, or prevention of vascular restenosis), osteogenesis imperfecta (OI) or brittle bone disease, amyotrophic lateral sclerosis (ALS), lysosomal storage diseases (e.g., Hurler syndrome), steroid refractory Graft versus Host Disease (GVHD), and periodontitis and bone fractures [12].

A significant fraction of proteins in MSC exosomes are involved in the highly regulated and complex intracellular membrane trafficking and sorting through the biosynthetic and endocytotic pathways [58], and these processes probably reflect the biogenesis of exosomes. In fact, many of the proteins in these processes constitute a common set of proteins found in exosomes from different cell sources [59]. However, the biological significance of the other proteins remains to be uncovered. Exosomes have been implicated in an increasing number of important physiological and pathological processes such as disposal of unwanted protein [60], antigen presentation [42], genetic exchange [61], immune responses [38, 62] and tumor metastasis [38, 62–66]. Whether the proteome of MSC exosomes could sustain such processes remains to be determined.

Proteasome subunits have been previously reported to be present in exosomes [61, 67–69] but this is the first time that mass spectrometry analysis of exosome proteome had detected the entire protein complement of a 20S proteasome with very high confidence. In addition, we also demonstrated that exosome had the lactacystin-sensitive proteolytic activity of 20S proteasome. Since decreased proteasome activity resulting in accumulation of misfolded proteins has been recognized as an important contributor to tissue damage during myocardial ischemia/reperfusion injury [44–48] (reviewed [49]) and MSC exosomes reproducibly reduce IS/AAR by ~40% in a mouse model of myocardial ischemia/reperfusion injury [23, 24, 52] we tested if cardioprotective MSC exosomes exert some of their therapeutic effects through proteolytic degradation of misfolded proteins. The level of denatured or misfolded protein was assayed using an antibody against oligomers. Using this antibody, we demonstrated that accumulation of misfolded proteins or oligomers was reduced in the heart tissues of a mouse model of myocardial ischemia/reperfusion injury that had been treated with MSC exosomes. This positive correlation between exosome-mediated decreased accumulation of misfolded proteins and exosome-mediated reduction in IS/AAR supports a therapeutic role the 20S proteasome in MSC exosome.

20S proteasome is responsible for the degradation of about 90% of all intracellular oxidatively damaged proteins [70] and reduced proteasomal activity has been postulated to be a contributing factor in the pathogenesis of aging-related neurodegenerative diseases such as Alzheimer's disease and Parkinson's disease [71, 72] or cardiovascular disease [73–75]. The presence of all seven *α* and *β* subunits of the 20S proteasome, the three beta subunits of “immunoproteasome” and validation of 20S proteasome enzymatic activity in the exosomes *in vitro* and *in vivo* suggested that the cardioprotective activity of MSC exosomes [35] could be partly attributed to the presence of 20S proteasome. This further implied that MSC exosomes have the potential to therapeutically correct for proteasome insufficiency. Many debilitating neurodegenerative diseases such as Alzheimer's, Parkinson's or prion disease that are caused by accumulation of denatured or misfolded proteins could thus be alleviated by MSC exosomes carrying functional proteasomes. The use of exosomes as a delivery vehicle for proteasome has the added advantage of being able to transcend the blood-brain-barrier. This barrier is the main cause in the intractability of neurodegenerative diseases to medical intervention. Unlike most conventional drugs, exosomes could cross the blood-brain-barrier as demonstrated by the recent knockdown of the gene target in the brain by exosomes loaded with exogenous siRNA [76].

The presence of 20S proteasome in MSC exosomes also suggested that cells extruded 20S proteasome through exosomes. Consistent with this, we observed that plasma proteasomes had the flotation density (1.10–1.19 g/mL) and pI of exosomes, and were associated with GM1 gangliosides, a glycolipid found to be enriched in the membrane of exosome [77]. This finding supported our hypothesis that cellular extrusion of 20S proteasomes via exosomes is a normal physiological process.

Together this study demonstrated that MSC and plasma exosomes contain functional 20S proteasome, and cells secrete proteasome via the exosome pathway. MSC exosomes could reduce misfolded proteins in the heart after ischemia/reperfusion injury suggesting that they could also be used to alleviate diseases precipitated by accumulation of denatured proteins such as Alzheimer's disease, Parkinson's disease or prion disease.

In conclusion, MSC exosome has functional 20S proteamsomes and its presence correlated with a modest but significant reduction in oligomerized protein in a mouse model of myocardial infarction. Therefore, 20S proteasome is a candidate exosome protein that could synergize with other constituents in the exosome to ameliorate tissue damage.

## Supplementary Material

Supplementary material provides the raw data of the detected 20S proteasome in 3 independent batches of exosomes. Supplementary data 1 provides a summary of number of detected peptides in 3 independent batches of exosomes; Supplementary data 2–4 provide the raw data of detected 20S proteasome peptides in 3 independent batches of exosomes respectively; Supplementary data 5–7 provide the Mascot peptide view of unique 20S proteasome peptides in 3 independent batches of exosomes respectively.

## Figures and Tables

**Table 1:** Proteomic profile of 3 independently prepared exosomes as determined by LC MS/MS and antibody arrays.

A2M	C11orf59	COPS4	FBXW8	HNRNPA1	KRT16	MMP3	PRR4	RPS3	TGFB2
ABI3BP	C1orf78	COPS8	FEN1	HP	KRT17	MOS	PRSS23	RPS4X	TGFBI
ACAA2	C1R	CPS1	FER1L3	HPX	KRT18	MPO	PSMA1	RPS5	TGM2
ACAT2	C1S	CREG1	FGA	HRSP12	KRT19	MPZL1	PSMA2	RPSA	TGOLN2
ACLY	C20orf114	CRIPT	FGB	**HSP90AA1**	KRT2	MRC2	PSMA3	RRAS2	THBS1
ACSL1	C3	CRTAP	FGF16	**HSP90AB1**	KRT27	**MSN**	PSMA4	RTN4	**THBS2**
**ACTA1**	C5orf24	CSF1	FGF18	**HSP90B1**	KRT28	MXRA5	PSMA5	RUVBL1	THY1
**ACTA2**	C9orf19	CSF2	FGF19	**HSPA1A**	KRT3	MYADM	PSMA6	S100A11	TIMP1
**ACTB**	C9orf91	CSF3	FGFRL1	**HSPA1L**	KRT4	MYCBPAP	PSMA7	S100A13	TIMP2
**ACTG2**	CACNA2D1	CSPG4	FGG	**HSPA5**	KRT5	MYH14	PSMB1	S100A8	TIMP3
ACTN1	CACNA2D4	CST4	FLG2	**HSPA6**	KRT6A	MYH9	PSMB10	S100P	TKT
ACTN2	CALR	CTA-221G9.4	FLJ13197	**HSPA8**	KRT6B	MYL6B	PSMB2	SAA4	TLN1
ACTN3	CAND1	CTBP2	FLJ22184	HSPB1	KRT6C	MYO1C	PSMB3	SASS6	TMBIM1
ACTN4	CAP1	CTNNA1	FLJ32784	HSPD1	KRT7	NBL1	PSMB4	SCAMP3	TMED10
ACTR1A	CAPNS1	CTNNA2	FLNA	HSPG2	KRT72	NEFH	PSMB5	SCGB2A1	TMED9
ACTR2	CAPZA1	CTNNB1	FLNB	HTRA1	KRT73	NEK10	PSMB6	SCYE1	TMEM16B
ACTR3	CASP14	CTNND1	FLNC	HYI	KRT74	NID1	PSMB7	SDC1	TMEM2
ADAM10	CAT	CTSG	FLOT1	**ICAM1**	KRT76	NLRP8	PSMB8	SDC2	TMEM47
ADAM9	CAV1	CXCL16	FLOT2	ICAM5	KRT77	NME1	PSMB9	SDC4	TMEM51
ADAMTS12	CCDC129	CXCL2	FLT1	IDH3B	KRT78	NOMO1	PSMC5	SDCBP	TNC
AEBP1	CCDC64B	CXorf39	FN1	IFITM2	KRT79	NRAS	PSMD11	SEC14L4	TNFRSF11B
AFM	CCL2	CYBRD1	FREM3	IFNG	KRT8	NRG2	PSMD14	SEMA5A	TNFRSF12A
AGRN	CCL20	DBF4B	**FST**	IFRD1	KRT80	NRLN1	PSMD6	SEPT2	TNFRSF1A
AHCY	CCL28	DCD	FTL	IFT140	KRT84	NRP1	PSMD7	SEPT7	TNFSF18
AHNAK2	CCL7	DCHS2	FUCA2	IGF2R	KRT9	NT5E	PTGFRN	SERINC5	TNFSF5
AHSG	CCR4	DCLK2	GALNT5	IGFBP3	LACRT	NTF5	PTK7	SERPINA1	TPBG
AKR1B1	CCR5	**DCN**	GANAB	IGFBP4	LAMA4	NUSAP1	PTPRK	SERPINB3	TPI1
AKR7A2	CCT5	DCTN1	**GAPDH**	IGFBP6	LAMB1	OBFC1	PTRF	SERPINE1	TRAP1
ALB	CCT6A	DECR1	GAPDHS	**IGFBP7**	LAMC1	ODZ3	PTTG1IP	SERPINE2	TREM1
ALCAM	CD109	DEFA1	GARS	IGHA1	LAMP1	OFD1	**PTX3**	SERPINF1	TREML2P
ALDH2	CD151	DIP2B	GAS6	IGHA2	LAMP2	OPRM1	PXDN	SFN	TRIM40
ALDH3A2	CD248	DIRAS2	GDF1	IGHG1	LAP3	OSM	PZP	SFRP1	TRIM41
ALDH6A1	CD276	DKFZp686D0972	GDF11	IGHG2	LCN1	OTC	QPCTL	SFRP4	TSN
ALDH7A1	CD44	DKK1	GDF3	IGHG4	LCN2	OXNAD1	QSOX1	SHANK3	TSNAX
ALDH9A1	CD47	DKK3	GDF5	IGHM	LDHA	OXTR	**RAB10**	SLAIN1	TSPAN14
ALDOA	CD59	DMBT1	GDF8	IGJ	LDHAL6B	P4HB	**RAB11B**	SLC16A1	TSPAN4
ALDOB	**CD63**	DNASE1L1	GDF9	IGKC	LDHB	PAICS	**RAB14**	SLC16A3	TSPAN6
ALDOC	**CD81**	DNPEP	GDI1	IGKV1-5	LEPRE1	PAN3	**RAB15**	SLC1A4	TSPAN9
ALOX12P2	CD82	DPYS	GDI2	IGL@	LGALS1	PAPPA	**RAB1A**	SLC1A5	TSTA3
ANG	**CD9**	DPYSL2	GFRA3	IGLV4-3	LGALS3	PARP10	**RAB1B**	SLC22A2	TTLL3
ANGPTL2	CDC2L5	DSP	GLDC	IGSF8	LGALS3BP	PARP16	**RAB2A**	SLC25A10	TTN
ANPEP	CDC42	DULLARD	GLUD1	IL10	LGALS8	PARVG	**RAB33B**	SLC25A13	TTYH3
**ANXA1**	CDH13	ECM1	**GNA13**	IL11	LGR6	PC	**RAB35**	SLC2A1	**TUBA1A**
**ANXA11**	CDIPT	ED1	GNAI2	IL13	LIF	PCOLCE	**RAB39B**	SLC2A3	**TUBA1B**
**ANXA2**	CDK5R2	EDG2	GNAL	IL15RA	LMNA	PDCD6	**RAB5A**	SLC38A2	**TUBA1C**
**ANXA2P1**	CEACAM8	EDIL3	GNAS	IL17B	LOC124220	**PDCD6IP**	**RAB5B**	SLC38A3	**TUBB**
**ANXA3**	CFB	EEA1	GNAT3	IL17R	LOC283523	PDGFA	**RAB5C**	SLC39A14	**TUBB2A**
**ANXA4**	CFI	**EEF1A1**	GNB1	IL19	LOC284297	PDGFC	**RAB6A**	SLC3A2	**TUBB2C**
**ANXA5**	CFL1	EEF1G	GNB2	IL1F9	LOC388344	PDGFRB	**RAB7A**	SLC44A1	**TUBB3**
**ANXA6**	CFL2	EEF2	GNB4	IL1RAP	LOC389827	PDIA3	**RAB8A**	SLC44A2	**TUBB6**
**ANXA7**	CFTR	EFEMP2	GNG12	IL1RAPL1	LOC442497	PEBP1	**RAB8B**	SLC7A10	UBA52
AP1S1	CHMP2A	EHD1	GNPDA1	IL1RL2	LOC653269	PFAS	RAC1	SLC7A5	UBB
APEH	CHST12	EHD2	GOT2	IL22RA1	LOC727942	PFKFB3	RAC2	SMARCA4	UBE1
APOA1	CITED1	EHD4	GPC1	IL23A	LOC728320	PFN1	RAD21	SMC1A	UBE2N
APOE	CLASP2	EIF4A1	GPC5	IL3	LOC728378	PFN2	RALA	SORT1	UGP2
APP	CLDN1	EMILIN1	GPI	IL5	LOC730013	PGAM2	RAN	SPACA1	UNC13B
ARF1	CLEC11A	ENG	GPR112	IL6ST	LRP1	PGD	RAP1A	SPARC	UNC45A
ARF4	CLIC1	ENO1	GREM1	IL7	LRP6	PGK1	RAP1B	SPOCK1	VAMP3
ARF5	CLIC6	ENO2	GRM2	IL8	LRRFIP2	PGLYRP2	RAP1B	SPRY4	VANGL1
ARHGAP18	CLPX	ENO3	GRM3	INHBA	**LTBP1**	PIGR	RAP2C	SPTAN1	**VASN**
ARHGAP23	CLSTN1	ENTPD4	GRM7	INHBB	LTBP2	PIP	RARRES1	SPTBN1	VAT1
ARHGDIA	**CLTA**	ENTPD4;LOXL2	GSN	INSR	LTF	**PKM2**	RASA1	SPTBN4	VCAN
ARHGEF1	**CLTC**	EPB41L3	GSTM1	IQGAP1	LYAR	PLAB	RASA4	SRGN	VCL
ARL6IP5	**CLTCL1**	EPHA2	GSTM2	ITGA11	LYZ	PLAU	RB1CC1	SRI	VCP
ARMS2	CLU	EPO	GSTM5	ITGA2	MADH4	PLEC1	RCOR2	SRPX2	VEGFC
ARPC3	CMIP	ESM1	GSTO1	ITGA3	MAMDC2	PLEKHG3	RDH5	ST6GALNAC6	VIL1
ARPC4	CNGB1	ETFB	GSTP1	ITGA4	MAP1A	PLOD1	RFTN1	STAT1	**VIL2**
ARPC5	COL12A1	F2R	GTPBP2	ITGA5	MAP2K6	PLOD2	RGN	STC1	VIM
ASH1L	COL14A1	**F3**	GYLTL1B	ITGAL	MAP3K1	PLOD3	RHOC	STC2	VTI1A
ASL	COL18A1	F8	GZMA	ITGAV	MARCKS	PLP2	RMND5A	STOM	VTN
ATP1A1	COL1A1	FADD	H2AFV	ITGB1	MARCKSL1	PLSCR3	RNF123	STOML3	WDR49
ATP1B3	COL1A2	FAH	H2AFX	ITGB5	MAT1A	PLTP	RNF40	STX12	WDR52
ATP2B1	COL2A1	FAM108A1	HBB	ITIH2	MBD3	PLUNC	RPL10A	STX2	WNT5A
ATP2B4	COL3A1	FAM129B	HBE1	ITIH4	MCC	PNO1	RPL12	SURF4	YBX1
ATP5A1	COL4A1	FAM29A	HDAC5	ITPR2	MCM10	PODN	RPL15	SVEP1	**YWHAB**
ATP5B	COL4A2	FAM3B	HERC5	JUP	MDH1	POLN	RPL18	SYT1	**YWHAE**
ATP8B3	COL4A3	FAM64A	HGF	KIAA0146	MDH2	POSTN	RPL23	SYT9	**YWHAG**
ATRN	COL5A1	FAM71F1	HGFR	KIAA0256	ME1	POTE2	RPL29	TAAR2	**YWHAQ**
ATXN1	COL5A2	FAP	HISPPD2A	KIAA0467	MECP2	PPIA	RPL35A	TAGLN	**YWHAZ**
AXL	COL6A1	FASN	HIST1H2AE	KIAA1881	MFAP4	PPIB	RPLP0	TALDO1	ZBTB4
BASP1	COL6A2	FAT	HIST1H2BA	KPNB1	**MFGE8**	PPME1	RPS10	TAS2R60	ZNF134
BDNF	COL6A3	FAT2	HIST1H2BL	KRT1	MFSD2	PPP1CC	RPS16	TCN1	ZNF503
BGN	COL7A1	FAT4	HIST1H4H	KRT10	MIF	PRDM16	RPS18	TF	ZNF614
BHMT2	COMP	FBLN1	HIST2H2BE	KRT13	MMP1	PRDX1	RPS2	TFG	
BRMS1	COPB1	FBN1	**HLAA**	KRT14	MMP10	PRDX6	RPS24	TFRC	
BSG	COPS3	FBN2	HMGCS2	KRT15	**MMP2**	PRNP	RPS27A	TGFB1	

Regular font: identified by LC MS/MS.

Underline: identified by antibody arrays.

**Bold and Underline:** identified by both LC MS/MS and antibody arrays.

**Bold:** identified by LC MS/MS and was found to be present in at least 50% of exosomes characterized.
