# Supplementary material for: Proteolytic Potential of the MSC Exosome Proteome: Implications for an Exosome-Mediated Delivery of Therapeutic Proteasome
Source: Int J Proteomics. 2012 Jul 18;2012:971907. doi: 10.1155/2012/971907 (PMC3407643; doi:10.1155/2012/971907)
Supplement: Supplementary file 1 — Supplementary material provides the raw data of the detected 20S proteasome in 3 independent batches of exosomes. Supplementary data 1 provides a summary of number of detected peptides in 3 independent batches of exosomes; Supplementary data 2–4 provide the raw data of detected 20S proteasome peptides in 3 independent batches of exosomes respectively; Supplementary data 5–7 provide the Mascot peptide view of unique 20S proteasome peptides in 3 independent batches of exosomes respectively. [file 971907.f1.zip › SupplementaryData5_Exosome1_MascotPeptideView_UniqueProteasomePeptides.pdf]

Mascot Search Results

Results Generated by a Script Modified from Mascot Pepitde View
by Newman Sze, School of Biological Sciences, Nanyang Technological University

---

Spectrum No: 1; Query: 12332; Rank: 1

Peptide View

MS/MS Fragmentation of **GYSFSLTTFSPSGK**
Found in **IPI00219622**, Tax_Id=9606 Gene_Symbol=PSMA2 Proteasome subunit alpha type-2

Match to Query 12332: 1477.712608 from(739.863580,2+)
Title: 090324LimSK_Exosome1_A.11819.11819.2.dta
Data file C:\Temp\Mascoti\LSK_2D\090324LimSK_Exosome1_A_2D.mgf

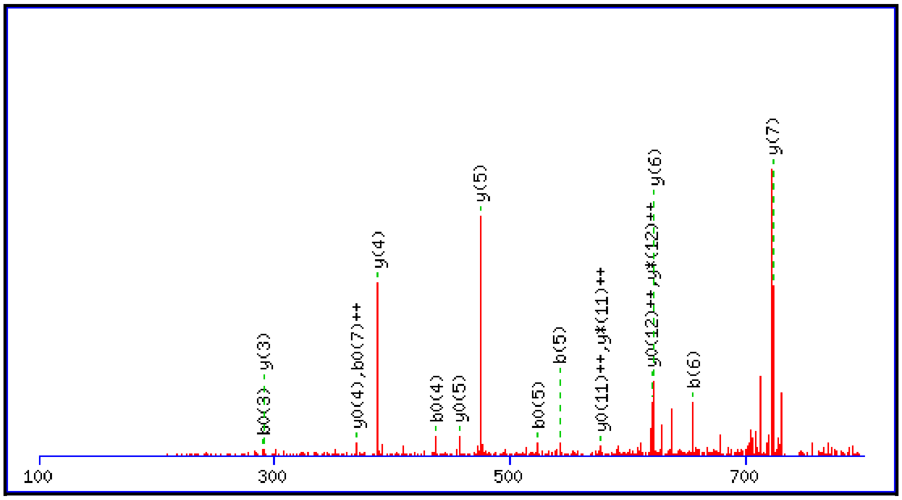

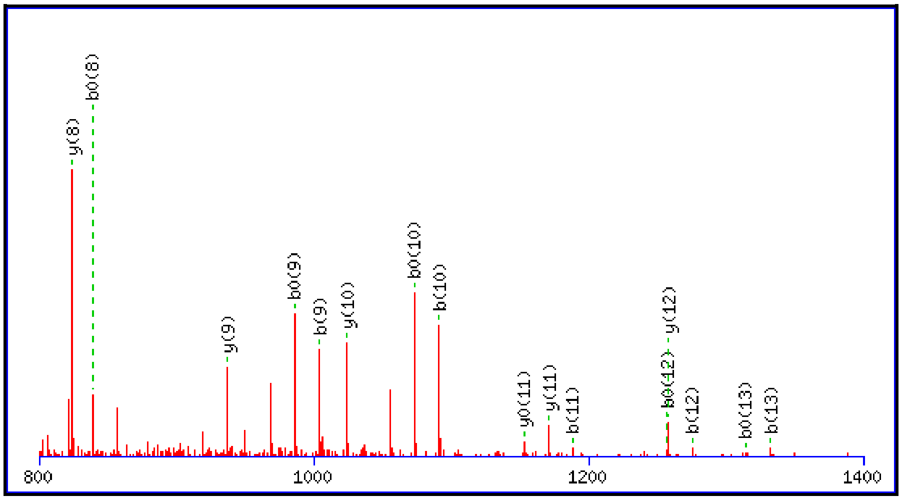

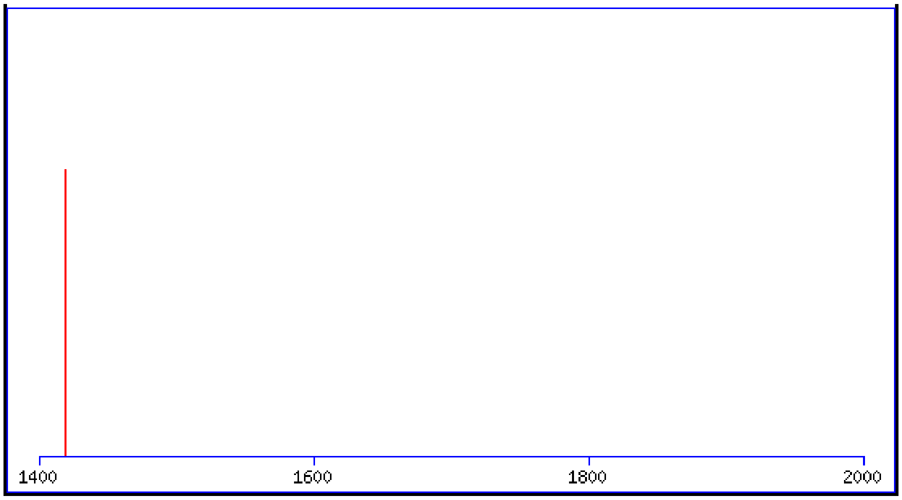

Monoisotopic mass of neutral peptide Mr(calc): 1477.7089
Fixed modifications: Carbamidomethyl (C)
Ions Score: 90 **Expect:** 1.9e-007
Matches (Bold Red): 33/122 fragment ions using 43 most intense peaks

#	b	b^{++}	b^{0}	b^{0++}	Seq.	y	y^{++}	y^{*}	y^{*++}	y^{0}	y^{0++}	#
1	58.0287	29.5180			G							14
2	221.0921	111.0497			Y	1421.6947	711.3510	1404.6682	702.8377	1403.6842	702.3457	13
3	308.1241	154.5657	**290.1135**	145.5604	S	**1258.6314**	629.8193	1241.6048	**621.3061**	1240.6208	**620.8141**	12
4	455.1925	228.0999	**437.1819**	219.0946	F	**1171.5994**	586.3033	1154.5728	**577.7900**	**1153.5888**	**577.2980**	11
5	**542.2245**	271.6159	**524.2140**	262.6106	S	**1024.5310**	512.7691	1007.5044	504.2558	1006.5204	503.7638	10
6	**655.3086**	328.1579	637.2980	319.1527	L	**937.4989**	469.2531	920.4724	460.7398	919.4884	460.2478	9
7	756.3563	378.6818	738.3457	**369.6765**	T	**824.4149**	412.7111	807.3883	404.1978	806.4043	403.7058	8
8	857.4040	429.2056	**839.3934**	420.2003	T	**723.3672**	362.1872	706.3406	353.6740	705.3566	353.1819	7
9	**1004.4724**	502.7398	**986.4618**	493.7345	F	**622.3195**	311.6634	605.2930	303.1501	604.3089	302.6581	6
10	**1091.5044**	546.2558	**1073.4938**	537.2506	S	**475.2511**	238.1292	458.2245	229.6159	**457.2405**	229.1239	5
11	**1188.5572**	594.7822	1170.5466	585.7769	P	**388.2191**	194.6132	371.1925	186.0999	**370.2085**	185.6079	4
12	**1275.5892**	638.2982	**1257.5786**	629.2930	S	**291.1663**	146.0868	274.1397	137.5735	273.1557	137.0815	3
13	**1332.6107**	666.8090	**1314.6001**	657.8037	G	204.1343	102.5708	187.1077	94.0575			2
14					K	147.1128	74.0600	130.0863	65.5468			1

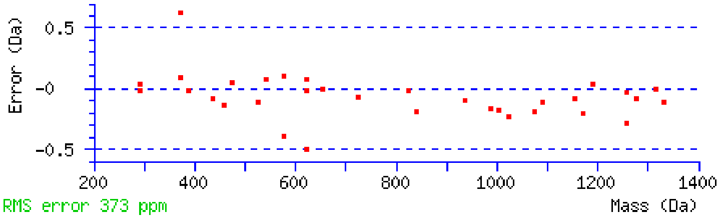

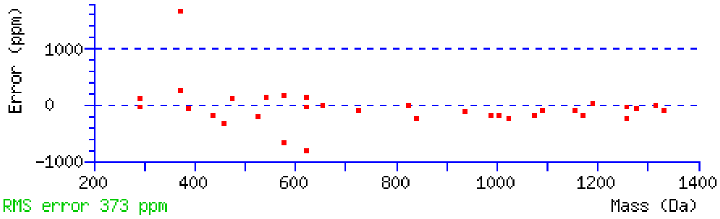

All matches to this query

Score	Mr(calc):	Delta	Sequence
90.0	1477.7089	0.0037	GYSFSLTTFSPSGK
10.9	1475.7061	2.0065	VKFASLEFSPGSK
9.5	1476.6974	1.0153	SDIPSGTSKHLQK
6.1	1477.7112	0.0014	MLLAPPSTPSRGR
4.3	1477.7040	0.0086	GYLQMSPLFKAK
2.5	1476.6974	1.0153	SDIPSGTSKHLQK
2.2	1477.7112	0.0014	MLLAPPSTPSRGR
1.1	1477.7112	0.0014	LSMGGLHSSRPLK

0.5	1477.7121	0.0005	SRSGSSPEVDSKSR

Spectrum No: 2; Query: 30832; Rank: 1

Peptide View

MS/MS Fragmentation of **KLAQQYYLVYQEPIPTAQLVQR**
Found in **IPI00219622**, Tax_Id=9606 Gene_Symbol=PSMA2 Proteasome subunit alpha type-2

Match to Query 30832: 2648.442102 from(883.821310,3+)
Title: 090324LimSK_Exosome1_B.13230.13230.3.dta
Data file C:\Temp\Mascoti\LSK_2D\090324LimSK_Exosome1_A_2D.mgf

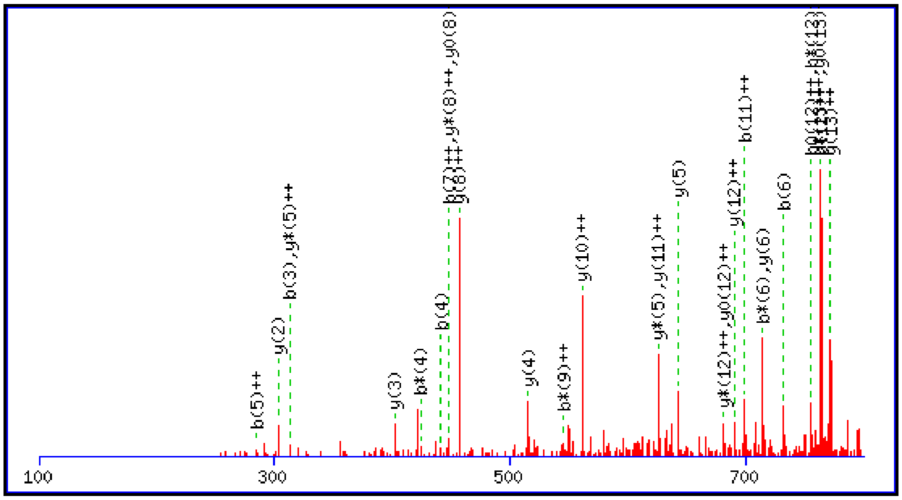

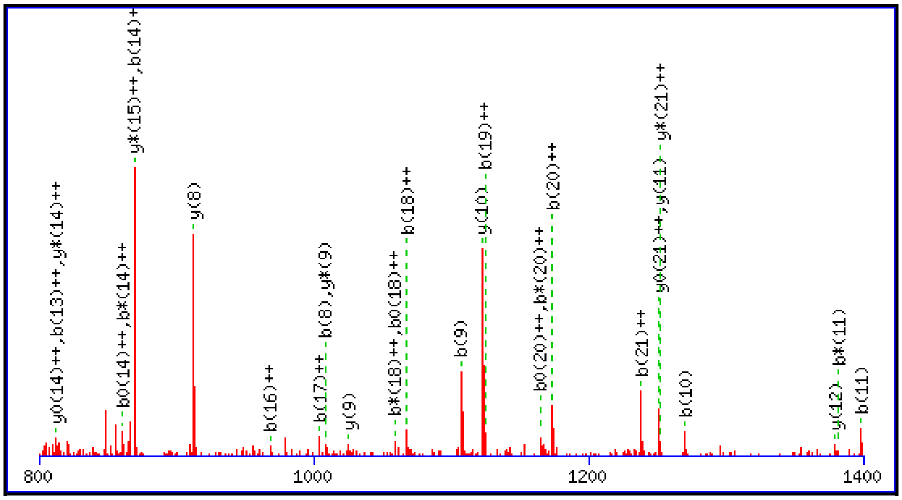

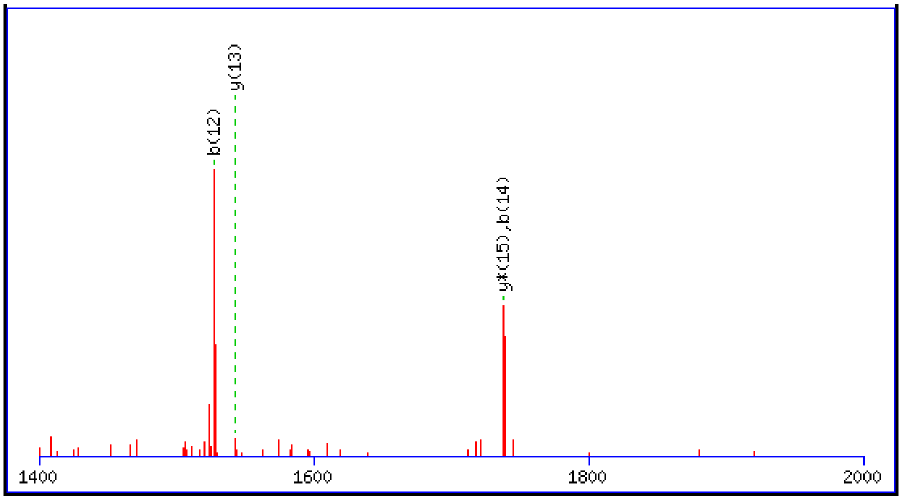

Monoisotopic mass of neutral peptide Mr(calc): 2648.4326
Fixed modifications: Carbamidomethyl (C)
Ions Score: 60 **Expect:** 5.1e-005
Matches (Bold Red): 65/218 fragment ions using 111 most intense peaks

#	b	b^{++}	b^{*}	b^{*++}	b^{0}	b^{0++}	Seq.	y	y^{++}	y^{*}	y^{*++}	y^{0}	y^{0++}	#
1	129.1022	65.0548	112.0757	56.5415			K							22
2	242.1863	121.5968	225.1598	113.0835			L	2521.3449	1261.1761	2504.3184	**1252.6628**	2503.3344	**1252.1708**	21
3	**313.2234**	157.1153	296.1969	148.6021			A	2408.2609	1204.6341	2391.2343	1196.1208	2390.2503	1195.6288	20
4	**441.2820**	221.1446	**424.2554**	212.6314			Q	2337.2238	1169.1155	2320.1972	1160.6022	2319.2132	1160.1102	19
5	569.3406	**285.1739**	552.3140	276.6607			Q	2209.1652	1105.0862	2192.1386	1096.5730	2191.1546	1096.0809	18
6	**732.4039**	366.7056	**715.3774**	358.1923			Y	2081.1066	1041.0569	2064.0801	1032.5437	2063.0960	1032.0517	17
7	895.4672	**448.2373**	878.4407	439.7240			Y	1918.0433	959.5253	1901.0167	951.0120	1900.0327	950.5200	16
8	**1008.5513**	504.7793	991.5247	496.2660			L	1754.9799	877.9936	**1737.9534**	**869.4803**	1736.9694	**868.9883**	15
9	**1107.6197**	554.3135	1090.5932	**545.8002**			V	1641.8959	821.4516	1624.8693	**812.9383**	1623.8853	**812.4463**	14
10	**1270.6830**	635.8452	1253.6565	627.3319			Y	**1542.8275**	**771.9174**	1525.8009	**763.4041**	1524.8169	**762.9121**	13
11	**1398.7416**	**699.8744**	**1381.7151**	691.3612			Q	**1379.7641**	**690.3857**	1362.7376	**681.8724**	1361.7536	**681.3804**	12
12	**1527.7842**	**764.3957**	1510.7577	**755.8825**	1509.7736	**755.3905**	E	**1251.7056**	**626.3564**	1234.6790	617.8431	1233.6950	617.3511	11
13	1624.8370	**812.9221**	1607.8104	804.4089	1606.8264	803.9168	P	**1122.6630**	**561.8351**	1105.6364	553.3218	1104.6524	552.8298	10
14	**1737.9210**	**869.4642**	1720.8945	**860.9509**	1719.9105	**860.4589**	I	**1025.6102**	513.3087	**1008.5837**	504.7955	1007.5996	504.3035	9
15	1834.9738	917.9905	1817.9473	909.4773	1816.9632	908.9853	P	**912.5261**	**456.7667**	895.4996	**448.2534**	894.5156	**447.7614**	8
16	1936.0215	**968.5144**	1918.9949	960.0011	1918.0109	959.5091	T	815.4734	408.2403	798.4468	399.7271	797.4628	399.2350	7
17	2007.0586	**1004.0329**	1990.0320	995.5197	1989.0480	995.0277	A	**714.4257**	357.7165	697.3992	349.2032			6
18	2135.1172	**1068.0622**	2118.0906	**1059.5489**	2117.1066	**1059.0569**	Q	**643.3886**	322.1979	**626.3620**	**313.6847**			5
19	2248.2012	**1124.6043**	2231.1747	1116.0910	2230.1907	1115.5990	L	**515.3300**	258.1686	498.3035	249.6554			4
20	2347.2696	**1174.1385**	2330.2431	**1165.6252**	2329.2591	**1165.1332**	V	**402.2459**	201.6266	385.2194	193.1133			3
21	2475.3282	**1238.1678**	2458.3017	1229.6545	2457.3177	1229.1625	Q	**303.1775**	152.0924	286.1510	143.5791			2
22							R	175.1190	88.0631	158.0924	79.5498			1

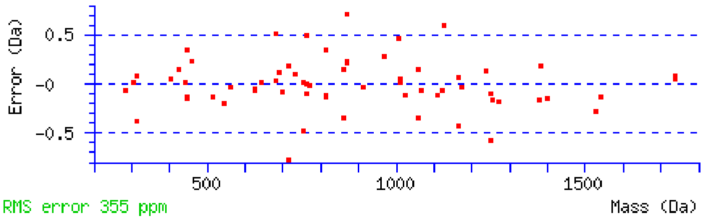

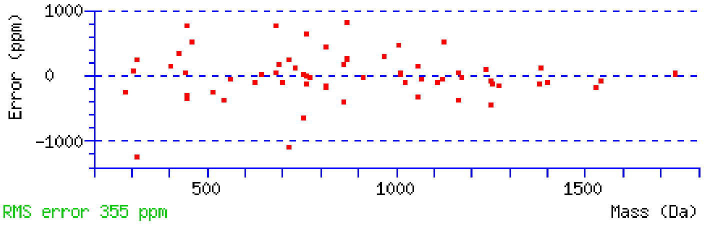

All matches to this query

Score	Mr(calc):	Delta	Sequence

60.1	2648.4326	0.0095	KLAQQYYLVYQEPIPTAQLVQR
9.4	2648.4320	0.0101	VTIAELGVQLVAGMSLSLQPHRADK
6.2	2647.4495	0.9926	ISYLMIALTVVGCIFHWLLRAR
4.7	2648.4262	0.0159	GSSSSLVVAGERSGDGLVLKLGLVLR
4.0	2647.4293	1.0128	GSSDVQELQRIGQLLIHLQEKEK
3.1	2646.4260	2.0161	FIFEAVGFIVSSVGLLLGIALVMR
3.1	2646.4260	2.0161	FIFEAVGFIVSSVGLLLGIALVMR
2.2	2648.4286	0.0135	NALTGILTQFEQIVAVFNASTRQK
1.3	2646.4397	2.0024	KFLILITDGEAQDIVKEPAVVLR
1.0	2648.4262	0.0159	GSSSSLVVAGERSGDGLVLKLGLVLR

Spectrum No: 3; Query: 29471; Rank: 1

Peptide View

MS/MS Fragmentation of **LAQQYYLVYQEPIPTAQLVQR**
Found in **IPI00219622**, Tax_Id=9606 Gene_Symbol=PSMA2 Proteasome subunit alpha type-2

Match to Query 29471: 2520.346932 from(841.122920,3+)
Title: 090324LimSK_Exosome1_B.14261.14261.3.dta
Data file C:\Temp\Mascoti\LSK_2D\090324LimSK_Exosome1_A_2D.mgf

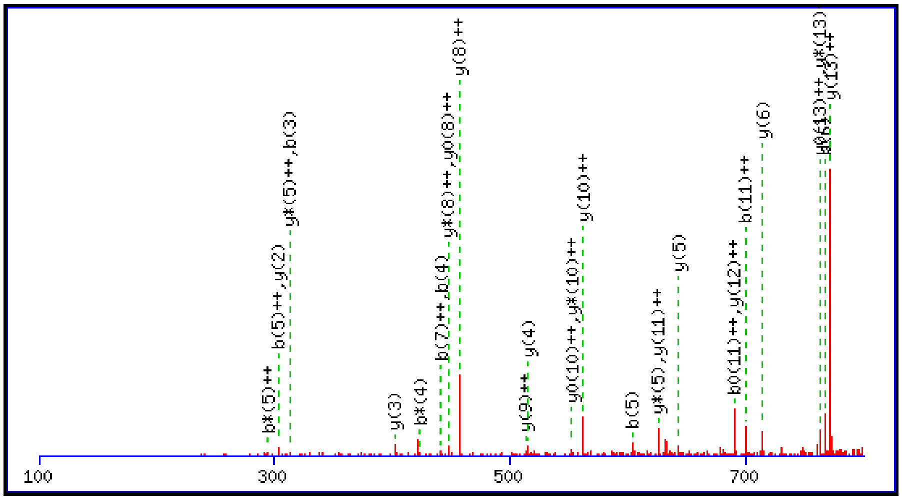

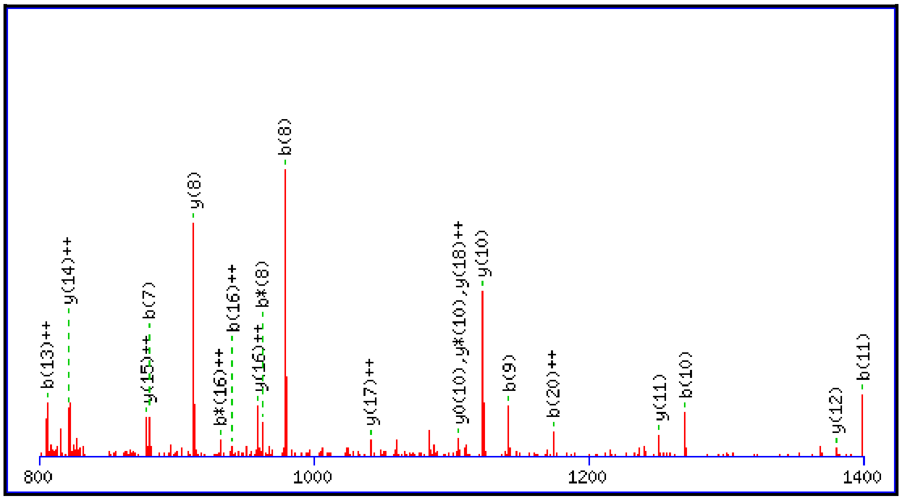

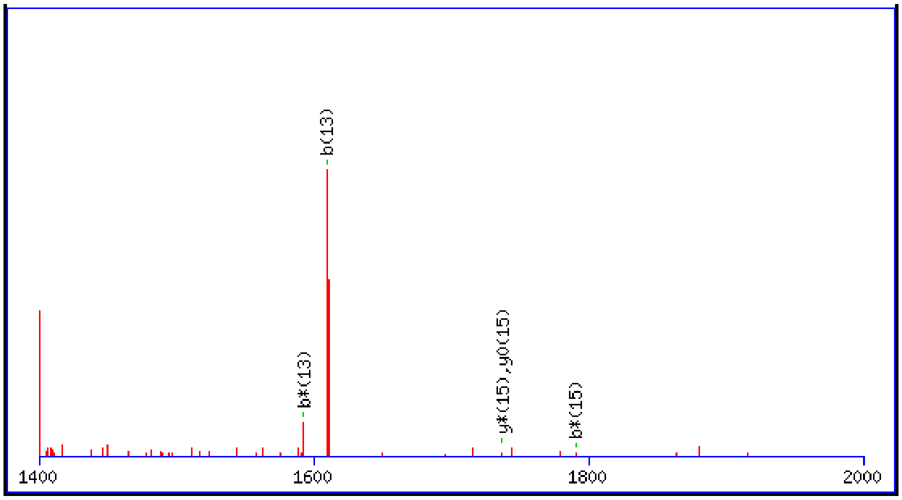

Monoisotopic mass of neutral peptide Mr(calc): 2520.3376
Fixed modifications: Carbamidomethyl (C)
Ions Score: 57 **Expect:** 0.00018
Matches (Bold Red): 55/204 fragment ions using 85 most intense peaks

#	b	b^{++}	b*	b^{*++}	b^{0}	b^{0++}	Seq.	y	y^{++}	y*	y^{*++}	y^{0}	y^{0++}	#
1	114.0913	57.5493					L							21
2	185.1285	93.0679					A	2408.2609	1204.6341	2391.2343	1196.1208	2390.2503	1195.6288	20
3	**313.1870**	157.0972	296.1605	148.5839			Q	2337.2238	1169.1155	2320.1972	1160.6022	2319.2132	1160.1102	19
4	**441.2456**	221.1264	**424.2191**	212.6132			Q	2209.1652	**1105.0862**	2192.1386	1096.5730	2191.1546	1096.0809	18
5	**604.3089**	**302.6581**	587.2824	**294.1448**			Y	2081.1066	**1041.0569**	2064.0801	1032.5437	2063.0960	1032.0517	17
6	**767.3723**	384.1898	750.3457	375.6765			Y	1918.0433	**959.5253**	1901.0167	951.0120	1900.0327	950.5200	16
7	**880.4563**	**440.7318**	863.4298	432.2185			L	1754.9799	**877.9936**	**1737.9534**	869.4803	**1736.9694**	868.9883	15
8	**979.5247**	490.2660	**962.4982**	481.7527			V	1641.8959	**821.4516**	1624.8693	812.9383	1623.8853	812.4463	14
9	**1142.5881**	571.7977	1125.5615	563.2844			Y	1542.8275	**771.9174**	1525.8009	**763.4041**	1524.8169	**762.9121**	13
10	**1270.6467**	635.8270	1253.6201	627.3137			Q	**1379.7641**	**690.3857**	1362.7376	681.8724	1361.7536	681.3804	12
11	**1399.6892**	**700.3483**	1382.6627	691.8350	1381.6787	**691.3430**	E	**1251.7056**	**626.3564**	1234.6790	617.8431	1233.6950	617.3511	11
12	1496.7420	748.8746	1479.7155	740.3614	1478.7314	739.8694	P	**1122.6630**	**561.8351**	**1105.6364**	**553.3218**	**1104.6524**	**552.8298**	10
13	**1609.8261**	**805.4167**	**1592.7995**	796.9034	1591.8155	796.4114	I	1025.6102	**513.3087**	1008.5837	504.7955	1007.5996	504.3035	9
14	1706.8788	853.9431	1689.8523	845.4298	1688.8683	844.9378	P	**912.5261**	**456.7667**	895.4996	**448.2534**	894.5156	**447.7614**	8
15	1807.9265	904.4669	**1790.9000**	895.9536	1789.9160	895.4616	T	815.4734	408.2403	798.4468	399.7271	797.4628	399.2350	7
16	1878.9636	**939.9855**	1861.9371	**931.4722**	1860.9531	930.9802	A	**714.4257**	357.7165	697.3992	349.2032			6
17	2007.0222	1004.0147	1989.9957	995.5015	1989.0116	995.0095	Q	**643.3886**	322.1979	**626.3620**	**313.6847**			5
18	2120.1063	1060.5568	2103.0797	1052.0435	2102.0957	1051.5515	L	**515.3300**	258.1686	498.3035	249.6554			4
19	2219.1747	1110.0910	2202.1481	1101.5777	2201.1641	1101.0857	V	**402.2459**	201.6266	385.2194	193.1133			3
20	2347.2333	**1174.1203**	2330.2067	1165.6070	2329.2227	1165.1150	Q	**303.1775**	152.0924	286.1510	143.5791			2
21							R	175.1190	88.0631	158.0924	79.5498			1

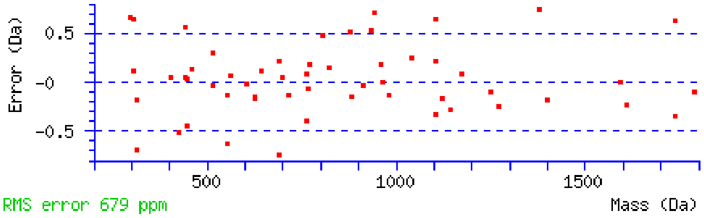

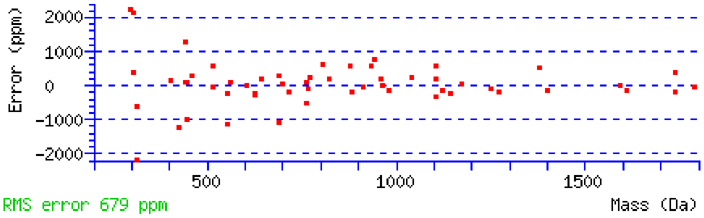

All matches to this query

Score	Mr(calc):	Delta	Sequence
57.0	2520.3376	0.0093	LAQQYYLVYQEPIPTAQLVQR

11.6	2519.3400	1.0069	DLAVVLETAPILTALDIFVDRR
11.2	2518.3501	1.9968	WQLALAVGAPLLLGAGAIYLWSR
10.9	2519.3486	0.9984	VPGRKPAPGLSVGAQITTNPAPRR
5.3	2519.3458	1.0012	TRYTALVTLMTQYIKFAGDSLK
4.2	2520.3457	0.0012	YLAIFDAFITPFLNPVIYTFR
1.7	2520.3477	-0.0008	KTLPSMLILSGLTAGMLMTDAGRK
0.6	2519.3434	1.0035	LVATAMKLHGVIDILSLSITNSK
0.4	2520.3353	0.0117	TVILVKNLPAGTLAAELQETFGR
0.4	2520.3376	0.0093	SKLPSLWQFVTSAPGNQYKELK

Spectrum No: 4; Query: 12592; Rank: 1

Peptide View

MS/MS Fragmentation of **LTPTEVKDYLAAIA**
Found in **IPI00219622**, Tax_Id=9606 Gene_Symbol=PSMA2 Proteasome subunit alpha type-2

Match to Query 12592: 1503.821768 from(752.918160,2+)
Title: 090324LimSK_Exosome1_A.16279.16279.2.dta
Data file C:\Temp\Mascoti\LSK_2D\090324LimSK_Exosome1_A_2D.mgf

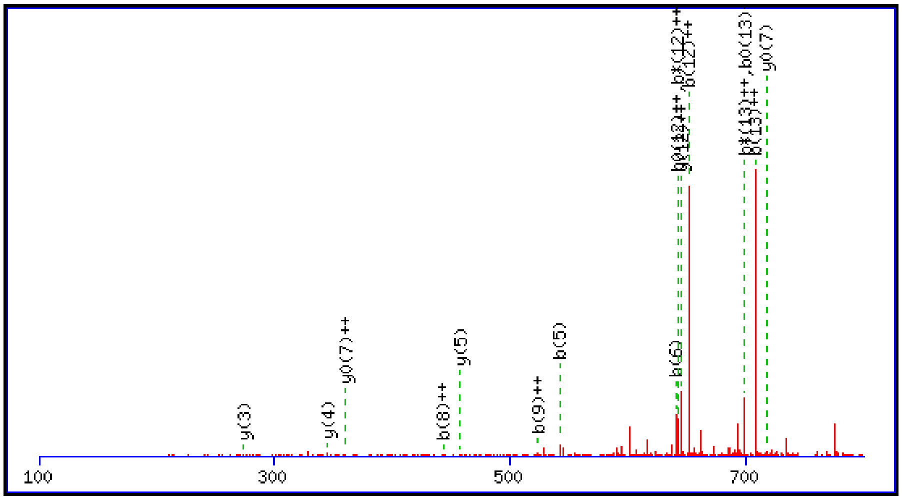

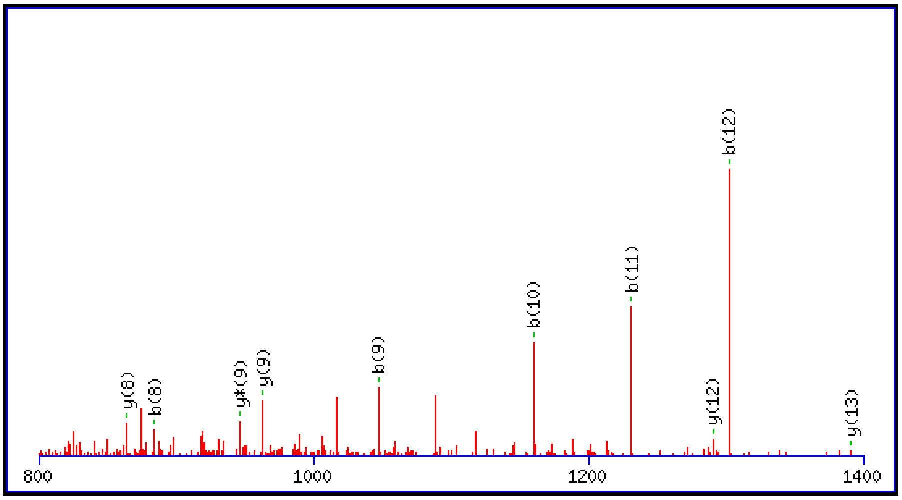

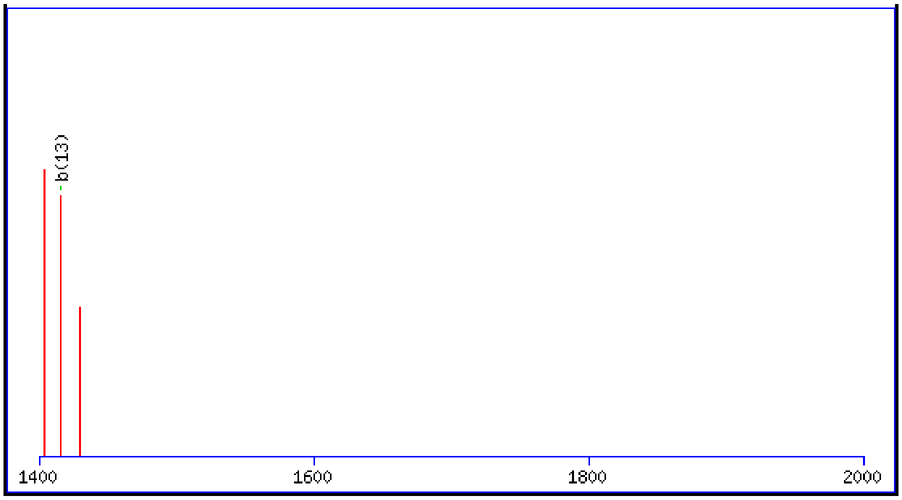

Monoisotopic mass of neutral peptide Mr(calc): 1503.8184
Fixed modifications: Carbamidomethyl (C)
Ions Score: 40 **Expect:** 0.012
Matches (Bold Red): 28/116 fragment ions using 61 most intense peaks

#	b	b++	b*	b*++	b0	b0++	Seq.	y	y++	y*	y*++	y0	y0++	#
1	114.0913	57.5493					L							14
2	215.1390	108.0731			197.1285	99.0679	T	**1391.7417**	696.3745	1374.7151	687.8612	1373.7311	687.3692	13
3	312.1918	156.5995			294.1812	147.5942	P	**1290.6940**	**645.8506**	1273.6674	637.3374	1272.6834	636.8454	12
4	413.2395	207.1234			395.2289	198.1181	T	1193.6412	597.3243	1176.6147	588.8110	1175.6307	588.3190	11
5	**542.2821**	271.6447			**524.2715**	262.6394	E	1092.5936	546.8004	1075.5670	538.2871	1074.5830	537.7951	10
6	**641.3505**	321.1789			623.3399	312.1736	V	**963.5510**	482.2791	**946.5244**	473.7658	945.5404	473.2738	9
7	769.4454	385.2264	752.4189	376.7131	751.4349	376.2211	K	**864.4825**	432.7449	847.4560	424.2316	846.4720	423.7396	8
8	**884.4724**	**442.7398**	867.4458	434.2266	866.4618	433.7345	D	736.3876	368.6974			**718.3770**	**359.6921**	7
9	**1047.5357**	**524.2715**	1030.5092	515.7582	1029.5251	515.2662	Y	621.3606	311.1840					6
10	**1160.6198**	580.8135	1143.5932	572.3002	1142.6092	571.8082	L	**458.2973**	229.6523					5
11	**1231.6569**	616.3321	1214.6303	607.8188	1213.6463	607.3268	A	**345.2132**	173.1103					4
12	**1302.6940**	**651.8506**	1285.6674	**643.3374**	1284.6834	**642.8454**	A	**274.1761**	137.5917					3
13	**1415.7781**	**708.3927**	1398.7515	**699.8794**	1397.7675	**699.3874**	I	203.1390	102.0731					2
14							A	90.0550	45.5311					1

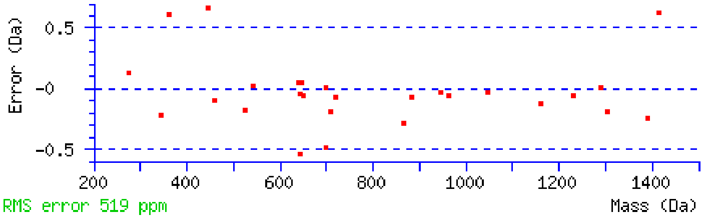

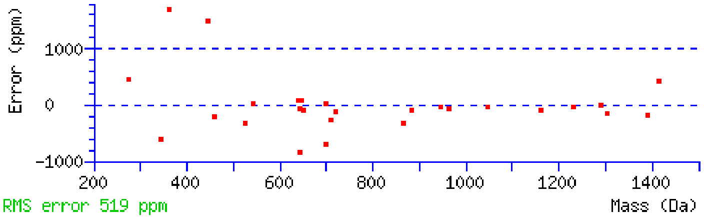

All matches to this query

Score	Mr(calc):	Delta	Sequence
39.7	1503.8184	0.0033	LTPTEVKDYLAAIA
7.5	1502.8239	0.9979	SNLREVAQMLKSK
3.4	1502.8052	1.0165	SLNANTDITSLARK
2.2	1501.8286	1.9932	SNIREIEKLCLK
2.1	1501.8075	2.0143	ASLALCAFVAVPQR
2.1	1501.8293	1.9925	SPSSLLSLFLPWR
1.9	1502.8066	1.0152	SLTGARWQSRSVR
1.8	1502.8286	0.9932	SFLKNIFNVFFK

0.9	1501.8018	2.0200	SIKPIQPASQVVR
0.6	1501.8212	2.0005	SLSNSPQLKSRTGK

Spectrum No: 5; Query: 22429; Rank: 1

Peptide View

MS/MS Fragmentation of **LVQIEYALAAVAGGAPSVGIK**
Found in **IPI00219622**, Tax_Id=9606 Gene_Symbol=PSMA2 Proteasome subunit alpha type-2

Match to Query 22429: 2026.148292 from(676.390040,3+)
Title: 090324LimSK_Exosome1_A.17718.17718.3.dta
Data file C:\Temp\Mascoti\LSK_2D\090324LimSK_Exosome1_A_2D.mgf

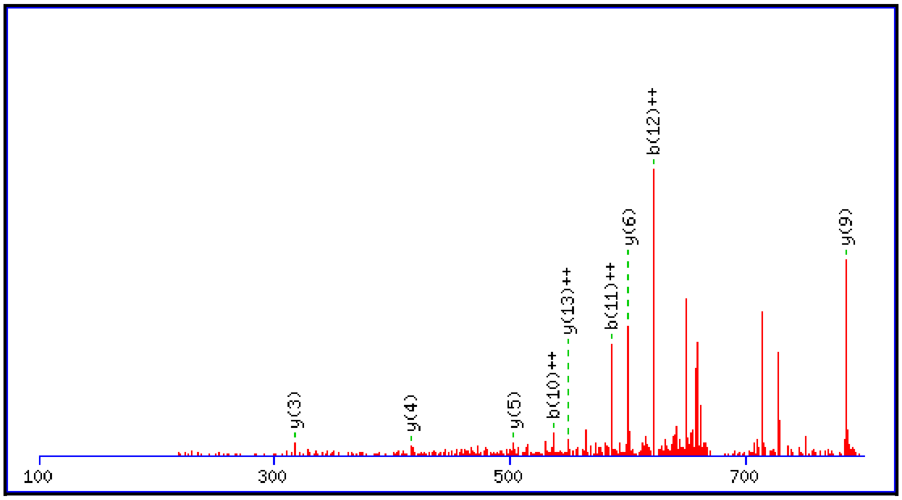

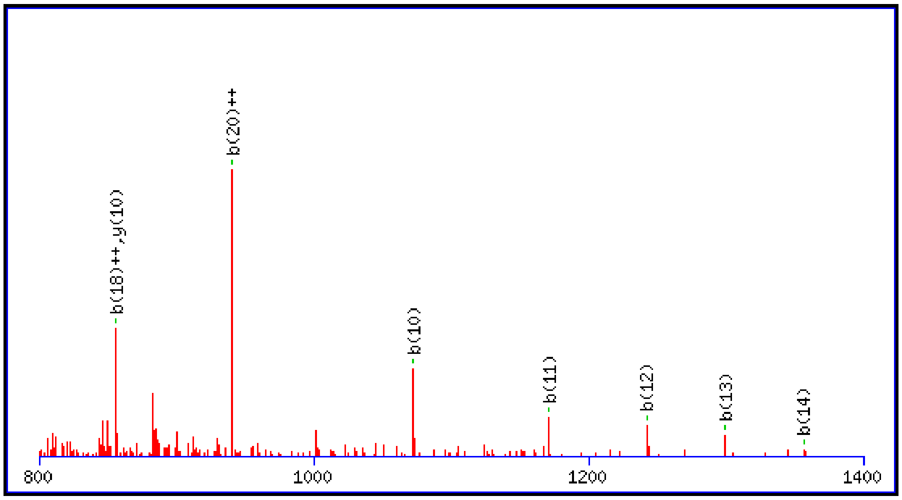

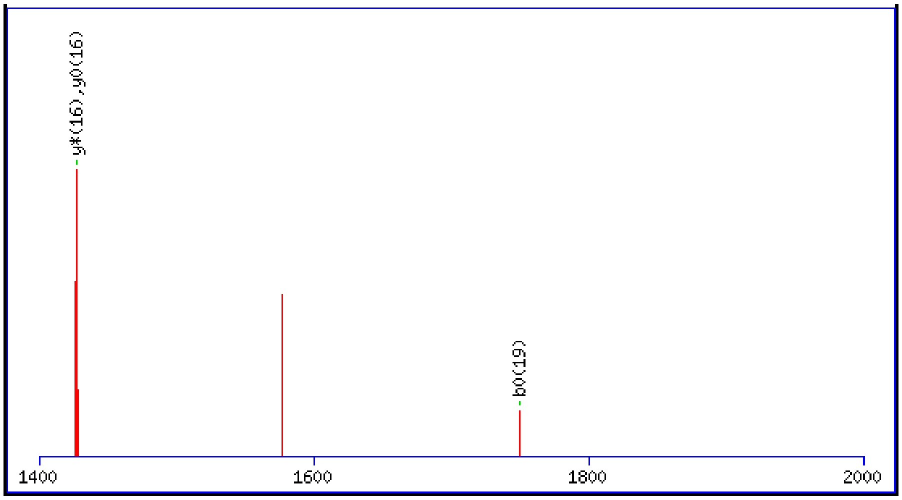

Monoisotopic mass of neutral peptide Mr(calc): 2026.1463
Fixed modifications: Carbamidomethyl (C)
Ions Score: 54 **Expect:** 0.00014
Matches (Bold Red): 21/220 fragment ions using 22 most intense peaks

#	b	b^{++}	b*	b^{*++}	b^{0}	b^{0++}	Seq.	y	y^{++}	y*	y^{*++}	y^{0}	y^{0++}	#
1	114.0913	57.5493					L							21
2	213.1598	107.0835					V	1914.0695	957.5384	1897.0429	949.0251	1896.0589	948.5331	20
3	341.2183	171.1128	324.1918	162.5995			Q	1815.0011	908.0042	1797.9745	899.4909	1796.9905	898.9989	19
4	454.3024	227.6548	437.2758	219.1416			I	1686.9425	843.9749	1669.9160	835.4616	1668.9319	834.9696	18
5	583.3450	292.1761	566.3184	283.6629	565.3344	283.1709	E	1573.8584	787.4329	1556.8319	778.9196	1555.8479	778.4276	17
6	746.4083	373.7078	729.3818	365.1945	728.3978	364.7025	Y	1444.8158	722.9116	**1427.7893**	714.3983	**1426.8053**	713.9063	16
7	817.4454	409.2264	800.4189	400.7131	799.4349	400.2211	A	1281.7525	641.3799	1264.7260	632.8666	1263.7419	632.3746	15
8	930.5295	465.7684	913.5029	457.2551	912.5189	456.7631	L	1210.7154	605.8613	1193.6889	597.3481	1192.7048	596.8561	14
9	1001.5666	501.2869	984.5401	492.7737	983.5560	492.2817	A	1097.6313	**549.3193**	1080.6048	540.8060	1079.6208	540.3140	13
10	**1072.6037**	**536.8055**	1055.5772	528.2922	1054.5932	527.8002	A	1026.5942	513.8007	1009.5677	505.2875	1008.5837	504.7955	12
11	**1171.6721**	**586.3397**	1154.6456	577.8264	1153.6616	577.3344	V	955.5571	478.2822	938.5306	469.7689	937.5465	469.2769	11
12	**1242.7093**	**621.8583**	1225.6827	613.3450	1224.6987	612.8530	A	**856.4887**	428.7480	839.4621	420.2347	838.4781	419.7427	10
13	**1299.7307**	650.3690	1282.7042	641.8557	1281.7202	641.3637	G	**785.4516**	393.2294	768.4250	384.7162	767.4410	384.2241	9
14	**1356.7522**	678.8797	1339.7256	670.3665	1338.7416	669.8744	G	728.4301	364.7187	711.4036	356.2054	710.4196	355.7134	8
15	**1427.7893**	714.3983	1410.7627	705.8850	1409.7787	705.3930	A	671.4087	336.2080	654.3821	327.6947	653.3981	327.2027	7
16	1524.8421	762.9247	1507.8155	754.4114	1506.8315	753.9194	P	**600.3715**	300.6894	583.3450	292.1761	582.3610	291.6841	6
17	1611.8741	806.4407	1594.8475	797.9274	1593.8635	797.4354	S	**503.3188**	252.1630	486.2922	243.6498	485.3082	243.1577	5
18	1710.9425	**855.9749**	1693.9160	847.4616	1692.9319	846.9696	V	**416.2867**	208.6470	399.2602	200.1337			4
19	1767.9640	884.4856	1750.9374	875.9723	**1749.9534**	875.4803	G	**317.2183**	159.1128	300.1918	150.5995			3
20	1881.0480	**941.0277**	1864.0215	932.5144	1863.0375	932.0224	I	260.1969	130.6021	243.1703	122.0888			2
21							K	147.1128	74.0600	130.0863	65.5468			1

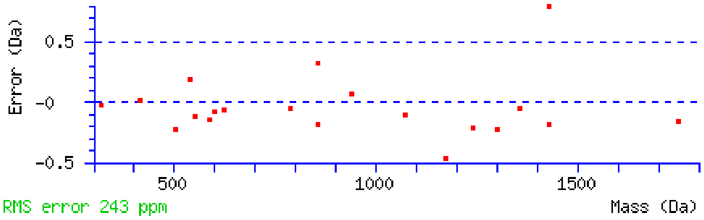

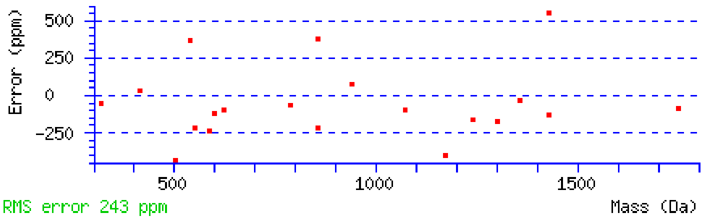

All matches to this query

Score	Mr(calc):	Delta	Sequence
54.5	2026.1463	0.0020	LVQIEYALAAVAGGAPSVGIK

13.3	2026.1649	-0.0166	ELIMIAGGNQSILLWIKK
2.6	2026.1384	0.0099	AEKDLTLLIAMEPLINVK
2.1	2025.1443	1.0040	GSTAVGIRGLTADARVVINR
1.2	2026.1310	0.0173	ELKTQVEDLNENLLKLK
1.2	2026.1438	0.0045	LPSLPLPHGCLQLPLPFK
0.7	2026.1414	0.0069	LTLLPAVVMHLKKQDLK
0.5	2026.1310	0.0173	IEETNPSLAQDTVIIKKK
0.2	2025.1622	0.9860	VATALTLLRFELLPDPTR
0.0	2024.1517	1.9966	ELQVKLLELQEVDSLLR

Spectrum No: 6; Query: 10308; Rank: 1

Peptide View

MS/MS Fragmentation of **FILNLPTFSVR**
Found in **IPI00028006**, Tax_Id=9606 Gene_Symbol=PSMB2 Proteasome subunit beta type-2

Match to Query 10308: 1305.746668 from(653.880610,2+)
Title: 090324LimSK_Exosome1_B.17094.17094.2.dta
Data file C:\Temp\Mascoti\LSK_2D\090324LimSK_Exosome1_A_2D.mgf

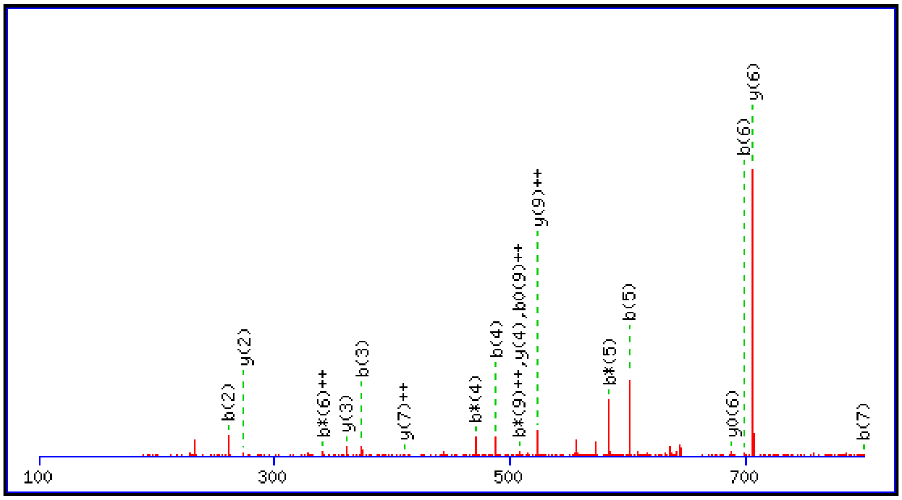

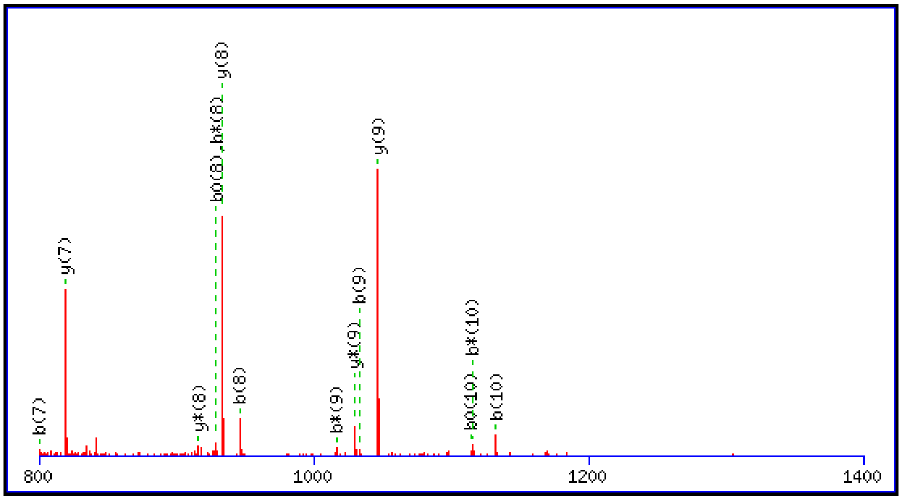

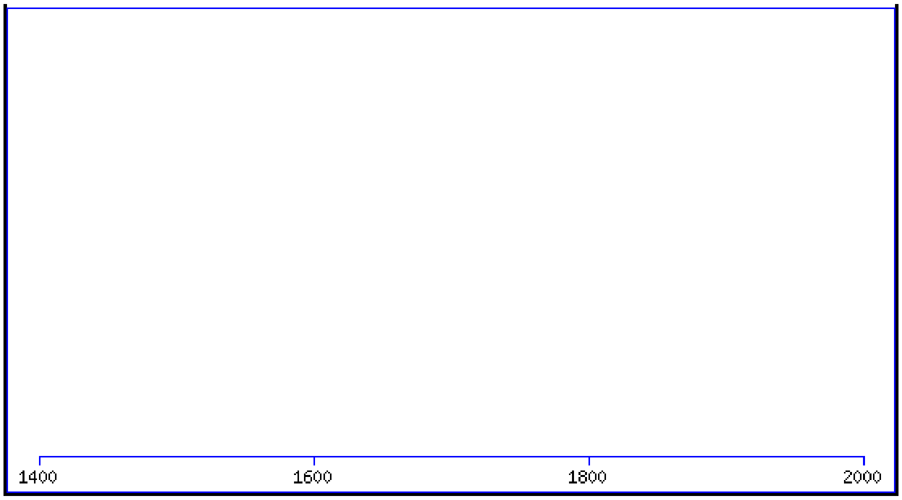

Monoisotopic mass of neutral peptide Mr(calc): 1305.7445
Fixed modifications: Carbamidomethyl (C)
Ions Score: 52 **Expect:** 0.00037
Matches (Bold Red): 31/98 fragment ions using 64 most intense peaks

#	b	b^{++}	b*	b^{*++}	b^{0}	b^{0++}	Seq.	y	y^{++}	y*	y^{*++}	y^{0}	y^{0++}	#
1	148.0757	74.5415					F							11
2	**261.1598**	131.0835					I	1159.6834	580.3453	1142.6568	571.8320	1141.6728	571.3400	10
3	**374.2438**	187.6255					L	**1046.5993**	**523.8033**	**1029.5728**	515.2900	1028.5887	514.7980	9
4	**488.2867**	244.6470	**471.2602**	236.1337			N	**933.5152**	467.2613	**916.4887**	458.7480	915.5047	458.2560	8
5	**601.3708**	301.1890	**584.3443**	292.6758			L	**819.4723**	**410.2398**	802.4458	401.7265	801.4617	401.2345	7
6	**698.4236**	349.7154	681.3970	**341.2022**			P	**706.3883**	353.6978	689.3617	345.1845	**688.3777**	344.6925	6
7	**799.4713**	400.2393	782.4447	391.7260	781.4607	391.2340	T	609.3355	305.1714	592.3089	296.6581	591.3249	296.1661	5
8	**946.5397**	473.7735	**929.5131**	465.2602	**928.5291**	464.7682	F	**508.2878**	254.6475	491.2613	246.1343	490.2772	245.6423	4
9	**1033.5717**	517.2895	**1016.5451**	**508.7762**	1015.5611	**508.2842**	S	**361.2194**	181.1133	344.1928	172.6001	343.2088	172.1081	3
10	**1132.6401**	566.8237	**1115.6136**	558.3104	**1114.6295**	557.8184	V	**274.1874**	137.5973	257.1608	129.0840			2
11							R	175.1190	88.0631	158.0924	79.5498			1

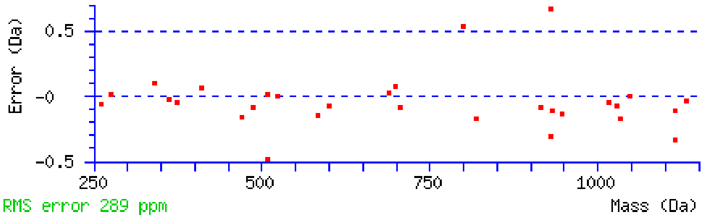

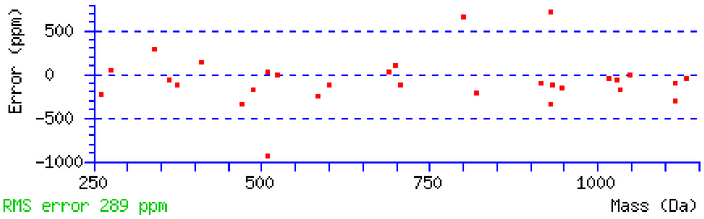

All matches to this query

Score	Mr(calc):	Delta	Sequence
52.2	1305.7445	0.0022	FILNLPTFSVR
10.5	1305.7517	-0.0051	NAGQVVHLTLVR
8.1	1305.7478	-0.0012	RIYLCLDLLK
5.3	1304.7452	1.0015	IHETQPLLNLK
5.0	1305.7478	-0.0012	MLEFVSLLARK
3.9	1304.7340	1.0127	QVFEGLTTGLLK
2.5	1305.7517	-0.0050	QAPIPAELRGVR
2.5	1305.7517	-0.0050	HRAAEAAINILK
2.3	1303.7500	1.9967	TLEGFRLLVEK
2.2	1305.7378	0.0089	RNHNLALGRQK

Spectrum No: 7; Query: 20562; Rank: 1

Peptide View

MS/MS Fragmentation of **IIDKNGIHDLDNISFPK**
Found in **IPI00028006**, Tax_Id=9606 Gene_Symbol=PSMB2 Proteasome subunit beta type-2

Match to Query 20562: 1938.026892 from(647.016240,3+)
Title: 090324LimSK_Exosome1_A.10059.10059.3.dta
Data file C:\Temp\Mascoti\LSK_2D\090324LimSK_Exosome1_A_2D.mgf

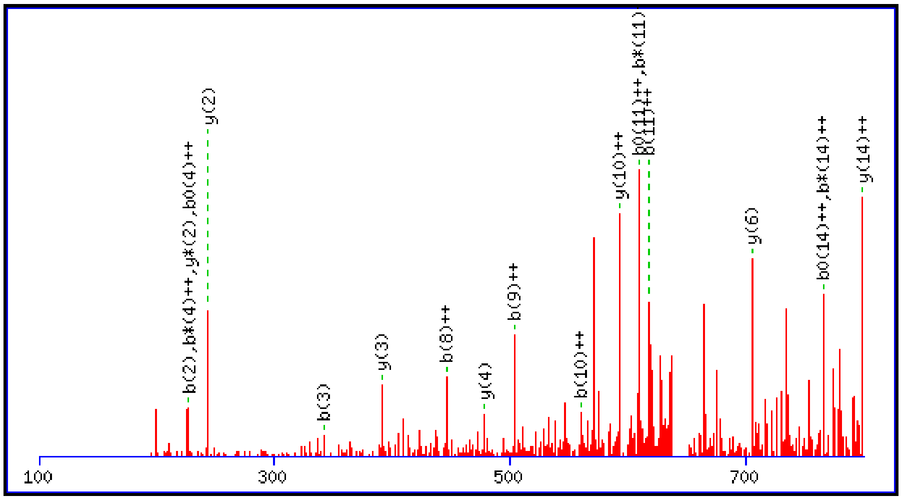

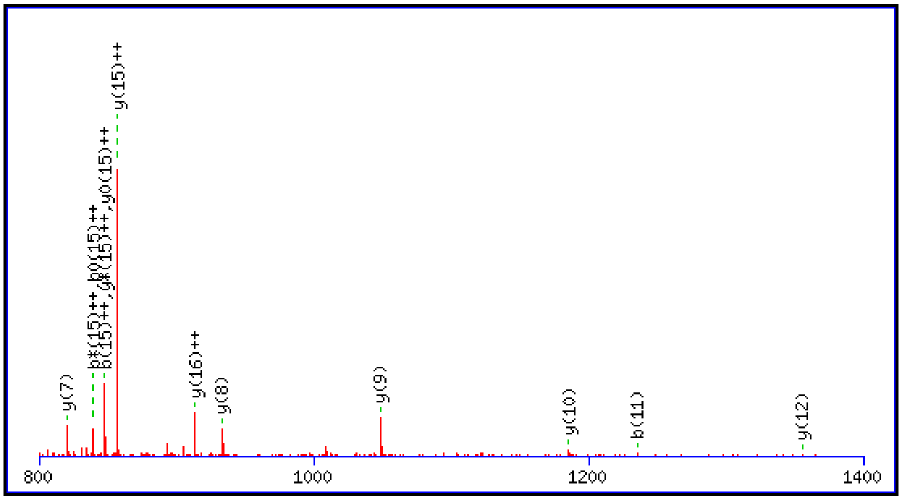

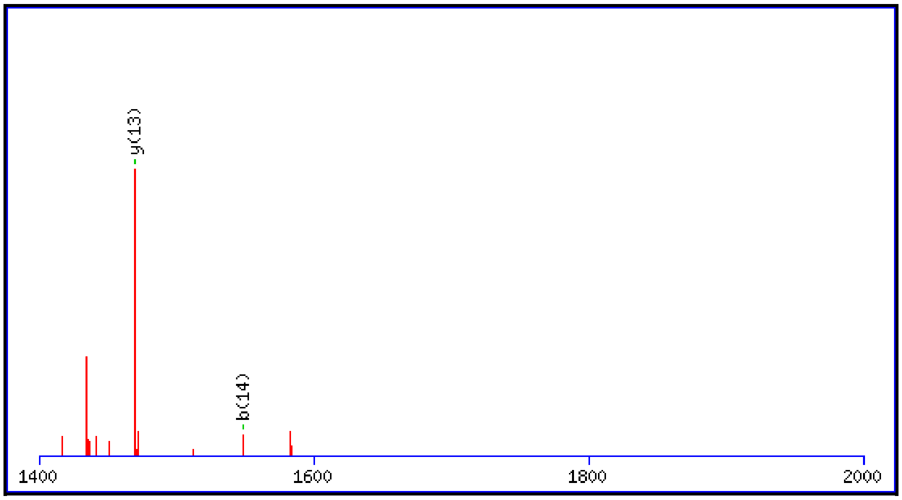

Monoisotopic mass of neutral peptide Mr(calc): 1938.0211

Fixed modifications: Carbamidomethyl (C)
Ions Score: 68 **Expect:** 1.8e-005
Matches (Bold Red): 34/176 fragment ions using 38 most intense peaks

#	b	b^{++}	b*	b*$^{++}$	b^{0}	b^{0++}	Seq.	y	y^{++}	y*	y*$^{++}$	y^{0}	y^{0++}	#
1	114.0913	57.5493					I							17
2	**227.1754**	114.0913					I	1825.9443	**913.4758**	1808.9177	904.9625	1807.9337	904.4705	16
3	**342.2023**	171.6048			324.1918	162.5995	D	1712.8602	**856.9338**	1695.8337	**848.4205**	1694.8497	**847.9285**	15
4	470.2973	235.6523	453.2708	**227.1390**	452.2867	**226.6470**	K	1597.8333	**799.4203**	1580.8067	790.9070	1579.8227	790.4150	14
5	584.3402	292.6738	567.3137	284.1605	566.3297	283.6685	N	**1469.7383**	735.3728	1452.7118	726.8595	1451.7278	726.3675	13
6	641.3617	321.1845	624.3352	312.6712	623.3511	312.1792	G	**1355.6954**	678.3513	1338.6688	669.8381	1337.6848	669.3461	12
7	754.4458	377.7265	737.4192	369.2132	736.4352	368.7212	I	1298.6739	649.8406	1281.6474	641.3273	1280.6634	640.8353	11
8	891.5047	**446.2560**	874.4781	437.7427	873.4941	437.2507	H	**1185.5899**	**593.2986**	1168.5633	584.7853	1167.5793	584.2933	10
9	1006.5316	**503.7694**	989.5051	495.2562	988.5211	494.7642	D	**1048.5310**	524.7691	1031.5044	516.2558	1030.5204	515.7638	9
10	1119.6157	**560.3115**	1102.5891	551.7982	1101.6051	551.3062	L	**933.5040**	467.2556	916.4775	458.7424	915.4934	458.2504	8
11	**1234.6426**	**617.8250**	1217.6161	**609.3117**	1216.6321	**608.8197**	D	**820.4199**	410.7136	803.3934	402.2003	802.4094	401.7083	7
12	1348.6856	674.8464	1331.6590	666.3331	1330.6750	665.8411	N	**705.3930**	353.2001	688.3665	344.6869	687.3824	344.1949	6
13	1461.7696	731.3884	1444.7431	722.8752	1443.7591	722.3832	I	591.3501	296.1787	574.3235	287.6654	573.3395	287.1734	5
14	**1548.8016**	774.9045	1531.7751	**766.3912**	1530.7911	**765.8992**	S	**478.2660**	239.6366	461.2395	231.1234	460.2554	230.6314	4
15	1695.8701	**848.4387**	1678.8435	**839.9254**	1677.8595	**839.4334**	F	**391.2340**	196.1206	374.2074	187.6074			3
16	1792.9228	896.9651	1775.8963	888.4518	1774.9123	887.9598	P	**244.1656**	122.5864	**227.1390**	114.0731			2
17							K	147.1128	74.0600	130.0863	65.5468			1

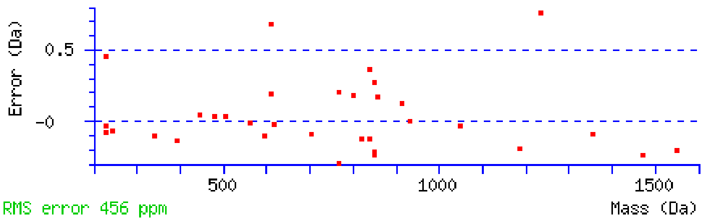

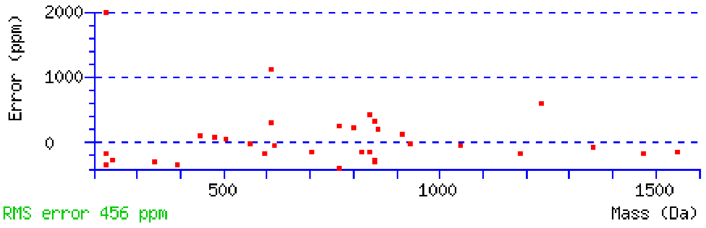

All matches to this query

Score	Mr(calc):	Delta	Sequence
68.1	1938.0211	0.0058	IIDKNGIHDLDNISFPK
9.7	1937.0148	1.0121	LLMLKQQITMEMSALR
7.1	1937.0135	1.0134	KYLSLTERSQIAHALK
6.2	1937.0365	0.9903	LLNSTAVKALFMMEKLE
4.8	1937.0148	1.0121	LLMLKQQITMEMSALR
2.3	1937.0421	0.9848	GALEISQTLLGIIVRMK
2.3	1938.0274	-0.0005	LLRMYDLNKPKARPK
2.2	1937.0321	0.9948	LILNSKLWAQMKIQR
2.2	1937.0119	1.0150	LIWEQIHQERDQSKK
2.0	1938.0283	-0.0014	QVZLVZSGGGAVZPGRSLR

Spectrum No: 8; Query: 14367; Rank: 1

Peptide View

MS/MS Fragmentation of **NGYELSPTAAANFTR**
Found in **IPI00028006**, Tax_Id=9606 Gene_Symbol=PSMB2 Proteasome subunit beta type-2

Match to Query 14367: 1610.777828 from(806.396190,2+)

Title: 090324LimSK_Exosome1_A.8075.8075.2.dta
Data file C:\Temp\Mascoti\LSK_2D\090324LimSK_Exosome1_A_2D.mgf

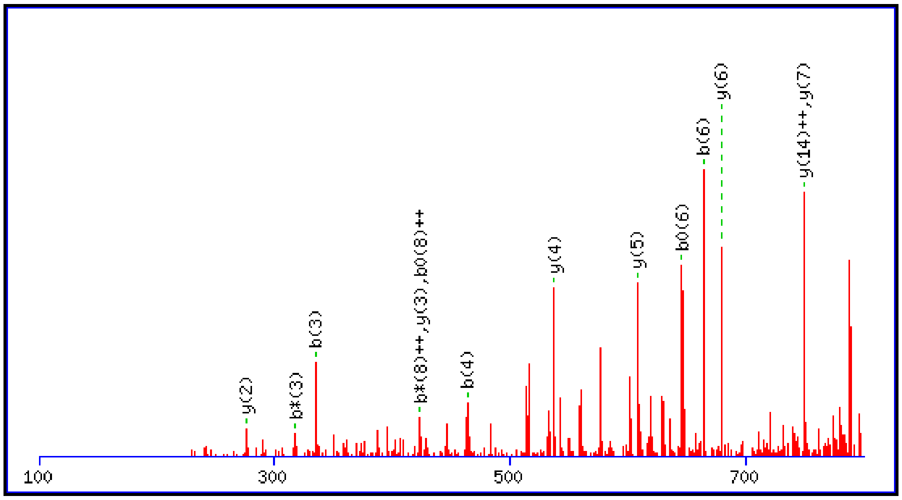

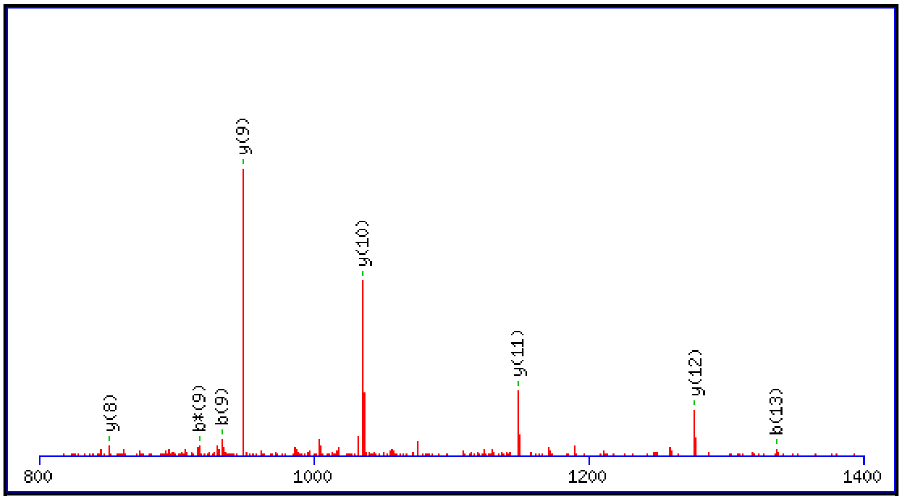

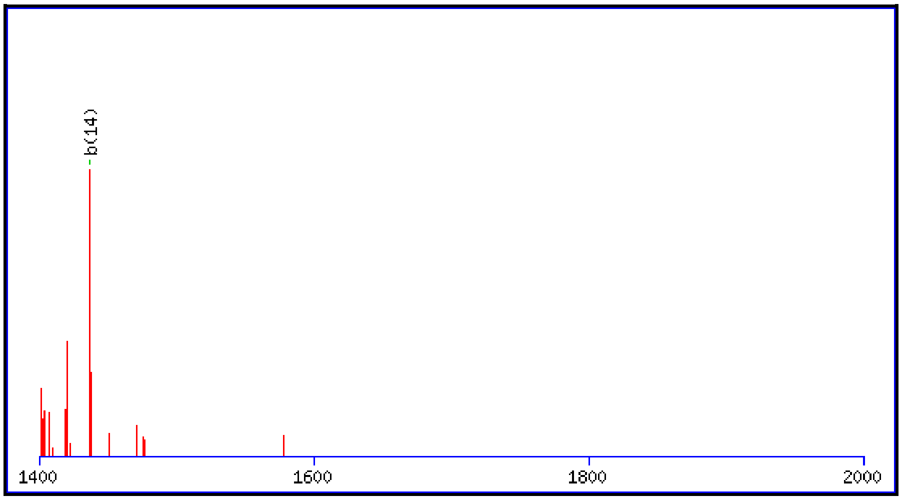

Monoisotopic mass of neutral peptide Mr(calc): 1610.7689
Fixed modifications: Carbamidomethyl (C)
Ions Score: 91 **Expect:** 1.5e-007
Matches (Bold Red): 23/160 fragment ions using 33 most intense peaks

#	b	b^{++}	b*	b^{*++}	b^0	b^{0++}	Seq.	y	y^{++}	y*	y^{*++}	y^0	y^{0++}	#
1	115.0502	58.0287	98.0237	49.5155			N							15
2	172.0717	86.5395	155.0451	78.0262			G	1497.7332	**749.3703**	1480.7067	740.8570	1479.7227	740.3650	14
3	**335.1350**	168.0711	**318.1084**	159.5579			Y	1440.7118	720.8595	1423.6852	712.3462	1422.7012	711.8542	13
4	**464.1776**	232.5924	447.1510	224.0792	446.1670	223.5872	E	**1277.6484**	639.3279	1260.6219	630.8146	1259.6379	630.3226	12

**5**	577.2617	289.1345	560.2351	280.6212	559.2511	280.1292	**L**	**1148.6058**	574.8066	1131.5793	566.2933	1130.5953	565.8013	**11**
**6**	**664.2937**	332.6505	647.2671	324.1372	**646.2831**	323.6452	**S**	**1035.5218**	518.2645	1018.4952	509.7513	1017.5112	509.2592	**10**
**7**	761.3464	381.1769	744.3199	372.6636	743.3359	372.1716	**P**	**948.4898**	474.7485	931.4632	466.2352	930.4792	465.7432	**9**
**8**	862.3941	431.7007	845.3676	**423.1874**	844.3836	**422.6954**	**T**	**851.4370**	426.2221	834.4104	417.7089	833.4264	417.2169	**8**
**9**	**933.4312**	467.2193	**916.4047**	458.7060	915.4207	458.2140	**A**	**750.3893**	375.6983	733.3628	367.1850	732.3787	366.6930	**7**
**10**	1004.4684	502.7378	987.4418	494.2245	986.4578	493.7325	**A**	**679.3522**	340.1797	662.3257	331.6665	661.3416	331.1745	**6**
**11**	1075.5055	538.2564	1058.4789	529.7431	1057.4949	529.2511	**A**	**608.3151**	304.6612	591.2885	296.1479	590.3045	295.6559	**5**
**12**	1189.5484	595.2778	1172.5218	586.7646	1171.5378	586.2726	**N**	**537.2780**	269.1426	520.2514	260.6293	519.2674	260.1373	**4**
**13**	**1336.6168**	668.8120	1319.5903	660.2988	1318.6062	659.8068	**F**	**423.2350**	212.1212	406.2085	203.6079	405.2245	203.1159	**3**
**14**	**1437.6645**	719.3359	1420.6379	710.8226	1419.6539	710.3306	**T**	**276.1666**	138.5870	259.1401	130.0737	258.1561	129.5817	**2**
**15**							**R**	175.1190	88.0631	158.0924	79.5498			**1**

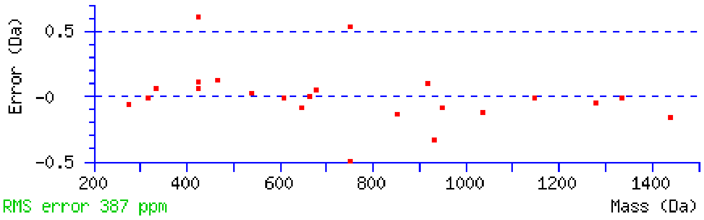

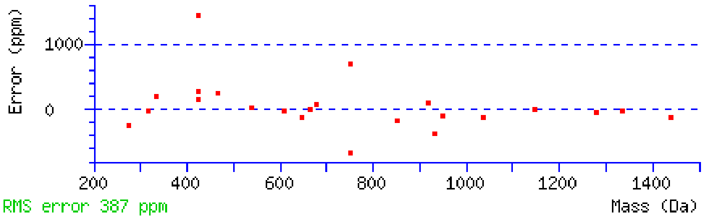

All matches to this query

Score	Mr(calc):	Delta	Sequence
91.4	1610.7689	0.0090	NGYELSPTAAANFTR
6.4	1610.7851	-0.0073	LRKGGATALMDAAEK
3.6	1610.7900	-0.0121	SSGSEESILDLRYR
1.0	1610.7665	0.0113	LKESQTQDNITVR
1.0	1610.7678	0.0100	IRQQQEATPPAHR
0.8	1610.7804	-0.0026	LTTALQKLEEEEK
0.8	1610.7804	-0.0026	LTTALQKLEEEEK
0.5	1608.7872	1.9906	EKRVTVLEQNGEK
0.0	1608.7752	2.0026	KMPYKCNPGGNSLK

Spectrum No: 9; Query: 17295; Rank: 1

Peptide View

MS/MS Fragmentation of **NGYELSPTAAANFTRR**
Found in **IPI00028006**, Tax_Id=9606 Gene_Symbol=PSMB2 Proteasome subunit beta type-2

Match to Query 17295: 1766.874582 from(589.965470,3+)
Title: 090324LimSK_Exosome1_B.6881.6881.3.dta
Data file C:\Temp\Mascoti\LSK_2D\090324LimSK_Exosome1_A_2D.mgf

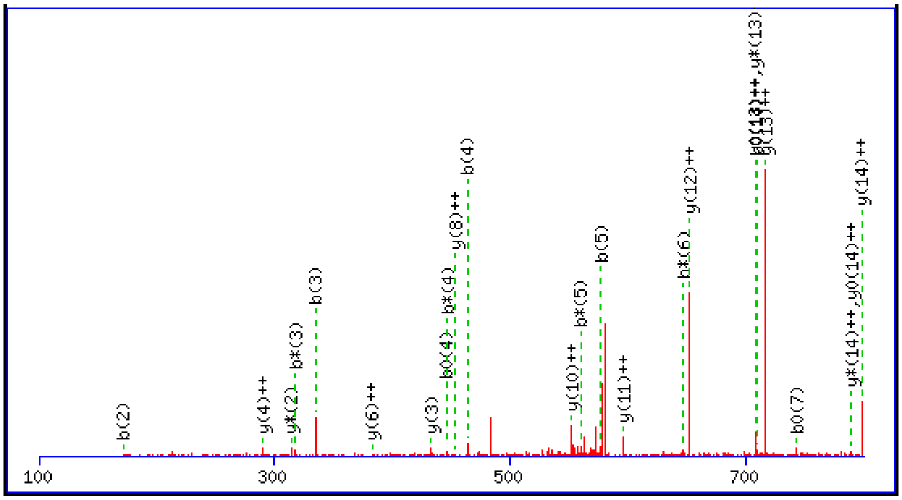

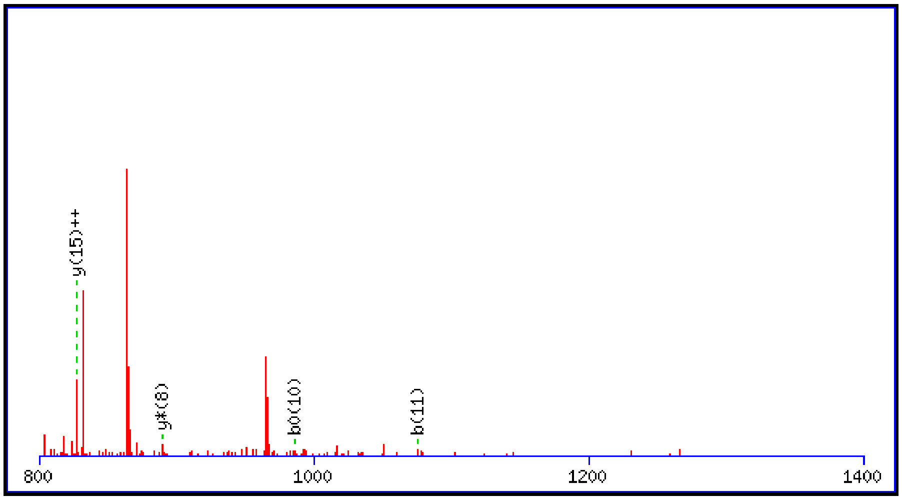

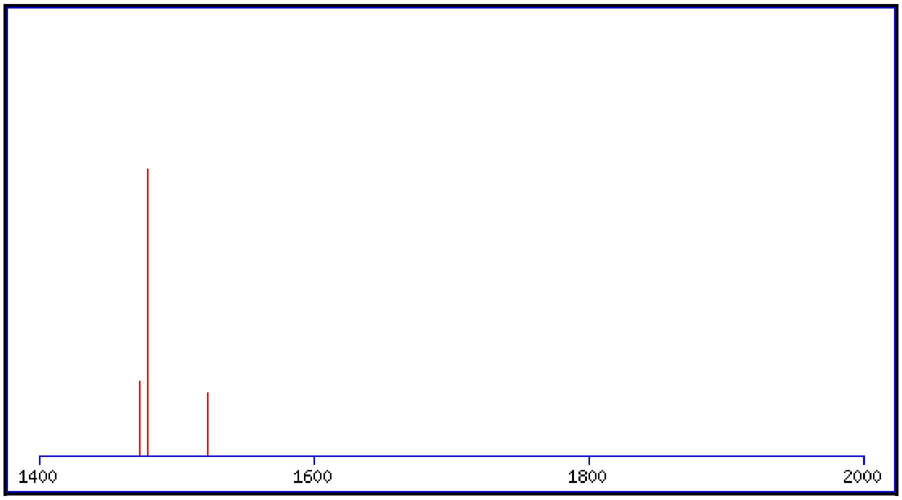

Monoisotopic mass of neutral peptide Mr(calc): 1766.8700
Fixed modifications: Carbamidomethyl (C)
Ions Score: 22 **Expect:** 1.5
Matches (Bold Red): 29/170 fragment ions using 97 most intense peaks

#	b	b^{++}	b*	b^{*++}	b^{0}	b^{0++}	Seq.	y	y^{++}	y*	y^{*++}	y^{0}	y^{0++}	#
1	115.0502	58.0287	98.0237	49.5155			N							16
2	**172.0717**	86.5395	155.0451	78.0262			G	1653.8343	**827.4208**	1636.8078	818.9075	1635.8238	818.4155	15
3	**335.1350**	168.0711	**318.1084**	159.5579			Y	1596.8129	**798.9101**	1579.7863	**790.3968**	1578.8023	**789.9048**	14
4	**464.1776**	232.5924	**447.1510**	224.0792	**446.1670**	223.5872	E	1433.7496	**717.3784**	1416.7230	**708.8651**	1415.7390	**708.3731**	13
5	**577.2617**	289.1345	**560.2351**	280.6212	559.2511	280.1292	L	1304.7070	**652.8571**	1287.6804	644.3438	1286.6964	643.8518	12
6	664.2937	332.6505	**647.2671**	324.1372	646.2831	323.6452	S	1191.6229	**596.3151**	1174.5963	587.8018	1173.6123	587.3098	11

**7**	761.3464	381.1769	744.3199	372.6636	**743.3359**	372.1716	**P**	1104.5909	**552.7991**	1087.5643	544.2858	1086.5803	543.7938	**10**
**8**	862.3941	431.7007	845.3676	423.1874	844.3836	422.6954	**T**	1007.5381	504.2727	990.5116	495.7594	989.5275	495.2674	**9**
**9**	933.4312	467.2193	916.4047	458.7060	915.4207	458.2140	**A**	906.4904	**453.7489**	**889.4639**	445.2356	888.4799	444.7436	**8**
**10**	1004.4684	502.7378	987.4418	494.2245	**986.4578**	493.7325	**A**	835.4533	418.2303	818.4268	409.7170	817.4427	409.2250	**7**
**11**	**1075.5055**	538.2564	1058.4789	529.7431	1057.4949	529.2511	**A**	764.4162	**382.7117**	747.3896	374.1985	746.4056	373.7065	**6**
**12**	1189.5484	595.2778	1172.5218	586.7646	1171.5378	586.2726	**N**	693.3791	347.1932	676.3525	338.6799	675.3685	338.1879	**5**
**13**	1336.6168	668.8120	1319.5903	660.2988	1318.6062	659.8068	**F**	579.3362	**290.1717**	562.3096	281.6584	561.3256	281.1664	**4**
**14**	1437.6645	719.3359	1420.6379	710.8226	1419.6539	**710.3306**	**T**	**432.2677**	216.6375	415.2412	208.1242	414.2572	207.6322	**3**
**15**	1593.7656	797.3864	1576.7390	788.8732	1575.7550	788.3812	**R**	331.2201	166.1137	**314.1935**	157.6004			**2**
**16**							**R**	175.1190	88.0631	158.0924	79.5498			**1**

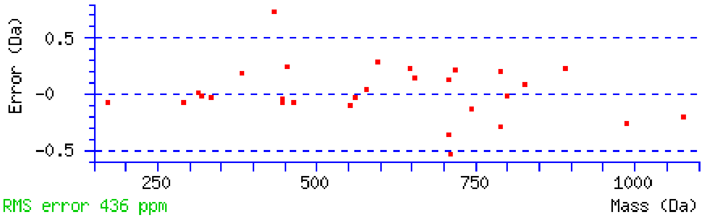

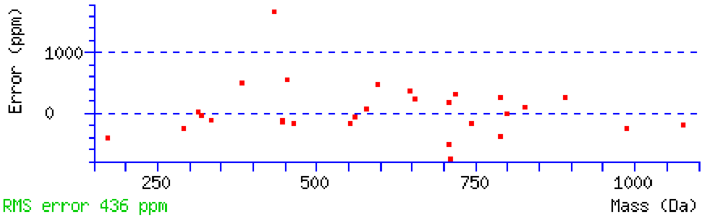

All matches to this query

Score	Mr(calc):	Delta	Sequence
21.6	1766.8700	0.0046	NGYELSPTAAANFTRR
13.7	1765.8734	1.0012	NLDDLVKQYESESVK
12.0	1765.8682	1.0064	HQKIHTGEKPFECR
7.1	1765.8764	0.9982	AQSLTLGHFKEQLSK
6.5	1765.8611	1.0135	NTLPTKETIEQEKR
6.5	1764.8560	2.0186	LVGSQSFSPGGPNGIIR
6.5	1764.8560	2.0186	LVGSQSFSPGGPNGIIR
4.5	1764.8533	2.0213	DSPPGSPRRRPGPGPR
3.4	1766.8845	-0.0099	TLVLIGASGVGRSHIK
2.7	1766.8604	0.0142	SQEVLKNSEIINWK

Spectrum No: 10; Query: 15786; Rank: 1

Peptide View

MS/MS Fragmentation of **ALRETLPAEQDLTTK**
Found in **IPI00016832**, Tax_Id=9606 Gene_Symbol=PSMA1 Isoform Short of Proteasome subunit alpha type-1

Match to Query 15786: 1684.904708 from(843.459630,2+)
Title: 090324LimSK_Exosome1_A.5291.5291.2.dta
Data file C:\Temp\Mascoti\LSK_2D\090324LimSK_Exosome1_A_2D.mgf

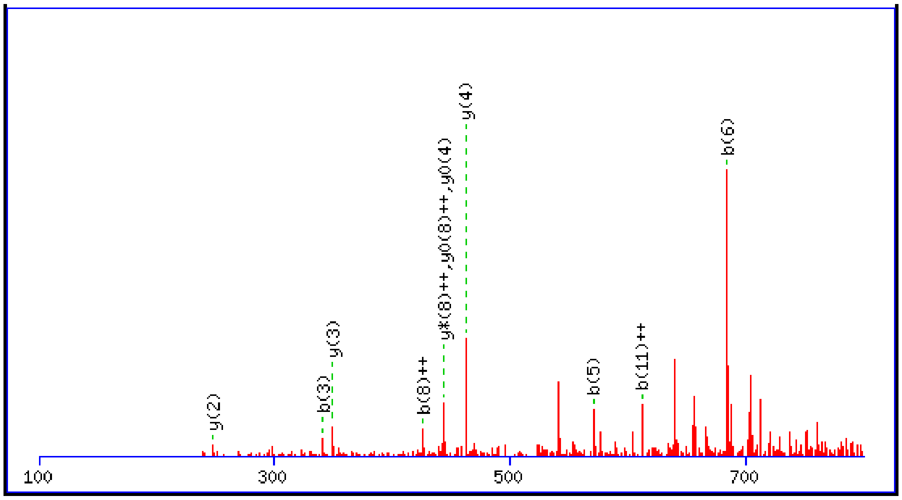

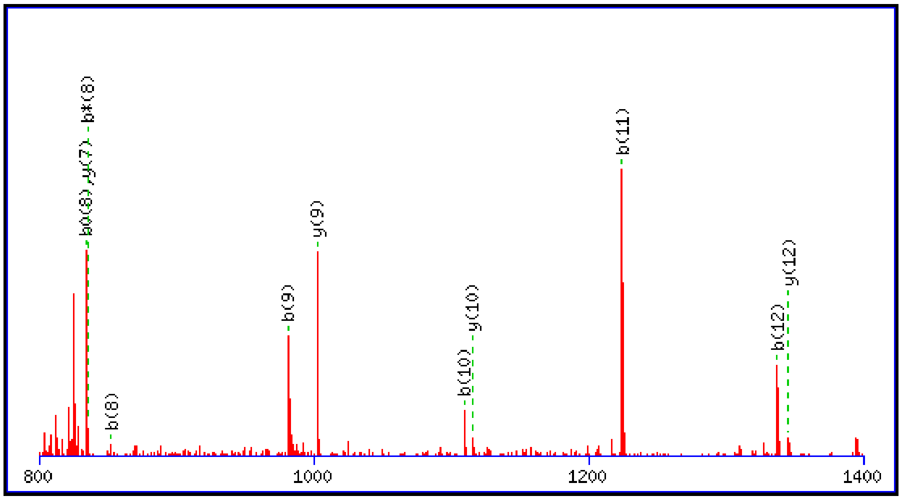

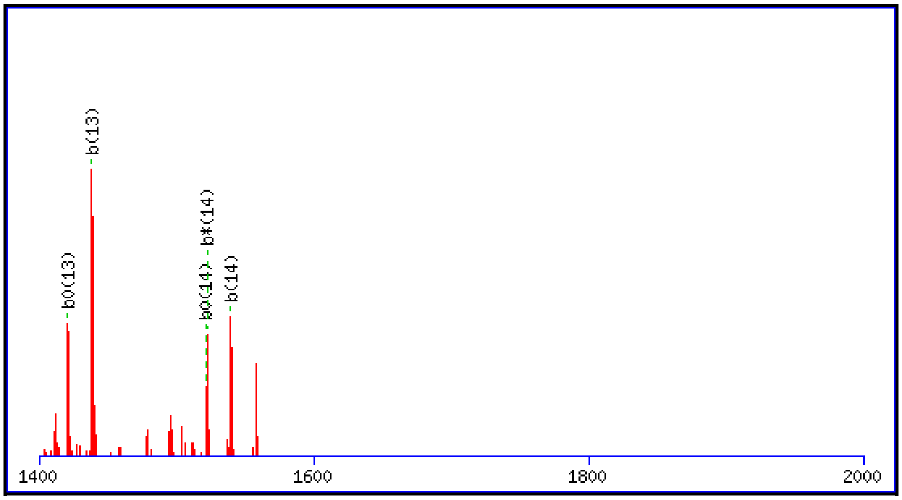

Monoisotopic mass of neutral peptide Mr(calc): 1684.8995
Fixed modifications: Carbamidomethyl (C)
Ions Score: 58 **Expect:** 0.00018
Matches (Bold Red): 27/156 fragment ions using 46 most intense peaks

#	b	b++	b*	b*++	b0	b0++	Seq.	y	y++	y*	y*++	y0	y0++	#
**1**	72.0444	36.5258					**A**							**15**
**2**	185.1285	93.0679					**L**	1614.8697	807.9385	1597.8432	799.4252	1596.8592	798.9332	**14**
**3**	**341.2296**	171.1184	324.2030	162.6051			**R**	1501.7857	751.3965	1484.7591	742.8832	1483.7751	742.3912	**13**
**4**	470.2722	235.6397	453.2456	227.1264	452.2616	226.6344	**E**	**1345.6846**	673.3459	1328.6580	664.8326	1327.6740	664.3406	**12**
**5**	**571.3198**	286.1636	554.2933	277.6503	553.3093	277.1583	**T**	1216.6420	608.8246	1199.6154	600.3113	1198.6314	599.8193	**11**
**6**	**684.4039**	342.7056	667.3774	334.1923	666.3933	333.7003	**L**	**1115.5943**	558.3008	1098.5677	549.7875	1097.5837	549.2955	**10**

**7**	781.4567	391.2320	764.4301	382.7187	763.4461	382.2267	**P**	**1002.5102**	501.7587	985.4837	493.2455	984.4997	492.7535	**9**
**8**	**852.4938**	**426.7505**	**835.4672**	418.2373	**834.4832**	417.7452	**A**	905.4575	453.2324	888.4309	**444.7191**	887.4469	**444.2271**	**8**
**9**	**981.5364**	491.2718	964.5098	482.7585	963.5258	482.2665	**E**	**834.4203**	417.7138	817.3938	409.2005	816.4098	408.7085	**7**
**10**	**1109.5949**	555.3011	1092.5684	546.7878	1091.5844	546.2958	**Q**	705.3777	353.1925	688.3512	344.6792	687.3672	344.1872	**6**
**11**	**1224.6219**	**612.8146**	1207.5953	604.3013	1206.6113	603.8093	**D**	577.3192	289.1632	560.2926	280.6499	559.3086	280.1579	**5**
**12**	**1337.7060**	669.3566	1320.6794	660.8433	1319.6954	660.3513	**L**	**462.2922**	231.6498	445.2657	223.1365	**444.2817**	222.6445	**4**
**13**	**1438.7536**	719.8805	1421.7271	711.3672	**1420.7431**	710.8752	**T**	**349.2082**	175.1077	332.1816	166.5944	331.1976	166.1024	**3**
**14**	**1539.8013**	770.4043	**1522.7748**	761.8910	**1521.7907**	761.3990	**T**	**248.1605**	124.5839	231.1339	116.0706	230.1499	115.5786	**2**
**15**							**K**	147.1128	74.0600	130.0863	65.5468			**1**

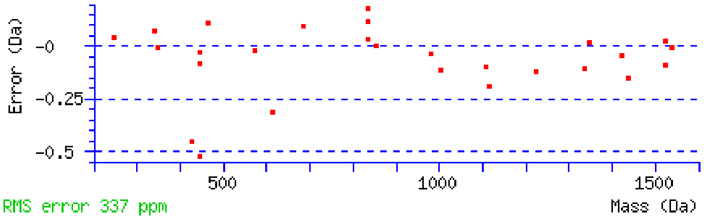

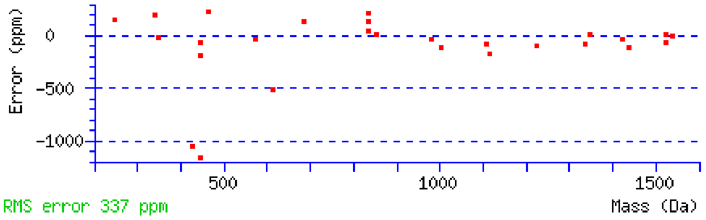

All matches to this query

Score	Mr(calc):	Delta	Sequence
58.0	1684.8995	0.0052	ALRETLPAEQDLTTK
14.2	1684.8937	0.0110	WLNKYFSLGNSKTK
9.0	1684.8930	0.0117	KPDTIEVXQMKAQAR
6.7	1684.8996	0.0052	DQLSLGNAALQITDVK
5.1	1684.8913	0.0134	KGIFSSLKGLDTLAR
3.8	1684.8913	0.0134	GIFSSLKGLDTLARK
3.8	1684.8913	0.0134	GIFSSLKGLDTLARK
3.5	1683.8903	1.0144	LQQDKKQLQEEAAR
2.7	1684.8896	0.0151	WEKPSPSNVTERKK
2.4	1683.8960	1.0087	ELKELASRVAFLTK

Spectrum No: 11; Query: 10661; Rank: 1

Peptide View

MS/MS Fragmentation of **ETLPAEQDLTTK**
Found in **IPI00016832**, Tax_Id=9606 Gene_Symbol=PSMA1 Isoform Short of Proteasome subunit alpha type-1

Match to Query 10661: 1345.682648 from(673.848600,2+)
Title: 090324LimSK_Exosome1_A.3959.3959.2.dta
Data file C:\Temp\Mascoti\LSK_2D\090324LimSK_Exosome1_A_2D.mgf

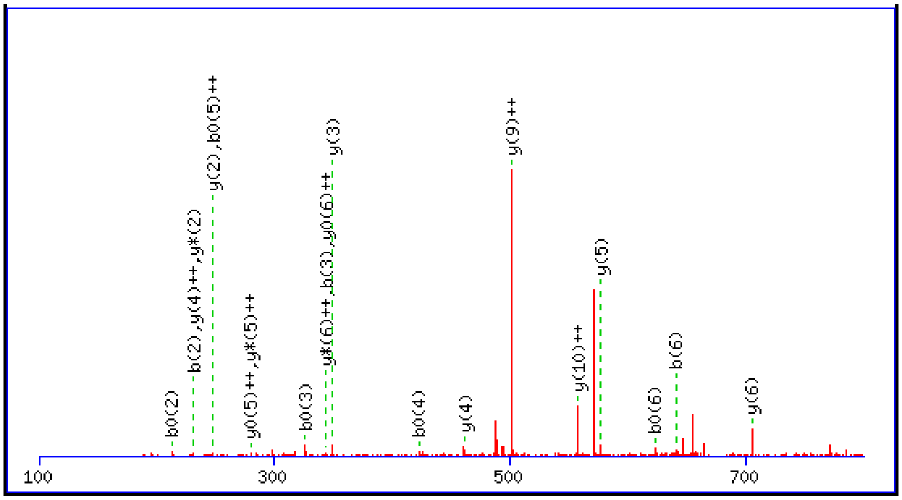

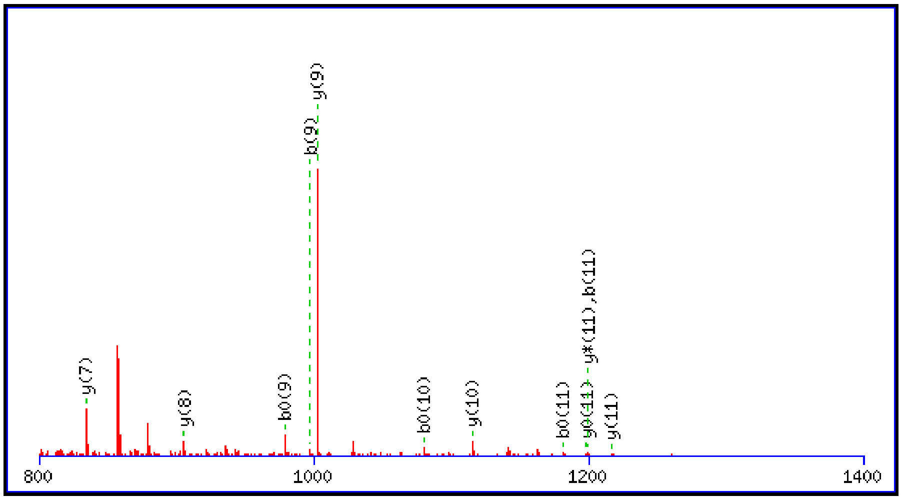

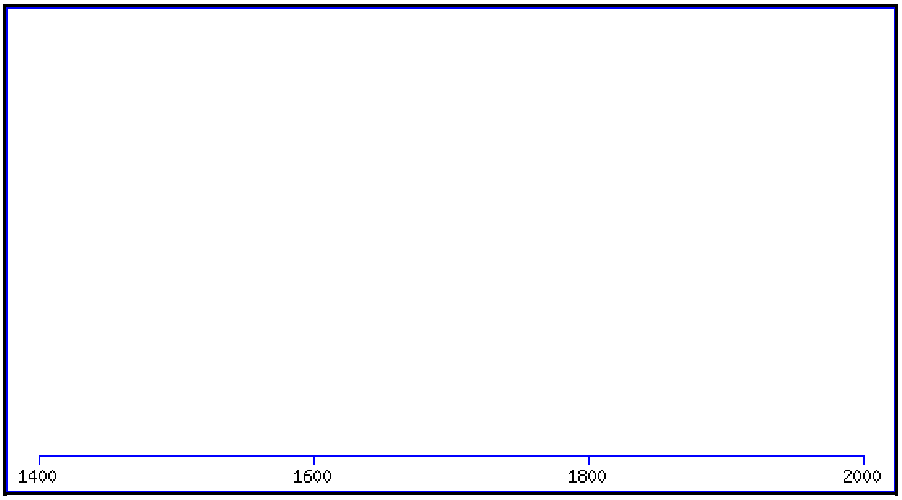

Monoisotopic mass of neutral peptide Mr(calc): 1344.6773
Fixed modifications: Carbamidomethyl (C)
Ions Score: 49 **Expect:** 0.0024
Matches (Bold Red): 33/118 fragment ions using 69 most intense peaks

#	b	b^{++}	b*	b^{*++}	b^{0}	b^{0++}	Seq.	y	y^{++}	y*	y^{*++}	y^{0}	y^{0++}	#
1	130.0499	65.5286			112.0393	56.5233	E							12
2	**231.0975**	116.0524			**213.0870**	107.0471	T	**1216.6420**	608.8246	**1199.6154**	600.3113	**1198.6314**	599.8193	11
3	**344.1816**	172.5944			**326.1710**	163.5892	L	**1115.5943**	**558.3008**	1098.5677	549.7875	1097.5837	549.2955	10
4	441.2344	221.1208			**423.2238**	212.1155	P	**1002.5102**	**501.7587**	985.4837	493.2455	984.4997	492.7535	9
5	512.2715	256.6394			494.2609	**247.6341**	A	**905.4575**	453.2324	888.4309	444.7191	887.4469	444.2271	8
6	**641.3141**	321.1607			**623.3035**	312.1554	E	**834.4203**	417.7138	817.3938	409.2005	816.4098	408.7085	7

7	769.3727	385.1900	752.3461	376.6767	751.3621	376.1847	Q	**705.3777**	353.1925	688.3512	**344.6792**	687.3672	**344.1872**	6
8	884.3996	442.7034	867.3731	434.1902	866.3890	433.6982	D	**577.3192**	289.1632	560.2926	**280.6499**	559.3086	**280.1579**	5
9	**997.4837**	499.2455	980.4571	490.7322	**979.4731**	490.2402	L	**462.2922**	**231.6498**	445.2657	223.1365	444.2817	222.6445	4
10	1098.5313	549.7693	1081.5048	541.2560	**1080.5208**	540.7640	T	**349.2082**	175.1077	332.1816	166.5944	331.1976	166.1024	3
11	**1199.5790**	600.2932	1182.5525	591.7799	**1181.5685**	591.2879	T	**248.1605**	124.5839	**231.1339**	116.0706	230.1499	115.5786	2
12							K	147.1128	74.0600	130.0863	65.5468			1

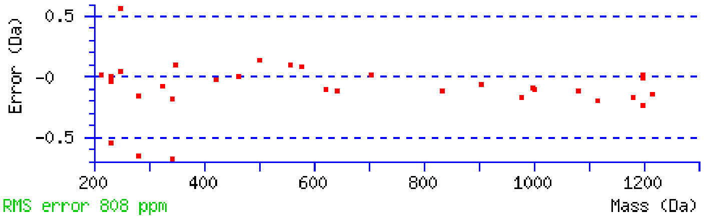

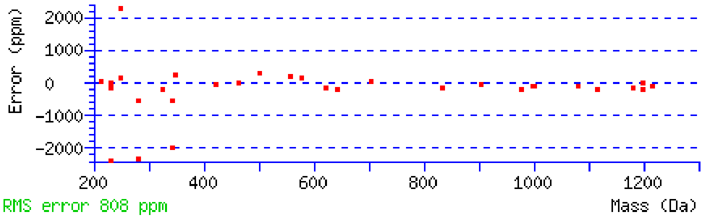

All matches to this query

Score	Mr(calc):	Delta	Sequence
48.5	1344.6773	1.0054	ETLPAEQDLTTK
11.8	1343.6850	1.9977	TKILNNSSIFK
10.2	1344.6826	1.0000	DVERFFKGYGK
7.1	1343.6655	2.0171	TLKWLISPVK
6.9	1343.6850	1.9977	TKILNNSSIFK
3.6	1343.6867	1.9959	NVQLICEGNGLK
2.2	1343.6738	2.0089	TIKGFETLVEK
1.9	1343.6721	2.0105	TSKGSYFLGAEGK
1.8	1344.6876	0.9950	VKYMATQLLAK
1.8	1345.6811	0.0016	GRGRSDTGAGSLGR

Spectrum No: 12; Query: 10569; Rank: 1

Peptide View

MS/MS Fragmentation of **FVFDRPLPVSR**
Found in **IPI00016832**, Tax_Id=9606 Gene_Symbol=PSMA1 Isoform Short of Proteasome subunit alpha type-1

Match to Query 10569: 1331.739432 from(444.920420,3+)
Title: 090324LimSK_Exosome1_A.7869.7869.3.dta
Data file C:\Temp\Mascoti\LSK_2D\090324LimSK_Exosome1_A_2D.mgf

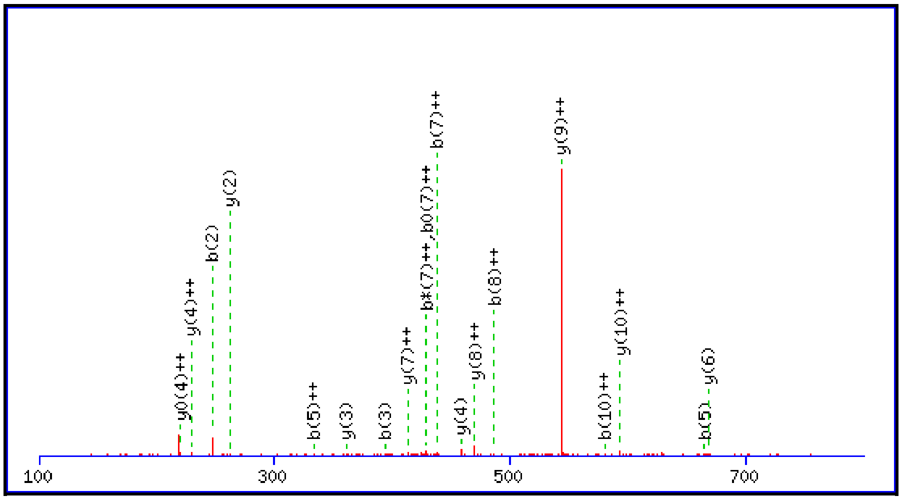

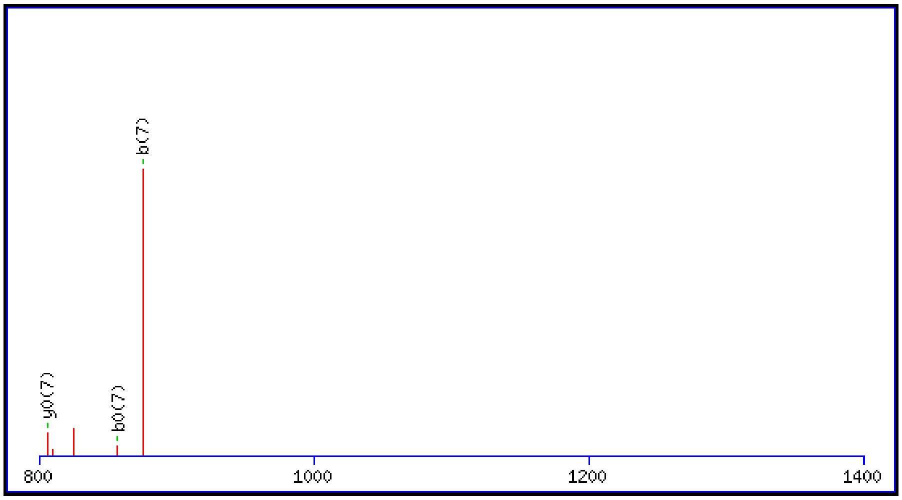

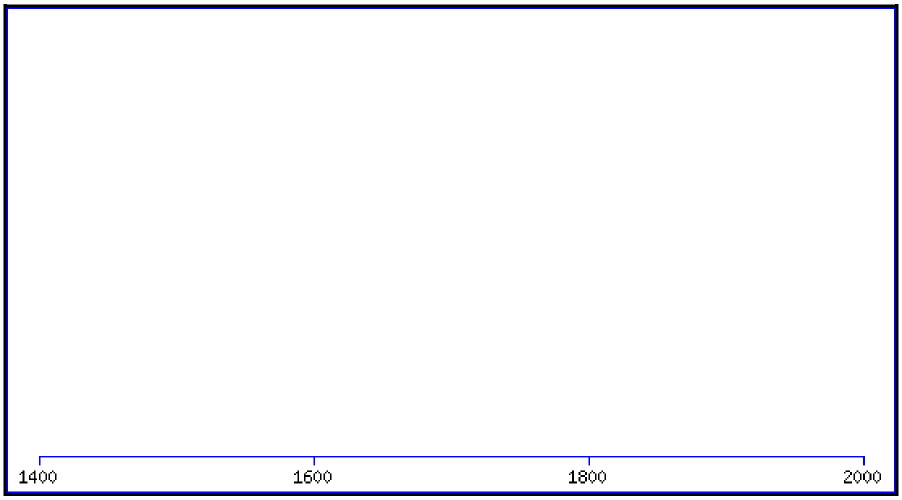

Monoisotopic mass of neutral peptide Mr(calc): 1331.7350
Fixed modifications: Carbamidomethyl (C)
Ions Score: 32 **Expect:** 0.063
Matches (Bold Red): 22/104 fragment ions using 41 most intense peaks

#	b	b++	b*	b*++	b0	b0++	Seq.	y	y++	y*	y*++	y0	y0++	#
1	148.0757	74.5415					F							11
2	**247.1441**	124.0757					V	1185.6739	**593.3406**	1168.6473	584.8273	1167.6633	584.3353	10
3	**394.2125**	197.6099					F	1086.6055	**543.8064**	1069.5789	535.2931	1068.5949	534.8011	9
4	509.2395	255.1234			491.2289	246.1181	D	939.5370	**470.2722**	922.5105	461.7589	921.5265	461.2669	8
5	**665.3406**	**333.1739**	648.3140	324.6606	647.3300	324.1686	R	824.5101	**412.7587**	807.4835	404.2454	**806.4995**	403.7534	7
6	762.3933	381.7003	745.3668	373.1870	744.3828	372.6950	P	**668.4090**	334.7081	651.3824	326.1949	650.3984	325.7028	6
7	**875.4774**	**438.2423**	858.4509	**429.7291**	**857.4668**	**429.2371**	L	571.3562	286.1817	554.3297	277.6685	553.3457	277.1765	5
8	972.5302	**486.7687**	955.5036	478.2554	954.5196	477.7634	P	**458.2722**	**229.6397**	441.2456	221.1264	440.2616	**220.6344**	4
9	1071.5986	536.3029	1054.5720	527.7897	1053.5880	527.2976	V	**361.2194**	181.1133	344.1928	172.6001	343.2088	172.1081	3
10	1158.6306	**579.8189**	1141.6041	571.3057	1140.6200	570.8137	S	**262.1510**	131.5791	245.1244	123.0659	244.1404	122.5738	2
11							R	175.1190	88.0631	158.0924	79.5498			1

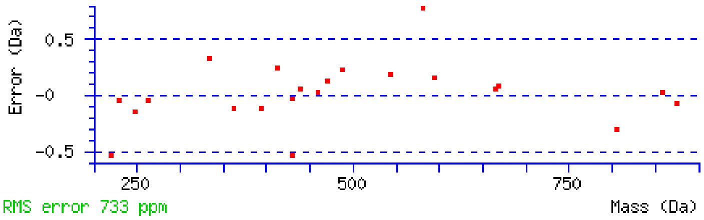

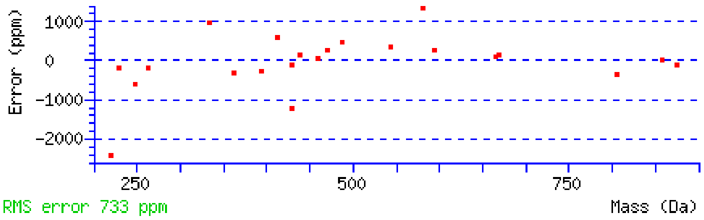

All matches to this query

Score	Mr(calc):	Delta	Sequence
32.0	1331.7350	0.0044	FVFDRPLPVSR
16.0	1330.7245	1.0149	XPTTSRPALDVK
14.4	1330.7357	1.0037	DPRAGPAVPGGLPK
14.2	1330.7235	1.0160	SGRHVTRVLVK
14.0	1330.7456	0.9938	DEAAKLQTTKVK
14.0	1330.7431	0.9963	DFRVINLMVPK
14.0	1329.7252	2.0142	TSGLQQKNVEVK
14.0	1329.7252	2.0142	VSEEVEKGRAVK
10.4	1331.7271	0.0123	LVPLASDMIFAR
8.6	1329.7252	2.0142	QTVSQVVNAKEK

Spectrum No: 13; Query: 14861; Rank: 1

Peptide View

MS/MS Fragmentation of **LVSLIGSKTQIPTQR**
Found in **IPI00016832**, Tax_Id=9606 Gene_Symbol=PSMA1 Isoform Short of Proteasome subunit alpha type-1

Match to Query 14861: 1639.963842 from(547.661890,3+)
Title: 090324LimSK_Exosome1_B.5938.5938.3.dta
Data file C:\Temp\Mascoti\LSK_2D\090324LimSK_Exosome1_A_2D.mgf

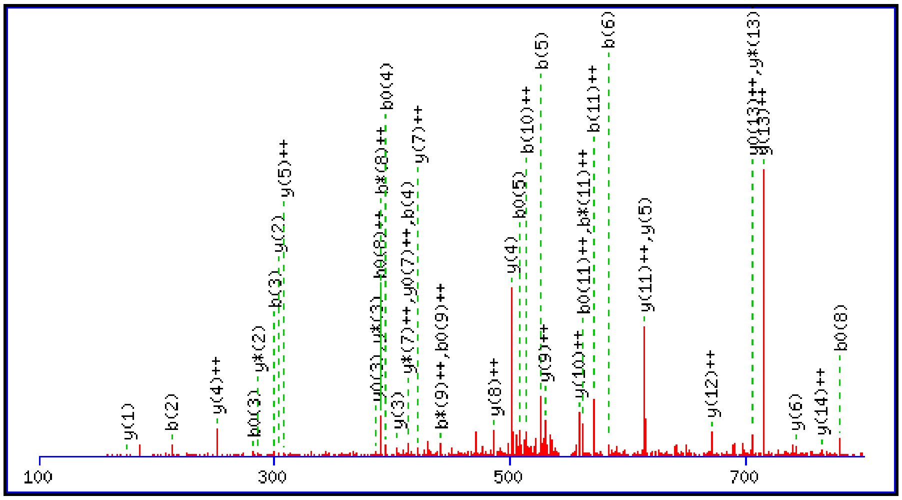

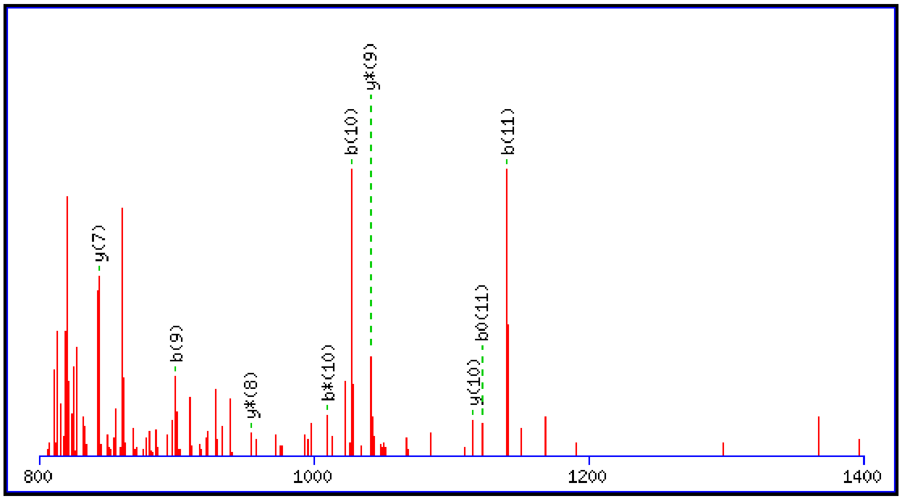

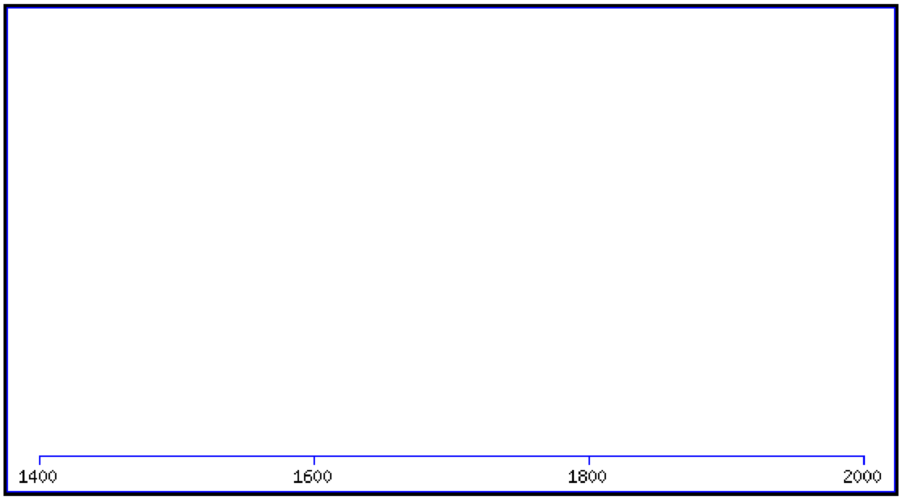

Monoisotopic mass of neutral peptide Mr(calc): 1639.9621
Fixed modifications: Carbamidomethyl (C)
Ions Score: 61 **Expect:** 2.1e-005
Matches (Bold Red): 49/146 fragment ions using 88 most intense peaks

#	b	b^{++}	b*	b^{*++}	b^0	b^{0++}	Seq.	y	y^{++}	y*	y^{*++}	y^0	y^{0++}	#
1	114.0913	57.5493					L							15
2	**213.1598**	107.0835					V	1527.8853	**764.4463**	1510.8588	755.9330	1509.8748	755.4410	14
3	**300.1918**	150.5995			**282.1812**	141.5942	S	1428.8169	**714.9121**	1411.7904	**706.3988**	1410.8063	**705.9068**	13
4	**413.2758**	207.1416			**395.2653**	198.1363	L	1341.7849	**671.3961**	1324.7583	662.8828	1323.7743	662.3908	12
5	**526.3599**	263.6836			**508.3493**	254.6783	I	1228.7008	**614.8540**	1211.6743	606.3408	1210.6902	605.8488	11
6	**583.3814**	292.1943			565.3708	283.1890	G	**1115.6167**	**558.3120**	1098.5902	549.7987	1097.6062	549.3067	10
7	670.4134	335.7103			652.4028	326.7051	S	1058.5953	**529.8013**	**1041.5687**	521.2880	1040.5847	520.7960	9
8	798.5084	399.7578	781.4818	**391.2445**	**780.4978**	**390.7525**	K	971.5633	**486.2853**	**954.5367**	477.7720	953.5527	477.2800	8
9	**899.5560**	450.2817	882.5295	**441.7684**	881.5455	**441.2764**	T	**843.4683**	**422.2378**	826.4417	**413.7245**	825.4577	**413.2325**	7
10	**1027.6146**	**514.3109**	**1010.5881**	505.7977	1009.6041	505.3057	Q	**742.4206**	371.7139	725.3941	363.2007	724.4100	362.7087	6
11	**1140.6987**	**570.8530**	1123.6721	**562.3397**	**1122.6881**	**561.8477**	I	**614.3620**	**307.6847**	597.3355	299.1714	596.3515	298.6794	5
12	1237.7515	619.3794	1220.7249	610.8661	1219.7409	610.3741	P	**501.2780**	**251.1426**	484.2514	242.6293	483.2674	242.1373	4
13	1338.7991	669.9032	1321.7726	661.3899	1320.7886	660.8979	T	**404.2252**	202.6162	**387.1987**	194.1030	**386.2146**	193.6110	3
14	1466.8577	733.9325	1449.8312	725.4192	1448.8471	724.9272	Q	**303.1775**	152.0924	**286.1510**	143.5791			2
15							R	**175.1190**	88.0631	158.0924	79.5498			1

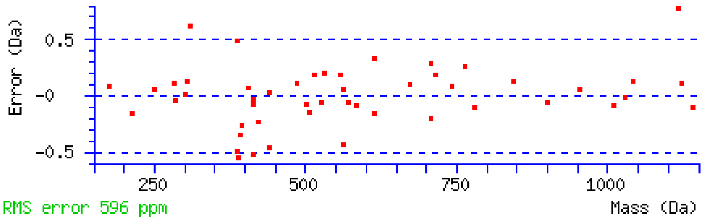

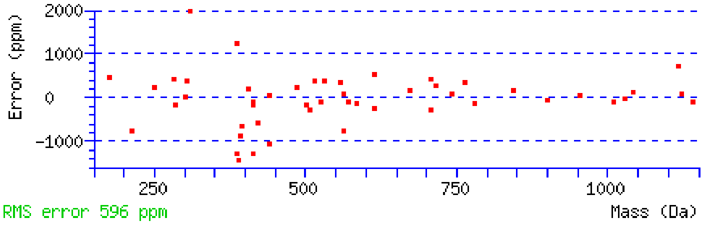

All matches to this query

Score	Mr(calc):	Delta	Sequence
60.9	1639.9621	0.0017	LVSLIGSKTQIPTQR
4.3	1639.9482	0.0157	QRGLTESRVLVGGLR
4.3	1638.9569	1.0069	EAIGKYHRALLQLK
4.0	1637.9464	2.0174	VNAPILTNTTLNVIR
1.9	1639.9701	-0.0063	SRVLFFIYIIAISV
1.8	1637.9577	2.0062	IKVVGSRGTIEINPR
1.4	1638.9531	1.0108	LAMAIPDKWIQLLK

0.9	1638.9668	0.9970	VITVLVGNKIDLAER
0.8	1638.9668	0.9970	SRKEVELIIQVTPK

Spectrum No: 14; Query: 17562; Rank: 1

Peptide View

MS/MS Fragmentation of **NQYDNDVTVWSPQGR**
Found in **IPI00016832**, Tax_Id=9606 Gene_Symbol=PSMA1 Isoform Short of Proteasome subunit alpha type-1

Match to Query 17562: 1777.802128 from(889.908340,2+)
Title: 090324LimSK_Exosome1_B.6246.6246.2.dta
Data file C:\Temp\Mascoti\LSK_2D\090324LimSK_Exosome1_A_2D.mgf

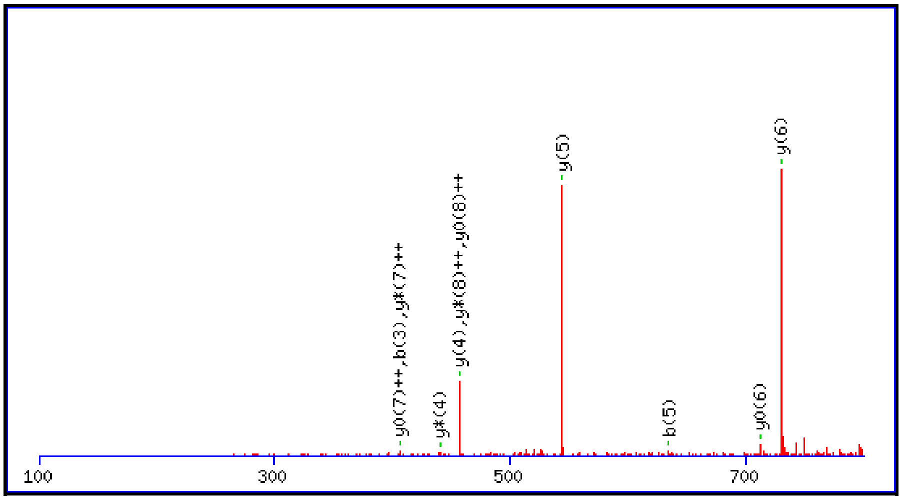

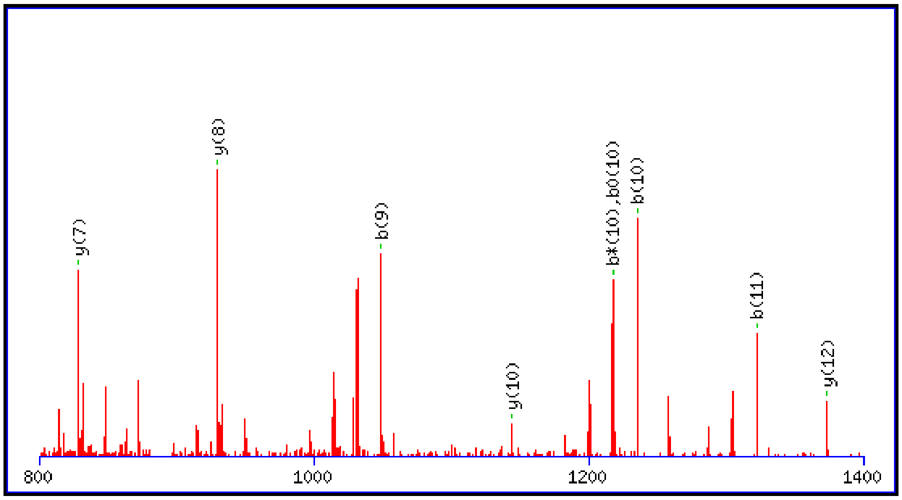

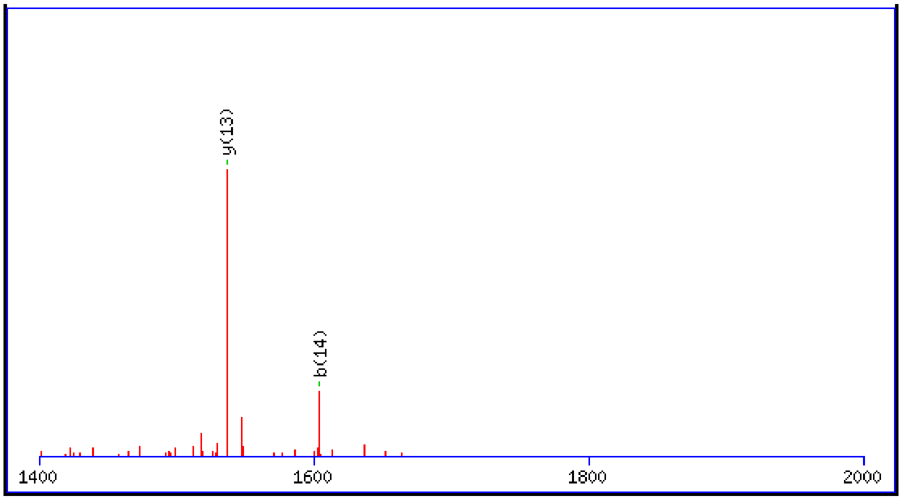

Monoisotopic mass of neutral peptide Mr(calc): 1777.8020
Fixed modifications: Carbamidomethyl (C)
Ions Score: 73 **Expect:** 1.2e-005
Matches (Bold Red): 22/154 fragment ions using 26 most intense peaks

#	b	b++	b*	b*++	b0	b0++	Seq.	y	y++	y*	y*++	y0	y0++	#
1	115.0502	58.0287	98.0237	49.5155			N							15
2	243.1088	122.0580	226.0822	113.5448			Q	1664.7663	832.8868	1647.7398	824.3735	1646.7558	823.8815	14
3	**406.1721**	203.5897	389.1456	195.0764			Y	**1536.7077**	768.8575	1519.6812	760.3442	1518.6972	759.8522	13
4	521.1991	261.1032	504.1725	252.5899	503.1885	252.0979	D	**1373.6444**	687.3258	1356.6179	678.8126	1355.6339	678.3206	12
5	**635.2420**	318.1246	618.2154	309.6114	617.2314	309.1193	N	1258.6175	629.8124	1241.5909	621.2991	1240.6069	620.8071	11
6	750.2689	375.6381	733.2424	367.1248	732.2584	366.6328	D	**1144.5745**	572.7909	1127.5480	564.2776	1126.5640	563.7856	10
7	849.3373	425.1723	832.3108	416.6590	831.3268	416.1670	V	1029.5476	515.2774	1012.5211	506.7642	1011.5370	506.2722	9
8	950.3850	475.6961	933.3585	467.1829	932.3745	466.6909	T	**930.4792**	465.7432	913.4526	**457.2300**	912.4686	**456.7380**	8
9	**1049.4534**	525.2304	1032.4269	516.7171	1031.4429	516.2251	V	**829.4315**	415.2194	812.4050	**406.7061**	811.4209	**406.2141**	7
10	**1235.5327**	618.2700	**1218.5062**	609.7567	**1217.5222**	609.2647	W	**730.3631**	365.6852	713.3366	357.1719	**712.3525**	356.6799	6
11	**1322.5648**	661.7860	1305.5382	653.2727	1304.5542	652.7807	S	**544.2838**	272.6455	527.2572	264.1323	526.2732	263.6402	5
12	1419.6175	710.3124	1402.5910	701.7991	1401.6070	701.3071	P	**457.2518**	229.1295	**440.2252**	220.6162			4
13	1547.6761	774.3417	1530.6496	765.8284	1529.6655	765.3364	Q	360.1990	180.6031	343.1724	172.0899			3
14	**1604.6976**	802.8524	1587.6710	794.3392	1586.6870	793.8471	G	232.1404	116.5738	215.1139	108.0606			2
15							R	175.1190	88.0631	158.0924	79.5498			1

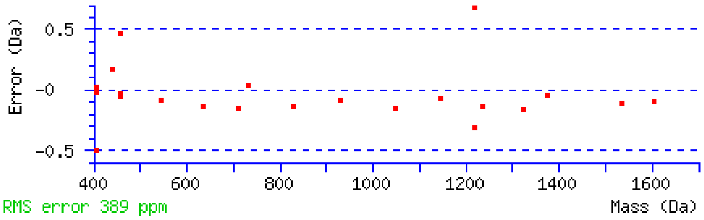

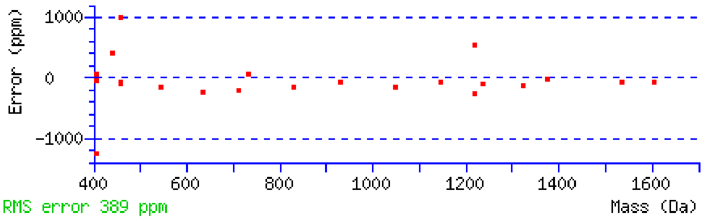

All matches to this query

Score	Mr(calc):	Delta	Sequence
73.1	1777.8020	0.0001	NQYDNDVTVWSPQGR
5.3	1777.7913	0.0108	VAKTGSSNAYLHSKR
5.3	1777.7913	0.0108	VAKTGSSNAYLHSKR
5.3	1777.7913	0.0108	VAKTGSSNAYLHSKR
4.7	1777.7915	0.0106	MISTLATFPPFLHK
2.2	1777.8070	-0.0048	ELMGFSKSVNEARSK
1.8	1777.7971	0.0050	NPFWSMSTSSVRKR

1.6	1776.8068	0.9953	MKSLPCLLLSAQTR
1.6	1777.8182	-0.0161	QNITMKDHLSDIQR
1.3	1776.7921	1.0100	KVRSGGGSGGSGGQVSLK

Spectrum No: 15; Query: 28457; Rank: 1

Peptide View

MS/MS Fragmentation of **AITVFSPDGHLFQVEYAQEAVK**
Found in **IPI00024175**, Tax_Id=9606 Gene_Symbol=PSMA7 Isoform 1 of Proteasome subunit alpha type-7

Match to Query 28457: 2448.238122 from(817.086650,3+)
Title: 090324LimSK_Exosome1_B.14855.14855.3.dta
Data file C:\Temp\Mascoti\LSK_2D\090324LimSK_Exosome1_A_2D.mgf

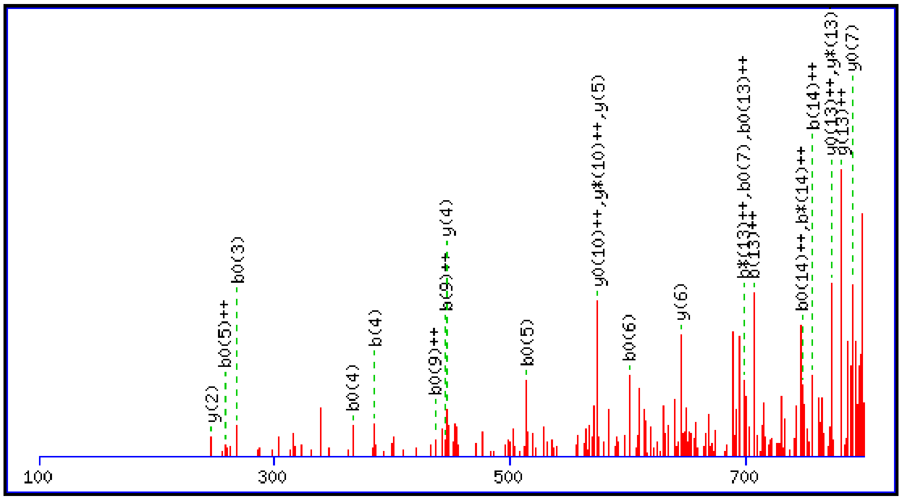

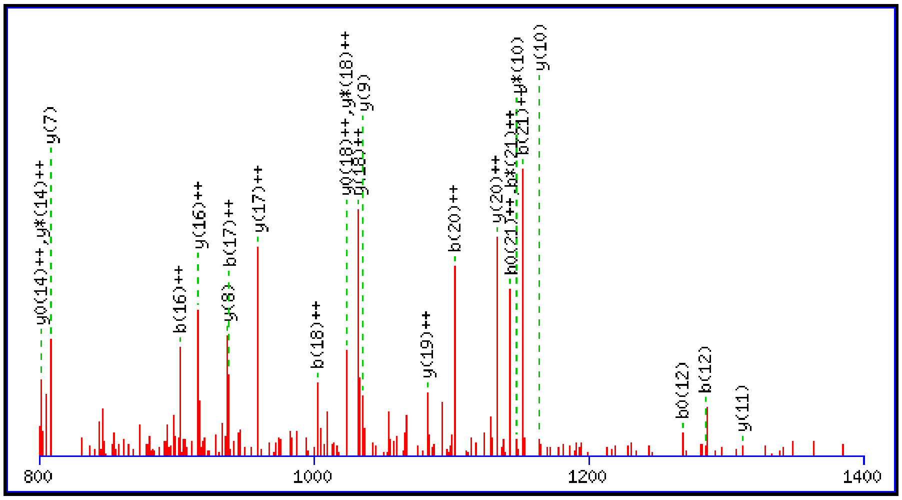

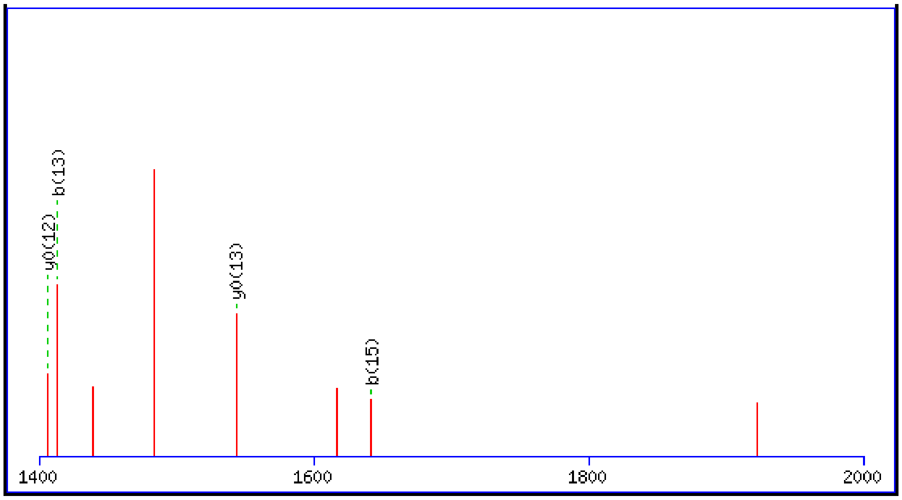

Monoisotopic mass of neutral peptide Mr(calc): 2448.2325
Fixed modifications: Carbamidomethyl (C)
Ions Score: 53 **Expect:** 0.0011
Matches (Bold Red): 53/218 fragment ions using 82 most intense peaks

#	b	b^{++}	b*	b^{*++}	b^{0}	b^{0++}	Seq.	y	y^{++}	y*	y^{*++}	y^{0}	y^{0++}	#
1	72.0444	36.5258					A							22
2	185.1285	93.0679					I	2378.2027	1189.6050	2361.1761	1181.0917	2360.1921	1180.5997	21
3	286.1761	143.5917			**268.1656**	134.5864	T	2265.1186	**1133.0630**	2248.0921	1124.5497	2247.1081	1124.0577	20
4	**385.2445**	193.1259			**367.2340**	184.1206	V	2164.0709	**1082.5391**	2147.0444	1074.0258	2146.0604	1073.5338	19
5	532.3130	266.6601			**514.3024**	**257.6548**	F	2065.0025	**1033.0049**	2047.9760	**1024.4916**	2046.9920	**1023.9996**	18
6	619.3450	310.1761			**601.3344**	301.1709	S	1917.9341	**959.4707**	1900.9076	950.9574	1899.9236	950.4654	17
7	716.3978	358.7025			**698.3872**	349.6972	P	1830.9021	**915.9547**	1813.8755	907.4414	1812.8915	906.9494	16
8	831.4247	416.2160			813.4141	407.2107	D	1733.8493	867.4283	1716.8228	858.9150	1715.8388	858.4230	15
9	888.4462	**444.7267**			870.4356	**435.7214**	G	1618.8224	809.9148	1601.7958	**801.4016**	1600.8118	**800.9095**	14
10	1025.5051	513.2562			1007.4945	504.2509	H	1561.8009	**781.4041**	1544.7744	**772.8908**	**1543.7904**	**772.3988**	13
11	1138.5891	569.7982			1120.5786	560.7929	L	1424.7420	712.8746	1407.7155	704.3614	**1406.7314**	703.8694	12
12	**1285.6576**	643.3324			**1267.6470**	634.3271	F	**1311.6579**	656.3326	1294.6314	647.8193	1293.6474	647.3273	11
13	**1413.7161**	**707.3617**	1396.6896	**698.8484**	1395.7056	**698.3564**	Q	**1164.5895**	582.7984	**1147.5630**	**574.2851**	1146.5790	**573.7931**	10
14	1512.7845	**756.8959**	1495.7580	**748.3826**	1494.7740	**747.8906**	V	**1036.5310**	518.7691	1019.5044	510.2558	1018.5204	509.7638	9
15	**1641.8271**	821.4172	1624.8006	812.9039	1623.8166	812.4119	E	**937.4625**	469.2349	920.4360	460.7216	919.4520	460.2296	8
16	1804.8905	**902.9489**	1787.8639	894.4356	1786.8799	893.9436	Y	**808.4199**	404.7136	791.3934	396.2003	**790.4094**	395.7083	7
17	1875.9276	**938.4674**	1858.9010	929.9542	1857.9170	929.4621	A	**645.3566**	323.1819	628.3301	314.6687	627.3461	314.1767	6
18	2003.9862	**1002.4967**	1986.9596	993.9834	1985.9756	993.4914	Q	**574.3195**	287.6634	557.2930	279.1501	556.3089	278.6581	5
19	2133.0287	1067.0180	2116.0022	1058.5047	2115.0182	1058.0127	E	**446.2609**	223.6341	429.2344	215.1208	428.2504	214.6288	4
20	2204.0659	**1102.5366**	2187.0393	1094.0233	2186.0553	1093.5313	A	317.2183	159.1128	300.1918	150.5995			3
21	2303.1343	**1152.0708**	2286.1077	**1143.5575**	2285.1237	**1143.0655**	V	**246.1812**	123.5942	229.1547	115.0810			2
22							K	147.1128	74.0600	130.0863	65.5468			1

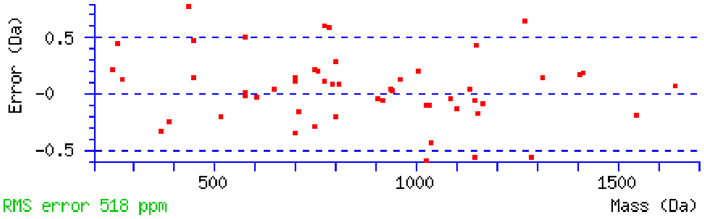

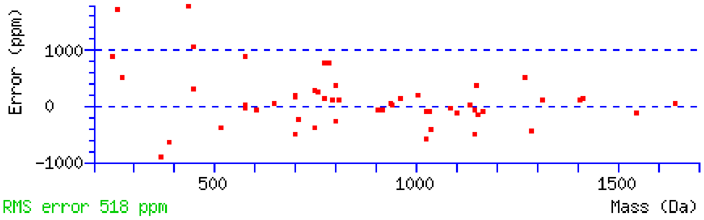

All matches to this query

Score	Mr(calc):	Delta	Sequence

52.9	2448.2325	0.0056	AITVFSPDGHLFQVEYAQEAVK
3.6	2447.2324	1.0057	ERVPFVLTPDFLFVMGTSGKK
3.6	2447.2324	1.0057	ERVPFVLTPDFLFVMGTSGKK
3.2	2448.2196	0.0185	NDGTRPKMTPGPAVLATKAAAGTK
2.7	2446.2151	2.0230	TAPAANLASRIPAASAAAMNLASAR
2.3	2447.2154	1.0227	LSDYLFTLARYAAMKEGNQEK
1.9	2448.2279	0.0103	VCSKATSPTSQRGQEVISTPTSK
1.6	2448.2342	0.0039	DLSSGFPSFLTSSILWKSAVVK
1.6	2447.2495	0.9887	SDMNKPHLISELLTKLQLSGK
1.2	2447.2553	0.9829	VFEAKVIDGTLCGPETLAICVR

Spectrum No: 16; Query: 30235; Rank: 1

Peptide View

MS/MS Fragmentation of **AITVFSPDGHLFQVEYAQEAVKK**
Found in **IPI00024175**, Tax_Id=9606 Gene_Symbol=PSMA7 Isoform 1 of Proteasome subunit alpha type-7

Match to Query 30235: 2576.332122 from(859.784650,3+)
Title: 090324LimSK_Exosome1_B.13777.13777.3.dta
Data file C:\Temp\Mascoti\LSK_2D\090324LimSK_Exosome1_A_2D.mgf

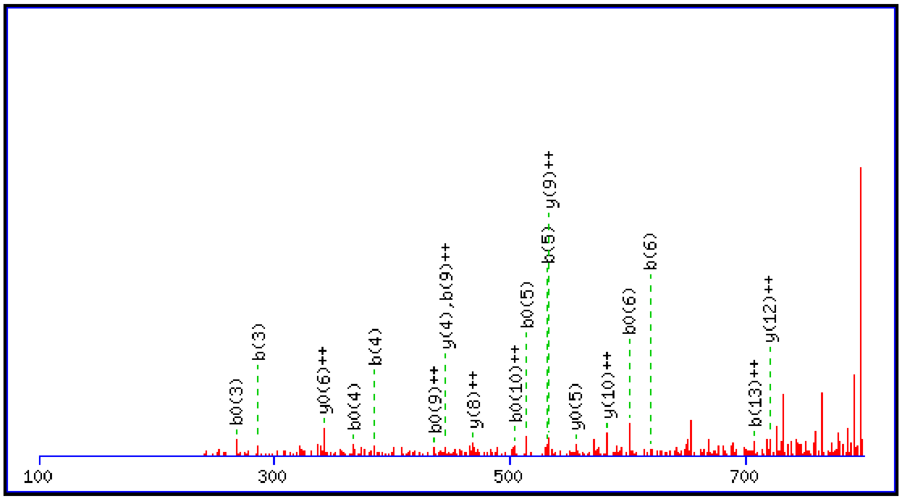

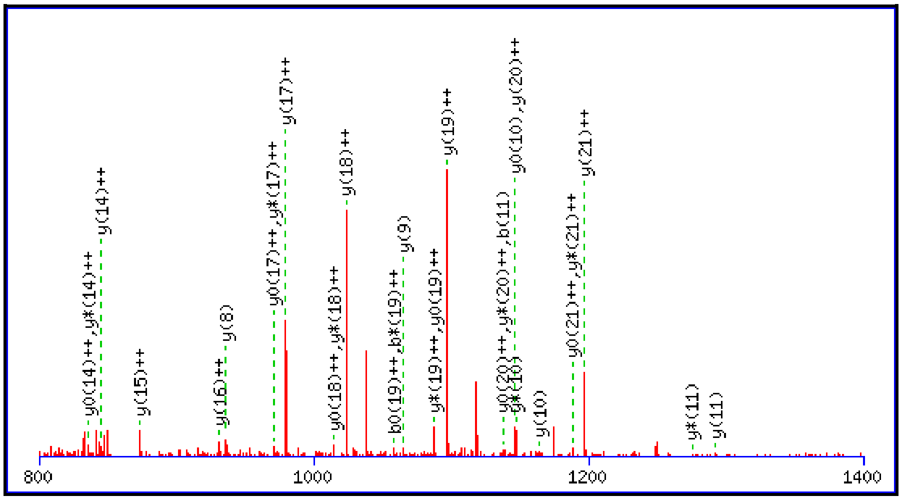

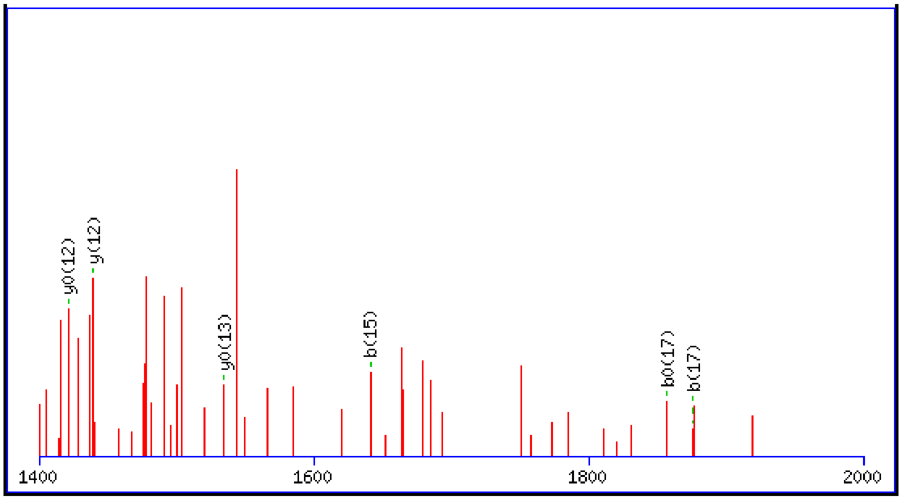

Monoisotopic mass of neutral peptide Mr(calc): 2576.3275
Fixed modifications: Carbamidomethyl (C)
Ions Score: 27 **Expect:** 0.3
Matches (Bold Red): 55/228 fragment ions using 158 most intense peaks

#	b	b^{++}	b*	b^{*++}	b^{0}	b^{0++}	Seq.	y	y^{++}	y*	y^{*++}	y^{0}	y^{0++}	#
1	72.0444	36.5258					A							23
2	185.1285	93.0679					I	2506.2977	1253.6525	2489.2711	1245.1392	2488.2871	1244.6472	22
3	**286.1761**	143.5917			**268.1656**	134.5864	T	2393.2136	**1197.1104**	2376.1870	**1188.5972**	2375.2030	**1188.1052**	21
4	**385.2445**	193.1259			**367.2340**	184.1206	V	2292.1659	**1146.5866**	2275.1394	**1138.0733**	2274.1553	**1137.5813**	20
5	**532.3130**	266.6601			**514.3024**	257.6548	F	2193.0975	**1097.0524**	2176.0709	**1088.5391**	2175.0869	**1088.0471**	19
6	**619.3450**	310.1761			**601.3344**	301.1709	S	2046.0291	**1023.5182**	2029.0025	**1015.0049**	2028.0185	**1014.5129**	18
7	716.3978	358.7025			698.3872	349.6972	P	1958.9971	**980.0022**	1941.9705	**971.4889**	1940.9865	**970.9969**	17
8	831.4247	416.2160			813.4141	407.2107	D	1861.9443	**931.4758**	1844.9177	922.9625	1843.9337	922.4705	16
9	888.4462	**444.7267**			870.4356	**435.7214**	G	1746.9173	**873.9623**	1729.8908	865.4490	1728.9068	864.9570	15
10	1025.5051	513.2562			1007.4945	**504.2509**	H	1689.8959	**845.4516**	1672.8693	**836.9383**	1671.8853	**836.4463**	14
11	**1138.5891**	569.7982			1120.5786	560.7929	L	1552.8370	776.9221	1535.8104	768.4088	**1534.8264**	767.9168	13
12	1285.6576	643.3324			1267.6470	634.3271	F	**1439.7529**	**720.3801**	1422.7264	711.8668	**1421.7423**	711.3748	12
13	1413.7161	**707.3617**	1396.6896	698.8484	1395.7056	698.3564	Q	**1292.6845**	646.8459	**1275.6579**	638.3326	1274.6739	637.8406	11
14	1512.7845	756.8959	1495.7580	748.3826	1494.7740	747.8906	V	**1164.6259**	**582.8166**	**1147.5994**	574.3033	**1146.6154**	573.8113	10
15	**1641.8271**	821.4172	1624.8006	812.9039	1623.8166	812.4119	E	**1065.5575**	**533.2824**	1048.5310	524.7691	1047.5469	524.2771	9
16	1804.8905	902.9489	1787.8639	894.4356	1786.8799	893.9436	Y	**936.5149**	**468.7611**	919.4884	460.2478	918.5043	459.7558	8
17	**1875.9276**	938.4674	1858.9010	929.9542	**1857.9170**	929.4621	A	773.4516	387.2294	756.4250	378.7162	755.4410	378.2241	7
18	2003.9862	1002.4967	1986.9596	993.9834	1985.9756	993.4914	Q	702.4145	351.7109	685.3879	343.1976	684.4039	**342.7056**	6
19	2133.0287	1067.0180	2116.0022	**1058.5047**	2115.0182	**1058.0127**	E	574.3559	287.6816	557.3293	279.1683	**556.3453**	278.6763	5
20	2204.0659	1102.5366	2187.0393	1094.0233	2186.0553	1093.5313	A	**445.3133**	223.1603	428.2867	214.6470			4
21	2303.1343	1152.0708	2286.1077	1143.5575	2285.1237	1143.0655	V	374.2762	187.6417	357.2496	179.1285			3
22	2431.2292	1216.1183	2414.2027	1207.6050	2413.2187	1207.1130	K	275.2078	138.1075	258.1812	129.5942			2
23							K	147.1128	74.0600	130.0863	65.5468			1

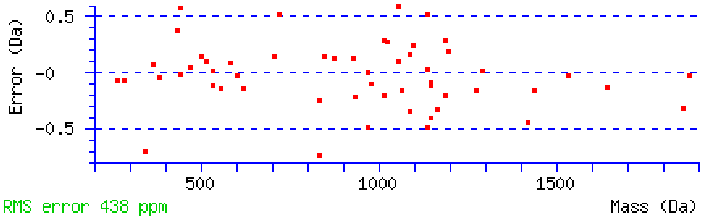

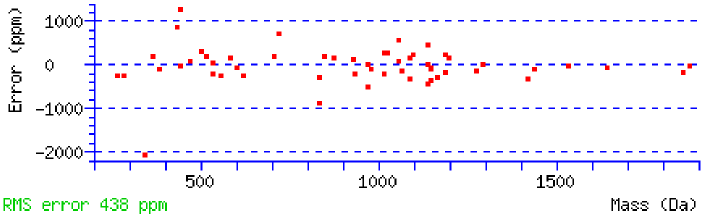

All matches to this query

Score	Mr(calc):	Delta	Sequence
26.7	2576.3275	0.0046	AITVFSPDGHLFQVEYAQEAVKK
3.5	2575.3457	0.9864	NLMNVASVRKHLASIPNLSYIR
1.6	2575.3345	0.9976	TALLLRNLGFTAIPLHGQMSQSK
1.6	2575.3345	0.9976	TALLLRNLGFTAIPLHGQMSQSK
1.1	2575.3258	1.0063	GSIPTSLTALSLASASPPLSGRSTPK
1.0	2576.3241	0.0081	GPGKVNSSIPLTSVGLRPQLLQR
0.8	2576.3129	0.0192	QQKMGQKSQRPSTANFPLSNSVK
0.3	2575.3031	1.0290	ATLPFAPGKTSRVPSDSGVFSSDPR
0.2	2574.3288	2.0034	WGLQPTQPTLPLPSLSLFFPQK
0.0	2576.3224	0.0097	HQPSLKHTKGGTQEAVAGISVVPR

Spectrum No: 17; Query: 8378; Rank: 1

Peptide View

MS/MS Fragmentation of **ALLEVVQSGGK**
Found in **IPI00024175**, Tax_Id=9606 Gene_Symbol=PSMA7 Isoform 1 of Proteasome subunit alpha type-7

Match to Query 8378: 1099.625288 from(550.819920,2+)
Title: 090324LimSK_Exosome1_A.4867.4867.2.dta
Data file C:\Temp\Mascoti\LSK_2D\090324LimSK_Exosome1_A_2D.mgf

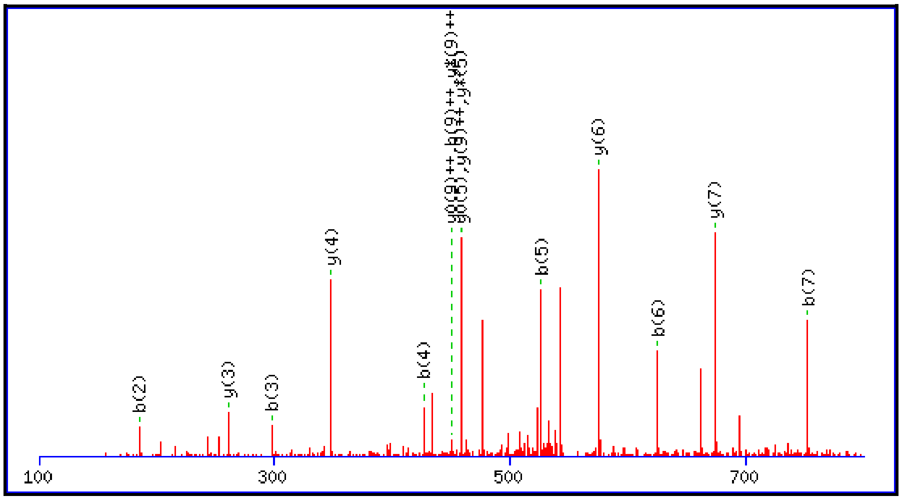

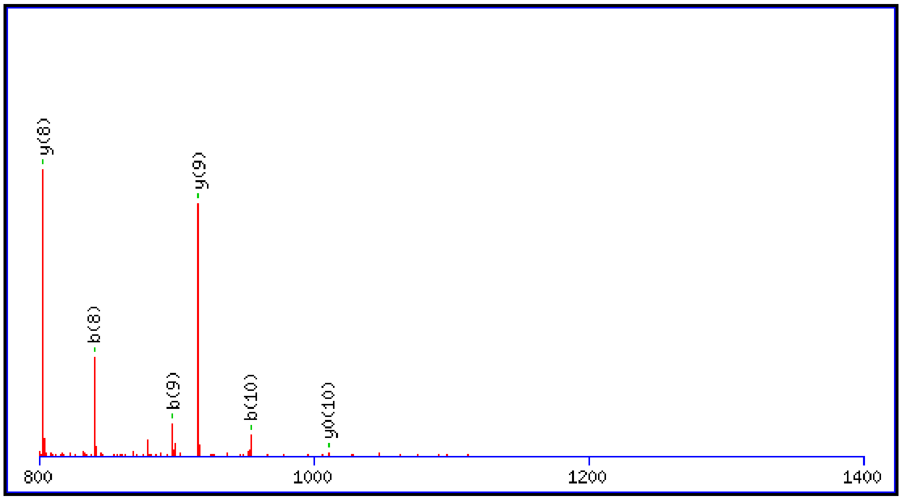

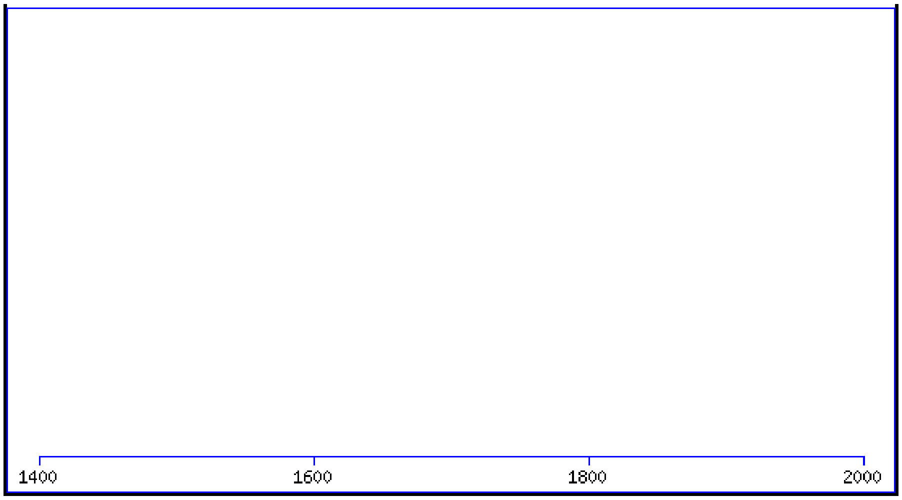

Monoisotopic mass of neutral peptide Mr(calc): 1099.6237
Fixed modifications: Carbamidomethyl (C)
Ions Score: 74 **Expect:** 3.8e-006
Matches (Bold Red): 22/96 fragment ions using 32 most intense peaks

#	b	b^{++}	b*	b^{*++}	b^{0}	b^{0++}	Seq.	y	y^{++}	y*	y^{*++}	y^{0}	y^{0++}	#
1	72.0444	36.5258					A							11
2	**185.1285**	93.0679					L	1029.5939	515.3006	1012.5673	506.7873	**1011.5833**	506.2953	10
3	**298.2125**	149.6099					L	**916.5098**	**458.7585**	899.4833	**450.2453**	898.4993	**449.7533**	9
4	**427.2551**	214.1312			409.2445	205.1259	E	**803.4258**	402.2165	786.3992	393.7032	785.4152	393.2112	8
5	**526.3235**	263.6654			508.3130	254.6601	V	**674.3832**	337.6952	657.3566	329.1819	656.3726	328.6899	7
6	**625.3919**	313.1996			607.3814	304.1943	V	**575.3148**	288.1610	558.2882	279.6477	557.3042	279.1557	6
7	**753.4505**	377.2289	736.4240	368.7156	735.4400	368.2236	Q	476.2463	238.6268	**459.2198**	230.1135	**458.2358**	229.6215	5
8	**840.4825**	420.7449	823.4560	412.2316	822.4720	411.7396	S	**348.1878**	174.5975	331.1612	166.0842	330.1772	165.5922	4
9	**897.5040**	**449.2556**	880.4775	440.7424	879.4934	440.2504	G	**261.1557**	131.0815	244.1292	122.5682			3
10	**954.5255**	477.7664	937.4989	469.2531	936.5149	468.7611	G	204.1343	102.5708	187.1077	94.0575			2
11							K	147.1128	74.0600	130.0863	65.5468			1

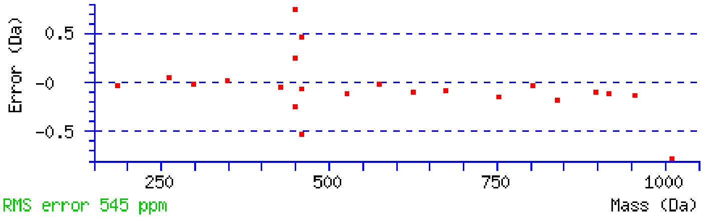

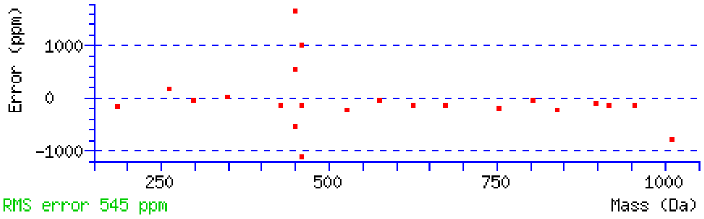

All matches to this query

Score	Mr(calc):	Delta	Sequence
74.1	1099.6237	0.0016	ALLEVVQSGGK
18.2	1099.6349	-0.0096	LALEKERNK
4.7	1099.6237	0.0016	LAPVITGTNSK
4.3	1099.6237	0.0016	ALQVLVDQSK
3.9	1099.6237	0.0016	ILNLSGNELK
3.9	1099.6172	0.0081	LMSSLHLKR
1.2	1098.6284	0.9968	SVPELKDALK
0.9	1099.6237	0.0016	AIPKTLKDSQ
0.3	1098.6284	0.9968	LLDLAQEGLK

Spectrum No: 18; Query: 14716; Rank: 1

Peptide View

MS/MS Fragmentation of **LTVEDPVTVEYITR**
Found in **IPI00024175**, Tax_Id=9606 Gene_Symbol=PSMA7 Isoform 1 of Proteasome subunit alpha type-7

Match to Query 14716: 1633.860668 from(817.937610,2+)
Title: 090324LimSK_Exosome1_B.10749.10749.2.dta
Data file C:\Temp\Mascoti\LSK_2D\090324LimSK_Exosome1_A_2D.mgf

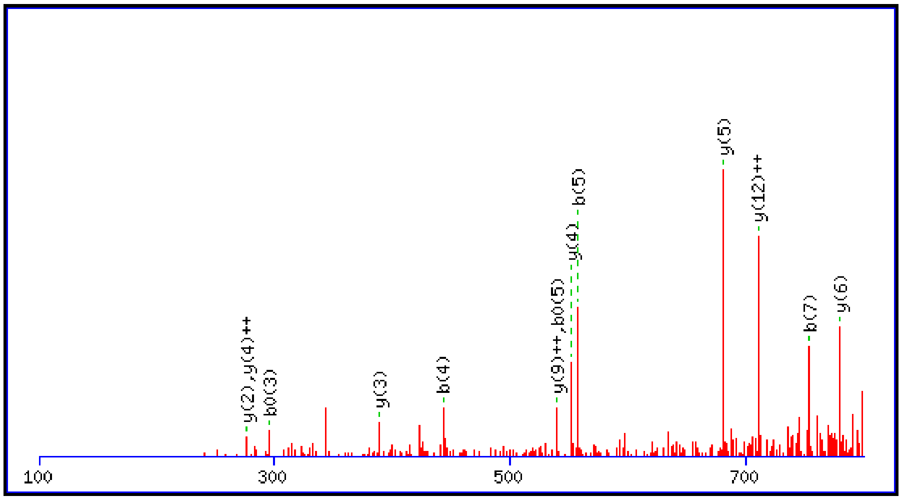

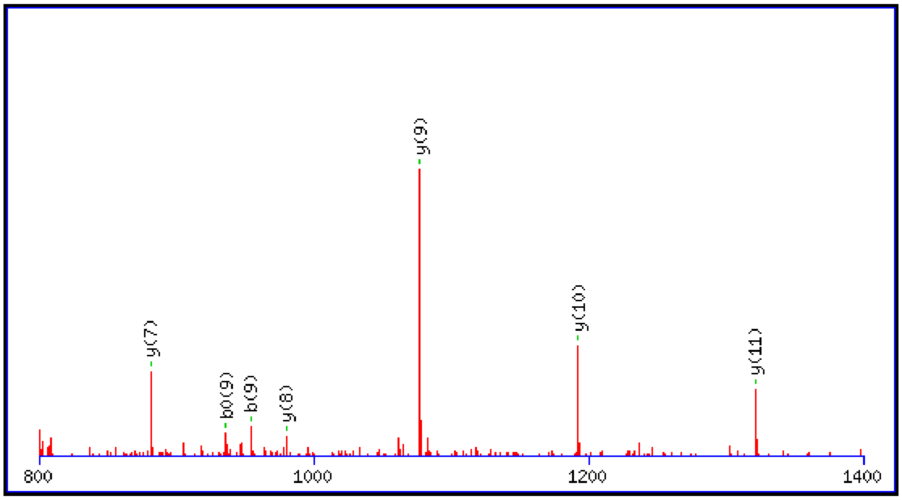

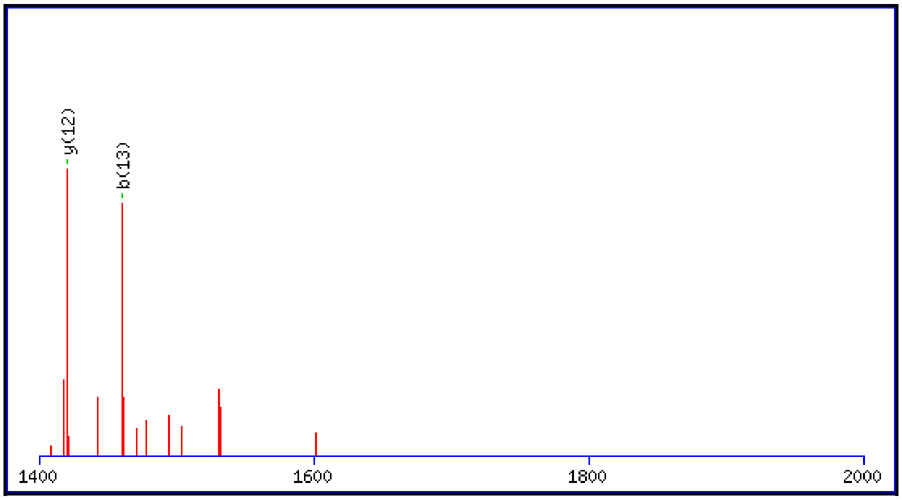

Monoisotopic mass of neutral peptide Mr(calc): 1633.8563
Fixed modifications: Carbamidomethyl (C)
Ions Score: 95 **Expect:** 4.1e-008

Matches (Bold Red): 22/126 fragment ions using 28 most intense peaks

#	b	b^{++}	b^{0}	b^{0++}	Seq.	y	y^{++}	y*	y^{*++}	y^{0}	y^{0++}	#
1	114.0913	57.5493			L							14
2	215.1390	108.0731	197.1285	99.0679	T	1521.7795	761.3934	1504.7530	752.8801	1503.7690	752.3881	13
3	314.2074	157.6074	**296.1969**	148.6021	V	**1420.7318**	**710.8696**	1403.7053	702.3563	1402.7213	701.8643	12
4	**443.2500**	222.1287	425.2395	213.1234	E	**1321.6634**	661.3353	1304.6369	652.8221	1303.6529	652.3301	11
5	**558.2770**	279.6421	**540.2664**	270.6368	D	**1192.6208**	596.8141	1175.5943	588.3008	1174.6103	587.8088	10
6	655.3297	328.1685	637.3192	319.1632	P	**1077.5939**	**539.3006**	1060.5673	530.7873	1059.5833	530.2953	9
7	**754.3981**	377.7027	736.3876	368.6974	V	**980.5411**	490.7742	963.5146	482.2609	962.5306	481.7689	8
8	855.4458	428.2266	837.4353	419.2213	T	**881.4727**	441.2400	864.4462	432.7267	863.4621	432.2347	7
9	**954.5142**	477.7608	**936.5037**	468.7555	V	**780.4250**	390.7162	763.3985	382.2029	762.4145	381.7109	6
10	1083.5568	542.2821	1065.5463	533.2768	E	**681.3566**	341.1819	664.3301	332.6687	663.3461	332.1767	5
11	1246.6202	623.8137	1228.6096	614.8084	Y	**552.3140**	**276.6607**	535.2875	268.1474	534.3035	267.6554	4
12	1359.7042	680.3558	1341.6937	671.3505	I	**389.2507**	195.1290	372.2241	186.6157	371.2401	186.1237	3
13	**1460.7519**	730.8796	1442.7413	721.8743	T	**276.1666**	138.5870	259.1401	130.0737	258.1561	129.5817	2
14					R	175.1190	88.0631	158.0924	79.5498			1

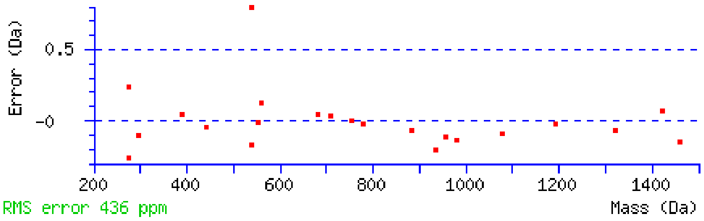

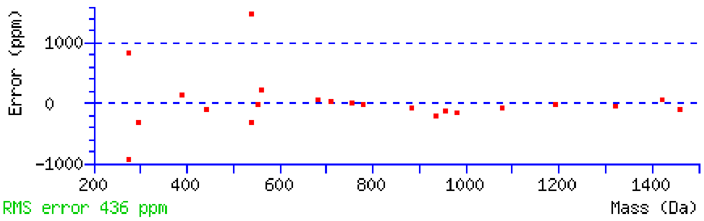

All matches to this query

Score	Mr(calc):	Delta	Sequence
95.0	1633.8563	0.0044	LTVEDPVTVEYITR
8.5	1632.8528	1.0079	TLNINTKYYVLVI
7.0	1632.8528	1.0079	TLNINTKYYVLVI
6.3	1632.8528	1.0079	TLNINTKYYVLVI
4.2	1633.8610	-0.0003	NRLVNIMPYELTR
2.8	1633.8480	0.0126	EKLTLLHYDPVVK
2.4	1631.8631	1.9976	VLETLVTVAEEHHR
2.3	1631.8661	1.9946	QVRFRLTPPSPVR
2.3	1633.8552	0.0054	ESAGGDLLLALLARR
0.8	1631.8494	2.0113	GTDLPWGPVIFKMR

Spectrum No: 19; Query: 15299; Rank: 1

Peptide View

MS/MS Fragmentation of **LYQTDPSGTYHAWK**
Found in **IPI00024175**, Tax_Id=9606 Gene_Symbol=PSMA7 Isoform 1 of Proteasome subunit alpha type-7

Match to Query 15299: 1665.783648 from(833.899100,2+)
Title: 090324LimSK_Exosome1_A.4426.4426.2.dta
Data file C:\Temp\Mascoti\LSK_2D\090324LimSK_Exosome1_A_2D.mgf

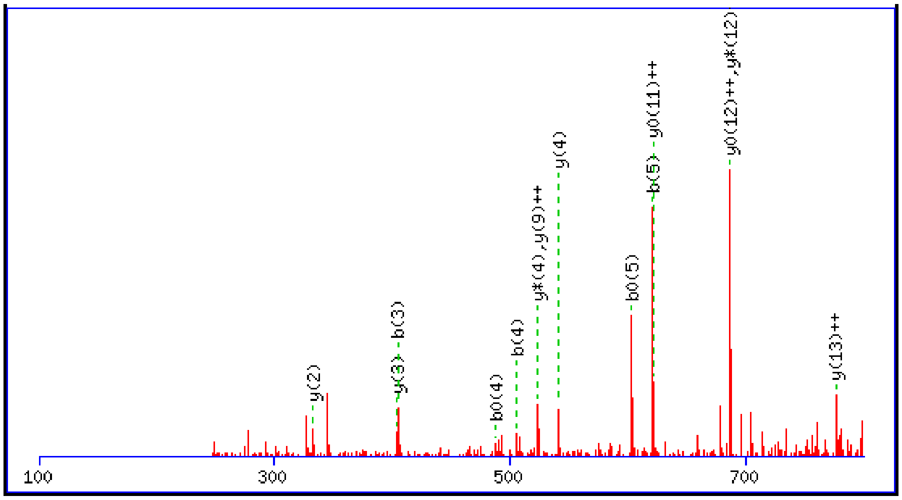

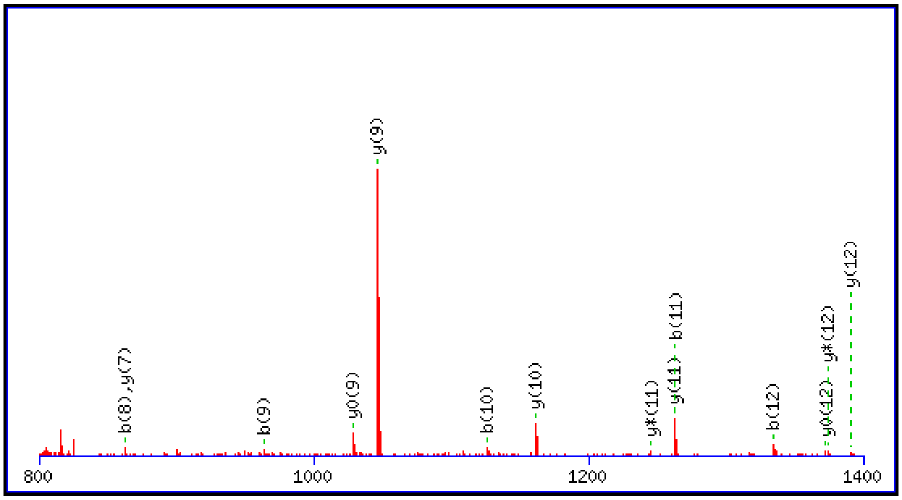

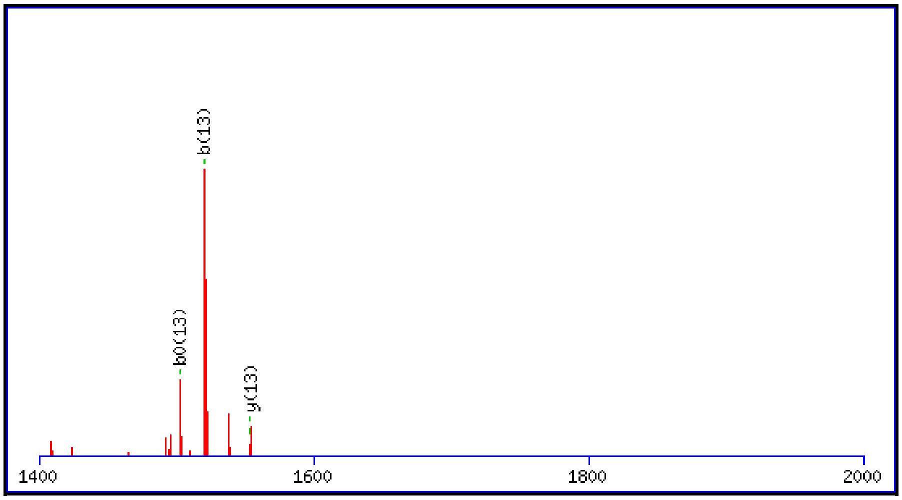

Monoisotopic mass of neutral peptide Mr(calc): 1665.7787
Fixed modifications: Carbamidomethyl (C)
Ions Score: 60 **Expect:** 0.00024
Matches (Bold Red): 33/136 fragment ions using 52 most intense peaks

#	b	b^{++}	b*	b^{*++}	b^0	b^{0++}	Seq.	y	y^{++}	y*	y^{*++}	y^0	y^{0++}	#
1	114.0913	57.5493					L							14
2	277.1547	139.0810					Y	**1553.7019**	**777.3546**	1536.6754	768.8413	1535.6914	768.3493	13
3	**405.2132**	203.1103	388.1867	194.5970			Q	**1390.6386**	695.8229	**1373.6121**	**687.3097**	**1372.6280**	**686.8177**	12
4	**506.2609**	253.6341	489.2344	245.1208	**488.2504**	244.6288	T	**1262.5800**	631.7937	**1245.5535**	623.2804	1244.5695	**622.7884**	11
5	**621.2879**	311.1476	604.2613	302.6343	**603.2773**	302.1423	D	**1161.5324**	581.2698	1144.5058	572.7565	1143.5218	572.2645	10
6	718.3406	359.6740	701.3141	351.1607	700.3301	350.6687	P	**1046.5054**	**523.7563**	1029.4789	515.2431	**1028.4948**	514.7511	9

**7**	805.3727	403.1900	788.3461	394.6767	787.3621	394.1847	**S**	949.4526	475.2300	932.4261	466.7167	931.4421	466.2247	**8**
**8**	**862.3941**	431.7007	845.3676	423.1874	844.3836	422.6954	**G**	**862.4206**	431.7139	845.3941	423.2007	844.4100	422.7087	**7**
**9**	**963.4418**	482.2245	946.4153	473.7113	945.4312	473.2193	**T**	805.3992	403.2032	788.3726	394.6899	787.3886	394.1979	**6**
**10**	**1126.5051**	563.7562	1109.4786	555.2429	1108.4946	554.7509	**Y**	704.3515	352.6794	**687.3249**	344.1661			**5**
**11**	**1263.5640**	632.2857	1246.5375	623.7724	**1245.5535**	623.2804	**H**	**541.2881**	271.1477	**524.2616**	262.6344			**4**
**12**	**1334.6012**	667.8042	1317.5746	659.2909	1316.5906	658.7989	**A**	**404.2292**	202.6183	387.2027	194.1050			**3**
**13**	**1520.6805**	760.8439	1503.6539	752.3306	**1502.6699**	751.8386	**W**	**333.1921**	167.0997	316.1656	158.5864			**2**
**14**							**K**	147.1128	74.0600	130.0863	65.5468			**1**

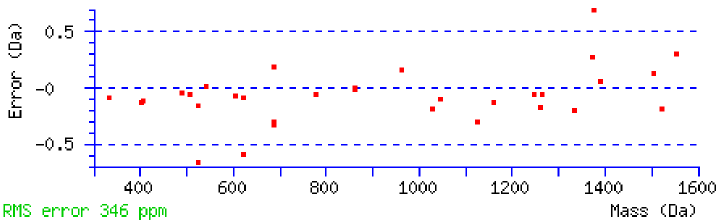

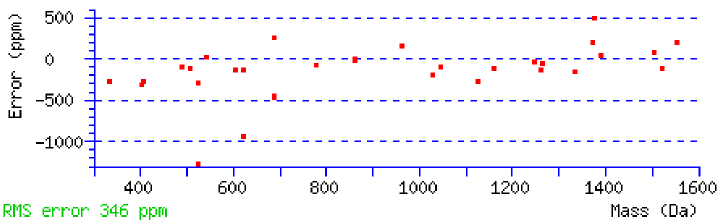

All matches to this query

Score	Mr(calc):	Delta	Sequence
59.6	1665.7787	0.0049	LYQTDPSGTYHAWK
14.0	1665.7974	-0.0138	EETKAEAPLLTQTR
12.4	1665.7767	0.0069	MEIDDLASNVETVSK
9.3	1664.7658	1.0178	EESLATEPAPGTQKK
8.2	1665.7954	-0.0117	GSSVEMLQDVIDVMK
7.9	1665.7702	0.0135	MNDRLGEDESLLMK
5.7	1665.7876	-0.0039	GHIPTPSRSESKYK
5.7	1665.7876	-0.0039	GHIPTPSRSESKYK
5.7	1665.7876	-0.0039	GHIPTPSRSESKYK
5.6	1664.7885	0.9952	LVETMYFKGIEAGK

Spectrum No: 20; Query: 16765; Rank: 1

Peptide View

MS/MS Fragmentation of **NYTDEAIETDDLTIK**
Found in **IPI00024175**, Tax_Id=9606 Gene_Symbol=PSMA7 Isoform 1 of Proteasome subunit alpha type-7

Match to Query 16765: 1739.812268 from(870.913410,2+)
Title: 090324LimSK_Exosome1_B.8164.8164.2.dta
Data file C:\Temp\Mascoti\LSK_2D\090324LimSK_Exosome1_A_2D.mgf

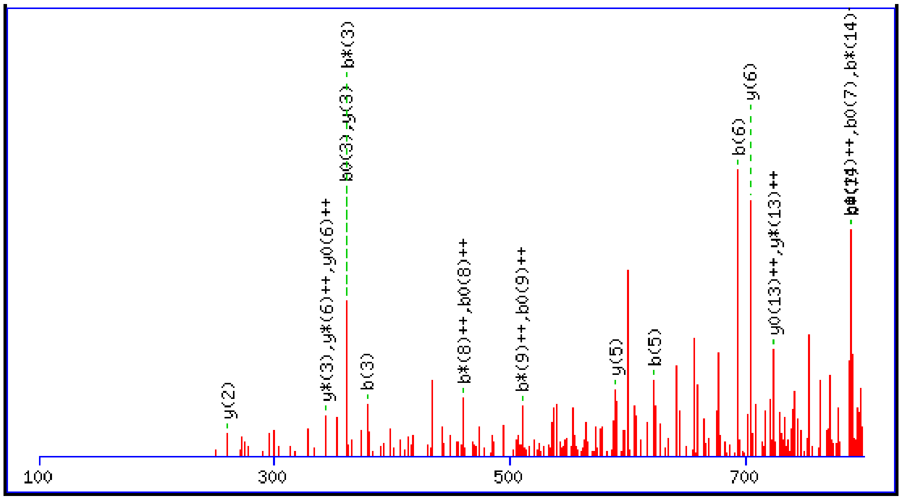

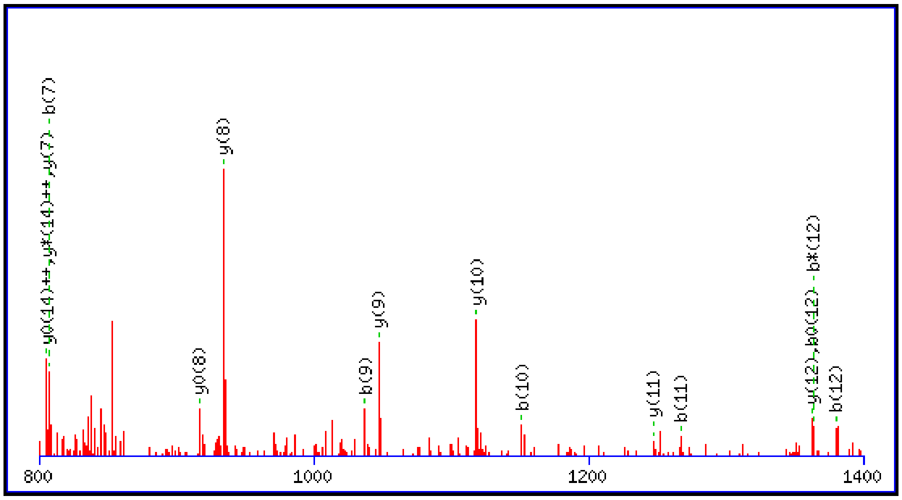

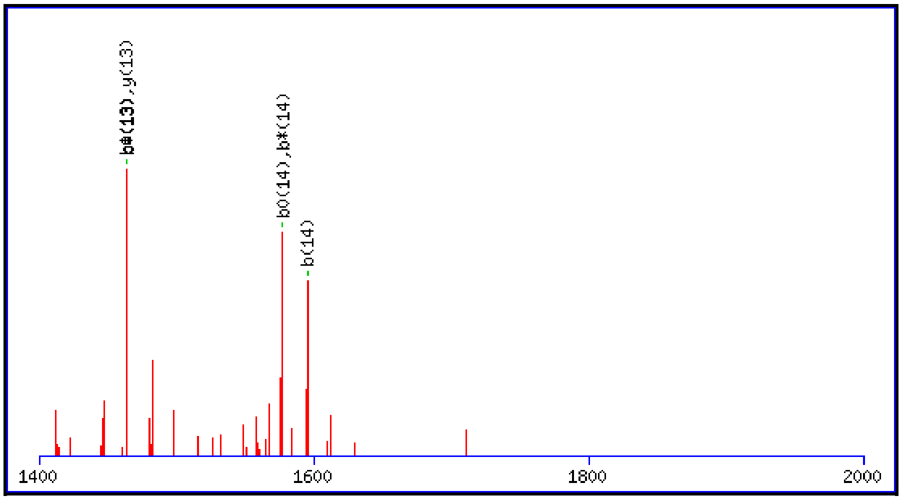

Monoisotopic mass of neutral peptide Mr(calc): 1739.8101
Fixed modifications: Carbamidomethyl (C)
Ions Score: 70 **Expect:** 2e-005
Matches (Bold Red): 45/160 fragment ions using 62 most intense peaks

#	b	b++	b*	b*++	b0	b0++	Seq.	y	y++	y*	y*++	y0	y0++	#
1	115.0502	58.0287	98.0237	49.5155			N							15
2	278.1135	139.5604	261.0870	131.0471			Y	1626.7745	813.8909	1609.7479	**805.3776**	1608.7639	**804.8856**	14
3	**379.1612**	190.0842	**362.1347**	181.5710	**361.1506**	181.0790	T	**1463.7112**	732.3592	1446.6846	**723.8459**	1445.7006	**723.3539**	13
4	494.1882	247.5977	477.1616	239.0844	476.1776	238.5924	D	**1362.6635**	681.8354	1345.6369	673.3221	1344.6529	672.8301	12
5	**623.2307**	312.1190	606.2042	303.6057	605.2202	303.1137	E	**1247.6365**	624.3219	1230.6100	615.8086	1229.6260	615.3166	11
6	**694.2679**	347.6376	677.2413	339.1243	676.2573	338.6323	A	**1118.5939**	559.8006	1101.5674	551.2873	1100.5834	550.7953	10

7	**807.3519**	404.1796	**790.3254**	395.6663	**789.3414**	395.1743	I	**1047.5568**	524.2821	1030.5303	515.7688	1029.5463	515.2768	9
8	936.3945	468.7009	919.3680	**460.1876**	918.3840	**459.6956**	E	**934.4728**	467.7400	917.4462	459.2267	**916.4622**	458.7347	8
9	**1037.4422**	519.2247	1020.4156	**510.7115**	1019.4316	**510.2195**	T	**805.4302**	403.2187	**788.4036**	394.7055	787.4196	394.2134	7
10	**1152.4691**	576.7382	1135.4426	568.2249	1134.4586	567.7329	D	**704.3825**	352.6949	687.3559	**344.1816**	686.3719	**343.6896**	6
11	**1267.4961**	634.2517	1250.4695	625.7384	1249.4855	625.2464	D	**589.3556**	295.1814	572.3290	286.6681	571.3450	286.1761	5
12	**1380.5801**	690.7937	**1363.5536**	682.2804	**1362.5696**	681.7884	L	474.3286	237.6679	457.3021	229.1547	456.3180	228.6627	4
13	1481.6278	741.3176	**1464.6013**	732.8043	**1463.6173**	732.3123	T	**361.2445**	181.1259	**344.2180**	172.6126	343.2340	172.1206	3
14	**1594.7119**	797.8596	**1577.6853**	**789.3463**	**1576.7013**	**788.8543**	I	**260.1969**	130.6021	243.1703	122.0888			2
15							K	147.1128	74.0600	130.0863	65.5468			1

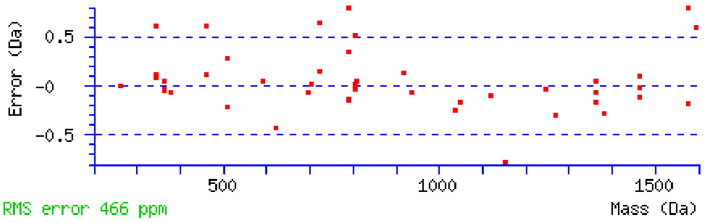

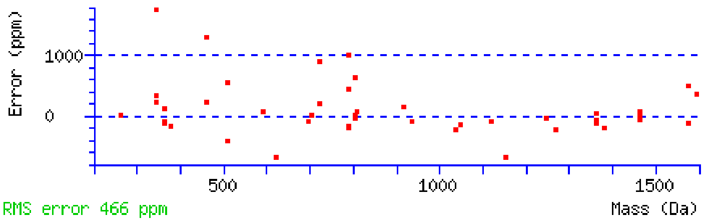

All matches to this query

Score	Mr(calc):	Delta	Sequence
70.5	1739.8101	0.0021	NYTDEAIETDDLTIK
16.9	1739.7953	0.0169	YLGKYACESNLKSK
12.5	1739.7953	0.0169	YLGKYACESNLKSK
9.9	1739.8222	-0.0100	MKQYASPMPTQTDVK
5.6	1739.7953	0.0169	YLGKYACESNLKSK
5.4	1739.8248	-0.0125	MLTGDTTVTSGDATVAGK
4.7	1739.8066	0.0057	DEGHRVLIFSQMTK
4.3	1739.8066	0.0057	DEGHRVLIFSQMTK
3.0	1738.8010	1.0113	KQSTQFSGGPTEDTEK
3.0	1739.8025	0.0097	AASANNEKMRLDVNK

Spectrum No: 21; Query: 10715; Rank: 1

Peptide View

MS/MS Fragmentation of **DIREEAEKYAK**
Found in **IPI00171199**, Tax_Id=9606 Gene_Symbol=PSMA3 Isoform 2 of Proteasome subunit alpha type-3

Match to Query 10715: 1350.679512 from(451.233780,3+)
Title: 090324LimSK_Exosome1_B.3246.3246.3.dta
Data file C:\Temp\Mascoti\LSK_2D\090324LimSK_Exosome1_A_2D.mgf

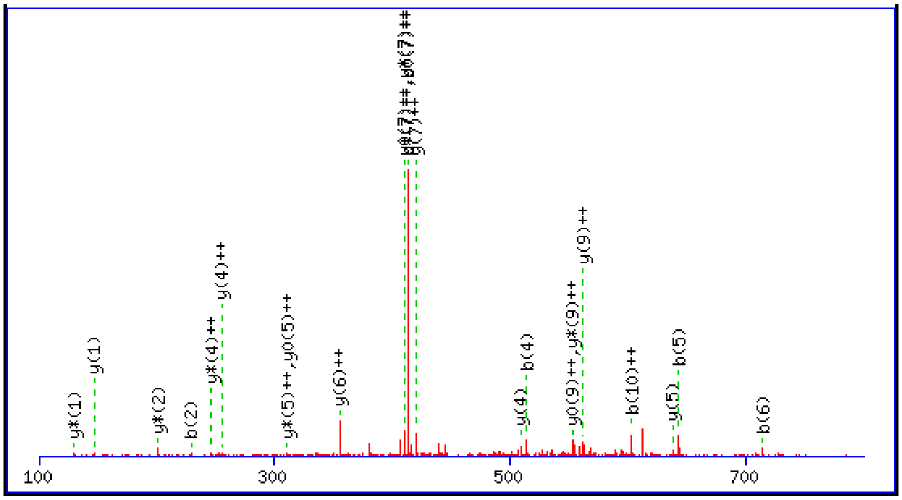

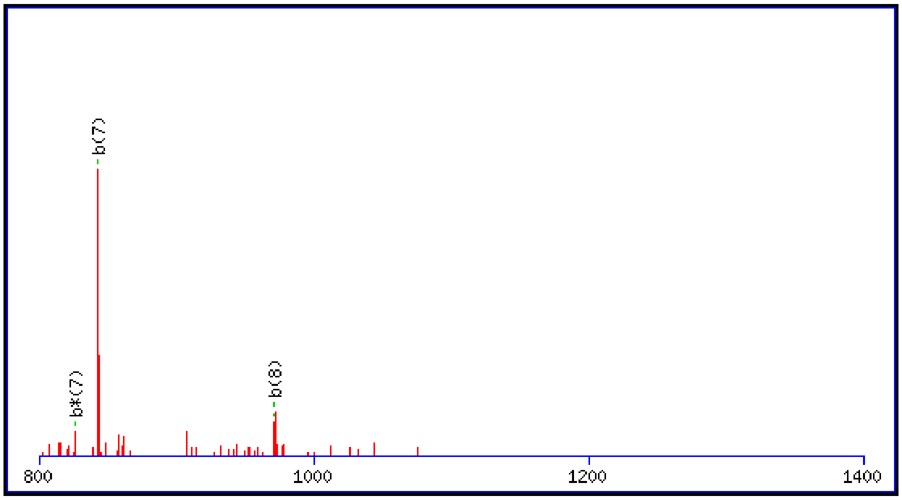

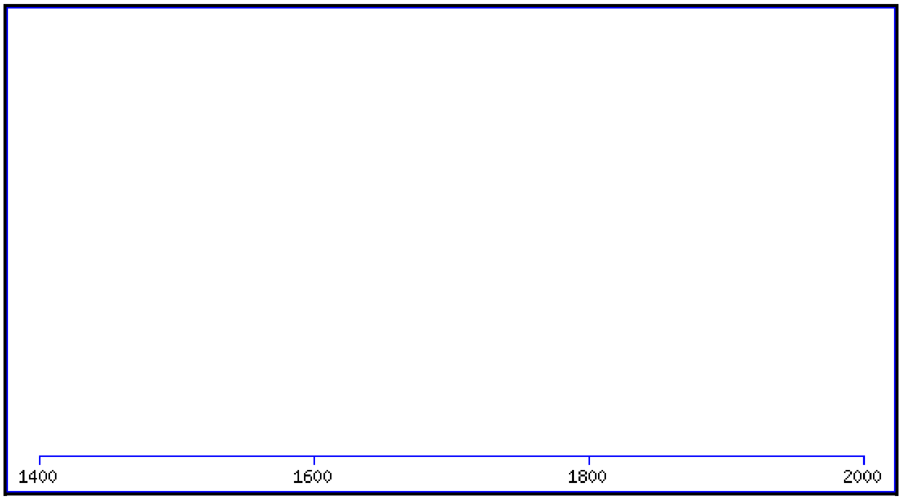

Monoisotopic mass of neutral peptide Mr(calc): 1350.6779
Fixed modifications: Carbamidomethyl (C)
Ions Score: 29 **Expect:** 0.2
Matches (Bold Red): 26/108 fragment ions using 45 most intense peaks

#	b	b^{++}	b*	b^{*++}	b^0	b^{0++}	Seq.	y	y^{++}	y*	y^{*++}	y^0	y^{0++}	#
1	116.0342	58.5207			98.0237	49.5155	D							11
2	**229.1183**	115.0628			211.1077	106.0575	I	1236.6583	618.8328	1219.6317	610.3195	1218.6477	609.8275	10
3	385.2194	193.1133	368.1928	184.6001	367.2088	184.1081	R	1123.5742	**562.2907**	1106.5477	**553.7775**	1105.5636	**553.2855**	9
4	**514.2620**	257.6346	497.2354	249.1214	496.2514	248.6293	E	967.4731	484.2402	950.4466	475.7269	949.4625	475.2349	8
5	**643.3046**	322.1559	626.2780	313.6427	625.2940	313.1506	E	838.4305	**419.7189**	821.4040	**411.2056**	820.4199	**410.7136**	7
6	**714.3417**	357.6745	697.3151	349.1612	696.3311	348.6692	A	709.3879	**355.1976**	692.3614	346.6843	691.3774	346.1923	6

7	**843.3843**	422.1958	**826.3577**	**413.6825**	825.3737	**413.1905**	E	**638.3508**	319.6790	621.3243	**311.1658**	620.3402	**310.6738**	5
8	**971.4793**	486.2433	954.4527	477.7300	953.4687	477.2380	K	**509.3082**	**255.1577**	492.2817	**246.6445**			4
9	1134.5426	567.7749	1117.5160	559.2617	1116.5320	558.7696	Y	381.2132	191.1103	364.1867	182.5970			3
10	1205.5797	**603.2935**	1188.5531	594.7802	1187.5691	594.2882	A	218.1499	109.5786	**201.1234**	101.0653			2
11							K	**147.1128**	74.0600	**130.0863**	65.5468			1

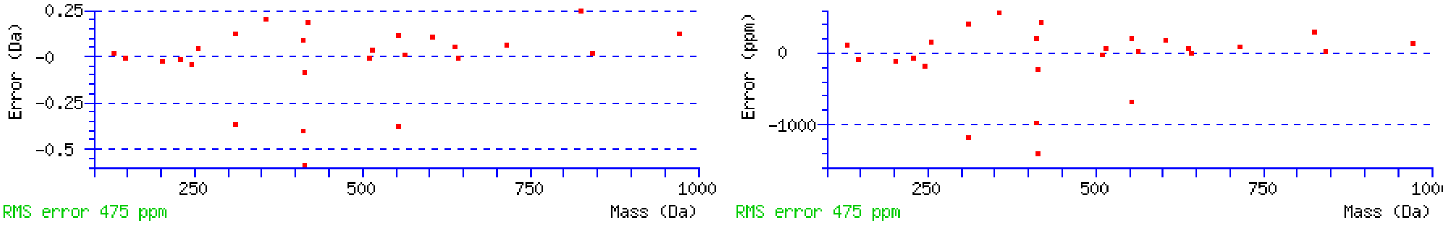

All matches to this query

Score	Mr(calc):	Delta	Sequence
29.2	1350.6779	0.0016	DIREEAEKYAK
7.6	1348.6768	2.0027	RSEEKAAAMAASK
7.5	1349.6827	0.9968	EEFTEALRDLK
6.7	1350.6721	0.0075	XFYRTLEGFR
6.0	1350.6908	-0.0113	EVLDASKALLGR
4.2	1350.6796	-0.0000	LIALGEENATLK
3.2	1349.6649	1.0146	RADEIYPVMEK
2.8	1348.6809	1.9986	LNQGQFIELCK
2.8	1349.6688	1.0108	SFSQSSSLSRHK
2.6	1349.6802	0.9994	YMFIRDYKSK

Spectrum No: 22; Query: 13721; Rank: 1

Peptide View

MS/MS Fragmentation of **SLADIAREEASNFR**
Found in **IPI00171199**, Tax_Id=9606 Gene_Symbol=PSMA3 Isoform 2 of Proteasome subunit alpha type-3

Match to Query 13721: 1577.793028 from(789.903790,2+)
Title: 090324LimSK_Exosome1_A.12008.12008.2.dta
Data file C:\Temp\Mascoti\LSK_2D\090324LimSK_Exosome1_A_2D.mgf

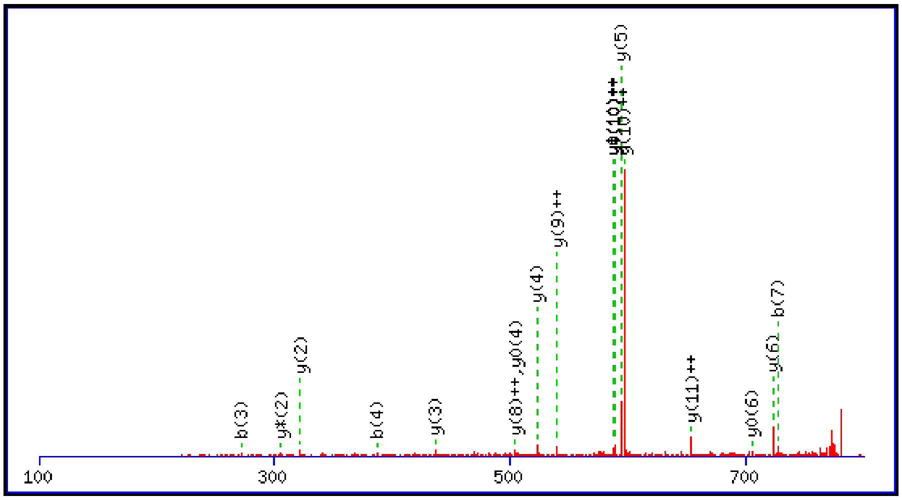

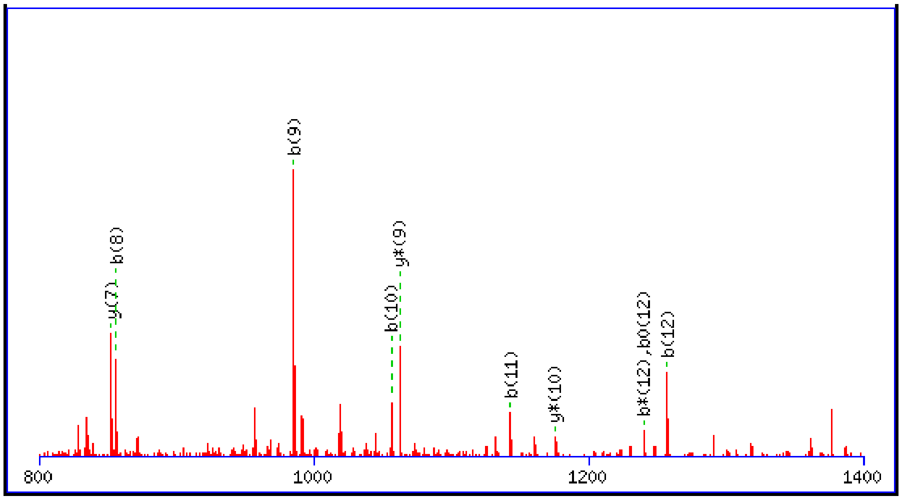

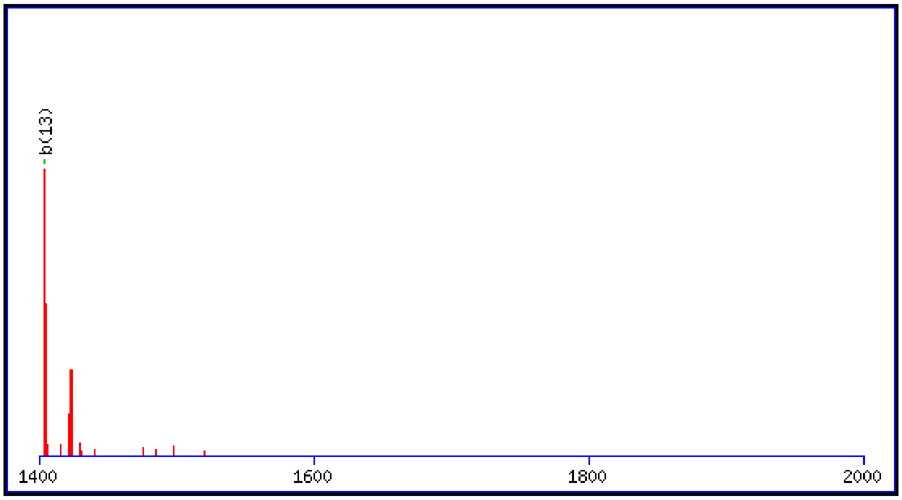

Monoisotopic mass of neutral peptide Mr(calc): 1577.7797
Fixed modifications: Carbamidomethyl (C)
Ions Score: 64 **Expect:** 7.6e-005
Matches (Bold Red): 28/138 fragment ions using 42 most intense peaks

#	b	b^{++}	b*	b^{*++}	b^0	b^{0++}	Seq.	y	y^{++}	y*	y^{*++}	y^0	y^{0++}	#
1	88.0393	44.5233			70.0287	35.5180	S							14
2	201.1234	101.0653			183.1128	92.0600	L	1491.7550	746.3812	1474.7285	737.8679	1473.7445	737.3759	13
3	**272.1605**	136.5839			254.1499	127.5786	A	1378.6710	689.8391	1361.6444	681.3258	1360.6604	680.8338	12
4	**387.1874**	194.0974			369.1769	185.0921	D	1307.6339	**654.3206**	1290.6073	645.8073	1289.6233	645.3153	11
5	500.2715	250.6394			482.2609	241.6341	I	1192.6069	**596.8071**	**1175.5804**	**588.2938**	1174.5963	**587.8018**	10
6	571.3086	286.1579			553.2980	277.1527	A	1079.5228	**540.2651**	**1062.4963**	531.7518	1061.5123	531.2598	9
7	**727.4097**	364.2085	710.3832	355.6952	709.3991	355.2032	R	1008.4857	**504.7465**	991.4592	496.2332	990.4752	495.7412	8
8	**856.4523**	428.7298	839.4258	420.2165	838.4417	419.7245	E	**852.3846**	426.6959	835.3581	418.1827	834.3741	417.6907	7
9	**985.4949**	493.2511	968.4684	484.7378	967.4843	484.2458	E	**723.3420**	362.1747	706.3155	353.6614	**705.3315**	353.1694	6
10	**1056.5320**	528.7696	1039.5055	520.2564	1038.5214	519.7644	A	**594.2994**	297.6534	577.2729	289.1401	576.2889	288.6481	5
11	**1143.5640**	572.2857	1126.5375	563.7724	1125.5535	563.2804	S	**523.2623**	262.1348	506.2358	253.6215	**505.2518**	253.1295	4
12	**1257.6070**	629.3071	**1240.5804**	620.7938	**1239.5964**	620.3018	N	**436.2303**	218.6188	419.2037	210.1055			3
13	**1404.6754**	702.8413	1387.6488	694.3281	1386.6648	693.8360	F	**322.1874**	161.5973	**305.1608**	153.0840			2
14							R	175.1190	88.0631	158.0924	79.5498			1

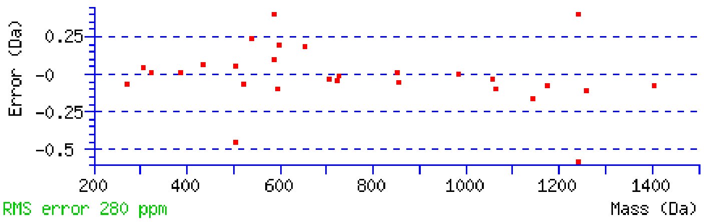

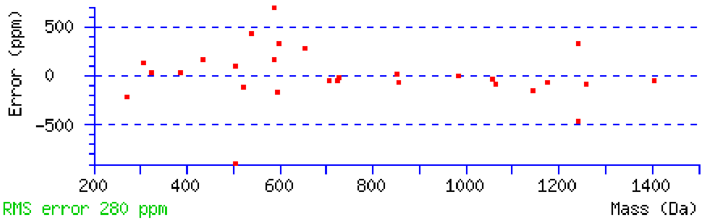

All matches to this query

Score	Mr(calc):	Delta	Sequence
63.7	1577.7797	0.0133	SLADIAREEASNFR
13.3	1577.8022	-0.0092	AAEVPGAQGQQGPRGR
8.5	1575.7893	2.0038	DGPPPGAEIPTLAGER
6.5	1577.7861	0.0069	GPSCRSSLLRPIR
6.2	1577.7997	-0.0067	RRPWAPPAASGCPR
6.1	1577.7861	0.0069	GPSCRSSLLRPIR
6.1	1577.7926	0.0004	EQRQTPGKGLISGK
3.6	1577.7861	0.0069	GPSCRSSLLRPIR
3.6	1577.7787	0.0143	QAASNRRAQELVR
3.0	1577.7959	-0.0029	MGFKHPKIMGNFR

Spectrum No: 23; Query: 8790; Rank: 1

Peptide View

MS/MS Fragmentation of **SNFGYNIPLK**
Found in **IPI00171199**, Tax_Id=9606 Gene_Symbol=PSMA3 Isoform 2 of Proteasome subunit alpha type-3

Match to Query 8790: 1151.598068 from(576.806310,2+)
Title: 090324LimSK_Exosome1_A.7793.7793.2.dta
Data file C:\Temp\Mascoti\LSK_2D\090324LimSK_Exosome1_A_2D.mgf

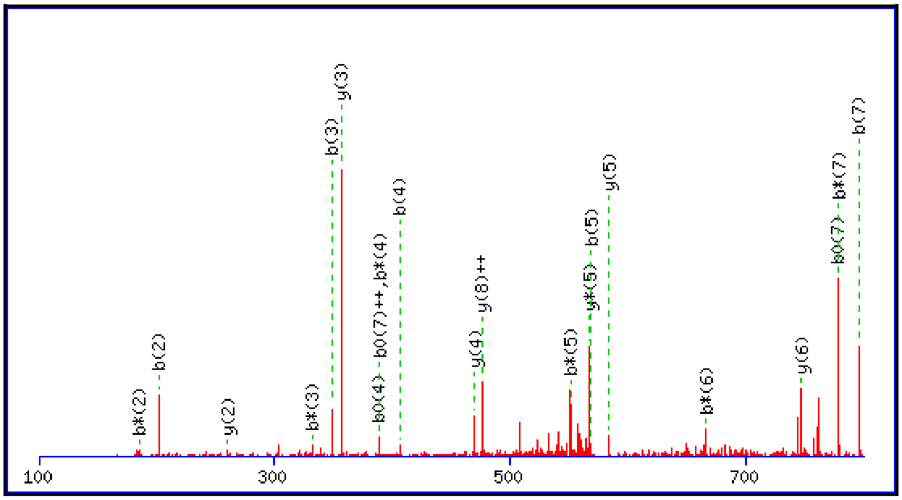

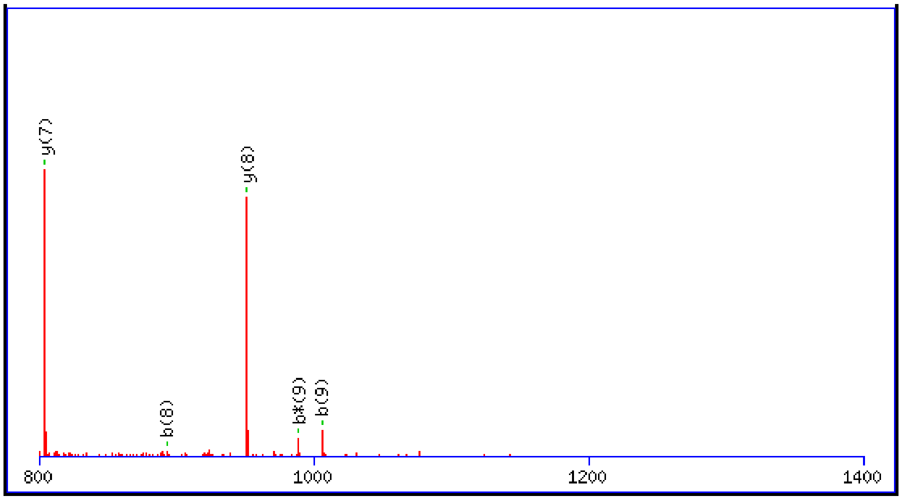

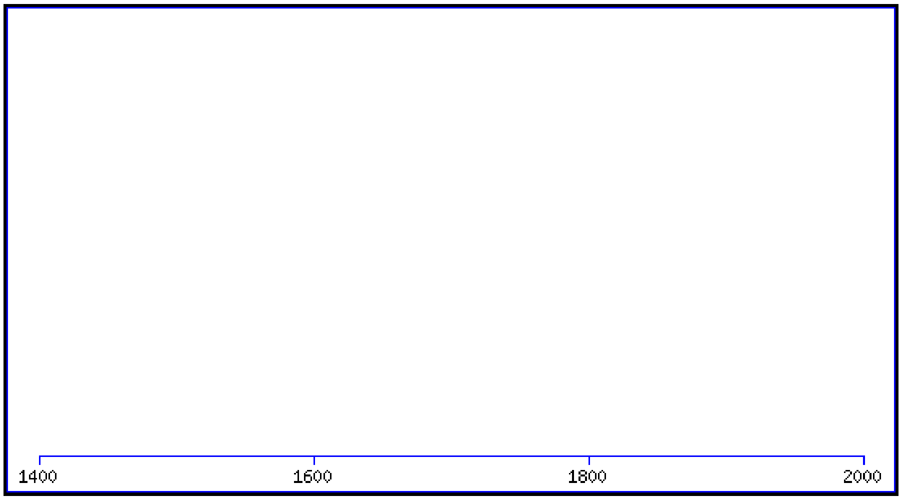

Monoisotopic mass of neutral peptide Mr(calc): 1151.5975
Fixed modifications: Carbamidomethyl (C)
Ions Score: 44 **Expect:** 0.0064
Matches (Bold Red): 26/88 fragment ions using 45 most intense peaks

#	b	b^{++}	b*	b^{*++}	b^0	b^{0++}	Seq.	y	y^{++}	y*	y^{*++}	#
1	88.0393	44.5233			70.0287	35.5180	S					10
2	**202.0822**	101.5448	**185.0557**	93.0315	184.0717	92.5395	N	1065.5728	533.2900	1048.5462	524.7767	9
3	**349.1506**	175.0790	**332.1241**	166.5657	331.1401	166.0737	F	**951.5298**	**476.2686**	934.5033	467.7553	8
4	**406.1721**	203.5897	**389.1456**	195.0764	**388.1615**	194.5844	G	**804.4614**	402.7343	787.4349	394.2211	7
5	**569.2354**	285.1214	**552.2089**	276.6081	551.2249	276.1161	Y	**747.4400**	374.2236	730.4134	365.7103	6
6	683.2784	342.1428	**666.2518**	333.6295	665.2678	333.1375	N	**584.3766**	292.6919	**567.3501**	284.1787	5
7	**796.3624**	398.6849	**779.3359**	390.1716	**778.3519**	**389.6796**	I	**470.3337**	235.6705	453.3071	227.1572	4
8	**893.4152**	447.2112	876.3886	438.6980	875.4046	438.2060	P	**357.2496**	179.1285	340.2231	170.6152	3
9	**1006.4993**	503.7533	**989.4727**	495.2400	988.4887	494.7480	L	**260.1969**	130.6021	243.1703	122.0888	2
10							K	147.1128	74.0600	130.0863	65.5468	1

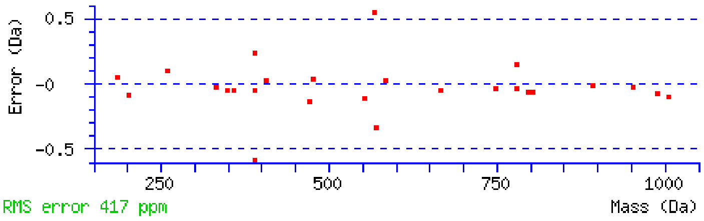

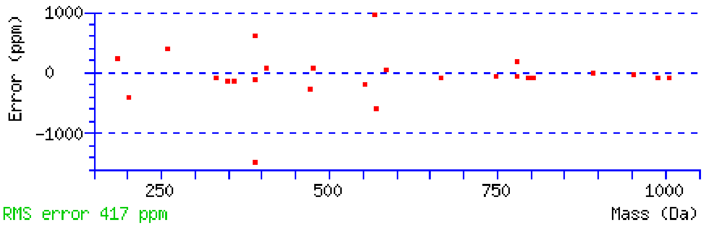

All matches to this query

Score	Mr(calc):	Delta	Sequence
43.8	1151.5975	0.0006	SNFGYNIPLK
11.4	1151.6063	-0.0083	KLDQTIARK
6.2	1150.5860	1.0121	LQDGRITIR
6.0	1151.6063	-0.0083	RGELQTKLK
4.6	1151.5896	0.0085	MEYQLLDIK
4.5	1150.5860	1.0121	SPRVKIDTR
4.2	1151.6064	-0.0083	NVLRTTQLK
3.1	1150.5860	1.0121	NLQLGVGRSK
3.0	1151.5951	0.0029	SVLAVGTANIK
3.0	1150.5999	0.9982	EQLLVTIQK

Spectrum No: 24; Query: 10081; Rank: 1

Peptide View

MS/MS Fragmentation of **AINQGGLTSVAVR**
Found in **IPI00029623**, Tax_Id=9606 Gene_Symbol=PSMA6 Proteasome subunit alpha type-6

Match to Query 10081: 1284.716068 from(643.365310,2+)
Title: 090324LimSK_Exosome1_A.4273.4273.2.dta
Data file C:\Temp\Mascoti\LSK_2D\090324LimSK_Exosome1_A_2D.mgf

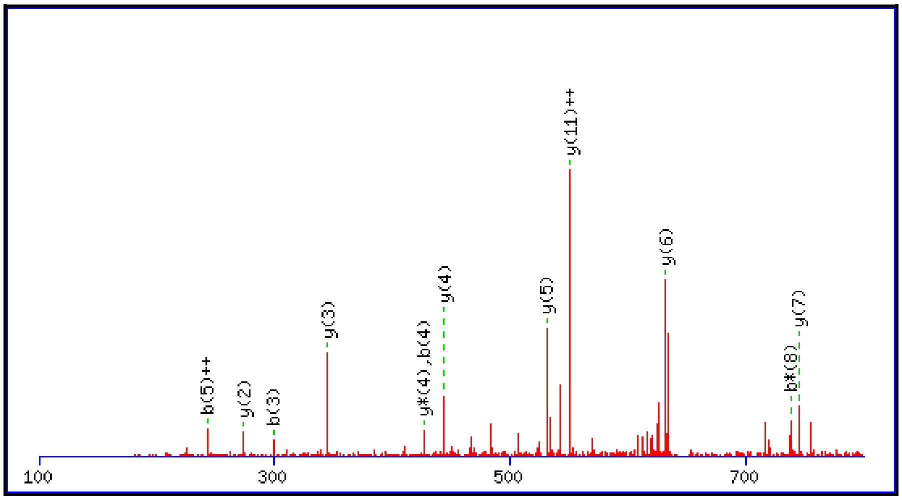

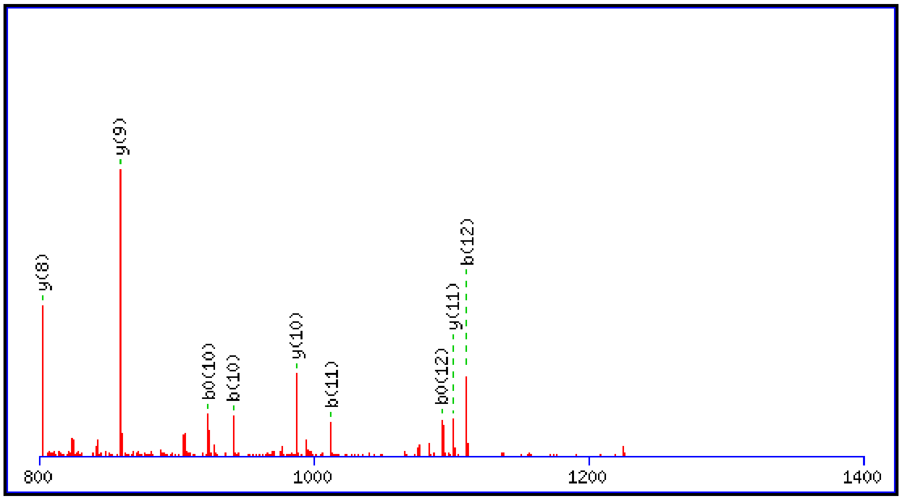

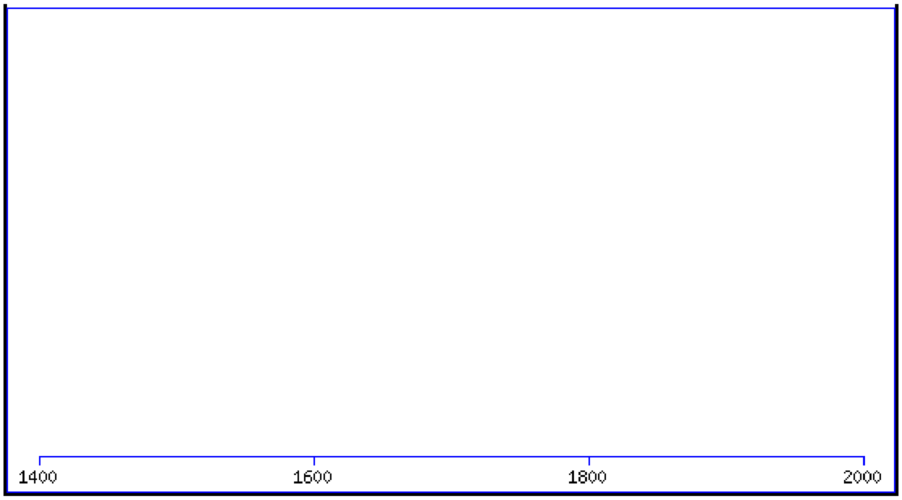

Monoisotopic mass of neutral peptide Mr(calc): 1284.7150
Fixed modifications: Carbamidomethyl (C)
Ions Score: 96 **Expect:** 2.4e-008
Matches (Bold Red): 21/118 fragment ions using 24 most intense peaks

#	b	b^{++}	b*	b^{*++}	b^0	b^{0++}	Seq.	y	y^{++}	y*	y^{*++}	y^0	y^{0++}	#
1	72.0444	36.5258					A							13
2	185.1285	93.0679					I	1214.6852	607.8462	1197.6586	599.3329	1196.6746	598.8409	12
3	**299.1714**	150.0893	282.1448	141.5761			N	**1101.6011**	**551.3042**	1084.5745	542.7909	1083.5905	542.2989	11
4	**427.2300**	214.1186	410.2034	205.6053			Q	**987.5582**	494.2827	970.5316	485.7694	969.5476	485.2774	10
5	484.2514	**242.6293**	467.2249	234.1161			G	**859.4996**	430.2534	842.4730	421.7402	841.4890	421.2482	9
6	541.2729	271.1401	524.2463	262.6268			G	**802.4781**	401.7427	785.4516	393.2294	784.4676	392.7374	8
7	654.3570	327.6821	637.3304	319.1688			L	**745.4567**	373.2320	728.4301	364.7187	727.4461	364.2267	7
8	755.4046	378.2060	**738.3781**	369.6927	737.3941	369.2007	T	**632.3726**	316.6899	615.3461	308.1767	614.3620	307.6847	6
9	842.4367	421.7220	825.4101	413.2087	824.4261	412.7167	S	**531.3249**	266.1661	514.2984	257.6528	513.3144	257.1608	5
10	**941.5051**	471.2562	924.4785	462.7429	**923.4945**	462.2509	V	**444.2929**	222.6501	**427.2663**	214.1368			4
11	**1012.5422**	506.7747	995.5156	498.2615	994.5316	497.7694	A	**345.2245**	173.1159	328.1979	164.6026			3
12	**1111.6106**	556.3089	1094.5841	547.7957	**1093.6000**	547.3037	V	**274.1874**	137.5973	257.1608	129.0840			2
13							R	175.1190	88.0631	158.0924	79.5498			1

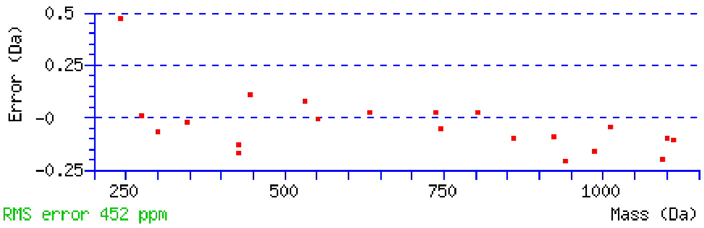

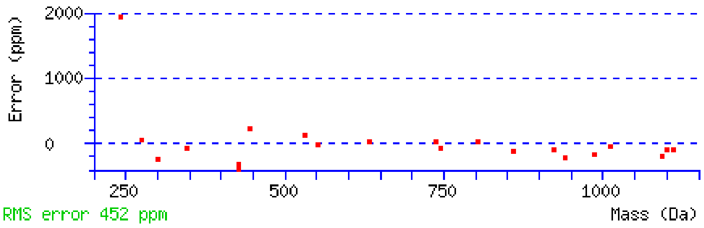

All matches to this query

Score	Mr(calc):	Delta	Sequence
95.6	1284.7150	0.0011	AINQGGLTSVAVR
9.0	1284.7262	-0.0101	LQEVRESIRR
5.9	1284.7150	0.0011	LRELVAGDSAVR
5.1	1284.7224	-0.0063	MKEEVKGIPVR
2.2	1284.7262	-0.0101	ELEQRVSLRR
1.9	1284.7051	0.0110	SWDVTRPLRR
0.6	1282.7033	2.0127	AAGPQAWSALLAK
0.4	1284.7262	-0.0101	RVREEAVTVAR

Spectrum No: 25; Query: 17306; Rank: 1

Peptide View

MS/MS Fragmentation of **ATAAGVKQTESTSFLEK**
Found in **IPI00029623**, Tax_Id=9606 Gene_Symbol=PSMA6 Proteasome subunit alpha type-6

Match to Query 17306: 1766.910582 from(589.977470,3+)
Title: 090324LimSK_Exosome1_B.2933.2933.3.dta
Data file C:\Temp\Mascoti\LSK_2D\090324LimSK_Exosome1_A_2D.mgf

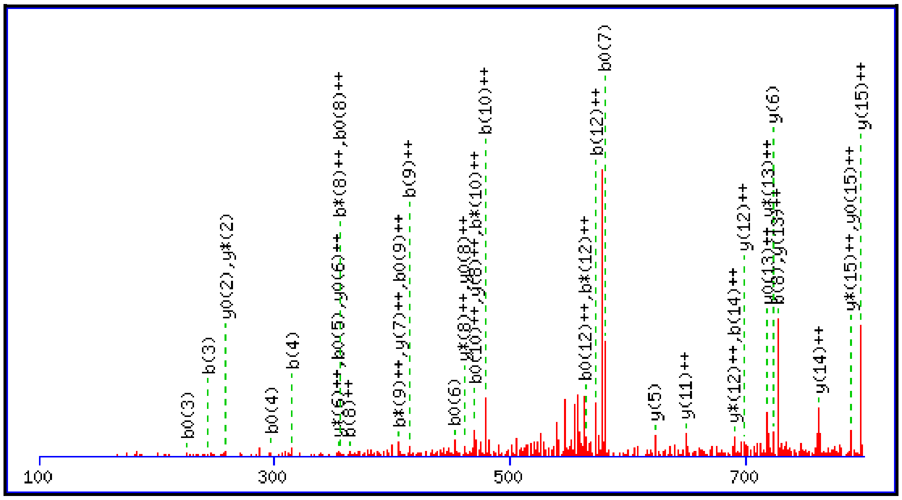

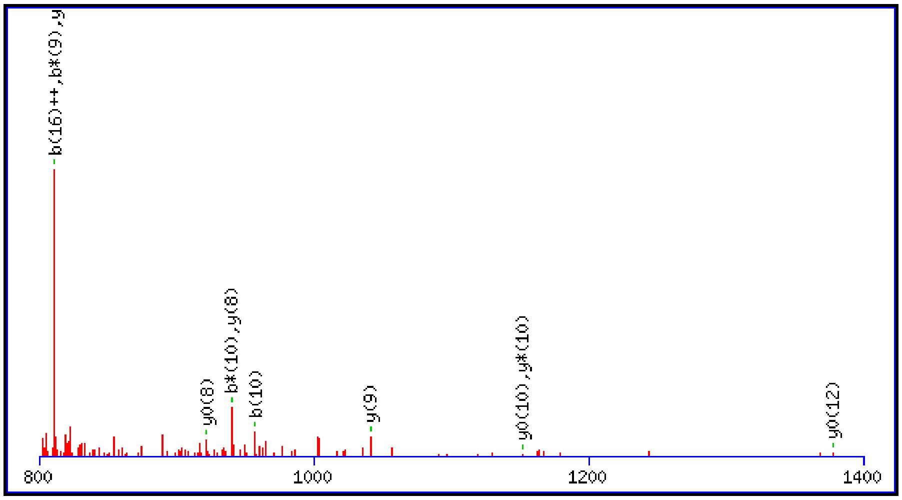

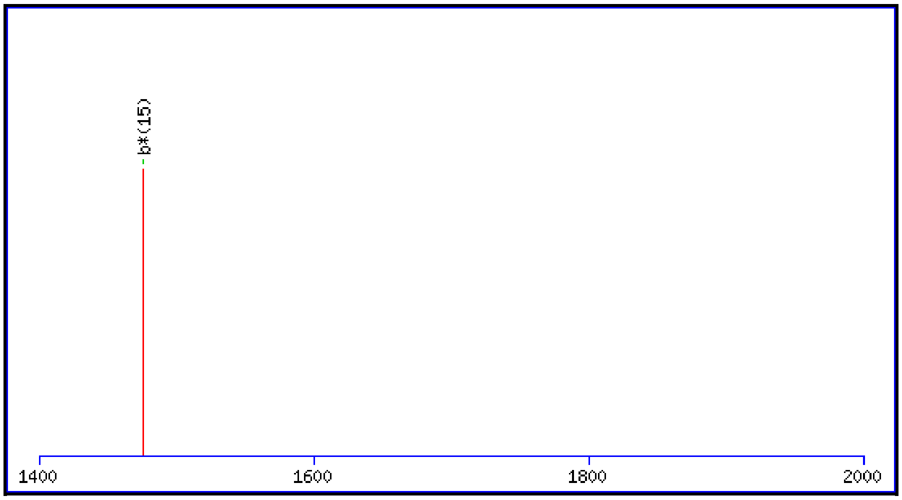

Monoisotopic mass of neutral peptide Mr(calc): 1766.9050

Fixed modifications: Carbamidomethyl (C)
Ions Score: 24 **Expect:** 0.62
Matches (Bold Red): 53/176 fragment ions using 110 most intense peaks

#	b	b^{++}	b*	b^{*++}	b^0	b^{0++}	Seq.	y	y^{++}	y*	y^{*++}	y^0	y^{0++}	#
1	72.0444	36.5258					A							17
2	173.0921	87.0497			155.0815	78.0444	T	1696.8752	848.9412	1679.8487	840.4280	1678.8646	839.9360	16
3	**244.1292**	122.5682			**226.1186**	113.5629	A	1595.8275	**798.4174**	1578.8010	**789.9041**	1577.8170	**789.4121**	15
4	**315.1663**	158.0868			**297.1557**	149.0815	A	1524.7904	**762.8988**	1507.7639	754.3856	1506.7799	753.8936	14
5	372.1878	186.5975			**354.1772**	177.5922	G	1453.7533	**727.3803**	1436.7268	**718.8670**	1435.7427	**718.3750**	13
6	471.2562	236.1317			**453.2456**	227.1264	V	1396.7318	**698.8696**	1379.7053	**690.3563**	**1378.7213**	689.8643	12
7	599.3511	300.1792	582.3246	291.6659	**581.3406**	291.1739	K	1297.6634	**649.3353**	1280.6369	640.8221	1279.6529	640.3301	11
8	**727.4097**	**364.2085**	710.3832	**355.6952**	709.3992	**355.2032**	Q	1169.5685	585.2879	**1152.5419**	576.7746	**1151.5579**	576.2826	10
9	828.4574	**414.7323**	**811.4308**	**406.2191**	810.4468	**405.7271**	T	**1041.5099**	521.2586	1024.4833	512.7453	1023.4993	512.2533	9
10	**957.5000**	**479.2536**	**940.4734**	**470.7404**	939.4894	**470.2483**	E	**940.4622**	**470.7347**	923.4357	**462.2215**	**922.4516**	**461.7295**	8
11	1044.5320	522.7696	1027.5055	514.2564	1026.5215	513.7644	S	**811.4196**	**406.2134**	794.3931	397.7002	793.4090	397.2082	7
12	1145.5797	**573.2935**	1128.5531	**564.7802**	1127.5691	**564.2882**	T	**724.3876**	362.6974	707.3610	**354.1842**	706.3770	**353.6921**	6
13	1232.6117	616.8095	1215.5852	608.2962	1214.6012	607.8042	S	**623.3399**	312.1736	606.3134	303.6603	605.3293	303.1683	5
14	1379.6801	**690.3437**	1362.6536	681.8304	1361.6696	681.3384	F	536.3079	268.6576	519.2813	260.1443	518.2973	259.6523	4
15	1492.7642	746.8857	**1475.7377**	738.3725	1474.7536	737.8805	L	389.2395	195.1234	372.2129	186.6101	371.2289	186.1181	3
16	1621.8068	**811.4070**	1604.7802	802.8938	1603.7962	802.4018	E	276.1554	138.5813	**259.1288**	130.0681	**258.1448**	129.5761	2
17							K	147.1128	74.0600	130.0863	65.5468			1

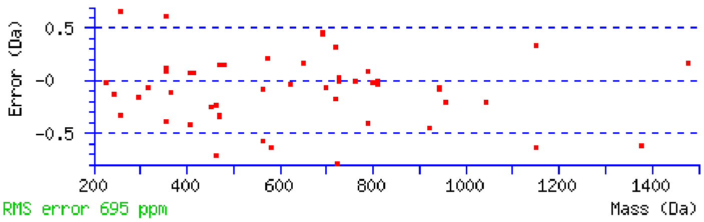

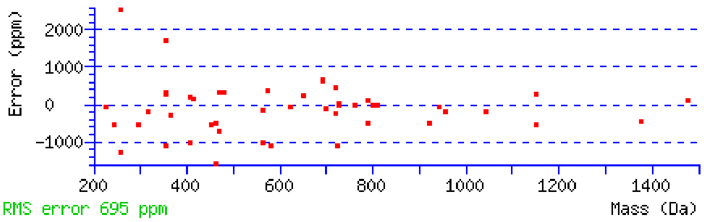

All matches to this query

Score	Mr(calc):	Delta	Sequence
24.4	1766.9050	0.0056	ATAAGVKQTESTSFLEK
8.9	1766.9080	0.0026	RSAGLELTILPGAFSR
8.3	1765.9210	0.9896	GSLGPTLTTEAPAAAQPGK
6.5	1764.9080	2.0026	KLLDADAAYEELMKR
4.0	1765.9128	0.9978	KLSTIHGKLQEAFSK
2.5	1766.9040	0.0066	NSIQLSAKTIKQNSR
1.7	1766.9066	0.0040	EEKETLLLEKQISK
1.6	1766.9067	0.0039	LVSDVTADSKVLEALK
1.6	1766.9001	0.0105	CDQLIKTKIQLEAK
1.5	1766.8938	0.0168	SDLGELDHVVEILVET

Spectrum No: 26; Query: 10583; Rank: 1

Peptide View

MS/MS Fragmentation of **CDPAGYYCGFK**
Found in **IPI00029623**, Tax_Id=9606 Gene_Symbol=PSMA6 Proteasome subunit alpha type-6

Match to Query 10583: 1336.526748 from(669.270650,2+)

Title: 090324LimSK_Exosome1_A.5110.5110.2.dta
Data file C:\Temp\Mascoti\LSK_2D\090324LimSK_Exosome1_A_2D.mgf

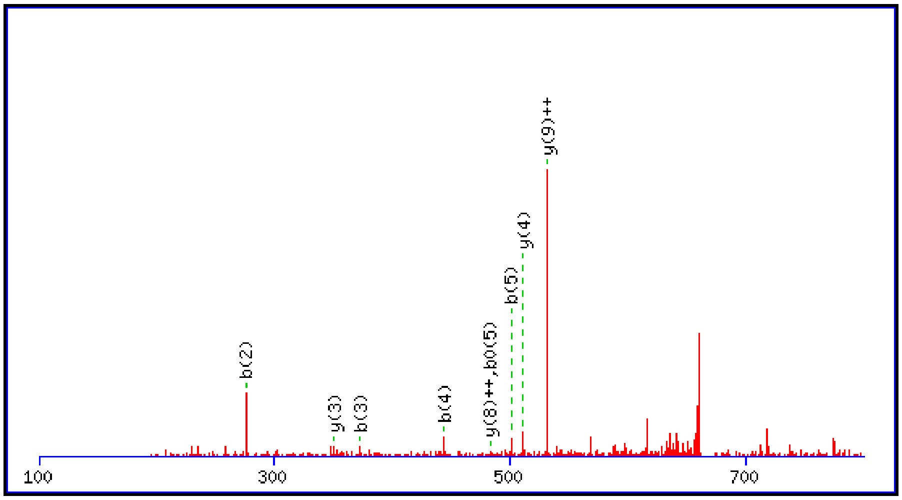

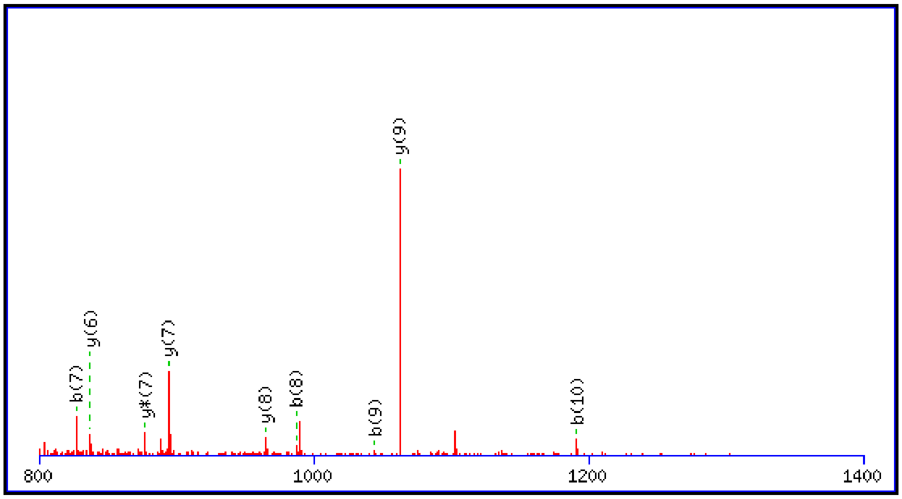

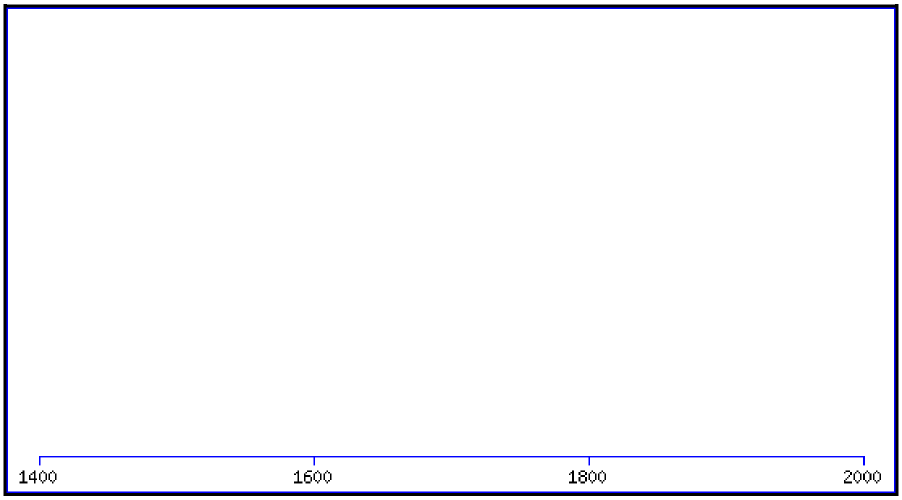

Monoisotopic mass of neutral peptide Mr(calc): 1336.5217
Fixed modifications: Carbamidomethyl (C)
Ions Score: 38 **Expect:** 0.017
Matches (Bold Red): 18/80 fragment ions using 46 most intense peaks

#	b	b^{++}	b^0	b^{0++}	Seq.	y	y^{++}	y*	y^{*++}	y^0	y^{0++}	#
1	161.0379	81.0226			C							11
2	**276.0649**	138.5361	258.0543	129.5308	D	1177.4983	589.2528	1160.4717	580.7395	1159.4877	580.2475	10
3	**373.1176**	187.0625	355.1071	178.0572	P	**1062.4713**	**531.7393**	1045.4448	523.2260			9
4	**444.1547**	222.5810	426.1442	213.5757	A	**965.4186**	**483.2129**	948.3920	474.6996			8

**5**	**501.1762**	251.0917	**483.1656**	242.0865	**G**	**894.3815**	447.6944	**877.3549**	439.1811			**7**
**6**	664.2395	332.6234	646.2290	323.6181	**Y**	**837.3600**	419.1836	820.3334	410.6704			**6**
**7**	**827.3029**	414.1551	809.2923	405.1498	**Y**	674.2967	337.6520	657.2701	329.1387			**5**
**8**	**987.3335**	494.1704	969.3230	485.1651	**C**	**511.2333**	256.1203	494.2068	247.6070			**4**
**9**	**1044.3550**	522.6811	1026.3444	513.6758	**G**	**351.2027**	176.1050	334.1761	167.5917			**3**
**10**	**1191.4234**	596.2153	1173.4128	587.2101	**F**	294.1812	147.5942	277.1547	139.0810			**2**
**11**					**K**	147.1128	74.0600	130.0863	65.5468			**1**

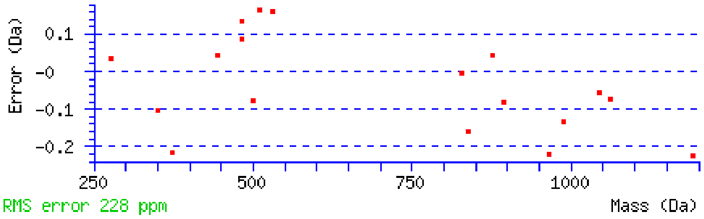

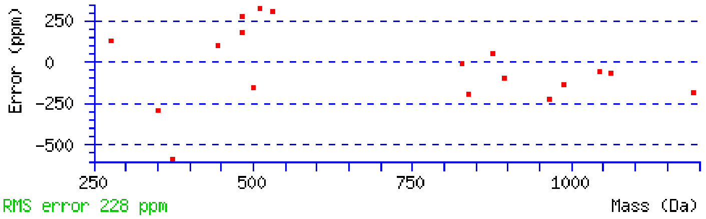

All matches to this query

Score	Mr(calc):	Delta	Sequence
38.0	1336.5217	0.0051	CDPAGYYCGFK
20.3	1336.5387	-0.0119	MKSTEVLPEK
14.7	1335.5331	0.9936	MQPAMMMFSSK
14.7	1335.5331	0.9936	MQPAMMMFSSK
14.0	1336.5257	0.0010	YAESLSEAMEK
12.7	1335.5331	0.9936	MQPAMMMFSSK
9.5	1336.5257	0.0010	YAESLSEAMEK
8.1	1336.5313	-0.0045	TGGTNTEKLEK
8.1	1336.5313	-0.0045	TGGTNTEKLEK
7.9	1336.5257	0.0010	YAESLSEAMEK

Spectrum No: 27; Query: 20522; Rank: 1

Peptide View

MS/MS Fragmentation of **GSSAGFDRHITIFSPEGR**
Found in **IPI00029623**, Tax_Id=9606 Gene_Symbol=PSMA6 Proteasome subunit alpha type-6

Match to Query 20522: 1933.945782 from(645.655870,3+)
Title: 090324LimSK_Exosome1_A.7753.7753.3.dta
Data file C:\Temp\Mascoti\LSK_2D\090324LimSK_Exosome1_A_2D.mgf

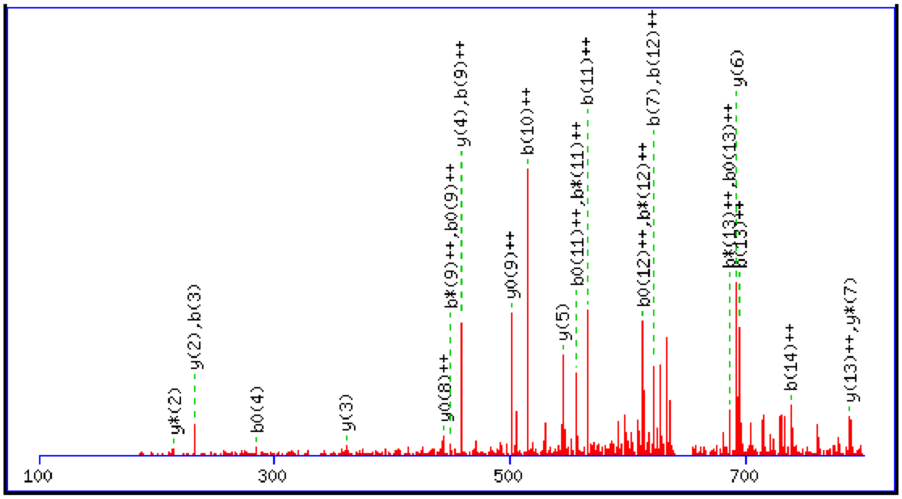

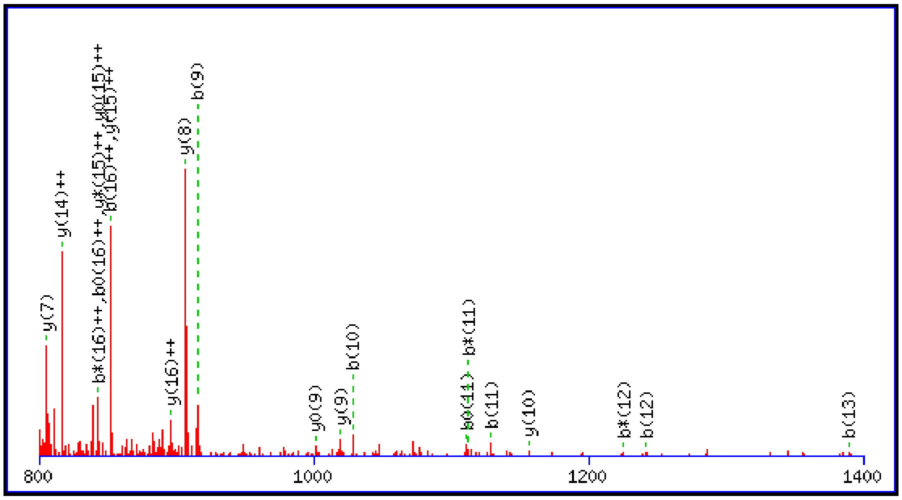

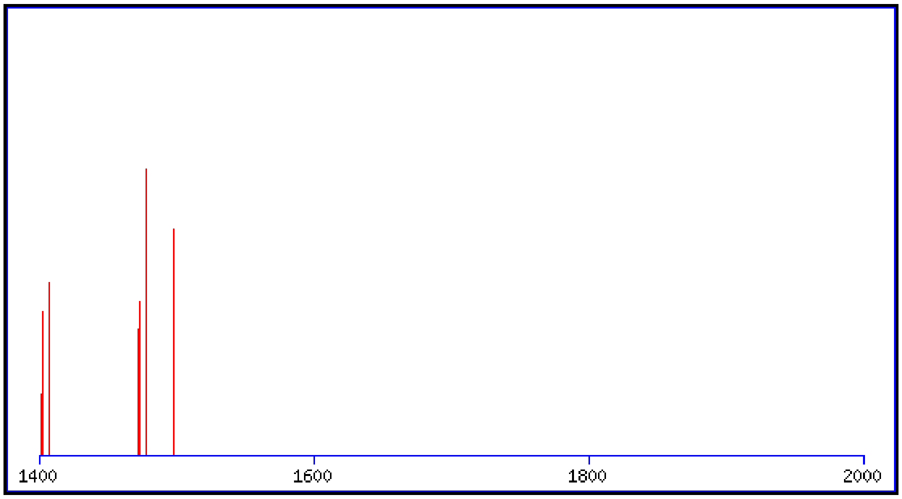

Monoisotopic mass of neutral peptide Mr(calc): 1932.9442
Fixed modifications: Carbamidomethyl (C)
Ions Score: 44 **Expect:** 0.0094
Matches (Bold Red): 48/184 fragment ions using 71 most intense peaks

#	b	b^{++}	b*	b^{*++}	b^0	b^{0++}	Seq.	y	y^{++}	y*	y^{*++}	y^0	y^{0++}	#
1	58.0287	29.5180					G							18
2	145.0608	73.0340			127.0502	64.0287	S	1876.9300	938.9687	1859.9035	930.4554	1858.9195	929.9634	17
3	**232.0928**	116.5500			214.0822	107.5448	S	1789.8980	**895.4526**	1772.8715	886.9394	1771.8874	886.4474	16
4	303.1299	152.0686			**285.1193**	143.0633	A	1702.8660	**851.9366**	1685.8394	**843.4234**	1684.8554	**842.9313**	15
5	360.1514	180.5793			342.1408	171.5740	G	1631.8289	**816.4181**	1614.8023	807.9048	1613.8183	807.4128	14
6	507.2198	254.1135			489.2092	245.1082	F	1574.8074	**787.9073**	1557.7809	779.3941	1556.7968	778.9021	13

7	622.2467	311.6270			604.2362	302.6217	D	1427.7390	714.3731	1410.7124	705.8599	1409.7284	705.3678	12
8	778.3478	389.6776	761.3213	381.1643	760.3373	380.6723	R	1312.7120	656.8597	1295.6855	648.3464	1294.7015	647.8544	11
9	915.4068	458.2070	898.3802	449.6937	897.3962	449.2017	H	1156.6109	578.8091	1139.5844	570.2958	1138.6004	569.8038	10
10	1028.4908	514.7490	1011.4643	506.2358	1010.4803	505.7438	I	1019.5520	510.2796	1002.5255	501.7664	1001.5415	501.2744	9
11	1129.5385	565.2729	1112.5119	556.7596	1111.5279	556.2676	T	906.4680	453.7376	889.4414	445.2243	888.4574	444.7323	8
12	1242.6226	621.8149	1225.5960	613.3016	1224.6120	612.8096	I	805.4203	403.2138	788.3937	394.7005	787.4097	394.2085	7
13	1389.6910	695.3491	1372.6644	686.8359	1371.6804	686.3438	F	692.3362	346.6717	675.3097	338.1585	674.3257	337.6665	6
14	1476.7230	738.8651	1459.6965	730.3519	1458.7124	729.8599	S	545.2678	273.1375	528.2413	264.6243	527.2572	264.1323	5
15	1573.7758	787.3915	1556.7492	778.8782	1555.7652	778.3862	P	458.2358	229.6215	441.2092	221.1083	440.2252	220.6162	4
16	1702.8184	851.9128	1685.7918	843.3995	1684.8078	842.9075	E	361.1830	181.0951	344.1565	172.5819	343.1724	172.0899	3
17	1759.8398	880.4235	1742.8133	871.9103	1741.8293	871.4183	G	232.1404	116.5738	215.1139	108.0606			2
18							R	175.1190	88.0631	158.0924	79.5498			1

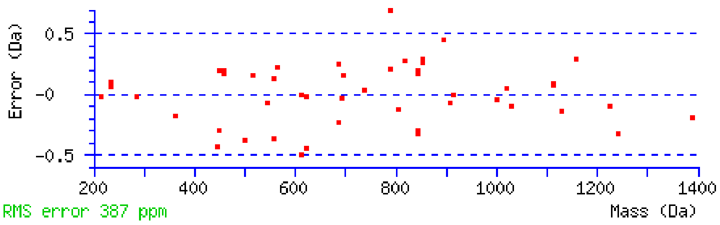

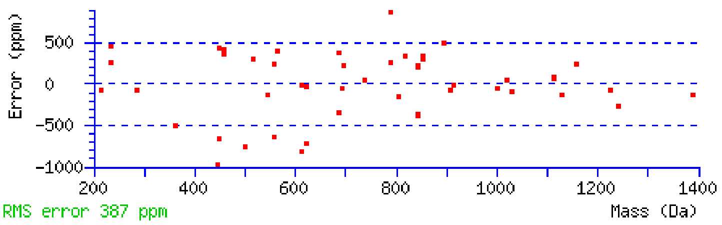

All matches to this query

Score	Mr(calc):	Delta	Sequence
44.0	1932.9442	1.0016	GSSAGFDRHITIFSPEGR
8.0	1932.9541	0.9917	HEGLDIIENAVDNLEPR
5.0	1931.9474	1.9983	YCLLRTLKQCQTLR
4.4	1932.9411	1.0047	RKGTANAQMPGNVTWMR
4.0	1933.9350	0.0108	VRGNVNTMPKEGAGVDMK
3.6	1931.9337	2.0121	GERAEELENELHHLEK
2.1	1932.9386	1.0072	FYAAEIASALGYLHSIK

Spectrum No: 28; Query: 21293; Rank: 1

Peptide View

MS/MS Fragmentation of **ILTEAEIDAHLVALAERD**
Found in **IPI00029623**, Tax_Id=9606 Gene_Symbol=PSMA6 Proteasome subunit alpha type-6

Match to Query 21293: 1978.042932 from(660.354920,3+)
Title: 090324LimSK_Exosome1_A.14962.14962.3.dta
Data file C:\Temp\Mascoti\LSK_2D\090324LimSK_Exosome1_A_2D.mgf

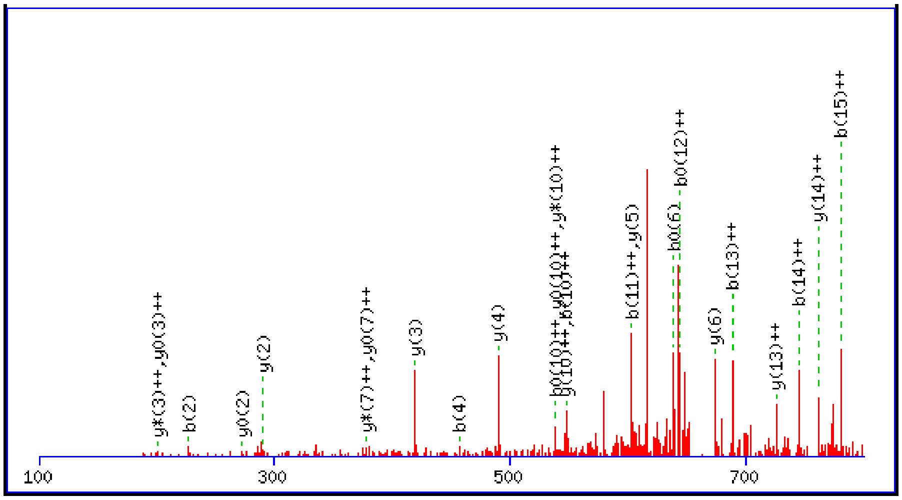

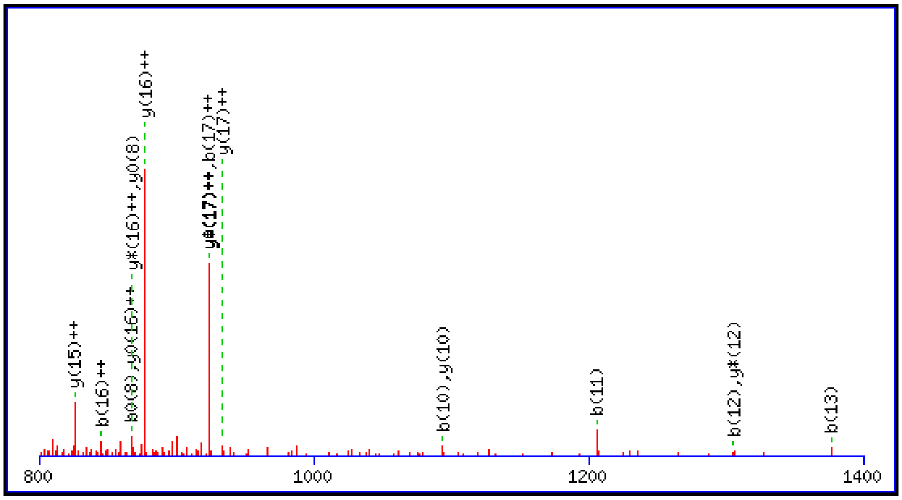

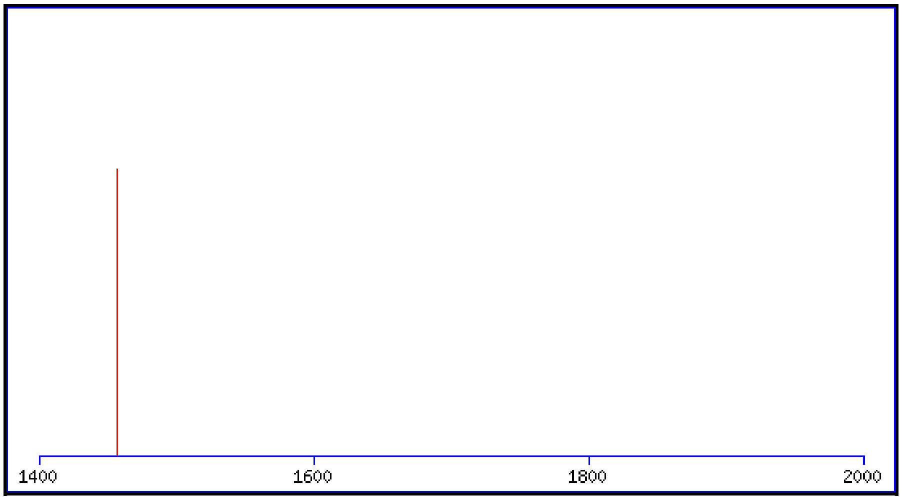

Monoisotopic mass of neutral peptide Mr(calc): 1978.0371
Fixed modifications: Carbamidomethyl (C)
Ions Score: 36 **Expect:** 0.031
Matches (Bold Red): 42/166 fragment ions using 79 most intense peaks

#	b	b^{++}	b*	b^{*++}	b^{0}	b^{0++}	Seq.	y	y^{++}	y*	y^{*++}	y^{0}	y^{0++}	#
1	114.0913	57.5493					I							18
2	**227.1754**	114.0913					L	1865.9603	**933.4838**	1848.9338	**924.9705**	1847.9498	**924.4785**	17
3	328.2231	164.6152			310.2125	155.6099	T	1752.8763	**876.9418**	1735.8497	**868.4285**	1734.8657	**867.9365**	16
4	**457.2657**	229.1365			439.2551	220.1312	E	1651.8286	**826.4179**	1634.8020	817.9047	1633.8180	817.4127	15
5	528.3028	264.6550			510.2922	255.6498	A	1522.7860	**761.8966**	1505.7594	753.3834	1504.7754	752.8914	14
6	657.3454	329.1763			**639.3348**	320.1710	E	1451.7489	**726.3781**	1434.7223	717.8648	1433.7383	717.3728	13

**7**	770.4294	385.7184			752.4189	376.7131	**I**	1322.7063	661.8568	**1305.6797**	653.3435	1304.6957	652.8515	**12**
**8**	885.4564	443.2318			**867.4458**	434.2266	**D**	1209.6222	605.3148	1192.5957	596.8015	1191.6117	596.3095	**11**
**9**	956.4935	478.7504			938.4829	469.7451	**A**	**1094.5953**	**547.8013**	1077.5687	**539.2880**	1076.5847	**538.7960**	**10**
**10**	**1093.5524**	**547.2798**			1075.5419	**538.2746**	**H**	1023.5582	512.2827	1006.5316	503.7694	1005.5476	503.2774	**9**
**11**	**1206.6365**	**603.8219**			1188.6259	594.8166	**L**	886.4993	443.7533	869.4727	435.2400	**868.4887**	434.7480	**8**
**12**	**1305.7049**	653.3561			1287.6943	**644.3508**	**V**	773.4152	387.2112	756.3886	**378.6980**	755.4046	**378.2060**	**7**
**13**	**1376.7420**	**688.8746**			1358.7314	679.8694	**A**	**674.3468**	337.6770	657.3202	329.1638	656.3362	328.6717	**6**
**14**	1489.8261	**745.4167**			1471.8155	736.4114	**L**	**603.3097**	302.1585	586.2831	293.6452	585.2991	293.1532	**5**
**15**	1560.8632	**780.9352**			1542.8526	771.9299	**A**	**490.2256**	245.6164	473.1991	237.1032	472.2150	236.6112	**4**
**16**	1689.9058	**845.4565**			1671.8952	836.4512	**E**	**419.1885**	210.0979	402.1619	**201.5846**	401.1779	**201.0926**	**3**
**17**	1846.0069	**923.5071**	1828.9803	914.9938	1827.9963	914.5018	**R**	**290.1459**	145.5766	273.1193	137.0633	**272.1353**	136.5713	**2**
**18**							**D**	134.0448	67.5260			116.0342	58.5207	**1**

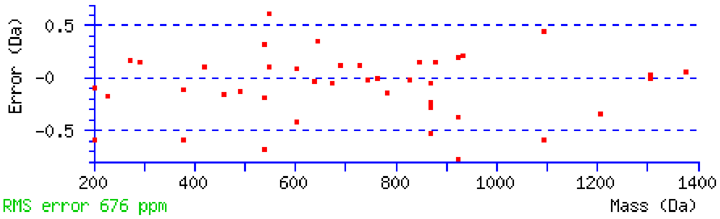

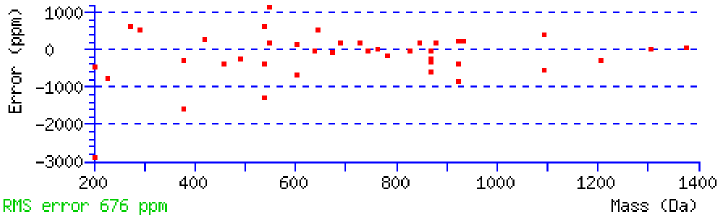

All matches to this query

Score	Mr(calc):	Delta	Sequence
35.8	1978.0371	0.0059	ILTEAEIDAHLVALAERD
9.7	1978.0587	-0.0158	MAPLGGAPRLVLLFSGKR
6.9	1976.0343	2.0086	EVGLTENTGLPRKLLEK
6.9	1976.0343	2.0086	EVGLTENTGLPRKLLEK
5.0	1978.0248	0.0181	SIKENASAGVERLLLGNK
4.8	1978.0305	0.0124	NIYDLMELKNTARELR
3.7	1978.0418	0.0012	MAAEALRNYRNTQAILK
2.4	1978.0401	0.0029	EPELFLSRSRPVAAKAK
1.8	1976.0335	2.0094	MLETKWSLVQQQKMAR
1.7	1976.0190	2.0240	KNHFQVTVYIGMLGEPK

Spectrum No: 29; Query: 20399; Rank: 1

Peptide View

MS/MS Fragmentation of **KVPDKLLDSSTVTHLFK**
Found in **IPI00029623**, Tax_Id=9606 Gene_Symbol=PSMA6 Proteasome subunit alpha type-6

Match to Query 20399: 1927.083736 from(482.778210,4+)
Title: 090324LimSK_Exosome1_B.10752.10752.4.dta
Data file C:\Temp\Mascoti\LSK_2D\090324LimSK_Exosome1_A_2D.mgf

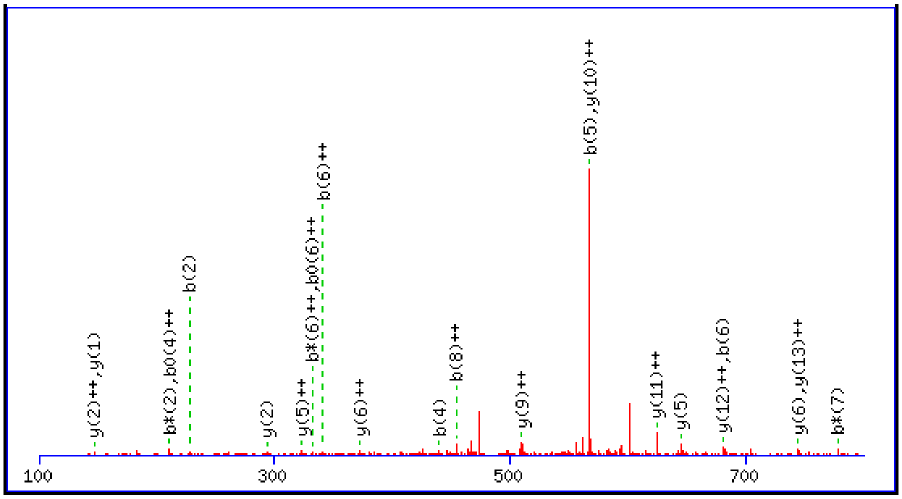

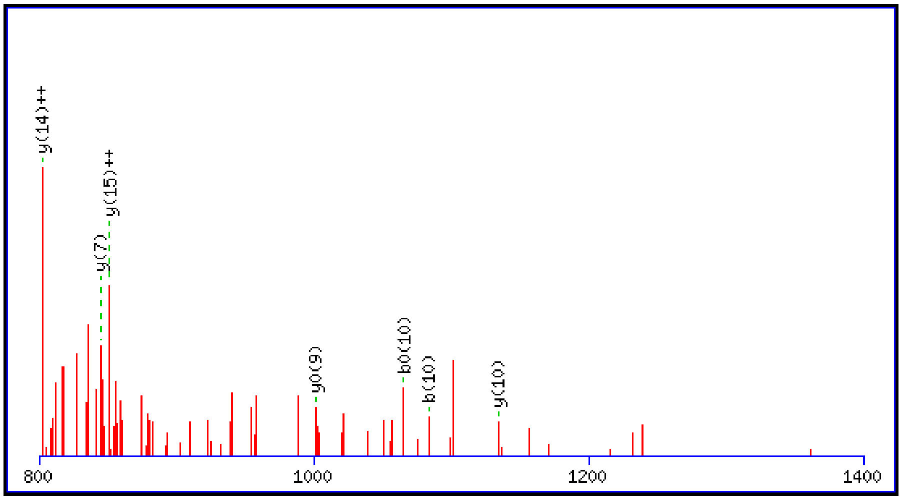

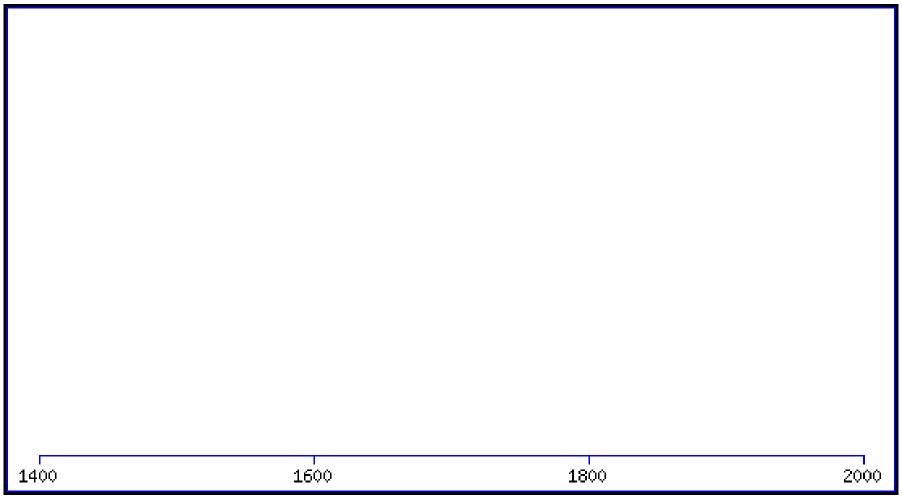

Monoisotopic mass of neutral peptide Mr(calc): 1927.0779
Fixed modifications: Carbamidomethyl (C)
Ions Score: 55 **Expect:** 0.00014
Matches (Bold Red): 30/178 fragment ions using 53 most intense peaks

#	b	b^{++}	b*	b^{*++}	b^0	b^{0++}	Seq.	y	y^{++}	y*	y^{*++}	y^0	y^{0++}	#
1	129.1022	65.0548	112.0757	56.5415			K							17
2	**228.1707**	114.5890	**211.1441**	106.0757			V	1799.9902	900.4987	1782.9636	891.9855	1781.9796	891.4934	16
3	325.2234	163.1153	308.1969	154.6021			P	1700.9218	**850.9645**	1683.8952	842.4512	1682.9112	841.9592	15
4	**440.2504**	220.6288	423.2238	212.1155	422.2398	**211.6235**	D	1603.8690	**802.4381**	1586.8425	793.9249	1585.8584	793.4329	14
5	**568.3453**	284.6763	551.3188	276.1630	550.3348	275.6710	K	1488.8421	**744.9247**	1471.8155	736.4114	1470.8315	735.9194	13
6	**681.4294**	**341.2183**	664.4028	**332.7051**	663.4188	**332.2130**	L	1360.7471	**680.8772**	1343.7205	672.3639	1342.7365	671.8719	12

**7**	794.5135	397.7604	**777.4869**	389.2471	776.5029	388.7551	**L**	1247.6630	**624.3352**	1230.6365	615.8219	1229.6525	615.3299	**11**
**8**	909.5404	**455.2738**	892.5138	446.7606	891.5298	446.2686	**D**	**1134.5790**	**567.7931**	1117.5524	559.2798	1116.5684	558.7878	**10**
**9**	996.5724	498.7898	979.5459	490.2766	978.5619	489.7846	**S**	1019.5520	**510.2796**	1002.5255	501.7664	**1001.5415**	501.2744	**9**
**10**	**1083.6045**	542.3059	1066.5779	533.7926	**1065.5939**	533.3006	**S**	932.5200	466.7636	915.4934	458.2504	914.5094	457.7584	**8**
**11**	1184.6521	592.8297	1167.6256	584.3164	1166.6416	583.8244	**T**	**845.4880**	423.2476	828.4614	414.7343	827.4774	414.2423	**7**
**12**	1283.7205	642.3639	1266.6940	633.8506	1265.7100	633.3586	**V**	**744.4403**	**372.7238**	727.4137	364.2105	726.4297	363.7185	**6**
**13**	1384.7682	692.8877	1367.7417	684.3745	1366.7577	683.8825	**T**	**645.3719**	**323.1896**	628.3453	314.6763	627.3613	314.1843	**5**
**14**	1521.8271	761.4172	1504.8006	752.9039	1503.8166	752.4119	**H**	544.3242	272.6657	527.2976	264.1525			**4**
**15**	1634.9112	817.9592	1617.8846	809.4460	1616.9006	808.9540	**L**	407.2653	204.1363	390.2387	195.6230			**3**
**16**	1781.9796	891.4934	1764.9531	882.9802	1763.9690	882.4882	**F**	**294.1812**	**147.5942**	277.1547	139.0810			**2**
**17**							**K**	**147.1128**	74.0600	130.0863	65.5468			**1**

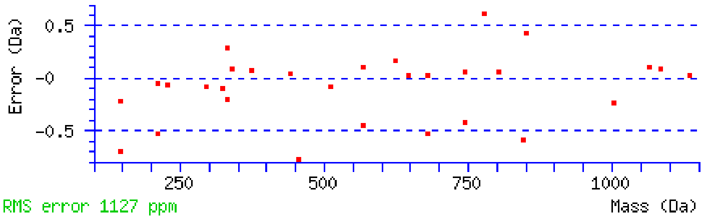

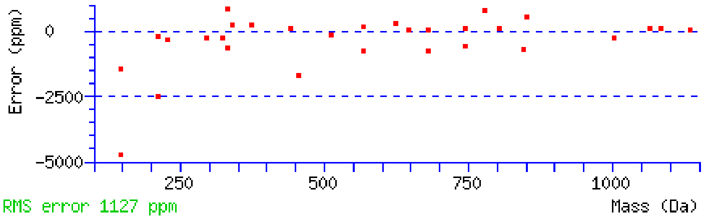

All matches to this query

Score	Mr(calc):	Delta	Sequence
55.1	1927.0779	0.0059	KVPDKLLDSSTVTHLFK
12.9	1927.0666	0.0171	AAPVTLQLLFLDGEEALK
7.9	1926.0703	1.0134	IKSEASPRPLKSVIPPK
5.4	1926.0703	1.0134	IKSEASPRPLKSVIPPK
3.2	1926.0727	1.0110	VKHLIDSLIDFLNFPR
3.1	1925.0638	2.0199	KSISDIPVSKIASFIIK
1.9	1926.0655	1.0182	GAAALLALLCVACALRAGR
1.8	1925.0847	1.9991	QPPQVLKTQTKFQAGVR
1.5	1925.0945	1.9892	GKILVGTRNAEIIEVGEK
1.4	1925.0692	2.0145	VFFRNIKILIPATWK

Spectrum No: 30; Query: 8869; Rank: 1

Peptide View

MS/MS Fragmentation of **LYQVEYAFK**
Found in **IPI00029623**, Tax_Id=9606 Gene_Symbol=PSMA6 Proteasome subunit alpha type-6

Match to Query 8869: 1159.593288 from(580.803920,2+)
Title: 090324LimSK_Exosome1_A.7816.7816.2.dta
Data file C:\Temp\Mascoti\LSK_2D\090324LimSK_Exosome1_A_2D.mgf

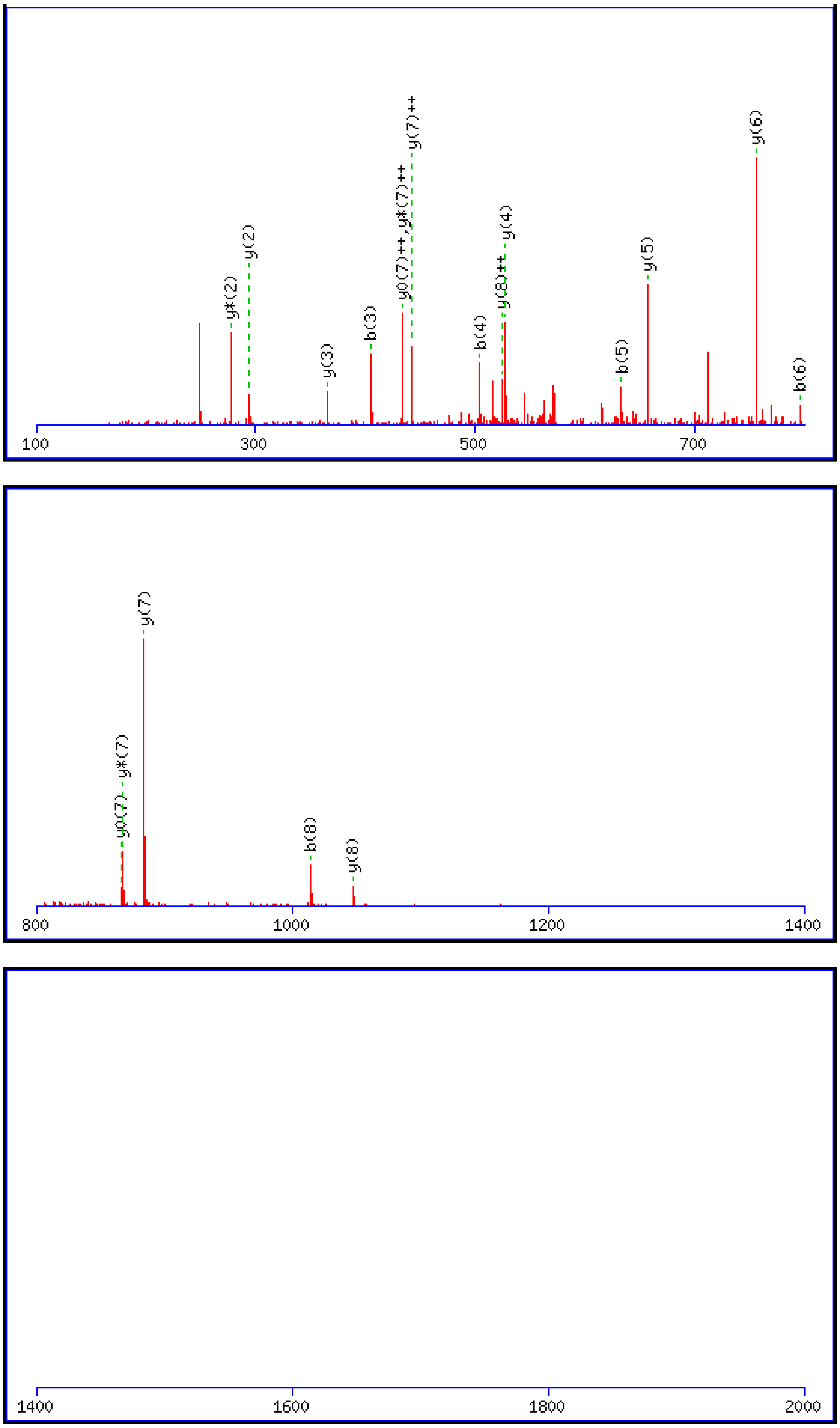

Monoisotopic mass of neutral peptide Mr(calc): 1159.5913
Fixed modifications: Carbamidomethyl (C)
Ions Score: 51 **Expect:** 0.0011
Matches (Bold Red): 22/76 fragment ions using 32 most intense peaks

#	b	b^{++}	b*	b^{*++}	b^{0}	b^{0++}	Seq.	y	y^{++}	y*	y^{*++}	y^{0}	y^{0++}	#
1	114.0913	57.5493					L							9
2	**277.1547**	139.0810					Y	**1047.5146**	**524.2609**	1030.4880	515.7477	1029.5040	515.2556	8
3	**405.2132**	203.1103	388.1867	194.5970			Q	**884.4512**	**442.7293**	**867.4247**	**434.2160**	**866.4407**	**433.7240**	7
4	**504.2817**	252.6445	487.2551	244.1312			V	**756.3927**	378.7000	739.3661	370.1867	738.3821	369.6947	6
5	**633.3243**	317.1658	616.2977	308.6525	615.3137	308.1605	E	**657.3243**	329.1658	640.2977	320.6525	639.3137	320.1605	5
6	**796.3876**	398.6974	779.3610	390.1842	778.3770	389.6921	Y	**528.2817**	264.6445	511.2551	256.1312			4

**7**	**867.4247**	**434.2160**	850.3981	425.7027	849.4141	425.2107	**A**	**365.2183**	183.1128	348.1918	174.5995			**3**
**8**	**1014.4931**	507.7502	997.4666	499.2369	996.4825	498.7449	**F**	**294.1812**	147.5942	**277.1547**	139.0810			**2**
**9**							**K**	147.1128	74.0600	130.0863	65.5468			**1**

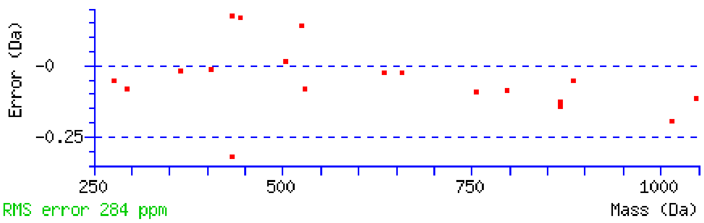

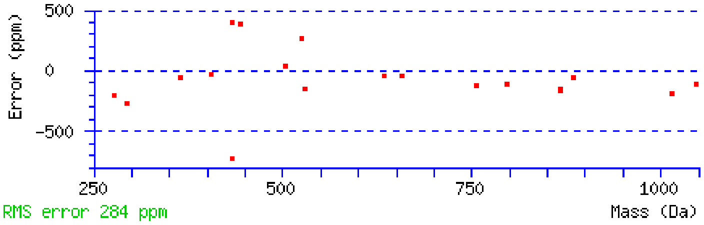

All matches to this query

Score	Mr(calc):	Delta	Sequence
51.1	1159.5913	0.0019	LYQVEYAFK
15.3	1157.5829	2.0104	AENAWKPSQK
14.9	1159.5986	-0.0053	EFKAGGEVPAR
12.2	1159.5985	-0.0053	ETLRAWQEK
11.9	1158.5992	0.9940	NEINREKEK
11.8	1159.5890	0.0043	LYKTLSEVK
11.5	1159.5890	0.0043	LYKTLSEVK
11.5	1158.5993	0.9940	AAGGQDKAGKEK
11.5	1158.5993	0.9940	GREANKLDEK
10.1	1159.5833	0.0100	EKGAGAGGEEKK

Spectrum No: 31; Query: 14916; Rank: 1

Peptide View

MS/MS Fragmentation of **YGYEIPVDMLCKR**
Found in **IPI00029623**, Tax_Id=9606 Gene_Symbol=PSMA6 Proteasome subunit alpha type-6

Match to Query 14916: 1644.796392 from(549.272740,3+)
Title: 090324LimSK_Exosome1_A.11054.11054.3.dta
Data file C:\Temp\Mascoti\LSK_2D\090324LimSK_Exosome1_A_2D.mgf

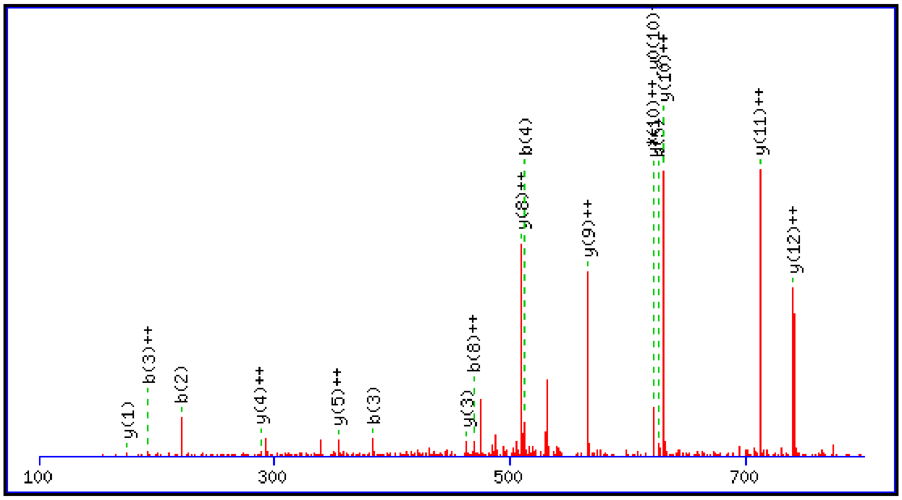

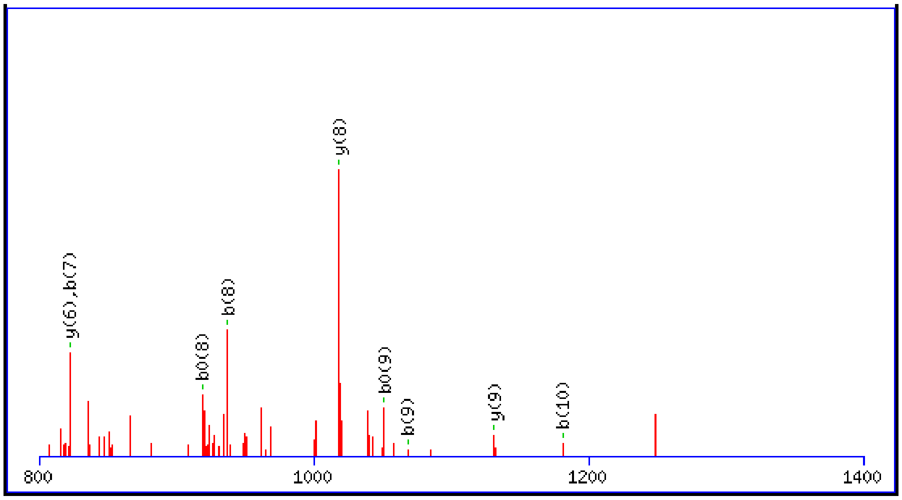

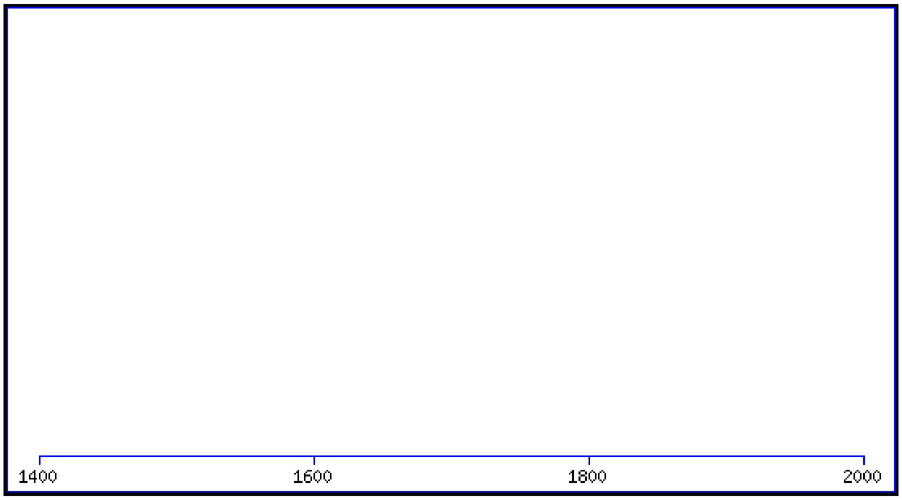

Monoisotopic mass of neutral peptide Mr(calc): 1642.7847
Fixed modifications: Carbamidomethyl (C)
Ions Score: 36 **Expect:** 0.055
Matches (Bold Red): 26/106 fragment ions using 57 most intense peaks

#	b	b^{++}	b*	b^{*++}	b^{0}	b^{0++}	Seq.	y	y^{++}	y*	y^{*++}	y^{0}	y^{0++}	#
1	164.0706	82.5389					Y							13
2	**221.0921**	111.0497					G	1480.7287	**740.8680**	1463.7021	732.3547	1462.7181	731.8627	12
3	**384.1554**	**192.5813**					Y	1423.7072	**712.3572**	1406.6807	703.8440	1405.6967	703.3520	11
4	**513.1980**	257.1026			495.1874	248.0974	E	1260.6439	**630.8256**	1243.6173	**622.3123**	1242.6333	**621.8203**	10
5	**626.2821**	313.6447			608.2715	304.6394	I	**1131.6013**	**566.3043**	1114.5747	557.7910	1113.5907	557.2990	9
6	723.3348	362.1710			705.3243	353.1658	P	**1018.5172**	**509.7623**	1001.4907	501.2490	1000.5067	500.7570	8
7	**822.4032**	411.7053			804.3927	402.7000	V	921.4645	461.2359	904.4379	452.7226	903.4539	452.2306	7
8	**937.4302**	**469.2187**			**919.4196**	460.2134	D	**822.3961**	411.7017	805.3695	403.1884	804.3855	402.6964	6
9	**1068.4707**	534.7390			**1050.4601**	525.7337	M	707.3691	**354.1882**	690.3426	345.6749			5
10	**1181.5547**	591.2810			1163.5442	582.2757	L	576.3286	**288.6680**	559.3021	280.1547			4
11	1341.5854	671.2963			1323.5748	662.2910	C	**463.2446**	232.1259	446.2180	223.6126			3
12	1469.6803	735.3438	1452.6538	726.8305	1451.6698	726.3385	K	303.2139	152.1106	286.1874	143.5973			2
13							R	**175.1190**	88.0631	158.0924	79.5498			1

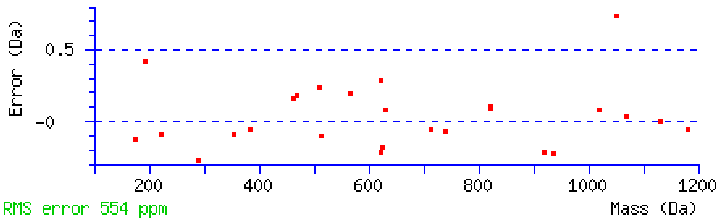

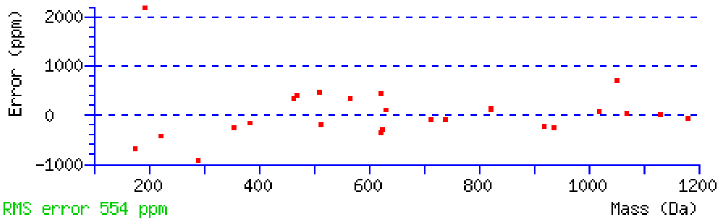

All matches to this query

Score	Mr(calc):	Delta	Sequence
35.7	1642.7847	2.0117	YGYEIPVDMLCKR
13.3	1643.8032	0.9932	NSTLIFTRENTIR
9.2	1643.7975	0.9989	QAGKEARQEAAESNR
8.3	1643.7944	1.0020	VFHGADSDIEWLQK
8.3	1644.7913	0.0051	LRFLSASAVPFTQE
8.1	1644.8001	-0.0037	XALALLNSKQNTGK
6.8	1644.7902	0.0062	VTWLPTRTVXRR
6.4	1644.7913	0.0051	EKLLTQGFTNWTK
5.8	1643.7950	1.0014	KRVFPLSPASNLR
5.2	1643.7936	1.0028	IQSIAPSLQVITSK

Spectrum No: 32; Query: 17258; Rank: 1

Peptide View

MS/MS Fragmentation of **FGPYYTEPVIAGLDPK**
Found in **IPI00028004**, Tax_Id=9606 Gene_Symbol=PSMB3 Proteasome subunit beta type-3

Match to Query 17258: 1765.902428 from(883.958490,2+)
Title: 090324LimSK_Exosome1_A.12970.12970.2.dta
Data file C:\Temp\Mascoti\LSK_2D\090324LimSK_Exosome1_A_2D.mgf

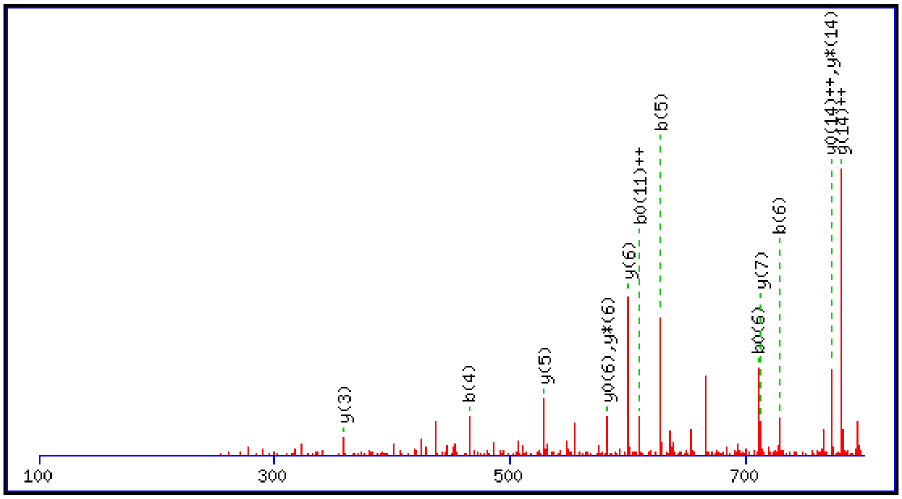

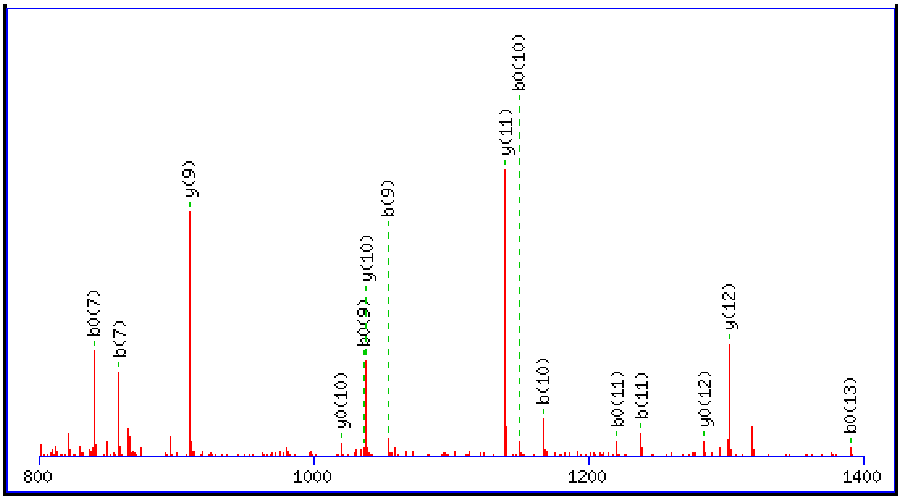

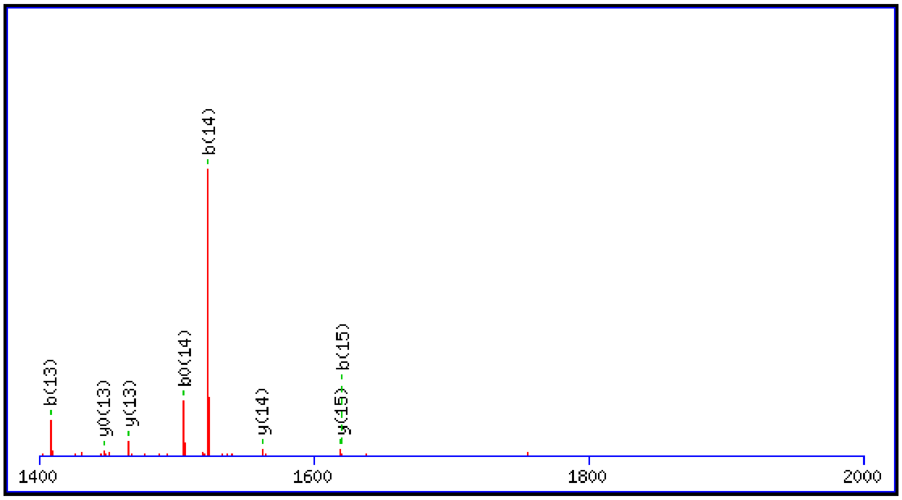

Monoisotopic mass of neutral peptide Mr(calc): 1765.8927
Fixed modifications: Carbamidomethyl (C)
Ions Score: 77 **Expect:** 3.3e-006
Matches (Bold Red): 37/136 fragment ions using 60 most intense peaks

#	b	b^{++}	b^0	b^{0++}	Seq.	y	y^{++}	y*	y^{*++}	y^0	y^{0++}	#
1	148.0757	74.5415			F							16
2	205.0972	103.0522			G	**1619.8316**	810.4194	1602.8050	801.9061	1601.8210	801.4141	15
3	302.1499	151.5786			P	**1562.8101**	**781.9087**	1545.7835	**773.3954**	1544.7995	**772.9034**	14
4	**465.2132**	233.1103			Y	**1465.7573**	733.3823	1448.7308	724.8690	**1447.7468**	724.3770	13
5	**628.2766**	314.6419			Y	**1302.6940**	651.8506	1285.6674	643.3374	**1284.6834**	642.8454	12
6	**729.3243**	365.1658	**711.3137**	356.1605	T	**1139.6307**	570.3190	1122.6041	561.8057	1121.6201	561.3137	11
7	**858.3668**	429.6871	**840.3563**	420.6818	E	**1038.5830**	519.7951	1021.5564	511.2819	**1020.5724**	510.7898	10
8	955.4196	478.2134	937.4090	469.2082	P	**909.5404**	455.2738	892.5138	446.7606	891.5298	446.2686	9
9	**1054.4880**	527.7477	**1036.4775**	518.7424	V	812.4876	406.7475	795.4611	398.2342	794.4771	397.7422	8
10	**1167.5721**	584.2897	**1149.5615**	575.2844	I	**713.4192**	357.2132	696.3927	348.7000	695.4087	348.2080	7
11	**1238.6092**	619.8082	**1220.5986**	**610.8030**	A	**600.3352**	300.6712	**583.3086**	292.1579	**582.3246**	291.6659	6
12	1295.6307	648.3190	1277.6201	639.3137	G	**529.2980**	265.1527	512.2715	256.6394	511.2875	256.1474	5
13	**1408.7147**	704.8610	**1390.7042**	695.8557	L	472.2766	236.6419	455.2500	228.1287	454.2660	227.6366	4
14	**1523.7417**	762.3745	**1505.7311**	753.3692	D	**359.1925**	180.0999	342.1660	171.5866	341.1819	171.0946	3
15	**1620.7944**	810.9009	1602.7839	801.8956	P	244.1656	122.5864	227.1390	114.0731			2
16					K	147.1128	74.0600	130.0863	65.5468			1

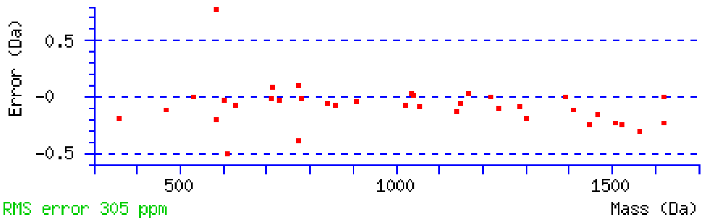

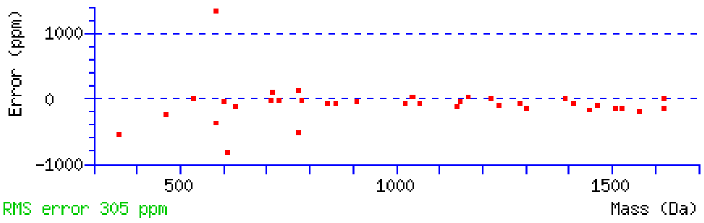

All matches to this query

Score	Mr(calc):	Delta	Sequence
77.5	1765.8927	0.0097	FGPYYTEPVIAGLDPK
16.7	1763.9070	1.9954	SGGGEQLDILSVGILVK
16.7	1764.9135	0.9889	VSNGLKAIRVEAVSDK
6.8	1763.9117	1.9907	MQGLLREGISIAQIR
5.3	1765.8862	0.0162	IIDKTQLDEENLKK
4.1	1764.8981	1.0043	QNTGQPLCHLYPLPK
3.7	1764.9036	0.9988	EPLPSRPLNARPGPGK
3.3	1765.8975	0.0050	IEATKKAESEIQAIR
1.2	1765.8975	0.0050	IEATKKAESEIQAIR
0.8	1765.9161	-0.0137	EILQIMNKTISKPR

Spectrum No: 33; Query: 9564; Rank: 1

Peptide View

MS/MS Fragmentation of **LNLYELKEGR**
Found in **IPI00028004**, Tax_Id=9606 Gene_Symbol=PSMB3 Proteasome subunit beta type-3

Match to Query 9564: 1233.673962 from(412.231930,3+)
Title: 090324LimSK_Exosome1_A.5688.5688.3.dta
Data file C:\Temp\Mascoti\LSK_2D\090324LimSK_Exosome1_A_2D.mgf

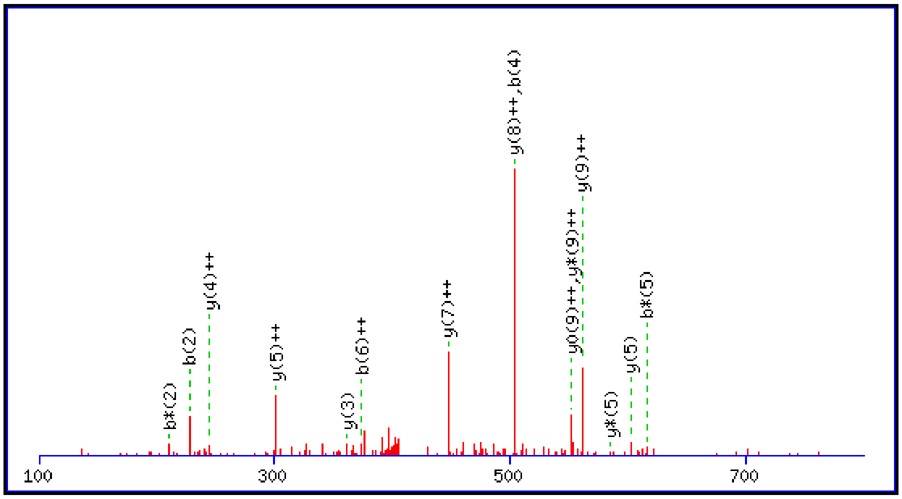

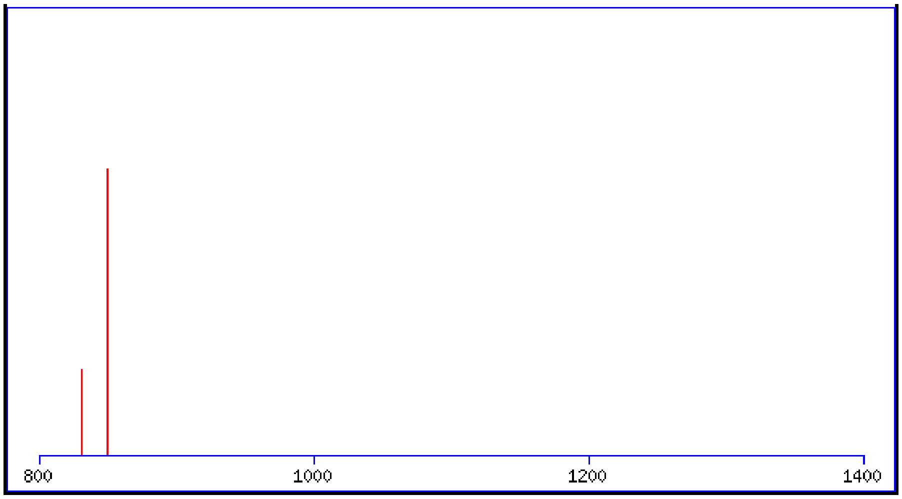

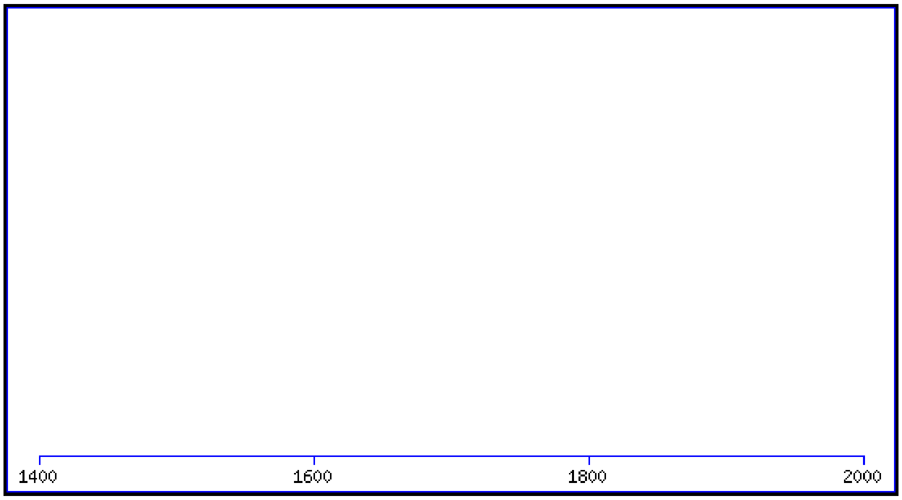

Monoisotopic mass of neutral peptide Mr(calc): 1233.6717
Fixed modifications: Carbamidomethyl (C)
Ions Score: 20 **Expect:** 0.93
Matches (Bold Red): 15/94 fragment ions using 33 most intense peaks

#	b	b^{++}	b*	b^{*++}	b^0	b^{0++}	Seq.	y	y^{++}	y*	y^{*++}	y^0	y^{0++}	#
1	114.0913	57.5493					L							10
2	**228.1343**	114.5708	**211.1077**	106.0575			N	1121.5949	**561.3011**	1104.5684	**552.7878**	1103.5844	**552.2958**	9
3	341.2183	171.1128	324.1918	162.5995			L	1007.5520	**504.2796**	990.5255	495.7664	989.5415	495.2744	8
4	**504.2817**	252.6445	487.2551	244.1312			Y	894.4680	**447.7376**	877.4414	439.2243	876.4574	438.7323	7
5	633.3243	317.1658	**616.2977**	308.6525	615.3137	308.1605	E	731.4046	366.2060	714.3781	357.6927	713.3941	357.2007	6
6	746.4083	**373.7078**	729.3818	365.1945	728.3978	364.7025	L	**602.3620**	**301.6847**	**585.3355**	293.1714	584.3515	292.6794	5
7	874.5033	437.7553	857.4767	429.2420	856.4927	428.7500	K	489.2780	**245.1426**	472.2514	236.6293	471.2674	236.1373	4
8	1003.5459	502.2766	986.5193	493.7633	985.5353	493.2713	E	**361.1830**	181.0951	344.1565	172.5819	343.1724	172.0899	3
9	1060.5673	530.7873	1043.5408	522.2740	1042.5568	521.7820	G	232.1404	116.5738	215.1139	108.0606			2
10							R	175.1190	88.0631	158.0924	79.5498			1

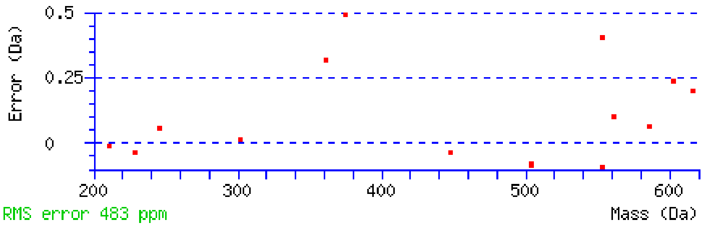

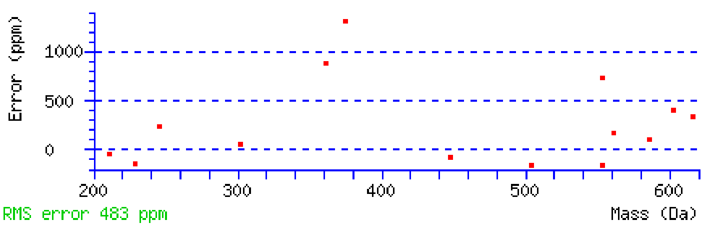

All matches to this query

Score	Mr(calc):	Delta	Sequence
20.5	1233.6717	0.0023	LNLYELKEGR
12.8	1233.6734	0.0006	LIDAKTGLPVK
12.4	1232.6765	0.9975	DFLAGGIAAAISK
11.9	1232.6738	1.0002	EPGPTPRARPR
10.8	1233.6734	0.0006	KVISALLEGPK
10.6	1232.6812	0.9928	QPLRCPPIPR
9.3	1232.6642	1.0098	LNLKPRSTPK
9.2	1231.6561	2.0179	EIPPPPPTAASR
9.2	1232.6642	1.0098	KPKKAAGGATPK
7.6	1233.6846	-0.0107	VKVVTAPATIR

Spectrum No: 34; Query: 20295; Rank: 1

Peptide View

MS/MS Fragmentation of **RFGPYYTEPVIAGLDPK**
Found in **IPI00028004**, Tax_Id=9606 Gene_Symbol=PSMB3 Proteasome subunit beta type-3

Match to Query 20295: 1921.998672 from(641.673500,3+)
Title: 090324LimSK_Exosome1_B.11804.11804.3.dta
Data file C:\Temp\Mascoti\LSK_2D\090324LimSK_Exosome1_A_2D.mgf

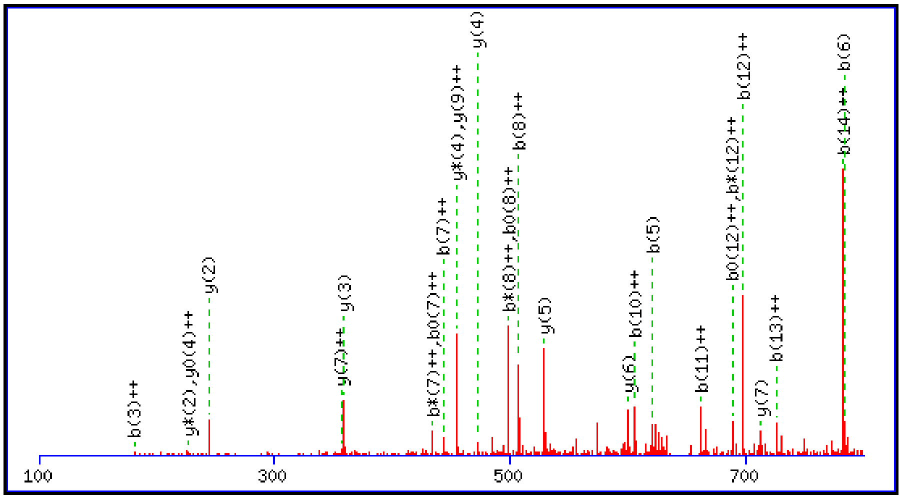

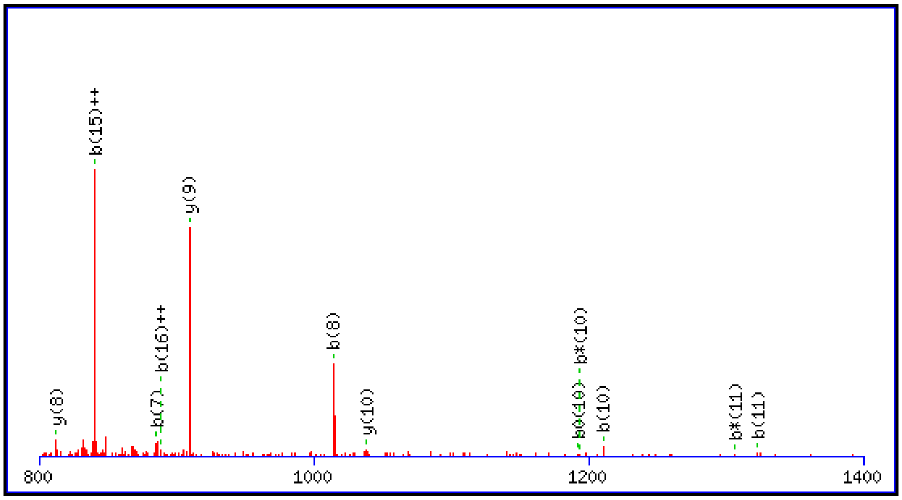

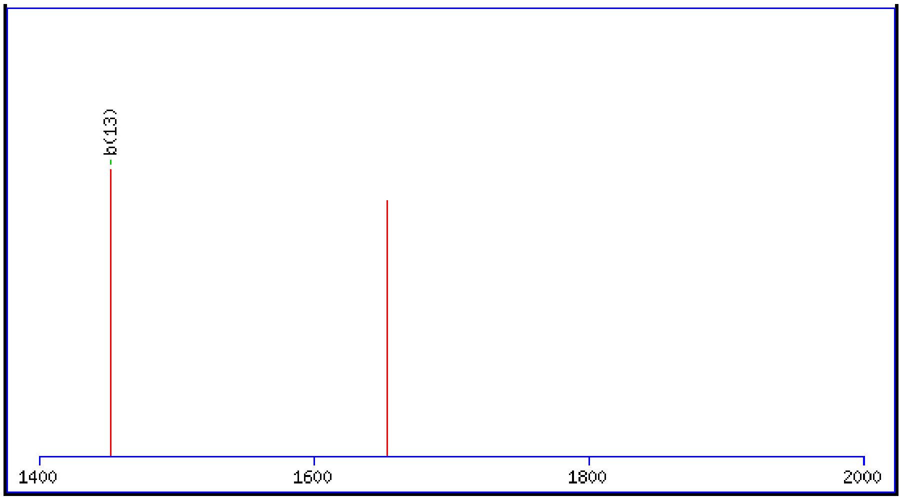

Monoisotopic mass of neutral peptide Mr(calc): 1921.9938
Fixed modifications: Carbamidomethyl (C)
Ions Score: 46 **Expect:** 0.0039
Matches (Bold Red): 40/176 fragment ions using 79 most intense peaks

#	b	b^{++}	b*	b^{*++}	b^{0}	b^{0++}	Seq.	y	y^{++}	y*	y^{*++}	y^{0}	y^{0++}	#
1	157.1084	79.0578	140.0818	70.5446			R							17
2	304.1768	152.5920	287.1503	144.0788			F	1766.9000	883.9536	1749.8734	875.4403	1748.8894	874.9483	16
3	361.1983	**181.1028**	344.1717	172.5895			G	1619.8316	810.4194	1602.8050	801.9061	1601.8210	801.4141	15
4	458.2510	229.6292	441.2245	221.1159			P	1562.8101	781.9087	1545.7835	773.3954	1544.7995	772.9034	14
5	**621.3144**	311.1608	604.2878	302.6475			Y	1465.7573	733.3823	1448.7308	724.8690	1447.7468	724.3770	13
6	**784.3777**	392.6925	767.3511	384.1792			Y	1302.6940	651.8506	1285.6674	643.3374	1284.6834	642.8454	12
7	**885.4254**	**443.2163**	868.3988	**434.7030**	867.4148	**434.2110**	T	1139.6307	570.3190	1122.6041	561.8057	1121.6201	561.3137	11
8	**1014.4680**	**507.7376**	997.4414	**499.2243**	996.4574	**498.7323**	E	**1038.5830**	519.7951	1021.5564	511.2819	1020.5724	510.7898	10
9	1111.5207	556.2640	1094.4942	547.7507	1093.5102	547.2587	P	**909.5404**	**455.2738**	892.5138	446.7606	891.5298	446.2686	9
10	**1210.5891**	**605.7982**	**1193.5626**	597.2849	**1192.5786**	596.7929	V	**812.4876**	406.7475	795.4611	398.2342	794.4771	397.7422	8
11	**1323.6732**	**662.3402**	**1306.6467**	653.8270	1305.6626	653.3350	I	**713.4192**	**357.2132**	696.3927	348.7000	695.4087	348.2080	7
12	1394.7103	**697.8588**	1377.6838	**689.3455**	1376.6998	**688.8535**	A	**600.3352**	300.6712	583.3086	292.1579	582.3246	291.6659	6
13	**1451.7318**	**726.3695**	1434.7052	717.8563	1433.7212	717.3642	G	**529.2980**	265.1527	512.2715	256.6394	511.2875	256.1474	5
14	1564.8158	**782.9116**	1547.7893	774.3983	1546.8053	773.9063	L	**472.2766**	236.6419	**455.2500**	228.1287	454.2660	**227.6366**	4
15	1679.8428	**840.4250**	1662.8162	831.9118	1661.8322	831.4197	D	**359.1925**	180.0999	342.1660	171.5866	341.1819	171.0946	3
16	1776.8956	**888.9514**	1759.8690	880.4381	1758.8850	879.9461	P	**244.1656**	122.5864	**227.1390**	114.0731			2
17							K	147.1128	74.0600	130.0863	65.5468			1

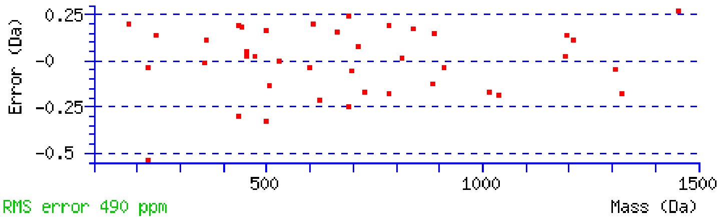

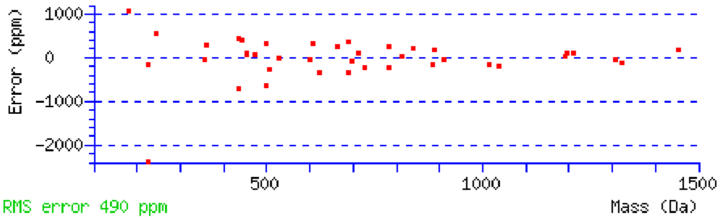

All matches to this query

Score	Mr(calc):	Delta	Sequence
46.0	1921.9938	0.0049	RFGPYYTEPVIAGLDPK
9.4	1921.9874	0.0113	TQGSEKAALQLQLNTIK
7.0	1921.9874	0.0113	KSEVENGPSSVVTRILK
6.6	1921.9874	0.0113	KSEVENGPSSVVTRILK
5.3	1919.9731	2.0256	HHPSSPLPGSKTSKRPK

5.3	1919.9731	2.0256	HHPSSPLPGSKTSKRPK
5.2	1919.9903	2.0083	ELETEIIKIGRVMGPR
4.7	1922.0108	-0.0122	LNELQEGAAPLEQEIIR
4.5	1921.9874	0.0113	QLEEKEALISQLTRGK
3.8	1920.9904	1.0082	LDELEGALHQAKEELAR

Spectrum No: 35; Query: 21792; Rank: 1

Peptide View

MS/MS Fragmentation of **AGGSASAMLQPLLDNQVGFK**
Found in **IPI00025019**, Tax_Id=9606 Gene_Symbol=PSMB1 Proteasome subunit beta type-1

Match to Query 21792: 2003.021708 from(1002.518130,2+)
Title: 090324LimSK_Exosome1_A.14810.14810.2.dta
Data file C:\Temp\Mascoti\LSK_2D\090324LimSK_Exosome1_A_2D.mgf

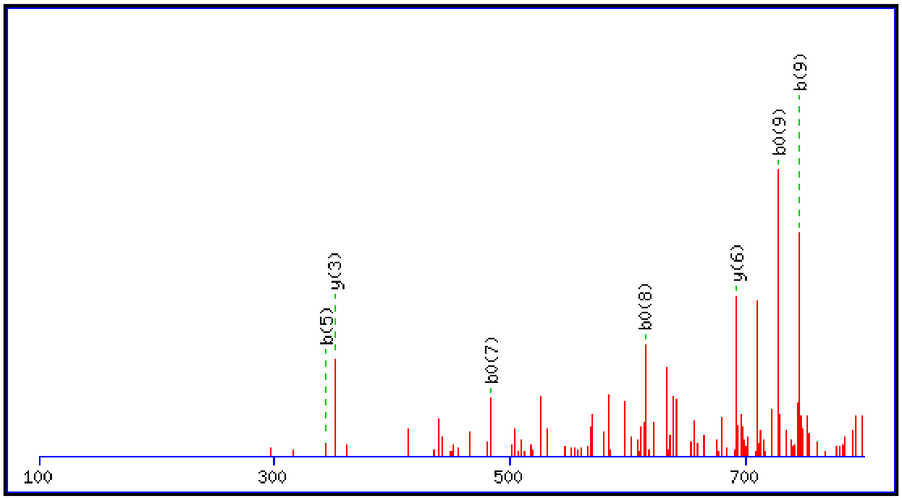

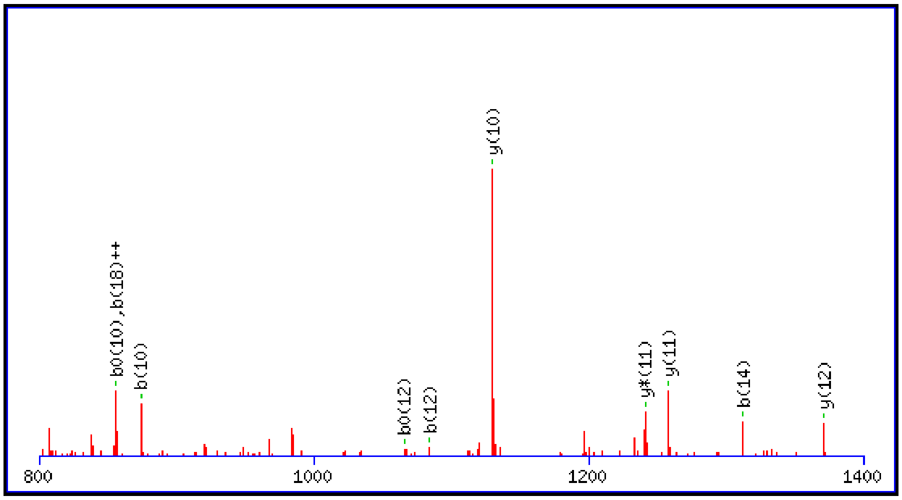

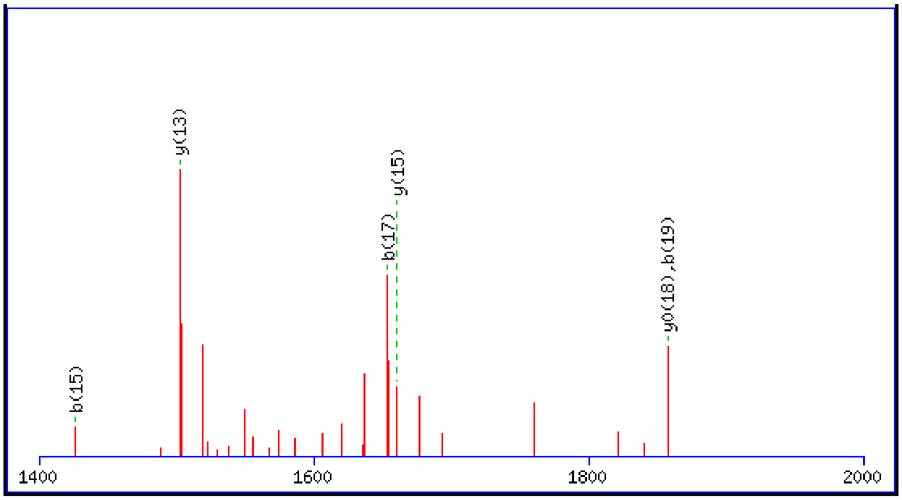

Monoisotopic mass of neutral peptide Mr(calc): 2003.0146
Fixed modifications: Carbamidomethyl (C)
Ions Score: 55 **Expect:** 0.00051
Matches (Bold Red): 23/192 fragment ions using 34 most intense peaks

#	b	b++	b*	b*++	b0	b0++	Seq.	y	y++	y*	y*++	y0	y0++	#
1	72.0444	36.5258					A							20
2	129.0659	65.0366					G	1932.9848	966.9960	1915.9582	958.4828	1914.9742	957.9907	19
3	186.0873	93.5473					G	1875.9633	938.4853	1858.9368	929.9720	**1857.9527**	929.4800	18
4	273.1193	137.0633			255.1088	128.0580	S	1818.9418	909.9746	1801.9153	901.4613	1800.9313	900.9693	17
5	**344.1565**	172.5819			326.1459	163.5766	A	1731.9098	866.4585	1714.8833	857.9453	1713.8993	857.4533	16
6	431.1885	216.0979			413.1779	207.0926	S	**1660.8727**	830.9400	1643.8462	822.4267	1642.8621	821.9347	15
7	502.2256	251.6164			**484.2150**	242.6112	A	1573.8407	787.4240	1556.8141	778.9107	1555.8301	778.4187	14
8	633.2661	317.1367			**615.2555**	308.1314	M	**1502.8036**	751.9054	1485.7770	743.3921	1484.7930	742.9001	13
9	**746.3502**	373.6787			**728.3396**	364.6734	L	**1371.7631**	686.3852	1354.7365	677.8719	1353.7525	677.3799	12
10	**874.4087**	437.7080	857.3822	429.1947	**856.3982**	428.7027	Q	**1258.6790**	629.8431	**1241.6525**	621.3299	1240.6684	620.8379	11
11	971.4615	486.2344	954.4349	477.7211	953.4509	477.2291	P	**1130.6204**	565.8139	1113.5939	557.3006	1112.6099	556.8086	10
12	**1084.5456**	542.7764	1067.5190	534.2631	**1066.5350**	533.7711	L	1033.5677	517.2875	1016.5411	508.7742	1015.5571	508.2822	9
13	1197.6296	599.3184	1180.6031	590.8052	1179.6191	590.3132	L	920.4836	460.7454	903.4571	452.2322	902.4730	451.7402	8
14	**1312.6566**	656.8319	1295.6300	648.3186	1294.6460	647.8266	D	807.3995	404.2034	790.3730	395.6901	789.3890	395.1981	7
15	**1426.6995**	713.8534	1409.6729	705.3401	1408.6889	704.8481	N	**692.3726**	346.6899	675.3461	338.1767			6
16	1554.7581	777.8827	1537.7315	769.3694	1536.7475	768.8774	Q	578.3297	289.6685	561.3031	281.1552			5
17	**1653.8265**	827.4169	1636.7999	818.9036	1635.8159	818.4116	V	450.2711	225.6392	433.2445	217.1259			4
18	1710.8479	**855.9276**	1693.8214	847.4143	1692.8374	846.9223	G	**351.2027**	176.1050	334.1761	167.5917			3
19	**1857.9164**	929.4618	1840.8898	920.9485	1839.9058	920.4565	F	294.1812	147.5942	277.1547	139.0810			2
20							K	147.1128	74.0600	130.0863	65.5468			1

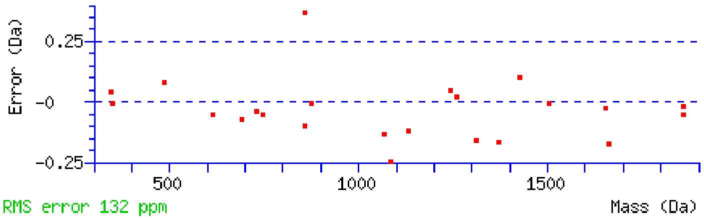

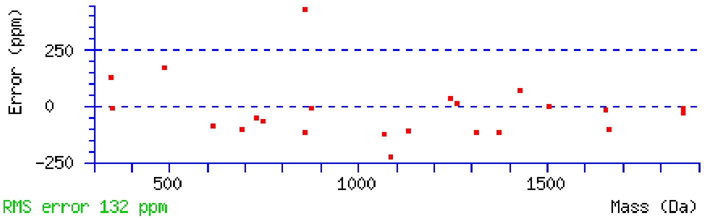

All matches to this query

Score	Mr(calc):	Delta	Sequence
55.2	2003.0146	0.0071	AGGSASAMLQPLLDNQVGFK
4.1	2003.0281	-0.0064	HSFLSNAFFLKVTALTK

3.1	2003.0288	-0.0071	MPVLSRPRPWRGNTLK
2.5	2003.0292	-0.0075	DKEAMALANSVQGCLIRK
1.1	2002.0119	1.0098	EVKYGAPQATDGLAGALDAR
0.6	2002.0210	1.0007	LEALHQILVLLSGMEEK
0.5	2003.0364	-0.0147	FNAHEFATLVIDILSDAK
0.2	2002.0306	0.9911	SVLPSSRQMPASQSWLTK
0.1	2003.0315	-0.0098	VGIIPPKGCLLYGPPGTGK

Spectrum No: 36; Query: 13148; Rank: 1

Peptide View

MS/MS Fragmentation of **GAVYSFDPVGSYQR**
Found in **IPI00025019**, Tax_Id=9606 Gene_Symbol=PSMB1 Proteasome subunit beta type-1

Match to Query 13148: 1544.729408 from(773.371980,2+)
Title: 090324LimSK_Exosome1_B.8125.8125.2.dta
Data file C:\Temp\Mascoti\LSK_2D\090324LimSK_Exosome1_A_2D.mgf

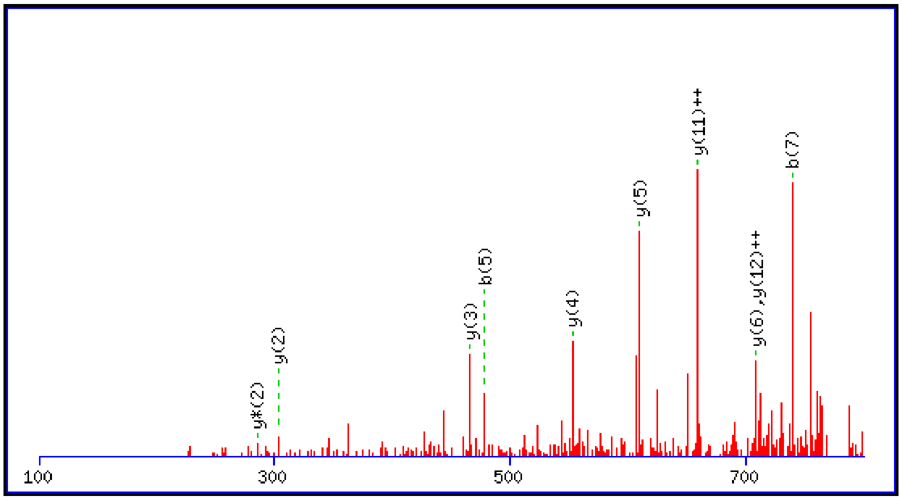

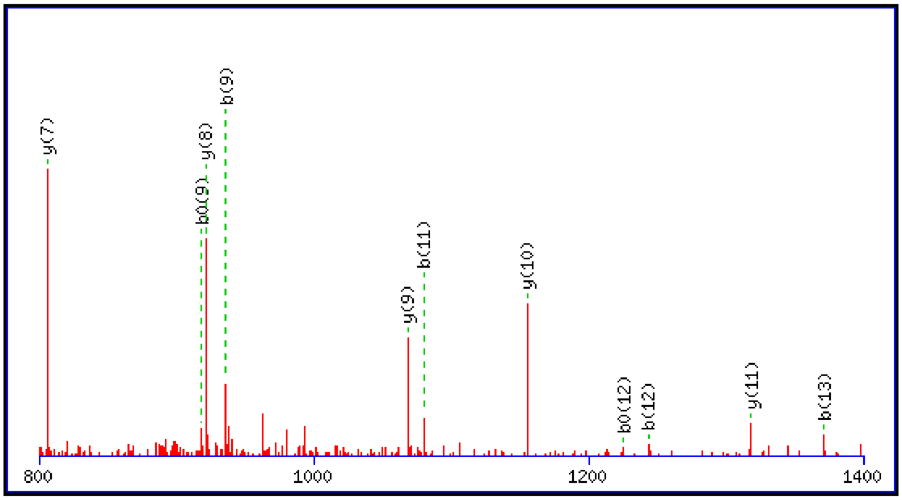

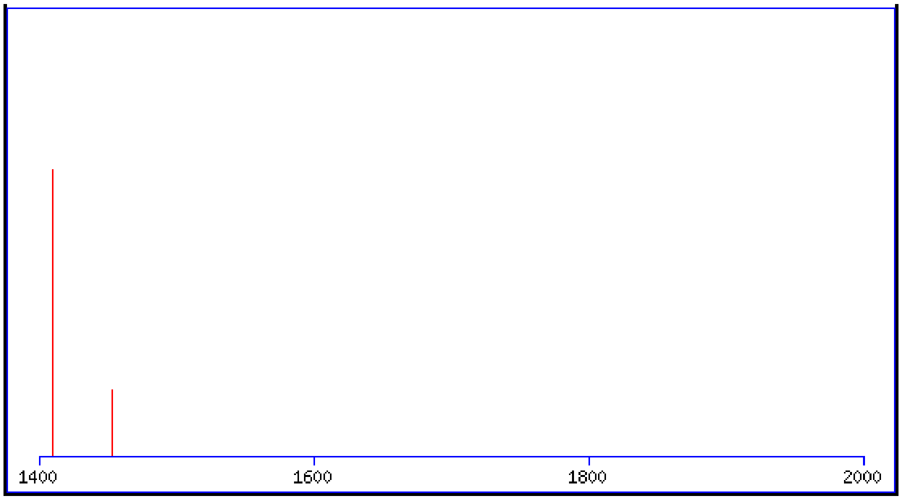

Monoisotopic mass of neutral peptide Mr(calc): 1544.7260
Fixed modifications: Carbamidomethyl (C)
Ions Score: 90 **Expect:** 2.2e-007
Matches (Bold Red): 21/118 fragment ions using 27 most intense peaks

#	b	b^{++}	b*	b^{*++}	b^{0}	b^{0++}	Seq.	y	y^{++}	y*	y^{*++}	y^{0}	y^{0++}	#
1	58.0287	29.5180					G							14
2	129.0659	65.0366					A	1488.7118	744.8595	1471.6852	736.3462	1470.7012	735.8542	13
3	228.1343	114.5708					V	1417.6747	**709.3410**	1400.6481	700.8277	1399.6641	700.3357	12
4	391.1976	196.1024					Y	**1318.6062**	**659.8068**	1301.5797	651.2935	1300.5957	650.8015	11
5	**478.2296**	239.6185			460.2191	230.6132	S	**1155.5429**	578.2751	1138.5164	569.7618	1137.5323	569.2698	10
6	625.2980	313.1527			607.2875	304.1474	F	**1068.5109**	534.7591	1051.4843	526.2458	1050.5003	525.7538	9
7	**740.3250**	370.6661			722.3144	361.6608	D	**921.4425**	461.2249	904.4159	452.7116	903.4319	452.2196	8
8	837.3777	419.1925			819.3672	410.1872	P	**806.4155**	403.7114	789.3890	395.1981	788.4050	394.7061	7
9	**936.4462**	468.7267			**918.4356**	459.7214	V	**709.3628**	355.1850	692.3362	346.6717	691.3522	346.1797	6
10	993.4676	497.2375			975.4571	488.2322	G	**610.2944**	305.6508	593.2678	297.1375	592.2838	296.6455	5
11	**1080.4997**	540.7535			1062.4891	531.7482	S	**553.2729**	277.1401	536.2463	268.6268	535.2623	268.1348	4
12	**1243.5630**	622.2851			**1225.5524**	613.2798	Y	**466.2409**	233.6241	449.2143	225.1108			3
13	**1371.6216**	686.3144	1354.5950	677.8011	1353.6110	677.3091	Q	**303.1775**	152.0924	**286.1510**	143.5791			2
14							R	175.1190	88.0631	158.0924	79.5498			1

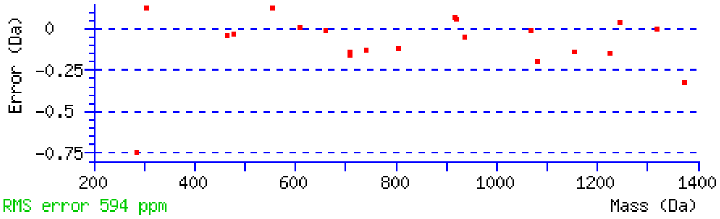

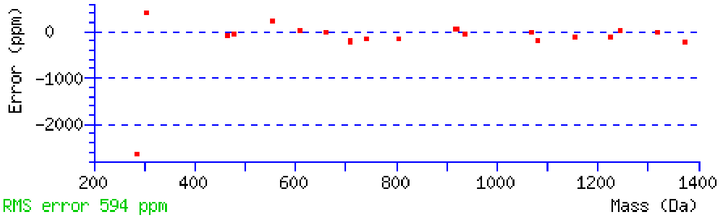

All matches to this query

Score	Mr(calc):	Delta	Sequence
89.8	1544.7260	0.0034	GAVYSFDPVGSYQR
9.4	1544.7348	-0.0054	ITATSAENRVTFR
8.7	1544.7236	0.0059	SFQDSLEDIKKR
5.5	1542.7079	2.0215	DPLSALAREYGGSK
5.4	1544.7364	-0.0070	KPATLSQIGTNKK
5.3	1544.7348	-0.0054	ITATSAENRVTFR
4.0	1543.7283	1.0011	TLRYVPEESKDK
4.0	1543.7283	1.0011	TLRYVPEESKDK

3.9	1542.7320	1.9974	AKVRLLGTADSPR
3.8	1544.7388	-0.0094	YKTHVDTVLAYR

Spectrum No: 37; Query: 26844; Rank: 1

Peptide View

MS/MS Fragmentation of **LTDKTVIGCSGFHGDCLTLTK**
Found in **IPI00025019**, Tax_Id=9606 Gene_Symbol=PSMB1 Proteasome subunit beta type-1

Match to Query 26844: 2322.137532 from(775.053120,3+)
Title: 090324LimSK_Exosome1_B.8078.8078.3.dta
Data file C:\Temp\Mascoti\LSK_2D\090324LimSK_Exosome1_A_2D.mgf

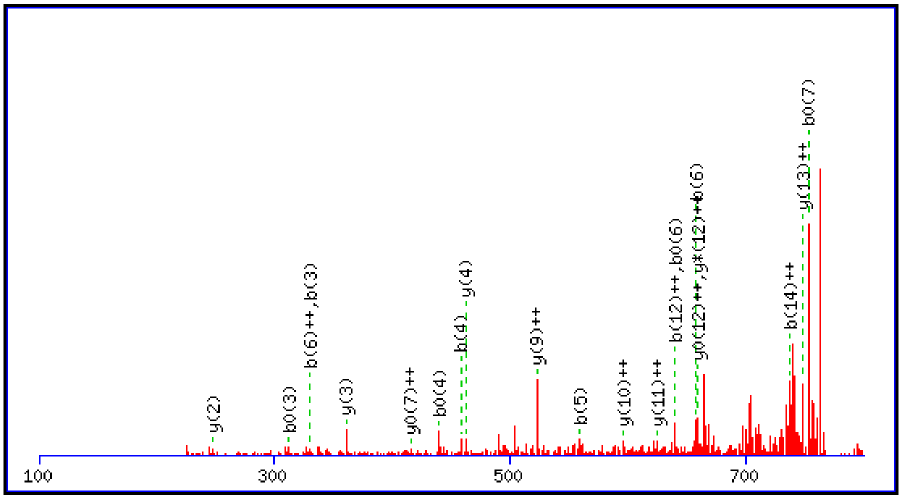

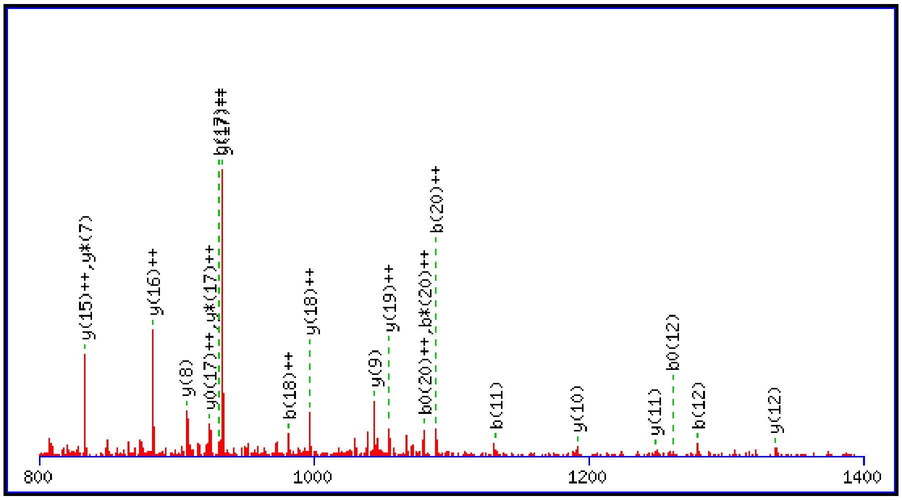

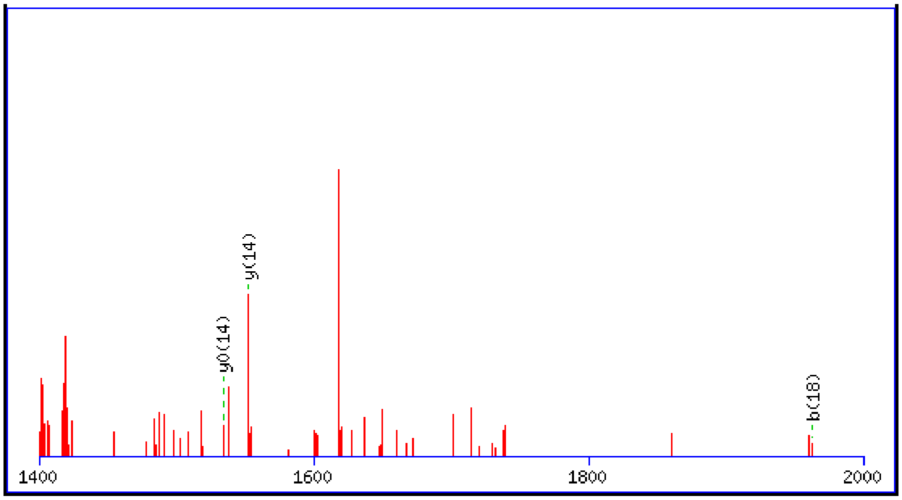

Monoisotopic mass of neutral peptide Mr(calc): 2322.1348
Fixed modifications: Carbamidomethyl (C)
Ions Score: 44 **Expect:** 0.011
Matches (Bold Red): 45/230 fragment ions using 100 most intense peaks

#	b	b^{++}	b*	b^{*++}	b^{0}	b^{0++}	Seq.	y	y^{++}	y*	y^{*++}	y^{0}	y^{0++}	#
1	114.0913	57.5493					L							21
2	215.1390	108.0731			197.1285	99.0679	T	2210.0580	1105.5326	2193.0315	1097.0194	2192.0475	1096.5274	20
3	**330.1660**	165.5866			**312.1554**	156.5813	D	2109.0103	**1055.0088**	2091.9838	1046.4955	2090.9998	1046.0035	19
4	**458.2609**	229.6341	441.2344	221.1208	**440.2504**	220.6288	K	1993.9834	**997.4953**	1976.9568	988.9821	1975.9728	988.4901	18
5	**559.3086**	280.1579	542.2821	271.6447	541.2980	271.1527	T	1865.8884	**933.4479**	1848.8619	**924.9346**	1847.8779	**924.4426**	17
6	**658.3770**	**329.6921**	641.3505	321.1789	**640.3665**	320.6869	V	1764.8408	**882.9240**	1747.8142	874.4107	1746.8302	873.9187	16
7	771.4611	386.2342	754.4345	377.7209	**753.4505**	377.2289	I	1665.7723	**833.3898**	1648.7458	824.8765	1647.7618	824.3845	15
8	828.4825	414.7449	811.4560	406.2316	810.4720	405.7396	G	**1552.6883**	776.8478	1535.6617	768.3345	**1534.6777**	767.8425	14
9	988.5132	494.7602	971.4866	486.2470	970.5026	485.7550	C	1495.6668	**748.3370**	1478.6403	739.8238	1477.6562	739.3318	13
10	1075.5452	538.2762	1058.5187	529.7630	1057.5347	529.2710	S	**1335.6362**	668.3217	1318.6096	**659.8084**	1317.6256	**659.3164**	12
11	**1132.5667**	566.7870	1115.5401	558.2737	1114.5561	557.7817	G	**1248.6041**	**624.8057**	1231.5776	616.2924	1230.5936	615.8004	11
12	**1279.6351**	**640.3212**	1262.6086	631.8079	**1261.6245**	631.3159	F	**1191.5827**	**596.2950**	1174.5561	587.7817	1173.5721	587.2897	10
13	1416.6940	708.8506	1399.6675	700.3374	1398.6834	699.8454	H	**1044.5143**	**522.7608**	1027.4877	514.2475	1026.5037	513.7555	9
14	1473.7155	**737.3614**	1456.6889	728.8481	1455.7049	728.3561	G	**907.4553**	454.2313	890.4288	445.7180	889.4448	445.2260	8
15	1588.7424	794.8748	1571.7159	786.3616	1570.7319	785.8696	D	850.4339	425.7206	**833.4073**	417.2073	832.4233	**416.7153**	7
16	1748.7731	874.8902	1731.7465	866.3769	1730.7625	865.8849	C	735.4069	368.2071	718.3804	359.6938	717.3964	359.2018	6
17	1861.8571	**931.4322**	1844.8306	922.9189	1843.8466	922.4269	L	575.3763	288.1918	558.3497	279.6785	557.3657	279.1865	5
18	**1962.9048**	**981.9560**	1945.8783	973.4428	1944.8942	972.9508	T	**462.2922**	231.6498	445.2657	223.1365	444.2817	222.6445	4
19	2075.9889	1038.4981	2058.9623	1029.9848	2057.9783	1029.4928	L	**361.2445**	181.1259	344.2180	172.6126	343.2340	172.1206	3
20	2177.0366	**1089.0219**	2160.0100	**1080.5086**	2159.0260	**1080.0166**	T	**248.1605**	124.5839	231.1339	116.0706	230.1499	115.5786	2
21							K	147.1128	74.0600	130.0863	65.5468			1

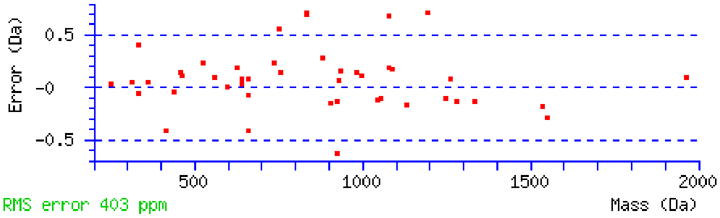

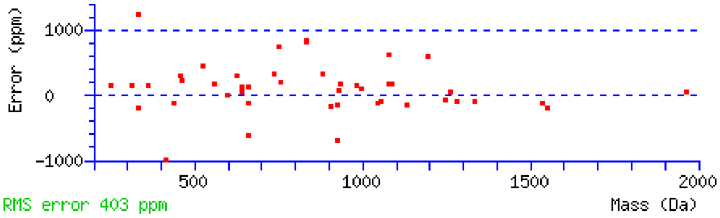

All matches to this query

Score	Mr(calc):	Delta	Sequence
43.6	2322.1348	0.0027	LTDKTVIGCSGFHGDCLTLTK

6.0	2321.1143	1.0232	TSLLENEALFPIGGKKAVMK
2.3	2320.1360	2.0015	KDTAQIMFMAGLPIGSLIFR
1.8	2321.1433	0.9942	GIGTPPNTTPIKNGSPEIKLK
1.7	2320.1382	1.9993	SRNVTVDSSAWKELCHLYR
0.6	2321.1473	0.9902	LTKDTTLPVTLTEWKLFR
0.4	2321.1545	0.9830	ELLHELALSVPGARSKVTNK
0.1	2321.1264	1.0111	LGQIRSEIDLTEAQETVPSR
0.0	2322.1151	0.0224	DSRGGRELTGPAVLAVELCSR

Spectrum No: 38; Query: 21039; Rank: 1

Peptide View

MS/MS Fragmentation of **AIGSASEGAQSSLQEVYHK**
Found in **IPI00291922**, Tax_Id=9606 Gene_Symbol=PSMA5 Proteasome subunit alpha type-5

Match to Query 21039: 1960.947828 from(981.481190,2+)
Title: 090324LimSK_Exosome1_B.4113.4113.2.dta
Data file C:\Temp\Mascoti\LSK_2D\090324LimSK_Exosome1_A_2D.mgf

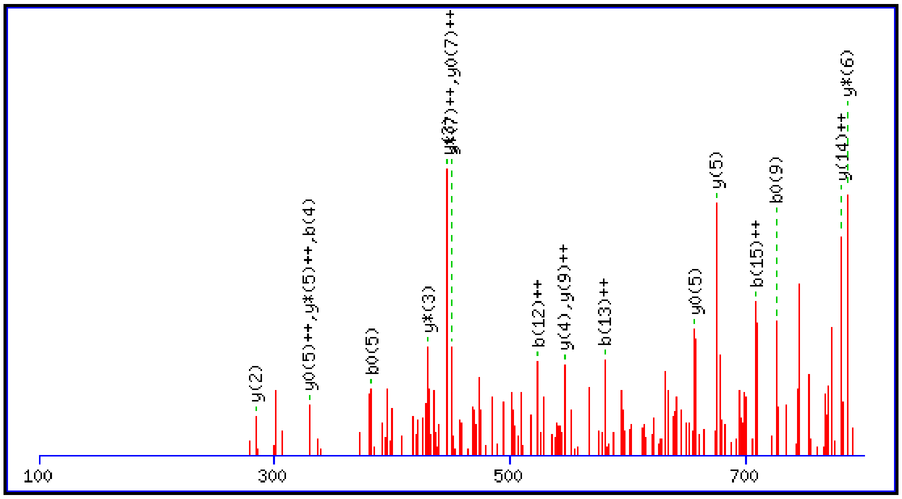

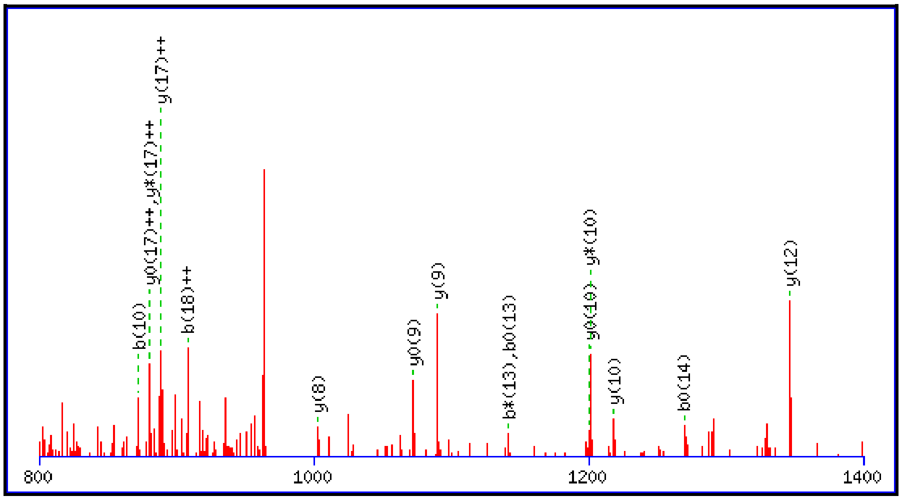

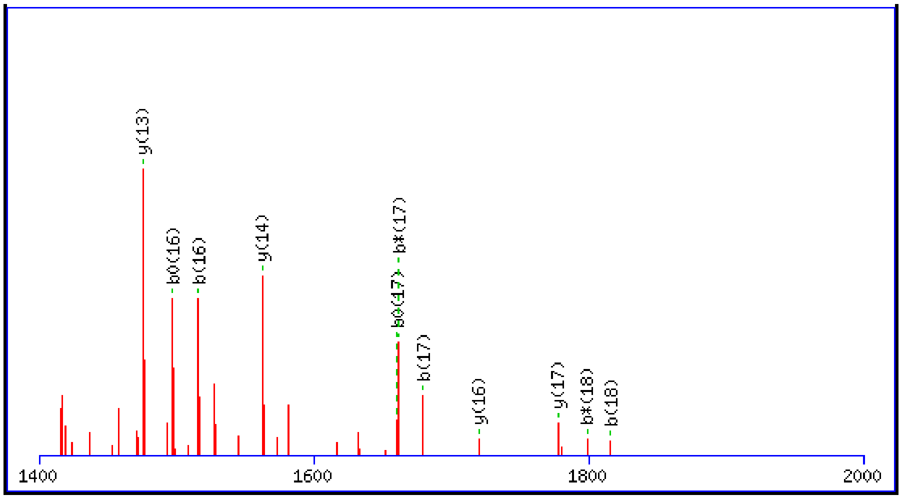

Monoisotopic mass of neutral peptide Mr(calc): 1960.9490
Fixed modifications: Carbamidomethyl (C)
Ions Score: 92 **Expect:** 1.4e-007
Matches (Bold Red): 45/184 fragment ions using 57 most intense peaks

#	b	b^{++}	b*	b^{*++}	b^0	b^{0++}	Seq.	y	y^{++}	y*	y^{*++}	y^0	y^{0++}	#
1	72.0444	36.5258					A							19
2	185.1285	93.0679					I	1890.9192	945.9632	1873.8926	937.4500	1872.9086	936.9580	18
3	242.1499	121.5786					G	**1777.8351**	**889.4212**	1760.8086	**880.9079**	1759.8246	**880.4159**	17
4	**329.1819**	165.0946			311.1714	156.0893	S	**1720.8137**	860.9105	1703.7871	852.3972	1702.8031	851.9052	16
5	400.2191	200.6132			**382.2085**	191.6079	A	1633.7816	817.3945	1616.7551	808.8812	1615.7711	808.3892	15
6	487.2511	244.1292			469.2405	235.1239	S	**1562.7445**	**781.8759**	1545.7180	773.3626	1544.7340	772.8706	14
7	616.2937	308.6505			598.2831	299.6452	E	**1475.7125**	738.3599	1458.6860	729.8466	1457.7019	729.3546	13
8	673.3151	337.1612			655.3046	328.1559	G	**1346.6699**	673.8386	1329.6434	665.3253	1328.6593	664.8333	12
9	744.3523	372.6798			**726.3417**	363.6745	A	1289.6484	645.3279	1272.6219	636.8146	1271.6379	636.3226	11
10	**872.4108**	436.7091	855.3843	428.1958	854.4003	427.7038	Q	**1218.6113**	609.8093	**1201.5848**	601.2960	**1200.6008**	600.8040	10
11	959.4429	480.2251	942.4163	471.7118	941.4323	471.2198	S	**1090.5528**	**545.7800**	1073.5262	537.2667	**1072.5422**	536.7747	9
12	1046.4749	**523.7411**	1029.4483	515.2278	1028.4643	514.7358	S	**1003.5207**	502.2640	986.4942	493.7507	985.5102	493.2587	8
13	1159.5590	**580.2831**	**1142.5324**	571.7698	**1141.5484**	571.2778	L	916.4887	458.7480	899.4621	**450.2347**	898.4781	**449.7427**	7
14	1287.6175	644.3124	1270.5910	635.7991	**1269.6070**	635.3071	Q	803.4046	402.2060	**786.3781**	393.6927	785.3941	393.2007	6
15	1416.6601	**708.8337**	1399.6336	700.3204	1398.6496	699.8284	E	**675.3461**	338.1767	658.3195	**329.6634**	**657.3355**	**329.1714**	5
16	**1515.7285**	758.3679	1498.7020	749.8546	**1497.7180**	749.3626	V	**546.3035**	273.6554	529.2769	265.1421			4
17	**1678.7919**	839.8996	**1661.7653**	831.3863	**1660.7813**	830.8943	Y	**447.2350**	224.1212	**430.2085**	215.6079			3
18	**1815.8508**	**908.4290**	**1798.8242**	899.9158	1797.8402	899.4237	H	**284.1717**	142.5895	267.1452	134.0762			2
19							K	147.1128	74.0600	130.0863	65.5468			1

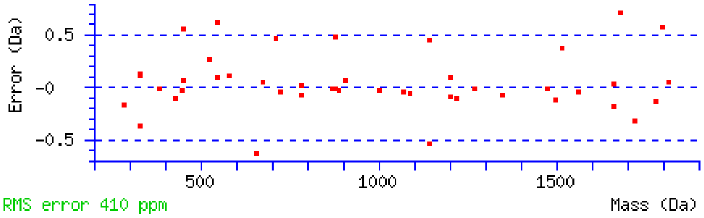

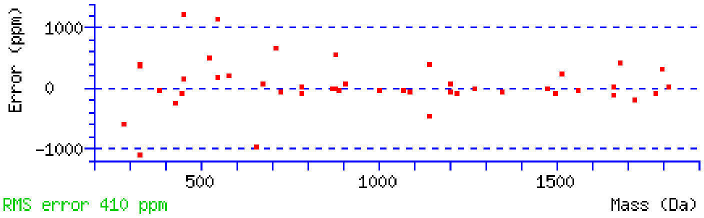

All matches to this query

Score	Mr(calc):	Delta	Sequence
92.3	1960.9490	-0.0012	AIGSASEGAQSSLQEVYHK
6.4	1960.9520	-0.0042	STLTVHQRTHGEKPYK
4.8	1958.9432	2.0046	EGGAIEEEAKEKTSEAPK

4.0	1960.9490	-0.0012	ATPELGSSENSASSPPRFK
3.0	1959.9481	0.9997	ANNVSPMTCLKKHWMK
2.4	1959.9455	1.0023	LPQFGISTPGSDLHVNAK
1.6	1960.9673	-0.0194	YGVSHPDAGAAWRLLLR
0.8	1959.9636	0.9842	LSKSNIDISSGLEDEEPK
0.3	1959.9455	1.0023	AINEFFRTEGAEKLQK
0.3	1960.9589	-0.0111	SDVMMKYMGLKLGPALK

Spectrum No: 39; Query: 11638; Rank: 1

Peptide View

MS/MS Fragmentation of **LFQVEYAIEAIK**
Found in **IPI00291922**, Tax_Id=9606 Gene_Symbol=PSMA5 Proteasome subunit alpha type-5

Match to Query 11638: 1422.778108 from(712.396330,2+)
Title: 090324LimSK_Exosome1_B.15141.15141.2.dta
Data file C:\Temp\Mascoti\LSK_2D\090324LimSK_Exosome1_A_2D.mgf

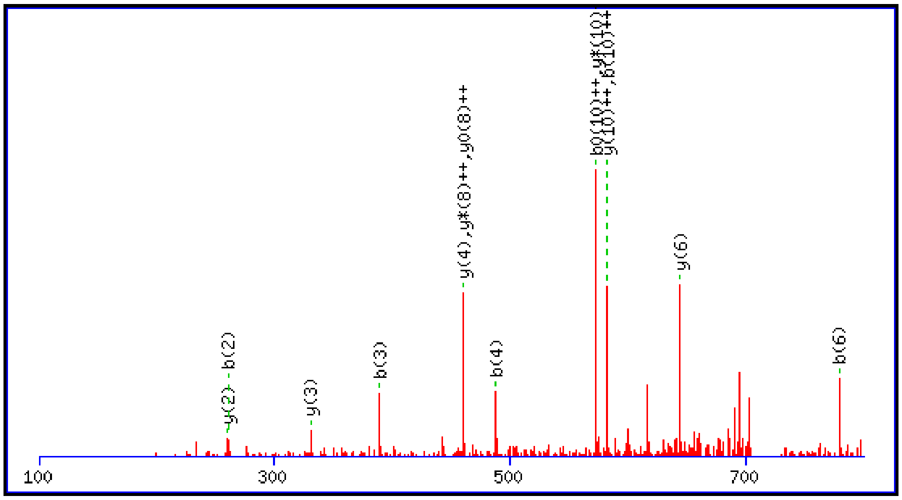

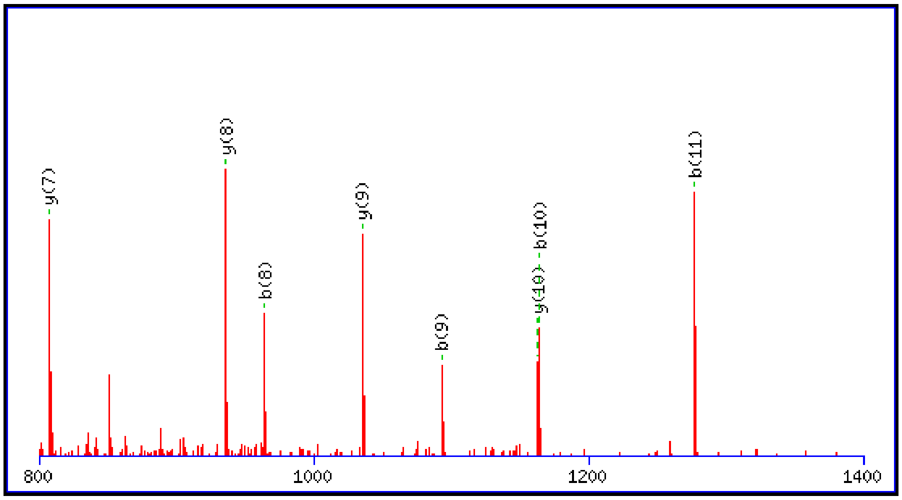

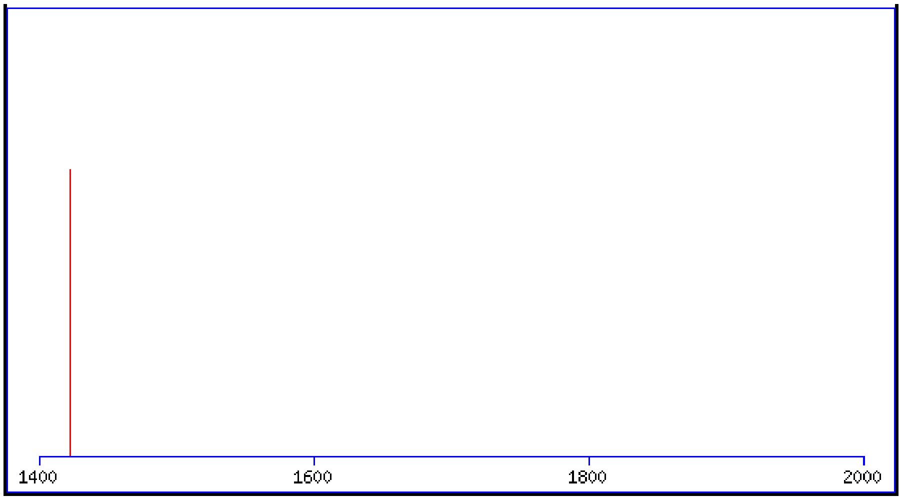

Monoisotopic mass of neutral peptide Mr(calc): 1422.7758
Fixed modifications: Carbamidomethyl (C)
Ions Score: 85 **Expect:** 2.6e-007
Matches (Bold Red): 24/114 fragment ions using 24 most intense peaks

#	b	b^{++}	b*	b^{*++}	b^0	b^{0++}	Seq.	y	y^{++}	y*	y^{*++}	y^0	y^{0++}	#
1	114.0913	57.5493					L							12
2	**261.1598**	131.0835					F	1310.6991	655.8532	1293.6725	647.3399	1292.6885	646.8479	11
3	**389.2183**	195.1128	372.1918	186.5995			Q	**1163.6307**	**582.3190**	1146.6041	**573.8057**	1145.6201	**573.3137**	10
4	**488.2867**	244.6470	471.2602	236.1337			V	**1035.5721**	518.2897	1018.5455	509.7764	1017.5615	509.2844	9
5	617.3293	309.1683	600.3028	300.6550	599.3188	300.1630	E	**936.5037**	468.7555	919.4771	**460.2422**	918.4931	**459.7502**	8
6	**780.3927**	390.7000	763.3661	382.1867	762.3821	381.6947	Y	**807.4611**	404.2342	790.4345	395.7209	789.4505	395.2289	7
7	851.4298	426.2185	834.4032	417.7053	833.4192	417.2132	A	**644.3978**	322.7025	627.3712	314.1892	626.3872	313.6972	6
8	**964.5138**	482.7606	947.4873	474.2473	946.5033	473.7553	I	**573.3606**	287.1840	556.3341	278.6707	555.3501	278.1787	5
9	**1093.5564**	547.2819	1076.5299	538.7686	1075.5459	538.2766	E	**460.2766**	230.6419	443.2500	222.1287	442.2660	221.6366	4
10	**1164.5936**	**582.8004**	1147.5670	574.2871	1146.5830	**573.7951**	A	**331.2340**	166.1206	314.2074	157.6074			3
11	**1277.6776**	639.3424	1260.6511	630.8292	1259.6671	630.3372	I	**260.1969**	130.6021	243.1703	122.0888			2
12							K	147.1128	74.0600	130.0863	65.5468			1

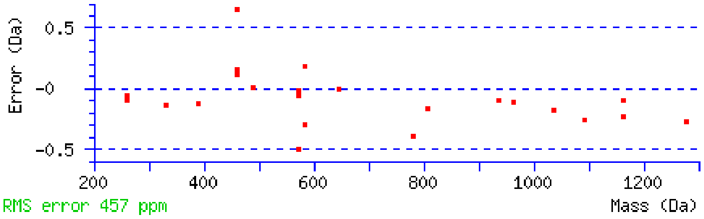

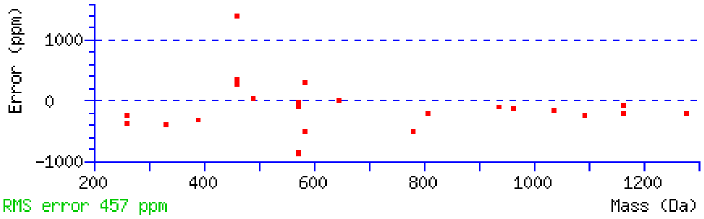

All matches to this query

Score	Mr(calc):	Delta	Sequence
85.3	1422.7758	0.0023	LFQVEYAIEAIK
15.3	1421.7643	1.0138	KNGTNVIVEKLK
14.8	1421.7739	1.0042	KQEQRHEVIQK
14.2	1422.7718	0.0063	AHLEEISALVVDK
13.5	1422.7752	0.0029	MLKGISSSSLKEK
13.2	1422.7710	0.0071	VVWMQTLLILK
13.0	1422.7878	-0.0096	VGHRMNIERALK
11.7	1422.7718	0.0063	AALDSPPGALAELAK
11.6	1421.7813	0.9968	SRLRMFLNELK
11.3	1421.7667	1.0114	APYRIFQTDAIK

Spectrum No: 40; Query: 16207; Rank: 1

Peptide View

MS/MS Fragmentation of **SEYDRGVNTFSPEGR**
Found in **IPI00291922**, Tax_Id=9606 Gene_Symbol=PSMA5 Proteasome subunit alpha type-5

Match to Query 16207: 1712.778882 from(571.933570,3+)
Title: 090324LimSK_Exosome1_A.3597.3597.3.dta
Data file C:\Temp\Mascoti\LSK_2D\090324LimSK_Exosome1_A_2D.mgf

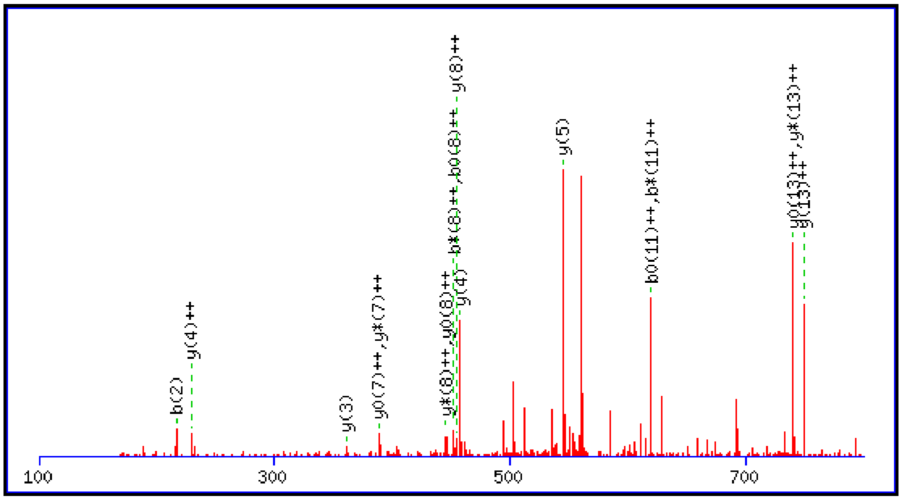

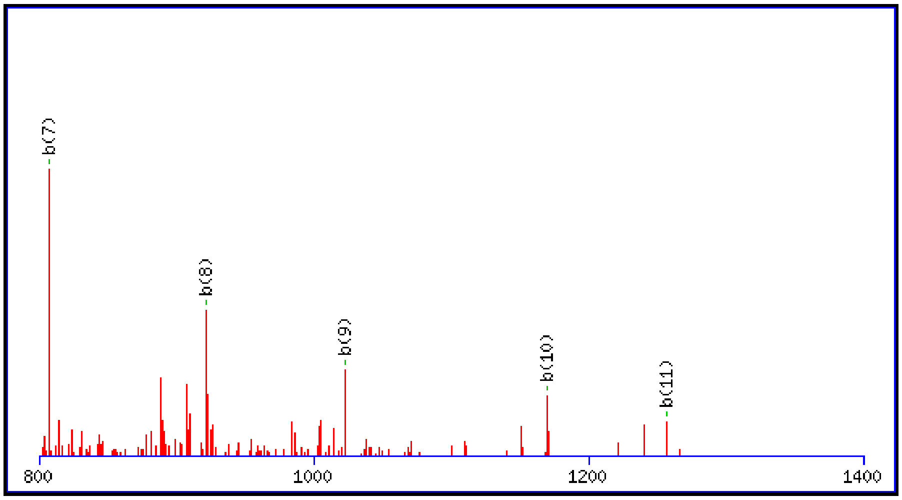

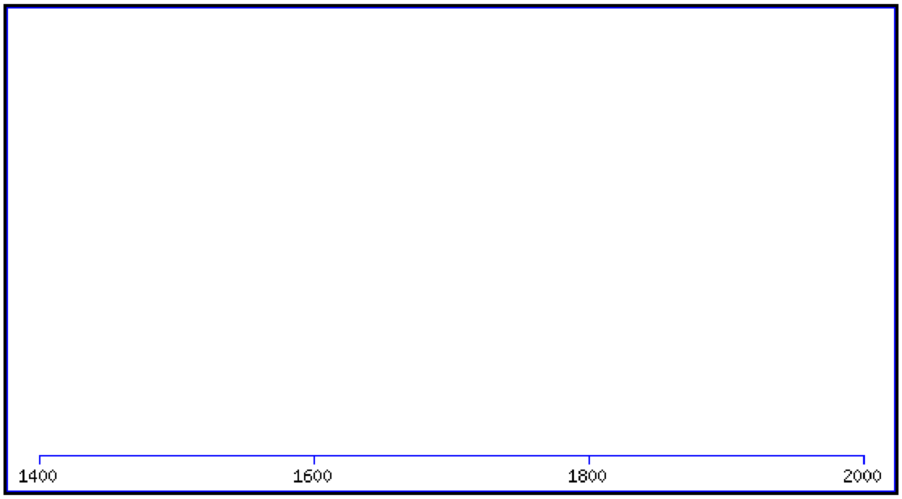

Monoisotopic mass of neutral peptide Mr(calc): 1712.7754
Fixed modifications: Carbamidomethyl (C)
Ions Score: 54 **Expect:** 0.00084
Matches (Bold Red): 22/156 fragment ions using 22 most intense peaks

#	b	b^{++}	b*	b^{*++}	b^{0}	b^{0++}	Seq.	y	y^{++}	y*	y^{*++}	y^{0}	y^{0++}	#
1	88.0393	44.5233			70.0287	35.5180	S							15
2	**217.0819**	109.0446			199.0713	100.0393	E	1626.7507	813.8790	1609.7241	805.3657	1608.7401	804.8737	14
3	380.1452	190.5763			362.1347	181.5710	Y	1497.7081	**749.3577**	1480.6815	**740.8444**	1479.6975	**740.3524**	13
4	495.1722	248.0897			477.1616	239.0844	D	1334.6448	667.8260	1317.6182	659.3127	1316.6342	658.8207	12
5	651.2733	326.1403	634.2467	317.6270	633.2627	317.1350	R	1219.6178	610.3125	1202.5913	601.7993	1201.6072	601.3073	11
6	708.2947	354.6510	691.2682	346.1377	690.2842	345.6457	G	1063.5167	532.2620	1046.4902	523.7487	1045.5061	523.2567	10
7	**807.3632**	404.1852	790.3366	395.6719	789.3526	395.1799	V	1006.4952	503.7513	989.4687	495.2380	988.4847	494.7460	9
8	**921.4061**	461.2067	904.3795	**452.6934**	903.3955	**452.2014**	N	907.4268	**454.2170**	890.4003	**445.7038**	889.4163	**445.2118**	8
9	**1022.4538**	511.7305	1005.4272	503.2172	1004.4432	502.7252	T	793.3839	397.1956	776.3573	**388.6823**	775.3733	**388.1903**	7
10	**1169.5222**	585.2647	1152.4956	576.7515	1151.5116	576.2594	F	692.3362	346.6717	675.3097	338.1585	674.3257	337.6665	6
11	**1256.5542**	628.7807	1239.5277	**620.2675**	1238.5436	**619.7755**	S	**545.2678**	273.1375	528.2413	264.6243	527.2572	264.1323	5
12	1353.6070	677.3071	1336.5804	668.7938	1335.5964	668.3018	P	**458.2358**	**229.6215**	441.2092	221.1082	440.2252	220.6162	4
13	1482.6496	741.8284	1465.6230	733.3151	1464.6390	732.8231	E	**361.1830**	181.0951	344.1565	172.5819	343.1724	172.0899	3
14	1539.6710	770.3392	1522.6445	761.8259	1521.6605	761.3339	G	232.1404	116.5738	215.1139	108.0606			2
15							R	175.1190	88.0631	158.0924	79.5498			1

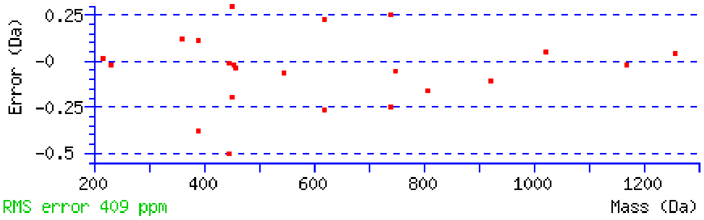

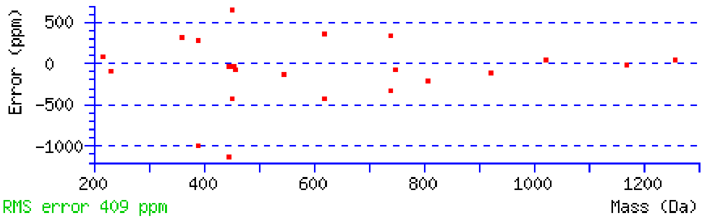

All matches to this query

Score	Mr(calc):	Delta	Sequence
54.2	1712.7754	0.0035	SEYDRGVNTFSPEGR
17.2	1712.7746	0.0043	AELCAFTLFYSRR
16.5	1710.7855	1.9934	SNGRPPTAAAVPKSAK
15.8	1712.7746	0.0043	AELCAFTLFYSRR
15.2	1710.7855	1.9934	SNGRPPTAAAVPKSAK
10.8	1710.7855	1.9933	TSVGSQRSRVSFLK
8.8	1712.7787	0.0002	TLNAETPKSSPLPAK
8.5	1710.7776	2.0012	KAGITSAMATRTSLK
7.7	1710.7807	1.9982	VGGSGMAGCRSRALPR
7.3	1711.7836	0.9953	NMSFVNDLTVTQDGR

Spectrum No: 41; Query: 34427; Rank: 1

Peptide View

MS/MS Fragmentation of **AATLLAARGAGPAPAWGPEAFTPDWESR**
Found in **IPI00000811**, Tax_Id=9606 Gene_Symbol=PSMB6 Proteasome subunit beta type-6

Match to Query 34427: 3027.348732 from(1010.123520,3+)
Title: 090324LimSK_Exosome1_B.9839.9839.3.dta
Data file C:\Temp\Mascoti\LSK_2D\090324LimSK_Exosome1_A_2D.mgf

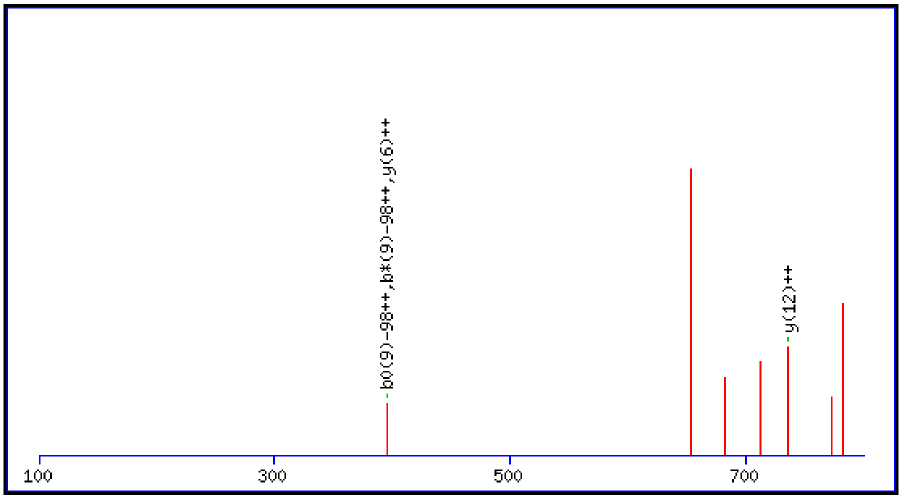

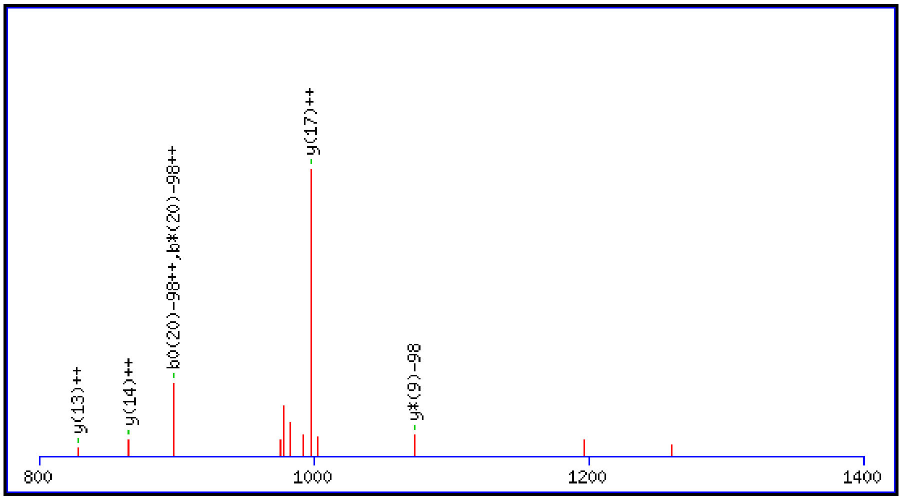

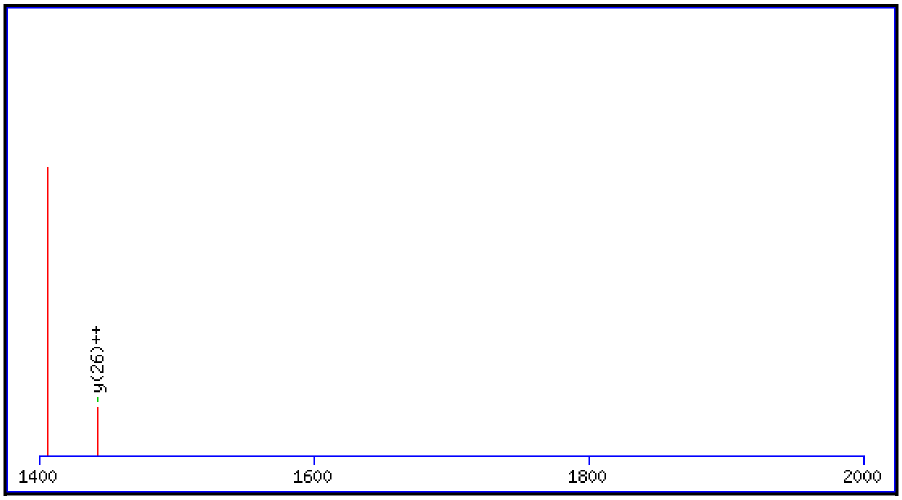

Monoisotopic mass of neutral peptide Mr(calc): 3025.3524
Fixed modifications: Carbamidomethyl (C)
Variable modifications:
T3 : Phospho (ST), with neutral losses 0.0000(shown in table), 97.9769
T22 : Phospho (ST), with neutral losses 0.0000(shown in table), 97.9769
Ions Score: 23 **Expect:** 1.7
Matches (Bold Red): 11/570 fragment ions using 16 most intense peaks

#	b	b^{++}	b*	b^{*++}	b^0	b^{0++}	Seq.	y	y^{++}	y*	y^{*++}	y^0	y^{0++}	#
1	72.0444	36.5258					A							28
2	143.0815	72.0444					A	2955.3226	1478.1650	2938.2961	1469.6517	2937.3121	1469.1597	27
3	324.0955	162.5514			306.0849	153.5461	T	2884.2855	**1442.6464**	2867.2590	1434.1331	2866.2750	1433.6411	26
4	437.1796	219.0934			419.1690	210.0881	L	2703.2715	1352.1394	2686.2450	1343.6261	2685.2610	1343.1341	25

**5**	550.2636	275.6355			532.2531	266.6302	**L**	2590.1875	1295.5974	2573.1609	1287.0841	2572.1769	1286.5921	**24**
**6**	621.3007	311.1540			603.2902	302.1487	**A**	2477.1034	1239.0553	2460.0768	1230.5421	2459.0928	1230.0501	**23**
**7**	692.3379	346.6726			674.3273	337.6673	**A**	2406.0663	1203.5368	2389.0397	1195.0235	2388.0557	1194.5315	**22**
**8**	848.4390	424.7231	831.4124	416.2099	830.4284	415.7178	**R**	2335.0292	1168.0182	2318.0026	1159.5049	2317.0186	1159.0129	**21**
**9**	905.4604	453.2339	888.4339	444.7206	887.4499	444.2286	**G**	2178.9281	1089.9677	2161.9015	1081.4544	2160.9175	1080.9624	**20**
**10**	976.4976	488.7524	959.4710	480.2391	958.4870	479.7471	**A**	2121.9066	1061.4569	2104.8800	1052.9437	2103.8960	1052.4517	**19**
**11**	1033.5190	517.2631	1016.4925	508.7499	1015.5085	508.2579	**G**	2050.8695	1025.9384	2033.8429	1017.4251	2032.8589	1016.9331	**18**
**12**	1130.5718	565.7895	1113.5452	557.2763	1112.5612	556.7842	**P**	1993.8480	**997.4276**	1976.8215	988.9144	1975.8374	988.4224	**17**
**13**	1201.6089	601.3081	1184.5823	592.7948	1183.5983	592.3028	**A**	1896.7952	948.9013	1879.7687	940.3880	1878.7847	939.8960	**16**
**14**	1298.6617	649.8345	1281.6351	641.3212	1280.6511	640.8292	**P**	1825.7581	913.3827	1808.7316	904.8694	1807.7476	904.3774	**15**
**15**	1369.6988	685.3530	1352.6722	676.8397	1351.6882	676.3477	**A**	1728.7054	**864.8563**	1711.6788	856.3430	1710.6948	855.8510	**14**
**16**	1555.7781	778.3927	1538.7515	769.8794	1537.7675	769.3874	**W**	1657.6683	**829.3378**	1640.6417	820.8245	1639.6577	820.3325	**13**
**17**	1612.7995	806.9034	1595.7730	798.3901	1594.7890	797.8981	**G**	1471.5889	**736.2981**	1454.5624	727.7848	1453.5784	727.2928	**12**
**18**	1709.8523	855.4298	1692.8258	846.9165	1691.8417	846.4245	**P**	1414.5675	707.7874	1397.5409	699.2741	1396.5569	698.7821	**11**
**19**	1838.8949	919.9511	1821.8684	911.4378	1820.8843	910.9458	**E**	1317.5147	659.2610	1300.4882	650.7477	1299.5042	650.2557	**10**
**20**	1909.9320	955.4696	1892.9055	946.9564	1891.9215	946.4644	**A**	1188.4721	594.7397	1171.4456	586.2264	1170.4616	585.7344	**9**
**21**	2057.0004	1029.0039	2039.9739	1020.4906	2038.9899	1019.9986	**F**	1117.4350	559.2211	1100.4085	550.7079	1099.4244	550.2159	**8**
**22**	2238.0144	1119.5109	2220.9879	1110.9976	2220.0039	1110.5056	**T**	970.3666	485.6869	953.3400	477.1737	952.3560	476.6817	**7**
**23**	2335.0672	1168.0372	2318.0407	1159.5240	2317.0566	1159.0320	**P**	789.3526	**395.1799**	772.3260	386.6667	771.3420	386.1747	**6**
**24**	2450.0941	1225.5507	2433.0676	1217.0374	2432.0836	1216.5454	**D**	692.2998	346.6536	675.2733	338.1403	674.2893	337.6483	**5**
**25**	2636.1735	1318.5904	2619.1469	1310.0771	2618.1629	1309.5851	**W**	577.2729	289.1401	560.2463	280.6268	559.2623	280.1348	**4**
**26**	2765.2160	1383.1117	2748.1895	1374.5984	2747.2055	1374.1064	**E**	391.1936	196.1004	374.1670	187.5872	373.1830	187.0951	**3**
**27**	2852.2481	1426.6277	2835.2215	1418.1144	2834.2375	1417.6224	**S**	262.1510	131.5791	245.1244	123.0659	244.1404	122.5738	**2**
**28**							**R**	175.1190	88.0631	158.0924	79.5498			**1**

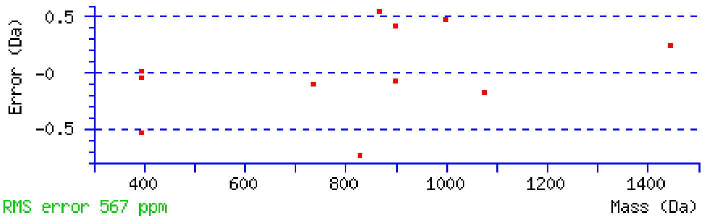

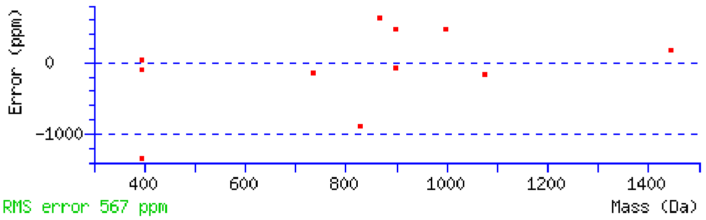

All matches to this query

Score	Mr(calc):	Delta	Sequence
23.0	3025.3524	1.9963	AATLLAARGAGPAPAWGPEAFTPDWESR
17.7	3027.3514	-0.0026	SPRGTGCSAGLLMTVGWLLLAGLQSAR
17.7	3027.3514	-0.0026	SPRGTGCSAGLLMTVGWLLLAGLQSAR
16.3	3027.3239	0.0249	ENLFSFQTATTTMQAISVFRGYAER
16.3	3027.3239	0.0249	ENLFSFQTATTTMQAISVFRGYAER
16.3	3027.3239	0.0249	ENLFSFQTATTTMQAISVFRGYAER
16.1	3027.3239	0.0249	ENLFSFQTATTTMQAISVFRGYAER
16.1	3027.3239	0.0249	ENLFSFQTATTTMQAISVFRGYAER
16.1	3027.3239	0.0249	ENLFSFQTATTTMQAISVFRGYAER
16.1	3027.3239	0.0249	ENLFSFQTATTTMQAISVFRGYAER

Spectrum No: 42; Query: 25181; Rank: 1

Peptide View

MS/MS Fragmentation of **LAAIAESGVERQVLLGDQIPK**
Found in **IPI00000811**, Tax_Id=9606 Gene_Symbol=PSMB6 Proteasome subunit beta type-6

Match to Query 25181: 2206.234332 from(736.418720,3+)
Title: 090324LimSK_Exosome1_B.11440.11440.3.dta
Data file C:\Temp\Mascoti\LSK_2D\090324LimSK_Exosome1_A_2D.mgf

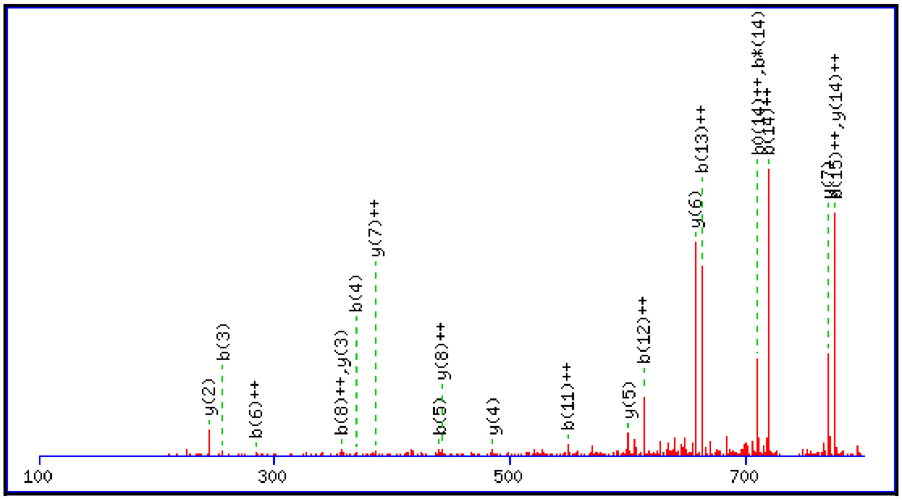

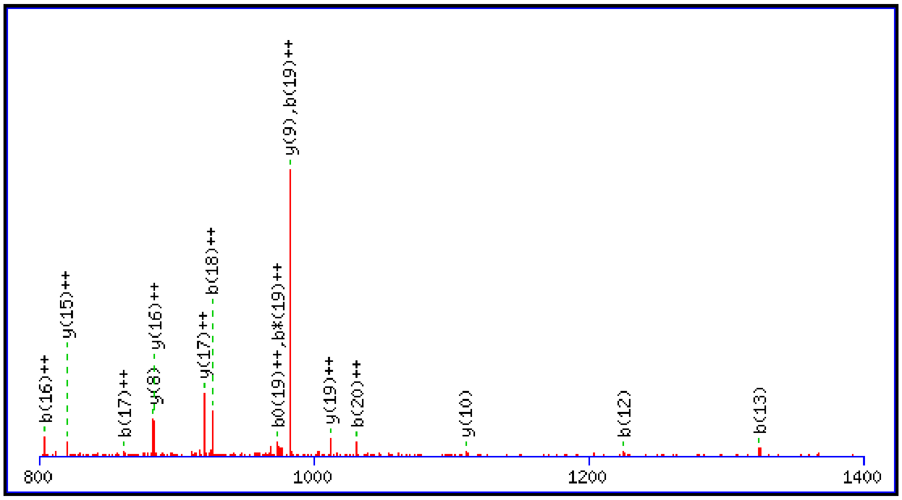

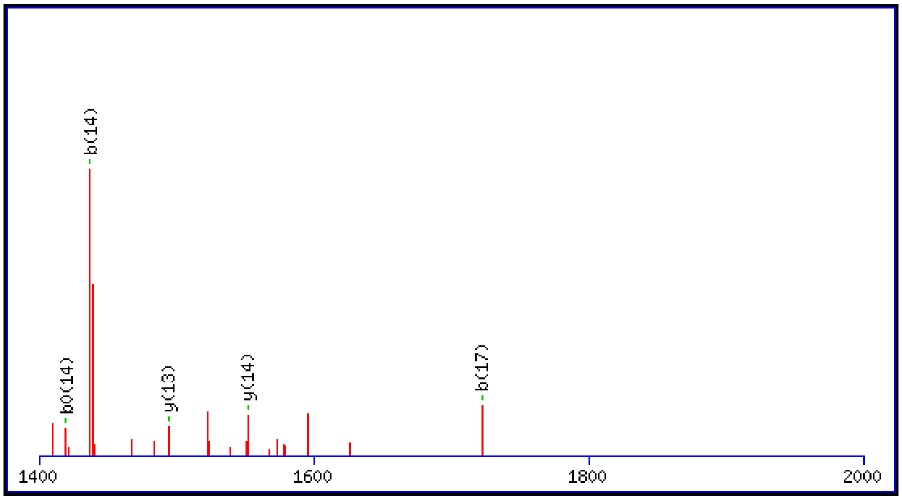

Monoisotopic mass of neutral peptide Mr(calc): 2206.2321
Fixed modifications: Carbamidomethyl (C)
Ions Score: 77 **Expect:** 6.9e-007
Matches (Bold Red): 42/202 fragment ions using 60 most intense peaks

#	b	b^{++}	b*	b^{*++}	b^0	b^{0++}	Seq.	y	y^{++}	y*	y^{*++}	y^0	y^{0++}	#
1	114.0913	57.5493					L							21

**2**	185.1285	93.0679					**A**	2094.1553	1047.5813	2077.1288	1039.0680	2076.1448	1038.5760	**20**
**3**	**256.1656**	128.5864					**A**	2023.1182	**1012.0628**	2006.0917	1003.5495	2005.1077	1003.0575	**19**
**4**	**369.2496**	185.1285					**I**	1952.0811	976.5442	1935.0546	968.0309	1934.0706	967.5389	**18**
**5**	**440.2867**	220.6470					**A**	1838.9971	**920.0022**	1821.9705	911.4889	1820.9865	910.9969	**17**
**6**	569.3293	**285.1683**			551.3188	276.1630	**E**	1767.9599	**884.4836**	1750.9334	875.9703	1749.9494	875.4783	**16**
**7**	656.3614	328.6843			638.3508	319.6790	**S**	1638.9173	**819.9623**	1621.8908	811.4490	1620.9068	810.9570	**15**
**8**	713.3828	**357.1951**			695.3723	348.1898	**G**	**1551.8853**	**776.4463**	1534.8588	767.9330	1533.8748	767.4410	**14**
**9**	812.4512	406.7293			794.4407	397.7240	**V**	**1494.8639**	747.9356	1477.8373	739.4223	1476.8533	738.9303	**13**
**10**	941.4938	471.2506			923.4833	462.2453	**E**	1395.7954	698.4014	1378.7689	689.8881	1377.7849	689.3961	**12**
**11**	1097.5949	**549.3011**	1080.5684	540.7878	1079.5844	540.2958	**R**	1266.7528	633.8801	1249.7263	625.3668	1248.7423	624.8748	**11**
**12**	**1225.6535**	**613.3304**	1208.6270	604.8171	1207.6430	604.3251	**Q**	**1110.6517**	555.8295	1093.6252	547.3162	1092.6412	546.8242	**10**
**13**	**1324.7219**	**662.8646**	1307.6954	654.3513	1306.7114	653.8593	**V**	**982.5932**	491.8002	965.5666	483.2869	964.5826	482.7949	**9**
**14**	**1437.8060**	**719.4066**	1420.7795	**710.8934**	**1419.7954**	**710.4014**	**L**	**883.5247**	**442.2660**	866.4982	433.7527	865.5142	433.2607	**8**
**15**	1550.8901	**775.9487**	1533.8635	767.4354	1532.8795	766.9434	**L**	**770.4407**	**385.7240**	753.4141	377.2107	752.4301	376.7187	**7**
**16**	1607.9115	**804.4594**	1590.8850	795.9461	1589.9010	795.4541	**G**	**657.3566**	329.1819	640.3301	320.6687	639.3461	320.1767	**6**
**17**	**1722.9385**	**861.9729**	1705.9119	853.4596	1704.9279	852.9676	**D**	**600.3352**	300.6712	583.3086	292.1579	582.3246	291.6659	**5**
**18**	1850.9971	**926.0022**	1833.9705	917.4889	1832.9865	916.9969	**Q**	**485.3082**	243.1577	468.2817	234.6445			**4**
**19**	1964.0811	**982.5442**	1947.0546	**974.0309**	1946.0706	**973.5389**	**I**	**357.2496**	179.1285	340.2231	170.6152			**3**
**20**	2061.1339	**1031.0706**	2044.1073	1022.5573	2043.1233	1022.0653	**P**	**244.1656**	122.5864	227.1390	114.0731			**2**
**21**							**K**	147.1128	74.0600	130.0863	65.5468			**1**

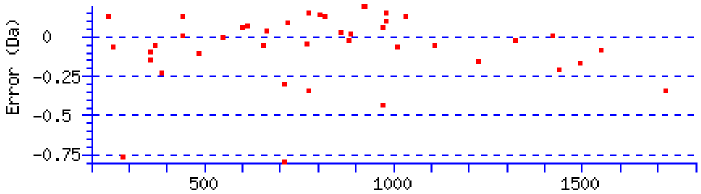

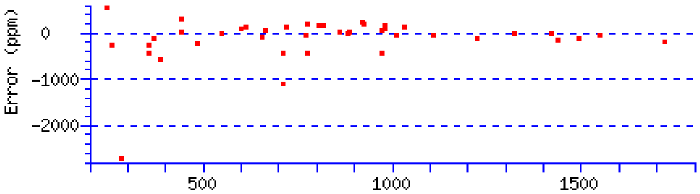

All matches to this query

Score	Mr(calc):	Delta	Sequence
77.3	2206.2321	0.0022	LAAIAESGVERQVLLGDQIPK
11.7	2205.2270	1.0073	ILVDQVTGVSRGVGFIRFDK
9.4	2204.2140	2.0204	TLGDPVALLVLLWCPEPRR
8.9	2205.2094	1.0250	KLLKEAVVLPMWMPEFFK
8.5	2205.2481	0.9862	GVTQRLDLPDLPLSDILKGR
6.9	2206.2222	0.0121	VGSQLEVLVHVQDFQRKPK
6.1	2205.2259	1.0084	GSLGAQLRPALLTLHLPGRR
5.4	2205.2382	0.9961	WVRVLEKQGAASQLALTHAK
4.8	2205.2133	1.0210	ARINESLSQLKTLILDALK
3.1	2204.2235	2.0109	IAVAFPGNLQLIFILRPSR

Spectrum No: 43; Query: 8449; Rank: 1

Peptide View

MS/MS Fragmentation of **QVLLGDQIPK**
Found in **IPI00000811**, Tax_Id=9606 Gene_Symbol=PSMB6 Proteasome subunit beta type-6

Match to Query 8449: 1109.646368 from(555.830460,2+)
Title: 090324LimSK_Exosome1_A.5640.5640.2.dta

Data file C:\Temp\Mascoti\LSK_2D\090324LimSK_Exosome1_A_2D.mgf

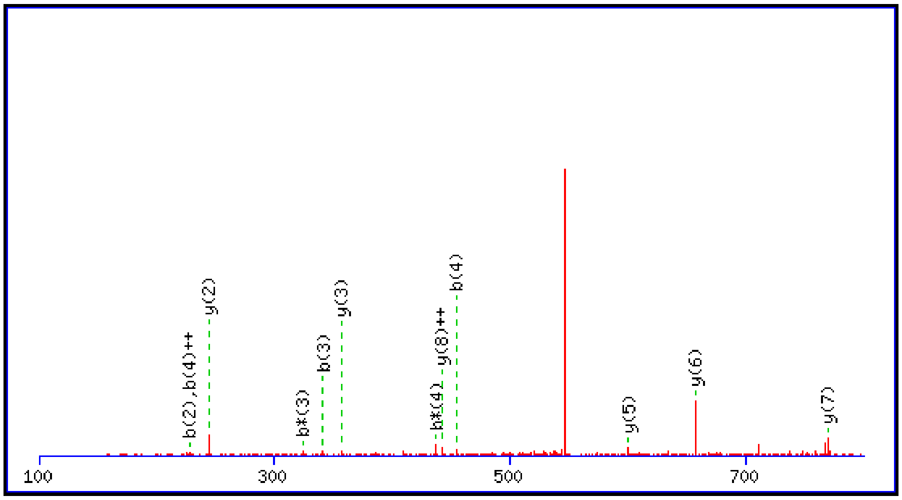

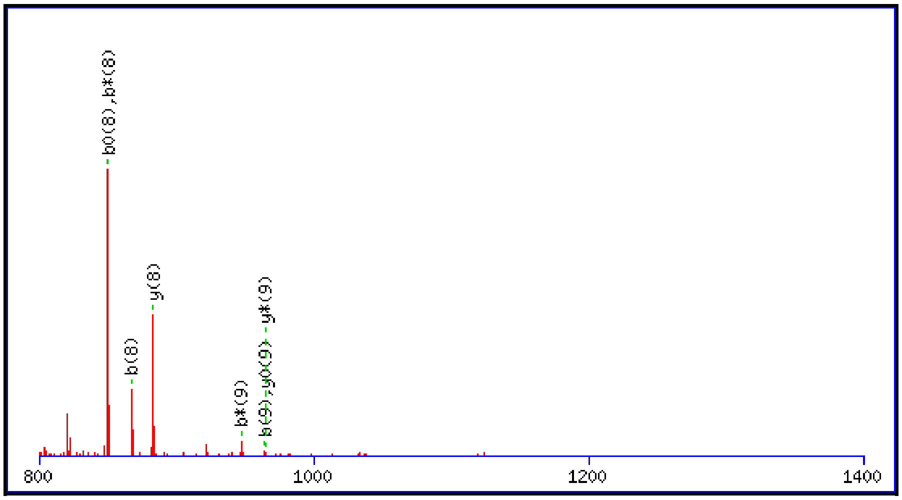

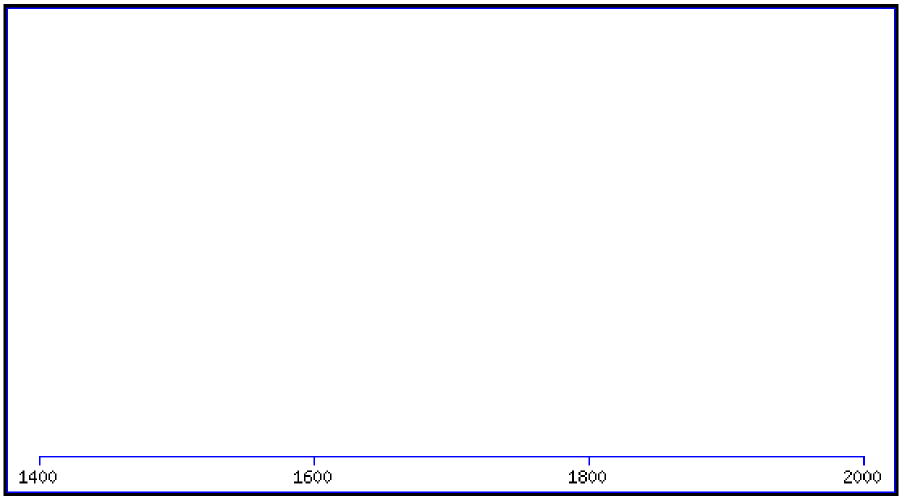

Monoisotopic mass of neutral peptide Mr(calc): 1109.6445
Fixed modifications: Carbamidomethyl (C)
Ions Score: 41 **Expect:** 0.0031
Matches (Bold Red): 20/90 fragment ions using 31 most intense peaks

#	b	b^{++}	b*	b^{*++}	b^0	b^{0++}	Seq.	y	y^{++}	y*	y^{*++}	y^0	y^{0++}	#
1	129.0659	65.0366	112.0393	56.5233			Q							10
2	**228.1343**	114.5708	211.1077	106.0575			V	982.5932	491.8002	**965.5666**	483.2869	**964.5826**	482.7949	9
3	**341.2183**	171.1128	**324.1918**	162.5995			L	**883.5247**	**442.2660**	866.4982	433.7527	865.5142	433.2607	8
4	**454.3024**	**227.6548**	**437.2758**	219.1416			L	**770.4407**	385.7240	753.4141	377.2107	752.4301	376.7187	7
5	511.3239	256.1656	494.2973	247.6523			G	**657.3566**	329.1819	640.3301	320.6687	639.3461	320.1767	6

6	626.3508	313.6790	609.3243	305.1658	608.3402	304.6738	D	**600.3352**	300.6712	583.3086	292.1579	582.3246	291.6659	5
7	754.4094	377.7083	737.3828	369.1951	736.3988	368.7030	Q	485.3082	243.1577	468.2817	234.6445			4
8	**867.4934**	434.2504	**850.4669**	425.7371	**849.4829**	425.2451	I	**357.2496**	179.1285	340.2231	170.6152			3
9	**964.5462**	482.7767	**947.5197**	474.2635	946.5356	473.7715	P	**244.1656**	122.5864	227.1390	114.0731			2
10							K	147.1128	74.0600	130.0863	65.5468			1

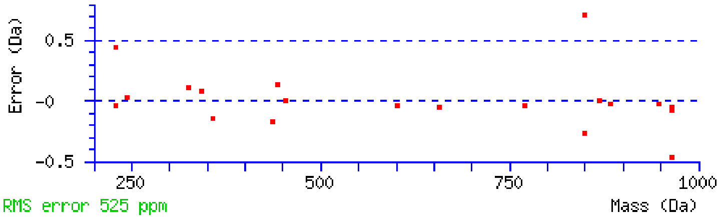

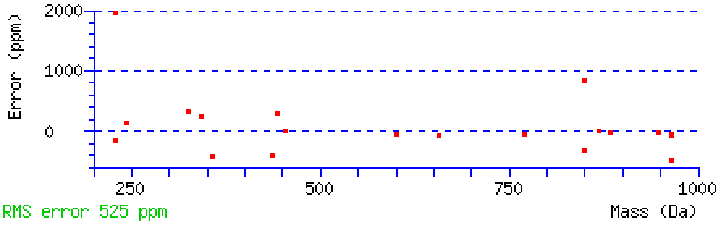

All matches to this query

Score	Mr(calc):	Delta	Sequence
41.5	1109.6445	0.0019	QVLLGDQIPK
18.0	1109.6444	0.0019	DKDGKPLLPK
11.6	1109.6557	-0.0093	RAPQITTPVK
10.6	1109.6557	-0.0093	LNREQLLPK
9.8	1109.6557	-0.0093	RAIPANVEIK
8.0	1109.6557	-0.0093	RALEQVPGLK
6.9	1109.6444	0.0019	GPKLDLKDPK
6.8	1109.6557	-0.0093	LLIRENQPK
3.9	1108.6492	0.9972	LIVQSPIDPK
3.5	1109.6557	-0.0093	GGLQPAVLSLR

Spectrum No: 44; Query: 22505; Rank: 1

Peptide View

MS/MS Fragmentation of **VTDKLTPIHDRIFCCR**
Found in **IPI00000811**, Tax_Id=9606 Gene_Symbol=PSMB6 Proteasome subunit beta type-6

Match to Query 22505: 2030.031016 from(508.515030,4+)
Title: 090324LimSK_Exosome1_B.5409.5409.4.dta
Data file C:\Temp\Mascoti\LSK_2D\090324LimSK_Exosome1_A_2D.mgf

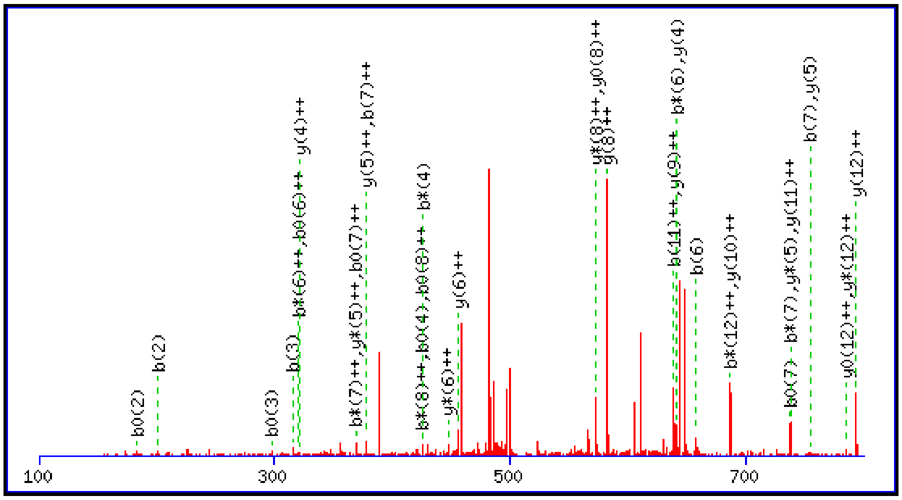

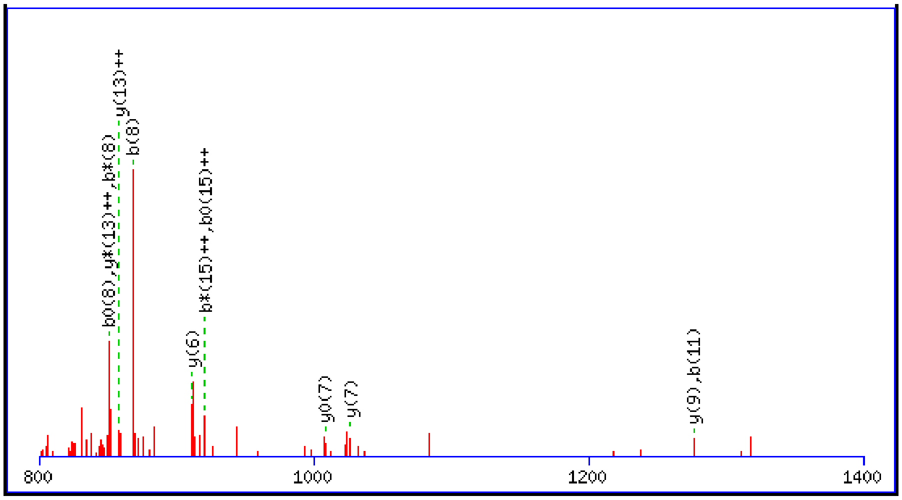

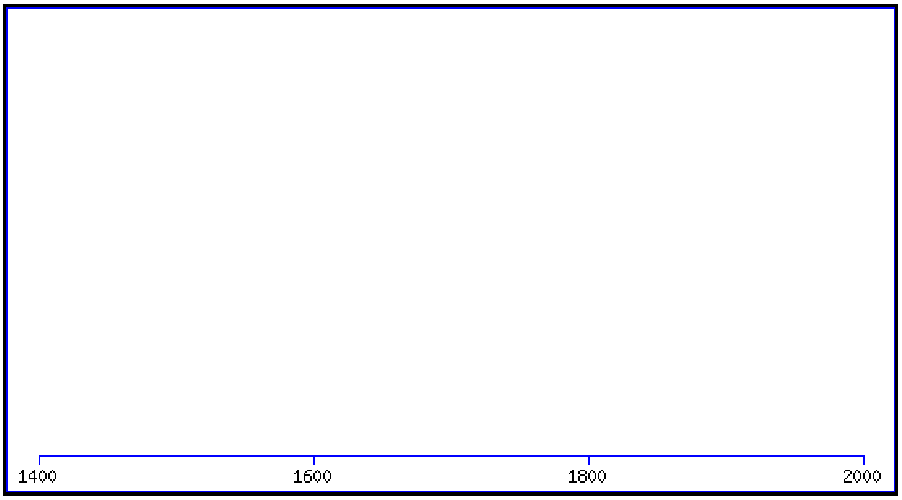

Monoisotopic mass of neutral peptide Mr(calc): 2030.0190
Fixed modifications: Carbamidomethyl (C)
Ions Score: 29 **Expect:** 0.26
Matches (Bold Red): 49/160 fragment ions using 89 most intense peaks

#	b	b^{++}	b*	b^{*++}	b^0	b^{0++}	Seq.	y	y^{++}	y*	y^{*++}	y^0	y^{0++}	#
1	100.0757	50.5415					V							16
2	**201.1234**	101.0653			**183.1128**	92.0600	T	1931.9579	966.4826	1914.9313	957.9693	1913.9473	957.4773	15
3	**316.1503**	158.5788			**298.1397**	149.5735	D	1830.9102	915.9587	1813.8836	907.4454	1812.8996	906.9534	14
4	444.2453	222.6263	**427.2187**	214.1130	**426.2347**	213.6210	K	1715.8832	**858.4453**	1698.8567	**849.9320**	1697.8727	849.4400	13
5	557.3293	279.1683	540.3028	270.6550	539.3188	270.1630	L	1587.7883	**794.3978**	1570.7617	**785.8845**	1569.7777	**785.3925**	12
6	**658.3770**	329.6921	**641.3505**	**321.1789**	640.3665	**320.6869**	T	1474.7042	**737.8557**	1457.6777	729.3425	1456.6936	728.8505	11
7	**755.4298**	**378.2185**	**738.4032**	**369.7053**	**737.4192**	**369.2132**	P	1373.6565	**687.3319**	1356.6300	678.8186	1355.6460	678.3266	10
8	**868.5138**	434.7606	**851.4873**	**426.2473**	**850.5033**	**425.7553**	I	**1276.6038**	**638.8055**	1259.5772	630.2922	1258.5932	629.8002	9
9	1005.5728	503.2900	988.5462	494.7767	987.5622	494.2847	H	1163.5197	**582.2635**	1146.4931	**573.7502**	1145.5091	**573.2582**	8
10	1120.5997	560.8035	1103.5732	552.2902	1102.5891	551.7982	D	**1026.4608**	513.7340	1009.4342	505.2208	**1008.4502**	504.7287	7
11	**1276.7008**	**638.8540**	1259.6743	630.3408	1258.6902	629.8488	R	**911.4338**	**456.2206**	894.4073	**447.7073**			6
12	1389.7849	695.3961	1372.7583	**686.8828**	1371.7743	686.3908	I	**755.3327**	**378.1700**	**738.3062**	**369.6567**			5
13	1536.8533	768.9303	1519.8267	760.4170	1518.8427	759.9250	F	**642.2487**	**321.6280**	625.2221	313.1147			4
14	1696.8839	848.9456	1679.8574	840.4323	1678.8734	839.9403	C	495.1803	248.0938	478.1537	239.5805			3
15	1856.9146	928.9609	1839.8880	**920.4477**	1838.9040	**919.9556**	C	335.1496	168.0784	318.1231	159.5652			2
16							R	175.1190	88.0631	158.0924	79.5498			1

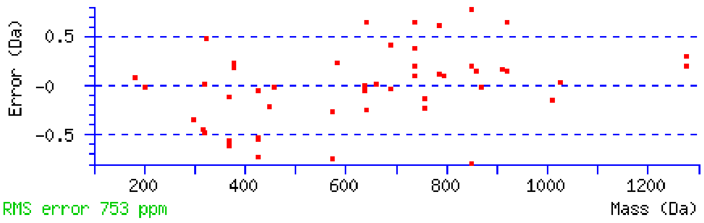

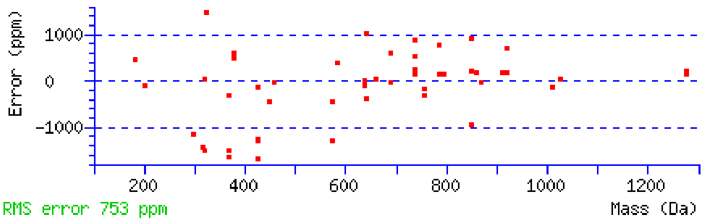

All matches to this query

Score	Mr(calc):	Delta	Sequence
28.5	2030.0190	0.0120	VTDKLTPIHDRIFCCR
8.4	2029.0332	0.9978	NVKSIVPMIHVSWNVAR
6.7	2030.0197	0.0113	LSRSAQLGDAVQLASLPPAG
6.5	2030.0198	0.0112	TPKRPGNSTVTSSPPVTPK
6.2	2030.0198	0.0112	TPKRPGNSTVTSSPPVTPK
5.4	2029.0116	1.0194	TPLAVELEVLDGHDPDPGR
5.4	2030.0197	0.0113	LSRSAQLGDAVQLASLPPAG
5.3	2028.0445	1.9865	KSFLSPRTALINFLVQD
4.4	2030.0216	0.0095	ILLMLLFTIYSISSLK
3.0	2030.0285	0.0025	RSRMEIPVPVQPSWLR

Spectrum No: 45; Query: 16425; Rank: 1

Peptide View

MS/MS Fragmentation of **EVLEKQPVLSQTEAR**
Found in **IPI00555956**, Tax_Id=9606 Gene_Symbol=PSMB4 Proteasome subunit beta type-4

Match to Query 16425: 1725.930282 from(576.317370,3+)
Title: 090324LimSK_Exosome1_A.3144.3144.3.dta
Data file C:\Temp\Mascoti\LSK_2D\090324LimSK_Exosome1_A_2D.mgf

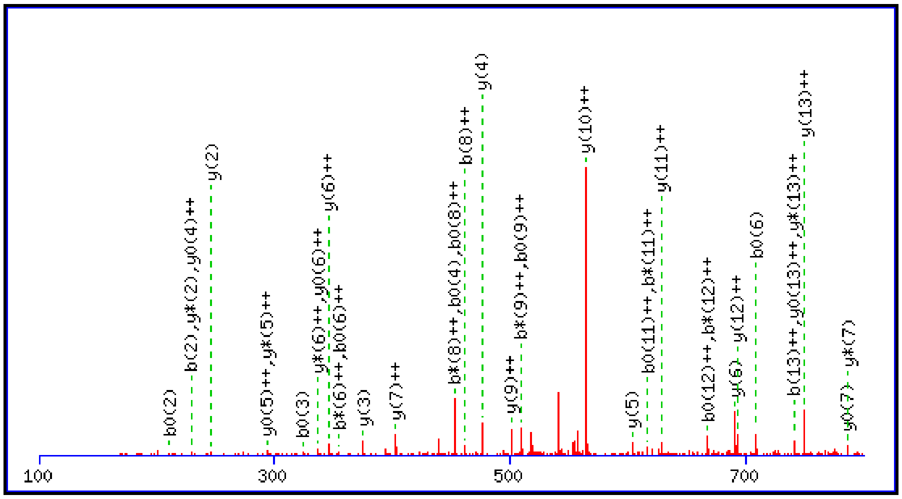

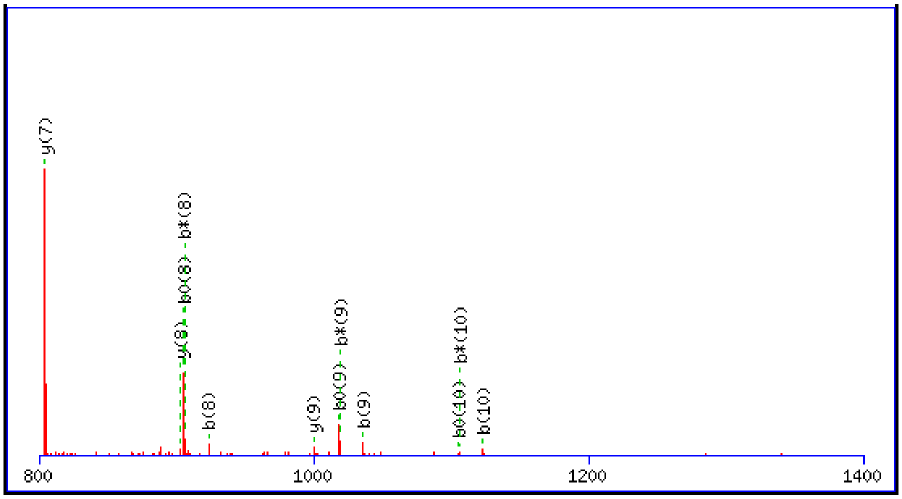

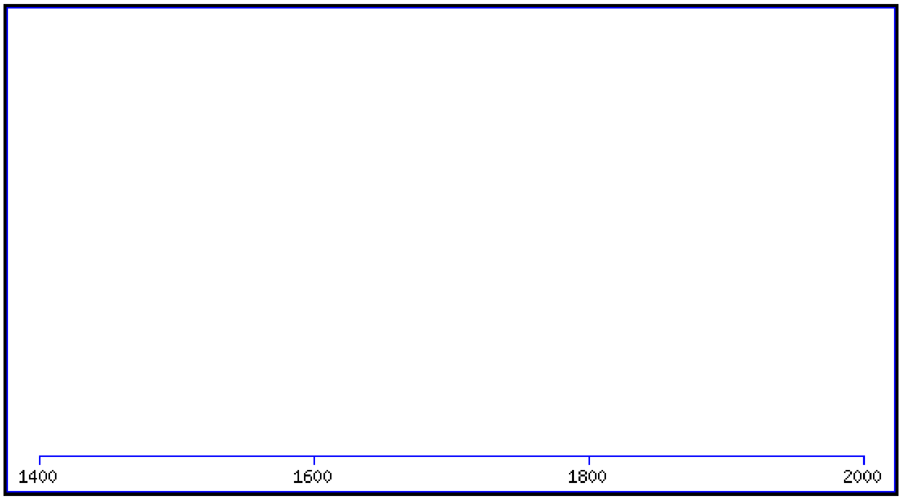

Monoisotopic mass of neutral peptide Mr(calc): 1725.9261
Fixed modifications: Carbamidomethyl (C)
Ions Score: 68 **Expect:** 1.4e-005
Matches (Bold Red): 51/156 fragment ions using 52 most intense peaks

#	b	b^{++}	b*	b^{*++}	b^0	b^{0++}	Seq.	y	y^{++}	y*	y^{*++}	y^0	y^{0++}	#
1	130.0499	65.5286			112.0393	56.5233	E							15
2	**229.1183**	115.0628			**211.1077**	106.0575	V	1597.8908	799.4490	1580.8642	790.9358	1579.8802	790.4438	14
3	342.2023	171.6048			**324.1918**	162.5995	L	1498.8224	**749.9148**	1481.7958	**741.4016**	1480.8118	**740.9095**	13
4	471.2449	236.1261			**453.2344**	227.1208	E	1385.7383	**693.3728**	1368.7118	684.8595	1367.7278	684.3675	12
5	599.3399	300.1736	582.3134	291.6603	581.3293	291.1683	K	1256.6957	**628.8515**	1239.6692	620.3382	1238.6852	619.8462	11
6	727.3985	364.2029	710.3719	**355.6896**	**709.3879**	**355.1976**	Q	1128.6008	**564.8040**	1111.5742	556.2907	1110.5902	555.7987	10
7	824.4512	412.7293	807.4247	404.2160	806.4407	403.7240	P	**1000.5422**	**500.7747**	983.5156	492.2615	982.5316	491.7694	9
8	**923.5197**	**462.2635**	**906.4931**	**453.7502**	**905.5091**	**453.2582**	V	**903.4894**	452.2483	886.4629	443.7351	885.4789	443.2431	8
9	**1036.6037**	518.8055	**1019.5772**	**510.2922**	**1018.5932**	**509.8002**	L	**804.4210**	**402.7141**	**787.3945**	394.2009	**786.4104**	393.7089	7
10	**1123.6358**	562.3215	**1106.6092**	553.8082	**1105.6252**	553.3162	S	**691.3369**	**346.1721**	674.3104	**337.6588**	673.3264	**337.1668**	6
11	1251.6943	626.3508	1234.6678	**617.8375**	1233.6838	**617.3455**	Q	**604.3049**	302.6561	587.2784	**294.1428**	586.2944	**293.6508**	5
12	1352.7420	676.8746	1335.7155	**668.3614**	1334.7314	**667.8694**	T	**476.2463**	238.6268	459.2198	230.1135	458.2358	**229.6215**	4
13	1481.7846	**741.3959**	1464.7581	732.8827	1463.7740	732.3907	E	**375.1987**	188.1030	358.1721	179.5897	357.1881	179.0977	3
14	1552.8217	776.9145	1535.7952	768.4012	1534.8112	767.9092	A	**246.1561**	123.5817	**229.1295**	115.0684			2
15							R	175.1190	88.0631	158.0924	79.5498			1

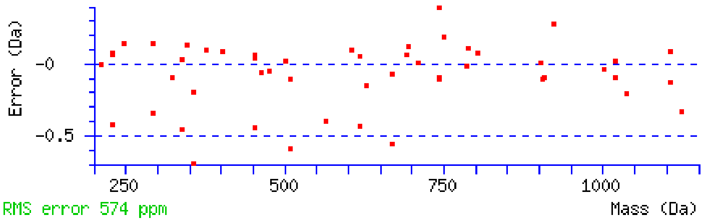

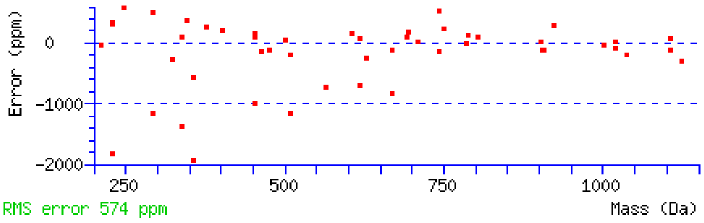

All matches to this query

Score	Mr(calc):	Delta	Sequence
68.4	1725.9261	0.0042	EVLEKQPVLSQTEAR
7.7	1723.9078	2.0224	VYKSLLLLAYLIR
6.6	1723.9297	2.0005	IIVFFSLPNIGDFSR
5.2	1723.9208	2.0095	MFLRAGLAALSPLLR
5.1	1724.9256	1.0047	SWAMSKLAAAHRGAIR
4.6	1724.9260	1.0043	KLVGTKVLMASVQGSK
4.4	1723.9134	2.0169	NLIQKAGYLNLRNK
4.2	1724.9226	1.0077	KNKDIVTVANAVFVK
4.2	1725.9195	0.0108	TLTKVLALVKEVPR
4.1	1725.9162	0.0141	RGLYLSDIPLHDATR

Spectrum No: 46; Query: 26990; Rank: 1

Peptide View

MS/MS Fragmentation of **EVLEKQPVLSQTEARDLVER**
Found in **IPI00555956**, Tax_Id=9606 Gene_Symbol=PSMB4 Proteasome subunit beta type-4

Match to Query 26990: 2338.269416 from(585.574630,4+)
Title: 090324LimSK_Exosome1_B.7520.7520.4.dta
Data file C:\Temp\Mascoti\LSK_2D\090324LimSK_Exosome1_A_2D.mgf

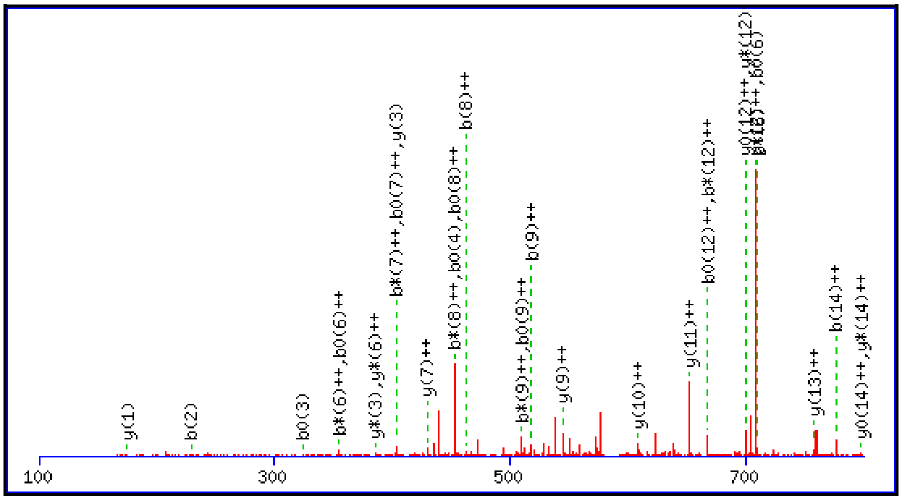

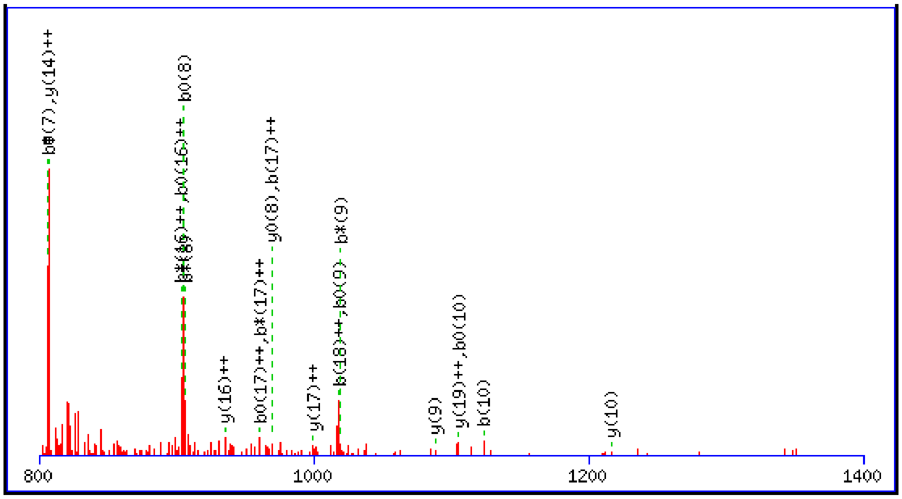

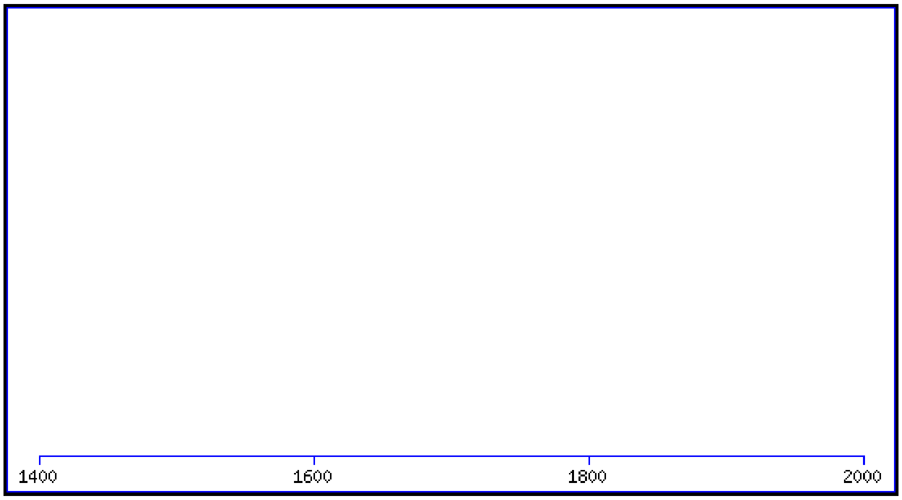

Monoisotopic mass of neutral peptide Mr(calc): 2338.2492
Fixed modifications: Carbamidomethyl (C)
Ions Score: 22 **Expect:** 0.44
Matches (Bold Red): 53/218 fragment ions using 95 most intense peaks

#	b	b^{++}	b*	b^{*++}	b^0	b^{0++}	Seq.	y	y^{++}	y*	y^{*++}	y^0	y^{0++}	#
1	130.0499	65.5286			112.0393	56.5233	E							20
2	**229.1183**	115.0628			211.1077	106.0575	V	2210.2139	**1105.6106**	2193.1874	1097.0973	2192.2034	1096.6053	19
3	342.2023	171.6048			**324.1918**	162.5995	L	2111.1455	1056.0764	2094.1190	1047.5631	2093.1349	1047.0711	18
4	471.2449	236.1261			**453.2344**	227.1208	E	1998.0614	**999.5344**	1981.0349	991.0211	1980.0509	990.5291	17
5	599.3399	300.1736	582.3134	291.6603	581.3293	291.1683	K	1869.0189	**935.0131**	1851.9923	926.4998	1851.0083	926.0078	16
6	727.3985	364.2029	**710.3719**	**355.6896**	**709.3879**	**355.1976**	Q	1740.9239	870.9656	1723.8973	862.4523	1722.9133	861.9603	15
7	824.4512	412.7293	**807.4247**	**404.2160**	**806.4407**	**403.7240**	P	1612.8653	**806.9363**	1595.8388	**798.4230**	1594.8547	**797.9310**	14
8	923.5197	**462.2635**	**906.4931**	**453.7502**	**905.5091**	**453.2582**	V	1515.8125	**758.4099**	1498.7860	749.8966	1497.8020	749.4046	13
9	1036.6037	**518.8055**	**1019.5772**	**510.2922**	**1018.5932**	**509.8002**	L	1416.7441	**708.8757**	1399.7176	**700.3624**	1398.7336	**699.8704**	12
10	**1123.6358**	562.3215	1106.6092	553.8082	**1105.6252**	553.3162	S	1303.6601	**652.3337**	1286.6335	643.8204	1285.6495	643.3284	11
11	1251.6943	626.3508	1234.6678	617.8375	1233.6838	617.3455	Q	**1216.6280**	**608.8177**	1199.6015	600.3044	1198.6175	599.8124	10
12	1352.7420	676.8746	1335.7155	**668.3614**	1334.7314	**667.8694**	T	**1088.5695**	**544.7884**	1071.5429	536.2751	1070.5589	535.7831	9
13	1481.7846	741.3959	1464.7581	732.8827	1463.7740	732.3907	E	987.5218	494.2645	970.4952	485.7513	**969.5112**	485.2592	8
14	1552.8217	**776.9145**	1535.7952	768.4012	1534.8112	767.9092	A	858.4792	**429.7432**	841.4526	421.2300	840.4686	420.7380	7
15	1708.9228	854.9651	1691.8963	846.4518	1690.9123	845.9598	R	787.4421	394.2247	770.4155	**385.7114**	769.4315	385.2194	6
16	1823.9498	912.4785	1806.9232	**903.9652**	1805.9392	**903.4732**	D	631.3410	316.1741	614.3144	307.6608	613.3304	307.1688	5
17	1937.0338	**969.0206**	1920.0073	**960.5073**	1919.0233	**960.0153**	L	516.3140	258.6606	499.2875	250.1474	498.3035	249.6554	4
18	2036.1022	**1018.5548**	2019.0757	1010.0415	2018.0917	1009.5495	V	**403.2300**	202.1186	**386.2034**	193.6053	385.2194	193.1133	3
19	2165.1448	1083.0761	2148.1183	1074.5628	2147.1343	1074.0708	E	304.1615	152.5844	287.1350	144.0711	286.1510	143.5791	2

20							R	175.1190	88.0631	158.0924	79.5498			1

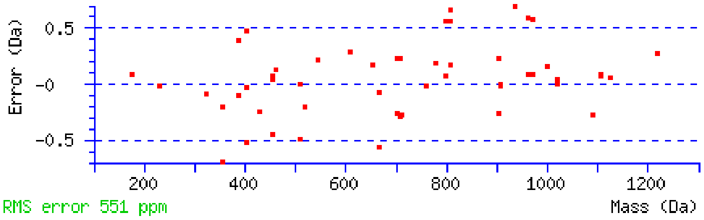

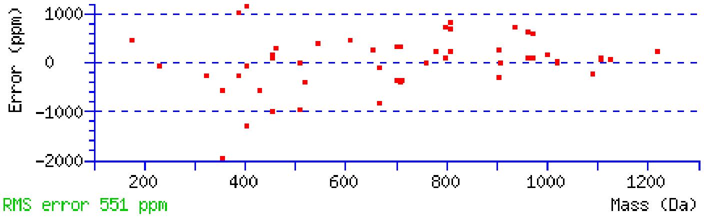

All matches to this query

Score	Mr(calc):	Delta	Sequence
22.0	2338.2492	0.0202	EVLEKQPVLSQTEARDLVER
9.6	2336.2731	1.9963	FLTLCTWLLLLGPGLLATVR
8.8	2337.2614	1.0080	VTMENTFVNSLASQIIKTLTK
4.7	2336.2614	2.0080	XFVHHLRNALDVLHREVPR
4.7	2338.2593	0.0101	XFVHHLRNALDVLHREVPR
4.0	2336.2457	2.0237	LPEIQQPLCRKEGLCQIVR
3.9	2337.2441	1.0254	LPNLSSPSAEGPPGPPSGPAPRKK
1.5	2336.2538	2.0156	XVRVLIQSGPLKISSVSEVMK
1.4	2337.2481	1.0213	YFSGPKPKAVLNQFRTDLEK
1.2	2338.2654	0.0040	KNSVVQGMILMFAGGKLIFGGR

Spectrum No: 47; Query: 16773; Rank: 1

Peptide View

MS/MS Fragmentation of **QPVLSQTEARDLVER**
Found in **IPI00555956**, Tax_Id=9606 Gene_Symbol=PSMB4 Proteasome subunit beta type-4

Match to Query 16773: 1739.925402 from(580.982410,3+)
Title: 090324LimSK_Exosome1_A.6390.6390.3.dta
Data file C:\Temp\Mascoti\LSK_2D\090324LimSK_Exosome1_A_2D.mgf

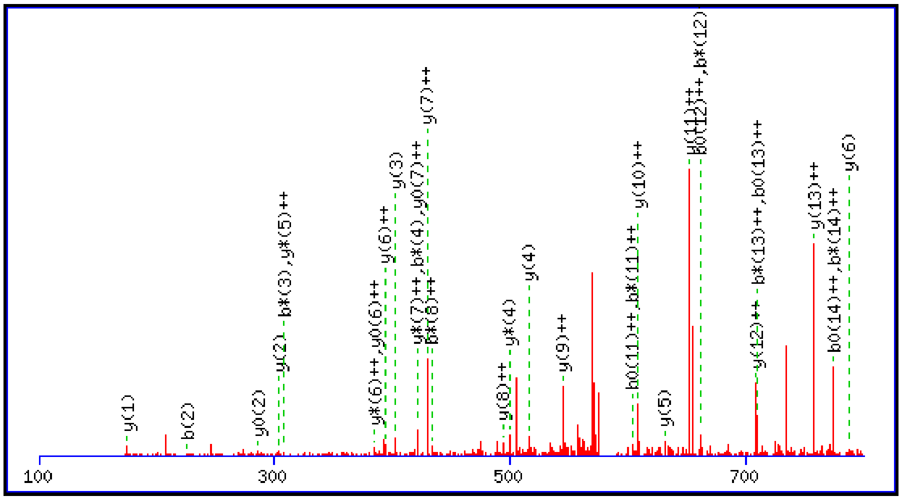

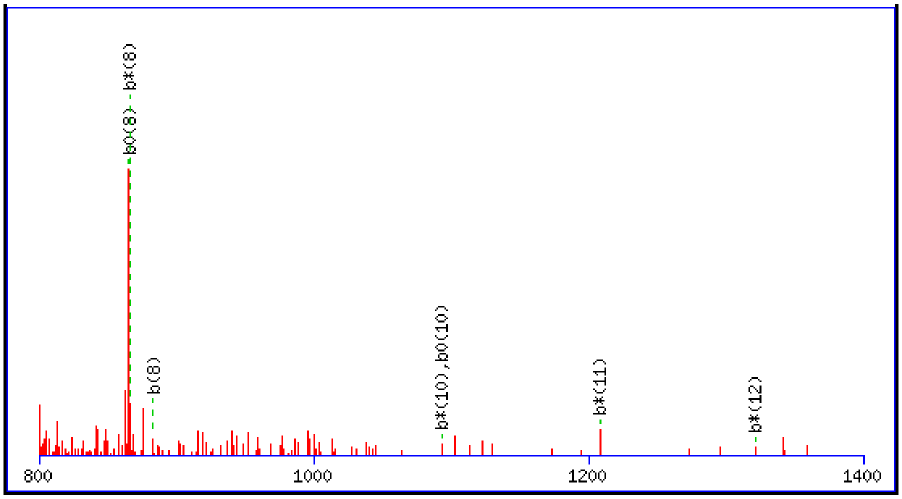

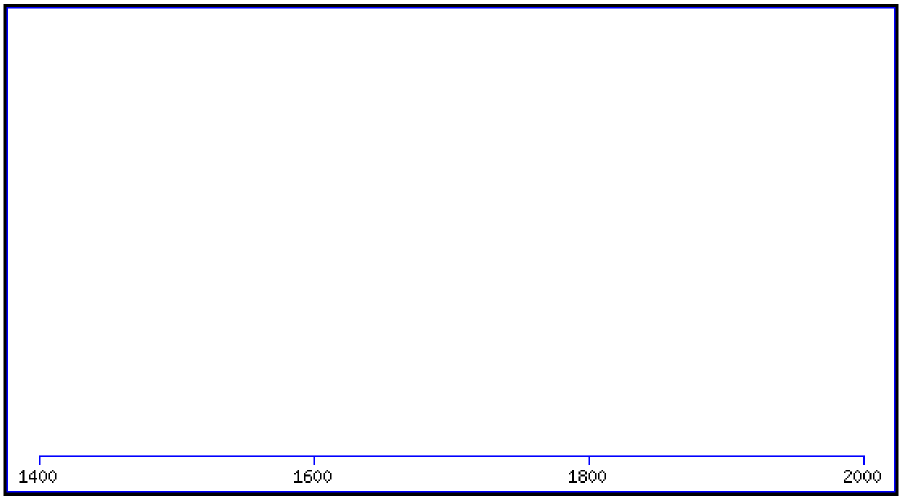

Monoisotopic mass of neutral peptide Mr(calc): 1739.9166
Fixed modifications: Carbamidomethyl (C)
Ions Score: 22 **Expect:** 0.68
Matches (Bold Red): 41/158 fragment ions using 90 most intense peaks

#	b	b^{++}	b*	b^{*++}	b^0	b^{0++}	Seq.	y	y^{++}	y*	y^{*++}	y^0	y^{0++}	#
1	129.0659	65.0366	112.0393	56.5233			Q							15
2	**226.1186**	113.5629	209.0921	105.0497			P	1612.8653	806.9363	1595.8388	798.4230	1594.8547	797.9310	14
3	325.1870	163.0972	**308.1605**	154.5839			V	1515.8125	**758.4099**	1498.7860	749.8966	1497.8020	749.4046	13
4	438.2711	219.6392	**421.2445**	211.1259			L	1416.7441	**708.8757**	1399.7176	700.3624	1398.7336	699.8704	12
5	525.3031	263.1552	508.2766	254.6419	507.2926	254.1499	S	1303.6601	**652.3337**	1286.6335	643.8204	1285.6495	643.3284	11
6	653.3617	327.1845	636.3352	318.6712	635.3511	318.1792	Q	1216.6280	**608.8177**	1199.6015	600.3044	1198.6175	599.8124	10
7	754.4094	377.7083	737.3828	369.1951	736.3988	368.7030	T	1088.5695	**544.7884**	1071.5429	536.2751	1070.5589	535.7831	9
8	**883.4520**	442.2296	**866.4254**	**433.7164**	**865.4414**	433.2243	E	987.5218	**494.2645**	970.4952	485.7513	969.5112	485.2592	8
9	954.4891	477.7482	937.4625	469.2349	936.4785	468.7429	A	858.4792	**429.7432**	841.4526	**421.2300**	840.4686	**420.7380**	7
10	1110.5902	555.7987	**1093.5637**	547.2855	**1092.5796**	546.7935	R	**787.4421**	**394.2247**	770.4155	**385.7114**	769.4315	**385.2194**	6
11	1225.6171	613.3122	**1208.5906**	**604.7989**	1207.6066	**604.3069**	D	**631.3410**	316.1741	614.3144	**307.6608**	613.3304	307.1688	5
12	1338.7012	669.8542	**1321.6747**	**661.3410**	1320.6906	**660.8490**	L	**516.3140**	258.6606	**499.2875**	250.1474	498.3035	249.6554	4
13	1437.7696	719.3884	1420.7431	**710.8752**	1419.7591	**710.3832**	V	**403.2300**	202.1186	386.2034	193.6053	**385.2194**	193.1133	3
14	1566.8122	783.9097	1549.7857	**775.3965**	1548.8016	**774.9045**	E	**304.1615**	152.5844	287.1350	144.0711	**286.1510**	143.5791	2
15							R	**175.1190**	88.0631	158.0924	79.5498			1

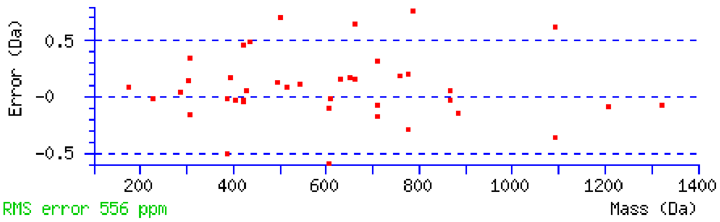

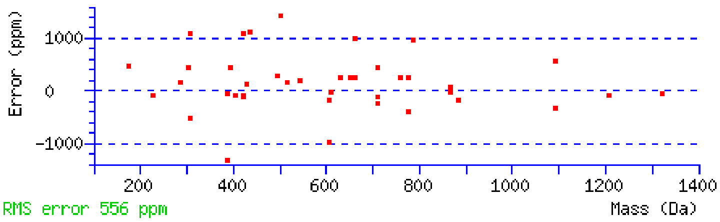

All matches to this query

Score	Mr(calc):	Delta	Sequence
22.5	1739.9166	0.0088	QPVLSQTEARDLVER
7.5	1737.9080	2.0174	XFIGFATTIHQIVR
4.5	1738.9148	1.0106	SSRLCVPAPLSTAPGAR
4.0	1737.9097	2.0157	LMTNLRTTHPHFVR
3.7	1738.9261	0.9993	QGPQRASQLGLGMAAVR
3.4	1739.9409	-0.0155	LLLKDKMPGTFLLR
3.3	1738.9366	0.9888	ALFLATERETYAAKR
2.9	1738.9165	1.0089	MVLADLGRKITSALR
2.8	1739.9256	-0.0002	TGISMSLLTVIEKLR
2.8	1739.9256	-0.0002	TGISMSLLTVIEKLR

Spectrum No: 48; Query: 34245; Rank: 1

Peptide View

MS/MS Fragmentation of **IYKLNEDMACSVAGITSDANVLTNELR**
Found in **IPI00299155**, Tax_Id=9606 Gene_Symbol=PSMA4 Proteasome subunit alpha type-4

Match to Query 34245: 2998.477152 from(1000.499660,3+)
Title: 090324LimSK_Exosome1_A.17902.17902.3.dta
Data file C:\Temp\Mascoti\LSK_2D\090324LimSK_Exosome1_A_2D.mgf

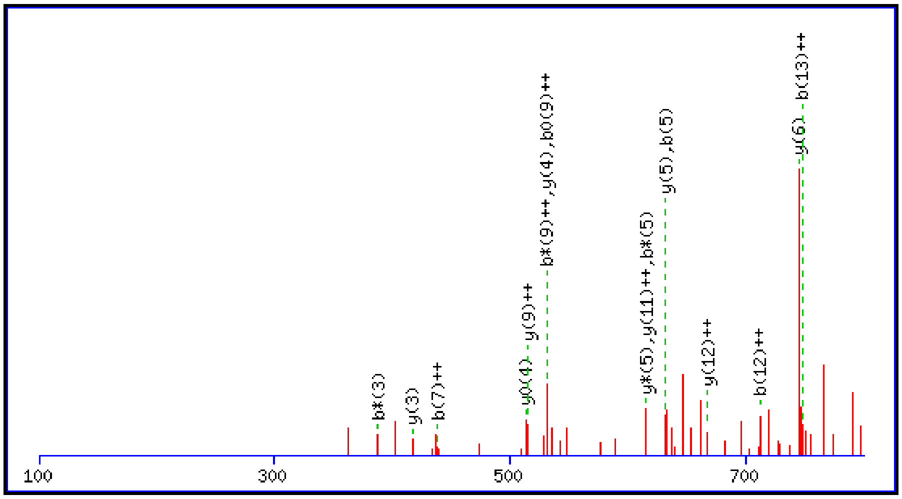

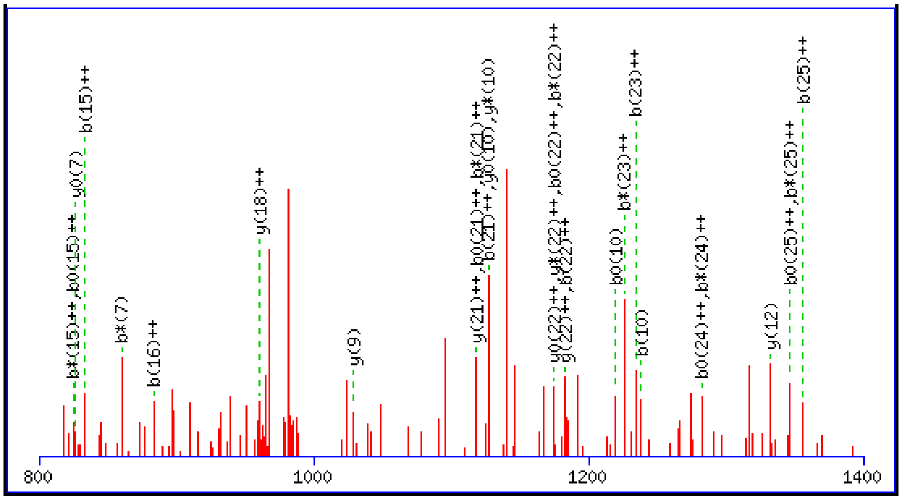

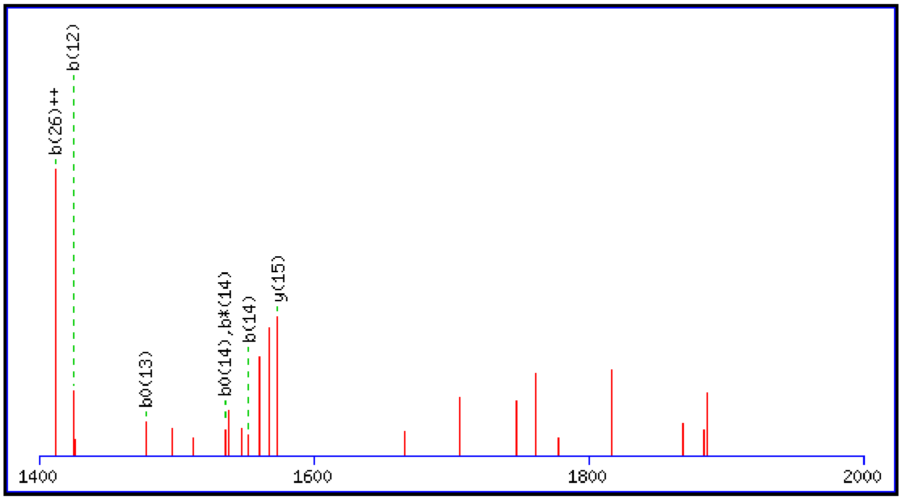

Monoisotopic mass of neutral peptide Mr(calc): 2996.4583
Fixed modifications: Carbamidomethyl (C)
Ions Score: 24 **Expect:** 0.93
Matches (Bold Red): 54/294 fragment ions using 109 most intense peaks

#	b	b^{++}	b*	b^{*++}	b^0	b^{0++}	Seq.	y	y^{++}	y*	y^{*++}	y^0	y^{0++}	#
1	114.0913	57.5493					I							27
2	277.1547	139.0810					Y	2884.3815	1442.6944	2867.3550	1434.1811	2866.3710	1433.6891	26
3	405.2496	203.1285	**388.2231**	194.6152			K	2721.3182	1361.1627	2704.2917	1352.6495	2703.3076	1352.1575	25
4	518.3337	259.6705	501.3071	251.1572			L	2593.2232	1297.1153	2576.1967	1288.6020	2575.2127	1288.1100	24
5	**632.3766**	316.6919	**615.3501**	308.1787			N	2480.1392	1240.5732	2463.1126	1232.0600	2462.1286	1231.5679	23
6	761.4192	381.2132	744.3927	372.7000	743.4087	372.2080	E	2366.0963	**1183.5518**	2349.0697	**1175.0385**	2348.0857	**1174.5465**	22
7	876.4462	**438.7267**	**859.4196**	430.2134	858.4356	429.7214	D	2237.0537	**1119.0305**	2220.0271	1110.5172	2219.0431	1110.0252	21
8	1007.4866	504.2470	990.4601	495.7337	989.4761	495.2417	M	2122.0267	1061.5170	2105.0002	1053.0037	2104.0162	1052.5117	20
9	1078.5238	539.7655	1061.4972	**531.2522**	1060.5132	**530.7602**	A	1990.9862	995.9968	1973.9597	987.4835	1972.9757	986.9915	19
10	**1238.5544**	619.7808	1221.5279	611.2676	**1220.5438**	610.7756	C	1919.9491	**960.4782**	1902.9226	951.9649	1901.9386	951.4729	18
11	1325.5864	663.2969	1308.5599	654.7836	1307.5759	654.2916	S	1759.9185	880.4629	1742.8919	871.9496	1741.9079	871.4576	17
12	**1424.6548**	**712.8311**	1407.6283	704.3178	1406.6443	703.8258	V	1672.8864	836.9469	1655.8599	828.4336	1654.8759	827.9416	16
13	1495.6920	**748.3496**	1478.6654	739.8363	**1477.6814**	739.3443	A	**1573.8180**	787.4127	1556.7915	778.8994	1555.8075	778.4074	15
14	**1552.7134**	776.8604	**1535.6869**	768.3471	**1534.7029**	767.8551	G	1502.7809	751.8941	1485.7544	743.3808	1484.7703	742.8888	14
15	1665.7975	**833.4024**	1648.7709	**824.8891**	1647.7869	**824.3971**	I	1445.7594	723.3834	1428.7329	714.8701	1427.7489	714.3781	13
16	1766.8452	**883.9262**	1749.8186	875.4129	1748.8346	874.9209	T	**1332.6754**	**666.8413**	1315.6488	658.3281	1314.6648	657.8360	12
17	1853.8772	927.4422	1836.8506	918.9290	1835.8666	918.4370	S	1231.6277	**616.3175**	1214.6012	607.8042	1213.6171	607.3122	11
18	1968.9041	984.9557	1951.8776	976.4424	1950.8936	975.9504	D	1144.5957	572.8015	**1127.5691**	564.2882	**1126.5851**	563.7962	10
19	2039.9413	1020.4743	2022.9147	1011.9610	2021.9307	1011.4690	A	**1029.5687**	**515.2880**	1012.5422	506.7747	1011.5582	506.2827	9

**20**	2153.9842	1077.4957	2136.9576	1068.9825	2135.9736	1068.4904	**N**	958.5316	479.7694	941.5051	471.2562	940.5211	470.7642	**8**
**21**	2253.0526	**1127.0299**	2236.0260	**1118.5167**	2235.0420	**1118.0247**	**V**	844.4887	422.7480	827.4621	414.2347	**826.4781**	413.7427	**7**
**22**	2366.1367	**1183.5720**	2349.1101	**1175.0587**	2348.1261	**1174.5667**	**L**	**745.4203**	373.2138	728.3937	364.7005	727.4097	364.2085	**6**
**23**	2467.1843	**1234.0958**	2450.1578	**1225.5825**	2449.1738	1225.0905	**T**	**632.3362**	316.6717	**615.3097**	308.1585	614.3257	307.6665	**5**
**24**	2581.2273	1291.1173	2564.2007	**1282.6040**	2563.2167	**1282.1120**	**N**	**531.2885**	266.1479	514.2620	257.6346	**513.2780**	257.1426	**4**
**25**	2710.2699	**1355.6386**	2693.2433	**1347.1253**	2692.2593	**1346.6333**	**E**	**417.2456**	209.1264	400.2191	200.6132	399.2350	200.1212	**3**
**26**	2823.3539	**1412.1806**	2806.3274	1403.6673	2805.3434	1403.1753	**L**	288.2030	144.6051	271.1765	136.0919			**2**
**27**							**R**	175.1190	88.0631	158.0924	79.5498			**1**

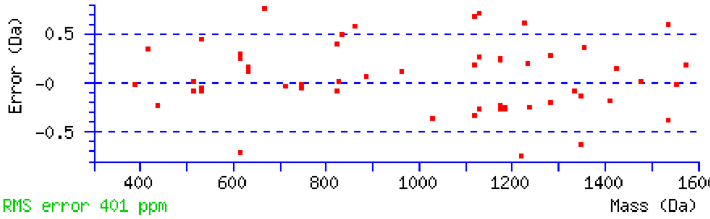

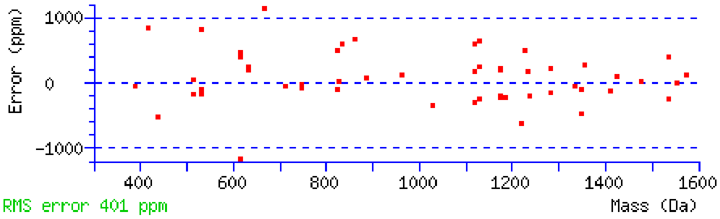

All matches to this query

Score	Mr(calc):	Delta	Sequence
24.0	2996.4583	2.0189	IYKLNEDMACSVAGITSDANVLTNELR
7.8	2997.4527	1.0245	MLFRTVLHLLSVDVSTAEMMPENLR
7.8	2997.4527	1.0245	MLFRTVLHLLSVDVSTAEMMPENLR
7.8	2997.4527	1.0245	MLFRTVLHLLSVDVSTAEMMPENLR
7.8	2997.4527	1.0245	MLFRTVLHLLSVDVSTAEMMPENLR
7.4	2997.4977	0.9794	TPFKSPLLKGISAPVISSNPATTEVQR
6.9	2997.4977	0.9794	TPFKSPLLKGISAPVISSNPATTEVQR
5.3	2997.4946	0.9826	TSPMTNLVLPSAVLTPNRTLKMAINR
5.3	2998.4998	-0.0227	SDGEMVLPGFPDADSFVKFALGSVVAVTK
5.2	2997.4467	1.0305	HHLVQHMVSAMQRLGFTPSVTIEQR

Spectrum No: 49; Query: 9479; Rank: 1

Peptide View

MS/MS Fragmentation of **LLDEVFFSEK**
Found in **IPI00299155**, Tax_Id=9606 Gene_Symbol=PSMA4 Proteasome subunit alpha type-4

Match to Query 9479: 1225.629028 from(613.821790,2+)
Title: 090324LimSK_Exosome1_B.11581.11581.2.dta
Data file C:\Temp\Mascoti\LSK_2D\090324LimSK_Exosome1_A_2D.mgf

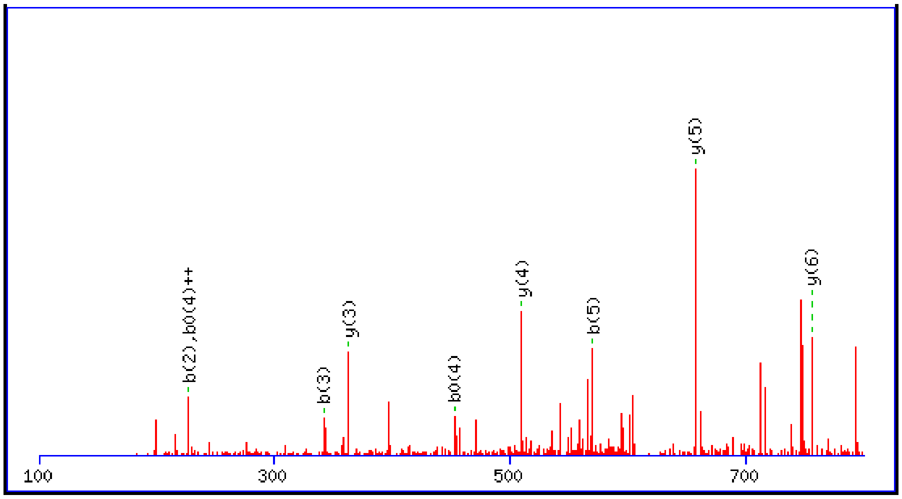

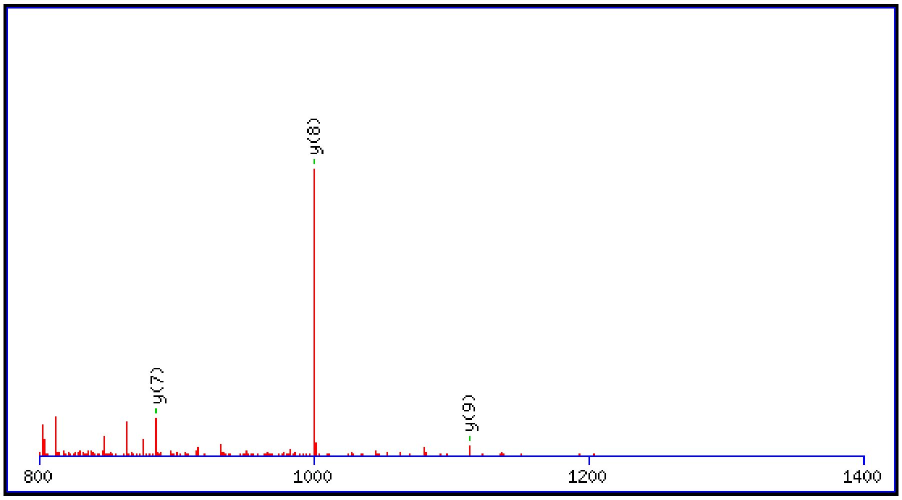

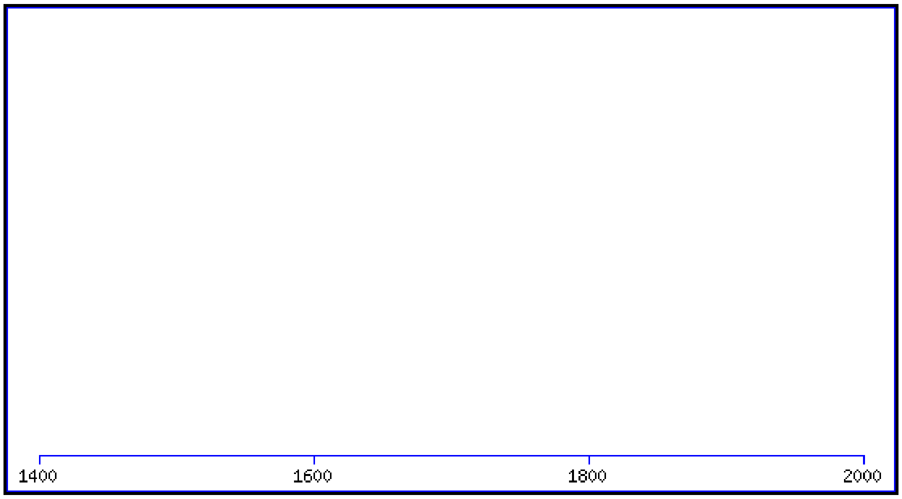

Monoisotopic mass of neutral peptide Mr(calc): 1225.6230
Fixed modifications: Carbamidomethyl (C)
Ions Score: 43 **Expect:** 0.0065
Matches (Bold Red): 12/84 fragment ions using 25 most intense peaks

#	b	b^{++}	b^0	b^{0++}	Seq.	y	y^{++}	y*	y^{*++}	y^0	y^{0++}	#
1	114.0913	57.5493			L							10
2	**227.1754**	114.0913			L	**1113.5463**	557.2768	1096.5197	548.7635	1095.5357	548.2715	9
3	**342.2023**	171.6048	324.1918	162.5995	D	**1000.4622**	500.7347	983.4357	492.2215	982.4516	491.7295	8
4	471.2449	236.1261	**453.2344**	**227.1208**	E	**885.4353**	443.2213	868.4087	434.7080	867.4247	434.2160	7
5	**570.3134**	285.6603	552.3028	276.6550	V	**756.3927**	378.7000	739.3661	370.1867	738.3821	369.6947	6
6	717.3818	359.1945	699.3712	350.1892	F	**657.3243**	329.1658	640.2977	320.6525	639.3137	320.1605	5

**7**	864.4502	432.7287	846.4396	423.7234	**F**	**510.2558**	255.6316	493.2293	247.1183	492.2453	246.6263	**4**
**8**	951.4822	476.2447	933.4716	467.2395	**S**	**363.1874**	182.0974	346.1609	173.5841	345.1769	173.0921	**3**
**9**	1080.5248	540.7660	1062.5142	531.7608	**E**	276.1554	138.5813	259.1288	130.0681	258.1448	129.5761	**2**
**10**					**K**	147.1128	74.0600	130.0863	65.5468			**1**

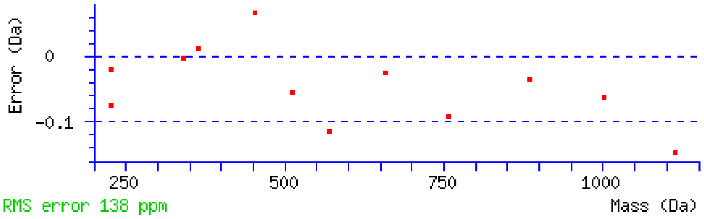

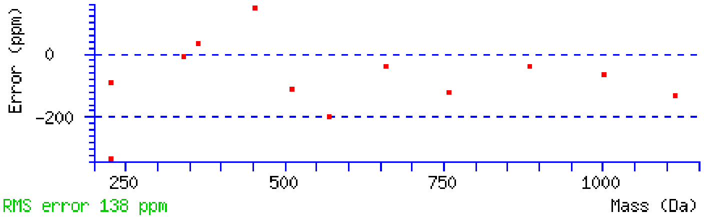

All matches to this query

Score	Mr(calc):	Delta	Sequence
43.5	1225.6230	0.0060	LLDEVFFSEK
3.9	1225.6220	0.0070	ILVAHDIHTK
3.0	1225.6349	-0.0059	LIHDTNMRAR
0.5	1225.6254	0.0037	KPVMKGAKSGK

Spectrum No: 50; Query: 11752; Rank: 1

Peptide View

MS/MS Fragmentation of **LSAEKVEIATLTR**
Found in **IPI00299155**, Tax_Id=9606 Gene_Symbol=PSMA4 Proteasome subunit alpha type-4

Match to Query 11752: 1429.821492 from(477.614440,3+)
Title: 090324LimSK_Exosome1_B.5418.5418.3.dta
Data file C:\Temp\Mascoti\LSK_2D\090324LimSK_Exosome1_A_2D.mgf

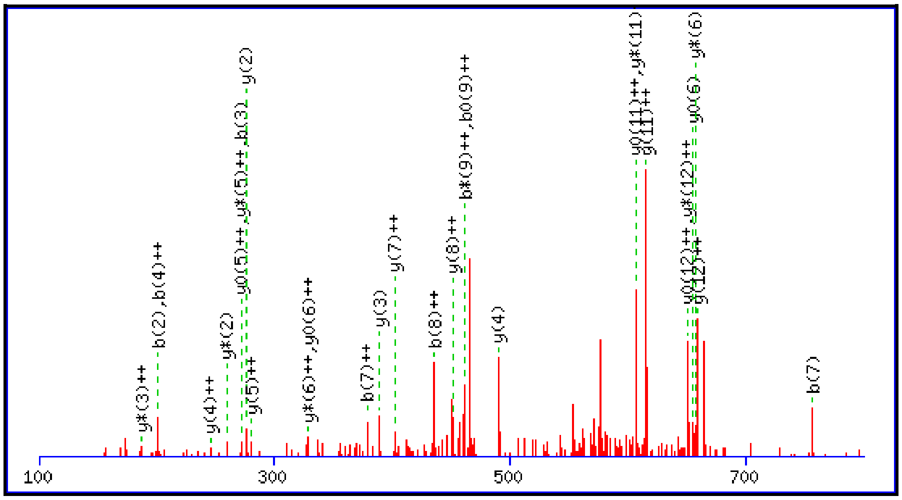

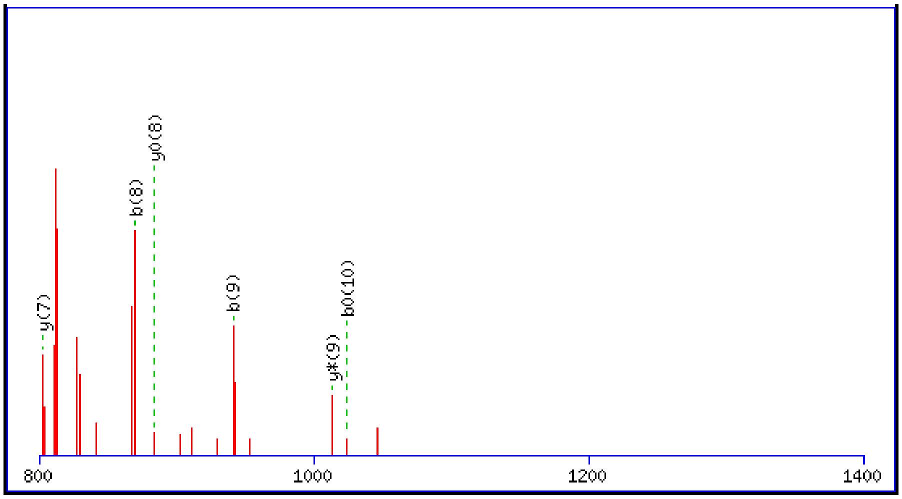

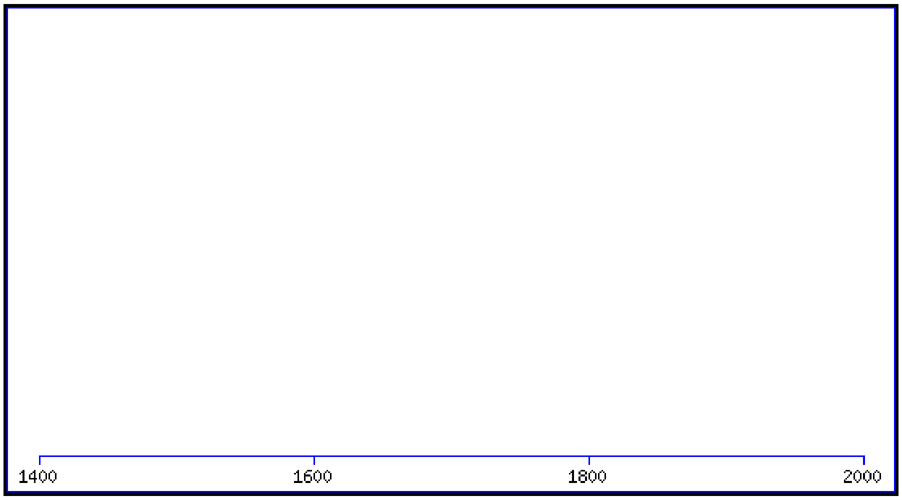

Monoisotopic mass of neutral peptide Mr(calc): 1429.8140
Fixed modifications: Carbamidomethyl (C)
Ions Score: 35 **Expect:** 0.02
Matches (Bold Red): 35/132 fragment ions using 52 most intense peaks

#	b	b^{++}	b*	b^{*++}	b^{0}	b^{0++}	Seq.	y	y^{++}	y*	y^{*++}	y^{0}	y^{0++}	#
1	114.0913	57.5493					L							13
2	**201.1234**	101.0653			183.1128	92.0600	S	1317.7373	**659.3723**	1300.7107	**650.8590**	1299.7267	**650.3670**	12
3	**272.1605**	136.5839			254.1499	127.5786	A	1230.7052	**615.8563**	1213.6787	**607.3430**	1212.6947	**606.8510**	11
4	401.2031	**201.1052**			383.1925	192.0999	E	1159.6681	580.3377	1142.6416	571.8244	1141.6575	571.3324	10
5	529.2980	265.1527	512.2715	256.6394	511.2875	256.1474	K	1030.6255	515.8164	**1013.5990**	507.3031	1012.6150	506.8111	9
6	628.3665	314.6869	611.3399	306.1736	610.3559	305.6816	V	902.5306	**451.7689**	885.5040	443.2556	**884.5200**	442.7636	8
7	**757.4090**	**379.2082**	740.3825	370.6949	739.3985	370.2029	E	**803.4621**	**402.2347**	786.4356	393.7214	785.4516	393.2294	7
8	**870.4931**	**435.7502**	853.4666	427.2369	852.4825	426.7449	I	674.4196	337.7134	**657.3930**	**329.2001**	**656.4090**	**328.7081**	6
9	**941.5302**	471.2687	924.5037	**462.7555**	923.5197	**462.2635**	A	561.3355	**281.1714**	544.3089	**272.6581**	543.3249	**272.1661**	5
10	1042.5779	521.7926	1025.5514	513.2793	**1024.5673**	512.7873	T	**490.2984**	**245.6528**	473.2718	237.1396	472.2878	236.6475	4
11	1155.6620	578.3346	1138.6354	569.8213	1137.6514	569.3293	L	**389.2507**	195.1290	372.2241	**186.6157**	371.2401	186.1237	3
12	1256.7096	628.8585	1239.6831	620.3452	1238.6991	619.8532	T	**276.1666**	138.5870	**259.1401**	130.0737	258.1561	129.5817	2
13							R	175.1190	88.0631	158.0924	79.5498			1

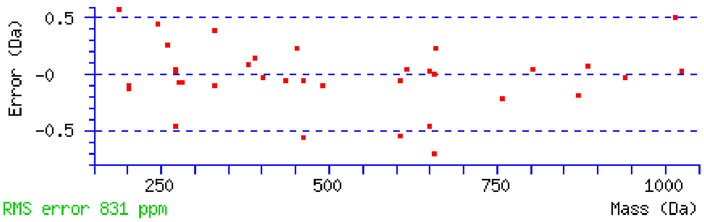

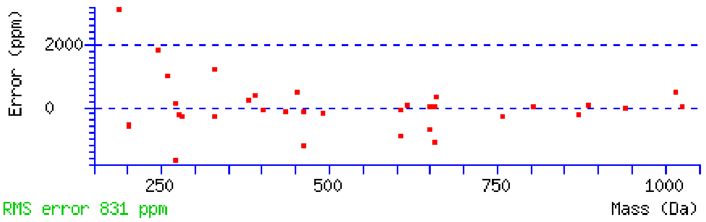

All matches to this query

Score	Mr(calc):	Delta	Sequence
34.9	1429.8140	0.0075	LSAEKVEIATLTR
8.2	1428.8235	0.9980	SLKMAQRQLLNK
4.9	1428.8300	0.9915	LSGVLTVKAGDTIR
3.5	1428.8201	1.0014	YRQILQKVEPR
2.5	1427.8096	2.0119	TDQRQQLLVSLK
2.0	1429.8154	0.0061	IFRSNGVQLLQR
1.6	1429.8187	0.0028	LKVCALGVTSRAR
1.1	1428.8235	0.9980	XAMLATLARVAALR
0.1	1429.8187	0.0028	ALLSLRAVREMR

Spectrum No: 51; Query: 31107; Rank: 1

Peptide View

MS/MS Fragmentation of **YLLQYQEPIPCEQLVTALCDIK**
Found in **IPI00299155**, Tax_Id=9606 Gene_Symbol=PSMA4 Proteasome subunit alpha type-4

Match to Query 31107: 2693.354502 from(898.792110,3+)
Title: 090324LimSK_Exosome1_A.18247.18247.3.dta
Data file C:\Temp\Mascoti\LSK_2D\090324LimSK_Exosome1_A_2D.mgf

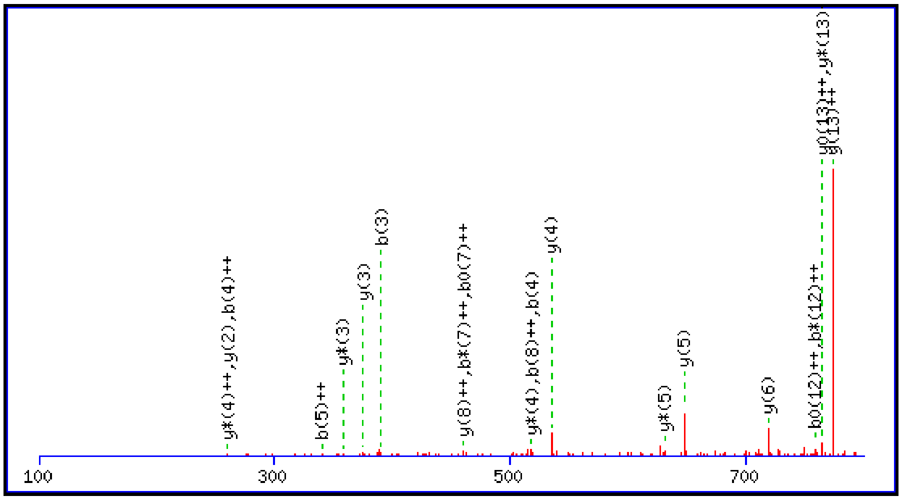

Mascot Search Results: Peptide View

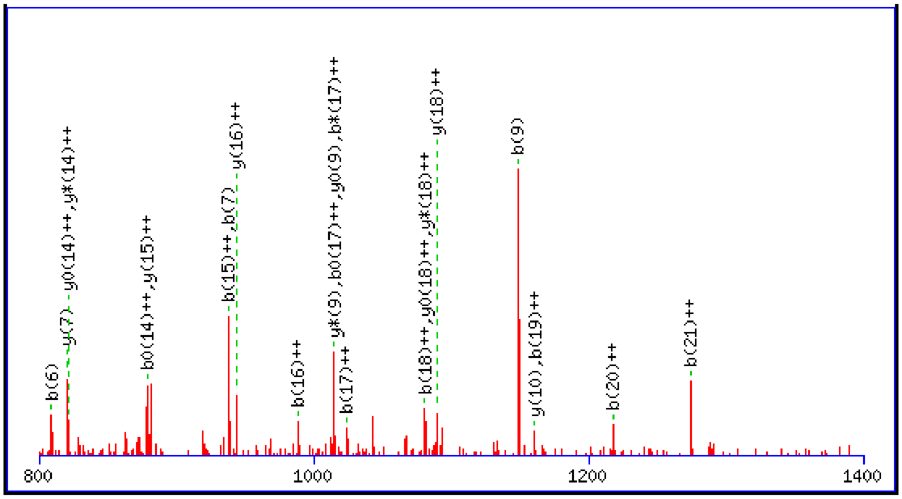

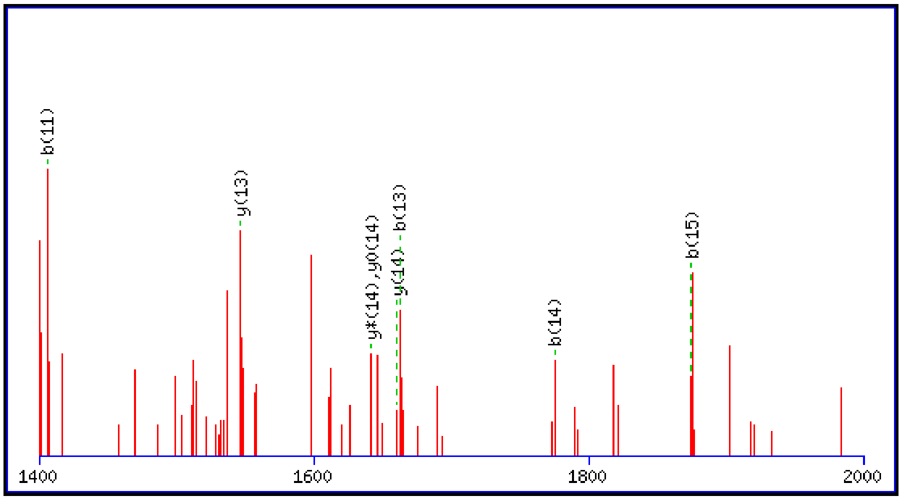

Monoisotopic mass of neutral peptide Mr(calc): 2693.3444
Fixed modifications: Carbamidomethyl (C)
Ions Score: 48 **Expect:** 0.0031
Matches (Bold Red): 54/230 fragment ions using 91 most intense peaks

#	b	b^{++}	b*	b^{*++}	b^0	b^{0++}	Seq.	y	y^{++}	y*	y^{*++}	y^0	y^{0++}	#
1	164.0706	82.5389					Y							22
2	277.1547	139.0810					L	2531.2884	1266.1478	2514.2619	1257.6346	2513.2778	1257.1426	21
3	**390.2387**	195.6230					L	2418.2043	1209.6058	2401.1778	1201.0925	2400.1938	1200.6005	20
4	**518.2973**	**259.6523**	501.2708	251.1390			Q	2305.1203	1153.0638	2288.0937	1144.5505	2287.1097	1144.0585	19
5	681.3606	**341.1840**	664.3341	332.6707			Y	2177.0617	**1089.0345**	2160.0352	**1080.5212**	2159.0511	**1080.0292**	18
6	**809.4192**	405.2132	792.3927	396.7000			Q	2013.9984	1007.5028	1996.9718	998.9896	1995.9878	998.4975	17
7	**938.4618**	469.7345	921.4353	**461.2213**	920.4512	**460.7293**	E	1885.9398	**943.4735**	1868.9133	934.9603	1867.9292	934.4683	16
8	1035.5146	**518.2609**	1018.4880	509.7477	1017.5040	509.2556	P	1756.8972	**878.9522**	1739.8707	870.4390	1738.8866	869.9470	15
9	**1148.5986**	574.8030	1131.5721	566.2897	1130.5881	565.7977	I	**1659.8444**	830.4259	**1642.8179**	**821.9126**	**1641.8339**	**821.4206**	14
10	1245.6514	623.3293	1228.6249	614.8161	1227.6408	614.3241	P	**1546.7604**	**773.8838**	1529.7338	**765.3706**	1528.7498	**764.8785**	13
11	**1405.6821**	703.3447	1388.6555	694.8314	1387.6715	694.3394	C	1449.7076	725.3574	1432.6811	716.8442	1431.6971	716.3522	12
12	1534.7246	767.8660	1517.6981	**759.3527**	1516.7141	**758.8607**	E	1289.6770	645.3421	1272.6504	636.8288	1271.6664	636.3368	11
13	**1662.7832**	831.8952	1645.7567	823.3820	1644.7727	822.8900	Q	**1160.6344**	580.8208	1143.6078	572.3076	1142.6238	571.8155	10
14	**1775.8673**	888.4373	1758.8407	879.9240	1757.8567	**879.4320**	L	1032.5758	516.7915	**1015.5492**	508.2783	**1014.5652**	507.7863	9
15	**1874.9357**	**937.9715**	1857.9092	929.4582	1856.9251	928.9662	V	919.4917	**460.2495**	902.4652	451.7362	901.4812	451.2442	8
16	1975.9834	**988.4953**	1958.9568	979.9821	1957.9728	979.4900	T	**820.4233**	410.7153	803.3968	402.2020	802.4128	401.7100	7
17	2047.0205	**1024.0139**	2029.9939	**1015.5006**	2029.0099	**1015.0086**	A	**719.3756**	360.1915	702.3491	351.6782	701.3651	351.1862	6
18	2160.1046	**1080.5559**	2143.0780	1072.0426	2142.0940	1071.5506	L	**648.3385**	324.6729	**631.3120**	316.1596	630.3280	315.6676	5
19	2320.1352	**1160.5712**	2303.1087	1152.0580	2302.1246	1151.5660	C	**535.2545**	268.1309	**518.2279**	**259.6176**	517.2439	259.1256	4

**20**	2435.1621	**1218.0847**	2418.1356	1209.5714	2417.1516	1209.0794	**D**	**375.2238**	188.1155	**358.1973**	179.6023	357.2132	179.1103	**3**
**21**	2548.2462	**1274.6267**	2531.2197	1266.1135	2530.2356	1265.6215	**I**	**260.1969**	130.6021	243.1703	122.0888			**2**
**22**							**K**	147.1128	74.0600	130.0863	65.5468			**1**

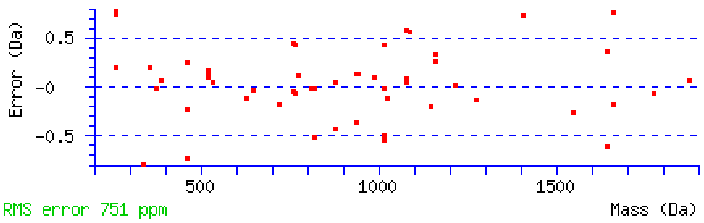

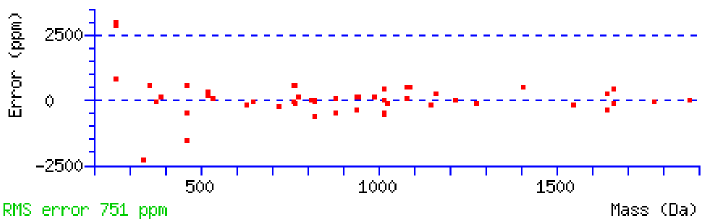

All matches to this query

Score	Mr(calc):	Delta	Sequence
47.9	2693.3444	0.0101	YLLQYQEPIPCEQLVTALCDIK
15.4	2692.3765	0.9780	TGVFLFTASSGGLPTDEITFAKLLK
15.4	2692.3765	0.9780	TGVFLFTASSGGLPTDEITFAKLLK
13.4	2692.3765	0.9780	TGVFLFTASSGGLPTDEITFAKLLK
13.3	2692.3765	0.9780	TGVFLFTASSGGLPTDEITFAKLLK
12.7	2692.3414	1.0131	NVNGYSYKVAVALSLFLGWLGADR
10.0	2692.3414	1.0131	NVNGYSYKVAVALSLFLGWLGADR
9.4	2692.3765	0.9780	TGVFLFTASSGGLPTDEITFAKLLK
7.4	2692.3414	1.0131	NVNGYSYKVAVALSLFLGWLGADR
5.6	2691.3415	2.0130	LQDPVVSVALGQTVRITCQGDSLR

Spectrum No: 52; Query: 12115; Rank: 1

Peptide View

MS/MS Fragmentation of **LDFLRPYTVPNK**
Found in **IPI00003217**, Tax_Id=9606 Gene_Symbol=PSMB7 Proteasome subunit beta type-7

Match to Query 12115: 1461.801762 from(488.274530,3+)
Title: 090324LimSK_Exosome1_A.9103.9103.3.dta
Data file C:\Temp\Mascoti\LSK_2D\090324LimSK_Exosome1_A_2D.mgf

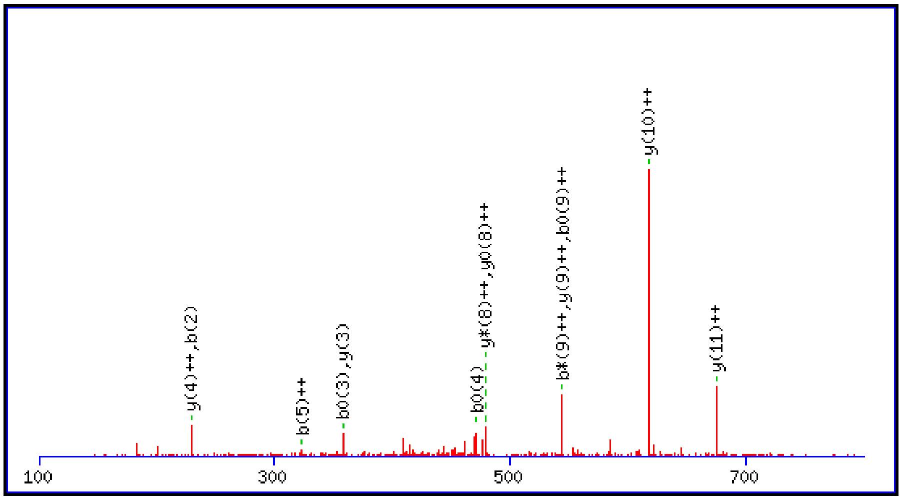

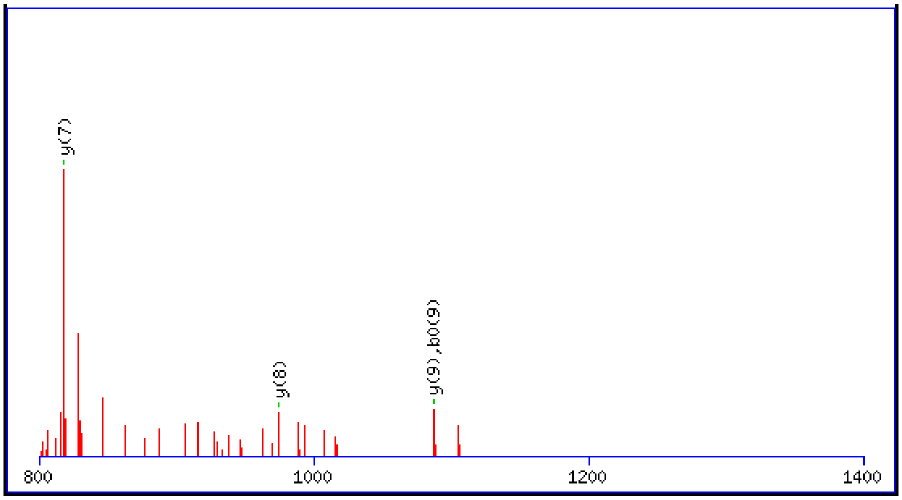

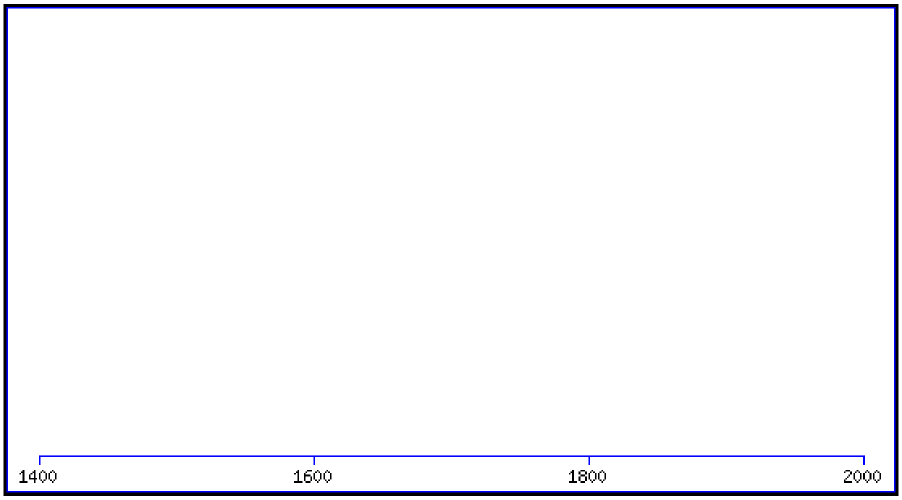

Monoisotopic mass of neutral peptide Mr(calc): 1461.7980
Fixed modifications: Carbamidomethyl (C)
Ions Score: 37 **Expect:** 0.021
Matches (Bold Red): 17/114 fragment ions using 17 most intense peaks

#	b	b^{++}	b*	b^{*++}	b^0	b^{0++}	Seq.	y	y^{++}	y*	y^{*++}	y^0	y^{0++}	#
1	114.0913	57.5493					L							12
2	**229.1183**	115.0628			211.1077	106.0575	D	1349.7212	**675.3642**	1332.6947	666.8510	1331.7106	666.3590	11
3	376.1867	188.5970			**358.1761**	179.5917	F	1234.6943	**617.8508**	1217.6677	609.3375	1216.6837	608.8455	10
4	489.2708	245.1390			**471.2602**	236.1337	L	**1087.6259**	**544.3166**	1070.5993	535.8033	1069.6153	535.3113	9
5	645.3719	**323.1896**	628.3453	314.6763	627.3613	314.1843	R	**974.5418**	487.7745	957.5152	**479.2613**	956.5312	**478.7693**	8
6	742.4246	371.7160	725.3981	363.2027	724.4141	362.7107	P	**818.4407**	409.7240	801.4141	401.2107	800.4301	400.7187	7
7	905.4880	453.2476	888.4614	444.7343	887.4774	444.2423	Y	721.3879	361.1976	704.3614	352.6843	703.3774	352.1923	6
8	1006.5356	503.7715	989.5091	495.2582	988.5251	494.7662	T	558.3246	279.6659	541.2980	271.1527	540.3140	270.6606	5
9	1105.6041	553.3057	1088.5775	**544.7924**	**1087.5935**	**544.3004**	V	457.2769	**229.1421**	440.2504	220.6288			4
10	1202.6568	601.8320	1185.6303	593.3188	1184.6463	592.8268	P	**358.2085**	179.6079	341.1819	171.0946			3
11	1316.6997	658.8535	1299.6732	650.3402	1298.6892	649.8482	N	261.1557	131.0815	244.1292	122.5682			2
12							K	147.1128	74.0600	130.0863	65.5468			1

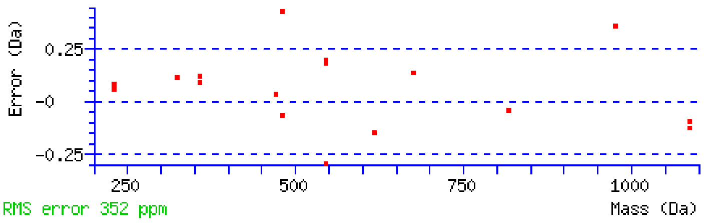

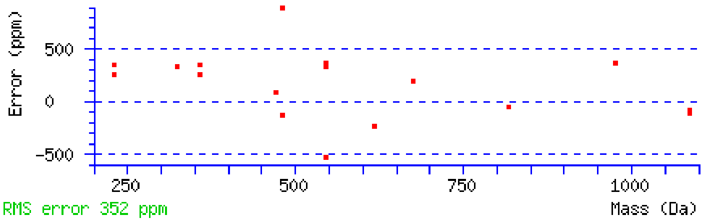

All matches to this query

Score	Mr(calc):	Delta	Sequence
36.7	1461.7980	0.0038	LDFLRPYTVPNK
17.1	1460.7875	1.0143	LLFSATLTQNPEK
12.7	1461.7874	0.0143	MPFTGERRLSLR
7.5	1460.8021	0.9997	LQKMKALEETVR
6.1	1461.7939	0.0078	NLFTALNAVEKSR
5.9	1461.7969	0.0048	RAALLVPPRYAR
5.7	1460.7977	1.0041	ILAVSPSRRINR
5.7	1460.7977	1.0041	ILAVSPSRRINR
4.1	1461.7948	0.0069	MLLCYRHLKTK
4.1	1459.7912	2.0106	SRLAKPKPNLEK

**Mascot:** http://www.matrixscience.com/
