# Supplementary material for: Proteolytic Potential of the MSC Exosome Proteome: Implications for an Exosome-Mediated Delivery of Therapeutic Proteasome
Source: Int J Proteomics. 2012 Jul 18;2012:971907. doi: 10.1155/2012/971907 (PMC3407643; doi:10.1155/2012/971907)
Supplement: Supplementary file 1 — Supplementary material provides the raw data of the detected 20S proteasome in 3 independent batches of exosomes. Supplementary data 1 provides a summary of number of detected peptides in 3 independent batches of exosomes; Supplementary data 2–4 provide the raw data of detected 20S proteasome peptides in 3 independent batches of exosomes respectively; Supplementary data 5–7 provide the Mascot peptide view of unique 20S proteasome peptides in 3 independent batches of exosomes respectively. [file 971907.f1.zip › SupplementaryData6_Exosome2_MascotPeptideView_UniqueProteasomePeptides.pdf]

Mascot Search Results

Results Generated by a Script Modified from Mascot Pepitde View
by Newman Sze, School of Biological Sciences, Nanyang Technological University

---

Spectrum No: 1; Query: 59524; Rank: 1

Peptide View

MS/MS Fragmentation of **AINQGGLTSVAVR**
Found in **IPI00029623**, Tax_Id=9606 Gene_Symbol=PSMA6 Proteasome subunit alpha type-6

Match to Query 59524: 1284.717728 from(643.366140,2+)
Title: 090702LimSK_Exosome2_06.3901.3901.2.dta
Data file C:\Temp\Mascoti\LSK_2D\090702LimSK_Exosome2_01_2D.mgf

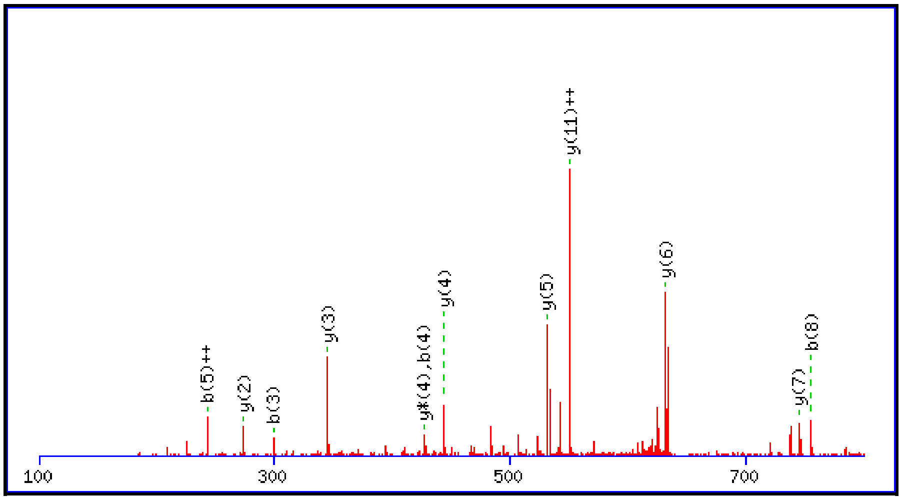

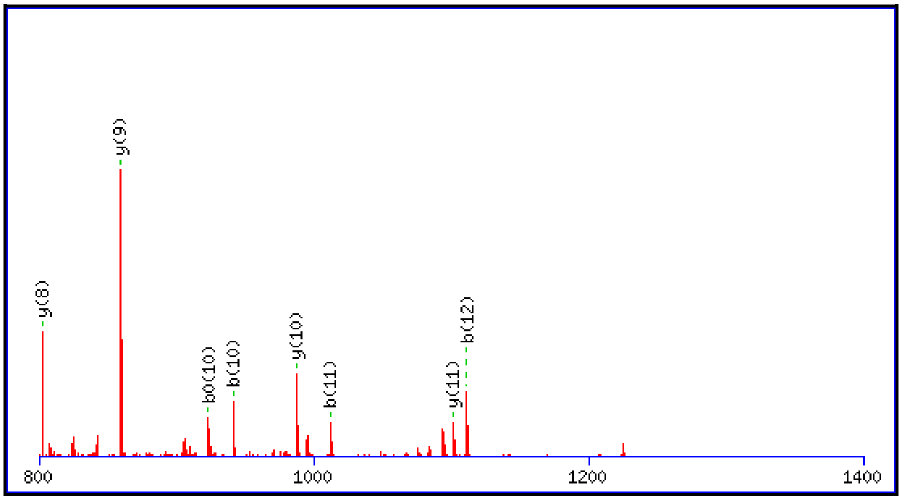

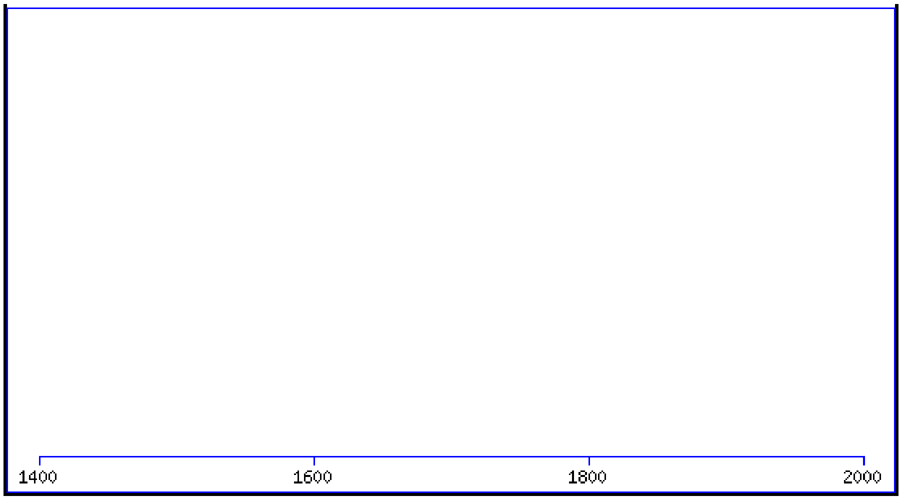

Monoisotopic mass of neutral peptide Mr(calc): 1284.7150
Fixed modifications: Carbamidomethyl (C)
Ions Score: 95 **Expect:** 2.5e-008
Matches (Bold Red): 20/118 fragment ions using 24 most intense peaks

#	b	b^{++}	b*	b^{*++}	b^0	b^{0++}	Seq.	y	y^{++}	y*	y^{*++}	y^0	y^{0++}	#
1	72.0444	36.5258					A							13
2	185.1285	93.0679					I	1214.6852	607.8462	1197.6586	599.3329	1196.6746	598.8409	12
3	**299.1714**	150.0893	282.1448	141.5761			N	**1101.6011**	**551.3042**	1084.5745	542.7909	1083.5905	542.2989	11
4	**427.2300**	214.1186	410.2034	205.6053			Q	**987.5582**	494.2827	970.5316	485.7694	969.5476	485.2774	10
5	484.2514	**242.6293**	467.2249	234.1161			G	**859.4996**	430.2534	842.4730	421.7402	841.4890	421.2482	9
6	541.2729	271.1401	524.2463	262.6268			G	**802.4781**	401.7427	785.4516	393.2294	784.4676	392.7374	8
7	654.3570	327.6821	637.3304	319.1688			L	**745.4567**	373.2320	728.4301	364.7187	727.4461	364.2267	7
8	**755.4046**	378.2060	738.3781	369.6927	737.3941	369.2007	T	**632.3726**	316.6899	615.3461	308.1767	614.3620	307.6847	6
9	842.4367	421.7220	825.4101	413.2087	824.4261	412.7167	S	**531.3249**	266.1661	514.2984	257.6528	513.3144	257.1608	5
10	**941.5051**	471.2562	924.4785	462.7429	**923.4945**	462.2509	V	**444.2929**	222.6501	**427.2663**	214.1368			4
11	**1012.5422**	506.7747	995.5156	498.2615	994.5316	497.7694	A	**345.2245**	173.1159	328.1979	164.6026			3
12	**1111.6106**	556.3089	1094.5841	547.7957	1093.6000	547.3037	V	**274.1874**	137.5973	257.1608	129.0840			2
13							R	175.1190	88.0631	158.0924	79.5498			1

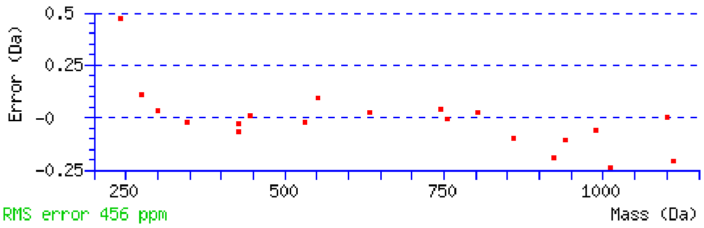

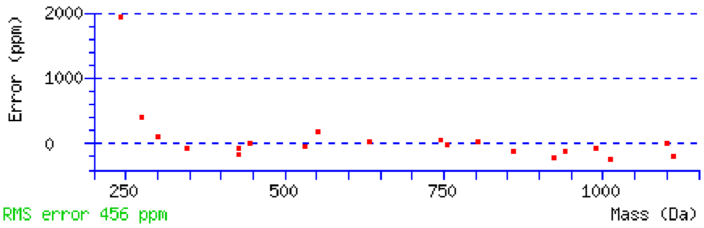

All matches to this query

Score	Mr(calc):	Delta	Sequence
95.3	1284.7150	0.0027	AINQGGLTSVAVR
5.5	1284.7150	0.0027	LRELVAGDSAVR
5.4	1284.7262	-0.0085	LQEVRESIRR
4.3	1284.7224	-0.0046	MKEEVKGIPVR
3.5	1283.7115	1.0062	TYRAVVKIVR
3.5	1283.7115	1.0062	TYRAVVKIVR
3.4	1284.7262	-0.0085	AIDARSIDRLR
3.1	1284.7262	-0.0085	SLRADIERVAR
2.6	1284.7262	-0.0085	ERRVLTATPSR

2.5	1284.7262	-0.0085	ELEQRVSLRR

Spectrum No: 2; Query: 62306; Rank: 1

Peptide View

MS/MS Fragmentation of **CDPAGYYCGFK**
Found in **IPI00029623**, Tax_Id=9606 Gene_Symbol=PSMA6 Proteasome subunit alpha type-6

Match to Query 62306: 1336.524428 from(669.269490,2+)
Title: 090702LimSK_Exosome2_05.4871.4871.2.dta
Data file C:\Temp\Mascoti\LSK_2D\090702LimSK_Exosome2_01_2D.mgf

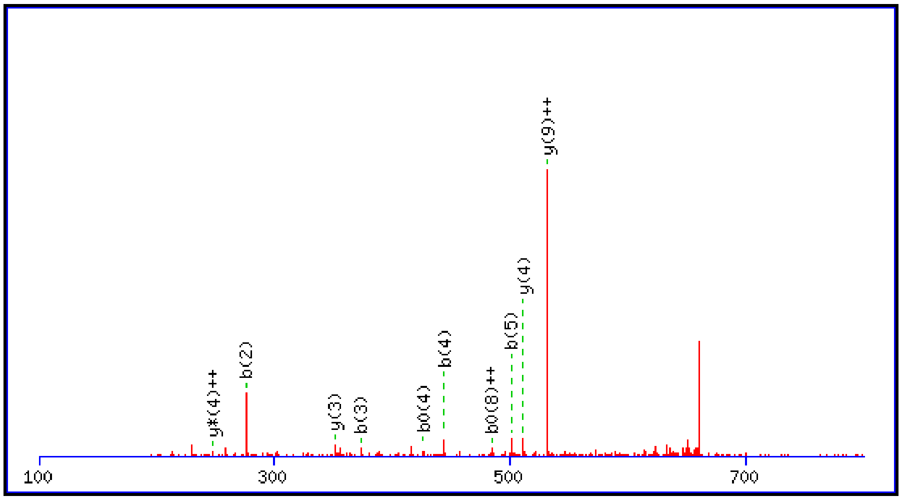

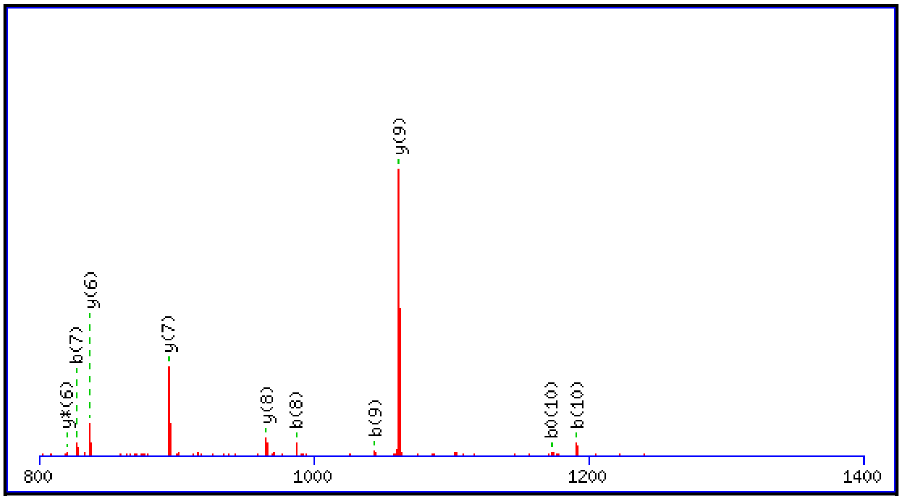

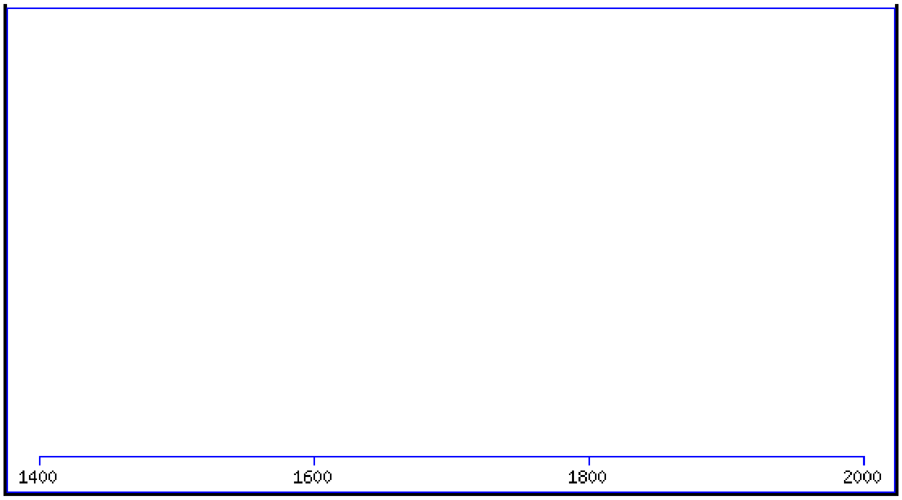

Monoisotopic mass of neutral peptide Mr(calc): 1336.5217
Fixed modifications: Carbamidomethyl (C)
Ions Score: 42 **Expect:** 0.0069
Matches (Bold Red): 20/80 fragment ions using 46 most intense peaks

#	b	b^{++}	b^{0}	b^{0++}	Seq.	y	y^{++}	y*	y^{*++}	y^{0}	y^{0++}	#
1	161.0379	81.0226			C							11
2	**276.0649**	138.5361	258.0543	129.5308	D	1177.4983	589.2528	1160.4717	580.7395	1159.4877	580.2475	10
3	**373.1176**	187.0625	355.1071	178.0572	P	**1062.4713**	**531.7393**	1045.4448	523.2260			9
4	**444.1547**	222.5810	**426.1442**	213.5757	A	**965.4186**	483.2129	948.3920	474.6996			8
5	**501.1762**	251.0917	483.1656	242.0865	G	**894.3815**	447.6944	877.3549	439.1811			7
6	664.2395	332.6234	646.2290	323.6181	Y	**837.3600**	419.1836	**820.3334**	410.6704			6
7	**827.3029**	414.1551	809.2923	405.1498	Y	674.2967	337.6520	657.2701	329.1387			5
8	**987.3335**	494.1704	969.3230	**485.1651**	C	**511.2333**	256.1203	494.2068	**247.6070**			4
9	**1044.3550**	522.6811	1026.3444	513.6758	G	**351.2027**	176.1050	334.1761	167.5917			3
10	**1191.4234**	596.2153	**1173.4128**	587.2101	F	294.1812	147.5942	277.1547	139.0810			2
11					K	147.1128	74.0600	130.0863	65.5468			1

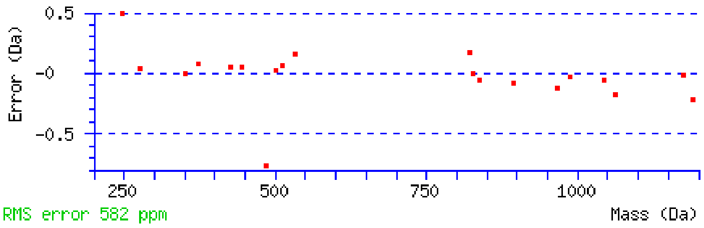

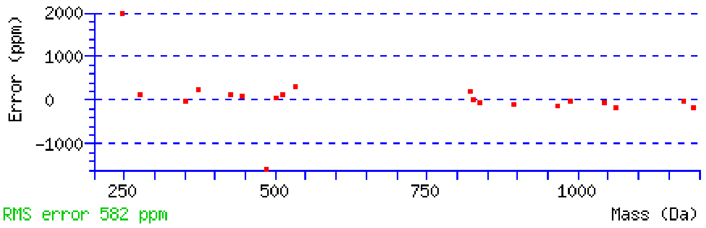

All matches to this query

Score	Mr(calc):	Delta	Sequence
41.8	1336.5217	0.0028	CDPAGYYCGFK
23.8	1336.5257	-0.0013	YAESLSEAMEK
16.4	1336.5193	0.0051	XCSFGVQLMGK
12.1	1336.5353	-0.0109	SFIDLAPASEK
12.1	1336.5135	0.0109	CDQGASLTVVK
11.8	1335.5261	0.9983	EEKKYHSQK
11.4	1336.5313	-0.0068	TGGTNTEKLEK
11.3	1336.5135	0.0109	KMTTSTDHLK
10.6	1336.5257	-0.0013	YAESLSEAMEK
10.3	1335.5331	0.9913	MQPAMMMFSSK

Spectrum No: 3; Query: 44526; Rank: 1

Peptide View

MS/MS Fragmentation of **DCAVIVTQK**
Found in **IPI00029623**, Tax_Id=9606 Gene_Symbol=PSMA6 Proteasome subunit alpha type-6

Match to Query 44526: 1032.530408 from(517.272480,2+)
Title: 090702LimSK_Exosome2_06.1366.1366.2.dta
Data file C:\Temp\Mascoti\LSK_2D\090702LimSK_Exosome2_01_2D.mgf

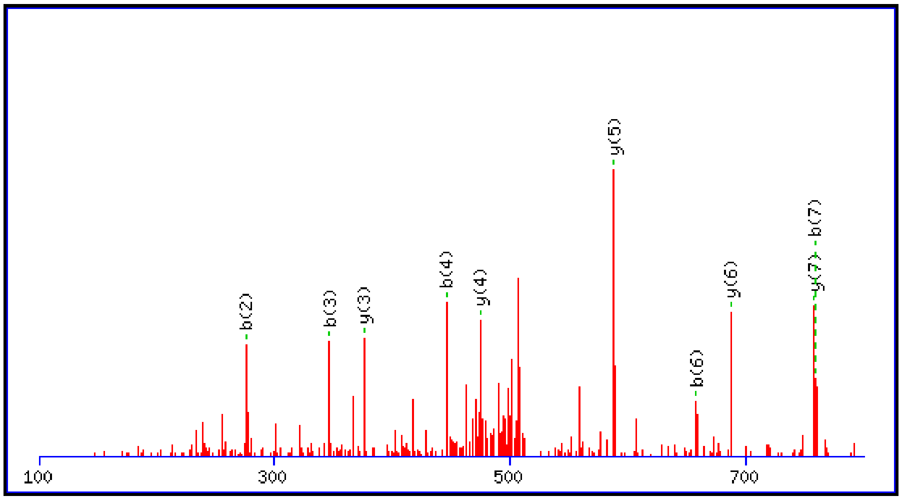

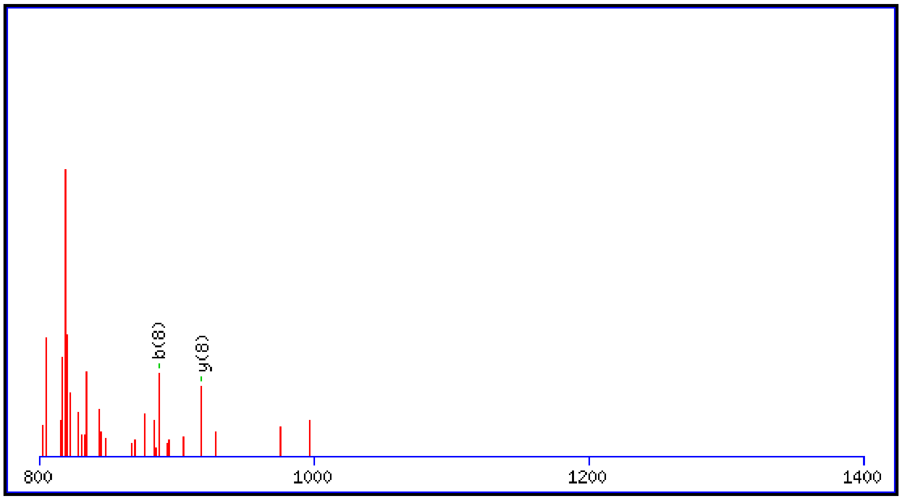

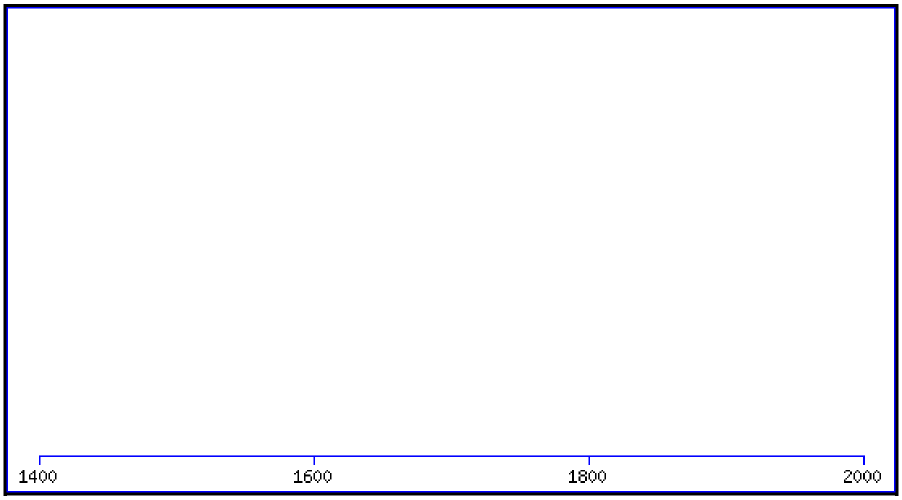

Monoisotopic mass of neutral peptide Mr(calc): 1032.5274

Fixed modifications: Carbamidomethyl (C)
Ions Score: 59 **Expect:** 0.00018
Matches (Bold Red): 12/78 fragment ions using 27 most intense peaks

#	b	b^{++}	b*	b^{*++}	b^{0}	b^{0++}	Seq.	y	y^{++}	y*	y^{*++}	y^{0}	y^{0++}	#
1	116.0342	58.5207			98.0237	49.5155	D							9
2	**276.0649**	138.5361			258.0543	129.5308	C	**918.5077**	459.7575	901.4812	451.2442	900.4972	450.7522	8
3	**347.1020**	174.0546			329.0914	165.0493	A	**758.4771**	379.7422	741.4505	371.2289	740.4665	370.7369	7
4	**446.1704**	223.5888			428.1598	214.5836	V	**687.4400**	344.2236	670.4134	335.7103	669.4294	335.2183	6
5	559.2545	280.1309			541.2439	271.1256	I	**588.3715**	294.6894	571.3450	286.1761	570.3610	285.6841	5
6	**658.3229**	329.6651			640.3123	320.6598	V	**475.2875**	238.1474	458.2609	229.6341	457.2769	229.1421	4
7	**759.3706**	380.1889			741.3600	371.1836	T	**376.2191**	188.6132	359.1925	180.0999	358.2085	179.6079	3
8	**887.4291**	444.2182	870.4026	435.7049	869.4186	435.2129	Q	275.1714	138.0893	258.1448	129.5761			2
9							K	147.1128	74.0600	130.0863	65.5468			1

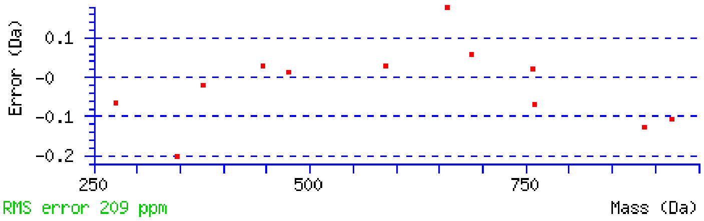

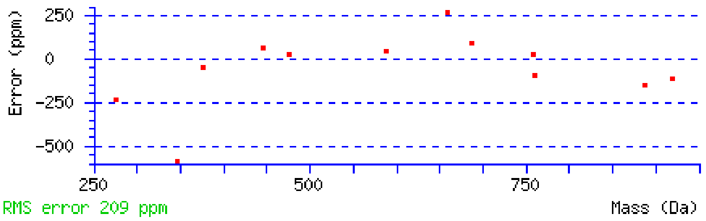

All matches to this query

Score	Mr(calc):	Delta	Sequence
59.1	1032.5274	0.0030	DCAVIVTQK
31.2	1032.5386	-0.0082	DCAQGVKKK
16.9	1032.5274	0.0030	MANSSPVLSK
10.8	1032.5274	0.0030	MAASQVLGEK
9.5	1032.5352	-0.0048	GWEERTKK
9.3	1032.5386	-0.0082	SALQRMAEK
9.1	1032.5352	-0.0048	EIAHVHAEK
8.9	1030.5229	2.0075	AAMAQVIGDR
8.3	1032.5386	-0.0082	KCSVNLANK
6.8	1031.5247	1.0057	GESGIGAKGEK

Spectrum No: 4; Query: 56461; Rank: 1

Peptide View

MS/MS Fragmentation of **GKDCAVIVTQK**
Found in **IPI00029623**, Tax_Id=9606 Gene_Symbol=PSMA6 Proteasome subunit alpha type-6

Match to Query 56461: 1217.648328 from(609.831440,2+)
Title: 090702LimSK_Exosome2_03.2956.2956.2.dta
Data file C:\Temp\Mascoti\LSK_2D\090702LimSK_Exosome2_01_2D.mgf

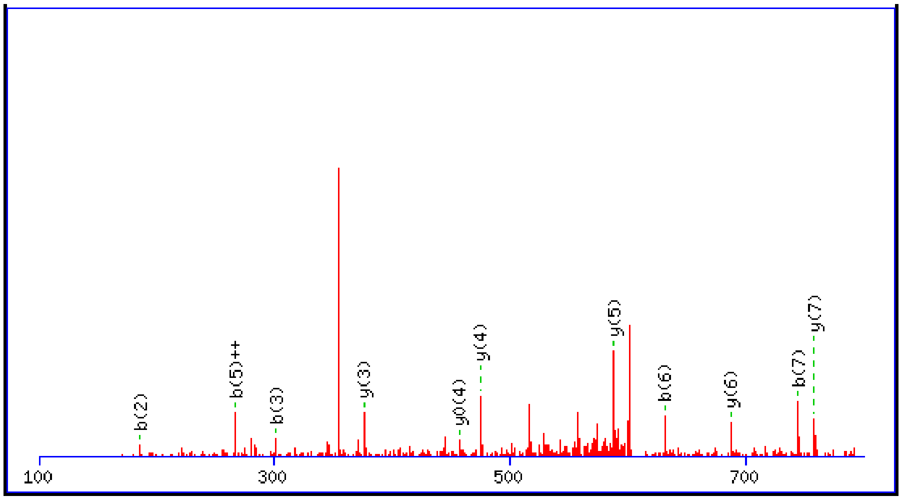

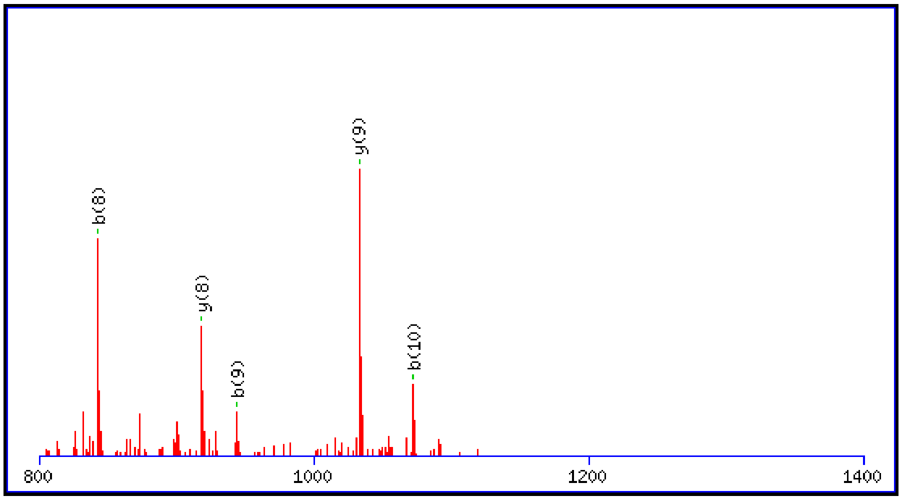

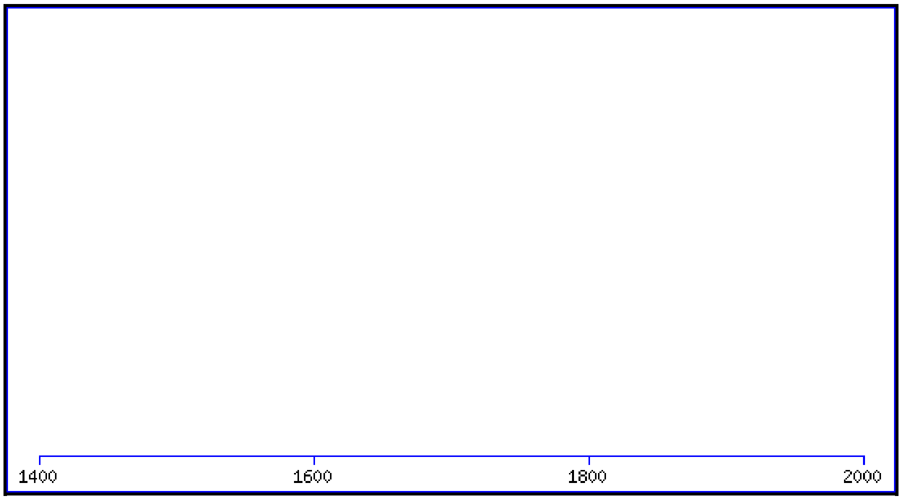

Monoisotopic mass of neutral peptide Mr(calc): 1217.6438
Fixed modifications: Carbamidomethyl (C)
Ions Score: 51 **Expect:** 0.0011
Matches (Bold Red): 16/110 fragment ions using 31 most intense peaks

#	b	b^{++}	b*	b^{*++}	b^0	b^{0++}	Seq.	y	y^{++}	y*	y^{*++}	y^0	y^{0++}	#
1	58.0287	29.5180					G							11
2	**186.1237**	93.5655	169.0972	85.0522			K	1161.6296	581.3184	1144.6031	572.8052	1143.6191	572.3132	10
3	**301.1506**	151.0790	284.1241	142.5657	283.1401	142.0737	D	**1033.5347**	517.2710	1016.5081	508.7577	1015.5241	508.2657	9
4	461.1813	231.0943	444.1547	222.5810	443.1707	222.0890	C	**918.5077**	459.7575	901.4812	451.2442	900.4972	450.7522	8
5	532.2184	**266.6128**	515.1919	258.0996	514.2078	257.6076	A	**758.4771**	379.7422	741.4505	371.2289	740.4665	370.7369	7
6	**631.2868**	316.1470	614.2603	307.6338	613.2763	307.1418	V	**687.4400**	344.2236	670.4134	335.7103	669.4294	335.2183	6

**7**	**744.3709**	372.6891	727.3443	364.1758	726.3603	363.6838	**I**	**588.3715**	294.6894	571.3450	286.1761	570.3610	285.6841	**5**
**8**	**843.4393**	422.2233	826.4128	413.7100	825.4287	413.2180	**V**	**475.2875**	238.1474	458.2609	229.6341	**457.2769**	229.1421	**4**
**9**	**944.4870**	472.7471	927.4604	464.2339	926.4764	463.7418	**T**	**376.2191**	188.6132	359.1925	180.0999	358.2085	179.6079	**3**
**10**	**1072.5456**	536.7764	1055.5190	528.2631	1054.5350	527.7711	**Q**	275.1714	138.0893	258.1448	129.5761			**2**
**11**							**K**	147.1128	74.0600	130.0863	65.5468			**1**

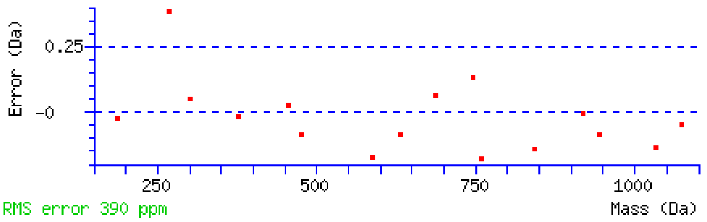

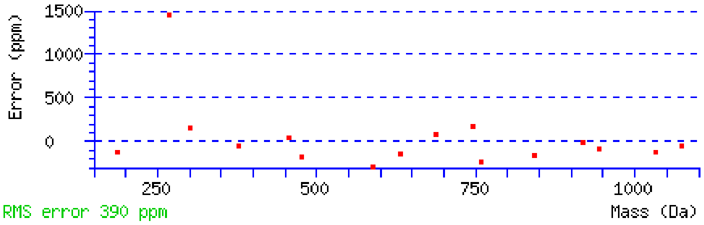

All matches to this query

Score	Mr(calc):	Delta	Sequence
51.2	1217.6438	0.0045	GKDCAVIVTQK
15.0	1217.6438	0.0045	AQMNLGSLIQK
9.4	1216.6468	1.0015	YVLLSSLSKK
7.7	1216.6411	1.0072	AVENSSTAIGIR
6.4	1217.6364	0.0119	NGERISVSAASK
5.5	1216.6468	1.0015	YVLLSSLSKK
4.3	1216.6458	1.0025	MAATRSPTRAR
4.0	1216.6411	1.0072	GQDRSEATLIK
2.8	1217.6438	0.0045	GKCTVLNADIK
2.7	1217.6405	0.0079	QGLPGPVGDPGPK

Spectrum No: 5; Query: 32383; Rank: 1

Peptide View

MS/MS Fragmentation of **GSSAGFDR**
Found in **IPI00029623**, Tax_Id=9606 Gene_Symbol=PSMA6 Proteasome subunit alpha type-6

Match to Query 32383: 795.352028 from(398.683290,2+)
Title: 090702LimSK_Exosome2_06.724.724.2.dta
Data file C:\Temp\Mascoti\LSK_2D\090702LimSK_Exosome2_01_2D.mgf

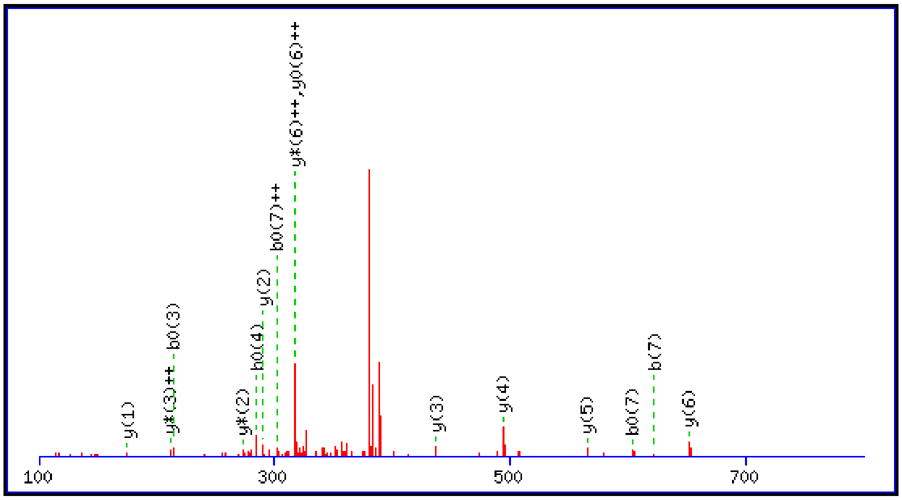

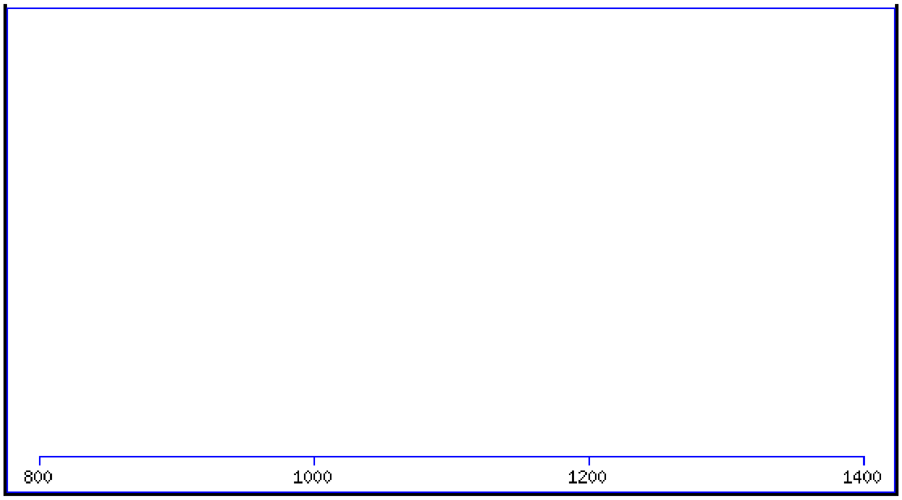

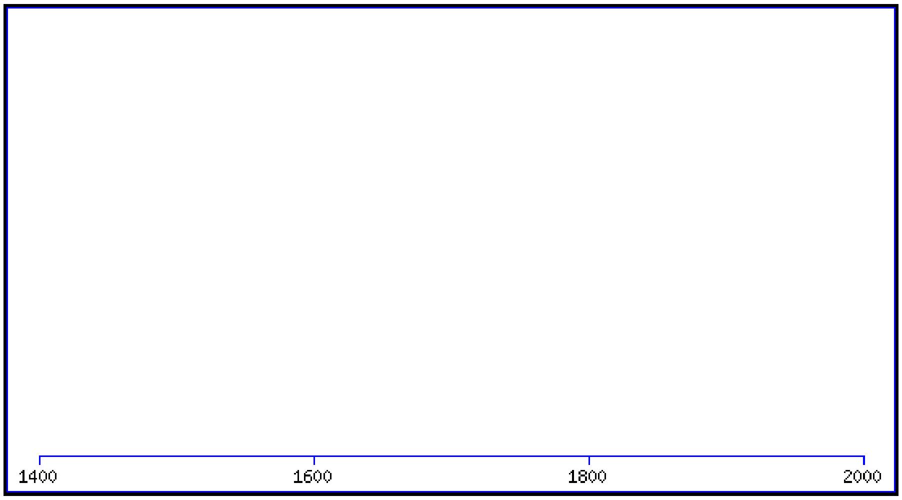

Monoisotopic mass of neutral peptide Mr(calc): 795.3511
Fixed modifications: Carbamidomethyl (C)
Ions Score: 42 **Expect:** 0.0043
Matches (Bold Red): 15/66 fragment ions using 21 most intense peaks

#	b	b^{++}	b^0	b^{0++}	Seq.	y	y^{++}	y*	y^{*++}	y^0	y^{0++}	#
1	58.0287	29.5180			G							8
2	145.0608	73.0340	127.0502	64.0287	S	739.3369	370.1721	722.3104	361.6588	721.3264	361.1668	7
3	232.0928	116.5500	**214.0822**	107.5448	S	**652.3049**	326.6561	635.2784	**318.1428**	634.2943	**317.6508**	6
4	303.1299	152.0686	**285.1193**	143.0633	A	**565.2729**	283.1401	548.2463	274.6268	547.2623	274.1348	5
5	360.1514	180.5793	342.1408	171.5740	G	**494.2358**	247.6215	477.2092	239.1082	476.2252	238.6162	4
6	507.2198	254.1135	489.2092	245.1082	F	**437.2143**	219.1108	420.1878	**210.5975**	419.2037	210.1055	3
7	**622.2467**	311.6270	**604.2362**	**302.6217**	D	**290.1459**	145.5766	**273.1193**	137.0633	272.1353	136.5713	2
8					R	**175.1190**	88.0631	158.0924	79.5498			1

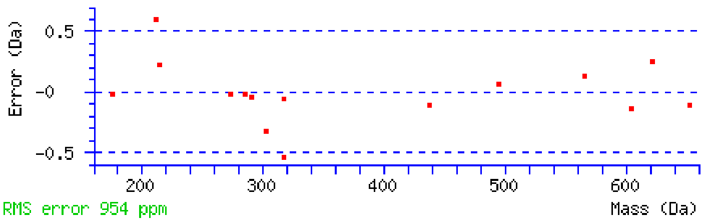

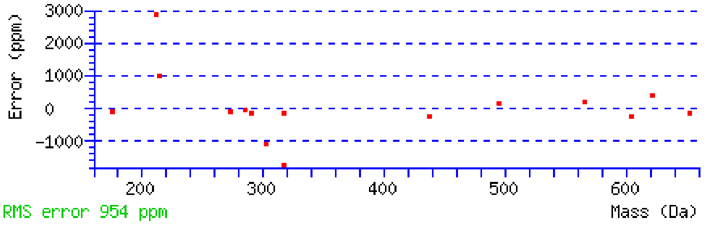

All matches to this query

Score	Mr(calc):	Delta	Sequence
41.9	795.3511	0.0009	GSSAGFDR

20.6	795.3545	-0.0025	SGSMKDR
9.9	793.3484	2.0037	TTIHSR
6.3	795.3545	-0.0025	SSCLSSR
5.7	795.3528	-0.0007	LTPTER
5.0	793.3389	2.0132	ATMQGDR
3.3	795.3585	-0.0065	MPYVDR
2.5	793.3454	2.0067	ASASPSTSS
1.8	795.3511	0.0009	DNSSFAR
1.5	794.3415	1.0106	XEMIAR

Spectrum No: 6; Query: 52504; Rank: 1

Peptide View

MS/MS Fragmentation of **HITIFSPEGR**
Found in **IPI00029623**, Tax_Id=9606 Gene_Symbol=PSMA6 Proteasome subunit alpha type-6

Match to Query 52504: 1155.605888 from(578.810220,2+)
Title: 090702LimSK_Exosome2_04.8854.8854.2.dta
Data file C:\Temp\Mascoti\LSK_2D\090702LimSK_Exosome2_01_2D.mgf

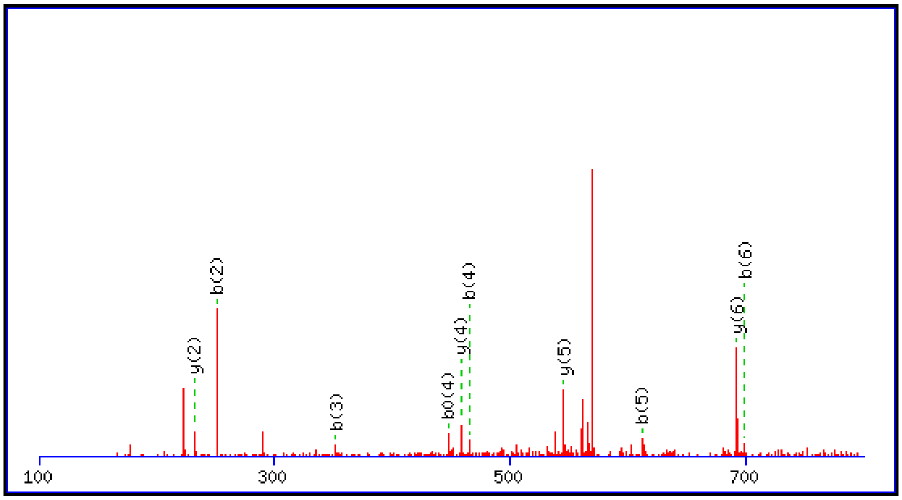

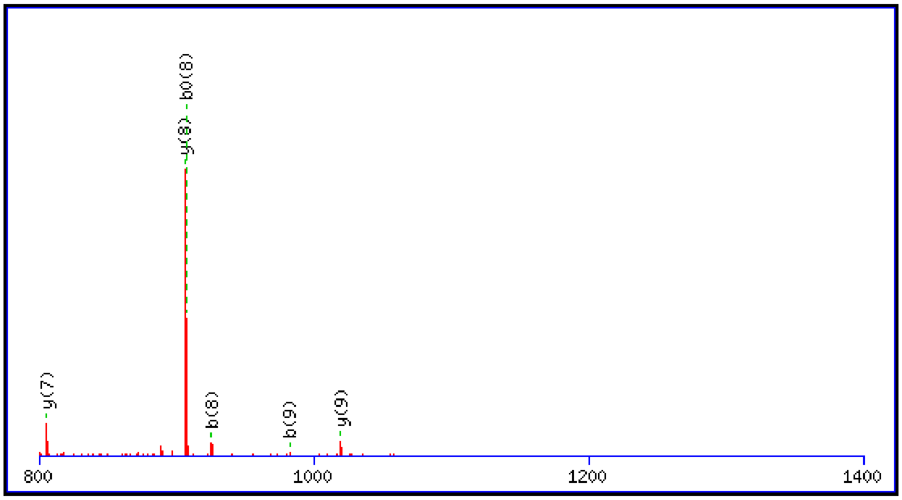

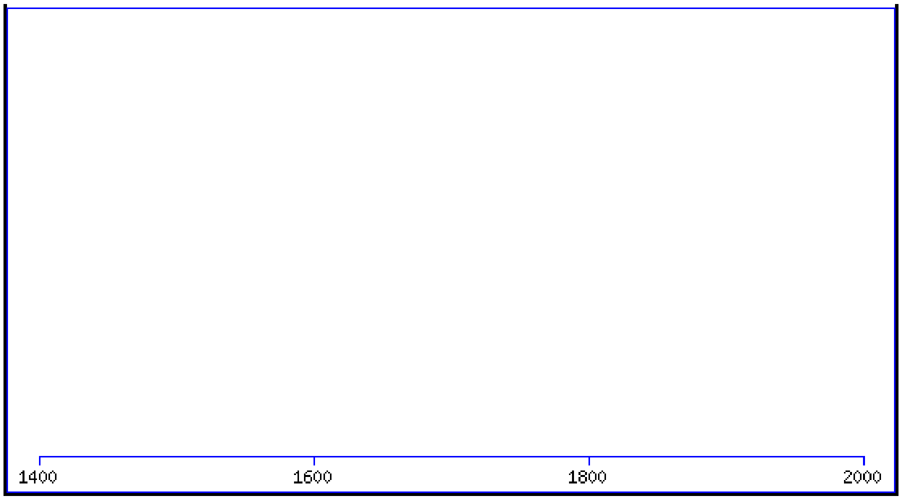

Monoisotopic mass of neutral peptide Mr(calc): 1155.6037
Fixed modifications: Carbamidomethyl (C)
Ions Score: 57 **Expect:** 0.0002
Matches (Bold Red): 16/82 fragment ions using 31 most intense peaks

#	b	b^{++}	b^{0}	b^{0++}	Seq.	y	y^{++}	y*	y^{*++}	y^{0}	y^{0++}	#
1	138.0662	69.5367			H							10
2	**251.1503**	126.0788			I	**1019.5520**	510.2796	1002.5255	501.7664	1001.5415	501.2744	9
3	**352.1979**	176.6026	334.1874	167.5973	T	**906.4680**	453.7376	889.4414	445.2243	888.4574	444.7323	8
4	**465.2820**	233.1446	**447.2714**	224.1394	I	**805.4203**	403.2138	788.3937	394.7005	787.4097	394.2085	7
5	**612.3504**	306.6788	594.3398	297.6736	F	**692.3362**	346.6717	675.3097	338.1585	674.3257	337.6665	6
6	**699.3824**	350.1949	681.3719	341.1896	S	**545.2678**	273.1375	528.2413	264.6243	527.2572	264.1323	5
7	796.4352	398.7212	778.4246	389.7160	P	**458.2358**	229.6215	441.2092	221.1083	440.2252	220.6162	4
8	**925.4778**	463.2425	**907.4672**	454.2373	E	361.1830	181.0951	344.1565	172.5819	343.1724	172.0899	3
9	**982.4993**	491.7533	964.4887	482.7480	G	**232.1404**	116.5738	215.1139	108.0606			2
10					R	175.1190	88.0631	158.0924	79.5498			1

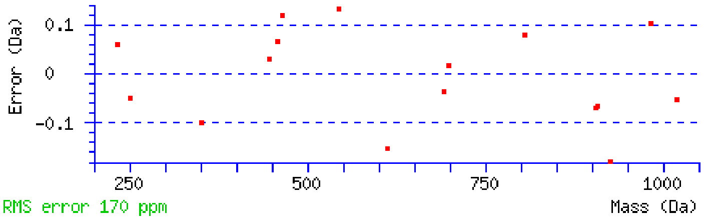

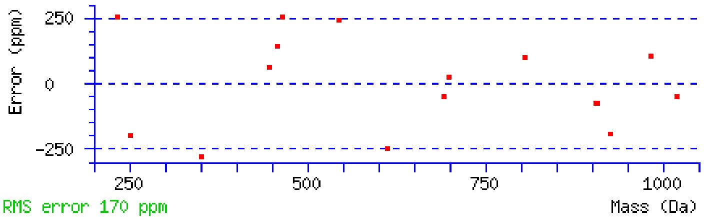

All matches to this query

Score	Mr(calc):	Delta	Sequence
57.5	1155.6037	0.0022	HITIFSPEGR
12.6	1155.6070	-0.0012	HLTQAVCTVK
4.0	1155.6037	0.0022	QHSIFGDPKK
3.4	1154.5948	1.0111	LITGTLSKDK
2.5	1155.5996	0.0063	HLSDSKSQVR
1.5	1155.6053	0.0006	SKYKVNPIK

Spectrum No: 7; Query: 84230; Rank: 1

Peptide View

MS/MS Fragmentation of **ILTEAEIDAHLVALAERD**
Found in **IPI00029623**, Tax_Id=9606 Gene_Symbol=PSMA6 Proteasome subunit alpha type-6

Match to Query 84230: 1978.044402 from(660.355410,3+)
Title: 090702LimSK_Exosome2_01.9709.9709.3.dta
Data file C:\Temp\Mascoti\LSK_2D\090702LimSK_Exosome2_01_2D.mgf

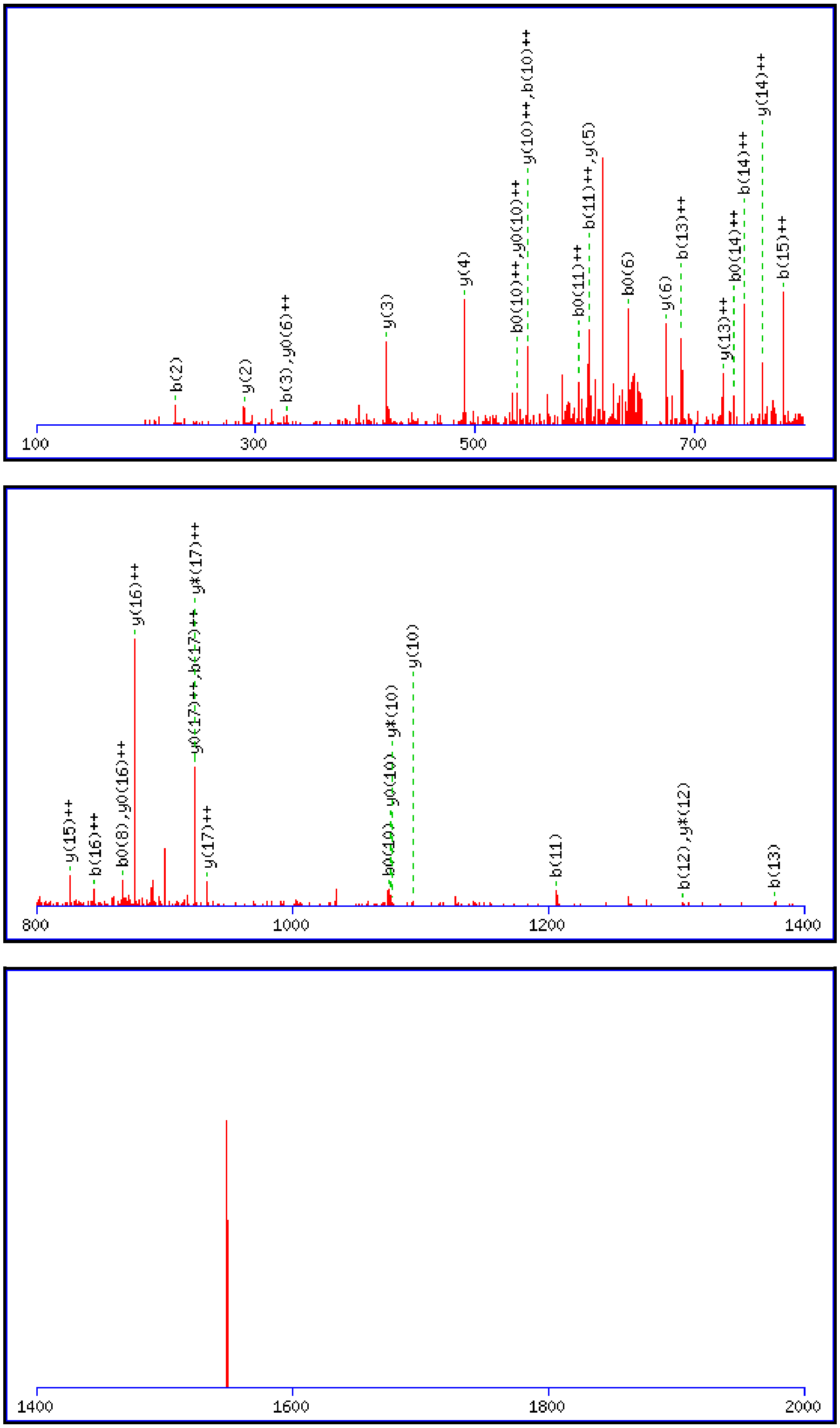

Monoisotopic mass of neutral peptide Mr(calc): 1978.0371
Fixed modifications: Carbamidomethyl (C)
Ions Score: 38 **Expect:** 0.017
Matches (Bold Red): 38/166 fragment ions using 78 most intense peaks

#	b	b++	b*	b*++	b0	b0++	Seq.	y	y++	y*	y*++	y0	y0++	#
1	114.0913	57.5493					I							18

2	**227.1754**	114.0913					L	1865.9603	**933.4838**	1848.9338	**924.9705**	1847.9498	**924.4785**	17
3	**328.2231**	164.6152			310.2125	155.6099	T	1752.8763	**876.9418**	1735.8497	868.4285	1734.8657	**867.9365**	16
4	457.2657	229.1365			439.2551	220.1312	E	1651.8286	**826.4179**	1634.8020	817.9047	1633.8180	817.4127	15
5	528.3028	264.6550			510.2922	255.6498	A	1522.7860	**761.8966**	1505.7594	753.3834	1504.7754	752.8914	14
6	657.3454	329.1763			**639.3348**	320.1710	E	1451.7489	**726.3781**	1434.7223	717.8648	1433.7383	717.3728	13
7	770.4294	385.7184			752.4189	376.7131	I	1322.7063	661.8568	**1305.6797**	653.3435	1304.6957	652.8515	12
8	885.4564	443.2318			**867.4458**	434.2266	D	1209.6222	605.3148	1192.5957	596.8015	1191.6117	596.3095	11
9	956.4935	478.7504			938.4829	469.7451	A	**1094.5953**	**547.8013**	**1077.5687**	539.2880	**1076.5847**	**538.7960**	10
10	1093.5524	**547.2798**			**1075.5419**	**538.2746**	H	1023.5582	512.2827	1006.5316	503.7694	1005.5476	503.2774	9
11	**1206.6365**	**603.8219**			1188.6259	**594.8166**	L	886.4993	443.7533	869.4727	435.2400	868.4887	434.7480	8
12	**1305.7049**	653.3561			1287.6943	644.3508	V	773.4152	387.2112	756.3886	378.6980	755.4046	378.2060	7
13	**1376.7420**	**688.8746**			1358.7314	679.8694	A	**674.3468**	337.6770	657.3202	329.1638	656.3362	**328.6717**	6
14	1489.8261	**745.4167**			1471.8155	**736.4114**	L	**603.3097**	302.1585	586.2831	293.6452	585.2991	293.1532	5
15	1560.8632	**780.9352**			1542.8526	771.9299	A	**490.2256**	245.6164	473.1991	237.1032	472.2150	236.6112	4
16	1689.9058	**845.4565**			1671.8952	836.4512	E	**419.1885**	210.0979	402.1619	201.5846	401.1779	201.0926	3
17	1846.0069	**923.5071**	1828.9803	914.9938	1827.9963	914.5018	R	**290.1459**	145.5766	273.1193	137.0633	272.1353	136.5713	2
18							D	134.0448	67.5260			116.0342	58.5207	1

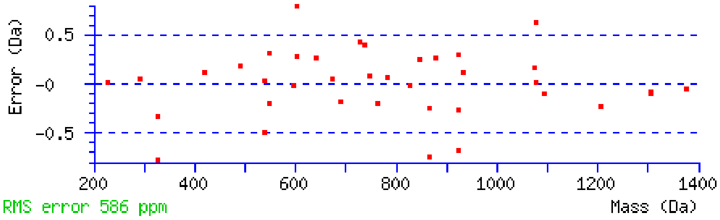

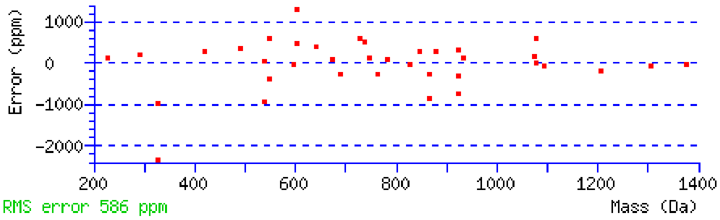

All matches to this query

Score	Mr(calc):	Delta	Sequence
38.4	1978.0371	0.0073	ILTEAEIDAHLVALAERD
5.3	1978.0564	-0.0120	LLTYEFVKQKYLDYR
2.8	1978.0418	0.0026	MAAEALRNYRNTQAILK
2.0	1976.0343	2.0101	EVGLTENTGLPRKLLEK
1.9	1976.0430	2.0014	LPLYLRLLQRMEHSK
1.8	1976.0343	2.0101	EVGLTENTGLPRKLLEK
1.6	1978.0398	0.0046	VQHLRDHPNYKYRPR
1.1	1978.0248	0.0196	ETQGIEKLVLINKSGNR
0.9	1977.0449	0.9995	GAPGPSGKPGIPGLQGLLGPK
0.9	1977.0482	0.9962	ILIEENRKVIAPMLSR

Spectrum No: 8; Query: 81971; Rank: 1

Peptide View

MS/MS Fragmentation of **ITENIGCVMTGMTADSR**
Found in **IPI00029623**, Tax_Id=9606 Gene_Symbol=PSMA6 Proteasome subunit alpha type-6

Match to Query 81971: 1854.829568 from(928.422060,2+)
Title: 090702LimSK_Exosome2_04.7093.7093.2.dta
Data file C:\Temp\Mascoti\LSK_2D\090702LimSK_Exosome2_01_2D.mgf

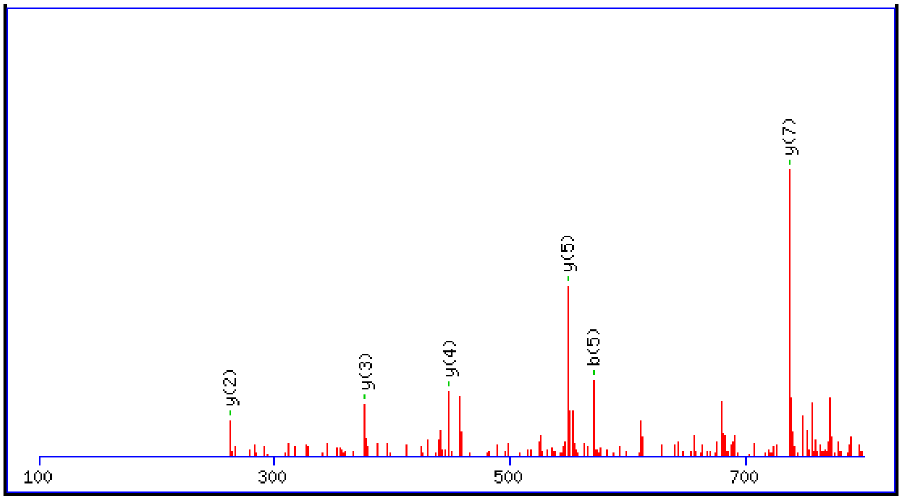

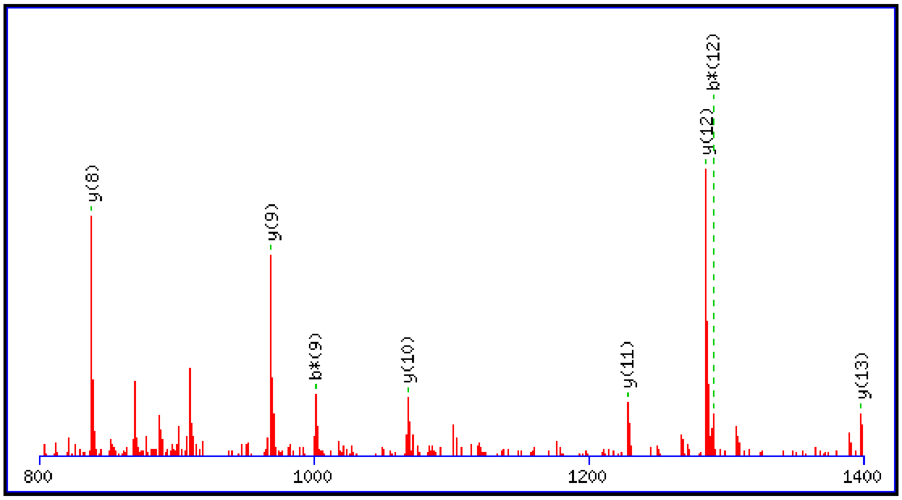

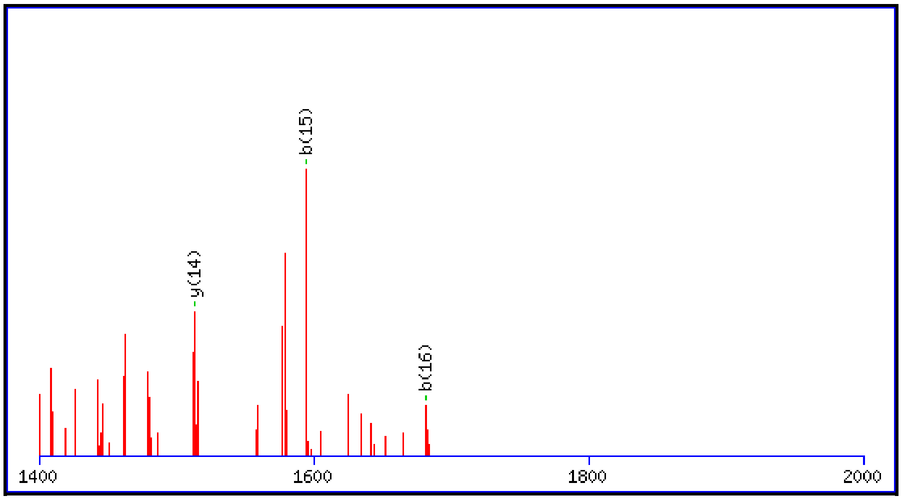

Monoisotopic mass of neutral peptide Mr(calc): 1854.8274
Fixed modifications: Carbamidomethyl (C)
Ions Score: 117 **Expect:** 4.4e-010
Matches (Bold Red): 17/182 fragment ions using 25 most intense peaks

#	b	b^{++}	b*	b^{*++}	b^0	b^{0++}	Seq.	y	y^{++}	y*	y^{*++}	y^0	y^{0++}	#
1	114.0913	57.5493					I							17
2	215.1390	108.0731			197.1285	99.0679	T	1742.7506	871.8789	1725.7241	863.3657	1724.7401	862.8737	16
3	344.1816	172.5944			326.1710	163.5892	E	1641.7029	821.3551	1624.6764	812.8418	1623.6924	812.3498	15
4	458.2245	229.6159	441.1980	221.1026	440.2140	220.6106	N	**1512.6603**	756.8338	1495.6338	748.3205	1494.6498	747.8285	14
5	**571.3086**	286.1579	554.2821	277.6447	553.2980	277.1527	I	**1398.6174**	699.8123	1381.5909	691.2991	1380.6069	690.8071	13
6	628.3301	314.6687	611.3035	306.1554	610.3195	305.6634	G	**1285.5334**	643.2703	1268.5068	634.7570	1267.5228	634.2650	12

7	788.3607	394.6840	771.3342	386.1707	770.3502	385.6787	C	1228.5119	614.7596	1211.4853	606.2463	1210.5013	605.7543	11
8	887.4291	444.2182	870.4026	435.7049	869.4186	435.2129	V	1068.4812	534.7443	1051.4547	526.2310	1050.4707	525.7390	10
9	1018.4696	509.7384	1001.4431	501.2252	1000.4591	500.7332	M	969.4128	485.2101	952.3863	476.6968	951.4023	476.2048	9
10	1119.5173	560.2623	1102.4907	551.7490	1101.5067	551.2570	T	838.3723	419.6898	821.3458	411.1765	820.3618	410.6845	8
11	1176.5388	588.7730	1159.5122	580.2597	1158.5282	579.7677	G	737.3247	369.1660	720.2981	360.6527	719.3141	360.1607	7
12	1307.5792	654.2933	1290.5527	645.7800	1289.5687	645.2880	M	680.3032	340.6552	663.2767	332.1420	662.2926	331.6500	6
13	1408.6269	704.8171	1391.6004	696.3038	1390.6164	695.8118	T	549.2627	275.1350	532.2362	266.6217	531.2522	266.1297	5
14	1479.6640	740.3357	1462.6375	731.8224	1461.6535	731.3304	A	448.2150	224.6112	431.1885	216.0979	430.2045	215.6059	4
15	1594.6910	797.8491	1577.6644	789.3359	1576.6804	788.8438	D	377.1779	189.0926	360.1514	180.5793	359.1674	180.0873	3
16	1681.7230	841.3651	1664.6965	832.8519	1663.7124	832.3599	S	262.1510	131.5791	245.1244	123.0659	244.1404	122.5738	2
17							R	175.1190	88.0631	158.0924	79.5498			1

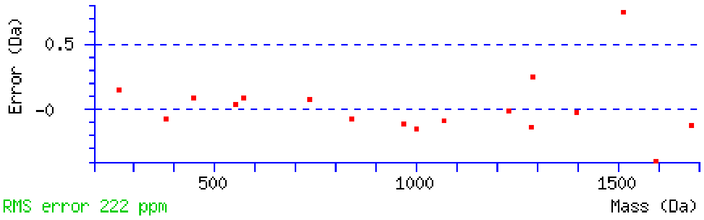

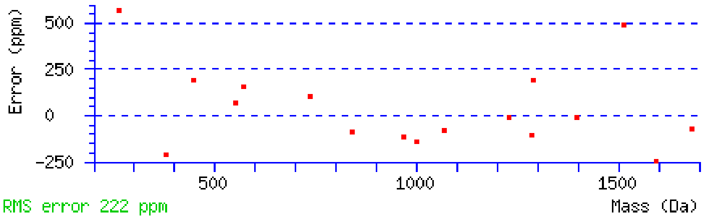

All matches to this query

Score	Mr(calc):	Delta	Sequence
117.2	1854.8274	0.0022	ITENIGCVMTGMTADSR
9.9	1854.8118	0.0178	CTSSHGCVTIIQSLGR
9.9	1854.8118	0.0178	CTSSHGCVTIIQSLGR
7.5	1852.8260	2.0035	DQLSDDVSKVIAYLK
3.9	1854.8473	-0.0177	AQTTGPSAGVTVTSGQSAR
3.9	1854.8473	-0.0177	AQTTGPSAGVTVTSGQSAR
3.9	1854.8473	-0.0177	AQTTGPSAGVTVTSGQSAR
2.8	1854.8122	0.0174	SWGAAGSARGSEGRPSSR
2.5	1853.8203	1.0092	AVDGEGARRGLCSPSSR
2.3	1853.8287	1.0009	MNIIADATIDLLFTK

Spectrum No: 9; Query: 63586; Rank: 1

Peptide View

MS/MS Fragmentation of **LLDSSTVTHLFK**
Found in **IPI00029623**, Tax_Id=9606 Gene_Symbol=PSMA6 Proteasome subunit alpha type-6

Match to Query 63586: 1359.742992 from(454.254940,3+)
Title: 090702LimSK_Exosome2_01.6742.6742.3.dta
Data file C:\Temp\Mascoti\LSK_2D\090702LimSK_Exosome2_01_2D.mgf

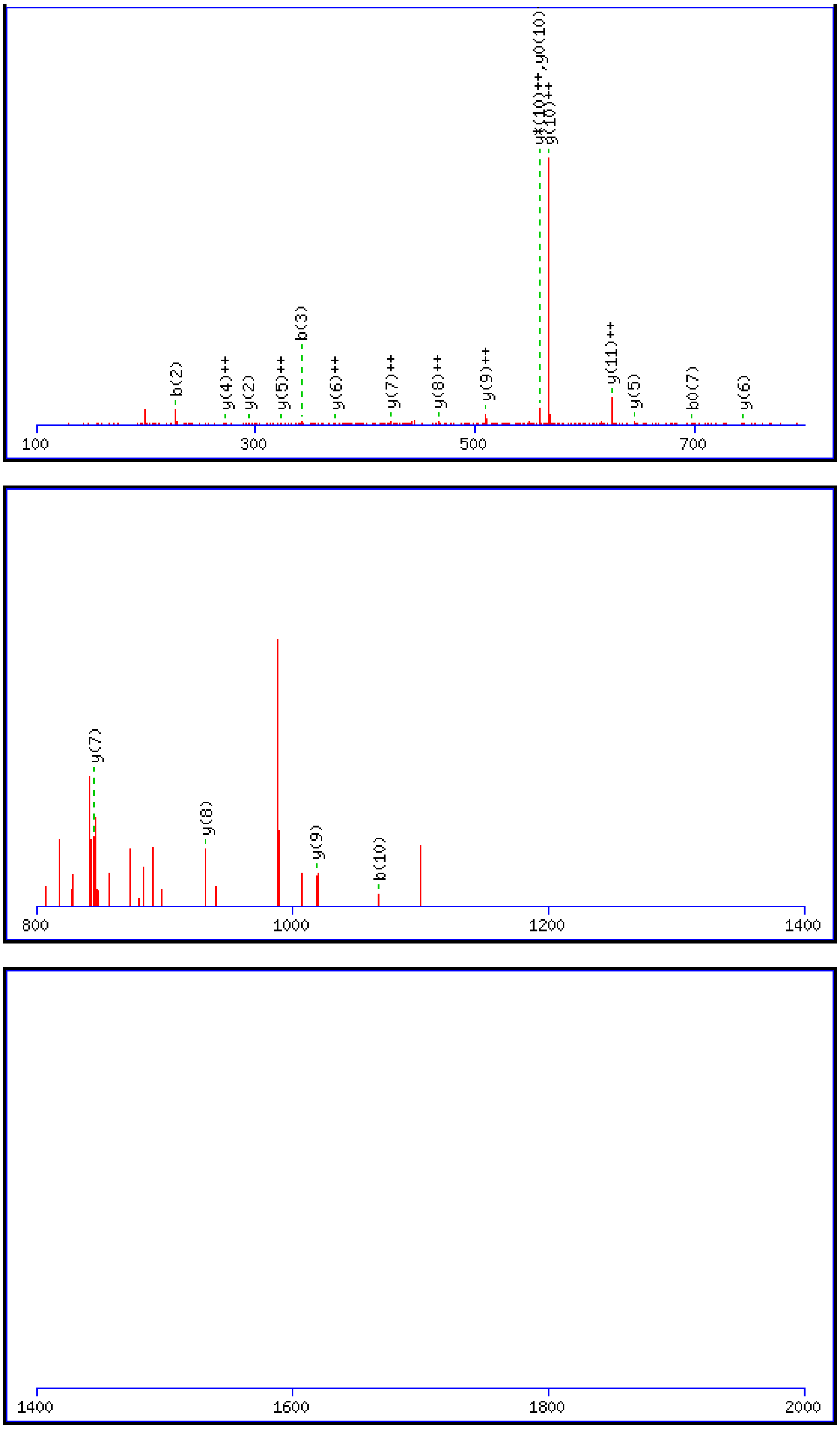

Monoisotopic mass of neutral peptide Mr(calc): 1359.7398
Fixed modifications: Carbamidomethyl (C)
Ions Score: 46 **Expect:** 0.0026
Matches (Bold Red): 20/98 fragment ions using 44 most intense peaks

#	b	b^{++}	b^{0}	b^{0++}	Seq.	y	y^{++}	y*	y^{*++}	y^{0}	y^{0++}	#
1	114.0913	57.5493			L							12
2	**227.1754**	114.0913			L	1247.6630	**624.3352**	1230.6365	615.8219	1229.6525	615.3299	11
3	**342.2023**	171.6048	324.1918	162.5995	D	1134.5790	**567.7931**	1117.5524	**559.2798**	1116.5684	**558.7878**	10
4	429.2344	215.1208	411.2238	206.1155	S	**1019.5520**	**510.2796**	1002.5255	501.7664	1001.5415	501.2744	9
5	516.2664	258.6368	498.2558	249.6316	S	**932.5200**	**466.7636**	915.4934	458.2504	914.5094	457.7584	8
6	617.3141	309.1607	599.3035	300.1554	T	**845.4880**	**423.2476**	828.4614	414.7343	827.4774	414.2423	7

7	716.3825	358.6949	698.3719	349.6896	V	744.4403	372.7238	727.4137	364.2105	726.4297	363.7185	6
8	817.4302	409.2187	799.4196	400.2134	T	645.3719	323.1896	628.3453	314.6763	627.3613	314.1843	5
9	954.4891	477.7482	936.4785	468.7429	H	544.3242	272.6657	527.2976	264.1525			4
10	1067.5732	534.2902	1049.5626	525.2849	L	407.2653	204.1363	390.2387	195.6230			3
11	1214.6416	607.8244	1196.6310	598.8191	F	294.1812	147.5942	277.1547	139.0810			2
12					K	147.1128	74.0600	130.0863	65.5468			1

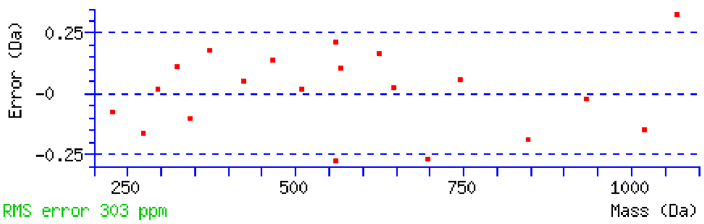

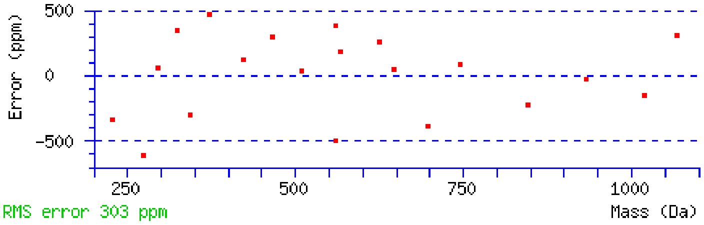

All matches to this query

Score	Mr(calc):	Delta	Sequence
46.1	1359.7398	0.0032	LLDSSTVTHLFK
12.9	1359.7527	-0.0097	ILDLLIPLTNR
11.2	1359.7371	0.0059	NLLSVGHQLSHR
9.6	1359.7398	0.0032	ILDVEIIFNER
9.6	1358.7445	0.9985	LLLYHTEETIK
9.3	1359.7358	0.0072	ARDTVTSELAAVK
8.9	1359.7299	0.0131	EPLHPATHALFK
8.3	1359.7319	0.0111	ILTMDGLIEDIK
7.6	1357.7231	2.0199	LLNKVSLGHAAR
5.0	1359.7371	0.0059	NLLSVGHQSLHR

Spectrum No: 10; Query: 52934; Rank: 1

Peptide View

MS/MS Fragmentation of **LYQVEYAFK**
Found in **IPI00029623**, Tax_Id=9606 Gene_Symbol=PSMA6 Proteasome subunit alpha type-6

Match to Query 52934: 1159.595948 from(580.805250,2+)
Title: 090702LimSK_Exosome2_04.6346.6346.2.dta
Data file C:\Temp\Mascoti\LSK_2D\090702LimSK_Exosome2_01_2D.mgf

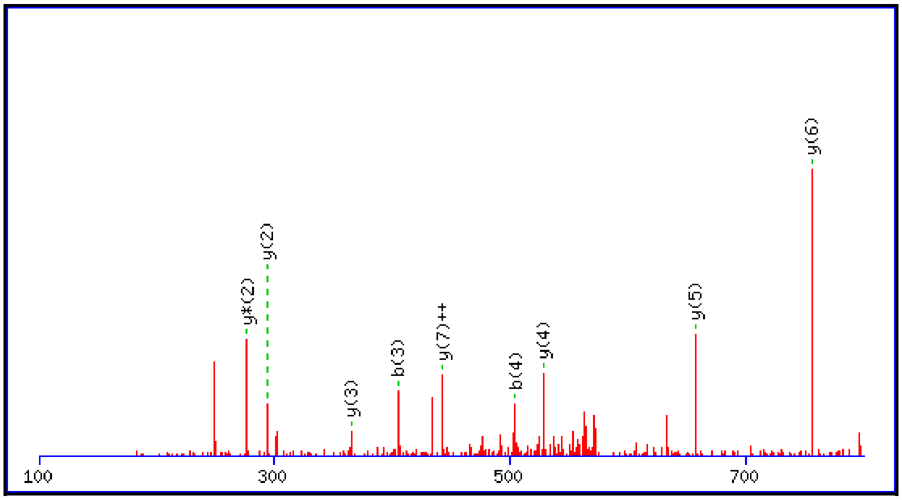

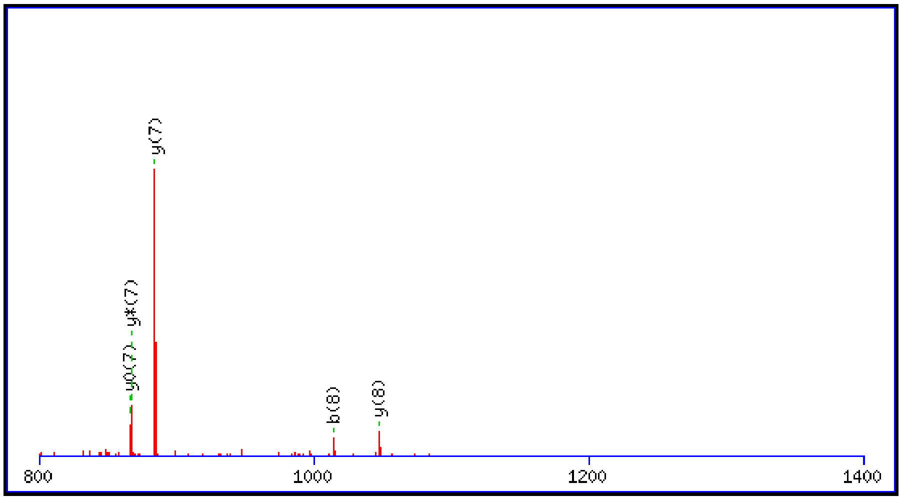

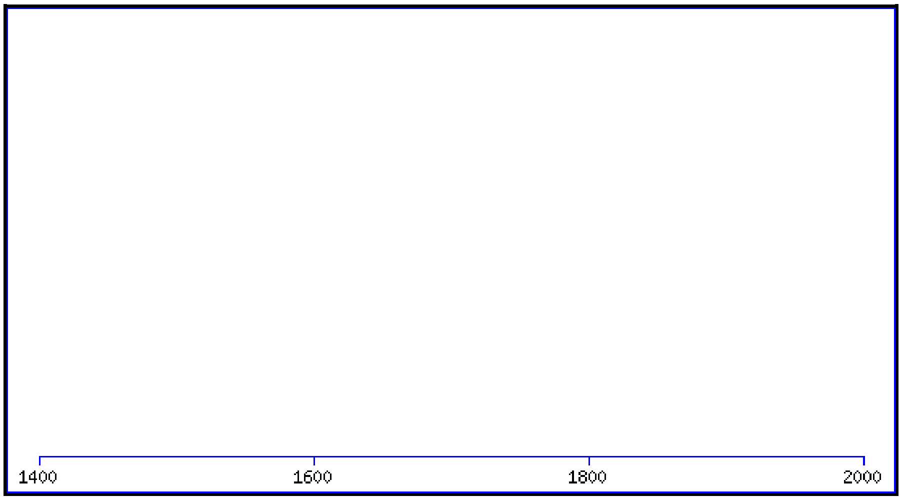

Monoisotopic mass of neutral peptide Mr(calc): 1159.5913
Fixed modifications: Carbamidomethyl (C)
Ions Score: 54 **Expect:** 0.00055
Matches (Bold Red): 16/76 fragment ions using 21 most intense peaks

#	b	b^{++}	b*	b^{*++}	b^{0}	b^{0++}	Seq.	y	y^{++}	y*	y^{*++}	y^{0}	y^{0++}	#
1	114.0913	57.5493					L							9
2	**277.1547**	139.0810					Y	**1047.5146**	524.2609	1030.4880	515.7477	1029.5040	515.2556	8
3	**405.2132**	203.1103	388.1867	194.5970			Q	**884.4512**	**442.7293**	**867.4247**	434.2160	**866.4407**	433.7240	7
4	**504.2817**	252.6445	487.2551	244.1312			V	**756.3927**	378.7000	739.3661	370.1867	738.3821	369.6947	6
5	633.3243	317.1658	616.2977	308.6525	615.3137	308.1605	E	**657.3243**	329.1658	640.2977	320.6525	639.3137	320.1605	5
6	796.3876	398.6974	779.3610	390.1842	778.3770	389.6921	Y	**528.2817**	264.6445	511.2551	256.1312			4
7	**867.4247**	434.2160	850.3981	425.7027	849.4141	425.2107	A	**365.2183**	183.1128	348.1918	174.5995			3
8	**1014.4931**	507.7502	997.4666	499.2369	996.4825	498.7449	F	**294.1812**	147.5942	**277.1547**	139.0810			2
9							K	147.1128	74.0600	130.0863	65.5468			1

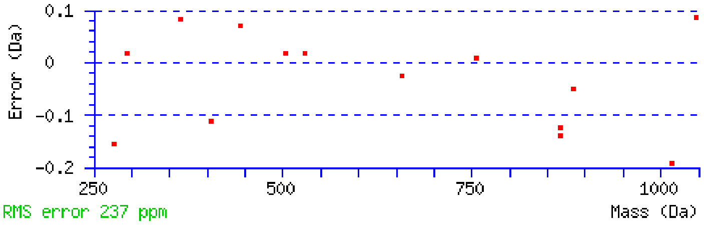

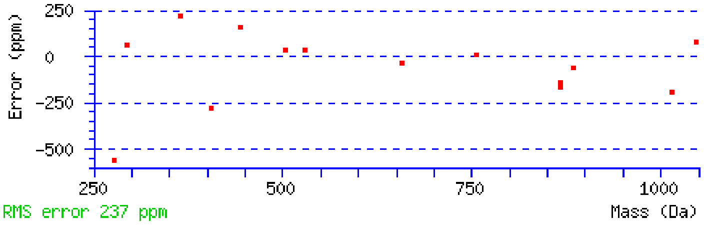

All matches to this query

Score	Mr(calc):	Delta	Sequence

54.1	1159.5913	0.0046	LYQVEYAFK
17.4	1159.5986	-0.0026	EFKAGGEVPAR
14.0	1159.5890	0.0070	LYKTLSEVK
12.7	1159.5890	0.0070	LYKTLSEVK
11.5	1158.6033	0.9926	XQQSLLQEK
10.6	1159.5873	0.0086	YIQAEPPTNK
8.4	1158.5928	1.0032	MGARASGGPLAR
7.9	1159.5972	-0.0013	LLELTADEEK
7.6	1158.5993	0.9967	ERLEAASQQK
6.7	1159.5985	-0.0026	ETLRAWQEK

Spectrum No: 11; Query: 53439; Rank: 1

Peptide View

MS/MS Fragmentation of **QTESTSFLEK**
Found in **IPI00029623**, Tax_Id=9606 Gene_Symbol=PSMA6 Proteasome subunit alpha type-6

Match to Query 53439: 1168.563148 from(585.288850,2+)
Title: 090702LimSK_Exosome2_05.2893.2893.2.dta
Data file C:\Temp\Mascoti\LSK_2D\090702LimSK_Exosome2_01_2D.mgf

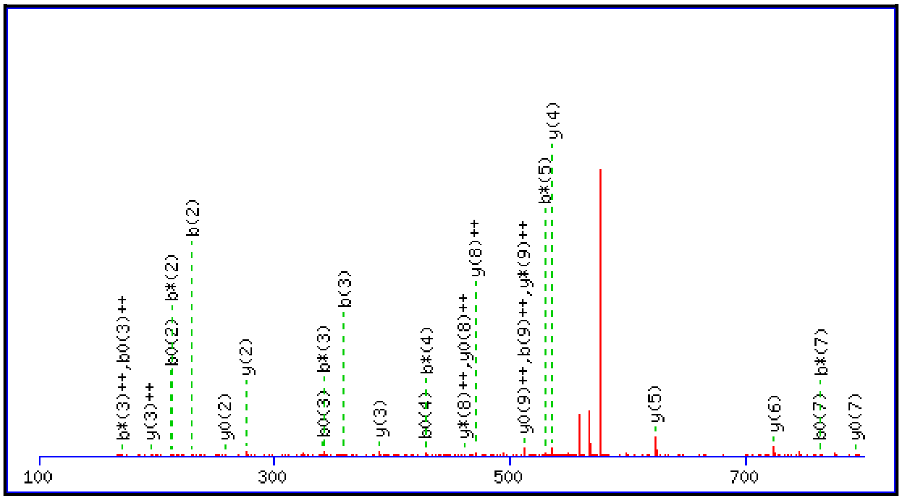

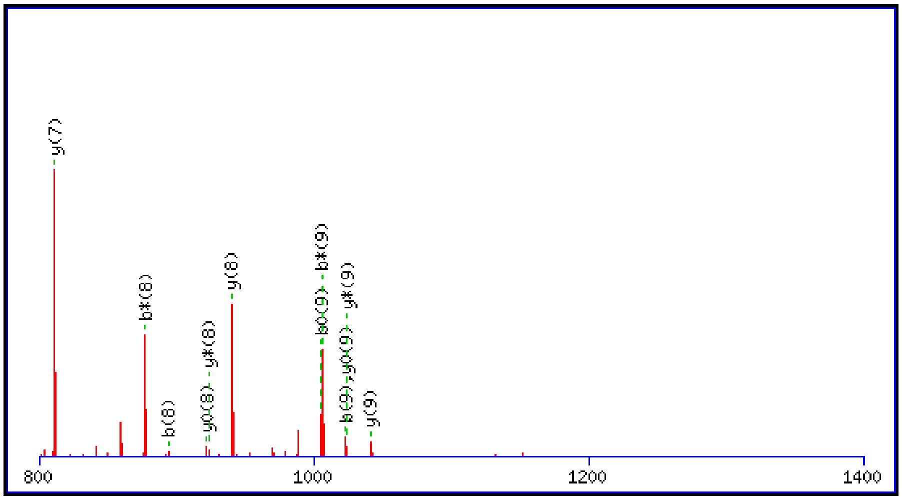

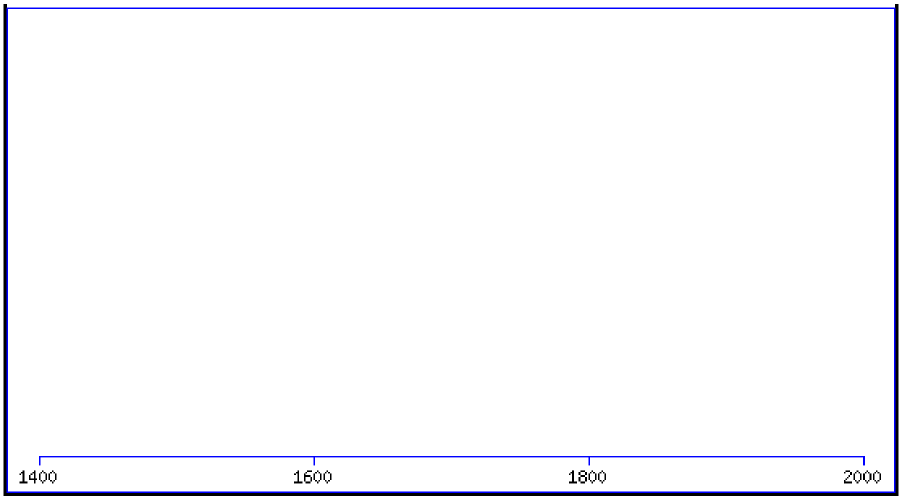

Monoisotopic mass of neutral peptide Mr(calc): 1168.5612
Fixed modifications: Carbamidomethyl (C)
Ions Score: 47 **Expect:** 0.0032
Matches (Bold Red): 39/104 fragment ions using 66 most intense peaks

#	b	b^{++}	b*	b^{*++}	b^{0}	b^{0++}	Seq.	y	y^{++}	y*	y^{*++}	y^{0}	y^{0++}	#
1	129.0659	65.0366	112.0393	56.5233			Q							10
2	**230.1135**	115.5604	**213.0870**	107.0471	**212.1030**	106.5551	T	**1041.5099**	521.2586	**1024.4833**	**512.7453**	**1023.4993**	**512.2533**	9
3	**359.1561**	180.0817	**342.1296**	**171.5684**	**341.1456**	**171.0764**	E	**940.4622**	**470.7347**	**923.4357**	**462.2215**	**922.4516**	**461.7295**	8
4	446.1882	223.5977	**429.1616**	215.0844	**428.1776**	214.5924	S	**811.4196**	406.2134	794.3931	397.7002	**793.4090**	397.2082	7
5	547.2358	274.1216	**530.2093**	265.6083	529.2253	265.1163	T	**724.3876**	362.6974	707.3610	354.1842	706.3770	353.6921	6
6	634.2679	317.6376	617.2413	309.1243	616.2573	308.6323	S	**623.3399**	312.1736	606.3134	303.6603	605.3293	303.1683	5
7	781.3363	391.1718	**764.3097**	382.6585	**763.3257**	382.1665	F	**536.3079**	268.6576	519.2813	260.1443	518.2973	259.6523	4
8	**894.4203**	447.7138	**877.3938**	439.2005	876.4098	438.7085	L	**389.2395**	**195.1234**	372.2129	186.6101	371.2289	186.1181	3
9	**1023.4629**	**512.2351**	**1006.4364**	503.7218	**1005.4524**	503.2298	E	**276.1554**	138.5813	259.1288	130.0681	**258.1448**	129.5761	2
10							K	147.1128	74.0600	130.0863	65.5468			1

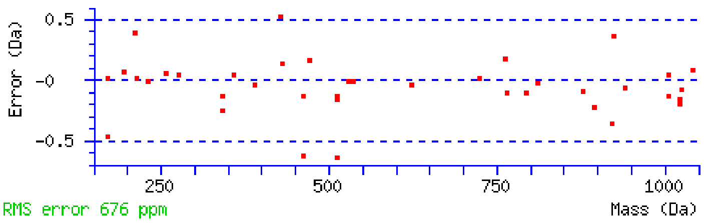

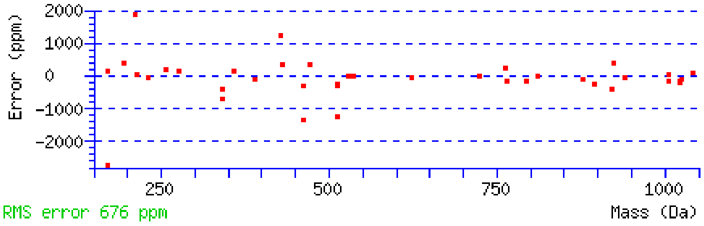

All matches to this query

Score	Mr(calc):	Delta	Sequence
47.4	1168.5612	0.0020	QTESTSFLEK
11.9	1166.5584	2.0048	VKEEASSPLK
10.8	1168.5740	-0.0109	KTEVAEALTK
10.2	1168.5741	-0.0109	EKTEGVVSIK
8.5	1167.5537	1.0095	SIGTGGIQDLK
8.1	1167.5594	1.0038	MPEEVHLGEK
8.1	1167.5634	0.9997	QFLPEEFMK
8.1	1168.5641	-0.0010	RLASSWLEK
8.0	1167.5594	1.0037	YQVQTICEK
7.6	1167.5537	1.0095	SDGTKKDPLK

Spectrum No: 12; Query: 60130; Rank: 1

Peptide View

MS/MS Fragmentation of **QTESTSFLEKK**
Found in **IPI00029623**, Tax_Id=9606 Gene_Symbol=PSMA6 Proteasome subunit alpha type-6

Match to Query 60130: 1296.658722 from(433.226850,3+)
Title: 090702LimSK_Exosome2_05.5284.5284.3.dta
Data file C:\Temp\Mascoti\LSK_2D\090702LimSK_Exosome2_01_2D.mgf

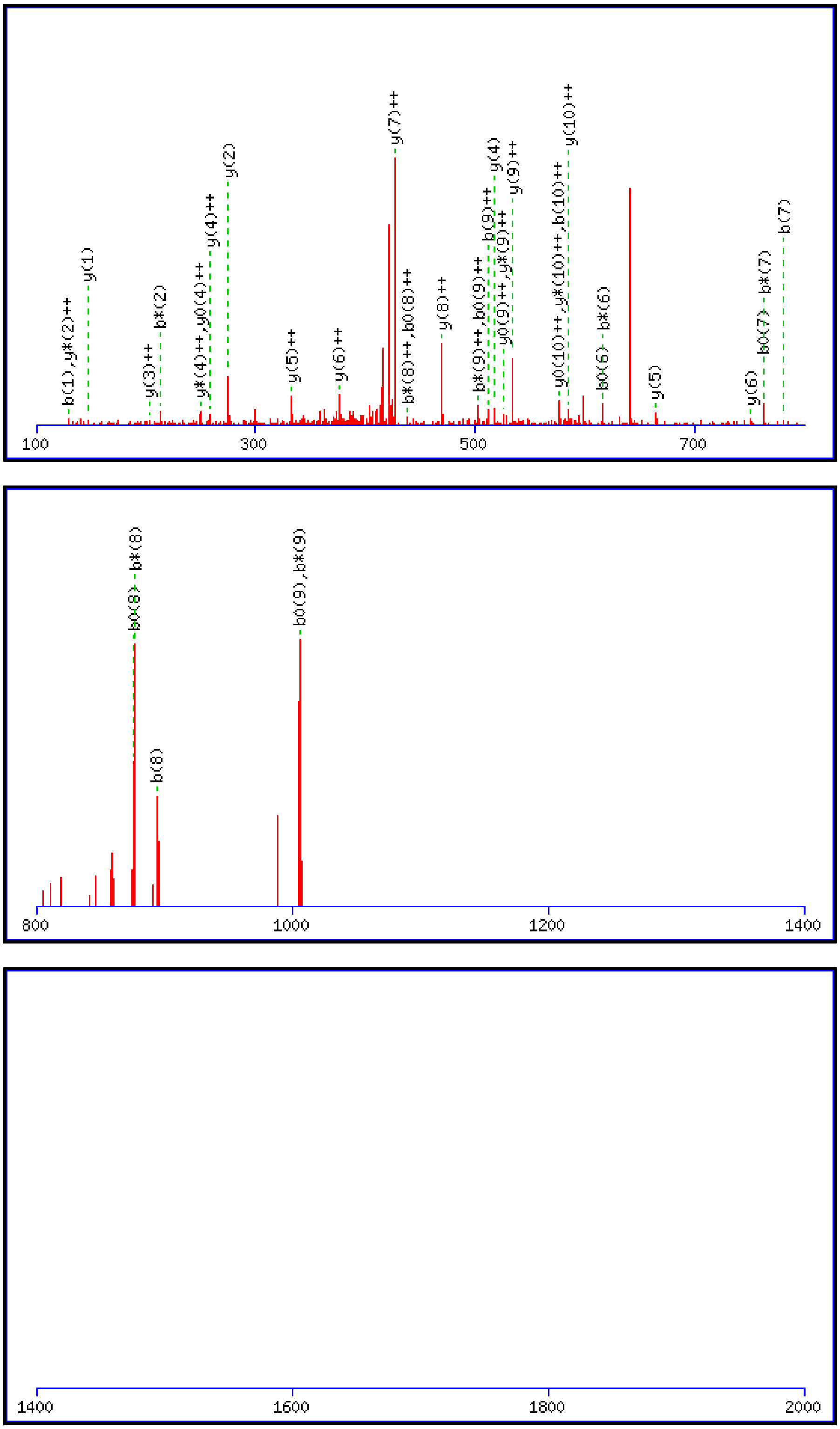

Monoisotopic mass of neutral peptide Mr(calc): 1296.6561
Fixed modifications: Carbamidomethyl (C)
Ions Score: 43 **Expect:** 0.0082

Matches (Bold Red): 38/114 fragment ions using 66 most intense peaks

#	b	b++	b*	b*++	b0	b0++	Seq.	y	y++	y*	y*++	y0	y0++	#
1	**129.0659**	65.0366	112.0393	56.5233			Q							11
2	230.1135	115.5604	**213.0870**	107.0471	212.1030	106.5551	T	1169.6048	**585.3061**	1152.5783	**576.7928**	1151.5943	**576.3008**	10
3	359.1561	180.0817	342.1296	171.5684	341.1456	171.0764	E	1068.5572	**534.7822**	1051.5306	**526.2689**	1050.5466	**525.7769**	9
4	446.1882	223.5977	429.1616	215.0844	428.1776	214.5924	S	939.5146	**470.2609**	922.4880	461.7477	921.5040	461.2556	8
5	547.2358	274.1216	530.2093	265.6083	529.2253	265.1163	T	852.4825	**426.7449**	835.4560	418.2316	834.4720	417.7396	7
6	634.2679	317.6376	**617.2413**	309.1243	**616.2573**	308.6323	S	**751.4349**	**376.2211**	734.4083	367.7078	733.4243	367.2158	6
7	**781.3363**	391.1718	**764.3097**	382.6585	**763.3257**	382.1665	F	**664.4028**	**332.7051**	647.3763	324.1918	646.3923	323.6998	5
8	**894.4203**	447.7138	**877.3938**	**439.2005**	**876.4098**	**438.7085**	L	**517.3344**	**259.1709**	500.3079	**250.6576**	499.3239	**250.1656**	4
9	1023.4629	**512.2351**	**1006.4364**	**503.7218**	**1005.4524**	**503.2298**	E	404.2504	**202.6288**	387.2238	194.1155	386.2398	193.6235	3
10	1151.5579	**576.2826**	1134.5313	567.7693	1133.5473	567.2773	K	**275.2078**	138.1075	258.1812	**129.5942**			2
11							K	**147.1128**	74.0600	130.0863	65.5468			1

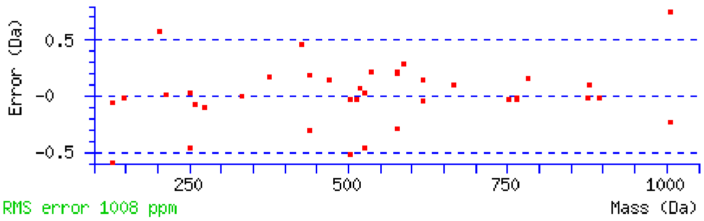

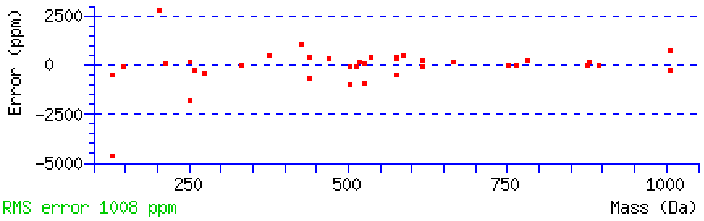

All matches to this query

Score	Mr(calc):	Delta	Sequence
43.1	1296.6561	0.0026	QTESTSFLEKK
13.5	1296.6479	0.0108	SKQELPPPPPK
8.7	1294.6507	2.0081	KQTNQRIATR
8.0	1296.6625	-0.0037	MQSSLLAKAIR
7.5	1295.6543	1.0044	EKYMIGNSVQK
7.3	1295.6486	1.0101	KQLTDNLKEK
6.9	1296.6496	0.0091	SSVSRVFDCLK
6.6	1295.6577	1.0010	AMVTETMTKLR
6.4	1295.6427	1.0160	KFFSRLYQK
6.2	1295.6599	0.9989	GSIKTVAGSGIAR

Spectrum No: 13; Query: 68569; Rank: 1

Peptide View

MS/MS Fragmentation of **YGYEIPVDMLCK**
Found in **IPI00029623**, Tax_Id=9606 Gene_Symbol=PSMA6 Proteasome subunit alpha type-6

Match to Query 68569: 1486.686348 from(744.350450,2+)
Title: 090702LimSK_Exosome2_06.8199.8199.2.dta
Data file C:\Temp\Mascoti\LSK_2D\090702LimSK_Exosome2_01_2D.mgf

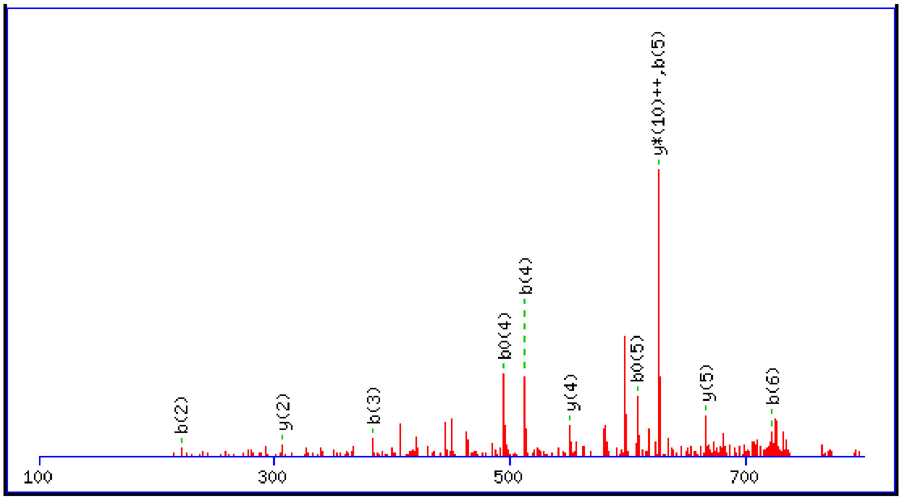

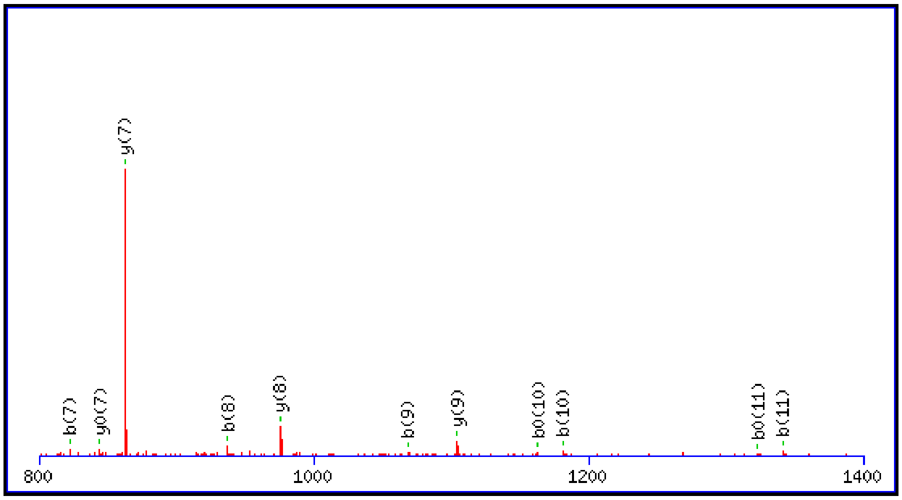

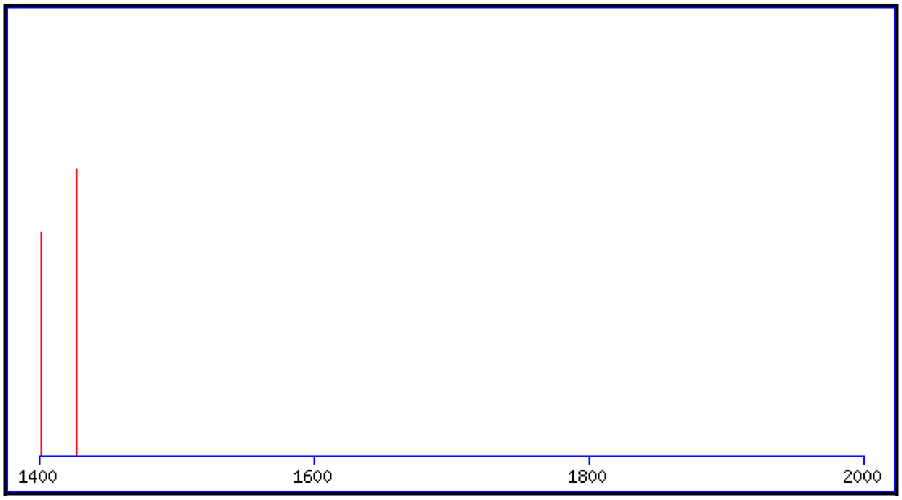

Monoisotopic mass of neutral peptide Mr(calc): 1486.6836
Fixed modifications: Carbamidomethyl (C)
Ions Score: 46 **Expect:** 0.005
Matches (Bold Red): 22/96 fragment ions using 64 most intense peaks

#	b	b^{++}	b^{0}	b^{0++}	Seq.	y	y^{++}	y*	y^{*++}	y^{0}	y^{0++}	#
1	164.0706	82.5389			Y							12
2	**221.0921**	111.0497			G	1324.6276	662.8174	1307.6010	654.3042	1306.6170	653.8121	11
3	**384.1554**	192.5813			Y	1267.6061	634.3067	1250.5796	**625.7934**	1249.5955	625.3014	10
4	**513.1980**	257.1026	**495.1874**	248.0974	E	**1104.5428**	552.7750	1087.5162	544.2618	1086.5322	543.7697	9
5	**626.2821**	313.6447	**608.2715**	304.6394	I	**975.5002**	488.2537	958.4736	479.7405	957.4896	479.2484	8
6	**723.3348**	362.1710	705.3243	353.1658	P	**862.4161**	431.7117	845.3896	423.1984	**844.4056**	422.7064	7

7	822.4032	411.7053	804.3927	402.7000	V	765.3634	383.1853	748.3368	374.6720	747.3528	374.1800	6
8	937.4302	469.2187	919.4196	460.2134	D	666.2949	333.6511	649.2684	325.1378	648.2844	324.6458	5
9	1068.4707	534.7390	1050.4601	525.7337	M	551.2680	276.1376	534.2415	267.6244			4
10	1181.5547	591.2810	1163.5442	582.2757	L	420.2275	210.6174	403.2010	202.1041			3
11	1341.5854	671.2963	1323.5748	662.2910	C	307.1435	154.0754	290.1169	145.5621			2
12					K	147.1128	74.0600	130.0863	65.5468			1

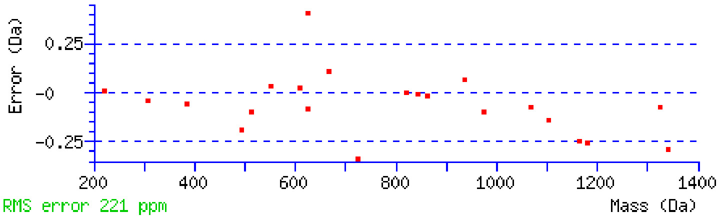

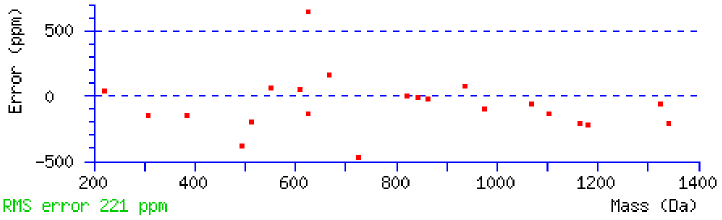

All matches to this query

Score	Mr(calc):	Delta	Sequence
46.1	1486.6836	0.0027	YGYEIPVDMLCK
7.0	1485.6877	0.9987	ELDMKTKMMEAK
7.0	1486.6973	-0.0110	VAEVLNDPENMEK
4.4	1486.6925	-0.0061	LLKVGDSLCQMK
2.8	1484.6701	2.0163	SFSFSHTIPPSAK
2.8	1484.6701	2.0163	SFSFSHTIPPSAK
2.8	1484.6701	2.0163	SFSFSHTIPPSAK
2.1	1486.6738	0.0125	ASSAKSAEMPTISK
1.7	1485.6898	0.9965	SMPSLDLSGRLSK
1.3	1486.6929	-0.0066	ARGPQPSPPGSSAAK

Spectrum No: 14; Query: 48102; Rank: 1

Peptide View

MS/MS Fragmentation of **ALLEVVQSGGK**
Found in **IPI00024175**, Tax_Id=9606 Gene_Symbol=PSMA7 Isoform 1 of Proteasome subunit alpha type-7

Match to Query 48102: 1099.625928 from(550.820240,2+)
Title: 090702LimSK_Exosome2_05.4658.4658.2.dta
Data file C:\Temp\Mascoti\LSK_2D\090702LimSK_Exosome2_01_2D.mgf

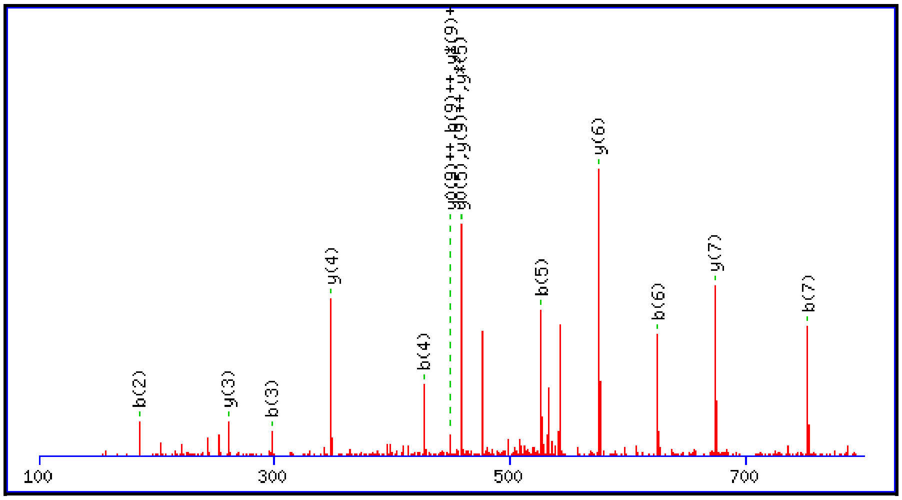

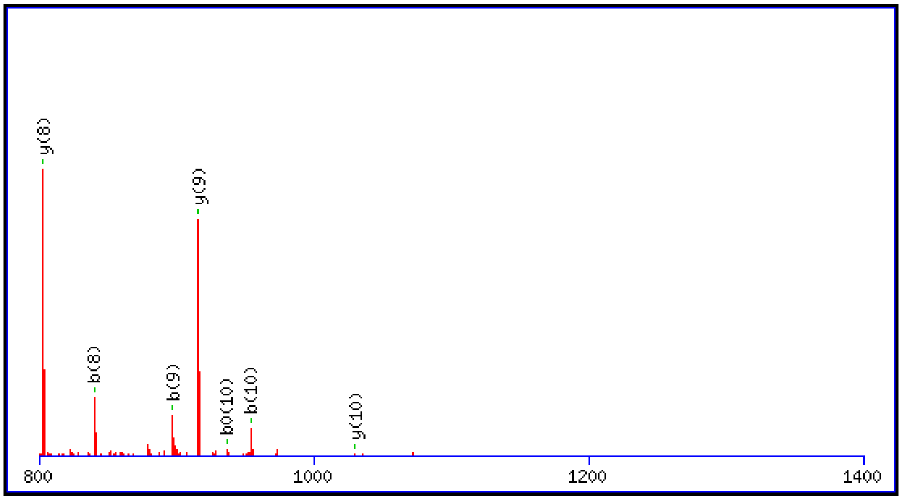

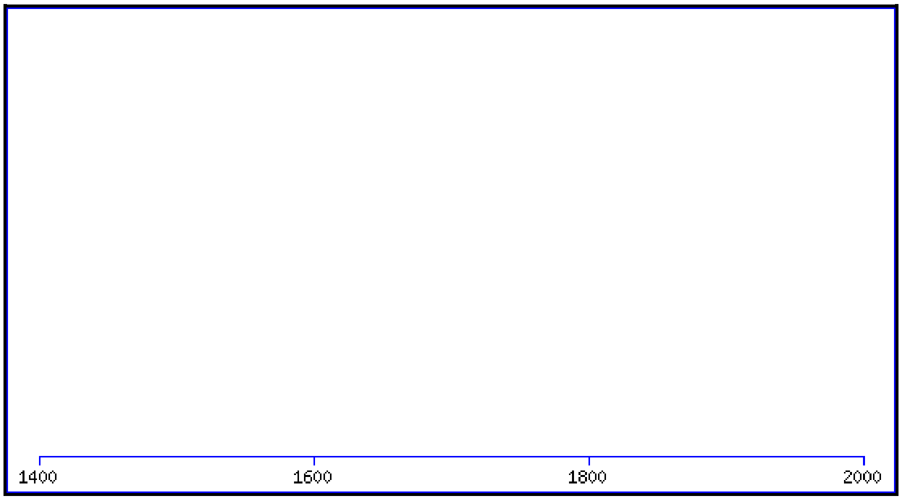

Monoisotopic mass of neutral peptide Mr(calc): 1099.6237
Fixed modifications: Carbamidomethyl (C)
Ions Score: 75 **Expect:** 2.7e-006
Matches (Bold Red): 23/96 fragment ions using 31 most intense peaks

#	b	b^{++}	b*	b^{*++}	b^{0}	b^{0++}	Seq.	y	y^{++}	y*	y^{*++}	y^{0}	y^{0++}	#
1	72.0444	36.5258					A							11
2	**185.1285**	93.0679					L	**1029.5939**	515.3006	1012.5673	506.7873	1011.5833	506.2953	10
3	**298.2125**	149.6099					L	**916.5098**	**458.7585**	899.4833	**450.2453**	898.4993	**449.7533**	9
4	**427.2551**	214.1312			409.2445	205.1259	E	**803.4258**	402.2165	786.3992	393.7032	785.4152	393.2112	8
5	**526.3235**	263.6654			508.3130	254.6601	V	**674.3832**	337.6952	657.3566	329.1819	656.3726	328.6899	7
6	**625.3919**	313.1996			607.3814	304.1943	V	**575.3148**	288.1610	558.2882	279.6477	557.3042	279.1557	6
7	**753.4505**	377.2289	736.4240	368.7156	735.4400	368.2236	Q	476.2463	238.6268	**459.2198**	230.1135	**458.2358**	229.6215	5
8	**840.4825**	420.7449	823.4560	412.2316	822.4720	411.7396	S	**348.1878**	174.5975	331.1612	166.0842	330.1772	165.5922	4
9	**897.5040**	**449.2556**	880.4775	440.7424	879.4934	440.2504	G	**261.1557**	131.0815	244.1292	122.5682			3
10	**954.5255**	477.7664	937.4989	469.2531	**936.5149**	468.7611	G	204.1343	102.5708	187.1077	94.0575			2
11							K	147.1128	74.0600	130.0863	65.5468			1

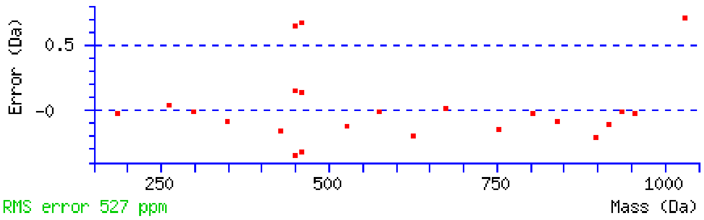

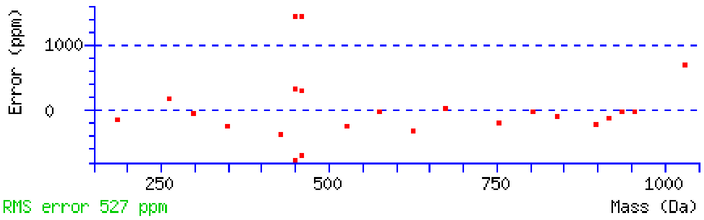

All matches to this query

Score	Mr(calc):	Delta	Sequence
75.5	1099.6237	0.0022	ALLEVVQSGGK
19.1	1099.6349	-0.0090	LALEKERNK
9.7	1099.6237	0.0022	LAPVITGTNSK
9.5	1099.6237	0.0022	AIPKTLKDSQ
8.0	1099.6237	0.0022	ALQVLVDQSK
6.0	1099.6349	-0.0090	ITRGIEAVGGK
4.5	1099.6349	-0.0090	RGTNEIVAIK
4.0	1099.6349	-0.0090	SPVKRGLESK
2.8	1099.6237	0.0022	KTLDTGAIPGK
2.3	1099.6349	-0.0090	VKNGAASPTKK

Spectrum No: 15; Query: 51319; Rank: 1

Peptide View

MS/MS Fragmentation of **ARVECQSHR**
Found in **IPI00024175**, Tax_Id=9606 Gene_Symbol=PSMA7 Isoform 1 of Proteasome subunit alpha type-7

Match to Query 51319: 1141.543482 from(381.521770,3+)
Title: 090702LimSK_Exosome2_02.226.226.3.dta
Data file C:\Temp\Mascoti\LSK_2D\090702LimSK_Exosome2_01_2D.mgf

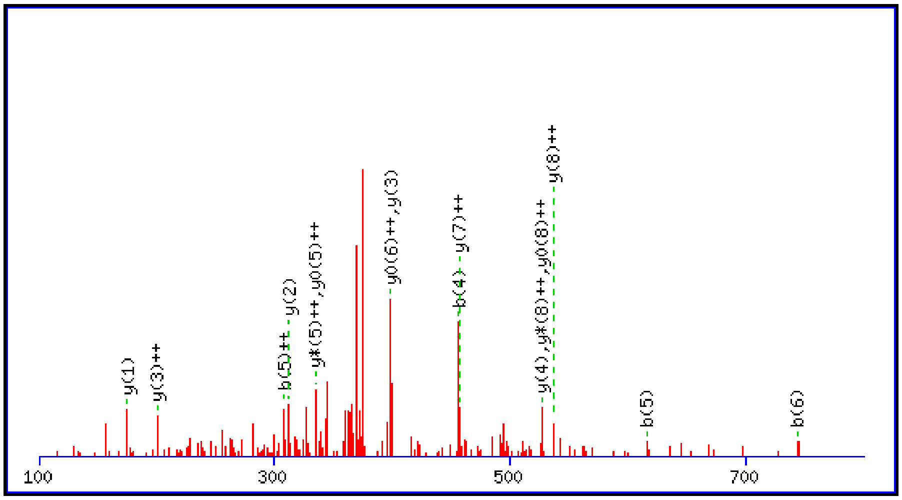

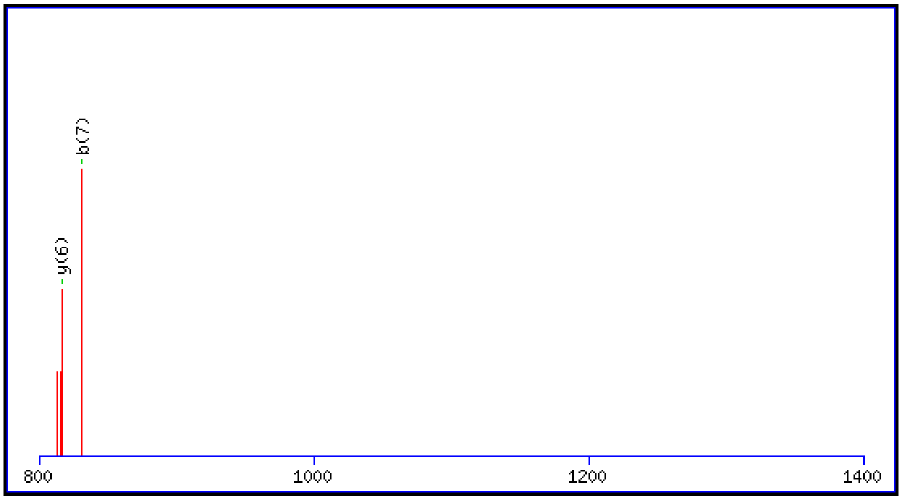

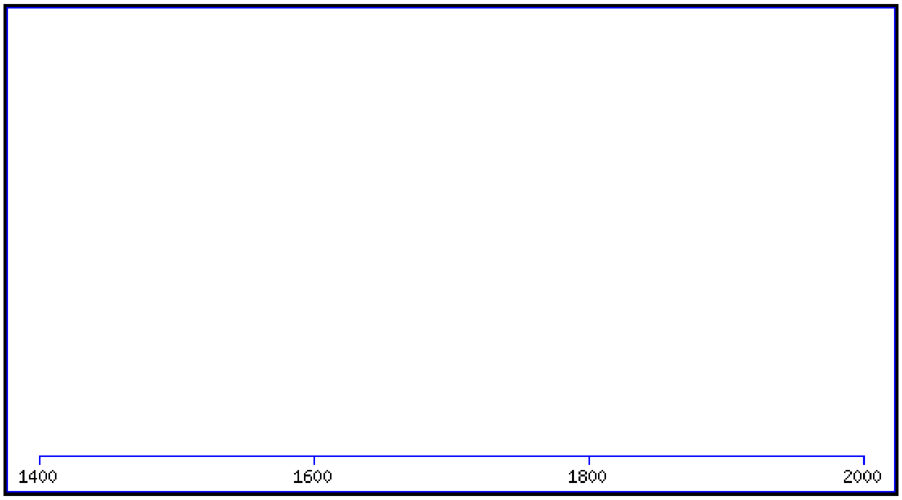

Monoisotopic mass of neutral peptide Mr(calc): 1141.5411
Fixed modifications: Carbamidomethyl (C)
Ions Score: 32 **Expect:** 0.097
Matches (Bold Red): 18/84 fragment ions using 20 most intense peaks

#	b	b^{++}	b*	b^{*++}	b^{0}	b^{0++}	Seq.	y	y^{++}	y*	y^{*++}	y^{0}	y^{0++}	#
1	72.0444	36.5258					A							9
2	228.1455	114.5764	211.1190	106.0631			R	1071.5112	**536.2593**	1054.4847	**527.7460**	1053.5007	**527.2540**	8
3	327.2139	164.1106	310.1874	155.5973			V	915.4101	**458.2087**	898.3836	449.6954	897.3996	449.2034	7
4	**456.2565**	228.6319	439.2300	220.1186	438.2459	219.6266	E	**816.3417**	408.6745	799.3152	400.1612	798.3311	**399.6692**	6
5	**616.2872**	**308.6472**	599.2606	300.1339	598.2766	299.6419	C	687.2991	344.1532	670.2726	**335.6399**	669.2886	**335.1479**	5
6	**744.3457**	372.6765	727.3192	364.1632	726.3352	363.6712	Q	**527.2685**	264.1379	510.2419	255.6246	509.2579	255.1326	4
7	**831.3778**	416.1925	814.3512	407.6792	813.3672	407.1872	S	**399.2099**	**200.1086**	382.1833	191.5953	381.1993	191.1033	3
8	968.4367	484.7220	951.4101	476.2087	950.4261	475.7167	H	**312.1779**	156.5926	295.1513	148.0793			2
9							R	**175.1190**	88.0631	158.0924	79.5498			1

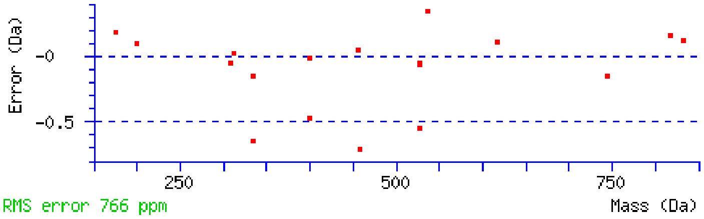

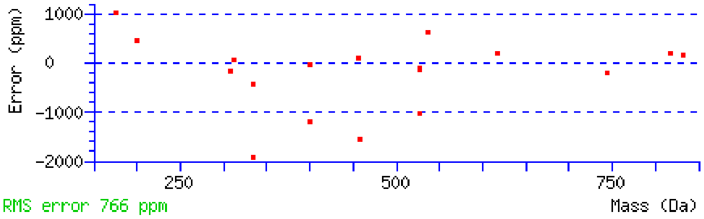

All matches to this query

Score	Mr(calc):	Delta	Sequence
32.1	1141.5411	0.0024	ARVECQSHR
10.9	1139.5270	2.0164	MPASQRSKR
10.5	1140.5362	1.0072	GVAMVTAVAAR
10.5	1139.5410	2.0025	TAMAAAKAPTK
10.4	1139.5376	2.0059	QVVESFVPR
9.2	1140.5329	1.0106	NIFTPEGKR
8.8	1141.5533	-0.0098	YGLLPSGSLR
8.7	1140.5475	0.9960	MVSVKREGR
8.4	1141.5427	0.0008	CRVIKSSGR
8.3	1140.5414	1.0021	ARAHAHSRR

Spectrum No: 16; Query: 41485; Rank: 1

Peptide View

MS/MS Fragmentation of **DIVVLGVEK**
Found in **IPI00024175**, Tax_Id=9606 Gene_Symbol=PSMA7 Isoform 1 of Proteasome subunit alpha type-7

Match to Query 41485: 970.572228 from(486.293390,2+)
Title: 090702LimSK_Exosome2_05.4828.4828.2.dta
Data file C:\Temp\Mascoti\LSK_2D\090702LimSK_Exosome2_01_2D.mgf

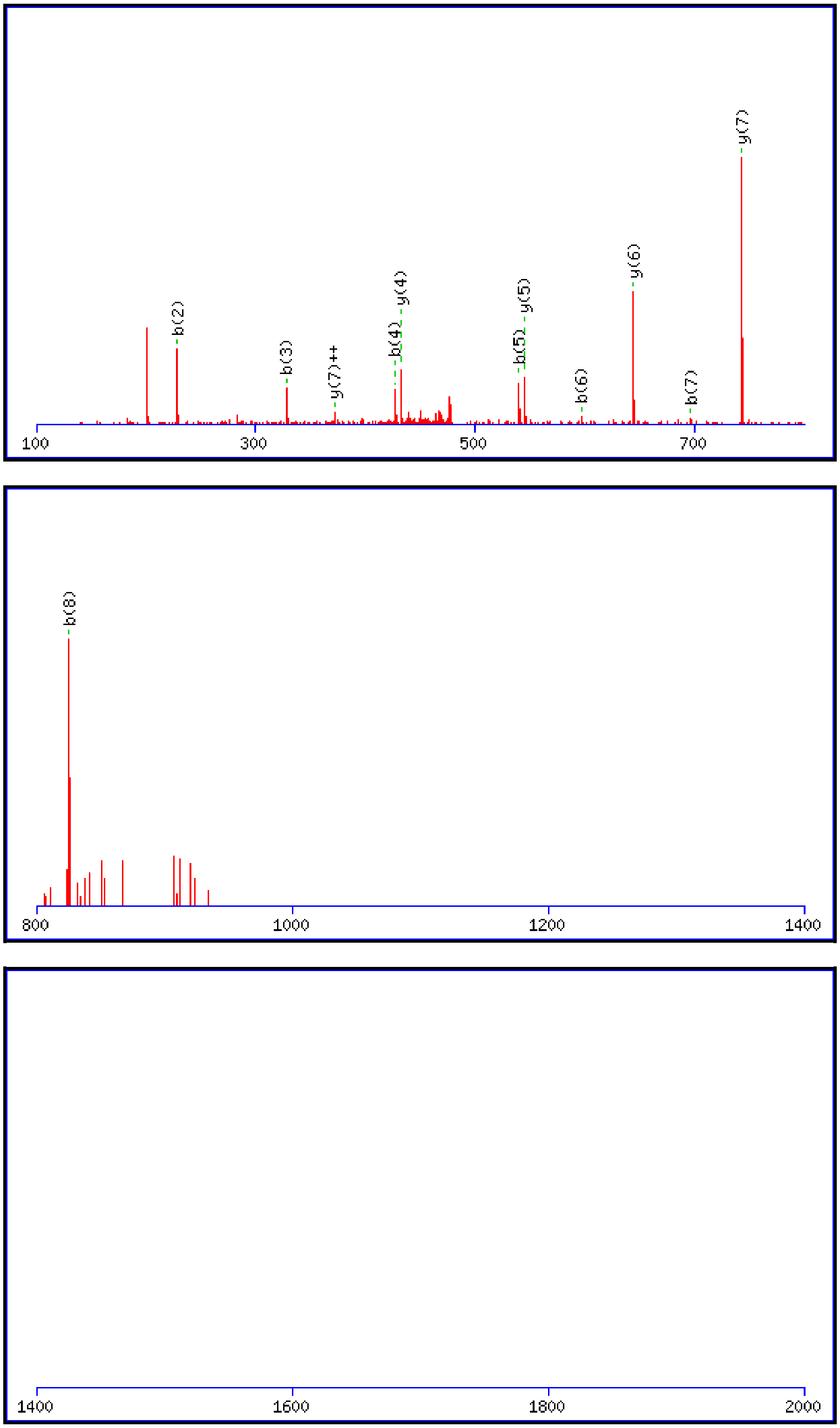

Monoisotopic mass of neutral peptide Mr(calc): 970.5699
Fixed modifications: Carbamidomethyl (C)
Ions Score: 50 **Expect:** 0.00073
Matches (Bold Red): 12/78 fragment ions using 26 most intense peaks

#	b	b^{++}	b^{0}	b^{0++}	Seq.	y	y^{++}	y^{*}	y^{*++}	y^{0}	y^{0++}	#
1	116.0342	58.5207	98.0237	49.5155	D							9
2	**229.1183**	115.0628	211.1077	106.0575	I	856.5502	428.7788	839.5237	420.2655	838.5397	419.7735	8
3	**328.1867**	164.5970	310.1761	155.5917	V	**743.4662**	**372.2367**	726.4396	363.7234	725.4556	363.2314	7
4	**427.2551**	214.1312	409.2445	205.1259	V	**644.3978**	322.7025	627.3712	314.1892	626.3872	313.6972	6
5	**540.3392**	270.6732	522.3286	261.6679	L	**545.3293**	273.1683	528.3028	264.6550	527.3188	264.1630	5
6	**597.3606**	299.1840	579.3501	290.1787	G	**432.2453**	216.6263	415.2187	208.1130	414.2347	207.6210	4
7	**696.4291**	348.7182	678.4185	339.7129	V	375.2238	188.1155	358.1973	179.6023	357.2132	179.1103	3
8	**825.4716**	413.2395	807.4611	404.2342	E	276.1554	138.5813	259.1288	130.0681	258.1448	129.5761	2
9					K	147.1128	74.0600	130.0863	65.5468			1

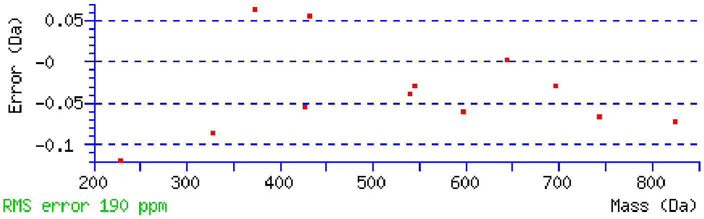

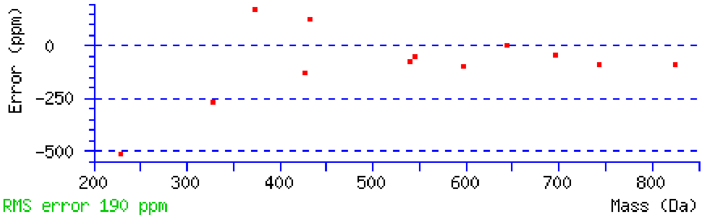

All matches to this query

Score	Mr(calc):	Delta	Sequence
49.8	970.5699	0.0023	DIVVLGVEK
9.5	970.5699	0.0024	IAELLGLDK
9.3	970.5672	0.0050	RNVGIREK
7.2	970.5811	-0.0089	SLLLLEQR
7.2	970.5634	0.0089	TVLLLPCR
6.7	970.5699	0.0023	IDVGDVILK
5.7	970.5811	-0.0089	ITLLVQER
5.1	970.5811	-0.0089	DVVILGRSL
2.9	969.5720	1.0003	QVVIGGRNK
2.6	970.5811	-0.0089	EKAKTPVAK

Spectrum No: 17; Query: 48059; Rank: 1

Peptide View

MS/MS Fragmentation of **DIVVLGVEKK**
Found in **IPI00024175**, Tax_Id=9606 Gene_Symbol=PSMA7 Isoform 1 of Proteasome subunit alpha type-7

Match to Query 48059: 1098.666348 from(550.340450,2+)
Title: 090702LimSK_Exosome2_05.6721.6721.2.dta
Data file C:\Temp\Mascoti\LSK_2D\090702LimSK_Exosome2_01_2D.mgf

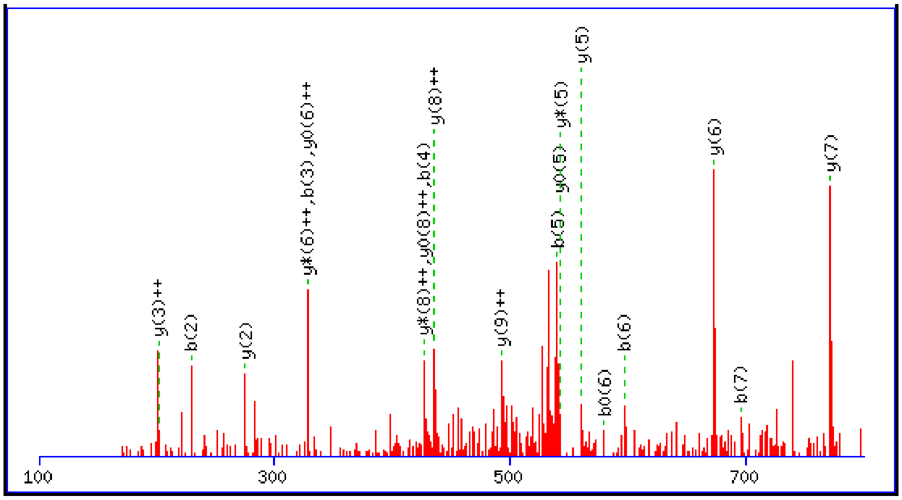

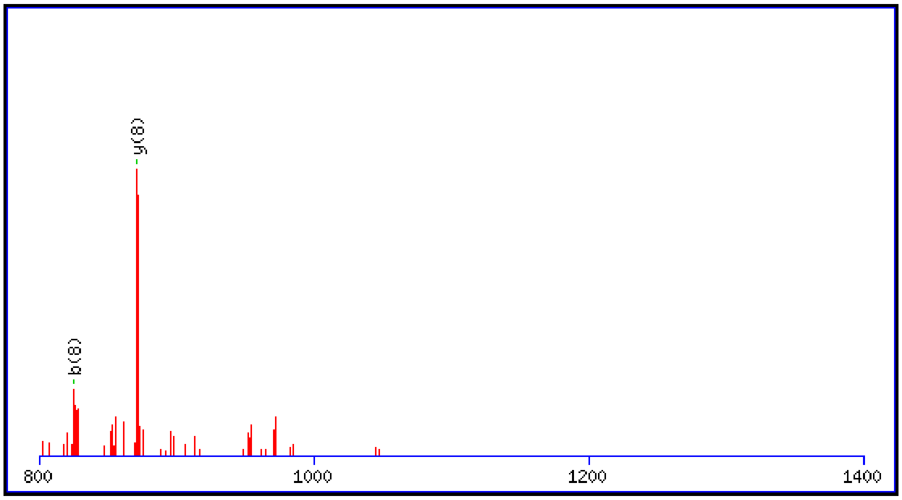

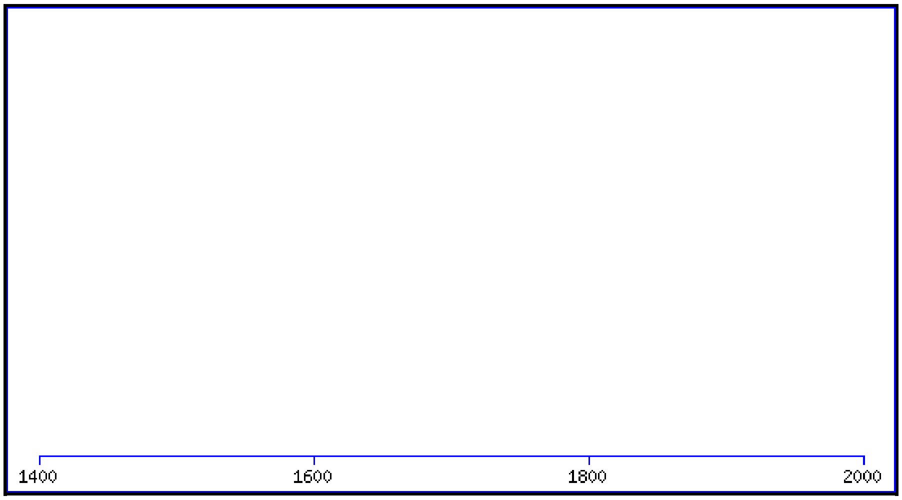

Monoisotopic mass of neutral peptide Mr(calc): 1098.6649
Fixed modifications: Carbamidomethyl (C)
Ions Score: 33 **Expect:** 0.024
Matches (Bold Red): 22/88 fragment ions using 51 most intense peaks

#	b	b^{++}	b*	b^{*++}	b^0	b^{0++}	Seq.	y	y^{++}	y*	y^{*++}	y^0	y^{0++}	#
1	116.0342	58.5207			98.0237	49.5155	D							10
2	**229.1183**	115.0628			211.1077	106.0575	I	984.6452	**492.8262**	967.6186	484.3130	966.6346	483.8210	9
3	**328.1867**	164.5970			310.1761	155.5917	V	**871.5611**	**436.2842**	854.5346	**427.7709**	853.5506	**427.2789**	8
4	**427.2551**	214.1312			409.2445	205.1259	V	**772.4927**	386.7500	755.4662	378.2367	754.4822	377.7447	7
5	**540.3392**	270.6732			522.3286	261.6679	L	**673.4243**	337.2158	656.3978	**328.7025**	655.4137	**328.2105**	6
6	**597.3606**	299.1840			**579.3501**	290.1787	G	**560.3402**	280.6738	**543.3137**	272.1605	**542.3297**	271.6685	5

**7**	**696.4291**	348.7182			678.4185	339.7129	**V**	503.3188	252.1630	486.2922	243.6498	485.3082	243.1577	**4**
**8**	**825.4716**	413.2395			807.4611	404.2342	**E**	404.2504	**202.6288**	387.2238	194.1155	386.2398	193.6235	**3**
**9**	953.5666	477.2869	936.5401	468.7737	935.5560	468.2817	**K**	**275.2078**	138.1075	258.1812	129.5942			**2**
**10**							**K**	147.1128	74.0600	130.0863	65.5468			**1**

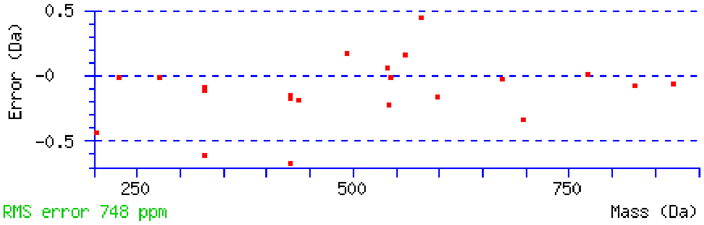

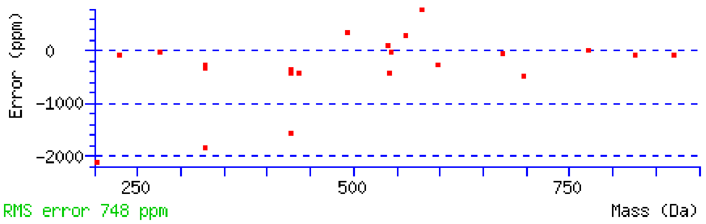

All matches to this query

Score	Mr(calc):	Delta	Sequence
32.5	1098.6649	0.0015	DIVVLGVEKK
15.8	1098.6761	-0.0097	DLVVKGSLLR
5.7	1098.6760	-0.0097	AEQKIAAIKK
1.6	1097.6669	0.9995	LNDLIKRAR

Spectrum No: 18; Query: 54341; Rank: 1

Peptide View

MS/MS Fragmentation of **GRDIVVLGVEK**
Found in **IPI00024175**, Tax_Id=9606 Gene_Symbol=PSMA7 Isoform 1 of Proteasome subunit alpha type-7

Match to Query 54341: 1183.695312 from(395.572380,3+)
Title: 090702LimSK_Exosome2_05.8433.8433.3.dta
Data file C:\Temp\Mascoti\LSK_2D\090702LimSK_Exosome2_01_2D.mgf

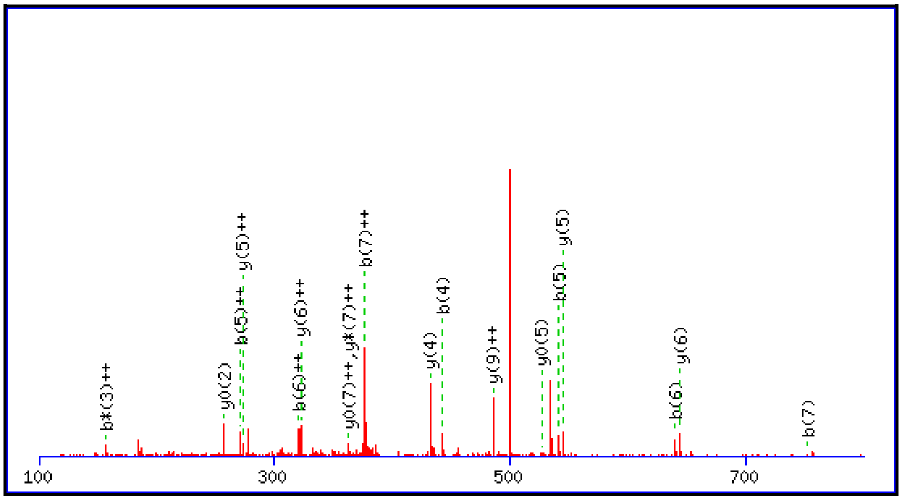

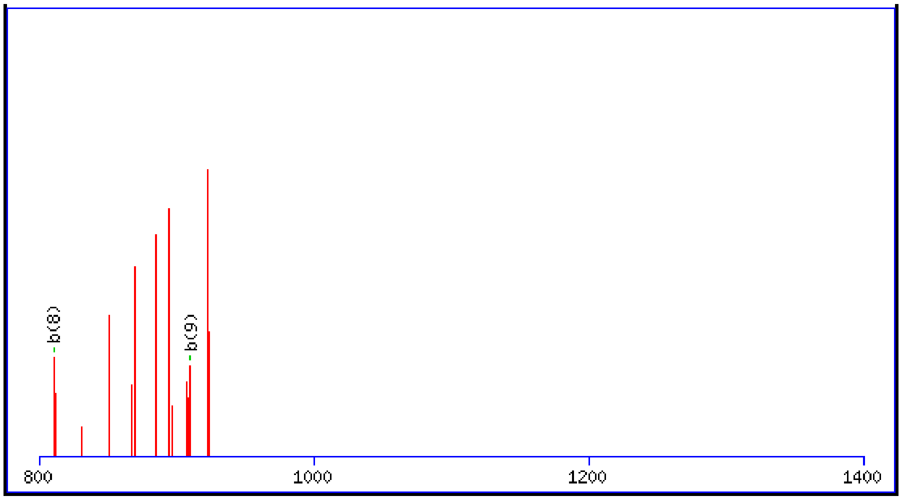

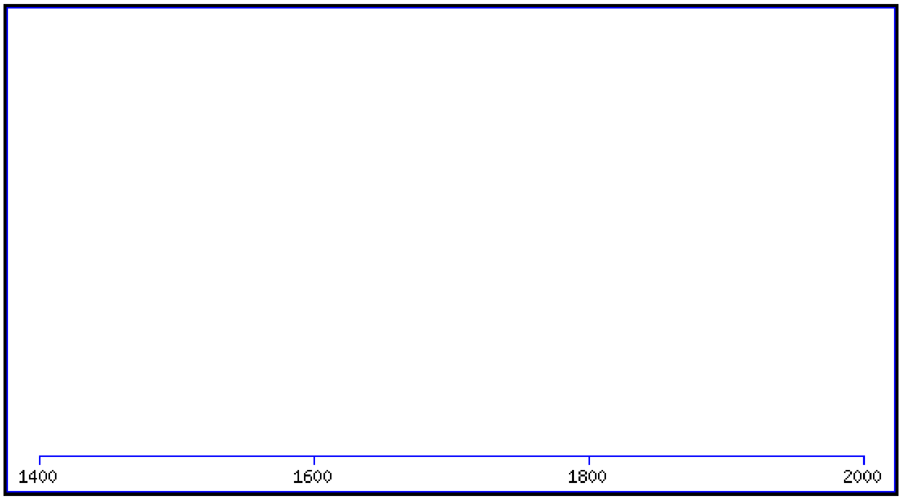

Monoisotopic mass of neutral peptide Mr(calc): 1183.6925
Fixed modifications: Carbamidomethyl (C)
Ions Score: 22 **Expect:** 0.37
Matches (Bold Red): 20/112 fragment ions using 51 most intense peaks

#	b	b++	b*	b*++	b0	b0++	Seq.	y	y++	y*	y*++	y0	y0++	#
1	58.0287	29.5180					G							11
2	214.1299	107.5686	197.1033	99.0553			R	1127.6783	564.3428	1110.6517	555.8295	1109.6677	555.3375	10
3	329.1568	165.0820	312.1302	**156.5688**	311.1462	156.0768	D	971.5772	**486.2922**	954.5506	477.7790	953.5666	477.2869	9
4	**442.2409**	221.6241	425.2143	213.1108	424.2303	212.6188	I	856.5502	428.7788	839.5237	420.2655	838.5397	419.7735	8
5	**541.3093**	**271.1583**	524.2827	262.6450	523.2987	262.1530	V	743.4662	372.2367	726.4396	**363.7234**	725.4556	**363.2314**	7
6	**640.3777**	**320.6925**	623.3511	312.1792	622.3671	311.6872	V	**644.3978**	**322.7025**	627.3712	314.1892	626.3872	313.6972	6
7	**753.4618**	**377.2345**	736.4352	368.7212	735.4512	368.2292	L	**545.3293**	**273.1683**	528.3028	264.6550	**527.3188**	264.1630	5
8	**810.4832**	405.7452	793.4567	397.2320	792.4726	396.7400	G	**432.2453**	216.6263	415.2187	208.1130	414.2347	207.6210	4
9	**909.5516**	455.2795	892.5251	446.7662	891.5411	446.2742	V	375.2238	188.1155	358.1973	179.6023	357.2132	179.1103	3
10	1038.5942	519.8007	1021.5677	511.2875	1020.5837	510.7955	E	276.1554	138.5813	259.1288	130.0681	**258.1448**	129.5761	2
11							K	147.1128	74.0600	130.0863	65.5468			1

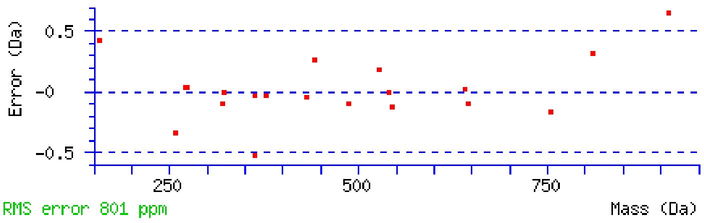

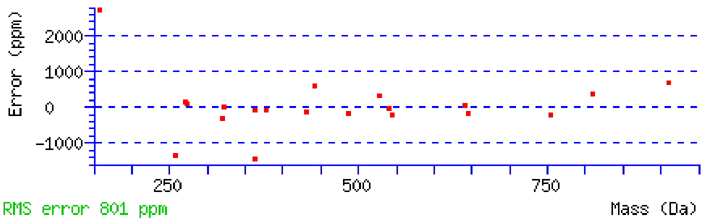

All matches to this query

Score	Mr(calc):	Delta	Sequence
21.5	1183.6925	0.0028	GRDIVVLGVEK
20.5	1183.6859	0.0094	MGRSLVGLPVR
9.7	1181.6768	2.0185	LADLEQRLPK
9.0	1183.7037	-0.0084	KVAENVIRQK
8.7	1182.6833	1.0120	GQLLEVGRGVR
7.7	1181.6768	2.0185	ALISPGTLSAPR
7.7	1181.6992	1.9961	AINAALAQRVR
5.8	1183.7037	-0.0084	QQGDLVRKLK
4.9	1182.6833	1.0120	XSSGSIAIRKK
2.1	1181.6768	2.0185	LADLLRADPAK

Spectrum No: 19; Query: 47028; Rank: 1

Peptide View

MS/MS Fragmentation of **ILNPEEIEK**
Found in **IPI00024175**, Tax_Id=9606 Gene_Symbol=PSMA7 Isoform 1 of Proteasome subunit alpha type-7

Match to Query 47028: 1083.583928 from(542.799240,2+)
Title: 090702LimSK_Exosome2_06.3945.3945.2.dta
Data file C:\Temp\Mascoti\LSK_2D\090702LimSK_Exosome2_01_2D.mgf

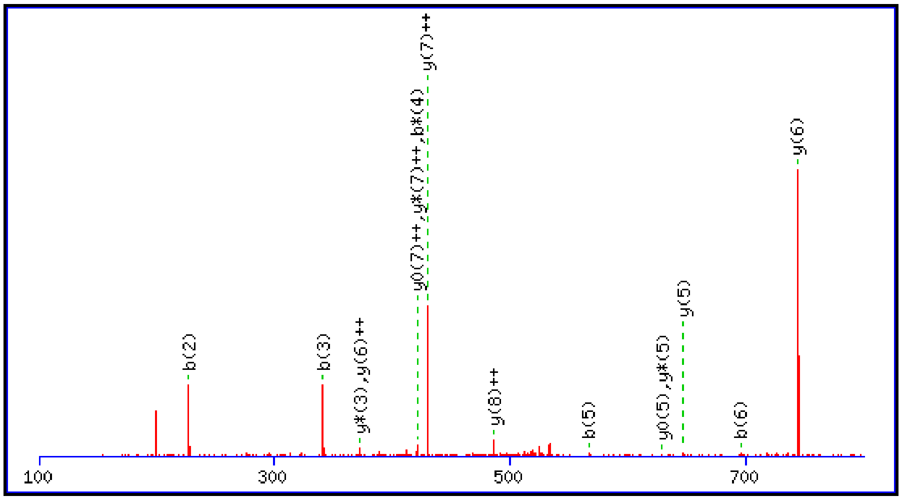

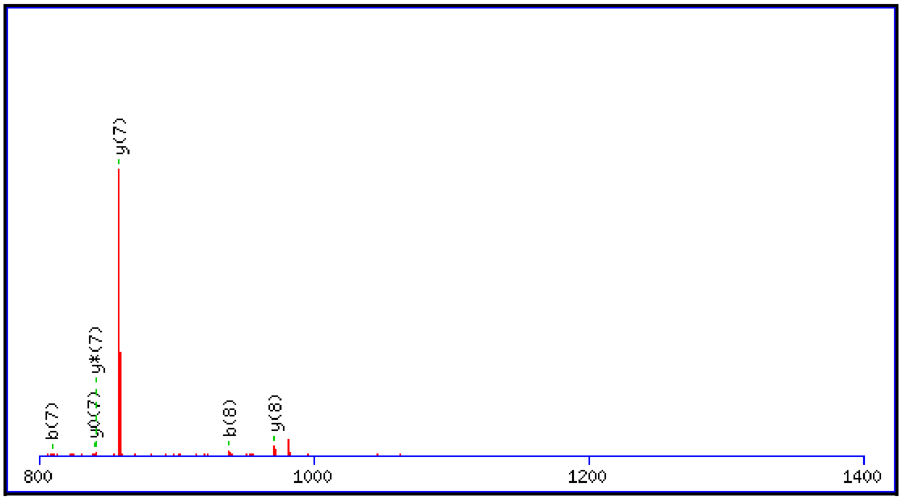

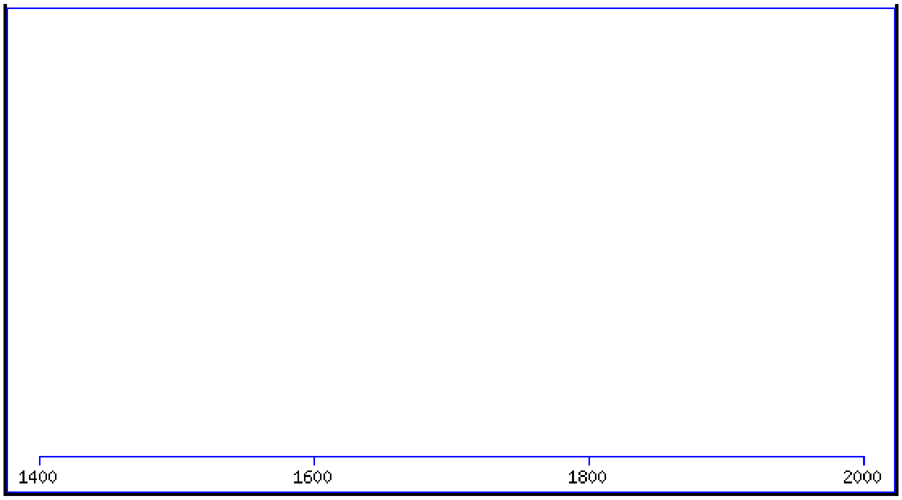

Monoisotopic mass of neutral peptide Mr(calc): 1083.5811
Fixed modifications: Carbamidomethyl (C)
Ions Score: 45 **Expect:** 0.0028
Matches (Bold Red): 21/82 fragment ions using 29 most intense peaks

#	b	b^{++}	b*	b^{*++}	b^0	b^{0++}	Seq.	y	y^{++}	y*	y^{*++}	y^0	y^{0++}	#
1	114.0913	57.5493					I							9
2	**227.1754**	114.0913					L	**971.5044**	**486.2558**	954.4779	477.7426	953.4938	477.2506	8
3	**341.2183**	171.1128	324.1918	162.5995			N	**858.4203**	**429.7138**	**841.3938**	**421.2005**	**840.4098**	**420.7085**	7
4	438.2711	219.6392	**421.2445**	211.1259			P	**744.3774**	**372.6923**	727.3509	364.1791	726.3668	363.6871	6
5	**567.3137**	284.1605	550.2871	275.6472	549.3031	275.1552	E	**647.3246**	324.1660	**630.2981**	315.6527	**629.3141**	315.1607	5
6	**696.3563**	348.6818	679.3297	340.1685	678.3457	339.6765	E	518.2821	259.6447	501.2555	251.1314	500.2715	250.6394	4
7	**809.4403**	405.2238	792.4138	396.7105	791.4298	396.2185	I	389.2395	195.1234	**372.2129**	186.6101	371.2289	186.1181	3
8	**938.4829**	469.7451	921.4564	461.2318	920.4724	460.7398	E	276.1554	138.5813	259.1288	130.0681	258.1448	129.5761	2
9							K	147.1128	74.0600	130.0863	65.5468			1

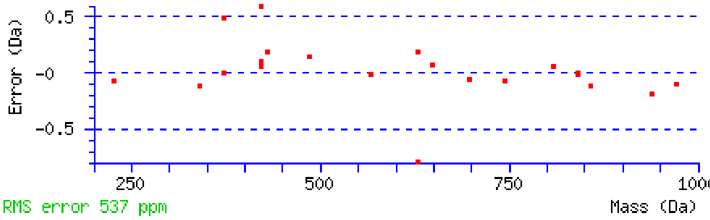

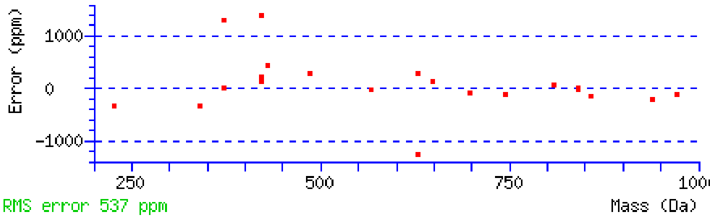

All matches to this query

Score	Mr(calc):	Delta	Sequence
45.3	1083.5811	0.0028	ILNPEEIEK
10.7	1083.5746	0.0093	LLAMENIHK
8.8	1082.5760	1.0079	LLIFGSYDR
6.5	1083.5924	-0.0085	NIQPKVEEK
5.3	1082.5873	0.9967	LLXPPPGNYR
4.7	1083.5746	0.0093	ILEMEGKHK
3.9	1083.5924	-0.0085	NLEANSVLPK
2.8	1083.5763	0.0076	LLATKAMLK
2.8	1083.5859	-0.0019	LLHSSQMLR
2.5	1083.5841	-0.0002	ILQLYARK

Spectrum No: 20; Query: 37893; Rank: 1

Peptide View

MS/MS Fragmentation of **KGSTAVGVR**
Found in **IPI00024175**, Tax_Id=9606 Gene_Symbol=PSMA7 Isoform 1 of Proteasome subunit alpha type-7

Match to Query 37893: 873.504608 from(437.759580,2+)
Title: 090702LimSK_Exosome2_06.2039.2039.2.dta
Data file C:\Temp\Mascoti\LSK_2D\090702LimSK_Exosome2_01_2D.mgf

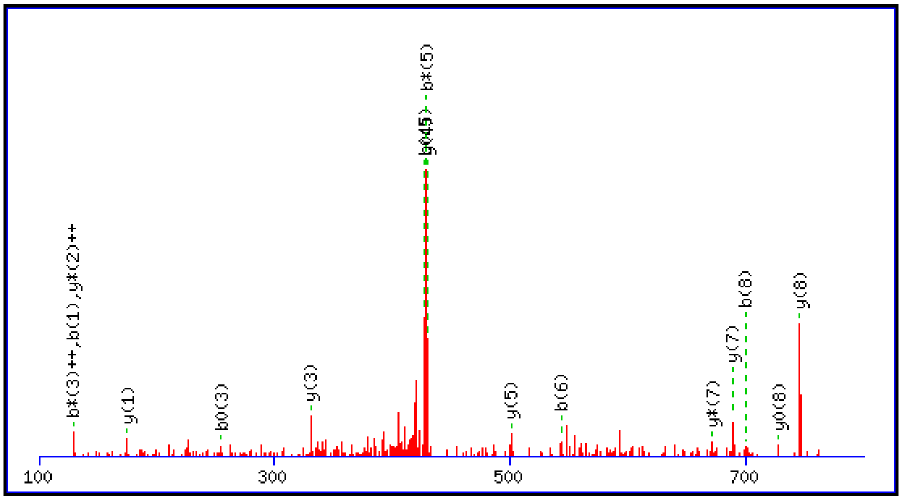

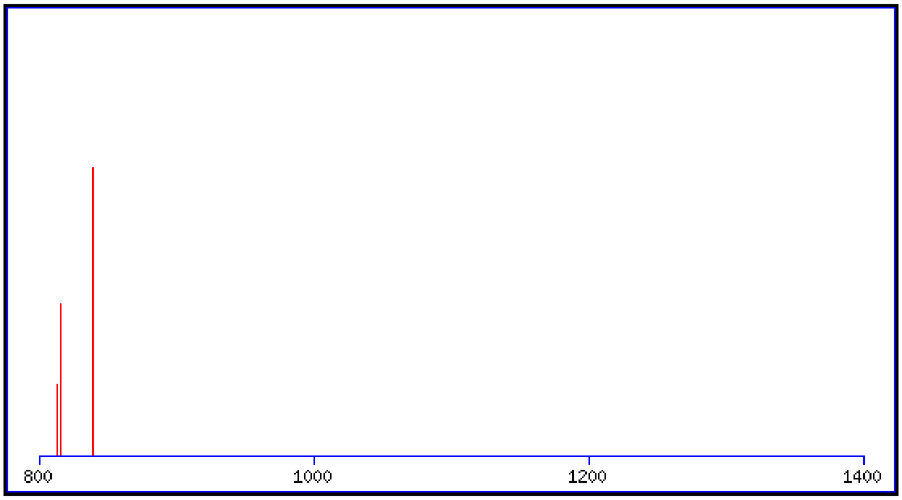

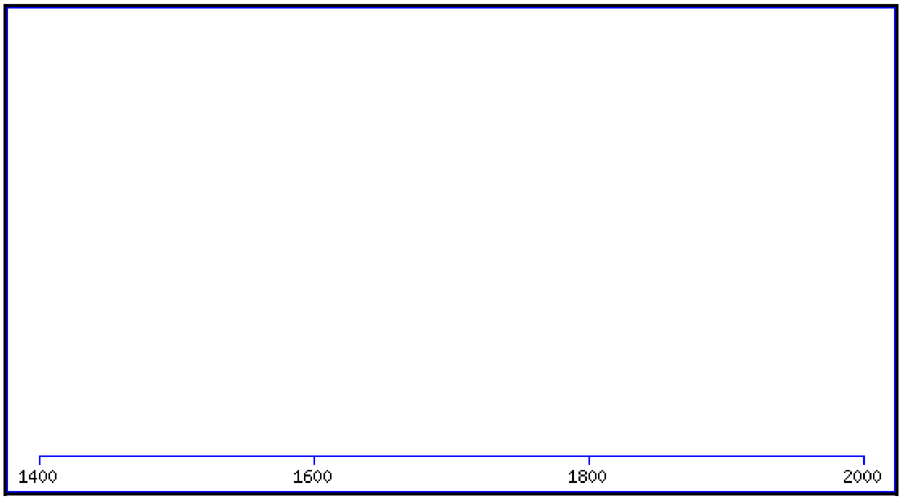

Monoisotopic mass of neutral peptide Mr(calc): 873.5032
Fixed modifications: Carbamidomethyl (C)
Ions Score: 30 **Expect:** 0.15
Matches (Bold Red): 16/82 fragment ions using 43 most intense peaks

#	b	b^{++}	b*	b^{*++}	b^{0}	b^{0++}	Seq.	y	y^{++}	y*	y^{*++}	y^{0}	y^{0++}	#
1	**129.1022**	65.0548	112.0757	56.5415			K							9
2	186.1237	93.5655	169.0972	85.0522			G	**746.4155**	373.7114	729.3890	365.1981	**728.4050**	364.7061	8
3	273.1557	137.0815	256.1292	**128.5682**	**255.1452**	128.0762	S	**689.3941**	345.2007	**672.3675**	336.6874	671.3835	336.1954	7
4	374.2034	187.6053	357.1769	179.0921	356.1928	178.6001	T	602.3620	301.6847	585.3355	293.1714	584.3515	292.6794	6
5	445.2405	223.1239	**428.2140**	214.6106	**427.2300**	214.1186	A	**501.3144**	251.1608	484.2878	242.6475			5
6	**544.3089**	272.6581	527.2824	264.1448	526.2984	263.6528	V	**430.2772**	215.6423	413.2507	207.1290			4
7	601.3304	301.1688	584.3039	292.6556	583.3198	292.1636	G	**331.2088**	166.1081	314.1823	157.5948			3
8	**700.3988**	350.7030	683.3723	342.1898	682.3883	341.6978	V	274.1874	137.5973	257.1608	**129.0840**			2
9							R	**175.1190**	88.0631	158.0924	79.5498			1

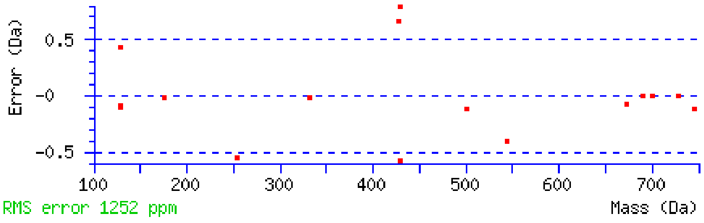

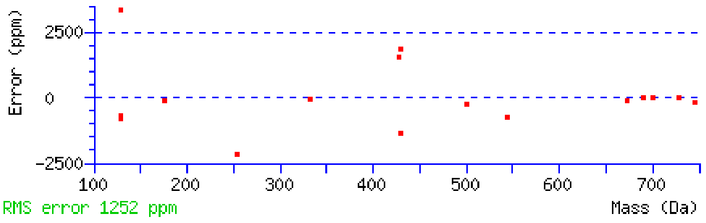

All matches to this query

Score	Mr(calc):	Delta	Sequence
29.6	873.5032	0.0014	KGSTAVGVR
21.9	872.5080	0.9966	LLVQTGSR
16.1	873.5072	-0.0026	LISIWSR
13.4	873.5032	0.0014	LISETRR
13.3	873.5072	-0.0026	LAFLASPR
12.7	873.5032	0.0014	KDKQSIR
12.3	873.5032	0.0014	KDSQIKR
11.9	873.5032	0.0014	KGGNVGKSK
11.0	873.5106	-0.0060	ILQTMLR
10.6	873.5106	-0.0060	LISLICR

Spectrum No: 21; Query: 75013; Rank: 1

Peptide View

MS/MS Fragmentation of **LTVEDPVTVEYITR**
Found in **IPI00024175**, Tax_Id=9606 Gene_Symbol=PSMA7 Isoform 1 of Proteasome subunit alpha type-7

Match to Query 75013: 1634.862988 from(818.438770,2+)
Title: 090702LimSK_Exosome2_08.6823.6823.2.dta
Data file C:\Temp\Mascoti\LSK_2D\090702LimSK_Exosome2_01_2D.mgf

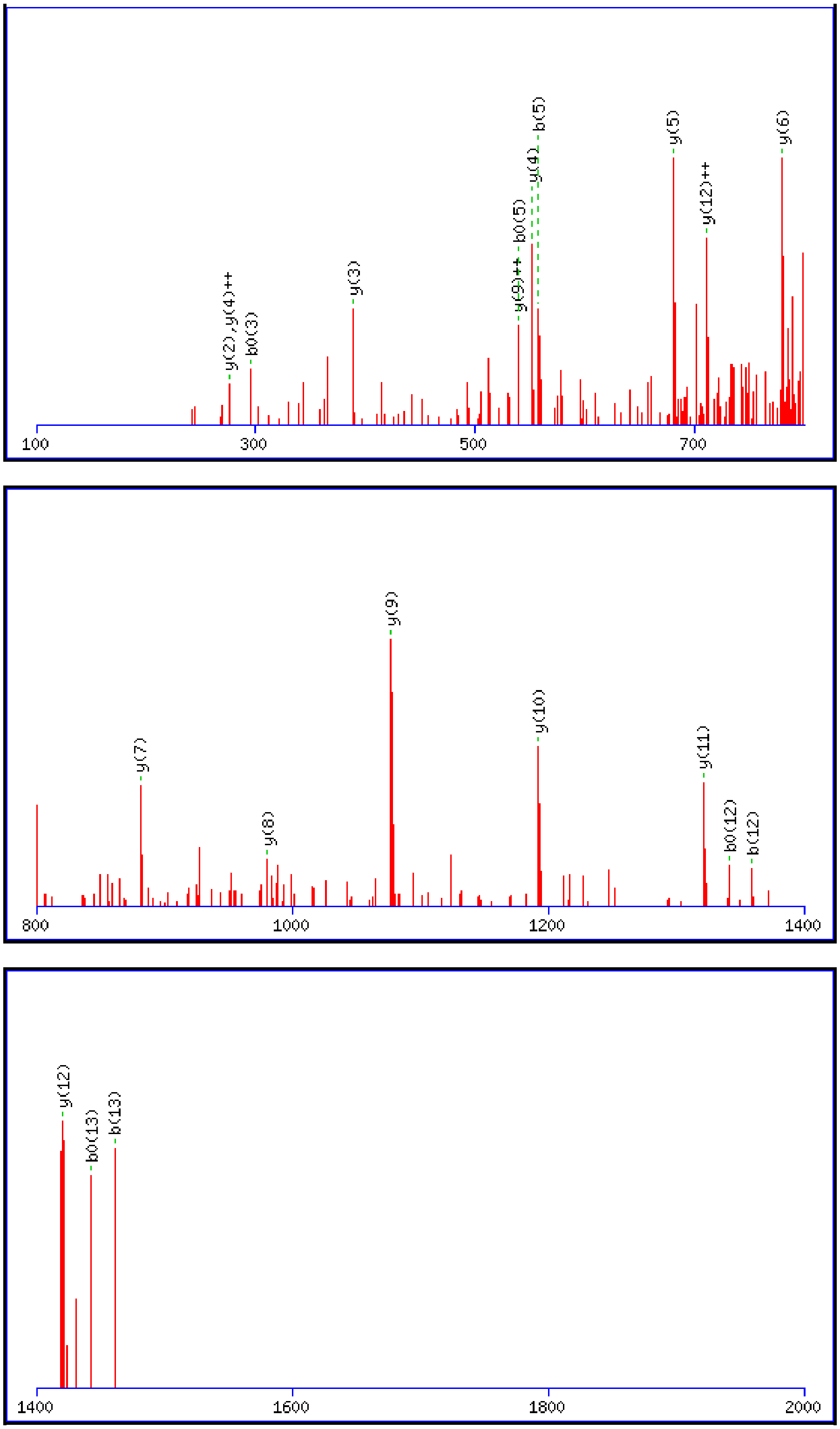

Monoisotopic mass of neutral peptide Mr(calc): 1633.8563
Fixed modifications: Carbamidomethyl (C)
Ions Score: 96 **Expect:** 3.6e-008
Matches (Bold Red): 21/126 fragment ions using 28 most intense peaks

#	b	b^{++}	b^0	b^{0++}	Seq.	y	y^{++}	y^*	y^{*++}	y^0	y^{0++}	#
1	114.0913	57.5493			L							14
2	215.1390	108.0731	197.1285	99.0679	T	1521.7795	761.3934	1504.7530	752.8801	1503.7690	752.3881	13
3	314.2074	157.6074	**296.1969**	148.6021	V	**1420.7318**	**710.8696**	1403.7053	702.3563	1402.7213	701.8643	12
4	443.2500	222.1287	425.2395	213.1234	E	**1321.6634**	661.3353	1304.6369	652.8221	1303.6529	652.3301	11
5	**558.2770**	279.6421	**540.2664**	270.6368	D	**1192.6208**	596.8141	1175.5943	588.3008	1174.6103	587.8088	10
6	655.3297	328.1685	637.3192	319.1632	P	**1077.5939**	**539.3006**	1060.5673	530.7873	1059.5833	530.2953	9

**7**	754.3981	377.7027	736.3876	368.6974	**V**	**980.5411**	490.7742	963.5146	482.2609	962.5306	481.7689	**8**
**8**	855.4458	428.2266	837.4353	419.2213	**T**	**881.4727**	441.2400	864.4462	432.7267	863.4621	432.2347	**7**
**9**	954.5142	477.7608	936.5037	468.7555	**V**	**780.4250**	390.7162	763.3985	382.2029	762.4145	381.7109	**6**
**10**	1083.5568	542.2821	1065.5463	533.2768	**E**	**681.3566**	341.1819	664.3301	332.6687	663.3461	332.1767	**5**
**11**	1246.6202	623.8137	1228.6096	614.8084	**Y**	**552.3140**	**276.6607**	535.2875	268.1474	534.3035	267.6554	**4**
**12**	**1359.7042**	680.3558	**1341.6937**	671.3505	**I**	**389.2507**	195.1290	372.2241	186.6157	371.2401	186.1237	**3**
**13**	**1460.7519**	730.8796	**1442.7413**	721.8743	**T**	**276.1666**	138.5870	259.1401	130.0737	258.1561	129.5817	**2**
**14**					**R**	175.1190	88.0631	158.0924	79.5498			**1**

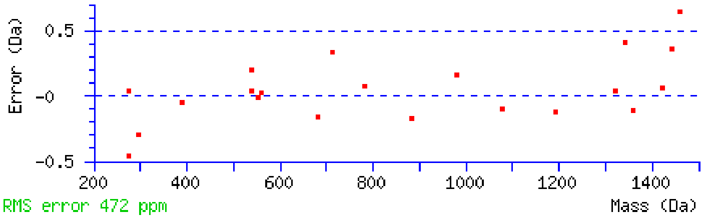

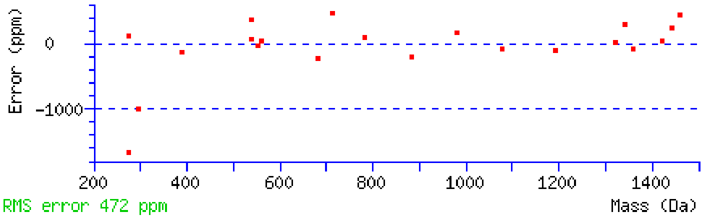

All matches to this query

Score	Mr(calc):	Delta	Sequence
95.8	1633.8563	1.0067	LTVEDPVTVEYITR
7.5	1633.8480	1.0150	EKLTLLHYDPVVK
3.7	1634.8701	-0.0071	MKLYIDNAAPDKLK
2.9	1634.8505	0.0125	SLLADTRQLAAQLR
2.2	1633.8610	1.0020	NRLVNIMPYELTR
2.0	1632.8501	2.0129	KFLPSTANRGLPPR
2.0	1632.8501	2.0129	KFLPSTANRGLPPR
0.8	1633.8552	1.0077	ELTGVGGREALLLAR
0.7	1633.8675	0.9954	LTPLTKFGEGDSTIR

Spectrum No: 22; Query: 75819; Rank: 1

Peptide View

MS/MS Fragmentation of **LYQTDPSGTYHAWK**
Found in **IPI00024175**, Tax_Id=9606 Gene_Symbol=PSMA7 Isoform 1 of Proteasome subunit alpha type-7

Match to Query 75819: 1665.782622 from(556.268150,3+)
Title: 090702LimSK_Exosome2_01.3401.3401.3.dta
Data file C:\Temp\Mascoti\LSK_2D\090702LimSK_Exosome2_01_2D.mgf

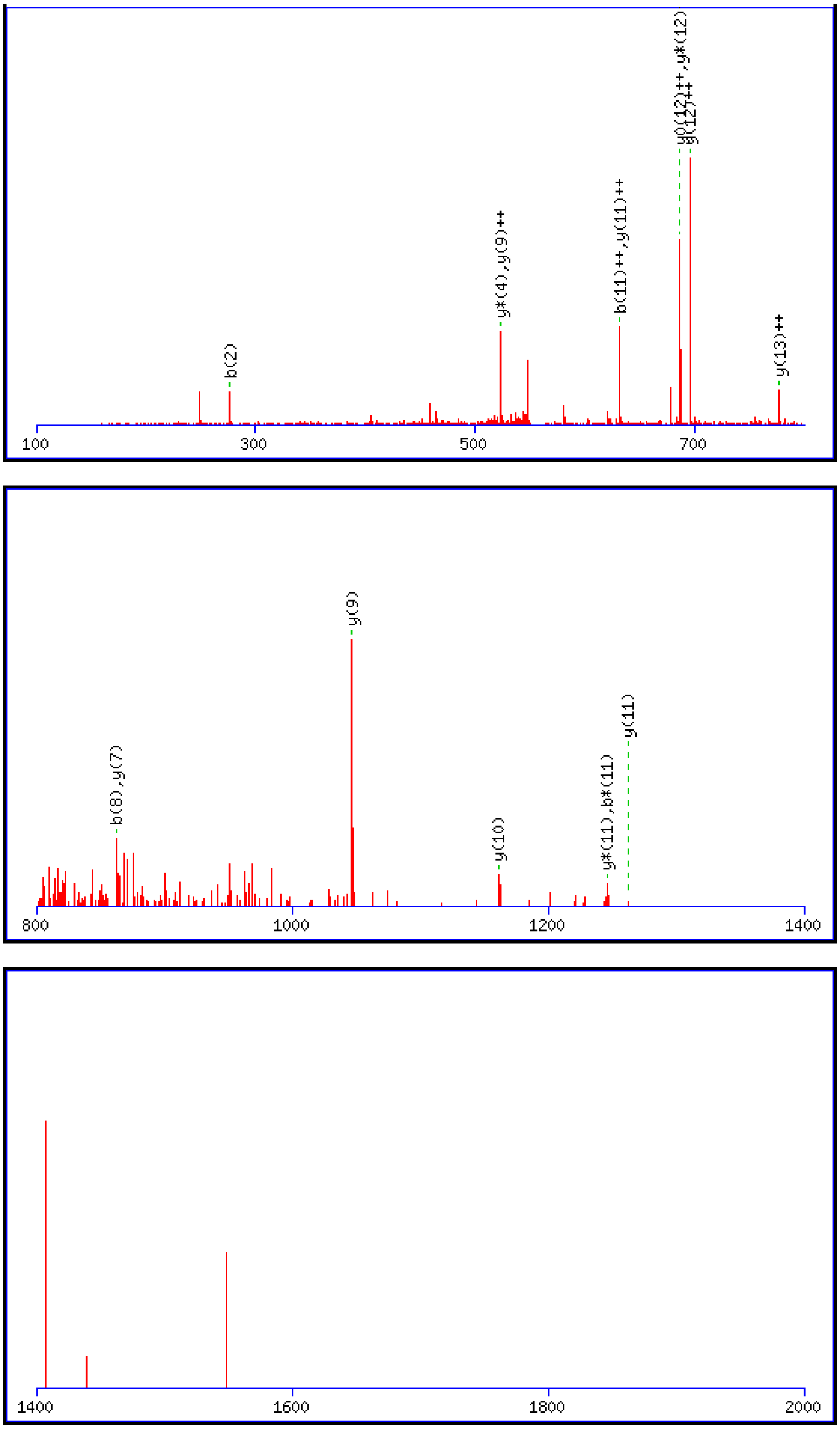

Monoisotopic mass of neutral peptide Mr(calc): 1665.7787
Fixed modifications: Carbamidomethyl (C)
Ions Score: 25 **Expect:** 0.78
Matches (Bold Red): 18/136 fragment ions using 27 most intense peaks

#	b	b^{++}	b*	b^{*++}	b^0	b^{0++}	Seq.	y	y^{++}	y*	y^{*++}	y^0	y^{0++}	#
1	114.0913	57.5493					L							14
2	**277.1547**	139.0810					Y	1553.7019	**777.3546**	1536.6754	768.8413	1535.6914	768.3493	13
3	405.2132	203.1103	388.1867	194.5970			Q	1390.6386	**695.8229**	1373.6121	**687.3097**	1372.6280	**686.8177**	12
4	506.2609	253.6341	489.2344	245.1208	488.2504	244.6288	T	**1262.5800**	**631.7937**	**1245.5535**	623.2804	1244.5695	622.7884	11
5	621.2879	311.1476	604.2613	302.6343	603.2773	302.1423	D	**1161.5324**	581.2698	1144.5058	572.7565	1143.5218	572.2645	10
6	718.3406	359.6740	701.3141	351.1607	700.3301	350.6687	P	**1046.5054**	**523.7563**	1029.4789	515.2431	1028.4948	514.7511	9

7	805.3727	403.1900	788.3461	394.6767	787.3621	394.1847	S	949.4526	475.2300	932.4261	466.7167	931.4421	466.2247	8
8	**862.3941**	431.7007	845.3676	423.1874	844.3836	422.6954	G	**862.4206**	431.7139	845.3941	423.2007	844.4100	422.7087	7
9	963.4418	482.2245	946.4153	473.7113	945.4312	473.2193	T	805.3992	403.2032	788.3726	394.6899	787.3886	394.1979	6
10	1126.5051	563.7562	1109.4786	555.2429	1108.4946	554.7509	Y	704.3515	352.6794	**687.3249**	344.1661			5
11	1263.5640	**632.2857**	**1246.5375**	623.7724	**1245.5535**	623.2804	H	541.2881	271.1477	**524.2616**	262.6344			4
12	1334.6012	667.8042	1317.5746	659.2909	1316.5906	658.7989	A	404.2292	202.6183	387.2027	194.1050			3
13	1520.6805	760.8439	1503.6539	752.3306	1502.6699	751.8386	W	333.1921	167.0997	316.1656	158.5864			2
14							K	147.1128	74.0600	130.0863	65.5468			1

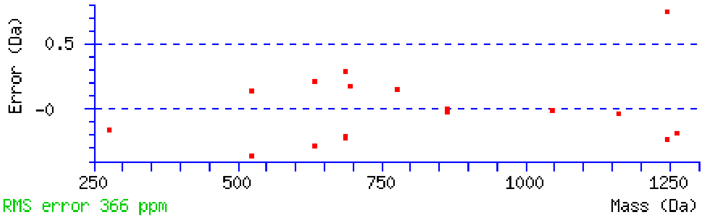

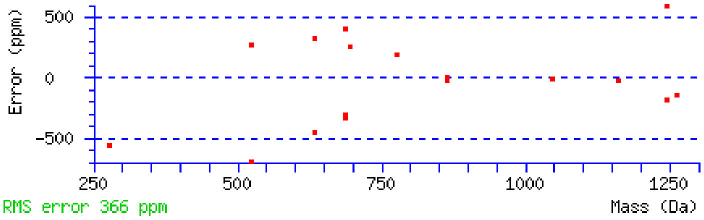

All matches to this query

Score	Mr(calc):	Delta	Sequence
24.7	1665.7787	0.0039	LYQTDPSGTYHAWK
11.7	1664.7707	1.0119	YLMVPAFQGALTMK
11.6	1664.7814	1.0012	EKELAEDIMSVEEK
10.8	1663.7848	1.9978	ILKPASAHSPTQVR
8.3	1664.7667	1.0159	LYKSMTLLNTMDR
8.3	1665.7992	-0.0166	SRICDETANATSTLK
8.1	1664.7811	1.0016	EQPPALFSALQEQK
7.5	1665.7876	-0.0049	EYGKSFSRGSLVTR
7.2	1664.7667	1.0159	LYKSMTLLNTMDR
6.9	1663.7909	1.9917	KENMEELLNLCSGK

Spectrum No: 23; Query: 40489; Rank: 1

Peptide View

MS/MS Fragmentation of **NIELAVMR**
Found in **IPI00024175**, Tax_Id=9606 Gene_Symbol=PSMA7 Isoform 1 of Proteasome subunit alpha type-7

Match to Query 40489: 944.513648 from(473.264100,2+)
Title: 090702LimSK_Exosome2_05.5522.5522.2.dta
Data file C:\Temp\Mascoti\LSK_2D\090702LimSK_Exosome2_01_2D.mgf

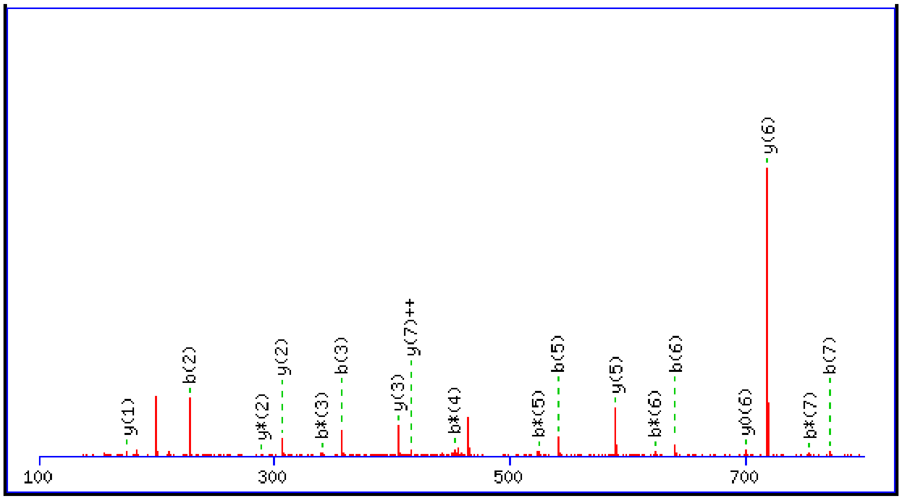

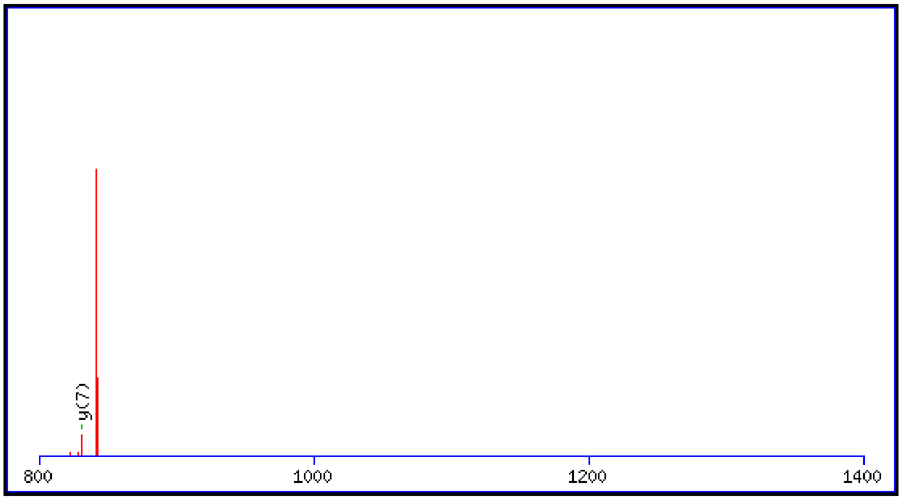

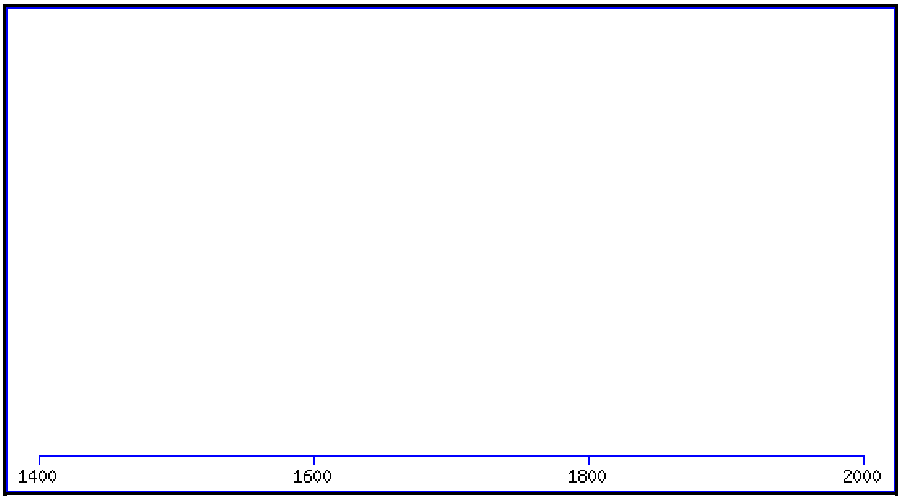

Monoisotopic mass of neutral peptide Mr(calc): 944.5113
Fixed modifications: Carbamidomethyl (C)
Ions Score: 36 **Expect:** 0.033
Matches (Bold Red): 19/70 fragment ions using 38 most intense peaks

#	b	b^{++}	b*	b^{*++}	b^0	b^{0++}	Seq.	y	y^{++}	y*	y^{*++}	y^0	y^{0++}	#
1	115.0502	58.0287	98.0237	49.5155			N							8
2	**228.1343**	114.5708	211.1077	106.0575			I	**831.4757**	**416.2415**	814.4491	407.7282	813.4651	407.2362	7
3	**357.1769**	179.0921	**340.1503**	170.5788	339.1663	170.0868	E	**718.3916**	359.6994	701.3651	351.1862	**700.3811**	350.6942	6
4	470.2609	235.6341	**453.2344**	227.1208	452.2504	226.6288	L	**589.3490**	295.1782	572.3225	286.6649			5
5	**541.2980**	271.1527	**524.2715**	262.6394	523.2875	262.1474	A	476.2650	238.6361	459.2384	230.1228			4
6	**640.3665**	320.6869	**623.3399**	312.1736	622.3559	311.6816	V	**405.2279**	203.1176	388.2013	194.6043			3

7	771.4069	386.2071	754.3804	377.6938	753.3964	377.2018	M	306.1594	153.5834	289.1329	145.0701			2
8							R	175.1190	88.0631	158.0924	79.5498			1

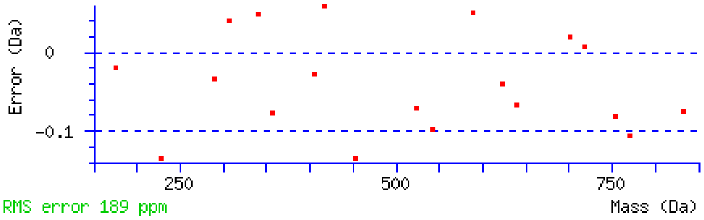

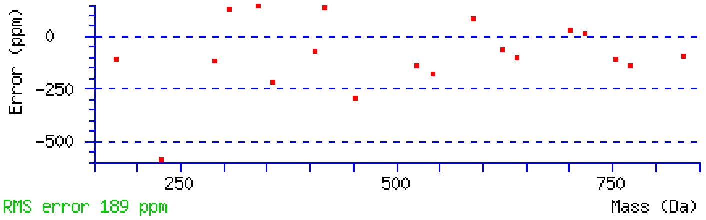

All matches to this query

Score	Mr(calc):	Delta	Sequence
35.7	944.5113	0.0023	NIELAVMR
8.0	944.5151	-0.0015	LNARAASSR
6.5	943.5087	1.0050	LLQTGQER
6.2	943.5087	1.0050	LLEEERR
6.2	943.5087	1.0050	LLEERER
4.2	944.5178	-0.0042	LNEELLSK
3.6	943.5100	1.0036	GRQGGRWK
3.5	944.5179	-0.0042	ITIXPDTSK
3.3	942.5134	2.0002	KDNLAVNAV
2.8	942.5134	2.0003	KPEEKANK

Spectrum No: 24; Query: 77824; Rank: 1

Peptide View

MS/MS Fragmentation of **NYTDEAIETDDLTIK**
Found in **IPI00024175**, Tax_Id=9606 Gene_Symbol=PSMA7 Isoform 1 of Proteasome subunit alpha type-7

Match to Query 77824: 1739.816108 from(870.915330,2+)
Title: 090702LimSK_Exosome2_06.6093.6093.2.dta
Data file C:\Temp\Mascoti\LSK_2D\090702LimSK_Exosome2_01_2D.mgf

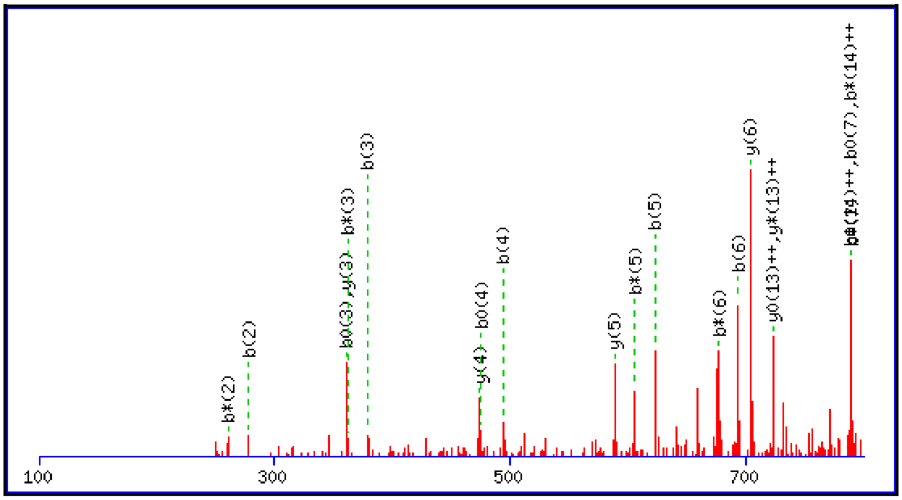

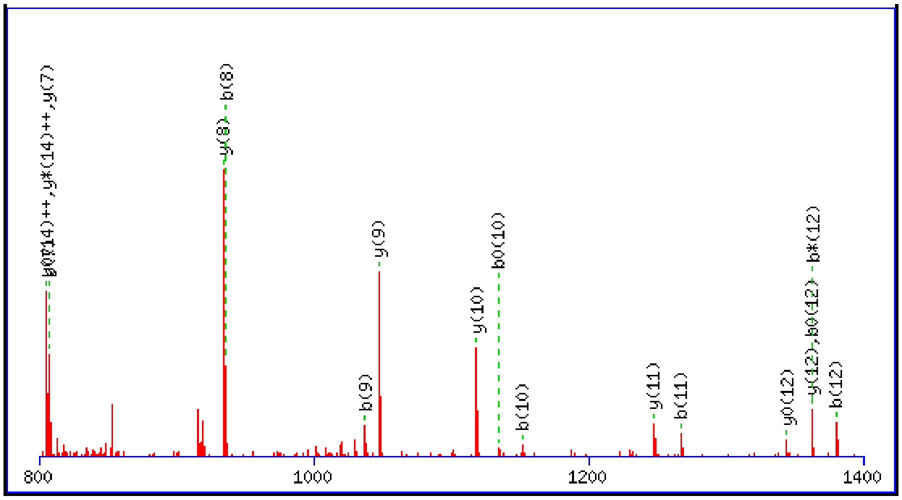

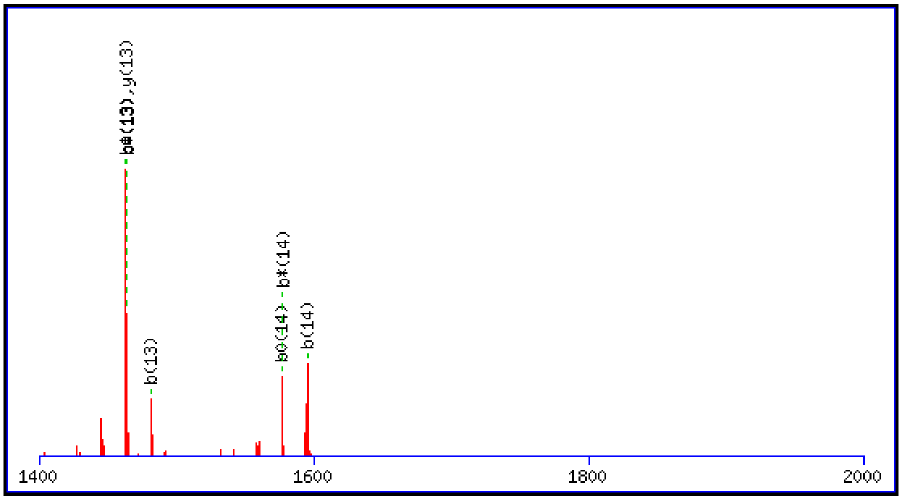

Monoisotopic mass of neutral peptide Mr(calc): 1739.8101
Fixed modifications: Carbamidomethyl (C)
Ions Score: 126 **Expect:** 6e-011
Matches (Bold Red): 46/160 fragment ions using 45 most intense peaks

#	b	b++	b*	b*++	b0	b0++	Seq.	y	y++	y*	y*++	y0	y0++	#
1	115.0502	58.0287	98.0237	49.5155			N							15
2	**278.1135**	139.5604	**261.0870**	131.0471			Y	1626.7745	813.8909	1609.7479	**805.3776**	1608.7639	**804.8856**	14
3	**379.1612**	190.0842	**362.1347**	181.5710	**361.1506**	181.0790	T	**1463.7112**	732.3592	1446.6846	**723.8459**	1445.7006	**723.3539**	13
4	**494.1882**	247.5977	477.1616	239.0844	**476.1776**	238.5924	D	**1362.6635**	681.8354	1345.6369	673.3221	**1344.6529**	672.8301	12
5	**623.2307**	312.1190	**606.2042**	303.6057	605.2202	303.1137	E	**1247.6365**	624.3219	1230.6100	615.8086	1229.6260	615.3166	11
6	**694.2679**	347.6376	**677.2413**	339.1243	676.2573	338.6323	A	**1118.5939**	559.8006	1101.5674	551.2873	1100.5834	550.7953	10
7	**807.3519**	404.1796	**790.3254**	395.6663	**789.3414**	395.1743	I	**1047.5568**	524.2821	1030.5303	515.7688	1029.5463	515.2768	9
8	**936.3945**	468.7009	919.3680	460.1876	918.3840	459.6956	E	**934.4728**	467.7400	917.4462	459.2267	916.4622	458.7347	8
9	**1037.4422**	519.2247	1020.4156	510.7115	1019.4316	510.2195	T	**805.4302**	403.2187	788.4036	394.7055	787.4196	394.2134	7
10	**1152.4691**	576.7382	1135.4426	568.2249	**1134.4586**	567.7329	D	**704.3825**	352.6949	687.3559	344.1816	686.3719	343.6896	6
11	**1267.4961**	634.2517	1250.4695	625.7384	1249.4855	625.2464	D	**589.3556**	295.1814	572.3290	286.6681	571.3450	286.1761	5
12	**1380.5801**	690.7937	**1363.5536**	682.2804	**1362.5696**	681.7884	L	**474.3286**	237.6679	457.3021	229.1547	456.3180	228.6627	4
13	**1481.6278**	741.3176	**1464.6013**	732.8043	**1463.6173**	732.3123	T	**361.2445**	181.1259	344.2180	172.6126	343.2340	172.1206	3
14	**1594.7119**	797.8596	**1577.6853**	**789.3463**	**1576.7013**	**788.8543**	I	260.1969	130.6021	243.1703	122.0888			2
15							K	147.1128	74.0600	130.0863	65.5468			1

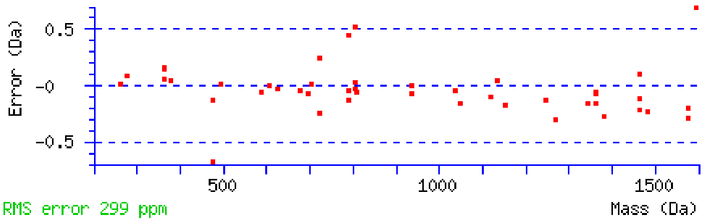

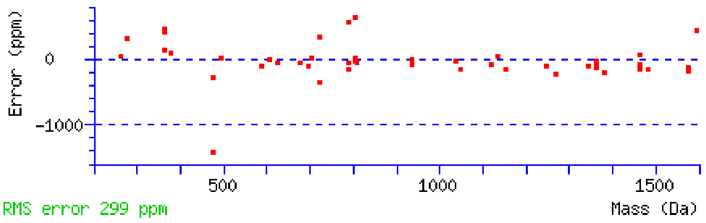

All matches to this query

Score	Mr(calc):	Delta	Sequence
125.6	1739.8101	0.0060	NYTDEAIETDDLTIK
17.8	1739.8222	-0.0061	IFTVAQMDDNSIQMK
13.1	1739.8222	-0.0061	MKQYASPMPTQTDVK
11.4	1738.8010	1.0151	KQSTQFSGGPTEDTEK
8.8	1739.8066	0.0095	DEGHRVLIFSQMTK
8.2	1739.8248	-0.0087	MLTGDTTVTSGDATVAGK
6.0	1739.8260	-0.0099	GLNGGITPLNSISPLK
5.6	1739.8260	-0.0099	GLNGGITPLNSISPLK
5.3	1739.8317	-0.0156	YQMPTGIKGPLPNTK
5.0	1739.8335	-0.0174	CNSVKDGAICYKPTK

Spectrum No: 25; Query: 26515; Rank: 1

Peptide View

MS/MS Fragmentation of **YIASLK**
Found in **IPI00024175**, Tax_Id=9606 Gene_Symbol=PSMA7 Isoform 1 of Proteasome subunit alpha type-7

Match to Query 26515: 693.407214 from(694.414490,1+)
Title: 090702LimSK_Exosome2_06.2018.2018.1.dta
Data file C:\Temp\Mascoti\LSK_2D\090702LimSK_Exosome2_01_2D.mgf

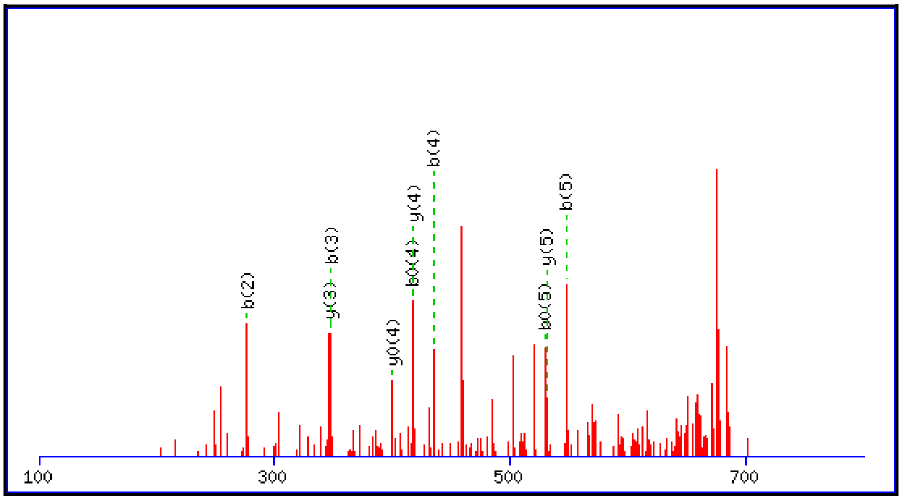

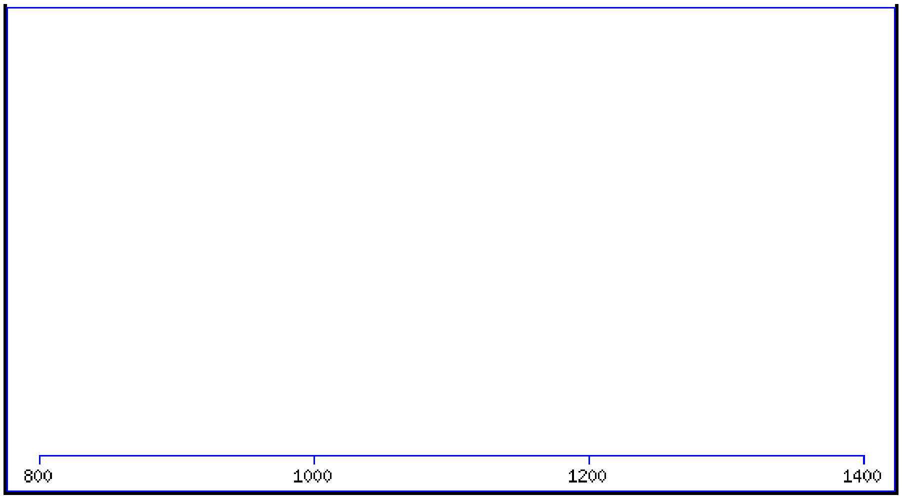

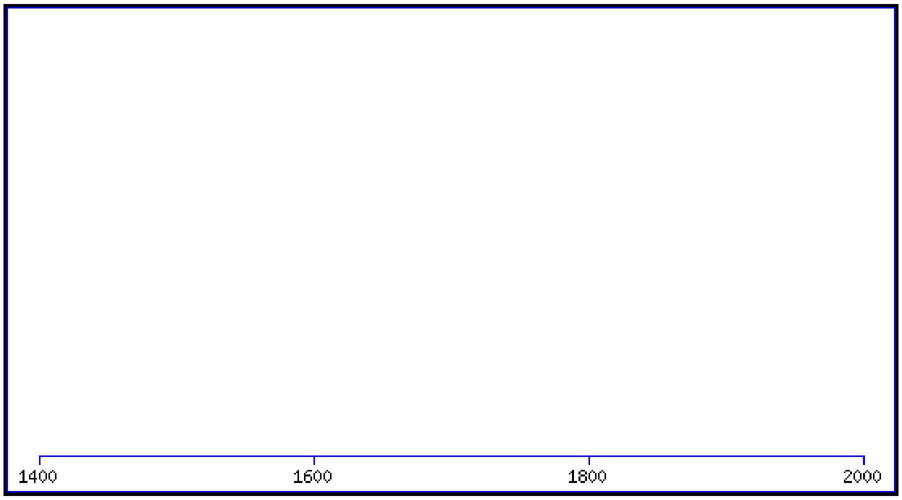

Monoisotopic mass of neutral peptide Mr(calc): 693.4061
Fixed modifications: Carbamidomethyl (C)
Ions Score: 25 **Expect:** 0.23
Matches (Bold Red): 10/20 fragment ions using 20 most intense peaks

#	b	b^0	Seq.	y	y*	y^0	#
1	164.0706		Y				6
2	**277.1547**		I	**531.3501**	514.3235	513.3395	5
3	**348.1918**		A	**418.2660**	401.2395	**400.2554**	4
4	**435.2238**	**417.2132**	S	**347.2289**	330.2023	329.2183	3
5	**548.3079**	**530.2973**	L	260.1969	243.1703		2
6			K	147.1128	130.0863		1

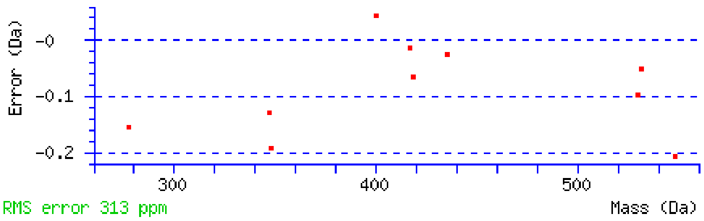

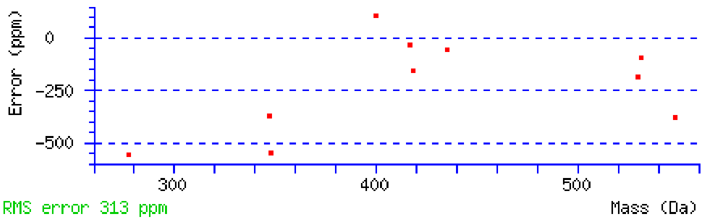

All matches to this query

Score	Mr(calc):	Delta	Sequence
24.7	693.4061	0.0011	YIASLK
12.7	693.4061	0.0011	YASLLK
10.1	693.4061	0.0011	IYVTAK

10.1	693.4061	0.0011	YIISAK
9.8	693.4061	0.0011	SFLSLK
7.5	693.4061	0.0011	LSFSLK
7.5	693.4061	0.0011	SLFSLK
5.8	693.4061	0.0011	TFSVIK
5.3	692.3969	1.0103	YLNRK
4.7	693.4061	0.0011	FLSLSK

Spectrum No: 26; Query: 77731; Rank: 1

Peptide View

MS/MS Fragmentation of **YVAEIEKEKEKEENEK**
Found in **IPI00024175**, Tax_Id=9606 Gene_Symbol=PSMA7 Isoform 1 of Proteasome subunit alpha type-7

Match to Query 77731: 1736.851122 from(579.957650,3+)
Title: 090702LimSK_Exosome2_01.1028.1028.3.dta
Data file C:\Temp\Mascoti\LSK_2D\090702LimSK_Exosome2_01_2D.mgf

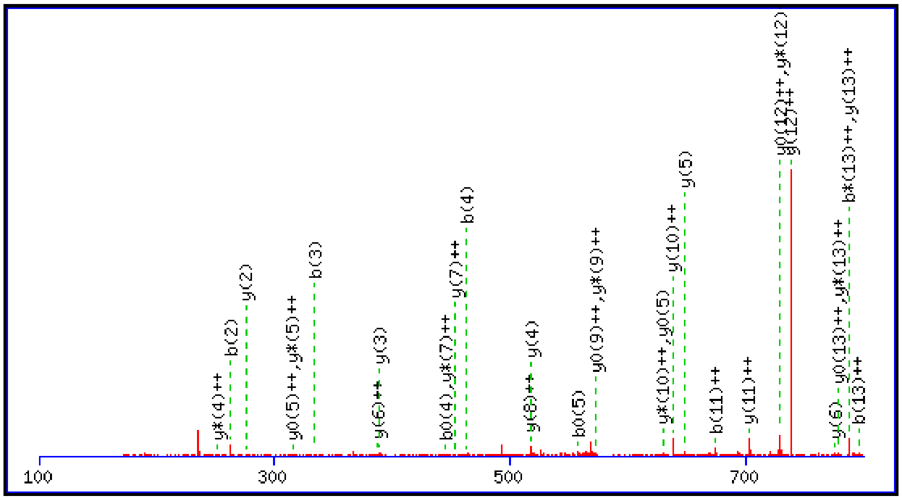

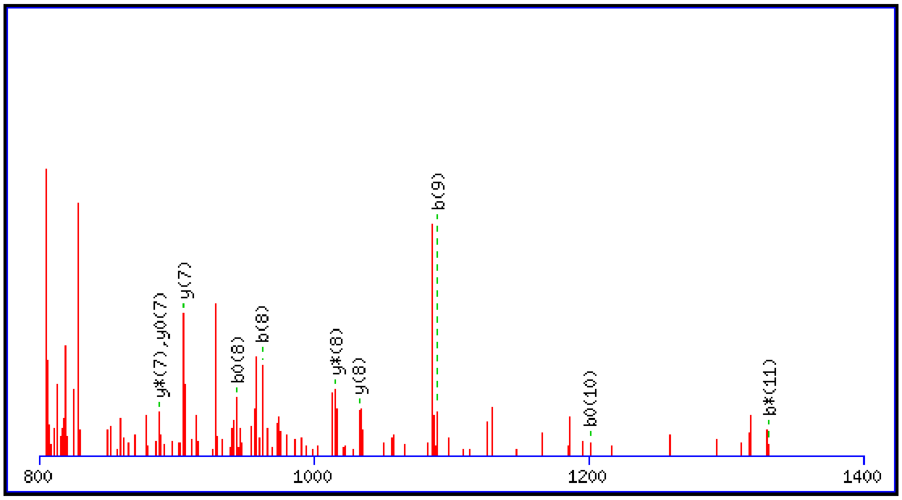

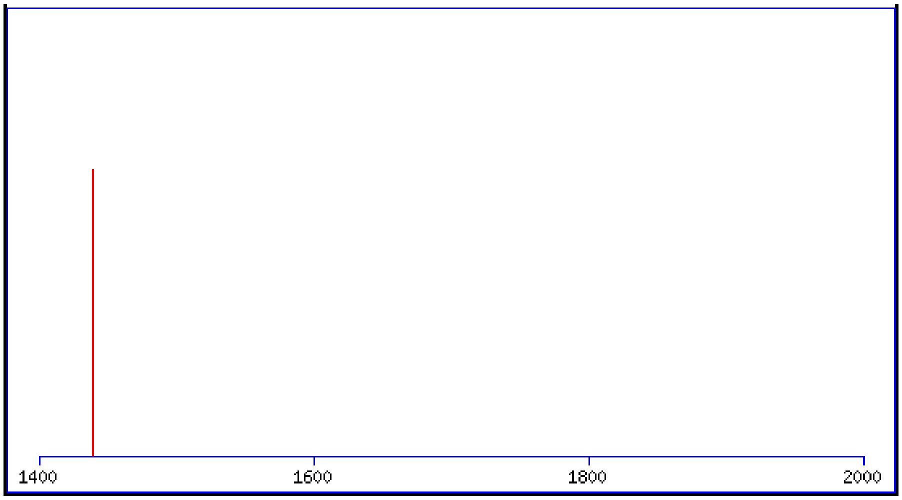

Monoisotopic mass of neutral peptide Mr(calc): 1736.8468
Fixed modifications: Carbamidomethyl (C)
Ions Score: 23 **Expect:** 0.97
Matches (Bold Red): 42/136 fragment ions using 117 most intense peaks

#	b	b^{++}	b^{*}	b^{*++}	b^{0}	b^{0++}	Seq.	y	y^{++}	y^{*}	y^{*++}	y^{0}	y^{0++}	#
1	164.0706	82.5389					Y							14
2	**263.1390**	132.0731					V	1574.7908	**787.8990**	1557.7643	**779.3858**	1556.7802	**778.8938**	13
3	**334.1761**	167.5917					A	1475.7224	**738.3648**	1458.6958	**729.8516**	1457.7118	**729.3596**	12
4	**463.2187**	232.1130			**445.2082**	223.1077	E	1404.6853	**702.8463**	1387.6587	694.3330	1386.6747	693.8410	11
5	576.3028	288.6550			**558.2922**	279.6498	I	1275.6427	**638.3250**	1258.6161	**629.8117**	1257.6321	629.3197	10
6	705.3454	353.1763			687.3348	344.1710	E	1162.5586	581.7829	1145.5321	**573.2697**	1144.5481	**572.7777**	9
7	833.4403	417.2238	816.4138	408.7105	815.4298	408.2185	K	**1033.5160**	**517.2617**	**1016.4895**	508.7484	1015.5055	508.2564	8
8	**962.4829**	481.7451	945.4564	473.2318	**944.4724**	472.7398	E	**905.4211**	**453.2142**	**888.3945**	**444.7009**	**887.4105**	444.2089	7
9	**1090.5779**	545.7926	1073.5514	537.2793	1072.5673	536.7873	K	**776.3785**	**388.6929**	759.3519	380.1796	758.3679	379.6876	6
10	1219.6205	610.3139	1202.5939	601.8006	**1201.6099**	601.3086	E	**648.2835**	324.6454	631.2570	**316.1321**	**630.2729**	**315.6401**	5
11	1348.6631	**674.8352**	**1331.6365**	666.3219	1330.6525	665.8299	E	**519.2409**	260.1241	502.2144	**251.6108**	501.2304	251.1188	4
12	1462.7060	731.8566	1445.6795	723.3434	1444.6955	722.8514	N	**390.1983**	195.6028	373.1718	187.0895	372.1878	186.5975	3
13	1591.7486	**796.3779**	1574.7221	**787.8647**	1573.7380	787.3727	E	**276.1554**	138.5813	259.1288	130.0681	258.1448	129.5761	2
14							K	147.1128	74.0600	130.0863	65.5468			1

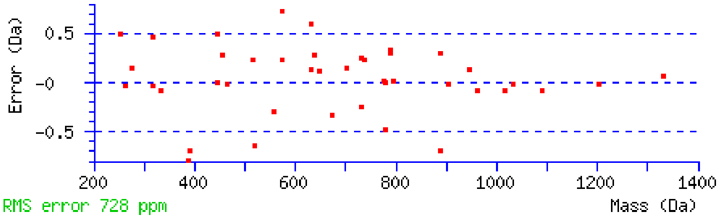

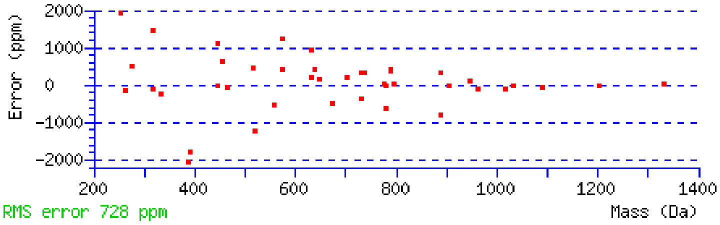

All matches to this query

Score	Mr(calc):	Delta	Sequence
23.4	1736.8468	0.0043	YVAEIEKEKEENEK
7.2	1736.8359	0.0152	IRSNNHLAQTVYGGK
7.1	1735.8519	0.9992	SDHLAKHIKTHQNK
6.6	1735.8519	0.9992	VRLVSHLNNYASQR
6.2	1736.8532	-0.0021	AIDATLMSPRGIAVDK
5.8	1734.8553	1.9958	RLGPISADSTTAPLEK
5.5	1734.8389	2.0122	GLAMLHVTRGVWGSR
5.5	1734.8389	2.0122	GLAMLHVTRGVWGSR

4.9	1734.8277	2.0235	CVRFSKVISSYPGR
4.9	1734.8618	1.9894	CTRAPSMTTRGTALGR

Spectrum No: 27; Query: 42160; Rank: 1

Peptide View

MS/MS Fragmentation of **AIYQATYR**
Found in **IPI00479306**, Tax_Id=9606 Gene_Symbol=PSMB5 Proteasome subunit beta type-5

Match to Query 42160: 984.504108 from(493.259330,2+)
Title: 090702LimSK_Exosome2_06.2416.2416.2.dta
Data file C:\Temp\Mascoti\LSK_2D\090702LimSK_Exosome2_01_2D.mgf

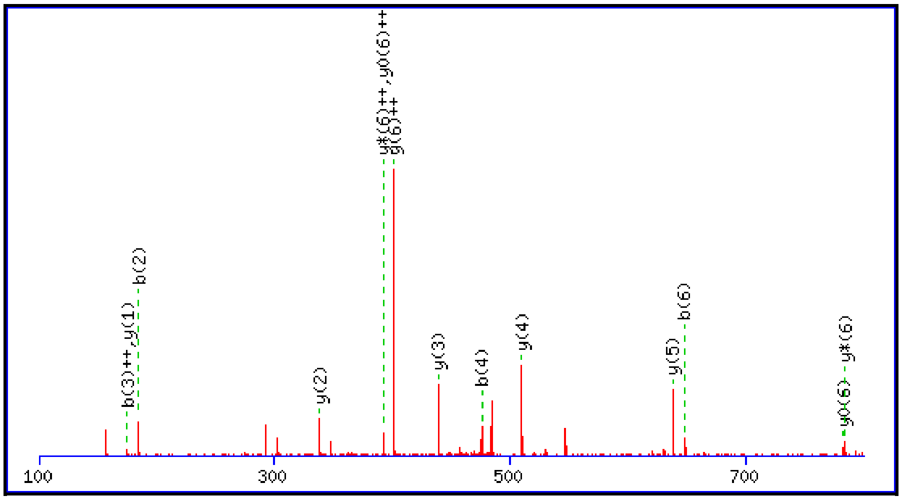

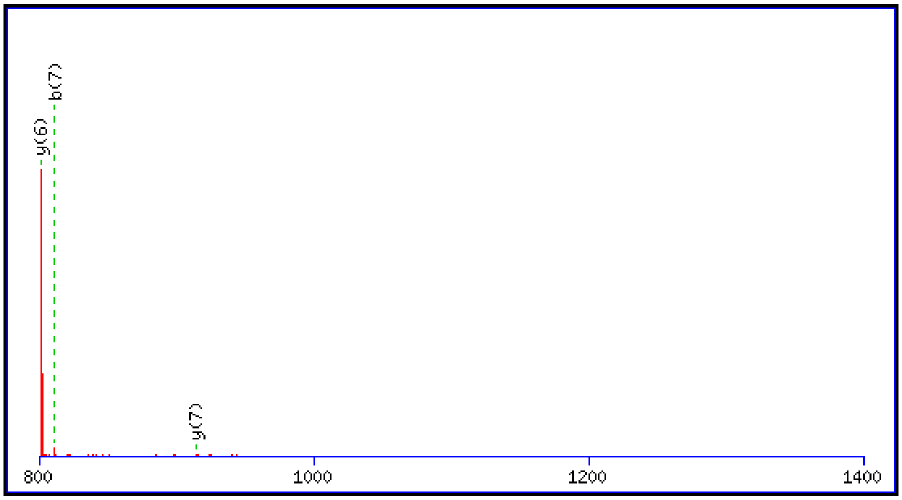

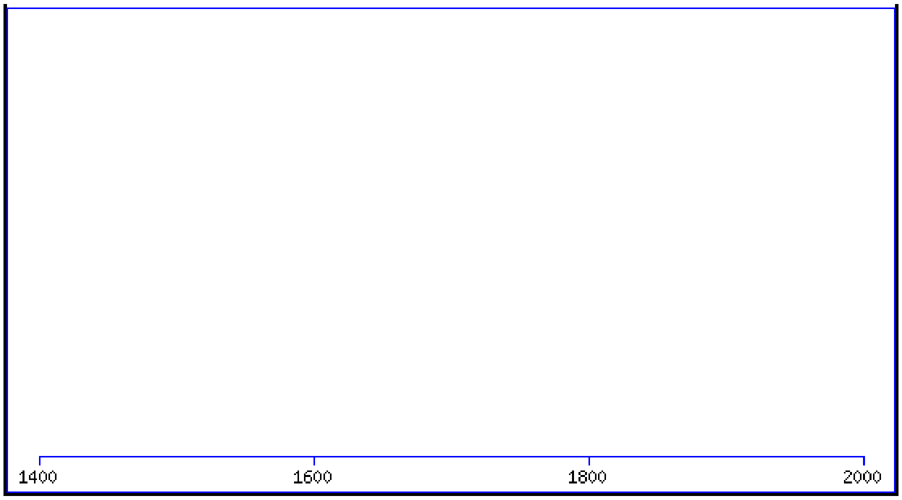

Monoisotopic mass of neutral peptide Mr(calc): 984.5029
Fixed modifications: Carbamidomethyl (C)
Ions Score: 50 **Expect:** 0.00091
Matches (Bold Red): 17/64 fragment ions using 29 most intense peaks

#	b	b++	b*	b*++	b0	b0++	Seq.	y	y++	y*	y*++	y0	y0++	#
1	72.0444	36.5258					A							8
2	**185.1285**	93.0679					I	**914.4730**	457.7402	897.4465	449.2269	896.4625	448.7349	7
3	348.1918	**174.5995**					Y	**801.3890**	**401.1981**	**784.3624**	**392.6849**	**783.3784**	**392.1928**	6
4	**476.2504**	238.6288	459.2238	230.1155			Q	**638.3257**	319.6665	621.2991	311.1532	620.3151	310.6612	5
5	547.2875	274.1474	530.2609	265.6341			A	**510.2671**	255.6372	493.2405	247.1239	492.2565	246.6319	4
6	**648.3352**	324.6712	631.3086	316.1579	630.3246	315.6659	T	**439.2300**	220.1186	422.2034	211.6053	421.2194	211.1133	3
7	**811.3985**	406.2029	794.3719	397.6896	793.3879	397.1976	Y	**338.1823**	169.5948	321.1557	161.0815			2
8							R	**175.1190**	88.0631	158.0924	79.5498			1

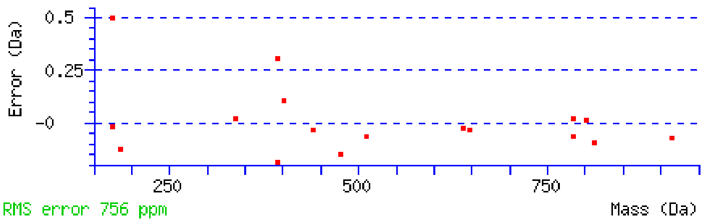

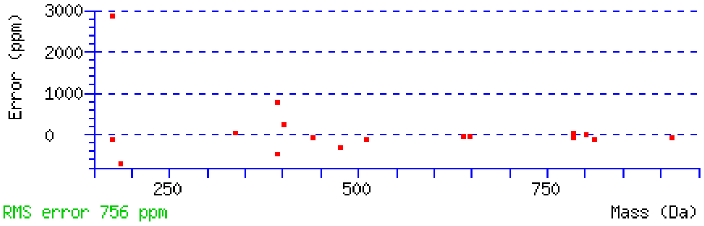

All matches to this query

Score	Mr(calc):	Delta	Sequence
50.5	984.5029	0.0013	AIYQATYR
7.8	984.4988	0.0053	SPVAGAADAAR
7.0	984.5018	0.0023	ALRSRFR
5.9	982.5018	2.0023	KCGKAYTR
5.4	984.5101	-0.0060	SPRQQVDR
5.0	983.4913	1.0128	RGRSTVTK
5.0	984.5045	-0.0004	ALYGLIQK
3.0	984.4988	0.0053	SSEKELHR
1.8	983.5036	1.0005	SLQESPAPR
0.9	984.5103	-0.0062	ALMFTQFK

Spectrum No: 28; Query: 59383; Rank: 1

Peptide View

MS/MS Fragmentation of **ATAGAYIASQTVK**
Found in **IPI00383971**, Tax_Id=9606 Gene_Symbol=PSMB5 proteasome beta 5 subunit isoform 3

Match to Query 59383: 1279.680608 from(640.847580,2+)
Title: 090702LimSK_Exosome2_04.3499.3499.2.dta
Data file C:\Temp\Mascoti\LSK_2D\090702LimSK_Exosome2_01_2D.mgf

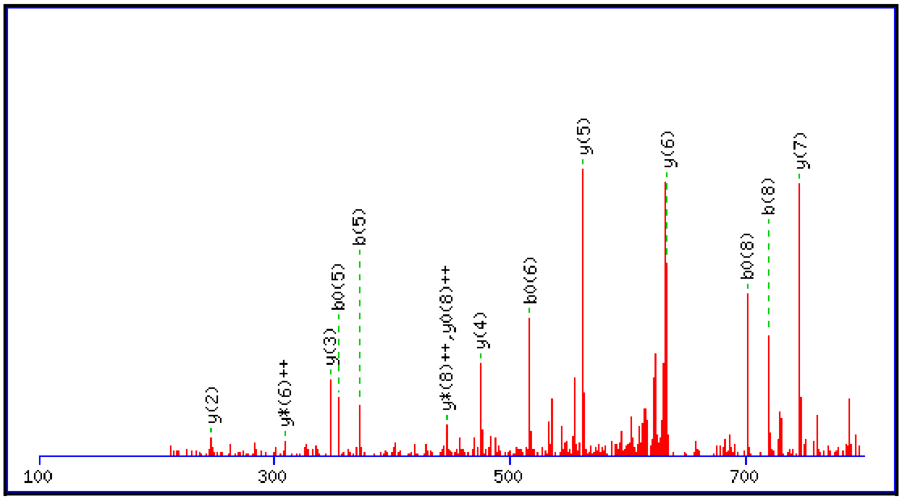

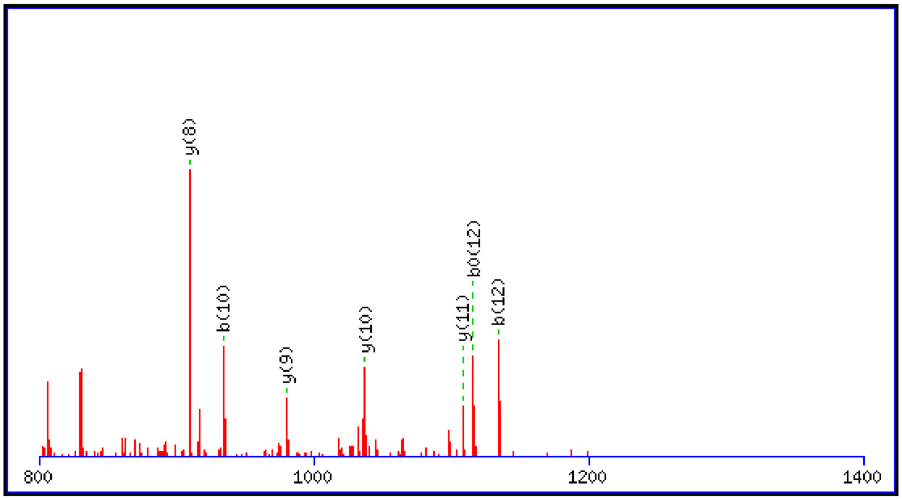

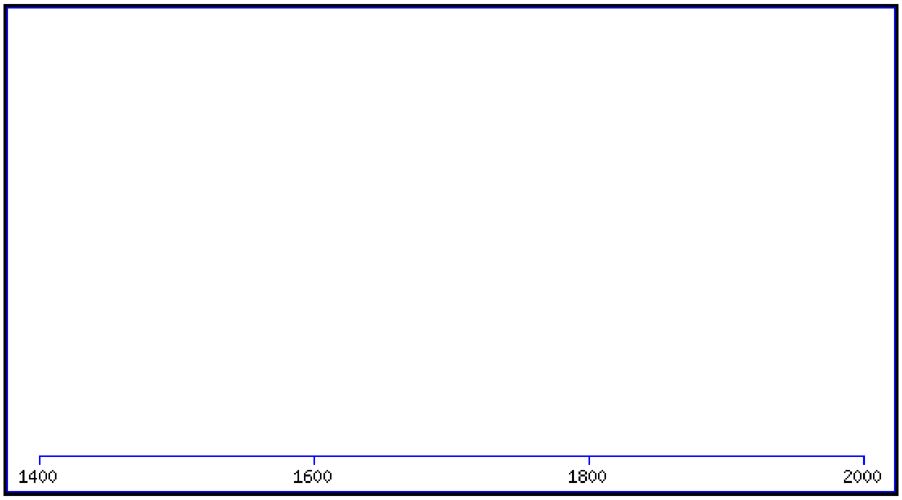

Monoisotopic mass of neutral peptide Mr(calc): 1279.6772
Fixed modifications: Carbamidomethyl (C)
Ions Score: 107 **Expect:** 2.8e-009
Matches (Bold Red): 21/120 fragment ions using 22 most intense peaks

		++		++	0	0++			++		++	0	0++	

#	b	b	b*	b*	b	b	Seq.	y	y	y*	y*	y	y	#
1	72.0444	36.5258					A							13
2	173.0921	87.0497			155.0815	78.0444	T	1209.6474	605.3273	1192.6208	596.8141	1191.6368	596.3220	12
3	244.1292	122.5682			226.1186	113.5629	A	**1108.5997**	554.8035	1091.5732	546.2902	1090.5891	545.7982	11
4	301.1506	151.0790			283.1401	142.0737	G	**1037.5626**	519.2849	1020.5360	510.7717	1019.5520	510.2796	10
5	**372.1878**	186.5975			**354.1772**	177.5922	A	**980.5411**	490.7742	963.5146	482.2609	962.5306	481.7689	9
6	535.2511	268.1292			**517.2405**	259.1239	Y	**909.5040**	455.2556	892.4775	**446.7424**	891.4934	**446.2504**	8
7	648.3352	324.6712			630.3246	315.6659	I	**746.4407**	373.7240	729.4141	365.2107	728.4301	364.7187	7
8	**719.3723**	360.1898			**701.3617**	351.1845	A	**633.3566**	317.1819	616.3301	**308.6687**	615.3461	308.1767	6
9	806.4043	403.7058			788.3937	394.7005	S	**562.3195**	281.6634	545.2930	273.1501	544.3089	272.6581	5
10	**934.4629**	467.7351	917.4363	459.2218	916.4523	458.7298	Q	**475.2875**	238.1474	458.2609	229.6341	457.2769	229.1421	4
11	1035.5106	518.2589	1018.4840	509.7456	1017.5000	509.2536	T	**347.2289**	174.1181	330.2023	165.6048	329.2183	165.1128	3
12	**1134.5790**	567.7931	1117.5524	559.2798	**1116.5684**	558.7878	V	**246.1812**	123.5942	229.1547	115.0810			2
13							K	147.1128	74.0600	130.0863	65.5468			1

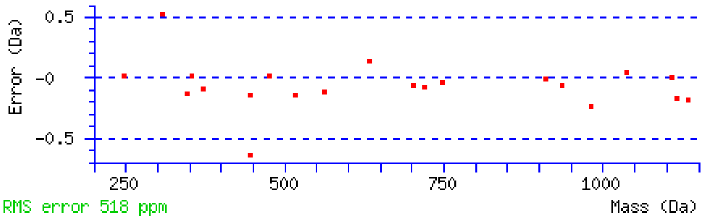

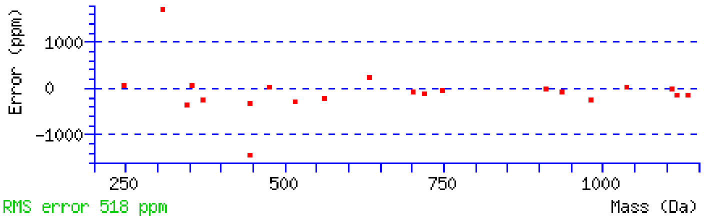

All matches to this query

Score	Mr(calc):	Delta	Sequence
107.1	1279.6772	0.0034	ATAGAYIASQTVK
10.9	1279.6901	-0.0095	KPTQASITKVK
6.9	1277.6723	2.0083	VMMAELEKTVK
6.4	1279.6772	0.0034	NVFTVSASSIQK
5.5	1279.6687	0.0120	GWQGRFRFAR
4.9	1279.6772	0.0034	TGGFTGNVSITVK
4.6	1277.6761	2.0045	ESKSRICATVK
4.2	1279.6901	-0.0095	KPTQASITKVK
2.0	1278.6849	0.9957	XHVITALRVK
1.5	1278.6697	1.0109	RPSLKTLQEK

Spectrum No: 29; Query: 64963; Rank: 1

Peptide View

MS/MS Fragmentation of **ATAGAYIASQTVKK**
Found in **IPI00383971**, Tax_Id=9606 Gene_Symbol=PSMB5 proteasome beta 5 subunit isoform 3

Match to Query 64963: 1407.770748 from(704.892650,2+)
Title: 090702LimSK_Exosome2_06.5066.5066.2.dta
Data file C:\Temp\Mascoti\LSK_2D\090702LimSK_Exosome2_01_2D.mgf

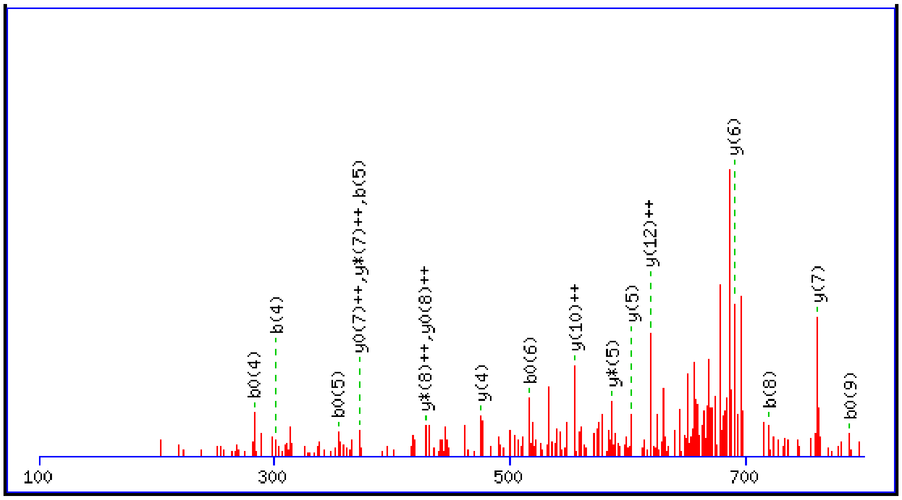

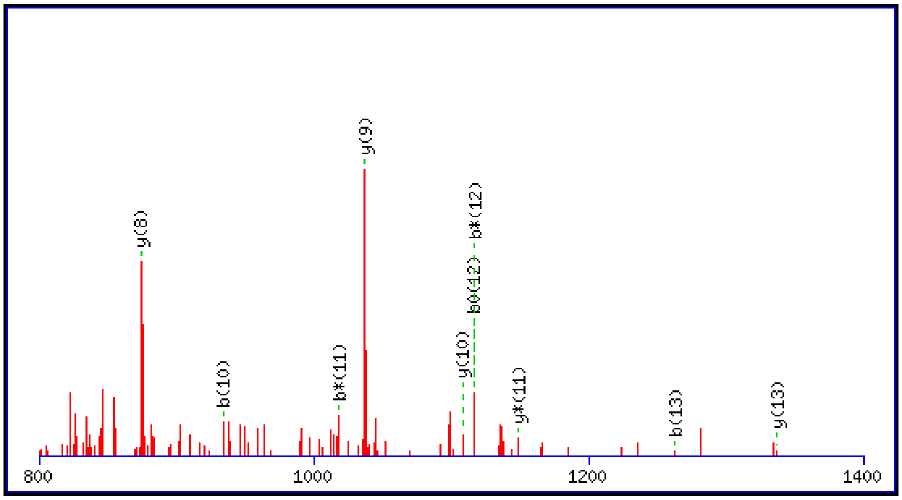

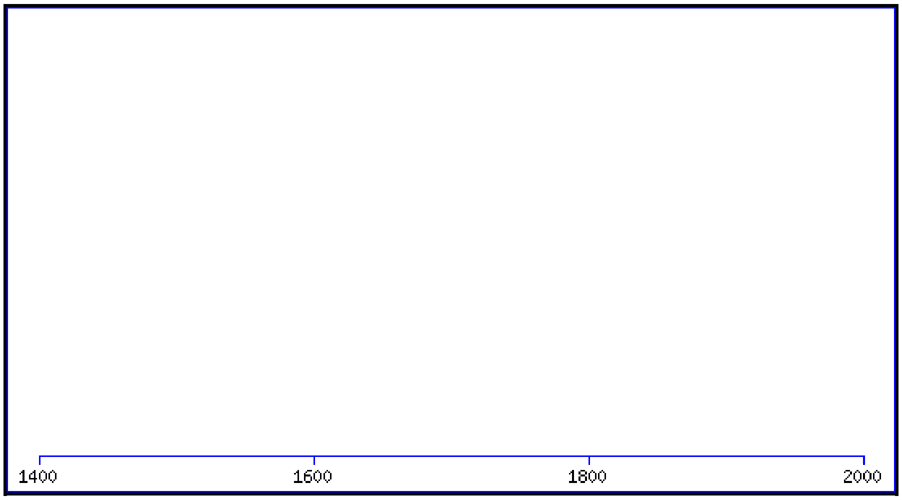

Monoisotopic mass of neutral peptide Mr(calc): 1407.7722
Fixed modifications: Carbamidomethyl (C)
Ions Score: 29 **Expect:** 0.11
Matches (Bold Red): 28/130 fragment ions using 69 most intense peaks

#	b	b^{++}	b*	b^{*++}	b^0	b^{0++}	Seq.	y	y^{++}	y*	y^{*++}	y^0	y^{0++}	#
1	72.0444	36.5258					A							14
2	173.0921	87.0497			155.0815	78.0444	T	**1337.7423**	669.3748	1320.7158	660.8615	1319.7318	660.3695	13
3	244.1292	122.5682			226.1186	113.5629	A	1236.6947	**618.8510**	1219.6681	610.3377	1218.6841	609.8457	12
4	**301.1506**	151.0790			**283.1401**	142.0737	G	1165.6576	583.3324	**1148.6310**	574.8191	1147.6470	574.3271	11
5	**372.1878**	186.5975			**354.1772**	177.5922	A	**1108.6361**	**554.8217**	1091.6095	546.3084	1090.6255	545.8164	10
6	535.2511	268.1292			**517.2405**	259.1239	Y	**1037.5990**	519.3031	1020.5724	510.7899	1019.5884	510.2978	9

7	648.3352	324.6712			630.3246	315.6659	I	**874.5356**	437.7715	857.5091	**429.2582**	856.5251	**428.7662**	8
8	**719.3723**	360.1898			701.3617	351.1845	A	**761.4516**	381.2294	744.4250	**372.7162**	743.4410	**372.2241**	7
9	806.4043	403.7058			**788.3937**	394.7005	S	**690.4145**	345.7109	673.3879	337.1976	672.4039	336.7056	6
10	**934.4629**	467.7351	917.4363	459.2218	916.4523	458.7298	Q	**603.3824**	302.1949	**586.3559**	293.6816	585.3719	293.1896	5
11	1035.5106	518.2589	**1018.4840**	509.7456	1017.5000	509.2536	T	**475.3239**	238.1656	458.2973	229.6523	457.3133	229.1603	4
12	1134.5790	567.7931	**1117.5524**	559.2798	**1116.5684**	558.7878	V	374.2762	187.6417	357.2496	179.1285			3
13	**1262.6739**	631.8406	1245.6474	623.3273	1244.6634	622.8353	K	275.2078	138.1075	258.1812	129.5942			2
14							K	147.1128	74.0600	130.0863	65.5468			1

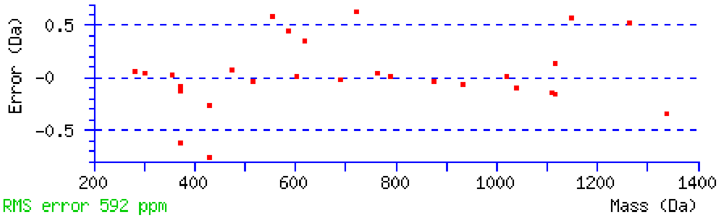

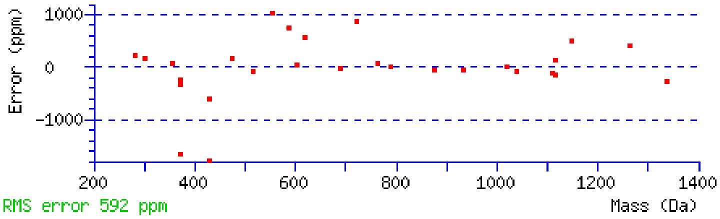

All matches to this query

Score	Mr(calc):	Delta	Sequence
29.4	1407.7722	-0.0014	ATAGAYIASQTVKK
1.3	1406.7686	1.0021	AILQGAKLIWSK
0.7	1405.7595	2.0113	XIRLKLIYDR
0.0	1406.7646	1.0061	SLLLKAVVSGNAR

Spectrum No: 30; Query: 70211; Rank: 1

Peptide View

MS/MS Fragmentation of **DAYSGGAVNLYHVR**
Found in **IPI00479306**, Tax_Id=9606 Gene_Symbol=PSMB5 Proteasome subunit beta type-5

Match to Query 70211: 1520.740588 from(761.377570,2+)
Title: 090702LimSK_Exosome2_01.3832.3832.2.dta
Data file C:\Temp\Mascoti\LSK_2D\090702LimSK_Exosome2_01_2D.mgf

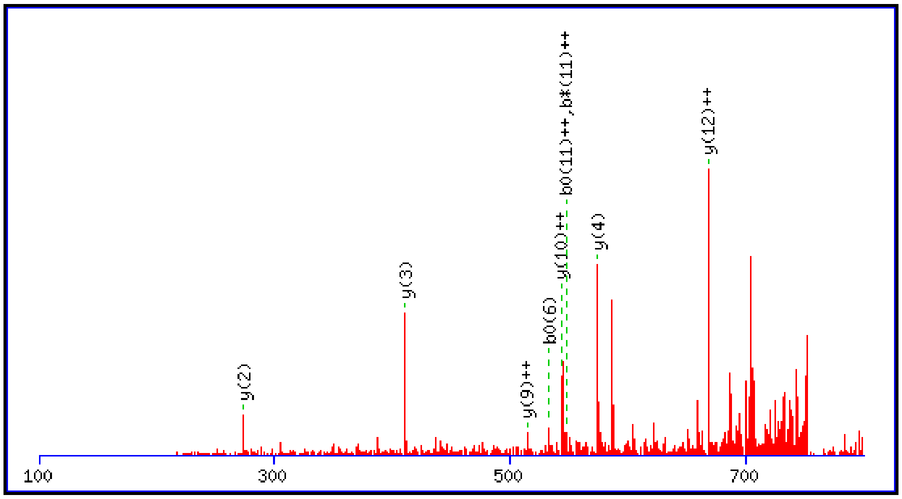

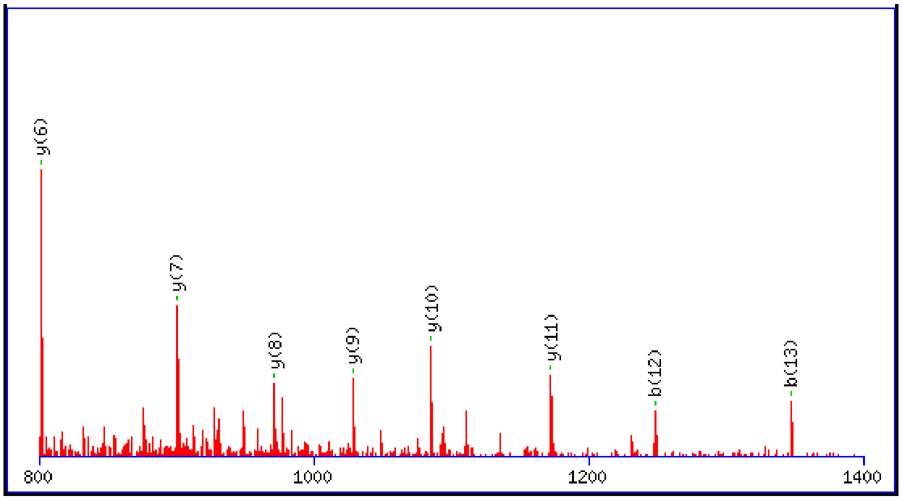

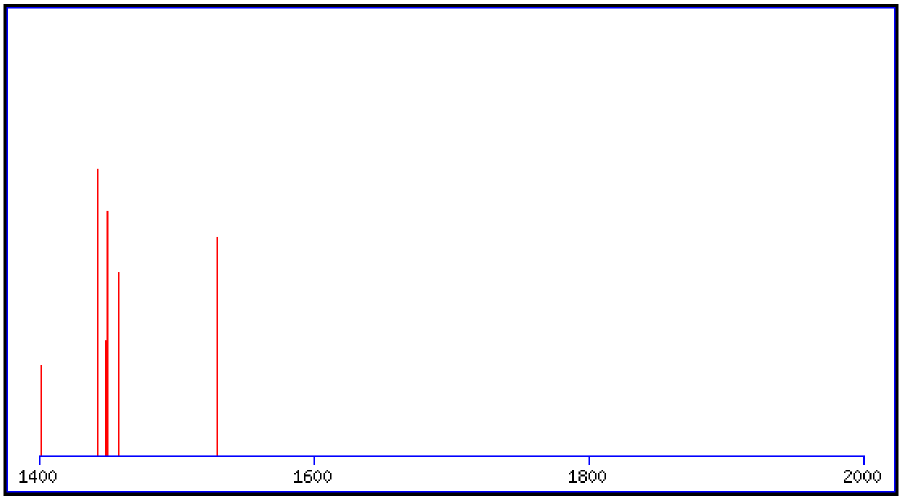

Monoisotopic mass of neutral peptide Mr(calc): 1520.7372
Fixed modifications: Carbamidomethyl (C)
Ions Score: 75 **Expect:** 6.9e-006
Matches (Bold Red): 17/120 fragment ions using 26 most intense peaks

#	b	b^{++}	b*	b^{*++}	b^{0}	b^{0++}	Seq.	y	y^{++}	y*	y^{*++}	y^{0}	y^{0++}	#
1	116.0342	58.5207			98.0237	49.5155	D							14
2	187.0713	94.0393			169.0608	85.0340	A	1406.7175	703.8624	1389.6910	695.3491	1388.7070	694.8571	13
3	350.1347	175.5710			332.1241	166.5657	Y	1335.6804	**668.3438**	1318.6539	659.8306	1317.6698	659.3386	12
4	437.1667	219.0870			419.1561	210.0817	S	**1172.6171**	586.8122	1155.5905	578.2989	1154.6065	577.8069	11
5	494.1882	247.5977			476.1776	238.5924	G	**1085.5851**	**543.2962**	1068.5585	534.7829			10
6	551.2096	276.1084			**533.1991**	267.1032	G	**1028.5636**	**514.7854**	1011.5370	506.2722			9
7	622.2467	311.6270			604.2362	302.6217	A	**971.5421**	486.2747	954.5156	477.7614			8
8	721.3151	361.1612			703.3046	352.1559	V	**900.5050**	450.7561	883.4785	442.2429			7
9	835.3581	418.1827	818.3315	409.6694	817.3475	409.1774	N	**801.4366**	401.2219	784.4100	392.7087			6
10	948.4421	474.7247	931.4156	466.2114	930.4316	465.7194	L	687.3937	344.2005	670.3671	335.6872			5
11	1111.5055	556.2564	1094.4789	**547.7431**	1093.4949	**547.2511**	Y	**574.3096**	287.6584	557.2831	279.1452			4
12	**1248.5644**	624.7858	1231.5378	616.2726	1230.5538	615.7805	H	**411.2463**	206.1268	394.2197	197.6135			3
13	**1347.6328**	674.3200	1330.6062	665.8068	1329.6222	665.3148	V	**274.1874**	137.5973	257.1608	129.0840			2
14							R	175.1190	88.0631	158.0924	79.5498			1

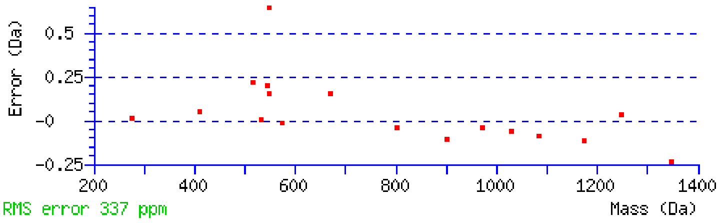

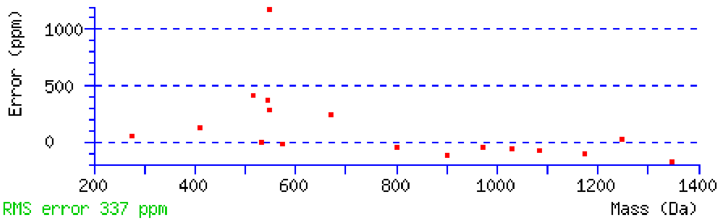

All matches to this query

Score	Mr(calc):	Delta	Sequence
74.7	1520.7372	0.0034	DAYSGGAVNLYHVR
9.9	1519.7297	1.0109	AFSAHSSLVTHKR
5.3	1519.7341	1.0065	LAEGEFTPEMQLR
3.8	1519.7274	1.0132	NGTASRGCADRSLR
3.6	1520.7406	0.0000	GLTCLASIWGSGGSR
3.0	1520.7501	-0.0095	IHSPSPHKQVPSK
1.2	1519.7267	1.0139	YPVDASASPAAAASSR
0.3	1519.7419	0.9986	WYKDGQPVTPSSR

Spectrum No: 31; Query: 65648; Rank: 1

Peptide View

MS/MS Fragmentation of **GPGLYYVDSEGNR**
Found in **IPI00479306**, Tax_Id=9606 Gene_Symbol=PSMB5 Proteasome subunit beta type-5

Match to Query 65648: 1425.654088 from(713.834320,2+)
Title: 090702LimSK_Exosome2_04.4492.4492.2.dta
Data file C:\Temp\Mascoti\LSK_2D\090702LimSK_Exosome2_01_2D.mgf

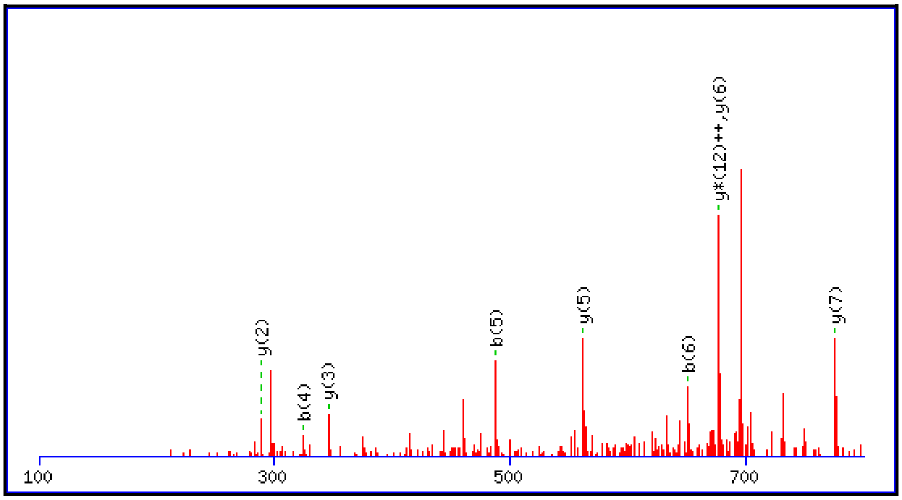

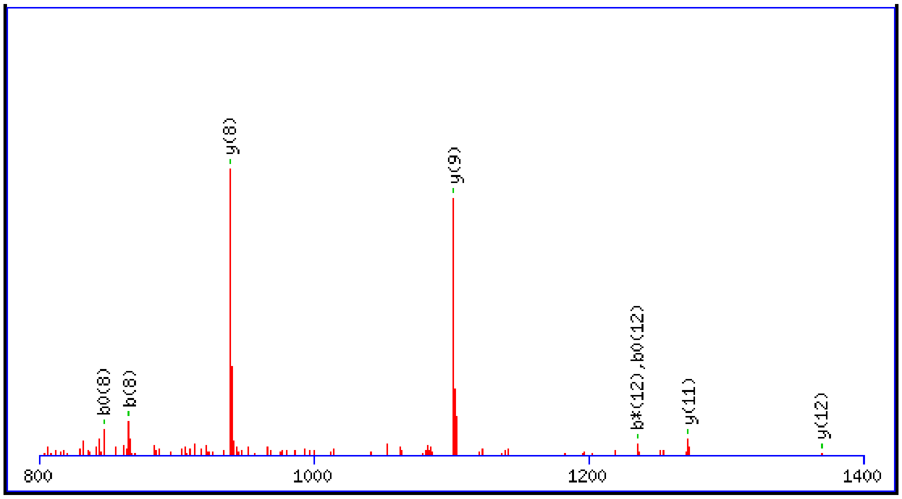

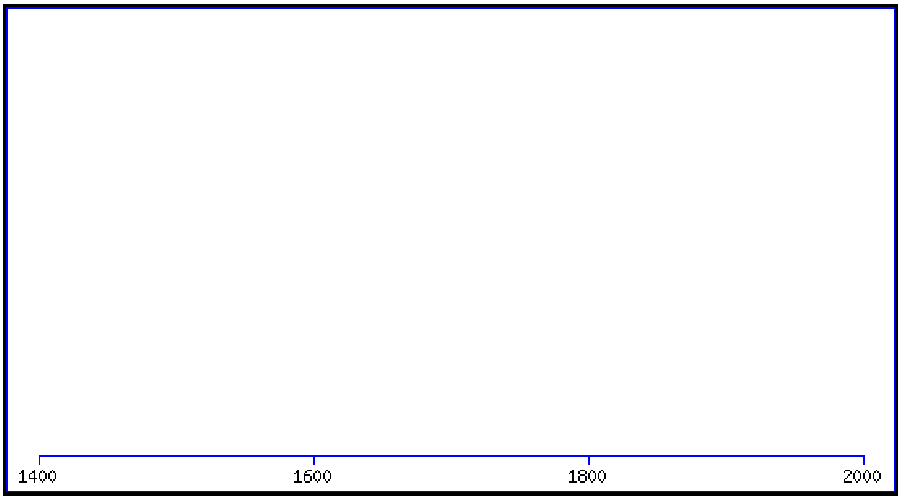

Monoisotopic mass of neutral peptide Mr(calc): 1425.6525
Fixed modifications: Carbamidomethyl (C)
Ions Score: 70 **Expect:** 2.2e-005
Matches (Bold Red): 17/102 fragment ions using 27 most intense peaks

#	b	b^{++}	b*	b^{*++}	b^{0}	b^{0++}	Seq.	y	y^{++}	y*	y^{*++}	y^{0}	y^{0++}	#
1	58.0287	29.5180					G							13
2	155.0815	78.0444					P	**1369.6383**	685.3228	1352.6117	**676.8095**	1351.6277	676.3175	12
3	212.1030	106.5551					G	**1272.5855**	636.7964	1255.5590	628.2831	1254.5749	627.7911	11
4	**325.1870**	163.0972					L	1215.5640	608.2857	1198.5375	599.7724	1197.5535	599.2804	10
5	**488.2504**	244.6288					Y	**1102.4800**	551.7436	1085.4534	543.2304	1084.4694	542.7383	9
6	**651.3137**	326.1605					Y	**939.4166**	470.2120	922.3901	461.6987	921.4061	461.2067	8
7	750.3821	375.6947					V	**776.3533**	388.6803	759.3268	380.1670	758.3428	379.6750	7
8	**865.4090**	433.2082			**847.3985**	424.2029	D	**677.2849**	339.1461	660.2584	330.6328	659.2743	330.1408	6
9	952.4411	476.7242			934.4305	467.7189	S	**562.2580**	281.6326	545.2314	273.1193	544.2474	272.6273	5
10	1081.4837	541.2455			1063.4731	532.2402	E	475.2259	238.1166	458.1994	229.6033	457.2154	229.1113	4
11	1138.5051	569.7562			1120.4946	560.7509	G	**346.1833**	173.5953	329.1568	165.0820			3
12	1252.5481	626.7777	**1235.5215**	618.2644	**1234.5375**	617.7724	N	**289.1619**	145.0846	272.1353	136.5713			2
13							R	175.1190	88.0631	158.0924	79.5498			1

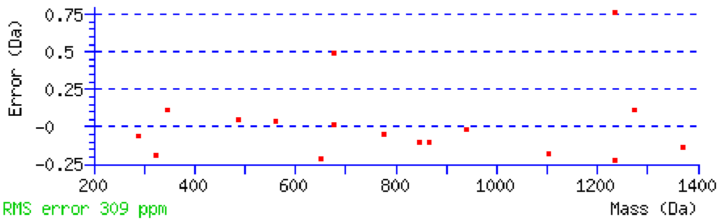

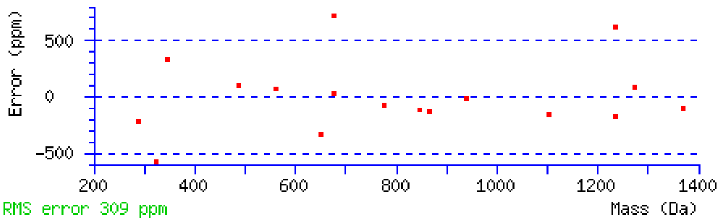

All matches to this query

Score	Mr(calc):	Delta	Sequence
69.6	1425.6525	0.0016	GPGLYYVDSEGNR
7.8	1423.6465	2.0076	XPPSTPRQMKR
7.8	1423.6465	2.0076	XPPSTPRQMKR
7.3	1425.6677	-0.0136	GQVTWFGYESPR
7.1	1425.6493	0.0048	CQGPAPSPSPCLR
4.6	1425.6592	-0.0051	SLSGCTDMSSILR
2.3	1423.6531	2.0010	SVTHANALTVMGK
2.2	1423.6531	2.0010	SVTHANALTVMGK

Spectrum No: 32; Query: 43990; Rank: 1

Peptide View

MS/MS Fragmentation of **HGVIVAADSR**
Found in **IPI00383971**, Tax_Id=9606 Gene_Symbol=PSMB5 proteasome beta 5 subunit isoform 3

Match to Query 43990: 1023.548968 from(512.781760,2+)
Title: 090702LimSK_Exosome2_06.3245.3245.2.dta
Data file C:\Temp\Mascoti\LSK_2D\090702LimSK_Exosome2_01_2D.mgf

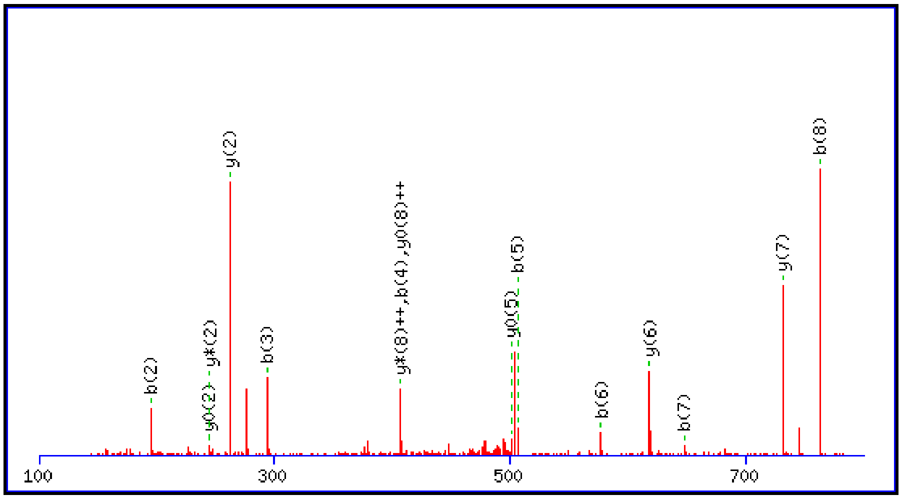

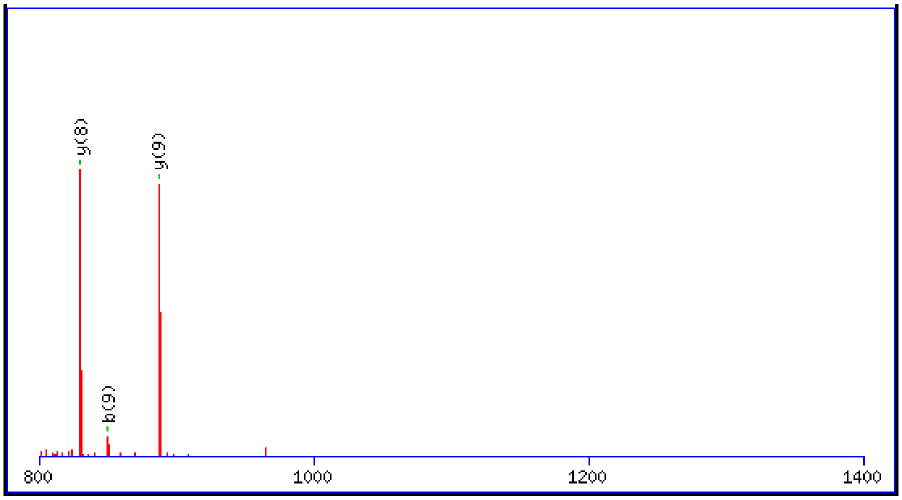

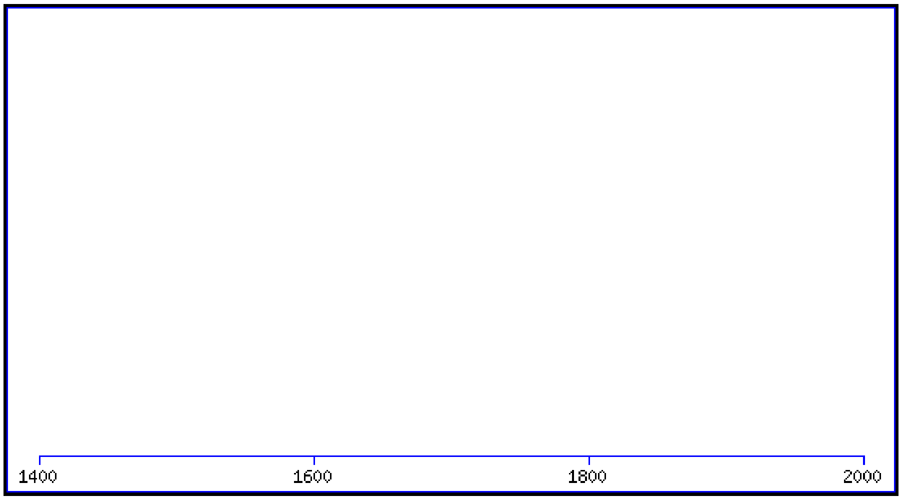

Monoisotopic mass of neutral peptide Mr(calc): 1023.5461
Fixed modifications: Carbamidomethyl (C)
Ions Score: 64 **Expect:** 3.9e-005
Matches (Bold Red): 18/74 fragment ions using 30 most intense peaks

#	b	b^{++}	b^{0}	b^{0++}	Seq.	y	y^{++}	y*	y^{*++}	y^{0}	y^{0++}	#
1	138.0662	69.5367			H							10
2	**195.0877**	98.0475			G	**887.4945**	444.2509	870.4680	435.7376	869.4839	435.2456	9
3	**294.1561**	147.5817			V	**830.4730**	415.7402	813.4465	**407.2269**	812.4625	**406.7349**	8
4	**407.2401**	204.1237			I	**731.4046**	366.2060	714.3781	357.6927	713.3941	357.2007	7
5	**506.3085**	253.6579			V	**618.3206**	309.6639	601.2940	301.1506	600.3100	300.6586	6
6	**577.3457**	289.1765			A	519.2522	260.1297	502.2256	251.6164	**501.2416**	251.1244	5
7	**648.3828**	324.6950			A	448.2150	224.6112	431.1885	216.0979	430.2045	215.6059	4
8	**763.4097**	382.2085	745.3991	373.2032	D	377.1779	189.0926	360.1514	180.5793	359.1674	180.0873	3
9	**850.4417**	425.7245	832.4312	416.7192	S	**262.1510**	131.5791	**245.1244**	123.0659	**244.1404**	122.5738	2
10					R	175.1190	88.0631	158.0924	79.5498			1

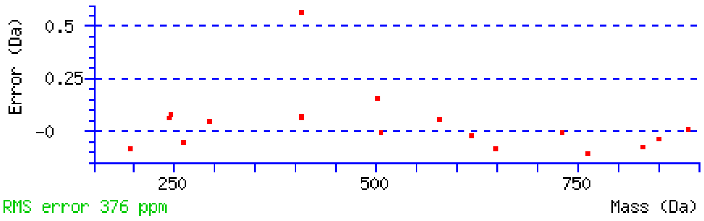

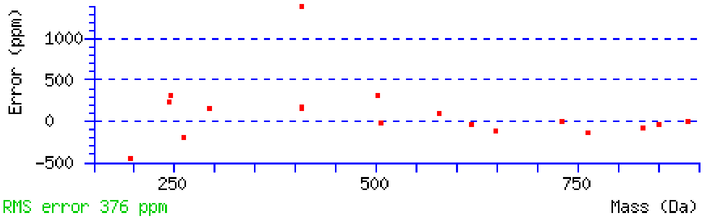

All matches to this query

Score	Mr(calc):	Delta	Sequence
64.4	1023.5461	0.0028	HGVIVAADSR
10.1	1023.5461	0.0028	YSRGVLSSR
9.9	1023.5389	0.0101	YIEIFPSR
9.1	1023.5423	0.0067	KDDKMLFK
7.8	1023.5569	-0.0079	MKVKMLSR
7.4	1021.5444	2.0046	GPAVPPELDK
4.4	1023.5461	0.0028	SSSHPLQLR
4.2	1021.5379	2.0111	YGGLKMPTR
3.4	1022.5470	1.0019	ISATMPLFK
3.0	1021.5474	2.0016	RHLYILK

Spectrum No: 33; Query: 29505; Rank: 1

Peptide View

MS/MS Fragmentation of **ISVAAASK**
Found in **IPI00383971**, Tax_Id=9606 Gene_Symbol=PSMB5 proteasome beta 5 subunit isoform 3

Match to Query 29505: 745.434448 from(373.724500,2+)
Title: 090702LimSK_Exosome2_06.942.942.2.dta
Data file C:\Temp\Mascoti\LSK_2D\090702LimSK_Exosome2_01_2D.mgf

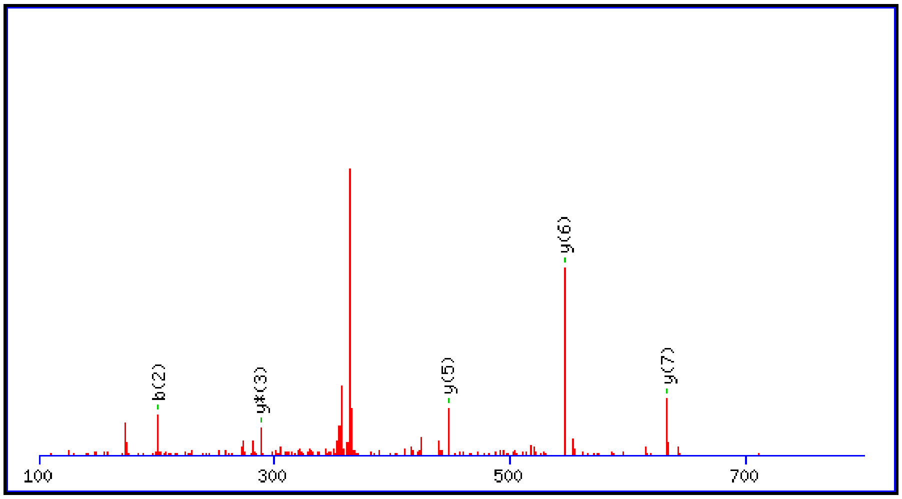

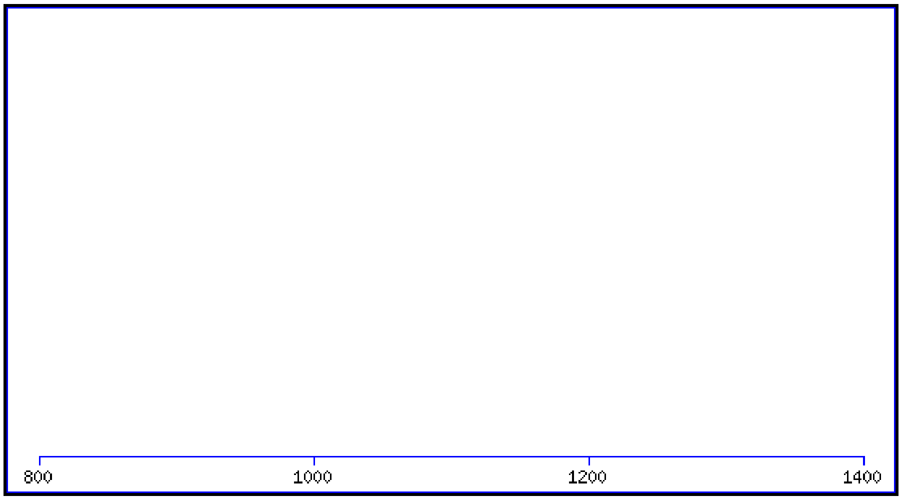

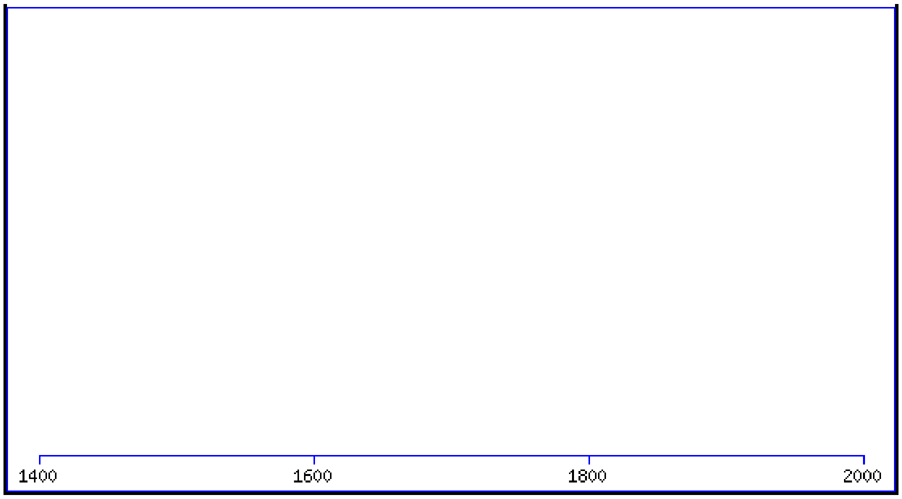

Monoisotopic mass of neutral peptide Mr(calc): 745.4334
Fixed modifications: Carbamidomethyl (C)
Ions Score: 21 **Expect:** 1.1
Matches (Bold Red): 5/66 fragment ions using 12 most intense peaks

#	b	b^{++}	b^{0}	b^{0++}	Seq.	y	y^{++}	y*	y^{*++}	y^{0}	y^{0++}	#
1	114.0913	57.5493			I							8
2	**201.1234**	101.0653	183.1128	92.0600	S	**633.3566**	317.1819	616.3301	308.6687	615.3461	308.1767	7
3	300.1918	150.5995	282.1812	141.5942	V	**546.3246**	273.6659	529.2980	265.1527	528.3140	264.6606	6
4	371.2289	186.1181	353.2183	177.1128	A	**447.2562**	224.1317	430.2296	215.6185	429.2456	215.1264	5
5	442.2660	221.6366	424.2554	212.6314	A	376.2191	188.6132	359.1925	180.0999	358.2085	179.6079	4
6	513.3031	257.1552	495.2926	248.1499	A	305.1819	153.0946	**288.1554**	144.5813	287.1714	144.0893	3
7	600.3352	300.6712	582.3246	291.6659	S	234.1448	117.5761	217.1183	109.0628	216.1343	108.5708	2
8					K	147.1128	74.0600	130.0863	65.5468			1

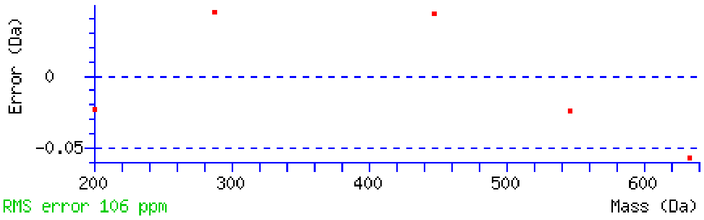

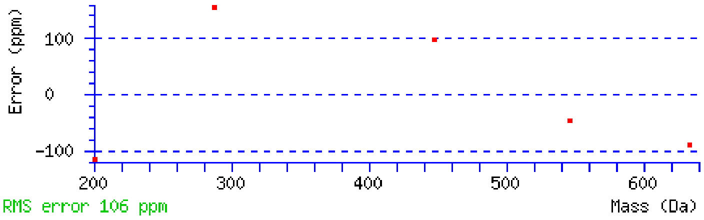

All matches to this query

Score	Mr(calc):	Delta	Sequence
20.9	745.4334	0.0011	ISVAAASK
18.4	745.4334	0.0011	LSSALQK
8.7	743.4290	2.0055	QSLQIR
7.8	743.4290	2.0055	TPVGSRK
7.7	745.4334	0.0011	LANSLTK
7.7	744.4242	1.0102	KVAQGSR
6.5	745.4334	0.0011	LVSKDGK
6.2	745.4334	0.0010	TVGTGLAK
6.1	743.4290	2.0055	XSGLALR
5.5	743.4290	2.0055	TVQQLR

Spectrum No: 34; Query: 57634; Rank: 1

Peptide View

MS/MS Fragmentation of **LLANMVYQYK**
Found in **IPI00383971**, Tax_Id=9606 Gene_Symbol=PSMB5 proteasome beta 5 subunit isoform 3

Match to Query 57634: 1241.649948 from(621.832250,2+)
Title: 090702LimSK_Exosome2_04.6492.6492.2.dta
Data file C:\Temp\Mascoti\LSK_2D\090702LimSK_Exosome2_01_2D.mgf

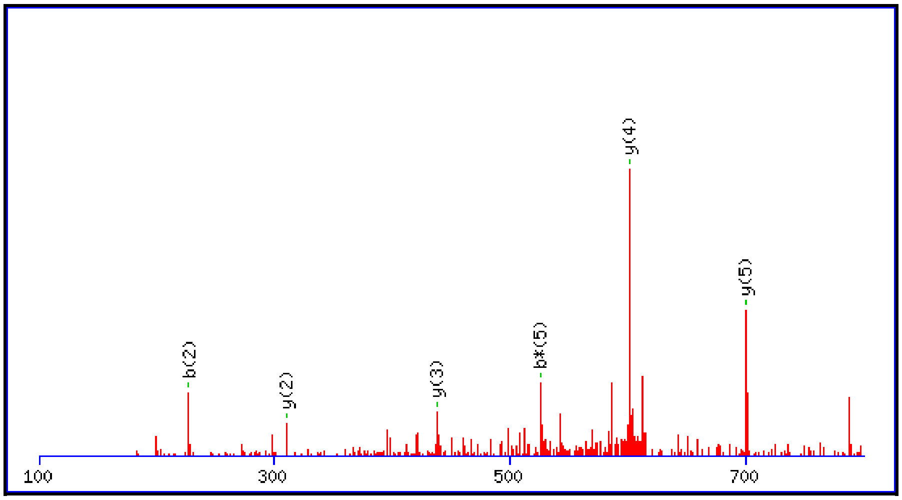

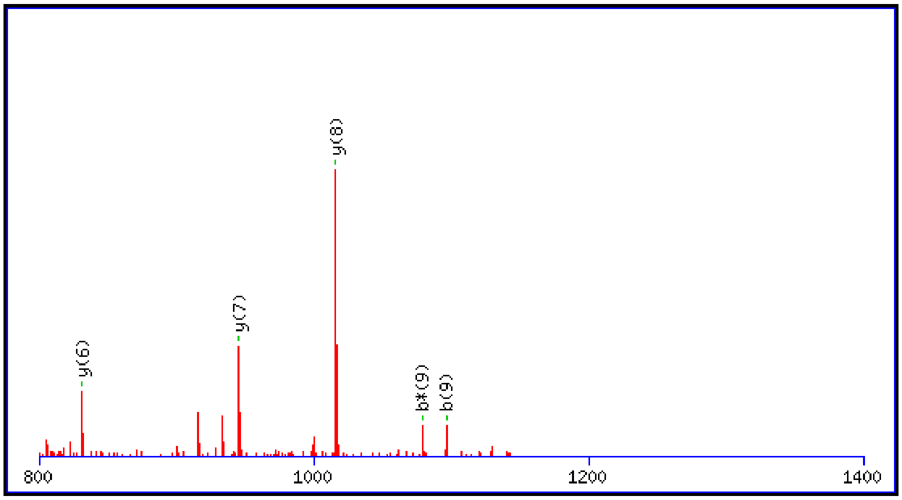

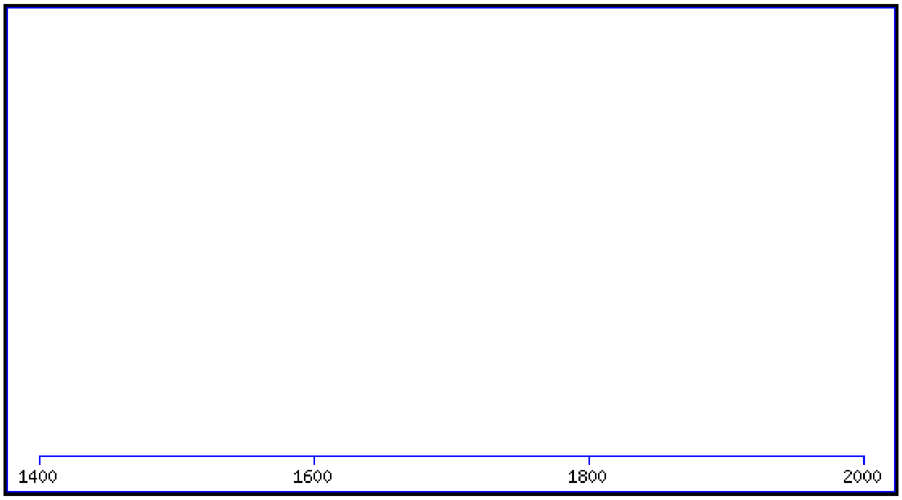

Monoisotopic mass of neutral peptide Mr(calc): 1241.6478
Fixed modifications: Carbamidomethyl (C)
Ions Score: 72 **Expect:** 7.5e-006
Matches (Bold Red): 11/66 fragment ions using 12 most intense peaks

		++		++			++		++	

#	b	b	b*	b*	Seq.	y	y	y*	y*	#
1	114.0913	57.5493			L					10
2	**227.1754**	114.0913			L	1129.5710	565.2892	1112.5445	556.7759	9
3	298.2125	149.6099			A	**1016.4870**	508.7471	999.4604	500.2339	8
4	412.2554	206.6314	395.2289	198.1181	N	**945.4499**	473.2286	928.4233	464.7153	7
5	543.2959	272.1516	**526.2694**	263.6383	M	**831.4069**	416.2071	814.3804	407.6938	6
6	642.3643	321.6858	625.3378	313.1725	V	**700.3665**	350.6869	683.3399	342.1736	5
7	805.4277	403.2175	788.4011	394.7042	Y	**601.2980**	301.1527	584.2715	292.6394	4
8	933.4863	467.2468	916.4597	458.7335	Q	**438.2347**	219.6210	421.2082	211.1077	3
9	**1096.5496**	548.7784	**1079.5230**	540.2652	Y	**310.1761**	155.5917	293.1496	147.0784	2
10					K	147.1128	74.0600	130.0863	65.5468	1

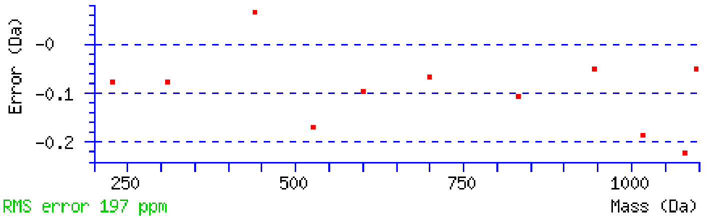

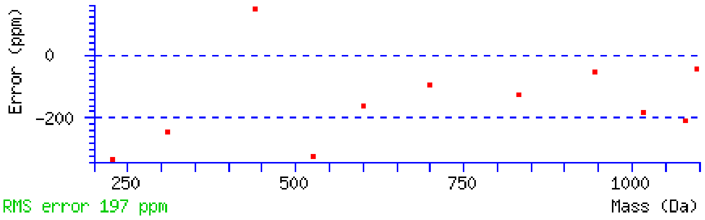

All matches to this query

Score	Mr(calc):	Delta	Sequence
72.0	1241.6478	0.0022	LLANMVYQYK
5.4	1240.6411	1.0088	EPVQALPSSASR
3.4	1241.6380	0.0119	SRTKEVITTK
1.6	1240.6524	0.9976	GRNPSSTPLGQK

Spectrum No: 35; Query: 51245; Rank: 1

Peptide View

MS/MS Fragmentation of **RAIYQATYR**
Found in **IPI00479306**, Tax_Id=9606 Gene_Symbol=PSMB5 Proteasome subunit beta type-5

Match to Query 51245: 1140.606568 from(571.310560,2+)
Title: 090702LimSK_Exosome2_06.5766.5766.2.dta
Data file C:\Temp\Mascoti\LSK_2D\090702LimSK_Exosome2_01_2D.mgf

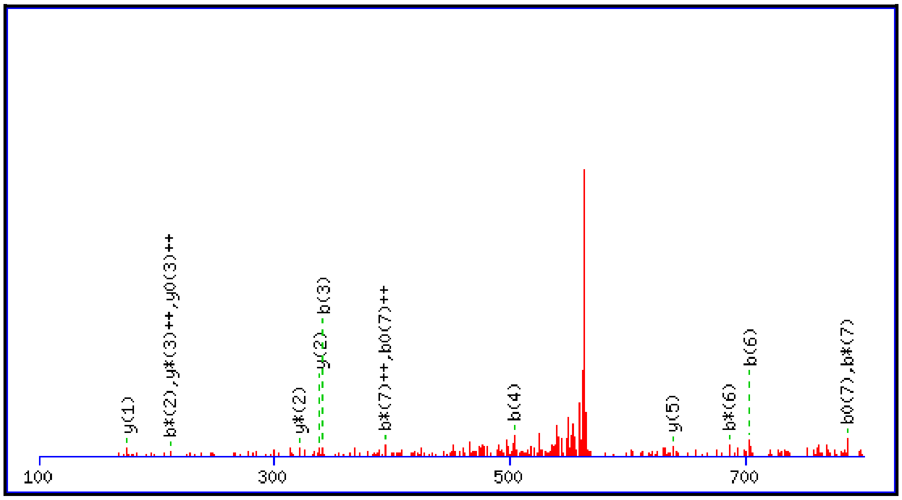

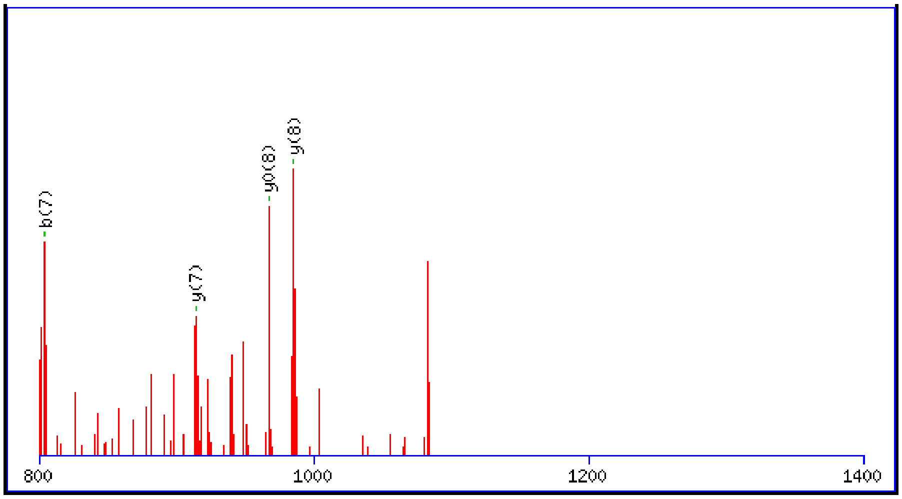

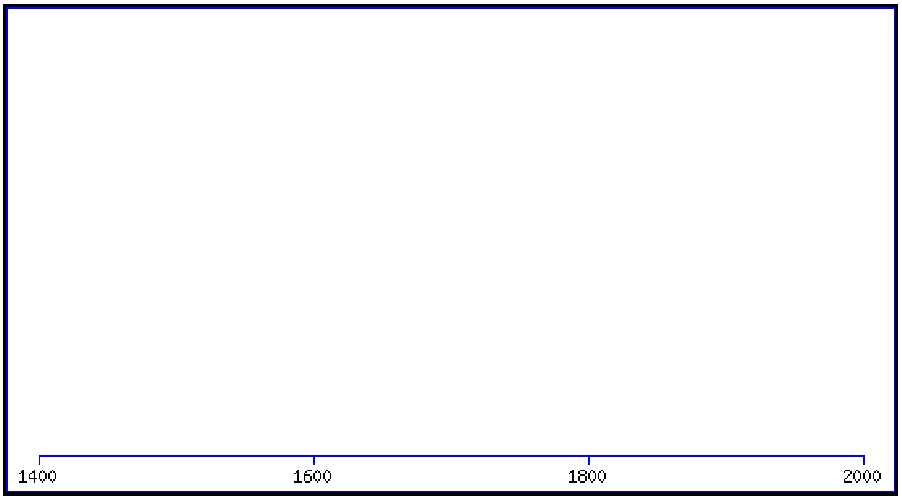

Monoisotopic mass of neutral peptide Mr(calc): 1140.6040
Fixed modifications: Carbamidomethyl (C)
Ions Score: 40 **Expect:** 0.012
Matches (Bold Red): 20/80 fragment ions using 42 most intense peaks

#	b	b^{++}	b*	b^{*++}	b^0	b^{0++}	Seq.	y	y^{++}	y*	y^{*++}	y^0	y^{0++}	#
1	157.1084	79.0578	140.0818	70.5446			R							9
2	228.1455	114.5764	**211.1190**	106.0631			A	**985.5102**	493.2587	968.4836	484.7454	**967.4996**	484.2534	8
3	**341.2296**	171.1184	324.2030	162.6051			I	**914.4730**	457.7402	897.4465	449.2269	896.4625	448.7349	7
4	**504.2929**	252.6501	487.2663	244.1368			Y	801.3890	401.1981	784.3624	392.6849	783.3784	392.1928	6
5	632.3515	316.6794	615.3249	308.1661			Q	**638.3257**	319.6665	621.2991	311.1532	620.3151	310.6612	5
6	**703.3886**	352.1979	**686.3620**	343.6847			A	510.2671	255.6372	493.2405	247.1239	492.2565	246.6319	4
7	**804.4363**	402.7218	**787.4097**	**394.2085**	**786.4257**	**393.7165**	T	439.2300	220.1186	422.2034	**211.6053**	421.2194	**211.1133**	3
8	**967.4996**	484.2534	950.4730	475.7402	949.4890	475.2482	Y	**338.1823**	169.5948	**321.1557**	161.0815			2
9							R	**175.1190**	88.0631	158.0924	79.5498			1

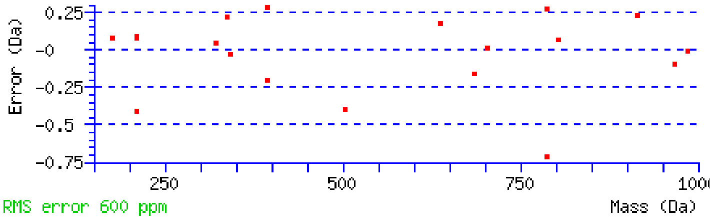

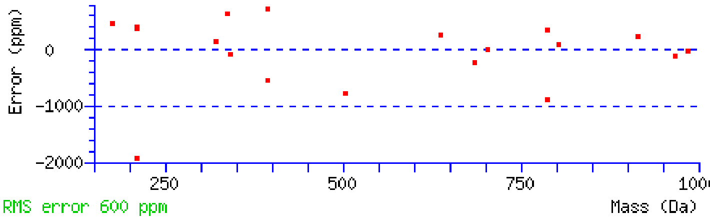

All matches to this query

Score	Mr(calc):	Delta	Sequence

39.8	1140.6040	0.0026	RAIYQATYR
15.8	1139.6087	0.9978	LLXPPPGNYR
13.3	1138.6095	1.9971	GPGKPLQEGTR
11.3	1140.6152	-0.0086	RSFLHAAPSR
8.8	1140.6139	-0.0073	LLQAPDTDLR
7.8	1140.5961	0.0105	YSSQIMLKR
7.5	1140.6090	-0.0024	LKGSLKMLR
6.0	1140.6056	0.0009	IIQKVFASR
5.9	1139.5975	1.0091	GDYLKELFR
5.8	1139.5951	1.0115	LLKTLETSR

Spectrum No: 36; Query: 73367; Rank: 1

Peptide View

MS/MS Fragmentation of **RGPGLYYVDSEGNR**
Found in **IPI00479306**, Tax_Id=9606 Gene_Symbol=PSMB5 Proteasome subunit beta type-5

Match to Query 73367: 1582.758612 from(528.593480,3+)
Title: 090702LimSK_Exosome2_05.7714.7714.3.dta
Data file C:\Temp\Mascoti\LSK_2D\090702LimSK_Exosome2_01_2D.mgf

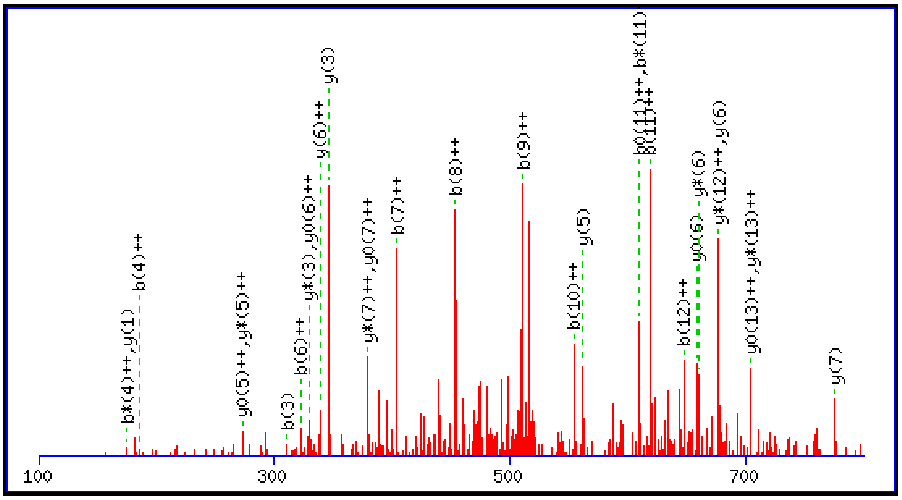

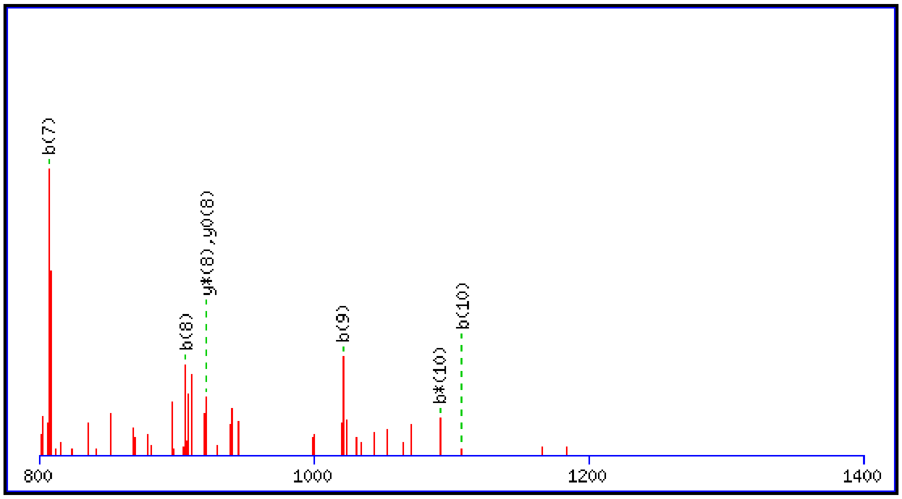

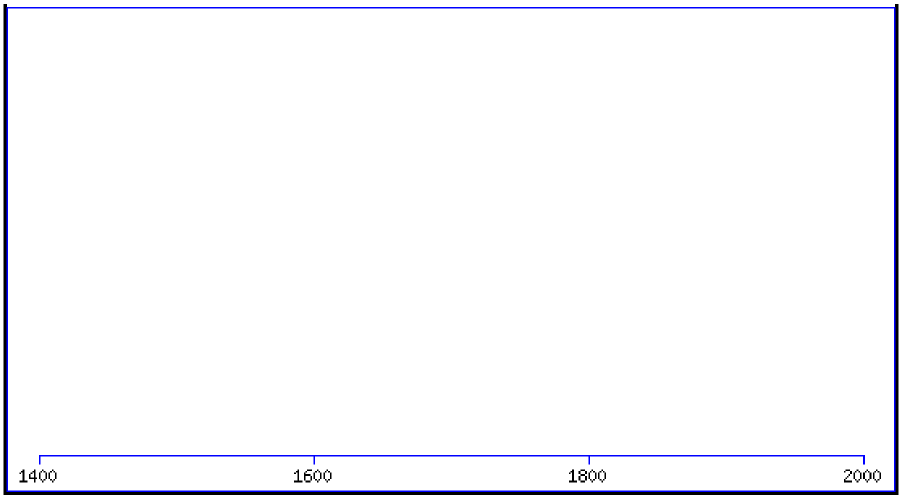

Monoisotopic mass of neutral peptide Mr(calc): 1581.7536
Fixed modifications: Carbamidomethyl (C)
Ions Score: 57 **Expect:** 0.00044
Matches (Bold Red): 37/134 fragment ions using 49 most intense peaks

#	b	b++	b*	b*++	b0	b0++	Seq.	y	y++	y*	y*++	y0	y0++	#
1	157.1084	79.0578	140.0818	70.5446			R							14
2	214.1299	107.5686	197.1033	99.0553			G	1426.6597	713.8335	1409.6332	**705.3202**	1408.6492	**704.8282**	13
3	**311.1826**	156.0949	294.1561	147.5817			P	1369.6383	685.3228	1352.6117	**676.8095**	1351.6277	676.3175	12
4	368.2041	**184.6057**	351.1775	**176.0924**			G	1272.5855	636.7964	1255.5590	628.2831	1254.5749	627.7911	11
5	481.2881	241.1477	464.2616	232.6344			L	1215.5640	608.2857	1198.5375	599.7724	1197.5535	599.2804	10
6	644.3515	**322.6794**	627.3249	314.1661			Y	1102.4800	551.7436	1085.4534	543.2304	1084.4694	542.7383	9
7	**807.4148**	**404.2110**	790.3883	395.6978			Y	939.4166	470.2120	**922.3901**	461.6987	**921.4061**	461.2067	8
8	**906.4832**	**453.7452**	889.4567	445.2320			V	**776.3533**	388.6803	759.3268	**380.1670**	758.3428	**379.6750**	7
9	**1021.5102**	**511.2587**	1004.4836	502.7454	1003.4996	502.2534	D	**677.2849**	**339.1461**	**660.2584**	330.6328	**659.2743**	**330.1408**	6
10	**1108.5422**	**554.7747**	**1091.5156**	546.2615	1090.5316	545.7694	S	**562.2580**	281.6326	545.2314	**273.1193**	544.2474	**272.6273**	5
11	1237.5848	**619.2960**	1220.5582	**610.7828**	1219.5742	**610.2907**	E	475.2259	238.1166	458.1994	229.6033	457.2154	229.1113	4
12	1294.6062	**647.8068**	1277.5797	639.2935	1276.5957	638.8015	G	**346.1833**	173.5953	**329.1568**	165.0820			3
13	1408.6492	**704.8282**	1391.6226	696.3149	1390.6386	695.8229	N	289.1619	145.0846	272.1353	136.5713			2
14							R	**175.1190**	88.0631	158.0924	79.5498			1

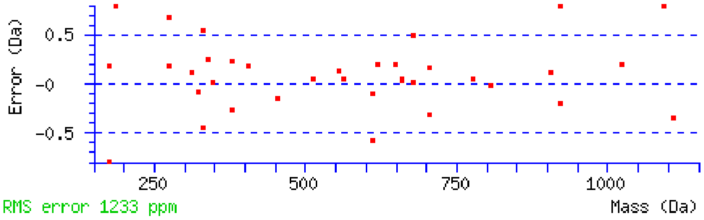

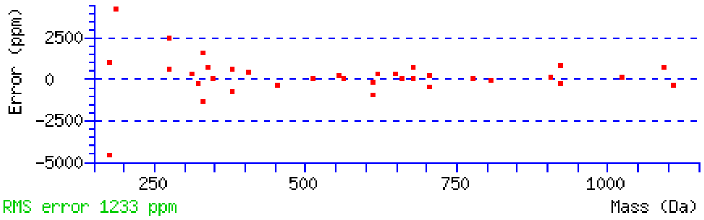

All matches to this query

Score	Mr(calc):	Delta	Sequence
56.6	1581.7536	1.0051	RGPGLYYVDSEGNR
18.4	1581.7651	0.9935	SLPPSDSKKTVESK
18.4	1581.7651	0.9935	XLPPSDSKKTVESK
16.2	1581.7651	0.9935	SLPPSDSKKTVESK
16.2	1581.7651	0.9935	XLPPSDSKKTVESK
10.5	1580.7364	2.0222	SPSKTYIQLKTR
10.5	1582.7483	0.0103	SVTEMEVMQFLNR
9.8	1582.7505	0.0082	GNLSNLVFLDAAGGR

7.7	1581.7651	0.9935	DDALKEVNTLKEK
7.2	1580.7426	2.0160	MVVEVDSMPAASSVK

Spectrum No: 37; Query: 61021; Rank: 1

Peptide View

MS/MS Fragmentation of **VSSDNVADLHEK**
Found in **IPI00479306**, Tax_Id=9606 Gene_Symbol=PSMB5 Proteasome subunit beta type-5

Match to Query 61021: 1312.630108 from(657.322330,2+)
Title: 090702LimSK_Exosome2_06.3316.3316.2.dta
Data file C:\Temp\Mascoti\LSK_2D\090702LimSK_Exosome2_01_2D.mgf

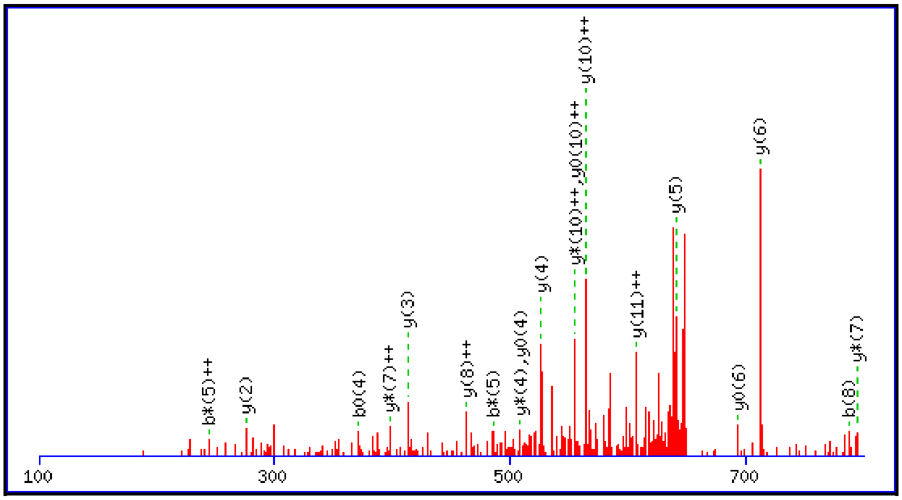

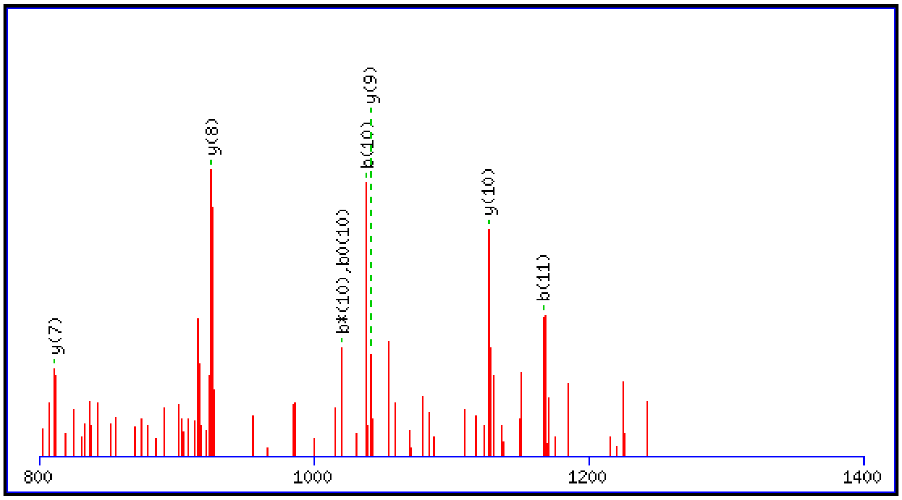

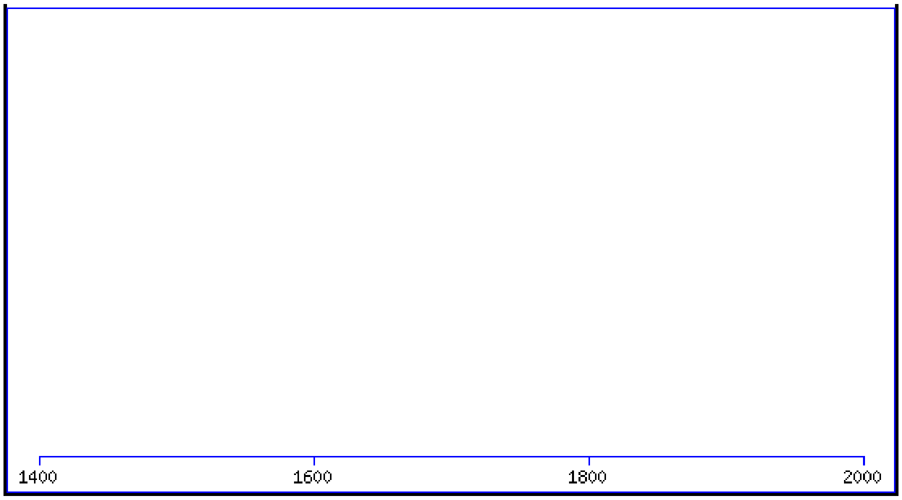

Monoisotopic mass of neutral peptide Mr(calc): 1312.6259
Fixed modifications: Carbamidomethyl (C)
Ions Score: 57 **Expect:** 0.00032
Matches (Bold Red): 27/120 fragment ions using 50 most intense peaks

#	b	b^{++}	b*	b^{*++}	b^0	b^{0++}	Seq.	y	y^{++}	y*	y^{*++}	y^0	y^{0++}	#
1	100.0757	50.5415					V							12
2	187.1077	94.0575			169.0972	85.0522	S	1214.5648	**607.7860**	1197.5382	599.2727	1196.5542	598.7807	11
3	274.1397	137.5735			256.1292	128.5682	S	**1127.5327**	**564.2700**	1110.5062	**555.7567**	1109.5222	**555.2647**	10
4	389.1667	195.0870			**371.1561**	186.0817	D	**1040.5007**	520.7540	1023.4742	512.2407	1022.4901	511.7487	9
5	503.2096	252.1084	**486.1831**	**243.5952**	485.1991	243.1032	N	**925.4738**	**463.2405**	908.4472	454.7272	907.4632	454.2352	8
6	602.2780	301.6427	585.2515	293.1294	584.2675	292.6374	V	**811.4308**	406.2191	**794.4043**	**397.7058**	793.4203	397.2138	7
7	673.3151	337.1612	656.2886	328.6479	655.3046	328.1559	A	**712.3624**	356.6849	695.3359	348.1716	**694.3519**	347.6796	6
8	**788.3421**	394.6747	771.3155	386.1614	770.3315	385.6694	D	**641.3253**	321.1663	624.2988	312.6530	623.3148	312.1610	5
9	901.4262	451.2167	884.3996	442.7034	883.4156	442.2114	L	**526.2984**	263.6528	**509.2718**	255.1396	**508.2878**	254.6475	4
10	**1038.4851**	519.7462	**1021.4585**	511.2329	**1020.4745**	510.7409	H	**413.2143**	207.1108	396.1878	198.5975	395.2037	198.1055	3
11	**1167.5277**	584.2675	1150.5011	575.7542	1149.5171	575.2622	E	**276.1554**	138.5813	259.1288	130.0681	258.1448	129.5761	2
12							K	147.1128	74.0600	130.0863	65.5468			1

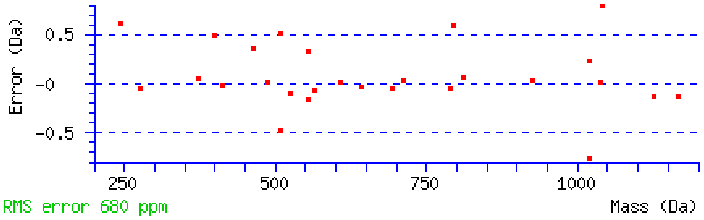

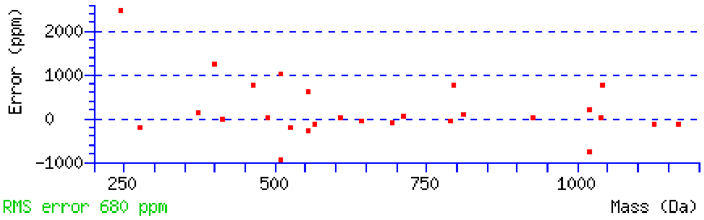

All matches to this query

Score	Mr(calc):	Delta	Sequence
57.2	1312.6259	0.0042	VSSDNVADLHEK
12.0	1310.6116	2.0185	SPNRFDAHDPR
7.8	1312.6275	0.0026	SVKATETEQLK
7.7	1312.6275	0.0026	SVKATETEQLK
7.4	1312.6428	-0.0127	GAFLDKPKTEK
5.5	1311.6296	1.0005	ARSTQGEVTRK
5.5	1311.6296	1.0005	ARSTQGEVTRK
4.4	1310.6119	2.0182	SPDSDVAATLKK
4.1	1312.6340	-0.0039	SVRMASSRMTR
3.7	1311.6354	0.9947	SGNSAAVFMRTR

Spectrum No: 38; Query: 76670; Rank: 1

Peptide View

MS/MS Fragmentation of **ALRETLPAEQDLTTK**
Found in **IPI00016832**, Tax_Id=9606 Gene_Symbol=PSMA1 Isoform Short of Proteasome subunit alpha type-1

Match to Query 76670: 1684.904772 from(562.642200,3+)
Title: 090702LimSK_Exosome2_05.7138.7138.3.dta
Data file C:\Temp\Mascoti\LSK_2D\090702LimSK_Exosome2_01_2D.mgf

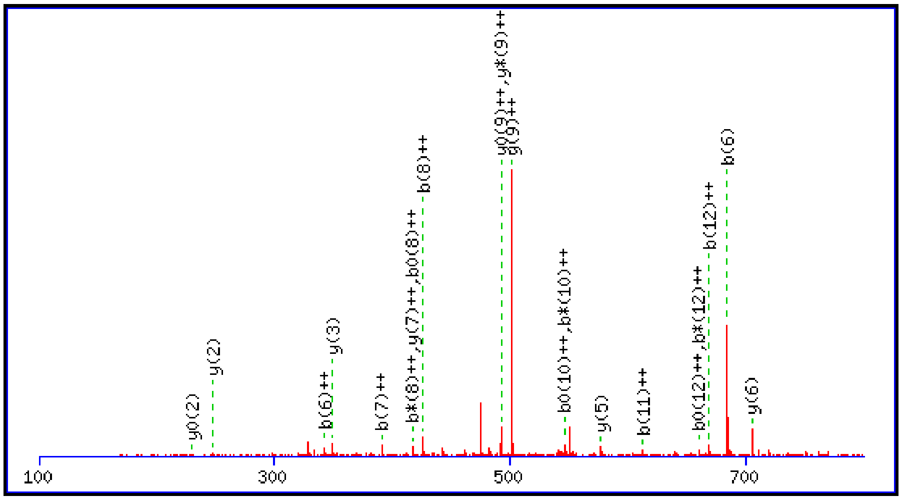

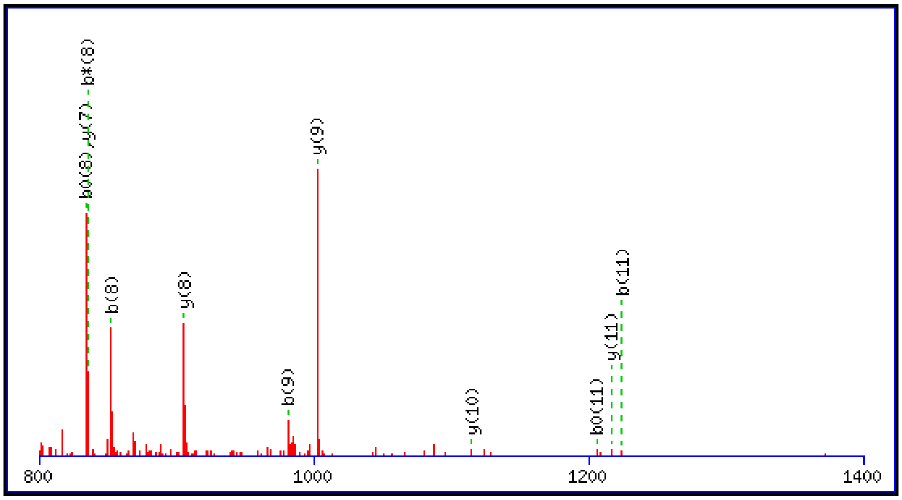

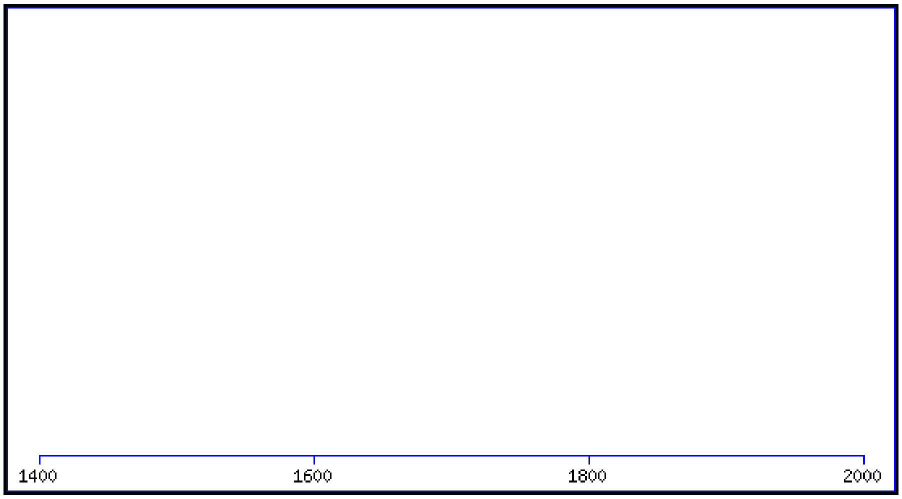

Monoisotopic mass of neutral peptide Mr(calc): 1684.8995
Fixed modifications: Carbamidomethyl (C)
Ions Score: 62 **Expect:** 6.9e-005
Matches (Bold Red): 32/156 fragment ions using 42 most intense peaks

#	b	b^{++}	b*	b^{*++}	b^{0}	b^{0++}	Seq.	y	y^{++}	y*	y^{*++}	y^{0}	y^{0++}	#
1	72.0444	36.5258					A							15
2	185.1285	93.0679					L	1614.8697	807.9385	1597.8432	799.4252	1596.8592	798.9332	14
3	341.2296	171.1184	324.2030	162.6051			R	1501.7857	751.3965	1484.7591	742.8832	1483.7751	742.3912	13
4	470.2722	235.6397	453.2456	227.1264	452.2616	226.6344	E	1345.6846	673.3459	1328.6580	664.8326	1327.6740	664.3406	12
5	571.3198	286.1636	554.2933	277.6503	553.3093	277.1583	T	**1216.6420**	608.8246	1199.6154	600.3113	1198.6314	599.8193	11
6	**684.4039**	**342.7056**	667.3774	334.1923	666.3933	333.7003	L	**1115.5943**	558.3008	1098.5677	549.7875	1097.5837	549.2955	10
7	781.4567	**391.2320**	764.4301	382.7187	763.4461	382.2267	P	**1002.5102**	**501.7587**	985.4837	**493.2455**	984.4997	**492.7535**	9
8	**852.4938**	**426.7505**	**835.4672**	**418.2373**	**834.4832**	**417.7452**	A	**905.4575**	453.2324	888.4309	444.7191	887.4469	444.2271	8
9	**981.5364**	491.2718	964.5098	482.7585	963.5258	482.2665	E	**834.4203**	**417.7138**	817.3938	409.2005	816.4098	408.7085	7
10	1109.5949	555.3011	1092.5684	**546.7878**	1091.5844	**546.2958**	Q	**705.3777**	353.1925	688.3512	344.6792	687.3672	344.1872	6
11	**1224.6219**	**612.8146**	1207.5953	604.3013	**1206.6113**	603.8093	D	**577.3192**	289.1632	560.2926	280.6499	559.3086	280.1579	5
12	1337.7060	**669.3566**	1320.6794	**660.8433**	1319.6954	**660.3513**	L	462.2922	231.6498	445.2657	223.1365	444.2817	222.6445	4
13	1438.7536	719.8805	1421.7271	711.3672	1420.7431	710.8752	T	**349.2082**	175.1077	332.1816	166.5944	331.1976	166.1024	3
14	1539.8013	770.4043	1522.7748	761.8910	1521.7907	761.3990	T	**248.1605**	124.5839	231.1339	116.0706	**230.1499**	115.5786	2
15							K	147.1128	74.0600	130.0863	65.5468			1

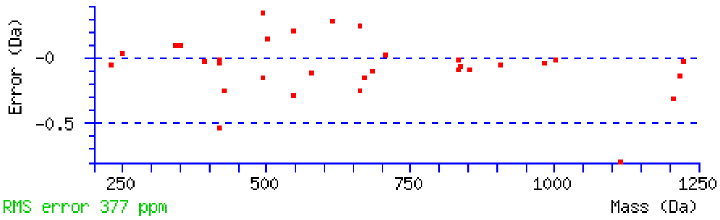

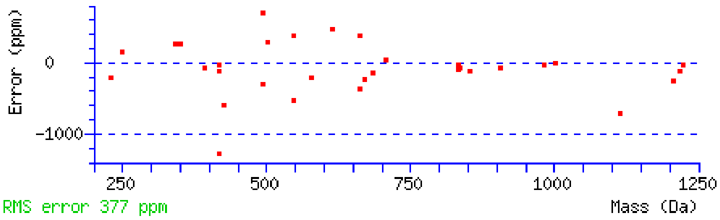

All matches to this query

Score	Mr(calc):	Delta	Sequence
62.0	1684.8995	0.0052	ALRETLPAEQDLTTK
14.6	1684.8892	0.0156	MKQISTKFLLMDSK
8.6	1683.9056	0.9991	VRSHGQDSPAVYLKK
8.4	1683.8944	1.0103	GEQGPPGIPGPQGLPGVK
8.0	1684.9182	-0.0134	TDPSILGGMIVRIGEK
7.8	1684.8937	0.0111	WLNKYFSLGNSKTK
7.5	1683.8960	1.0087	ELKELASRVAFLTK
7.1	1683.8961	1.0087	DLLLSLVVPAPSQPR
6.4	1682.9104	1.9944	APQAPFAGLEQLSKAR
5.7	1683.8960	1.0087	ELKELASRVAFLTK

Spectrum No: 39; Query: 27154; Rank: 1

Peptide View

MS/MS Fragmentation of **AMSIGAR**
Found in **IPI00016832**, Tax_Id=9606 Gene_Symbol=PSMA1 Isoform Short of Proteasome subunit alpha type-1

Match to Query 27154: 704.365128 from(353.189840,2+)
Title: 090702LimSK_Exosome2_05.1354.1354.2.dta
Data file C:\Temp\Mascoti\LSK_2D\090702LimSK_Exosome2_01_2D.mgf

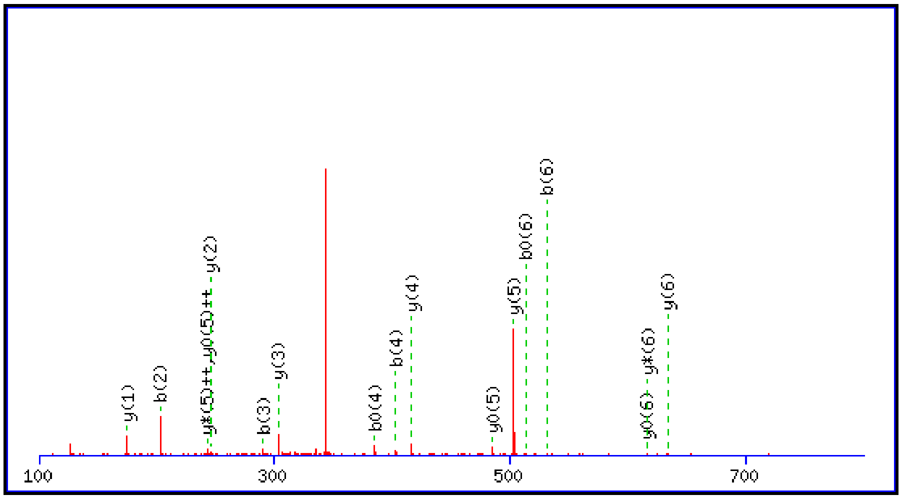

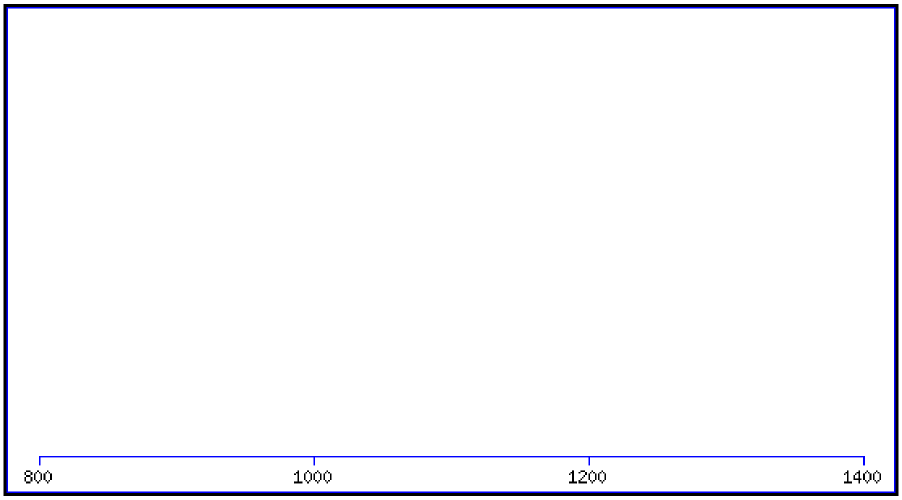

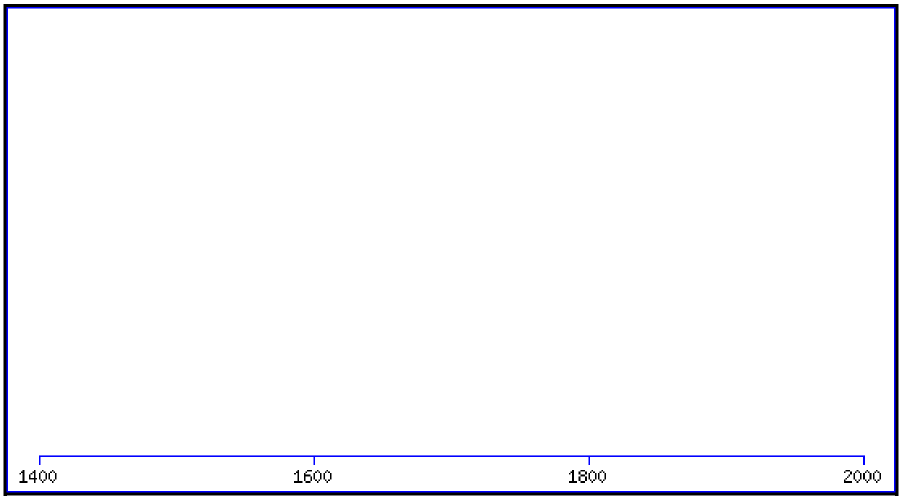

Monoisotopic mass of neutral peptide Mr(calc): 704.3639
Fixed modifications: Carbamidomethyl (C)
Ions Score: 46 **Expect:** 0.003
Matches (Bold Red): 17/48 fragment ions using 30 most intense peaks

#	b	b^{++}	b^{0}	b^{0++}	Seq.	y	y^{++}	y*	y^{*++}	y^{0}	y^{0++}	#
1	72.0444	36.5258			A							7
2	**203.0849**	102.0461			M	**634.3341**	317.6707	**617.3076**	309.1574	**616.3235**	308.6654	6
3	**290.1169**	145.5621	272.1063	136.5568	S	**503.2936**	252.1504	486.2671	**243.6372**	**485.2831**	**243.1452**	5
4	**403.2010**	202.1041	**385.1904**	193.0988	I	**416.2616**	208.6344	399.2350	200.1212			4
5	460.2224	230.6149	442.2119	221.6096	G	**303.1775**	152.0924	286.1510	143.5791			3
6	**531.2595**	266.1334	**513.2490**	257.1281	A	**246.1561**	123.5817	229.1295	115.0684			2

7					R	175.1190	88.0631	158.0924	79.5498			1

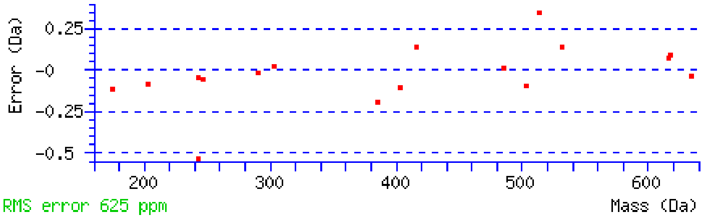

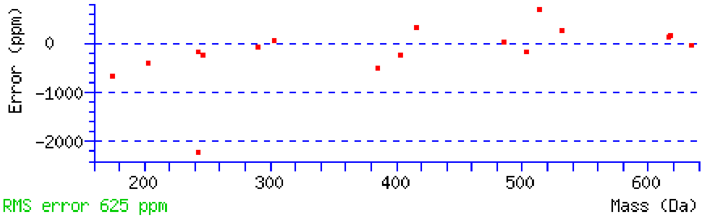

All matches to this query

Score	Mr(calc):	Delta	Sequence
45.9	704.3639	0.0012	AMSIGAR
35.9	704.3639	0.0012	AMEAKR
31.5	703.3613	1.0038	SNSIGAR
29.1	704.3639	0.0012	MASLQR
26.0	704.3639	0.0012	AMASVAR
20.7	704.3639	0.0012	MAAEKR
17.4	704.3639	0.0012	AMEARK
14.6	703.3613	1.0038	ATAASQR
14.3	704.3639	0.0012	ECIKR
14.1	704.3639	0.0012	AEMAKR

Spectrum No: 40; Query: 62930; Rank: 1

Peptide View

MS/MS Fragmentation of **AQPAQPADEPAEK**
Found in **IPI00016832**, Tax_Id=9606 Gene_Symbol=PSMA1 Isoform Short of Proteasome subunit alpha type-1

Match to Query 62930: 1350.643328 from(676.328940,2+)
Title: 090702LimSK_Exosome2_05.856.856.2.dta
Data file C:\Temp\Mascoti\LSK_2D\090702LimSK_Exosome2_01_2D.mgf

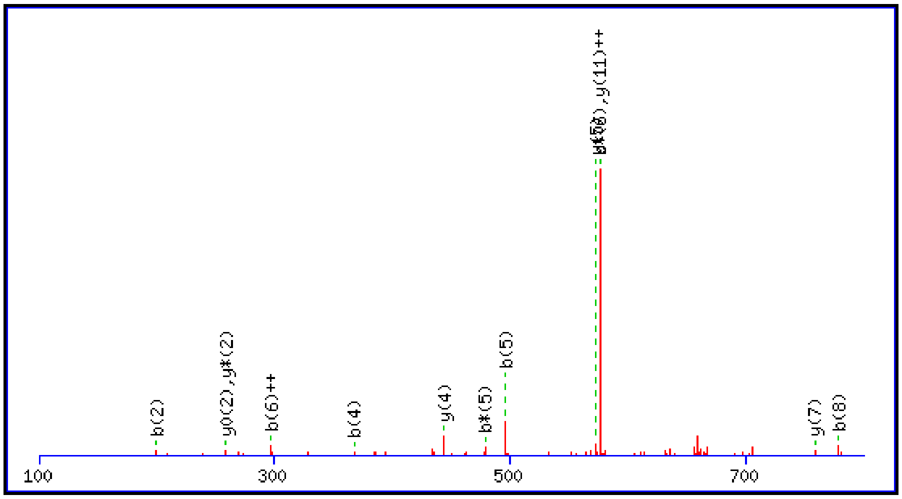

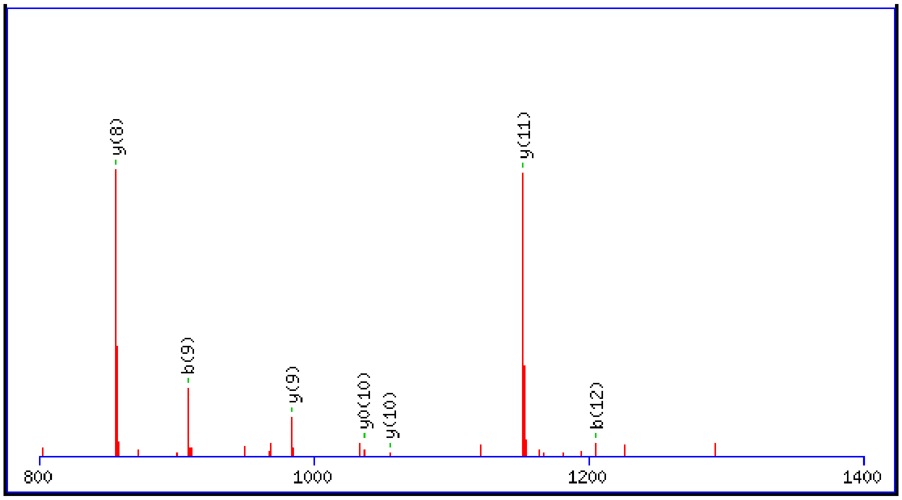

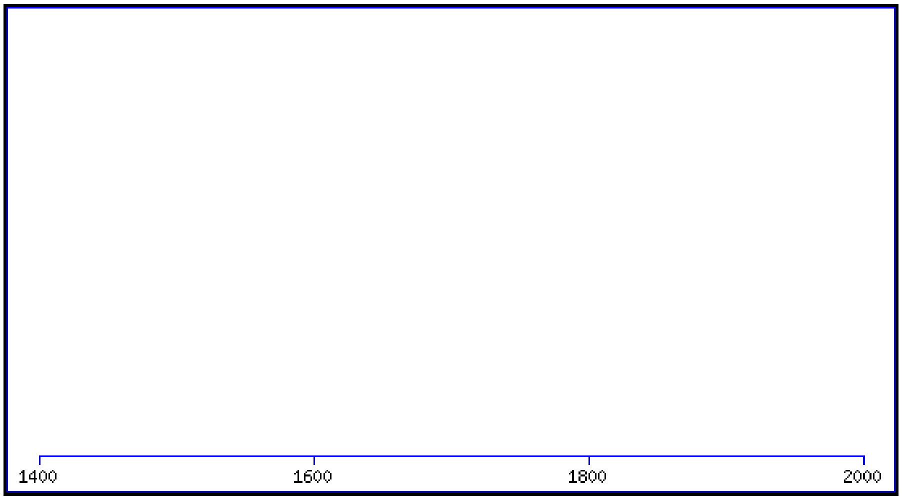

Monoisotopic mass of neutral peptide Mr(calc): 1350.6415
Fixed modifications: Carbamidomethyl (C)
Ions Score: 47 **Expect:** 0.0035
Matches (Bold Red): 21/126 fragment ions using 37 most intense peaks

#	b	b^{++}	b*	b^{*++}	b^{0}	b^{0++}	Seq.	y	y^{++}	y*	y^{*++}	y^{0}	y^{0++}	#
1	72.0444	36.5258					A							13
2	**200.1030**	100.5551	183.0764	92.0418			Q	1280.6117	640.8095	1263.5852	632.2962	1262.6012	631.8042	12
3	**297.1557**	149.0815	280.1292	140.5682			P	**1152.5531**	**576.7802**	1135.5266	568.2669	1134.5426	567.7749	11
4	**368.1928**	184.6001	351.1663	176.0868			A	**1055.5004**	528.2538	1038.4738	519.7406	**1037.4898**	519.2485	10
5	**496.2514**	248.6293	**479.2249**	240.1161			Q	**984.4633**	492.7353	967.4367	484.2220	966.4527	483.7300	9
6	593.3042	**297.1557**	**576.2776**	288.6425			P	**856.4047**	428.7060	839.3781	420.1927	838.3941	419.7007	8
7	664.3413	332.6743	647.3148	324.1610			A	**759.3519**	380.1796	742.3254	371.6663	741.3414	371.1743	7
8	**779.3682**	390.1878	762.3417	381.6745	761.3577	381.1825	D	688.3148	344.6610	671.2883	336.1478	670.3042	335.6558	6
9	**908.4108**	454.7091	891.3843	446.1958	890.4003	445.7038	E	**573.2879**	287.1476	556.2613	278.6343	555.2773	278.1423	5
10	1005.4636	503.2354	988.4371	494.7222	987.4530	494.2302	P	**444.2453**	222.6263	427.2187	214.1130	426.2347	213.6210	4
11	1076.5007	538.7540	1059.4742	530.2407	1058.4902	529.7487	A	347.1925	174.0999	330.1660	165.5866	329.1819	165.0946	3
12	**1205.5433**	603.2753	1188.5168	594.7620	1187.5327	594.2700	E	276.1554	138.5813	**259.1288**	130.0681	**258.1448**	129.5761	2
13							K	147.1128	74.0600	130.0863	65.5468			1

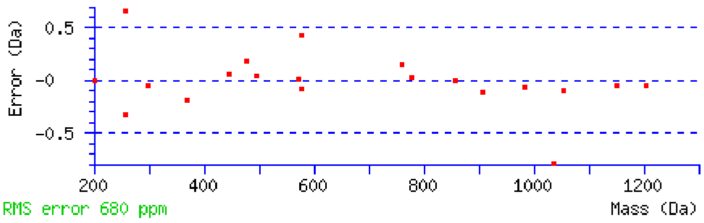

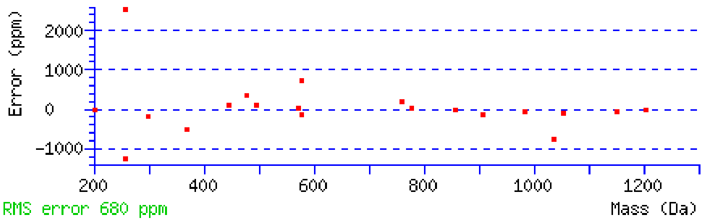

All matches to this query

Score	Mr(calc):	Delta	Sequence
47.0	1350.6415	0.0018	AQPAQPADEPAEK
7.0	1348.6388	2.0045	VPESSPIGSAIGR
6.3	1350.6489	-0.0056	TPQPAYLSEAMK
3.8	1350.6528	-0.0094	AQGAGAETPPEPAR
3.3	1350.6496	-0.0063	AQRMATDMQRK
3.3	1348.6388	2.0045	VPESSPIGSAIGR
2.6	1350.6544	-0.0111	AEKSPKSPNSVK
2.5	1350.6350	0.0083	EWKSQVQSGMR
2.4	1350.6544	-0.0111	TALLEANSTPVR
2.4	1350.6544	-0.0111	TALLEANSTPVR

Spectrum No: 41; Query: 87591; Rank: 1

Peptide View

MS/MS Fragmentation of **AQPAQPADEPAEKADEPMEH**
Found in **IPI00016832**, Tax_Id=9606 Gene_Symbol=PSMA1 Isoform Short of Proteasome subunit alpha type-1

Match to Query 87591: 2159.948382 from(720.990070,3+)
Title: 090702LimSK_Exosome2_03.3006.3006.3.dta
Data file C:\Temp\Mascoti\LSK_2D\090702LimSK_Exosome2_01_2D.mgf

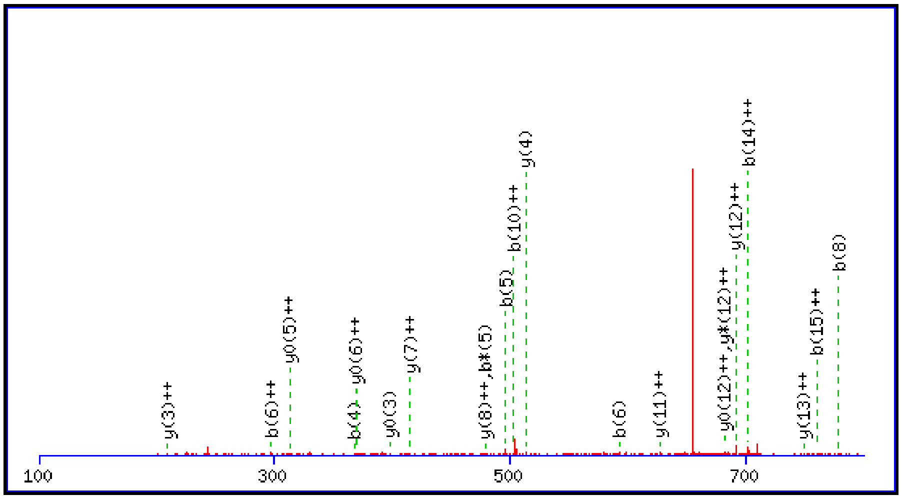

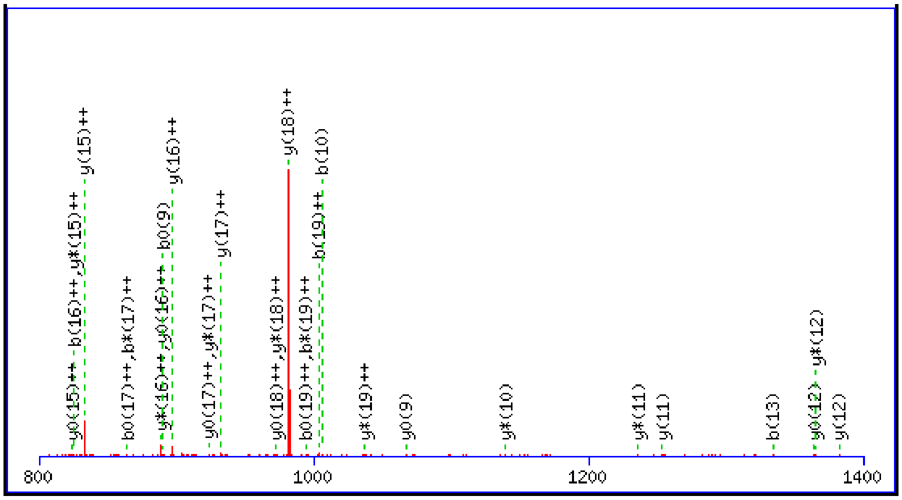

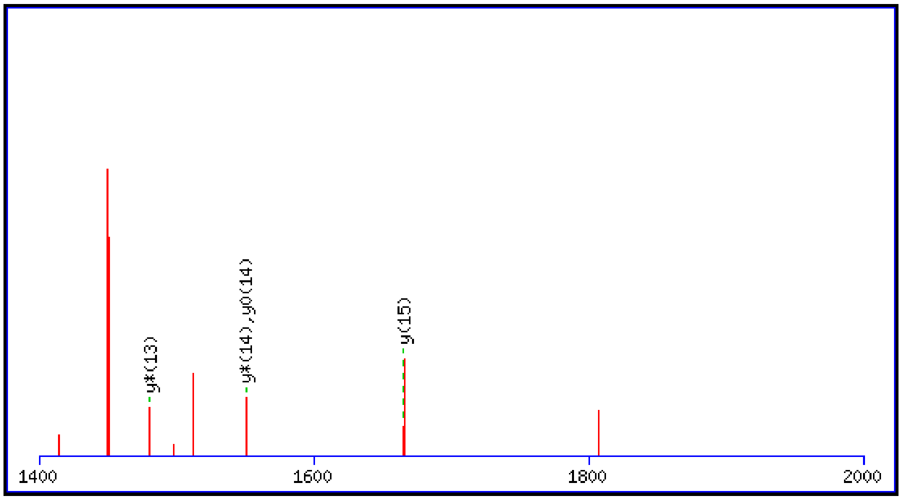

Monoisotopic mass of neutral peptide Mr(calc): 2159.9429
Fixed modifications: Carbamidomethyl (C)
Ions Score: 38 **Expect:** 0.037
Matches (Bold Red): 55/196 fragment ions using 113 most intense peaks

#	b	b^{++}	b*	b^{*++}	b^{0}	b^{0++}	Seq.	y	y^{++}	y*	y^{*++}	y^{0}	y^{0++}	#
1	72.0444	36.5258					A							20
2	200.1030	100.5551	183.0764	92.0418			Q	2089.9131	1045.4602	2072.8866	**1036.9469**	2071.9026	1036.4549	19
3	**297.1557**	149.0815	280.1292	140.5682			P	1961.8545	**981.4309**	1944.8280	**972.9176**	1943.8440	**972.4256**	18
4	**368.1928**	184.6001	351.1663	176.0868			A	1864.8018	**932.9045**	1847.7752	**924.3913**	1846.7912	**923.8992**	17
5	**496.2514**	248.6293	**479.2249**	240.1161			Q	1793.7647	**897.3860**	1776.7381	**888.8727**	1775.7541	**888.3807**	16
6	**593.3042**	**297.1557**	576.2776	288.6425			P	**1665.7061**	**833.3567**	1648.6795	**824.8434**	1647.6955	**824.3514**	15
7	664.3413	332.6743	647.3148	324.1610			A	1568.6533	784.8303	**1551.6268**	776.3170	**1550.6428**	775.8250	14
8	**779.3682**	390.1878	762.3417	381.6745	761.3577	381.1825	D	1497.6162	**749.3117**	**1480.5897**	740.7985	1479.6057	740.3065	13
9	908.4108	454.7091	891.3843	446.1958	**890.4003**	445.7038	E	**1382.5893**	**691.7983**	**1365.5627**	**683.2850**	**1364.5787**	**682.7930**	12
10	**1005.4636**	**503.2354**	988.4371	494.7222	987.4530	494.2302	P	**1253.5467**	**627.2770**	**1236.5201**	618.7637	1235.5361	618.2717	11
11	1076.5007	538.7540	1059.4742	530.2407	1058.4902	529.7487	A	1156.4939	578.7506	**1139.4674**	570.2373	1138.4834	569.7453	10
12	1205.5433	603.2753	1188.5168	594.7620	1187.5327	594.2700	E	1085.4568	543.2320	1068.4303	534.7188	**1067.4462**	534.2268	9
13	**1333.6383**	667.3228	1316.6117	658.8095	1315.6277	658.3175	K	956.4142	**478.7107**	939.3877	470.1975	938.4036	469.7055	8
14	1404.6754	**702.8413**	1387.6488	694.3281	1386.6648	693.8360	A	828.3192	**414.6633**			810.3087	405.6580	7
15	1519.7023	**760.3548**	1502.6758	751.8415	1501.6918	751.3495	D	757.2821	379.1447			739.2716	**370.1394**	6
16	1648.7449	**824.8761**	1631.7184	816.3628	1630.7344	815.8708	E	642.2552	321.6312			624.2446	**312.6259**	5
17	1745.7977	873.4025	1728.7711	**864.8892**	1727.7871	**864.3972**	P	**513.2126**	257.1099			495.2020	248.1047	4
18	1876.8382	938.9227	1859.8116	930.4094	1858.8276	929.9174	M	416.1598	**208.5836**			**398.1493**	199.5783	3
19	2005.8808	**1003.4440**	1988.8542	**994.9307**	1987.8702	**994.4387**	E	285.1193	143.0633			267.1088	134.0580	2

**20**							**H**	156.0768	78.5420					**1**

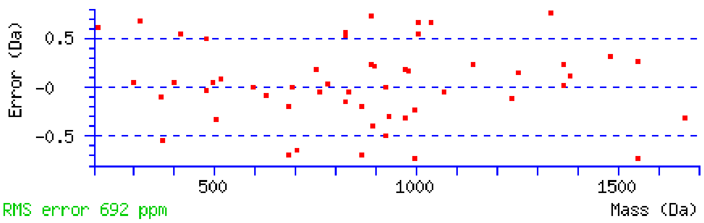

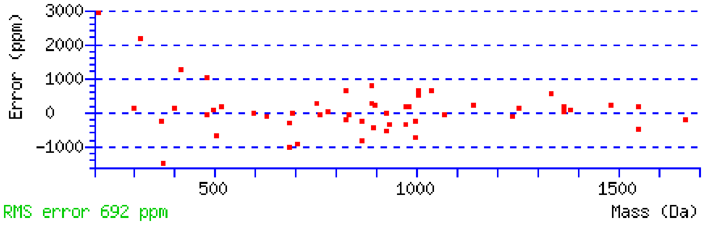

All matches to this query

Score	Mr(calc):	Delta	Sequence
38.4	2159.9429	0.0055	AQPAQPADEPAEKADEPMEH
12.1	2158.9622	0.9861	LSDPGVSPAVLSLEMLTDR
9.8	2157.9432	2.0052	LLASWVSGSSGRSGGFMRK
9.8	2159.9574	-0.0090	YSFFVAMGYLTICHISR
8.2	2158.9530	0.9954	YFLSEASVVWYGRPPTK
8.0	2157.9432	2.0052	LLASWVSGSSGRSGGFMRK
7.8	2159.9359	0.0125	STSRGSHAASDAAGGGGWHRR
7.8	2159.9359	0.0125	STSRGSHAASDAAGGGGWHRR
7.2	2158.9530	0.9954	YFLSEASVVWYGRPPTK
7.0	2159.9405	0.0079	SLVSDKTSISEKVLASEK

Spectrum No: 42; Query: 46864; Rank: 1

Peptide View

MS/MS Fragmentation of **AQSELAAHQK**
Found in **IPI00016832**, Tax_Id=9606 Gene_Symbol=PSMA1 Isoform Short of Proteasome subunit alpha type-1

Match to Query 46864: 1081.553952 from(361.525260,3+)
Title: 090702LimSK_Exosome2_05.1679.1679.3.dta
Data file C:\Temp\Mascoti\LSK_2D\090702LimSK_Exosome2_01_2D.mgf

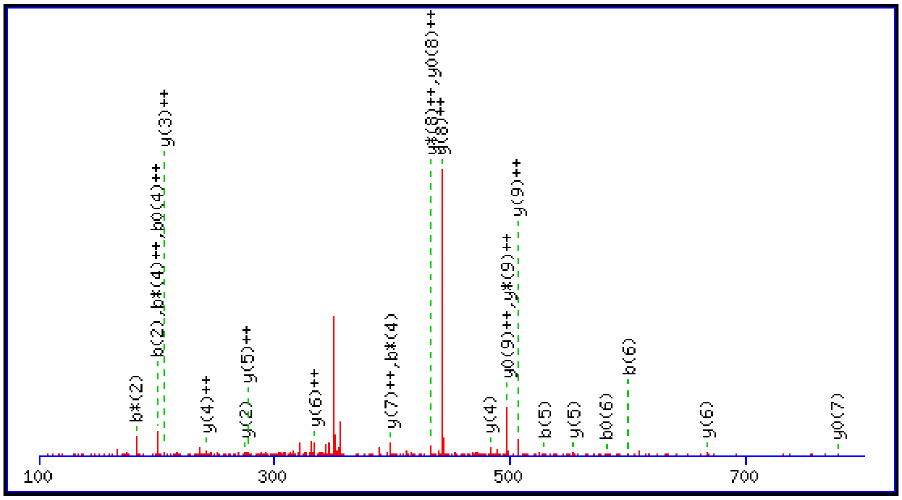

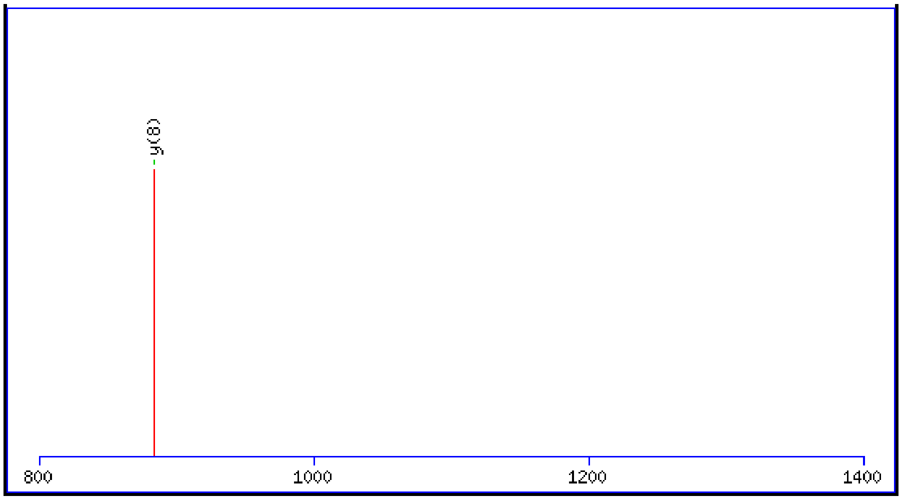

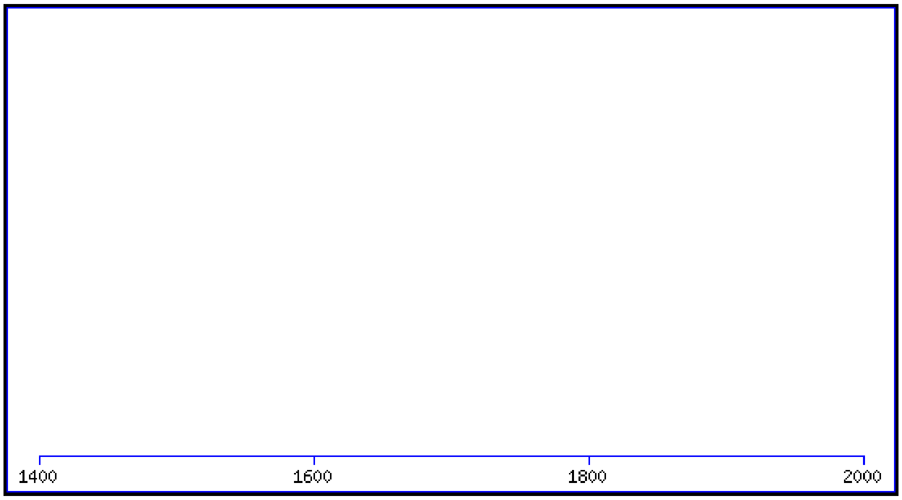

Monoisotopic mass of neutral peptide Mr(calc): 1081.5516
Fixed modifications: Carbamidomethyl (C)
Ions Score: 39 **Expect:** 0.021
Matches (Bold Red): 25/90 fragment ions using 45 most intense peaks

#	b	b^{++}	b*	b^{*++}	b^0	b^{0++}	Seq.	y	y^{++}	y*	y^{*++}	y^0	y^{0++}	#
1	72.0444	36.5258					A							10
2	**200.1030**	100.5551	**183.0764**	92.0418			Q	1011.5218	**506.2645**	994.4952	**497.7513**	993.5112	**497.2592**	9
3	287.1350	144.0711	270.1084	135.5579	269.1244	135.0659	S	**883.4632**	**442.2352**	866.4367	**433.7220**	865.4526	**433.2300**	8
4	416.1776	208.5924	**399.1510**	**200.0792**	398.1670	**199.5872**	E	796.4312	**398.7192**	779.4046	390.2060	**778.4206**	389.7139	7
5	**529.2617**	265.1345	512.2351	256.6212	511.2511	256.1292	L	**667.3886**	**334.1979**	650.3620	325.6847			6
6	**600.2988**	300.6530	583.2722	292.1397	**582.2882**	291.6477	A	**554.3045**	**277.6559**	537.2780	269.1426			5
7	671.3359	336.1716	654.3093	327.6583	653.3253	327.1663	A	**483.2674**	**242.1373**	466.2409	233.6241			4
8	808.3948	404.7010	791.3682	396.1878	790.3842	395.6958	H	412.2303	**206.6188**	395.2037	198.1055			3
9	936.4534	468.7303	919.4268	460.2170	918.4428	459.7250	Q	**275.1714**	138.0893	258.1448	129.5761			2
10							K	147.1128	74.0600	130.0863	65.5468			1

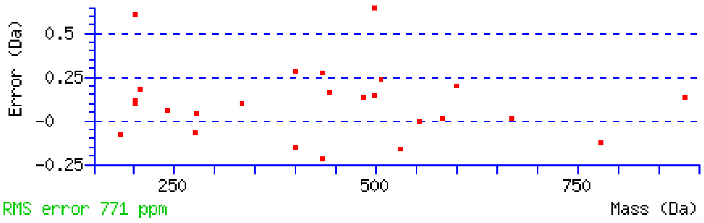

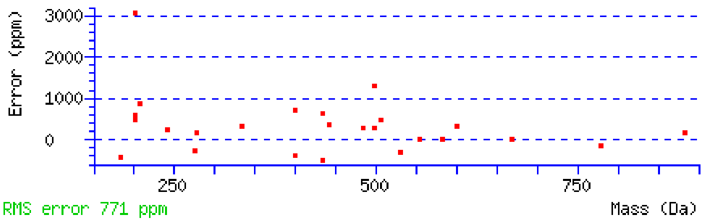

All matches to this query

Score	Mr(calc):	Delta	Sequence
38.5	1081.5516	0.0024	AQSELAAHQK
13.3	1080.5580	0.9959	TALITEVVR
11.5	1081.5532	0.0007	LSKISQAQK
10.4	1081.5628	-0.0089	LSSGANQHLR
9.3	1081.5628	-0.0089	AQQDKHSLR
8.3	1080.5580	0.9960	VATSPKSALK
6.7	1079.5499	2.0041	LDDKDLFSK
6.6	1081.5645	-0.0105	KINSSRVAK
6.4	1080.5580	0.9959	VPAKTSTLGK
6.3	1079.5529	2.0011	VIWLQSVR

Spectrum No: 43; Query: 62702; Rank: 1

Peptide View

MS/MS Fragmentation of **ETLPAEQDLTTK**
Found in **IPI00016832**, Tax_Id=9606 Gene_Symbol=PSMA1 Isoform Short of Proteasome subunit alpha type-1

Match to Query 62702: 1344.680808 from(673.347680,2+)
Title: 090702LimSK_Exosome2_06.3587.3587.2.dta
Data file C:\Temp\Mascoti\LSK_2D\090702LimSK_Exosome2_01_2D.mgf

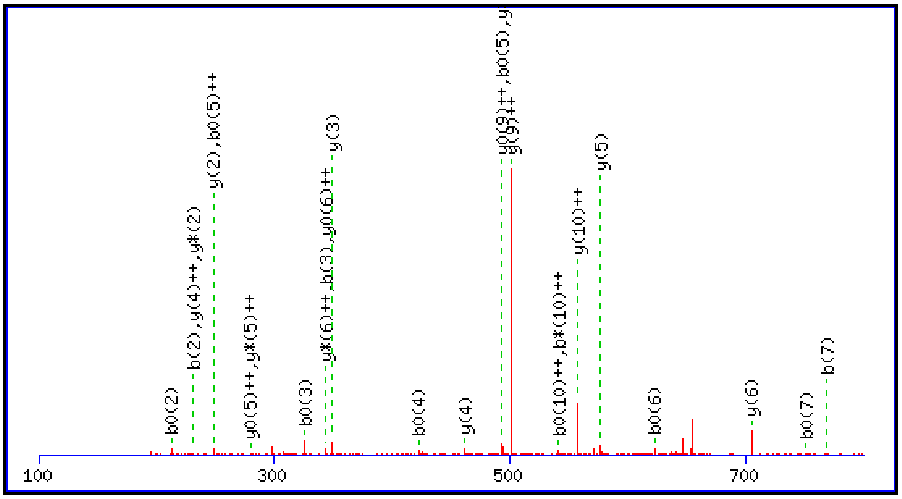

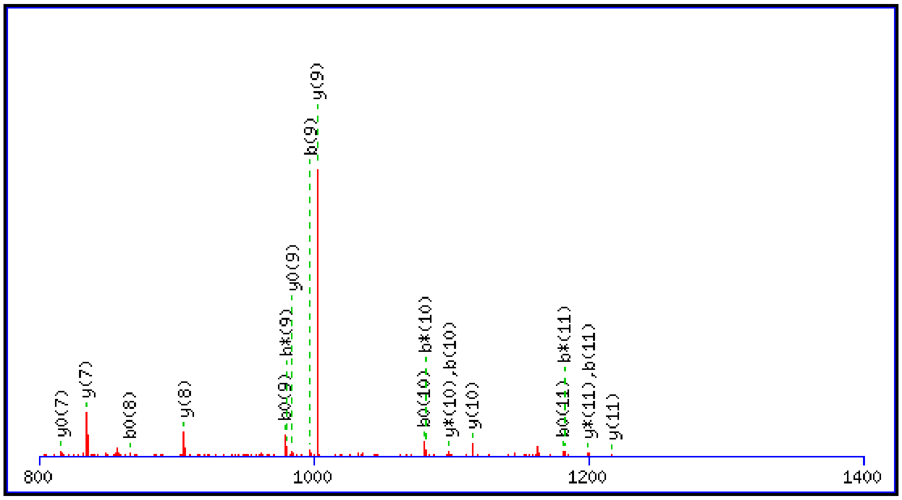

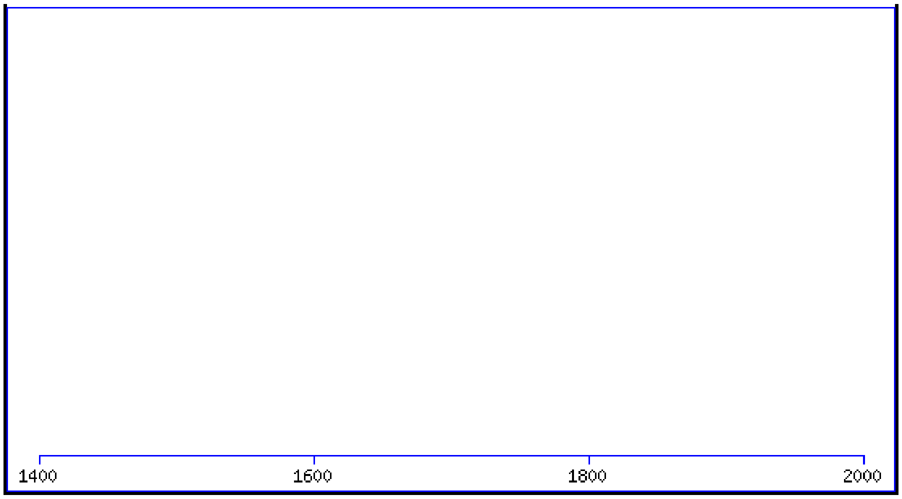

Monoisotopic mass of neutral peptide Mr(calc): 1344.6773
Fixed modifications: Carbamidomethyl (C)
Ions Score: 78 **Expect:** 3e-006
Matches (Bold Red): 46/118 fragment ions using 54 most intense peaks

#	b	b^{++}	b*	b^{*++}	b^0	b^{0++}	Seq.	y	y^{++}	y*	y^{*++}	y^0	y^{0++}	#
1	130.0499	65.5286			112.0393	56.5233	E							12
2	**231.0975**	116.0524			**213.0870**	107.0471	T	**1216.6420**	608.8246	**1199.6154**	600.3113	1198.6314	599.8193	11
3	**344.1816**	172.5944			**326.1710**	163.5892	L	**1115.5943**	**558.3008**	**1098.5677**	549.7875	1097.5837	549.2955	10
4	441.2344	221.1208			**423.2238**	212.1155	P	**1002.5102**	**501.7587**	985.4837	**493.2455**	**984.4997**	**492.7535**	9
5	512.2715	256.6394			**494.2609**	**247.6341**	A	**905.4575**	453.2324	888.4309	444.7191	887.4469	444.2271	8
6	641.3141	321.1607			**623.3035**	312.1554	E	**834.4203**	417.7138	817.3938	409.2005	**816.4098**	408.7085	7
7	**769.3727**	385.1900	752.3461	376.6767	**751.3621**	376.1847	Q	**705.3777**	353.1925	688.3512	**344.6792**	687.3672	**344.1872**	6
8	884.3996	442.7034	867.3731	434.1902	**866.3890**	433.6982	D	**577.3192**	289.1632	560.2926	**280.6499**	559.3086	**280.1579**	5
9	**997.4837**	499.2455	**980.4571**	490.7322	**979.4731**	490.2402	L	**462.2922**	**231.6498**	445.2657	223.1365	444.2817	222.6445	4
10	**1098.5313**	549.7693	**1081.5048**	**541.2560**	**1080.5208**	**540.7640**	T	**349.2082**	175.1077	332.1816	166.5944	331.1976	166.1024	3
11	**1199.5790**	600.2932	**1182.5525**	591.7799	**1181.5685**	591.2879	T	**248.1605**	124.5839	**231.1339**	116.0706	230.1499	115.5786	2
12							K	147.1128	74.0600	130.0863	65.5468			1

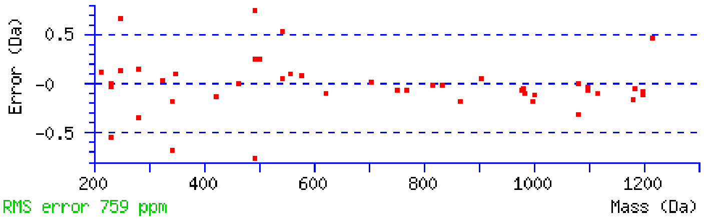

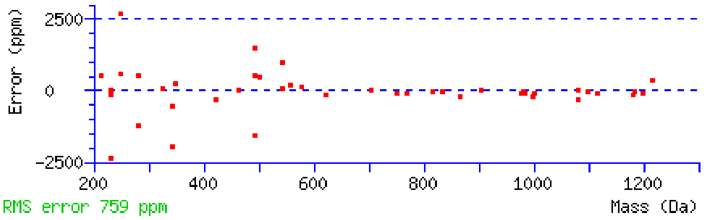

All matches to this query

Score	Mr(calc):	Delta	Sequence
77.6	1344.6773	0.0035	ETLPAEQDLTTK
17.0	1344.6885	-0.0077	VLLANSTQADEGK
11.9	1342.6646	2.0162	DLLAPPSPALGGR
11.3	1344.6826	-0.0018	DVERFFKGYGK
11.0	1344.6915	-0.0107	DPLSAPALRLGR
10.4	1343.6850	0.9958	TKILNNSSIFK
8.6	1343.6655	1.0153	TLKWLISPVK
8.5	1343.6711	1.0097	LLGPGGRPSSPAR
7.6	1342.6662	2.0146	KVPVSVNLLSK
6.8	1344.6737	0.0071	MNVSPVPPLRR

Spectrum No: 44; Query: 62270; Rank: 1

Peptide View

MS/MS Fragmentation of **FVFDRPLPVSR**
Found in **IPI00016832**, Tax_Id=9606 Gene_Symbol=PSMA1 Isoform Short of Proteasome subunit alpha type-1

Match to Query 62270: 1331.739462 from(444.920430,3+)
Title: 090702LimSK_Exosome2_01.5025.5025.3.dta
Data file C:\Temp\Mascoti\LSK_2D\090702LimSK_Exosome2_01_2D.mgf

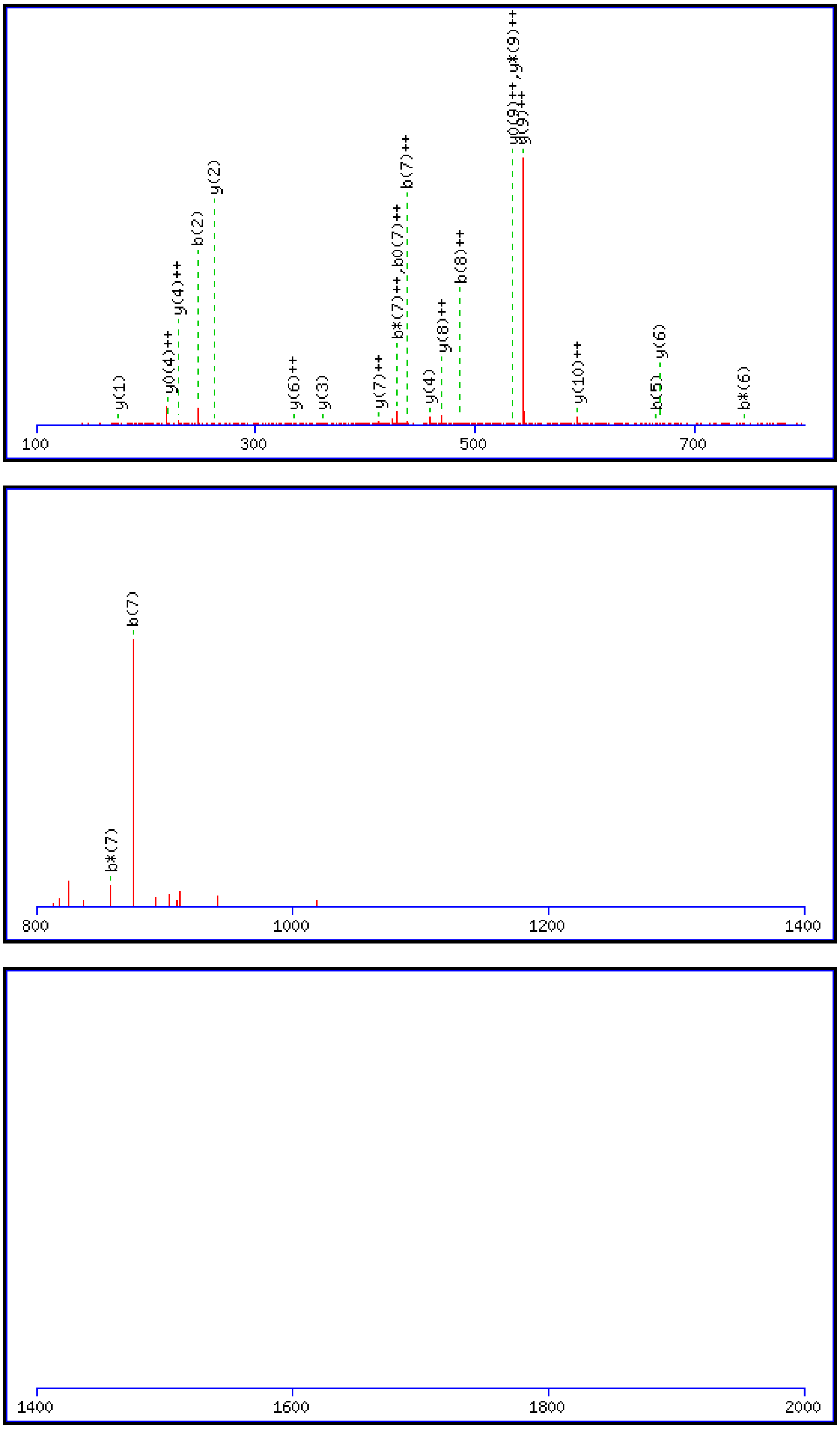

Monoisotopic mass of neutral peptide Mr(calc): 1331.7350
Fixed modifications: Carbamidomethyl (C)
Ions Score: 32 **Expect:** 0.063
Matches (Bold Red): 23/104 fragment ions using 52 most intense peaks

#	b	b^{++}	b*	b^{*++}	b^{0}	b^{0++}	Seq.	y	y^{++}	y*	y^{*++}	y^{0}	y^{0++}	#
1	148.0757	74.5415					F							11
2	**247.1441**	124.0757					V	1185.6739	**593.3406**	1168.6473	584.8273	1167.6633	584.3353	10
3	394.2125	197.6099					F	1086.6055	**543.8064**	1069.5789	**535.2931**	1068.5949	**534.8011**	9
4	509.2395	255.1234			491.2289	246.1181	D	939.5370	**470.2722**	922.5105	461.7589	921.5265	461.2669	8
5	**665.3406**	333.1739	648.3140	324.6606	647.3300	324.1686	R	824.5101	**412.7587**	807.4835	404.2454	806.4995	403.7534	7
6	762.3933	381.7003	**745.3668**	373.1870	744.3828	372.6950	P	**668.4090**	**334.7081**	651.3824	326.1949	650.3984	325.7028	6
7	**875.4774**	**438.2423**	**858.4509**	**429.7291**	857.4668	**429.2371**	L	571.3562	286.1817	554.3297	277.6685	553.3457	277.1765	5
8	972.5302	**486.7687**	955.5036	478.2554	954.5196	477.7634	P	**458.2722**	**229.6397**	441.2456	221.1264	440.2616	**220.6344**	4
9	1071.5986	536.3029	1054.5720	527.7897	1053.5880	527.2976	V	**361.2194**	181.1133	344.1928	172.6001	343.2088	172.1081	3
10	1158.6306	579.8189	1141.6041	571.3057	1140.6200	570.8137	S	**262.1510**	131.5791	245.1244	123.0659	244.1404	122.5738	2
11							R	**175.1190**	88.0631	158.0924	79.5498			1

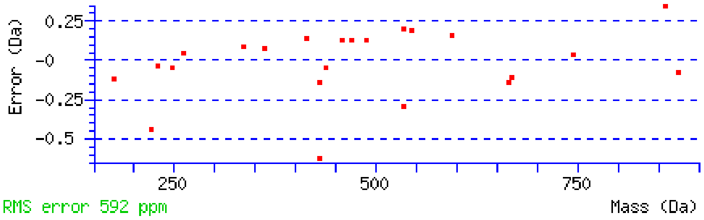

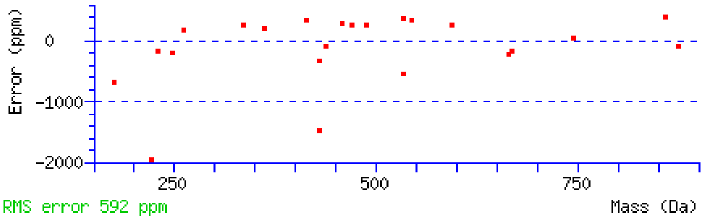

All matches to this query

Score	Mr(calc):	Delta	Sequence
32.0	1331.7350	0.0044	FVFDRPLPVSR
12.8	1331.7271	0.0123	LVPLASDMIFAR
11.6	1330.7235	1.0160	SGRHVTRVLVK
9.5	1329.7405	1.9990	VKLDFVAPATGGR
6.6	1330.7357	1.0037	DPRAGPAVPGGLPK
4.9	1329.7210	2.0185	YLGLKALGFLR
4.8	1329.7405	1.9990	RSFLGQKDPALV
4.7	1330.7245	1.0150	XPTTSRPALDVK
4.5	1330.7391	1.0004	GSSLNIMRNLVK
4.5	1329.7438	1.9956	MALIKDVLQQR

Spectrum No: 45; Query: 66075; Rank: 1

Peptide View

MS/MS Fragmentation of **IHQIEYAMEAVK**
Found in **IPI00016832**, Tax_Id=9606 Gene_Symbol=PSMA1 Isoform Short of Proteasome subunit alpha type-1

Match to Query 66075: 1431.725948 from(716.870250,2+)
Title: 090702LimSK_Exosome2_05.8826.8826.2.dta
Data file C:\Temp\Mascoti\LSK_2D\090702LimSK_Exosome2_01_2D.mgf

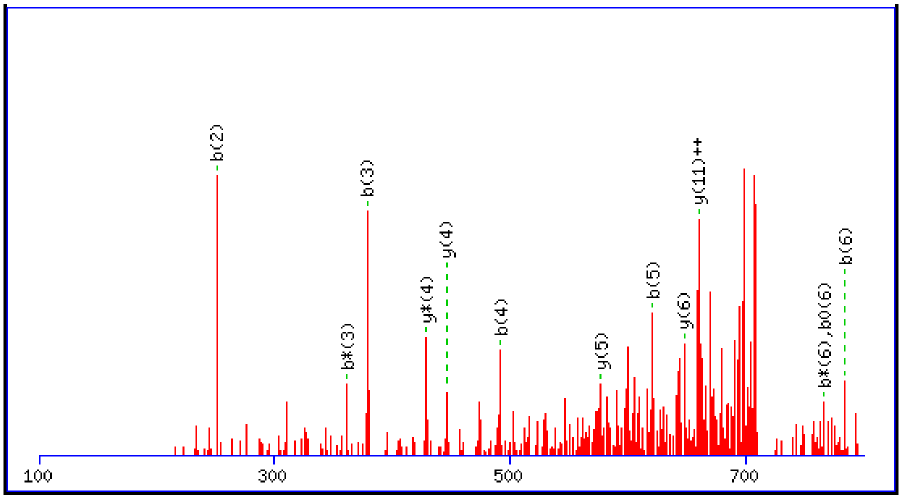

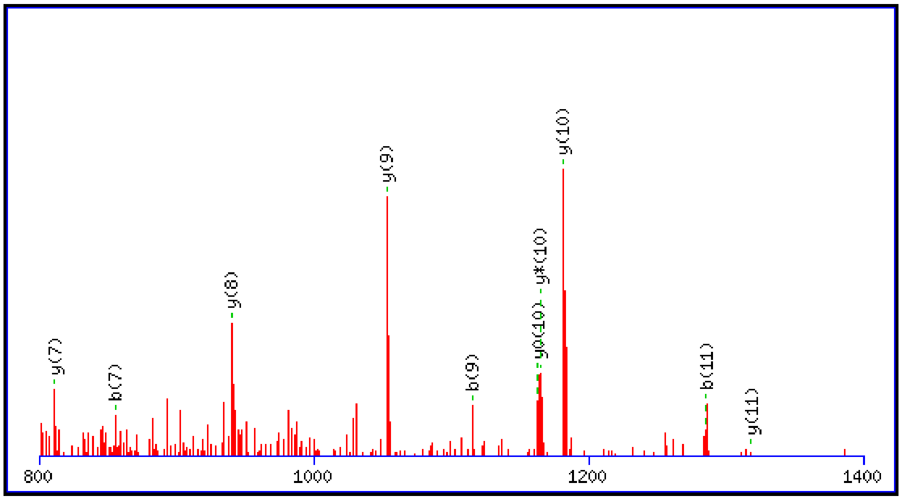

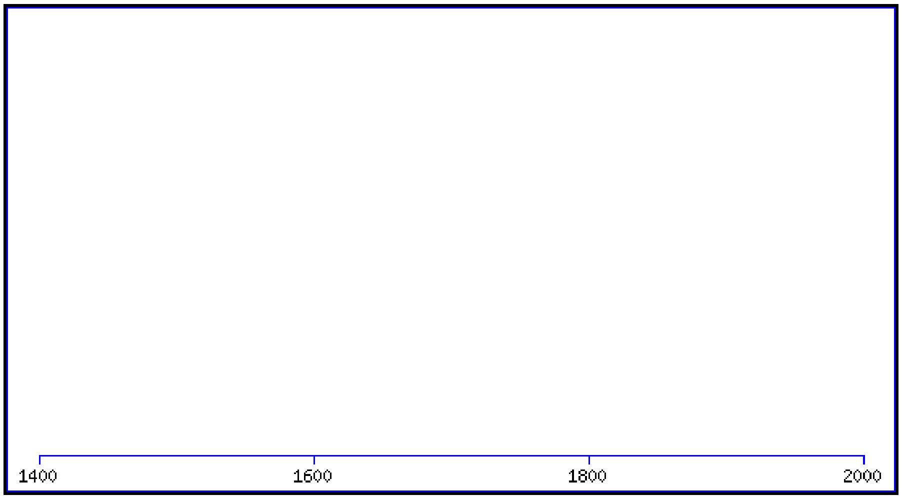

Monoisotopic mass of neutral peptide Mr(calc): 1430.7227
Fixed modifications: Carbamidomethyl (C)
Ions Score: 41 **Expect:** 0.013
Matches (Bold Red): 23/114 fragment ions using 65 most intense peaks

#	b	b^{++}	b*	b^{*++}	b^0	b^{0++}	Seq.	y	y^{++}	y*	y^{*++}	y^0	y^{0++}	#
1	114.0913	57.5493					I							12
2	**251.1503**	126.0788					H	**1318.6460**	**659.8266**	1301.6195	651.3134	1300.6354	650.8214	11
3	**379.2088**	190.1081	**362.1823**	181.5948			Q	**1181.5871**	591.2972	**1164.5605**	582.7839	**1163.5765**	582.2919	10
4	**492.2929**	246.6501	475.2663	238.1368			I	**1053.5285**	527.2679	1036.5020	518.7546	1035.5179	518.2626	9
5	**621.3355**	311.1714	604.3089	302.6581	603.3249	302.1661	E	**940.4444**	470.7259	923.4179	462.2126	922.4339	461.7206	8
6	**784.3988**	392.7030	**767.3723**	384.1898	**766.3883**	383.6978	Y	**811.4019**	406.2046	794.3753	397.6913	793.3913	397.1993	7

**7**	**855.4359**	428.2216	838.4094	419.7083	837.4254	419.2163	**A**	**648.3385**	324.6729	631.3120	316.1596	630.3280	315.6676	**6**
**8**	986.4764	493.7418	969.4499	485.2286	968.4659	484.7366	**M**	**577.3014**	289.1543	560.2749	280.6411	559.2908	280.1491	**5**
**9**	**1115.5190**	558.2631	1098.4925	549.7499	1097.5084	549.2579	**E**	**446.2609**	223.6341	**429.2344**	215.1208	428.2504	214.6288	**4**
**10**	1186.5561	593.7817	1169.5296	585.2684	1168.5456	584.7764	**A**	317.2183	159.1128	300.1918	150.5995			**3**
**11**	**1285.6245**	643.3159	1268.5980	634.8026	1267.6140	634.3106	**V**	246.1812	123.5942	229.1547	115.0810			**2**
**12**							**K**	147.1128	74.0600	130.0863	65.5468			**1**

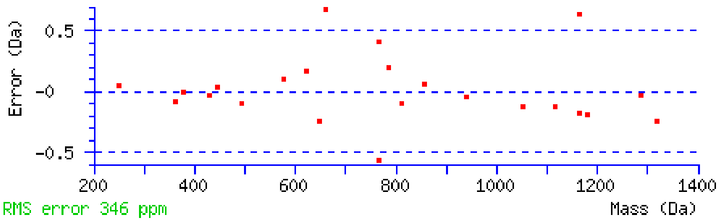

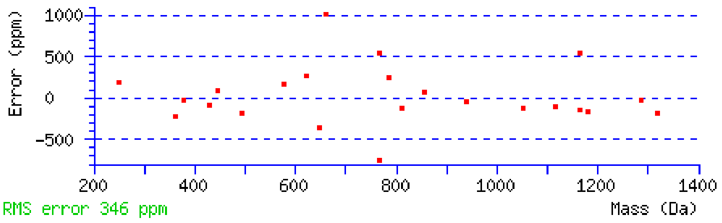

All matches to this query

Score	Mr(calc):	Delta	Sequence
41.3	1430.7227	1.0032	IHQIEYAMEAVK
7.1	1431.7220	0.0039	MQLFIYEIGFR
6.3	1431.7220	0.0039	YSKDKMWYLAK
6.1	1431.7180	0.0079	NHVFTVTMENLK
5.2	1430.7089	1.0171	IHQNITYQVCR
4.7	1431.7258	0.0001	SYEAFYARARAK
4.7	1429.7248	2.0011	KVSNMFNLHAGGR
4.1	1431.7136	0.0124	IHKAASRGQAWK
3.8	1430.7340	0.9920	IHTGEKPYKCAK
3.3	1429.7174	2.0085	NFLKSHGRDNSR

Spectrum No: 46; Query: 89851; Rank: 1

Peptide View

MS/MS Fragmentation of **KAQPAQPADEPAEKADEPMEH**
Found in **IPI00016832**, Tax_Id=9606 Gene_Symbol=PSMA1 Isoform Short of Proteasome subunit alpha type-1

Match to Query 89851: 2288.043672 from(763.688500,3+)
Title: 090702LimSK_Exosome2_01.1142.1142.3.dta
Data file C:\Temp\Mascoti\LSK_2D\090702LimSK_Exosome2_01_2D.mgf

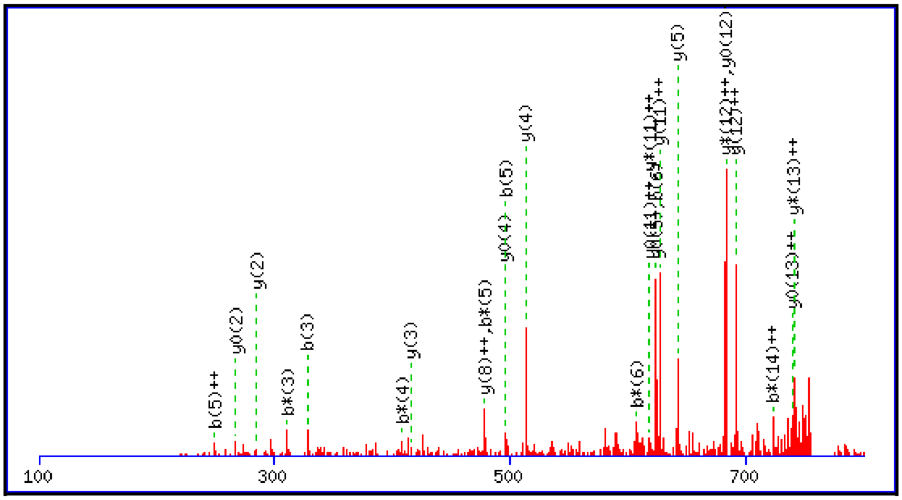

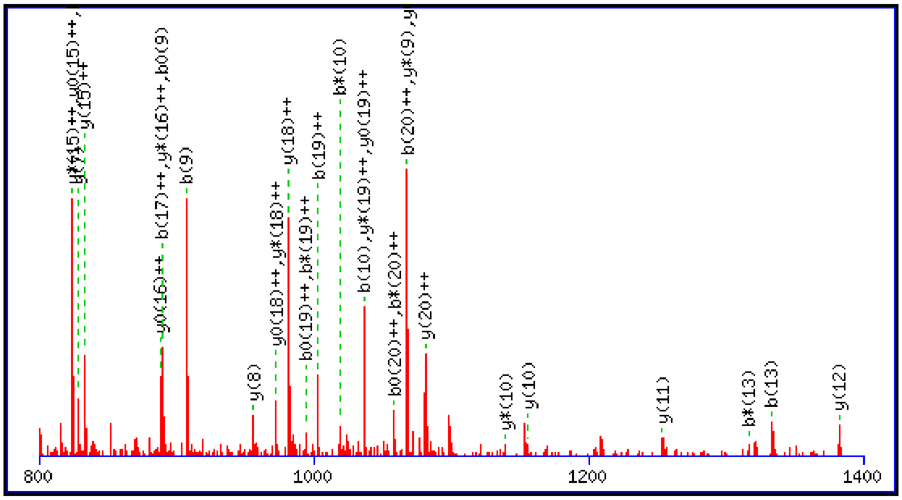

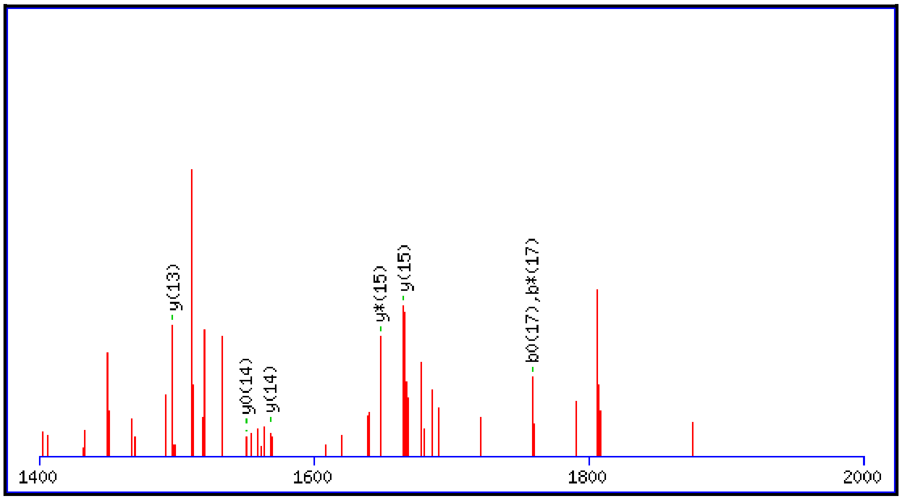

Monoisotopic mass of neutral peptide Mr(calc): 2288.0379
Fixed modifications: Carbamidomethyl (C)
Ions Score: 45 **Expect:** 0.0095
Matches (Bold Red): 65/208 fragment ions using 134 most intense peaks

#	b	b++	b*	b*++	b0	b0++	Seq.	y	y++	y*	y*++	y0	y0++	#
1	129.1022	65.0548	112.0757	56.5415			K							21
2	200.1394	100.5733	183.1128	92.0600			A	2160.9502	**1080.9788**	2143.9237	1072.4655	2142.9397	1071.9735	20
3	**328.1979**	164.6026	**311.1714**	156.0893			Q	2089.9131	1045.4602	2072.8866	**1036.9469**	2071.9026	**1036.4549**	19
4	425.2507	213.1290	**408.2241**	204.6157			P	1961.8545	**981.4309**	1944.8280	**972.9176**	1943.8440	**972.4256**	18
5	**496.2878**	**248.6475**	**479.2613**	240.1343			A	1864.8018	932.9045	1847.7752	924.3913	1846.7912	923.8992	17
6	**624.3464**	312.6768	**607.3198**	304.1636			Q	1793.7647	897.3860	1776.7381	**888.8727**	1775.7541	**888.3807**	16
7	721.3992	361.2032	704.3726	352.6899			P	**1665.7061**	**833.3567**	**1648.6795**	**824.8434**	1647.6955	**824.3514**	15
8	792.4363	396.7218	775.4097	388.2085			A	**1568.6533**	784.8303	1551.6268	776.3170	**1550.6428**	775.8250	14
9	**907.4632**	454.2352	890.4367	445.7220	**889.4526**	445.2300	D	**1497.6162**	749.3117	1480.5897	**740.7985**	1479.6057	**740.3065**	13
10	**1036.5058**	518.7565	**1019.4793**	510.2433	1018.4952	509.7513	E	**1382.5893**	**691.7983**	1365.5627	**683.2850**	1364.5787	**682.7930**	12
11	1133.5586	567.2829	1116.5320	558.7696	1115.5480	558.2776	P	**1253.5467**	**627.2770**	1236.5201	**618.7637**	1235.5361	**618.2717**	11
12	1204.5957	602.8015	1187.5691	594.2882	1186.5851	593.7962	A	**1156.4939**	578.7506	**1139.4674**	570.2373	1138.4834	569.7453	10
13	**1333.6383**	667.3228	**1316.6117**	658.8095	1315.6277	658.3175	E	1085.4568	543.2320	**1068.4303**	534.7188	**1067.4462**	534.2268	9
14	1461.7332	731.3703	1444.7067	**722.8570**	1443.7227	722.3650	K	**956.4142**	**478.7107**	939.3877	470.1975	938.4036	469.7055	8
15	1532.7703	766.8888	1515.7438	758.3755	1514.7598	757.8835	A	**828.3192**	414.6633			810.3087	405.6580	7
16	1647.7973	**824.4023**	1630.7707	815.8890	1629.7867	815.3970	D	757.2821	379.1447			739.2716	370.1394	6
17	1776.8399	**888.9236**	**1759.8133**	880.4103	**1758.8293**	879.9183	E	**642.2552**	321.6312			**624.2446**	312.6259	5
18	1873.8926	937.4500	1856.8661	928.9367	1855.8821	928.4447	P	**513.2126**	257.1099			**495.2020**	248.1047	4
19	2004.9331	**1002.9702**	1987.9066	**994.4569**	1986.9226	**993.9649**	M	**416.1598**	208.5836			398.1493	199.5783	3

**20**	2133.9757	**1067.4915**	2116.9492	**1058.9782**	2115.9652	**1058.4862**	**E**	**285.1193**	143.0633			**267.1088**	134.0580	**2**
**21**							**H**	156.0768	78.5420					**1**

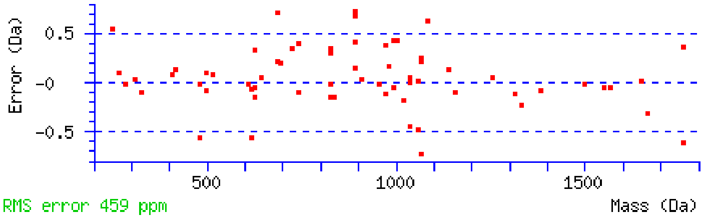

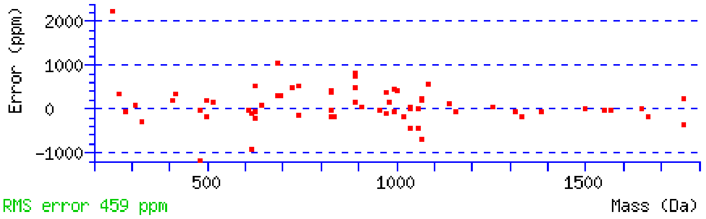

All matches to this query

Score	Mr(calc):	Delta	Sequence
44.9	2288.0379	0.0058	KAQPAQPADEPAEKADEPMEH
4.7	2287.0490	0.9946	GVSALNHMEVSFLGSRCTVK
4.5	2288.0603	-0.0166	ALPTPLYSTLPVESQGRGSR
3.5	2286.0384	2.0053	DVQSLSLRIMLAEILTTK
2.9	2288.0273	0.0164	TEITSSKRISSSFLAQSMR
2.8	2286.0391	2.0045	YYRTLEDIEKNYMSPLR
2.7	2287.0287	1.0150	DQVTTPWVPSSALIVSSNAR
2.5	2287.0230	1.0207	GVADQTRDGPEPEAEREPVR
2.4	2287.0594	0.9843	DLVQRETGKVQTAEEDHPR
2.0	2288.0273	0.0164	TEITSSKRISSSFLAQSMR

Spectrum No: 47; Query: 40866; Rank: 1

Peptide View

MS/MS Fragmentation of **LLCNFMR**
Found in **IPI00016832**, Tax_Id=9606 Gene_Symbol=PSMA1 Isoform Short of Proteasome subunit alpha type-1

Match to Query 40866: 952.463888 from(477.239220,2+)
Title: 090702LimSK_Exosome2_05.5807.5807.2.dta
Data file C:\Temp\Mascoti\LSK_2D\090702LimSK_Exosome2_01_2D.mgf

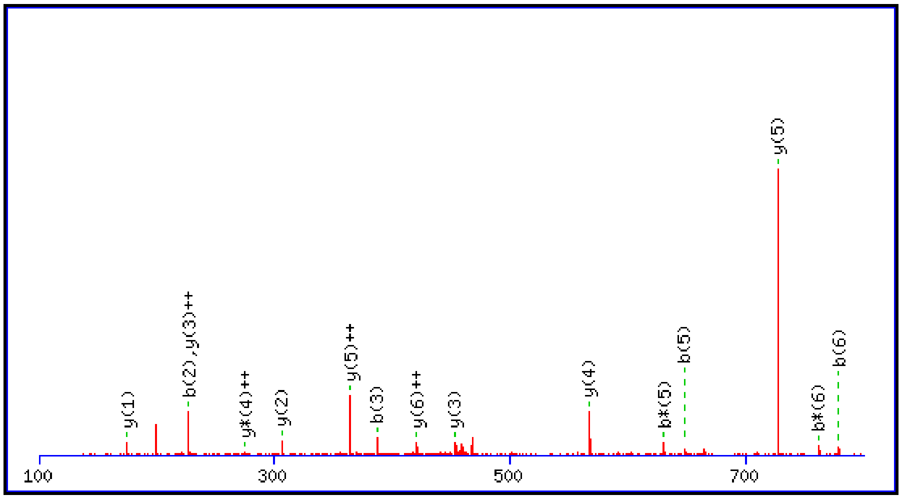

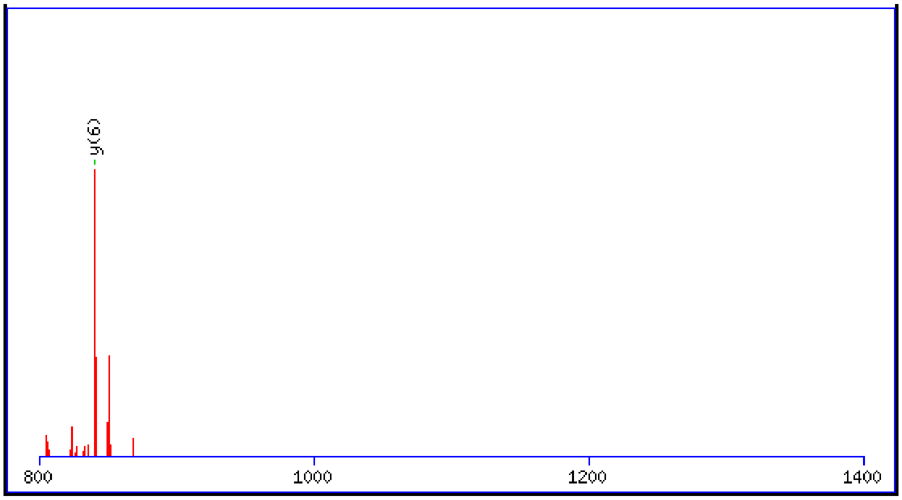

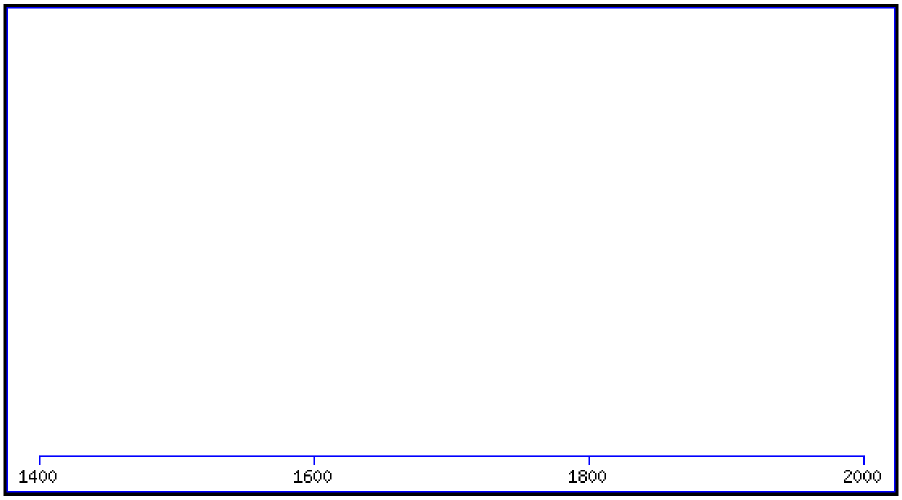

Monoisotopic mass of neutral peptide Mr(calc): 952.4623
Fixed modifications: Carbamidomethyl (C)
Ions Score: 46 **Expect:** 0.0029
Matches (Bold Red): 16/42 fragment ions using 25 most intense peaks

#	b	b++	b*	b*++	Seq.	y	y++	y*	y*++	#
1	114.0913	57.5493			L					7
2	**227.1754**	114.0913			L	**840.3855**	**420.6964**	823.3589	412.1831	6
3	**387.2061**	194.1067			C	**727.3014**	**364.1544**	710.2749	355.6411	5
4	501.2490	251.1281	484.2224	242.6149	N	**567.2708**	284.1390	550.2442	**275.6258**	4
5	**648.3174**	324.6623	**631.2908**	316.1491	F	**453.2279**	**227.1176**	436.2013	218.6043	3
6	**779.3579**	390.1826	**762.3313**	381.6693	M	**306.1594**	153.5834	289.1329	145.0701	2
7					R	**175.1190**	88.0631	158.0924	79.5498	1

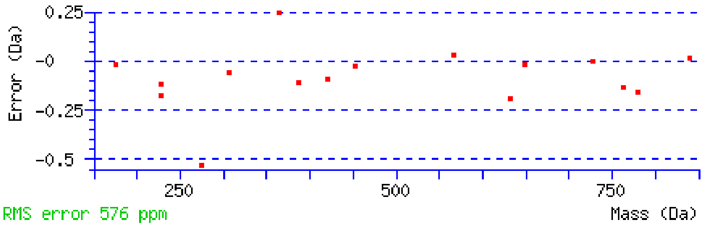

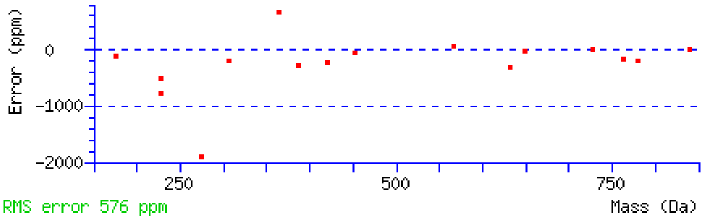

All matches to this query

Score	Mr(calc):	Delta	Sequence
46.5	952.4623	0.0016	LLCNFMR
13.2	952.4630	0.0009	LLDNSALK

13.1	952.4630	0.0009	LIIEETR
13.1	952.4630	0.0009	LLELETR
9.7	952.4630	0.0008	ILVQESGK
9.5	952.4565	0.0074	LLRSCPK
9.2	952.4630	0.0008	IIEVSGQK
9.1	952.4630	0.0009	ILTLEER
9.0	951.4539	1.0100	GSGGRGLLVG
8.3	951.4579	1.0060	GSPVFLPR

Spectrum No: 48; Query: 34191; Rank: 1

Peptide View

MS/MS Fragmentation of **LVSLIGSK**
Found in **IPI00016832**, Tax_Id=9606 Gene_Symbol=PSMA1 Isoform Short of Proteasome subunit alpha type-1

Match to Query 34191: 815.512948 from(408.763750,2+)
Title: 090702LimSK_Exosome2_04.3967.3967.2.dta
Data file C:\Temp\Mascoti\LSK_2D\090702LimSK_Exosome2_01_2D.mgf

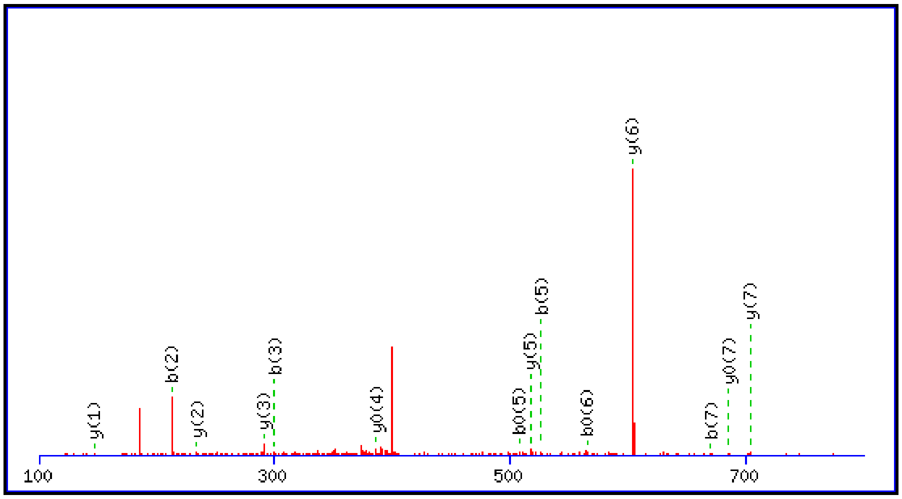

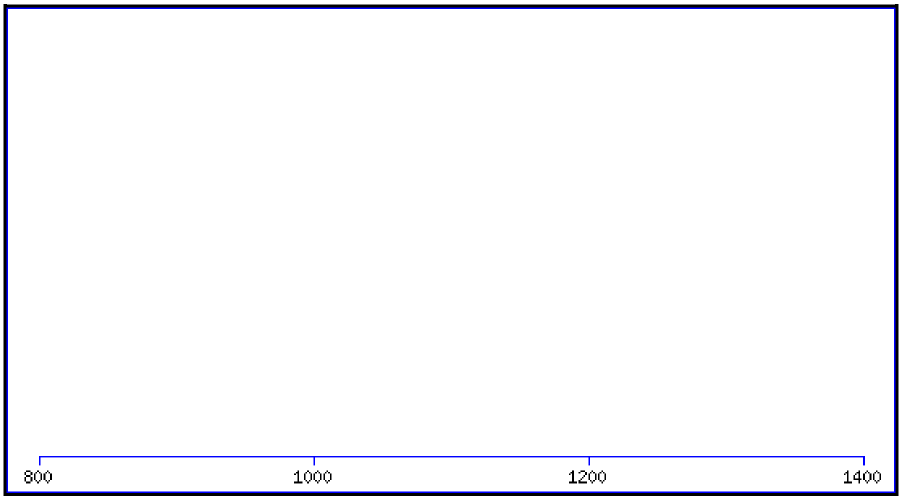

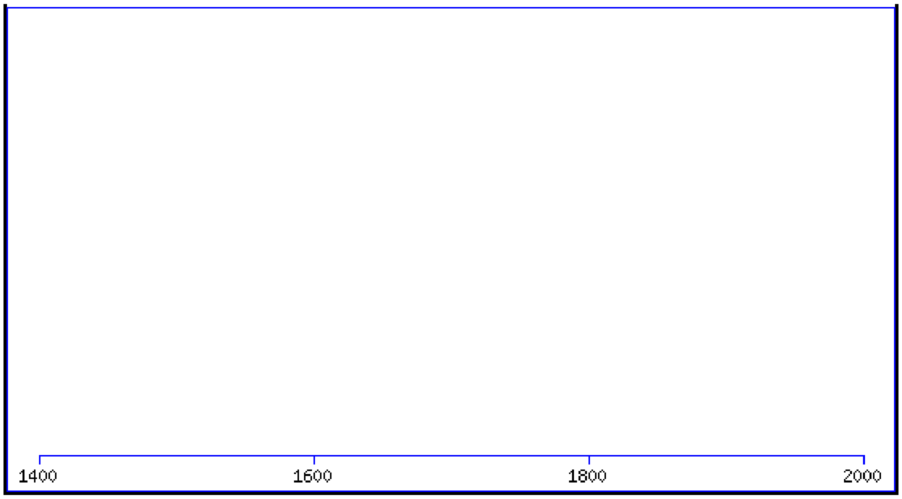

Monoisotopic mass of neutral peptide Mr(calc): 815.5116
Fixed modifications: Carbamidomethyl (C)
Ions Score: 39 **Expect:** 0.0061
Matches (Bold Red): 14/64 fragment ions using 36 most intense peaks

#	b	b^{++}	b^{0}	b^{0++}	Seq.	y	y^{++}	y*	y^{*++}	y^{0}	y^{0++}	#
1	114.0913	57.5493			L							8
2	**213.1598**	107.0835			V	**703.4349**	352.2211	686.4083	343.7078	**685.4243**	343.2158	7
3	**300.1918**	150.5995	282.1812	141.5942	S	**604.3665**	302.6869	587.3399	294.1736	586.3559	293.6816	6
4	413.2758	207.1416	395.2653	198.1363	L	**517.3344**	259.1709	500.3079	250.6576	499.3239	250.1656	5
5	**526.3599**	263.6836	**508.3493**	254.6783	I	404.2504	202.6288	387.2238	194.1155	**386.2398**	193.6235	4
6	583.3814	292.1943	**565.3708**	283.1890	G	**291.1663**	146.0868	274.1397	137.5735	273.1557	137.0815	3
7	**670.4134**	335.7103	652.4028	326.7051	S	**234.1448**	117.5761	217.1183	109.0628	216.1343	108.5708	2
8					K	**147.1128**	74.0600	130.0863	65.5468			1

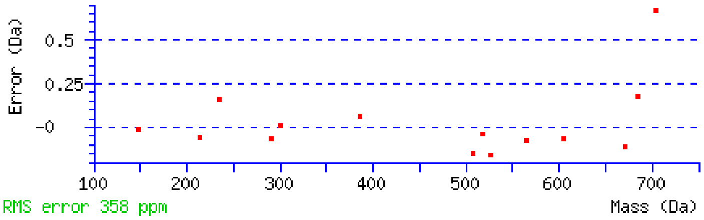

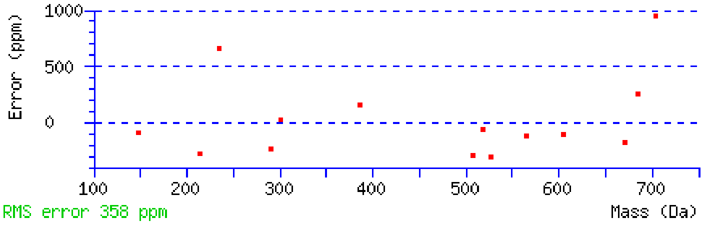

All matches to this query

Score	Mr(calc):	Delta	Sequence
39.2	815.5116	0.0013	LVSLIGSK
24.7	815.5116	0.0013	IVSISLGK
9.3	815.5116	0.0013	VISKLEK
8.7	813.5072	2.0057	LPSTLRK
7.0	815.5116	0.0013	IVTVEKK
7.0	815.5116	0.0013	LVVTKEK
6.4	815.5116	0.0013	IVELSKK
5.3	814.5025	1.0105	QILARSK
5.1	813.5072	2.0057	AILQKNK
5.0	814.5025	1.0105	KKLGGANK

Spectrum No: 49; Query: 78840; Rank: 1

Peptide View

MS/MS Fragmentation of **NQYDNDVTVWSPQGR**
Found in **IPI00016832**, Tax_Id=9606 Gene_Symbol=PSMA1 Isoform Short of Proteasome subunit alpha type-1

Match to Query 78840: 1777.805768 from(889.910160,2+)
Title: 090702LimSK_Exosome2_05.5589.5589.2.dta
Data file C:\Temp\Mascoti\LSK_2D\090702LimSK_Exosome2_01_2D.mgf

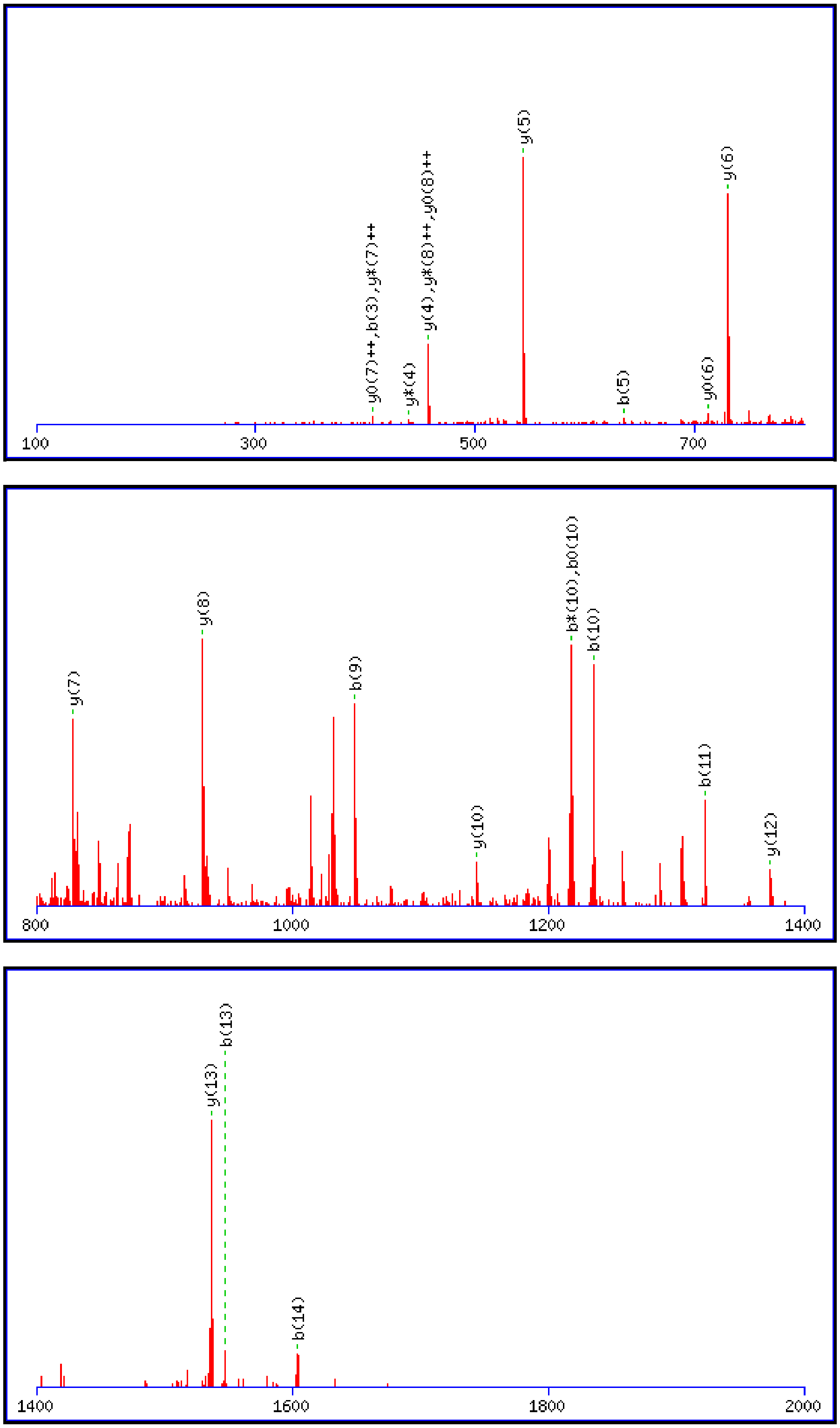

Monoisotopic mass of neutral peptide Mr(calc): 1777.8020
Fixed modifications: Carbamidomethyl (C)
Ions Score: 68 **Expect:** 4.1e-005
Matches (Bold Red): 23/154 fragment ions using 37 most intense peaks

		++		++	0	0++			++		++	0	0++	

#	b	b	b*	b*	b	b	Seq.	y	y	y*	y*	y	y	#
1	115.0502	58.0287	98.0237	49.5155			N							15
2	243.1088	122.0580	226.0822	113.5448			Q	1664.7663	832.8868	1647.7398	824.3735	1646.7558	823.8815	14
3	**406.1721**	203.5897	389.1456	195.0764			Y	**1536.7077**	768.8575	1519.6812	760.3442	1518.6972	759.8522	13
4	521.1991	261.1032	504.1725	252.5899	503.1885	252.0979	D	**1373.6444**	687.3258	1356.6179	678.8126	1355.6339	678.3206	12
5	**635.2420**	318.1246	618.2154	309.6114	617.2314	309.1193	N	1258.6175	629.8124	1241.5909	621.2991	1240.6069	620.8071	11
6	750.2689	375.6381	733.2424	367.1248	732.2584	366.6328	D	**1144.5745**	572.7909	1127.5480	564.2776	1126.5640	563.7856	10
7	849.3373	425.1723	832.3108	416.6590	831.3268	416.1670	V	1029.5476	515.2774	1012.5211	506.7642	1011.5370	506.2722	9
8	950.3850	475.6961	933.3585	467.1829	932.3745	466.6909	T	**930.4792**	465.7432	913.4526	**457.2300**	912.4686	**456.7380**	8
9	**1049.4534**	525.2304	1032.4269	516.7171	1031.4429	516.2251	V	**829.4315**	415.2194	812.4050	**406.7061**	811.4209	**406.2141**	7
10	**1235.5327**	618.2700	**1218.5062**	609.7567	**1217.5222**	609.2647	W	**730.3631**	365.6852	713.3366	357.1719	**712.3525**	356.6799	6
11	**1322.5648**	661.7860	1305.5382	653.2727	1304.5542	652.7807	S	**544.2838**	272.6455	527.2572	264.1323	526.2732	263.6402	5
12	1419.6175	710.3124	1402.5910	701.7991	1401.6070	701.3071	P	**457.2518**	229.1295	**440.2252**	220.6162			4
13	**1547.6761**	774.3417	1530.6496	765.8284	1529.6655	765.3364	Q	360.1990	180.6031	343.1724	172.0899			3
14	**1604.6976**	802.8524	1587.6710	794.3392	1586.6870	793.8471	G	232.1404	116.5738	215.1139	108.0606			2
15							R	175.1190	88.0631	158.0924	79.5498			1

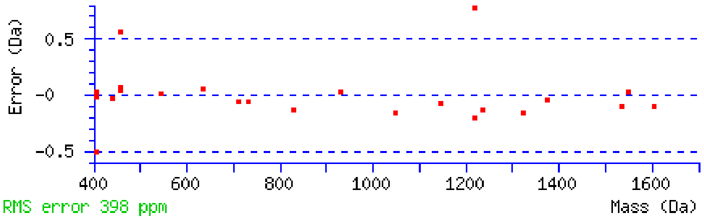

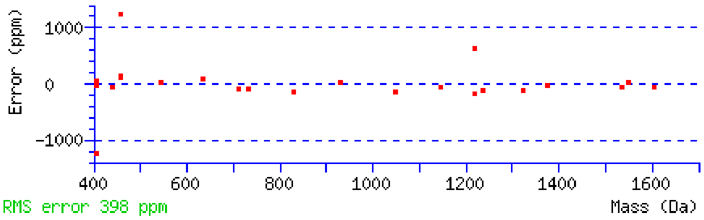

All matches to this query

Score	Mr(calc):	Delta	Sequence
67.8	1777.8020	0.0038	NQYDNDVTVWSPQGR
4.3	1777.7897	0.0161	NRHGKQVLDGNSNPY
3.9	1775.7909	2.0149	XLKMETPGMTTPSLK
3.6	1777.8031	0.0027	NYILMLTNSLEEMK
3.1	1775.7909	2.0149	XLKMETPGMTTPSLK
2.9	1775.7909	2.0149	XLKMETPGMTTPSLK
2.7	1777.8031	0.0027	NYILMLTNSLEEMK
2.2	1777.7913	0.0144	VAKTGSSNAYLHSKR
2.2	1777.7913	0.0144	VAKTGSSNAYLHSKR
2.2	1777.7913	0.0144	VAKTGSSNAYLHSKR

Spectrum No: 50; Query: 38300; Rank: 1

Peptide View

MS/MS Fragmentation of **NVSIGIVGK**
Found in **IPI00016832**, Tax_Id=9606 Gene_Symbol=PSMA1 Isoform Short of Proteasome subunit alpha type-1

Match to Query 38300: 885.530528 from(443.772540,2+)
Title: 090702LimSK_Exosome2_05.3738.3738.2.dta
Data file C:\Temp\Mascoti\LSK_2D\090702LimSK_Exosome2_01_2D.mgf

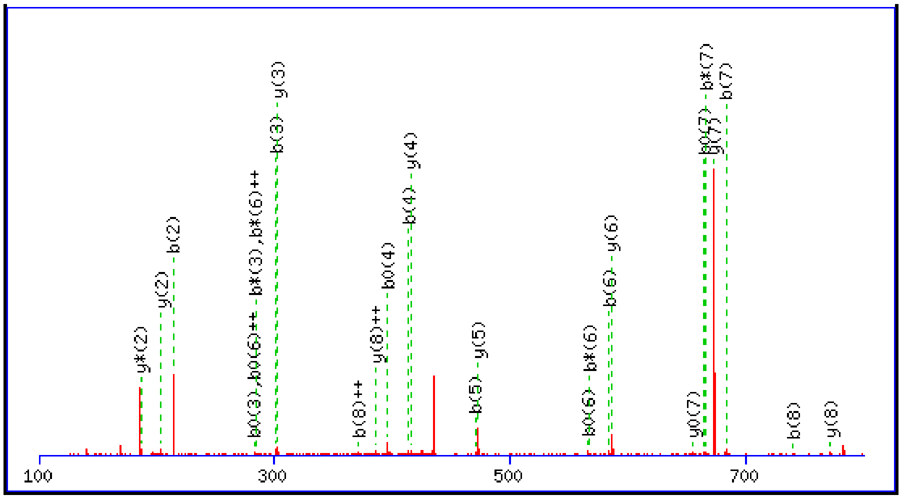

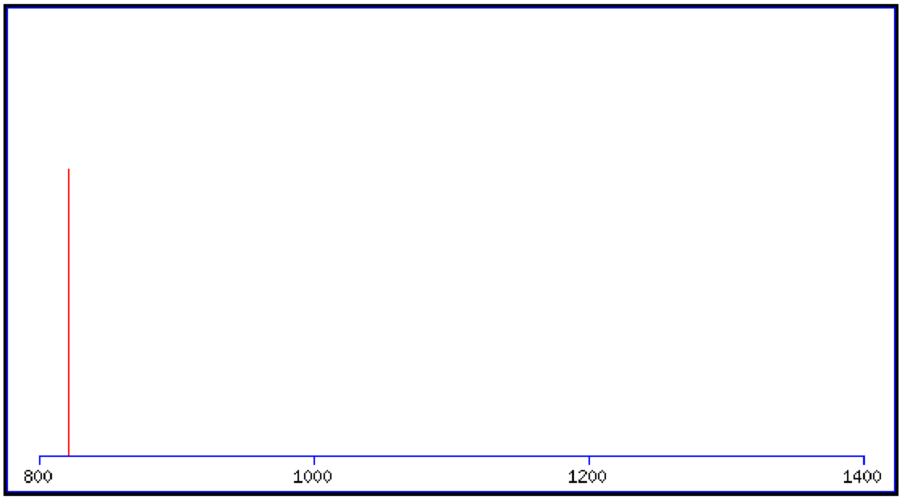

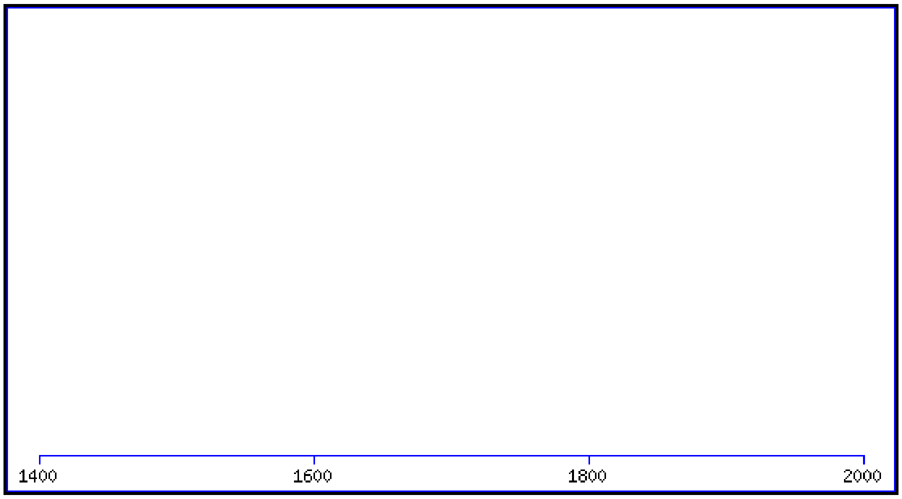

Monoisotopic mass of neutral peptide Mr(calc): 885.5284
Fixed modifications: Carbamidomethyl (C)
Ions Score: 52 **Expect:** 0.00047
Matches (Bold Red): 27/80 fragment ions using 45 most intense peaks

#	b	b^{++}	b*	b^{*++}	b^0	b^{0++}	Seq.	y	y^{++}	y*	y^{*++}	y^0	y^{0++}	#
1	115.0502	58.0287	98.0237	49.5155			N							9
2	**214.1186**	107.5629	197.0921	99.0497			V	**772.4927**	**386.7500**	755.4662	378.2367	754.4822	377.7447	8
3	**301.1506**	151.0790	**284.1241**	142.5657	**283.1401**	142.0737	S	**673.4243**	337.2158	656.3978	328.7025	**655.4137**	328.2105	7
4	**414.2347**	207.6210	397.2082	199.1077	**396.2241**	198.6157	I	**586.3923**	293.6998	569.3657	285.1865			6
5	**471.2562**	236.1317	454.2296	227.6185	453.2456	227.1264	G	**473.3082**	237.1577	456.2817	228.6445			5
6	**584.3402**	292.6738	**567.3137**	**284.1605**	**566.3297**	**283.6685**	I	**416.2867**	208.6470	399.2602	200.1337			4

**7**	**683.4087**	342.2080	**666.3821**	333.6947	**665.3981**	333.2027	**V**	**303.2027**	152.1050	286.1761	143.5917			**3**
**8**	**740.4301**	**370.7187**	723.4036	362.2054	722.4195	361.7134	**G**	**204.1343**	102.5708	**187.1077**	94.0575			**2**
**9**							**K**	147.1128	74.0600	130.0863	65.5468			**1**

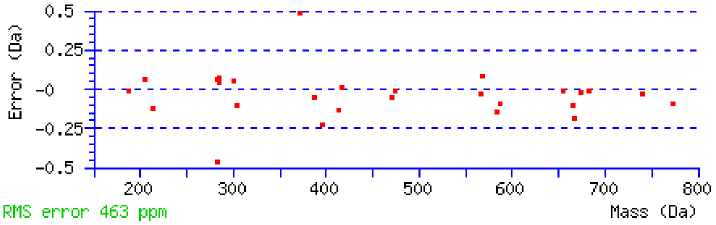

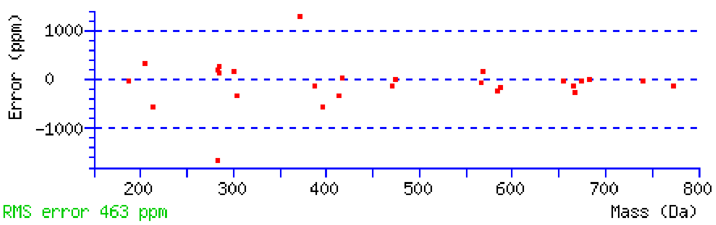

All matches to this query

Score	Mr(calc):	Delta	Sequence
52.0	885.5284	0.0022	NVSIGIVGK
12.0	885.5284	0.0022	LVIQTQGK
7.7	884.5232	1.0073	VLWLAQR
4.8	884.5232	1.0073	WLLGIAGR
4.7	885.5283	0.0022	VIEELRK
3.3	885.5283	0.0022	LLEDLRK
2.5	885.5283	0.0022	LEDLLRK
1.2	885.5284	0.0022	NVQVSLVK
1.0	885.5284	0.0021	VGNLTVVGK
0.5	885.5297	0.0008	WRQLRK

Spectrum No: 51; Query: 36965; Rank: 1

Peptide View

MS/MS Fragmentation of **QGSATVGLK**
Found in **IPI00016832**, Tax_Id=9606 Gene_Symbol=PSMA1 Isoform Short of Proteasome subunit alpha type-1

Match to Query 36965: 859.477368 from(430.745960,2+)
Title: 090702LimSK_Exosome2_05.1058.1058.2.dta
Data file C:\Temp\Mascoti\LSK_2D\090702LimSK_Exosome2_01_2D.mgf

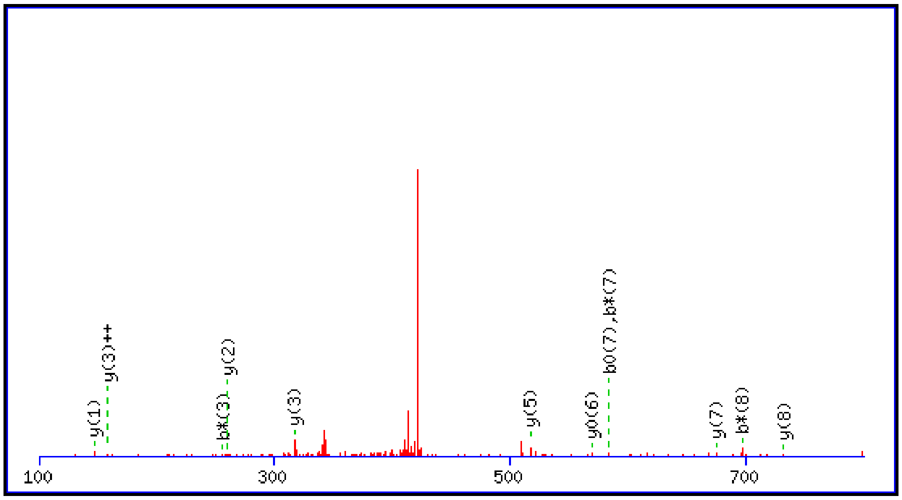

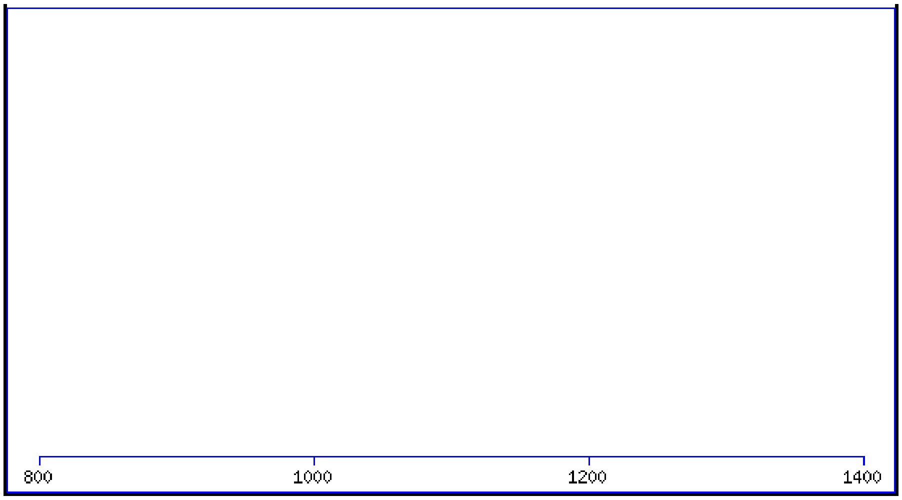

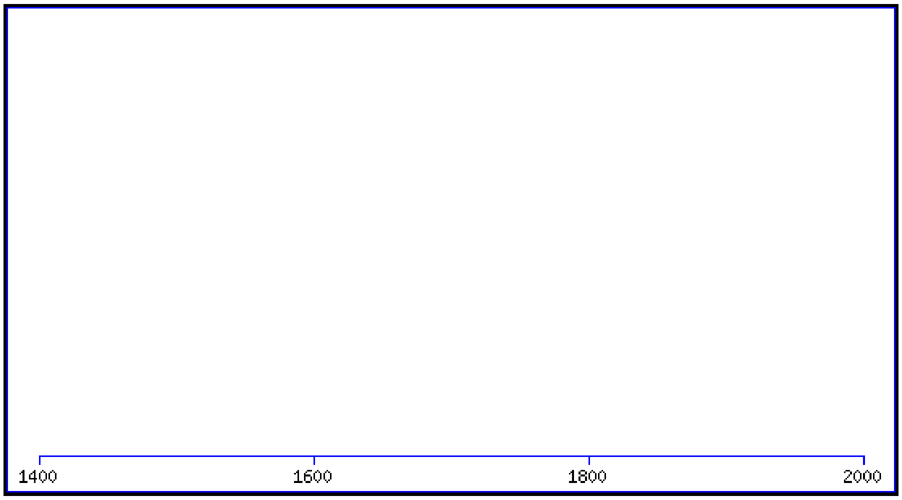

Monoisotopic mass of neutral peptide Mr(calc): 859.4763
Fixed modifications: Carbamidomethyl (C)
Ions Score: 31 **Expect:** 0.12
Matches (Bold Red): 12/84 fragment ions using 26 most intense peaks

#	b	b^{++}	b*	b^{*++}	b^{0}	b^{0++}	Seq.	y	y^{++}	y*	y^{*++}	y^{0}	y^{0++}	#
1	129.0659	65.0366	112.0393	56.5233			Q							9
2	186.0873	93.5473	169.0608	85.0340			G	**732.4250**	366.7162	715.3985	358.2029	714.4145	357.7109	8
3	273.1193	137.0633	**256.0928**	128.5500	255.1088	128.0580	S	**675.4036**	338.2054	658.3770	329.6921	657.3930	329.2001	7
4	344.1565	172.5819	327.1299	164.0686	326.1459	163.5766	A	588.3715	294.6894	571.3450	286.1761	**570.3610**	285.6841	6
5	445.2041	223.1057	428.1776	214.5924	427.1936	214.1004	T	**517.3344**	259.1709	500.3079	250.6576	499.3239	250.1656	5
6	544.2726	272.6399	527.2460	264.1266	526.2620	263.6346	V	416.2867	208.6470	399.2602	200.1337			4
7	601.2940	301.1506	**584.2675**	292.6374	**583.2835**	292.1454	G	**317.2183**	**159.1128**	300.1918	150.5995			3
8	714.3781	357.6927	**697.3515**	349.1794	696.3675	348.6874	L	**260.1969**	130.6021	243.1703	122.0888			2
9							K	**147.1128**	74.0600	130.0863	65.5468			1

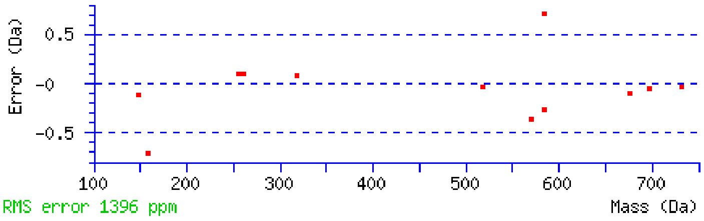

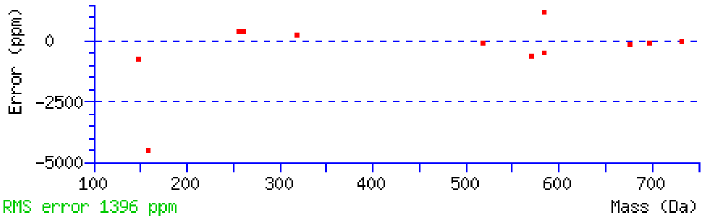

All matches to this query

Score	Mr(calc):	Delta	Sequence

31.1	859.4763	0.0010	QGSATVGLK
20.8	859.4763	0.0011	QGSAKLEK
14.4	859.4763	0.0010	QGLQVSTK
13.5	859.4763	0.0011	ASALSLGNK
11.4	859.4763	0.0010	QVNTASIK
10.9	859.4763	0.0010	KGSDVVAGK
10.7	858.4810	0.9963	LAASQIEK
10.0	859.4763	0.0011	QKSEIQK
10.0	859.4763	0.0011	QQSELKK
9.8	859.4763	0.0010	TGGQLSLGK

Spectrum No: 52; Query: 36070; Rank: 1

Peptide View

MS/MS Fragmentation of **TQIPTQR**
Found in **IPI00016832**, Tax_Id=9606 Gene_Symbol=PSMA1 Isoform Short of Proteasome subunit alpha type-1

Match to Query 36070: 842.461788 from(422.238170,2+)
Title: 090702LimSK_Exosome2_05.1036.1036.2.dta
Data file C:\Temp\Mascoti\LSK_2D\090702LimSK_Exosome2_01_2D.mgf

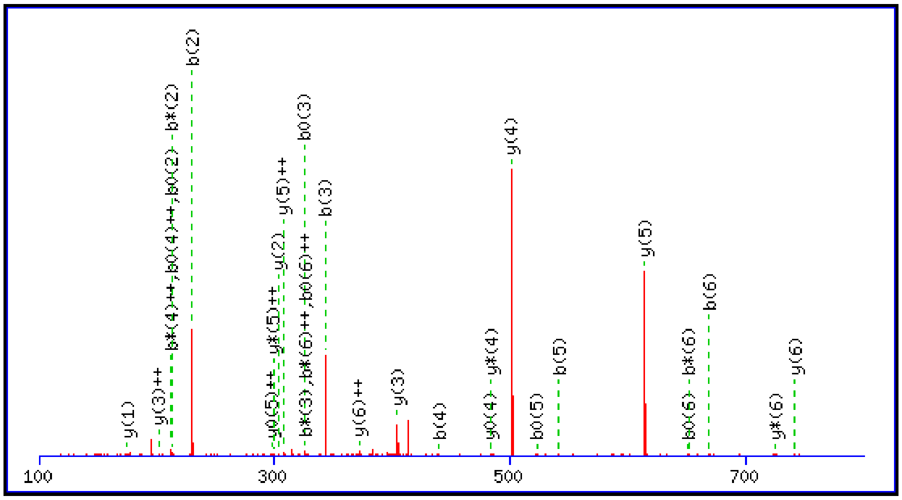

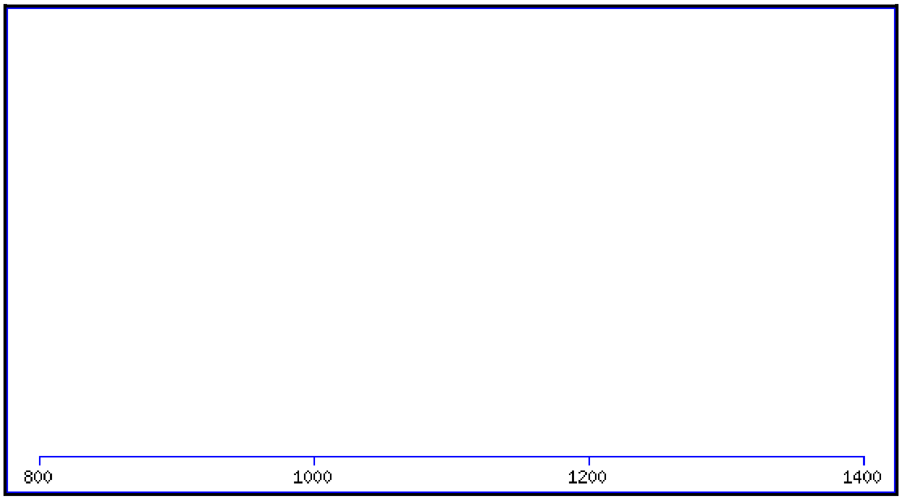

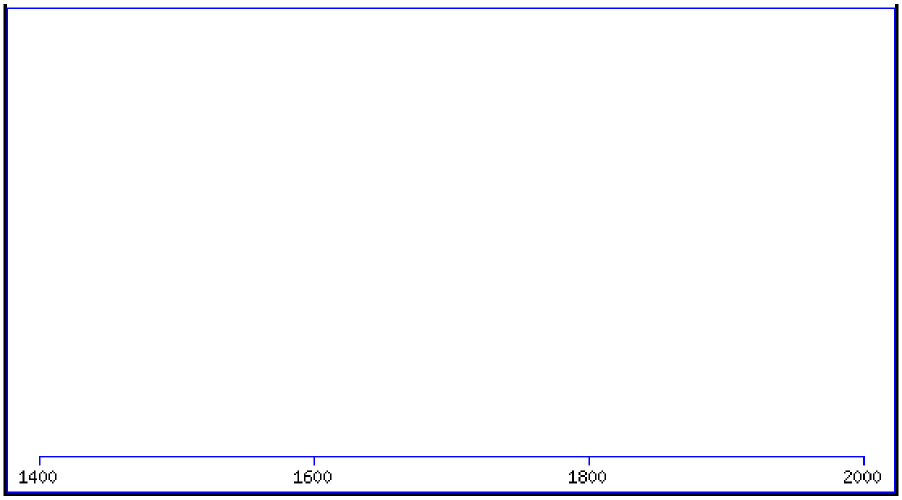

Monoisotopic mass of neutral peptide Mr(calc): 842.4610
Fixed modifications: Carbamidomethyl (C)
Ions Score: 31 **Expect:** 0.055
Matches (Bold Red): 30/66 fragment ions using 61 most intense peaks

#	b	b^{++}	b*	b^{*++}	b^0	b^{0++}	Seq.	y	y^{++}	y*	y^{*++}	y^0	y^{0++}	#
1	102.0550	51.5311			84.0444	42.5258	T							7
2	**230.1135**	115.5604	**213.0870**	107.0471	**212.1030**	106.5551	Q	**742.4206**	**371.7139**	**725.3941**	363.2007	724.4101	362.7087	6
3	**343.1976**	172.1024	**326.1710**	163.5892	**325.1870**	163.0972	I	**614.3620**	**307.6847**	597.3355	**299.1714**	596.3515	**298.6794**	5
4	**440.2504**	220.6288	423.2238	**212.1155**	422.2398	**211.6235**	P	**501.2780**	251.1426	**484.2514**	242.6294	**483.2674**	242.1373	4
5	**541.2980**	271.1527	524.2715	262.6394	**523.2875**	262.1474	T	**404.2252**	**202.6162**	387.1987	194.1030	386.2146	193.6110	3
6	**669.3566**	335.1819	**652.3301**	**326.6687**	**651.3461**	**326.1767**	Q	**303.1775**	152.0924	286.1510	143.5791			2
7							R	**175.1190**	88.0631	158.0924	79.5498			1

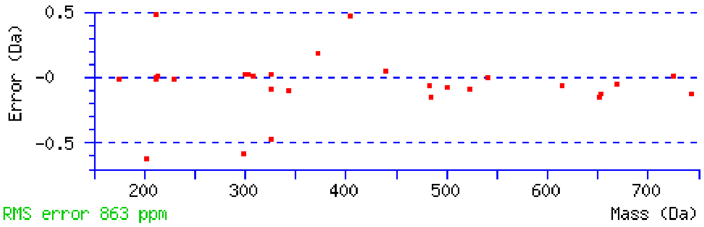

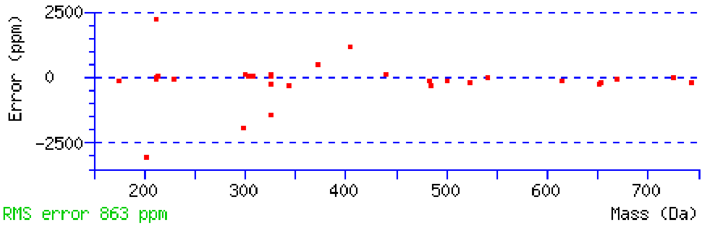

All matches to this query

Score	Mr(calc):	Delta	Sequence
31.5	842.4610	0.0008	TQIPTQR
25.1	842.4610	0.0008	TKLDNPR
22.1	842.4610	0.0008	ASALTQPR
9.4	840.4470	2.0148	KLSKGTK
9.0	842.4610	0.0008	LSGRSNPI
7.7	840.4566	2.0052	XGAGPERR
7.4	842.4610	0.0008	DPEAKKR
6.3	842.4610	0.0008	KVNPETR
4.7	841.4658	0.9960	TQLPLDR
4.7	842.4610	0.0008	ANELLQR

Spectrum No: 53; Query: 74154; Rank: 1

Peptide View

MS/MS Fragmentation of **ATCIGNNSAAAVSMLK**
Found in **IPI00299155**, Tax_Id=9606 Gene_Symbol=PSMA4 Proteasome subunit alpha type-4

Match to Query 74154: 1606.784248 from(804.399400,2+)
Title: 090702LimSK_Exosome2_04.5721.5721.2.dta
Data file C:\Temp\Mascoti\LSK_2D\090702LimSK_Exosome2_01_2D.mgf

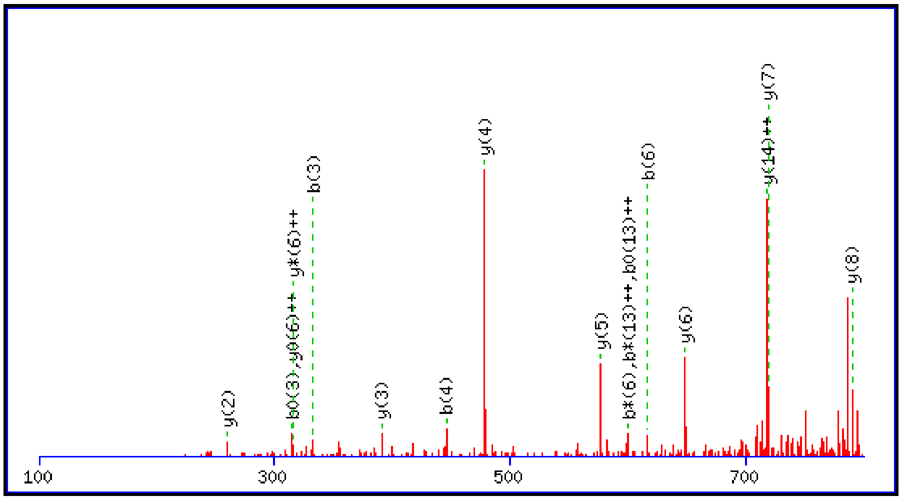

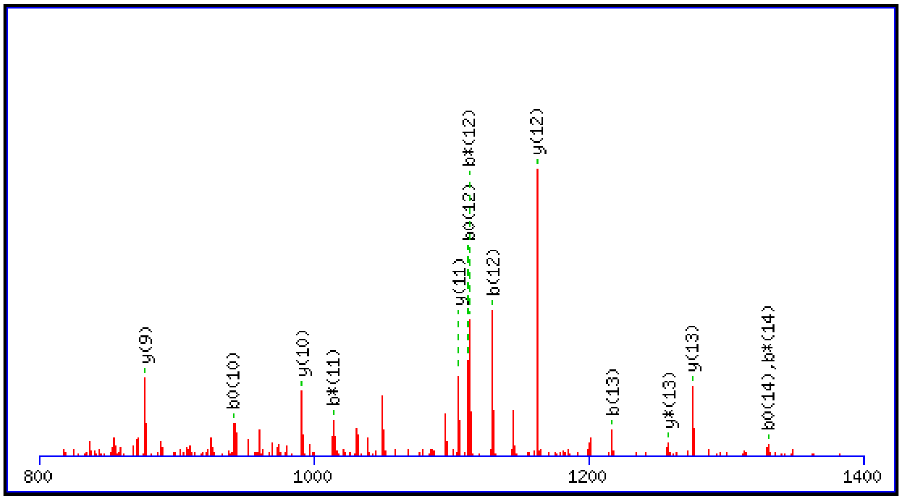

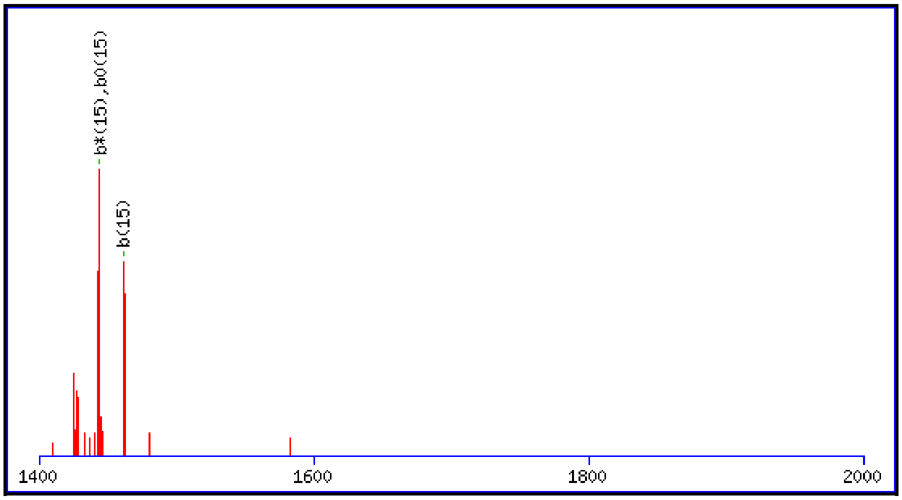

Monoisotopic mass of neutral peptide Mr(calc): 1606.7807
Fixed modifications: Carbamidomethyl (C)
Ions Score: 103 **Expect:** 1e-008
Matches (Bold Red): 34/162 fragment ions using 50 most intense peaks

#	b	b^{++}	b*	b^{*++}	b^0	b^{0++}	Seq.	y	y^{++}	y*	y^{*++}	y^0	y^{0++}	#
1	72.0444	36.5258					A							16

2	173.0921	87.0497			155.0815	78.0444	T	1536.7509	768.8791	1519.7243	760.3658	1518.7403	759.8738	15
3	**333.1227**	167.0650			**315.1122**	158.0597	C	1435.7032	**718.3552**	1418.6766	709.8420	1417.6926	709.3500	14
4	**446.2068**	223.6070			428.1962	214.6017	I	**1275.6725**	638.3399	**1258.6460**	629.8266	1257.6620	629.3346	13
5	503.2282	252.1178			485.2177	243.1125	G	**1162.5885**	581.7979	1145.5619	573.2846	1144.5779	572.7926	12
6	**617.2712**	309.1392	**600.2446**	300.6260	599.2606	300.1339	N	**1105.5670**	553.2871	1088.5405	544.7739	1087.5565	544.2819	11
7	731.3141	366.1607	714.2876	357.6474	713.3035	357.1554	N	**991.5241**	496.2657	974.4975	487.7524	973.5135	487.2604	10
8	818.3461	409.6767	801.3196	401.1634	800.3356	400.6714	S	**877.4812**	439.2442	860.4546	430.7309	859.4706	430.2389	9
9	889.3832	445.1953	872.3567	436.6820	871.3727	436.1900	A	**790.4491**	395.7282	773.4226	387.2149	772.4386	386.7229	8
10	960.4204	480.7138	943.3938	472.2005	**942.4098**	471.7085	A	**719.4120**	360.2096	702.3855	351.6964	701.4015	351.2044	7
11	1031.4575	516.2324	**1014.4309**	507.7191	1013.4469	507.2271	A	**648.3749**	324.6911	631.3484	**316.1778**	630.3643	**315.6858**	6
12	**1130.5259**	565.7666	**1113.4993**	557.2533	**1112.5153**	556.7613	V	**577.3378**	289.1725	560.3112	280.6593	559.3272	280.1673	5
13	**1217.5579**	609.2826	1200.5314	**600.7693**	1199.5473	**600.2773**	S	**478.2694**	239.6383	461.2428	231.1251	460.2588	230.6330	4
14	1348.5984	674.8028	**1331.5718**	666.2896	**1330.5878**	665.7976	M	**391.2374**	196.1223	374.2108	187.6090			3
15	**1461.6825**	731.3449	**1444.6559**	722.8316	**1443.6719**	722.3396	L	**260.1969**	130.6021	243.1703	122.0888			2
16							K	147.1128	74.0600	130.0863	65.5468			1

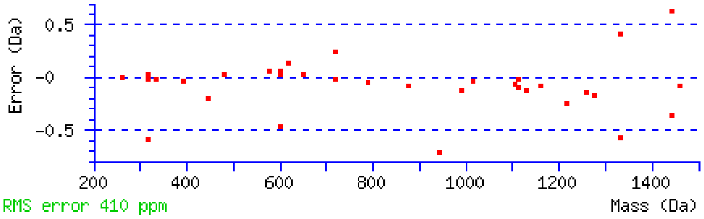

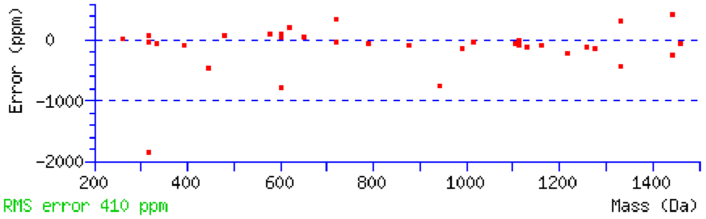

All matches to this query

Score	Mr(calc):	Delta	Sequence
103.2	1606.7807	0.0036	ATCIGNNSAAAVSMLK
15.1	1606.7838	0.0004	YINSTLPNDSENIK
3.8	1606.7789	0.0053	EKLALAHDTALSMK
3.4	1606.7789	0.0053	EKLALAHDTALSMK
2.4	1606.7913	-0.0070	FVGTDPASDEVVLMK
1.3	1606.7790	0.0053	TLPSHLCTSALLSK
1.1	1604.7646	2.0196	RNMYTAHPLPIGR
0.7	1606.7881	-0.0038	SMPKDAQMMAQILK
0.6	1606.7895	-0.0053	YLSDLFTTLVDLK
0.6	1606.7895	-0.0053	YLSDLFTTLVDLK

Spectrum No: 54; Query: 36836; Rank: 1

Peptide View

MS/MS Fragmentation of **EVEQLIK**
Found in **IPI00299155**, Tax_Id=9606 Gene_Symbol=PSMA4 Proteasome subunit alpha type-4

Match to Query 36836: 857.487188 from(429.750870,2+)
Title: 090702LimSK_Exosome2_03.2471.2471.2.dta
Data file C:\Temp\Mascoti\LSK_2D\090702LimSK_Exosome2_01_2D.mgf

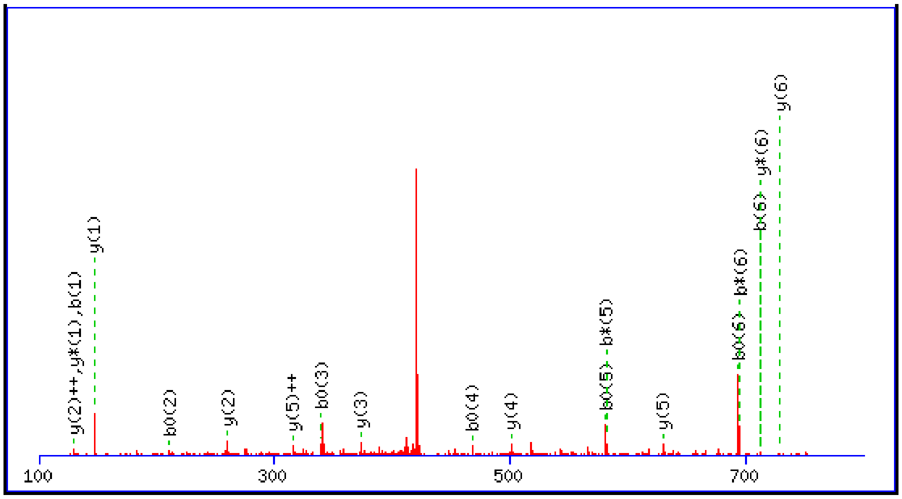

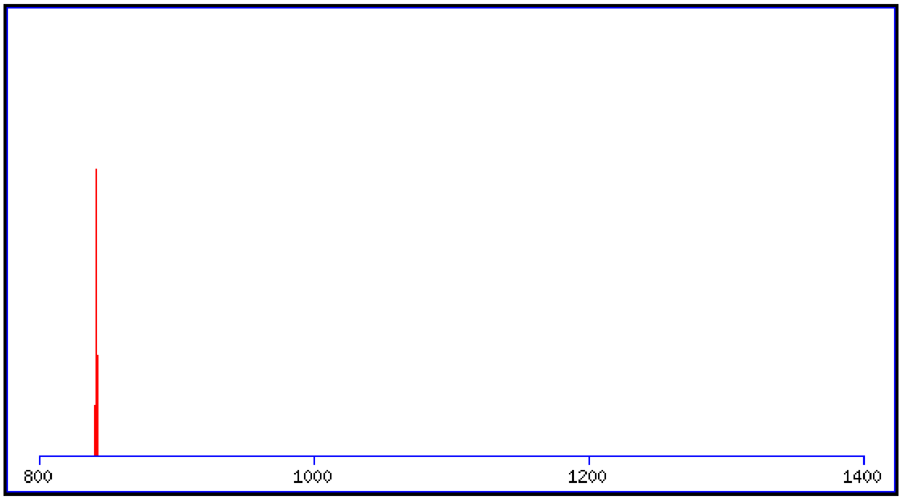

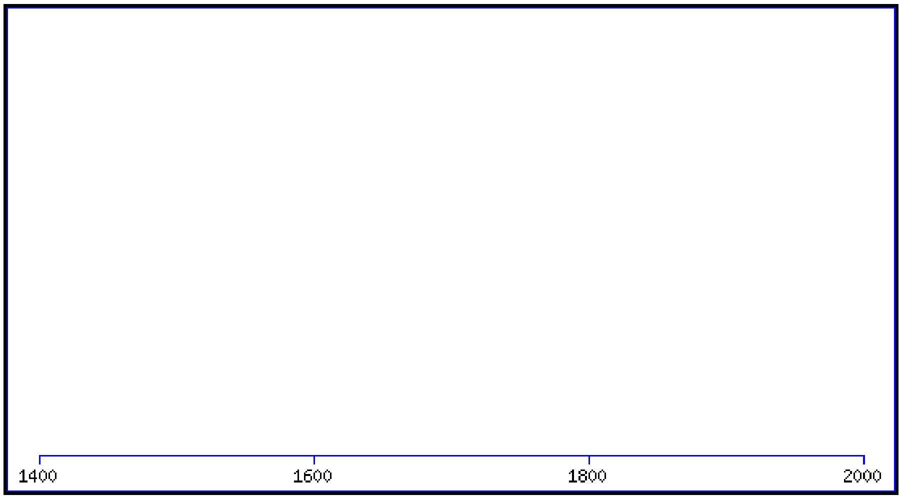

Monoisotopic mass of neutral peptide Mr(calc): 857.4858
Fixed modifications: Carbamidomethyl (C)
Ions Score: 25 **Expect:** 0.34
Matches (Bold Red): 19/58 fragment ions using 43 most intense peaks

#	b	b^{++}	b*	b^{*++}	b^0	b^{0++}	Seq.	y	y^{++}	y*	y^{*++}	y^0	y^{0++}	#
1	**130.0499**	65.5286			112.0393	56.5233	E							7
2	229.1183	115.0628			**211.1077**	106.0575	V	**729.4505**	365.2289	**712.4240**	356.7156	711.4400	356.2236	6
3	358.1609	179.5841			**340.1503**	170.5788	E	**630.3821**	**315.6947**	613.3556	307.1814	612.3715	306.6894	5
4	486.2195	243.6134	469.1929	235.1001	**468.2089**	234.6081	Q	**501.3395**	251.1734	484.3130	242.6601			4
5	599.3035	300.1554	**582.2770**	291.6421	**581.2930**	291.1501	L	**373.2809**	187.1441	356.2544	178.6308			3
6	**712.3876**	356.6974	**695.3610**	348.1842	**694.3770**	347.6921	I	**260.1969**	**130.6021**	243.1703	122.0888			2

7							K	147.1128	74.0600	130.0863	65.5468		1

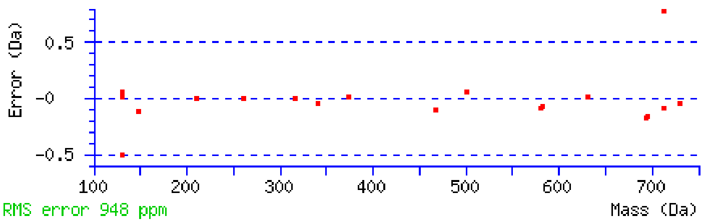

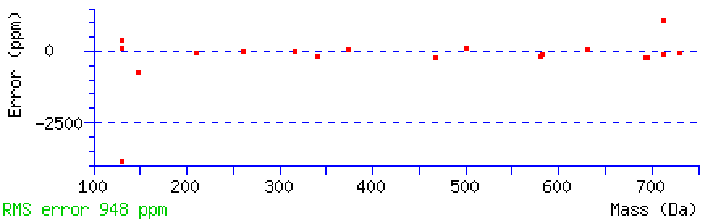

All matches to this query

Score	Mr(calc):	Delta	Sequence
24.7	857.4858	0.0014	EVEQLIK
17.1	856.4767	1.0105	NLSLGPTR
15.8	857.4858	0.0014	EVLLQEK
14.7	856.4823	1.0049	LYKLLK
14.6	857.4858	0.0014	IQVLEEK
14.4	856.4841	1.0031	LPGGLCIK
14.4	856.4879	0.9993	LSRQPTR
13.7	857.4858	0.0014	NIILEEK
13.7	857.4858	0.0014	NILLEEK
13.7	857.4858	0.0014	NLILEEK

Spectrum No: 55; Query: 56921; Rank: 1

Peptide View

MS/MS Fragmentation of **LLDEVFFSEK**
Found in **IPI00299155**, Tax_Id=9606 Gene_Symbol=PSMA4 Proteasome subunit alpha type-4

Match to Query 56921: 1225.625528 from(613.820040,2+)
Title: 090702LimSK_Exosome2_05.7883.7883.2.dta
Data file C:\Temp\Mascoti\LSK_2D\090702LimSK_Exosome2_01_2D.mgf

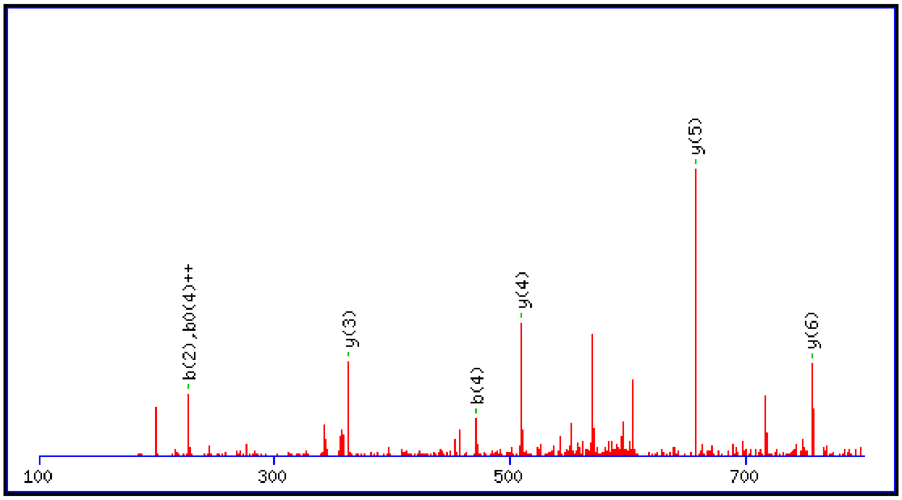

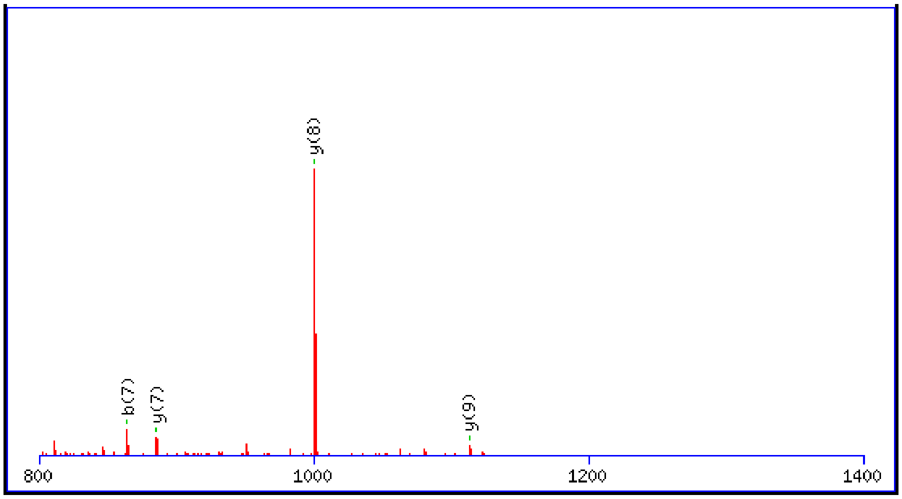

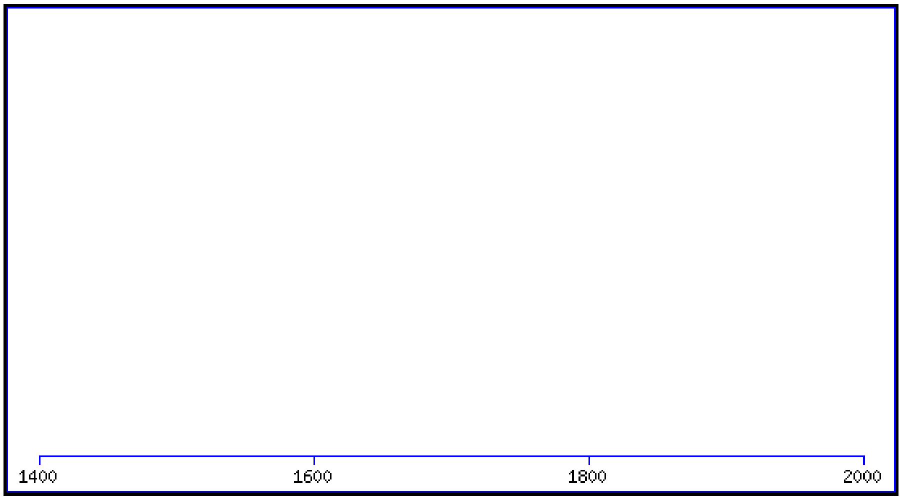

Monoisotopic mass of neutral peptide Mr(calc): 1225.6230
Fixed modifications: Carbamidomethyl (C)
Ions Score: 73 **Expect:** 8.2e-006
Matches (Bold Red): 11/84 fragment ions using 11 most intense peaks

#	b	b^{++}	b^0	b^{0++}	Seq.	y	y^{++}	y^*	y^{*++}	y^0	y^{0++}	#
1	114.0913	57.5493			L							10
2	**227.1754**	114.0913			L	**1113.5463**	557.2768	1096.5197	548.7635	1095.5357	548.2715	9
3	342.2023	171.6048	324.1918	162.5995	D	**1000.4622**	500.7347	983.4357	492.2215	982.4516	491.7295	8
4	**471.2449**	236.1261	453.2344	**227.1208**	E	**885.4353**	443.2213	868.4087	434.7080	867.4247	434.2160	7
5	570.3134	285.6603	552.3028	276.6550	V	**756.3927**	378.7000	739.3661	370.1867	738.3821	369.6947	6
6	717.3818	359.1945	699.3712	350.1892	F	**657.3243**	329.1658	640.2977	320.6525	639.3137	320.1605	5
7	**864.4502**	432.7287	846.4396	423.7234	F	**510.2558**	255.6316	493.2293	247.1183	492.2453	246.6263	4
8	951.4822	476.2447	933.4716	467.2395	S	**363.1874**	182.0974	346.1609	173.5841	345.1769	173.0921	3
9	1080.5248	540.7660	1062.5142	531.7608	E	276.1554	138.5813	259.1288	130.0681	258.1448	129.5761	2
10					K	147.1128	74.0600	130.0863	65.5468			1

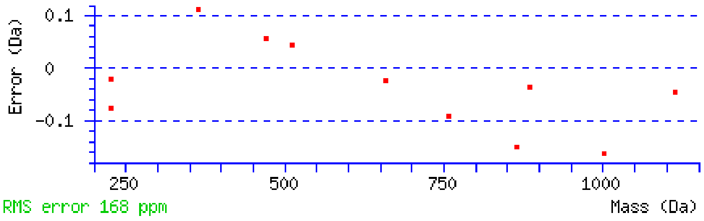

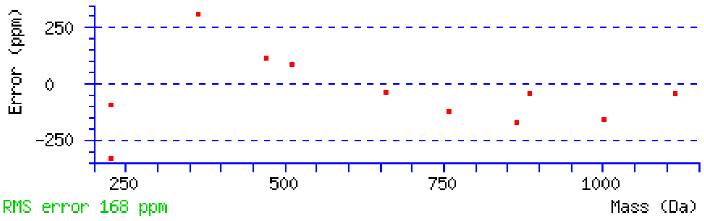

All matches to this query

Score	Mr(calc):	Delta	Sequence
72.7	1225.6230	0.0025	LLDEVFFSEK
8.9	1224.6340	0.9916	KLTGAVRSSAR
8.9	1224.6340	0.9916	KLTGAVRSSAR
7.8	1224.6115	1.0140	AGKAELLTSGAK
7.4	1225.6141	0.0114	KPMDLSTVKK
6.8	1223.6275	1.9981	NKKVDAAASIK
5.7	1224.6189	1.0066	LKLTLVSPMSG
5.6	1225.6254	0.0002	KPVMKGAKSGK
5.4	1224.6115	1.0140	KKGDLDTAAVK
4.5	1225.6343	-0.0087	EPFPPSSPLQK

Spectrum No: 56; Query: 93279; Rank: 1

Peptide View

MS/MS Fragmentation of **LNEDMACSVAGITSDANVLTNELR**
Found in **IPI00299155**, Tax_Id=9606 Gene_Symbol=PSMA4 Proteasome subunit alpha type-4

Match to Query 93279: 2592.230968 from(1297.122760,2+)
Title: 090702LimSK_Exosome2_01.9893.9893.2.dta
Data file C:\Temp\Mascoti\LSK_2D\090702LimSK_Exosome2_01_2D.mgf

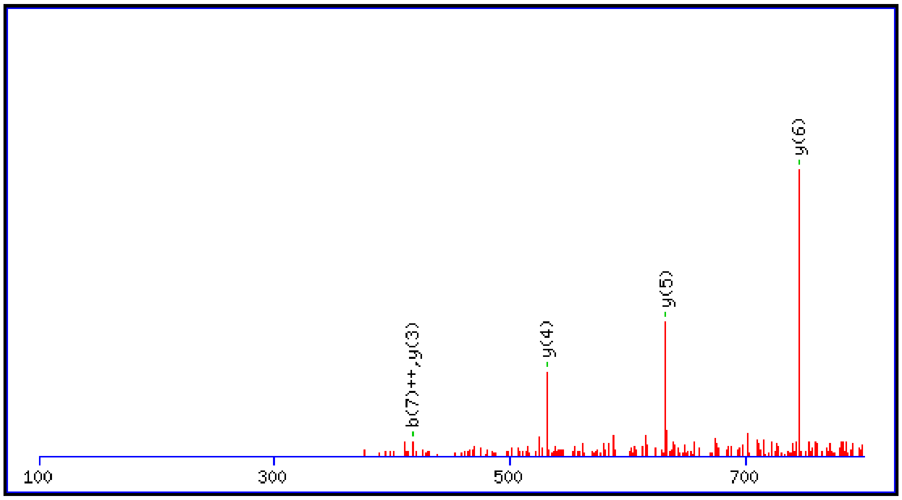

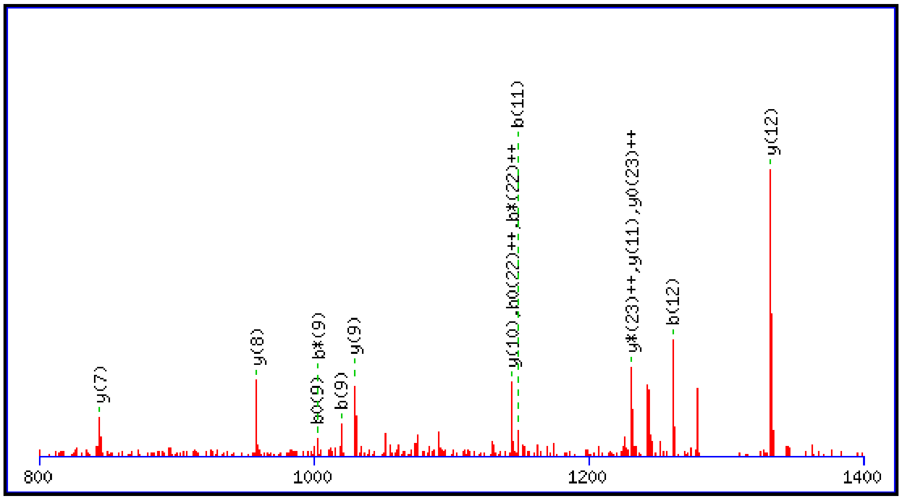

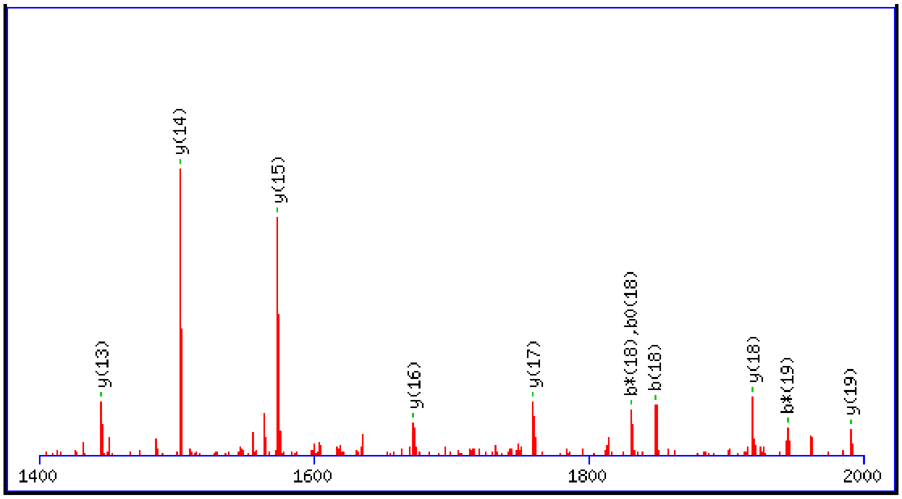

Monoisotopic mass of neutral peptide Mr(calc): 2592.2159
Fixed modifications: Carbamidomethyl (C)
Ions Score: 132 **Expect:** 1.8e-011
Matches (Bold Red): 31/266 fragment ions using 39 most intense peaks

#	b	b^{++}	b*	b^{*++}	b^0	b^{0++}	Seq.	y	y^{++}	y*	y^{*++}	y^0	y^{0++}	#
1	114.0913	57.5493					L							24
2	228.1343	114.5708	211.1077	106.0575			N	2480.1392	1240.5732	2463.1126	**1232.0600**	2462.1286	**1231.5679**	23
3	357.1769	179.0921	340.1503	170.5788	339.1663	170.0868	E	2366.0963	1183.5518	2349.0697	1175.0385	2348.0857	1174.5465	22
4	472.2038	236.6055	455.1773	228.0923	454.1932	227.6003	D	2237.0537	1119.0305	2220.0271	1110.5172	2219.0431	1110.0252	21
5	603.2443	302.1258	586.2177	293.6125	585.2337	293.1205	M	2122.0267	1061.5170	2105.0002	1053.0037	2104.0162	1052.5117	20
6	674.2814	337.6443	657.2549	329.1311	656.2708	328.6391	A	**1990.9862**	995.9968	1973.9597	987.4835	1972.9757	986.9915	19
7	834.3121	**417.6597**	817.2855	409.1464	816.3015	408.6544	C	**1919.9491**	960.4782	1902.9226	951.9649	1901.9386	951.4729	18
8	921.3441	461.1757	904.3175	452.6624	903.3335	452.1704	S	**1759.9185**	880.4629	1742.8919	871.9496	1741.9079	871.4576	17
9	**1020.4125**	510.7099	**1003.3859**	502.1966	**1002.4019**	501.7046	V	**1672.8864**	836.9469	1655.8599	828.4336	1654.8759	827.9416	16
10	1091.4496	546.2284	1074.4231	537.7152	1073.4390	537.2232	A	**1573.8180**	787.4127	1556.7915	778.8994	1555.8075	778.4074	15
11	**1148.4711**	574.7392	1131.4445	566.2259	1130.4605	565.7339	G	**1502.7809**	751.8941	1485.7544	743.3808	1484.7703	742.8888	14
12	**1261.5551**	631.2812	1244.5286	622.7679	1243.5446	622.2759	I	**1445.7594**	723.3834	1428.7329	714.8701	1427.7489	714.3781	13
13	1362.6028	681.8050	1345.5763	673.2918	1344.5922	672.7998	T	**1332.6754**	666.8413	1315.6488	658.3281	1314.6648	657.8360	12
14	1449.6348	725.3211	1432.6083	716.8078	1431.6243	716.3158	S	**1231.6277**	616.3175	1214.6012	607.8042	1213.6171	607.3122	11
15	1564.6618	782.8345	1547.6352	774.3213	1546.6512	773.8292	D	**1144.5957**	572.8015	1127.5691	564.2882	1126.5851	563.7962	10
16	1635.6989	818.3531	1618.6724	809.8398	1617.6883	809.3478	A	**1029.5687**	515.2880	1012.5422	506.7747	1011.5582	506.2827	9
17	1749.7418	875.3746	1732.7153	866.8613	1731.7313	866.3693	N	**958.5316**	479.7694	941.5051	471.2562	940.5211	470.7642	8
18	**1848.8102**	924.9088	**1831.7837**	916.3955	**1830.7997**	915.9035	V	**844.4887**	422.7480	827.4621	414.2347	826.4781	413.7427	7
19	1961.8943	981.4508	**1944.8678**	972.9375	1943.8837	972.4455	L	**745.4203**	373.2138	728.3937	364.7005	727.4097	364.2085	6
20	2062.9420	1031.9746	2045.9154	1023.4614	2044.9314	1022.9693	T	**632.3362**	316.6717	615.3097	308.1585	614.3257	307.6665	5
21	2176.9849	1088.9961	2159.9584	1080.4828	2158.9743	1079.9908	N	**531.2885**	266.1479	514.2620	257.6346	513.2780	257.1426	4
22	2306.0275	1153.5174	2289.0010	**1145.0041**	2288.0169	**1144.5121**	E	**417.2456**	209.1264	400.2191	200.6132	399.2350	200.1212	3
23	2419.1116	1210.0594	2402.0850	1201.5461	2401.1010	1201.0541	L	288.2030	144.6051	271.1765	136.0919			2
24							R	175.1190	88.0631	158.0924	79.5498			1

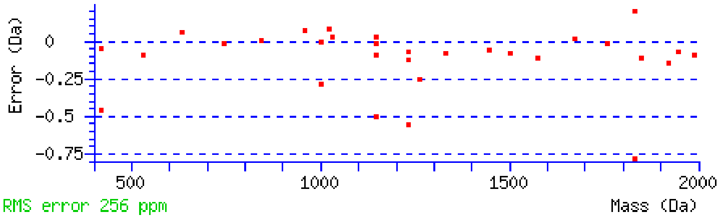

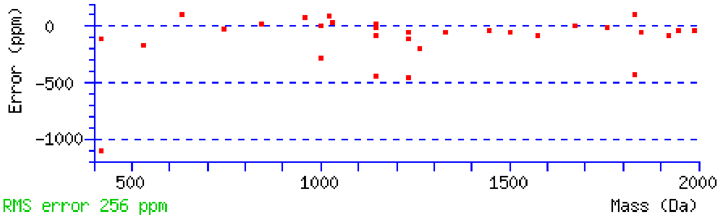

All matches to this query

Score	Mr(calc):	Delta	Sequence
131.8	2592.2159	0.0150	LNEDMACSVAGITSDANVLTNELR
9.8	2592.2561	-0.0252	GAVTGSVEKTKSVVSGSINTVLGSR
7.5	2590.2388	1.9922	NADLTVHRLGALSLGDGGTTATNEK
4.8	2590.2388	1.9922	NADLTVHRLGALSLGDGGTTATNEK
2.6	2591.2172	1.0137	SKPDSLEVLLTNLNPGTTYEIK
2.0	2590.2136	2.0173	SQDPPALARSTPGSNSSRGEEIVR
2.0	2592.2226	0.0084	HANRANKCIFIFVSILTWSR
2.0	2592.2226	0.0084	HANRANKCIFIFVSILTWSR

Spectrum No: 57; Query: 65926; Rank: 1

Peptide View

MS/MS Fragmentation of **LSAEKVEIATLTR**
Found in **IPI00299155**, Tax_Id=9606 Gene_Symbol=PSMA4 Proteasome subunit alpha type-4

Match to Query 65926: 1429.816722 from(477.612850,3+)
Title: 090702LimSK_Exosome2_03.8513.8513.3.dta
Data file C:\Temp\Mascoti\LSK_2D\090702LimSK_Exosome2_01_2D.mgf

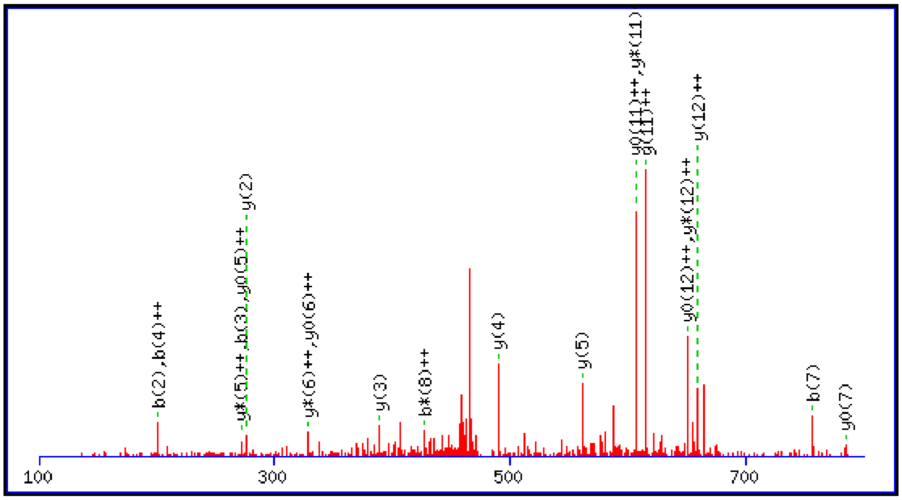

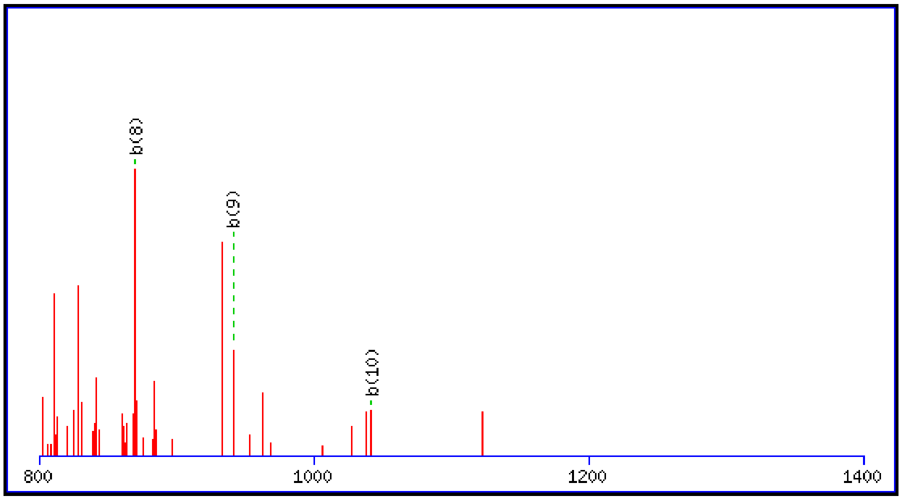

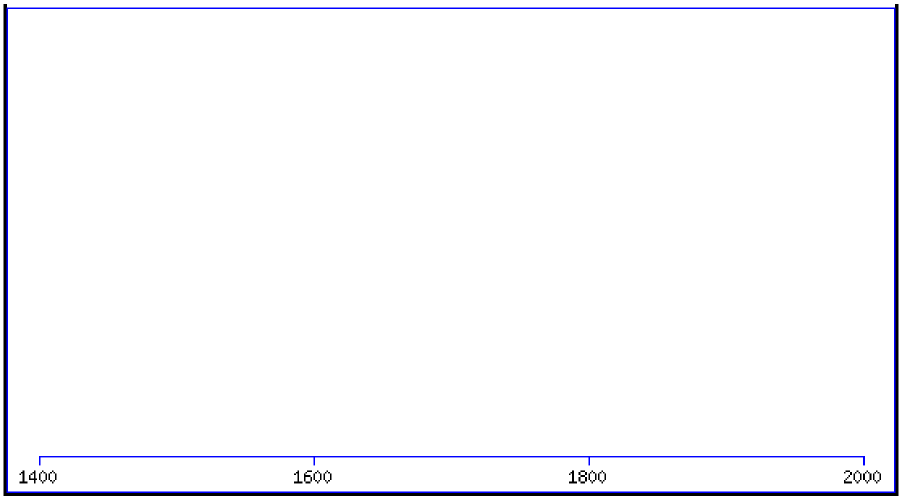

Monoisotopic mass of neutral peptide Mr(calc): 1429.8140
Fixed modifications: Carbamidomethyl (C)
Ions Score: 29 **Expect:** 0.092
Matches (Bold Red): 23/132 fragment ions using 31 most intense peaks

#	b	b^{++}	b*	b^{*++}	b^0	b^{0++}	Seq.	y	y^{++}	y*	y^{*++}	y^0	y^{0++}	#
1	114.0913	57.5493					L							13
2	**201.1234**	101.0653			183.1128	92.0600	S	1317.7373	**659.3723**	1300.7107	**650.8590**	1299.7267	**650.3670**	12
3	**272.1605**	136.5839			254.1499	127.5786	A	1230.7052	**615.8563**	1213.6787	**607.3430**	1212.6947	**606.8510**	11
4	401.2031	**201.1052**			383.1925	192.0999	E	1159.6681	580.3377	1142.6416	571.8244	1141.6575	571.3324	10
5	529.2980	265.1527	512.2715	256.6394	511.2875	256.1474	K	1030.6255	515.8164	1013.5990	507.3031	1012.6150	506.8111	9
6	628.3665	314.6869	611.3399	306.1736	610.3559	305.6816	V	902.5306	451.7689	885.5040	443.2556	884.5200	442.7636	8
7	**757.4090**	379.2082	740.3825	370.6949	739.3985	370.2029	E	803.4621	402.2347	786.4356	393.7214	**785.4516**	393.2294	7
8	**870.4931**	435.7502	853.4666	**427.2369**	852.4825	426.7449	I	674.4196	337.7134	657.3930	**329.2001**	656.4090	**328.7081**	6
9	**941.5302**	471.2687	924.5037	462.7555	923.5197	462.2635	A	**561.3355**	281.1714	544.3089	**272.6581**	543.3249	**272.1661**	5
10	**1042.5779**	521.7926	1025.5514	513.2793	1024.5673	512.7873	T	**490.2984**	245.6528	473.2718	237.1396	472.2878	236.6475	4
11	1155.6620	578.3346	1138.6354	569.8213	1137.6514	569.3293	L	**389.2507**	195.1290	372.2241	186.6157	371.2401	186.1237	3
12	1256.7096	628.8585	1239.6831	620.3452	1238.6991	619.8532	T	**276.1666**	138.5870	259.1401	130.0737	258.1561	129.5817	2
13							R	175.1190	88.0631	158.0924	79.5498			1

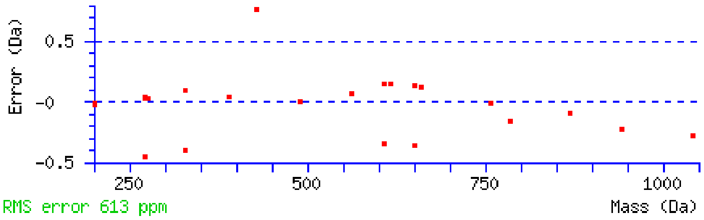

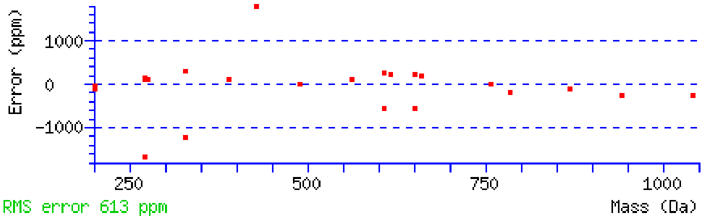

All matches to this query

Score	Mr(calc):	Delta	Sequence
28.6	1429.8140	0.0027	LSAEKVEIATLTR
4.0	1427.8037	2.0130	KALWAHLFSSLR
1.7	1427.7983	2.0184	TIAEQLAEKINAK
0.3	1427.8096	2.0071	TDQRQQLLVSLK

Spectrum No: 58; Query: 42738; Rank: 1

Peptide View

MS/MS Fragmentation of **QAYTQFGGK**
Found in **IPI00299155**, Tax_Id=9606 Gene_Symbol=PSMA4 Proteasome subunit alpha type-4

Match to Query 42738: 998.483908 from(500.249230,2+)
Title: 090702LimSK_Exosome2_03.1980.1980.2.dta
Data file C:\Temp\Mascoti\LSK_2D\090702LimSK_Exosome2_01_2D.mgf

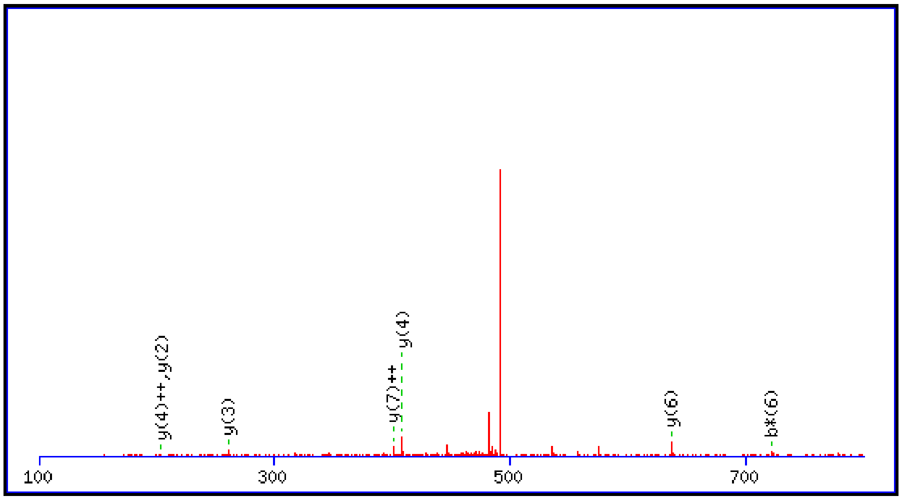

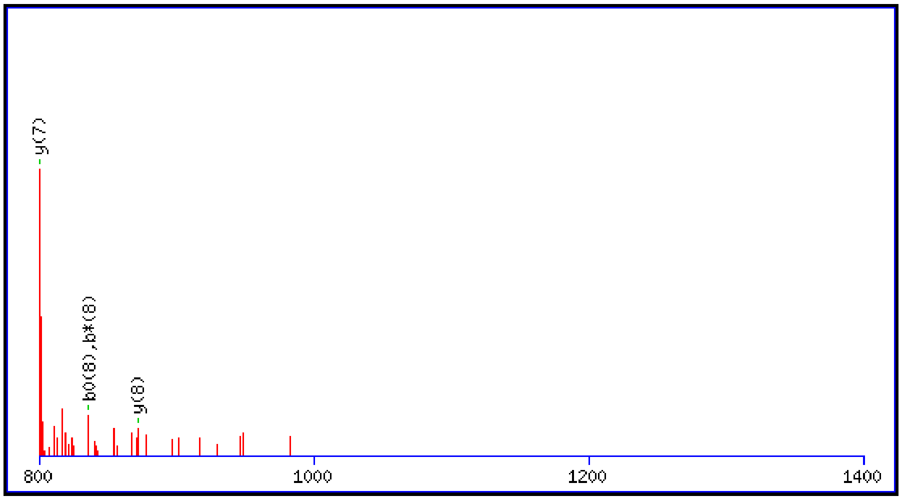

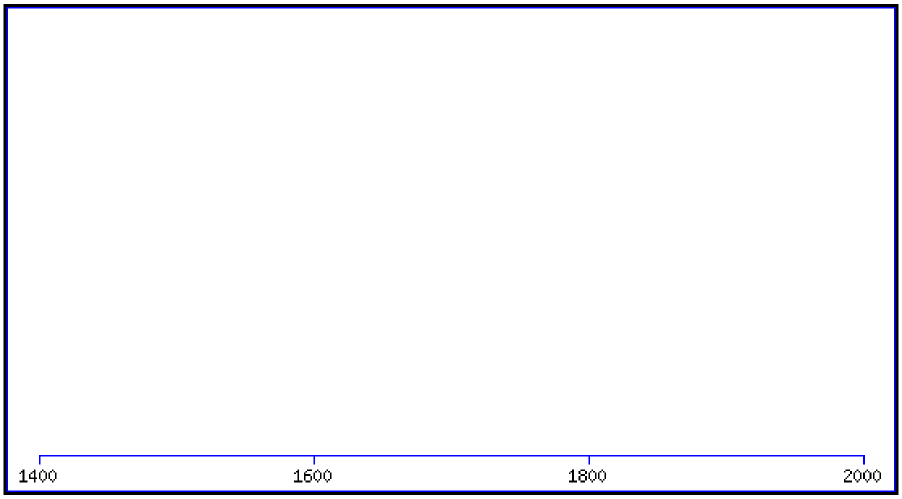

Monoisotopic mass of neutral peptide Mr(calc): 998.4821
Fixed modifications: Carbamidomethyl (C)
Ions Score: 56 **Expect:** 0.00031
Matches (Bold Red): 11/80 fragment ions using 19 most intense peaks

#	b	b++	b*	b*++	b0	b0++	Seq.	y	y++	y*	y*++	y0	y0++	#
1	129.0659	65.0366	112.0393	56.5233			Q							9

2	200.1030	100.5551	183.0764	92.0418			A	**871.4308**	436.2191	854.4043	427.7058	853.4203	427.2138	8
3	363.1663	182.0868	346.1397	173.5735			Y	**800.3937**	**400.7005**	783.3672	392.1872	782.3832	391.6952	7
4	464.2140	232.6106	447.1874	224.0974	446.2034	223.6053	T	**637.3304**	319.1688	620.3039	310.6556	619.3198	310.1636	6
5	592.2726	296.6399	575.2460	288.1266	574.2620	287.6346	Q	536.2827	268.6450	519.2562	260.1317			5
6	739.3410	370.1741	**722.3144**	361.6608	721.3304	361.1688	F	**408.2241**	**204.6157**	391.1976	196.1024			4
7	796.3624	398.6849	779.3359	390.1716	778.3519	389.6796	G	**261.1557**	131.0815	244.1292	122.5682			3
8	853.3839	427.1956	**836.3573**	418.6823	**835.3733**	418.1903	G	**204.1343**	102.5708	187.1077	94.0575			2
9							K	147.1128	74.0600	130.0863	65.5468			1

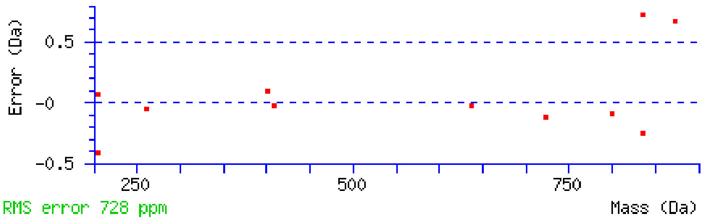

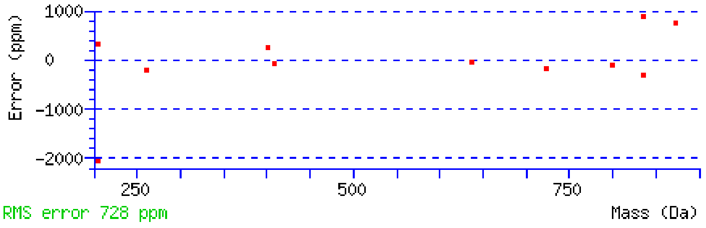

All matches to this query

Score	Mr(calc):	Delta	Sequence
55.7	998.4821	0.0018	QAYTQFGGK
30.2	998.4855	-0.0016	QAPEKQMAP
14.9	997.4902	0.9937	YVAEKMGGK
10.5	997.4845	0.9994	AQLSTSLAK
8.5	997.4845	0.9994	AKVTISDGK
8.2	997.4845	0.9994	DTKKIEGK
4.4	998.4756	0.0083	XAFGGCFGR
4.1	998.4872	-0.0032	LITLMGQK
4.1	997.4758	1.0081	MLMSKMNK
4.0	997.4845	0.9994	SVLKSAEGK

Spectrum No: 59; Query: 31569; Rank: 1

Peptide View

MS/MS Fragmentation of **SALALAIK**
Found in **IPI00299155**, Tax_Id=9606 Gene_Symbol=PSMA4 Proteasome subunit alpha type-4

Match to Query 31569: 785.501868 from(393.758210,2+)
Title: 090702LimSK_Exosome2_05.4740.4740.2.dta
Data file C:\Temp\Mascoti\LSK_2D\090702LimSK_Exosome2_01_2D.mgf

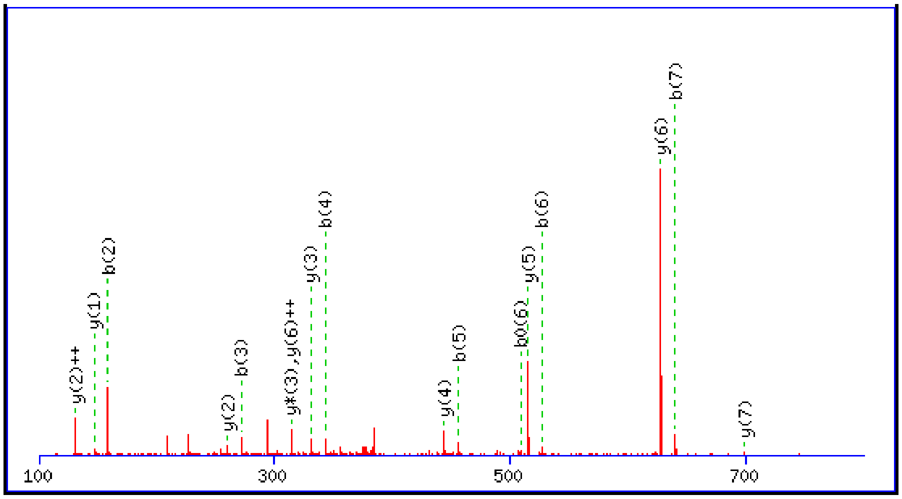

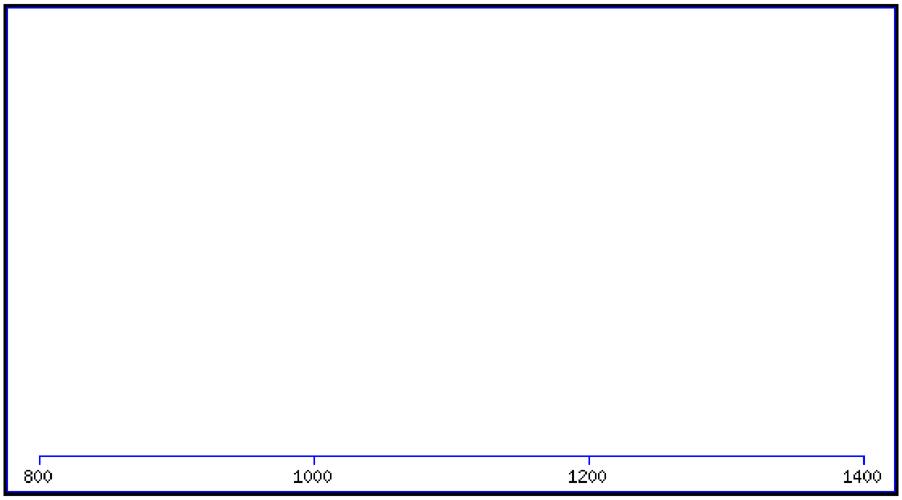

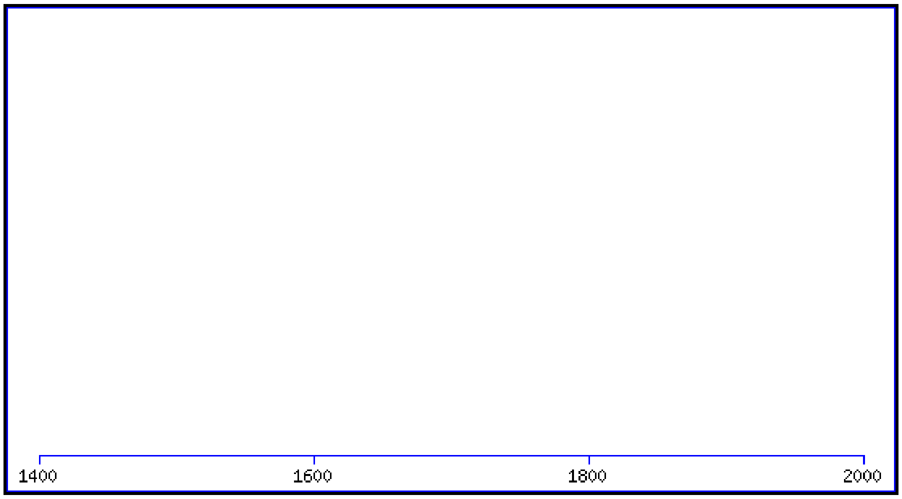

Monoisotopic mass of neutral peptide Mr(calc): 785.5011
Fixed modifications: Carbamidomethyl (C)
Ions Score: 51 **Expect:** 0.00035
Matches (Bold Red): 17/56 fragment ions using 31 most intense peaks

#	b	b^{++}	b^{0}	b^{0++}	Seq.	y	y^{++}	y*	y*$^{++}$	#
1	88.0393	44.5233	70.0287	35.5180	S					8
2	**159.0764**	80.0418	141.0659	71.0366	A	**699.4763**	350.2418	682.4498	341.7285	7
3	**272.1605**	136.5839	254.1499	127.5786	L	**628.4392**	**314.7233**	611.4127	306.2100	6
4	**343.1976**	172.1024	325.1870	163.0972	A	**515.3552**	258.1812	498.3286	249.6679	5
5	**456.2817**	228.6445	438.2711	219.6392	L	**444.3180**	222.6627	427.2915	214.1494	4
6	**527.3188**	264.1630	**509.3082**	255.1577	A	**331.2340**	166.1206	**314.2074**	157.6074	3

7	640.4028	320.7051	622.3923	311.6998	I	260.1969	130.6021	243.1703	122.0888	2
8					K	147.1128	74.0600	130.0863	65.5468	1

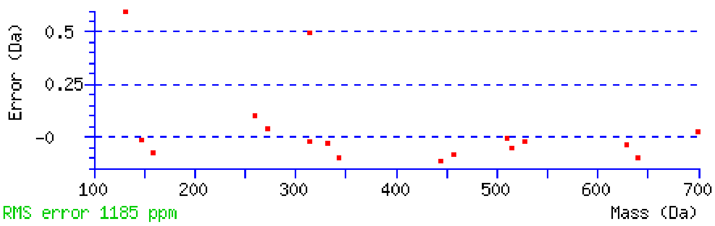

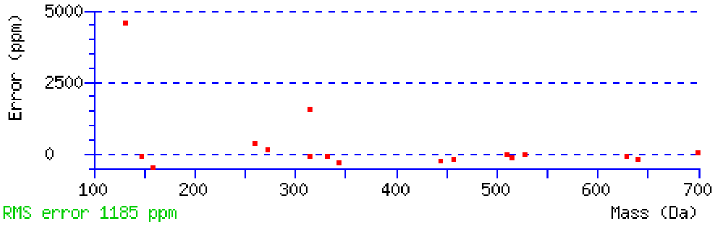

All matches to this query

Score	Mr(calc):	Delta	Sequence
51.3	785.5011	0.0008	SALALAIK
23.1	785.5011	0.0008	GLTLAALK
15.8	783.4966	2.0052	RLSPALK
12.0	785.5011	0.0008	KVDALLK
11.5	785.5011	0.0008	EVKVALK
10.1	785.5011	0.0008	TVNLVLK
9.3	785.5011	0.0008	AITAVALK
7.0	784.4919	1.0100	RLGQLAK
7.0	784.4919	1.0099	GVRVGGLK
6.4	785.5011	0.0008	TVLIVNK

Spectrum No: 60; Query: 43155; Rank: 1

Peptide View

MS/MS Fragmentation of **TTIFSPEGR**
Found in **IPI00299155**, Tax_Id=9606 Gene_Symbol=PSMA4 Proteasome subunit alpha type-4

Match to Query 43155: 1006.511888 from(504.263220,2+)
Title: 090702LimSK_Exosome2_05.3253.3253.2.dta
Data file C:\Temp\Mascoti\LSK_2D\090702LimSK_Exosome2_01_2D.mgf

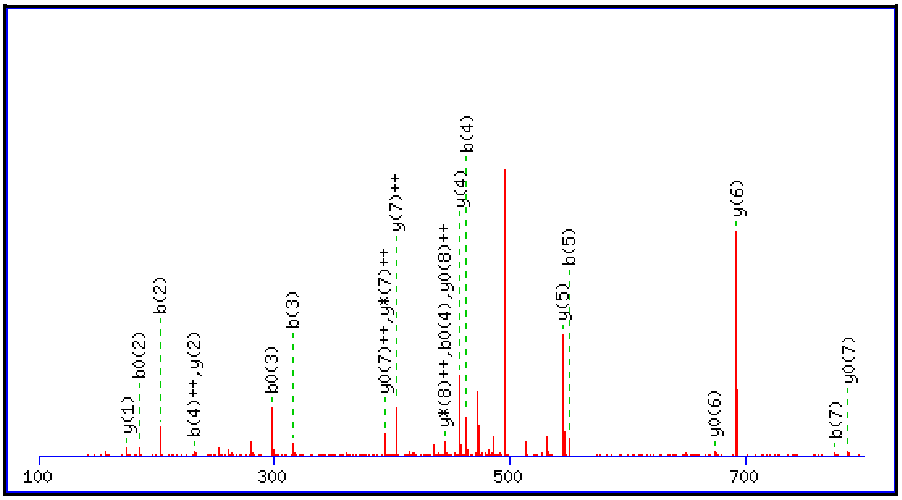

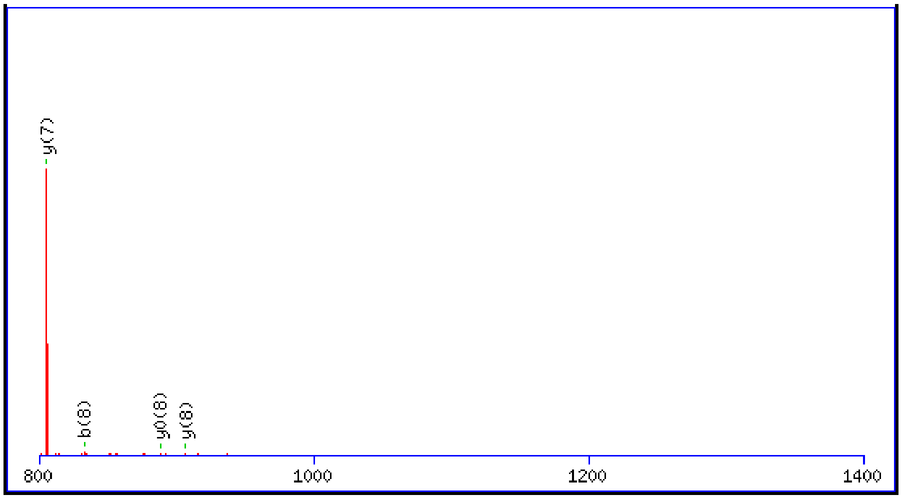

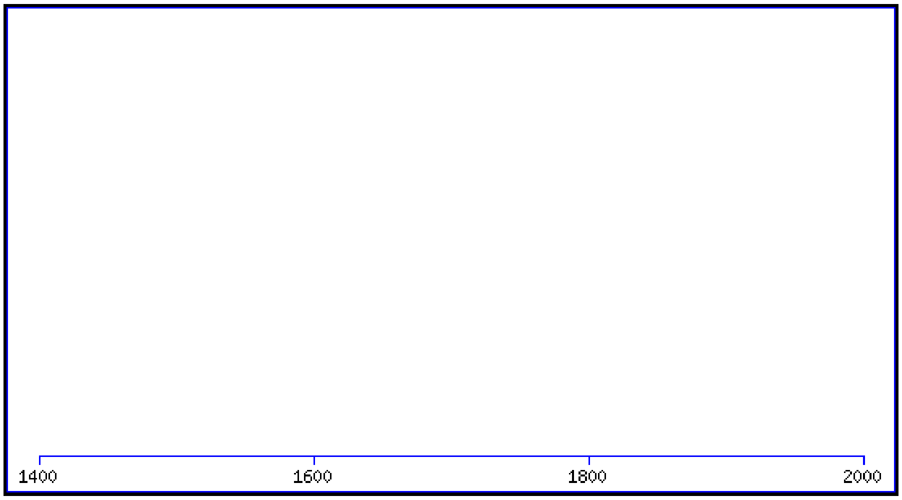

Monoisotopic mass of neutral peptide Mr(calc): 1006.5084
Fixed modifications: Carbamidomethyl (C)
Ions Score: 49 **Expect:** 0.0016
Matches (Bold Red): 25/76 fragment ions using 34 most intense peaks

#	b	b^{++}	b^{0}	b^{0++}	Seq.	y	y^{++}	y*	y^{*++}	y^{0}	y^{0++}	#
1	102.0550	51.5311	84.0444	42.5258	T							9
2	**203.1026**	102.0550	**185.0921**	93.0497	T	**906.4680**	453.7376	889.4414	**445.2243**	**888.4574**	**444.7323**	8
3	**316.1867**	158.5970	**298.1761**	149.5917	I	**805.4203**	**403.2138**	788.3937	**394.7005**	**787.4097**	**394.2085**	7
4	**463.2551**	**232.1312**	**445.2445**	223.1259	F	**692.3362**	346.6717	675.3097	338.1585	**674.3257**	337.6665	6
5	**550.2871**	275.6472	532.2766	266.6419	S	**545.2678**	273.1375	528.2413	264.6243	527.2572	264.1323	5
6	647.3399	324.1736	629.3293	315.1683	P	**458.2358**	229.6215	441.2092	221.1083	440.2252	220.6162	4
7	**776.3825**	388.6949	758.3719	379.6896	E	361.1830	181.0951	344.1565	172.5819	343.1724	172.0899	3
8	**833.4040**	417.2056	815.3934	408.2003	G	**232.1404**	116.5738	215.1139	108.0606			2
9					R	**175.1190**	88.0631	158.0924	79.5498			1

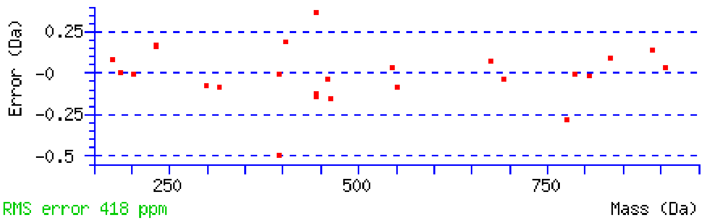

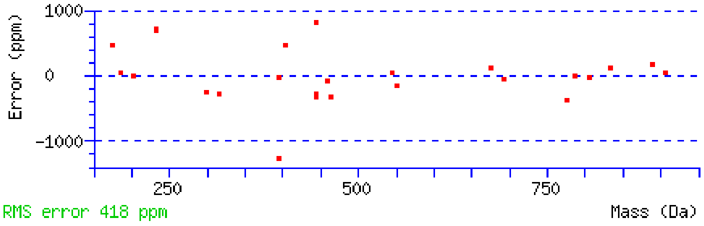

All matches to this query

Score	Mr(calc):	Delta	Sequence

49.4	1006.5084	0.0035	TTIFSPEGR
17.5	1004.4960	2.0159	EALERMEK
13.2	1006.5157	-0.0039	MAYVAVAVSP
13.1	1006.5083	0.0036	ADEAYLIGR
10.8	1004.4960	2.0158	SILQEMER
9.8	1004.5039	2.0080	AQNPGVYTR
8.1	1004.5152	1.9967	LSIGHHGER
7.5	1004.4961	2.0158	VTVVDADMR
6.8	1004.5113	2.0006	ELASMLWR
6.7	1004.5147	1.9972	SLLCMPIR

Spectrum No: 61; Query: 39024; Rank: 1

Peptide View

MS/MS Fragmentation of **VEIATLTR**
Found in **IPI00299155**, Tax_Id=9606 Gene_Symbol=PSMA4 Proteasome subunit alpha type-4

Match to Query 39024: 901.524588 from(451.769570,2+)
Title: 090702LimSK_Exosome2_02.2933.2933.2.dta
Data file C:\Temp\Mascoti\LSK_2D\090702LimSK_Exosome2_01_2D.mgf

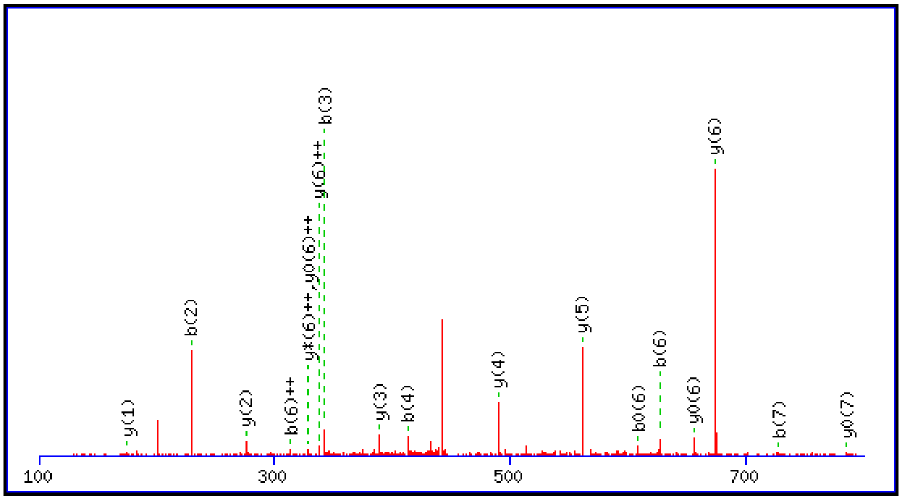

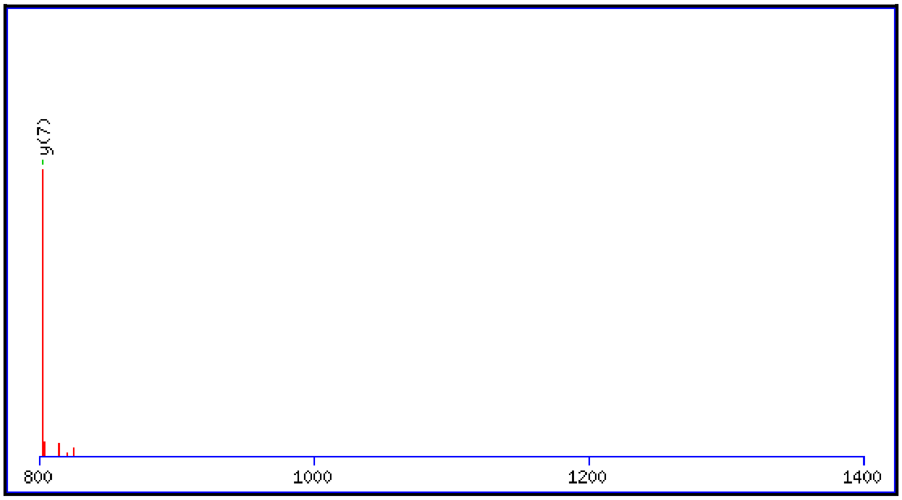

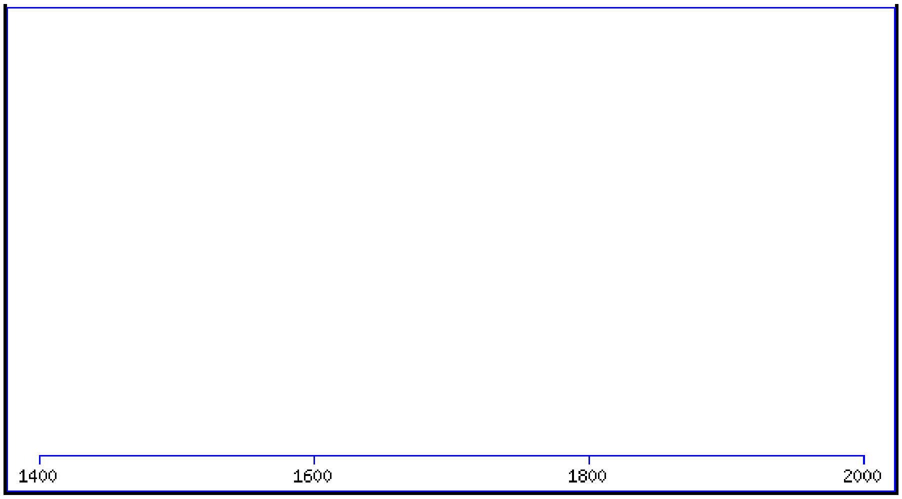

Monoisotopic mass of neutral peptide Mr(calc): 901.5233
Fixed modifications: Carbamidomethyl (C)
Ions Score: 56 **Expect:** 0.00026
Matches (Bold Red): 19/66 fragment ions using 28 most intense peaks

#	b	b++	b0	b0++	Seq.	y	y++	y*	y*++	y0	y0++	#
1	100.0757	50.5415			V							8
2	**229.1183**	115.0628	211.1077	106.0575	E	**803.4621**	402.2347	786.4356	393.7214	**785.4516**	393.2294	7
3	**342.2023**	171.6048	324.1918	162.5995	I	**674.4196**	**337.7134**	657.3930	**329.2001**	**656.4090**	**328.7081**	6
4	**413.2395**	207.1234	395.2289	198.1181	A	**561.3355**	281.1714	544.3089	272.6581	543.3249	272.1661	5
5	514.2871	257.6472	496.2766	248.6419	T	**490.2984**	245.6528	473.2718	237.1396	472.2878	236.6475	4
6	**627.3712**	**314.1892**	**609.3606**	305.1840	L	**389.2507**	195.1290	372.2241	186.6157	371.2401	186.1237	3
7	**728.4189**	364.7131	710.4083	355.7078	T	**276.1666**	138.5870	259.1401	130.0737	258.1561	129.5817	2
8					R	**175.1190**	88.0631	158.0924	79.5498			1

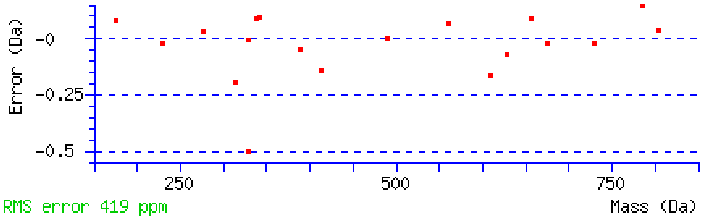

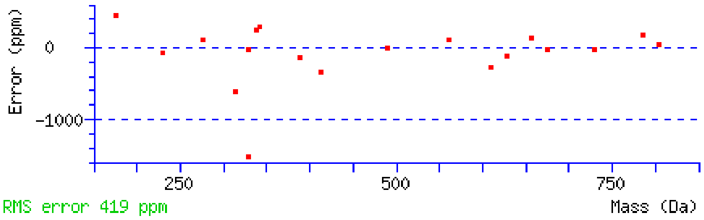

All matches to this query

Score	Mr(calc):	Delta	Sequence
56.4	901.5233	0.0013	VEIATLTR
26.9	901.5233	0.0013	VTLVDVTR
19.3	900.5280	0.9966	GAVIATELK
15.4	901.5232	0.0013	AEKKVAEK
13.1	901.5233	0.0013	LDLSAKQK
10.7	901.5233	0.0013	DLVISISR
10.7	900.5280	0.9966	IISAELQK
9.8	900.5181	1.0065	KVLWEAR
9.3	900.5280	0.9966	ALESILQK
9.3	901.5233	0.0013	GLTGIKGEK

Spectrum No: 62; Query: 93566; Rank: 1

Peptide View

MS/MS Fragmentation of **YLLQYQEPIPCEQLVTALCDIK**
Found in **IPI00299155**, Tax_Id=9606 Gene_Symbol=PSMA4 Proteasome subunit alpha type-4

Match to Query 93566: 2693.349132 from(898.790320,3+)
Title: 090702LimSK_Exosome2_03.8909.8909.3.dta
Data file C:\Temp\Mascoti\LSK_2D\090702LimSK_Exosome2_01_2D.mgf

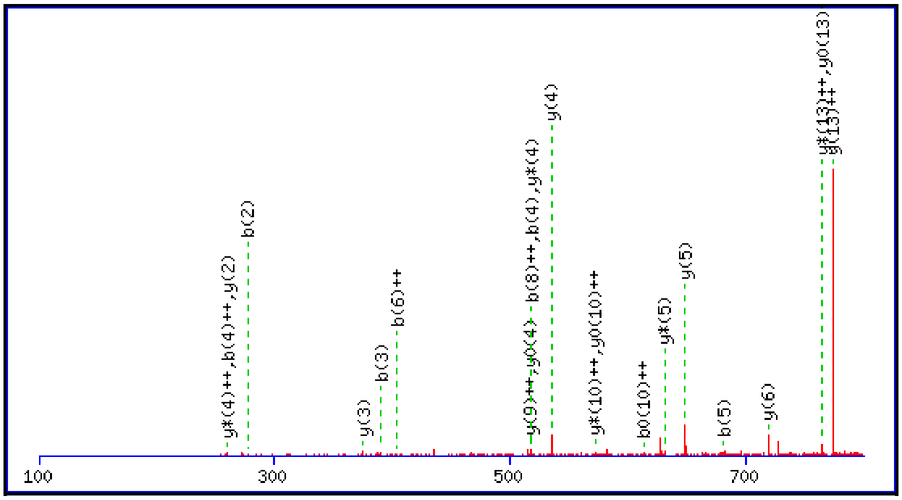

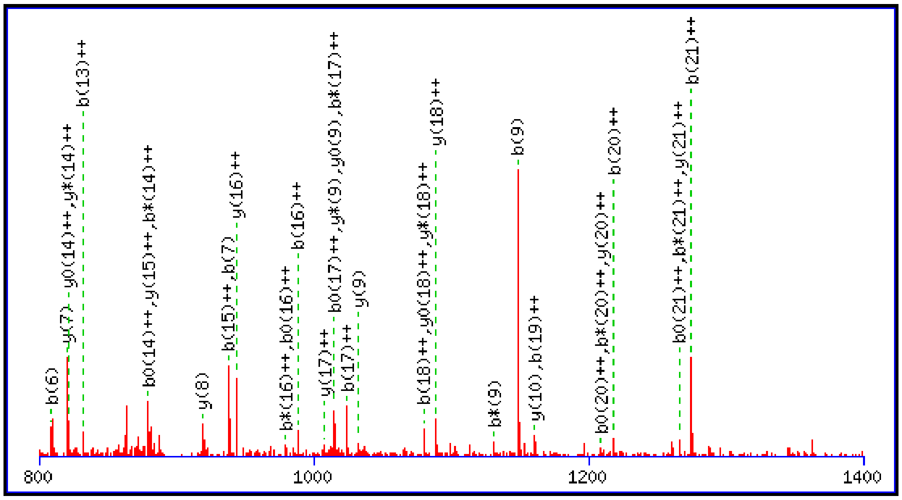

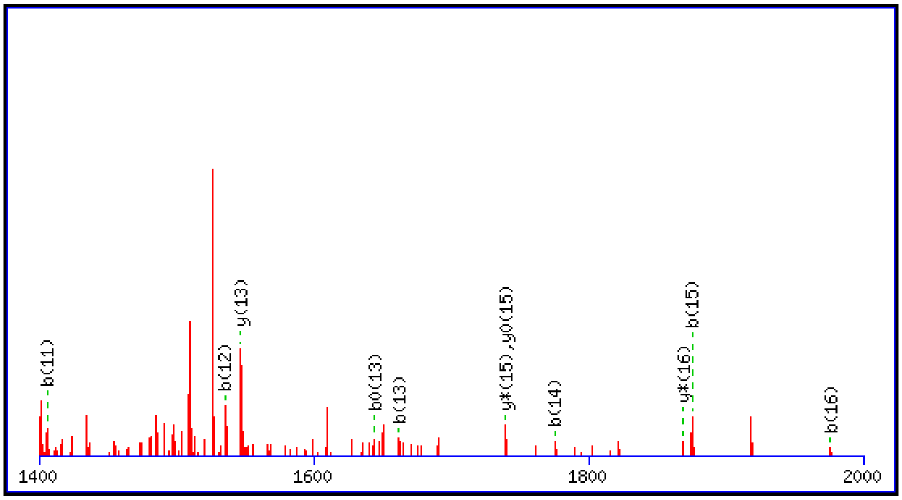

Monoisotopic mass of neutral peptide Mr(calc): 2693.3444
Fixed modifications: Carbamidomethyl (C)
Ions Score: 58 **Expect:** 0.0003
Matches (Bold Red): 72/230 fragment ions using 144 most intense peaks

		++		++	0	0++			++		++	0	0++	

#	b	b	b*	b*	b	b	Seq.	y	y	y*	y*	y	y	#
1	164.0706	82.5389					Y							22
2	**277.1547**	139.0810					L	2531.2884	**1266.1478**	2514.2619	1257.6346	2513.2778	1257.1426	21
3	**390.2387**	195.6230					L	2418.2043	**1209.6058**	2401.1778	1201.0925	2400.1938	1200.6005	20
4	**518.2973**	**259.6523**	501.2708	251.1390			Q	2305.1203	1153.0638	2288.0937	1144.5505	2287.1097	1144.0585	19
5	**681.3606**	341.1840	664.3341	332.6707			Y	2177.0617	**1089.0345**	2160.0352	**1080.5212**	2159.0511	**1080.0292**	18
6	**809.4192**	**405.2132**	792.3927	396.7000			Q	2013.9984	**1007.5028**	1996.9718	998.9896	1995.9878	998.4975	17
7	**938.4618**	469.7345	921.4353	461.2213	920.4512	460.7293	E	1885.9398	**943.4735**	**1868.9133**	934.9603	1867.9292	934.4683	16
8	1035.5146	**518.2609**	1018.4880	509.7477	1017.5040	509.2556	P	1756.8972	**878.9522**	**1739.8707**	870.4390	**1738.8866**	869.9470	15
9	**1148.5986**	574.8030	**1131.5721**	566.2897	1130.5881	565.7977	I	1659.8444	830.4259	1642.8179	**821.9126**	1641.8339	**821.4206**	14
10	1245.6514	623.3293	1228.6249	614.8161	1227.6408	**614.3241**	P	**1546.7604**	**773.8838**	1529.7338	**765.3706**	1528.7498	**764.8785**	13
11	**1405.6821**	703.3447	1388.6555	694.8314	1387.6715	694.3394	C	1449.7076	725.3574	1432.6811	716.8442	1431.6971	716.3522	12
12	**1534.7246**	767.8660	1517.6981	759.3527	1516.7141	758.8607	E	1289.6770	645.3421	1272.6504	636.8288	1271.6664	636.3368	11
13	**1662.7832**	**831.8952**	1645.7567	823.3820	**1644.7727**	822.8900	Q	**1160.6344**	580.8208	1143.6078	**572.3076**	1142.6238	**571.8155**	10
14	**1775.8673**	888.4373	1758.8407	**879.9240**	1757.8567	**879.4320**	L	**1032.5758**	**516.7915**	**1015.5492**	508.2783	**1014.5652**	507.7863	9
15	**1874.9357**	**937.9715**	1857.9092	929.4582	1856.9251	928.9662	V	**919.4917**	460.2495	902.4652	451.7362	901.4812	451.2442	8
16	**1975.9834**	**988.4953**	1958.9568	**979.9821**	1957.9728	**979.4900**	T	**820.4233**	410.7153	803.3968	402.2020	802.4128	401.7100	7
17	2047.0205	**1024.0139**	2029.9939	**1015.5006**	2029.0099	**1015.0086**	A	**719.3756**	360.1915	702.3491	351.6782	701.3651	351.1862	6
18	2160.1046	**1080.5559**	2143.0780	1072.0426	2142.0940	1071.5506	L	**648.3385**	324.6729	**631.3120**	316.1596	630.3280	315.6676	5
19	2320.1352	**1160.5712**	2303.1087	1152.0580	2302.1246	1151.5660	C	**535.2545**	268.1309	**518.2279**	**259.6176**	**517.2439**	259.1256	4
20	2435.1621	**1218.0847**	2418.1356	**1209.5714**	2417.1516	**1209.0794**	D	**375.2238**	188.1155	358.1973	179.6023	357.2132	179.1103	3
21	2548.2462	**1274.6267**	2531.2197	**1266.1135**	2530.2356	**1265.6215**	I	**260.1969**	130.6021	243.1703	122.0888			2
22							K	147.1128	74.0600	130.0863	65.5468			1

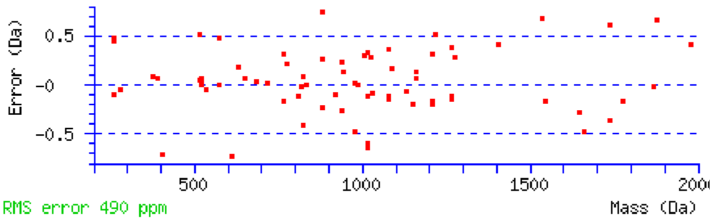

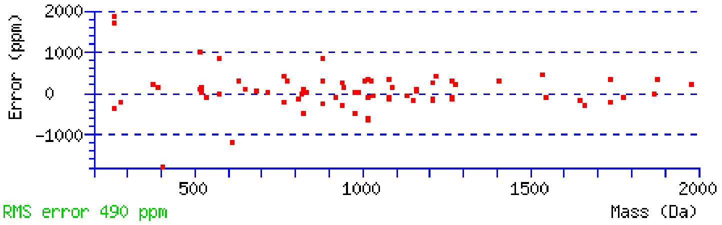

All matches to this query

Score	Mr(calc):	Delta	Sequence
58.2	2693.3444	0.0047	YLLQYQEPIPCEQLVTALCDIK
10.3	2692.3235	1.0257	VHPGQPAAPAEPAPGAPALRSGPSQPR
6.9	2692.3235	1.0257	VHPGQPAAPAEPAPGAPALRSGPSQPR
3.7	2692.3410	1.0082	SLGCFLLLHLVYIMASPFSITSK
2.6	2691.3204	2.0288	ITTHFELKHLSSGDLLRDNMLR
2.6	2691.3204	2.0288	ITTHFELKHLSSGDLLRDNMLR
2.3	2693.3533	-0.0042	TKGVTAYRLSTGLMITSVAVELCK
2.3	2693.3533	-0.0042	TKGVTAYRLSTGLMITSVAVELCK
2.3	2693.3533	-0.0042	TKGVTAYRLSTGLMITSVAVELCK
1.9	2691.3690	1.9801	RNQPDYYEVVSQPIDLMKIQQK

Spectrum No: 63; Query: 56327; Rank: 1

Peptide View

MS/MS Fragmentation of **AVENSSTAIGIR**

Found in **IPI00171199**, Tax_Id=9606 Gene_Symbol=PSMA3 Isoform 2 of Proteasome subunit alpha type-3

Match to Query 56327: 1216.643448 from(609.329000,2+)
Title: 090702LimSK_Exosome2_03.2496.2496.2.dta
Data file C:\Temp\Mascoti\LSK_2D\090702LimSK_Exosome2_01_2D.mgf

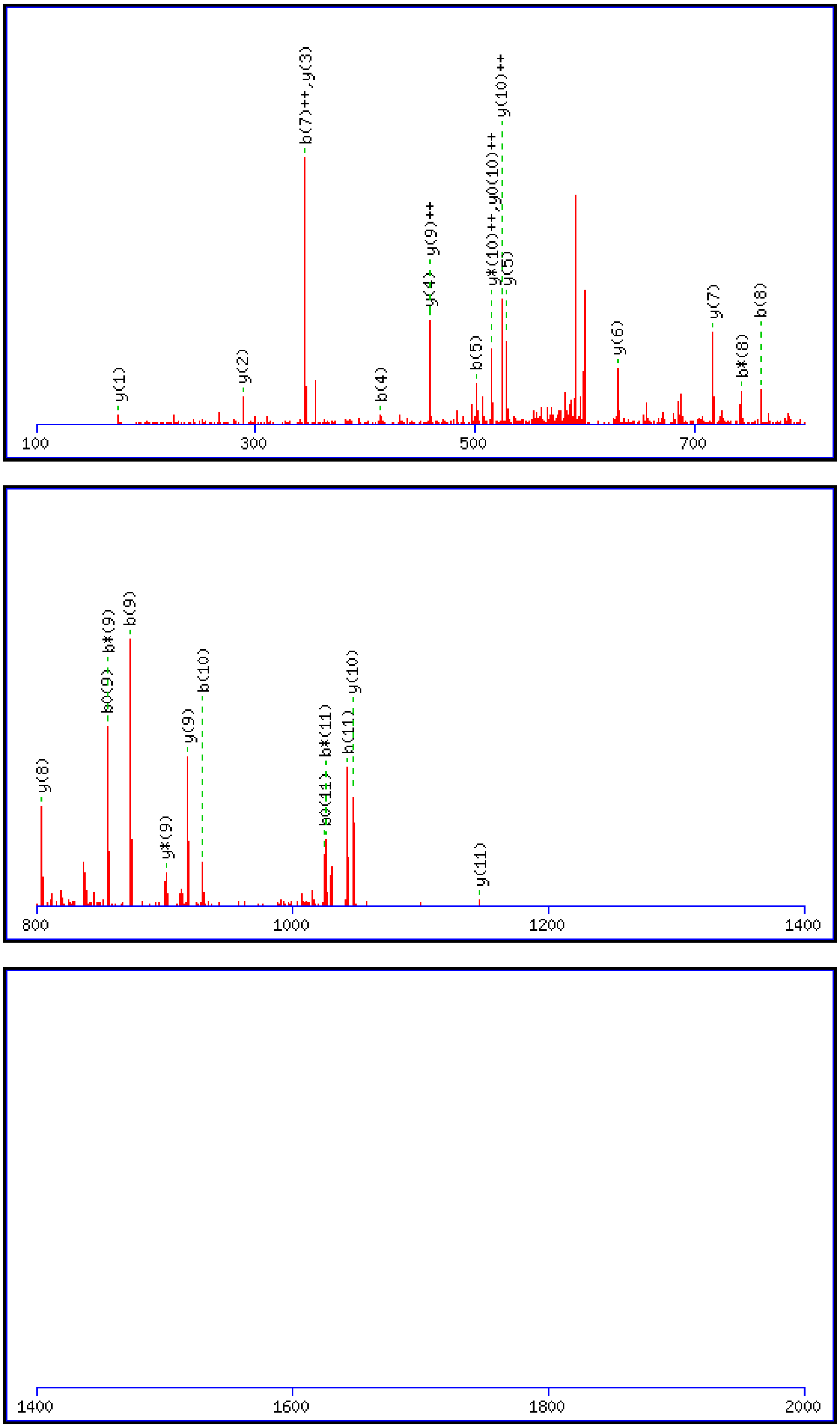

Monoisotopic mass of neutral peptide Mr(calc): 1216.6411
Fixed modifications: Carbamidomethyl (C)
Ions Score: 96 **Expect:** 3.7e-008
Matches (Bold Red): 28/112 fragment ions using 42 most intense peaks

#	b	b^{++}	b*	b^{*++}	b^{0}	b^{0++}	Seq.	y	y^{++}	y*	y^{*++}	y^{0}	y^{0++}	#
1	72.0444	36.5258					A							12
2	171.1128	86.0600					V	**1146.6113**	573.8093	1129.5848	565.2960	1128.6008	564.8040	11

**3**	300.1554	150.5813			282.1448	141.5761	**E**	**1047.5429**	**524.2751**	1030.5164	**515.7618**	1029.5323	**515.2698**	**10**
**4**	**414.1983**	207.6028	397.1718	199.0895	396.1878	198.5975	**N**	**918.5003**	**459.7538**	**901.4738**	451.2405	900.4898	450.7485	**9**
**5**	**501.2304**	251.1188	484.2038	242.6055	483.2198	242.1135	**S**	**804.4574**	402.7323	787.4308	394.2191	786.4468	393.7271	**8**
**6**	588.2624	294.6348	571.2358	286.1216	570.2518	285.6295	**S**	**717.4254**	359.2163	700.3988	350.7030	699.4148	350.2110	**7**
**7**	689.3101	**345.1587**	672.2835	336.6454	671.2995	336.1534	**T**	**630.3933**	315.7003	613.3668	307.1870	612.3828	306.6950	**6**
**8**	**760.3472**	380.6772	**743.3206**	372.1640	742.3366	371.6719	**A**	**529.3457**	265.1765	512.3191	256.6632			**5**
**9**	**873.4312**	437.2193	**856.4047**	428.7060	**855.4207**	428.2140	**I**	**458.3085**	229.6579	441.2820	221.1446			**4**
**10**	**930.4527**	465.7300	913.4262	457.2167	912.4421	456.7247	**G**	**345.2245**	173.1159	328.1979	164.6026			**3**
**11**	**1043.5368**	522.2720	**1026.5102**	513.7587	**1025.5262**	513.2667	**I**	**288.2030**	144.6051	271.1765	136.0919			**2**
**12**							**R**	**175.1190**	88.0631	158.0924	79.5498			**1**

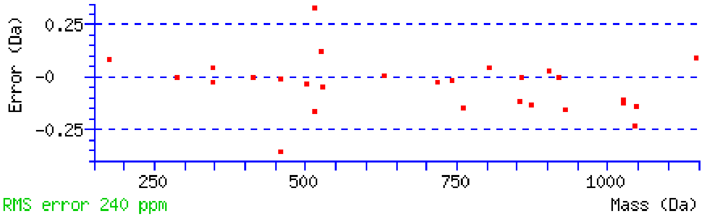

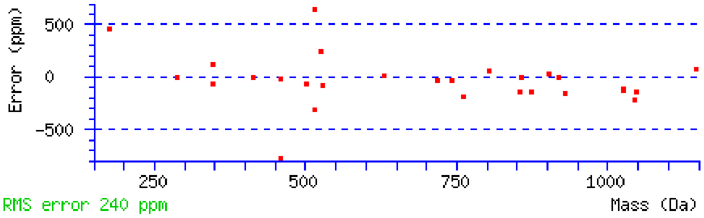

All matches to this query

Score	Mr(calc):	Delta	Sequence
96.0	1216.6411	0.0023	AVENSSTAIGIR
16.3	1216.6524	-0.0089	EPKSSSRTLGR
12.2	1215.6360	1.0074	RDWTAELGIR
11.0	1216.6346	0.0088	VAEATRMLNGR
10.3	1216.6425	0.0010	WTGRDLASRR
8.7	1216.6353	0.0082	LYWQGGPALGR
8.7	1216.6524	-0.0089	TRSTGQLELGR
8.6	1215.6489	0.9946	ARLPLPGSGLR
7.9	1215.6499	0.9935	YHLDTVKIEV
7.9	1216.6411	0.0023	ADTLNGSLSALR

Spectrum No: 64; Query: 57406; Rank: 1

Peptide View

MS/MS Fragmentation of **CKDGVVFGVEK**
Found in **IPI00171199**, Tax_Id=9606 Gene_Symbol=PSMA3 Isoform 2 of Proteasome subunit alpha type-3

Match to Query 57406: 1236.618188 from(619.316370,2+)
Title: 090702LimSK_Exosome2_06.7852.7852.2.dta
Data file C:\Temp\Mascoti\LSK_2D\090702LimSK_Exosome2_01_2D.mgf

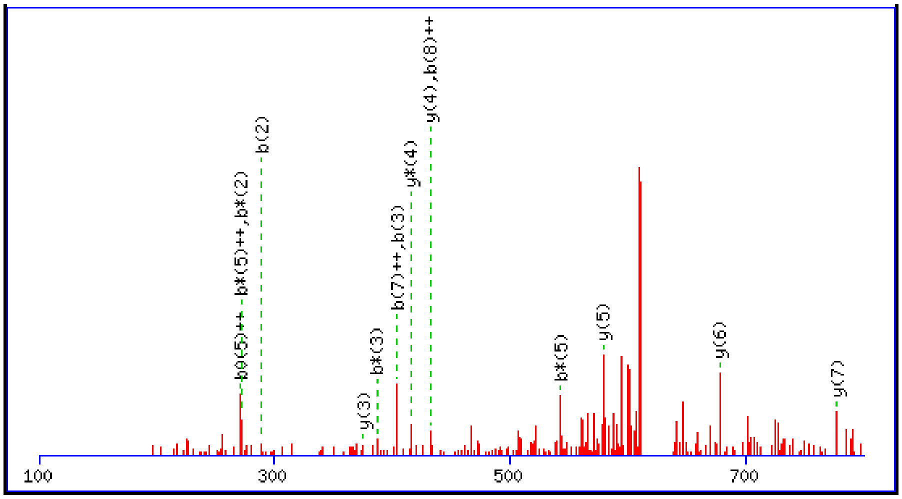

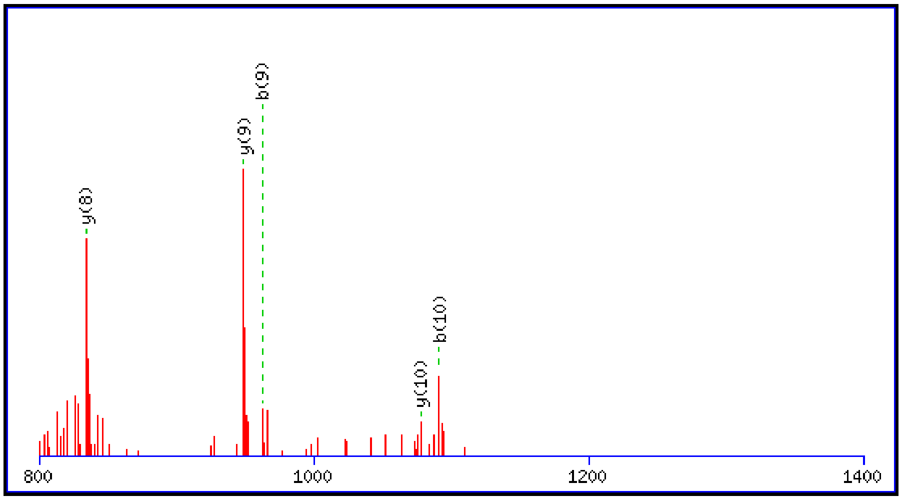

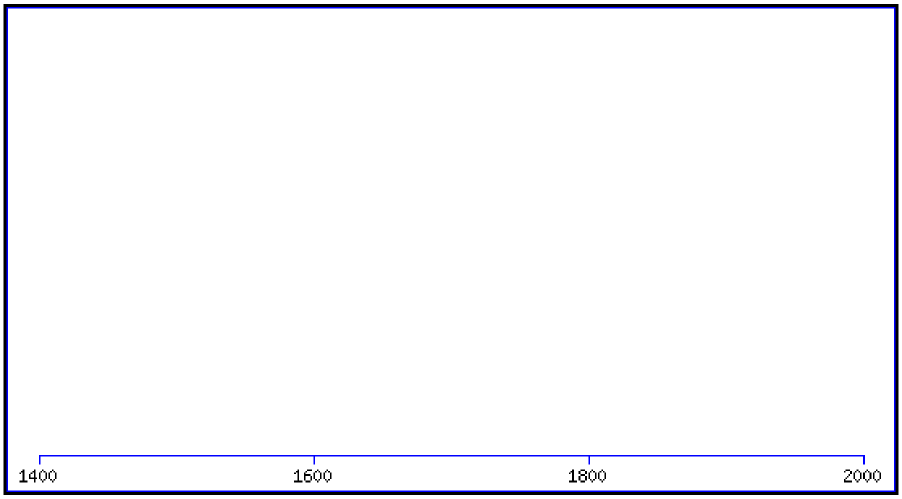

Monoisotopic mass of neutral peptide Mr(calc): 1236.6173
Fixed modifications: Carbamidomethyl (C)
Ions Score: 39 **Expect:** 0.016
Matches (Bold Red): 20/112 fragment ions using 48 most intense peaks

#	b	b^{++}	b*	b^{*++}	b^0	b^{0++}	Seq.	y	y^{++}	y*	y^{*++}	y^0	y^{0++}	#
1	161.0379	81.0226					C							11
2	**289.1329**	145.0701	**272.1063**	136.5568			K	**1077.5939**	539.3006	1060.5673	530.7873	1059.5833	530.2953	10
3	**404.1598**	202.5836	**387.1333**	194.0703	386.1493	193.5783	D	**949.4989**	475.2531	932.4724	466.7398	931.4884	466.2478	9
4	461.1813	231.0943	444.1547	222.5810	443.1707	222.0890	G	**834.4720**	417.7396	817.4454	409.2264	816.4614	408.7343	8
5	560.2497	280.6285	**543.2232**	**272.1152**	542.2391	**271.6232**	V	**777.4505**	389.2289	760.4240	380.7156	759.4400	380.2236	7
6	659.3181	330.1627	642.2916	321.6494	641.3076	321.1574	V	**678.3821**	339.6947	661.3556	331.1814	660.3715	330.6894	6

7	806.3865	**403.6969**	789.3600	395.1836	788.3760	394.6916	F	**579.3137**	290.1605	562.2871	281.6472	561.3031	281.1552	5
8	863.4080	**432.2076**	846.3815	423.6944	845.3974	423.2024	G	**432.2453**	216.6263	**415.2187**	208.1130	414.2347	207.6210	4
9	**962.4764**	481.7418	945.4499	473.2286	944.4658	472.7366	V	**375.2238**	188.1155	358.1973	179.6023	357.2132	179.1103	3
10	**1091.5190**	546.2631	1074.4925	537.7499	1073.5084	537.2579	E	276.1554	138.5813	259.1288	130.0681	258.1448	129.5761	2
11							K	147.1128	74.0600	130.0863	65.5468			1

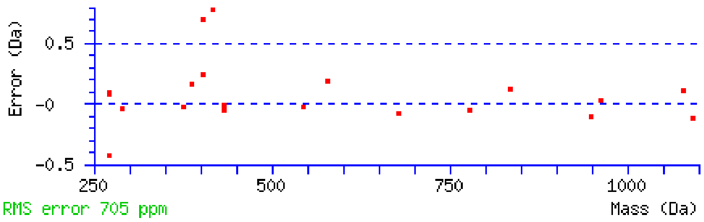

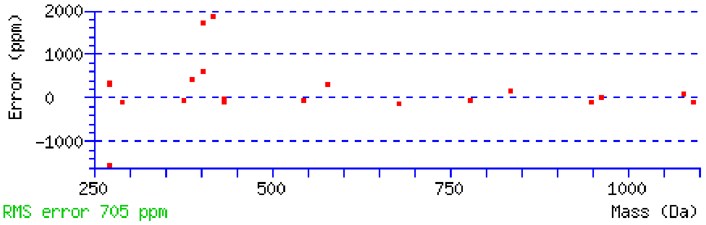

All matches to this query

Score	Mr(calc):	Delta	Sequence
39.5	1236.6173	0.0009	CKDGVVFGVEK
10.7	1236.6073	0.0108	KCNLQYHFK
8.8	1236.6285	-0.0103	CKDKYGKPNK
8.7	1236.6206	-0.0024	KGTEMKGMVEK
7.6	1236.6284	-0.0103	ILREMYERQ
6.1	1236.6227	-0.0045	KPSKNQAEKK
6.1	1236.6115	0.0067	QAVKTEPEKK
5.3	1235.6146	1.0036	SPSESFIGREK
5.3	1236.6198	-0.0016	KTDSLTSGATEK
4.6	1236.6285	-0.0103	CQYQERLIK

Spectrum No: 65; Query: 40656; Rank: 1

Peptide View

MS/MS Fragmentation of **DGVVFGVEK**
Found in **IPI00171199**, Tax_Id=9606 Gene_Symbol=PSMA3 Isoform 2 of Proteasome subunit alpha type-3

Match to Query 40656: 948.494128 from(475.254340,2+)
Title: 090702LimSK_Exosome2_06.3546.3546.2.dta
Data file C:\Temp\Mascoti\LSK_2D\090702LimSK_Exosome2_01_2D.mgf

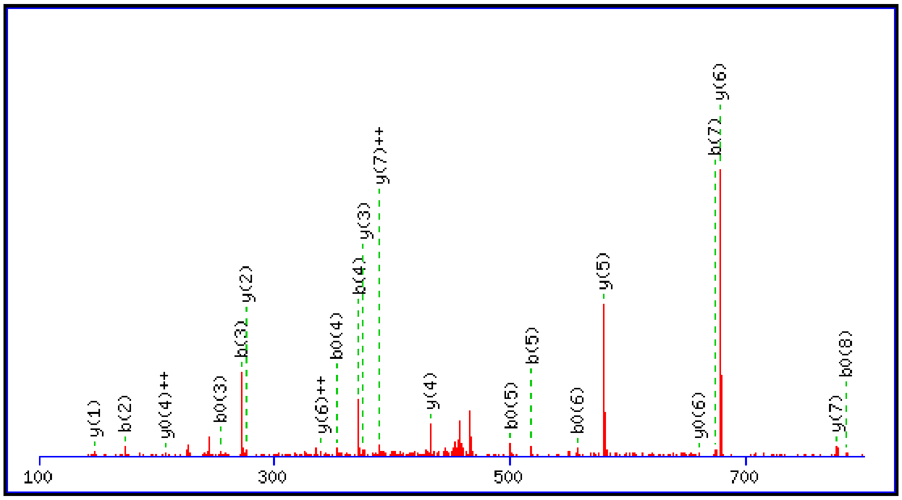

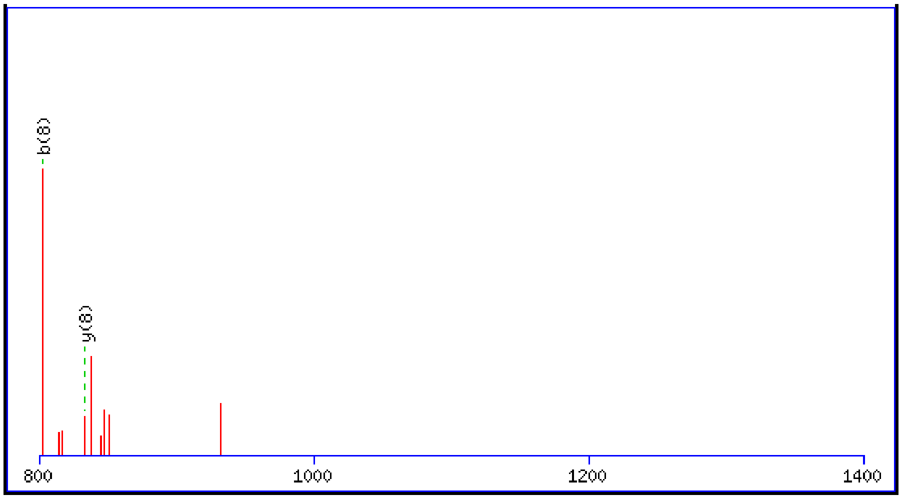

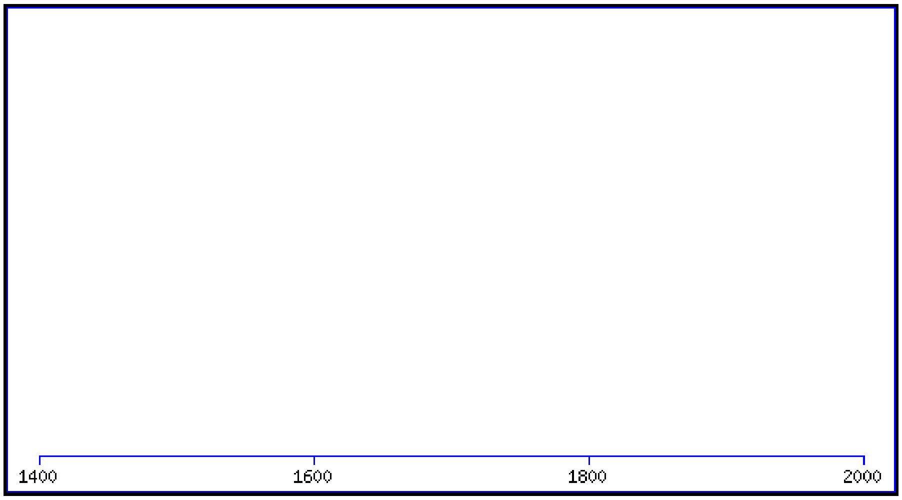

Monoisotopic mass of neutral peptide Mr(calc): 948.4917
Fixed modifications: Carbamidomethyl (C)
Ions Score: 46 **Expect:** 0.0036
Matches (Bold Red): 23/78 fragment ions using 66 most intense peaks

#	b	b^{++}	b^{0}	b^{0++}	Seq.	y	y^{++}	y*	y^{*++}	y^{0}	y^{0++}	#
1	116.0342	58.5207	98.0237	49.5155	D							9
2	**173.0557**	87.0315	155.0451	78.0262	G	**834.4720**	417.7396	817.4454	409.2264	816.4614	408.7343	8
3	**272.1241**	136.5657	**254.1135**	127.5604	V	**777.4505**	**389.2289**	760.4240	380.7156	759.4400	380.2236	7
4	**371.1925**	186.0999	**353.1819**	177.0946	V	**678.3821**	**339.6947**	661.3556	331.1814	**660.3715**	330.6894	6
5	**518.2609**	259.6341	**500.2504**	250.6288	F	**579.3137**	290.1605	562.2871	281.6472	561.3031	281.1552	5
6	575.2824	288.1448	**557.2718**	279.1396	G	**432.2453**	216.6263	415.2187	208.1130	414.2347	**207.6210**	4
7	**674.3508**	337.6790	656.3402	328.6738	V	**375.2238**	188.1155	358.1973	179.6023	357.2132	179.1103	3
8	**803.3934**	402.2003	**785.3828**	393.1951	E	**276.1554**	138.5813	259.1288	130.0681	258.1448	129.5761	2
9					K	**147.1128**	74.0600	130.0863	65.5468			1

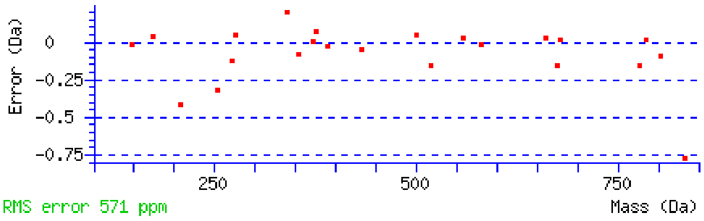

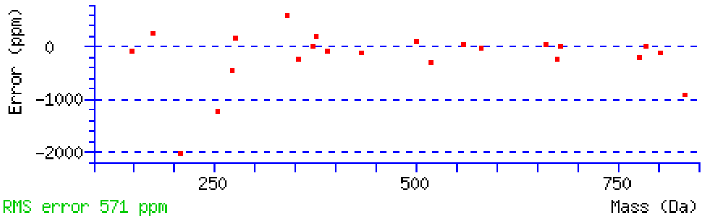

All matches to this query

Score	Mr(calc):	Delta	Sequence

45.9	948.4917	0.0025	DGVVFGVEK
12.8	948.5029	-0.0087	ESKYAVPR
12.8	948.4851	0.0090	MFVGQVPR
12.8	948.5018	-0.0077	VSRRVPR
10.6	948.4950	-0.0009	KMTSPLEK
6.2	948.5029	-0.0088	DGLFRVDK
5.6	948.5029	-0.0087	LAVNYNQK
5.5	948.4876	0.0065	SIAKNSDSK
5.1	948.4876	0.0065	SPSKGTSGTK
4.6	948.4906	0.0035	ALSRGLPR

Spectrum No: 66; Query: 42276; Rank: 1

Peptide View

MS/MS Fragmentation of **DIREEAEK**
Found in **IPI00171199**, Tax_Id=9606 Gene_Symbol=PSMA3 Isoform 2 of Proteasome subunit alpha type-3

Match to Query 42276: 988.484208 from(495.249380,2+)
Title: 090702LimSK_Exosome2_04.1308.1308.2.dta
Data file C:\Temp\Mascoti\LSK_2D\090702LimSK_Exosome2_01_2D.mgf

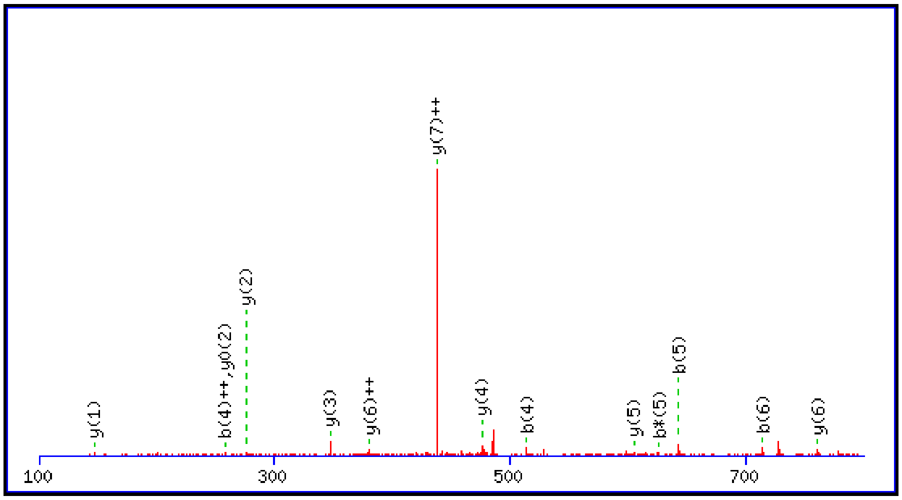

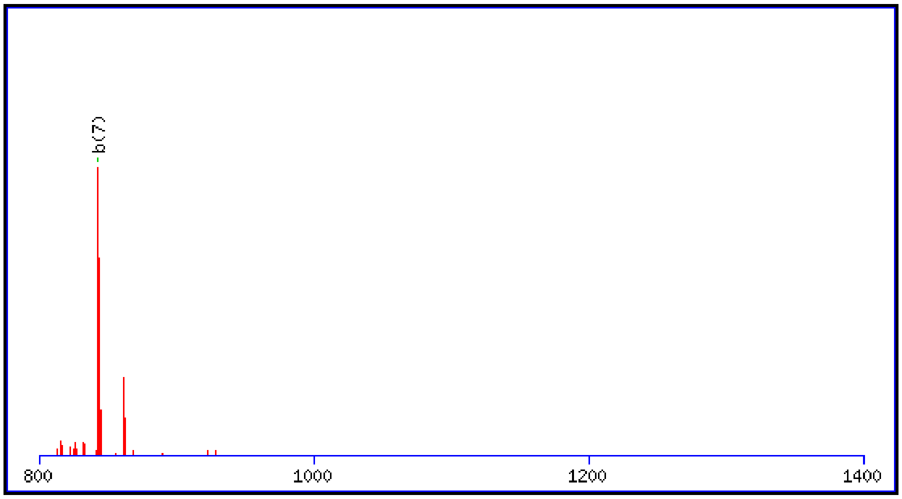

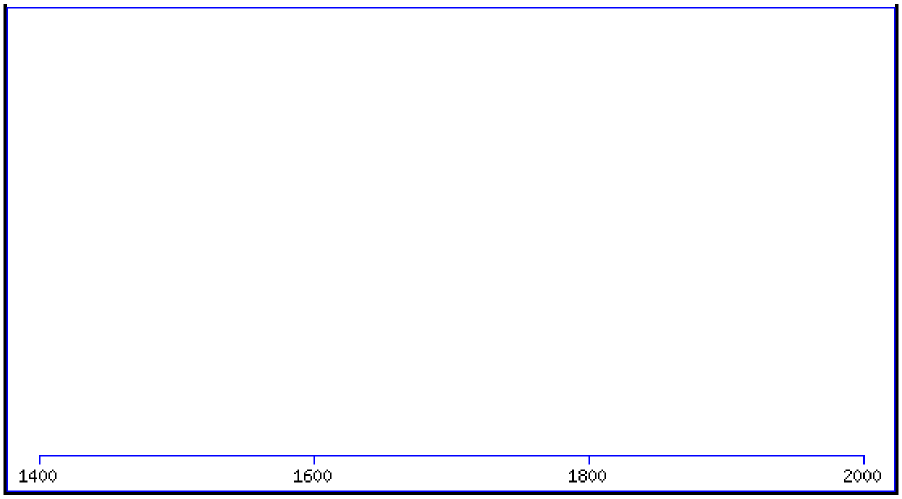

Monoisotopic mass of neutral peptide Mr(calc): 988.4825
Fixed modifications: Carbamidomethyl (C)
Ions Score: 31 **Expect:** 0.1
Matches (Bold Red): 15/78 fragment ions using 34 most intense peaks

#	b	b^{++}	b*	b^{*++}	b^0	b^{0++}	Seq.	y	y^{++}	y*	y^{*++}	y^0	y^{0++}	#
1	116.0342	58.5207			98.0237	49.5155	D							8
2	229.1183	115.0628			211.1077	106.0575	I	874.4629	**437.7351**	857.4363	429.2218	856.4523	428.7298	7
3	385.2194	193.1133	368.1928	184.6001	367.2088	184.1081	R	**761.3788**	**381.1930**	744.3523	372.6798	743.3682	372.1878	6
4	**514.2620**	**257.6346**	497.2354	249.1214	496.2514	248.6293	E	**605.2777**	303.1425	588.2511	294.6292	587.2671	294.1372	5
5	**643.3046**	322.1559	**626.2780**	313.6427	625.2940	313.1506	E	**476.2351**	238.6212	459.2086	230.1079	458.2245	229.6159	4
6	**714.3417**	357.6745	697.3151	349.1612	696.3311	348.6692	A	**347.1925**	174.0999	330.1660	165.5866	329.1819	165.0946	3
7	**843.3843**	422.1958	826.3577	413.6825	825.3737	413.1905	E	**276.1554**	138.5813	259.1288	130.0681	**258.1448**	129.5761	2
8							K	**147.1128**	74.0600	130.0863	65.5468			1

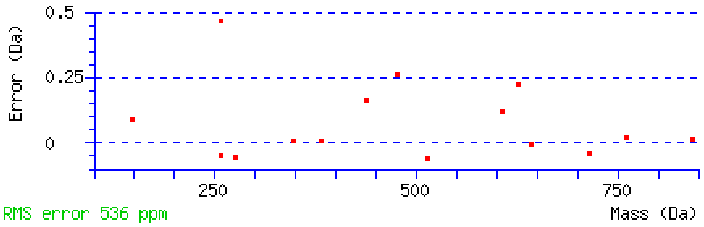

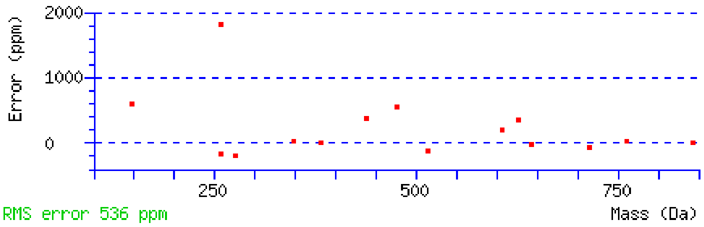

All matches to this query

Score	Mr(calc):	Delta	Sequence
30.9	988.4825	0.0017	DIREEAEK
27.7	988.4825	0.0017	QVAQSEAEK
12.5	988.4825	0.0017	QNDLKDEK
12.4	988.4825	0.0017	SLQEANAEK
12.3	987.4864	0.9978	LFIGMISK
10.8	988.4825	0.0017	QNVADSVEK
10.6	987.4790	1.0052	IIYTRDK
10.6	988.4825	0.0017	LEGVDEATR
9.5	986.4821	2.0021	HAFENLEK
9.3	986.4725	2.0117	LLLEYEK

Spectrum No: 67; Query: 39032; Rank: 1

Peptide View

MS/MS Fragmentation of **GAVIATELK**
Found in **IPI00006181**, Tax_Id=9606 Gene_Symbol=EIF3D Eukaryotic translation initiation factor 3 subunit D

Match to Query 39032: 901.525008 from(451.769780,2+)
Title: 090702LimSK_Exosome2_04.3602.3602.2.dta
Data file C:\Temp\Mascoti\LSK_2D\090702LimSK_Exosome2_01_2D.mgf

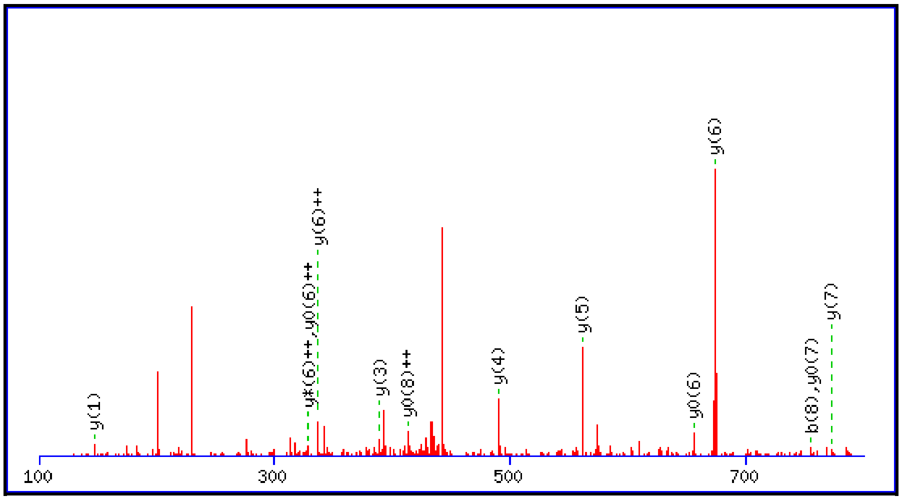

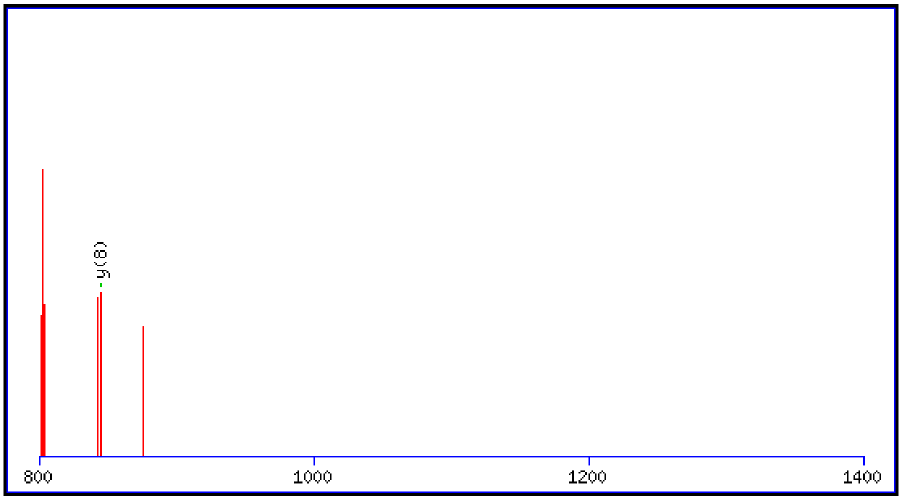

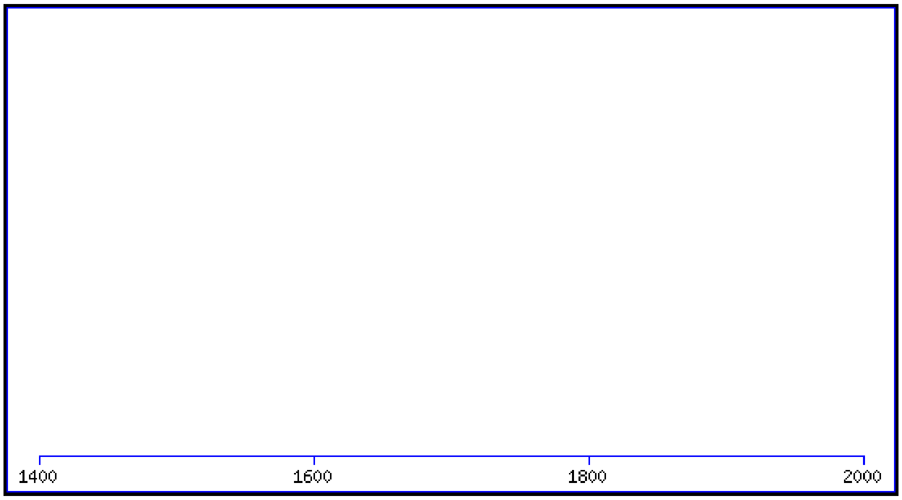

Monoisotopic mass of neutral peptide Mr(calc): 900.5280
Fixed modifications: Carbamidomethyl (C)
Ions Score: 29 **Expect:** 0.14
Matches (Bold Red): 14/66 fragment ions using 48 most intense peaks

		++	0	0++			++		++	0	0++	

#	b	b	b	b	Seq.	y	y	y*	y*	y	y	#
1	58.0287	29.5180			G							9
2	129.0659	65.0366			A	**844.5138**	422.7606	827.4873	414.2473	826.5033	**413.7553**	8
3	228.1343	114.5708			V	**773.4767**	387.2420	756.4502	378.7287	**755.4662**	378.2367	7
4	341.2183	171.1128			I	**674.4083**	**337.7078**	657.3818	**329.1945**	**656.3978**	**328.7025**	6
5	412.2554	206.6314			A	**561.3243**	281.1658	544.2977	272.6525	543.3137	272.1605	5
6	513.3031	257.1552	495.2926	248.1499	T	**490.2871**	245.6472	473.2606	237.1339	472.2766	236.6419	4
7	642.3457	321.6765	624.3352	312.6712	E	**389.2395**	195.1234	372.2129	186.6101	371.2289	186.1181	3
8	**755.4298**	378.2185	737.4192	369.2132	L	260.1969	130.6021	243.1703	122.0888			2
9					K	**147.1128**	74.0600	130.0863	65.5468			1

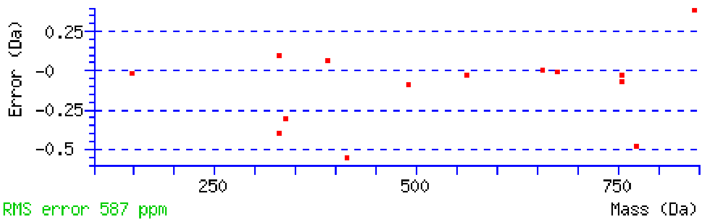

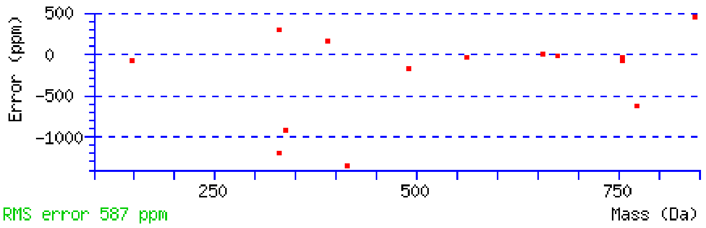

All matches to this query

Score	Mr(calc):	Delta	Sequence
28.8	900.5280	0.9970	GAVIATELK
27.3	900.5280	0.9970	DIVKEVAK
27.2	901.5233	0.0017	VEIATLTR
17.5	900.5141	1.0109	KAQEKAAR
16.2	899.5189	2.0061	DNVVRVAK
11.1	900.5181	1.0069	KVLWEAR
10.6	900.5280	0.9970	DILEGVKK
10.2	900.5280	0.9970	DIKEVGLK
9.9	899.5262	1.9988	VKPMQVAK
9.6	900.5280	0.9970	KIVDEAVK

Spectrum No: 68; Query: 36428; Rank: 1

Peptide View

MS/MS Fragmentation of **EEASNFR**
Found in **IPI00171199**, Tax_Id=9606 Gene_Symbol=PSMA3 Isoform 2 of Proteasome subunit alpha type-3

Match to Query 36428: 851.378468 from(426.696510,2+)
Title: 090702LimSK_Exosome2_05.853.853.2.dta
Data file C:\Temp\Mascoti\LSK_2D\090702LimSK_Exosome2_01_2D.mgf

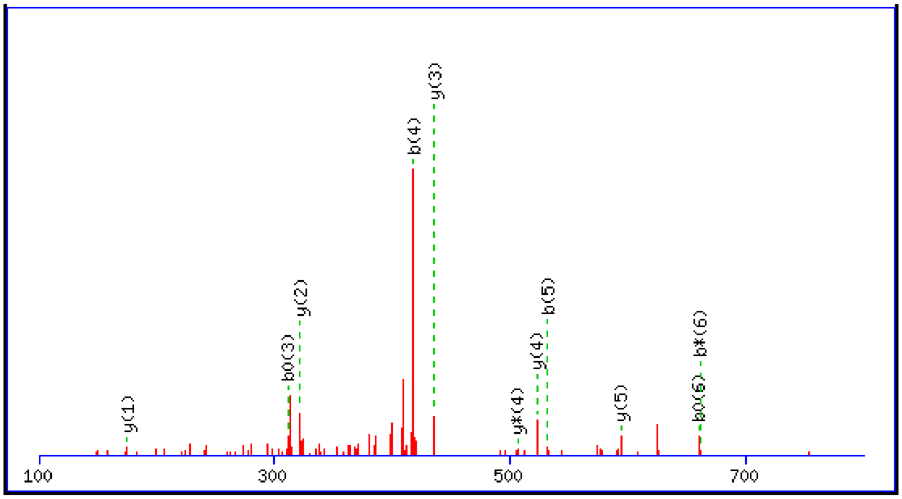

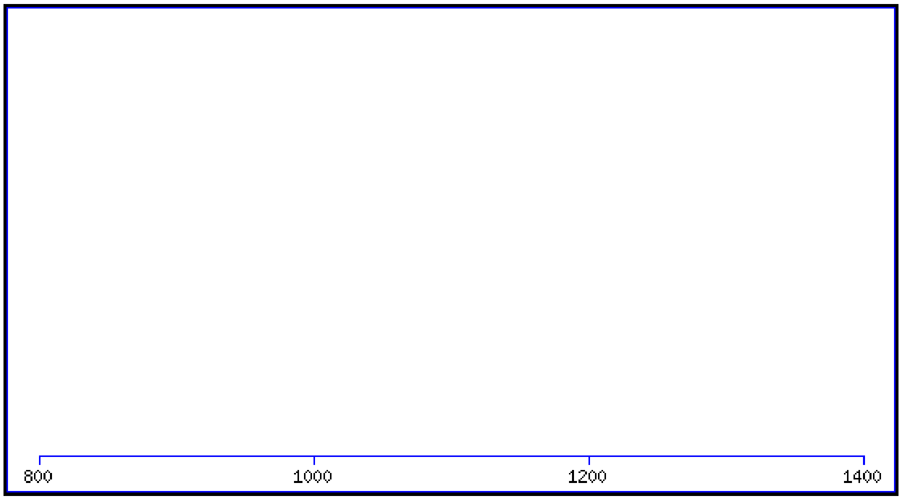

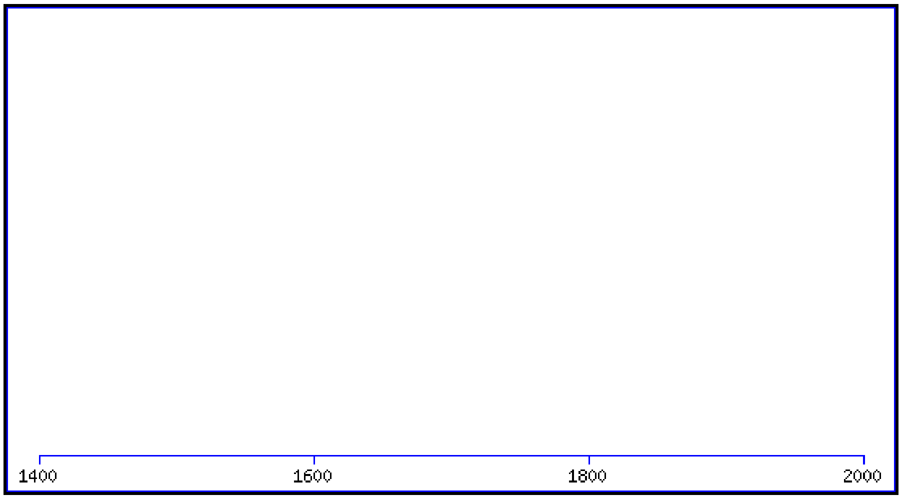

Monoisotopic mass of neutral peptide Mr(calc): 851.3773
Fixed modifications: Carbamidomethyl (C)
Ions Score: 36 **Expect:** 0.016
Matches (Bold Red): 11/58 fragment ions using 21 most intense peaks

#	b	b^{++}	b*	b^{*++}	b^{0}	b^{0++}	Seq.	y	y^{++}	y*	y^{*++}	y^{0}	y^{0++}	#
1	130.0499	65.5286			112.0393	56.5233	E							7
2	259.0925	130.0499			241.0819	121.0446	E	723.3420	362.1747	706.3155	353.6614	705.3315	353.1694	6
3	330.1296	165.5684			**312.1190**	156.5631	A	**594.2994**	297.6534	577.2729	289.1401	576.2889	288.6481	5
4	**417.1616**	209.0844			399.1510	200.0792	S	**523.2623**	262.1348	**506.2358**	253.6215	505.2518	253.1295	4
5	**531.2045**	266.1059	514.1780	257.5926	513.1940	257.1006	N	**436.2303**	218.6188	419.2037	210.1055			3
6	678.2729	339.6401	**661.2464**	331.1268	**660.2624**	330.6348	F	**322.1874**	161.5973	305.1608	153.0840			2

7							R	175.1190	88.0631	158.0924	79.5498			1

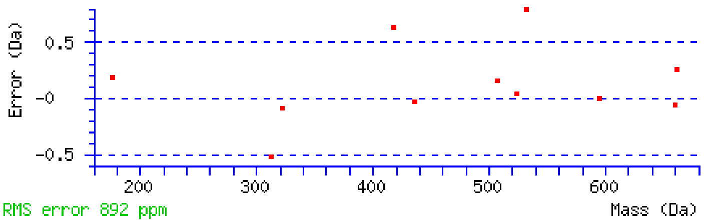

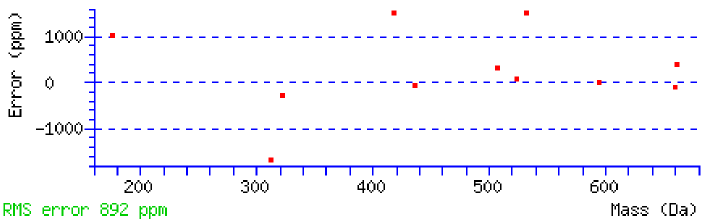

All matches to this query

Score	Mr(calc):	Delta	Sequence
36.4	851.3773	0.0011	EEASNFR
8.8	850.3738	1.0046	KSFSFR
8.7	850.3698	1.0086	LNGTGGPR
8.5	850.3716	1.0069	GRDSCTR
8.2	851.3807	-0.0022	MLSSNER
7.5	850.3698	1.0087	LQSPGNR
6.5	849.3651	2.0134	DGTCIER
6.5	850.3821	0.9964	EDPTSFR
6.5	850.3756	1.0029	MHSSSFR
6.1	851.3742	0.0043	CGKACTR

Spectrum No: 69; Query: 75870; Rank: 1

Peptide View

MS/MS Fragmentation of **ESLKEEDESDDDNM**
Found in **IPI00171199**, Tax_Id=9606 Gene_Symbol=PSMA3 Isoform 2 of Proteasome subunit alpha type-3

Match to Query 75870: 1670.611628 from(836.313090,2+)
Title: 090702LimSK_Exosome2_06.2092.2092.2.dta
Data file C:\Temp\Mascoti\LSK_2D\090702LimSK_Exosome2_01_2D.mgf

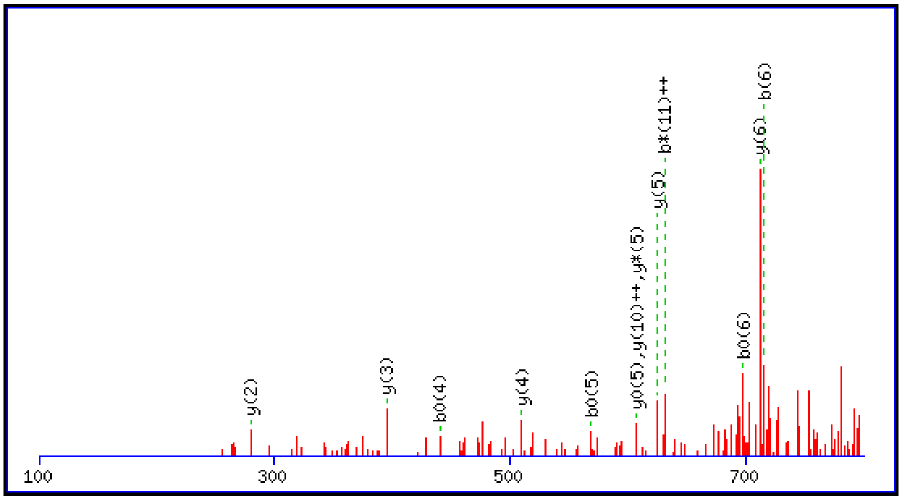

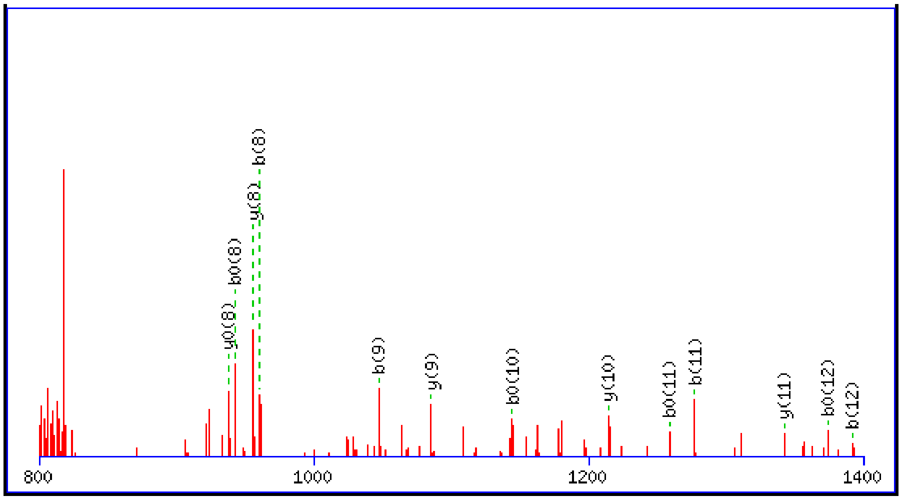

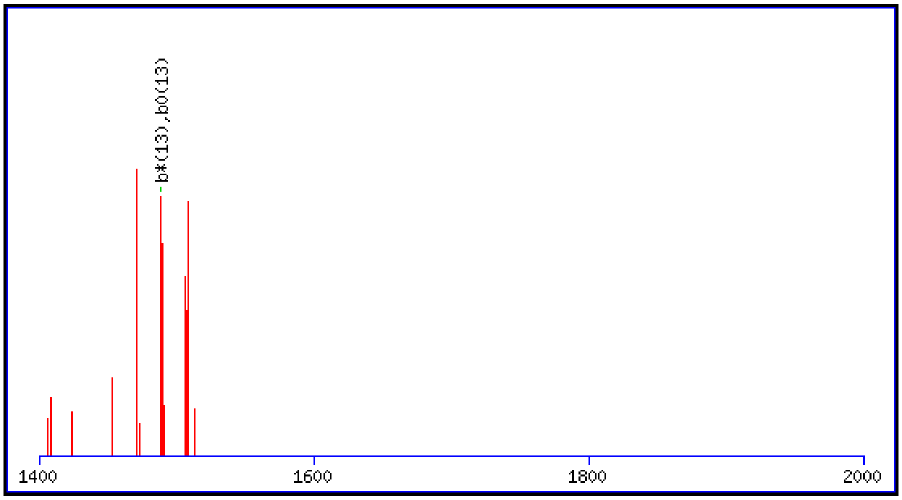

Monoisotopic mass of neutral peptide Mr(calc): 1670.6101
Fixed modifications: Carbamidomethyl (C)
Variable modifications:
M14 : Oxidation (M)
Ions Score: 58 **Expect:** 0.00014
Matches (Bold Red): 28/144 fragment ions using 42 most intense peaks

#	b	b++	b*	b*++	b0	b0++	Seq.	y	y++	y*	y*++	y0	y0++	#
1	130.0499	65.5286			112.0393	56.5233	E							14
2	217.0819	109.0446			199.0713	100.0393	S	1542.5748	771.7910	1525.5483	763.2778	1524.5642	762.7858	13
3	330.1660	165.5866			312.1554	156.5813	L	1455.5428	728.2750	1438.5162	719.7618	1437.5322	719.2697	12
4	458.2609	229.6341	441.2344	221.1208	**440.2504**	220.6288	K	**1342.4587**	671.7330	1325.4322	663.2197	1324.4481	662.7277	11
5	587.3035	294.1554	570.2770	285.6421	**569.2930**	285.1501	E	**1214.3638**	**607.6855**	1197.3372	599.1722	1196.3532	598.6802	10
6	**716.3461**	358.6767	699.3196	350.1634	**698.3355**	349.6714	E	**1085.3212**	543.1642	1068.2946	534.6509	1067.3106	534.1589	9
7	831.3731	416.1902	814.3465	407.6769	813.3625	407.1849	D	**956.2786**	478.6429	939.2520	470.1296	**938.2680**	469.6376	8
8	**960.4156**	480.7115	943.3891	472.1982	**942.4051**	471.7062	E	841.2516	421.1294	824.2251	412.6162	823.2411	412.1242	7
9	**1047.4477**	524.2275	1030.4211	515.7142	1029.4371	515.2222	S	**712.2090**	356.6082	695.1825	348.0949	694.1985	347.6029	6
10	1162.4746	581.7409	1145.4481	573.2277	**1144.4641**	572.7357	D	**625.1770**	313.0921	**608.1505**	304.5789	**607.1664**	304.0869	5
11	**1277.5016**	639.2544	1260.4750	**630.7411**	**1259.4910**	630.2491	D	**510.1501**	255.5787	493.1235	247.0654	492.1395	246.5734	4
12	**1392.5285**	696.7679	1375.5020	688.2546	**1374.5179**	687.7626	D	**395.1231**	198.0652	378.0966	189.5519	377.1126	189.0599	3
13	1506.5714	753.7894	**1489.5449**	745.2761	**1488.5609**	744.7841	N	**280.0962**	140.5517	263.0696	132.0384			2
14							M	166.0532	83.5303					1

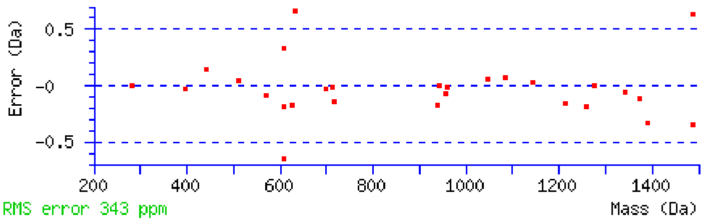

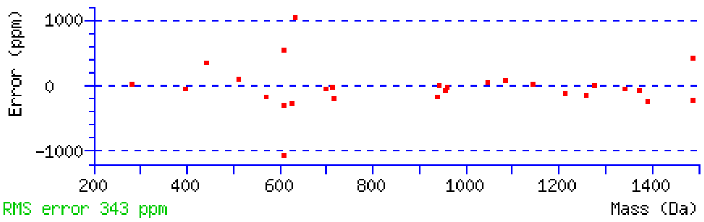

All matches to this query

Score	Mr(calc):	Delta	Sequence
58.0	1670.6101	0.0015	ESLKEEDESDDDNM
0.8	1669.6075	1.0042	NSVTPDMMEEMYK
0.8	1669.6075	1.0042	NSVTPDMMEEMYK

Spectrum No: 70; Query: 27935; Rank: 1

Peptide View

MS/MS Fragmentation of **HEIVPK**
Found in **IPI00171199**, Tax_Id=9606 Gene_Symbol=PSMA3 Isoform 2 of Proteasome subunit alpha type-3

Match to Query 27935: 721.413928 from(361.714240,2+)
Title: 090702LimSK_Exosome2_06.2880.2880.2.dta
Data file C:\Temp\Mascoti\LSK_2D\090702LimSK_Exosome2_01_2D.mgf

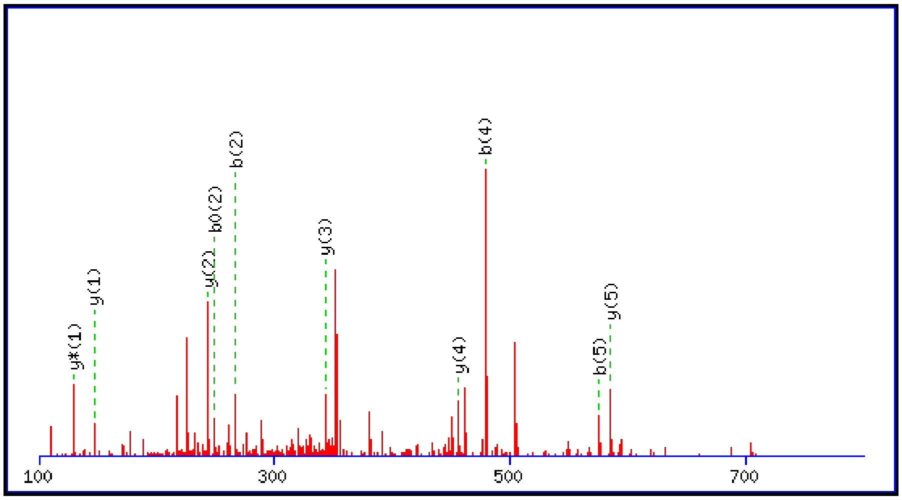

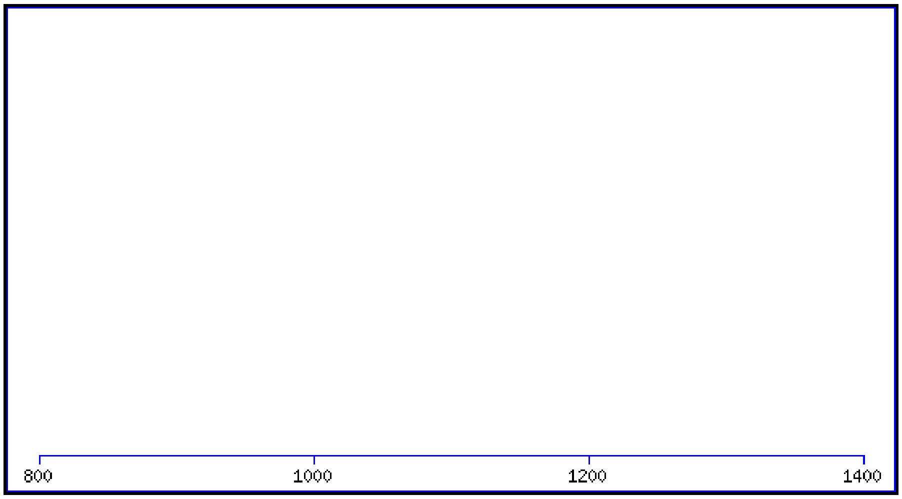

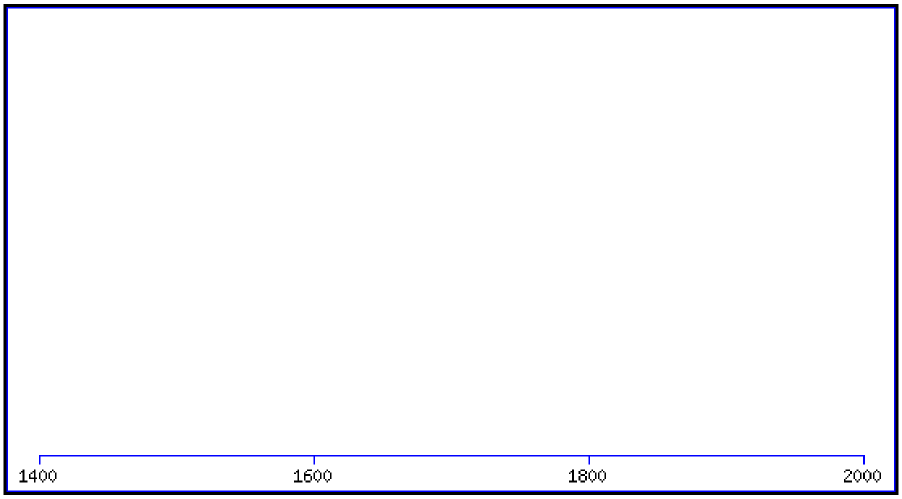

Monoisotopic mass of neutral peptide Mr(calc): 721.4123
Fixed modifications: Carbamidomethyl (C)
Ions Score: 31 **Expect:** 0.049
Matches (Bold Red): 10/40 fragment ions using 32 most intense peaks

#	b	b++	b0	b0++	Seq.	y	y++	y*	y*++	y0	y0++	#
1	138.0662	69.5367			H							6
2	**267.1088**	134.0580	**249.0982**	125.0527	E	**585.3606**	293.1840	568.3341	284.6707	567.3501	284.1787	5
3	380.1928	190.6001	362.1823	181.5948	I	**456.3180**	228.6627	439.2915	220.1494			4
4	**479.2613**	240.1343	461.2507	231.1290	V	**343.2340**	172.1206	326.2074	163.6074			3
5	**576.3140**	288.6606	558.3035	279.6554	P	**244.1656**	122.5864	227.1390	114.0731			2
6					K	**147.1128**	74.0600	**130.0863**	65.5468			1

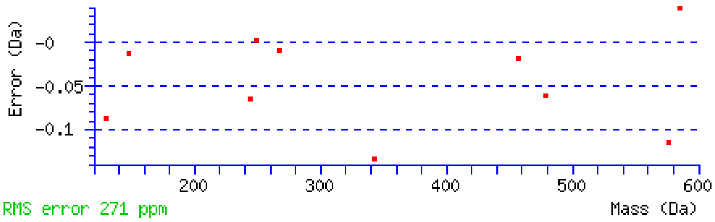

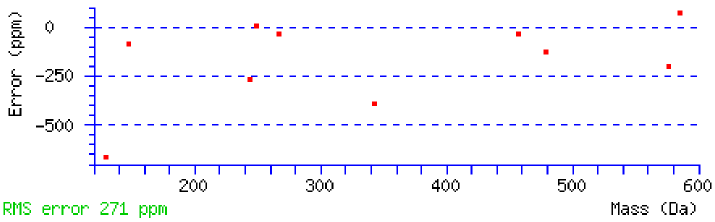

All matches to this query

Score	Mr(calc):	Delta	Sequence
30.9	721.4123	0.0017	HEIVPK
17.4	721.4123	0.0017	HILDPK
17.4	721.4123	0.0017	HLDIPK
17.4	721.4123	0.0017	HVEIPK
15.9	721.4163	-0.0024	FAFLPK
12.1	720.4170	0.9969	KLFDAK
9.8	721.4123	0.0017	ATFAGKK
9.7	721.4156	-0.0017	TAMKKK
7.8	721.4123	0.0017	XVIHPK
6.6	721.4156	-0.0017	AMTKKK

Spectrum No: 71; Query: 64016; Rank: 1

Peptide View

MS/MS Fragmentation of **HVGMAVAGLLADAR**
Found in **IPI00171199**, Tax_Id=9606 Gene_Symbol=PSMA3 Isoform 2 of Proteasome subunit alpha type-3

Match to Query 64016: 1379.738112 from(460.919980,3+)
Title: 090702LimSK_Exosome2_01.6447.6447.3.dta
Data file C:\Temp\Mascoti\LSK_2D\090702LimSK_Exosome2_01_2D.mgf

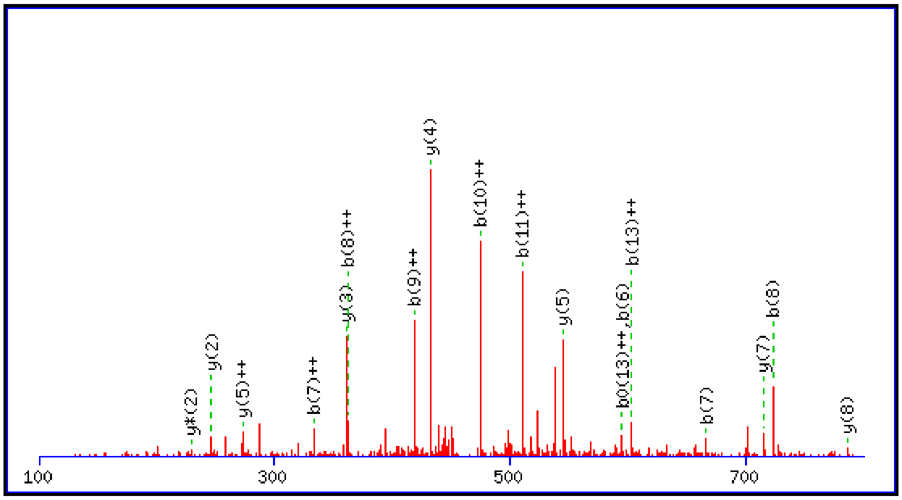

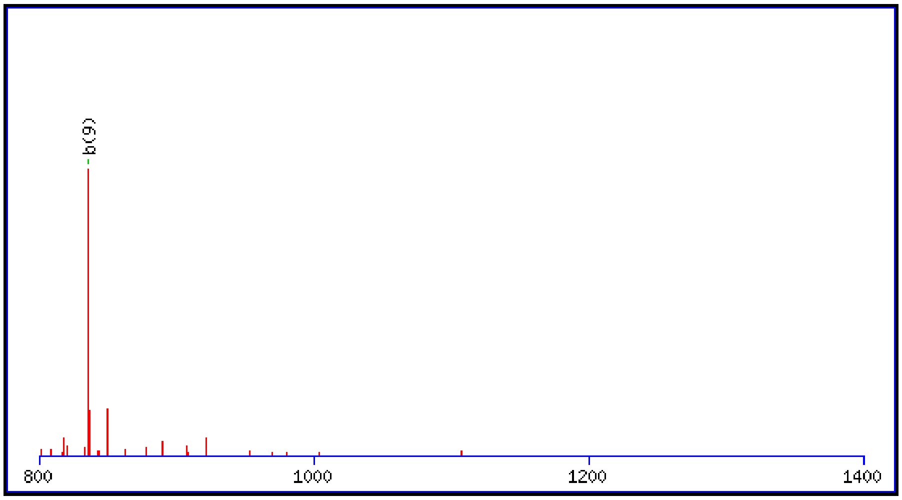

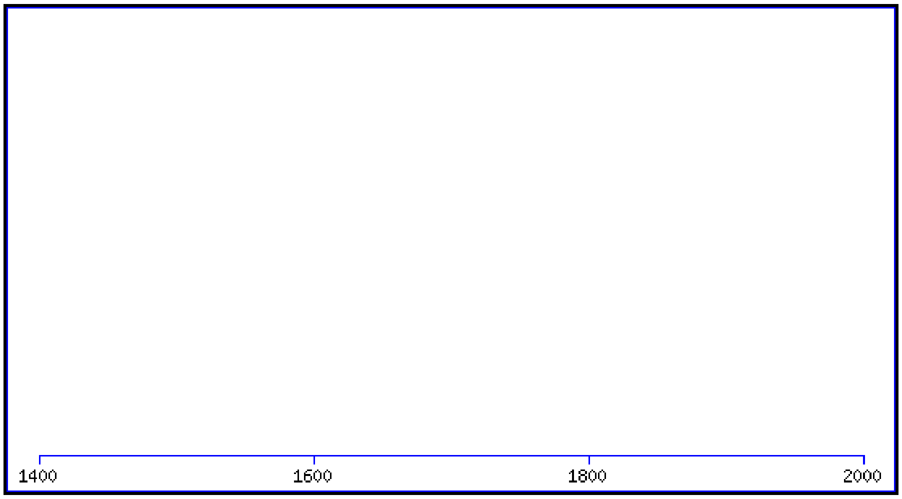

Monoisotopic mass of neutral peptide Mr(calc): 1379.7343
Fixed modifications: Carbamidomethyl (C)
Ions Score: 31 **Expect:** 0.083
Matches (Bold Red): 19/104 fragment ions using 41 most intense peaks

#	b	b^{++}	b^{0}	b^{0++}	Seq.	y	y^{++}	y^{*}	y^{*++}	y^{0}	y^{0++}	#
1	138.0662	69.5367			H							14

**2**	237.1346	119.0709			**V**	1243.6827	622.3450	1226.6562	613.8317	1225.6722	613.3397	**13**
**3**	294.1561	147.5817			**G**	1144.6143	572.8108	1127.5878	564.2975	1126.6037	563.8055	**12**
**4**	425.1966	213.1019			**M**	1087.5928	544.3001	1070.5663	535.7868	1069.5823	535.2948	**11**
**5**	496.2337	248.6205			**A**	956.5524	478.7798	939.5258	470.2665	938.5418	469.7745	**10**
**6**	**595.3021**	298.1547			**V**	885.5152	443.2613	868.4887	434.7480	867.5047	434.2560	**9**
**7**	**666.3392**	**333.6732**			**A**	**786.4468**	393.7271	769.4203	385.2138	768.4363	384.7218	**8**
**8**	**723.3607**	**362.1840**			**G**	**715.4097**	358.2085	698.3832	349.6952	697.3992	349.2032	**7**
**9**	**836.4447**	**418.7260**			**L**	658.3883	329.6978	641.3617	321.1845	640.3777	320.6925	**6**
**10**	949.5288	**475.2680**			**L**	**545.3042**	**273.1557**	528.2776	264.6425	527.2936	264.1504	**5**
**11**	1020.5659	**510.7866**			**A**	**432.2201**	216.6137	415.1936	208.1004	414.2096	207.6084	**4**
**12**	1135.5928	568.3001	1117.5823	559.2948	**D**	**361.1830**	181.0951	344.1565	172.5819	343.1724	172.0899	**3**
**13**	1206.6300	**603.8186**	1188.6194	**594.8133**	**A**	**246.1561**	123.5817	**229.1295**	115.0684			**2**
**14**					**R**	175.1190	88.0631	158.0924	79.5498			**1**

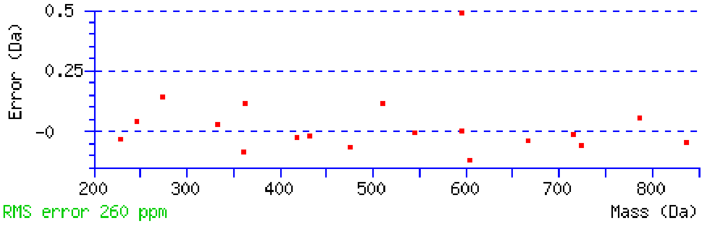

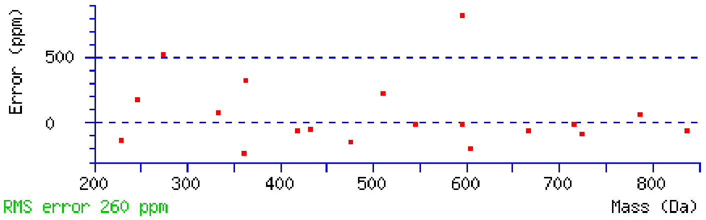

All matches to this query

Score	Mr(calc):	Delta	Sequence
31.5	1379.7343	0.0038	HVGMAVAGLLADAR
15.7	1379.7247	0.0134	SITPLMLSGIIR
11.3	1378.7333	1.0048	SLAVASKAKTPAR
11.2	1379.7382	-0.0000	ARKPGPDRAGAER
10.8	1379.7247	0.0134	SITPLMLSGIIR
9.1	1377.7404	1.9977	WLNEYKIKER
8.9	1378.7221	1.0160	LSSLIIGPSKER
8.1	1378.7317	1.0064	VAAQGARPLPEDR
7.8	1379.7343	0.0038	ACTIAIRYSAVR
7.7	1378.7429	0.9952	SLATNHPARKER

Spectrum No: 72; Query: 30498; Rank: 1

Peptide View

MS/MS Fragmentation of **LFNVDR**
Found in **IPI00171199**, Tax_Id=9606 Gene_Symbol=PSMA3 Isoform 2 of Proteasome subunit alpha type-3

Match to Query 30498: 762.403708 from(382.209130,2+)
Title: 090702LimSK_Exosome2_06.2765.2765.2.dta
Data file C:\Temp\Mascoti\LSK_2D\090702LimSK_Exosome2_01_2D.mgf

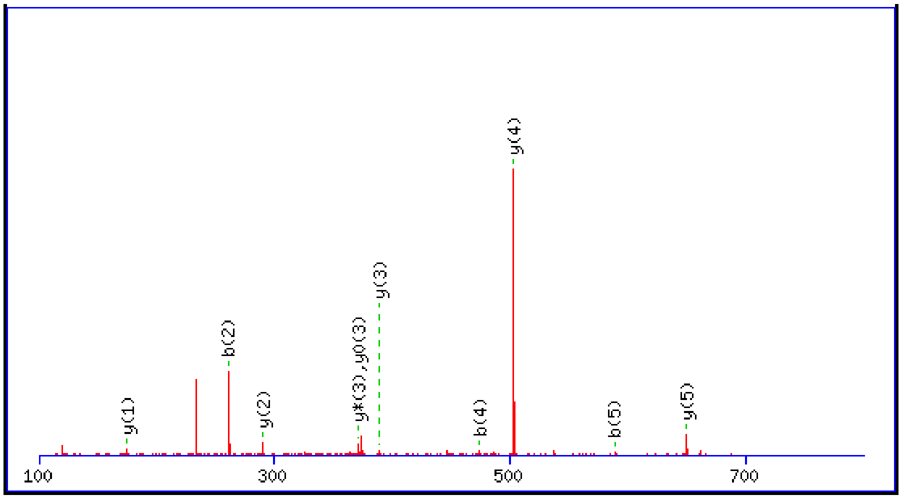

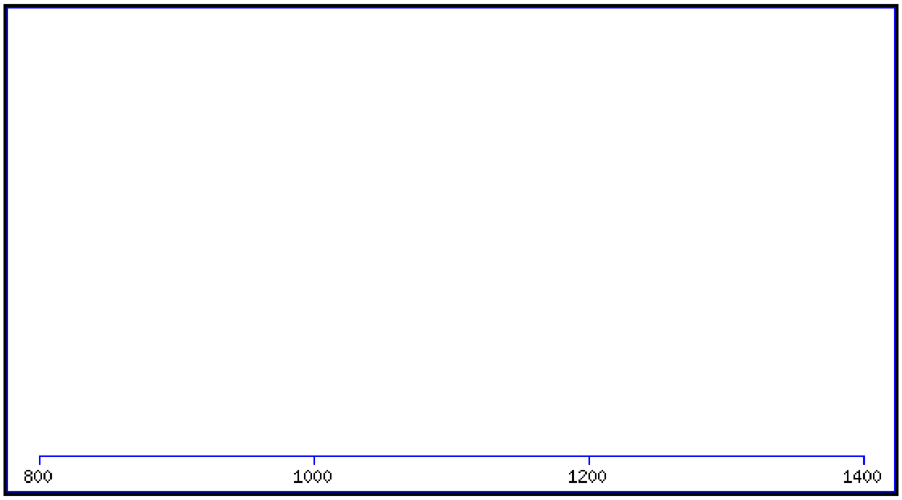

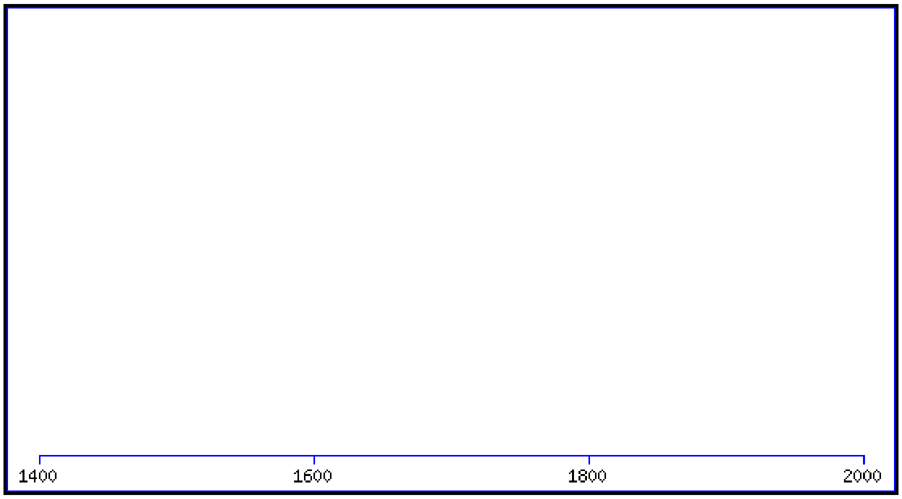

Monoisotopic mass of neutral peptide Mr(calc): 762.4024
Fixed modifications: Carbamidomethyl (C)
Ions Score: 35 **Expect:** 0.034
Matches (Bold Red): 10/46 fragment ions using 19 most intense peaks

#	b	b^{++}	b*	b^{*++}	b^{0}	b^{0++}	Seq.	y	y^{++}	y*	y^{*++}	y^{0}	y^{0++}	#
1	114.0913	57.5493					L							6
2	**261.1598**	131.0835					F	**650.3257**	325.6665	633.2991	317.1532	632.3151	316.6612	5
3	375.2027	188.1050	358.1761	179.5917			N	**503.2572**	252.1323	486.2307	243.6190	485.2467	243.1270	4
4	**474.2711**	237.6392	457.2445	229.1259			V	**389.2143**	195.1108	**372.1878**	186.5975	**371.2037**	186.1055	3
5	**589.2980**	295.1527	572.2715	286.6394	571.2875	286.1474	D	**290.1459**	145.5766	273.1193	137.0633	272.1353	136.5713	2
6							R	**175.1190**	88.0631	158.0924	79.5498			1

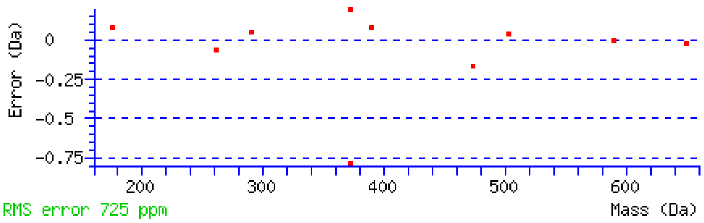

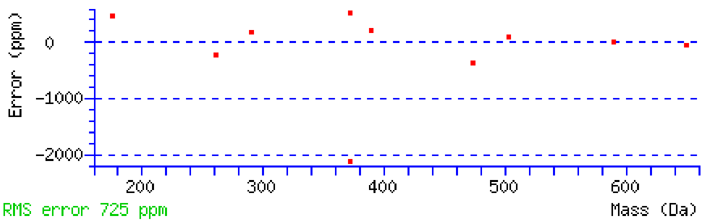

All matches to this query

Score	Mr(calc):	Delta	Sequence
35.4	762.4024	0.0013	LFNVDR
19.0	762.4058	-0.0021	LMEARK
18.8	761.3993	1.0044	EMLELK
18.8	761.3993	1.0044	MELEIK
18.1	762.4058	-0.0021	IMKEAR
18.1	762.4024	0.0013	LFQAER
18.0	762.4058	-0.0021	MEKISR
18.0	762.4058	-0.0021	MLQISR
16.3	762.4058	-0.0021	LMTNLR
16.2	762.4058	-0.0021	MLEKAR

Spectrum No: 73; Query: 47821; Rank: 1

Peptide View

MS/MS Fragmentation of **LYEEGSNKR**
Found in **IPI00171199**, Tax_Id=9606 Gene_Symbol=PSMA3 Isoform 2 of Proteasome subunit alpha type-3

Match to Query 47821: 1094.537228 from(548.275890,2+)
Title: 090702LimSK_Exosome2_06.1809.1809.2.dta
Data file C:\Temp\Mascoti\LSK_2D\090702LimSK_Exosome2_01_2D.mgf

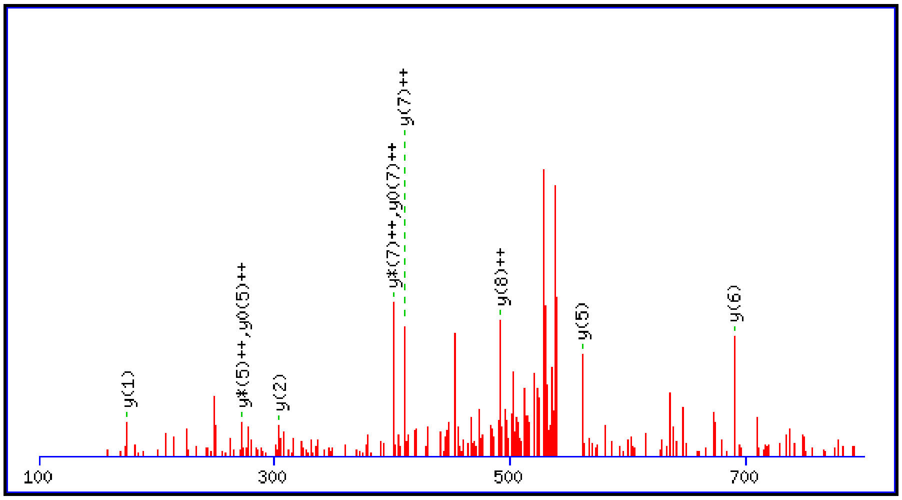

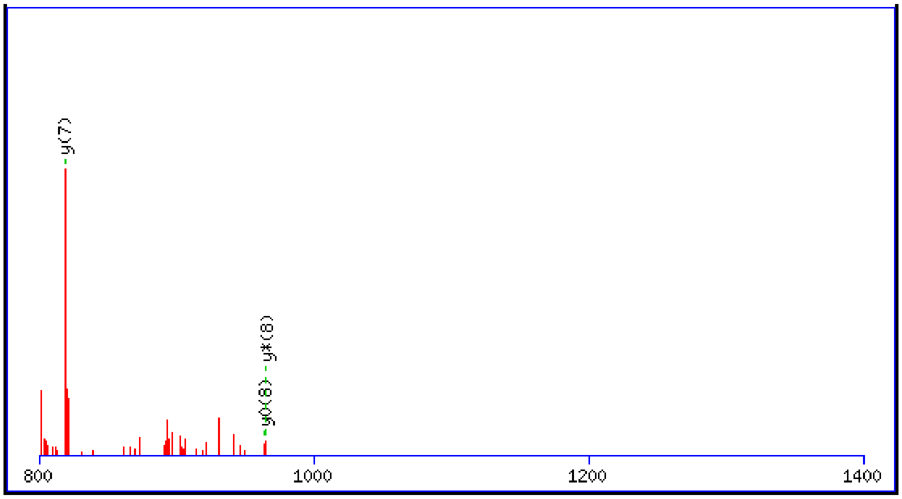

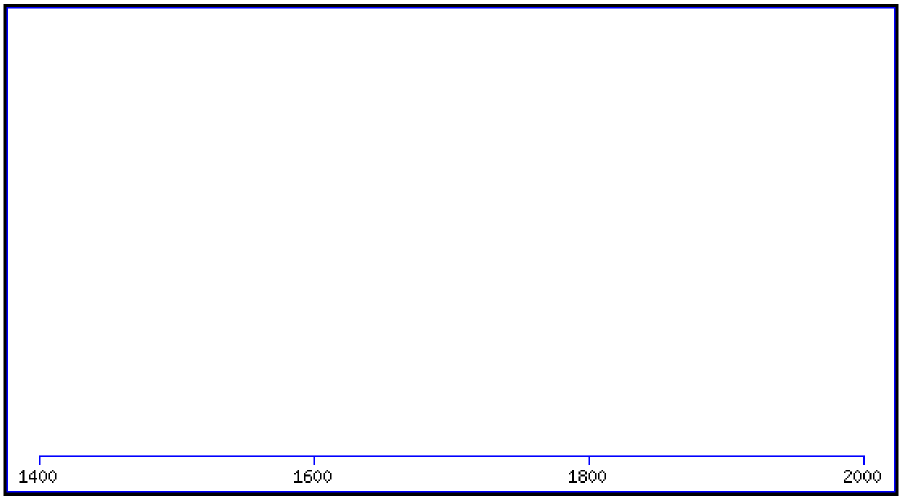

Monoisotopic mass of neutral peptide Mr(calc): 1094.5356
Fixed modifications: Carbamidomethyl (C)
Ions Score: 27 **Expect:** 0.3
Matches (Bold Red): 13/74 fragment ions using 22 most intense peaks

#	b	b^{++}	b*	b^{*++}	b^0	b^{0++}	Seq.	y	y^{++}	y*	y^{*++}	y^0	y^{0++}	#
1	114.0913	57.5493					L							9
2	277.1547	139.0810					Y	982.4588	**491.7331**	**965.4323**	483.2198	**964.4483**	482.7278	8
3	406.1973	203.6023			388.1867	194.5970	E	**819.3955**	**410.2014**	802.3690	**401.6881**	801.3850	**401.1961**	7
4	535.2399	268.1236			517.2293	259.1183	E	**690.3529**	345.6801	673.3264	337.1668	672.3424	336.6748	6
5	592.2613	296.6343			574.2508	287.6290	G	**561.3103**	281.1588	544.2838	**272.6455**	543.2998	**272.1535**	5
6	679.2933	340.1503			661.2828	331.1450	S	504.2889	252.6481	487.2623	244.1348	486.2783	243.6428	4
7	793.3363	397.1718	776.3097	388.6585	775.3257	388.1665	N	417.2568	209.1321	400.2303	200.6188			3
8	921.4312	461.2193	904.4047	452.7060	903.4207	452.2140	K	**303.2139**	152.1106	286.1874	143.5973			2
9							R	**175.1190**	88.0631	158.0924	79.5498			1

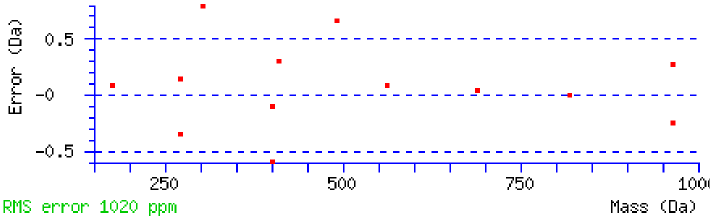

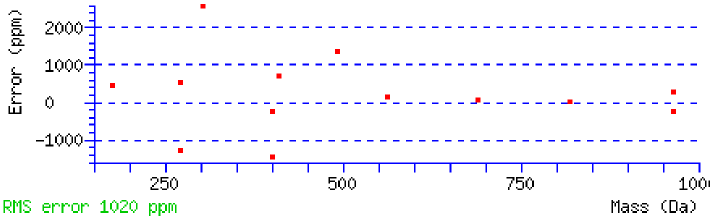

All matches to this query

Score	Mr(calc):	Delta	Sequence

26.6	1094.5356	0.0016	LYEEGSNKR
26.2	1092.5233	2.0139	QKMEEEKR
14.1	1092.5233	2.0139	DSRMAEEKK
11.3	1094.5291	0.0081	MAGGSGFGKQR
11.2	1093.5260	1.0113	MENGLMKQK
10.9	1093.5372	1.0000	KMKDCQLR
7.0	1093.5281	1.0091	EEKRTVPR
6.6	1093.5363	1.0009	SSNTSQSQKK
5.7	1092.5263	2.0109	MAASRKPPR
5.5	1093.5339	1.0034	KYGVGTCGPR

Spectrum No: 74; Query: 29419; Rank: 1

Peptide View

MS/MS Fragmentation of **SLADIAR**
Found in **IPI00171199**, Tax_Id=9606 Gene_Symbol=PSMA3 Isoform 2 of Proteasome subunit alpha type-3

Match to Query 29419: 744.413988 from(373.214270,2+)
Title: 090702LimSK_Exosome2_06.2385.2385.2.dta
Data file C:\Temp\Mascoti\LSK_2D\090702LimSK_Exosome2_01_2D.mgf

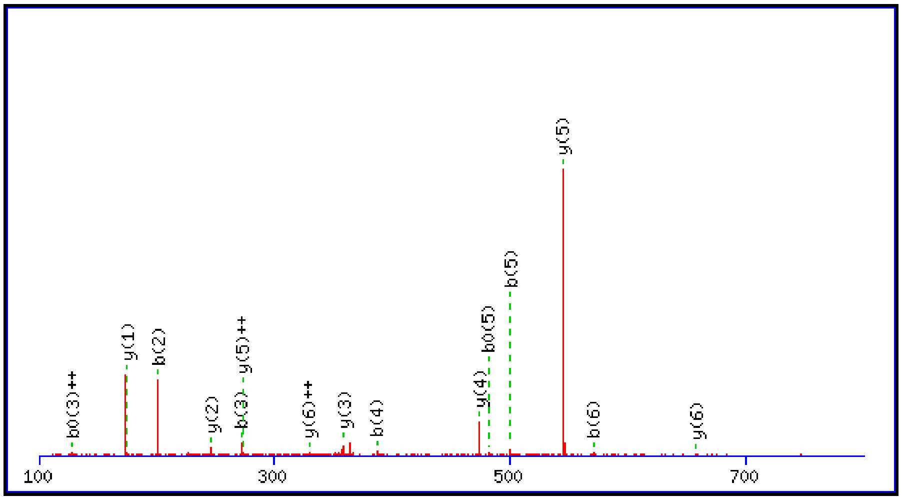

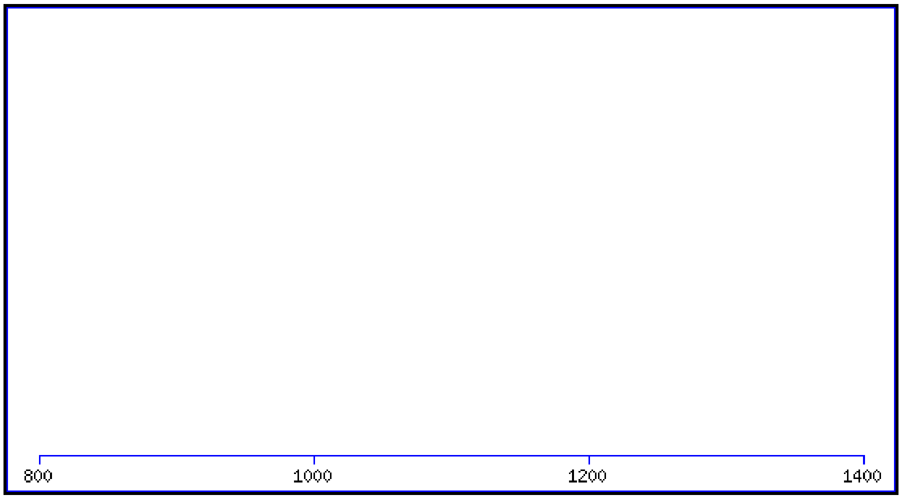

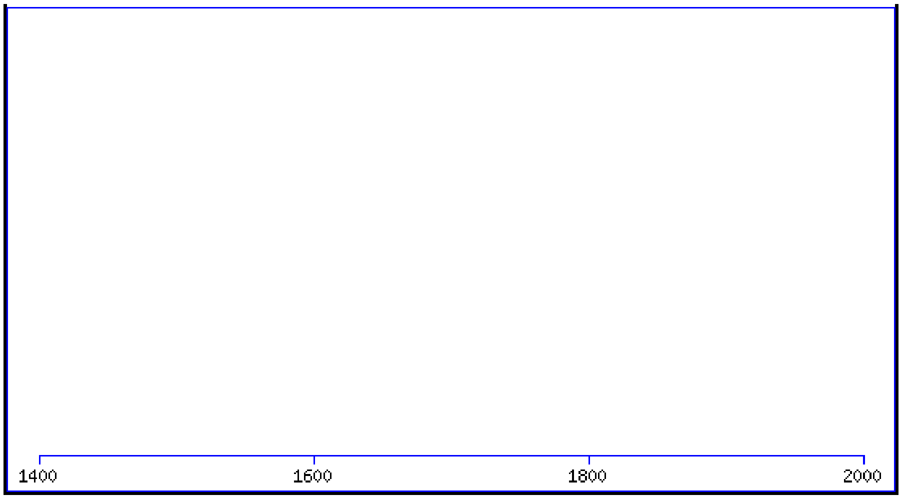

Monoisotopic mass of neutral peptide Mr(calc): 744.4130
Fixed modifications: Carbamidomethyl (C)
Ions Score: 48 **Expect:** 0.002
Matches (Bold Red): 15/54 fragment ions using 32 most intense peaks

#	b	b^{++}	b^{0}	b^{0++}	Seq.	y	y^{++}	y*	y^{*++}	y^{0}	y^{0++}	#
1	88.0393	44.5233	70.0287	35.5180	S							7
2	**201.1234**	101.0653	183.1128	92.0600	L	**658.3883**	**329.6978**	641.3617	321.1845	640.3777	320.6925	6
3	**272.1605**	136.5839	254.1499	**127.5786**	A	**545.3042**	**273.1557**	528.2776	264.6425	527.2936	264.1504	5
4	**387.1874**	194.0974	369.1769	185.0921	D	**474.2671**	237.6372	457.2405	229.1239	456.2565	228.6319	4
5	**500.2715**	250.6394	**482.2609**	241.6341	I	**359.2401**	180.1237	342.2136	171.6104			3
6	**571.3086**	286.1579	553.2980	277.1527	A	**246.1561**	123.5817	229.1295	115.0684			2
7					R	**175.1190**	88.0631	158.0924	79.5498			1

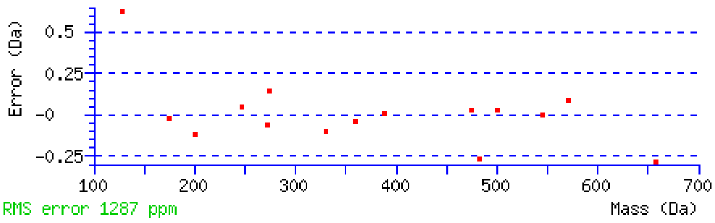

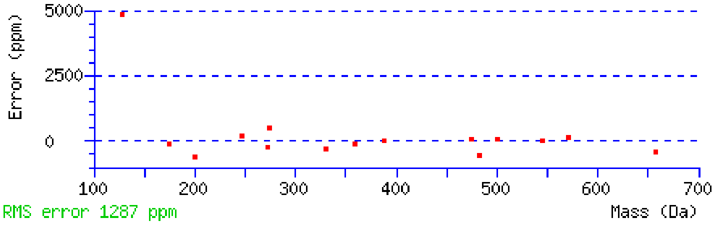

All matches to this query

Score	Mr(calc):	Delta	Sequence
48.3	744.4130	0.0010	SLADIAR
17.0	744.4130	0.0010	TVAAEVR
13.3	744.4130	0.0010	ISELGAR
13.0	743.4078	1.0062	AQALWR
12.9	744.4130	0.0010	TVAGELR
12.6	744.4130	0.0010	SLSPSVR
11.4	744.4130	0.0010	IASEVAR
10.8	744.4130	0.0010	TGLIDAR
10.8	744.4130	0.0010	ISVSPSR
10.4	744.4130	0.0010	TVDAAIR

Spectrum No: 75; Query: 52238; Rank: 1

Peptide View

MS/MS Fragmentation of **SNFGYNIPLK**
Found in **IPI00171199**, Tax_Id=9606 Gene_Symbol=PSMA3 Isoform 2 of Proteasome subunit alpha type-3

Match to Query 52238: 1151.598828 from(576.806690,2+)
Title: 090702LimSK_Exosome2_06.6362.6362.2.dta
Data file C:\Temp\Mascoti\LSK_2D\090702LimSK_Exosome2_01_2D.mgf

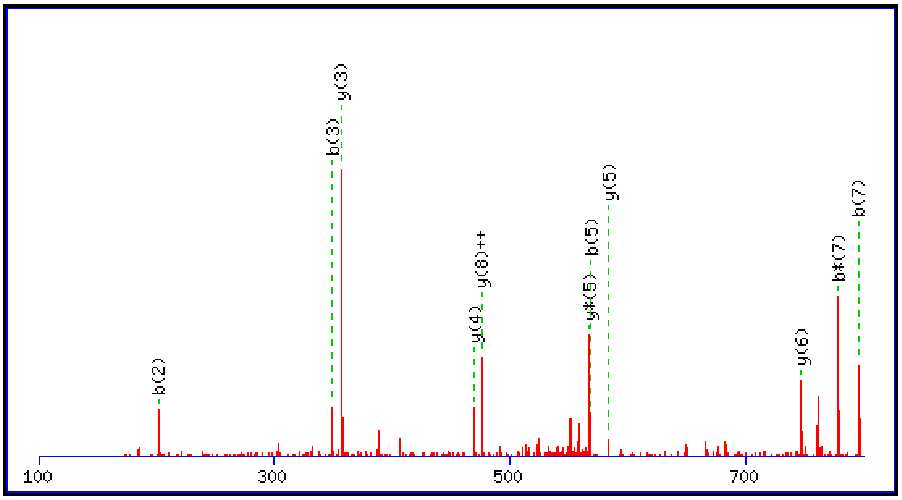

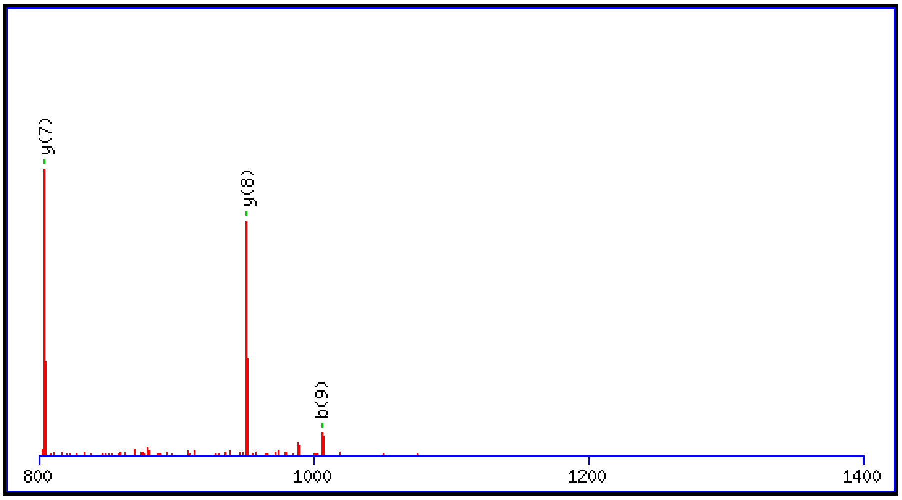

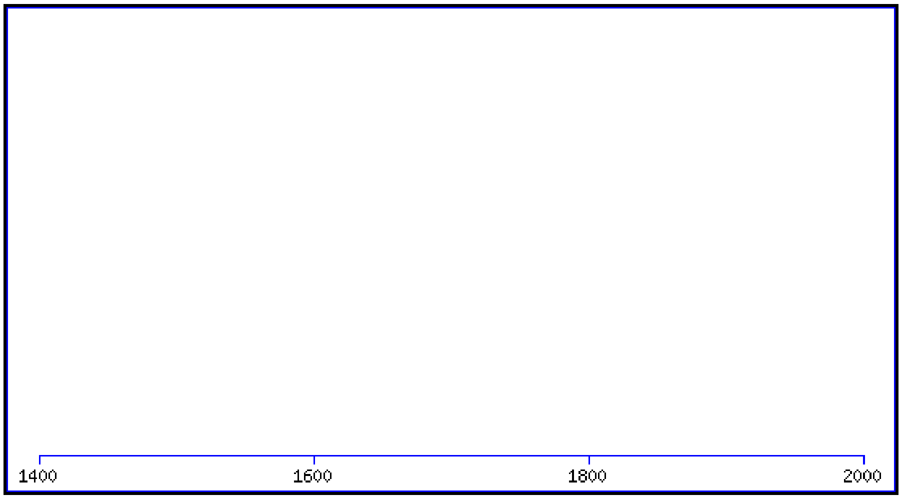

Monoisotopic mass of neutral peptide Mr(calc): 1151.5975
Fixed modifications: Carbamidomethyl (C)
Ions Score: 55 **Expect:** 0.00048
Matches (Bold Red): 14/88 fragment ions using 19 most intense peaks

#	b	b^{++}	b*	b^{*++}	b^0	b^{0++}	Seq.	y	y^{++}	y*	y^{*++}	#
1	88.0393	44.5233			70.0287	35.5180	S					10

2	**202.0822**	101.5448	185.0557	93.0315	184.0717	92.5395	N	1065.5728	533.2900	1048.5462	524.7767	9
3	**349.1506**	175.0790	332.1241	166.5657	331.1401	166.0737	F	**951.5298**	**476.2686**	934.5033	467.7553	8
4	406.1721	203.5897	389.1456	195.0764	388.1615	194.5844	G	**804.4614**	402.7343	787.4349	394.2211	7
5	**569.2354**	285.1214	552.2089	276.6081	551.2249	276.1161	Y	**747.4400**	374.2236	730.4134	365.7103	6
6	683.2784	342.1428	666.2518	333.6295	665.2678	333.1375	N	**584.3766**	292.6919	**567.3501**	284.1787	5
7	**796.3624**	398.6849	**779.3359**	390.1716	778.3519	389.6796	I	**470.3337**	235.6705	453.3071	227.1572	4
8	893.4152	447.2112	876.3886	438.6980	875.4046	438.2060	P	**357.2496**	179.1285	340.2231	170.6152	3
9	**1006.4993**	503.7533	989.4727	495.2400	988.4887	494.7480	L	260.1969	130.6021	243.1703	122.0888	2
10							K	147.1128	74.0600	130.0863	65.5468	1

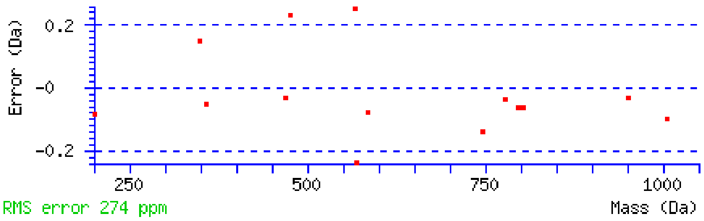

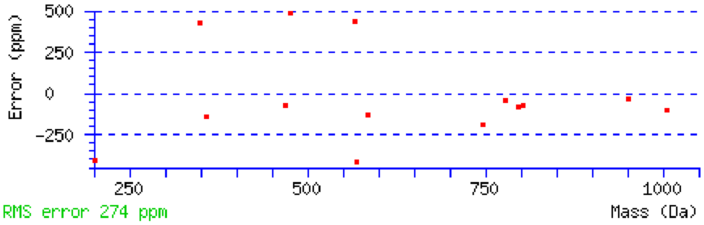

All matches to this query

Score	Mr(calc):	Delta	Sequence
55.1	1151.5975	0.0013	SNFGYNIPLK
10.5	1151.6063	-0.0075	KLDQTIARK
10.1	1151.5951	0.0037	VLKEGGSLAAK
7.1	1150.5860	1.0129	SPRVKIDTR
6.3	1151.5951	0.0037	QLTAGTLLQK
6.3	1151.6087	-0.0099	YGKHKTYQK
5.0	1151.6104	-0.0115	SALWKILNK
3.7	1149.6019	1.9969	NPVTRTKVR
3.4	1150.5860	1.0129	LQDGRITIR
3.1	1151.5951	0.0037	LNEQSKLLK

Spectrum No: 76; Query: 48974; Rank: 1

Peptide View

MS/MS Fragmentation of **VFQVEYAMK**
Found in **IPI00171199**, Tax_Id=9606 Gene_Symbol=PSMA3 Isoform 2 of Proteasome subunit alpha type-3

Match to Query 48974: 1113.554528 from(557.784540,2+)
Title: 090702LimSK_Exosome2_05.5580.5580.2.dta
Data file C:\Temp\Mascoti\LSK_2D\090702LimSK_Exosome2_01_2D.mgf

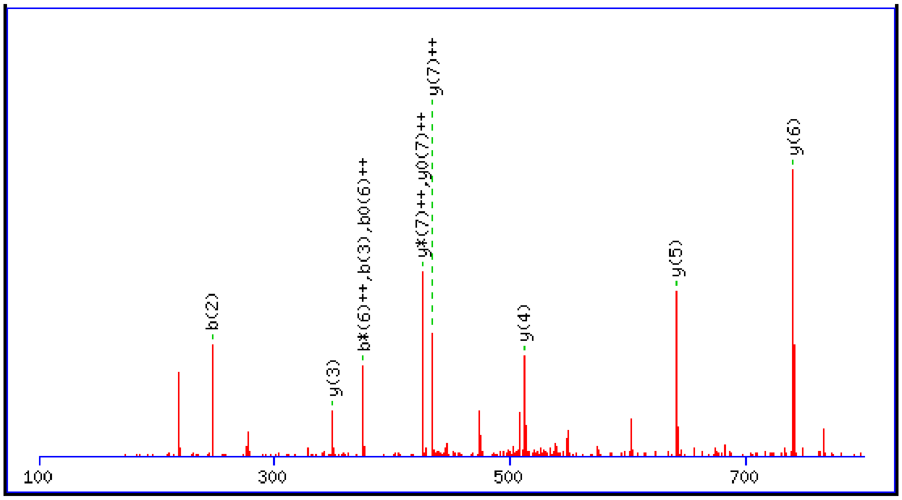

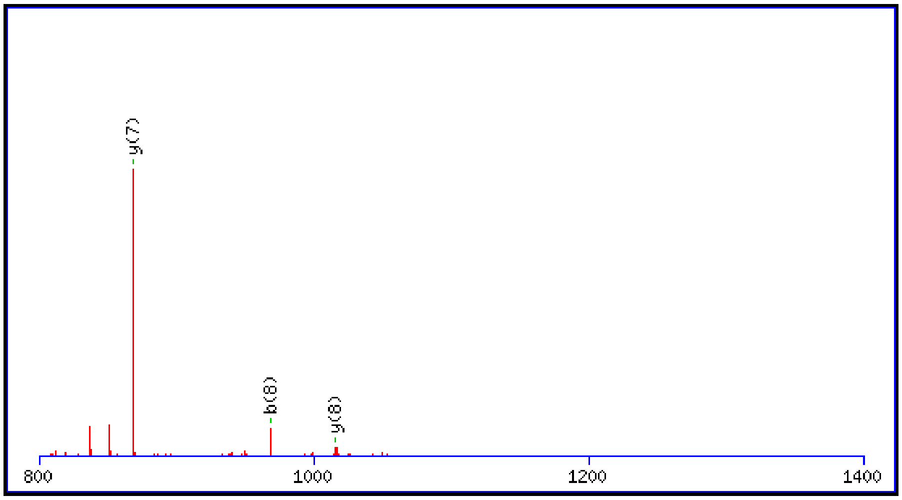

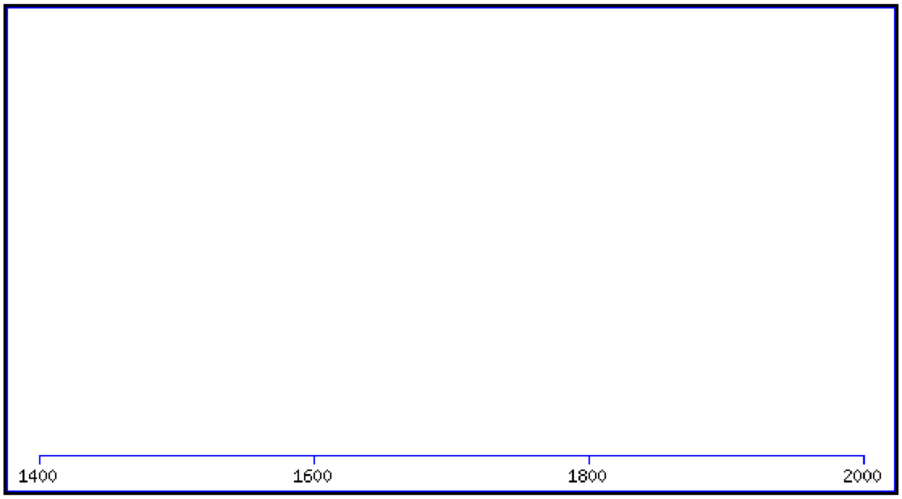

Monoisotopic mass of neutral peptide Mr(calc): 1113.5529
Fixed modifications: Carbamidomethyl (C)
Ions Score: 61 **Expect:** 0.0001
Matches (Bold Red): 14/76 fragment ions using 13 most intense peaks

#	b	b^{++}	b*	b^{*++}	b^0	b^{0++}	Seq.	y	y^{++}	y*	y^{*++}	y^0	y^{0++}	#
1	100.0757	50.5415					V							9
2	**247.1441**	124.0757					F	**1015.4917**	508.2495	998.4652	499.7362	997.4812	499.2442	8
3	**375.2027**	188.1050	358.1761	179.5917			Q	**868.4233**	**434.7153**	851.3968	**426.2020**	850.4128	**425.7100**	7
4	474.2711	237.6392	457.2445	229.1259			V	**740.3647**	370.6860	723.3382	362.1727	722.3542	361.6807	6
5	603.3137	302.1605	586.2871	293.6472	585.3031	293.1552	E	**641.2963**	321.1518	624.2698	312.6385	623.2858	312.1465	5
6	766.3770	383.6921	749.3505	**375.1789**	748.3665	**374.6869**	Y	**512.2537**	256.6305	495.2272	248.1172			4

7	837.4141	419.2107	820.3876	410.6974	819.4036	410.2054	A	349.1904	175.0988	332.1639	166.5856			3
8	968.4546	484.7309	951.4281	476.2177	950.4441	475.7257	M	278.1533	139.5803	261.1267	131.0670			2
9							K	147.1128	74.0600	130.0863	65.5468			1

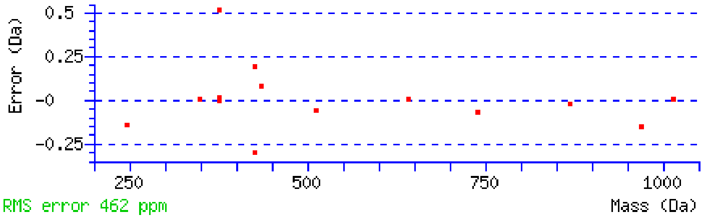

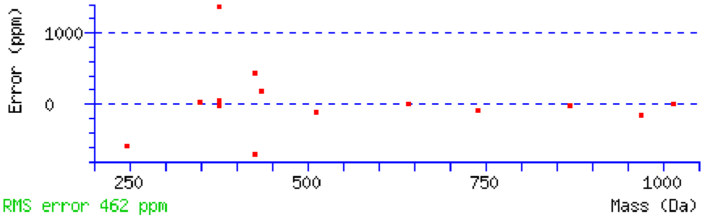

All matches to this query

Score	Mr(calc):	Delta	Sequence
61.4	1113.5529	0.0017	VFQVEYAMK
22.6	1111.5526	2.0019	LLTDSDLKK
19.3	1113.5444	0.0101	VFQGSGRRK
14.2	1111.5526	2.0019	LLTDSDLKK
13.7	1111.5526	2.0019	LITTTEQVK
13.7	1111.5526	2.0019	LITTTEQVK
13.7	1111.5526	2.0019	LITTTEQVK
13.7	1111.5387	2.0159	TSNKVEARK
13.7	1111.5387	2.0159	TSNKVEARK
13.2	1111.5526	2.0019	DLLTSSGVLK

Spectrum No: 77; Query: 84861; Rank: 1

Peptide View

MS/MS Fragmentation of **AGGSASAMLQPLLDNQVGFK**
Found in **IPI00025019**, Tax_Id=9606 Gene_Symbol=PSMB1 Proteasome subunit beta type-1

Match to Query 84861: 2003.018648 from(1002.516600,2+)
Title: 090702LimSK_Exosome2_06.8499.8499.2.dta
Data file C:\Temp\Mascoti\LSK_2D\090702LimSK_Exosome2_01_2D.mgf

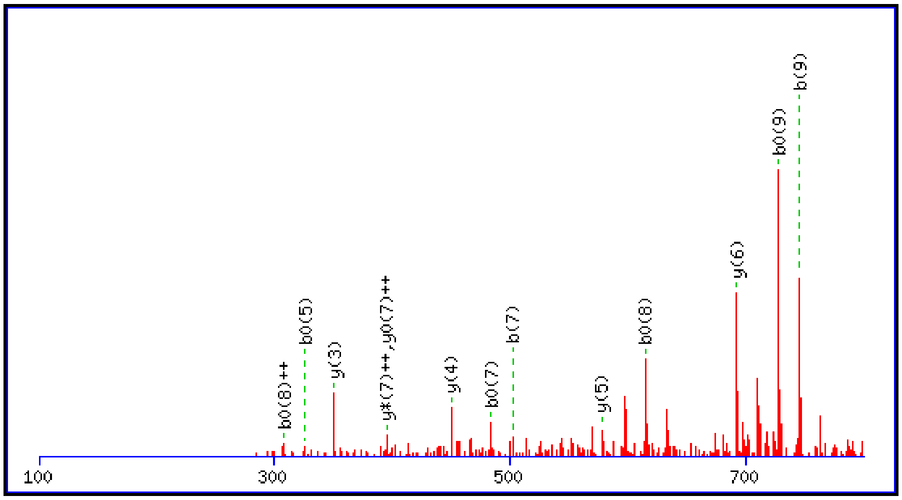

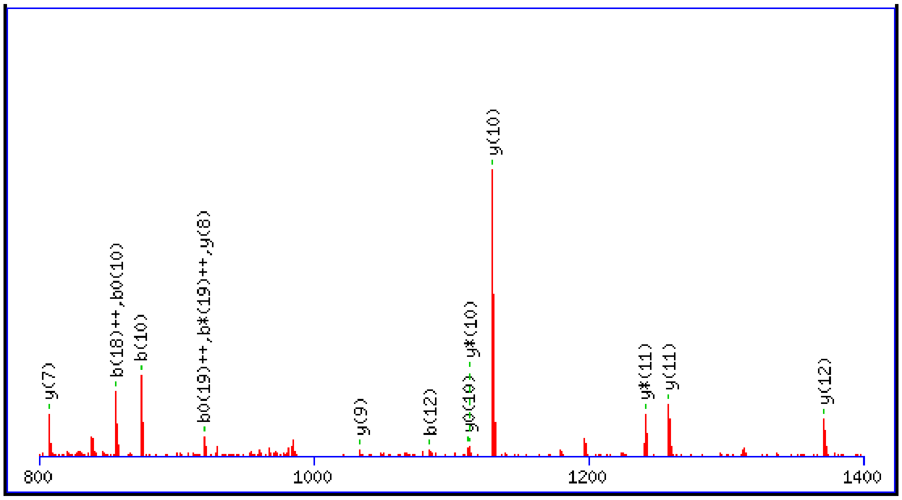

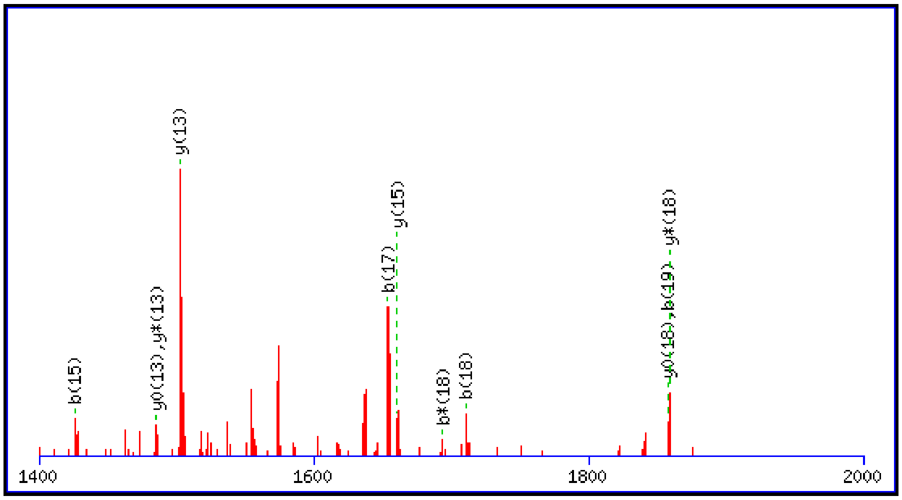

Monoisotopic mass of neutral peptide Mr(calc): 2003.0146
Fixed modifications: Carbamidomethyl (C)
Ions Score: 76 **Expect:** 4.9e-006
Matches (Bold Red): 39/192 fragment ions using 55 most intense peaks

#	b	b^{++}	b*	b*$^{++}$	b^{0}	b^{0++}	Seq.	y	y^{++}	y*	y*$^{++}$	y^{0}	y^{0++}	#
1	72.0444	36.5258					A							20
2	129.0659	65.0366					G	1932.9848	966.9960	1915.9582	958.4828	1914.9742	957.9907	19
3	186.0873	93.5473					G	1875.9633	938.4853	**1858.9368**	929.9720	**1857.9527**	929.4800	18
4	273.1193	137.0633			255.1088	128.0580	S	1818.9418	909.9746	1801.9153	901.4613	1800.9313	900.9693	17
5	344.1565	172.5819			**326.1459**	163.5766	A	1731.9098	866.4585	1714.8833	857.9453	1713.8993	857.4533	16
6	431.1885	216.0979			413.1779	207.0926	S	**1660.8727**	830.9400	1643.8462	822.4267	1642.8621	821.9347	15
7	**502.2256**	251.6164			**484.2150**	242.6112	A	1573.8407	787.4240	1556.8141	778.9107	1555.8301	778.4187	14
8	633.2661	317.1367			**615.2555**	**308.1314**	M	**1502.8036**	751.9054	**1485.7770**	743.3921	**1484.7930**	742.9001	13
9	**746.3502**	373.6787			**728.3396**	364.6734	L	**1371.7631**	686.3852	1354.7365	677.8719	1353.7525	677.3799	12
10	**874.4087**	437.7080	857.3822	429.1947	**856.3982**	428.7027	Q	**1258.6790**	629.8431	**1241.6525**	621.3299	1240.6684	620.8379	11
11	971.4615	486.2344	954.4349	477.7211	953.4509	477.2291	P	**1130.6204**	565.8139	**1113.5939**	557.3006	**1112.6099**	556.8086	10
12	**1084.5456**	542.7764	1067.5190	534.2631	1066.5350	533.7711	L	**1033.5677**	517.2875	1016.5411	508.7742	1015.5571	508.2822	9
13	1197.6296	599.3184	1180.6031	590.8052	1179.6191	590.3132	L	**920.4836**	460.7454	903.4571	452.2322	902.4730	451.7402	8
14	1312.6566	656.8319	1295.6300	648.3186	1294.6460	647.8266	D	**807.3995**	404.2034	790.3730	**395.6901**	789.3890	**395.1981**	7
15	**1426.6995**	713.8534	1409.6729	705.3401	1408.6889	704.8481	N	**692.3726**	346.6899	675.3461	338.1767			6
16	1554.7581	777.8827	1537.7315	769.3694	1536.7475	768.8774	Q	**578.3297**	289.6685	561.3031	281.1552			5
17	**1653.8265**	827.4169	1636.7999	818.9036	1635.8159	818.4116	V	**450.2711**	225.6392	433.2445	217.1259			4
18	**1710.8479**	**855.9276**	**1693.8214**	847.4143	1692.8374	846.9223	G	**351.2027**	176.1050	334.1761	167.5917			3
19	**1857.9164**	929.4618	1840.8898	**920.9485**	1839.9058	**920.4565**	F	294.1812	147.5942	277.1547	139.0810			2

**20**							**K**	147.1128	74.0600	130.0863	65.5468			**1**

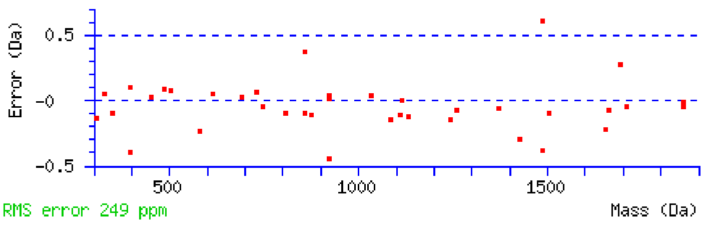

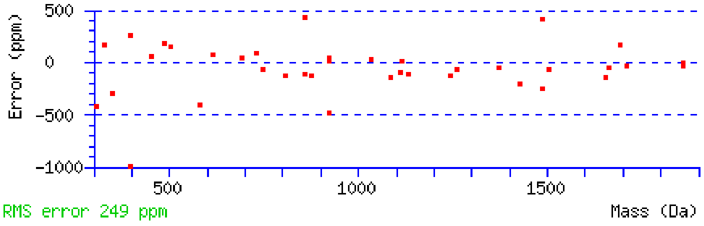

All matches to this query

Score	Mr(calc):	Delta	Sequence
75.7	2003.0146	0.0040	AGGSASAMLQPLLDNQVGFK
4.2	2003.0088	0.0098	RASPVADIQEEILNAAVK
1.4	2002.0032	1.0154	MDQQEFSIAMKLIKLK
0.0	2002.0119	1.0067	EVKYGAPQATDGLAGALDAR

Spectrum No: 78; Query: 43125; Rank: 1

Peptide View

MS/MS Fragmentation of **DVFISAAER**
Found in **IPI00025019**, Tax_Id=9606 Gene_Symbol=PSMB1 Proteasome subunit beta type-1

Match to Query 43125: 1006.510108 from(504.262330,2+)
Title: 090702LimSK_Exosome2_06.3543.3543.2.dta
Data file C:\Temp\Mascoti\LSK_2D\090702LimSK_Exosome2_01_2D.mgf

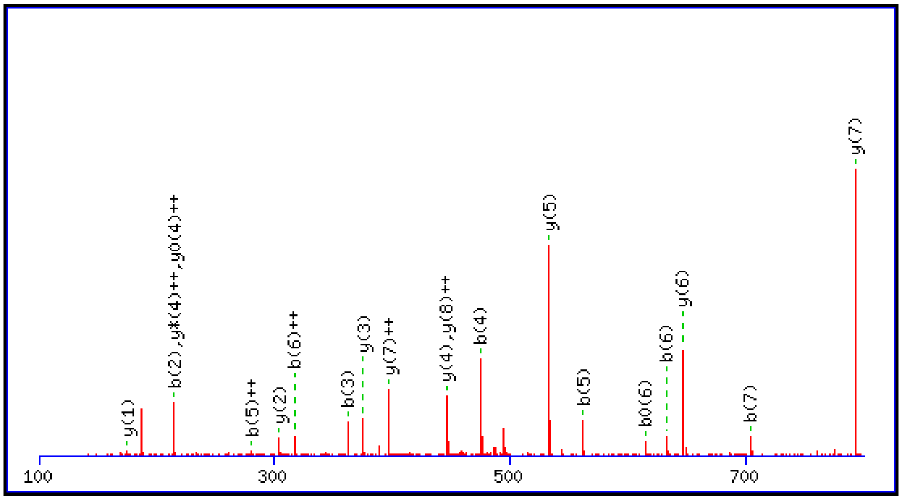

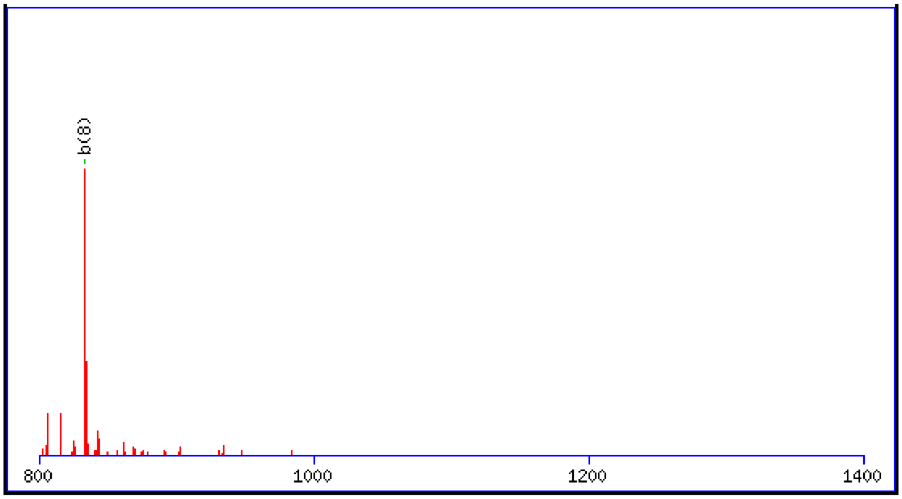

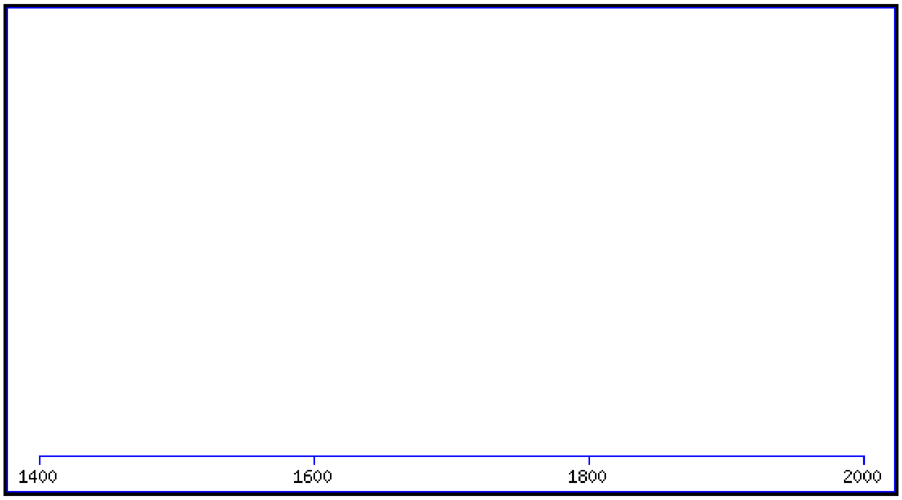

Monoisotopic mass of neutral peptide Mr(calc): 1006.5083
Fixed modifications: Carbamidomethyl (C)
Ions Score: 55 **Expect:** 0.00042
Matches (Bold Red): 21/78 fragment ions using 33 most intense peaks

#	b	b^{++}	b^{0}	b^{0++}	Seq.	y	y^{++}	y*	y^{*++}	y^{0}	y^{0++}	#
1	116.0342	58.5207	98.0237	49.5155	D							9
2	**215.1026**	108.0550	197.0921	99.0497	V	892.4887	**446.7480**	875.4621	438.2347	874.4781	437.7427	8
3	**362.1710**	181.5892	344.1605	172.5839	F	**793.4203**	**397.2138**	776.3937	388.7005	775.4097	388.2085	7
4	**475.2551**	238.1312	457.2445	229.1259	I	**646.3519**	323.6796	629.3253	315.1663	628.3413	314.6743	6
5	**562.2871**	**281.6472**	544.2766	272.6419	S	**533.2678**	267.1375	516.2413	258.6243	515.2572	258.1323	5
6	**633.3243**	**317.1658**	**615.3137**	308.1605	A	**446.2358**	223.6215	429.2092	**215.1083**	428.2252	**214.6162**	4
7	**704.3614**	352.6843	686.3508	343.6790	A	**375.1987**	188.1030	358.1721	179.5897	357.1881	179.0977	3
8	**833.4040**	417.2056	815.3934	408.2003	E	**304.1615**	152.5844	287.1350	144.0711	286.1510	143.5791	2
9					R	**175.1190**	88.0631	158.0924	79.5498			1

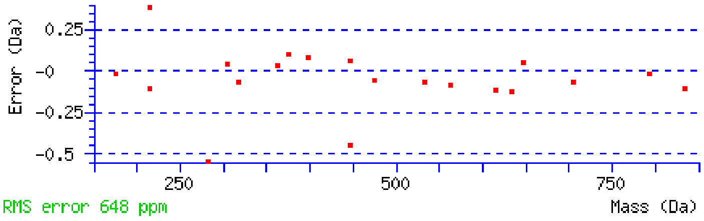

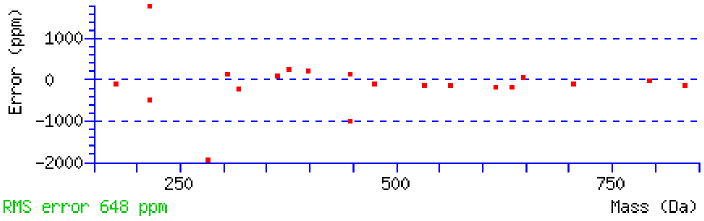

All matches to this query

Score	Mr(calc):	Delta	Sequence

55.0	1006.5083	0.0018	DVFISAAER
10.6	1006.5084	0.0017	DVAEVTAFR
10.3	1004.5073	2.0028	VLCKGSGER
9.1	1004.4960	2.0141	LVASMEEAR
7.3	1006.5117	-0.0016	DVVMANSKK
6.1	1006.5083	0.0018	FLNVTEER
4.4	1006.5117	-0.0016	ITTMQLER
4.0	1004.5096	2.0005	IFLTYIR
3.5	1005.5165	0.9936	VIMITGDNK
2.8	1004.4999	2.0102	SGRAGTGGAGSK

Spectrum No: 79; Query: 43308; Rank: 1

Peptide View

MS/MS Fragmentation of **DVYTGDALR**
Found in **IPI00025019**, Tax_Id=9606 Gene_Symbol=PSMB1 Proteasome subunit beta type-1

Match to Query 43308: 1008.488208 from(505.251380,2+)
Title: 090702LimSK_Exosome2_06.2438.2438.2.dta
Data file C:\Temp\Mascoti\LSK_2D\090702LimSK_Exosome2_01_2D.mgf

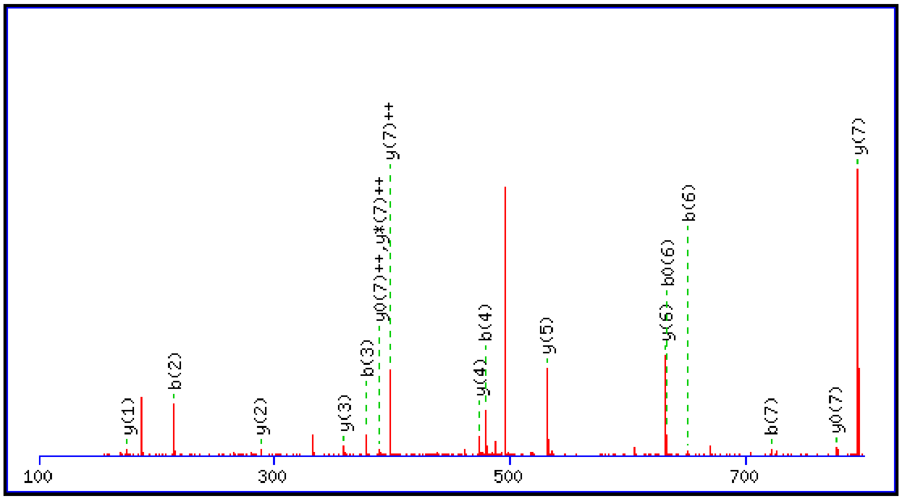

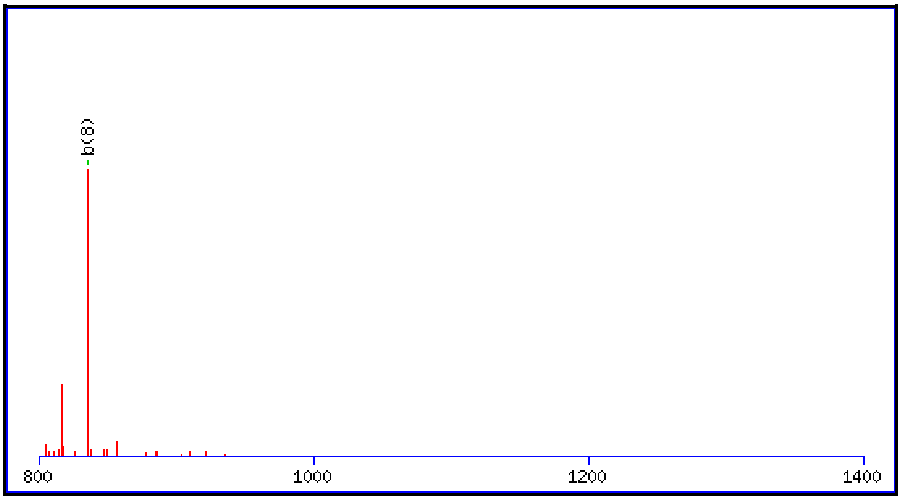

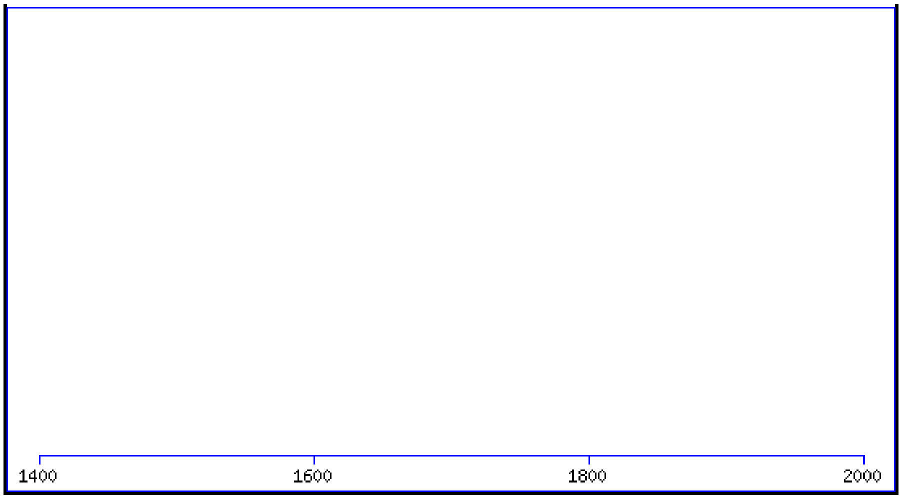

Monoisotopic mass of neutral peptide Mr(calc): 1008.4876
Fixed modifications: Carbamidomethyl (C)
Ions Score: 45 **Expect:** 0.0035
Matches (Bold Red): 18/74 fragment ions using 34 most intense peaks

#	b	b^{++}	b^{0}	b^{0++}	Seq.	y	y^{++}	y*	y^{*++}	y^{0}	y^{0++}	#
1	116.0342	58.5207	98.0237	49.5155	D							9
2	**215.1026**	108.0550	197.0921	99.0497	V	894.4680	447.7376	877.4414	439.2243	876.4574	438.7323	8
3	**378.1660**	189.5866	360.1554	180.5813	Y	**795.3995**	**398.2034**	778.3730	**389.6901**	**777.3890**	**389.1981**	7
4	**479.2136**	240.1105	461.2031	231.1052	T	**632.3362**	316.6717	615.3097	308.1585	614.3257	307.6665	6
5	536.2351	268.6212	518.2245	259.6159	G	**531.2885**	266.1479	514.2620	257.6346	513.2780	257.1426	5
6	**651.2620**	326.1347	**633.2515**	317.1294	D	**474.2671**	237.6372	457.2405	229.1239	456.2565	228.6319	4
7	**722.2992**	361.6532	704.2886	352.6479	A	**359.2401**	180.1237	342.2136	171.6104			3
8	**835.3832**	418.1953	817.3727	409.1900	L	**288.2030**	144.6051	271.1765	136.0919			2
9					R	**175.1190**	88.0631	158.0924	79.5498			1

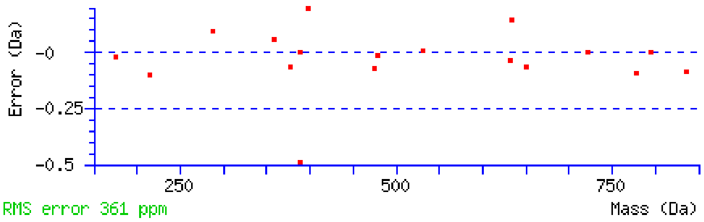

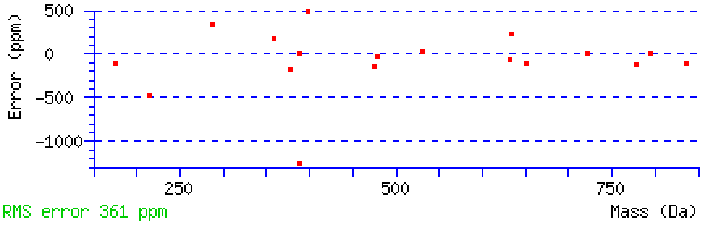

All matches to this query

Score	Mr(calc):	Delta	Sequence
45.0	1008.4876	0.0006	DVYTGDALR
11.7	1008.4876	0.0006	VDYGDVSVR
8.2	1007.4801	1.0081	VLGAPATSGR
6.1	1006.4866	2.0016	TLATCAAGSR
5.6	1007.4801	1.0081	STLTTHLR
5.5	1008.4845	0.0038	MDTMRSLR
4.8	1008.4827	0.0055	MPGVSVAIR
4.7	1008.4811	0.0071	CQATFNLR
4.1	1007.4801	1.0081	VLGAPATSGR
3.8	1007.4801	1.0081	APTEKLGGR

Spectrum No: 80; Query: 71749; Rank: 1

Peptide View

MS/MS Fragmentation of **GAVYSFDPVGSYQR**
Found in **IPI00025019**, Tax_Id=9606 Gene_Symbol=PSMB1 Proteasome subunit beta type-1

Match to Query 71749: 1544.728948 from(773.371750,2+)
Title: 090702LimSK_Exosome2_04.6424.6424.2.dta
Data file C:\Temp\Mascoti\LSK_2D\090702LimSK_Exosome2_01_2D.mgf

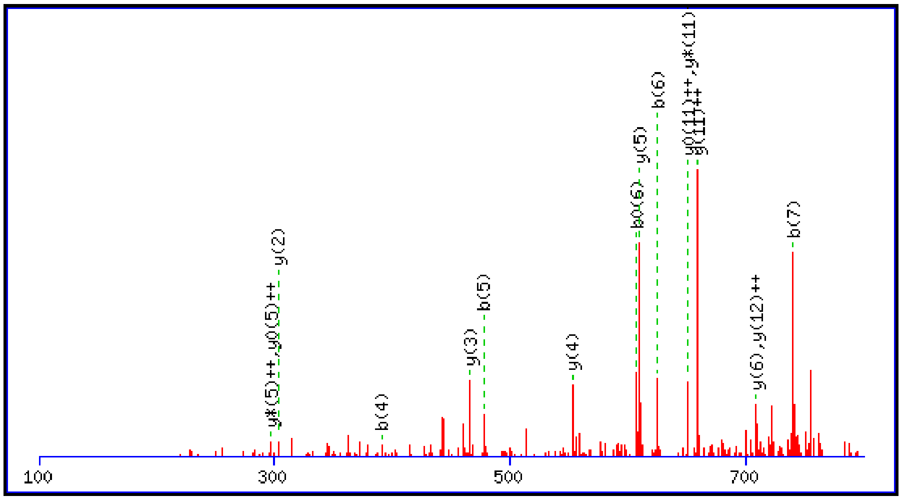

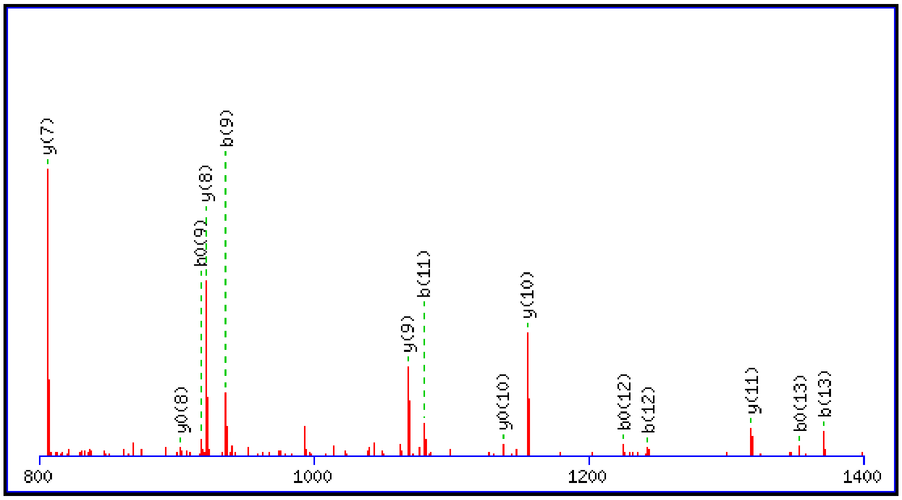

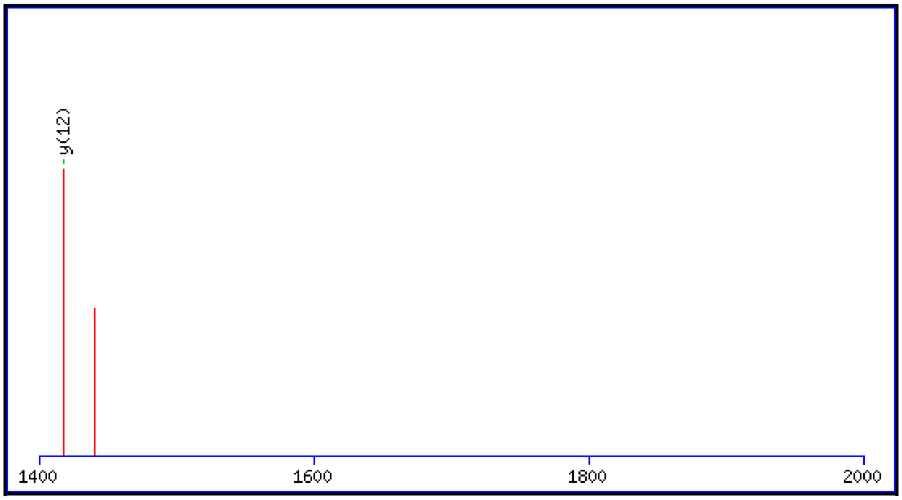

Monoisotopic mass of neutral peptide Mr(calc): 1544.7260
Fixed modifications: Carbamidomethyl (C)
Ions Score: 77 **Expect:** 4.1e-006
Matches (Bold Red): 31/118 fragment ions using 49 most intense peaks

#	b	b^{++}	b*	b^{*++}	b^{0}	b^{0++}	Seq.	y	y^{++}	y*	y^{*++}	y^{0}	y^{0++}	#
1	58.0287	29.5180					G							14
2	129.0659	65.0366					A	1488.7118	744.8595	1471.6852	736.3462	1470.7012	735.8542	13
3	228.1343	114.5708					V	**1417.6747**	**709.3410**	1400.6481	700.8277	1399.6641	700.3357	12
4	**391.1976**	196.1024					Y	**1318.6062**	**659.8068**	1301.5797	**651.2935**	1300.5957	**650.8015**	11
5	**478.2296**	239.6185			460.2191	230.6132	S	**1155.5429**	578.2751	1138.5164	569.7618	**1137.5323**	569.2698	10
6	**625.2980**	313.1527			**607.2875**	304.1474	F	**1068.5109**	534.7591	1051.4843	526.2458	1050.5003	525.7538	9
7	**740.3250**	370.6661			722.3144	361.6608	D	**921.4425**	461.2249	904.4159	452.7116	**903.4319**	452.2196	8
8	837.3777	419.1925			819.3672	410.1872	P	**806.4155**	403.7114	789.3890	395.1981	788.4050	394.7061	7
9	**936.4462**	468.7267			**918.4356**	459.7214	V	**709.3628**	355.1850	692.3362	346.6717	691.3522	346.1797	6
10	993.4676	497.2375			975.4571	488.2322	G	**610.2944**	305.6508	593.2678	**297.1375**	592.2838	**296.6455**	5
11	**1080.4997**	540.7535			1062.4891	531.7482	S	**553.2729**	277.1401	536.2463	268.6268	535.2623	268.1348	4
12	**1243.5630**	622.2851			**1225.5524**	613.2798	Y	**466.2409**	233.6241	449.2143	225.1108			3
13	**1371.6216**	686.3144	1354.5950	677.8011	**1353.6110**	677.3091	Q	**303.1775**	152.0924	286.1510	143.5791			2
14							R	175.1190	88.0631	158.0924	79.5498			1

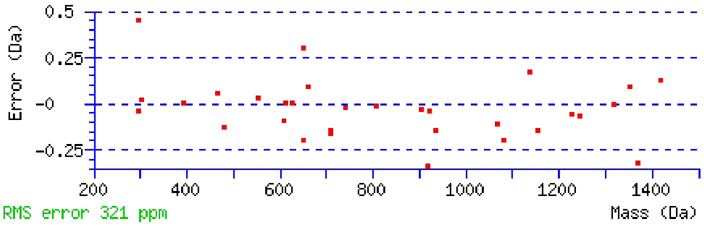

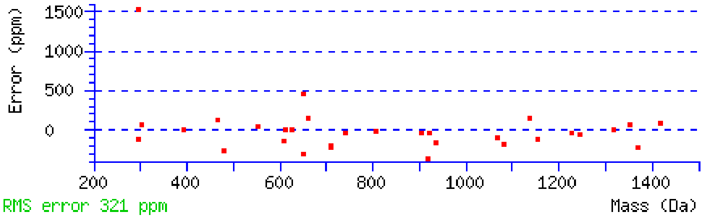

All matches to this query

Score	Mr(calc):	Delta	Sequence
77.1	1544.7260	0.0030	GAVYSFDPVGSYQR
11.4	1544.7364	-0.0075	KPATLSQIGTNKK
9.2	1543.7178	1.0112	ARSKSLVMGEQSR
5.6	1544.7388	-0.0099	YKTHVDTVLAYR
5.2	1544.7236	0.0054	SFQDSLEDIKKR
3.8	1544.7276	0.0014	LLTDDGNKWLYK
3.7	1544.7364	-0.0075	KPATLSQIGTNKK
3.3	1543.7330	0.9959	QATCFLGRSIGVR
3.2	1542.7249	2.0040	FCDRVQDAYTLR
2.3	1544.7357	-0.0067	KVVAVSMHPGPGMR

Spectrum No: 81; Query: 28291; Rank: 1

Peptide View

MS/MS Fragmentation of **ICIVTK**
Found in **IPI00025019**, Tax_Id=9606 Gene_Symbol=PSMB1 Proteasome subunit beta type-1

Match to Query 28291: 732.421328 from(367.217940,2+)
Title: 090702LimSK_Exosome2_06.1823.1823.2.dta
Data file C:\Temp\Mascoti\LSK_2D\090702LimSK_Exosome2_01_2D.mgf

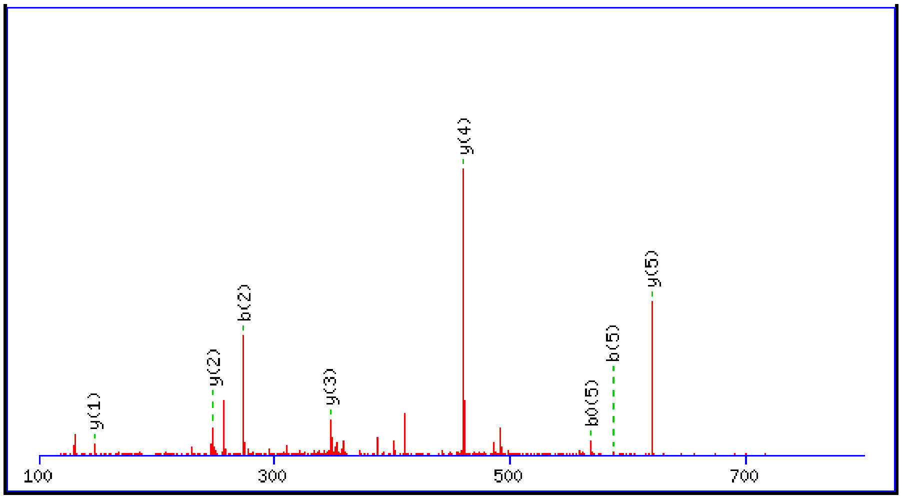

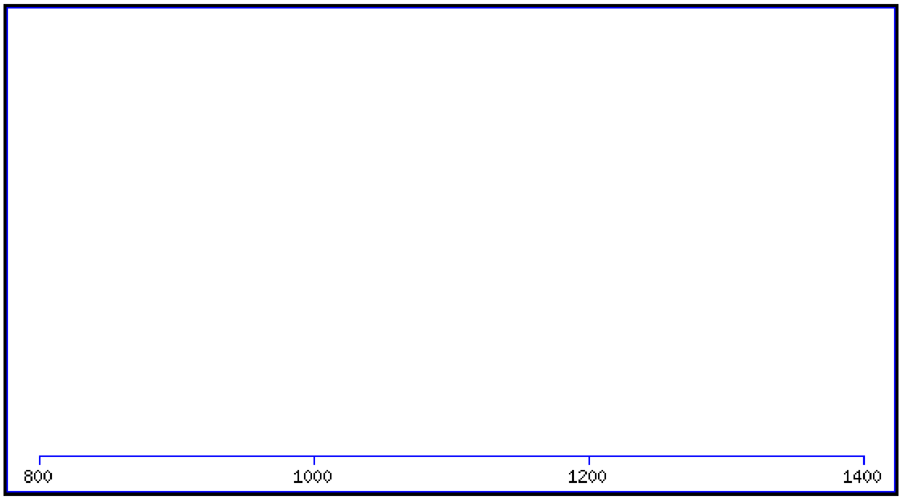

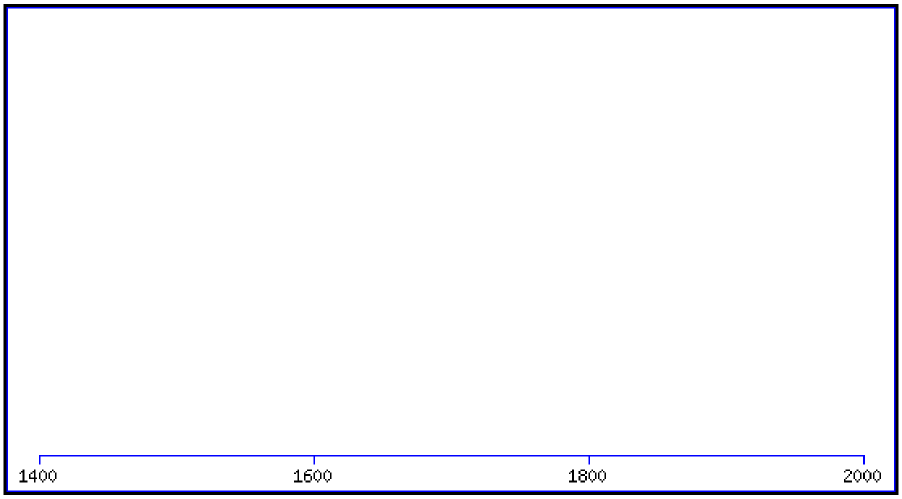

Monoisotopic mass of neutral peptide Mr(calc): 732.4204
Fixed modifications: Carbamidomethyl (C)
Ions Score: 33 **Expect:** 0.068
Matches (Bold Red): 8/40 fragment ions using 21 most intense peaks

#	b	b^{++}	b^0	b^{0++}	Seq.	y	y^{++}	y*	y^{*++}	y^0	y^{0++}	#
1	114.0913	57.5493			I							6
2	**274.1220**	137.5646			C	**620.3436**	310.6754	603.3171	302.1622	602.3330	301.6702	5
3	387.2061	194.1067			I	**460.3130**	230.6601	443.2864	222.1468	442.3024	221.6548	4
4	486.2745	243.6409			V	**347.2289**	174.1181	330.2023	165.6048	329.2183	165.1128	3
5	**587.3221**	294.1647	**569.3116**	285.1594	T	**248.1605**	124.5839	231.1339	116.0706	230.1499	115.5786	2
6					K	**147.1128**	74.0600	130.0863	65.5468			1

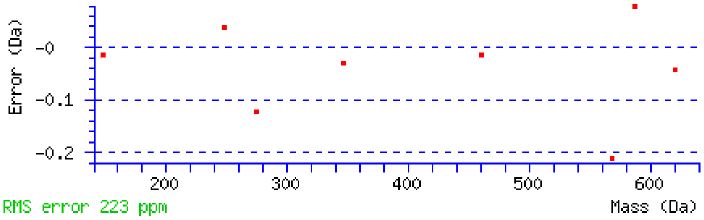

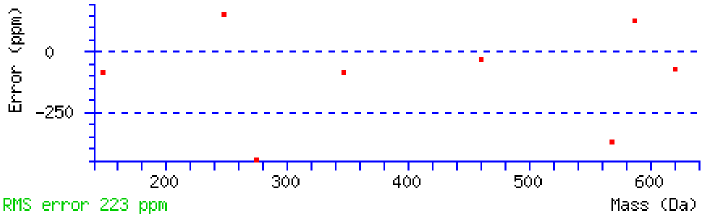

All matches to this query

Score	Mr(calc):	Delta	Sequence
32.8	732.4204	0.0009	ICIVTK
15.5	731.4178	1.0036	SGQLTVK
15.4	732.4283	-0.0069	QAFLVR
14.9	732.4204	0.0009	LKMPTK
14.9	732.4204	0.0009	LQIMTK
14.3	732.4283	-0.0069	LQAFVR
13.7	731.4178	1.0036	QSGIVTK
12.2	732.4204	0.0009	AMAVLTK
12.1	732.4170	0.0043	SWLSLK
11.9	731.4177	1.0036	XKEVTK

Spectrum No: 82; Query: 51927; Rank: 1

Peptide View

MS/MS Fragmentation of **LSEGFSIHTR**
Found in **IPI00025019**, Tax_Id=9606 Gene_Symbol=PSMB1 Proteasome subunit beta type-1

Match to Query 51927: 1146.589392 from(383.203740,3+)
Title: 090702LimSK_Exosome2_06.6594.6594.3.dta
Data file C:\Temp\Mascoti\LSK_2D\090702LimSK_Exosome2_01_2D.mgf

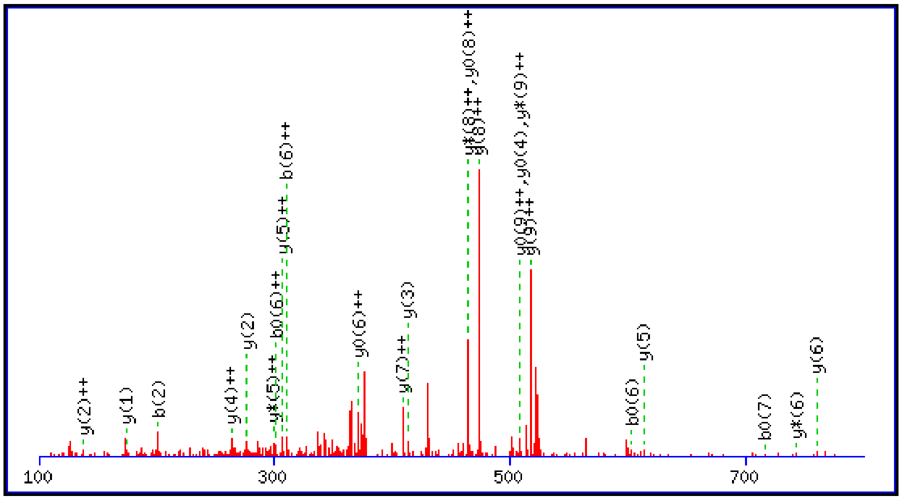

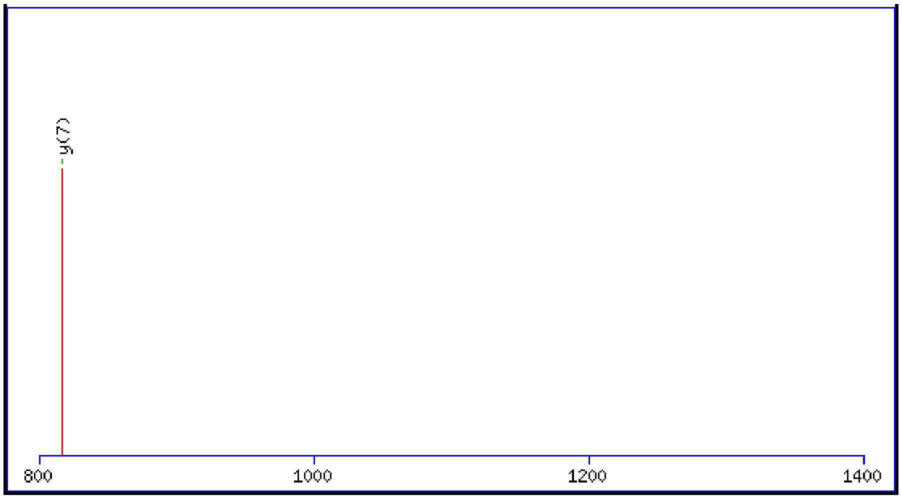

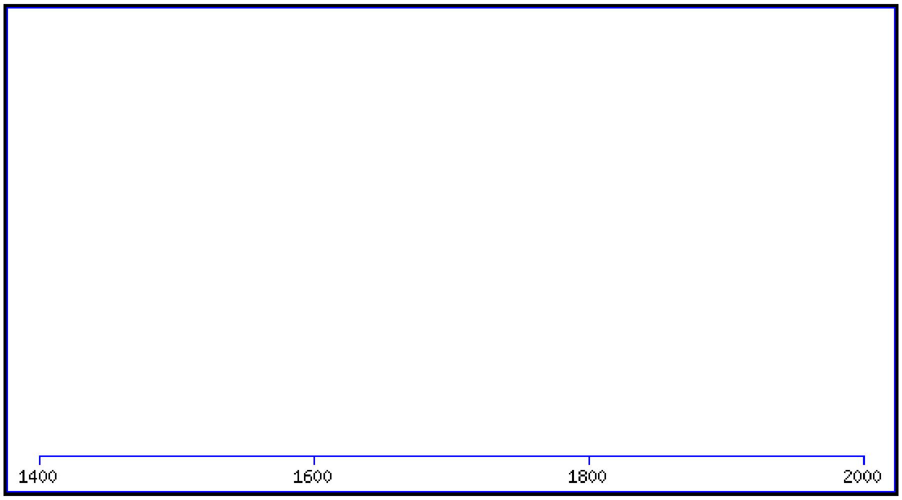

Monoisotopic mass of neutral peptide Mr(calc): 1145.5829
Fixed modifications: Carbamidomethyl (C)
Ions Score: 26 **Expect:** 0.37
Matches (Bold Red): 25/86 fragment ions using 63 most intense peaks

#	b	b^{++}	b^{0}	b^{0++}	Seq.	y	y^{++}	y*	y^{*++}	y^{0}	y^{0++}	#
1	114.0913	57.5493			L							10
2	**201.1234**	101.0653	183.1128	92.0600	S	1033.5061	**517.2567**	1016.4796	**508.7434**	1015.4956	**508.2514**	9
3	330.1660	165.5866	312.1554	156.5813	E	946.4741	**473.7407**	929.4476	**465.2274**	928.4635	**464.7354**	8
4	387.1874	194.0974	369.1769	185.0921	G	**817.4315**	**409.2194**	800.4050	400.7061	799.4209	400.2141	7
5	534.2558	267.6316	516.2453	258.6263	F	**760.4100**	380.7087	**743.3835**	372.1954	742.3995	**371.7034**	6
6	621.2879	**311.1476**	**603.2773**	**302.1423**	S	**613.3416**	**307.1745**	596.3151	**298.6612**	595.3311	298.1692	5
7	734.3719	367.6896	**716.3614**	358.6843	I	526.3096	**263.6584**	509.2831	255.1452	**508.2990**	254.6532	4
8	871.4308	436.2191	853.4203	427.2138	H	**413.2255**	207.1164	396.1990	198.6031	395.2150	198.1111	3
9	972.4785	486.7429	954.4680	477.7376	T	**276.1666**	**138.5870**	259.1401	130.0737	258.1561	129.5817	2
10					R	**175.1190**	88.0631	158.0924	79.5498			1

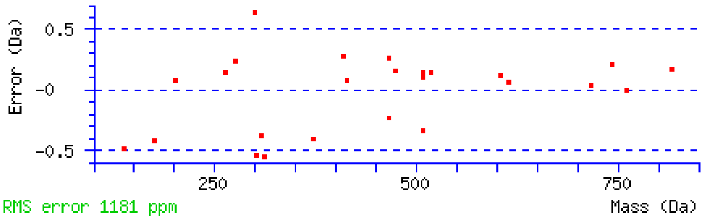

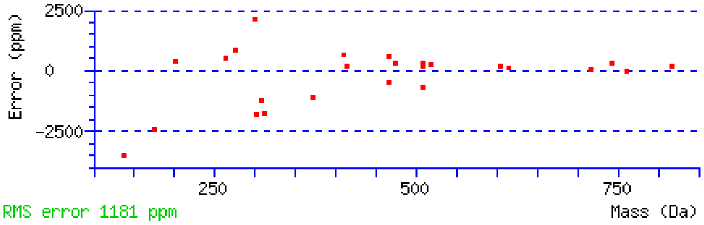

All matches to this query

Score	Mr(calc):	Delta	Sequence
26.4	1145.5829	1.0065	LSEGFSIHTR
10.3	1146.6006	-0.0112	NSSPRAFRGR
8.9	1146.5934	-0.0040	GFTAPSKHFR
7.1	1144.5798	2.0096	AMLSAELGPEK
5.5	1144.5754	2.0140	ISPGIRGTHK
5.1	1145.5869	1.0024	LQHFFTPEK
4.8	1145.5751	1.0143	VTMAVPEDLR
4.6	1144.5754	2.0140	TPSPARPALR
4.2	1144.5754	2.0140	VVLGAHNLSR
3.8	1146.5993	-0.0099	NSSELSVKQR

Spectrum No: 83; Query: 75489; Rank: 1

Peptide View

MS/MS Fragmentation of **NMQNVEHVPLSLDR**
Found in **IPI00025019**, Tax_Id=9606 Gene_Symbol=PSMB1 Proteasome subunit beta type-1

Match to Query 75489: 1650.817872 from(551.279900,3+)
Title: 090702LimSK_Exosome2_06.8377.8377.3.dta
Data file C:\Temp\Mascoti\LSK_2D\090702LimSK_Exosome2_01_2D.mgf

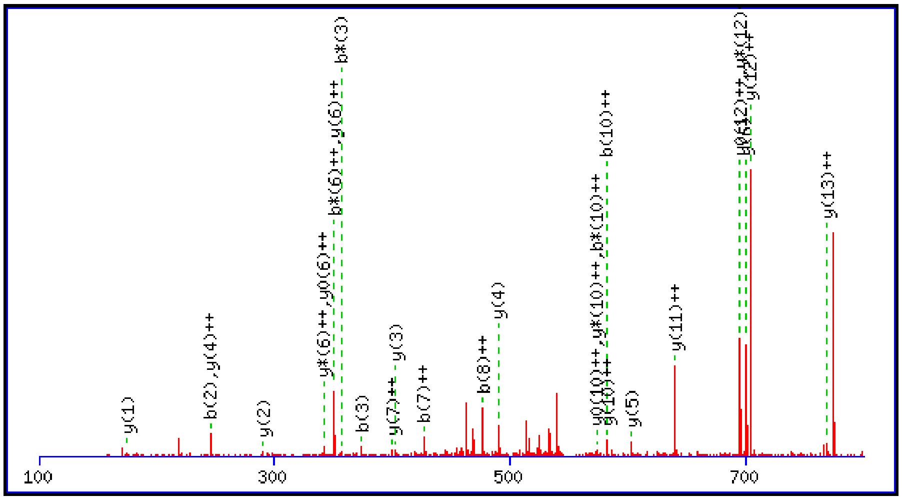

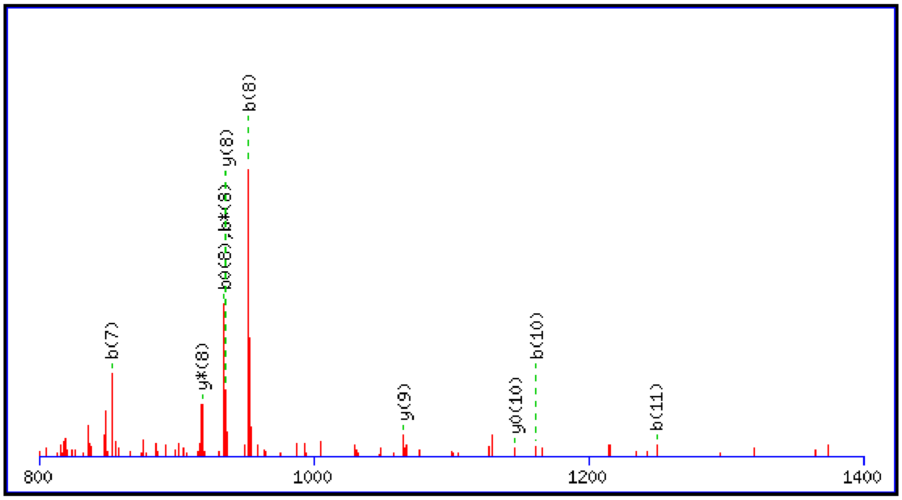

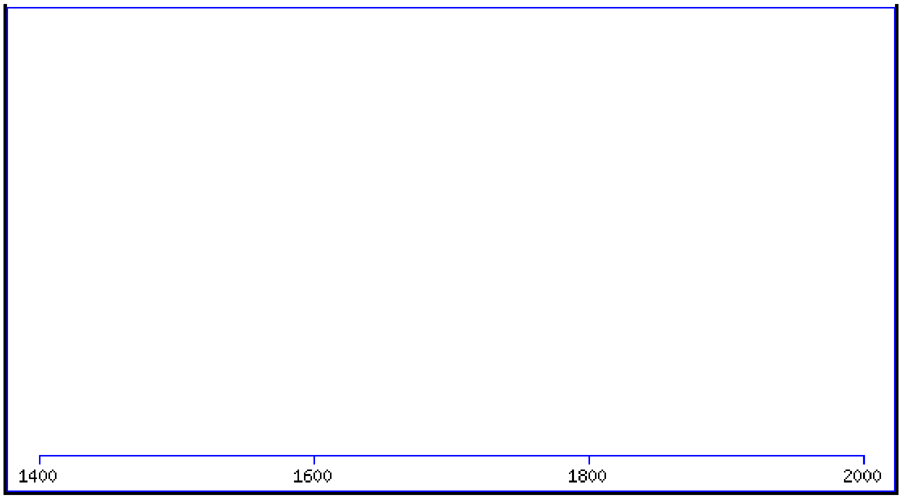

Monoisotopic mass of neutral peptide Mr(calc): 1650.8148
Fixed modifications: Carbamidomethyl (C)
Ions Score: 57 **Expect:** 0.00045
Matches (Bold Red): 37/144 fragment ions using 63 most intense peaks

#	b	b++	b*	b*++	b0	b0++	Seq.	y	y++	y*	y*++	y0	y0++	#
1	115.0502	58.0287	98.0237	49.5155			N							14
2	**246.0907**	123.5490	229.0641	115.0357			M	1537.7791	**769.3932**	1520.7526	760.8799	1519.7686	760.3879	13
3	**374.1493**	187.5783	**357.1227**	179.0650			Q	1406.7387	**703.8730**	1389.7121	**695.3597**	1388.7281	**694.8677**	12
4	488.1922	244.5997	471.1656	236.0865			N	1278.6801	**639.8437**	1261.6535	631.3304	1260.6695	630.8384	11
5	587.2606	294.1339	570.2341	285.6207			V	1164.6371	**582.8222**	1147.6106	**574.3089**	**1146.6266**	**573.8169**	10
6	716.3032	358.6552	699.2767	**350.1420**	698.2926	349.6500	E	**1065.5687**	533.2880	1048.5422	524.7747	1047.5582	524.2827	9
7	**853.3621**	**427.1847**	836.3356	418.6714	835.3515	418.1794	H	**936.5261**	468.7667	**919.4996**	460.2534	918.5156	459.7614	8
8	**952.4305**	**476.7189**	**935.4040**	468.2056	**934.4200**	467.7136	V	799.4672	**400.2373**	782.4407	391.7240	781.4567	391.2320	7
9	1049.4833	525.2453	1032.4567	516.7320	1031.4727	516.2400	P	**700.3988**	**350.7030**	683.3723	**342.1898**	682.3882	**341.6978**	6
10	**1162.5674**	**581.7873**	1145.5408	**573.2740**	1144.5568	572.7820	L	**603.3461**	302.1767	586.3195	293.6634	585.3355	293.1714	5
11	**1249.5994**	625.3033	1232.5728	616.7901	1231.5888	616.2980	S	**490.2620**	**245.6346**	473.2354	237.1214	472.2514	236.6293	4
12	1362.6834	681.8454	1345.6569	673.3321	1344.6729	672.8401	L	**403.2300**	202.1186	386.2034	193.6053	385.2194	193.1133	3
13	1477.7104	739.3588	1460.6838	730.8456	1459.6998	730.3535	D	**290.1459**	145.5766	273.1193	137.0633	272.1353	136.5713	2
14							R	**175.1190**	88.0631	158.0924	79.5498			1

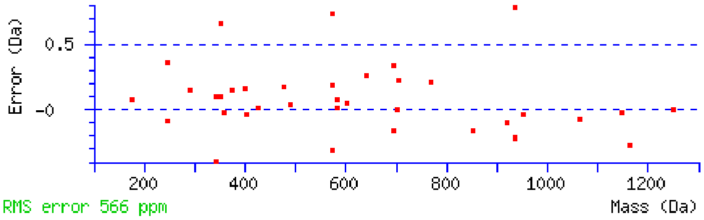

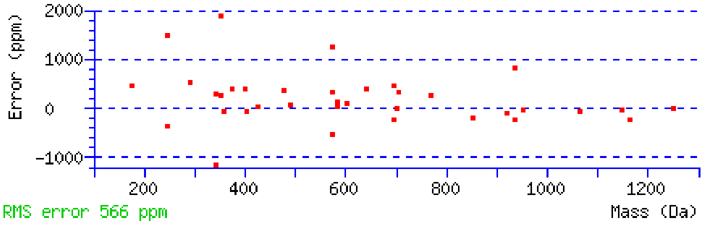

All matches to this query

Score	Mr(calc):	Delta	Sequence
56.6	1650.8148	0.0031	NMQNVEHVPLSLDR
15.3	1648.8169	2.0010	LKIDFGDSARADDAR
11.2	1649.8195	0.9983	SASTAGDIACAFRPVK
11.0	1650.8164	0.0014	LHKICSDKTITQK
11.0	1650.8164	0.0014	LHKICSDKTITQK
8.9	1650.8052	0.0127	MPKVDLKGPQTDVK
8.3	1649.8066	1.0113	FSSPSEIQGIALPPK
8.1	1648.8259	1.9920	EPKSVCKAPELLAK

7.7	1649.8212	0.9967	TMLLLSRSPEAQPK
7.7	1649.8212	0.9967	TMLLLSRSPEAQPK

Spectrum No: 84; Query: 29884; Rank: 1

Peptide View

MS/MS Fragmentation of **IFCCR**
Found in **IPI00000811**, Tax_Id=9606 Gene_Symbol=PSMB6 Proteasome subunit beta type-6

Match to Query 29884: 754.326648 from(378.170600,2+)
Title: 090702LimSK_Exosome2_06.1328.1328.2.dta
Data file C:\Temp\Mascoti\LSK_2D\090702LimSK_Exosome2_01_2D.mgf

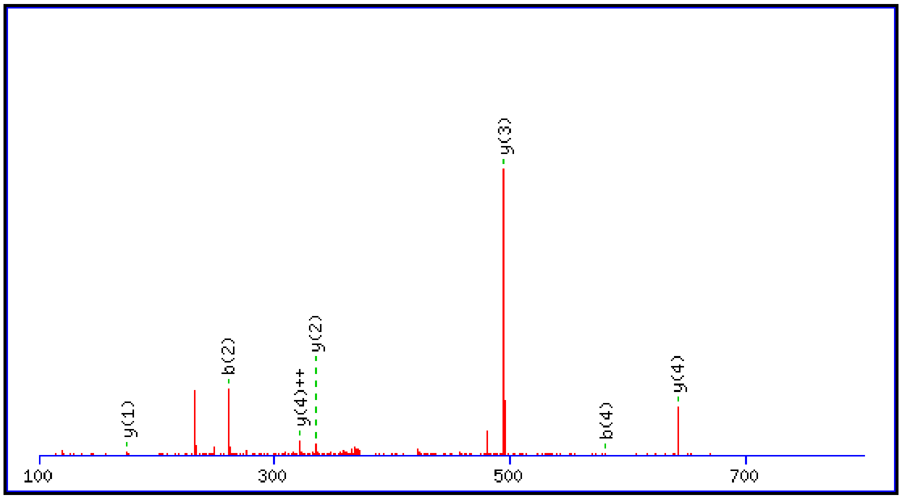

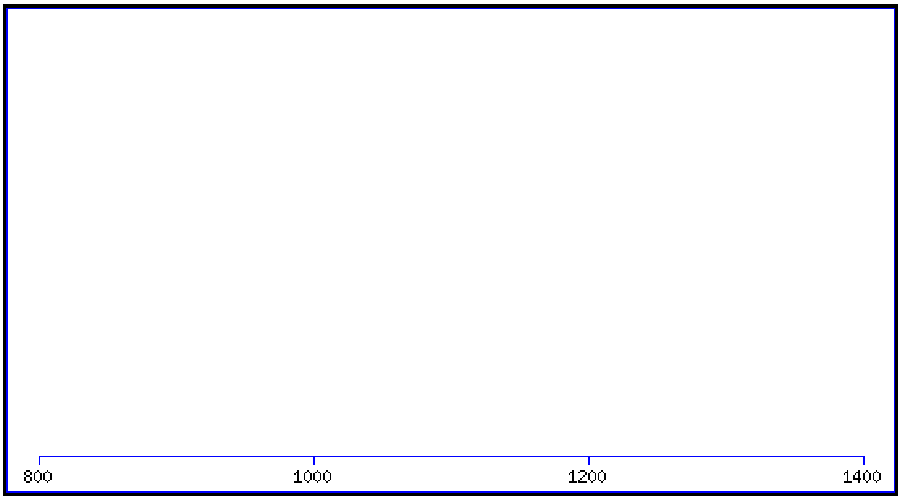

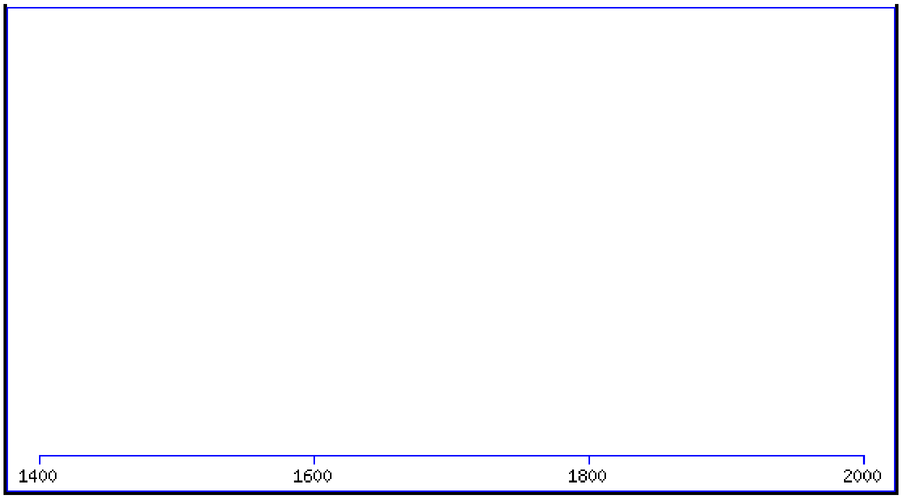

Monoisotopic mass of neutral peptide Mr(calc): 754.3255
Fixed modifications: Carbamidomethyl (C)
Ions Score: 25 **Expect:** 0.23
Matches (Bold Red): 7/24 fragment ions using 15 most intense peaks

#	b	b^{++}	Seq.	y	y^{++}	y*	y^{*++}	#
1	114.0913	57.5493	I					5
2	**261.1598**	131.0835	F	**642.2487**	**321.6280**	625.2221	313.1147	4
3	421.1904	211.0988	C	**495.1803**	248.0938	478.1537	239.5805	3
4	**581.2211**	291.1142	C	**335.1496**	168.0784	318.1231	159.5652	2
5			R	**175.1190**	88.0631	158.0924	79.5498	1

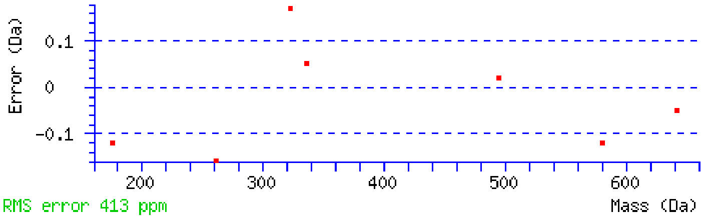

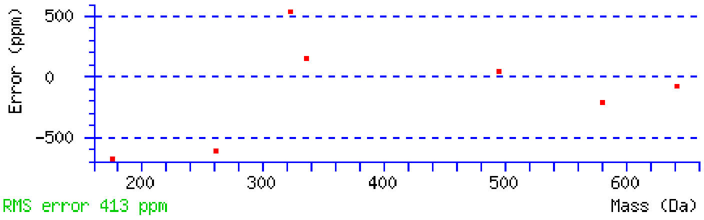

All matches to this query

Score	Mr(calc):	Delta	Sequence
25.2	754.3255	0.0012	IFCCR
25.2	754.3255	0.0012	LFCCR
12.2	754.3262	0.0004	LETANK
10.3	754.3262	0.0004	LDSVNK
5.0	753.3171	1.0096	GGAGGSLR
4.8	754.3262	0.0004	DIVSNK
4.5	752.3197	2.0070	QEMMSK
3.0	753.3228	1.0038	AGGSCFR
2.8	753.3171	1.0096	ANTVGGR
1.6	752.3218	2.0048	ASVSPGR

Spectrum No: 85; Query: 49045; Rank: 1

Peptide View

MS/MS Fragmentation of **LAAIAESGVER**
Found in **IPI00000811**, Tax_Id=9606 Gene_Symbol=PSMB6 Proteasome subunit beta type-6

Match to Query 49045: 1114.599008 from(558.306780,2+)
Title: 090702LimSK_Exosome2_06.2667.2667.2.dta
Data file C:\Temp\Mascoti\LSK_2D\090702LimSK_Exosome2_01_2D.mgf

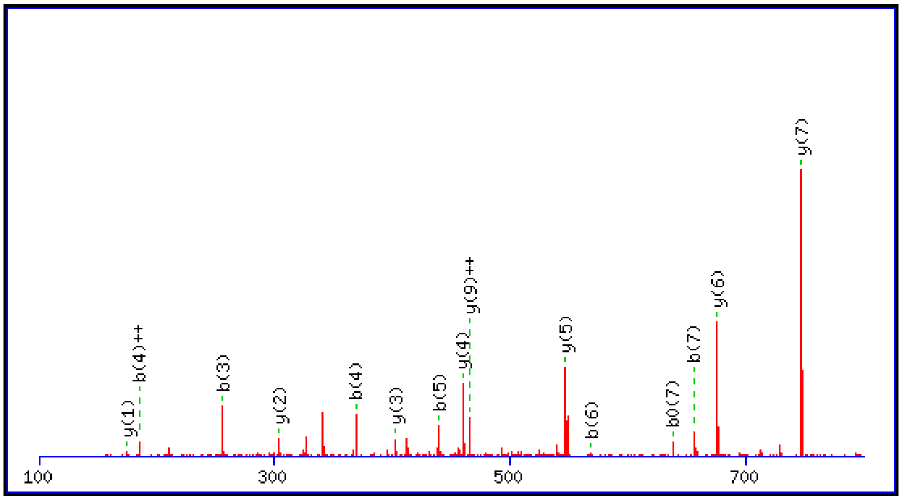

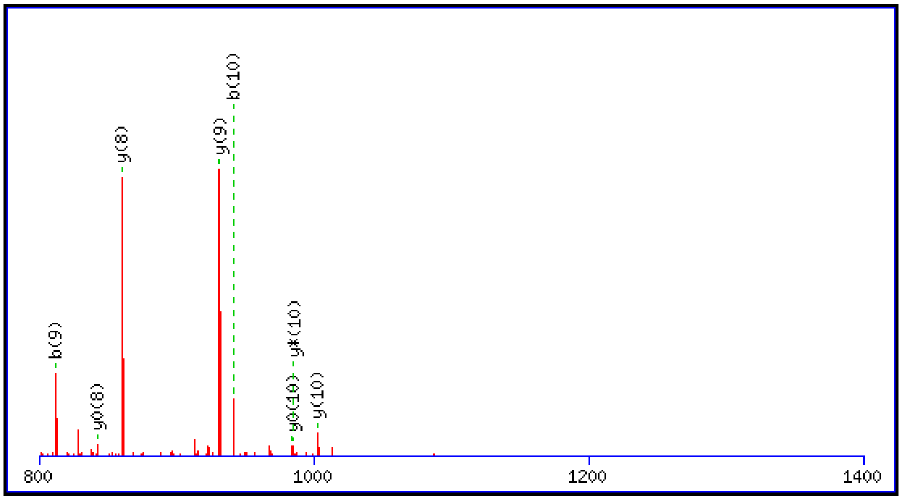

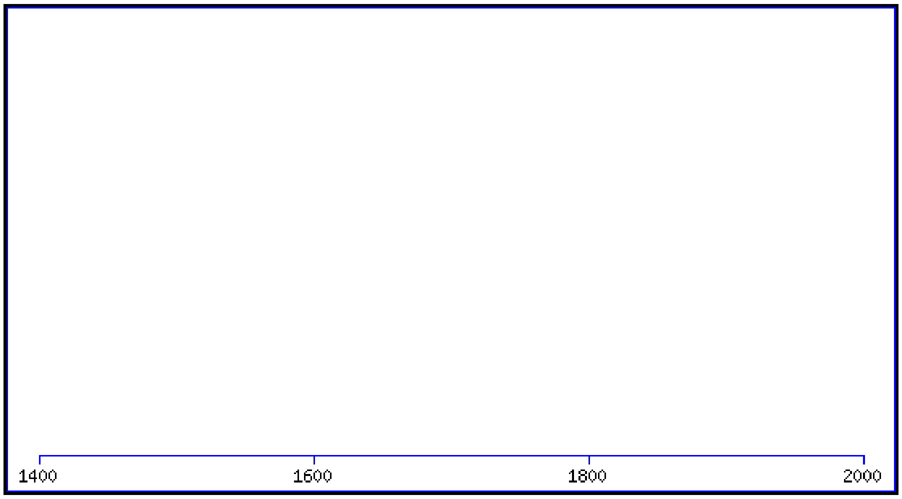

Monoisotopic mass of neutral peptide Mr(calc): 1114.5982
Fixed modifications: Carbamidomethyl (C)
Ions Score: 80 **Expect:** 1.4e-006
Matches (Bold Red): 24/88 fragment ions using 41 most intense peaks

#	b	b^{++}	b^0	b^{0++}	Seq.	y	y^{++}	y*	y^{*++}	y^0	y^{0++}	#
1	114.0913	57.5493			L							11
2	**185.1285**	93.0679			A	**1002.5215**	501.7644	**985.4949**	493.2511	**984.5109**	492.7591	10
3	**256.1656**	128.5864			A	**931.4843**	**466.2458**	914.4578	457.7325	913.4738	457.2405	9

4	369.2496	185.1285			I	860.4472	430.7272	843.4207	422.2140	842.4367	421.7220	8
5	440.2867	220.6470			A	747.3632	374.1852	730.3366	365.6719	729.3526	365.1799	7
6	569.3293	285.1683	551.3188	276.1630	E	676.3260	338.6667	659.2995	330.1534	658.3155	329.6614	6
7	656.3614	328.6843	638.3508	319.6790	S	547.2835	274.1454	530.2569	265.6321	529.2729	265.1401	5
8	713.3828	357.1951	695.3723	348.1898	G	460.2514	230.6293	443.2249	222.1161	442.2409	221.6241	4
9	812.4512	406.7293	794.4407	397.7240	V	403.2300	202.1186	386.2034	193.6053	385.2194	193.1133	3
10	941.4938	471.2506	923.4833	462.2453	E	304.1615	152.5844	287.1350	144.0711	286.1510	143.5791	2
11					R	175.1190	88.0631	158.0924	79.5498			1

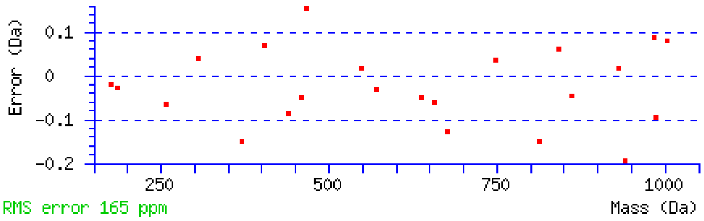

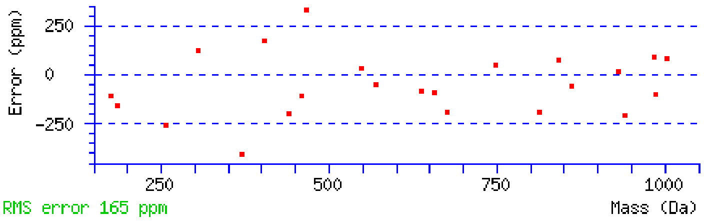

All matches to this query

Score	Mr(calc):	Delta	Sequence
79.5	1114.5982	0.0008	LAAIAESGVER
15.2	1114.5883	0.0107	LAVGFSTHQR
7.9	1114.6095	-0.0104	LAPSSDLRTR
4.3	1114.6094	-0.0104	ALDEKVRER
3.1	1114.6094	-0.0104	LDINLERSR
3.0	1114.5982	0.0008	LAKQEELER
2.7	1114.5883	0.0107	EVARLWGER
1.5	1112.5979	2.0012	TKAHVSSVVW
1.2	1114.6095	-0.0104	ILNVSNKGDR
0.8	1114.5957	0.0033	KMSIWTPPR

Spectrum No: 86; Query: 48747; Rank: 1

Peptide View

MS/MS Fragmentation of **QVLLGDQIPK**
Found in **IPI00000811**, Tax_Id=9606 Gene_Symbol=PSMB6 Proteasome subunit beta type-6

Match to Query 48747: 1109.645748 from(555.830150,2+)
Title: 090702LimSK_Exosome2_06.4875.4875.2.dta
Data file C:\Temp\Mascoti\LSK_2D\090702LimSK_Exosome2_01_2D.mgf

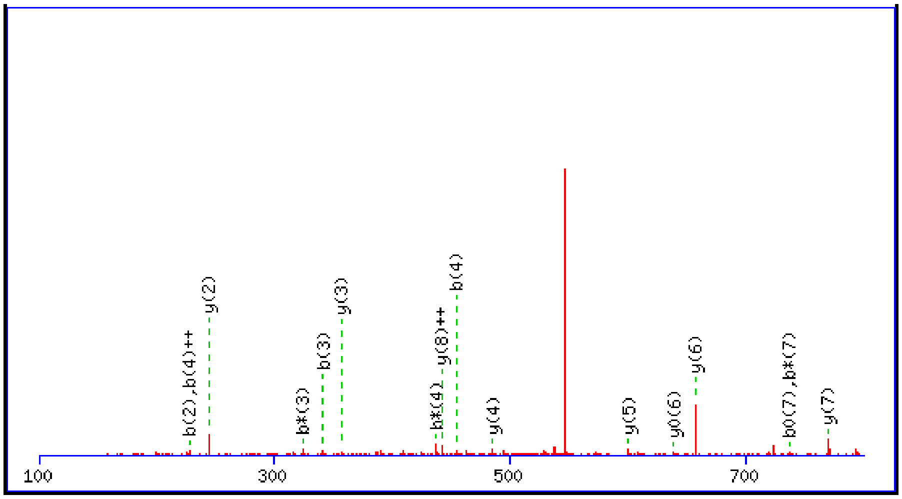

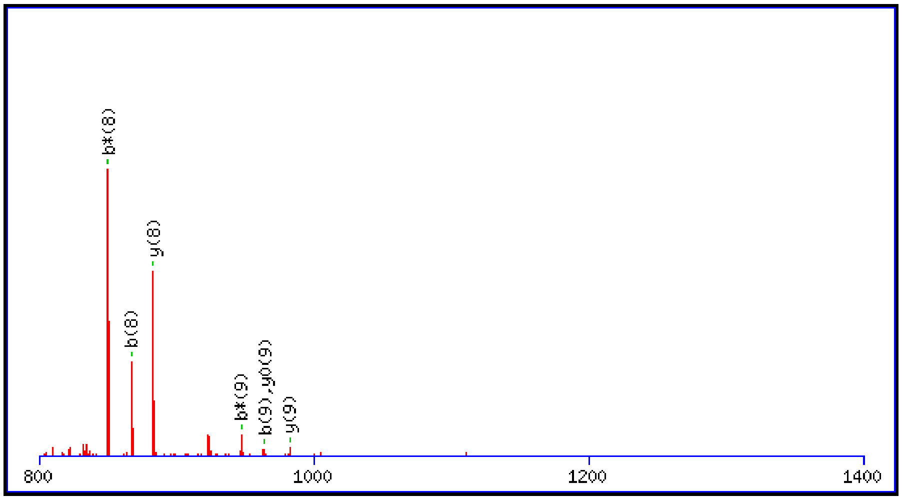

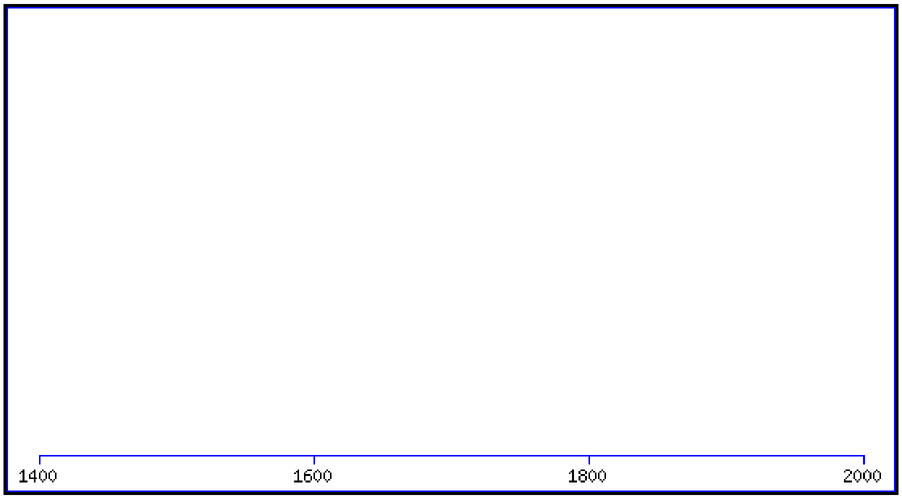

Monoisotopic mass of neutral peptide Mr(calc): 1109.6445
Fixed modifications: Carbamidomethyl (C)
Ions Score: 58 **Expect:** 7.7e-005
Matches (Bold Red): 23/90 fragment ions using 39 most intense peaks

#	b	b^{++}	b^{*}	b^{*++}	b^{0}	b^{0++}	Seq.	y	y^{++}	y^{*}	y^{*++}	y^{0}	y^{0++}	#
1	129.0659	65.0366	112.0393	56.5233			Q							10
2	**228.1343**	114.5708	211.1077	106.0575			V	**982.5932**	491.8002	965.5666	483.2869	**964.5826**	482.7949	9
3	**341.2183**	171.1128	**324.1918**	162.5995			L	**883.5247**	**442.2660**	866.4982	433.7527	865.5142	433.2607	8
4	**454.3024**	**227.6548**	**437.2758**	219.1416			L	**770.4407**	385.7240	753.4141	377.2107	752.4301	376.7187	7
5	511.3239	256.1656	494.2973	247.6523			G	**657.3566**	329.1819	640.3301	320.6687	**639.3461**	320.1767	6
6	626.3508	313.6790	609.3243	305.1658	608.3402	304.6738	D	**600.3352**	300.6712	583.3086	292.1579	582.3246	291.6659	5

**7**	754.4094	377.7083	**737.3828**	369.1951	**736.3988**	368.7030	**Q**	**485.3082**	243.1577	468.2817	234.6445			**4**
**8**	**867.4934**	434.2504	**850.4669**	425.7371	849.4829	425.2451	**I**	**357.2496**	179.1285	340.2231	170.6152			**3**
**9**	**964.5462**	482.7767	**947.5197**	474.2635	946.5356	473.7715	**P**	**244.1656**	122.5864	227.1390	114.0731			**2**
**10**							**K**	147.1128	74.0600	130.0863	65.5468			**1**

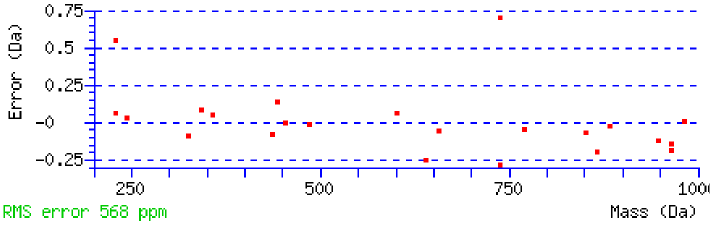

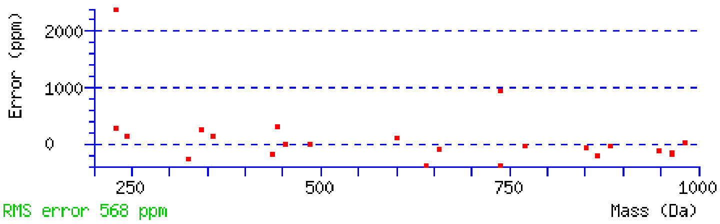

All matches to this query

Score	Mr(calc):	Delta	Sequence
57.6	1109.6445	0.0013	QVLLGDQIPK
14.1	1109.6557	-0.0099	RAIPANVEIK
11.4	1109.6557	-0.0099	LNREQLLPK
11.2	1109.6444	0.0013	DKDGKPLLPK
10.9	1109.6557	-0.0099	RALEQVPGLK
7.9	1109.6557	-0.0099	LLIRENQPK
7.8	1109.6444	0.0013	LDKIGLNLPAG
7.3	1108.6492	0.9965	ADVVPVTLAPK
6.3	1108.6353	1.0105	DKHINNLKK
6.0	1109.6557	-0.0099	GGLQPAVLSLR

Spectrum No: 87; Query: 46929; Rank: 1

Peptide View

MS/MS Fragmentation of **TTTGSYIANR**
Found in **IPI00000811**, Tax_Id=9606 Gene_Symbol=PSMB6 Proteasome subunit beta type-6

Match to Query 46929: 1082.536988 from(542.275770,2+)
Title: 090702LimSK_Exosome2_08b1.1084.1084.2.dta
Data file C:\Temp\Mascoti\LSK_2D\090702LimSK_Exosome2_01_2D.mgf

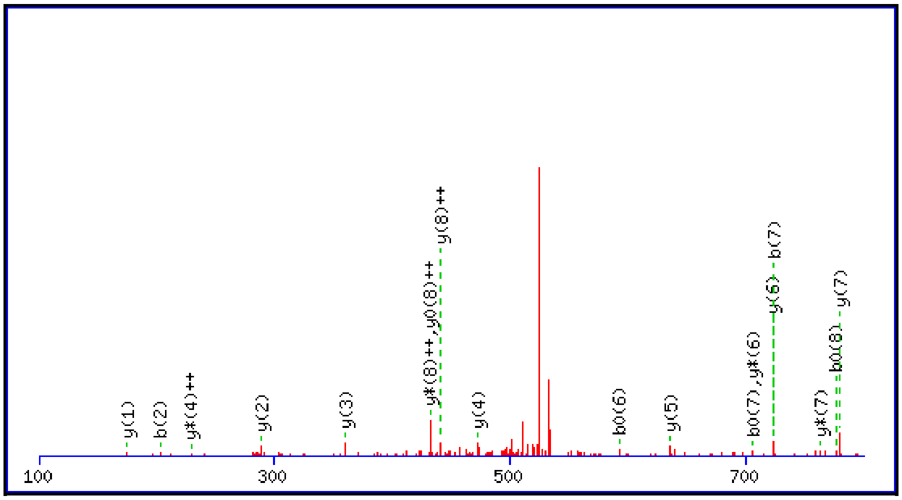

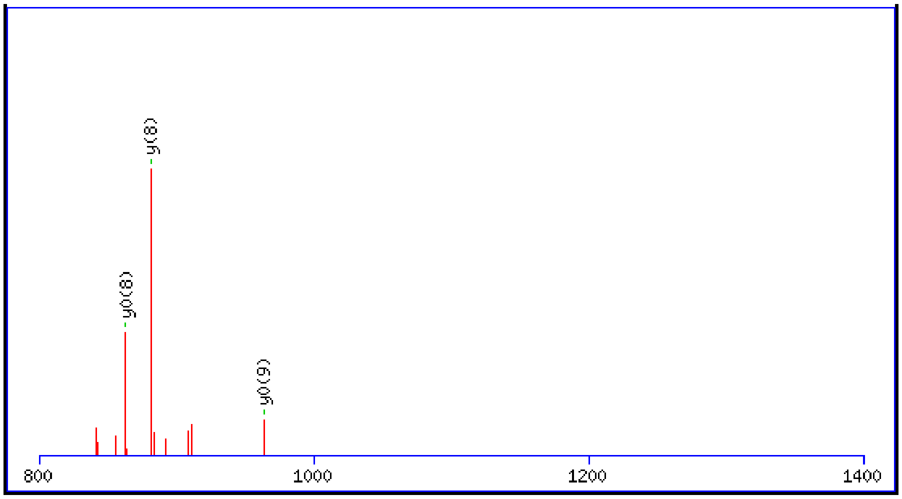

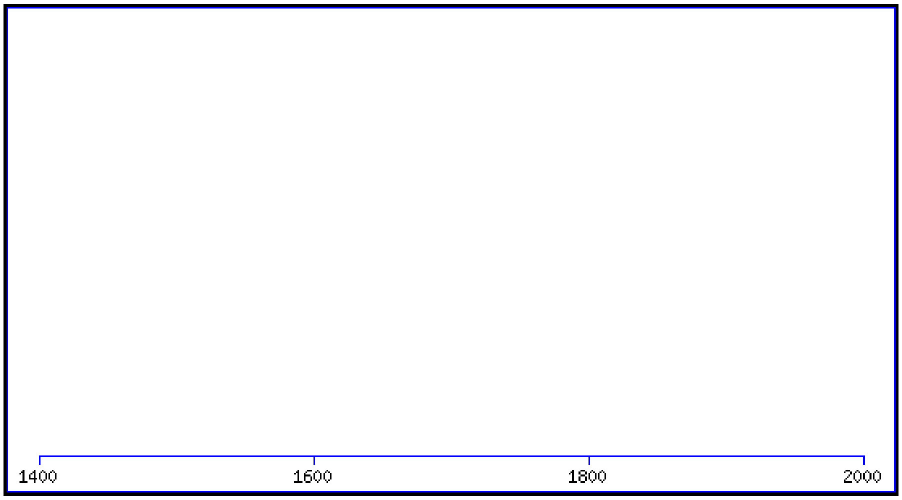

Monoisotopic mass of neutral peptide Mr(calc): 1082.5356
Fixed modifications: Carbamidomethyl (C)
Ions Score: 74 **Expect:** 5.8e-006
Matches (Bold Red): 21/82 fragment ions using 30 most intense peaks

#	b	b^{++}	b*	b^{*++}	b^0	b^{0++}	Seq.	y	y^{++}	y*	y^{*++}	y^0	y^{0++}	#
1	102.0550	51.5311			84.0444	42.5258	T							10
2	**203.1026**	102.0550			185.0921	93.0497	T	982.4952	491.7513	965.4687	483.2380	**964.4847**	482.7460	9
3	304.1503	152.5788			286.1397	143.5735	T	**881.4476**	**441.2274**	864.4210	**432.7141**	**863.4370**	**432.2221**	8
4	361.1718	181.0895			343.1612	172.0842	G	**780.3999**	390.7036	**763.3733**	382.1903	762.3893	381.6983	7
5	448.2038	224.6055			430.1932	215.6003	S	**723.3784**	362.1928	**706.3519**	353.6796	705.3678	353.1876	6
6	611.2671	306.1372			**593.2566**	297.1319	Y	**636.3464**	318.6768	619.3198	310.1636			5
7	**724.3512**	362.6792			**706.3406**	353.6740	I	**473.2831**	237.1452	456.2565	**228.6319**			4
8	795.3883	398.1978			**777.3777**	389.1925	A	**360.1990**	180.6031	343.1724	172.0899			3
9	909.4312	455.2193	892.4047	446.7060	891.4207	446.2140	N	**289.1619**	145.0846	272.1353	136.5713			2
10							R	**175.1190**	88.0631	158.0924	79.5498			1

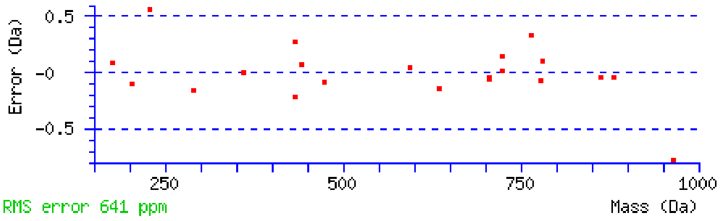

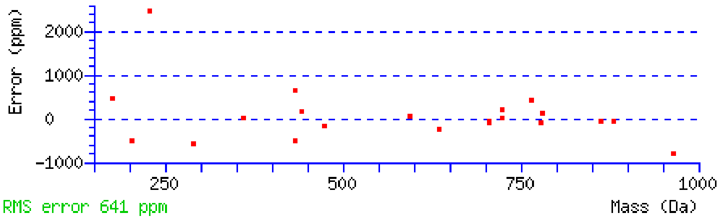

All matches to this query

Score	Mr(calc):	Delta	Sequence
74.1	1082.5356	0.0014	TTTGSYIANR
16.5	1082.5468	-0.0099	NSSLIQHER
14.7	1081.5355	1.0015	MAGTGLLALR
13.9	1080.5274	2.0096	MAASAPPPPDK
11.4	1081.5281	1.0089	SISRPSSLR
11.4	1081.5281	1.0089	SISRPSSLR
10.5	1082.5469	-0.0099	DSQLSLHQR
10.4	1082.5468	-0.0099	SSNLIEHQR
10.3	1081.5281	1.0089	SISRPSSLR
9.2	1081.5338	1.0031	SLRYCDLR

Spectrum No: 88; Query: 60094; Rank: 1

Peptide View

MS/MS Fragmentation of **VTDKLTPIHDR**
Found in **IPI00000811**, Tax_Id=9606 Gene_Symbol=PSMB6 Proteasome subunit beta type-6

Match to Query 60094: 1295.713902 from(432.911910,3+)
Title: 090702LimSK_Exosome2_07.6632.6632.3.dta
Data file C:\Temp\Mascoti\LSK_2D\090702LimSK_Exosome2_01_2D.mgf

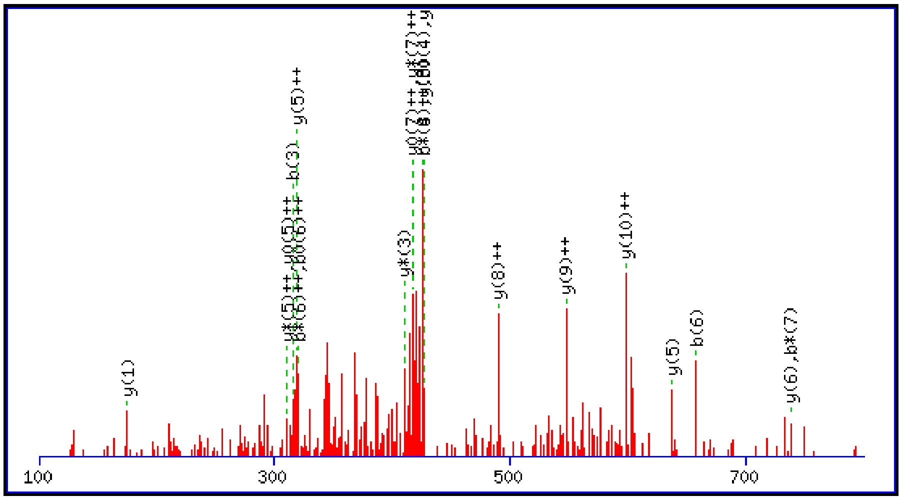

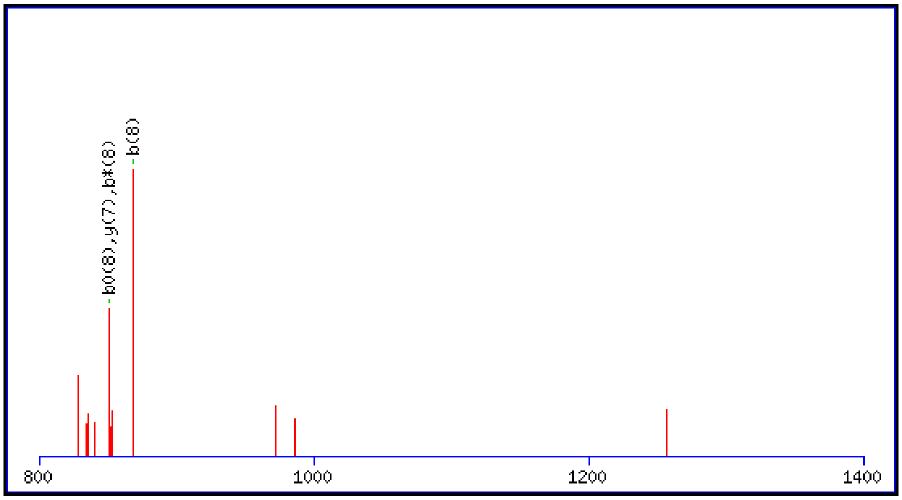

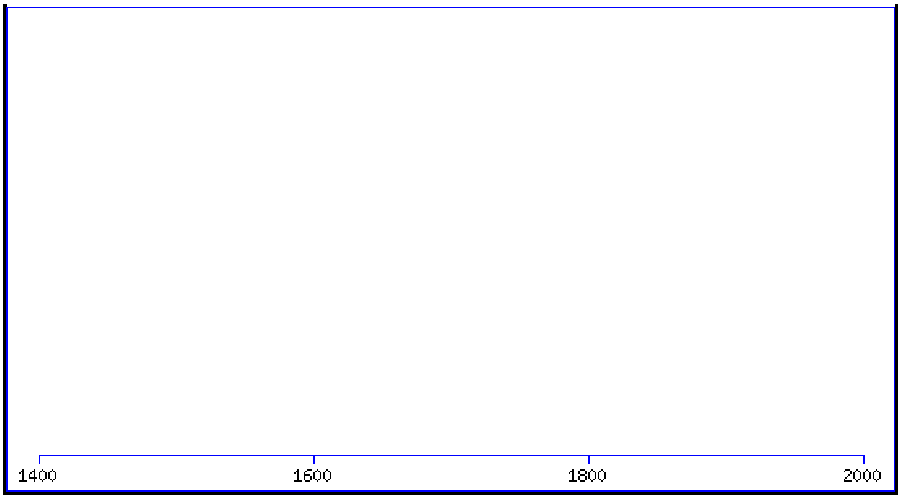

Monoisotopic mass of neutral peptide Mr(calc): 1293.7041
Fixed modifications: Carbamidomethyl (C)
Ions Score: 43 **Expect:** 0.0048
Matches (Bold Red): 27/110 fragment ions using 33 most intense peaks

#	b	b^{++}	b*	b^{*++}	b^{0}	b^{0++}	Seq.	y	y^{++}	y*	y^{*++}	y^{0}	y^{0++}	#
1	100.0757	50.5415					V							11
2	201.1234	101.0653			183.1128	92.0600	T	1195.6430	**598.3251**	1178.6164	589.8118	1177.6324	589.3198	10
3	**316.1503**	158.5788			298.1397	149.5735	D	1094.5953	**547.8013**	1077.5687	539.2880	1076.5847	538.7960	9
4	444.2453	222.6263	**427.2187**	214.1130	**426.2347**	213.6210	K	979.5683	**490.2878**	962.5418	481.7745	961.5578	481.2825	8
5	557.3293	279.1683	540.3028	270.6550	539.3188	270.1630	L	**851.4734**	**426.2403**	834.4468	**417.7271**	833.4628	**417.2350**	7
6	**658.3770**	329.6921	641.3505	**321.1789**	640.3665	**320.6869**	T	**738.3893**	369.6983	721.3628	361.1850	720.3787	360.6930	6
7	755.4298	378.2185	**738.4032**	369.7053	737.4192	369.2132	P	**637.3416**	**319.1745**	620.3151	**310.6612**	619.3311	**310.1692**	5
8	**868.5138**	434.7606	**851.4873**	**426.2473**	**850.5033**	**425.7553**	I	540.2889	270.6481	523.2623	262.1348	522.2783	261.6428	4
9	1005.5728	503.2900	988.5462	494.7767	987.5622	494.2847	H	**427.2048**	214.1060	**410.1783**	205.5928	409.1942	205.1008	3
10	1120.5997	560.8035	1103.5732	552.2902	1102.5891	551.7982	D	290.1459	145.5766	273.1193	137.0633	272.1353	136.5713	2
11							R	**175.1190**	88.0631	158.0924	79.5498			1

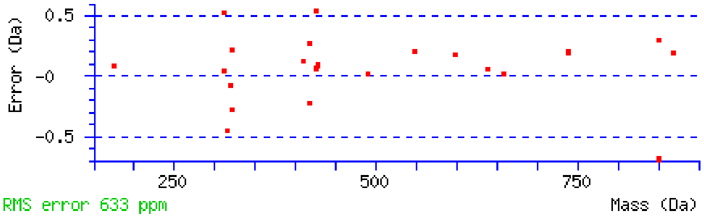

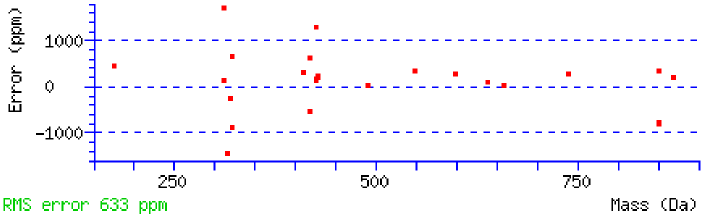

All matches to this query

Score	Mr(calc):	Delta	Sequence
42.7	1293.7041	2.0098	VTDKLTPIHDR
16.2	1294.7122	1.0017	LKSVRLLSGSR
13.5	1294.7179	0.9960	YMKSQTILRR
11.1	1294.7067	1.0072	VMTLITYNKGR
9.1	1294.7162	0.9977	ISFLPLTRLR
9.0	1293.7169	1.9970	VRALQSTLKAK
8.1	1295.7197	-0.0058	NPPINTKSQAVK
7.1	1293.7071	2.0068	XWTLTLLRR
6.3	1294.7122	1.0017	LKSVRLLSGSR
5.7	1293.7153	1.9986	SHALQLPKTSGR

Spectrum No: 89; Query: 26697; Rank: 1

Peptide View

MS/MS Fragmentation of **AVEILR**
Found in **IPI00291839**, Tax_Id=9606 Gene_Symbol=RAPGEF6 Isoform 2 of Rap guanine nucleotide exchange factor 6

Match to Query 26697: 699.428828 from(350.721690,2+)
Title: 090702LimSK_Exosome2_06.2732.2732.2.dta
Data file C:\Temp\Mascoti\LSK_2D\090702LimSK_Exosome2_01_2D.mgf

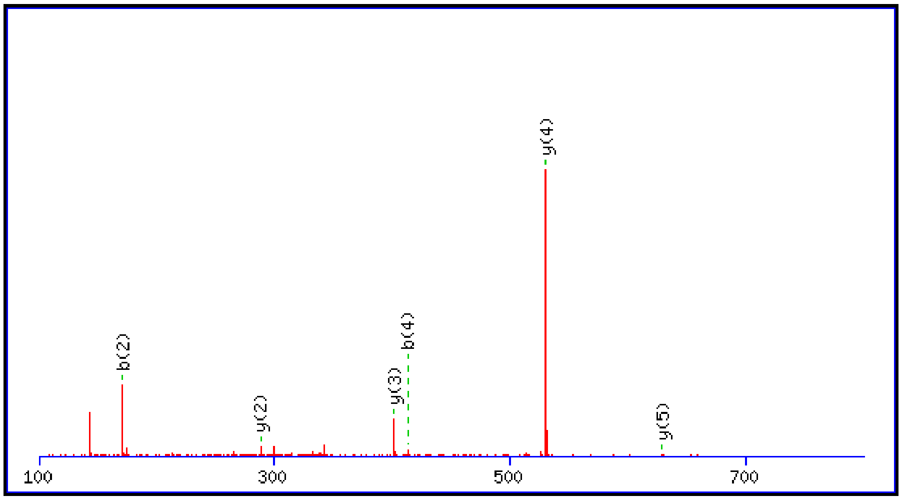

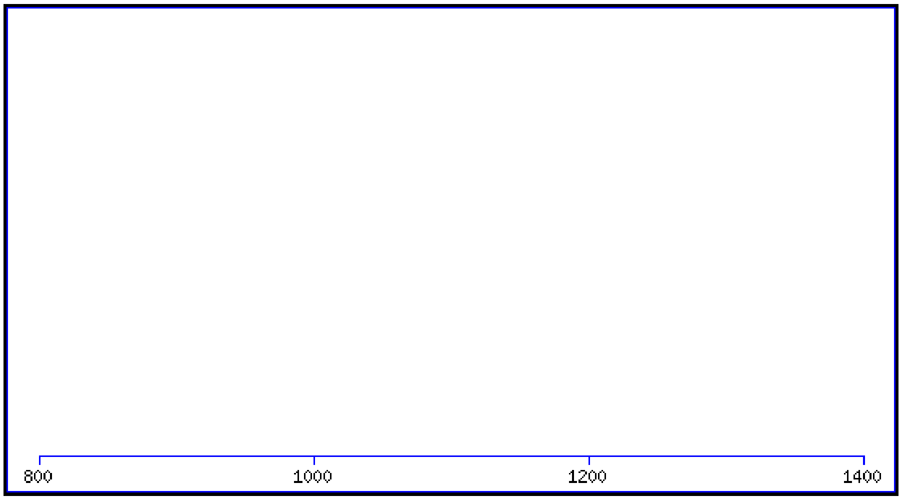

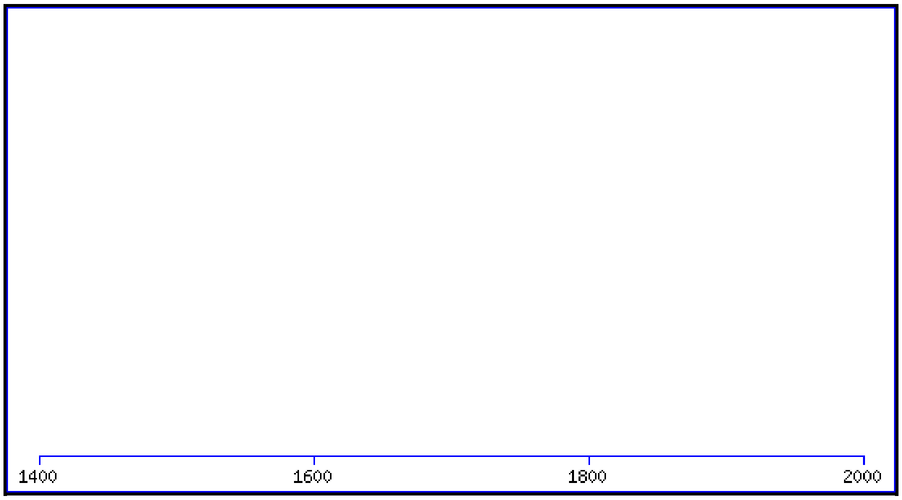

Monoisotopic mass of neutral peptide Mr(calc): 699.4279

Fixed modifications: Carbamidomethyl (C)
Ions Score: 43 **Expect:** 0.0028
Matches (Bold Red): 6/40 fragment ions using 8 most intense peaks

#	b	b^{++}	b^{0}	b^{0++}	Seq.	y	y^{++}	y*	y^{*++}	y^{0}	y^{0++}	#
1	72.0444	36.5258			A							6
2	**171.1128**	86.0600			V	**629.3981**	315.2027	612.3715	306.6894	611.3875	306.1974	5
3	300.1554	150.5813	282.1448	141.5761	E	**530.3297**	265.6685	513.3031	257.1552	512.3191	256.6632	4
4	**413.2395**	207.1234	395.2289	198.1181	I	**401.2871**	201.1472	384.2605	192.6339			3
5	526.3235	263.6654	508.3130	254.6601	L	**288.2030**	144.6051	271.1765	136.0919			2
6					R	175.1190	88.0631	158.0924	79.5498			1

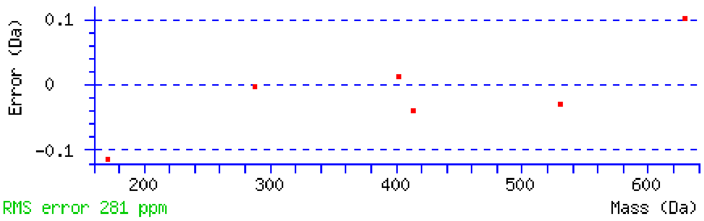

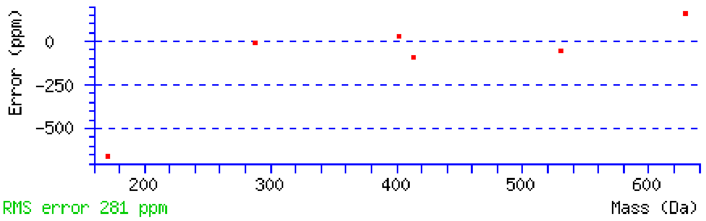

All matches to this query

Score	Mr(calc):	Delta	Sequence
42.7	699.4279	0.0009	AVEILR
42.7	699.4279	0.0009	AVELLR
21.8	699.4279	0.0009	ADILLR
16.9	699.4279	0.0009	ALDLLR
14.8	699.4279	0.0009	GLLELR
14.8	699.4279	0.0009	LGLEIR
14.2	699.4279	0.0009	LVEAIR
14.2	699.4279	0.0009	LVEALR
14.2	699.4279	0.0009	VIEAIR
14.2	699.4279	0.0009	VIEALR

Spectrum No: 90; Query: 34966; Rank: 1

Peptide View

MS/MS Fragmentation of **AVELLRK**
Found in **IPI00028006**, Tax_Id=9606 Gene_Symbol=PSMB2 Proteasome subunit beta type-2

Match to Query 34966: 827.524328 from(414.769440,2+)
Title: 090702LimSK_Exosome2_06.5079.5079.2.dta
Data file C:\Temp\Mascoti\LSK_2D\090702LimSK_Exosome2_01_2D.mgf

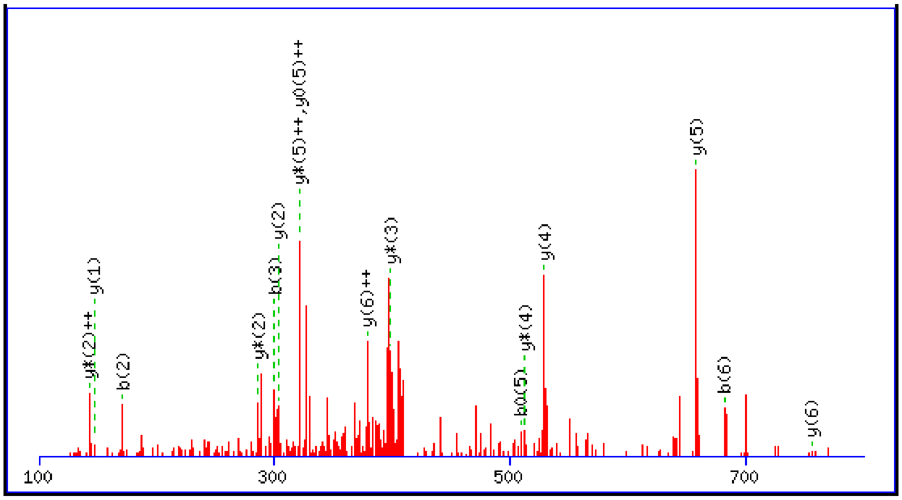

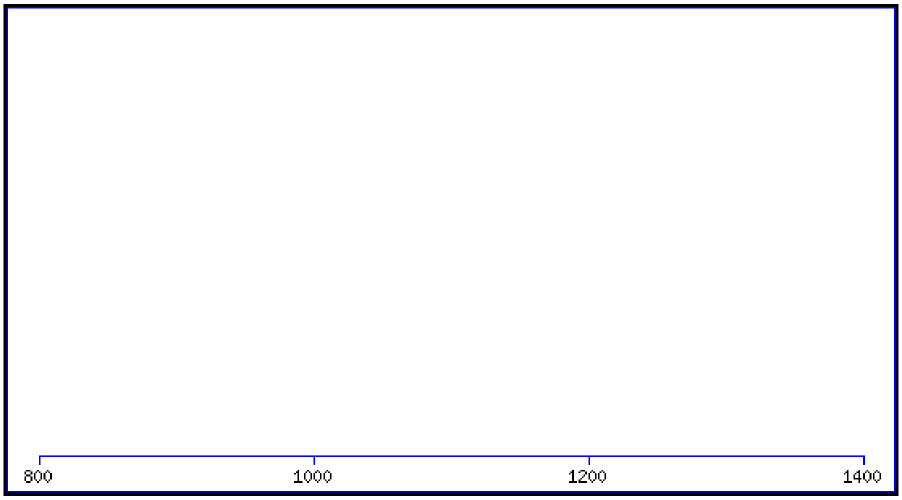

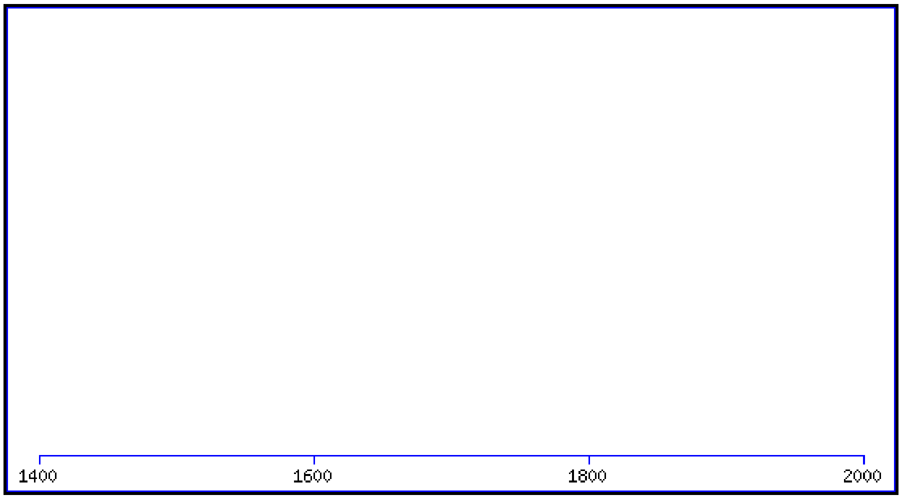

Monoisotopic mass of neutral peptide Mr(calc): 827.5229
Fixed modifications: Carbamidomethyl (C)
Ions Score: 24 **Expect:** 0.17
Matches (Bold Red): 16/50 fragment ions using 51 most intense peaks

#	b	b^{++}	b*	b^{*++}	b^0	b^{0++}	Seq.	y	y^{++}	y*	y^{*++}	y^0	y^{0++}	#
1	72.0444	36.5258					A							7
2	**171.1128**	86.0600					V	**757.4931**	**379.2502**	740.4665	370.7369	739.4825	370.2449	6
3	**300.1554**	150.5813			282.1448	141.5761	E	**658.4246**	329.7160	641.3981	**321.2027**	640.4141	**320.7107**	5
4	413.2395	207.1234			395.2289	198.1181	L	**529.3820**	265.1947	**512.3555**	256.6814			4
5	526.3235	263.6654			**508.3130**	254.6601	L	416.2980	208.6526	**399.2714**	200.1394			3
6	**682.4246**	341.7160	665.3981	333.2027	664.4141	332.7107	R	**303.2139**	152.1106	**286.1874**	**143.5973**			2

7							K	147.1128	74.0600	130.0863	65.5468			1

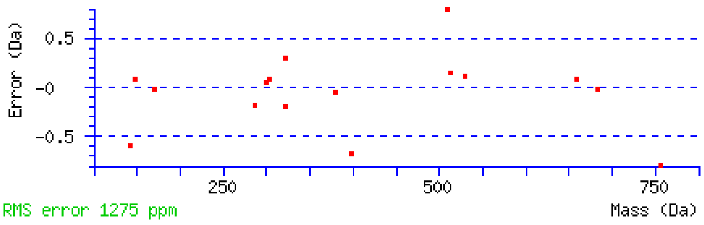

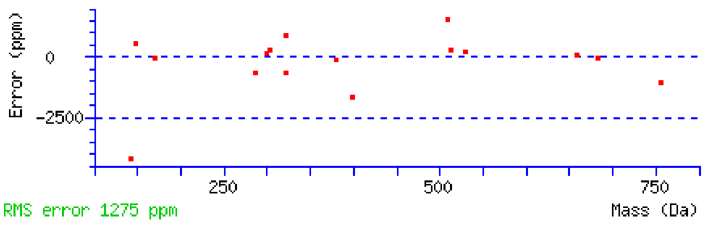

All matches to this query

Score	Mr(calc):	Delta	Sequence
24.1	827.5229	0.0015	AVELLRK
7.9	827.5229	0.0014	GLVQKGVK
7.0	827.5229	0.0015	IGELRIK
5.6	827.5229	0.0015	GLLVSALR
5.3	827.5229	0.0015	AEVRLLK
4.2	826.5137	1.0106	GLTPRRK
4.2	827.5229	0.0015	LGLEIRK
4.0	827.5229	0.0015	GLKIQAAK
2.3	825.5184	2.0059	GKPQLRK
2.1	826.5276	0.9967	VAGAIGIVK

Spectrum No: 91; Query: 39572; Rank: 1

Peptide View

MS/MS Fragmentation of **CLEELQK**
Found in **IPI00028006**, Tax_Id=9606 Gene_Symbol=PSMB2 Proteasome subunit beta type-2

Match to Query 39572: 918.449648 from(460.232100,2+)
Title: 090702LimSK_Exosome2_06.1845.1845.2.dta
Data file C:\Temp\Mascoti\LSK_2D\090702LimSK_Exosome2_01_2D.mgf

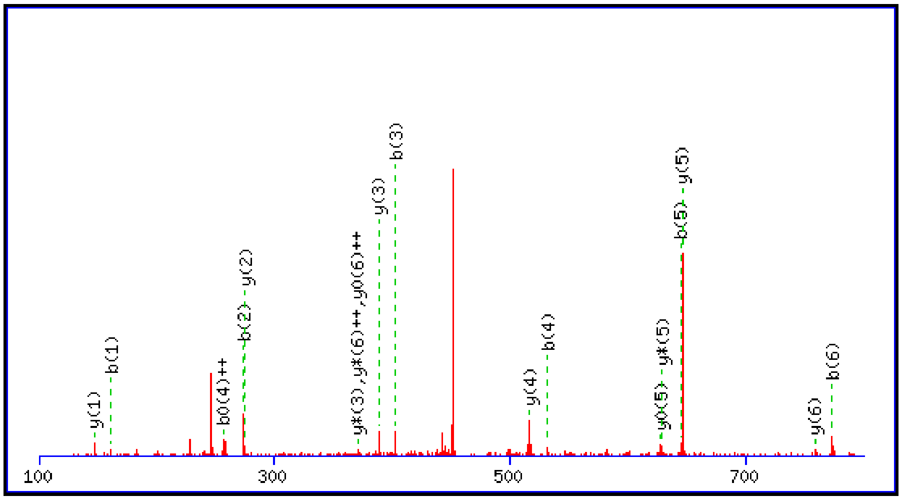

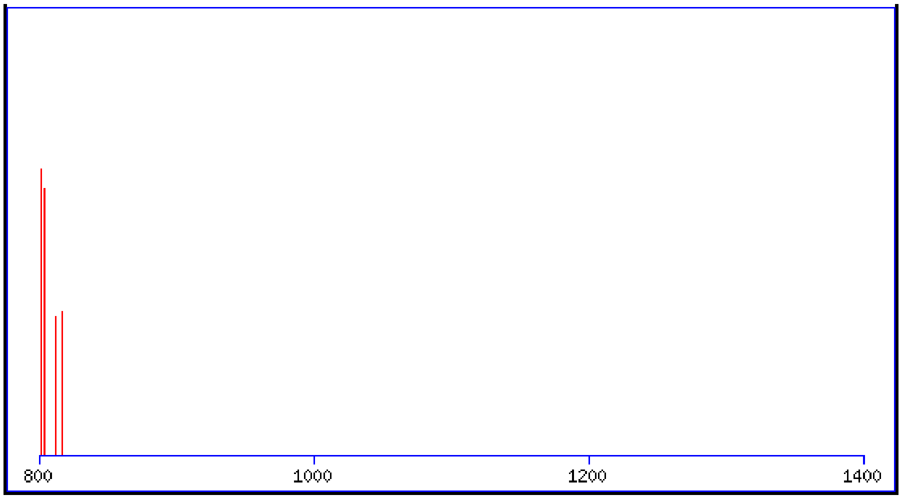

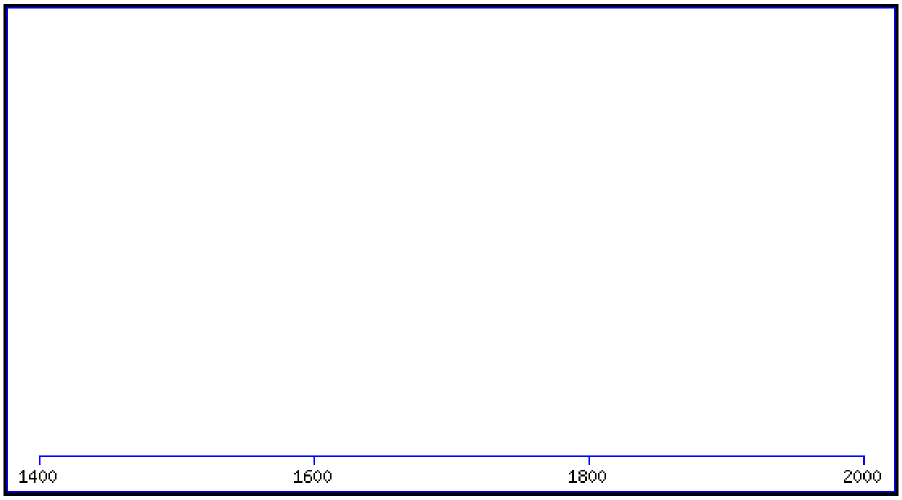

Monoisotopic mass of neutral peptide Mr(calc): 918.4480
Fixed modifications: Carbamidomethyl (C)
Ions Score: 52 **Expect:** 0.00072
Matches (Bold Red): 18/52 fragment ions using 23 most intense peaks

#	b	b^{++}	b^{*}	b^{*++}	b^{0}	b^{0++}	Seq.	y	y^{++}	y^{*}	y^{*++}	y^{0}	y^{0++}	#
1	**161.0379**	81.0226					C							7
2	**274.1220**	137.5646					L	**759.4247**	380.2160	742.3981	**371.7027**	741.4141	**371.2107**	6
3	**403.1646**	202.0859			385.1540	193.0806	E	**646.3406**	323.6740	**629.3141**	315.1607	**628.3301**	314.6687	5
4	**532.2072**	266.6072			514.1966	**257.6019**	E	**517.2980**	259.1527	500.2715	250.6394	499.2875	250.1474	4
5	**645.2912**	323.1493			627.2807	314.1440	L	**388.2554**	194.6314	**371.2289**	186.1181			3
6	**773.3498**	387.1785	756.3233	378.6653	755.3393	378.1733	Q	**275.1714**	138.0893	258.1448	129.5761			2
7							K	**147.1128**	74.0600	130.0863	65.5468			1

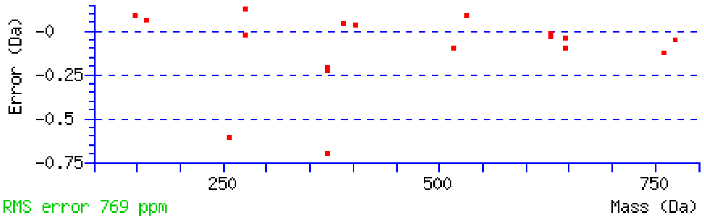

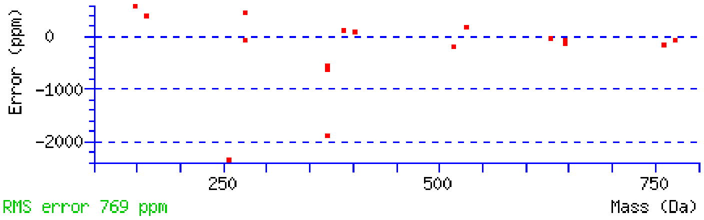

All matches to this query

Score	Mr(calc):	Delta	Sequence
52.1	918.4480	0.0016	CLEELQK
34.2	918.4480	0.0016	CLELEQK

24.1	918.4480	0.0016	AAEMELQK
21.3	917.4454	1.0042	GQSEEIQK
21.3	918.4480	0.0016	XLIEEQK
18.2	918.4454	0.0043	CNLGSRGR
16.1	918.4519	-0.0023	NGGGSGKSAGK
12.5	918.4406	0.0090	TRQEEEK
12.4	918.4559	-0.0063	DTWDRVK
11.1	916.4362	2.0134	DRAEAEAR

Spectrum No: 92; Query: 46440; Rank: 1

Peptide View

MS/MS Fragmentation of **CLEELQKR**
Found in **IPI00028006**, Tax_Id=9606 Gene_Symbol=PSMB2 Proteasome subunit beta type-2

Match to Query 46440: 1074.552248 from(538.283400,2+)
Title: 090702LimSK_Exosome2_06.4011.4011.2.dta
Data file C:\Temp\Mascoti\LSK_2D\090702LimSK_Exosome2_01_2D.mgf

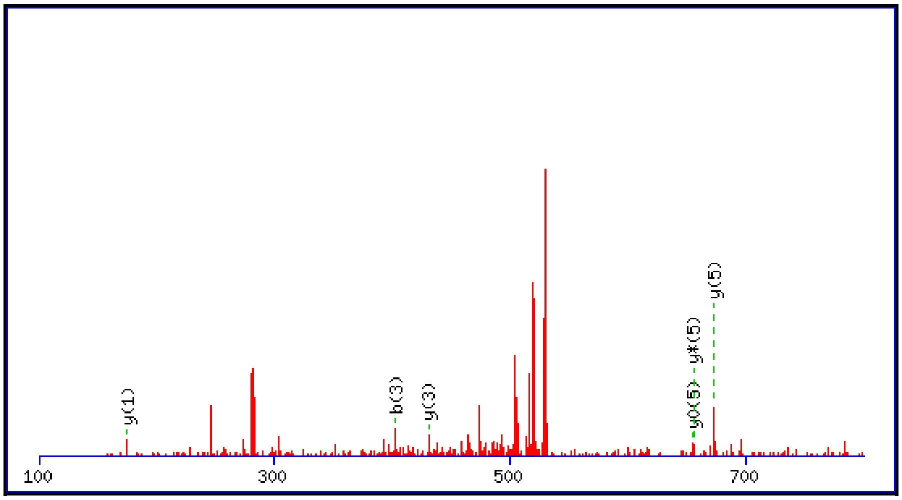

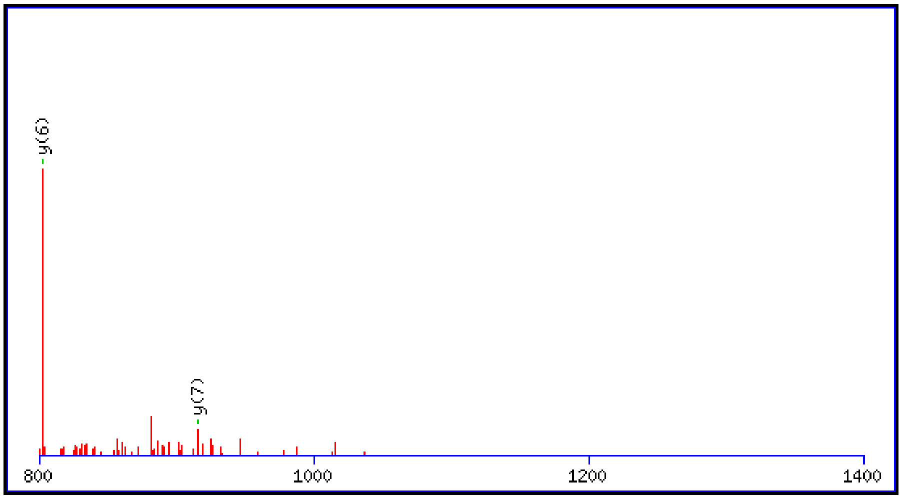

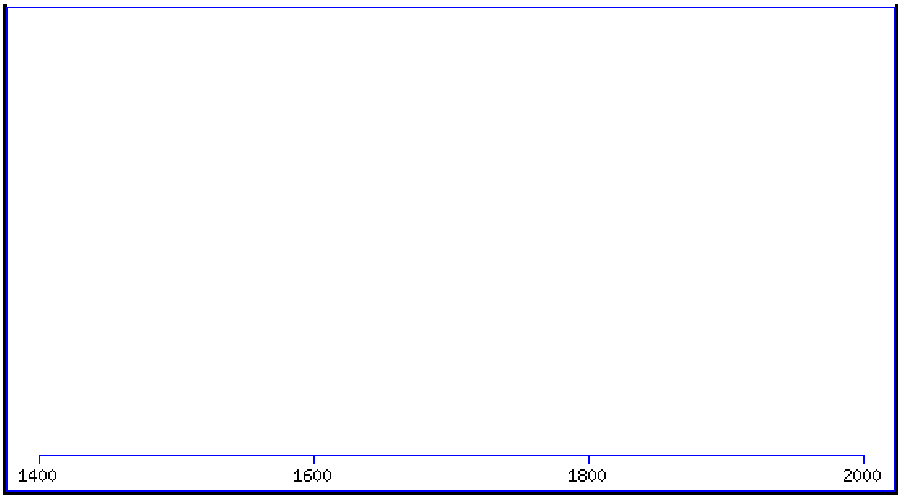

Monoisotopic mass of neutral peptide Mr(calc): 1074.5491
Fixed modifications: Carbamidomethyl (C)
Ions Score: 24 **Expect:** 0.63
Matches (Bold Red): 8/62 fragment ions using 32 most intense peaks

#	b	b^{++}	b*	b^{*++}	b^{0}	b^{0++}	Seq.	y	y^{++}	y*	y^{*++}	y^{0}	y^{0++}	#
1	161.0379	81.0226					C							8
2	274.1220	137.5646					L	**915.5258**	458.2665	898.4993	449.7533	897.5152	449.2613	7
3	**403.1646**	202.0859			385.1540	193.0806	E	**802.4417**	401.7245	785.4152	393.2112	784.4312	392.7192	6
4	532.2072	266.6072			514.1966	257.6019	E	**673.3991**	337.2032	**656.3726**	328.6899	**655.3886**	328.1979	5
5	645.2912	323.1493			627.2807	314.1440	L	544.3566	272.6819	527.3300	264.1686			4
6	773.3498	387.1785	756.3233	378.6653	755.3393	378.1733	Q	**431.2725**	216.1399	414.2459	207.6266			3
7	901.4448	451.2260	884.4182	442.7128	883.4342	442.2207	K	303.2139	152.1106	286.1874	143.5973			2
8							R	**175.1190**	88.0631	158.0924	79.5498			1

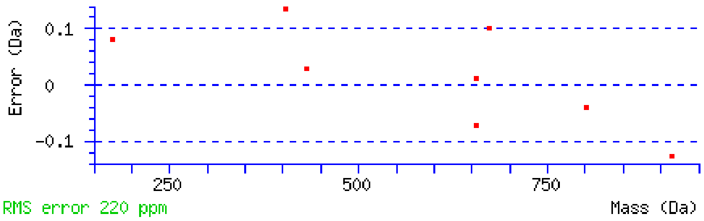

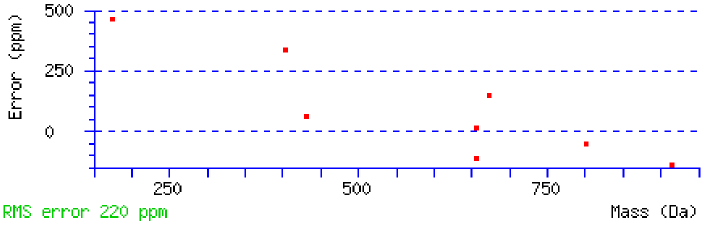

All matches to this query

Score	Mr(calc):	Delta	Sequence
23.7	1074.5491	0.0031	CLEELQKR
14.6	1074.5604	-0.0081	XAAMATARVR
12.3	1074.5492	0.0031	LECSGAVLAR
10.3	1074.5458	0.0064	SSVAFAGPSPR
9.3	1074.5491	0.0031	EICLEQRK
7.7	1074.5491	0.0031	MSSPLADAKR
7.2	1072.5512	2.0010	EAKKSEEPR
6.5	1074.5418	0.0105	EATSADARVR
6.0	1072.5513	2.0010	LDKENAIDR
6.0	1072.5513	2.0010	LDKENALDR

Spectrum No: 93; Query: 60584; Rank: 1

Peptide View

MS/MS Fragmentation of **FILNLPTFSVR**
Found in **IPI00028006**, Tax_Id=9606 Gene_Symbol=PSMB2 Proteasome subunit beta type-2

Match to Query 60584: 1305.747248 from(653.880900,2+)
Title: 090702LimSK_Exosome2_04.8920.8920.2.dta
Data file C:\Temp\Mascoti\LSK_2D\090702LimSK_Exosome2_01_2D.mgf

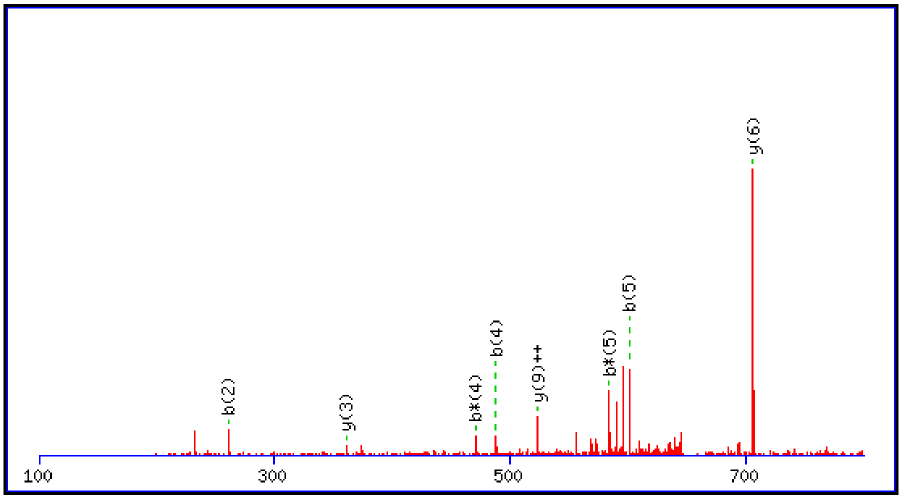

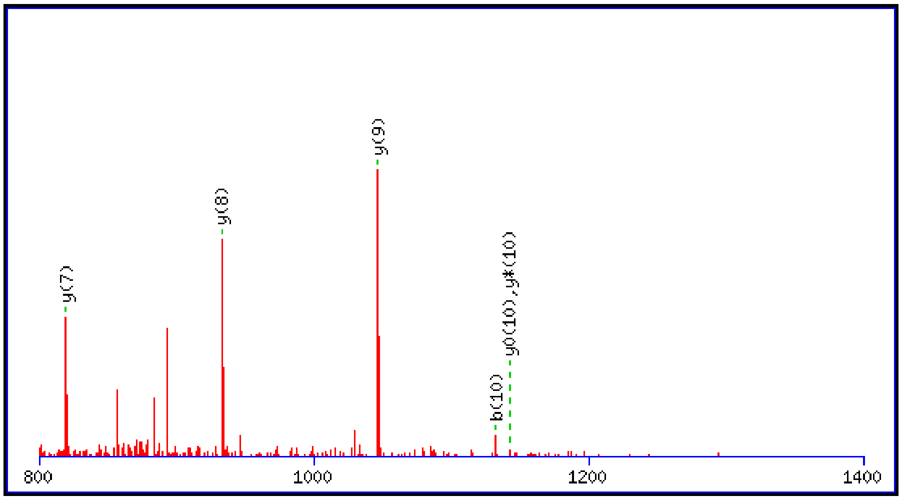

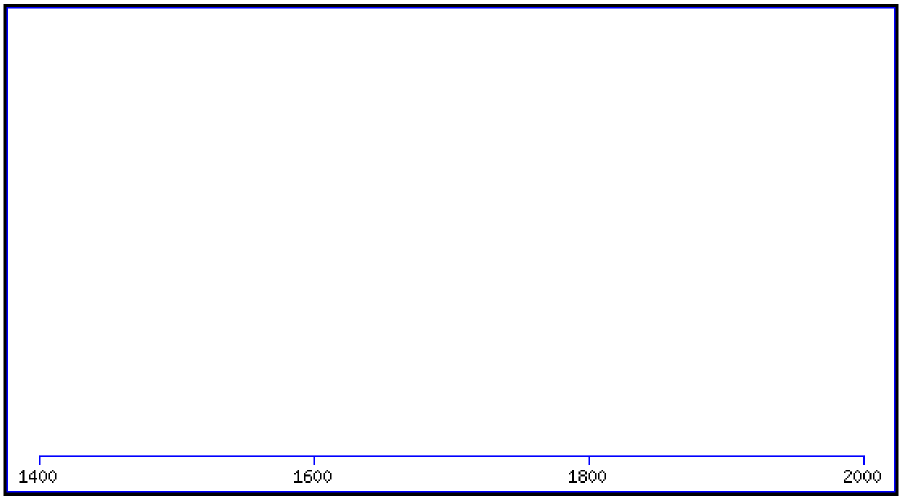

Monoisotopic mass of neutral peptide Mr(calc): 1305.7445
Fixed modifications: Carbamidomethyl (C)
Ions Score: 41 **Expect:** 0.0047
Matches (Bold Red): 14/98 fragment ions using 28 most intense peaks

		++		++	0	0++			++		++	0	0++	

#	b	b	b*	b*	b	b	Seq.	y	y	y*	y*	y	y	#
1	148.0757	74.5415					F							11
2	**261.1598**	131.0835					I	1159.6834	580.3453	**1142.6568**	571.8320	**1141.6728**	571.3400	10
3	374.2438	187.6255					L	**1046.5993**	**523.8033**	1029.5728	515.2900	1028.5887	514.7980	9
4	**488.2867**	244.6470	**471.2602**	236.1337			N	**933.5152**	467.2613	916.4887	458.7480	915.5047	458.2560	8
5	**601.3708**	301.1890	**584.3443**	292.6758			L	**819.4723**	410.2398	802.4458	401.7265	801.4617	401.2345	7
6	698.4236	349.7154	681.3970	341.2022			P	**706.3883**	353.6978	689.3617	345.1845	688.3777	344.6925	6
7	799.4713	400.2393	782.4447	391.7260	781.4607	391.2340	T	609.3355	305.1714	592.3089	296.6581	591.3249	296.1661	5
8	946.5397	473.7735	929.5131	465.2602	928.5291	464.7682	F	508.2878	254.6475	491.2613	246.1343	490.2772	245.6423	4
9	1033.5717	517.2895	1016.5451	508.7762	1015.5611	508.2842	S	**361.2194**	181.1133	344.1928	172.6001	343.2088	172.1081	3
10	**1132.6401**	566.8237	1115.6136	558.3104	1114.6295	557.8184	V	274.1874	137.5973	257.1608	129.0840			2
11							R	175.1190	88.0631	158.0924	79.5498			1

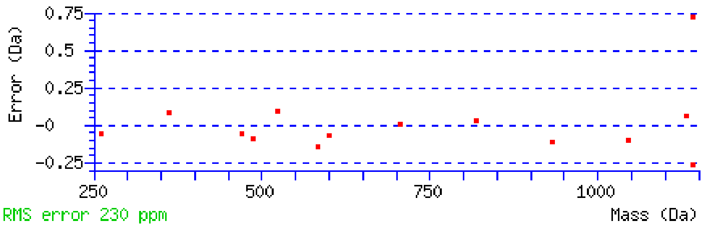

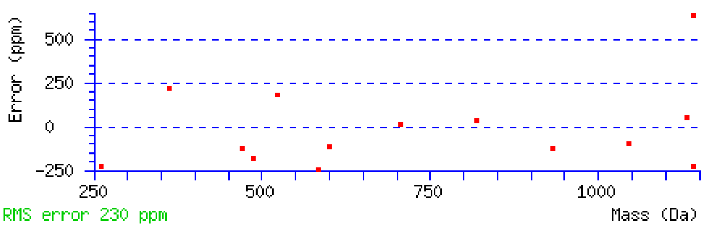

All matches to this query

Score	Mr(calc):	Delta	Sequence
41.1	1305.7445	0.0027	FILNLPTFSVR
14.3	1305.7517	-0.0045	NAGQVVHLTLVR
14.1	1305.7478	-0.0006	RIYLCLDLLK
6.4	1303.7500	1.9973	LDTRLLYPVSK
5.3	1305.7517	-0.0044	HRAAEAAINILK
4.2	1304.7452	1.0020	IHETQPLLNLK
2.5	1305.7378	0.0095	RNHNLALGRQK
1.4	1305.7478	-0.0006	MLEFVSLLARK

Spectrum No: 94; Query: 74279; Rank: 1

Peptide View

MS/MS Fragmentation of **NGYELSPTAAANFTR**
Found in **IPI00028006**, Tax_Id=9606 Gene_Symbol=PSMB2 Proteasome subunit beta type-2

Match to Query 74279: 1610.773028 from(806.393790,2+)
Title: 090702LimSK_Exosome2_06.6211.6211.2.dta
Data file C:\Temp\Mascoti\LSK_2D\090702LimSK_Exosome2_01_2D.mgf

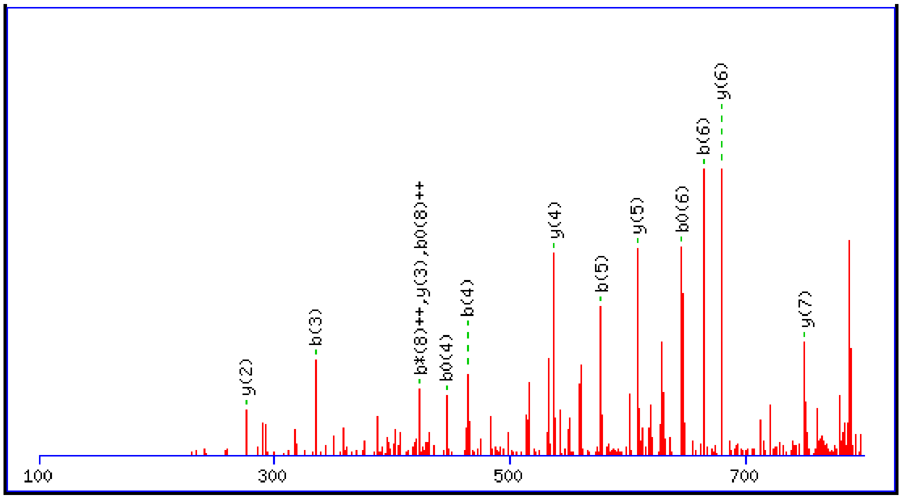

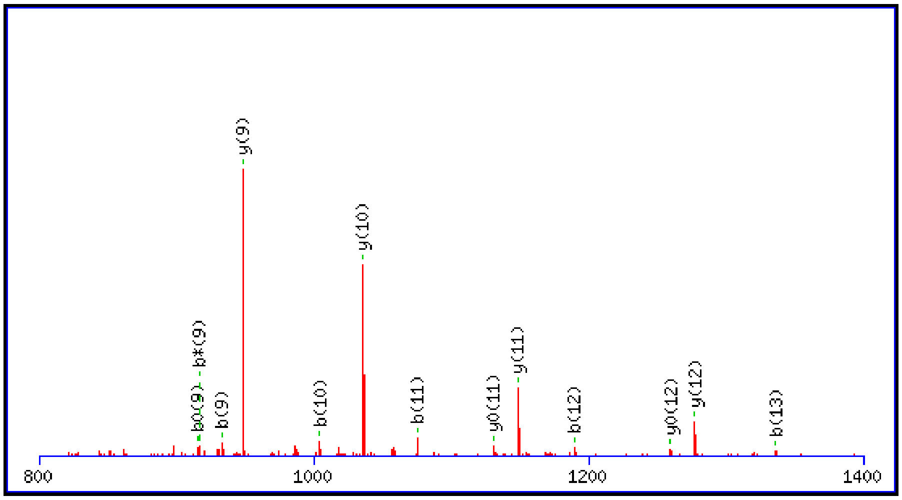

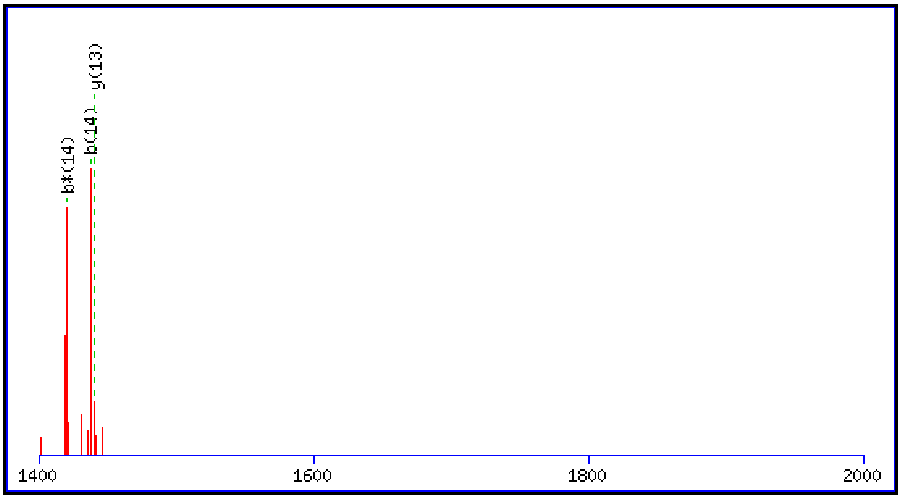

Monoisotopic mass of neutral peptide Mr(calc): 1610.7689
Fixed modifications: Carbamidomethyl (C)
Ions Score: 90 **Expect:** 1.9e-007
Matches (Bold Red): 30/160 fragment ions using 42 most intense peaks

#	b	b^{++}	b*	b^{*++}	b^0	b^{0++}	Seq.	y	y^{++}	y*	y^{*++}	y^0	y^{0++}	#
1	115.0502	58.0287	98.0237	49.5155			N							15
2	172.0717	86.5395	155.0451	78.0262			G	1497.7332	749.3703	1480.7067	740.8570	1479.7227	740.3650	14
3	**335.1350**	168.0711	318.1084	159.5579			Y	**1440.7118**	720.8595	1423.6852	712.3462	1422.7012	711.8542	13
4	**464.1776**	232.5924	447.1510	224.0792	**446.1670**	223.5872	E	**1277.6484**	639.3279	1260.6219	630.8146	**1259.6379**	630.3226	12
5	**577.2617**	289.1345	560.2351	280.6212	559.2511	280.1292	L	**1148.6058**	574.8066	1131.5793	566.2933	**1130.5953**	565.8013	11
6	**664.2937**	332.6505	647.2671	324.1372	**646.2831**	323.6452	S	**1035.5218**	518.2645	1018.4952	509.7513	1017.5112	509.2592	10

7	761.3464	381.1769	744.3199	372.6636	743.3359	372.1716	P	**948.4898**	474.7485	931.4632	466.2352	930.4792	465.7432	9
8	862.3941	431.7007	845.3676	**423.1874**	844.3836	**422.6954**	T	851.4370	426.2221	834.4104	417.7089	833.4264	417.2169	8
9	**933.4312**	467.2193	**916.4047**	458.7060	**915.4207**	458.2140	A	**750.3893**	375.6983	733.3628	367.1850	732.3787	366.6930	7
10	**1004.4684**	502.7378	987.4418	494.2245	986.4578	493.7325	A	**679.3522**	340.1797	662.3257	331.6665	661.3416	331.1745	6
11	**1075.5055**	538.2564	1058.4789	529.7431	1057.4949	529.2511	A	**608.3151**	304.6612	591.2885	296.1479	590.3045	295.6559	5
12	**1189.5484**	595.2778	1172.5218	586.7646	1171.5378	586.2726	N	**537.2780**	269.1426	520.2514	260.6293	519.2674	260.1373	4
13	**1336.6168**	668.8120	1319.5903	660.2988	1318.6062	659.8068	F	**423.2350**	212.1212	406.2085	203.6079	405.2245	203.1159	3
14	**1437.6645**	719.3359	**1420.6379**	710.8226	1419.6539	710.3306	T	**276.1666**	138.5870	259.1401	130.0737	258.1561	129.5817	2
15							R	175.1190	88.0631	158.0924	79.5498			1

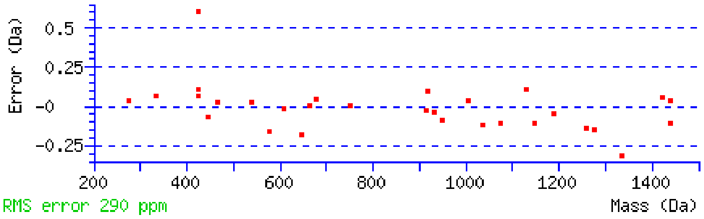

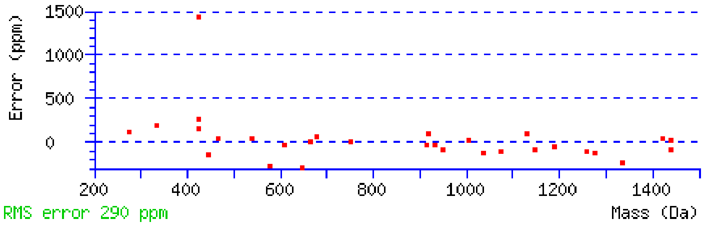

All matches to this query

Score	Mr(calc):	Delta	Sequence
90.2	1610.7689	0.0042	NGYELSPTAAANFTR
4.4	1610.7657	0.0073	EFGKNREVMGLCR
0.4	1608.7549	2.0182	SAVWLPDSVLEASR
0.4	1608.7549	2.0182	SAVWLPDSVLEASR

Spectrum No: 95; Query: 36994; Rank: 1

Peptide View

MS/MS Fragmentation of **NLADCLR**
Found in **IPI00028006**, Tax_Id=9606 Gene_Symbol=PSMB2 Proteasome subunit beta type-2

Match to Query 36994: 860.419688 from(431.217120,2+)
Title: 090702LimSK_Exosome2_05.2967.2967.2.dta
Data file C:\Temp\Mascoti\LSK_2D\090702LimSK_Exosome2_01_2D.mgf

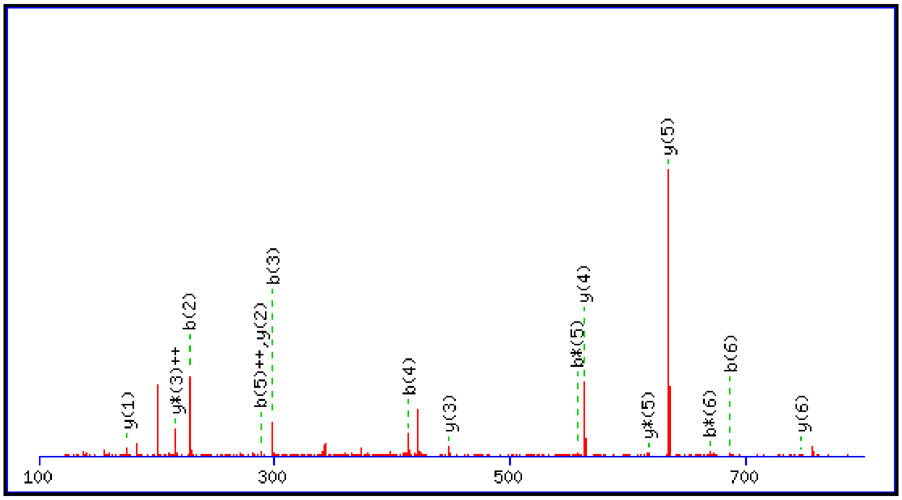

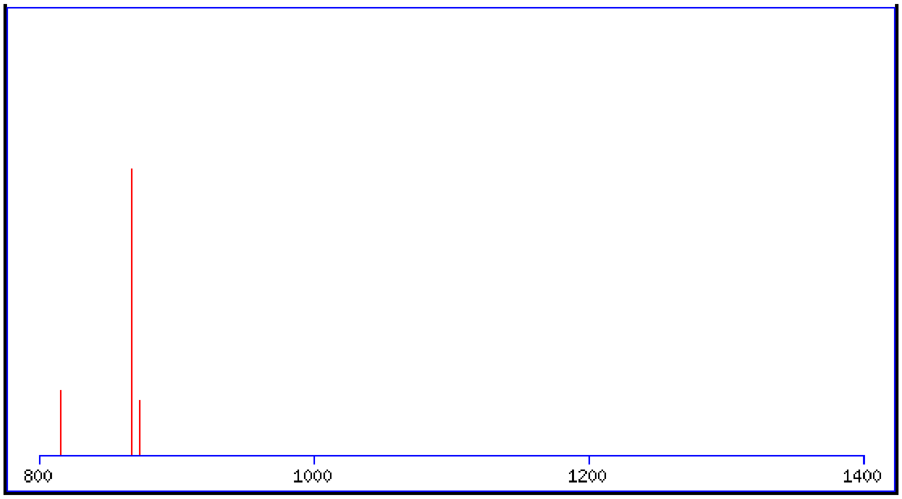

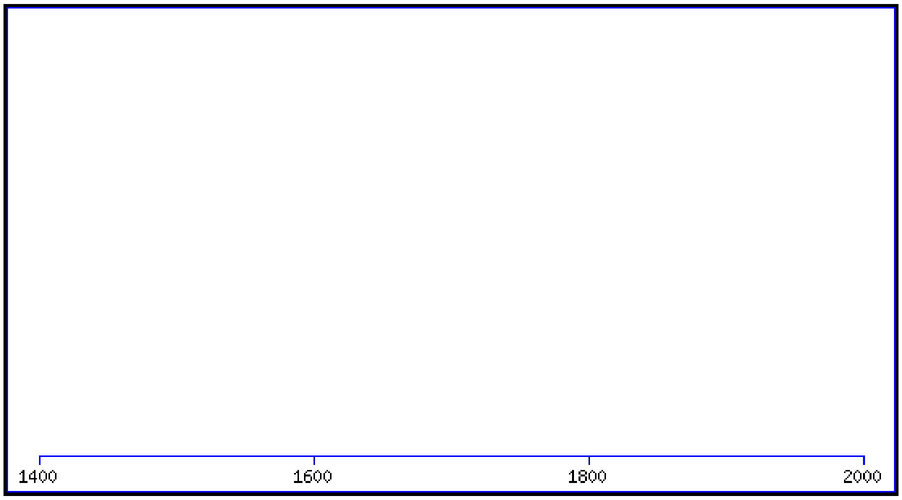

Monoisotopic mass of neutral peptide Mr(calc): 860.4174
Fixed modifications: Carbamidomethyl (C)
Ions Score: 41 **Expect:** 0.0073
Matches (Bold Red): 15/60 fragment ions using 28 most intense peaks

#	b	b++	b*	b*++	b0	b0++	Seq.	y	y++	y*	y*++	y0	y0++	#
1	115.0502	58.0287	98.0237	49.5155			N							7
2	**228.1343**	114.5708	211.1077	106.0575			L	**747.3818**	374.1945	730.3552	365.6813	729.3712	365.1892	6
3	**299.1714**	150.0893	282.1448	141.5761			A	**634.2977**	317.6525	**617.2712**	309.1392	616.2872	308.6472	5
4	**414.1983**	207.6028	397.1718	199.0895	396.1878	198.5975	D	**563.2606**	282.1339	546.2341	273.6207	545.2500	273.1287	4
5	574.2290	**287.6181**	**557.2024**	279.1049	556.2184	278.6128	C	**448.2337**	224.6205	431.2071	**216.1072**			3
6	**687.3130**	344.1602	**670.2865**	335.6469	669.3025	335.1549	L	**288.2030**	144.6051	271.1765	136.0919			2
7							R	**175.1190**	88.0631	158.0924	79.5498			1

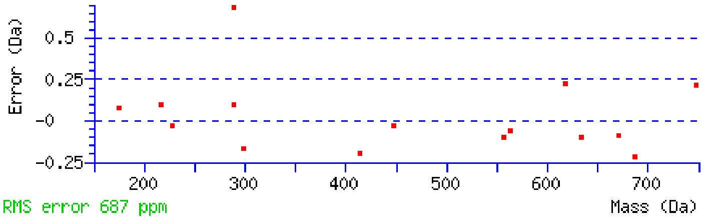

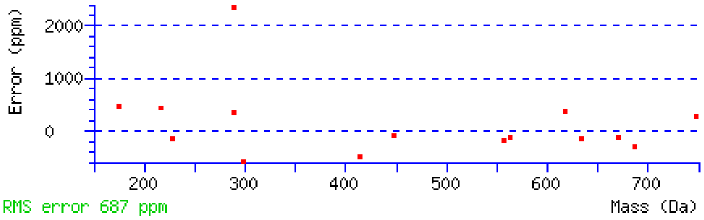

All matches to this query

Score	Mr(calc):	Delta	Sequence
41.1	860.4174	0.0023	NLADCLR
8.9	859.4204	0.9993	KAIYASK

8.9	859.4204	0.9993	KALYASK
8.9	859.4222	0.9975	LIPDSCR
6.8	860.4174	0.0023	IDQMQAR
6.0	858.4195	2.0002	LDADAAQR
6.0	860.4248	-0.0051	NLCMVPK
5.6	858.4170	2.0027	GAPWLCR
5.3	860.4174	0.0023	INVMNDR
5.2	860.4174	0.0023	NLPQMSR

Spectrum No: 96; Query: 30550; Rank: 1

Peptide View

MS/MS Fragmentation of **NVQLYK**
Found in **IPI00028006**, Tax_Id=9606 Gene_Symbol=PSMB2 Proteasome subunit beta type-2

Match to Query 30550: 763.424108 from(382.719330,2+)
Title: 090702LimSK_Exosome2_06.1888.1888.2.dta
Data file C:\Temp\Mascoti\LSK_2D\090702LimSK_Exosome2_01_2D.mgf

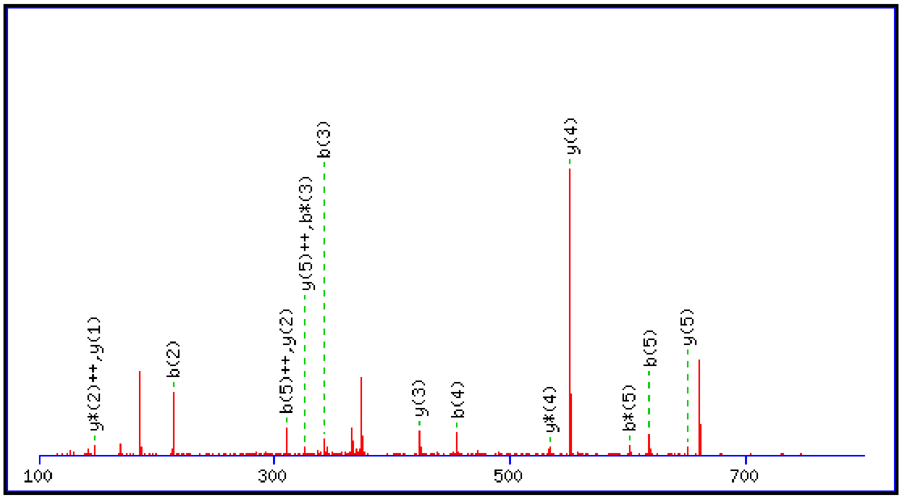

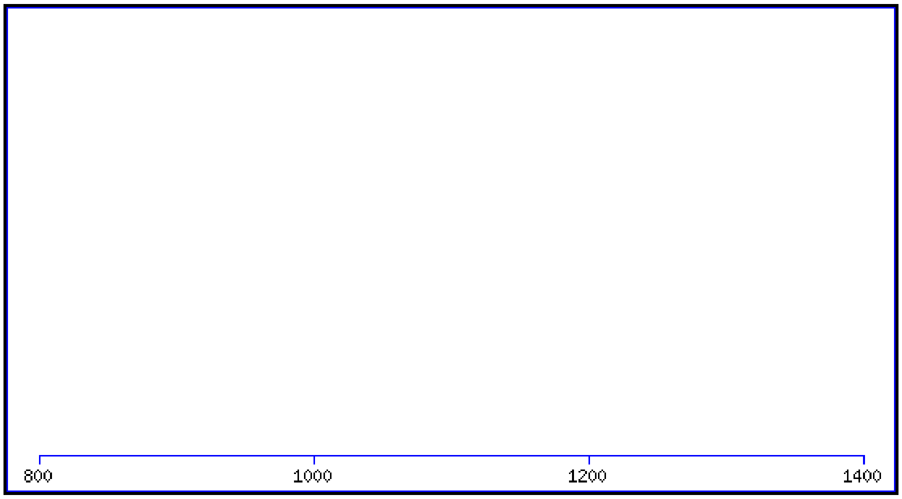

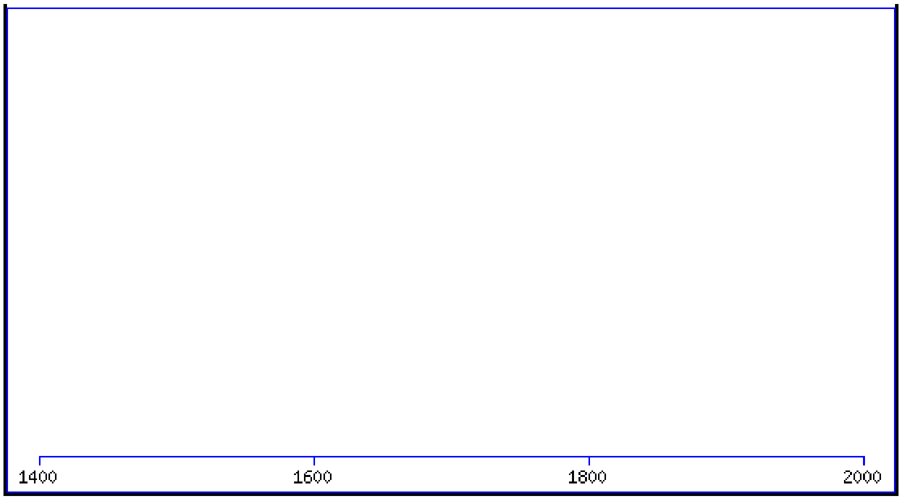

Monoisotopic mass of neutral peptide Mr(calc): 763.4228
Fixed modifications: Carbamidomethyl (C)
Ions Score: 32 **Expect:** 0.064
Matches (Bold Red): 15/40 fragment ions using 33 most intense peaks

#	b	b^{++}	b*	b^{*++}	Seq.	y	y^{++}	y*	y^{*++}	#
1	115.0502	58.0287	98.0237	49.5155	N					6
2	**214.1186**	107.5629	197.0921	99.0497	V	**650.3872**	**325.6972**	633.3606	317.1840	5
3	**342.1772**	171.5922	**325.1506**	163.0790	Q	**551.3188**	276.1630	**534.2922**	267.6498	4
4	**455.2613**	228.1343	438.2347	219.6210	L	**423.2602**	212.1337	406.2336	203.6205	3
5	**618.3246**	**309.6659**	**601.2980**	301.1527	Y	**310.1761**	155.5917	293.1496	**147.0784**	2
6					K	**147.1128**	74.0600	130.0863	65.5468	1

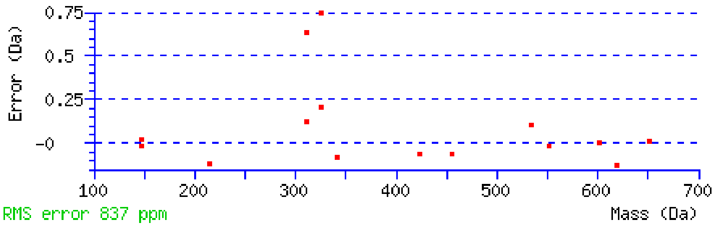

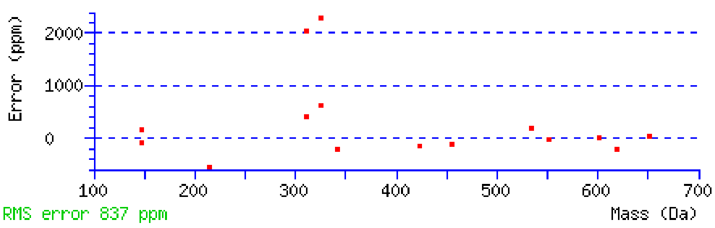

All matches to this query

Score	Mr(calc):	Delta	Sequence
31.8	763.4228	0.0013	NVQLYK
19.8	763.4228	0.0013	NVQYIK
17.9	762.4276	0.9965	DPKLYK
14.5	763.4228	0.0013	IVNGAYK
13.6	762.4276	0.9965	VLGAISGF
4.0	763.4228	0.0013	KGEGVFK
4.0	763.4262	-0.0021	KIKTMQ
4.0	763.4228	0.0013	QLQFTK
2.1	763.4228	0.0013	NKVEFK
1.3	763.4228	0.0013	YNLNIK

Spectrum No: 97; Query: 43596; Rank: 1

Peptide View

MS/MS Fragmentation of **RNLADCLR**
Found in **IPI00028006**, Tax_Id=9606 Gene_Symbol=PSMB2 Proteasome subunit beta type-2

Match to Query 43596: 1016.520208 from(509.267380,2+)
Title: 090702LimSK_Exosome2_06.6473.6473.2.dta
Data file C:\Temp\Mascoti\LSK_2D\090702LimSK_Exosome2_01_2D.mgf

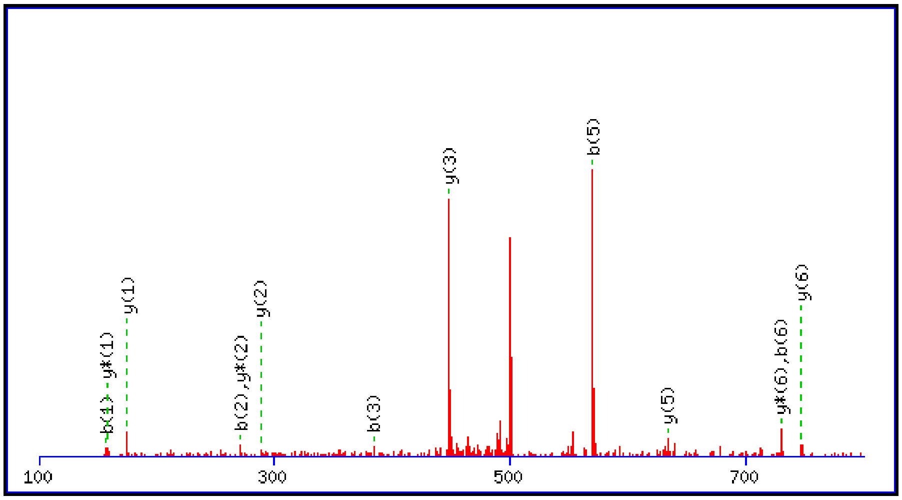

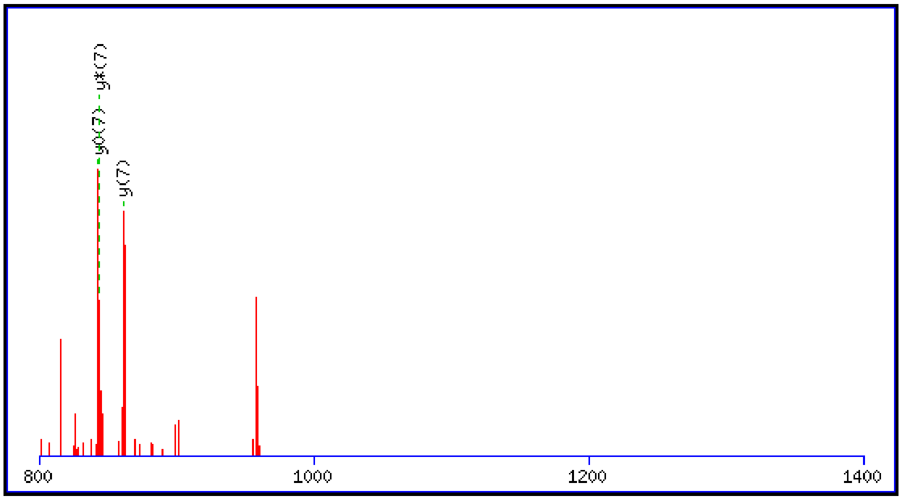

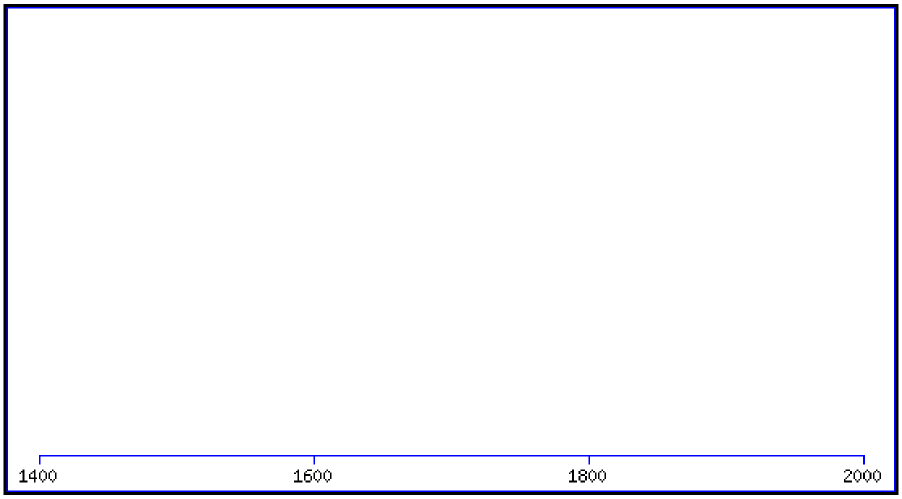

Monoisotopic mass of neutral peptide Mr(calc): 1016.5185
Fixed modifications: Carbamidomethyl (C)
Ions Score: 38 **Expect:** 0.019
Matches (Bold Red): 17/70 fragment ions using 35 most intense peaks

#	b	b^{++}	b*	b^{*++}	b^0	b^{0++}	Seq.	y	y^{++}	y*	y^{*++}	y^0	y^{0++}	#
1	**157.1084**	79.0578	140.0818	70.5446			R							8

2	**271.1513**	136.0793	254.1248	127.5660			N	**861.4247**	431.2160	**844.3982**	422.7027	**843.4141**	422.2107	7
3	**384.2354**	192.6213	367.2088	184.1081			L	**747.3818**	374.1945	**730.3552**	365.6813	729.3712	365.1892	6
4	455.2725	228.1399	438.2459	219.6266			A	**634.2977**	317.6525	617.2712	309.1392	616.2872	308.6472	5
5	**570.2994**	285.6534	553.2729	277.1401	552.2889	276.6481	D	563.2606	282.1339	546.2341	273.6207	545.2500	273.1287	4
6	**730.3301**	365.6687	713.3035	357.1554	712.3195	356.6634	C	**448.2337**	224.6205	431.2071	216.1072			3
7	**843.4141**	422.2107	826.3876	413.6974	825.4036	413.2054	L	**288.2030**	144.6051	**271.1765**	136.0919			2
8							R	**175.1190**	88.0631	**158.0924**	79.5498			1

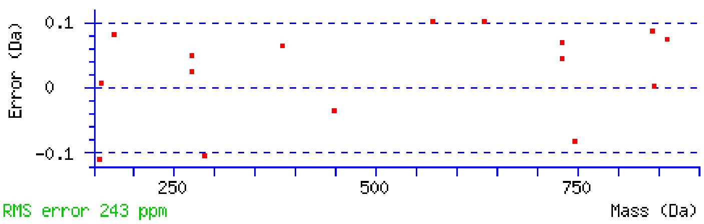

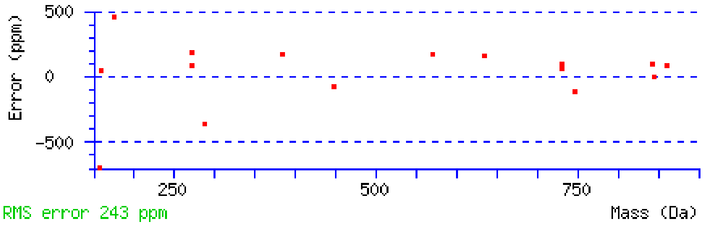

All matches to this query

Score	Mr(calc):	Delta	Sequence
38.2	1016.5185	0.0017	RNLADCLR
17.5	1016.5120	0.0082	RCPRICR
9.8	1016.5224	-0.0022	RGGQSSRGGR
9.5	1016.5251	-0.0048	LRDDTELR
9.2	1016.5266	-0.0064	MIWWPLR
9.2	1016.5147	0.0055	MMPGAPLLR
9.2	1016.5168	0.0034	TRYKTIR
8.8	1016.5185	0.0017	RLQEANMR
8.8	1016.5111	0.0091	RNERGETR
8.6	1016.5138	0.0064	GLLSDEDLR

Spectrum No: 98; Query: 45667; Rank: 1

Peptide View

MS/MS Fragmentation of **VAASNIVQMK**
Found in **IPI00028006**, Tax_Id=9606 Gene_Symbol=PSMB2 Proteasome subunit beta type-2

Match to Query 45667: 1059.577208 from(530.795880,2+)
Title: 090702LimSK_Exosome2_06.3112.3112.2.dta
Data file C:\Temp\Mascoti\LSK_2D\090702LimSK_Exosome2_01_2D.mgf

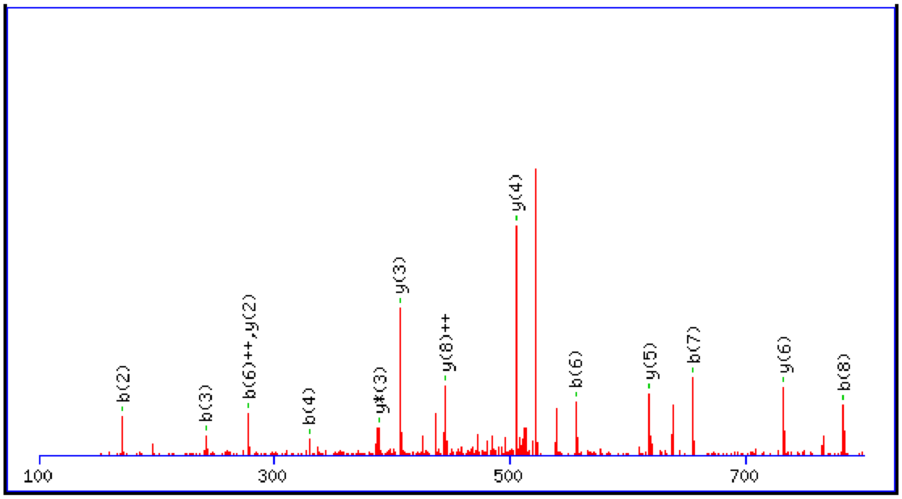

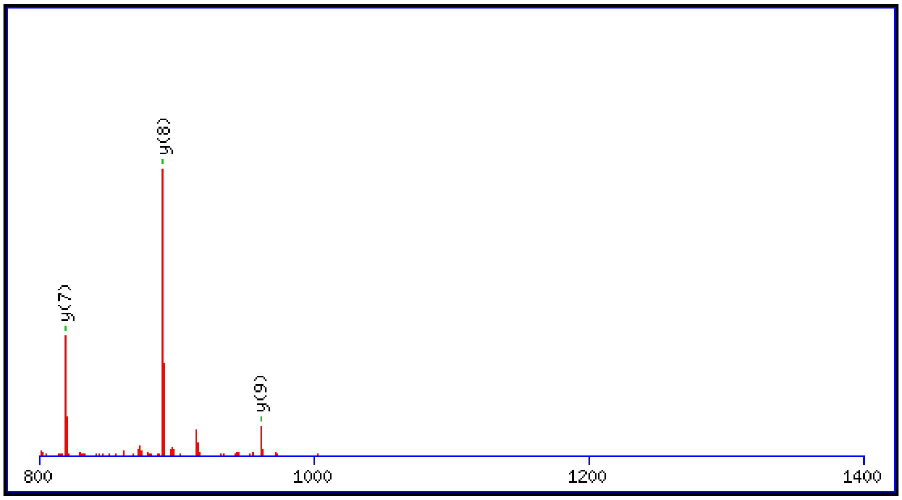

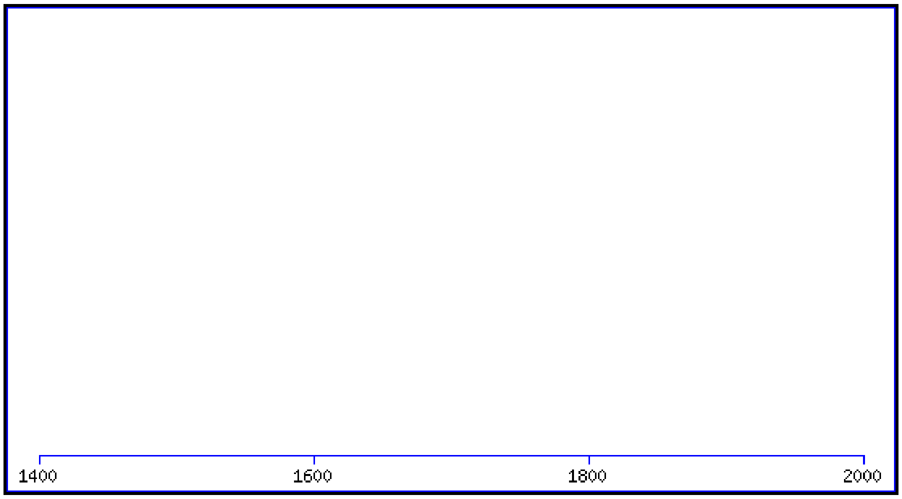

Monoisotopic mass of neutral peptide Mr(calc): 1059.5746
Fixed modifications: Carbamidomethyl (C)
Ions Score: 79 **Expect:** 2e-006
Matches (Bold Red): 17/82 fragment ions using 20 most intense peaks

#	b	b^{++}	b*	b^{*++}	b^{0}	b^{0++}	Seq.	y	y^{++}	y*	y^{*++}	y^{0}	y^{0++}	#
1	100.0757	50.5415					V							10
2	**171.1128**	86.0600					A	**961.5135**	481.2604	944.4870	472.7471	943.5030	472.2551	9
3	**242.1499**	121.5786					A	**890.4764**	**445.7418**	873.4499	437.2286	872.4658	436.7366	8
4	**329.1819**	165.0946			311.1714	156.0893	S	**819.4393**	410.2233	802.4128	401.7100	801.4287	401.2180	7
5	443.2249	222.1161	426.1983	213.6028	425.2143	213.1108	N	**732.4073**	366.7073	715.3807	358.1940			6
6	**556.3089**	**278.6581**	539.2824	270.1448	538.2984	269.6528	I	**618.3643**	309.6858	601.3378	301.1725			5

**7**	**655.3774**	328.1923	638.3508	319.6790	637.3668	319.1870	**V**	**505.2803**	253.1438	488.2537	244.6305			**4**
**8**	**783.4359**	392.2216	766.4094	383.7083	765.4254	383.2163	**Q**	**406.2119**	203.6096	**389.1853**	195.0963			**3**
**9**	914.4764	457.7418	897.4499	449.2286	896.4658	448.7366	**M**	**278.1533**	139.5803	261.1267	131.0670			**2**
**10**							**K**	147.1128	74.0600	130.0863	65.5468			**1**

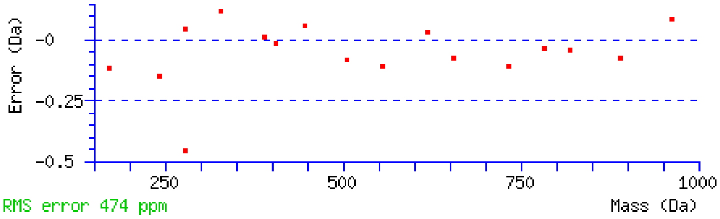

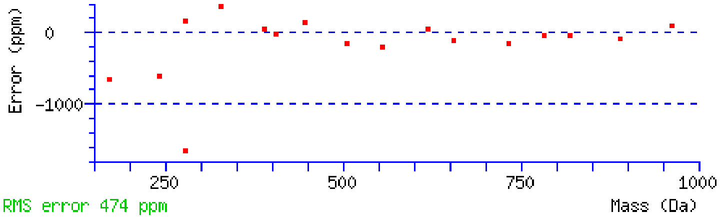

All matches to this query

Score	Mr(calc):	Delta	Sequence
78.8	1059.5746	0.0026	VAASNIVQMK
7.9	1059.5673	0.0099	KQEGIVGSSR
3.4	1058.5655	1.0117	XAAMATARVR
0.9	1057.5702	2.0070	GSLGCARPLK
0.5	1059.5798	-0.0026	VAEHRHRR
0.5	1059.5842	-0.0069	VAPSILKPR
0.5	1059.5785	-0.0013	VAQSESKRR
0.5	1059.5673	0.0099	VATKGTQAER
0.3	1057.5768	2.0005	QQIIATQEK

Spectrum No: 99; Query: 42830; Rank: 1

Peptide View

MS/MS Fragmentation of **YYTPTISR**
Found in **IPI00028006**, Tax_Id=9606 Gene_Symbol=PSMB2 Proteasome subunit beta type-2

Match to Query 42830: 999.505148 from(500.759850,2+)
Title: 090702LimSK_Exosome2_06.3090.3090.2.dta
Data file C:\Temp\Mascoti\LSK_2D\090702LimSK_Exosome2_01_2D.mgf

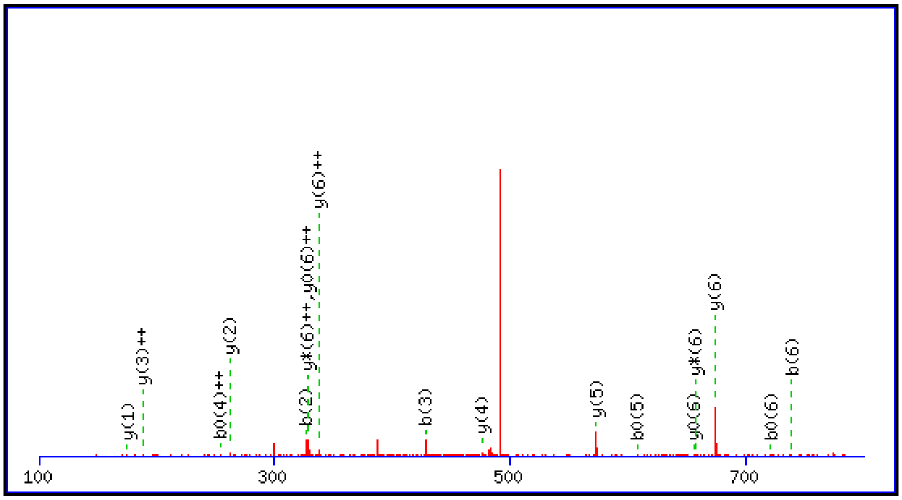

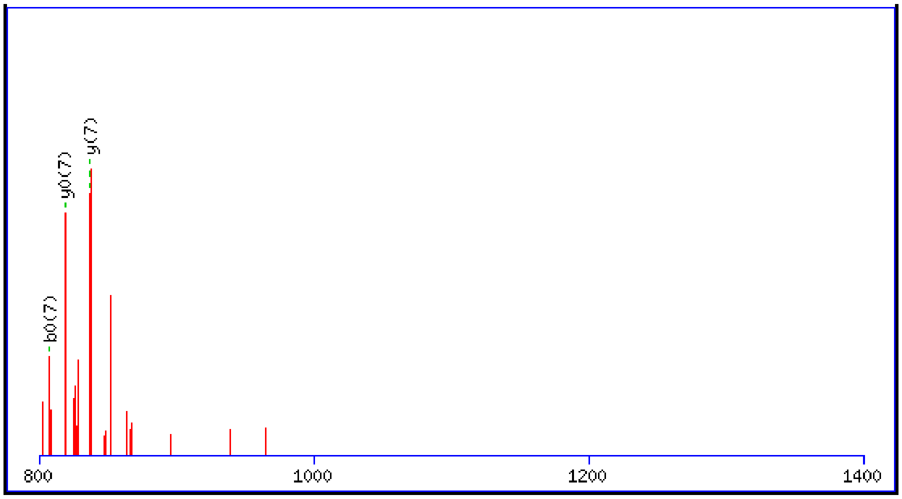

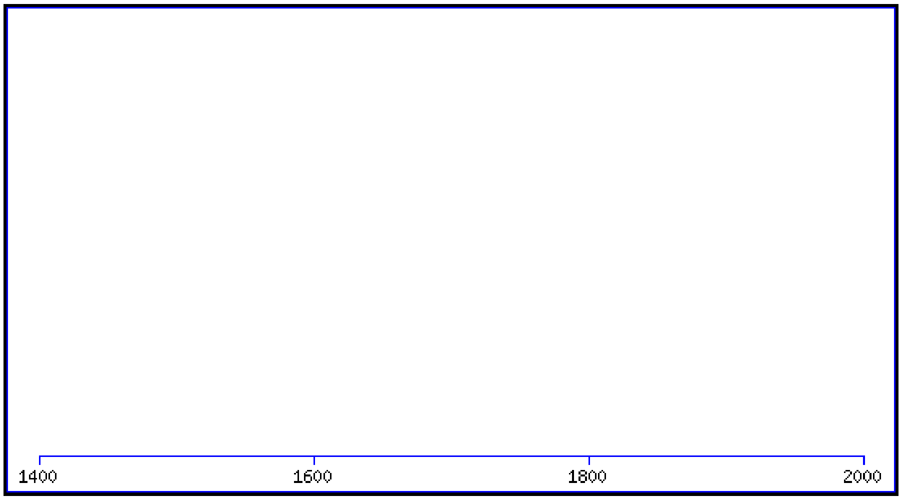

Monoisotopic mass of neutral peptide Mr(calc): 999.5025
Fixed modifications: Carbamidomethyl (C)
Ions Score: 24 **Expect:** 0.43
Matches (Bold Red): 20/64 fragment ions using 60 most intense peaks

#	b	b^{++}	b^{0}	b^{0++}	Seq.	y	y^{++}	y*	y^{*++}	y^{0}	y^{0++}	#
1	164.0706	82.5389			Y							8
2	**327.1339**	164.0706			Y	**837.4465**	419.2269	820.4199	410.7136	**819.4359**	410.2216	7
3	**428.1816**	214.5944	410.1710	205.5892	T	**674.3832**	**337.6952**	**657.3566**	**329.1819**	**656.3726**	**328.6899**	6
4	525.2344	263.1208	507.2238	**254.1155**	P	**573.3355**	287.1714	556.3089	278.6581	555.3249	278.1661	5
5	626.2821	313.6447	**608.2715**	304.6394	T	**476.2827**	238.6450	459.2562	230.1317	458.2722	229.6397	4
6	**739.3661**	370.1867	**721.3556**	361.1814	I	375.2350	**188.1212**	358.2085	179.6079	357.2245	179.1159	3
7	826.3981	413.7027	**808.3876**	404.6974	S	**262.1510**	131.5791	245.1244	123.0659	244.1404	122.5738	2
8					R	**175.1190**	88.0631	158.0924	79.5498			1

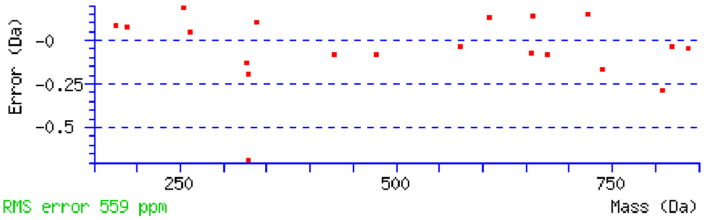

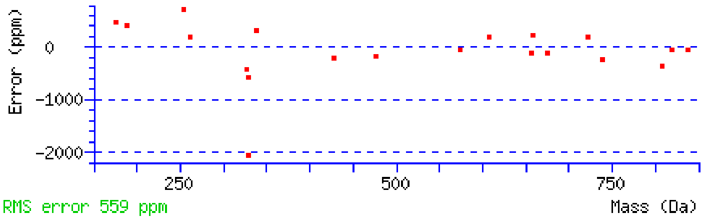

All matches to this query

Score	Mr(calc):	Delta	Sequence
23.9	999.5025	0.0026	YYTPTISR

14.8	998.4950	1.0101	GAVVTGFLR
14.7	999.4985	0.0066	EPVVGQDTR
12.6	998.4950	1.0101	KPVLGSYR
11.5	998.4968	1.0084	KPVQDCPR
11.5	998.4950	1.0101	KPVLGSYR
9.2	998.5046	1.0006	AGPWAEGRR
8.9	999.5076	-0.0024	ILVSLTMK
8.9	999.5076	-0.0024	ILVSLTMK
5.1	999.5015	0.0037	ARVYKQR

Spectrum No: 100; Query: 83887; Rank: 1

Peptide View

MS/MS Fragmentation of **AIGSASEGAQSSLQEVYHK**
Found in **IPI00291922**, Tax_Id=9606 Gene_Symbol=PSMA5 Proteasome subunit alpha type-5

Match to Query 83887: 1960.955052 from(654.658960,3+)
Title: 090702LimSK_Exosome2_03.5952.5952.3.dta
Data file C:\Temp\Mascoti\LSK_2D\090702LimSK_Exosome2_01_2D.mgf

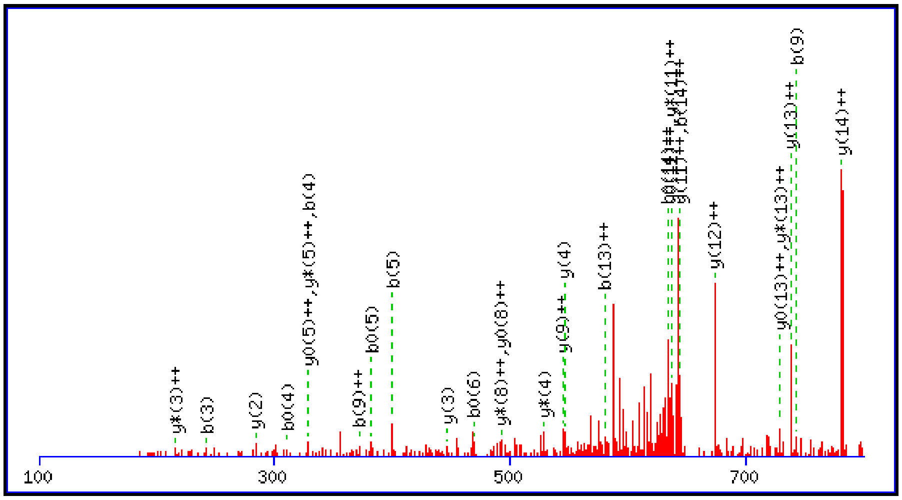

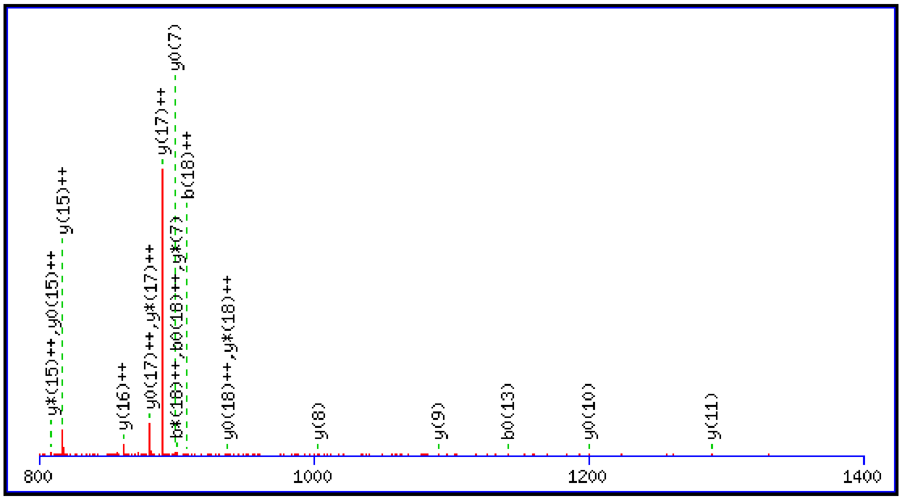

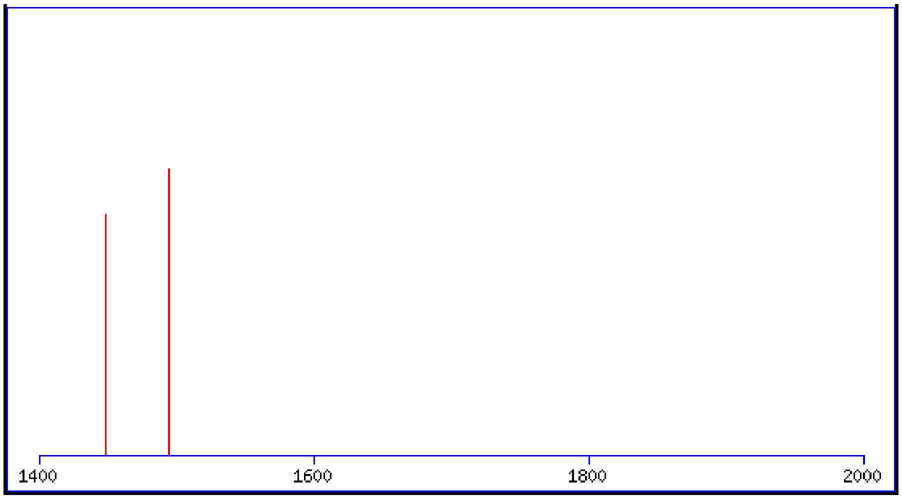

Monoisotopic mass of neutral peptide Mr(calc): 1960.9490
Fixed modifications: Carbamidomethyl (C)
Ions Score: 40 **Expect:** 0.025
Matches (Bold Red): 48/184 fragment ions using 98 most intense peaks

#	b	b^{++}	b^{*}	b^{*++}	b^{0}	b^{0++}	Seq.	y	y^{++}	y^{*}	y^{*++}	y^{0}	y^{0++}	#
1	72.0444	36.5258					A							19
2	185.1285	93.0679					I	1890.9192	945.9632	1873.8926	**937.4500**	1872.9086	**936.9580**	18
3	**242.1499**	121.5786					G	1777.8351	**889.4212**	1760.8086	**880.9079**	1759.8246	**880.4159**	17
4	**329.1819**	165.0946			**311.1714**	156.0893	S	1720.8137	**860.9105**	1703.7871	852.3972	1702.8031	851.9052	16
5	**400.2191**	200.6132			**382.2085**	191.6079	A	1633.7816	**817.3945**	1616.7551	**808.8812**	1615.7711	**808.3892**	15
6	487.2511	244.1292			**469.2405**	235.1239	S	1562.7445	**781.8759**	1545.7180	773.3626	1544.7340	772.8706	14
7	616.2937	308.6505			598.2831	299.6452	E	1475.7125	**738.3599**	1458.6860	**729.8466**	1457.7019	**729.3546**	13
8	673.3151	337.1612			655.3046	328.1559	G	1346.6699	**673.8386**	1329.6434	665.3253	1328.6593	664.8333	12
9	**744.3523**	**372.6798**			726.3417	363.6745	A	**1289.6484**	**645.3279**	1272.6219	**636.8146**	1271.6379	**636.3226**	11
10	872.4108	436.7091	855.3843	428.1958	854.4003	427.7038	Q	1218.6113	609.8093	1201.5848	601.2960	**1200.6008**	600.8040	10
11	959.4429	480.2251	942.4163	471.7118	941.4323	471.2198	S	**1090.5528**	**545.7800**	1073.5262	537.2667	1072.5422	536.7747	9
12	1046.4749	523.7411	1029.4483	515.2278	1028.4643	514.7358	S	**1003.5207**	502.2640	986.4942	**493.7507**	985.5102	**493.2587**	8
13	1159.5590	**580.2831**	1142.5324	571.7698	**1141.5484**	571.2778	L	916.4887	458.7480	**899.4621**	450.2347	**898.4781**	449.7427	7
14	1287.6175	**644.3124**	1270.5910	635.7991	1269.6070	**635.3071**	Q	803.4046	402.2060	786.3781	393.6927	785.3941	393.2007	6
15	1416.6601	708.8337	1399.6336	700.3204	1398.6496	699.8284	E	675.3461	338.1767	658.3195	**329.6634**	657.3355	**329.1714**	5
16	1515.7285	758.3679	1498.7020	749.8546	1497.7180	749.3626	V	**546.3035**	273.6554	**529.2769**	265.1421			4
17	1678.7919	839.8996	1661.7653	831.3863	1660.7813	830.8943	Y	**447.2350**	224.1212	430.2085	**215.6079**			3
18	1815.8508	**908.4290**	1798.8242	**899.9158**	1797.8402	**899.4237**	H	**284.1717**	142.5895	267.1452	134.0762			2
19							K	147.1128	74.0600	130.0863	65.5468			1

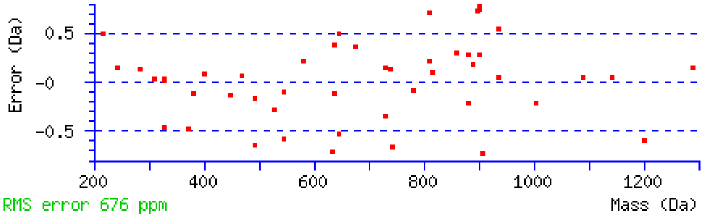

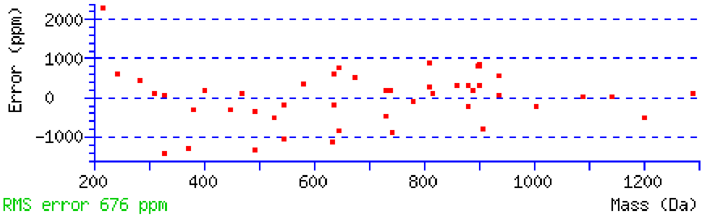

All matches to this query

Score	Mr(calc):	Delta	Sequence
39.7	1960.9490	0.0060	AIGSASEGAQSSLQEVYHK
10.6	1958.9462	2.0088	DLREKSSPSGLLTSSFR
8.4	1960.9592	-0.0042	SAGGRPGSGPQLGTGRGTLR

6.0	1959.9376	1.0174	AILNISKAMEQEFSATK
4.9	1960.9408	0.0143	SEHKVWSPLVTEEGKR
4.7	1960.9547	0.0004	STLINSLFLTDLYPER
4.6	1959.9415	1.0136	SPARPSESRLAPTPTEGK
4.1	1960.9710	-0.0160	QLSAQGQMTQLCELINK
3.5	1959.9336	1.0214	SKTSLTKGATTDNGIQMK
3.2	1960.9592	-0.0042	SAGGRPGSGPQLGTGRGTLR

Spectrum No: 101; Query: 56288; Rank: 1

Peptide View

MS/MS Fragmentation of **EELEEVIKDI**
Found in **IPI00291922**, Tax_Id=9606 Gene_Symbol=PSMA5 Proteasome subunit alpha type-5

Match to Query 56288: 1215.626268 from(608.820410,2+)
Title: 090702LimSK_Exosome2_06.8439.8439.2.dta
Data file C:\Temp\Mascoti\LSK_2D\090702LimSK_Exosome2_01_2D.mgf

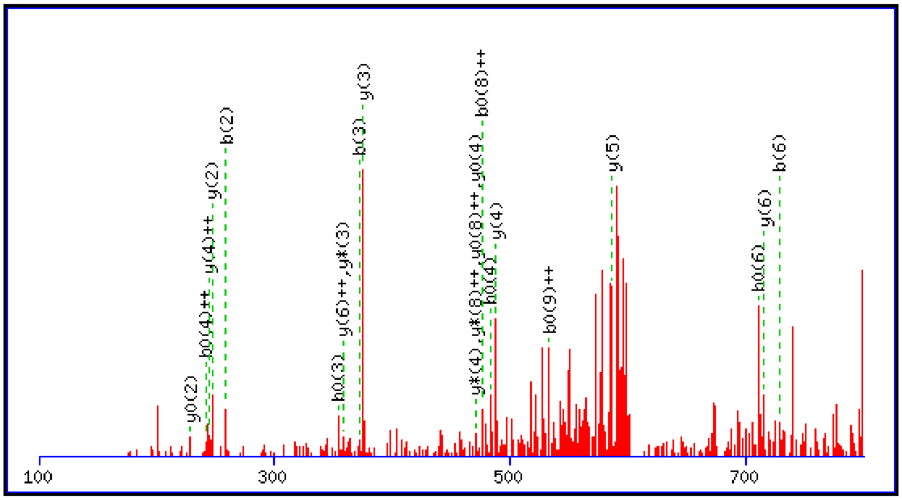

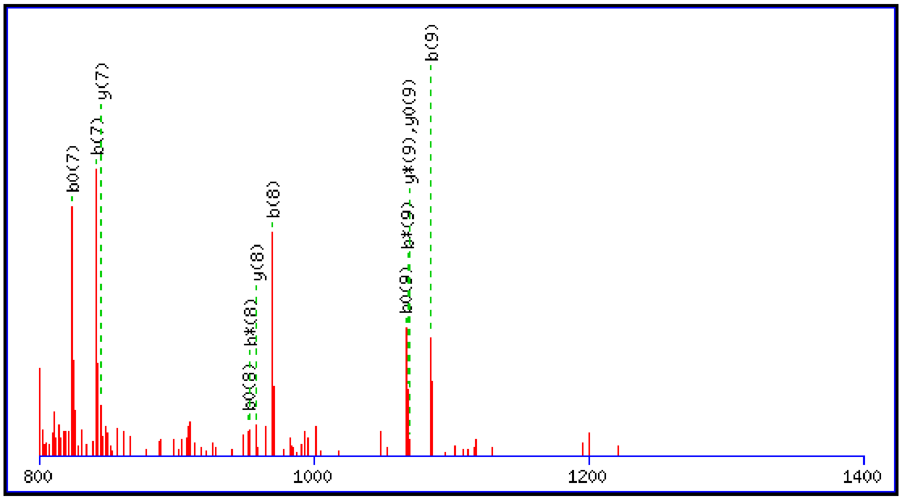

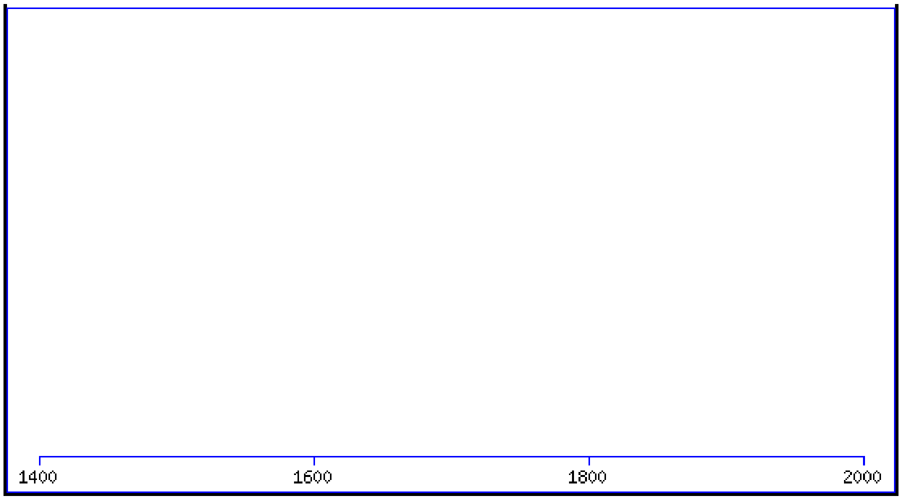

Monoisotopic mass of neutral peptide Mr(calc): 1215.6234
Fixed modifications: Carbamidomethyl (C)
Ions Score: 22 **Expect:** 0.87
Matches (Bold Red): 34/88 fragment ions using 95 most intense peaks

#	b	b^{++}	b*	b^{*++}	b^0	b^{0++}	Seq.	y	y^{++}	y*	y^{*++}	y^0	y^{0++}	#
1	130.0499	65.5286			112.0393	56.5233	E							10
2	**259.0925**	130.0499			241.0819	121.0446	E	1087.5881	544.2977	**1070.5616**	535.7844	**1069.5776**	535.2924	9
3	**372.1765**	186.5919			**354.1660**	177.5866	L	**958.5455**	479.7764	941.5190	**471.2631**	940.5350	**470.7711**	8
4	501.2191	251.1132			**483.2086**	**242.1079**	E	**845.4615**	423.2344	828.4349	414.7211	827.4509	414.2291	7
5	630.2617	315.6345			612.2511	306.6292	E	**716.4189**	**358.7131**	699.3923	350.1998	698.4083	349.7078	6
6	**729.3301**	365.1687			**711.3196**	356.1634	V	**587.3763**	294.1918	570.3497	285.6785	569.3657	285.1865	5
7	**842.4142**	421.7107			**824.4036**	412.7055	I	**488.3079**	**244.6576**	**471.2813**	236.1443	**470.2973**	235.6523	4
8	**970.5092**	485.7582	**953.4826**	477.2449	**952.4986**	**476.7529**	K	**375.2238**	188.1155	**358.1973**	179.6023	357.2132	179.1103	3
9	**1085.5361**	543.2717	**1068.5095**	534.7584	**1067.5255**	**534.2664**	D	**247.1288**	124.0681			**229.1183**	115.0628	2
10							I	132.1019	66.5546					1

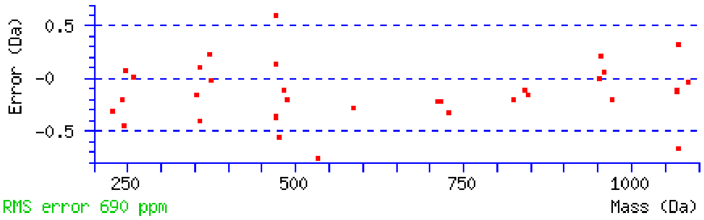

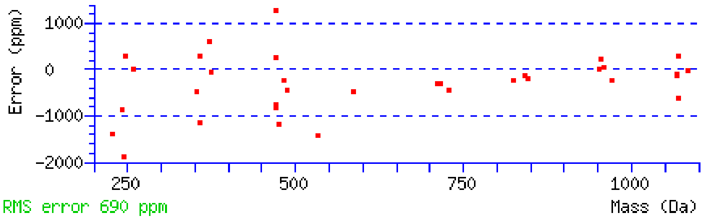

All matches to this query

Score	Mr(calc):	Delta	Sequence
22.2	1215.6234	0.0029	EELEEVIKDI
6.6	1213.6294	1.9969	MVKLVWKSK
6.4	1213.6220	2.0043	RYSLAVGLQK
5.8	1213.6107	2.0155	YTPKEQLKK
5.2	1214.6190	1.0073	MAEGSRIPQAR
4.0	1213.6076	2.0187	MSLRVCLKK
2.0	1215.6281	-0.0019	MALTPTNLNNK
1.9	1214.6156	1.0107	EELRGAGWAAR
1.8	1213.6108	2.0155	SLNILTAFQK
1.6	1213.6190	2.0072	ELLGLSDEGGPK

Spectrum No: 102; Query: 87669; Rank: 1

Peptide View

MS/MS Fragmentation of **GPQLFHMDPSGTFVQCDAR**
Found in **IPI00291922**, Tax_Id=9606 Gene_Symbol=PSMA5 Proteasome subunit alpha type-5

Match to Query 87669: 2161.975272 from(721.665700,3+)
Title: 090702LimSK_Exosome2_01.6911.6911.3.dta
Data file C:\Temp\Mascoti\LSK_2D\090702LimSK_Exosome2_01_2D.mgf

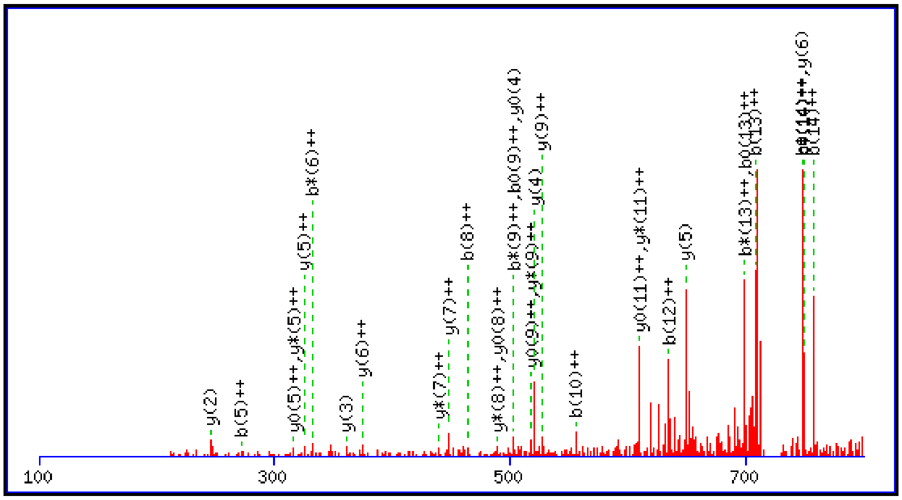

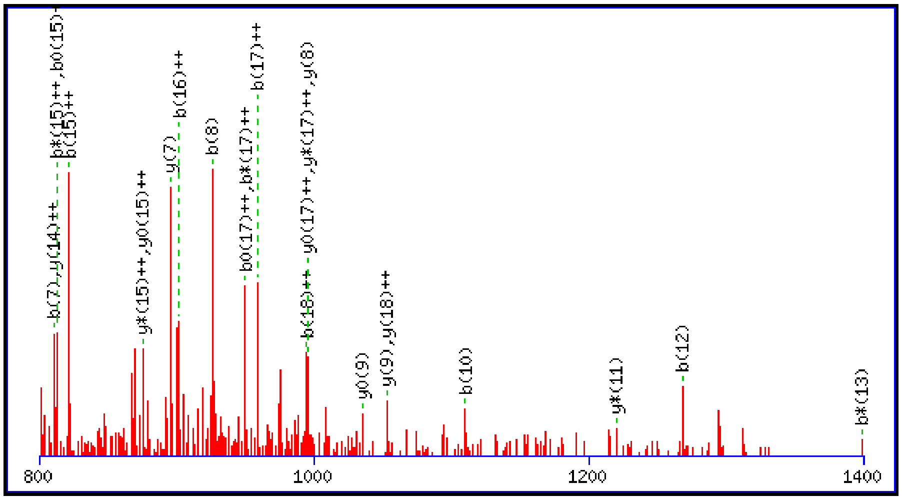

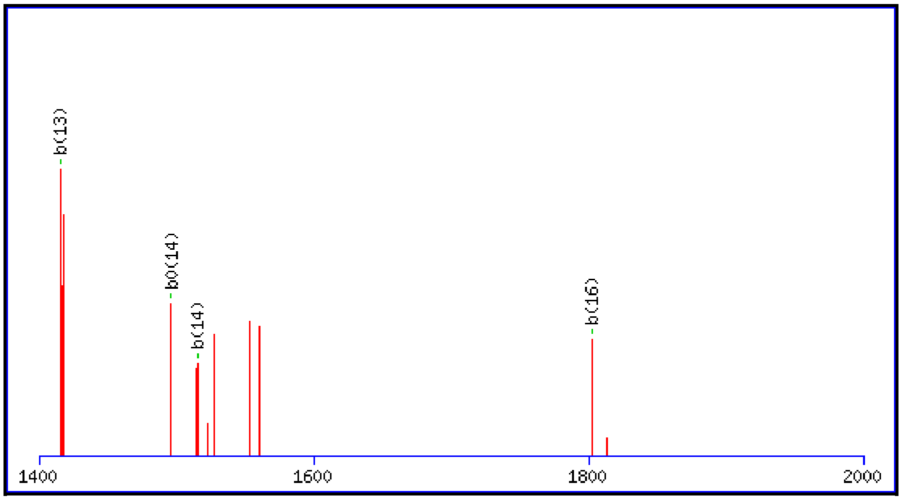

Monoisotopic mass of neutral peptide Mr(calc): 2161.9674
Fixed modifications: Carbamidomethyl (C)
Ions Score: 39 **Expect:** 0.033

Matches (Bold Red): 60/194 fragment ions using 99 most intense peaks

#	b	b^{++}	b*	b^{*++}	b^0	b^{0++}	Seq.	y	y^{++}	y*	y^{*++}	y^0	y^{0++}	#
1	58.0287	29.5180					G							19
2	155.0815	78.0444					P	2105.9532	**1053.4802**	2088.9266	1044.9669	2087.9426	1044.4749	18
3	283.1401	142.0737	266.1135	133.5604			Q	2008.9004	1004.9538	1991.8738	**996.4406**	1990.8898	**995.9486**	17
4	396.2241	198.6157	379.1976	190.1024			L	1880.8418	940.9245	1863.8153	932.4113	1862.8313	931.9193	16
5	543.2926	**272.1499**	526.2660	263.6366			F	1767.7578	884.3825	1750.7312	**875.8692**	1749.7472	**875.3772**	15
6	680.3515	340.6794	663.3249	**332.1661**			H	1620.6893	**810.8483**	1603.6628	802.3350	1602.6788	801.8430	14
7	**811.3920**	406.1996	794.3654	397.6863			M	1483.6304	742.3189	1466.6039	733.8056	1465.6199	733.3136	13
8	**926.4189**	**463.7131**	909.3924	455.1998	908.4083	454.7078	D	1352.5899	676.7986	1335.5634	668.2853	1334.5794	667.7933	12
9	1023.4717	512.2395	1006.4451	**503.7262**	1005.4611	**503.2342**	P	1237.5630	619.2851	**1220.5365**	**610.7719**	1219.5524	**610.2799**	11
10	**1110.5037**	**555.7555**	1093.4771	547.2422	1092.4931	546.7502	S	1140.5102	570.7588	1123.4837	562.2455	1122.4997	561.7535	10
11	1167.5252	584.2662	1150.4986	575.7529	1149.5146	575.2609	G	**1053.4782**	**527.2427**	1036.4517	**518.7295**	**1035.4676**	**518.2375**	9
12	**1268.5728**	**634.7901**	1251.5463	626.2768	1250.5623	625.7848	T	**996.4567**	498.7320	979.4302	**490.2187**	978.4462	**489.7267**	8
13	**1415.6412**	**708.3243**	**1398.6147**	**699.8110**	1397.6307	**699.3190**	F	**895.4091**	**448.2082**	878.3825	**439.6949**	877.3985	439.2029	7
14	**1514.7097**	**757.8585**	1497.6831	**749.3452**	**1496.6991**	**748.8532**	V	**748.3407**	**374.6740**	731.3141	366.1607	730.3301	365.6687	6
15	1642.7682	**821.8878**	1625.7417	**813.3745**	1624.7577	**812.8825**	Q	**649.2722**	**325.1398**	632.2457	**316.6265**	631.2617	**316.1345**	5
16	**1802.7989**	**901.9031**	1785.7723	893.3898	1784.7883	892.8978	C	**521.2137**	261.1105	504.1871	252.5972	**503.2031**	252.1052	4
17	1917.8258	**959.4166**	1900.7993	**950.9033**	1899.8153	**950.4113**	D	**361.1830**	181.0951	344.1565	172.5819	343.1724	172.0899	3
18	1988.8629	**994.9351**	1971.8364	986.4218	1970.8524	985.9298	A	**246.1561**	123.5817	229.1295	115.0684			2
19							R	175.1190	88.0631	158.0924	79.5498			1

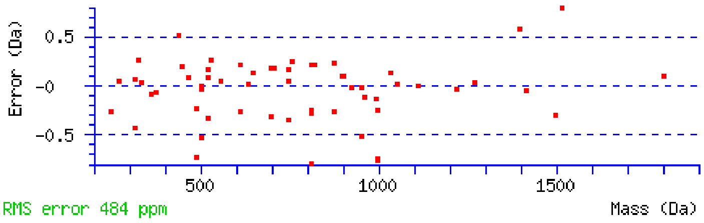

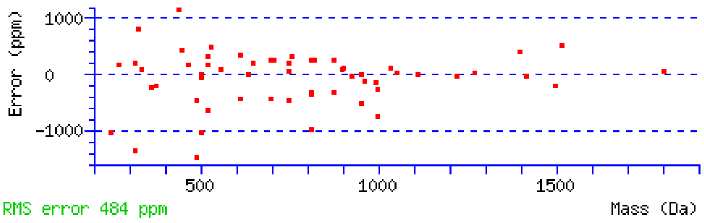

All matches to this query

Score	Mr(calc):	Delta	Sequence
39.3	2161.9674	0.0079	GPQLFHMDPSGTFVQCDAR
15.7	2160.9924	0.9829	IMNMGTPCTPLKFPFTAR
8.9	2161.9728	0.0024	VRPDSLCFGPAGSGGLHEAR
8.0	2160.9929	0.9823	RSLPNLSRTSNTQVDSVK
5.6	2161.9892	-0.0140	DLPLPNTIQDVENEKDSR
4.3	2161.9745	0.0008	NRPDSVISSKMFLSIHR
4.3	2160.9720	1.0032	GVFWASPILSSGCLSVPPK
4.3	2160.9762	0.9991	ASASYHISNLLEKMTSSDK
4.2	2159.9785	1.9968	MIECLRSNFIYGSYLSK
4.0	2160.9720	1.0032	GVFWASPILSSGCLSVPPK

Spectrum No: 103; Query: 45746; Rank: 1

Peptide View

MS/MS Fragmentation of **GVNTFSPEGR**
Found in **IPI00291922**, Tax_Id=9606 Gene_Symbol=PSMA5 Proteasome subunit alpha type-5

Match to Query 45746: 1062.510528 from(532.262540,2+)
Title: 090702LimSK_Exosome2_06.2262.2262.2.dta
Data file C:\Temp\Mascoti\LSK_2D\090702LimSK_Exosome2_01_2D.mgf

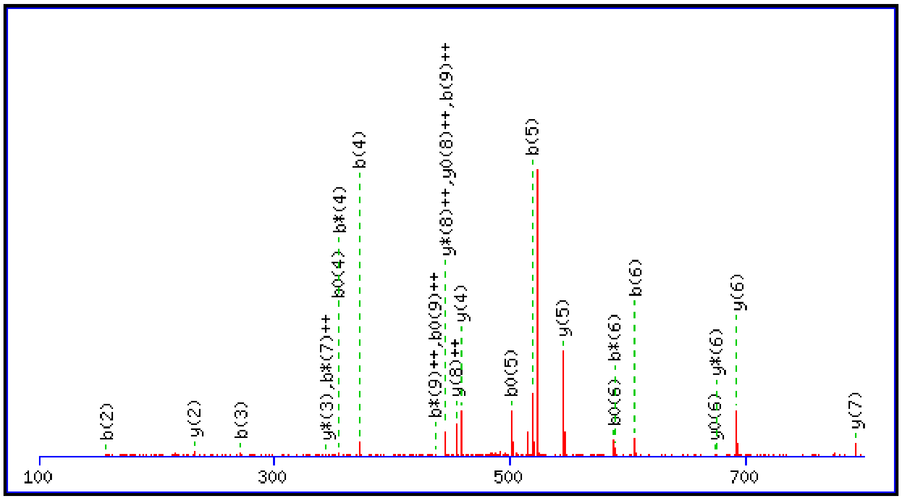

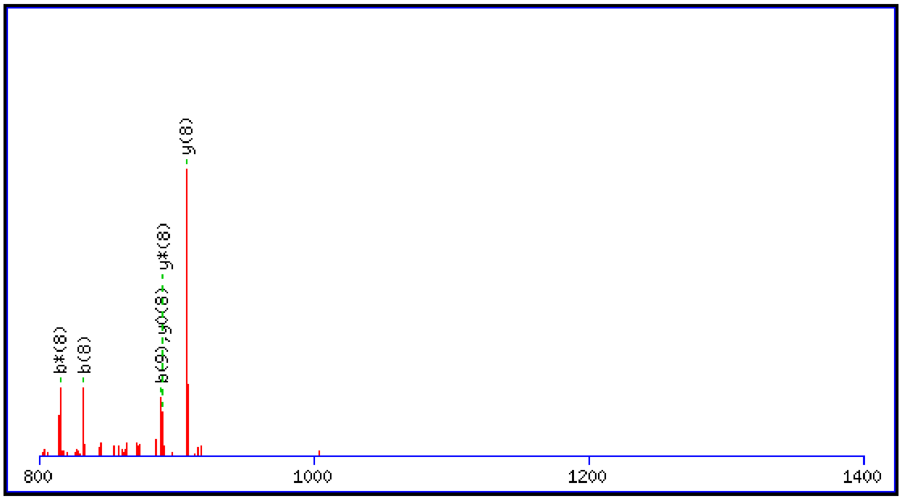

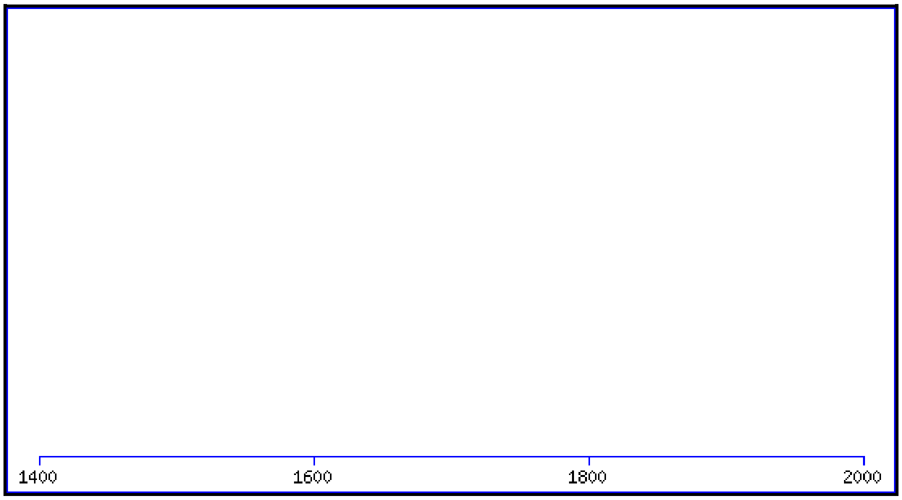

Monoisotopic mass of neutral peptide Mr(calc): 1062.5094
Fixed modifications: Carbamidomethyl (C)
Ions Score: 57 **Expect:** 0.00031
Matches (Bold Red): 31/94 fragment ions using 37 most intense peaks

#	b	b^{++}	b*	b^{*++}	b^{0}	b^{0++}	Seq.	y	y^{++}	y*	y^{*++}	y^{0}	y^{0++}	#
1	58.0287	29.5180					G							10
2	**157.0972**	79.0522					V	1006.4952	503.7513	989.4687	495.2380	988.4847	494.7460	9
3	**271.1401**	136.0737	254.1135	127.5604			N	**907.4268**	**454.2170**	**890.4003**	**445.7038**	**889.4163**	**445.2118**	8

**4**	**372.1878**	186.5975	**355.1612**	178.0842	**354.1772**	177.5922	**T**	**793.3839**	397.1956	776.3573	388.6823	775.3733	388.1903	**7**
**5**	**519.2562**	260.1317	502.2296	251.6185	**501.2456**	251.1264	**F**	**692.3362**	346.6717	**675.3097**	338.1585	**674.3257**	337.6665	**6**
**6**	**606.2882**	303.6477	**589.2617**	295.1345	**588.2776**	294.6425	**S**	**545.2678**	273.1375	528.2413	264.6243	527.2572	264.1323	**5**
**7**	703.3410	352.1741	686.3144	**343.6608**	685.3304	343.1688	**P**	**458.2358**	229.6215	441.2092	221.1083	440.2252	220.6162	**4**
**8**	**832.3836**	416.6954	**815.3570**	408.1821	814.3730	407.6901	**E**	361.1830	181.0951	**344.1565**	172.5819	343.1724	172.0899	**3**
**9**	**889.4050**	**445.2061**	872.3785	**436.6929**	871.3945	**436.2009**	**G**	**232.1404**	116.5738	215.1139	108.0606			**2**
**10**							**R**	175.1190	88.0631	158.0924	79.5498			**1**

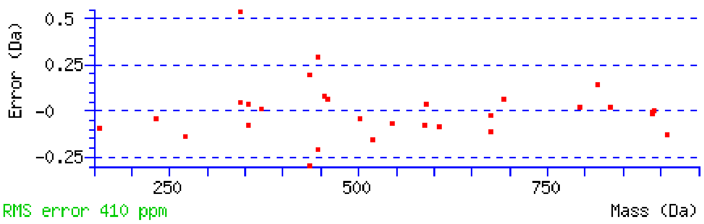

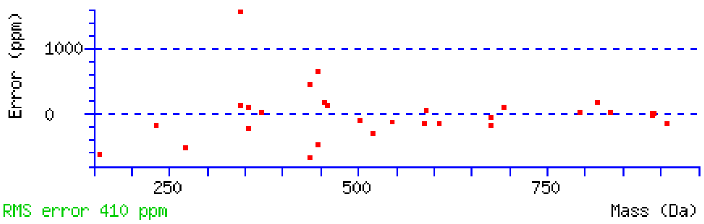

All matches to this query

Score	Mr(calc):	Delta	Sequence
56.6	1062.5094	0.0011	GVNTFSPEGR
11.5	1060.4938	2.0168	GPPPSYGGSSR
11.0	1062.5094	0.0011	GAKDFASDPR
9.9	1062.5128	-0.0022	NKEMEQLR
9.4	1062.5101	0.0004	GSARGCGRSR
8.6	1062.5101	0.0004	CRGAGSRGSR
8.5	1061.5019	1.0086	GIHSKTPSR
7.0	1062.5084	0.0022	RATHSAGKR
6.5	1062.5094	0.0011	VATNPSFDGR
5.9	1061.5019	1.0087	KANAAPSAPR

Spectrum No: 104; Query: 65973; Rank: 1

Peptide View

MS/MS Fragmentation of **ITSPLMEPSSIEK**
Found in **IPI00291922**, Tax_Id=9606 Gene_Symbol=PSMA5 Proteasome subunit alpha type-5

Match to Query 65973: 1430.735448 from(716.375000,2+)
Title: 090702LimSK_Exosome2_06.5431.5431.2.dta
Data file C:\Temp\Mascoti\LSK_2D\090702LimSK_Exosome2_01_2D.mgf

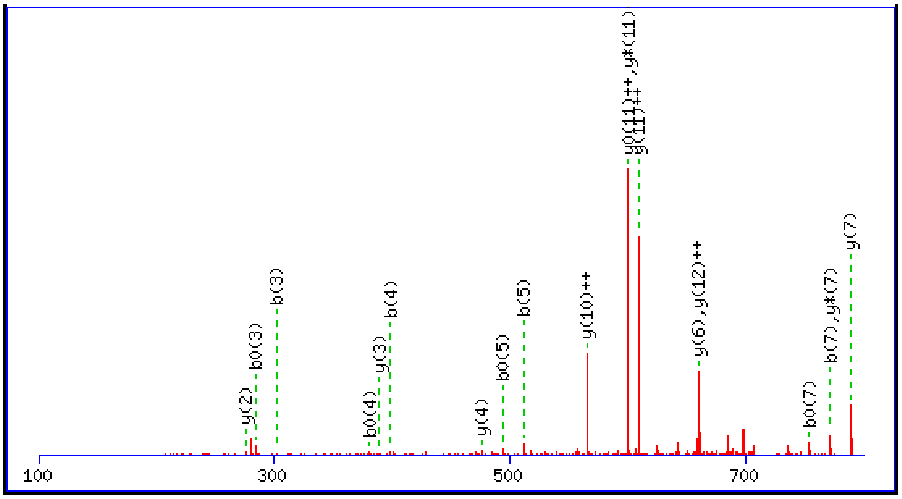

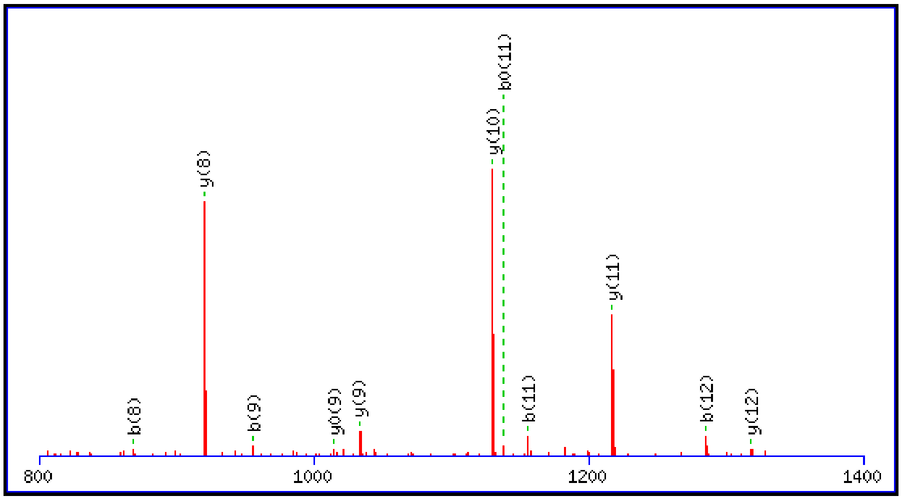

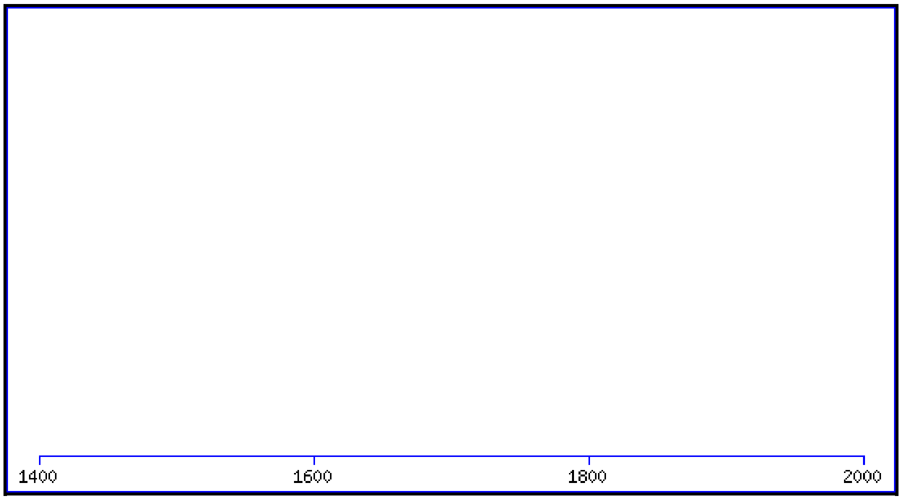

Monoisotopic mass of neutral peptide Mr(calc): 1430.7327
Fixed modifications: Carbamidomethyl (C)
Ions Score: 74 **Expect:** 6.2e-006
Matches (Bold Red): 30/116 fragment ions using 50 most intense peaks

#	b	b++	b0	b0++	Seq.	y	y++	y*	y*++	y0	y0++	#
1	114.0913	57.5493			I							13
2	215.1390	108.0731	197.1285	99.0679	T	**1318.6559**	**659.8316**	1301.6293	651.3183	1300.6453	650.8263	12
3	**302.1710**	151.5892	**284.1605**	142.5839	S	**1217.6082**	**609.3077**	1200.5817	**600.7945**	1199.5976	**600.3025**	11
4	**399.2238**	200.1155	**381.2132**	191.1103	P	**1130.5762**	**565.7917**	1113.5496	557.2785	1112.5656	556.7864	10
5	**512.3079**	256.6576	**494.2973**	247.6523	L	**1033.5234**	517.2653	1016.4969	508.7521	**1015.5129**	508.2601	9
6	643.3484	322.1778	625.3378	313.1725	M	**920.4394**	460.7233	903.4128	452.2100	902.4288	451.7180	8

7	772.3910	386.6991	754.3804	377.6938	E	789.3989	395.2031	772.3723	386.6898	771.3883	386.1978	7
8	869.4437	435.2255	851.4332	426.2202	P	660.3563	330.6818	643.3297	322.1685	642.3457	321.6765	6
9	956.4757	478.7415	938.4652	469.7362	S	563.3035	282.1554	546.2770	273.6421	545.2930	273.1501	5
10	1043.5078	522.2575	1025.4972	513.2522	S	476.2715	238.6394	459.2449	230.1261	458.2609	229.6341	4
11	1156.5918	578.7996	1138.5813	569.7943	I	389.2395	195.1234	372.2129	186.6101	371.2289	186.1181	3
12	1285.6344	643.3209	1267.6239	634.3156	E	276.1554	138.5813	259.1288	130.0681	258.1448	129.5761	2
13					K	147.1128	74.0600	130.0863	65.5468			1

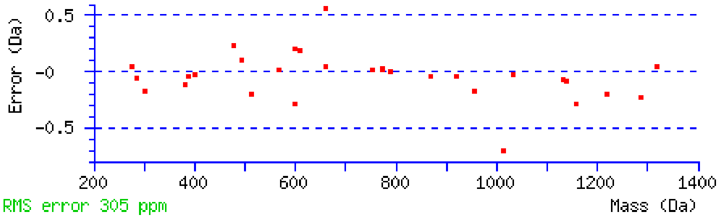

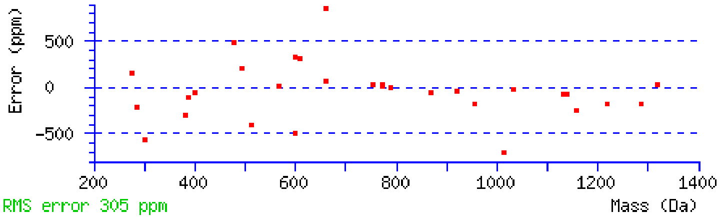

All matches to this query

Score	Mr(calc):	Delta	Sequence
73.9	1430.7327	0.0028	ITSPLMEPSSIEK
10.7	1429.7460	0.9895	VNSPIRMKNGNGK
7.7	1430.7226	0.0129	DVSDLERGAGRTR
5.2	1430.7327	0.0028	GMITVTDPDLIEK
3.8	1429.7330	1.0024	ALVLNTPNNPLGK
3.7	1430.7283	0.0072	ELQIVRTLDHK
2.9	1430.7365	-0.0010	RELDSIEAELTR
1.7	1429.7193	1.0162	LVLACVIPYFR
1.5	1429.7330	1.0025	NIANSLPNVALPK
0.9	1430.7365	-0.0010	LTIRKEEDGDAGK

Spectrum No: 105; Query: 65480; Rank: 1

Peptide View

MS/MS Fragmentation of **LFQVEYAIEAIK**
Found in **IPI00291922**, Tax_Id=9606 Gene_Symbol=PSMA5 Proteasome subunit alpha type-5

Match to Query 65480: 1422.776948 from(712.395750,2+)
Title: 090702LimSK_Exosome2_06.8551.8551.2.dta
Data file C:\Temp\Mascoti\LSK_2D\090702LimSK_Exosome2_01_2D.mgf

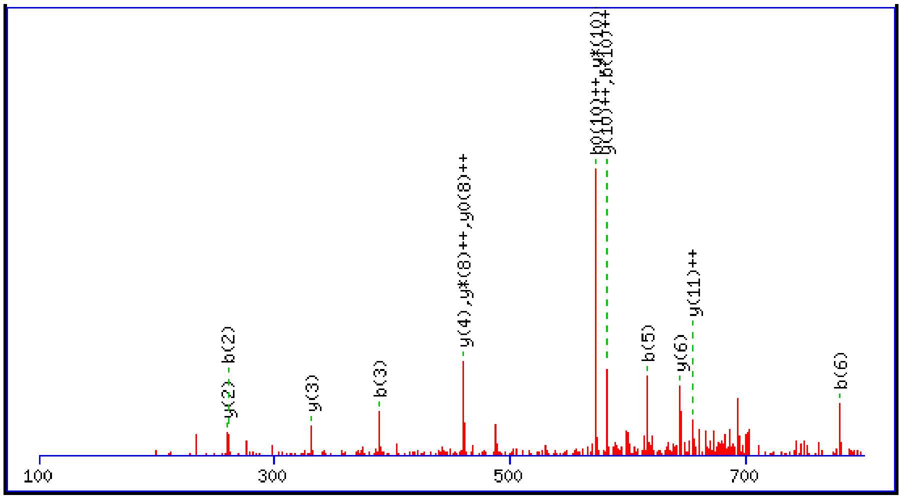

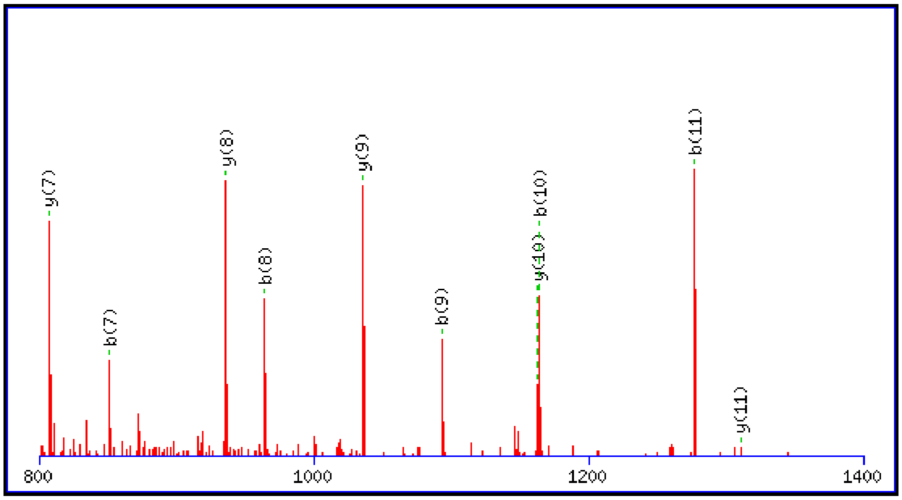

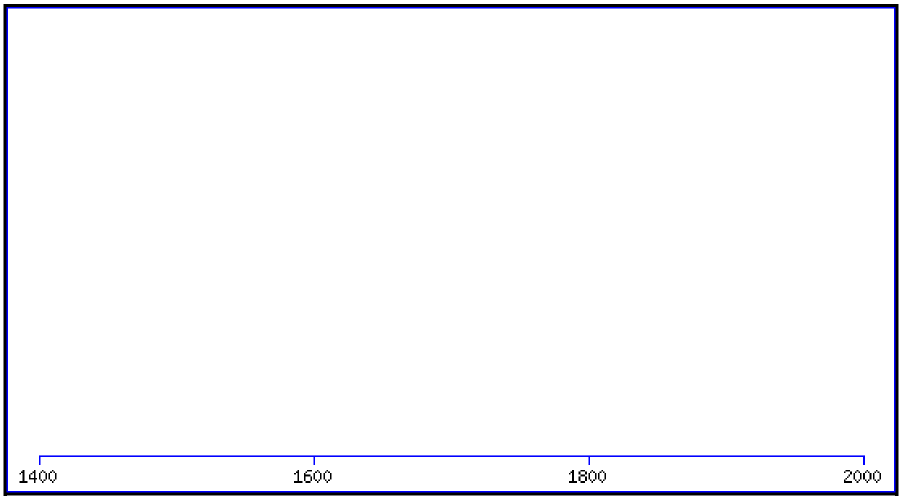

Monoisotopic mass of neutral peptide Mr(calc): 1422.7758
Fixed modifications: Carbamidomethyl (C)
Ions Score: 92 **Expect:** 5.8e-008
Matches (Bold Red): 28/114 fragment ions using 30 most intense peaks

#	b	b^{++}	b*	b^{*++}	b^{0}	b^{0++}	Seq.	y	y^{++}	y*	y^{*++}	y^{0}	y^{0++}	#
1	114.0913	57.5493					L							12
2	**261.1598**	131.0835					F	**1310.6991**	**655.8532**	1293.6725	647.3399	1292.6885	646.8479	11
3	**389.2183**	195.1128	372.1918	186.5995			Q	**1163.6307**	**582.3190**	1146.6041	**573.8057**	1145.6201	**573.3137**	10
4	488.2867	244.6470	471.2602	236.1337			V	**1035.5721**	518.2897	1018.5455	509.7764	1017.5615	509.2844	9
5	**617.3293**	309.1683	600.3028	300.6550	599.3188	300.1630	E	**936.5037**	468.7555	919.4771	**460.2422**	918.4931	**459.7502**	8
6	**780.3927**	390.7000	763.3661	382.1867	762.3821	381.6947	Y	**807.4611**	404.2342	790.4345	395.7209	789.4505	395.2289	7

7	851.4298	426.2185	834.4032	417.7053	833.4192	417.2132	A	644.3978	322.7025	627.3712	314.1892	626.3872	313.6972	6
8	964.5138	482.7606	947.4873	474.2473	946.5033	473.7553	I	573.3606	287.1840	556.3341	278.6707	555.3501	278.1787	5
9	1093.5564	547.2819	1076.5299	538.7686	1075.5459	538.2766	E	460.2766	230.6419	443.2500	222.1287	442.2660	221.6366	4
10	1164.5936	582.8004	1147.5670	574.2871	1146.5830	573.7951	A	331.2340	166.1206	314.2074	157.6074			3
11	1277.6776	639.3424	1260.6511	630.8292	1259.6671	630.3372	I	260.1969	130.6021	243.1703	122.0888			2
12							K	147.1128	74.0600	130.0863	65.5468			1

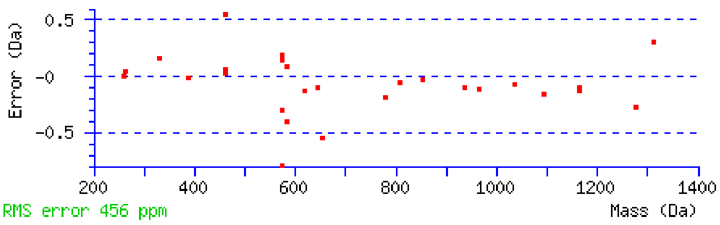

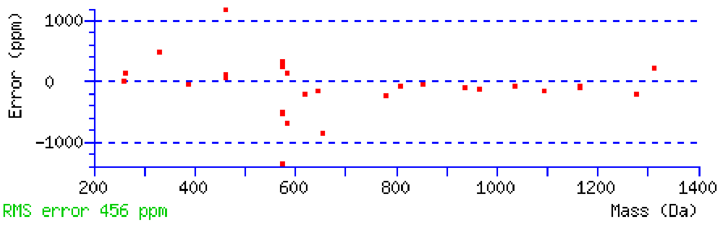

All matches to this query

Score	Mr(calc):	Delta	Sequence
92.1	1422.7758	0.0011	LFQVEYAIEAIK
14.1	1421.7739	1.0031	KQEQRHEVIQK
11.4	1422.7718	0.0051	AHLEEISALVVDK
11.0	1421.7813	0.9957	SRLRMFLNELK
10.7	1421.7643	1.0127	KNGTNVIVEKLK
9.1	1422.7878	-0.0108	VGHRMNIERALK
9.0	1422.7752	0.0018	MLKGISSSSLKEK
8.9	1422.7904	-0.0135	IMKGLNAYLEKK
8.5	1422.7732	0.0038	QFGHPQIEARIK
8.3	1422.7710	0.0060	VVWMQTLLILK

Spectrum No: 106; Query: 31051; Rank: 1

Peptide View

MS/MS Fragmentation of **SSLIILK**
Found in **IPI00291922**, Tax_Id=9606 Gene_Symbol=PSMA5 Proteasome subunit alpha type-5

Match to Query 31051: 772.506708 from(387.260630,2+)
Title: 090702LimSK_Exosome2_06.4990.4990.2.dta
Data file C:\Temp\Mascoti\LSK_2D\090702LimSK_Exosome2_01_2D.mgf

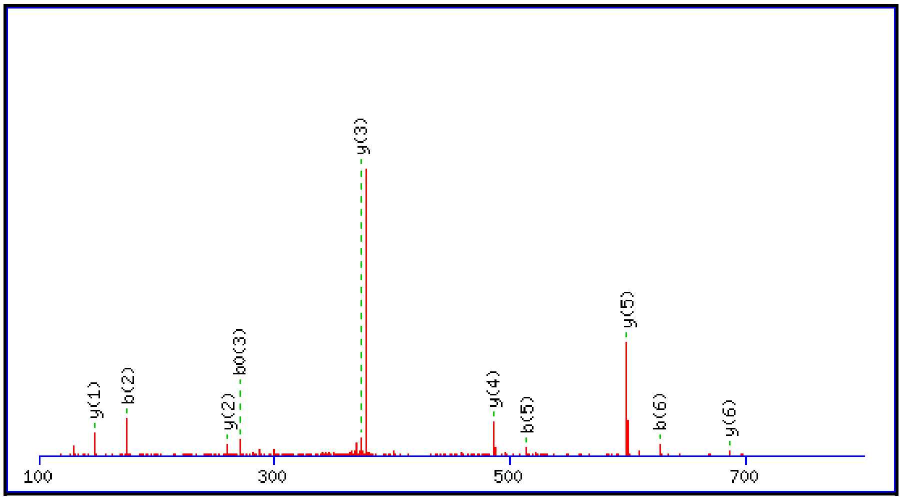

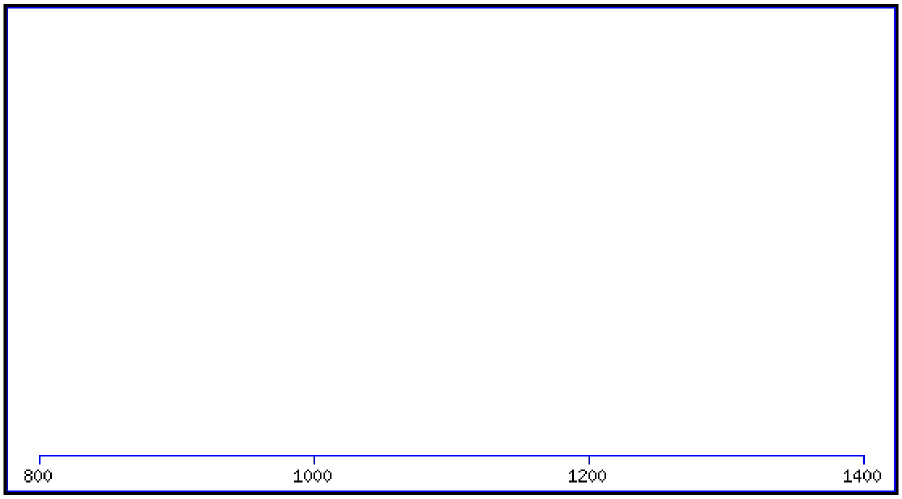

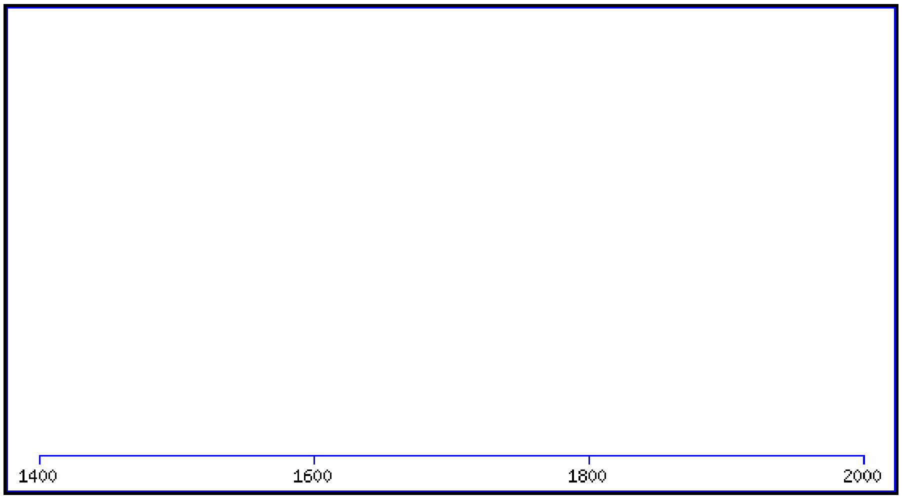

Monoisotopic mass of neutral peptide Mr(calc): 772.5058
Fixed modifications: Carbamidomethyl (C)
Ions Score: 67 **Expect:** 1e-005
Matches (Bold Red): 10/50 fragment ions using 12 most intense peaks

#	b	b^{++}	b^0	b^{0++}	Seq.	y	y^{++}	y*	y^{*++}	y^0	y^{0++}	#
1	88.0393	44.5233	70.0287	35.5180	S							7
2	**175.0713**	88.0393	157.0608	79.0340	S	**686.4811**	343.7442	669.4545	335.2309	668.4705	334.7389	6
3	288.1554	144.5813	**270.1448**	135.5761	L	**599.4491**	300.2282	582.4225	291.7149			5
4	401.2395	201.1234	383.2289	192.1181	I	**486.3650**	243.6861	469.3384	235.1729			4
5	**514.3235**	257.6654	496.3130	248.6601	I	**373.2809**	187.1441	356.2544	178.6308			3
6	**627.4076**	314.2074	609.3970	305.2021	L	**260.1969**	130.6021	243.1703	122.0888			2
7					K	**147.1128**	74.0600	130.0863	65.5468			1

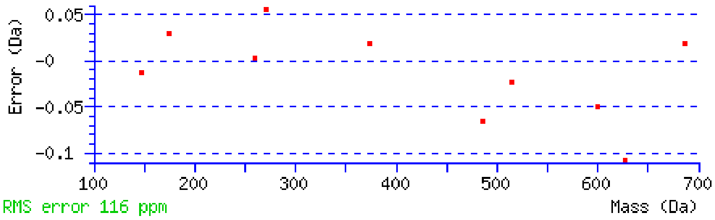

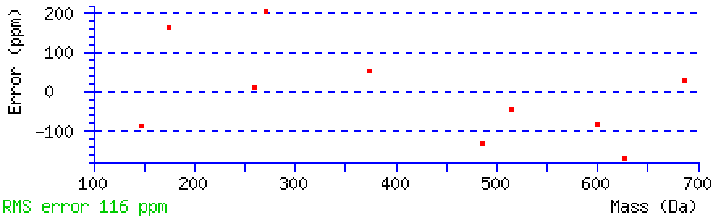

All matches to this query

Score	Mr(calc):	Delta	Sequence
66.6	772.5058	0.0009	SSLIILK
44.0	772.5058	0.0009	SLSLLLK

44.0	772.5058	0.0009	SVTLLIK
29.6	772.4993	0.0074	RMLILK
23.9	772.4993	0.0074	RLMLLK
22.3	770.5014	2.0053	ERLLLK
21.2	772.4993	0.0074	LRMLIK
21.2	772.4993	0.0074	LRMLLK
17.1	772.5058	0.0009	ISISLLK
15.5	770.5014	2.0053	NGLVKIK

Spectrum No: 107; Query: 46313; Rank: 1

Peptide View

MS/MS Fragmentation of **AANGVVLATEK**
Found in **IPI00219622**, Tax_Id=9606 Gene_Symbol=PSMA2 Proteasome subunit alpha type-2

Match to Query 46313: 1071.594128 from(536.804340,2+)
Title: 090702LimSK_Exosome2_05.2128.2128.2.dta
Data file C:\Temp\Mascoti\LSK_2D\090702LimSK_Exosome2_01_2D.mgf

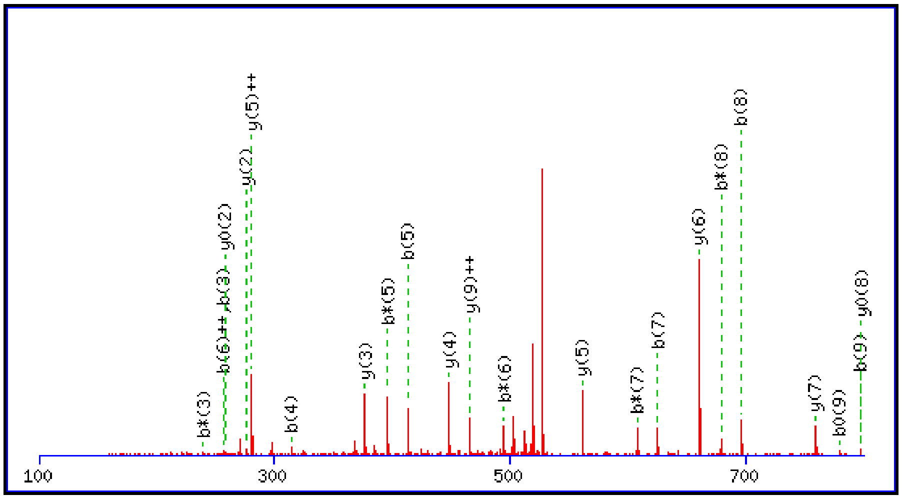

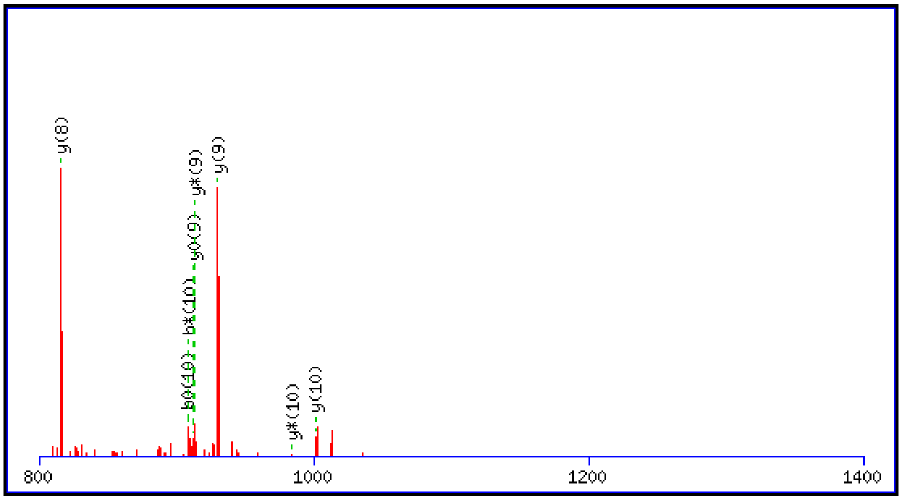

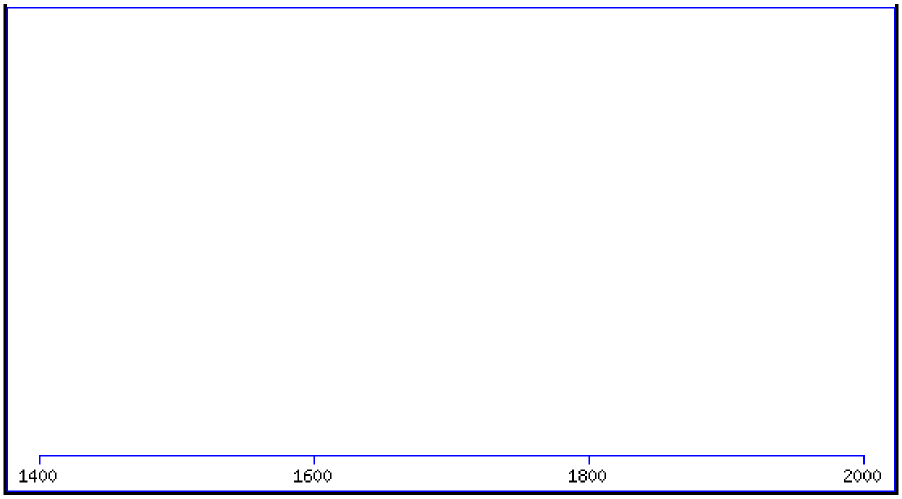

Monoisotopic mass of neutral peptide Mr(calc): 1071.5924
Fixed modifications: Carbamidomethyl (C)
Ions Score: 61 **Expect:** 9.1e-005
Matches (Bold Red): 31/98 fragment ions using 58 most intense peaks

#	b	b^{++}	b*	b^{*++}	b^{0}	b^{0++}	Seq.	y	y^{++}	y*	y^{*++}	y^{0}	y^{0++}	#
1	72.0444	36.5258					A							11
2	143.0815	72.0444					A	**1001.5626**	501.2849	**984.5360**	492.7717	983.5520	492.2796	10
3	**257.1244**	129.0659	**240.0979**	120.5526			N	**930.5255**	**465.7664**	**913.4989**	457.2531	**912.5149**	456.7611	9
4	**314.1459**	157.5766	297.1193	149.0633			G	**816.4825**	408.7449	799.4560	400.2316	**798.4720**	399.7396	8
5	**413.2143**	207.1108	**396.1878**	198.5975			V	**759.4611**	380.2342	742.4345	371.7209	741.4505	371.2289	7
6	512.2827	**256.6450**	**495.2562**	248.1317			V	**660.3927**	330.7000	643.3661	322.1867	642.3821	321.6947	6
7	**625.3668**	313.1870	**608.3402**	304.6738			L	**561.3243**	**281.1658**	544.2977	272.6525	543.3137	272.1605	5
8	**696.4039**	348.7056	**679.3774**	340.1923			A	**448.2402**	224.6237	431.2136	216.1105	430.2296	215.6185	4
9	**797.4516**	399.2294	780.4250	390.7162	**779.4410**	390.2241	T	**377.2031**	189.1052	360.1765	180.5919	359.1925	180.0999	3
10	926.4942	463.7507	**909.4676**	455.2375	**908.4836**	454.7454	E	**276.1554**	138.5813	259.1288	130.0681	**258.1448**	129.5761	2
11							K	147.1128	74.0600	130.0863	65.5468			1

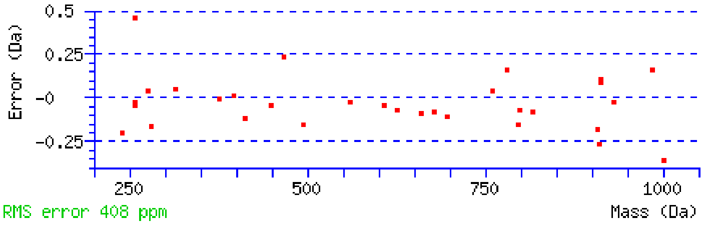

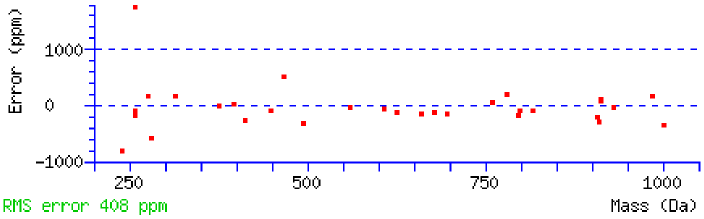

All matches to this query

Score	Mr(calc):	Delta	Sequence
60.6	1071.5924	0.0017	AANGVVLATEK
9.0	1069.5880	2.0062	ALEQALQAAR
3.9	1069.5880	2.0061	GERPLTLER
2.0	1071.5978	-0.0037	HFKVWSLR
1.6	1071.5897	0.0044	AAAAGAKSRGGR
1.0	1070.5985	0.9956	KRPEIWSR

Spectrum No: 108; Query: 55342; Rank: 1

Peptide View

MS/MS Fragmentation of **AANGVVLATEKK**
Found in **IPI00219622**, Tax_Id=9606 Gene_Symbol=PSMA2 Proteasome subunit alpha type-2

Match to Query 55342: 1199.688102 from(400.903310,3+)
Title: 090702LimSK_Exosome2_06.3788.3788.3.dta
Data file C:\Temp\Mascoti\LSK_2D\090702LimSK_Exosome2_01_2D.mgf

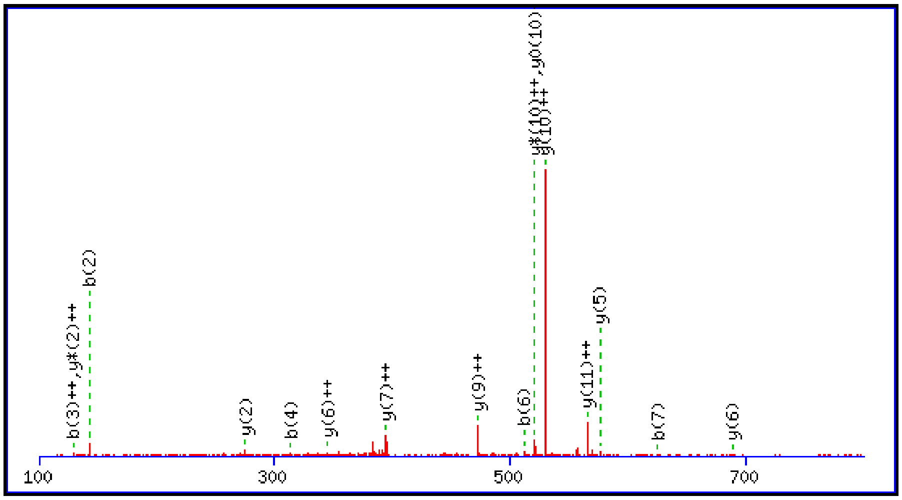

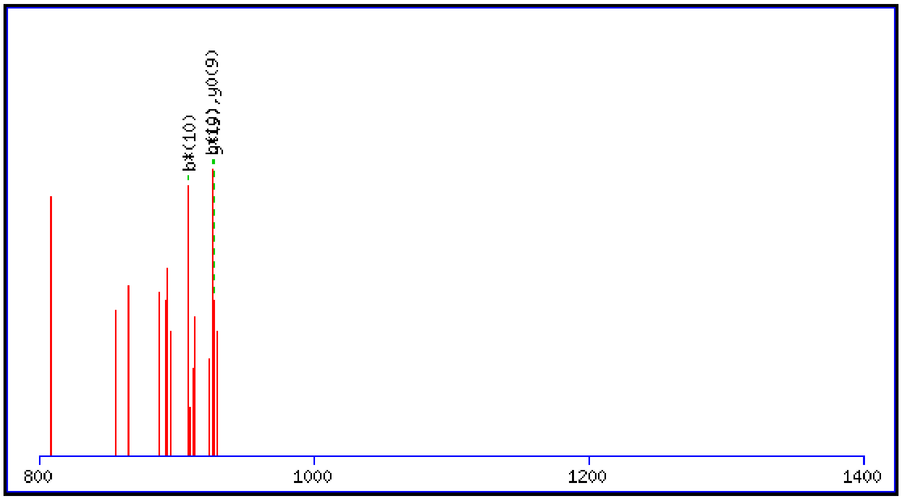

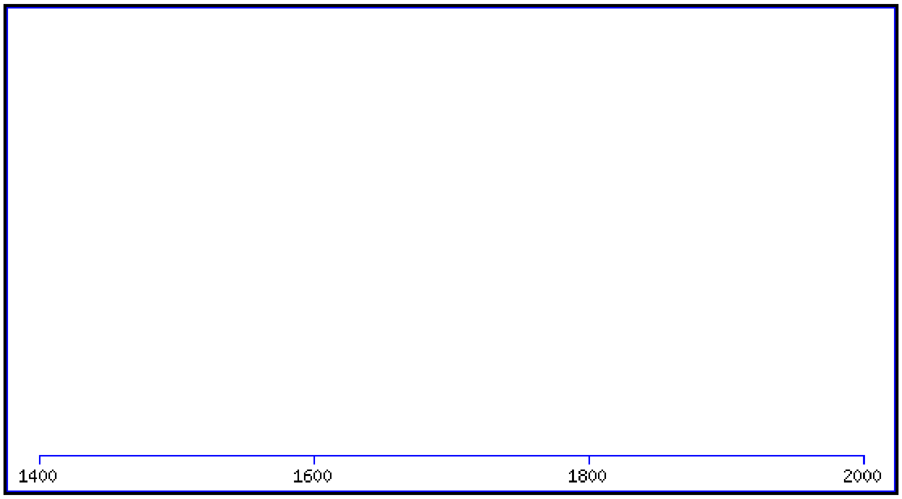

Monoisotopic mass of neutral peptide Mr(calc): 1199.6874
Fixed modifications: Carbamidomethyl (C)
Ions Score: 47 **Expect:** 0.0016
Matches (Bold Red): 20/108 fragment ions using 36 most intense peaks

#	b	b^{++}	b*	b^{*++}	b^{0}	b^{0++}	Seq.	y	y^{++}	y*	y^{*++}	y^{0}	y^{0++}	#
1	72.0444	36.5258					A							12

**2**	**143.0815**	72.0444					**A**	1129.6576	**565.3324**	1112.6310	556.8191	1111.6470	556.3271	**11**
**3**	257.1244	**129.0659**	240.0979	120.5526			**N**	1058.6204	**529.8139**	1041.5939	**521.3006**	1040.6099	**520.8086**	**10**
**4**	**314.1459**	157.5766	297.1193	149.0633			**G**	944.5775	**472.7924**	**927.5510**	464.2791	**926.5669**	463.7871	**9**
**5**	413.2143	207.1108	396.1878	198.5975			**V**	887.5560	444.2817	870.5295	435.7684	869.5455	435.2764	**8**
**6**	**512.2827**	256.6450	495.2562	248.1317			**V**	788.4876	**394.7475**	771.4611	386.2342	770.4771	385.7422	**7**
**7**	**625.3668**	313.1870	608.3402	304.6738			**L**	**689.4192**	**345.2132**	672.3927	336.7000	671.4087	336.2080	**6**
**8**	696.4039	348.7056	679.3774	340.1923			**A**	**576.3352**	288.6712	559.3086	280.1579	558.3246	279.6659	**5**
**9**	797.4516	399.2294	780.4250	390.7162	779.4410	390.2241	**T**	505.2980	253.1527	488.2715	244.6394	487.2875	244.1474	**4**
**10**	**926.4942**	463.7507	**909.4676**	455.2375	908.4836	454.7454	**E**	404.2504	202.6288	387.2238	194.1155	386.2398	193.6235	**3**
**11**	1054.5891	527.7982	1037.5626	519.2849	1036.5786	518.7929	**K**	**275.2078**	138.1075	258.1812	**129.5942**			**2**
**12**							**K**	147.1128	74.0600	130.0863	65.5468			**1**

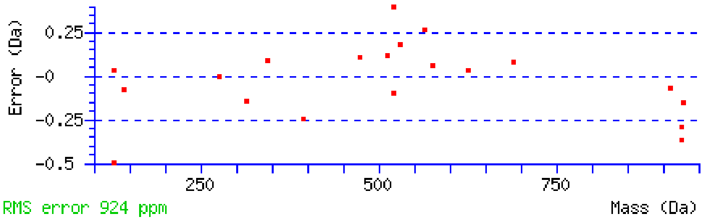

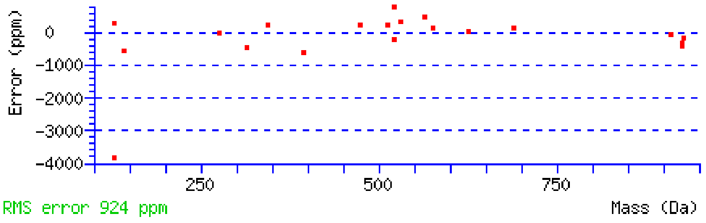

All matches to this query

Score	Mr(calc):	Delta	Sequence
47.1	1199.6874	0.0007	AANGVVLATEKK
8.6	1198.6856	1.0025	AAKKLLHSGMK
5.4	1199.6874	0.0007	SILPGDKTNKK
4.3	1199.6986	-0.0105	DSVLNIARGKK
3.5	1198.6743	1.0138	AKPLMELIER
3.1	1199.6808	0.0073	LKLGEMTPRR
3.1	1199.6887	-0.0006	AHVKRPDLHK
2.9	1197.6829	2.0052	AQERLAAALQK
2.8	1199.6775	0.0106	SSTPWLVRVR
2.8	1199.6914	-0.0033	AAPLIVEVFNK

Spectrum No: 109; Query: 28709; Rank: 1

Peptide View

MS/MS Fragmentation of **DYLAAIA**
Found in **IPI00219622**, Tax_Id=9606 Gene_Symbol=PSMA2 Proteasome subunit alpha type-2

Match to Query 28709: 735.382014 from(736.389290,1+)
Title: 090702LimSK_Exosome2_06.4730.4730.1.dta
Data file C:\Temp\Mascoti\LSK_2D\090702LimSK_Exosome2_01_2D.mgf

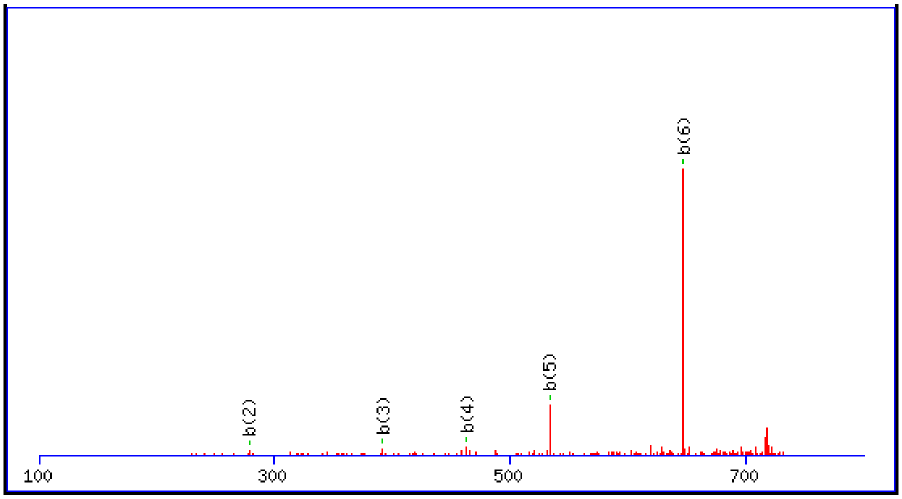

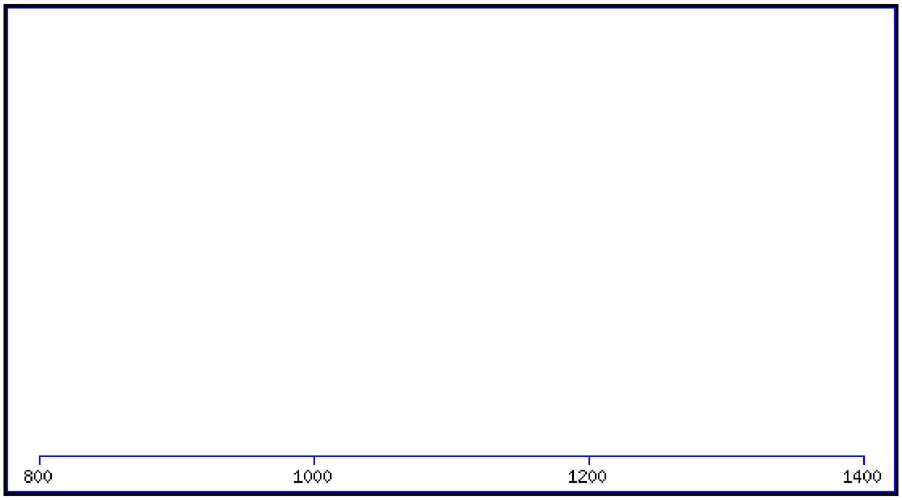

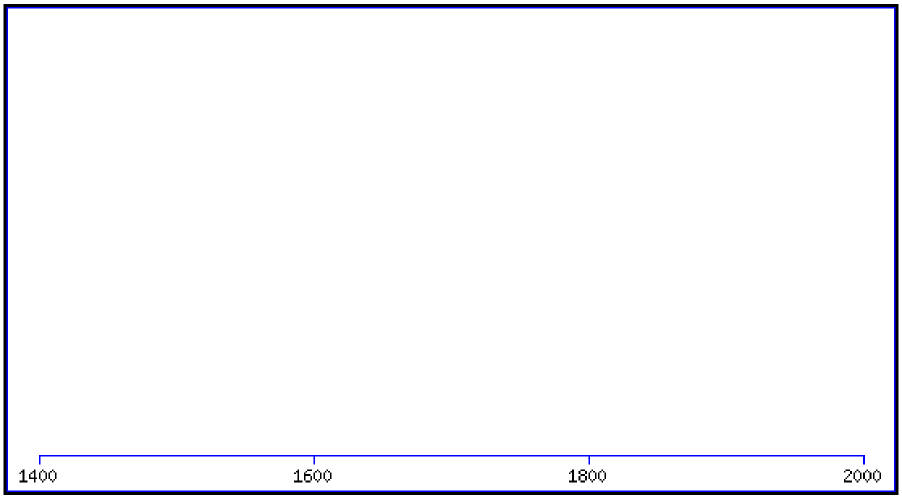

Monoisotopic mass of neutral peptide Mr(calc): 735.3803
Fixed modifications: Carbamidomethyl (C)
Ions Score: 50 **Expect:** 0.0009
Matches (Bold Red): 5/18 fragment ions using 26 most intense peaks

#	b	b^0	Seq.	y	#
1	116.0342	98.0237	D		7
2	**279.0975**	261.0870	Y	621.3606	6
3	**392.1816**	374.1710	L	458.2973	5
4	**463.2187**	445.2082	A	345.2132	4
5	**534.2558**	516.2453	A	274.1761	3
6	**647.3399**	629.3293	I	203.1390	2

7			A	90.0550	1

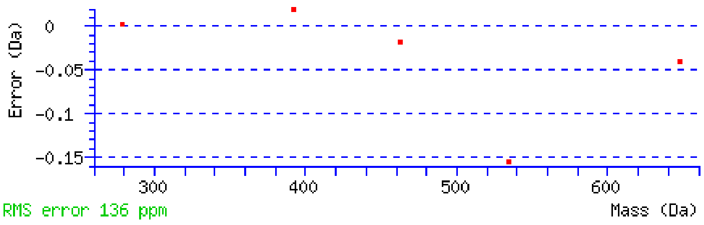

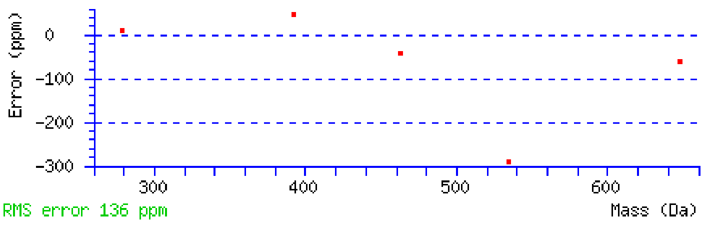

All matches to this query

Score	Mr(calc):	Delta	Sequence
50.3	735.3803	0.0017	DYLAAIA
6.2	733.3792	2.0028	XDIMIR
5.5	733.3792	2.0028	SLICNK
5.5	733.3793	2.0028	SLQCVK
5.5	733.3792	2.0028	SNCLLK
4.9	733.3792	2.0028	SPSGKMK
3.7	733.3759	2.0061	TVDFRP
2.0	733.3792	2.0028	SCLNIK

Spectrum No: 110; Query: 68369; Rank: 1

Peptide View

MS/MS Fragmentation of **GYSFSLTTFSPSGK**
Found in **IPI00219622**, Tax_Id=9606 Gene_Symbol=PSMA2 Proteasome subunit alpha type-2

Match to Query 68369: 1477.713428 from(739.863990,2+)
Title: 090702LimSK_Exosome2_06.8139.8139.2.dta
Data file C:\Temp\Mascoti\LSK_2D\090702LimSK_Exosome2_01_2D.mgf

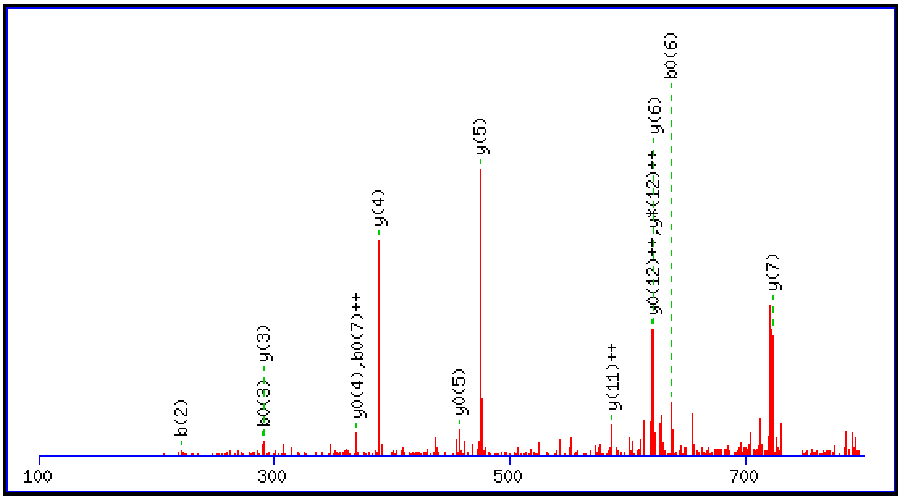

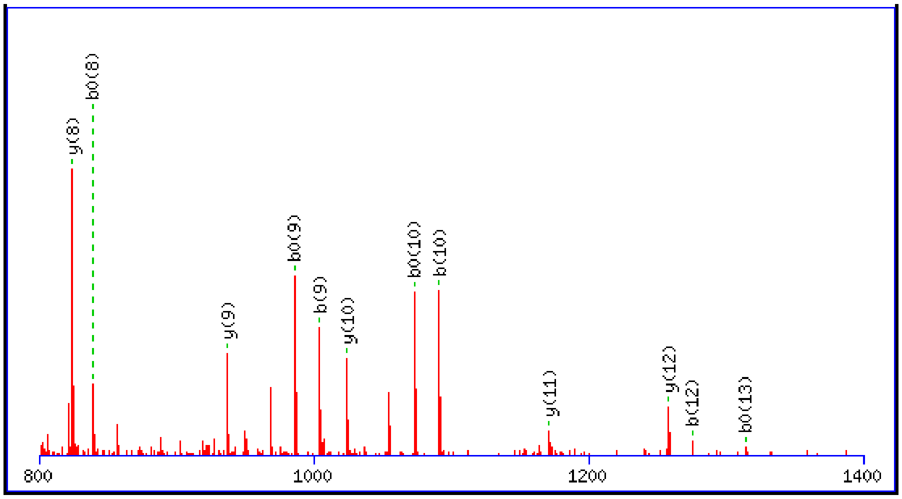

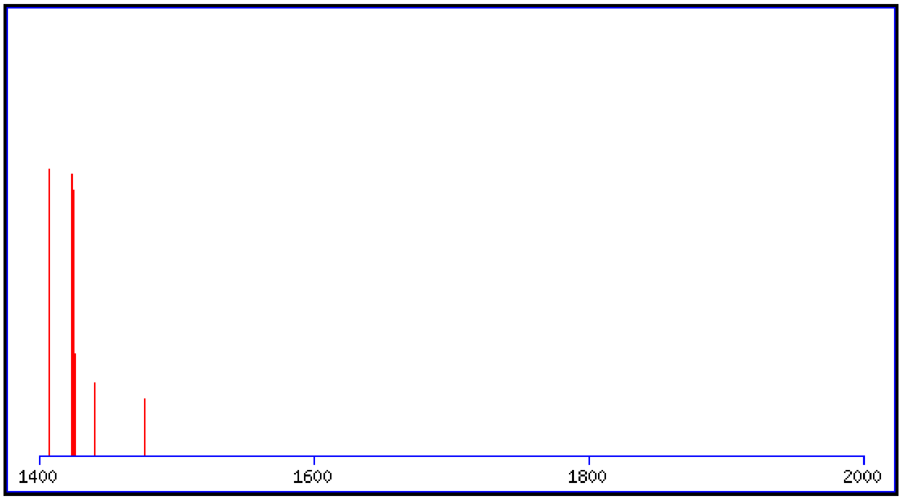

Monoisotopic mass of neutral peptide Mr(calc): 1477.7089
Fixed modifications: Carbamidomethyl (C)
Ions Score: 74 **Expect:** 7.1e-006
Matches (Bold Red): 26/122 fragment ions using 42 most intense peaks

#	b	b^{++}	b^0	b^{0++}	Seq.	y	y^{++}	y*	y^{*++}	y^0	y^{0++}	#
1	58.0287	29.5180			G							14
2	**221.0921**	111.0497			Y	1421.6947	711.3510	1404.6682	702.8377	1403.6842	702.3457	13
3	308.1241	154.5657	**290.1135**	145.5604	S	**1258.6314**	629.8193	1241.6048	**621.3061**	1240.6208	**620.8141**	12
4	455.1925	228.0999	437.1819	219.0946	F	**1171.5994**	**586.3033**	1154.5728	577.7900	1153.5888	577.2980	11
5	542.2245	271.6159	524.2140	262.6106	S	**1024.5310**	512.7691	1007.5044	504.2558	1006.5204	503.7638	10
6	655.3086	328.1579	**637.2980**	319.1527	L	**937.4989**	469.2531	920.4724	460.7398	919.4884	460.2478	9
7	756.3563	378.6818	738.3457	**369.6765**	T	**824.4149**	412.7111	807.3883	404.1978	806.4043	403.7058	8
8	857.4040	429.2056	**839.3934**	420.2003	T	**723.3672**	362.1872	706.3406	353.6740	705.3566	353.1819	7
9	**1004.4724**	502.7398	**986.4618**	493.7345	F	**622.3195**	311.6634	605.2930	303.1501	604.3089	302.6581	6
10	**1091.5044**	546.2558	**1073.4938**	537.2506	S	**475.2511**	238.1292	458.2245	229.6159	**457.2405**	229.1239	5
11	1188.5572	594.7822	1170.5466	585.7769	P	**388.2191**	194.6132	371.1925	186.0999	**370.2085**	185.6079	4
12	**1275.5892**	638.2982	1257.5786	629.2930	S	**291.1663**	146.0868	274.1397	137.5735	273.1557	137.0815	3
13	1332.6107	666.8090	**1314.6001**	657.8037	G	204.1343	102.5708	187.1077	94.0575			2
14					K	147.1128	74.0600	130.0863	65.5468			1

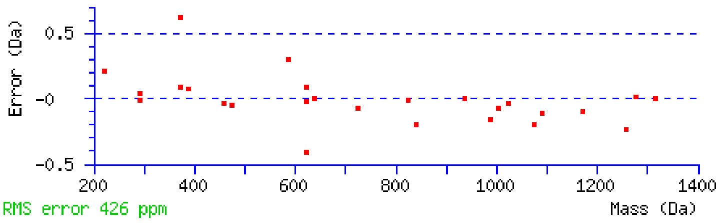

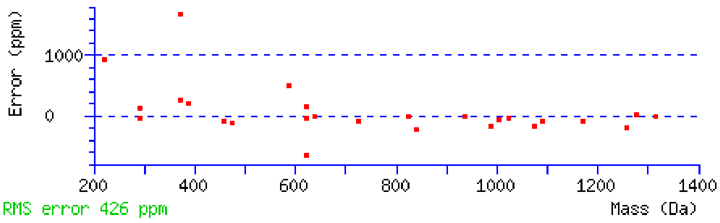

All matches to this query

Score	Mr(calc):	Delta	Sequence
74.3	1477.7089	0.0045	GYSFSLTTFSPSGK
9.1	1475.7061	2.0073	VKFASLEFSPGSK
3.3	1476.7130	1.0005	EKMQLVQESEEK
3.0	1475.6956	2.0178	AVACTTTHIPGLR
2.2	1476.7143	0.9992	KTFVLAPSSVLR
0.1	1477.7112	0.0022	MLLAPPSTPSRGR
0.1	1477.7112	0.0022	MLLAPPSTPSRGR

Spectrum No: 111; Query: 72559; Rank: 1

Peptide View

MS/MS Fragmentation of **HIGLVYSGMGPDYR**
Found in **IPI00219622**, Tax_Id=9606 Gene_Symbol=PSMA2 Proteasome subunit alpha type-2

Match to Query 72559: 1564.757562 from(522.593130,3+)
Title: 090702LimSK_Exosome2_06.9090.9090.3.dta
Data file C:\Temp\Mascoti\LSK_2D\090702LimSK_Exosome2_01_2D.mgf

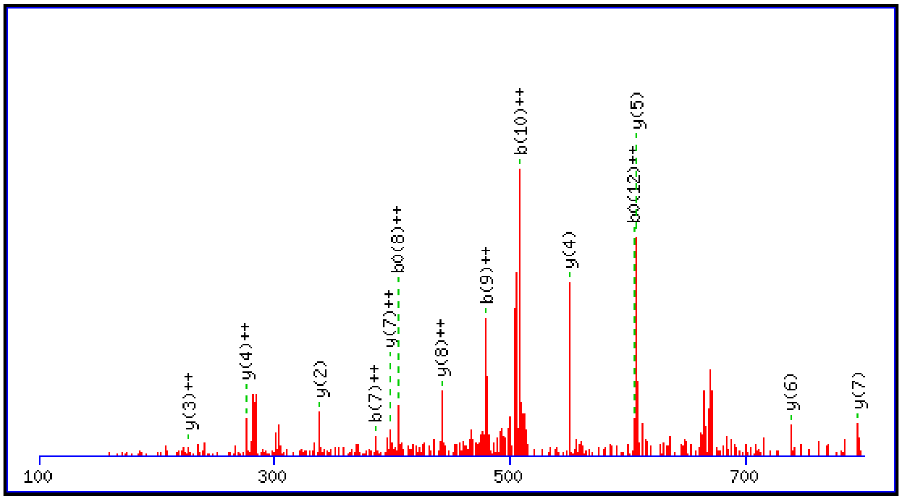

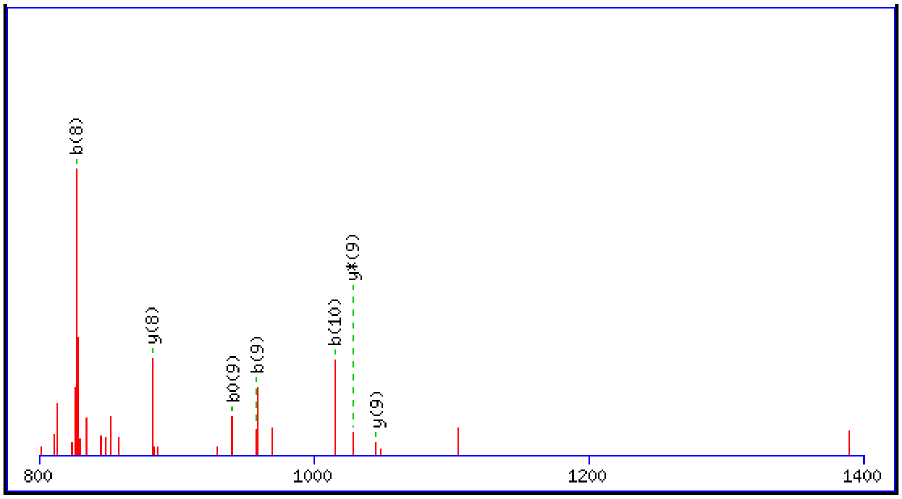

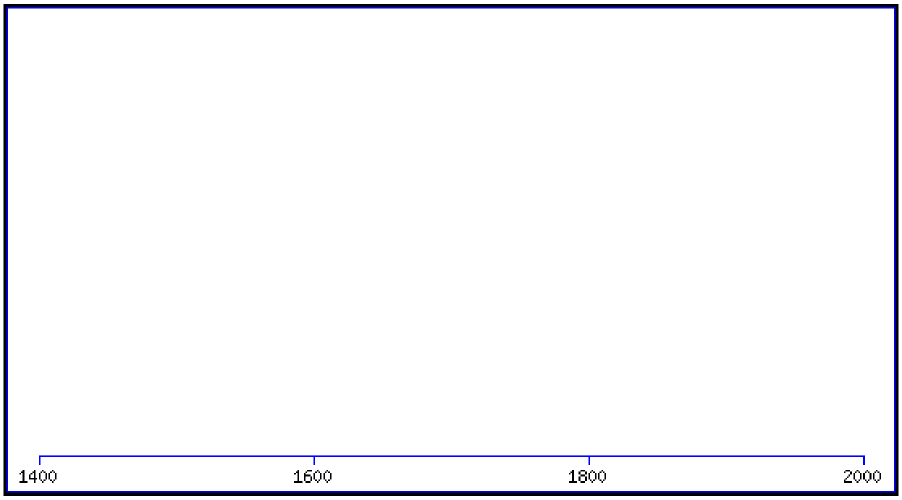

Monoisotopic mass of neutral peptide Mr(calc): 1563.7504
Fixed modifications: Carbamidomethyl (C)
Ions Score: 29 **Expect:** 0.27
Matches (Bold Red): 21/114 fragment ions using 50 most intense peaks

#	b	b^{++}	b^{0}	b^{0++}	Seq.	y	y^{++}	y*	y^{*++}	y^{0}	y^{0++}	#
1	138.0662	69.5367			H							14
2	251.1503	126.0788			I	1427.6988	714.3530	1410.6722	705.8397	1409.6882	705.3477	13
3	308.1717	154.5895			G	1314.6147	657.8110	1297.5882	649.2977	1296.6041	648.8057	12
4	421.2558	211.1315			L	1257.5932	629.3003	1240.5667	620.7870	1239.5827	620.2950	11
5	520.3242	260.6657			V	1144.5092	572.7582	1127.4826	564.2449	1126.4986	563.7529	10
6	683.3875	342.1974			Y	**1045.4408**	523.2240	**1028.4142**	514.7107	1027.4302	514.2187	9
7	770.4196	**385.7134**	752.4090	376.7081	S	**882.3774**	**441.6924**	865.3509	433.1791	864.3669	432.6871	8
8	**827.4410**	414.2241	809.4305	**405.2189**	G	**795.3454**	**398.1763**	778.3189	389.6631	777.3348	389.1711	7
9	**958.4815**	**479.7444**	**940.4709**	470.7391	M	**738.3239**	369.6656	721.2974	361.1523	720.3134	360.6603	6
10	**1015.5030**	**508.2551**	997.4924	499.2498	G	**607.2835**	304.1454	590.2569	295.6321	589.2729	295.1401	5
11	1112.5557	556.7815	1094.5452	547.7762	P	**550.2620**	**275.6346**	533.2354	267.1214	532.2514	266.6293	4
12	1227.5827	614.2950	1209.5721	**605.2897**	D	453.2092	**227.1083**	436.1827	218.5950	435.1987	218.1030	3
13	1390.6460	695.8266	1372.6354	686.8214	Y	**338.1823**	169.5948	321.1557	161.0815			2
14					R	175.1190	88.0631	158.0924	79.5498			1

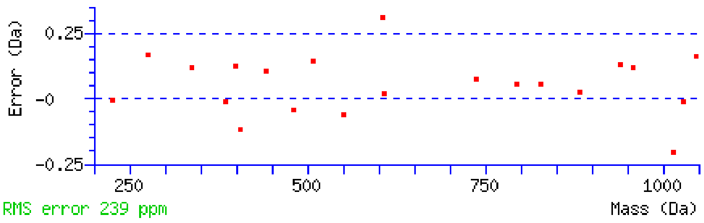

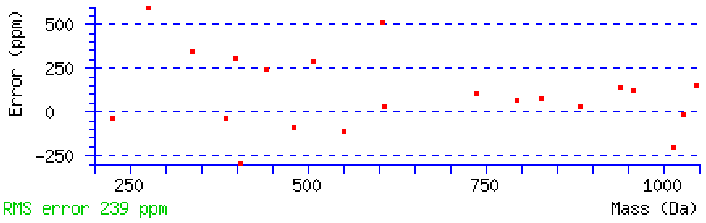

All matches to this query

Score	Mr(calc):	Delta	Sequence
28.8	1563.7504	1.0072	HIGLVYSGMGPDYR
9.7	1562.7552	2.0024	YDGLVGMFDPKGHK
7.1	1562.7626	1.9949	MSVSSLSSITSLKK
6.5	1562.7626	1.9949	MSVSSLSSITSLKK
5.0	1564.7545	0.0031	GARPAMSRAVSAPSK
3.8	1564.7545	0.0031	GARPAMSRAVSAPSK
2.8	1563.7406	1.0169	VQIIQTSTNTSHR
2.1	1563.7430	1.0146	SFSWSSSLDKHQR
1.3	1562.7494	2.0082	ASPHDVLETIFVR
0.8	1564.7650	-0.0075	KNLPKSPNPSFEK

Spectrum No: 112; Query: 92798; Rank: 1

Peptide View

MS/MS Fragmentation of **LAQQYYLVYQEPIPTAQLVQR**
Found in **IPI00219622**, Tax_Id=9606 Gene_Symbol=PSMA2 Proteasome subunit alpha type-2

Match to Query 92798: 2521.343112 from(841.454980,3+)
Title: 090702LimSK_Exosome2_05.8259.8259.3.dta
Data file C:\Temp\Mascoti\LSK_2D\090702LimSK_Exosome2_01_2D.mgf

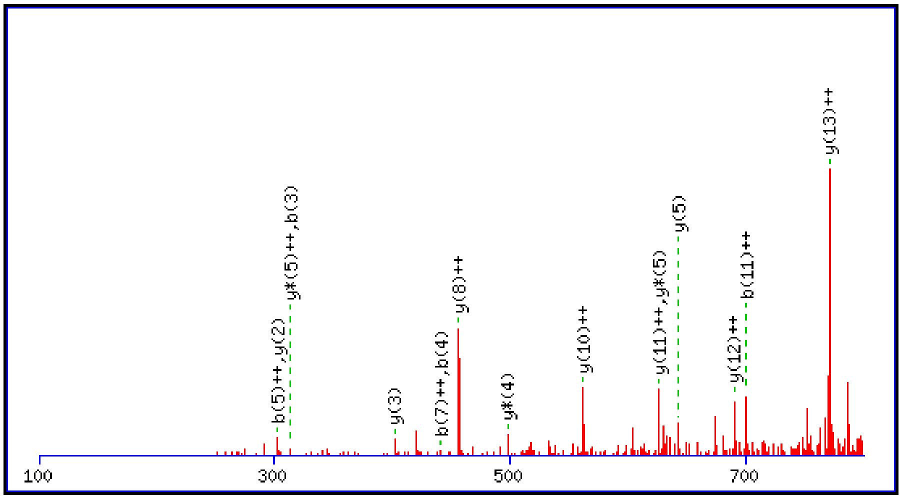

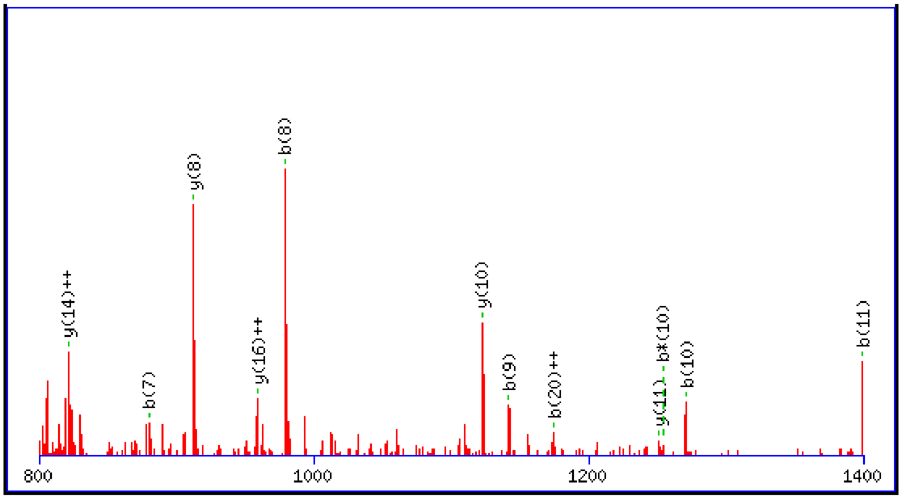

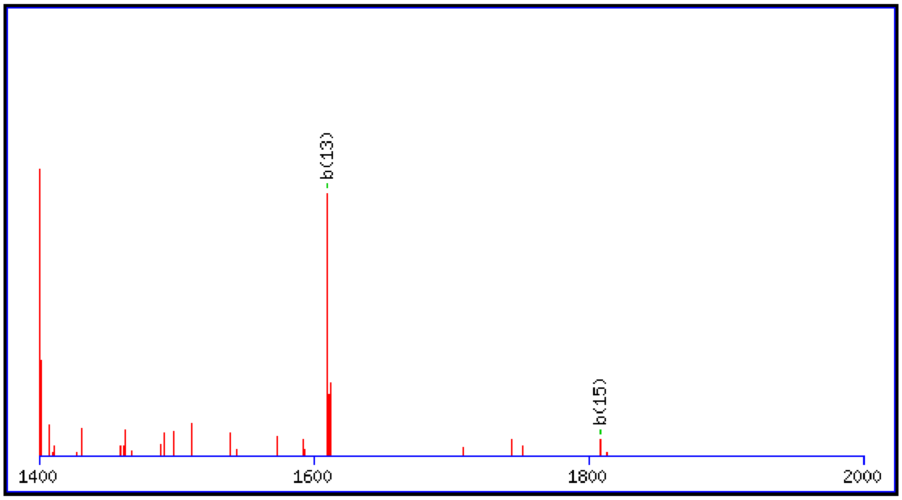

Monoisotopic mass of neutral peptide Mr(calc): 2520.3376
Fixed modifications: Carbamidomethyl (C)
Ions Score: 48 **Expect:** 0.0014
Matches (Bold Red): 30/204 fragment ions using 55 most intense peaks

#	b	b^{++}	b*	b^{*++}	b^0	b^{0++}	Seq.	y	y^{++}	y*	y^{*++}	y^0	y^{0++}	#
1	114.0913	57.5493					L							21
2	185.1285	93.0679					A	2408.2609	1204.6341	2391.2343	1196.1208	2390.2503	1195.6288	20
3	**313.1870**	157.0972	296.1605	148.5839			Q	2337.2238	1169.1155	2320.1972	1160.6022	2319.2132	1160.1102	19
4	**441.2456**	221.1264	424.2191	212.6132			Q	2209.1652	1105.0862	2192.1386	1096.5730	2191.1546	1096.0809	18
5	604.3089	**302.6581**	587.2824	294.1448			Y	2081.1066	1041.0569	2064.0801	1032.5437	2063.0960	1032.0517	17
6	767.3723	384.1898	750.3457	375.6765			Y	1918.0433	**959.5253**	1901.0167	951.0120	1900.0327	950.5200	16
7	**880.4563**	**440.7318**	863.4298	432.2185			L	1754.9799	877.9936	1737.9534	869.4803	1736.9694	868.9883	15
8	**979.5247**	490.2660	962.4982	481.7527			V	1641.8959	**821.4516**	1624.8693	812.9383	1623.8853	812.4463	14
9	**1142.5881**	571.7977	1125.5615	563.2844			Y	1542.8275	**771.9174**	1525.8009	763.4041	1524.8169	762.9121	13
10	**1270.6467**	635.8270	**1253.6201**	627.3137			Q	1379.7641	**690.3857**	1362.7376	681.8724	1361.7536	681.3804	12
11	**1399.6892**	**700.3483**	1382.6627	691.8350	1381.6787	691.3430	E	**1251.7056**	**626.3564**	1234.6790	617.8431	1233.6950	617.3511	11
12	1496.7420	748.8746	1479.7155	740.3614	1478.7314	739.8694	P	**1122.6630**	**561.8351**	1105.6364	553.3218	1104.6524	552.8298	10
13	**1609.8261**	805.4167	1592.7995	796.9034	1591.8155	796.4114	I	1025.6102	513.3087	1008.5837	504.7955	1007.5996	504.3035	9
14	1706.8788	853.9431	1689.8523	845.4298	1688.8683	844.9378	P	**912.5261**	**456.7667**	895.4996	448.2534	894.5156	447.7614	8
15	**1807.9265**	904.4669	1790.9000	895.9536	1789.9160	895.4616	T	815.4734	408.2403	798.4468	399.7271	797.4628	399.2350	7
16	1878.9636	939.9855	1861.9371	931.4722	1860.9531	930.9802	A	714.4257	357.7165	697.3992	349.2032			6
17	2007.0222	1004.0147	1989.9957	995.5015	1989.0116	995.0095	Q	**643.3886**	322.1979	**626.3620**	**313.6847**			5
18	2120.1063	1060.5568	2103.0797	1052.0435	2102.0957	1051.5515	L	515.3300	258.1686	**498.3035**	249.6554			4
19	2219.1747	1110.0910	2202.1481	1101.5777	2201.1641	1101.0857	V	**402.2459**	201.6266	385.2194	193.1133			3

**20**	2347.2333	**1174.1203**	2330.2067	1165.6070	2329.2227	1165.1150	**Q**	**303.1775**	152.0924	286.1510	143.5791			**2**
**21**							**R**	175.1190	88.0631	158.0924	79.5498			**1**

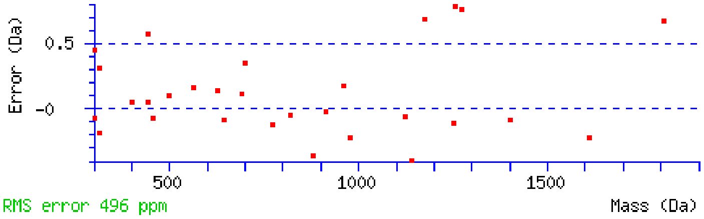

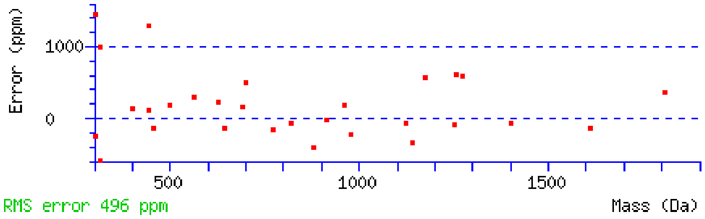

All matches to this query

Score	Mr(calc):	Delta	Sequence
48.2	2520.3376	1.0055	LAQQYYLVYQEPIPTAQLVQR
11.5	2519.3486	1.9945	VPGRKPAPGLSVGAQITTNPAPRR
6.0	2519.3400	2.0031	DLAVVLETAPILTALDIFVDRR
5.9	2521.3183	0.0248	SSARPGSPTAKMISPAARLGSHSQK
4.2	2519.3434	1.9997	LVATAMKLHGVIDILSLSITNSK
4.2	2521.3549	-0.0118	MKIKYFSPIGCVSLISGHLSLR
4.0	2519.3496	1.9935	LPRPPQVLGLQAEEEFNIEKGR
3.6	2520.3353	1.0078	TVILVKNLPAGTLAAELQETFGR
3.3	2520.3215	1.0216	IYTMIYRNLVVFPLVDLSIR
3.0	2520.3353	1.0078	TVILVKNLPAGTLAAELQETFGR

Spectrum No: 113; Query: 69468; Rank: 1

Peptide View

MS/MS Fragmentation of **LTPTEVKDYLAAIA**
Found in **IPI00219622**, Tax_Id=9606 Gene_Symbol=PSMA2 Proteasome subunit alpha type-2

Match to Query 69468: 1503.821828 from(752.918190,2+)
Title: 090702LimSK_Exosome2_01.9848.9848.2.dta
Data file C:\Temp\Mascoti\LSK_2D\090702LimSK_Exosome2_01_2D.mgf

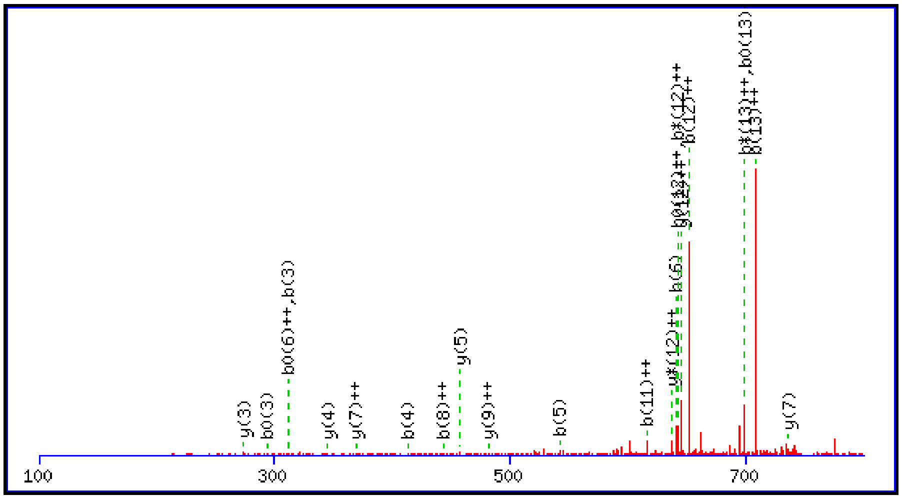

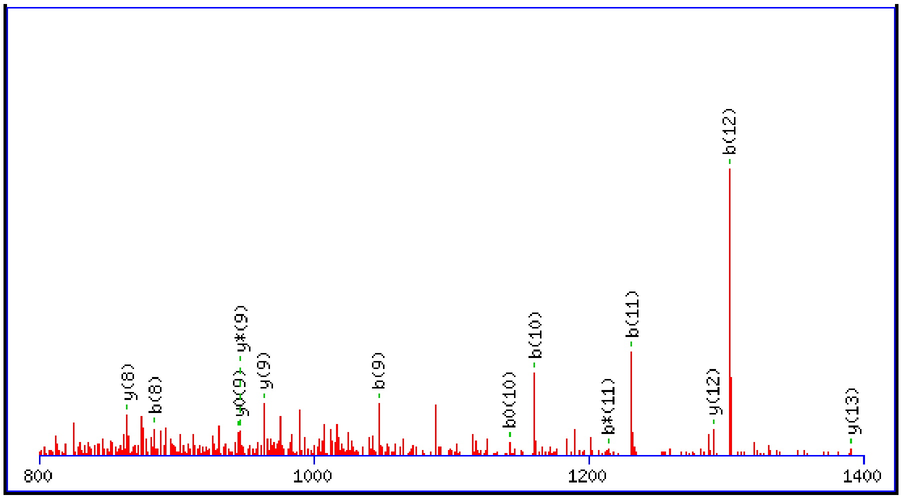

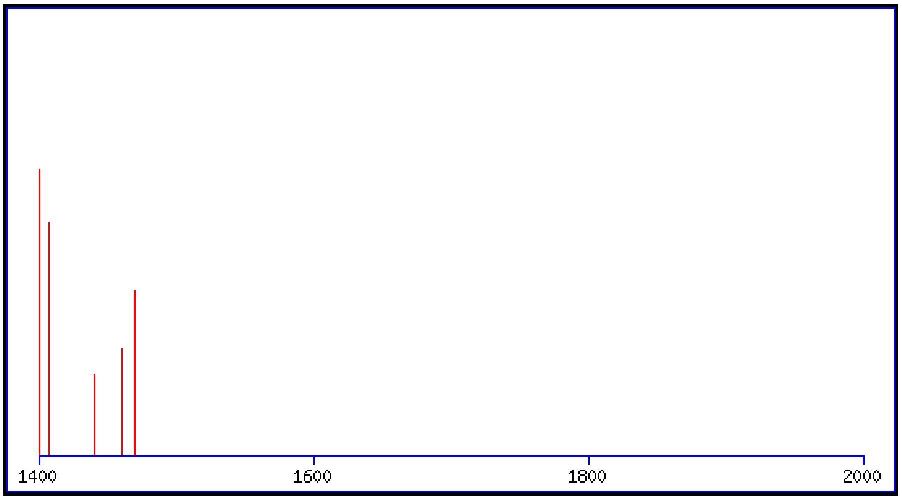

Monoisotopic mass of neutral peptide Mr(calc): 1503.8184
Fixed modifications: Carbamidomethyl (C)
Ions Score: 27 **Expect:** 0.2
Matches (Bold Red): 35/116 fragment ions using 125 most intense peaks

#	b	b^{++}	b*	b^{*++}	b^{0}	b^{0++}	Seq.	y	y^{++}	y*	y^{*++}	y^{0}	y^{0++}	#
1	114.0913	57.5493					L							14
2	215.1390	108.0731			197.1285	99.0679	T	**1391.7417**	696.3745	1374.7151	687.8612	1373.7311	687.3692	13
3	**312.1918**	156.5995			**294.1812**	147.5942	P	**1290.6940**	**645.8506**	1273.6674	**637.3374**	1272.6834	636.8454	12
4	**413.2395**	207.1234			395.2289	198.1181	T	1193.6412	597.3243	1176.6147	588.8110	1175.6307	588.3190	11
5	**542.2821**	271.6447			524.2715	262.6394	E	1092.5936	546.8004	1075.5670	538.2871	1074.5830	537.7951	10
6	**641.3505**	321.1789			623.3399	**312.1736**	V	**963.5510**	**482.2791**	**946.5244**	473.7658	**945.5404**	473.2738	9
7	769.4454	385.2264	752.4189	376.7131	751.4349	376.2211	K	**864.4825**	432.7449	847.4560	424.2316	846.4720	423.7396	8
8	**884.4724**	**442.7398**	867.4458	434.2266	866.4618	433.7345	D	**736.3876**	**368.6974**			718.3770	359.6921	7
9	**1047.5357**	524.2715	1030.5092	515.7582	1029.5251	515.2662	Y	621.3606	311.1840					6
10	**1160.6198**	580.8135	1143.5932	572.3002	**1142.6092**	571.8082	L	**458.2973**	229.6523					5
11	**1231.6569**	**616.3321**	**1214.6303**	607.8188	1213.6463	607.3268	A	**345.2132**	173.1103					4
12	**1302.6940**	**651.8506**	1285.6674	**643.3374**	1284.6834	**642.8454**	A	**274.1761**	137.5917					3
13	1415.7781	**708.3927**	1398.7515	**699.8794**	1397.7675	**699.3874**	I	203.1390	102.0731					2
14							A	90.0550	45.5311					1

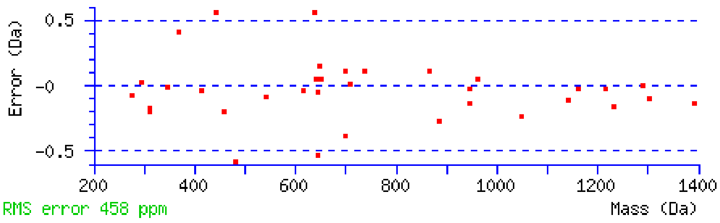

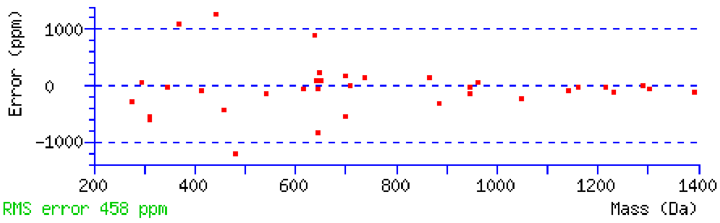

All matches to this query

Score	Mr(calc):	Delta	Sequence
27.3	1503.8184	0.0034	LTPTEVKDYLAAIA
7.7	1502.8239	0.9980	SNLREVAQMLKSK
6.3	1502.8052	1.0166	SLNANTDITSLARK
2.5	1502.8133	1.0085	STLKKVFGFDSFK
2.3	1501.8075	2.0143	ASLALCAFVAVPQR
1.7	1501.8212	2.0006	SLSNSPQLKSRTGK
1.6	1502.8066	1.0152	SLTGARWQSRSVR
1.5	1501.8286	1.9932	SNIREIEKLCLK
1.4	1502.8205	1.0013	SIYEIRNKDLPR

Spectrum No: 114; Query: 85691; Rank: 1

Peptide View

MS/MS Fragmentation of **NHLVEIPPNLPSSLVELR**
Found in **IPI00010790**, Tax_Id=9606 Gene_Symbol=BGN Biglycan

Match to Query 85691: 2027.130822 from(676.717550,3+)
Title: 090702LimSK_Exosome2_08b1.5743.5743.3.dta
Data file C:\Temp\Mascoti\LSK_2D\090702LimSK_Exosome2_01_2D.mgf

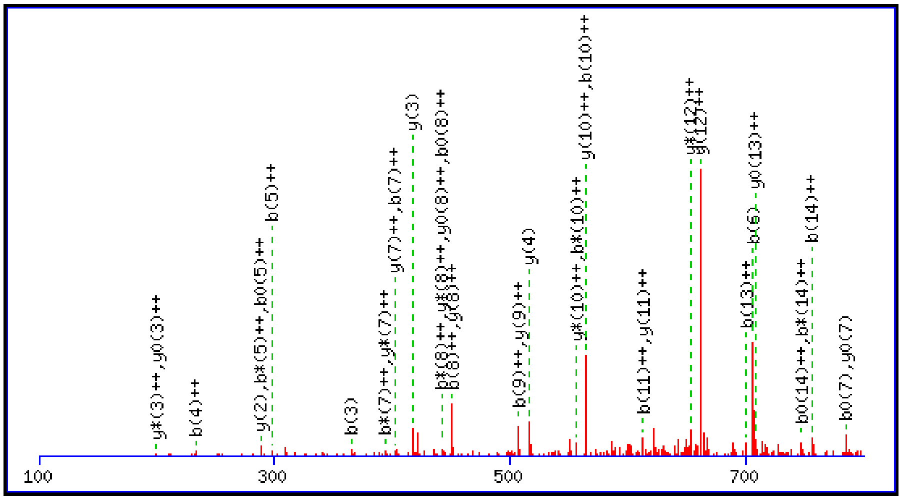

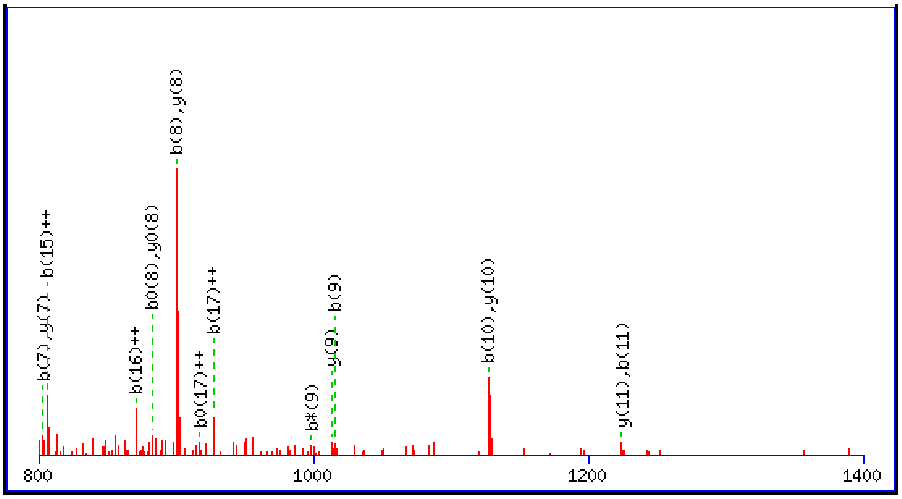

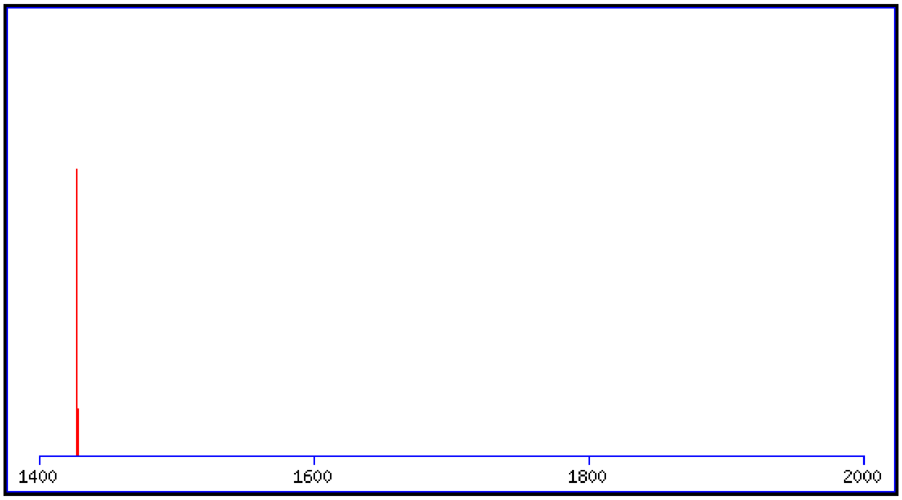

Monoisotopic mass of neutral peptide Mr(calc): 2026.1211
Fixed modifications: Carbamidomethyl (C)
Ions Score: 50 **Expect:** 0.00055
Matches (Bold Red): 55/192 fragment ions using 93 most intense peaks

#	b	b^{++}	b*	b^{*++}	b^0	b^{0++}	Seq.	y	y^{++}	y*	y^{*++}	y^0	y^{0++}	#
1	115.0502	58.0287	98.0237	49.5155			N							18
2	252.1091	126.5582	235.0826	118.0449			H	1913.0855	957.0464	1896.0589	948.5331	1895.0749	948.0411	17
3	**365.1932**	183.1002	348.1666	174.5870			L	1776.0266	888.5169	1759.0000	880.0036	1758.0160	879.5116	16
4	464.2616	**232.6344**	447.2350	224.1212			V	1662.9425	831.9749	1645.9159	823.4616	1644.9319	822.9696	15
5	593.3042	**297.1557**	576.2776	**288.6425**	575.2936	**288.1504**	E	1563.8741	782.4407	1546.8475	773.9274	1545.8635	773.4354	14
6	**706.3883**	353.6978	689.3617	345.1845	688.3777	344.6925	I	1434.8315	717.9194	1417.8049	709.4061	1416.8209	**708.9141**	13
7	**803.4410**	**402.2241**	786.4145	**393.7109**	**785.4304**	393.2189	P	1321.7474	**661.3774**	1304.7209	**652.8641**	1303.7369	652.3721	12
8	**900.4938**	**450.7505**	883.4672	**442.2373**	**882.4832**	**441.7452**	P	**1224.6947**	**612.8510**	1207.6681	604.3377	1206.6841	603.8457	11
9	**1014.5367**	**507.7720**	**997.5102**	499.2587	996.5261	498.7667	N	**1127.6419**	**564.3246**	1110.6153	**555.8113**	1109.6313	555.3193	10
10	**1127.6208**	**564.3140**	1110.5942	**555.8007**	1109.6102	555.3087	L	**1013.5990**	**507.3031**	996.5724	498.7898	995.5884	498.2978	9
11	**1224.6735**	**612.8404**	1207.6470	604.3271	1206.6630	603.8351	P	**900.5149**	**450.7611**	883.4884	**442.2478**	**882.5043**	**441.7558**	8
12	1311.7056	656.3564	1294.6790	647.8431	1293.6950	647.3511	S	**803.4621**	**402.2347**	786.4356	**393.7214**	**785.4516**	393.2294	7
13	1398.7376	**699.8724**	1381.7110	691.3592	1380.7270	690.8672	S	716.4301	358.7187	699.4036	350.2054	698.4195	349.7134	6
14	1511.8217	**756.4145**	1494.7951	**747.9012**	1493.8111	**747.4092**	L	629.3981	315.2027	612.3715	306.6894	611.3875	306.1974	5
15	1610.8901	**805.9487**	1593.8635	797.4354	1592.8795	796.9434	V	**516.3140**	258.6606	499.2875	250.1474	498.3035	249.6554	4
16	1739.9327	**870.4700**	1722.9061	861.9567	1721.9221	861.4647	E	**417.2456**	209.1264	400.2191	**200.6132**	399.2350	**200.1212**	3
17	1853.0167	**927.0120**	1835.9902	918.4987	1835.0062	**918.0067**	L	**288.2030**	144.6051	271.1765	136.0919			2
18							R	175.1190	88.0631	158.0924	79.5498			1

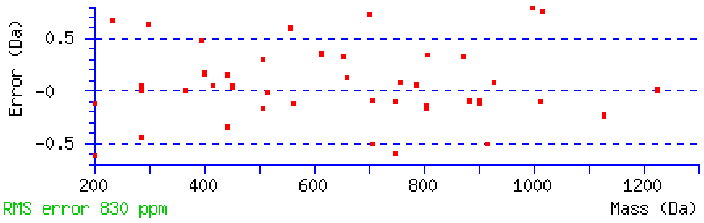

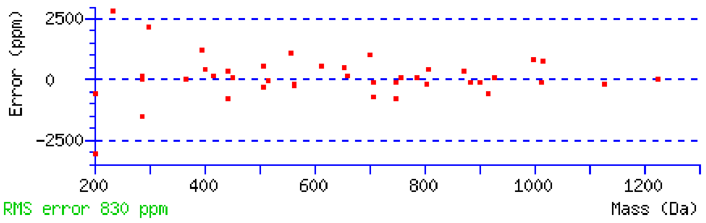

All matches to this query

Score	Mr(calc):	Delta	Sequence
49.9	2026.1211	1.0097	NHLVEIPPNLPSSLVELR
33.3	2027.1163	0.0145	APEYTEPLGGLQRSLKIR
21.6	2026.1463	0.9846	LVQIEYALAAVAGGAPSVGIK
10.1	2027.1165	0.0143	GFWILAILTILMYSTAAK
9.9	2027.1163	0.0145	LPRPLPAVPGELTEATPNR
8.1	2027.1197	0.0111	XSLMGLGDIISQQLVERR
8.1	2027.1197	0.0111	XSLMGLGDIISQQLVERR
6.2	2026.1364	0.9944	TGQLFLKIIHTSVWAGQK
6.1	2026.1211	1.0097	VKDQKAIITENWVLNQK
5.7	2025.1272	2.0036	QVKPPRLPPPPWGRSGEK

Spectrum No: 115; Query: 38639; Rank: 1

Peptide View

MS/MS Fragmentation of **SILYDER**
Found in **IPI00219622**, Tax_Id=9606 Gene_Symbol=PSMA2 Proteasome subunit alpha type-2

Match to Query 38639: 894.445968 from(448.230260,2+)
Title: 090702LimSK_Exosome2_06.2493.2493.2.dta
Data file C:\Temp\Mascoti\LSK_2D\090702LimSK_Exosome2_01_2D.mgf

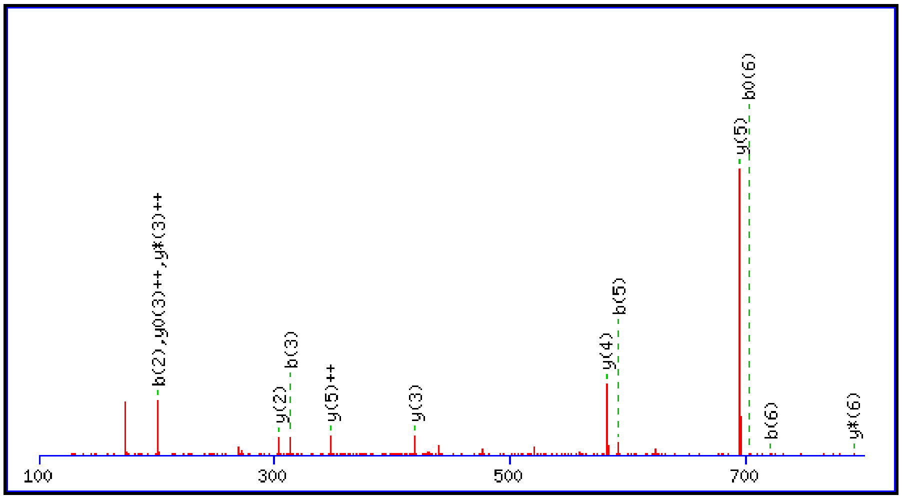

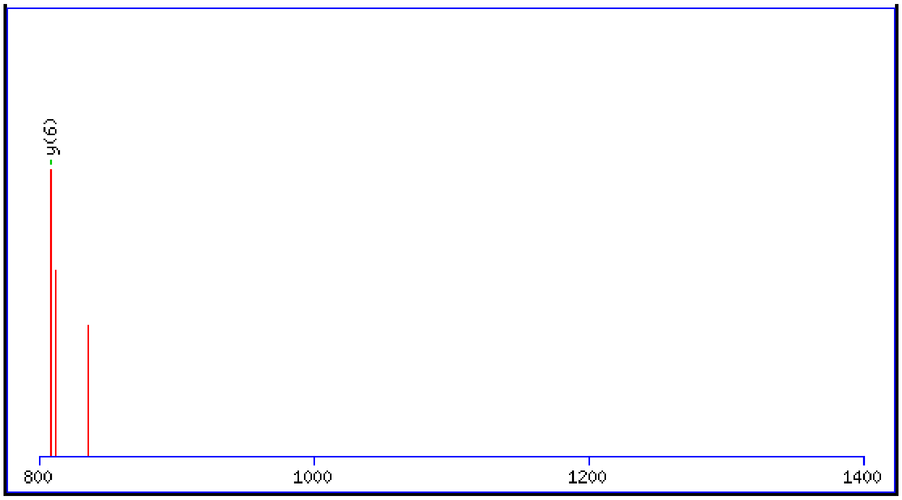

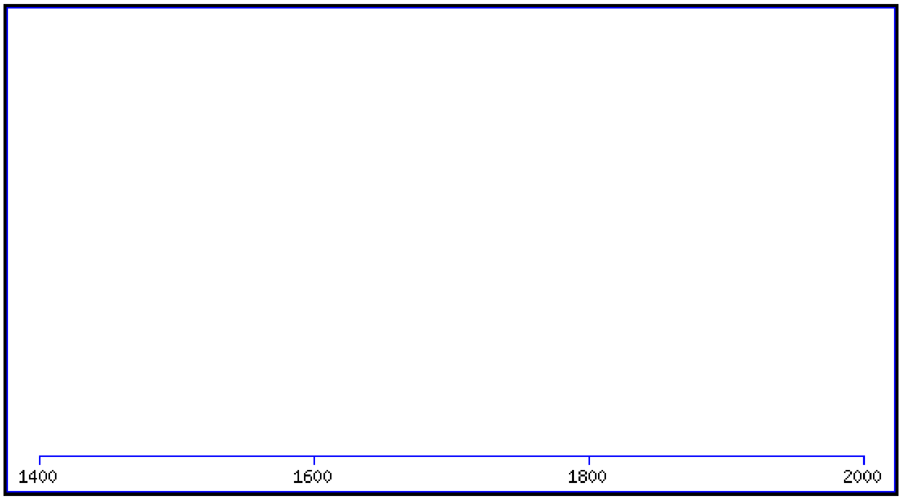

Monoisotopic mass of neutral peptide Mr(calc): 894.4447
Fixed modifications: Carbamidomethyl (C)
Ions Score: 37 **Expect:** 0.023
Matches (Bold Red): 14/58 fragment ions using 19 most intense peaks

#	b	b^{++}	b^{0}	b^{0++}	Seq.	y	y^{++}	y*	y^{*++}	y^{0}	y^{0++}	#
1	88.0393	44.5233	70.0287	35.5180	S							7
2	**201.1234**	101.0653	183.1128	92.0600	I	**808.4199**	404.7136	**791.3934**	396.2003	790.4094	395.7083	6
3	**314.2074**	157.6074	296.1969	148.6021	L	**695.3359**	**348.1716**	678.3093	339.6583	677.3253	339.1663	5
4	477.2708	239.1390	459.2602	230.1337	Y	**582.2518**	291.6295	565.2253	283.1163	564.2413	282.6243	4
5	**592.2977**	296.6525	574.2871	287.6472	D	**419.1885**	210.0979	402.1619	**201.5846**	401.1779	**201.0926**	3
6	**721.3403**	361.1738	**703.3297**	352.1685	E	**304.1615**	152.5844	287.1350	144.0711	286.1510	143.5791	2
7					R	175.1190	88.0631	158.0924	79.5498			1

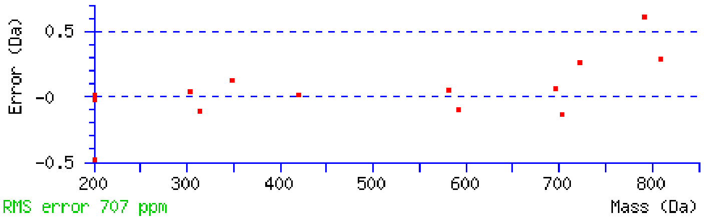

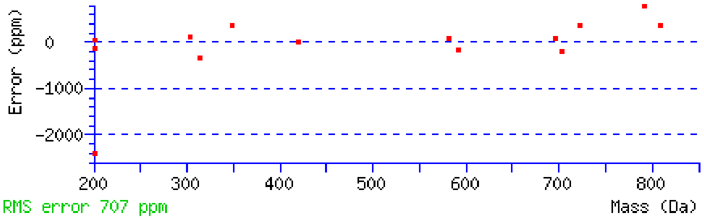

All matches to this query

Score	Mr(calc):	Delta	Sequence
37.3	894.4447	0.0013	SILYDER
10.6	892.4419	2.0040	VVIPTER

9.2	893.4484	0.9976	KAGGTPRK
8.7	892.4419	2.0040	VVLETPR
8.5	894.4447	0.0013	SLYDELR
7.7	893.4372	1.0088	TLGIASPR
6.3	893.4372	1.0088	TLGIASPR
5.9	893.4372	1.0088	LSLTAGPR
5.7	893.4484	0.9976	XLTAPRR
4.8	893.4454	1.0005	SLSSTSAGGK

Spectrum No: 116; Query: 50793; Rank: 1

Peptide View

MS/MS Fragmentation of **SVHKVEPITK**
Found in **IPI00219622**, Tax_Id=9606 Gene_Symbol=PSMA2 Proteasome subunit alpha type-2

Match to Query 50793: 1136.657952 from(379.893260,3+)
Title: 090702LimSK_Exosome2_06.5633.5633.3.dta
Data file C:\Temp\Mascoti\LSK_2D\090702LimSK_Exosome2_01_2D.mgf

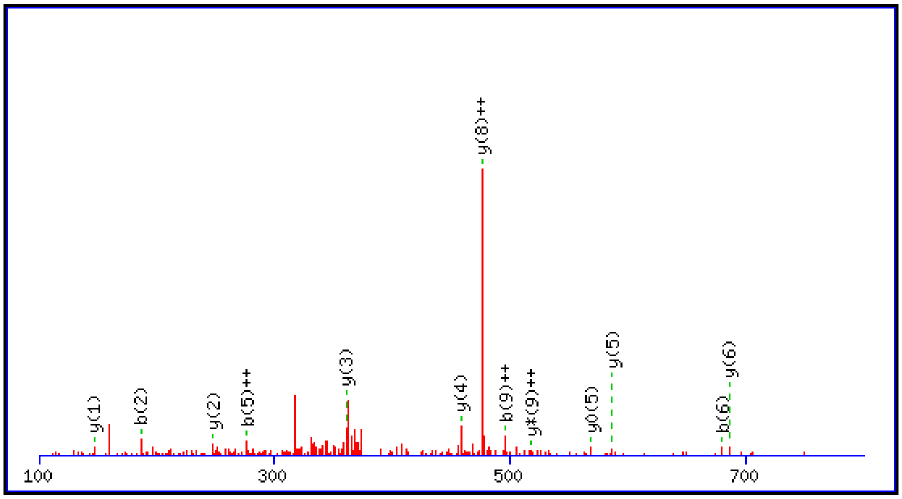

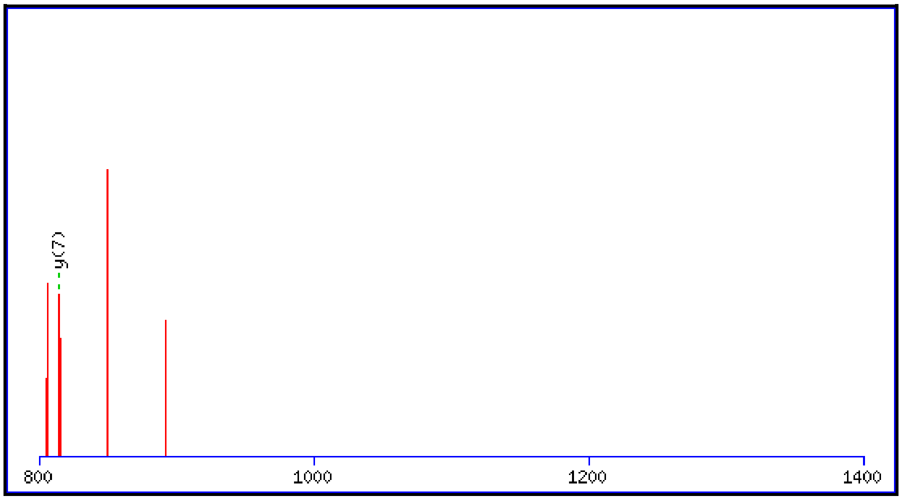

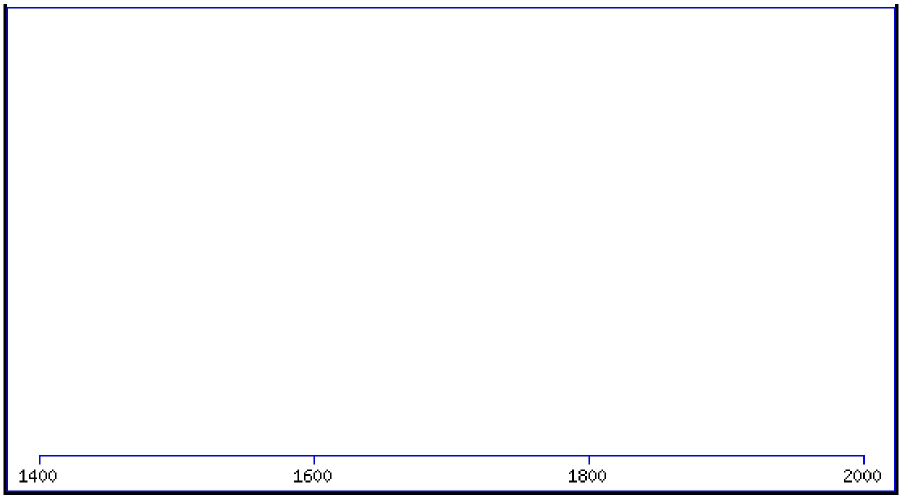

Monoisotopic mass of neutral peptide Mr(calc): 1136.6554
Fixed modifications: Carbamidomethyl (C)
Ions Score: 44 **Expect:** 0.0015
Matches (Bold Red): 14/100 fragment ions using 26 most intense peaks

#	b	b^{++}	b*	b^{*++}	b^0	b^{0++}	Seq.	y	y^{++}	y*	y^{*++}	y^0	y^{0++}	#
1	88.0393	44.5233			70.0287	35.5180	S							10
2	**187.1077**	94.0575			169.0972	85.0522	V	1050.6306	525.8189	1033.6041	**517.3057**	1032.6200	516.8137	9
3	324.1666	162.5870			306.1561	153.5817	H	951.5622	**476.2847**	934.5356	467.7715	933.5516	467.2795	8
4	452.2616	226.6344	435.2350	218.1212	434.2510	217.6292	K	**814.5033**	407.7553	797.4767	399.2420	796.4927	398.7500	7
5	551.3300	**276.1686**	534.3035	267.6554	533.3194	267.1634	V	**686.4083**	343.7078	669.3818	335.1945	668.3978	334.7025	6
6	**680.3726**	340.6899	663.3461	332.1767	662.3620	331.6847	E	**587.3399**	294.1736	570.3134	285.6603	**569.3293**	285.1683	5
7	777.4254	389.2163	760.3988	380.7030	759.4148	380.2110	P	**458.2973**	229.6523	441.2708	221.1390	440.2867	220.6470	4
8	890.5094	445.7584	873.4829	437.2451	872.4989	436.7531	I	**361.2445**	181.1259	344.2180	172.6126	343.2340	172.1206	3
9	991.5571	**496.2822**	974.5306	487.7689	973.5465	487.2769	T	**248.1605**	124.5839	231.1339	116.0706	230.1499	115.5786	2
10							K	**147.1128**	74.0600	130.0863	65.5468			1

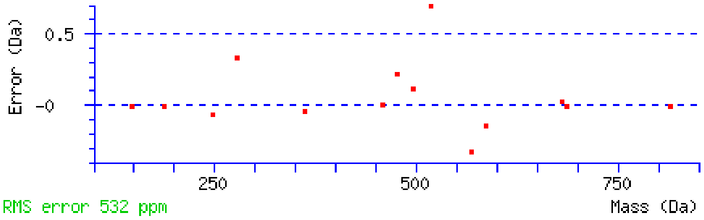

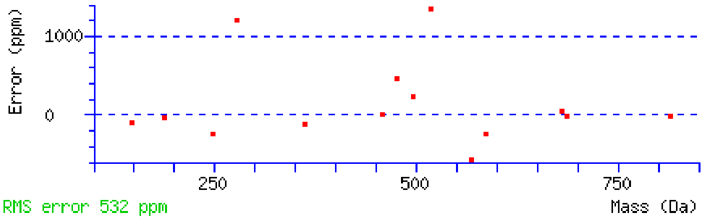

All matches to this query

Score	Mr(calc):	Delta	Sequence
44.5	1136.6554	0.0026	SVHKVEPITK
7.1	1136.6666	-0.0086	VHREIVSGLK
5.3	1135.6489	1.0091	ISDSVLVYIK
5.1	1136.6594	-0.0014	FPFISLASKK
4.8	1135.6574	1.0005	SVLIQHQRR
2.2	1134.6413	2.0166	KVLTPIKEK
1.7	1135.6601	0.9979	SVITNFSKLK
1.3	1134.6471	2.0109	AIPMYKIATK
0.7	1135.6601	0.9979	ELFSSLKKGK
0.3	1135.6574	1.0006	EGARAHLKVR

Spectrum No: 117; Query: 31954; Rank: 1

Peptide View

MS/MS Fragmentation of **TFLEKR**
Found in **IPI00219622**, Tax_Id=9606 Gene_Symbol=PSMA2 Proteasome subunit alpha type-2

Match to Query 31954: 792.450908 from(397.232730,2+)
Title: 090702LimSK_Exosome2_05.4039.4039.2.dta
Data file C:\Temp\Mascoti\LSK_2D\090702LimSK_Exosome2_01_2D.mgf

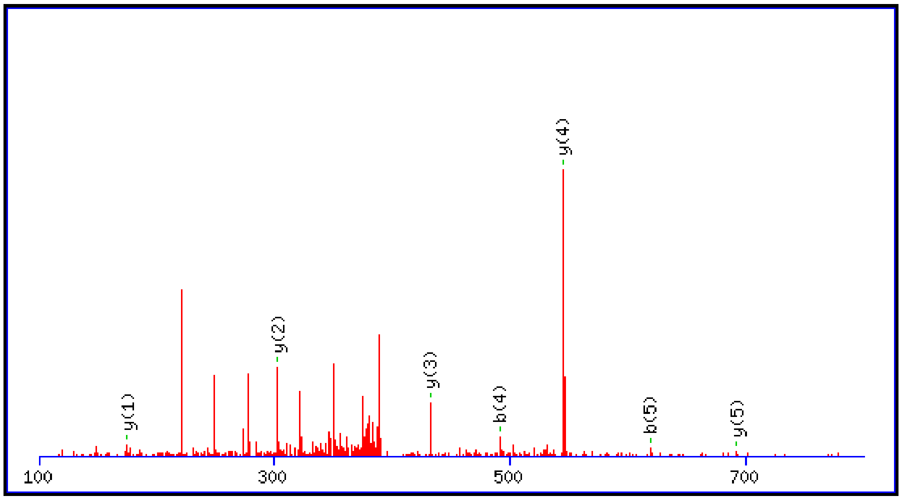

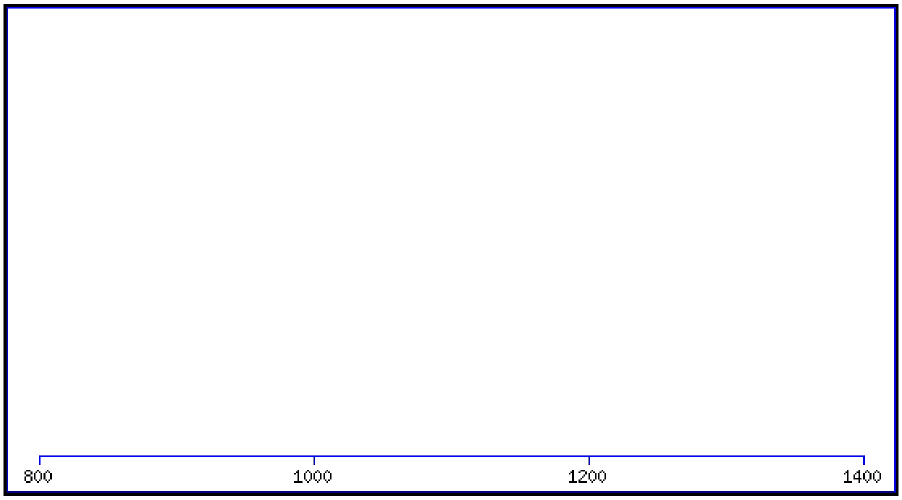

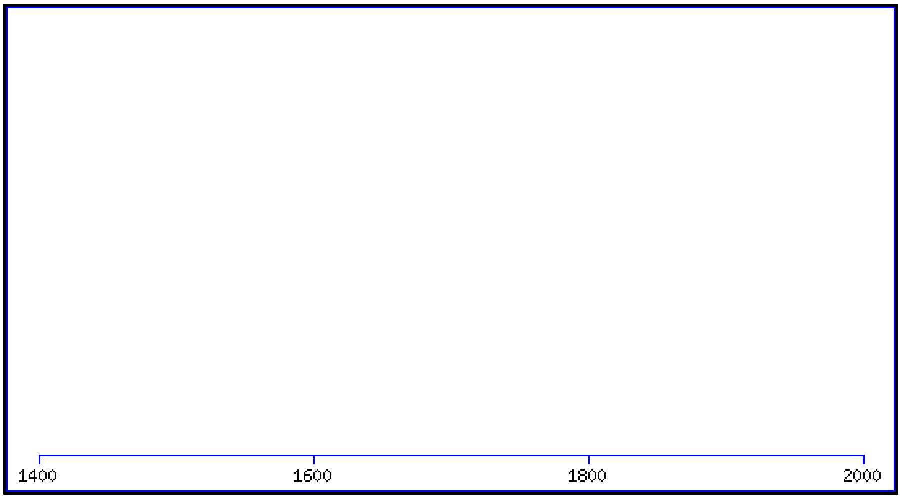

Monoisotopic mass of neutral peptide Mr(calc): 792.4494
Fixed modifications: Carbamidomethyl (C)
Ions Score: 33 **Expect:** 0.04

Matches (Bold Red): 7/48 fragment ions using 16 most intense peaks

#	b	b++	b*	b*++	b0	b0++	Seq.	y	y++	y*	y*++	y0	y0++	#
1	102.0550	51.5311			84.0444	42.5258	T							6
2	249.1234	125.0653			231.1128	116.0600	F	**692.4090**	346.7081	675.3824	338.1949	674.3984	337.7028	5
3	362.2074	181.6074			344.1969	172.6021	L	**545.3406**	273.1739	528.3140	264.6606	527.3300	264.1686	4
4	**491.2500**	246.1287			473.2395	237.1234	E	**432.2565**	216.6319	415.2300	208.1186	414.2459	207.6266	3
5	**619.3450**	310.1761	602.3184	301.6629	601.3344	301.1709	K	**303.2139**	152.1106	286.1874	143.5973			2
6							R	**175.1190**	88.0631	158.0924	79.5498			1

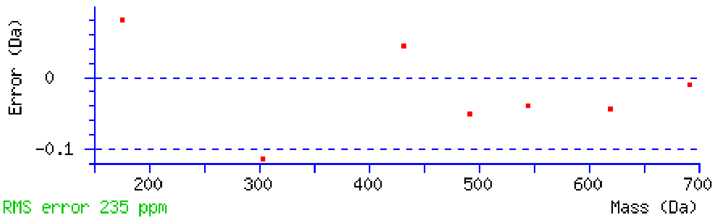

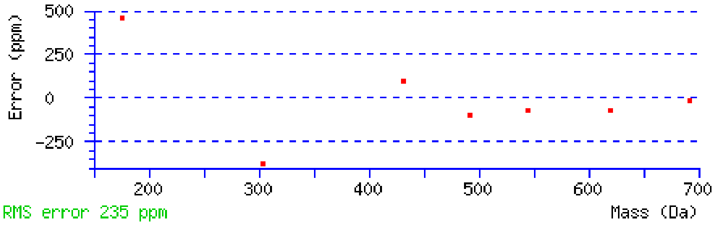

All matches to this query

Score	Mr(calc):	Delta	Sequence
32.8	792.4494	0.0015	TFLEKR
16.1	790.4450	2.0059	VFNQKR
15.9	792.4494	0.0015	TFIGTVR
15.9	792.4494	0.0015	TFLKER
8.5	790.4409	2.0100	TGSSRKR
8.0	790.4371	2.0138	MVSIGLR
8.0	792.4494	0.0015	FITEKR
7.5	792.4494	0.0015	TFELRK
5.0	792.4494	0.0015	FTKEIR
4.7	790.4371	2.0138	LVMEKR

Spectrum No: 118; Query: 88290; Rank: 1

Peptide View

MS/MS Fragmentation of **YNEDLELEDAIHTAILTLK**
Found in **IPI00219622**, Tax_Id=9606 Gene_Symbol=PSMA2 Proteasome subunit alpha type-2

Match to Query 88290: 2201.142042 from(734.721290,3+)
Title: 090702LimSK_Exosome2_01.10392.10392.3.dta
Data file C:\Temp\Mascoti\LSK_2D\090702LimSK_Exosome2_01_2D.mgf

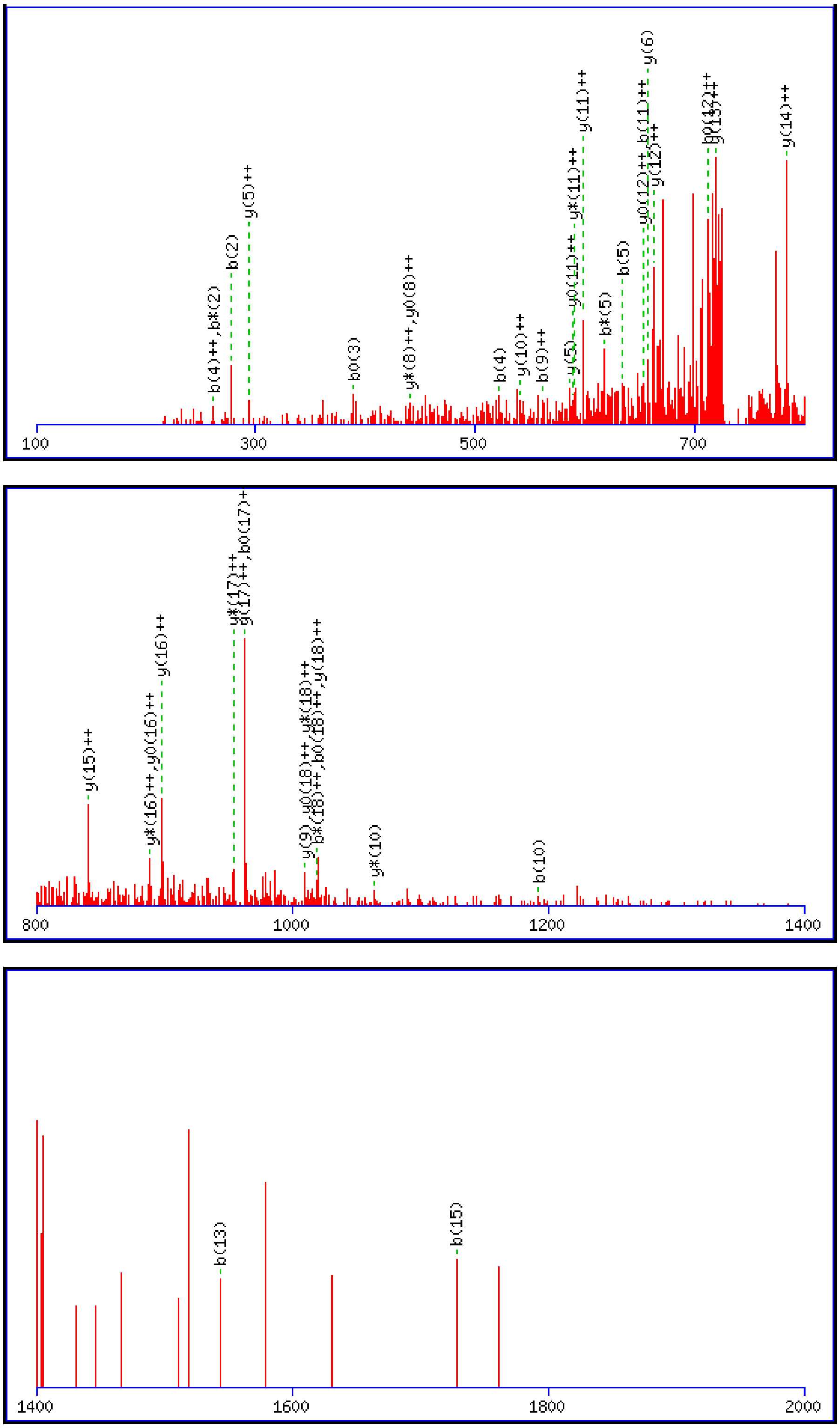

Monoisotopic mass of neutral peptide Mr(calc): 2200.1263
Fixed modifications: Carbamidomethyl (C)
Ions Score: 25 **Expect:** 0.45
Matches (Bold Red): 41/206 fragment ions using 85 most intense peaks

#	b	b^{++}	b*	b^{*++}	b^{0}	b^{0++}	Seq.	y	y^{++}	y*	y^{*++}	y^{0}	y^{0++}	#
1	164.0706	82.5389					Y							19
2	**278.1135**	139.5604	**261.0870**	131.0471			N	2038.0703	**1019.5388**	2021.0437	**1011.0255**	2020.0597	**1010.5335**	18
3	407.1561	204.0817	390.1296	195.5684	**389.1456**	195.0764	E	1924.0274	**962.5173**	1907.0008	**954.0040**	1906.0168	953.5120	17
4	**522.1831**	**261.5952**	505.1565	253.0819	504.1725	252.5899	D	1794.9848	**897.9960**	1777.9582	**889.4827**	1776.9742	**888.9907**	16
5	**635.2671**	318.1372	**618.2406**	309.6239	617.2566	309.1319	L	1679.9578	**840.4825**	1662.9313	831.9693	1661.9472	831.4773	15
6	764.3097	382.6585	747.2832	374.1452	746.2992	373.6532	E	1566.8738	**783.9405**	1549.8472	775.4272	1548.8632	774.9352	14

7	877.3938	439.2005	860.3672	430.6873	859.3832	430.1953	L	1437.8312	**719.4192**	1420.8046	710.9059	1419.8206	710.4139	13
8	1006.4364	503.7218	989.4098	495.2086	988.4258	494.7165	E	1324.7471	**662.8772**	1307.7205	654.3639	1306.7365	**653.8719**	12
9	1121.4633	**561.2353**	1104.4368	552.7220	1103.4528	552.2300	D	1195.7045	**598.3559**	1178.6780	**589.8426**	1177.6939	**589.3506**	11
10	**1192.5004**	596.7539	1175.4739	588.2406	1174.4899	587.7486	A	1080.6776	**540.8424**	**1063.6510**	532.3291	1062.6670	531.8371	10
11	1305.5845	**653.2959**	1288.5580	644.7826	1287.5739	644.2906	I	**1009.6404**	505.3239	992.6139	496.8106	991.6299	496.3186	9
12	1442.6434	721.8253	1425.6169	713.3121	1424.6329	**712.8201**	H	896.5564	448.7818	879.5298	**440.2686**	878.5458	**439.7765**	8
13	**1543.6911**	772.3492	1526.6645	763.8359	1525.6805	763.3439	T	759.4975	380.2524	742.4709	371.7391	741.4869	371.2471	7
14	1614.7282	807.8677	1597.7017	799.3545	1596.7176	798.8625	A	**658.4498**	329.7285	641.4232	321.2153	640.4392	320.7233	6
15	**1727.8123**	864.4098	1710.7857	855.8965	1709.8017	855.4045	I	**587.4127**	**294.2100**	570.3861	285.6967	569.4021	285.2047	5
16	1840.8963	920.9518	1823.8698	912.4385	1822.8858	911.9465	L	474.3286	237.6679	457.3021	229.1547	456.3180	228.6627	4
17	1941.9440	971.4756	1924.9175	**962.9624**	1923.9334	**962.4704**	T	361.2445	181.1259	344.2180	172.6126	343.2340	172.1206	3
18	2055.0281	1028.0177	2038.0015	**1019.5044**	2037.0175	**1019.0124**	L	260.1969	130.6021	243.1703	122.0888			2
19							K	147.1128	74.0600	130.0863	65.5468			1

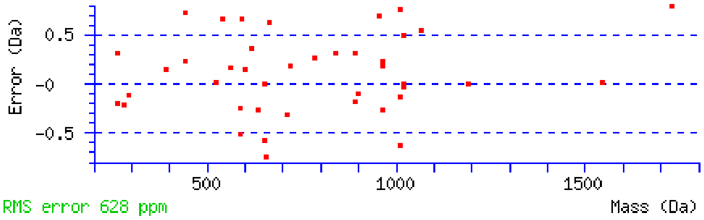

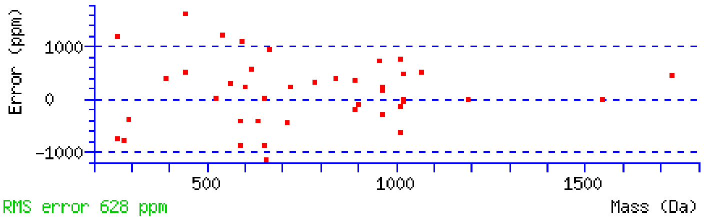

All matches to this query

Score	Mr(calc):	Delta	Sequence
25.1	2200.1263	1.0158	YNEDLELEDAIHTAILTLK
3.0	2199.1560	1.9860	QSLTWLLITRMKIIMEK
2.9	2199.1560	1.9860	QSLTWLLITRMKIIMEK
2.9	2199.1560	1.9860	QSLTWLLITRMKIIMEK
1.5	2200.1293	1.0127	LFGPTLSAKIQFNTDLSLR
1.2	2199.1487	1.9934	TSAMLTVRALPIKFTEGLR
0.5	2199.1341	2.0080	EFGPVVIDYGKVQSKVNLK

Spectrum No: 119; Query: 77553; Rank: 1

Peptide View

MS/MS Fragmentation of **EVLEKQPVLSQTEAR**
Found in **IPI00555956**, Tax_Id=9606 Gene_Symbol=PSMB4 Proteasome subunit beta type-4

Match to Query 77553: 1725.929862 from(576.317230,3+)
Title: 090702LimSK_Exosome2_05.5450.5450.3.dta
Data file C:\Temp\Mascoti\LSK_2D\090702LimSK_Exosome2_01_2D.mgf

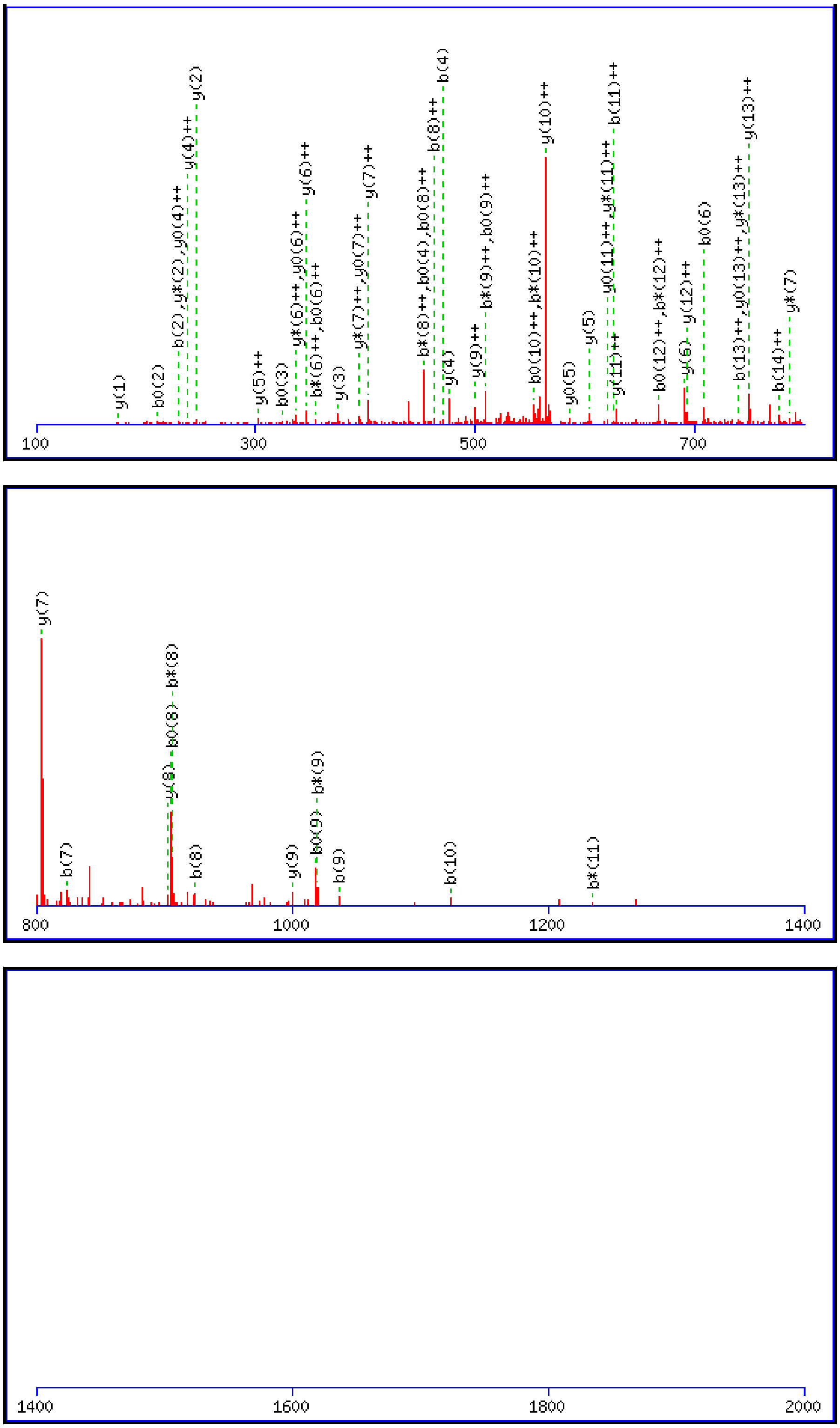

Monoisotopic mass of neutral peptide Mr(calc): 1725.9261
Fixed modifications: Carbamidomethyl (C)
Ions Score: 58 **Expect:** 0.00014
Matches (Bold Red): 59/156 fragment ions using 86 most intense peaks

#	b	b^{++}	b*	b^{*++}	b^0	b^{0++}	Seq.	y	y^{++}	y*	y^{*++}	y^0	y^{0++}	#
1	130.0499	65.5286			112.0393	56.5233	E							15
2	**229.1183**	115.0628			**211.1077**	106.0575	V	1597.8908	799.4490	1580.8642	790.9358	1579.8802	790.4438	14
3	342.2023	171.6048			**324.1918**	162.5995	L	1498.8224	**749.9148**	1481.7958	**741.4016**	1480.8118	**740.9095**	13
4	**471.2449**	236.1261			**453.2344**	227.1208	E	1385.7383	**693.3728**	1368.7118	684.8595	1367.7278	684.3675	12
5	599.3399	300.1736	582.3134	291.6603	581.3293	291.1683	K	1256.6957	**628.8515**	1239.6692	**620.3382**	1238.6852	**619.8462**	11
6	727.3985	364.2029	710.3719	**355.6896**	**709.3879**	**355.1976**	Q	1128.6008	**564.8040**	1111.5742	556.2907	1110.5902	555.7987	10

7	824.4512	412.7293	807.4247	404.2160	806.4407	403.7240	P	1000.5422	500.7747	983.5156	492.2615	982.5316	491.7694	9
8	923.5197	462.2635	906.4931	453.7502	905.5091	453.2582	V	903.4894	452.2483	886.4629	443.7351	885.4789	443.2431	8
9	1036.6037	518.8055	1019.5772	510.2922	1018.5932	509.8002	L	804.4210	402.7141	787.3945	394.2009	786.4104	393.7089	7
10	1123.6358	562.3215	1106.6092	553.8082	1105.6252	553.3162	S	691.3369	346.1721	674.3104	337.6588	673.3264	337.1668	6
11	1251.6943	626.3508	1234.6678	617.8375	1233.6838	617.3455	Q	604.3049	302.6561	587.2784	294.1428	586.2944	293.6508	5
12	1352.7420	676.8746	1335.7155	668.3614	1334.7314	667.8694	T	476.2463	238.6268	459.2198	230.1135	458.2358	229.6215	4
13	1481.7846	741.3959	1464.7581	732.8827	1463.7740	732.3907	E	375.1987	188.1030	358.1721	179.5897	357.1881	179.0977	3
14	1552.8217	776.9145	1535.7952	768.4012	1534.8112	767.9092	A	246.1561	123.5817	229.1295	115.0684			2
15							R	175.1190	88.0631	158.0924	79.5498			1

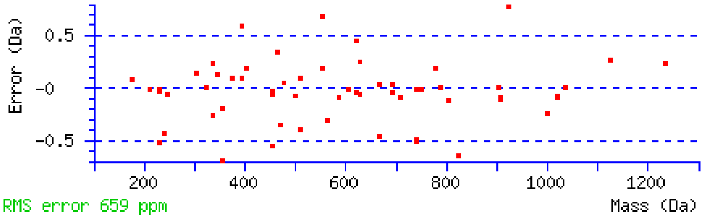

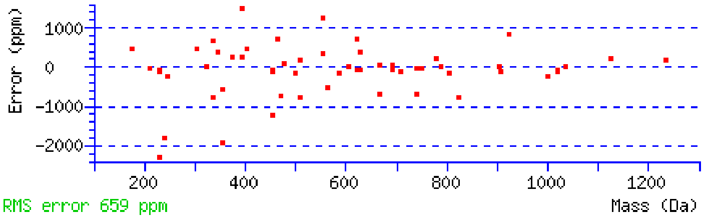

All matches to this query

Score	Mr(calc):	Delta	Sequence
58.4	1725.9261	0.0038	EVLEKQPVLSQTEAR
14.1	1725.9162	0.0137	RGLYLSDIPLHDATR
6.3	1724.9114	1.0185	KPKVQLVISYEDVK
6.0	1723.9208	2.0091	QKMWLKLGLAITAR
5.3	1724.9131	1.0168	SDMNKPHLISELLTK
4.3	1725.9195	0.0104	TLTKVLALVKEVPR
3.9	1723.9080	2.0219	VKNVLGIEGFTNFMR
3.6	1723.9134	2.0165	NLIQKAGYLNLRNK
3.6	1723.9297	2.0001	IIVFFSLPNIGDFSR
3.3	1724.9282	1.0017	AKDGTHVLQGLSSRTR

Spectrum No: 120; Query: 44573; Rank: 1

Peptide View

MS/MS Fragmentation of **FQIATVTEK**
Found in **IPI00555956**, Tax_Id=9606 Gene_Symbol=PSMB4 Proteasome subunit beta type-4

Match to Query 44573: 1035.560948 from(518.787750,2+)
Title: 090702LimSK_Exosome2_06.3978.3978.2.dta
Data file C:\Temp\Mascoti\LSK_2D\090702LimSK_Exosome2_01_2D.mgf

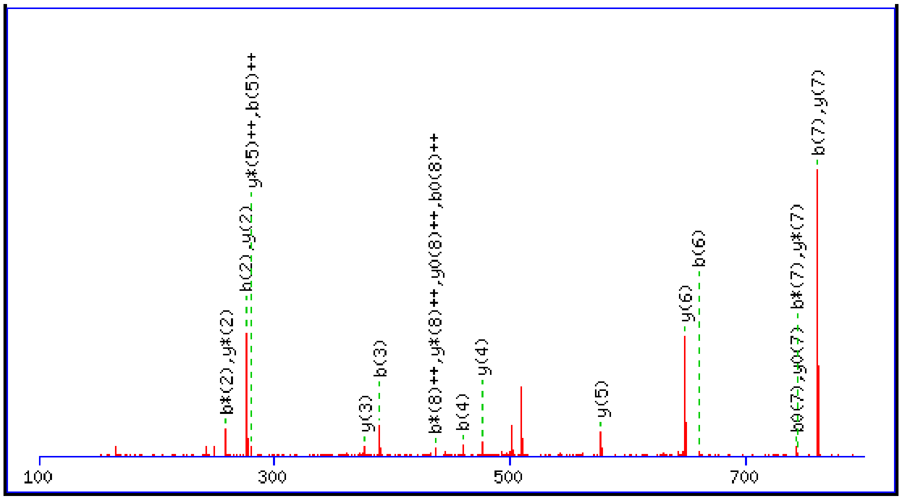

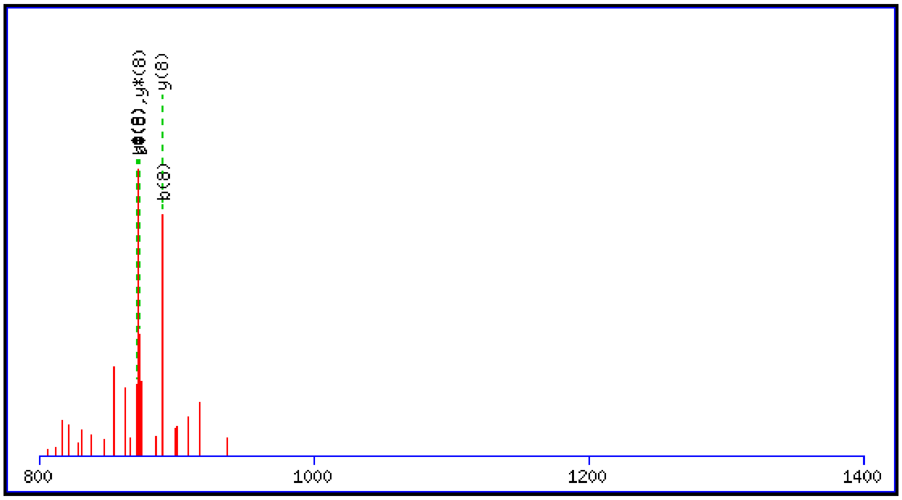

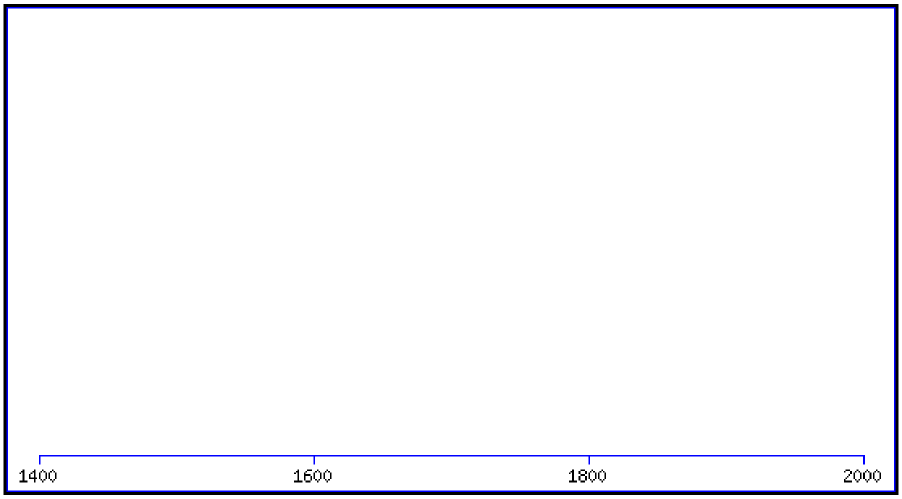

Monoisotopic mass of neutral peptide Mr(calc): 1035.5601
Fixed modifications: Carbamidomethyl (C)
Ions Score: 50 **Expect:** 0.0013
Matches (Bold Red): 29/84 fragment ions using 36 most intense peaks

#	b	b^{++}	b*	b^{*++}	b^0	b^{0++}	Seq.	y	y^{++}	y*	y^{*++}	y^0	y^{0++}	#
1	148.0757	74.5415					F							9
2	**276.1343**	138.5708	**259.1077**	130.0575			Q	**889.4989**	445.2531	**872.4724**	**436.7398**	**871.4884**	**436.2478**	8
3	**389.2183**	195.1128	372.1918	186.5995			I	**761.4403**	381.2238	**744.4138**	372.7105	**743.4298**	372.2185	7
4	**460.2554**	230.6314	443.2289	222.1181			A	**648.3563**	324.6818	631.3297	316.1685	630.3457	315.6765	6
5	561.3031	**281.1552**	544.2766	272.6419	543.2926	272.1499	T	**577.3192**	289.1632	560.2926	**280.6499**	559.3086	280.1579	5
6	**660.3715**	330.6894	643.3450	322.1761	642.3610	321.6841	V	**476.2715**	238.6394	459.2449	230.1261	458.2609	229.6341	4

7	761.4192	381.2132	744.3927	372.7000	743.4087	372.2080	T	377.2031	189.1052	360.1765	180.5919	359.1925	180.0999	3
8	890.4618	445.7345	873.4353	437.2213	872.4512	436.7293	E	276.1554	138.5813	259.1288	130.0681	258.1448	129.5761	2
9							K	147.1128	74.0600	130.0863	65.5468			1

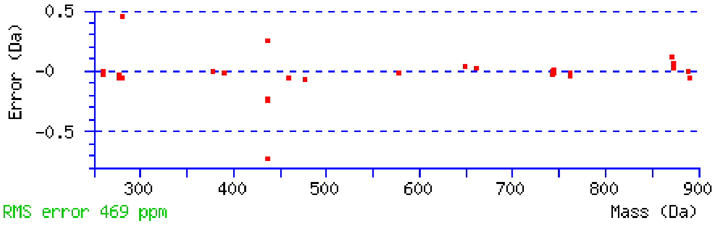

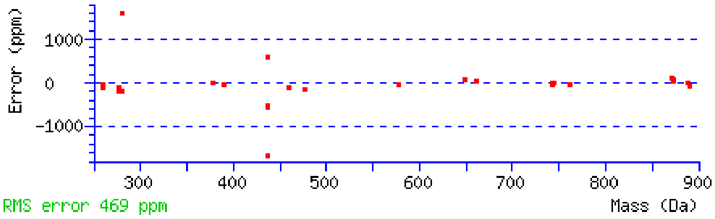

All matches to this query

Score	Mr(calc):	Delta	Sequence
49.5	1035.5601	0.0009	FQIATVTEK
25.6	1035.5600	0.0009	FKIDASVEK
20.9	1035.5600	0.0009	VAAYDKLEK
20.1	1034.5525	1.0084	GAITQPLKK
17.4	1034.5542	1.0067	RKSEMLQK
17.0	1035.5600	0.0009	EEKDFLKK
15.3	1035.5712	-0.0103	AYREKIEK
15.3	1035.5600	0.0009	IFKEDKEK
15.3	1035.5600	0.0009	KNLEYIEK
15.1	1034.5616	0.9993	KQMMLIQK

Spectrum No: 121; Query: 50086; Rank: 1

Peptide View

MS/MS Fragmentation of **QPVLSQTEAR**
Found in **IPI00555956**, Tax_Id=9606 Gene_Symbol=PSMB4 Proteasome subunit beta type-4

Match to Query 50086: 1127.597268 from(564.805910,2+)
Title: 090702LimSK_Exosome2_08b4.1367.1367.2.dta
Data file C:\Temp\Mascoti\LSK_2D\090702LimSK_Exosome2_01_2D.mgf

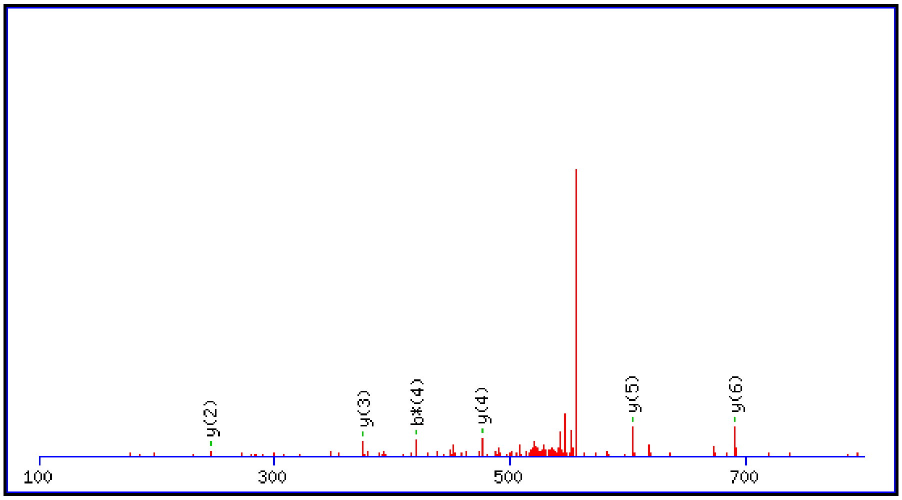

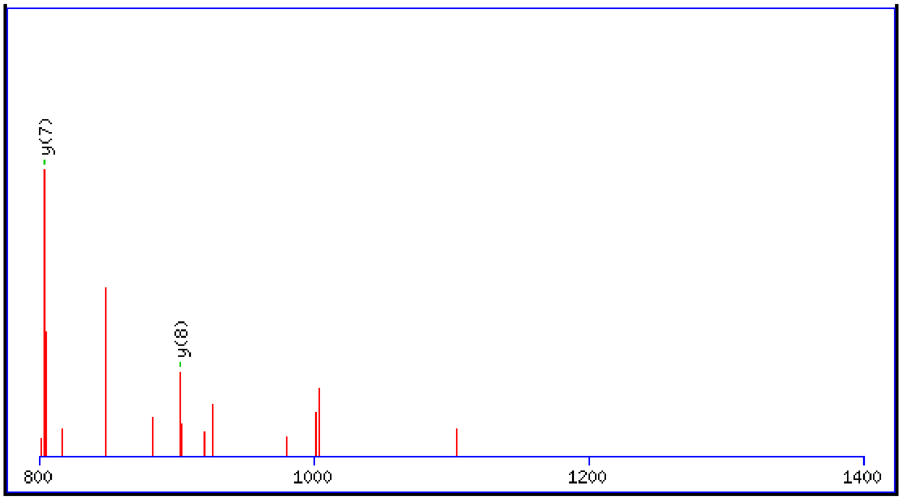

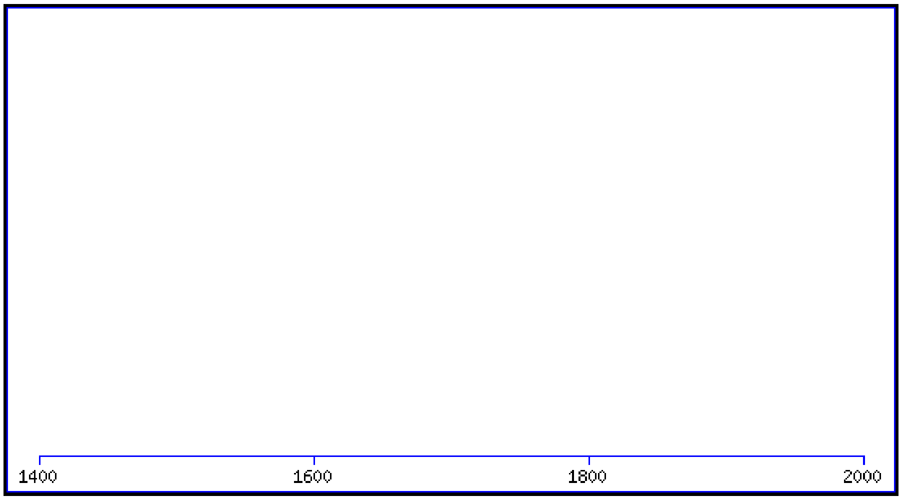

Monoisotopic mass of neutral peptide Mr(calc): 1127.5935
Fixed modifications: Carbamidomethyl (C)
Ions Score: 64 **Expect:** 3.7e-005
Matches (Bold Red): 8/96 fragment ions using 18 most intense peaks

#	b	b^{++}	b*	b^{*++}	b^0	b^{0++}	Seq.	y	y^{++}	y*	y^{*++}	y^0	y^{0++}	#
1	129.0659	65.0366	112.0393	56.5233			Q							10
2	226.1186	113.5629	209.0921	105.0497			P	1000.5422	500.7747	983.5156	492.2615	982.5316	491.7694	9
3	325.1870	163.0972	308.1605	154.5839			V	**903.4894**	452.2483	886.4629	443.7351	885.4789	443.2431	8
4	438.2711	219.6392	**421.2445**	211.1259			L	**804.4210**	402.7141	787.3945	394.2009	786.4104	393.7089	7
5	525.3031	263.1552	508.2766	254.6419	507.2926	254.1499	S	**691.3369**	346.1721	674.3104	337.6588	673.3264	337.1668	6
6	653.3617	327.1845	636.3352	318.6712	635.3511	318.1792	Q	**604.3049**	302.6561	587.2784	294.1428	586.2944	293.6508	5
7	754.4094	377.7083	737.3828	369.1951	736.3988	368.7030	T	**476.2463**	238.6268	459.2198	230.1135	458.2358	229.6215	4
8	883.4520	442.2296	866.4254	433.7164	865.4414	433.2243	E	**375.1987**	188.1030	358.1721	179.5897	357.1881	179.0977	3
9	954.4891	477.7482	937.4625	469.2349	936.4785	468.7429	A	**246.1561**	123.5817	229.1295	115.0684			2
10							R	175.1190	88.0631	158.0924	79.5498			1

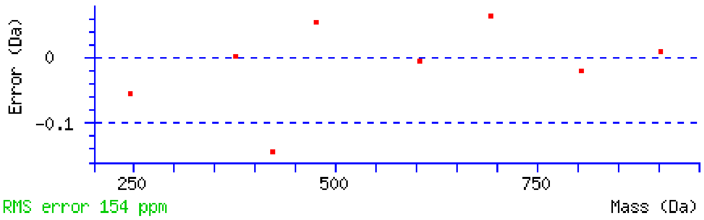

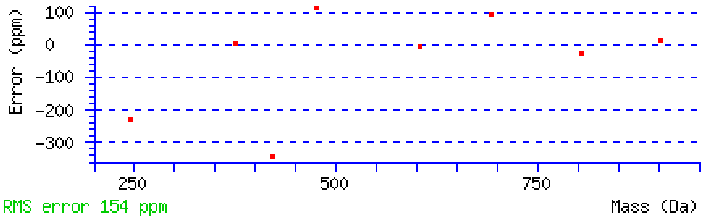

All matches to this query

Score	Mr(calc):	Delta	Sequence
64.2	1127.5935	0.0038	QPVLSQTEAR
13.3	1127.5992	-0.0019	KPVVTIYTK
11.6	1127.5992	-0.0019	KPVVTIYTK
6.6	1125.5931	2.0042	NFKTAATFAR
3.0	1125.5818	2.0154	FVLYKDGER
2.9	1127.6047	-0.0074	RAPVTPSSASR
2.2	1127.5975	-0.0002	AKQLYSAGYK
2.1	1126.5870	1.0102	LVSPPTGDDVK
2.1	1126.5982	0.9991	HSKLAESVEK
2.0	1126.5843	1.0130	DPREVREAR

Spectrum No: 122; Query: 71009; Rank: 1

Peptide View

MS/MS Fragmentation of **TQNPMVTGTSVLGVK**
Found in **IPI00555956**, Tax_Id=9606 Gene_Symbol=PSMB4 Proteasome subunit beta type-4

Match to Query 71009: 1530.811188 from(766.412870,2+)
Title: 090702LimSK_Exosome2_06.6288.6288.2.dta
Data file C:\Temp\Mascoti\LSK_2D\090702LimSK_Exosome2_01_2D.mgf

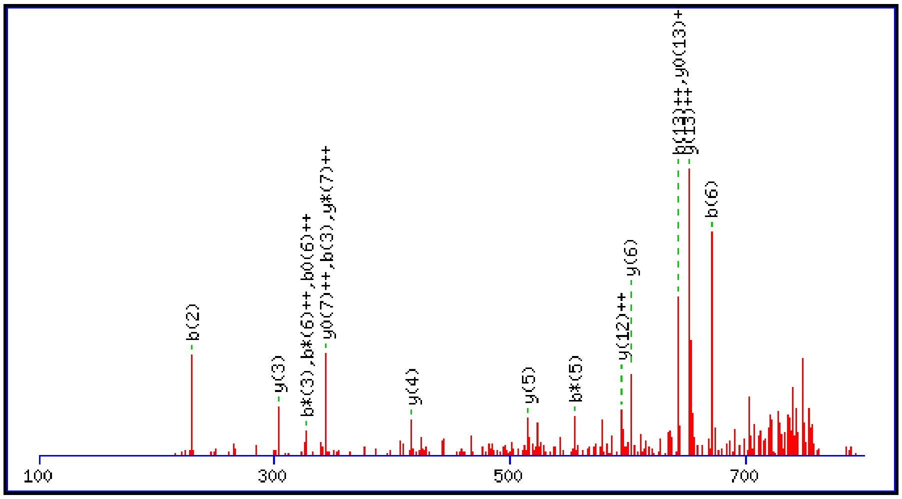

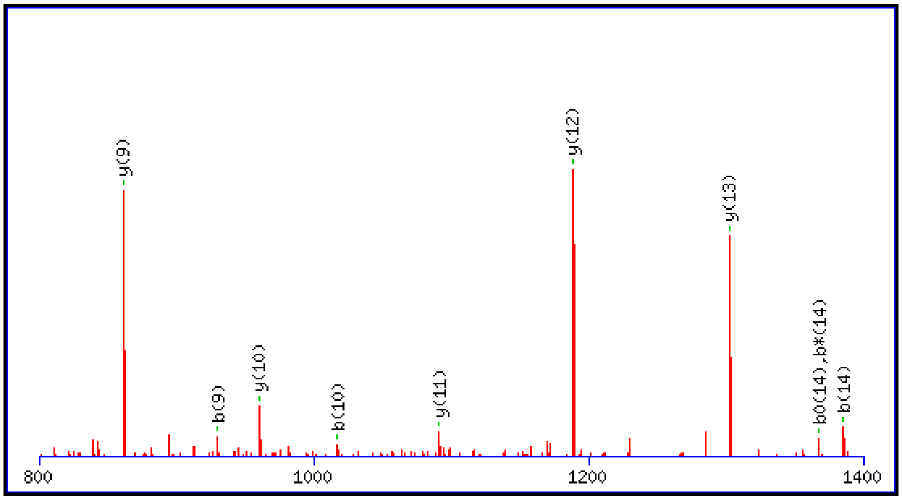

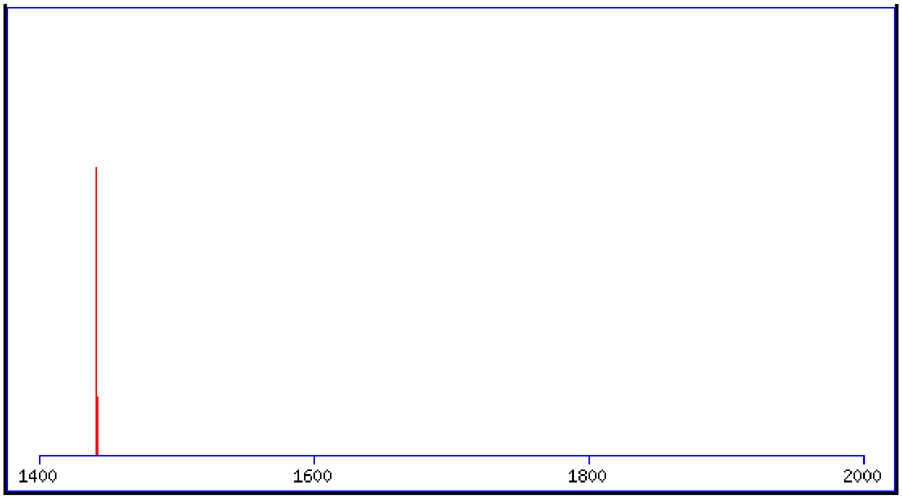

Monoisotopic mass of neutral peptide Mr(calc): 1530.8076
Fixed modifications: Carbamidomethyl (C)
Ions Score: 75 **Expect:** 4.3e-006
Matches (Bold Red): 28/156 fragment ions using 30 most intense peaks

#	b	b^{++}	b*	b^{*++}	b^{0}	b^{0++}	Seq.	y	y^{++}	y*	y^{*++}	y^{0}	y^{0++}	#
1	102.0550	51.5311			84.0444	42.5258	T							15
2	**230.1135**	115.5604	213.0870	107.0471	212.1030	106.5551	Q	1430.7672	715.8872	1413.7406	707.3740	1412.7566	706.8819	14
3	**344.1565**	172.5819	**327.1299**	164.0686	326.1459	163.5766	N	**1302.7086**	**651.8579**	1285.6821	**643.3447**	1284.6980	**642.8527**	13
4	441.2092	221.1083	424.1827	212.5950	423.1987	212.1030	P	**1188.6657**	**594.8365**	1171.6391	586.3232	1170.6551	585.8312	12
5	572.2497	286.6285	**555.2232**	278.1152	554.2391	277.6232	M	**1091.6129**	546.3101	1074.5864	537.7968	1073.6023	537.3048	11
6	**671.3181**	336.1627	654.2916	**327.6494**	653.3076	**327.1574**	V	**960.5724**	480.7899	943.5459	472.2766	942.5619	471.7846	10
7	772.3658	386.6865	755.3393	378.1733	754.3552	377.6813	T	**861.5040**	431.2556	844.4775	422.7424	843.4934	422.2504	9
8	829.3873	415.1973	812.3607	406.6840	811.3767	406.1920	G	760.4563	380.7318	743.4298	372.2185	742.4458	371.7265	8
9	**930.4349**	465.7211	913.4084	457.2078	912.4244	456.7158	T	703.4349	352.2211	686.4083	**343.7078**	685.4243	**343.2158**	7
10	**1017.4670**	509.2371	1000.4404	500.7239	999.4564	500.2318	S	**602.3872**	301.6972	585.3606	293.1840	584.3766	292.6919	6
11	1116.5354	558.7713	1099.5088	550.2581	1098.5248	549.7660	V	**515.3552**	258.1812	498.3286	249.6679			5
12	1229.6195	615.3134	1212.5929	606.8001	1211.6089	606.3081	L	**416.2867**	208.6470	399.2602	200.1337			4
13	1286.6409	**643.8241**	1269.6144	635.3108	1268.6303	634.8188	G	**303.2027**	152.1050	286.1761	143.5917			3
14	**1385.7093**	693.3583	**1368.6828**	684.8450	**1367.6988**	684.3530	V	246.1812	123.5942	229.1547	115.0810			2
15							K	147.1128	74.0600	130.0863	65.5468			1

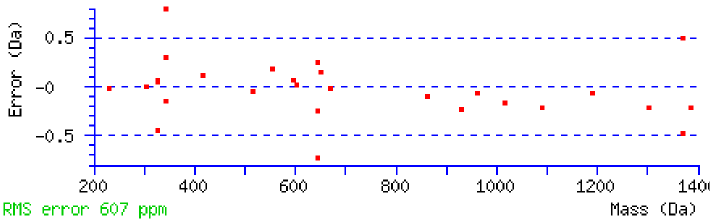

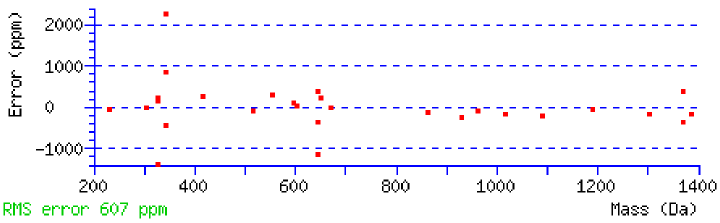

All matches to this query

Score	Mr(calc):	Delta	Sequence
74.9	1530.8076	0.0036	TQNPMVTGTSVLGVK
12.1	1528.8097	2.0015	LNNDLVGSTENLLK
9.8	1528.8167	1.9945	KVIPYIASQFRK
5.3	1529.7984	1.0128	EGRNIIGLQMGTNK
5.3	1529.8161	0.9951	LQTLQSENSNLRK
5.3	1530.8187	-0.0075	QTLKSLIIKLSK
4.7	1528.8167	1.9945	KVIPYIASQFRK

4.6	1529.8161	0.9951	INSTVNKDLENRK
4.3	1530.8089	0.0023	AVRCWGSLEGRLK
3.9	1530.8002	0.0110	TELRLESKDGEVR

Spectrum No: 123; Query: 27435; Rank: 1

Peptide View

MS/MS Fragmentation of **VLYYR**
Found in **IPI00555956**, Tax_Id=9606 Gene_Symbol=PSMB4 Proteasome subunit beta type-4

Match to Query 27435: 712.391808 from(357.203180,2+)
Title: 090702LimSK_Exosome2_06.2240.2240.2.dta
Data file C:\Temp\Mascoti\LSK_2D\090702LimSK_Exosome2_01_2D.mgf

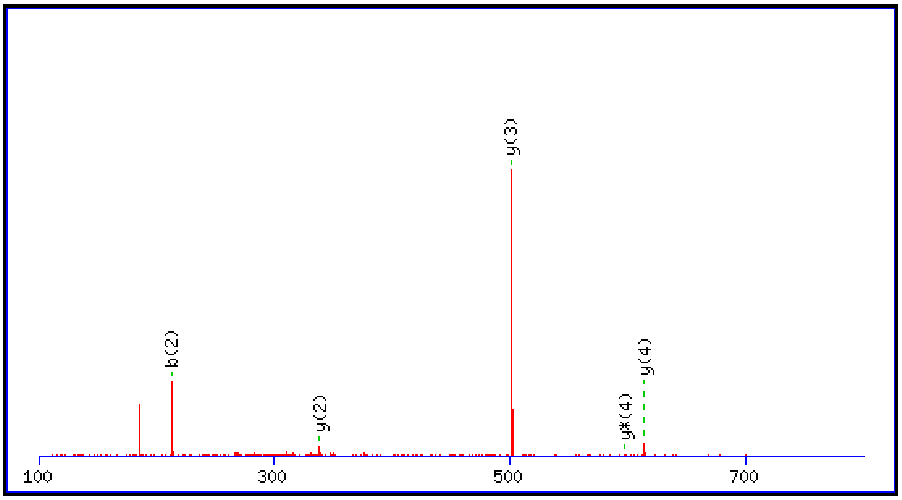

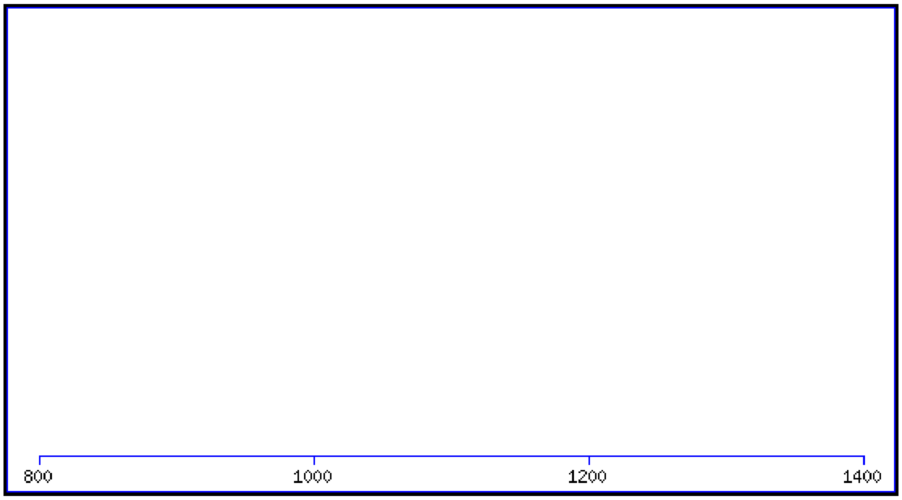

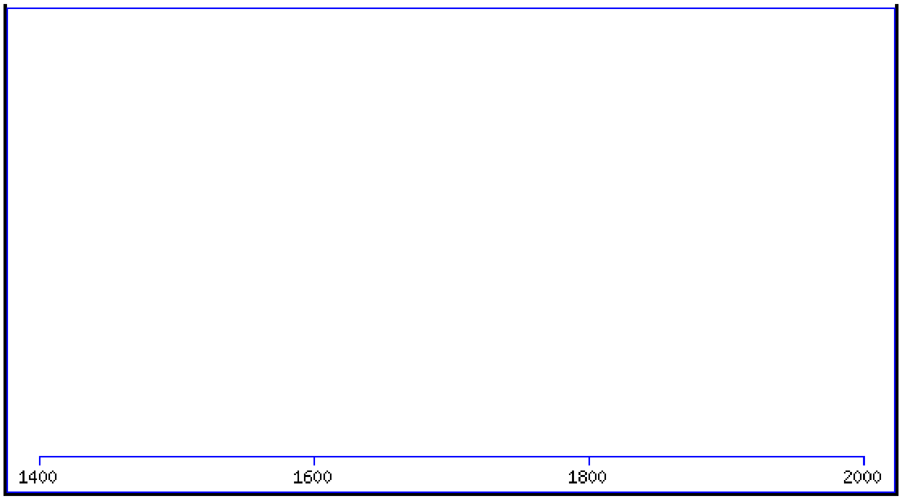

Monoisotopic mass of neutral peptide Mr(calc): 712.3908
Fixed modifications: Carbamidomethyl (C)
Ions Score: 23 **Expect:** 0.42
Matches (Bold Red): 5/24 fragment ions using 7 most intense peaks

#	b	b^{++}	Seq.	y	y^{++}	y*	y^{*++}	#
1	100.0757	50.5415	V					5
2	**213.1598**	107.0835	L	**614.3297**	307.6685	**597.3031**	299.1552	4
3	376.2231	188.6152	Y	**501.2456**	251.1264	484.2191	242.6132	3
4	539.2864	270.1468	Y	**338.1823**	169.5948	321.1557	161.0815	2
5			R	175.1190	88.0631	158.0924	79.5498	1

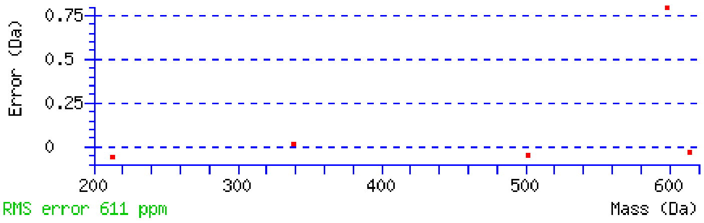

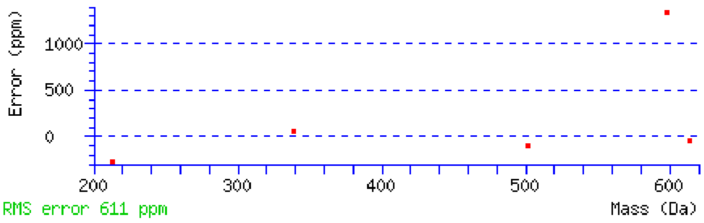

All matches to this query

Score	Mr(calc):	Delta	Sequence
22.7	712.3908	0.0010	VLYYR
19.2	712.3868	0.0050	DPGGVLR
11.6	712.3868	0.0050	DPAALAR
10.6	712.3868	0.0050	VIPDNR
10.2	712.3868	0.0050	DGVPGIR
5.4	712.3980	-0.0062	XRGPLR
4.5	712.3980	-0.0062	DRPLGR
3.1	712.3868	0.0050	TKPPDR
1.3	712.3868	0.0050	NPLDVR
1.1	710.3824	2.0095	ERGPPR

Spectrum No: 124; Query: 88232; Rank: 1

Peptide View

MS/MS Fragmentation of **VNNSTMLGASGDYADFQYLK**
Found in **IPI00555956**, Tax_Id=9606 Gene_Symbol=PSMB4 Proteasome subunit beta type-4

Match to Query 88232: 2193.009548 from(1097.512050,2+)
Title: 090702LimSK_Exosome2_06.8257.8257.2.dta
Data file C:\Temp\Mascoti\LSK_2D\090702LimSK_Exosome2_01_2D.mgf

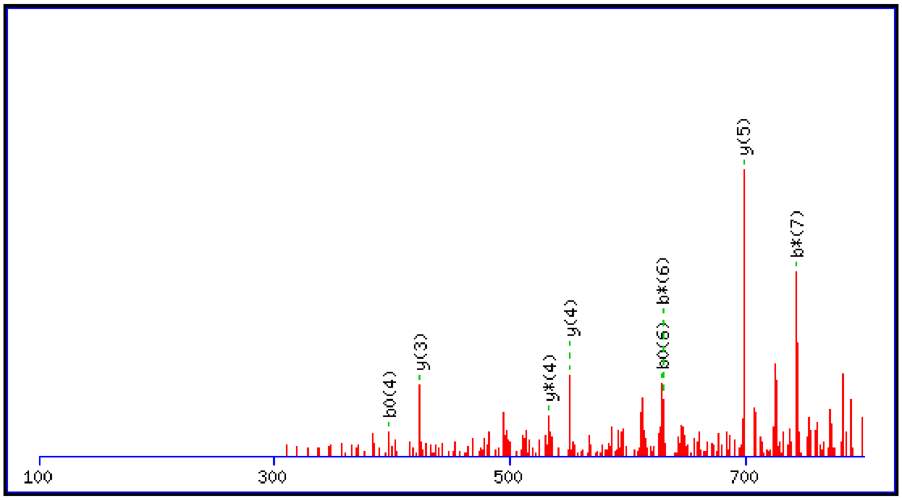

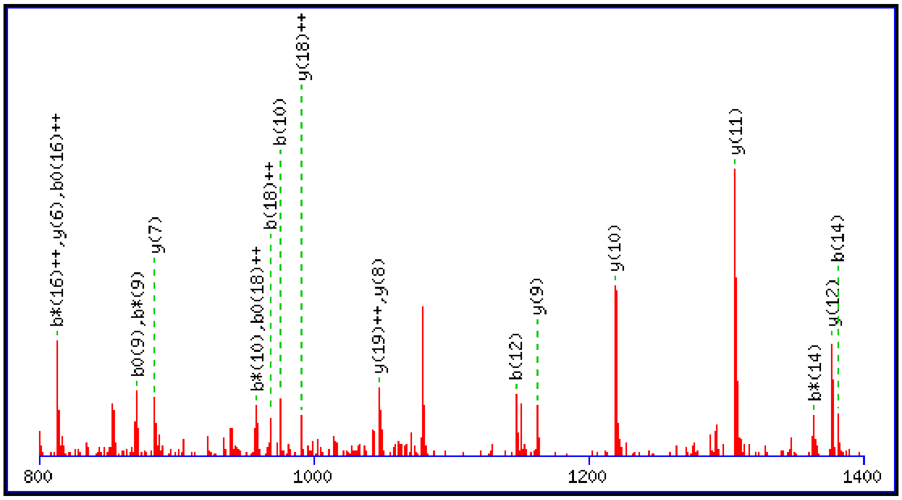

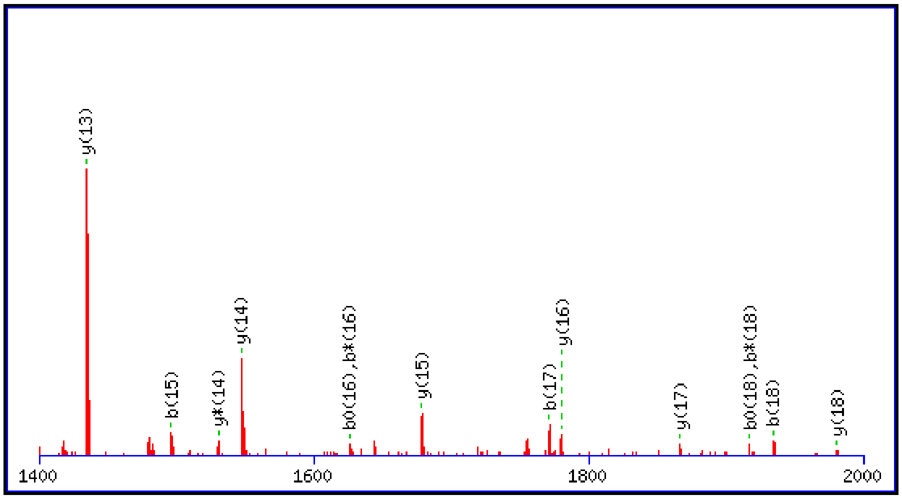

Monoisotopic mass of neutral peptide Mr(calc): 2193.0048
Fixed modifications: Carbamidomethyl (C)
Ions Score: 108 **Expect:** 4.7e-009
Matches (Bold Red): 42/210 fragment ions using 72 most intense peaks

#	b	b^{++}	b*	b^{*++}	b^0	b^{0++}	Seq.	y	y^{++}	y*	y^{*++}	y^0	y^{0++}	#
**1**	100.0757	50.5415					**V**							**20**
**2**	214.1186	107.5629	197.0921	99.0497			**N**	2094.9437	**1047.9755**	2077.9171	1039.4622	2076.9331	1038.9702	**19**
**3**	328.1615	164.5844	311.1350	156.0711			**N**	**1980.9008**	**990.9540**	1963.8742	982.4407	1962.8902	981.9487	**18**

**4**	415.1936	208.1004	398.1670	199.5871	**397.1830**	199.0951	**S**	**1866.8578**	933.9326	1849.8313	925.4193	1848.8473	924.9273	**17**
**5**	516.2413	258.6243	499.2147	250.1110	498.2307	249.6190	**T**	**1779.8258**	890.4165	1762.7993	881.9033	1761.8153	881.4113	**16**
**6**	647.2817	324.1445	**630.2552**	315.6312	**629.2712**	315.1392	**M**	**1678.7781**	839.8927	1661.7516	831.3794	1660.7676	830.8874	**15**
**7**	760.3658	380.6865	**743.3393**	372.1733	742.3552	371.6813	**L**	**1547.7377**	774.3725	**1530.7111**	765.8592	1529.7271	765.3672	**14**
**8**	817.3873	409.1973	800.3607	400.6840	799.3767	400.1920	**G**	**1434.6536**	717.8304	1417.6270	709.3172	1416.6430	708.8251	**13**
**9**	888.4244	444.7158	**871.3978**	436.2026	**870.4138**	435.7105	**A**	**1377.6321**	689.3197	1360.6056	680.8064	1359.6216	680.3144	**12**
**10**	**975.4564**	488.2318	**958.4299**	479.7186	957.4458	479.2266	**S**	**1306.5950**	653.8011	1289.5685	645.2879	1288.5844	644.7959	**11**
**11**	1032.4779	516.7426	1015.4513	508.2293	1014.4673	507.7373	**G**	**1219.5630**	610.2851	1202.5364	601.7719	1201.5524	601.2798	**10**
**12**	**1147.5048**	574.2560	1130.4783	565.7428	1129.4942	565.2508	**D**	**1162.5415**	581.7744	1145.5150	573.2611	1144.5310	572.7691	**9**
**13**	1310.5681	655.7877	1293.5416	647.2744	1292.5576	646.7824	**Y**	**1047.5146**	524.2609	1030.4880	515.7477	1029.5040	515.2556	**8**
**14**	**1381.6053**	691.3063	**1364.5787**	682.7930	1363.5947	682.3010	**A**	**884.4512**	442.7293	867.4247	434.2160	866.4407	433.7240	**7**
**15**	**1496.6322**	748.8197	1479.6057	740.3065	1478.6216	739.8145	**D**	**813.4141**	407.2107	796.3876	398.6974	795.4036	398.2054	**6**
**16**	1643.7006	822.3539	**1626.6741**	**813.8407**	**1625.6900**	**813.3487**	**F**	**698.3872**	349.6972	681.3606	341.1840			**5**
**17**	**1771.7592**	886.3832	1754.7326	877.8700	1753.7486	877.3780	**Q**	**551.3188**	276.1630	**534.2922**	267.6498			**4**
**18**	**1934.8225**	**967.9149**	**1917.7960**	959.4016	**1916.8120**	**958.9096**	**Y**	**423.2602**	212.1337	406.2336	203.6205			**3**
**19**	2047.9066	1024.4569	2030.8800	1015.9437	2029.8960	1015.4516	**L**	260.1969	130.6021	243.1703	122.0888			**2**
**20**							**K**	147.1128	74.0600	130.0863	65.5468			**1**

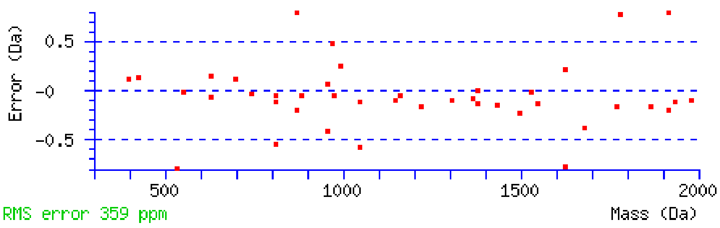

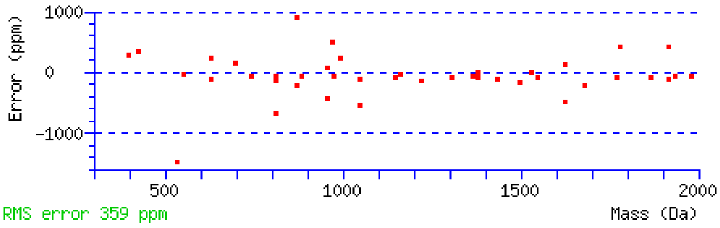

All matches to this query

Score	Mr(calc):	Delta	Sequence
107.9	2193.0048	0.0047	VNNSTMLGASGDYADFQYLK
1.2	2190.9857	2.0238	ETMSPAGGLVSISSDLRRR
1.2	2190.9857	2.0238	ETMSPAGGLVSISSDLRRR
1.2	2190.9857	2.0238	ETMSPAGGLVSISSDLRRR
0.5	2193.0071	0.0024	ATVEQEMELRHKNEMLR

Spectrum No: 125; Query: 74340; Rank: 1

Peptide View

MS/MS Fragmentation of **FGIQAQMVTTDFQK**
Found in **IPI00028004**, Tax_Id=9606 Gene_Symbol=PSMB3 Proteasome subunit beta type-3

Match to Query 74340: 1612.796388 from(807.405470,2+)
Title: 090702LimSK_Exosome2_06.7310.7310.2.dta
Data file C:\Temp\Mascoti\LSK_2D\090702LimSK_Exosome2_01_2D.mgf

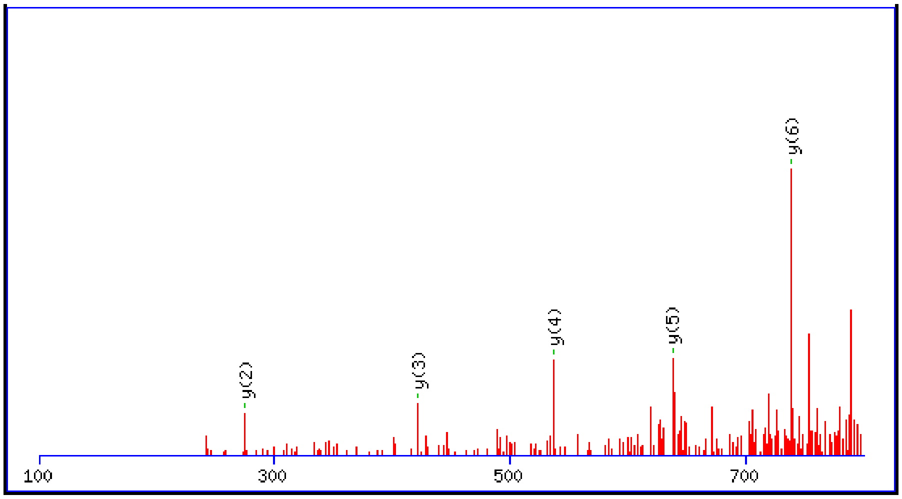

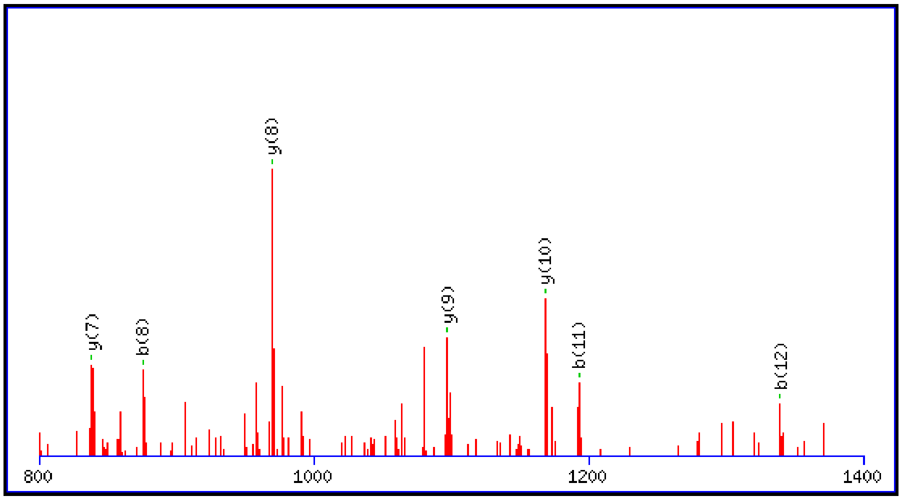

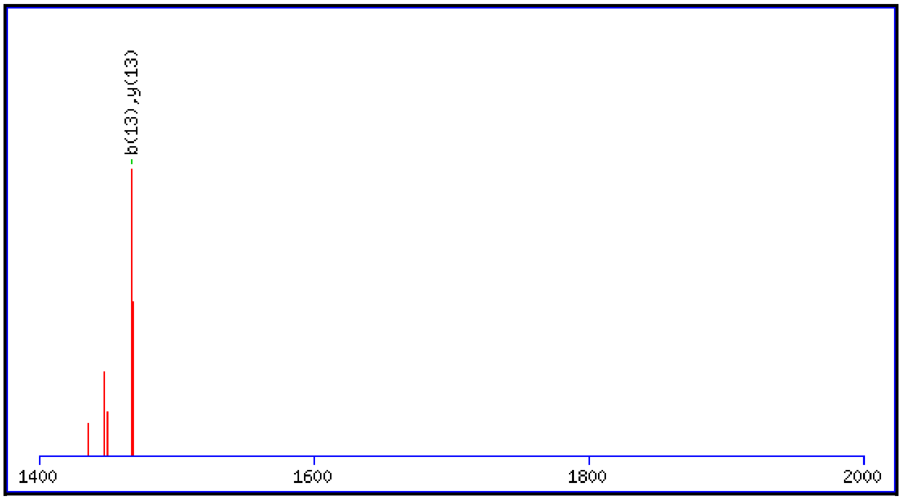

Monoisotopic mass of neutral peptide Mr(calc): 1612.7919
Fixed modifications: Carbamidomethyl (C)
Ions Score: 86 **Expect:** 4.7e-007
Matches (Bold Red): 14/128 fragment ions using 17 most intense peaks

#	b	b++	b*	b*++	b0	b0++	Seq.	y	y++	y*	y*++	y0	y0++	#
1	148.0757	74.5415					F							14
2	205.0972	103.0522					G	**1466.7308**	733.8690	1449.7042	725.3558	1448.7202	724.8638	13
3	318.1812	159.5942					I	1409.7093	705.3583	1392.6828	696.8450	1391.6988	696.3530	12
4	446.2398	223.6235	429.2132	215.1103			Q	1296.6253	648.8163	1279.5987	640.3030	1278.6147	639.8110	11
5	517.2769	259.1421	500.2504	250.6288			A	**1168.5667**	584.7870	1151.5401	576.2737	1150.5561	575.7817	10
6	645.3355	323.1714	628.3089	314.6581			Q	**1097.5296**	549.2684	1080.5030	540.7552	1079.5190	540.2631	9

**7**	776.3760	388.6916	759.3494	380.1784			**M**	**969.4710**	485.2391	952.4444	476.7259	951.4604	476.2339	**8**
**8**	**875.4444**	438.2258	858.4178	429.7126			**V**	**838.4305**	419.7189	821.4040	411.2056	820.4199	410.7136	**7**
**9**	976.4921	488.7497	959.4655	480.2364	958.4815	479.7444	**T**	**739.3621**	370.1847	722.3355	361.6714	721.3515	361.1794	**6**
**10**	1077.5397	539.2735	1060.5132	530.7602	1059.5292	530.2682	**T**	**638.3144**	319.6608	621.2879	311.1476	620.3039	310.6556	**5**
**11**	**1192.5667**	596.7870	1175.5401	588.2737	1174.5561	587.7817	**D**	**537.2667**	269.1370	520.2402	260.6237	519.2562	260.1317	**4**
**12**	**1339.6351**	670.3212	1322.6086	661.8079	1321.6245	661.3159	**F**	**422.2398**	211.6235	405.2132	203.1103			**3**
**13**	**1467.6937**	734.3505	1450.6671	725.8372	1449.6831	725.3452	**Q**	**275.1714**	138.0893	258.1448	129.5761			**2**
**14**							**K**	147.1128	74.0600	130.0863	65.5468			**1**

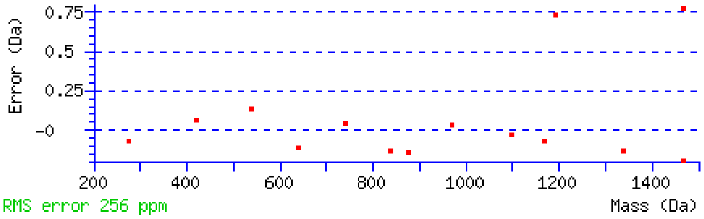

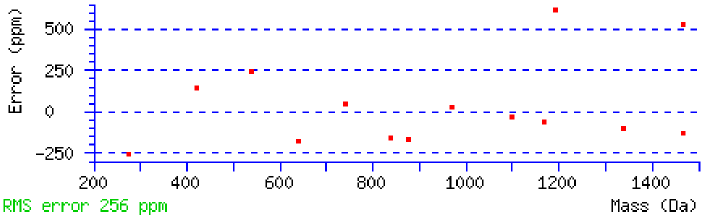

All matches to this query

Score	Mr(calc):	Delta	Sequence
86.2	1612.7919	0.0044	FGIQAQMVTTDFQK
8.6	1612.7837	0.0127	TRGLPMPAITWYK
5.0	1612.8113	-0.0149	IESLNEEIAFLKK
4.8	1611.7861	1.0103	NIQCVSCNKSFKK
1.9	1612.7840	0.0123	DMELSKDIMIQFK
0.3	1612.7879	0.0085	MQLSGEGAKTREYK
0.1	1612.7902	0.0062	IYYTGKYQSLGIK

Spectrum No: 126; Query: 78434; Rank: 1

Peptide View

MS/MS Fragmentation of **FGPYYTEPVIAGLDPK**
Found in **IPI00028004**, Tax_Id=9606 Gene_Symbol=PSMB3 Proteasome subunit beta type-3

Match to Query 78434: 1765.896568 from(883.955560,2+)
Title: 090702LimSK_Exosome2_03.8241.8241.2.dta
Data file C:\Temp\Mascoti\LSK_2D\090702LimSK_Exosome2_01_2D.mgf

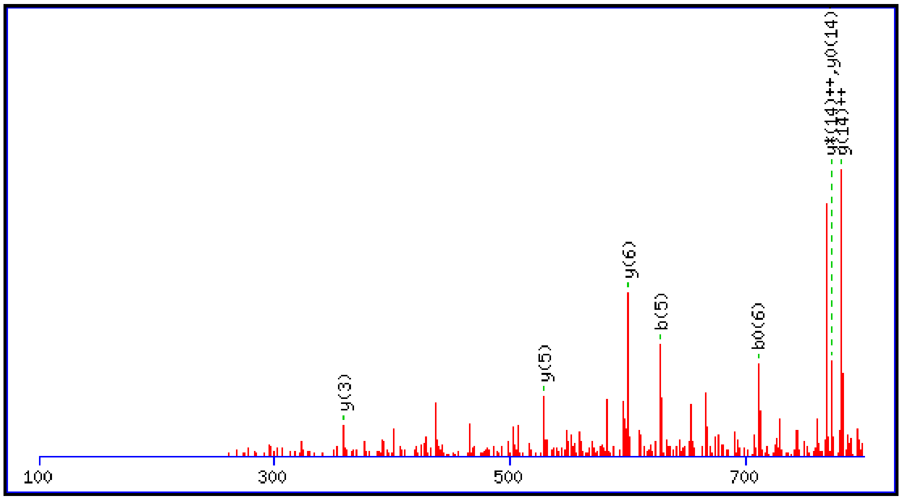

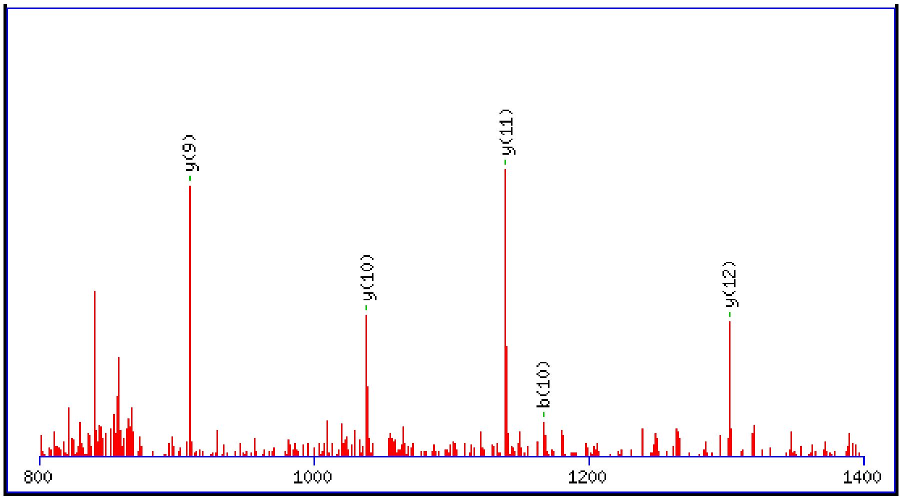

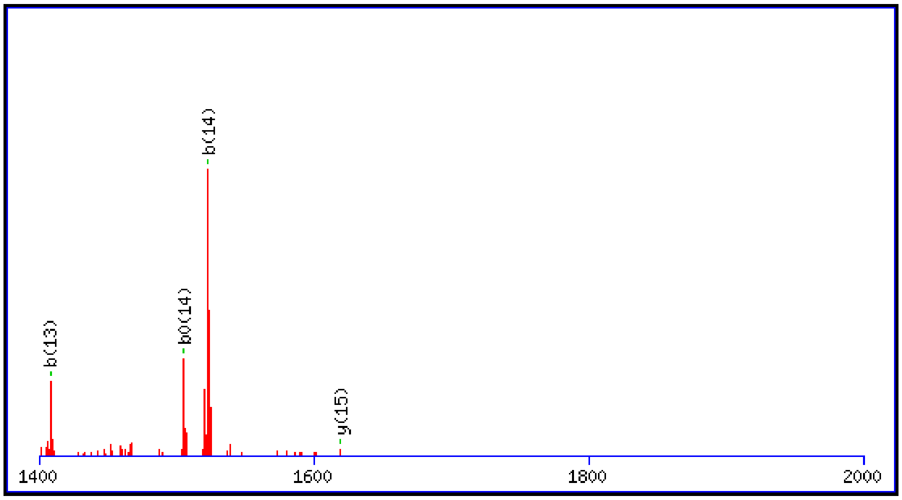

Monoisotopic mass of neutral peptide Mr(calc): 1765.8927
Fixed modifications: Carbamidomethyl (C)
Ions Score: 68 **Expect:** 3.3e-005
Matches (Bold Red): 17/136 fragment ions using 22 most intense peaks

#	b	b^{++}	b^{0}	b^{0++}	Seq.	y	y^{++}	y*	y^{*++}	y^{0}	y^{0++}	#
1	148.0757	74.5415			F							16
2	205.0972	103.0522			G	**1619.8316**	810.4194	1602.8050	801.9061	1601.8210	801.4141	15
3	302.1499	151.5786			P	1562.8101	**781.9087**	1545.7835	**773.3954**	1544.7995	**772.9034**	14
4	465.2132	233.1103			Y	1465.7573	733.3823	1448.7308	724.8690	1447.7468	724.3770	13
5	**628.2766**	314.6419			Y	**1302.6940**	651.8506	1285.6674	643.3374	1284.6834	642.8454	12
6	729.3243	365.1658	**711.3137**	356.1605	T	**1139.6307**	570.3190	1122.6041	561.8057	1121.6201	561.3137	11
7	858.3668	429.6871	840.3563	420.6818	E	**1038.5830**	519.7951	1021.5564	511.2819	1020.5724	510.7898	10
8	955.4196	478.2134	937.4090	469.2082	P	**909.5404**	455.2738	892.5138	446.7606	891.5298	446.2686	9
9	1054.4880	527.7477	1036.4775	518.7424	V	812.4876	406.7475	795.4611	398.2342	794.4771	397.7422	8
10	**1167.5721**	584.2897	1149.5615	575.2844	I	713.4192	357.2132	696.3927	348.7000	695.4087	348.2080	7
11	1238.6092	619.8082	1220.5986	610.8030	A	**600.3352**	300.6712	583.3086	292.1579	582.3246	291.6659	6
12	1295.6307	648.3190	1277.6201	639.3137	G	**529.2980**	265.1527	512.2715	256.6394	511.2875	256.1474	5
13	**1408.7147**	704.8610	1390.7042	695.8557	L	472.2766	236.6419	455.2500	228.1287	454.2660	227.6366	4
14	**1523.7417**	762.3745	**1505.7311**	753.3692	D	**359.1925**	180.0999	342.1660	171.5866	341.1819	171.0946	3
15	1620.7944	810.9009	1602.7839	801.8956	P	244.1656	122.5864	227.1390	114.0731			2
16					K	147.1128	74.0600	130.0863	65.5468			1

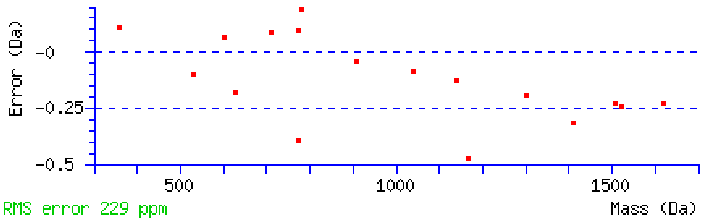

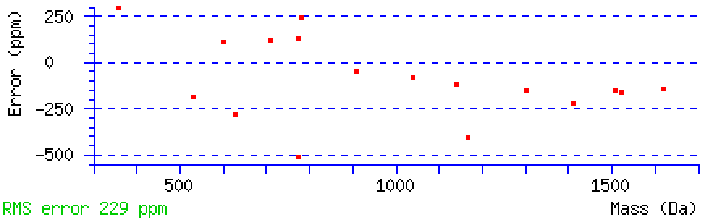

All matches to this query

Score	Mr(calc):	Delta	Sequence
67.6	1765.8927	0.0039	FGPYYTEPVIAGLDPK
16.8	1763.9070	1.9895	SGGGEQLDILSVGILVK
15.1	1764.9063	0.9903	GTDVFPLLGSVLKDPK
12.2	1764.8771	1.0195	RSQVEQQQLITVEK
8.4	1764.9063	0.9903	GTDVFPLLGSVLKDPK
7.6	1765.8862	0.0103	IIDKTQLDEENLKK
5.8	1764.8771	1.0195	DLLEVGTASRDIQLR
5.8	1764.8981	0.9985	QNTGQPLCHLYPLPK
4.4	1763.8794	2.0172	MFPHVQTDVLVRVK
3.3	1764.8811	1.0155	IEDPYSPRIQNLLK

Spectrum No: 127; Query: 35269; Rank: 1

Peptide View

MS/MS Fragmentation of **IFPMGDR**
Found in **IPI00028004**, Tax_Id=9606 Gene_Symbol=PSMB3 Proteasome subunit beta type-3

Match to Query 35269: 834.406448 from(418.210500,2+)
Title: 090702LimSK_Exosome2_06.3522.3522.2.dta
Data file C:\Temp\Mascoti\LSK_2D\090702LimSK_Exosome2_01_2D.mgf

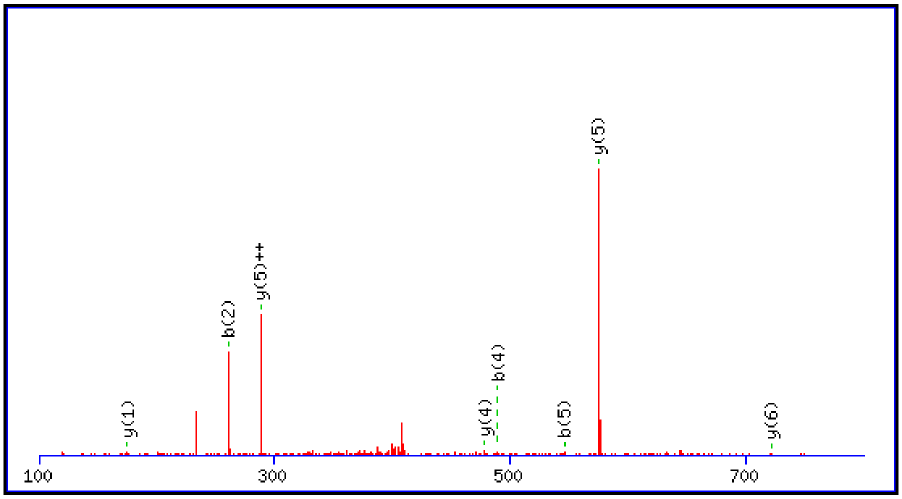

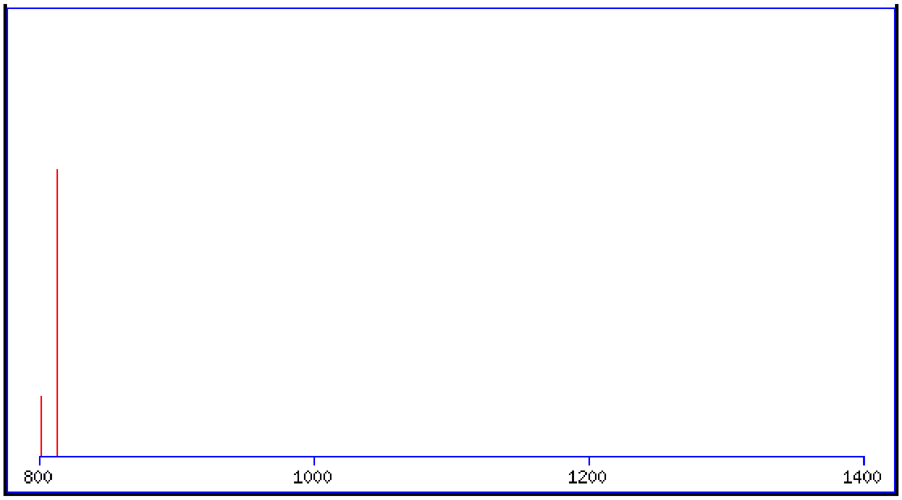

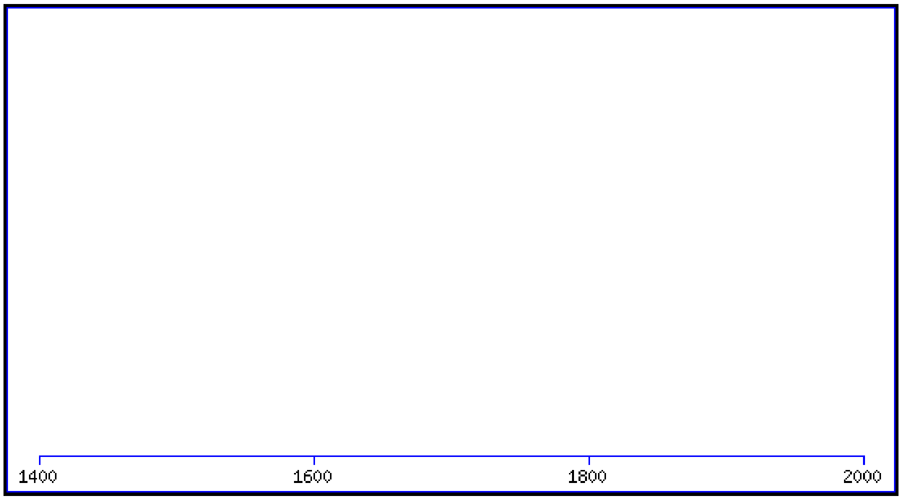

Monoisotopic mass of neutral peptide Mr(calc): 834.4058
Fixed modifications: Carbamidomethyl (C)
Ions Score: 24 **Expect:** 0.45
Matches (Bold Red): 8/48 fragment ions using 23 most intense peaks

#	b	b++	b0	b0++	Seq.	y	y++	y*	y*++	y0	y0++	#
1	114.0913	57.5493			I							7
2	**261.1598**	131.0835			F	**722.3290**	361.6681	705.3025	353.1549	704.3185	352.6629	6
3	358.2125	179.6099			P	**575.2606**	**288.1339**	558.2341	279.6207	557.2500	279.1287	5
4	**489.2530**	245.1301			M	**478.2078**	239.6076	461.1813	231.0943	460.1973	230.6023	4
5	**546.2745**	273.6409			G	347.1674	174.0873	330.1408	165.5740	329.1568	165.0820	3
6	661.3014	331.1543	643.2908	322.1491	D	290.1459	145.5766	273.1193	137.0633	272.1353	136.5713	2
7					R	**175.1190**	88.0631	158.0924	79.5498			1

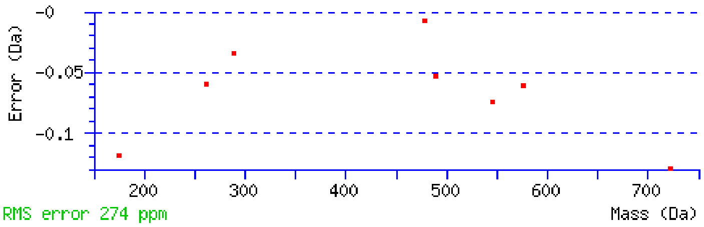

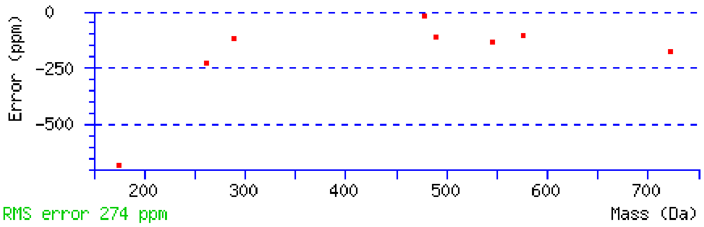

All matches to this query

Score	Mr(calc):	Delta	Sequence
24.5	834.4058	0.0006	IFPMGDR
20.1	832.4039	2.0025	STVGGQER

17.5	832.4039	2.0026	SGNTGELR
15.8	834.4123	-0.0059	LFPDESK
12.7	833.4088	0.9976	FIFSLK
12.7	833.4088	0.9976	LFFSLK
9.4	833.3953	1.0112	QMSSPLAT
8.7	832.3926	2.0138	EEADTLR
8.7	833.4048	1.0016	VPNPSLK
8.5	832.4039	2.0025	EGTGVTNR

Spectrum No: 128; Query: 38464; Rank: 1

Peptide View

MS/MS Fragmentation of **LNLYELK**
Found in **IPI00028004**, Tax_Id=9606 Gene_Symbol=PSMB3 Proteasome subunit beta type-3

Match to Query 38464: 891.508088 from(446.761320,2+)
Title: 090702LimSK_Exosome2_06.5954.5954.2.dta
Data file C:\Temp\Mascoti\LSK_2D\090702LimSK_Exosome2_01_2D.mgf

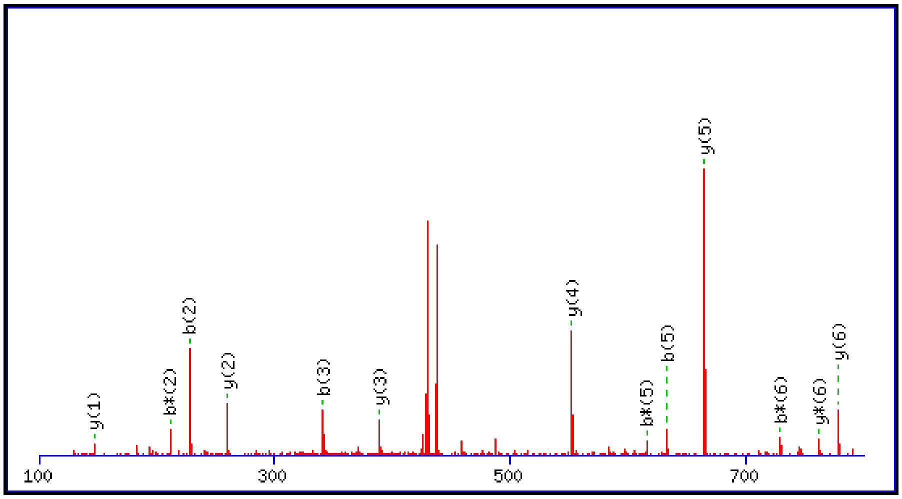

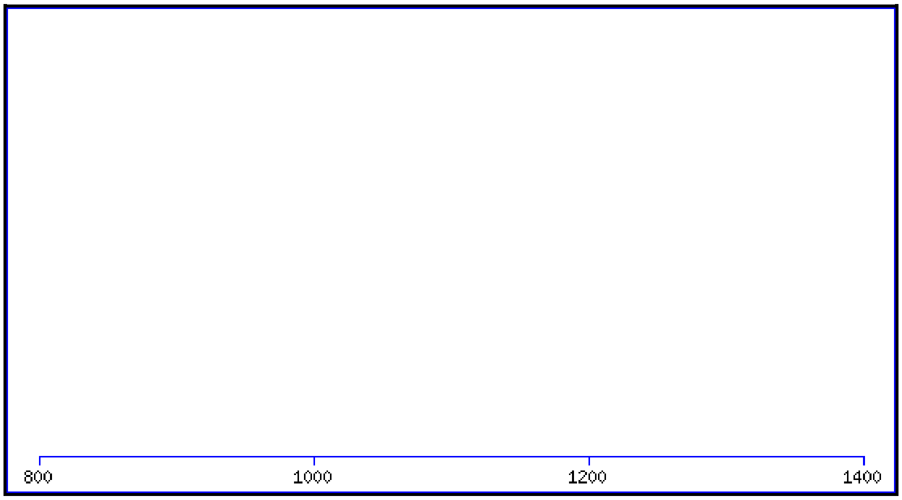

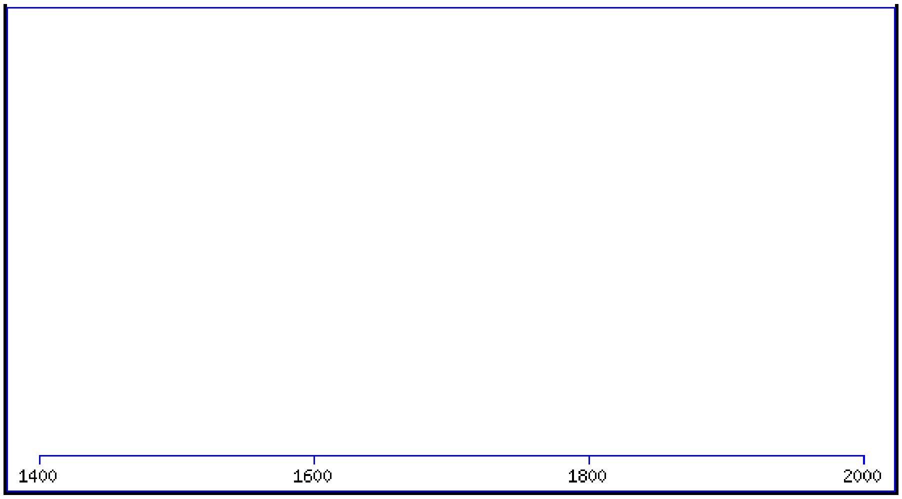

Monoisotopic mass of neutral peptide Mr(calc): 891.5065
Fixed modifications: Carbamidomethyl (C)
Ions Score: 50 **Expect:** 0.00077
Matches (Bold Red): 13/58 fragment ions using 22 most intense peaks

#	b	b++	b*	b*++	b0	b0++	Seq.	y	y++	y*	y*++	y0	y0++	#
1	114.0913	57.5493					L							7
2	**228.1343**	114.5708	**211.1077**	106.0575			N	**779.4298**	390.2185	**762.4032**	381.7053	761.4192	381.2132	6
3	**341.2183**	171.1128	324.1918	162.5995			L	**665.3869**	333.1971	648.3603	324.6838	647.3763	324.1918	5
4	504.2817	252.6445	487.2551	244.1312			Y	**552.3028**	276.6550	535.2762	268.1418	534.2922	267.6498	4
5	**633.3243**	317.1658	**616.2977**	308.6525	615.3137	308.1605	E	**389.2395**	195.1234	372.2129	186.6101	371.2289	186.1181	3
6	746.4083	373.7078	**729.3818**	365.1945	728.3978	364.7025	L	**260.1969**	130.6021	243.1703	122.0888			2
7							K	**147.1128**	74.0600	130.0863	65.5468			1

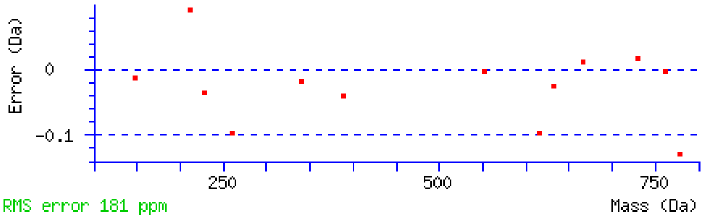

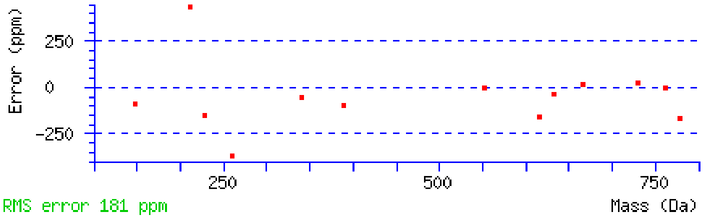

All matches to this query

Score	Mr(calc):	Delta	Sequence
50.0	891.5065	0.0016	LNLYELK
34.5	891.5065	0.0015	VQLYELK
21.4	891.5065	0.0016	NLYLEIK
20.1	891.5065	0.0015	LVAGEYLK
20.0	891.5065	0.0016	YNLLELK
17.2	891.5065	0.0015	IIGDAYLK
14.8	891.5065	0.0016	INYLIEK
12.6	891.5065	0.0015	LDFEKIK
12.3	891.5065	0.0015	IIDLGYAK
10.8	890.4990	1.0091	LLLLPSR

Spectrum No: 129; Query: 82476; Rank: 1

Peptide View

MS/MS Fragmentation of **LYIGLAGLATDVQTVAQR**
Found in **IPI00028004**, Tax_Id=9606 Gene_Symbol=PSMB3 Proteasome subunit beta type-3

Match to Query 82476: 1888.047008 from(945.030780,2+)
Title: 090702LimSK_Exosome2_01.9874.9874.2.dta
Data file C:\Temp\Mascoti\LSK_2D\090702LimSK_Exosome2_01_2D.mgf

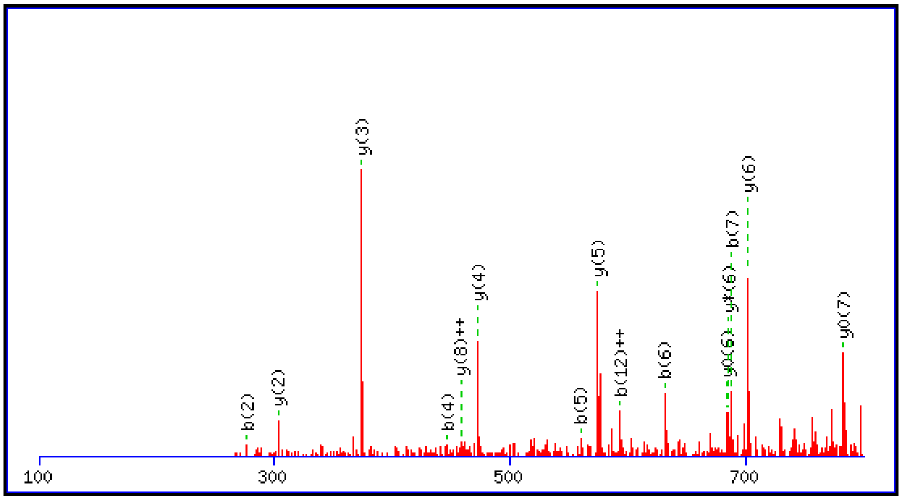

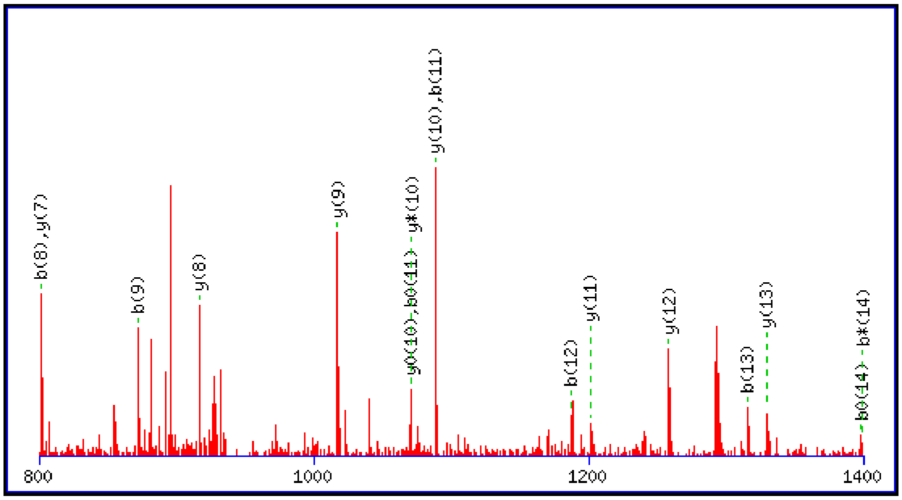

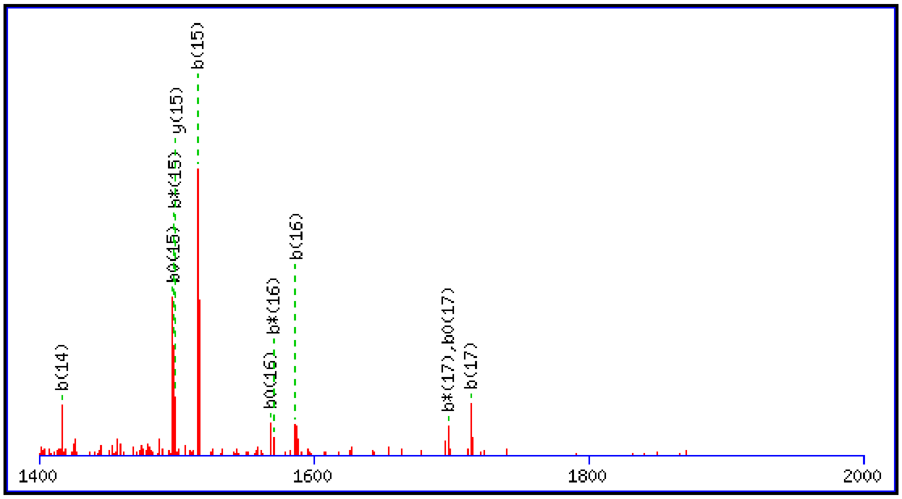

Monoisotopic mass of neutral peptide Mr(calc): 1888.0418
Fixed modifications: Carbamidomethyl (C)
Ions Score: 81 **Expect:** 4.3e-007
Matches (Bold Red): 43/154 fragment ions using 86 most intense peaks

#	b	b^{++}	b*	b^{*++}	b^0	b^{0++}	Seq.	y	y^{++}	y*	y^{*++}	y^0	y^{0++}	#
1	114.0913	57.5493					L							18

**2**	**277.1547**	139.0810					**Y**	1775.9650	888.4862	1758.9385	879.9729	1757.9545	879.4809	**17**
**3**	390.2387	195.6230					**I**	1612.9017	806.9545	1595.8752	798.4412	1594.8911	797.9492	**16**
**4**	**447.2602**	224.1337					**G**	**1499.8176**	750.4125	1482.7911	741.8992	1481.8071	741.4072	**15**
**5**	**560.3443**	280.6758					**L**	1442.7962	721.9017	1425.7696	713.3884	1424.7856	712.8964	**14**
**6**	**631.3814**	316.1943					**A**	**1329.7121**	665.3597	1312.6856	656.8464	1311.7015	656.3544	**13**
**7**	**688.4028**	344.7051					**G**	**1258.6750**	629.8411	1241.6484	621.3279	1240.6644	620.8359	**12**
**8**	**801.4869**	401.2471					**L**	**1201.6535**	601.3304	1184.6270	592.8171	1183.6430	592.3251	**11**
**9**	**872.5240**	436.7656					**A**	**1088.5695**	544.7884	**1071.5429**	536.2751	**1070.5589**	535.7831	**10**
**10**	973.5717	487.2895			955.5611	478.2842	**T**	**1017.5324**	509.2698	1000.5058	500.7565	999.5218	500.2645	**9**
**11**	**1088.5986**	544.8030			**1070.5881**	535.7977	**D**	**916.4847**	**458.7460**	899.4581	450.2327	898.4741	449.7407	**8**
**12**	**1187.6671**	**594.3372**			1169.6565	585.3319	**V**	**801.4577**	401.2325	784.4312	392.7192	**783.4472**	392.2272	**7**
**13**	**1315.7256**	658.3665	1298.6991	649.8532	1297.7151	649.3612	**Q**	**702.3893**	351.6983	**685.3628**	343.1850	**684.3787**	342.6930	**6**
**14**	**1416.7733**	708.8903	**1399.7468**	700.3770	**1398.7627**	699.8850	**T**	**574.3307**	287.6690	557.3042	279.1557	556.3202	278.6637	**5**
**15**	**1515.8417**	758.4245	**1498.8152**	749.9112	**1497.8312**	749.4192	**V**	**473.2831**	237.1452	456.2565	228.6319			**4**
**16**	**1586.8788**	793.9431	**1569.8523**	785.4298	**1568.8683**	784.9378	**A**	**374.2146**	187.6110	357.1881	179.0977			**3**
**17**	**1714.9374**	857.9723	**1697.9109**	849.4591	**1696.9269**	848.9671	**Q**	**303.1775**	152.0924	286.1510	143.5791			**2**
**18**							**R**	175.1190	88.0631	158.0924	79.5498			**1**

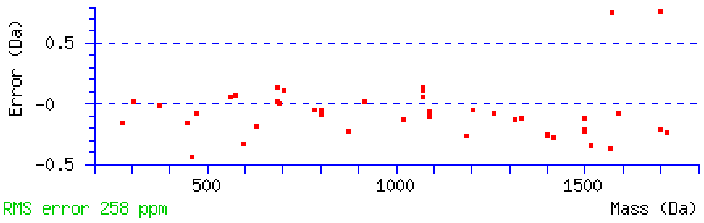

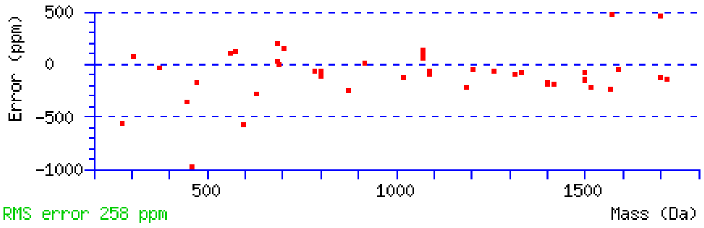

All matches to this query

Score	Mr(calc):	Delta	Sequence
81.3	1888.0418	0.0052	LYIGLAGLATDVQTVAQR
4.9	1886.0560	1.9910	TAPSLVMVLSWKSGVRR
3.2	1887.0578	0.9893	NALEVDLGYLIITAARR
0.8	1888.0431	0.0039	RLWGFSESLLIRGAAGR
0.6	1886.0448	2.0022	VLHPDMAKVPPASPKTAK

Spectrum No: 130; Query: 42285; Rank: 1

Peptide View

MS/MS Fragmentation of **NCVAIAADR**
Found in **IPI00028004**, Tax_Id=9606 Gene_Symbol=PSMB3 Proteasome subunit beta type-3

Match to Query 42285: 988.485128 from(495.249840,2+)
Title: 090702LimSK_Exosome2_06.1528.1528.2.dta
Data file C:\Temp\Mascoti\LSK_2D\090702LimSK_Exosome2_01_2D.mgf

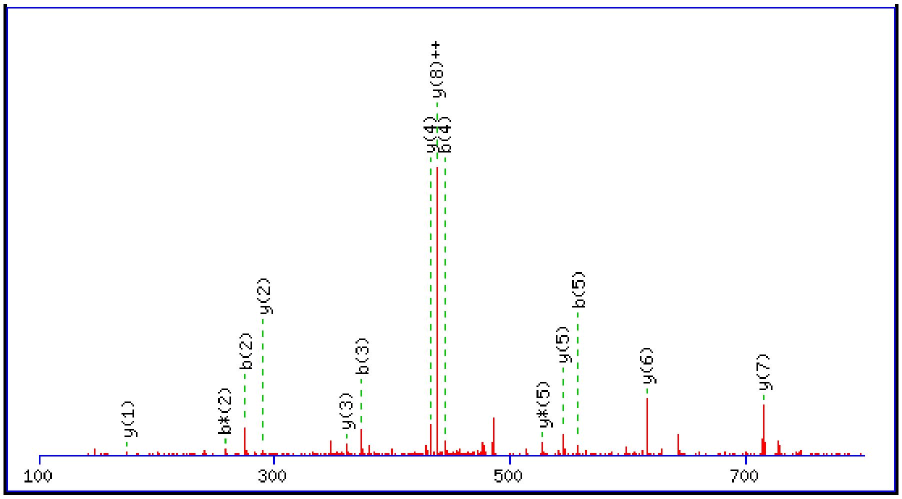

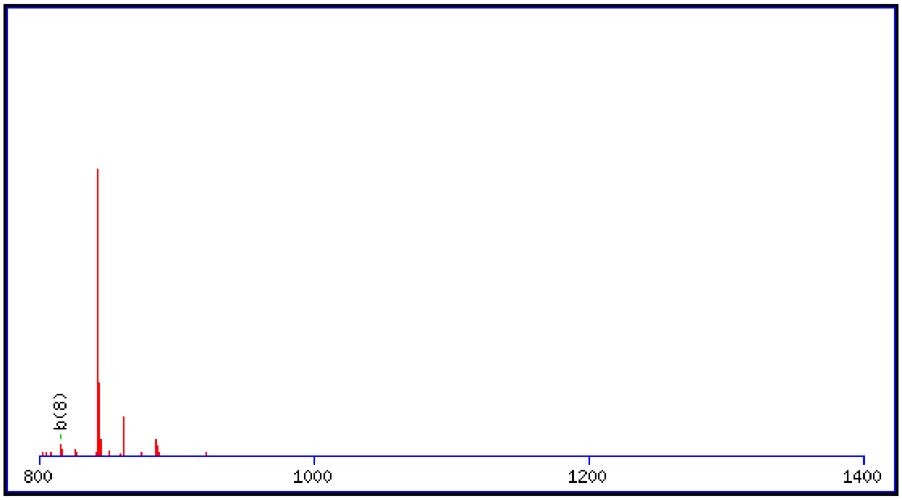

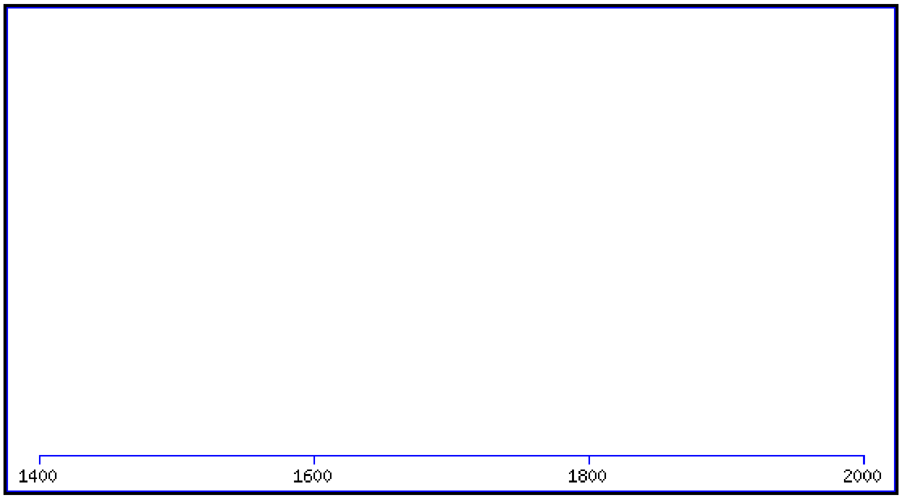

Monoisotopic mass of neutral peptide Mr(calc): 988.4760
Fixed modifications: Carbamidomethyl (C)
Ions Score: 34 **Expect:** 0.056
Matches (Bold Red): 15/80 fragment ions using 35 most intense peaks

#	b	b^{++}	b*	b^{*++}	b^0	b^{0++}	Seq.	y	y^{++}	y*	y^{*++}	y^0	y^{0++}	#
1	115.0502	58.0287	98.0237	49.5155			N							9
2	**275.0809**	138.0441	**258.0543**	129.5308			C	875.4404	**438.2238**	858.4138	429.7105	857.4298	429.2185	8
3	**374.1493**	187.5783	357.1227	179.0650			V	**715.4097**	358.2085	698.3832	349.6952	697.3991	349.2032	7
4	**445.1864**	223.0968	428.1598	214.5836			A	**616.3413**	308.6743	599.3148	300.1610	598.3307	299.6690	6
5	**558.2704**	279.6389	541.2439	271.1256			I	**545.3042**	273.1557	**528.2776**	264.6425	527.2936	264.1504	5
6	629.3076	315.1574	612.2810	306.6441			A	**432.2201**	216.6137	415.1936	208.1004	414.2096	207.6084	4

7	700.3447	350.6760	683.3181	342.1627			A	361.1830	181.0951	344.1565	172.5819	343.1724	172.0899	3
8	815.3716	408.1894	798.3451	399.6762	797.3610	399.1842	D	290.1459	145.5766	273.1193	137.0633	272.1353	136.5713	2
9							R	175.1190	88.0631	158.0924	79.5498			1

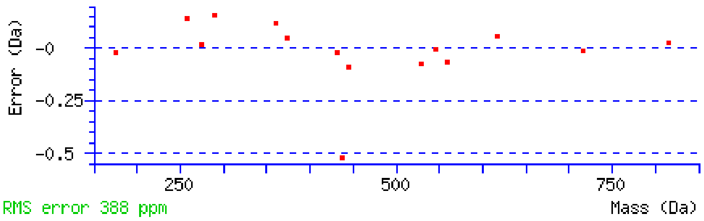

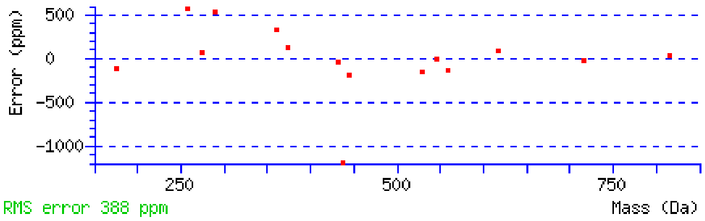

All matches to this query

Score	Mr(calc):	Delta	Sequence
33.5	988.4760	0.0091	NCVAIAADR
30.2	988.4825	0.0026	NEKDEVQK
19.4	988.4767	0.0085	IHDWYQK
15.6	988.4825	0.0026	KEDDLNQK
15.3	988.4938	-0.0086	DTGVNEKAR
14.6	988.4825	0.0026	DIREEAEK
14.0	988.4825	0.0026	QDLNDEKK
13.7	988.4800	0.0051	CFHLVGEK
13.0	988.4825	0.0026	QVAQSEAEK
12.8	988.4899	-0.0048	MLEIDPQK

Spectrum No: 131; Query: 51677; Rank: 1

Peptide View

MS/MS Fragmentation of **NCVAIAADRR**
Found in **IPI00028004**, Tax_Id=9606 Gene_Symbol=PSMB3 Proteasome subunit beta type-3

Match to Query 51677: 1144.579268 from(573.296910,2+)
Title: 090702LimSK_Exosome2_06.3084.3084.2.dta
Data file C:\Temp\Mascoti\LSK_2D\090702LimSK_Exosome2_01_2D.mgf

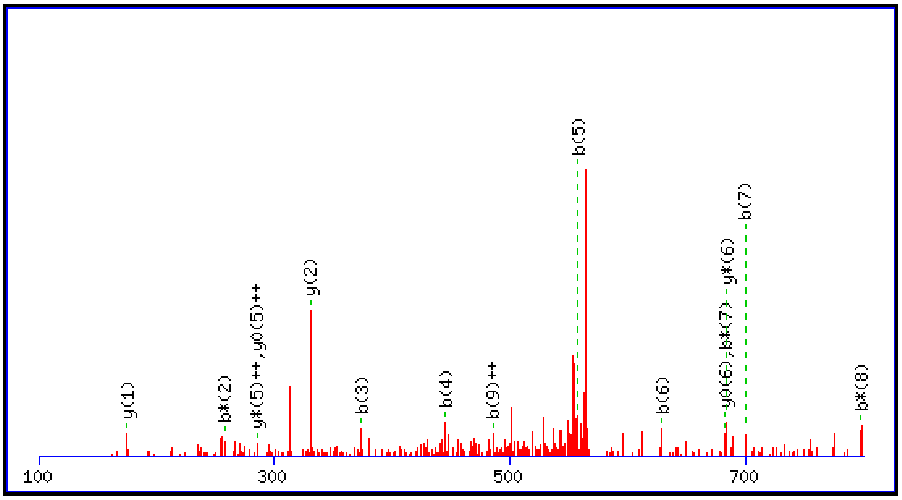

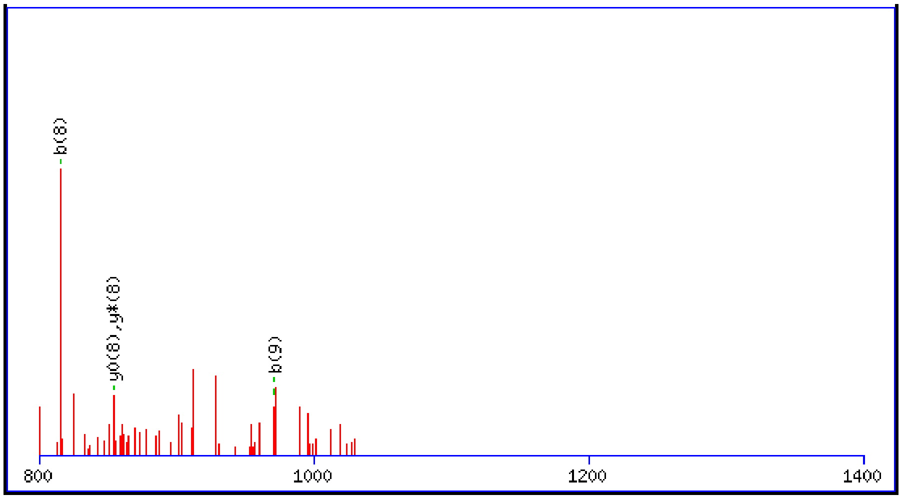

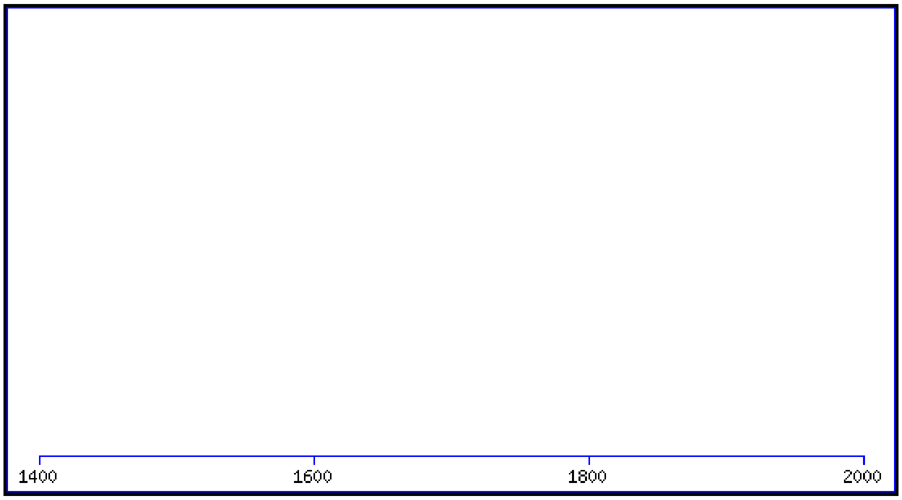

Monoisotopic mass of neutral peptide Mr(calc): 1144.5771
Fixed modifications: Carbamidomethyl (C)
Ions Score: 28 **Expect:** 0.23
Matches (Bold Red): 19/90 fragment ions using 40 most intense peaks

#	b	b^{++}	b*	b^{*++}	b^0	b^{0++}	Seq.	y	y^{++}	y*	y^{*++}	y^0	y^{0++}	#
1	115.0502	58.0287	98.0237	49.5155			N							10
2	275.0809	138.0441	**258.0543**	129.5308			C	1031.5415	516.2744	1014.5149	507.7611	1013.5309	507.2691	9
3	**374.1493**	187.5783	357.1227	179.0650			V	871.5108	436.2591	**854.4843**	427.7458	**853.5003**	427.2538	8
4	**445.1864**	223.0968	428.1598	214.5836			A	772.4424	386.7248	755.4159	378.2116	754.4318	377.7196	7
5	**558.2704**	279.6389	541.2439	271.1256			I	701.4053	351.2063	**684.3787**	342.6930	**683.3947**	342.2010	6
6	**629.3076**	315.1574	612.2810	306.6441			A	588.3212	294.6643	571.2947	**286.1510**	570.3107	**285.6590**	5
7	**700.3447**	350.6760	**683.3181**	342.1627			A	517.2841	259.1457	500.2576	250.6324	499.2736	250.1404	4
8	**815.3716**	408.1894	**798.3451**	399.6762	797.3610	399.1842	D	446.2470	223.6271	429.2205	215.1139	428.2364	214.6219	3
9	**971.4727**	**486.2400**	954.4462	477.7267	953.4622	477.2347	R	**331.2201**	166.1137	314.1935	157.6004			2
10							R	**175.1190**	88.0631	158.0924	79.5498			1

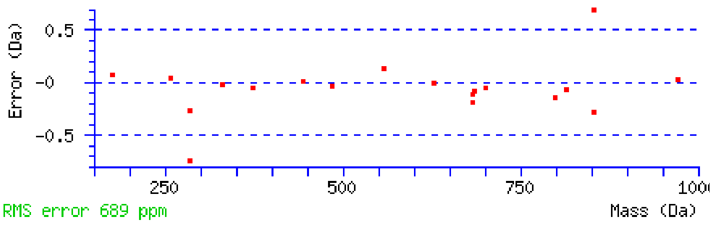

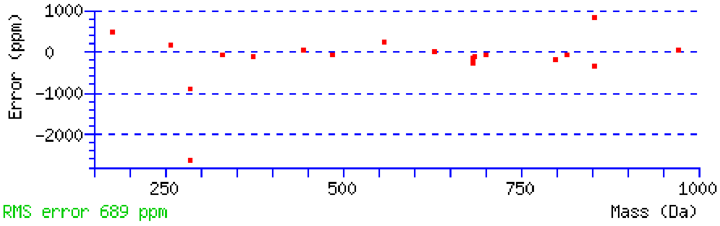

All matches to this query

Score	Mr(calc):	Delta	Sequence
27.7	1144.5771	0.0022	NCVAIAADRR
16.6	1144.5836	-0.0043	ELEEKADRR
13.0	1144.5836	-0.0043	SEVELEQRR
10.8	1144.5836	-0.0043	SSASPKAAEAAR
10.4	1144.5737	0.0055	TIENWGGGRR
9.8	1143.5679	1.0113	CNTPARNRR
9.8	1143.5857	0.9936	NRNDVTGGRR
9.5	1144.5698	0.0095	TLGGGGGGGSGGRR
8.5	1143.5818	0.9974	MEPAAGIQRR
8.3	1143.5801	0.9992	AIKYQSKAR

Spectrum No: 132; Query: 43789; Rank: 1

Peptide View

MS/MS Fragmentation of **ATEGMVVADK**
Found in **IPI00003217**, Tax_Id=9606 Gene_Symbol=PSMB7 Proteasome subunit beta type-7

Match to Query 43789: 1019.497308 from(510.755930,2+)
Title: 090702LimSK_Exosome2_05.1599.1599.2.dta
Data file C:\Temp\Mascoti\LSK_2D\090702LimSK_Exosome2_01_2D.mgf

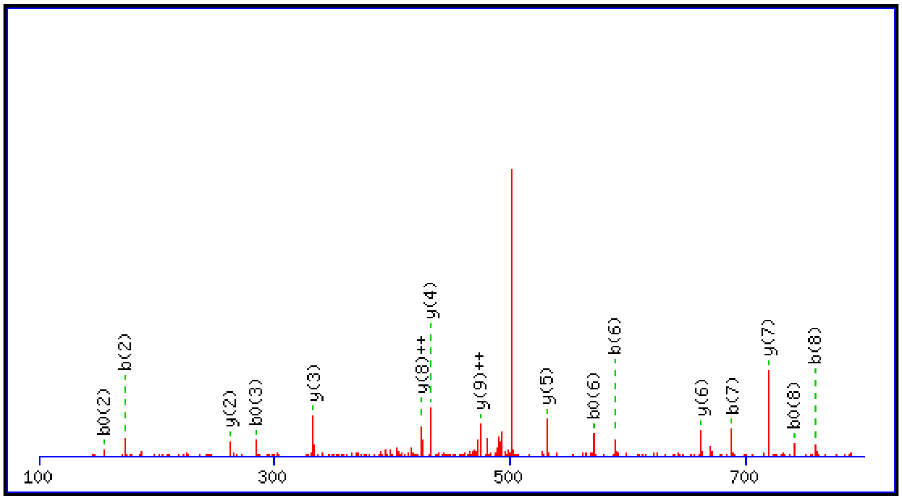

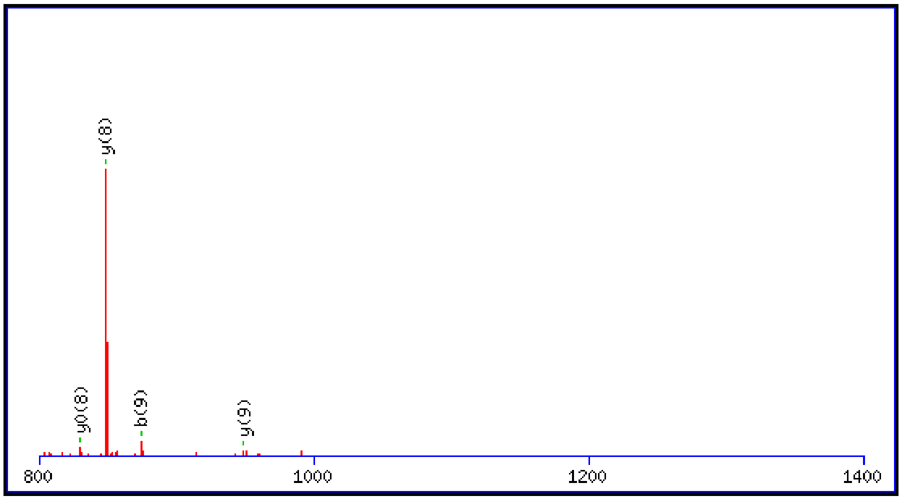

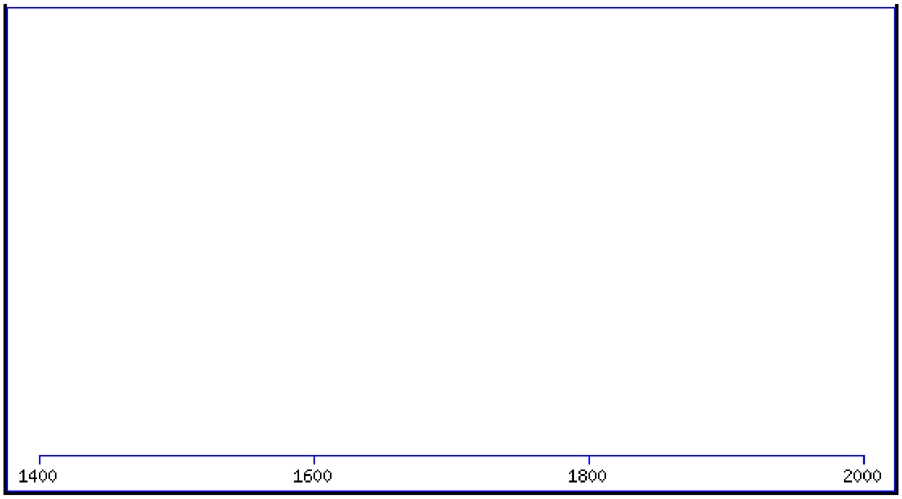

Monoisotopic mass of neutral peptide Mr(calc): 1019.4957
Fixed modifications: Carbamidomethyl (C)
Ions Score: 65 **Expect:** 3.7e-005
Matches (Bold Red): 20/86 fragment ions using 30 most intense peaks

#	b	b^{++}	b^{0}	b^{0++}	Seq.	y	y^{++}	y*	y^{*++}	y^{0}	y^{0++}	#
1	72.0444	36.5258			A							10
2	**173.0921**	87.0497	**155.0815**	78.0444	T	**949.4659**	**475.2366**	932.4394	466.7233	931.4553	466.2313	9
3	302.1347	151.5710	**284.1241**	142.5657	E	**848.4182**	**424.7128**	831.3917	416.1995	**830.4077**	415.7075	8
4	359.1561	180.0817	341.1456	171.0764	G	**719.3756**	360.1915	702.3491	351.6782	701.3651	351.1862	7
5	490.1966	245.6019	472.1860	236.5967	M	**662.3542**	331.6807	645.3276	323.1675	644.3436	322.6754	6
6	**589.2650**	295.1362	**571.2545**	286.1309	V	**531.3137**	266.1605	514.2871	257.6472	513.3031	257.1552	5
7	**688.3334**	344.6704	670.3229	335.6651	V	**432.2453**	216.6263	415.2187	208.1130	414.2347	207.6210	4
8	**759.3706**	380.1889	**741.3600**	371.1836	A	**333.1769**	167.0921	316.1503	158.5788	315.1663	158.0868	3
9	**874.3975**	437.7024	856.3869	428.6971	D	**262.1397**	131.5735	245.1132	123.0602	244.1292	122.5682	2
10					K	147.1128	74.0600	130.0863	65.5468			1

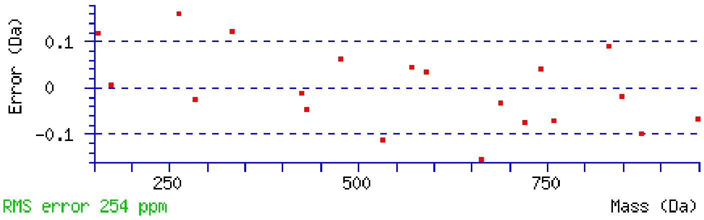

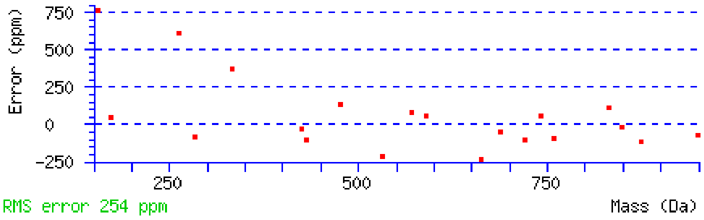

All matches to this query

Score	Mr(calc):	Delta	Sequence
65.4	1019.4957	0.0016	ATEGMVVADK
11.4	1019.4958	0.0015	SVTDSVMGPK
8.3	1018.4931	1.0042	ATNDSVVADK
5.3	1019.4883	0.0090	ATQESTLDR
5.2	1019.4897	0.0076	ATDHHVQGR
2.9	1019.5036	-0.0063	KPDQSFGGGK
2.9	1019.5036	-0.0063	GDFVGLQER
1.9	1018.4931	1.0042	VSNAESSTPK
1.3	1018.4961	1.0012	RTHIVADK
0.9	1018.4865	1.0108	EMKAQEQR

Spectrum No: 133; Query: 69571; Rank: 1

Peptide View

MS/MS Fragmentation of **ATEGMVVADKNCSK**
Found in **IPI00003217**, Tax_Id=9606 Gene_Symbol=PSMB7 Proteasome subunit beta type-7

Match to Query 69571: 1508.697088 from(755.355820,2+)
Title: 090702LimSK_Exosome2_06.2737.2737.2.dta
Data file C:\Temp\Mascoti\LSK_2D\090702LimSK_Exosome2_01_2D.mgf

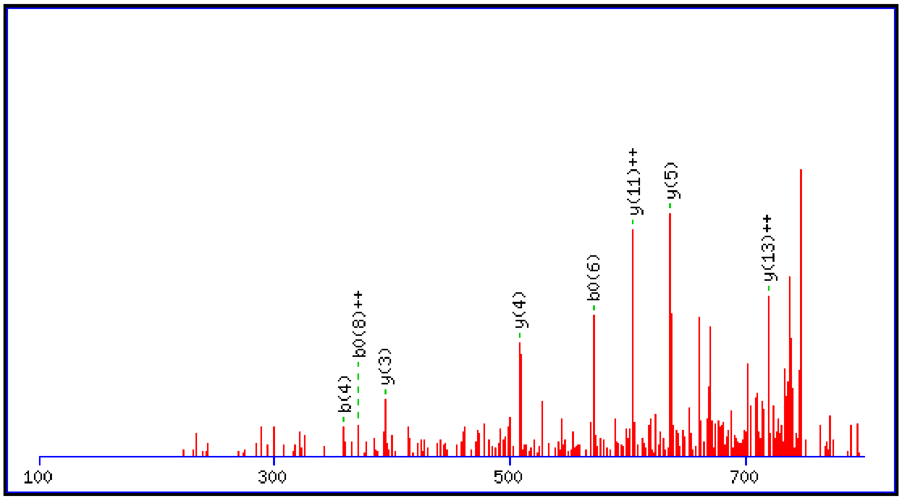

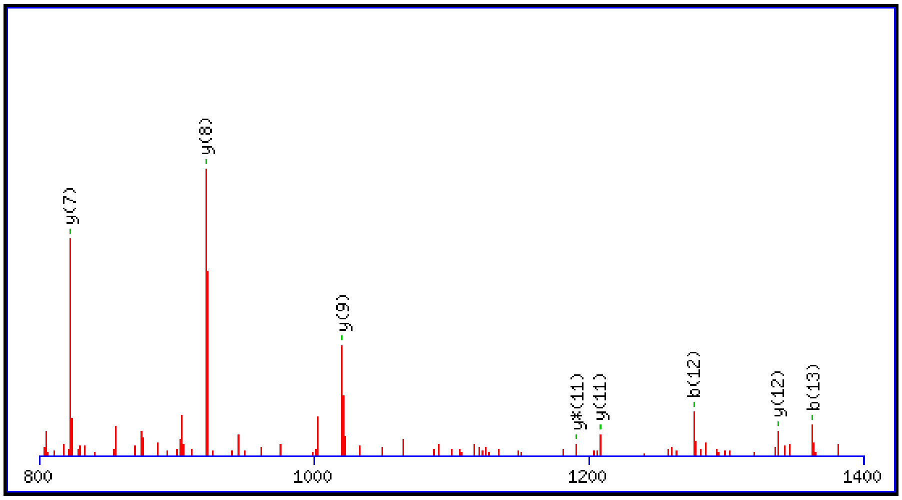

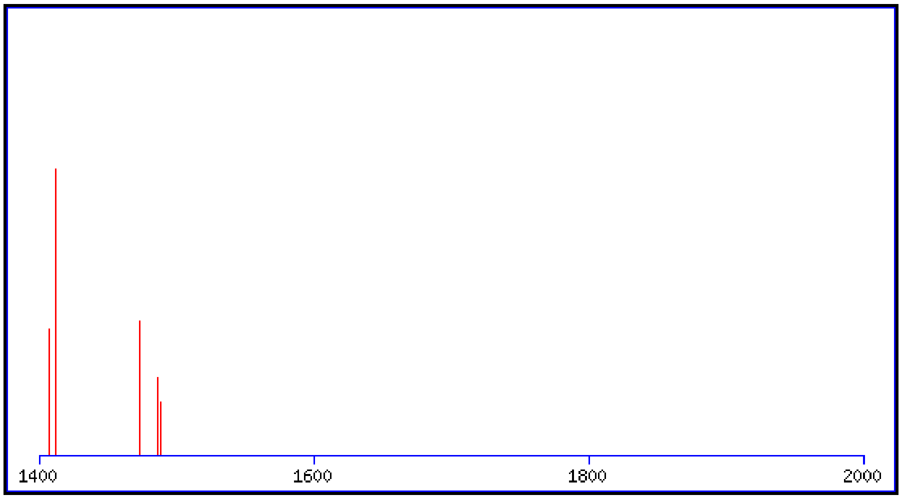

Monoisotopic mass of neutral peptide Mr(calc): 1508.6963
Fixed modifications: Carbamidomethyl (C)
Ions Score: 54 **Expect:** 0.00077

Matches (Bold Red): 16/134 fragment ions using 33 most intense peaks

#	b	b^{++}	b*	b^{*++}	b^{0}	b^{0++}	Seq.	y	y^{++}	y*	y^{*++}	y^{0}	y^{0++}	#
1	72.0444	36.5258					A							14
2	173.0921	87.0497			155.0815	78.0444	T	1438.6665	**719.8369**	1421.6399	711.3236	1420.6559	710.8316	13
3	302.1347	151.5710			284.1241	142.5657	E	**1337.6188**	669.3130	1320.5922	660.7998	1319.6082	660.3078	12
4	**359.1561**	180.0817			341.1456	171.0764	G	**1208.5762**	**604.7917**	**1191.5497**	596.2785	1190.5656	595.7865	11
5	490.1966	245.6019			472.1860	236.5967	M	1151.5547	576.2810	1134.5282	567.7677	1133.5442	567.2757	10
6	589.2650	295.1362			**571.2545**	286.1309	V	**1020.5143**	510.7608	1003.4877	502.2475	1002.5037	501.7555	9
7	688.3334	344.6704			670.3229	335.6651	V	**921.4458**	461.2266	904.4193	452.7133	903.4353	452.2213	8
8	759.3706	380.1889			741.3600	**371.1836**	A	**822.3774**	411.6924	805.3509	403.1791	804.3669	402.6871	7
9	874.3975	437.7024			856.3869	428.6971	D	751.3403	376.1738	734.3138	367.6605	733.3297	367.1685	6
10	1002.4925	501.7499	985.4659	493.2366	984.4819	492.7446	K	**636.3134**	318.6603	619.2868	310.1470	618.3028	309.6550	5
11	1116.5354	558.7713	1099.5088	550.2581	1098.5248	549.7660	N	**508.2184**	254.6128	491.1919	246.0996	490.2078	245.6076	4
12	**1276.5660**	638.7867	1259.5395	630.2734	1258.5555	629.7814	C	**394.1755**	197.5914	377.1489	189.0781	376.1649	188.5861	3
13	**1363.5981**	682.3027	1346.5715	673.7894	1345.5875	673.2974	S	234.1448	117.5761	217.1183	109.0628	216.1343	108.5708	2
14							K	147.1128	74.0600	130.0863	65.5468			1

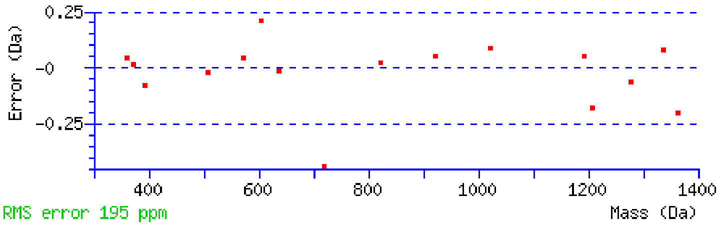

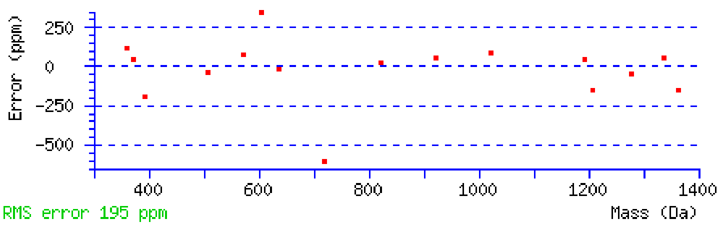

All matches to this query

Score	Mr(calc):	Delta	Sequence
54.0	1508.6963	0.0008	ATEGMVVADKNCSK
5.0	1508.7065	-0.0094	AFKYFSSLTTHK
4.1	1508.6946	0.0025	KTFMKDSVVGSSK
3.4	1508.7096	-0.0126	SIQNNVREAVSGR
3.3	1507.6893	1.0078	TSPGGRTSPEARGR
3.3	1507.6893	1.0078	TSPGGRTSPEARGR
3.0	1507.7045	0.9926	TGLRPDHRPDHK
0.9	1508.6872	0.0099	SVPGSEPSKTQGQK
0.1	1507.6837	1.0134	ELKSSKAPAYVR

Spectrum No: 134; Query: 43576; Rank: 1

Peptide View

MS/MS Fragmentation of **DGIVLGADTR**
Found in **IPI00003217**, Tax_Id=9606 Gene_Symbol=PSMB7 Proteasome subunit beta type-7

Match to Query 43576: 1015.531288 from(508.772920,2+)
Title: 090702LimSK_Exosome2_02.2420.2420.2.dta
Data file C:\Temp\Mascoti\LSK_2D\090702LimSK_Exosome2_01_2D.mgf

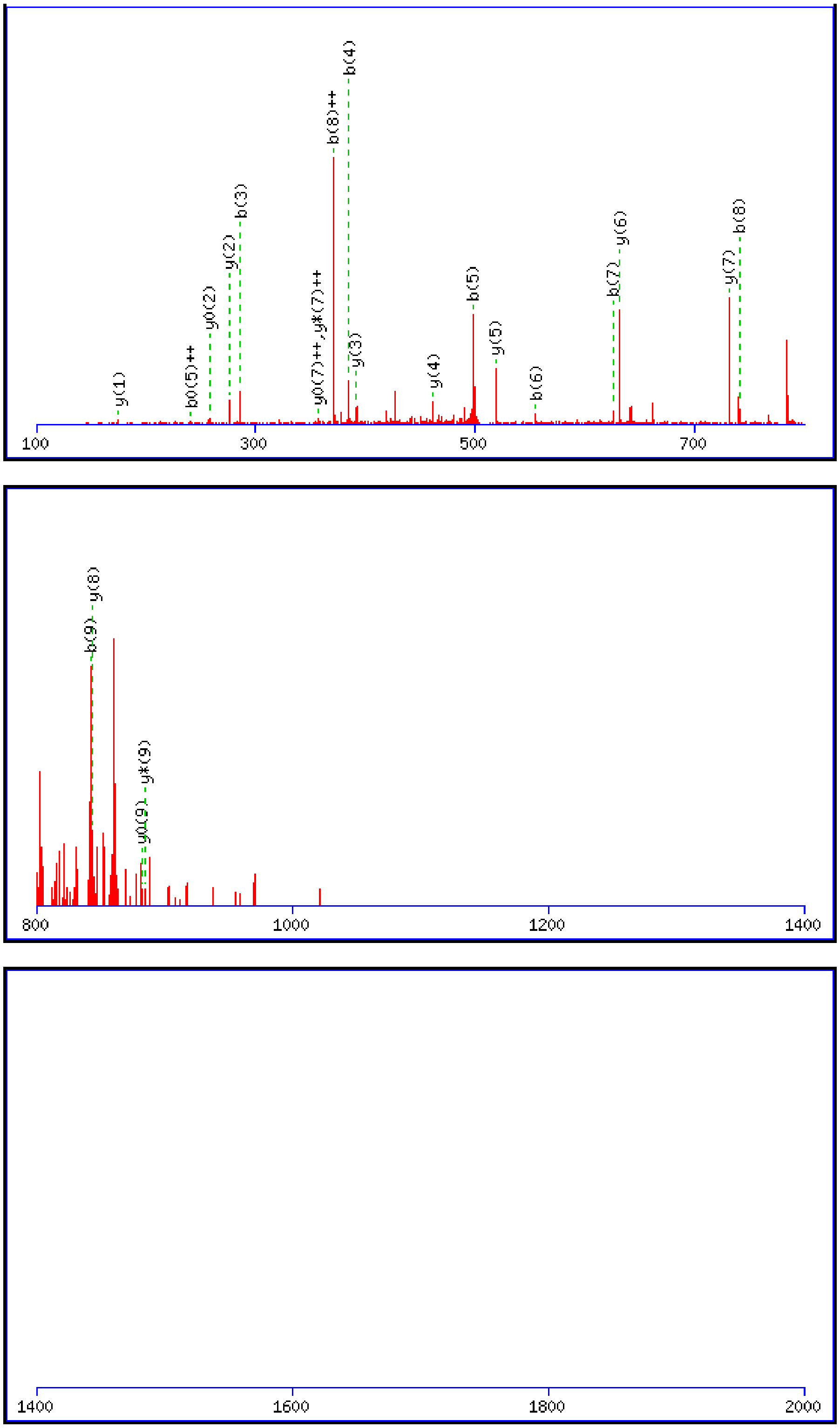

Monoisotopic mass of neutral peptide Mr(calc): 1015.5298
Fixed modifications: Carbamidomethyl (C)
Ions Score: 36 **Expect:** 0.031
Matches (Bold Red): 22/88 fragment ions using 59 most intense peaks

#	b	b^{++}	b^{0}	b^{0++}	Seq.	y	y^{++}	y*	y^{*++}	y^{0}	y^{0++}	#
1	116.0342	58.5207	98.0237	49.5155	D							10
2	173.0557	87.0315	155.0451	78.0262	G	901.5102	451.2587	**884.4836**	442.7454	**883.4996**	442.2534	9
3	**286.1397**	143.5735	268.1292	134.5682	I	**844.4887**	422.7480	827.4621	414.2347	826.4781	413.7427	8
4	**385.2082**	193.1077	367.1976	184.1024	V	**731.4046**	366.2060	714.3781	**357.6927**	713.3941	**357.2007**	7
5	**498.2922**	249.6498	480.2817	**240.6445**	L	**632.3362**	316.6717	615.3097	308.1585	614.3257	307.6665	6
6	**555.3137**	278.1605	537.3031	269.1552	G	**519.2522**	260.1297	502.2256	251.6164	501.2416	251.1244	5

7	626.3508	313.6790	608.3402	304.6738	A	462.2307	231.6190	445.2041	223.1057	444.2201	222.6137	4
8	741.3777	371.1925	723.3672	362.1872	D	391.1936	196.1004	374.1670	187.5872	373.1830	187.0951	3
9	842.4254	421.7164	824.4149	412.7111	T	276.1666	138.5870	259.1401	130.0737	258.1561	129.5817	2
10					R	175.1190	88.0631	158.0924	79.5498			1

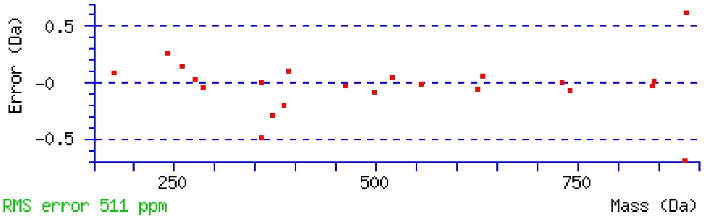

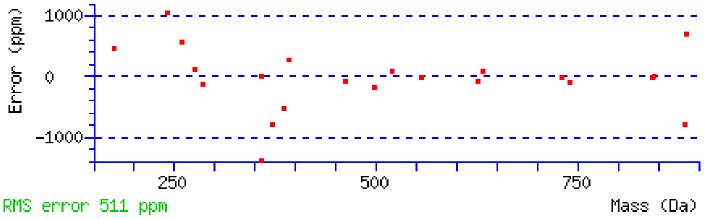

All matches to this query

Score	Mr(calc):	Delta	Sequence
36.1	1015.5298	0.0015	DGIVLGADTR
14.1	1015.5410	-0.0098	GQQALGSSLR
11.2	1013.5182	2.0131	YVSGYGLQK
10.5	1014.5319	0.9994	AGRVGGQSGAR
9.9	1014.5206	1.0106	TARPAGADTR
9.5	1015.5298	0.0015	LATVVNDER
7.8	1015.5298	0.0015	EQIGAAKDGK
7.1	1013.5310	2.0003	ILRLYEK
6.2	1013.5171	2.0142	KFRNLTR
6.1	1015.5216	0.0097	FKSIKTGR

Spectrum No: 135; Query: 64003; Rank: 1

Peptide View

MS/MS Fragmentation of **FRPDMEEEEAK**
Found in **IPI00003217**, Tax_Id=9606 Gene_Symbol=PSMB7 Proteasome subunit beta type-7

Match to Query 64003: 1379.607042 from(460.876290,3+)
Title: 090702LimSK_Exosome2_06.4814.4814.3.dta
Data file C:\Temp\Mascoti\LSK_2D\090702LimSK_Exosome2_01_2D.mgf

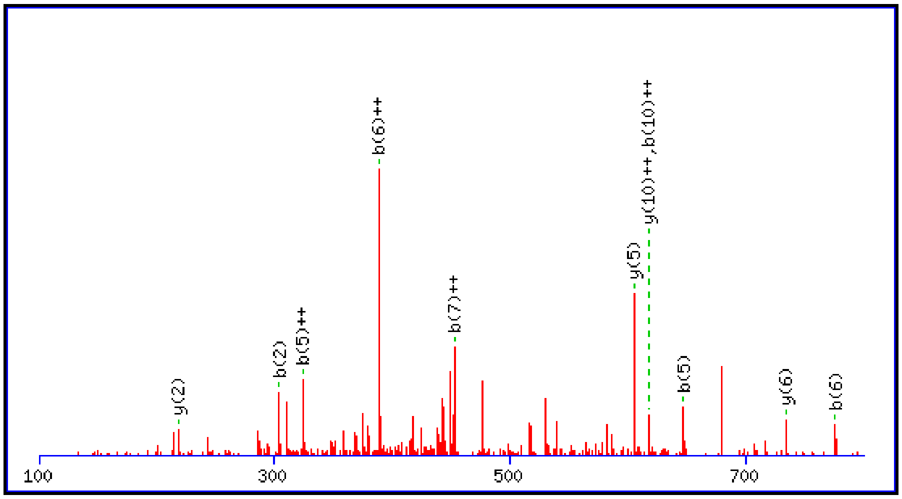

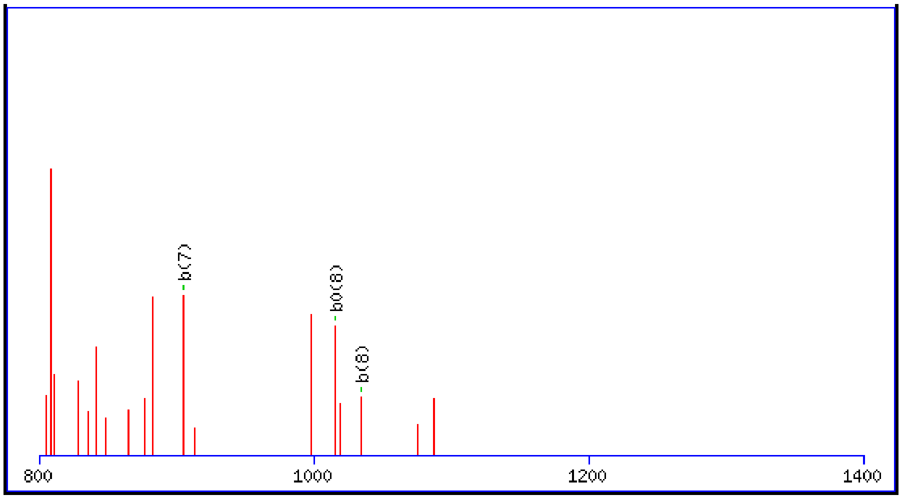

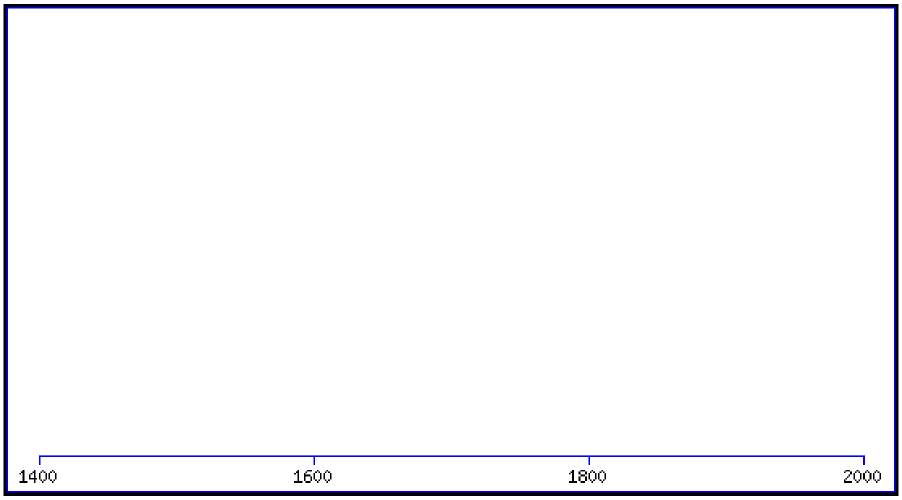

Monoisotopic mass of neutral peptide Mr(calc): 1379.6027
Fixed modifications: Carbamidomethyl (C)
Ions Score: 28 **Expect:** 0.31
Matches (Bold Red): 14/108 fragment ions using 22 most intense peaks

#	b	b++	b*	b*++	b0	b0++	Seq.	y	y++	y*	y*++	y0	y0++	#
1	148.0757	74.5415					F							11
2	**304.1768**	152.5920	287.1503	144.0788			R	1233.5416	**617.2744**	1216.5150	608.7612	1215.5310	608.2692	10
3	401.2296	201.1184	384.2030	192.6051			P	1077.4405	539.2239	1060.4139	530.7106	1059.4299	530.2186	9
4	516.2565	258.6319	499.2300	250.1186	498.2459	249.6266	D	980.3877	490.6975	963.3612	482.1842	962.3772	481.6922	8
5	**647.2970**	**324.1521**	630.2704	315.6389	629.2864	315.1469	M	865.3608	433.1840	848.3342	424.6708	847.3502	424.1787	7
6	**776.3396**	**388.6734**	759.3130	380.1602	758.3290	379.6681	E	**734.3203**	367.6638	717.2937	359.1505	716.3097	358.6585	6
7	**905.3822**	**453.1947**	888.3556	444.6815	887.3716	444.1894	E	**605.2777**	303.1425	588.2511	294.6292	587.2671	294.1372	5
8	**1034.4248**	517.7160	1017.3982	509.2027	**1016.4142**	508.7107	E	476.2351	238.6212	459.2086	230.1079	458.2245	229.6159	4
9	1163.4674	582.2373	1146.4408	573.7240	1145.4568	573.2320	E	347.1925	174.0999	330.1660	165.5866	329.1819	165.0946	3
10	1234.5045	**617.7559**	1217.4779	609.2426	1216.4939	608.7506	A	**218.1499**	109.5786	201.1234	101.0653			2
11							K	147.1128	74.0600	130.0863	65.5468			1

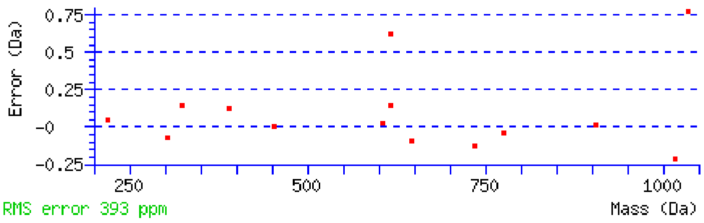

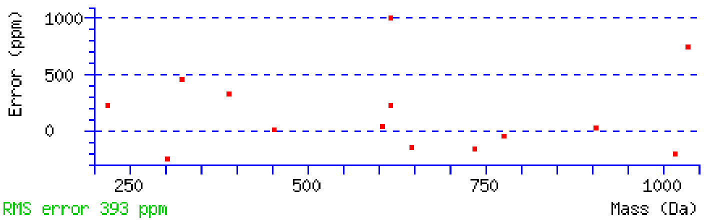

All matches to this query

Score	Mr(calc):	Delta	Sequence
27.7	1379.6027	0.0043	FRPDMEEEEAK
20.4	1378.5952	1.0118	SFCTAKESTIR
12.8	1378.5952	1.0118	SFCTAKESTIR
10.9	1379.6099	-0.0029	MADAAATAGAGGSGTR
10.6	1379.6172	-0.0102	ITTEKMLLQK
6.4	1377.6112	1.9959	AAAPAPVSEAVCR
6.2	1378.6129	0.9941	HVSAEEKEKDK
5.7	1379.6178	-0.0108	QNAHPGEQTDGAR
4.9	1379.6082	-0.0012	SSQHKQDLTEK
4.8	1379.6082	-0.0012	SSQHKQDLTEK

Spectrum No: 136; Query: 39582; Rank: 1

Peptide View

MS/MS Fragmentation of **GTTAVLTEK**
Found in **IPI00003217**, Tax_Id=9606 Gene_Symbol=PSMB7 Proteasome subunit beta type-7

Match to Query 39582: 918.503548 from(460.259050,2+)
Title: 090702LimSK_Exosome2_05.1432.1432.2.dta
Data file C:\Temp\Mascoti\LSK_2D\090702LimSK_Exosome2_01_2D.mgf

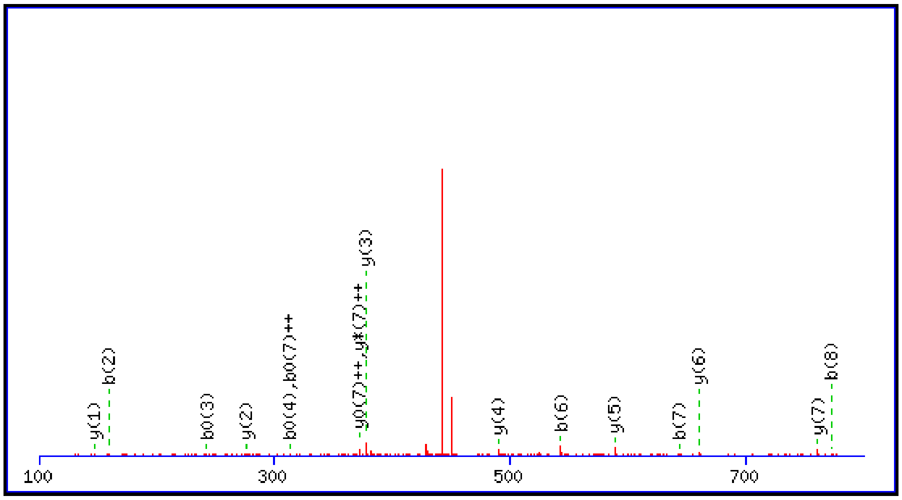

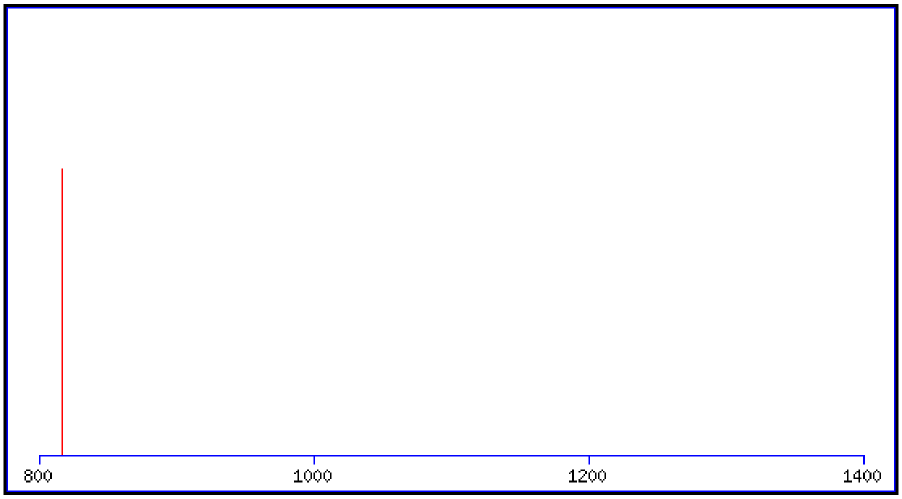

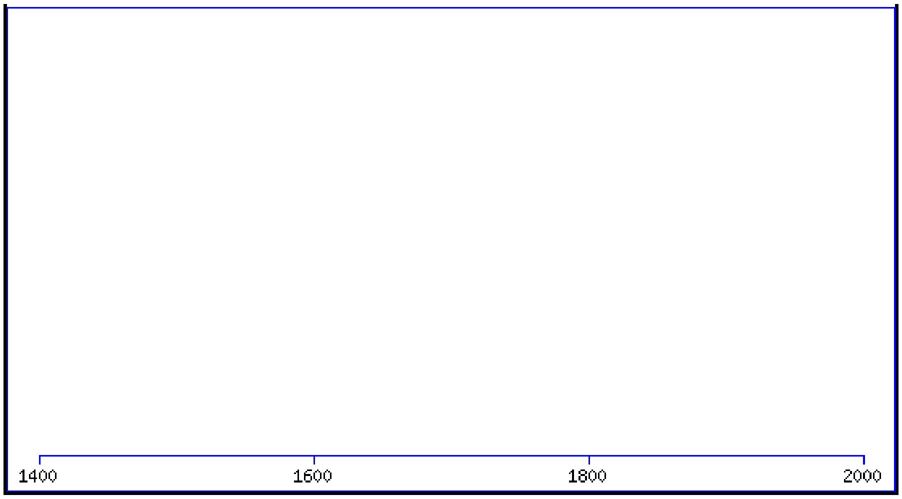

Monoisotopic mass of neutral peptide Mr(calc): 918.5022
Fixed modifications: Carbamidomethyl (C)
Ions Score: 67 **Expect:** 3e-005
Matches (Bold Red): 16/76 fragment ions using 22 most intense peaks

#	b	b^{++}	b^0	b^{0++}	Seq.	y	y^{++}	y*	y^{*++}	y^0	y^{0++}	#
1	58.0287	29.5180			G							9
2	**159.0764**	80.0418	141.0659	71.0366	T	862.4880	431.7477	845.4615	423.2344	844.4775	422.7424	8
3	260.1241	130.5657	**242.1135**	121.5604	T	**761.4403**	381.2238	744.4138	**372.7105**	743.4298	**372.2185**	7
4	331.1612	166.0842	**313.1506**	157.0790	A	**660.3927**	330.7000	643.3661	322.1867	642.3821	321.6947	6
5	430.2296	215.6185	412.2191	206.6132	V	**589.3556**	295.1814	572.3290	286.6681	571.3450	286.1761	5
6	**543.3137**	272.1605	525.3031	263.1552	L	**490.2871**	245.6472	473.2606	237.1339	472.2766	236.6419	4
7	**644.3614**	322.6843	626.3508	**313.6790**	T	**377.2031**	189.1052	360.1765	180.5919	359.1925	180.0999	3
8	**773.4040**	387.2056	755.3934	378.2003	E	**276.1554**	138.5813	259.1288	130.0681	258.1448	129.5761	2
9					K	**147.1128**	74.0600	130.0863	65.5468			1

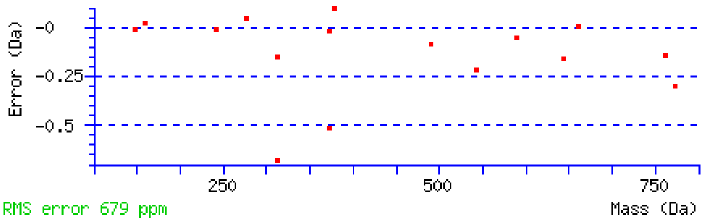

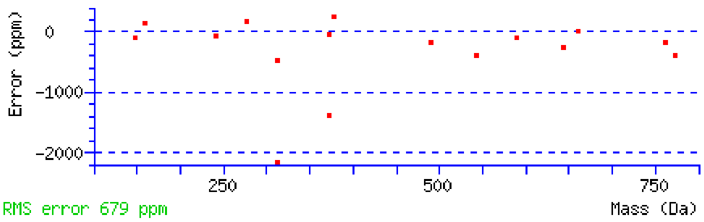

All matches to this query

Score	Mr(calc):	Delta	Sequence
66.6	918.5022	0.0013	GTTAVLTEK
20.6	918.5069	-0.0033	MAASIVRR
20.5	917.4930	1.0105	RKEQTEK
18.7	916.4978	2.0058	IAKETNNK
17.4	917.4930	1.0105	SKNVTNQK
15.7	916.5018	2.0017	QFVPAVEK
14.6	918.4957	0.0079	MENRLLK
14.2	917.4930	1.0105	REEQTKK
14.2	917.4930	1.0105	REQETKK
14.0	918.4957	0.0079	KMADAKQK

Spectrum No: 137; Query: 87481; Rank: 1

Peptide View

MS/MS Fragmentation of **ITPLEIEVLEETVQTMDTS**
Found in **IPI00003217**, Tax_Id=9606 Gene_Symbol=PSMB7 Proteasome subunit beta type-7

Match to Query 87481: 2147.062248 from(1074.538400,2+)
Title: 090702LimSK_Exosome2_01.10276.10276.2.dta
Data file C:\Temp\Mascoti\LSK_2D\090702LimSK_Exosome2_01_2D.mgf

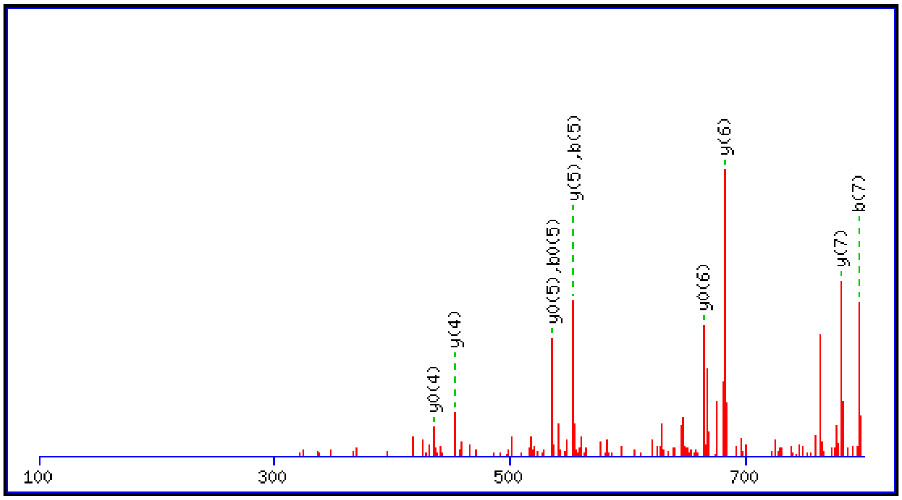

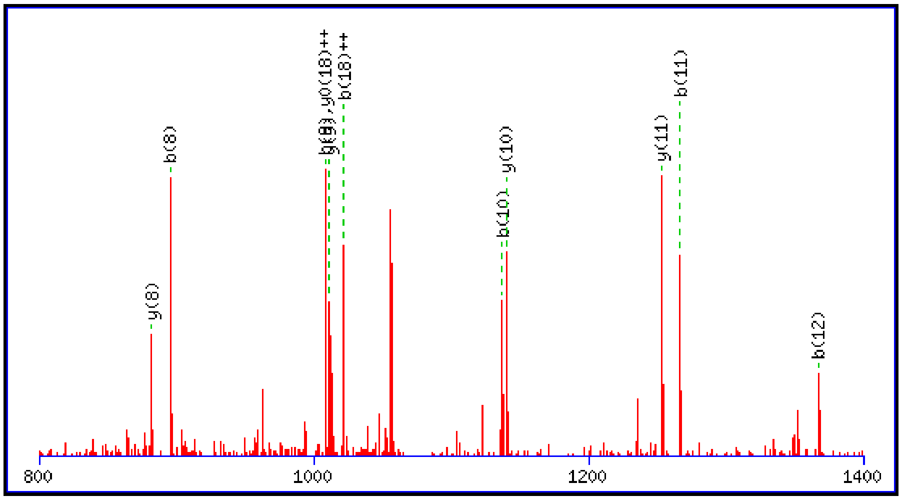

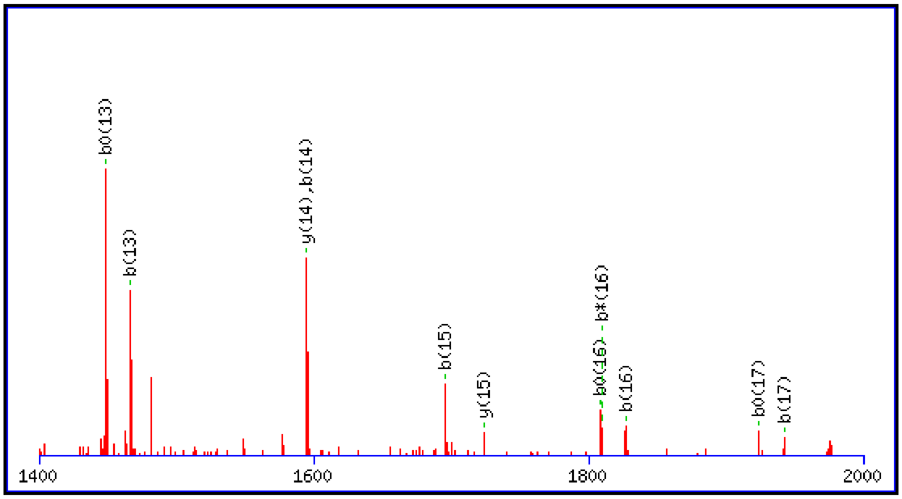

Monoisotopic mass of neutral peptide Mr(calc): 2147.0555
Fixed modifications: Carbamidomethyl (C)
Ions Score: 108 **Expect:** 3.7e-009
Matches (Bold Red): 32/178 fragment ions using 36 most intense peaks

#	b	b++	b*	b*++	b0	b0++	Seq.	y	y++	y*	y*++	y0	y0++	#
1	114.0913	57.5493					I							19
2	215.1390	108.0731			197.1285	99.0679	T	2034.9787	1017.9930	2017.9522	1009.4797	2016.9682	**1008.9877**	18
3	312.1918	156.5995			294.1812	147.5942	P	1933.9311	967.4692	1916.9045	958.9559	1915.9205	958.4639	17
4	425.2758	213.1416			407.2653	204.1363	L	1836.8783	918.9428	1819.8518	910.4295	1818.8677	909.9375	16
5	**554.3184**	277.6629			**536.3079**	268.6576	E	**1723.7942**	862.4008	1706.7677	853.8875	1705.7837	853.3955	15
6	667.4025	334.2049			649.3919	325.1996	I	**1594.7516**	797.8795	1577.7251	789.3662	1576.7411	788.8742	14
7	**796.4451**	398.7262			778.4345	389.7209	E	1481.6676	741.3374	1464.6410	732.8242	1463.6570	732.3321	13
8	**895.5135**	448.2604			877.5029	439.2551	V	1352.6250	676.8161	1335.5984	668.3029	1334.6144	667.8109	12
9	**1008.5976**	504.8024			990.5870	495.7971	L	**1253.5566**	627.2819	1236.5300	618.7687	1235.5460	618.2766	11
10	**1137.6402**	569.3237			1119.6296	560.3184	E	**1140.4725**	570.7399	1123.4460	562.2266	1122.4619	561.7346	10
11	**1266.6828**	633.8450			1248.6722	624.8397	E	**1011.4299**	506.2186	994.4034	497.7053	993.4194	497.2133	9
12	**1367.7304**	684.3689			1349.7199	675.3636	T	**882.3873**	441.6973	865.3608	433.1840	864.3768	432.6920	8
13	**1466.7989**	733.9031			**1448.7883**	724.8978	V	**781.3396**	391.1735	764.3131	382.6602	763.3291	382.1682	7
14	**1594.8574**	797.9324	1577.8309	789.4191	1576.8469	788.9271	Q	**682.2712**	341.6393	665.2447	333.1260	**664.2607**	332.6340	6
15	**1695.9051**	848.4562	1678.8786	839.9429	1677.8945	839.4509	T	**554.2127**	277.6100			**536.2021**	268.6047	5
16	**1826.9456**	913.9764	**1809.9190**	905.4632	**1808.9350**	904.9712	M	**453.1650**	227.0861			**435.1544**	218.0808	4
17	**1941.9725**	971.4899	1924.9460	962.9766	**1923.9620**	962.4846	D	322.1245	161.5659			304.1139	152.5606	3
18	2043.0202	**1022.0137**	2025.9937	1013.5005	2025.0097	1013.0085	T	207.0975	104.0524			189.0870	95.0471	2
19							S	106.0499	53.5286			88.0393	44.5233	1

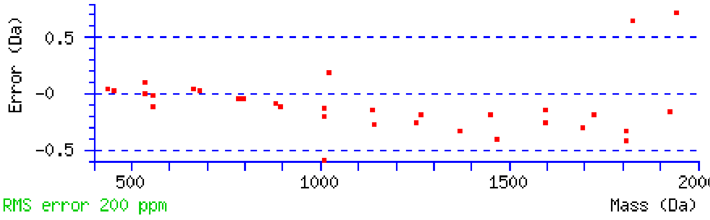

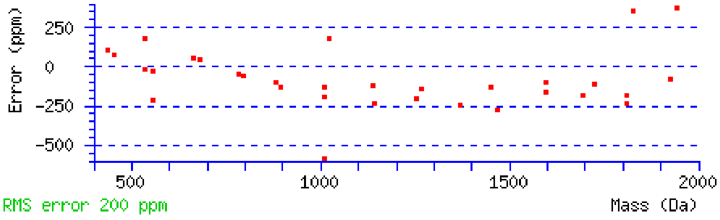

All matches to this query

Score	Mr(calc):	Delta	Sequence
108.1	2147.0555	0.0067	ITPLEIEVLEETVQTMDTS
8.9	2145.0620	2.0003	QQPFAYGTLTVRSLLDTR
7.5	2147.0615	0.0008	YGRLLPLDMILISLGASR
7.5	2147.0615	0.0008	YGRLLPLDMILISLGASR
7.3	2145.0579	2.0043	LQQTTQLIKEADEKHQR
6.9	2145.0483	2.0139	LEIDINKITVKTNESIR
6.3	2147.0736	-0.0114	DTTVGTLSQRITNQVHGLK
6.3	2147.0736	-0.0114	DTTVGTLSQRITNQVHGLK
5.4	2145.0467	2.0156	KLETTLNGAHSTSEGPAKPK
5.1	2145.0579	2.0043	LQQTTQLIKEADEKHQR

Spectrum No: 138; Query: 67537; Rank: 1

Peptide View

MS/MS Fragmentation of **LDFLRPYTVPNK**
Found in **IPI00003217**, Tax_Id=9606 Gene_Symbol=PSMB7 Proteasome subunit beta type-7

Match to Query 67537: 1461.800592 from(488.274140,3+)
Title: 090702LimSK_Exosome2_01.6003.6003.3.dta

Data file C:\Temp\Mascoti\LSK_2D\090702LimSK_Exosome2_01_2D.mgf

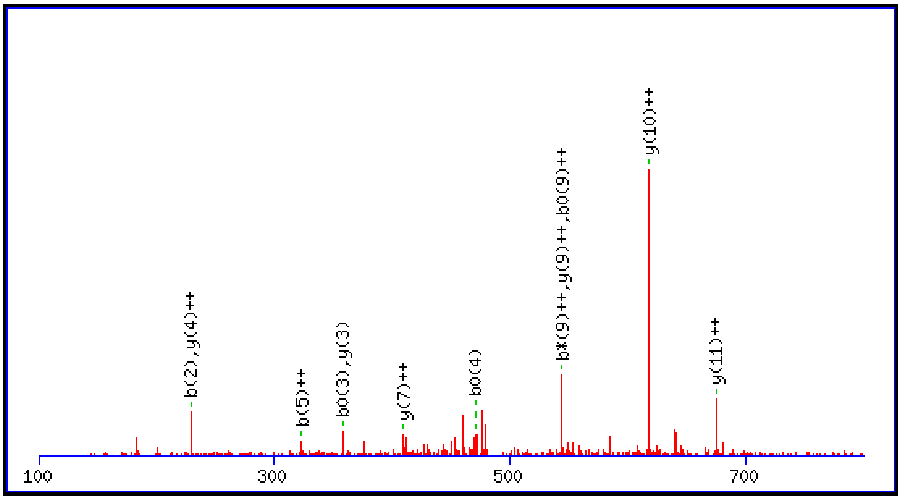

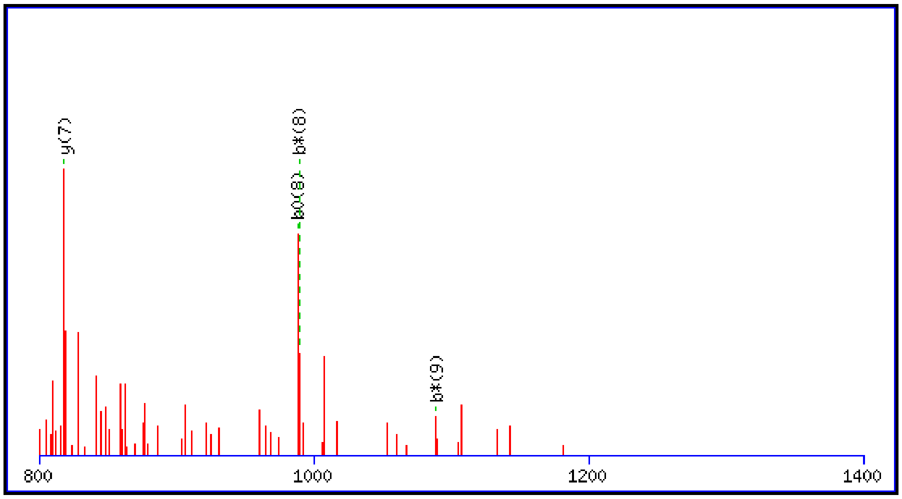

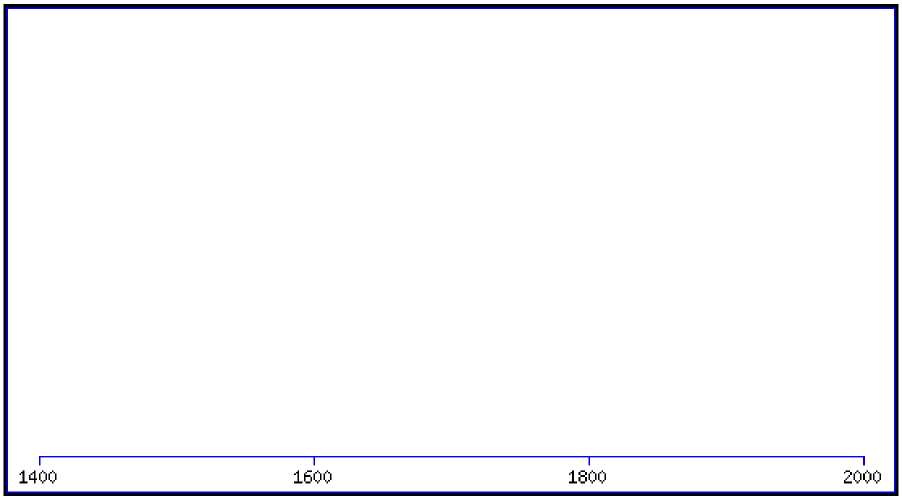

Monoisotopic mass of neutral peptide Mr(calc): 1461.7980
Fixed modifications: Carbamidomethyl (C)
Ions Score: 21 **Expect:** 0.78
Matches (Bold Red): 16/114 fragment ions using 33 most intense peaks

#	b	b^{++}	b*	b^{*++}	b^0	b^{0++}	Seq.	y	y^{++}	y*	y^{*++}	y^0	y^{0++}	#
1	114.0913	57.5493					L							12
2	**229.1183**	115.0628			211.1077	106.0575	D	1349.7212	**675.3642**	1332.6947	666.8510	1331.7106	666.3590	11
3	376.1867	188.5970			**358.1761**	179.5917	F	1234.6943	**617.8508**	1217.6677	609.3375	1216.6837	608.8455	10
4	489.2708	245.1390			**471.2602**	236.1337	L	1087.6259	**544.3166**	1070.5993	535.8033	1069.6153	535.3113	9
5	645.3719	**323.1896**	628.3453	314.6763	627.3613	314.1843	R	974.5418	487.7745	957.5152	479.2613	956.5312	478.7693	8

**6**	742.4246	371.7160	725.3981	363.2027	724.4141	362.7107	**P**	**818.4407**	**409.7240**	801.4141	401.2107	800.4301	400.7187	**7**
**7**	905.4880	453.2476	888.4614	444.7343	887.4774	444.2423	**Y**	721.3879	361.1976	704.3614	352.6843	703.3774	352.1923	**6**
**8**	1006.5356	503.7715	**989.5091**	495.2582	**988.5251**	494.7662	**T**	558.3246	279.6659	541.2980	271.1527	540.3140	270.6606	**5**
**9**	1105.6041	553.3057	**1088.5775**	**544.7924**	1087.5935	**544.3004**	**V**	457.2769	**229.1421**	440.2504	220.6288			**4**
**10**	1202.6568	601.8320	1185.6303	593.3188	1184.6463	592.8268	**P**	**358.2085**	179.6079	341.1819	171.0946			**3**
**11**	1316.6997	658.8535	1299.6732	650.3402	1298.6892	649.8482	**N**	261.1557	131.0815	244.1292	122.5682			**2**
**12**							**K**	147.1128	74.0600	130.0863	65.5468			**1**

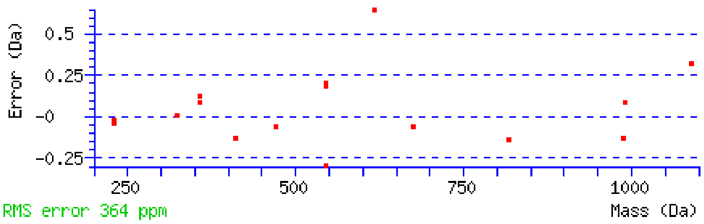

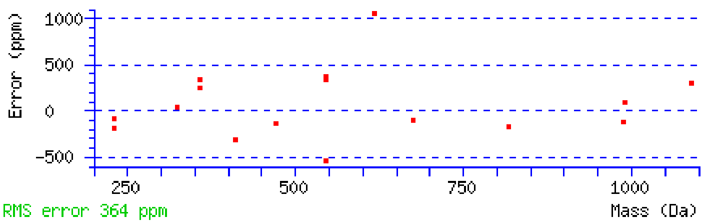

All matches to this query

Score	Mr(calc):	Delta	Sequence
21.2	1461.7980	0.0026	LDFLRPYTVPNK
14.0	1460.7875	1.0131	LLFSATLTQNPEK
13.9	1461.7861	0.0145	MPEVSSKGATISKK
8.3	1461.7874	0.0132	MPFTGERRLSLR
3.8	1461.7939	0.0066	NLFTALNAVEKSR
3.1	1460.8003	1.0003	LLERLLPGTIEK
3.1	1460.7977	1.0029	ILAVSPSRRINR
3.1	1460.7977	1.0029	ILAVSPSRRINR
2.4	1461.7861	0.0145	QTLKNMLDKEVK
1.8	1461.7861	0.0145	DLIISEMQRLTK

Spectrum No: 139; Query: 39012; Rank: 1

Peptide View

MS/MS Fragmentation of **AIAYATHR**
Found in **IPI00000783**, Tax_Id=9606 Gene_Symbol=PSMB8 Isoform 1 of Proteasome subunit beta type-8

Match to Query 39012: 901.479688 from(451.747120,2+)
Title: 090702LimSK_Exosome2_06.2469.2469.2.dta
Data file C:\Temp\Mascoti\LSK_2D\090702LimSK_Exosome2_01_2D.mgf

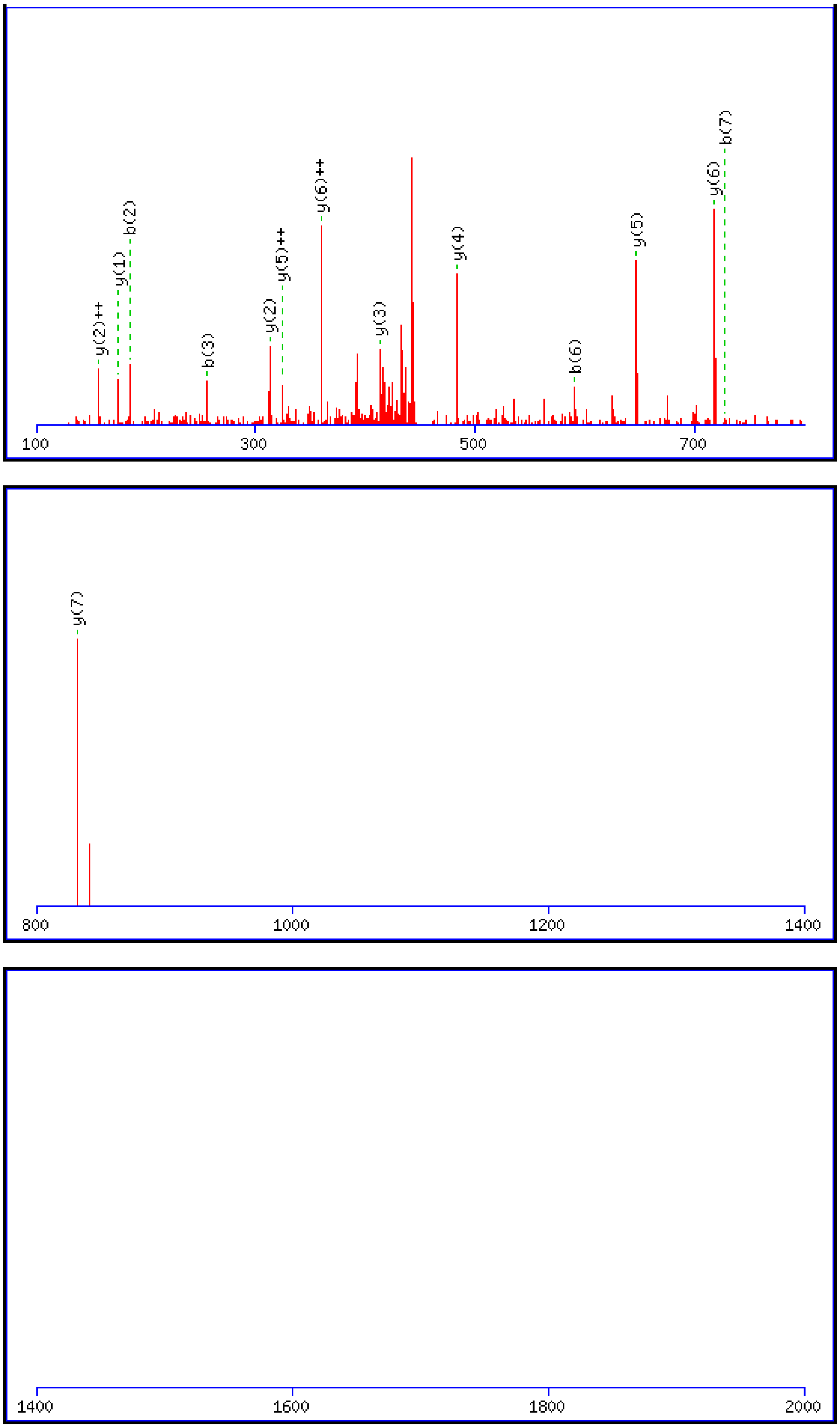

Monoisotopic mass of neutral peptide Mr(calc): 901.4770
Fixed modifications: Carbamidomethyl (C)
Ions Score: 58 **Expect:** 0.00022
Matches (Bold Red): 14/56 fragment ions using 26 most intense peaks

#	b	b++	b0	b0++	Seq.	y	y++	y*	y*++	y0	y0++	#
1	72.0444	36.5258			A							8
2	**185.1285**	93.0679			I	**831.4472**	416.2272	814.4206	407.7139	813.4366	407.2219	7
3	**256.1656**	128.5864			A	**718.3631**	**359.6852**	701.3366	351.1719	700.3525	350.6799	6
4	419.2289	210.1181			Y	**647.3260**	**324.1666**	630.2994	315.6534	629.3154	315.1613	5
5	490.2660	245.6366			A	**484.2627**	242.6350	467.2361	234.1217	466.2521	233.6297	4
6	**591.3137**	296.1605	573.3031	287.1552	T	**413.2255**	207.1164	396.1990	198.6031	395.2150	198.1111	3

7	728.3726	364.6899	710.3620	355.6847	H	312.1779	156.5926	295.1513	148.0793			2
8					R	175.1190	88.0631	158.0924	79.5498			1

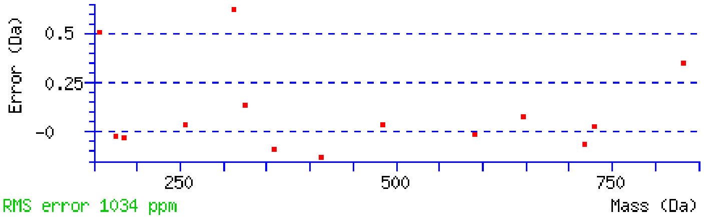

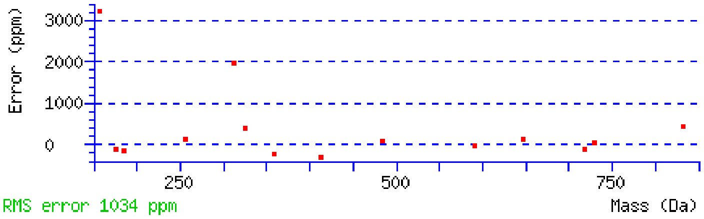

All matches to this query

Score	Mr(calc):	Delta	Sequence
57.6	901.4770	0.0027	AIAYATHR
17.2	901.4869	-0.0072	AIALETER
15.0	901.4868	-0.0072	AIEEANKK
10.3	901.4756	0.0040	ALDIDVEK
10.3	901.4869	-0.0072	ALVEQSQK
8.3	901.4730	0.0067	ALREGSGGR
8.0	901.4882	-0.0085	RREWQK
7.0	900.4739	1.0058	LAACEPLK
5.0	900.4777	1.0020	GINLSGGQR
5.0	900.4777	1.0020	GLNLSGGQR

Spectrum No: 140; Query: 47848; Rank: 1

Peptide View

MS/MS Fragmentation of **ASAGSYISALR**
Found in **IPI00000783**, Tax_Id=9606 Gene_Symbol=PSMB8 Isoform 1 of Proteasome subunit beta type-8

Match to Query 47848: 1094.574528 from(548.294540,2+)
Title: 090702LimSK_Exosome2_06.5353.5353.2.dta
Data file C:\Temp\Mascoti\LSK_2D\090702LimSK_Exosome2_01_2D.mgf

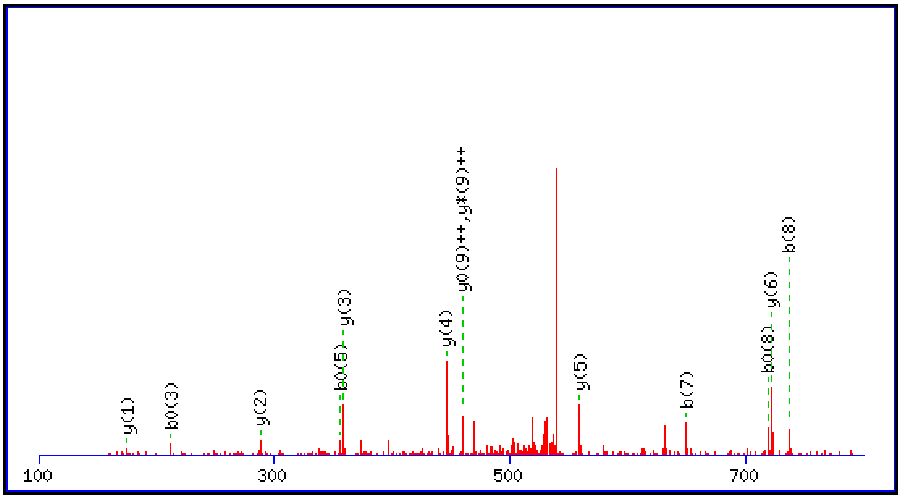

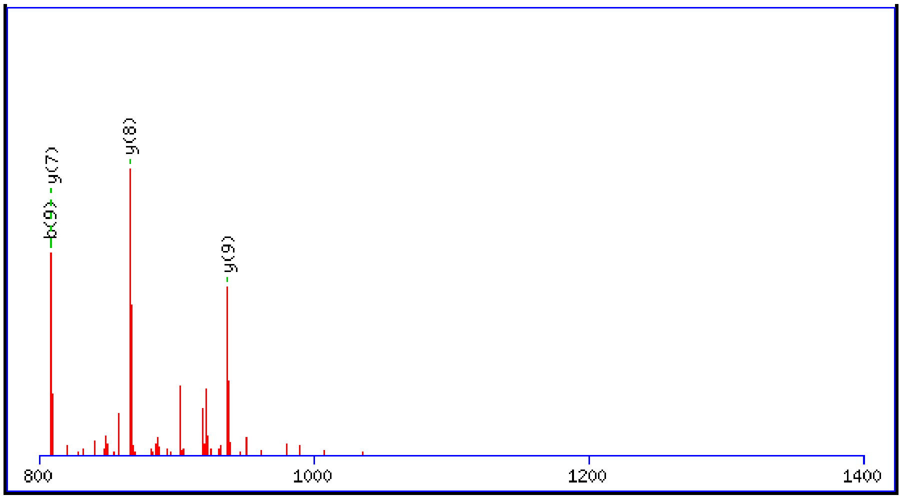

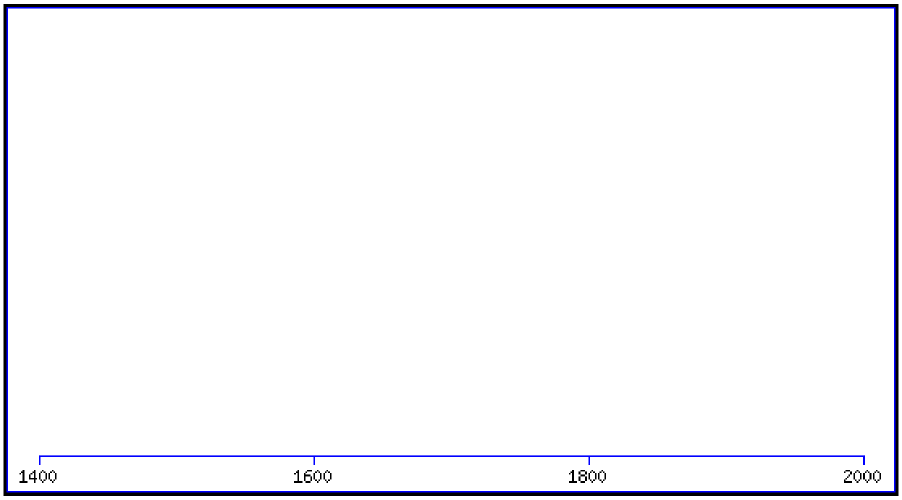

Monoisotopic mass of neutral peptide Mr(calc): 1094.5720
Fixed modifications: Carbamidomethyl (C)
Ions Score: 94 **Expect:** 5.1e-008
Matches (Bold Red): 17/92 fragment ions using 23 most intense peaks

#	b	b^{++}	b^{0}	b^{0++}	Seq.	y	y^{++}	y*	y^{*++}	y^{0}	y^{0++}	#
1	72.0444	36.5258			A							11
2	159.0764	80.0418	141.0659	71.0366	S	1024.5422	512.7747	1007.5156	504.2615	1006.5316	503.7694	10
3	230.1135	115.5604	**212.1030**	106.5551	A	**937.5102**	469.2587	920.4836	**460.7454**	919.4996	**460.2534**	9
4	287.1350	144.0711	269.1244	135.0659	G	**866.4730**	433.7402	849.4465	425.2269	848.4625	424.7349	8
5	374.1670	187.5872	**356.1565**	178.5819	S	**809.4516**	405.2294	792.4250	396.7162	791.4410	396.2241	7
6	537.2304	269.1188	519.2198	260.1135	Y	**722.4196**	361.7134	705.3930	353.2001	704.4090	352.7081	6
7	**650.3144**	325.6608	632.3039	316.6556	I	**559.3562**	280.1817	542.3297	271.6685	541.3457	271.1765	5
8	**737.3464**	369.1769	**719.3359**	360.1716	S	**446.2722**	223.6397	429.2456	215.1264	428.2616	214.6344	4
9	**808.3836**	404.6954	790.3730	395.6901	A	**359.2401**	180.1237	342.2136	171.6104			3
10	921.4676	461.2374	903.4571	452.2322	L	**288.2030**	144.6051	271.1765	136.0919			2
11					R	**175.1190**	88.0631	158.0924	79.5498			1

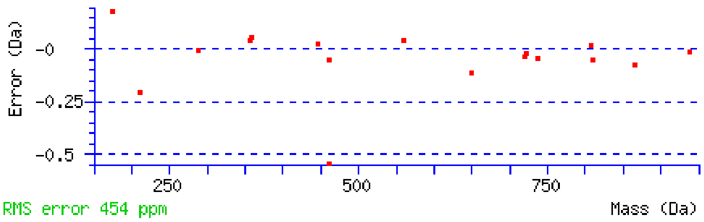

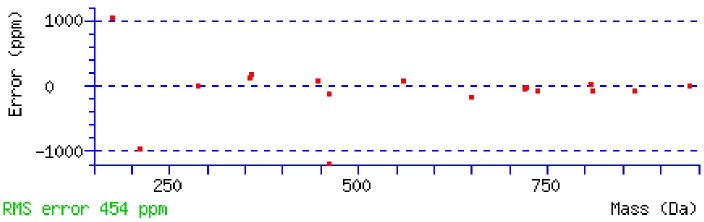

All matches to this query

Score	Mr(calc):	Delta	Sequence
93.9	1094.5720	0.0025	ASAGSYISALR
12.4	1093.5757	0.9988	RAVASGVKAR
12.2	1094.5849	-0.0104	ASKLTQVLR
12.1	1094.5849	-0.0104	TGKLNLLTR
11.7	1093.5669	1.0077	VWYSLDRR
11.0	1092.5692	2.0053	IQKTPGTLR
10.7	1093.5702	1.0043	DLMRQFLR
10.5	1092.5692	2.0053	INASGGLILR
10.4	1092.5750	1.9996	IYCNALALR
9.7	1093.5757	0.9988	RAGSGVAKIR

Spectrum No: 141; Query: 60187; Rank: 1

Peptide View

MS/MS Fragmentation of **FQHGVIAAVDSR**
Found in **IPI00000783**, Tax_Id=9606 Gene_Symbol=PSMB8 Isoform 1 of Proteasome subunit beta type-8

Match to Query 60187: 1298.675742 from(433.899190,3+)
Title: 090702LimSK_Exosome2_06.6897.6897.3.dta
Data file C:\Temp\Mascoti\LSK_2D\090702LimSK_Exosome2_01_2D.mgf

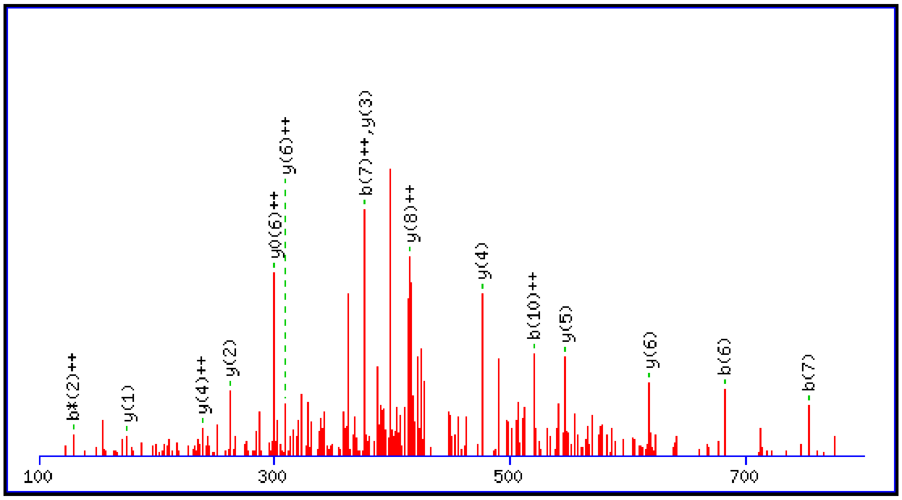

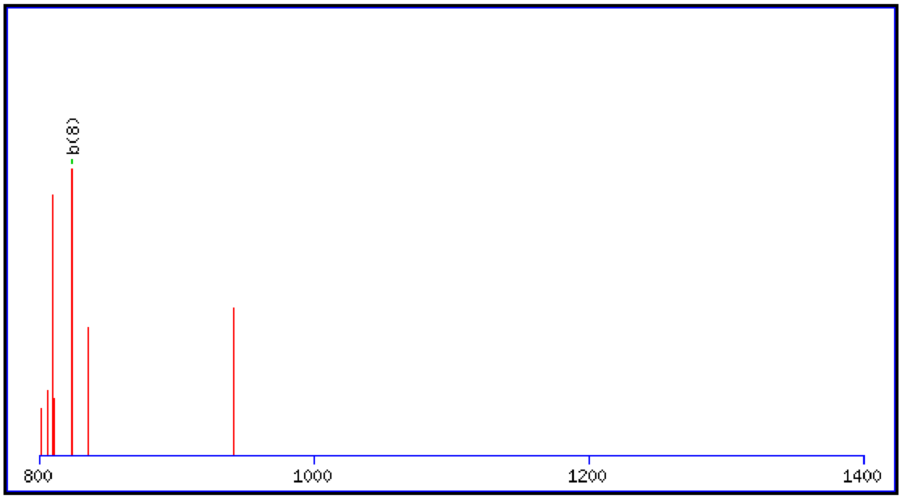

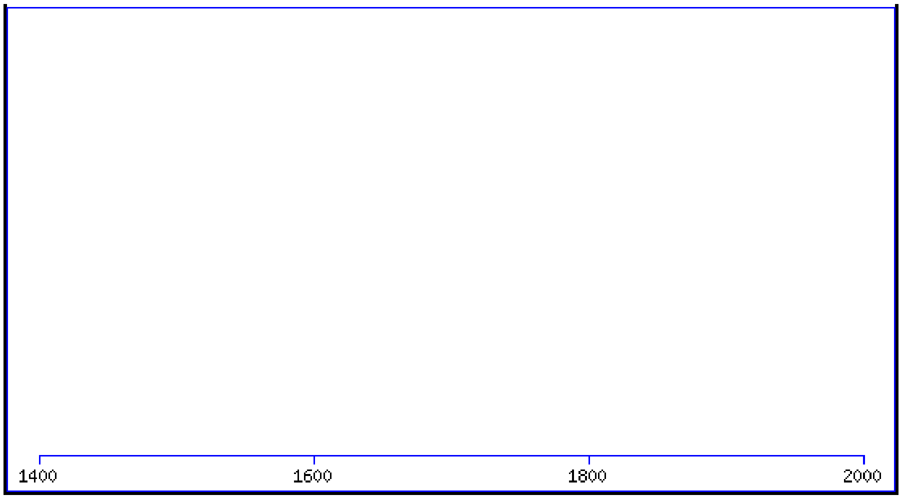

Monoisotopic mass of neutral peptide Mr(calc): 1298.6731
Fixed modifications: Carbamidomethyl (C)
Ions Score: 33 **Expect:** 0.059
Matches (Bold Red): 16/110 fragment ions using 31 most intense peaks

#	b	b^{++}	b*	b^{*++}	b^0	b^{0++}	Seq.	y	y^{++}	y*	y^{*++}	y^0	y^{0++}	#
1	148.0757	74.5415					F							12
2	276.1343	138.5708	259.1077	**130.0575**			Q	1152.6120	576.8096	1135.5854	568.2964	1134.6014	567.8044	11
3	413.1932	207.1002	396.1666	198.5870			H	1024.5534	512.7803	1007.5269	504.2671	1006.5429	503.7751	10
4	470.2146	235.6110	453.1881	227.0977			G	887.4945	444.2509	870.4680	435.7376	869.4839	435.2456	9
5	569.2831	285.1452	552.2565	276.6319			V	830.4730	**415.7402**	813.4465	407.2269	812.4625	406.7349	8
6	**682.3671**	341.6872	665.3406	333.1739			I	731.4046	366.2060	714.3781	357.6927	713.3941	357.2007	7
7	**753.4042**	**377.2058**	736.3777	368.6925			A	**618.3206**	**309.6639**	601.2940	301.1506	600.3100	**300.6586**	6
8	**824.4413**	412.7243	807.4148	404.2110			A	**547.2835**	274.1454	530.2569	265.6321	529.2729	265.1401	5
9	923.5098	462.2585	906.4832	453.7452			V	**476.2463**	**238.6268**	459.2198	230.1135	458.2358	229.6215	4
10	1038.5367	**519.7720**	1021.5102	511.2587	1020.5261	510.7667	D	**377.1779**	189.0926	360.1514	180.5793	359.1674	180.0873	3
11	1125.5687	563.2880	1108.5422	554.7747	1107.5582	554.2827	S	**262.1510**	131.5791	245.1244	123.0659	244.1404	122.5738	2
12							R	**175.1190**	88.0631	158.0924	79.5498			1

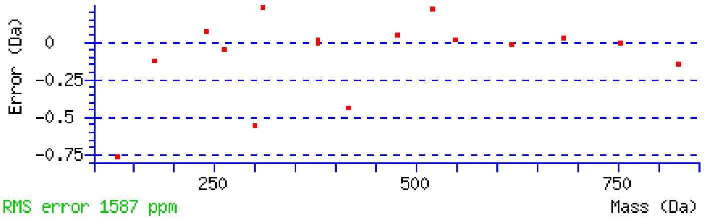

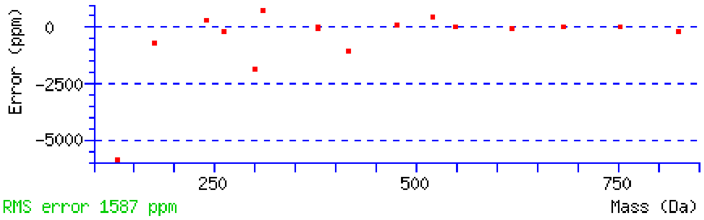

All matches to this query

Score	Mr(calc):	Delta	Sequence
33.5	1298.6731	0.0026	FQHGVIAAVDSR
8.8	1298.6747	0.0010	LARIVEIYSR
7.7	1296.6687	2.0071	YRHGAPAEQLR
6.5	1296.6737	2.0020	RGSLLGSMKLR
5.7	1298.6731	0.0026	RPWEPSALSTR
5.2	1296.6575	2.0183	QHPFEDGAKLR
5.0	1297.6779	0.9979	ERLAFYSSLGR
4.2	1296.6687	2.0070	XSPFRGPPGAAGAR
3.6	1297.6643	1.0115	SRVTISVDTLK
3.5	1296.6625	2.0133	QETKLKLMAR

Spectrum No: 142; Query: 30424; Rank: 1

Peptide View

MS/MS Fragmentation of **ISVSAASK**
Found in **IPI00000783**, Tax_Id=9606 Gene_Symbol=PSMB8 Isoform 1 of Proteasome subunit beta type-8

Match to Query 30424: 761.429448 from(381.722000,2+)
Title: 090702LimSK_Exosome2_06.933.933.2.dta
Data file C:\Temp\Mascoti\LSK_2D\090702LimSK_Exosome2_01_2D.mgf

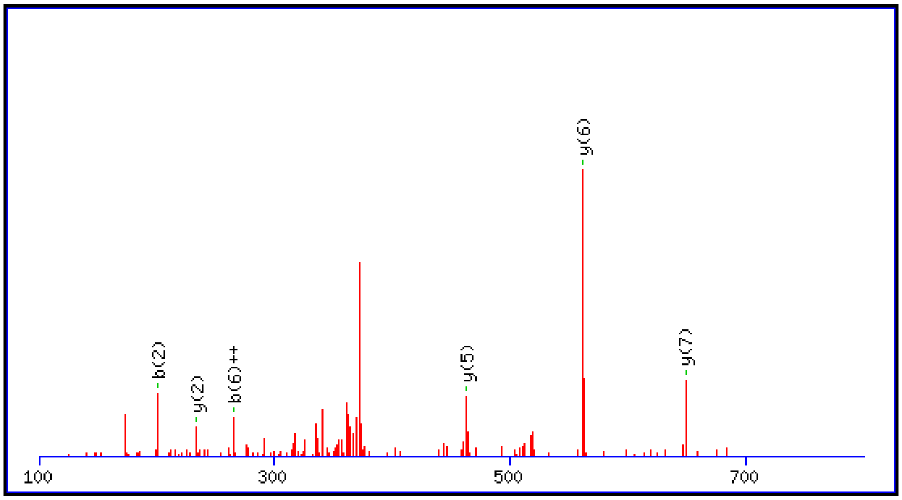

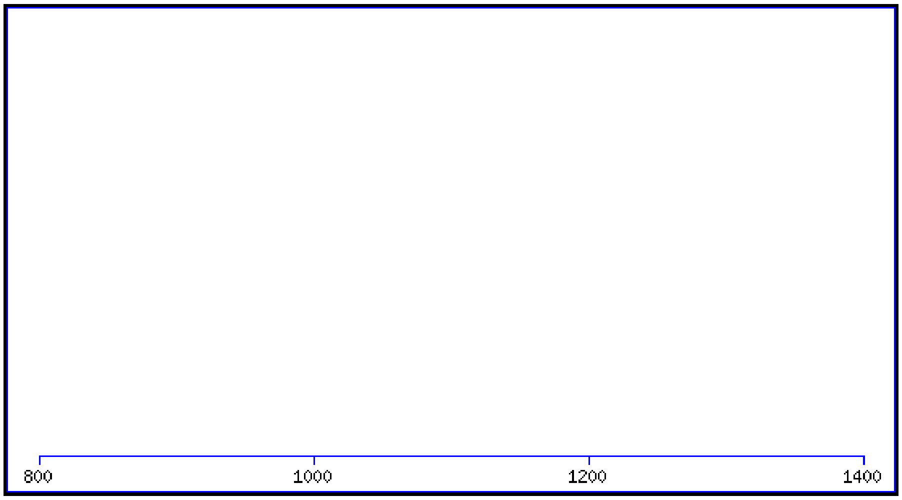

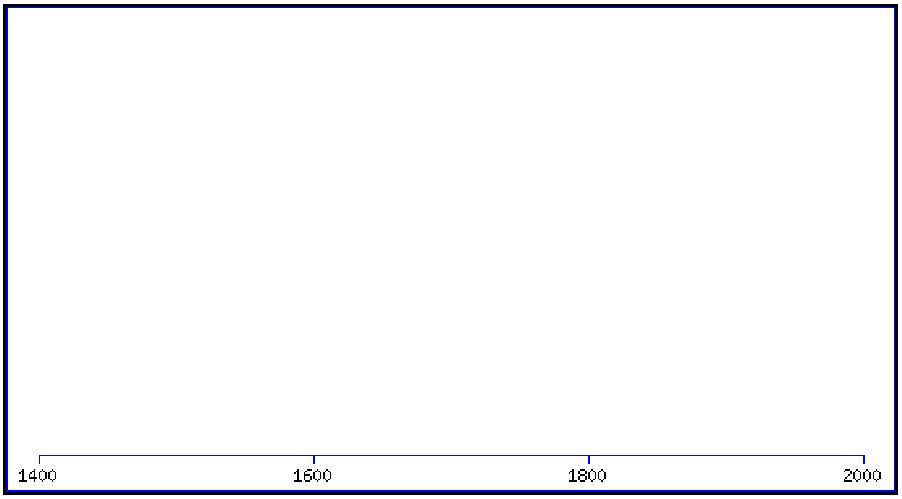

Monoisotopic mass of neutral peptide Mr(calc): 761.4283
Fixed modifications: Carbamidomethyl (C)
Ions Score: 21 **Expect:** 1.1
Matches (Bold Red): 6/66 fragment ions using 13 most intense peaks

#	b	b^{++}	b^0	b^{0++}	Seq.	y	y^{++}	y*	y^{*++}	y^0	y^{0++}	#
1	114.0913	57.5493			I							8
2	**201.1234**	101.0653	183.1128	92.0600	S	**649.3515**	325.1794	632.3250	316.6661	631.3410	316.1741	7
3	300.1918	150.5995	282.1812	141.5942	V	**562.3195**	281.6634	545.2930	273.1501	544.3089	272.6581	6
4	387.2238	194.1155	369.2132	185.1103	S	**463.2511**	232.1292	446.2245	223.6159	445.2405	223.1239	5
5	458.2609	229.6341	440.2504	220.6288	A	376.2191	188.6132	359.1925	180.0999	358.2085	179.6079	4
6	529.2980	**265.1527**	511.2875	256.1474	A	305.1819	153.0946	288.1554	144.5813	287.1714	144.0893	3
7	616.3301	308.6687	598.3195	299.6634	S	**234.1448**	117.5761	217.1183	109.0628	216.1343	108.5708	2
8					K	147.1128	74.0600	130.0863	65.5468			1

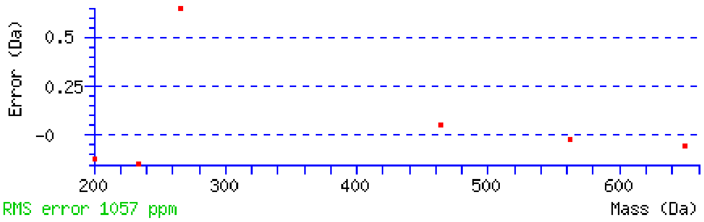

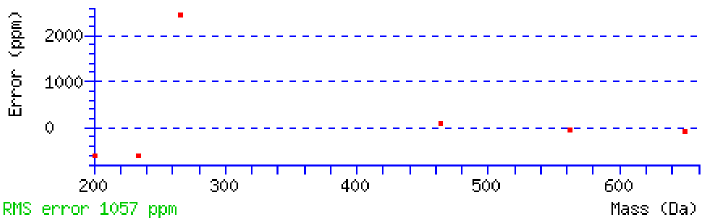

All matches to this query

Score	Mr(calc):	Delta	Sequence
20.8	761.4283	0.0011	ISVSAASK
10.9	759.4239	2.0056	LQRESK
9.8	759.4239	2.0056	IKSAEGR
9.5	761.4283	0.0011	SLGSAISK
8.7	761.4323	-0.0029	ISLPIGY
8.7	761.4283	0.0011	LSSTQVK
8.6	761.4283	0.0011	SIVTSQK
8.4	761.4283	0.0012	AELSKSK
5.5	760.4330	0.9964	ISEAKLT
5.5	760.4191	1.0103	KEGSKGR

Spectrum No: 143; Query: 61152; Rank: 1

Peptide View

MS/MS Fragmentation of **LLSNMMCQYR**
Found in **IPI00000783**, Tax_Id=9606 Gene_Symbol=PSMB8 Isoform 1 of Proteasome subunit beta type-8

Match to Query 61152: 1314.590268 from(658.302410,2+)
Title: 090702LimSK_Exosome2_06.5689.5689.2.dta
Data file C:\Temp\Mascoti\LSK_2D\090702LimSK_Exosome2_01_2D.mgf

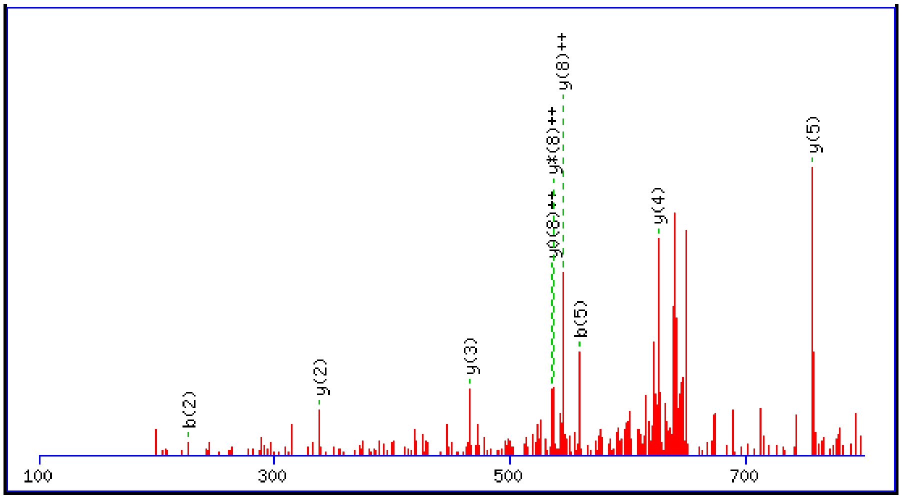

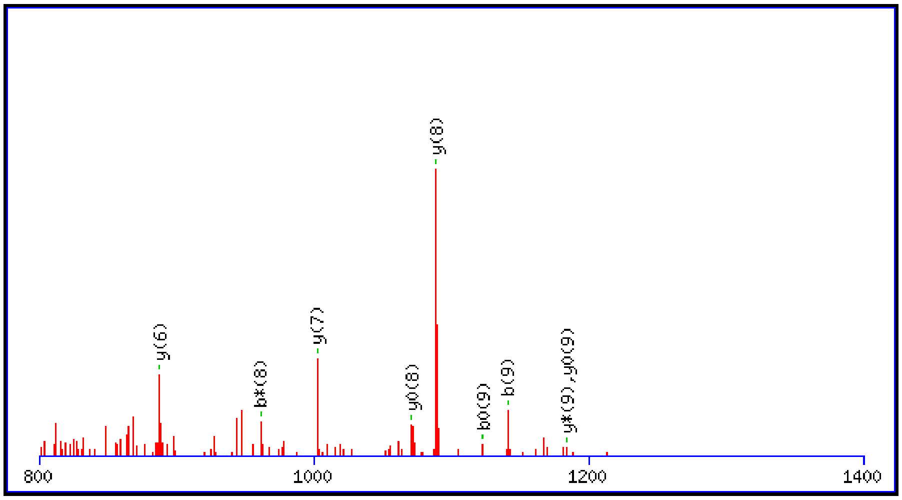

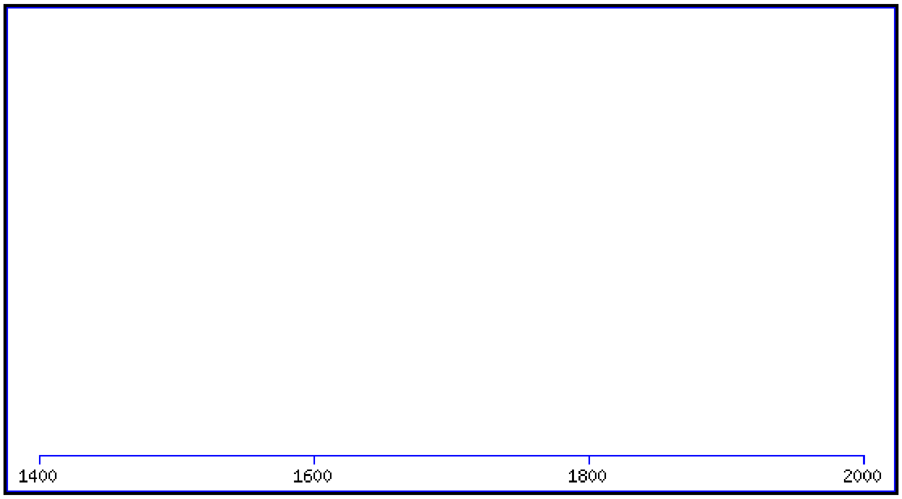

Monoisotopic mass of neutral peptide Mr(calc): 1314.5883
Fixed modifications: Carbamidomethyl (C)
Ions Score: 34 **Expect:** 0.073
Matches (Bold Red): 18/84 fragment ions using 47 most intense peaks

#	b	b^{++}	b*	b^{*++}	b^0	b^{0++}	Seq.	y	y^{++}	y*	y^{*++}	y^0	y^{0++}	#
1	114.0913	57.5493					L							10
2	**227.1754**	114.0913					L	1202.5115	601.7594	**1185.4849**	593.2461	**1184.5009**	592.7541	9
3	314.2074	157.6074			296.1969	148.6021	S	**1089.4274**	**545.2174**	1072.4009	**536.7041**	**1071.4169**	**536.2121**	8
4	428.2504	214.6288	411.2238	206.1155	410.2398	205.6235	N	**1002.3954**	501.7013	985.3689	493.1881			7
5	**559.2908**	280.1491	542.2643	271.6358	541.2803	271.1438	M	**888.3525**	444.6799	871.3259	436.1666			6
6	690.3313	345.6693	673.3048	337.1560	672.3208	336.6640	M	**757.3120**	379.1596	740.2854	370.6464			5

**7**	850.3620	425.6846	833.3354	417.1714	832.3514	416.6793	**C**	**626.2715**	313.6394	609.2450	305.1261			**4**
**8**	978.4206	489.7139	**961.3940**	481.2006	960.4100	480.7086	**Q**	**466.2409**	233.6241	449.2143	225.1108			**3**
**9**	**1141.4839**	571.2456	1124.4573	562.7323	**1123.4733**	562.2403	**Y**	**338.1823**	169.5948	321.1557	161.0815			**2**
**10**							**R**	175.1190	88.0631	158.0924	79.5498			**1**

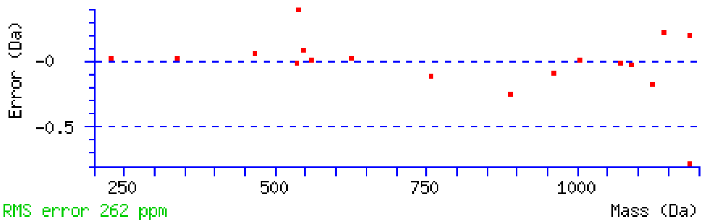

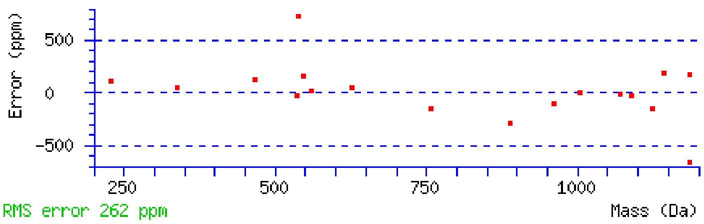

All matches to this query

Score	Mr(calc):	Delta	Sequence
33.6	1314.5883	0.0020	LLSNMMCQYR
10.5	1314.5986	-0.0083	TISPPTLGTLR
6.7	1314.6003	-0.0100	MTGKSVKDVDR
5.8	1314.5986	-0.0083	TISPPTLGTLR
5.8	1314.5986	-0.0083	TISPPTLGTLR
4.6	1314.5904	-0.0001	MLTVPHSHTGR
4.1	1314.5817	0.0086	SLSATTLDSGGAR
4.1	1314.5817	0.0086	SLSATTLDSGGAR
3.7	1314.5890	0.0012	LLETLSQMER
3.6	1314.6003	-0.0100	LLSLSCSVNSR

Spectrum No: 144; Query: 60079; Rank: 1

Peptide View

MS/MS Fragmentation of **FTTDAIALAMSR**
Found in **IPI00000787**, Tax_Id=9606 Gene_Symbol=PSMB9 Isoform LMP2.L of Proteasome subunit beta type-9

Match to Query 60079: 1295.657148 from(648.835850,2+)
Title: 090702LimSK_Exosome2_06.7631.7631.2.dta
Data file C:\Temp\Mascoti\LSK_2D\090702LimSK_Exosome2_01_2D.mgf

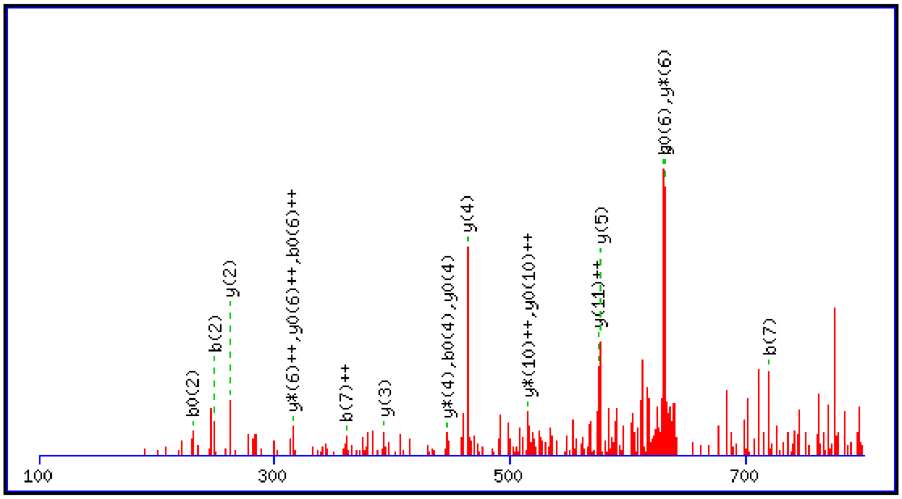

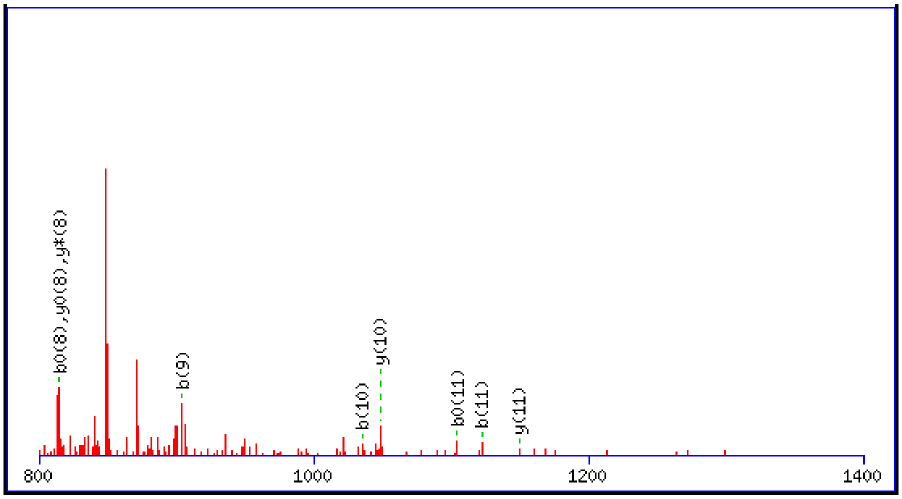

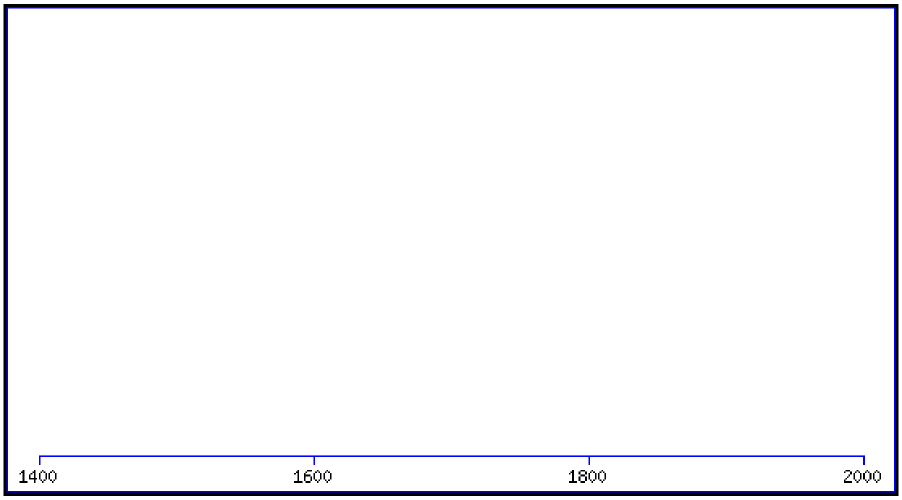

Monoisotopic mass of neutral peptide Mr(calc): 1295.6544
Fixed modifications: Carbamidomethyl (C)
Ions Score: 23 **Expect:** 0.92
Matches (Bold Red): 29/106 fragment ions using 47 most intense peaks

#	b	b++	b0	b0++	Seq.	y	y++	y*	y*++	y0	y0++	#
1	148.0757	74.5415			F							12
2	**249.1234**	125.0653	**231.1128**	116.0600	T	**1149.5932**	**575.3003**	1132.5667	566.7870	1131.5827	566.2950	11
3	350.1710	175.5892	332.1605	166.5839	T	**1048.5456**	524.7764	1031.5190	**516.2631**	1030.5350	**515.7711**	10
4	465.1980	233.1026	**447.1874**	224.0974	D	947.4979	474.2526	930.4713	465.7393	929.4873	465.2473	9
5	536.2351	268.6212	518.2245	259.6159	A	832.4709	416.7391	**815.4444**	408.2258	**814.4604**	407.7338	8
6	649.3192	325.1632	**631.3086**	**316.1579**	I	761.4338	381.2205	744.4073	372.7073	743.4233	372.2153	7
7	**720.3563**	**360.6818**	702.3457	351.6765	A	648.3498	324.6785	**631.3232**	**316.1652**	**630.3392**	**315.6732**	6
8	833.4403	417.2238	**815.4298**	408.2185	L	**577.3126**	289.1600	560.2861	280.6467	559.3021	280.1547	5
9	**904.4775**	452.7424	886.4669	443.7371	A	**464.2286**	232.6179	**447.2020**	224.1047	**446.2180**	223.6126	4
10	**1035.5179**	518.2626	1017.5074	509.2573	M	**393.1915**	197.0994	376.1649	188.5861	375.1809	188.0941	3
11	**1122.5500**	561.7786	**1104.5394**	552.7733	S	**262.1510**	131.5791	245.1244	123.0659	244.1404	122.5738	2
12					R	175.1190	88.0631	158.0924	79.5498			1

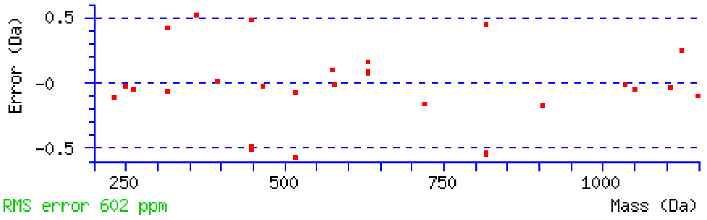

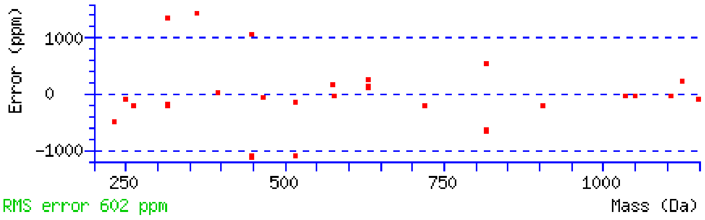

All matches to this query

Score	Mr(calc):	Delta	Sequence
22.6	1295.6544	0.0028	FTTDAIALAMSR
15.2	1294.6646	0.9926	LLGVRTEGDKK
7.5	1294.6547	1.0025	RGLHVKASGYK
4.7	1295.6696	-0.0125	TFMKLGPEAFR
2.8	1295.6445	0.0127	MAGPGPTFPLHR
1.8	1294.6517	1.0054	EAPQGELVPEAR
1.6	1293.6461	2.0110	MPAGVPMSTYLK
1.5	1294.6646	0.9926	LLRETSLQQK
0.7	1295.6622	-0.0051	EVVAHWIAESR
0.4	1293.6573	1.9998	MVVMHGYKAVK

Spectrum No: 145; Query: 42851; Rank: 1

Peptide View

MS/MS Fragmentation of **VSAGEAVVNR**
Found in **IPI00000787**, Tax_Id=9606 Gene_Symbol=PSMB9 Isoform LMP2.L of Proteasome subunit beta type-9

Match to Query 42851: 1000.531628 from(501.273090,2+)
Title: 090702LimSK_Exosome2_06.1339.1339.2.dta
Data file C:\Temp\Mascoti\LSK_2D\090702LimSK_Exosome2_01_2D.mgf

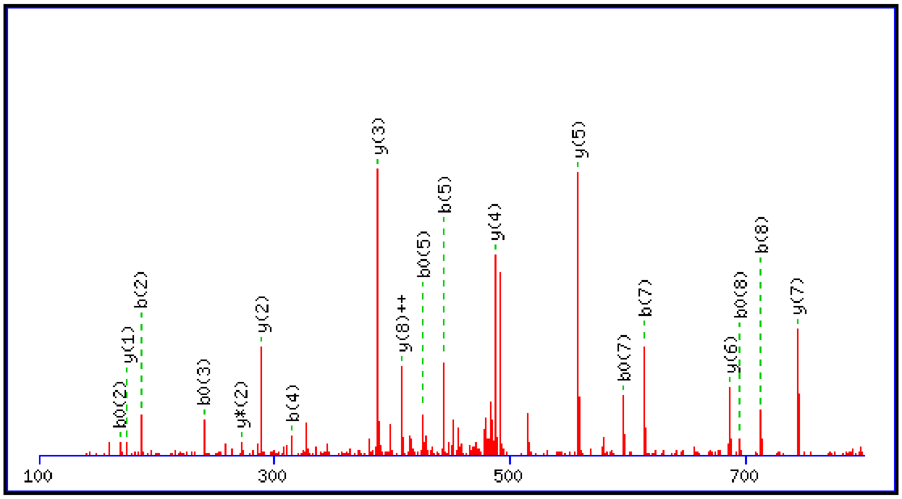

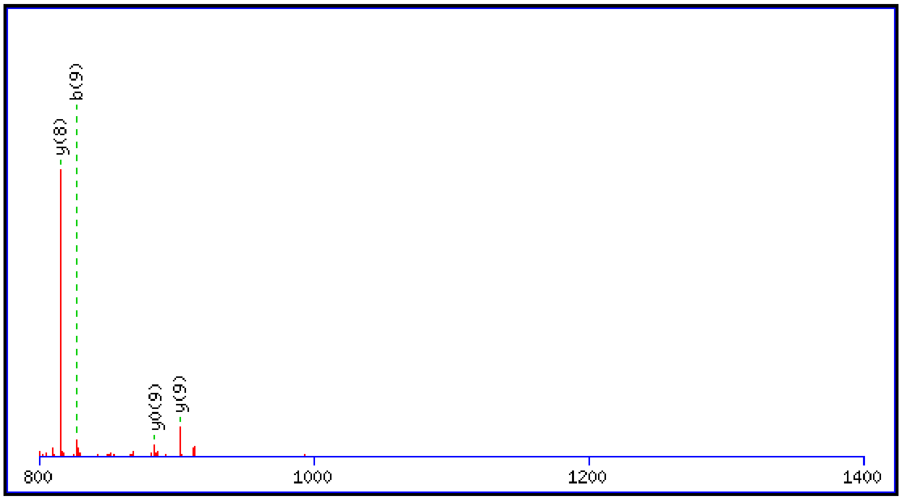

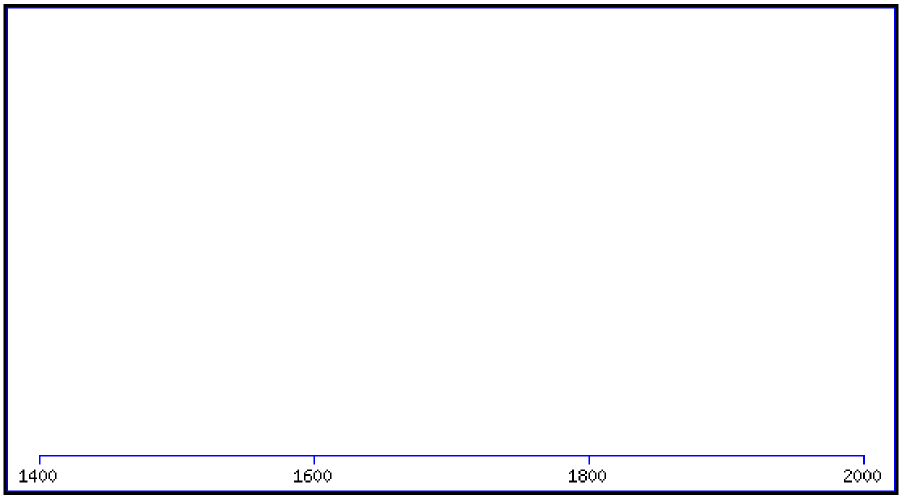

Monoisotopic mass of neutral peptide Mr(calc): 1000.5302
Fixed modifications: Carbamidomethyl (C)
Ions Score: 78 **Expect:** 1.5e-006
Matches (Bold Red): 23/80 fragment ions using 34 most intense peaks

#	b	b^{++}	b*	b^{*++}	b^{0}	b^{0++}	Seq.	y	y^{++}	y*	y^{*++}	y^{0}	y^{0++}	#
1	100.0757	50.5415					V							10
2	**187.1077**	94.0575			**169.0972**	85.0522	S	**902.4690**	451.7381	885.4425	443.2249	**884.4585**	442.7329	9
3	258.1448	129.5761			**240.1343**	120.5708	A	**815.4370**	**408.2221**	798.4104	399.7089	797.4264	399.2169	8
4	**315.1663**	158.0868			297.1557	149.0815	G	**744.3999**	372.7036	727.3733	364.1903	726.3893	363.6983	7
5	**444.2089**	222.6081			**426.1983**	213.6028	E	**687.3784**	344.1928	670.3519	335.6796	669.3678	335.1876	6
6	515.2460	258.1266			497.2354	249.1214	A	**558.3358**	279.6715	541.3093	271.1583			5
7	**614.3144**	307.6608			**596.3039**	298.6556	V	**487.2987**	244.1530	470.2722	235.6397			4
8	**713.3828**	357.1951			**695.3723**	348.1898	V	**388.2303**	194.6188	371.2037	186.1055			3
9	**827.4258**	414.2165	810.3992	405.7032	809.4152	405.2112	N	**289.1619**	145.0846	**272.1353**	136.5713			2
10							R	**175.1190**	88.0631	158.0924	79.5498			1

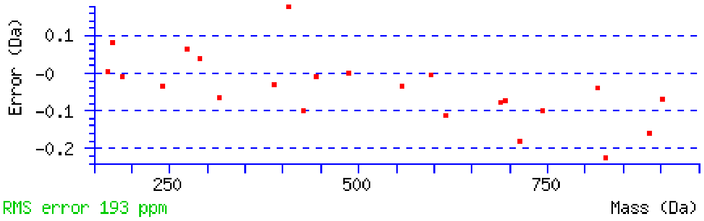

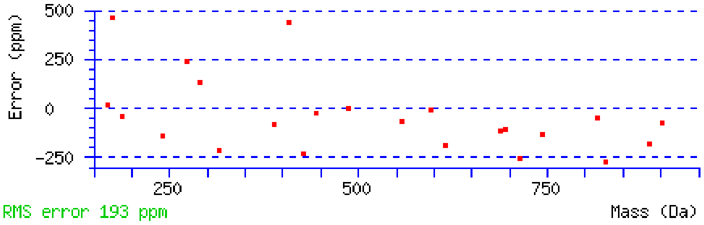

All matches to this query

Score	Mr(calc):	Delta	Sequence
78.5	1000.5302	0.0015	VSAGEAVVNR
30.8	1000.5302	0.0014	GVTGELGVGGR
13.3	1000.5301	0.0015	VSADLAQAAR
7.7	1000.5342	-0.0025	ADQWLLQK
7.0	1000.5301	0.0015	VQSGNINAAK
6.5	999.5284	1.0033	MRVIPDNR
6.1	1000.5414	-0.0098	SVLDQARGR
5.6	999.5322	0.9994	SPGGSRAGRR
5.5	1000.5414	-0.0098	VTGGPRTSAR
4.9	999.5349	0.9967	LTSGEAPGIR

Mascot: http://www.matrixscience.com/
