# Supplementary material for: Proteolytic Potential of the MSC Exosome Proteome: Implications for an Exosome-Mediated Delivery of Therapeutic Proteasome
Source: Int J Proteomics. 2012 Jul 18;2012:971907. doi: 10.1155/2012/971907 (PMC3407643; doi:10.1155/2012/971907)
Supplement: Supplementary file 1 — Supplementary material provides the raw data of the detected 20S proteasome in 3 independent batches of exosomes. Supplementary data 1 provides a summary of number of detected peptides in 3 independent batches of exosomes; Supplementary data 2–4 provide the raw data of detected 20S proteasome peptides in 3 independent batches of exosomes respectively; Supplementary data 5–7 provide the Mascot peptide view of unique 20S proteasome peptides in 3 independent batches of exosomes respectively. [file 971907.f1.zip › SupplementaryData7_Exosome3_MascotPeptideView_UniqueProteasomePeptides.pdf]

Mascot Search Results

Results Generated by a Script Modified from Mascot Pepitde View
by Newman Sze, School of Biological Sciences, Nanyang Technological University

---

Spectrum No: 1; Query: 54530; Rank: 1

Peptide View

MS/MS Fragmentation of **AITVFSPDGHLFQVEYAQEAVK**
Found in **IPI00024175**, Tax_Id=9606 Gene_Symbol=PSMA7 Isoform 1 of Proteasome subunit alpha type-7

Match to Query 54530: 2448.250902 from(817.090910,3+)
Title: 091224LimSK_Exosome3_06.6797.6797.3.dta
Data file C:\Temp\Mascoti\LSK_2D\091224LimSK_Exosome3_01_2D.mgf

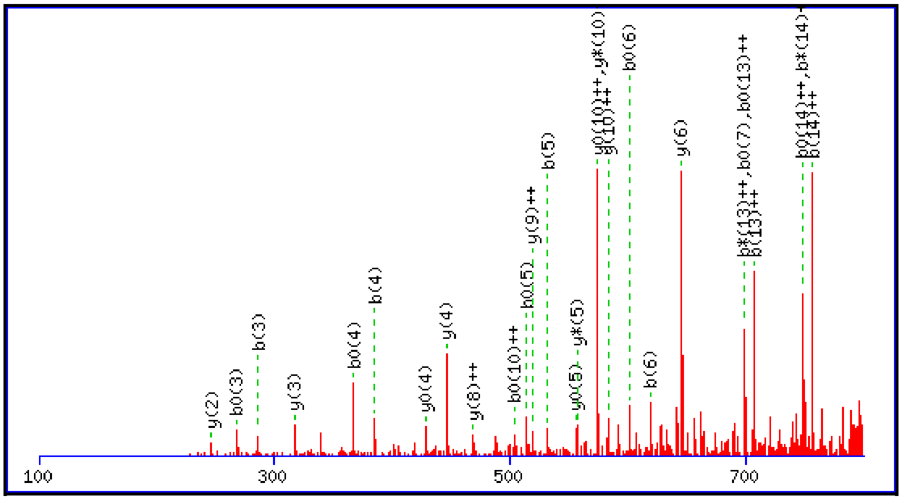

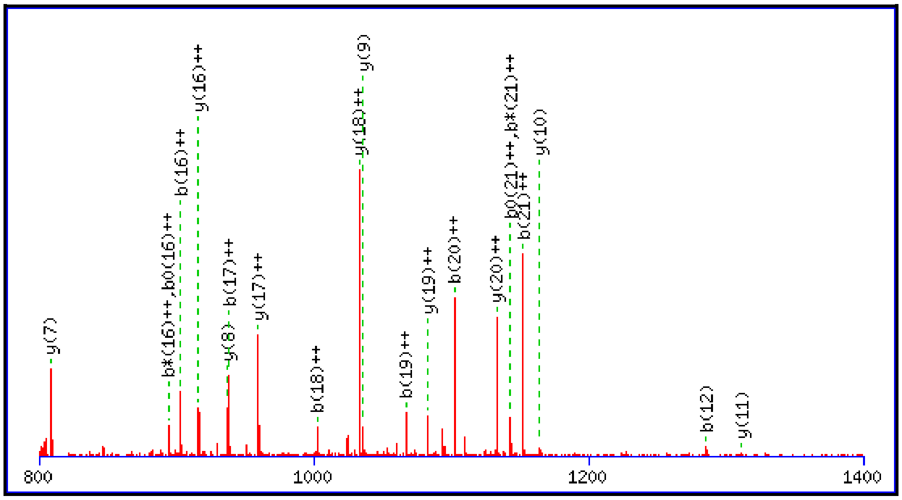

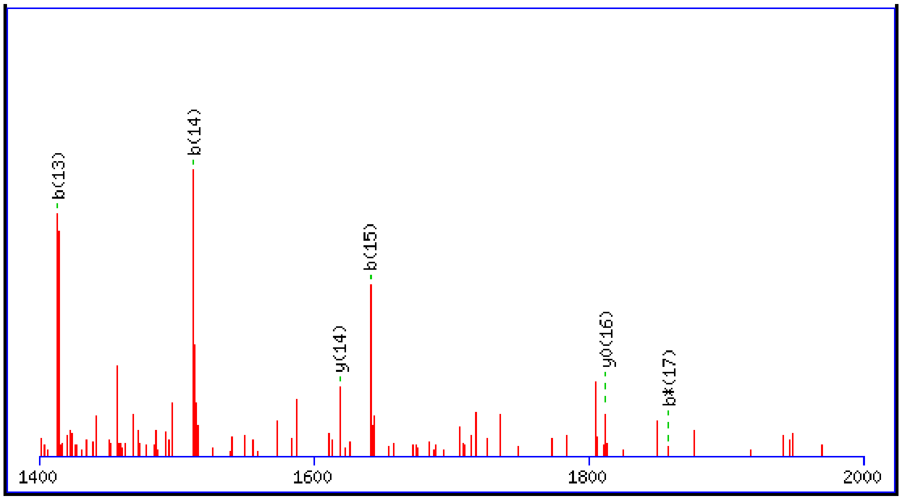

Monoisotopic mass of neutral peptide Mr(calc): 2448.2325
Fixed modifications: Carbamidomethyl (C)
Ions Score: 58 **Expect:** 0.00029
Matches (Bold Red): 56/218 fragment ions using 97 most intense peaks

#	b	b^{++}	b*	b^{*++}	b^{0}	b^{0++}	Seq.	y	y^{++}	y*	y^{*++}	y^{0}	y^{0++}	#
1	72.0444	36.5258					A							22
2	185.1285	93.0679					I	2378.2027	1189.6050	2361.1761	1181.0917	2360.1921	1180.5997	21
3	**286.1761**	143.5917			**268.1656**	134.5864	T	2265.1186	**1133.0630**	2248.0921	1124.5497	2247.1081	1124.0577	20
4	**385.2445**	193.1259			**367.2340**	184.1206	V	2164.0709	**1082.5391**	2147.0444	1074.0258	2146.0604	1073.5338	19
5	**532.3130**	266.6601			**514.3024**	257.6548	F	2065.0025	**1033.0049**	2047.9760	1024.4916	2046.9920	1023.9996	18
6	**619.3450**	310.1761			**601.3344**	301.1709	S	1917.9341	**959.4707**	1900.9076	950.9574	1899.9236	950.4654	17
7	716.3978	358.7025			**698.3872**	349.6972	P	1830.9021	**915.9547**	1813.8755	907.4414	**1812.8915**	906.9494	16
8	831.4247	416.2160			813.4141	407.2107	D	1733.8493	867.4283	1716.8228	858.9150	1715.8388	858.4230	15
9	888.4462	444.7267			870.4356	435.7214	G	**1618.8224**	809.9148	1601.7958	801.4016	1600.8118	800.9095	14
10	1025.5051	513.2562			1007.4945	**504.2509**	H	1561.8009	781.4041	1544.7744	772.8908	1543.7904	772.3988	13
11	1138.5891	569.7982			1120.5786	560.7929	L	1424.7420	712.8746	1407.7155	704.3614	1406.7314	703.8694	12
12	**1285.6576**	643.3324			1267.6470	634.3271	F	**1311.6579**	656.3326	1294.6314	647.8193	1293.6474	647.3273	11
13	**1413.7161**	**707.3617**	1396.6896	**698.8484**	1395.7056	**698.3564**	Q	**1164.5895**	**582.7984**	1147.5630	**574.2851**	1146.5790	**573.7931**	10
14	**1512.7845**	**756.8959**	1495.7580	**748.3826**	1494.7740	**747.8906**	V	**1036.5310**	**518.7691**	1019.5044	510.2558	1018.5204	509.7638	9
15	**1641.8271**	821.4172	1624.8006	812.9039	1623.8166	812.4119	E	**937.4625**	**469.2349**	920.4360	460.7216	919.4520	460.2296	8
16	1804.8905	**902.9489**	1787.8639	**894.4356**	1786.8799	**893.9436**	Y	**808.4199**	404.7136	791.3934	396.2003	790.4094	395.7083	7
17	1875.9276	**938.4674**	**1858.9010**	929.9542	1857.9170	929.4621	A	**645.3566**	323.1819	628.3301	314.6687	627.3461	314.1767	6
18	2003.9862	**1002.4967**	1986.9596	993.9834	1985.9756	993.4914	Q	**574.3195**	287.6634	**557.2930**	279.1501	**556.3089**	278.6581	5
19	2133.0287	**1067.0180**	2116.0022	1058.5047	2115.0182	1058.0127	E	**446.2609**	223.6341	429.2344	215.1208	**428.2504**	214.6288	4
20	2204.0659	**1102.5366**	2187.0393	1094.0233	2186.0553	1093.5313	A	**317.2183**	159.1128	300.1918	150.5995			3
21	2303.1343	**1152.0708**	2286.1077	**1143.5575**	2285.1237	**1143.0655**	V	**246.1812**	123.5942	229.1547	115.0810			2
22							K	147.1128	74.0600	130.0863	65.5468			1

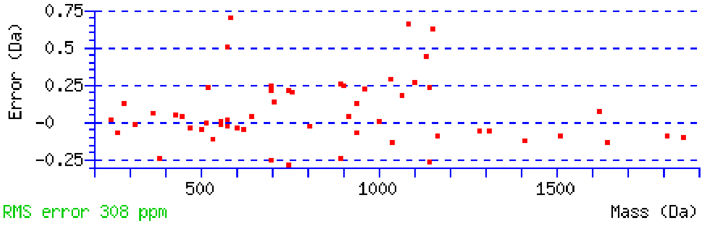

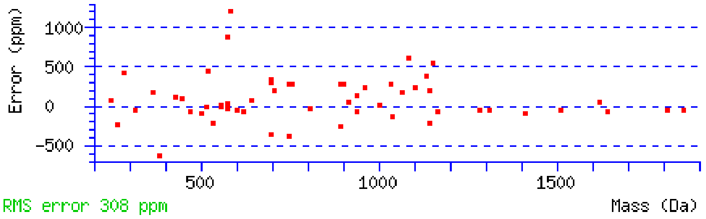

All matches to this query

Score	Mr(calc):	Delta	Sequence

57.9	2448.2325	0.0184	AITVFSPDGHLFQVEYAQEAVK
5.0	2448.2301	0.0208	QLEVVHTLDGKEYITPAQISK
4.3	2447.2492	1.0017	HFPGRGADPALGSLPAAGLSGLCAR
4.1	2448.2298	0.0211	YVNGHAKKHYEDAQVPLTNHK
4.1	2448.2301	0.0208	QLEVVHTLDGKEYITPAQISK
2.5	2448.2318	0.0191	LAAEEQFQALVKQMNQTLQDK
1.9	2446.2523	1.9986	FHHLVSPFVGQQVVKTGGSSKK
1.9	2446.2523	1.9986	FHHLVSPFVGQQVVKTGGSSKK
1.6	2448.2279	0.0230	VCSKATSPTSQRGQEVISTPTSK
1.6	2446.2509	2.0000	ELGIETYKVNVSERLVQYVK

Spectrum No: 2; Query: 4771; Rank: 1

Peptide View

MS/MS Fragmentation of **ALLEVVQSGGK**
Found in **IPI00024175**, Tax_Id=9606 Gene_Symbol=PSMA7 Isoform 1 of Proteasome subunit alpha type-7

Match to Query 4771: 1099.628208 from(550.821380,2+)
Title: 091224LimSK_Exosome3_06.2231.2231.2.dta
Data file C:\Temp\Mascoti\LSK_2D\091224LimSK_Exosome3_01_2D.mgf

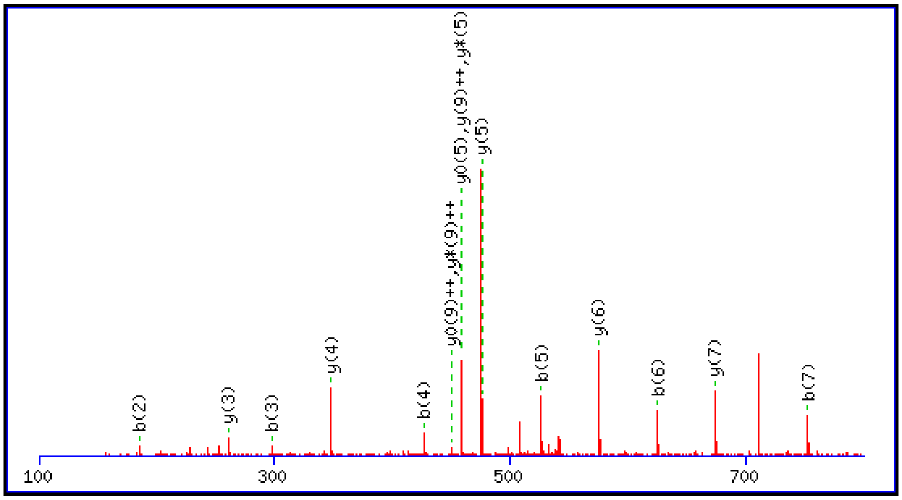

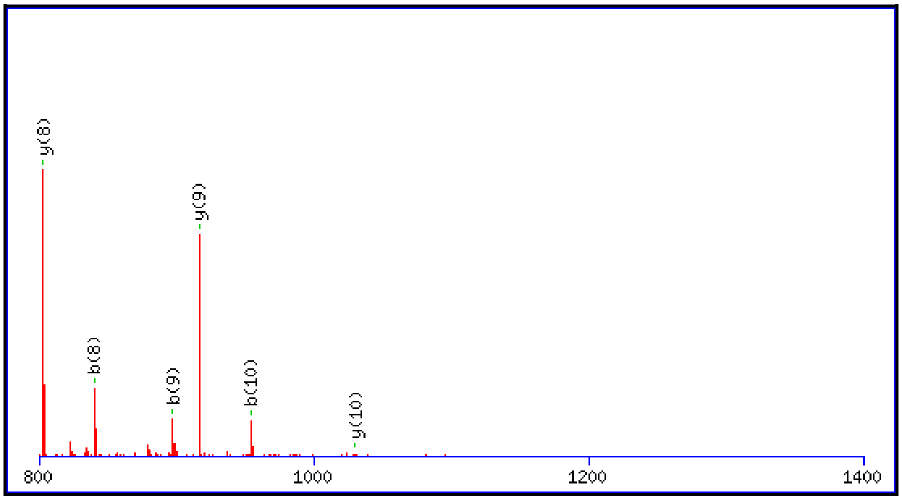

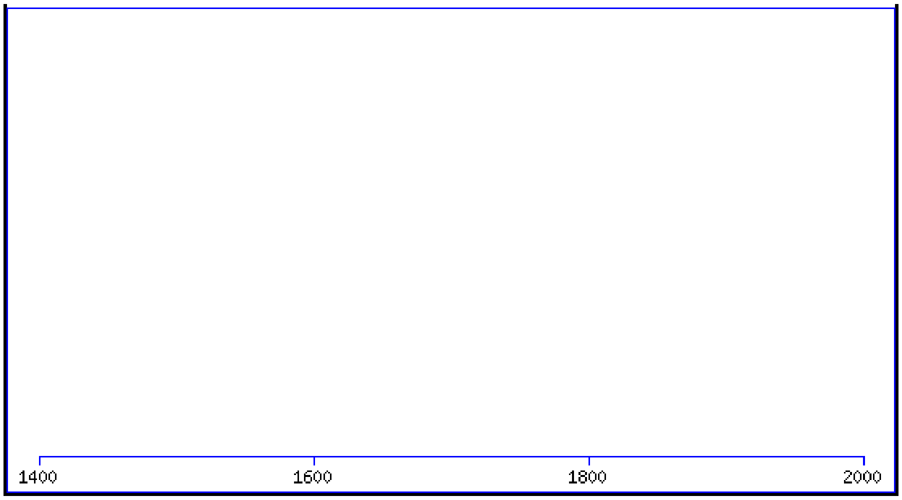

Monoisotopic mass of neutral peptide Mr(calc): 1099.6237
Fixed modifications: Carbamidomethyl (C)
Ions Score: 59 **Expect:** 0.00013
Matches (Bold Red): 22/96 fragment ions using 40 most intense peaks

#	b	b^{++}	b*	b^{*++}	b^0	b^{0++}	Seq.	y	y^{++}	y*	y^{*++}	y^0	y^{0++}	#
1	72.0444	36.5258					A							11
2	**185.1285**	93.0679					L	**1029.5939**	515.3006	1012.5673	506.7873	1011.5833	506.2953	10
3	**298.2125**	149.6099					L	**916.5098**	**458.7585**	899.4833	**450.2453**	898.4993	**449.7533**	9
4	**427.2551**	214.1312			409.2445	205.1259	E	**803.4258**	402.2165	786.3992	393.7032	785.4152	393.2112	8
5	**526.3235**	263.6654			508.3130	254.6601	V	**674.3832**	337.6952	657.3566	329.1819	656.3726	328.6899	7
6	**625.3919**	313.1996			607.3814	304.1943	V	**575.3148**	288.1610	558.2882	279.6477	557.3042	279.1557	6
7	**753.4505**	377.2289	736.4240	368.7156	735.4400	368.2236	Q	**476.2463**	238.6268	**459.2198**	230.1135	**458.2358**	229.6215	5
8	**840.4825**	420.7449	823.4560	412.2316	822.4720	411.7396	S	**348.1878**	174.5975	331.1612	166.0842	330.1772	165.5922	4
9	**897.5040**	449.2556	880.4775	440.7424	879.4934	440.2504	G	**261.1557**	131.0815	244.1292	122.5682			3
10	**954.5255**	477.7664	937.4989	469.2531	936.5149	468.7611	G	204.1343	102.5708	187.1077	94.0575			2
11							K	147.1128	74.0600	130.0863	65.5468			1

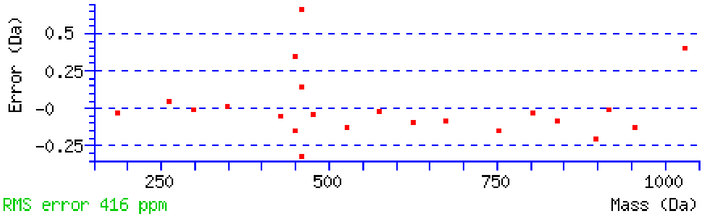

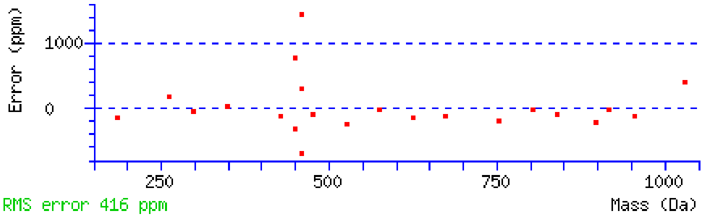

All matches to this query

Score	Mr(calc):	Delta	Sequence
58.6	1099.6237	0.0045	ALLEVVQSGGK
11.3	1099.6349	-0.0067	LALEKERNK
4.1	1099.6237	0.0045	ISQVLGNEIK
3.0	1099.6349	-0.0067	NLKNKIENK
3.0	1099.6349	-0.0067	NLKNKLENK
1.8	1099.6390	-0.0108	NIQGLFAPLK
1.6	1098.6145	1.0137	SKTASKHVNK
1.3	1099.6237	0.0045	AIPKTLKDSQ

Spectrum No: 3; Query: 2207; Rank: 1

Peptide View

MS/MS Fragmentation of **DIVVLGVEK**
Found in **IPI00024175**, Tax_Id=9606 Gene_Symbol=PSMA7 Isoform 1 of Proteasome subunit alpha type-7

Match to Query 2207: 970.574128 from(486.294340,2+)
Title: 091224LimSK_Exosome3_06.2990.2990.2.dta
Data file C:\Temp\Mascoti\LSK_2D\091224LimSK_Exosome3_01_2D.mgf

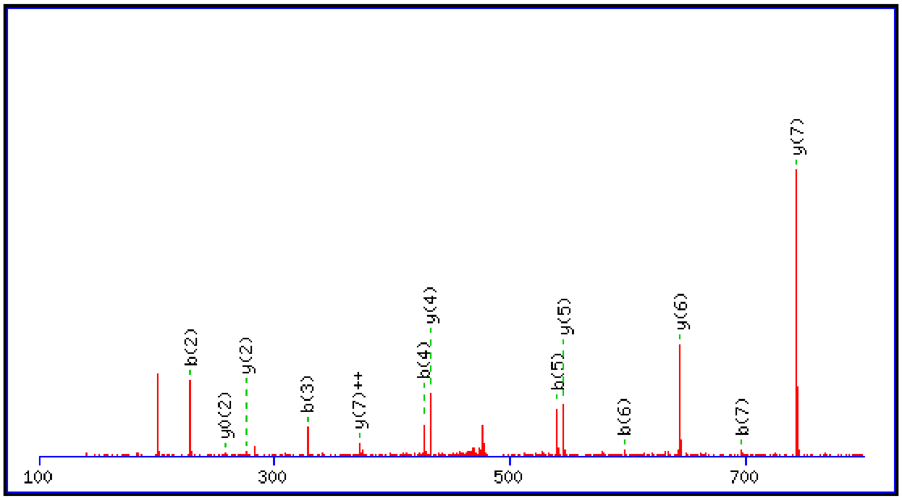

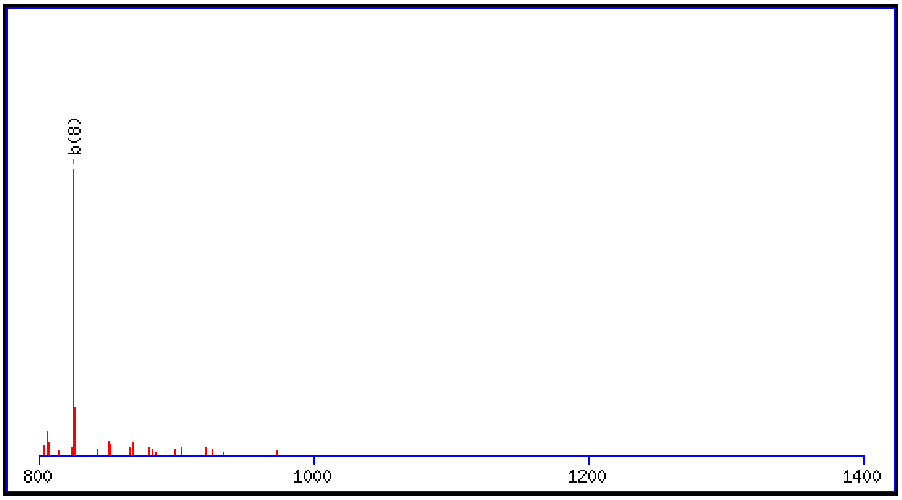

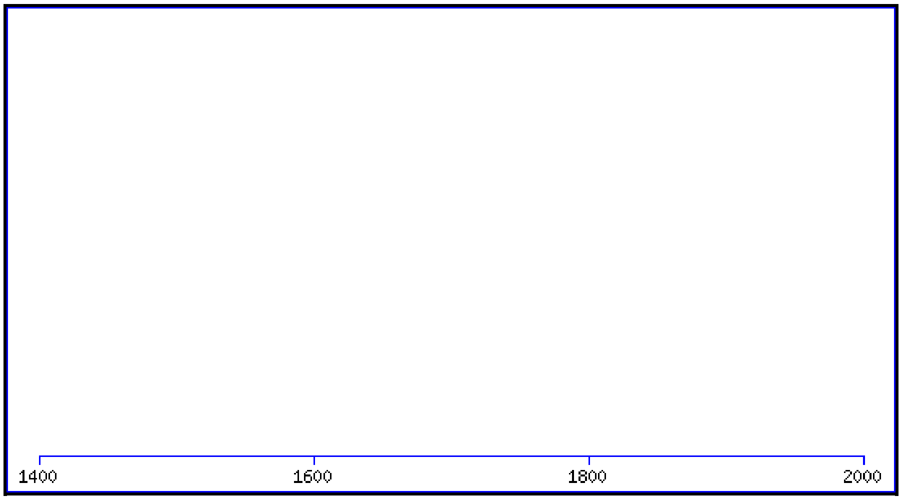

Monoisotopic mass of neutral peptide Mr(calc): 970.5699
Fixed modifications: Carbamidomethyl (C)
Ions Score: 44 **Expect:** 0.0028
Matches (Bold Red): 14/78 fragment ions using 35 most intense peaks

#	b	b^{++}	b^{0}	b^{0++}	Seq.	y	y^{++}	y^{*}	y^{*++}	y^{0}	y^{0++}	#
1	116.0342	58.5207	98.0237	49.5155	D							9
2	**229.1183**	115.0628	211.1077	106.0575	I	856.5502	428.7788	839.5237	420.2655	838.5397	419.7735	8
3	**328.1867**	164.5970	310.1761	155.5917	V	**743.4662**	**372.2367**	726.4396	363.7234	725.4556	363.2314	7
4	**427.2551**	214.1312	409.2445	205.1259	V	**644.3978**	322.7025	627.3712	314.1892	626.3872	313.6972	6
5	**540.3392**	270.6732	522.3286	261.6679	L	**545.3293**	273.1683	528.3028	264.6550	527.3188	264.1630	5
6	**597.3606**	299.1840	579.3501	290.1787	G	**432.2453**	216.6263	415.2187	208.1130	414.2347	207.6210	4
7	**696.4291**	348.7182	678.4185	339.7129	V	375.2238	188.1155	358.1973	179.6023	357.2132	179.1103	3
8	**825.4716**	413.2395	807.4611	404.2342	E	**276.1554**	138.5813	259.1288	130.0681	**258.1448**	129.5761	2
9					K	147.1128	74.0600	130.0863	65.5468			1

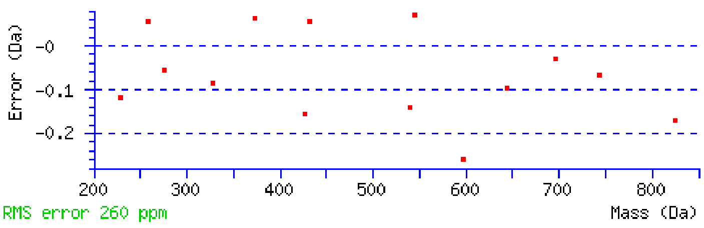

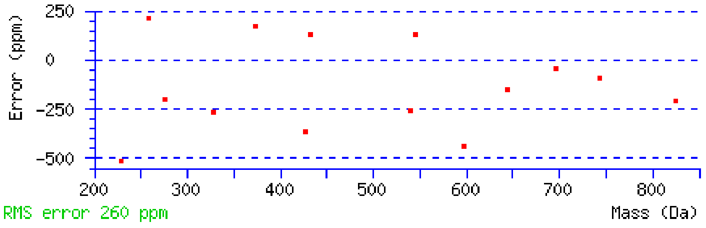

All matches to this query

Score	Mr(calc):	Delta	Sequence
43.8	970.5699	0.0042	DIVVLGVEK
10.1	970.5699	0.0043	IAELLGLDK
8.0	970.5672	0.0069	RNVGIREK
7.7	970.5811	-0.0070	SLLLLEQR
7.2	970.5699	0.0042	IDVGDVILK
5.8	970.5811	-0.0070	ITLLVQER
5.5	970.5811	-0.0070	DVVILGRSL
5.3	969.5720	1.0022	QVVIGGRNK
4.1	970.5699	0.0043	LDLQILEK
2.6	969.5719	1.0022	ARVENIIR

Spectrum No: 4; Query: 7382; Rank: 1

Peptide View

MS/MS Fragmentation of **GRDIVVLGVEK**
Found in **IPI00024175**, Tax_Id=9606 Gene_Symbol=PSMA7 Isoform 1 of Proteasome subunit alpha type-7

Match to Query 7382: 1183.698248 from(592.856400,2+)
Title: 091224LimSK_Exosome3_06.1747.1747.2.dta
Data file C:\Temp\Mascoti\LSK_2D\091224LimSK_Exosome3_01_2D.mgf

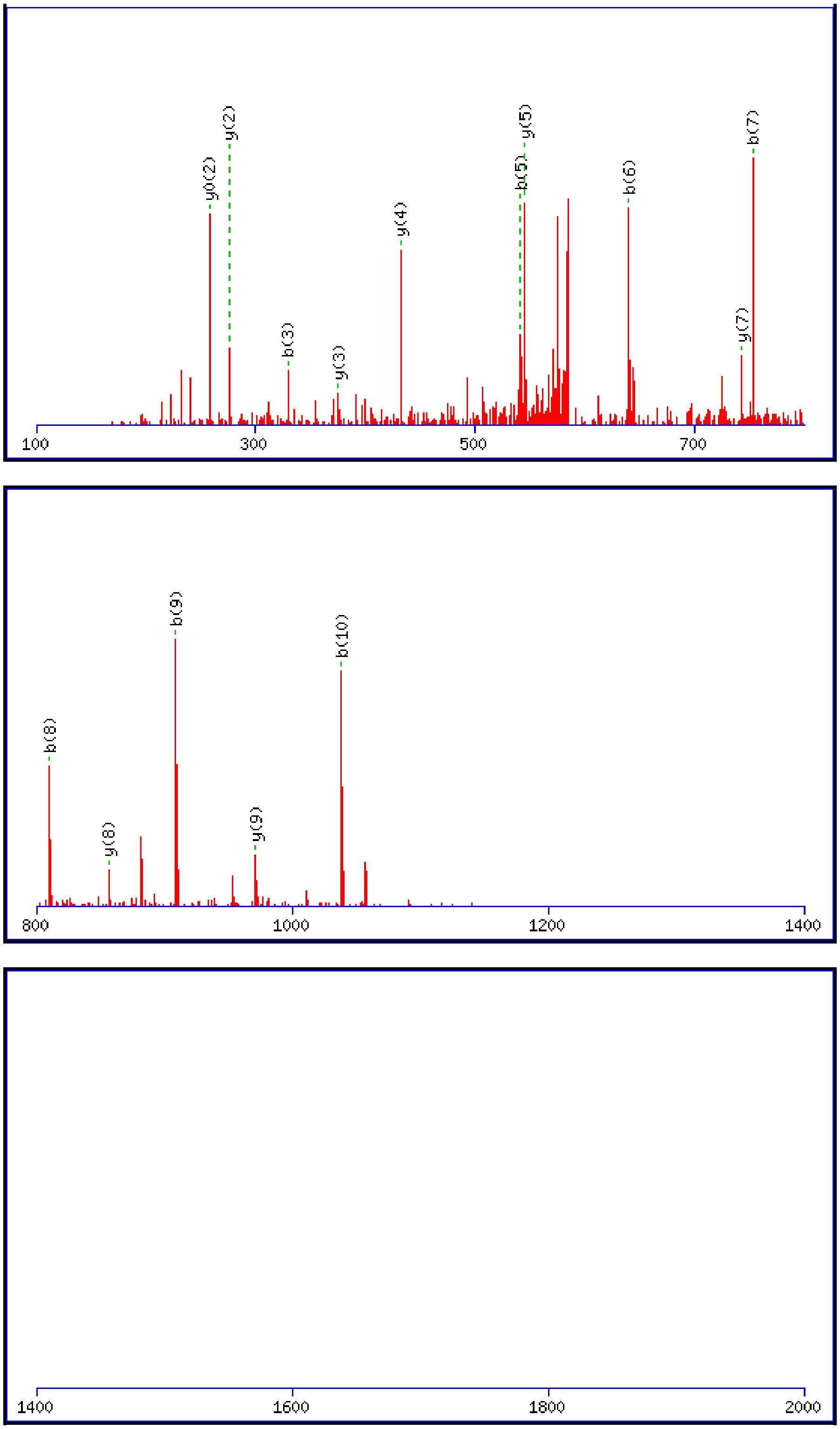

Monoisotopic mass of neutral peptide Mr(calc): 1183.6925
Fixed modifications: Carbamidomethyl (C)
Ions Score: 52 **Expect:** 0.00033
Matches (Bold Red): 15/112 fragment ions using 36 most intense peaks

#	b	b^{++}	b*	b^{*++}	b^0	b^{0++}	Seq.	y	y^{++}	y*	y^{*++}	y^0	y^{0++}	#
1	58.0287	29.5180					G							11
2	214.1299	107.5686	197.1033	99.0553			R	1127.6783	564.3428	1110.6517	555.8295	1109.6677	555.3375	10
3	**329.1568**	165.0820	312.1302	156.5688	311.1462	156.0768	D	**971.5772**	486.2922	954.5506	477.7790	953.5666	477.2869	9
4	442.2409	221.6241	425.2143	213.1108	424.2303	212.6188	I	**856.5502**	428.7788	839.5237	420.2655	838.5397	419.7735	8
5	**541.3093**	271.1583	524.2827	262.6450	523.2987	262.1530	V	**743.4662**	372.2367	726.4396	363.7234	725.4556	363.2314	7
6	**640.3777**	320.6925	623.3511	312.1792	622.3671	311.6872	V	644.3978	322.7025	627.3712	314.1892	626.3872	313.6972	6

7	**753.4618**	377.2345	736.4352	368.7212	735.4512	368.2292	**L**	**545.3293**	273.1683	528.3028	264.6550	527.3188	264.1630	5
8	**810.4832**	405.7452	793.4567	397.2320	792.4726	396.7400	**G**	**432.2453**	216.6263	415.2187	208.1130	414.2347	207.6210	4
9	**909.5516**	455.2795	892.5251	446.7662	891.5411	446.2742	**V**	**375.2238**	188.1155	358.1973	179.6023	357.2132	179.1103	3
10	**1038.5942**	519.8007	1021.5677	511.2875	1020.5837	510.7955	**E**	**276.1554**	138.5813	259.1288	130.0681	**258.1448**	129.5761	2
11							**K**	147.1128	74.0600	130.0863	65.5468			1

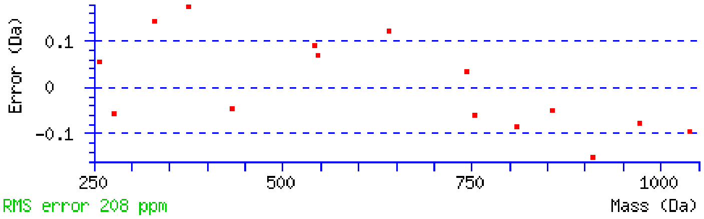

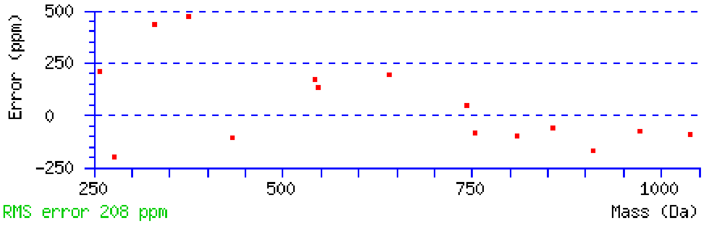

All matches to this query

Score	Mr(calc):	Delta	Sequence
51.9	1183.6925	0.0058	GRDIVVLGVEK
14.4	1183.7037	-0.0054	GNLKQVRIEK
12.0	1181.6992	1.9990	QQRKPLREK
11.8	1183.6924	0.0058	AEKQKPEVKK
4.7	1183.7037	-0.0054	RKALDLLNNK
4.6	1183.6965	0.0018	KEVVKPPYPK
3.3	1183.7037	-0.0054	LNTSIPQKRK
2.9	1183.7037	-0.0054	VLKNQDLARK
2.6	1183.6924	0.0058	GREILELVQK
2.5	1183.7037	-0.0054	NNTLVLQVRK

Spectrum No: 5; Query: 46207; Rank: 1

Peptide View

MS/MS Fragmentation of **ICALDDNVCMAFAGLTADAR**
Found in **IPI00024175**, Tax_Id=9606 Gene_Symbol=PSMA7 Isoform 1 of Proteasome subunit alpha type-7

Match to Query 46207: 2182.997328 from(1092.505940,2+)
Title: 091224LimSK_Exosome3_06.7435.7435.2.dta
Data file C:\Temp\Mascoti\LSK_2D\091224LimSK_Exosome3_01_2D.mgf

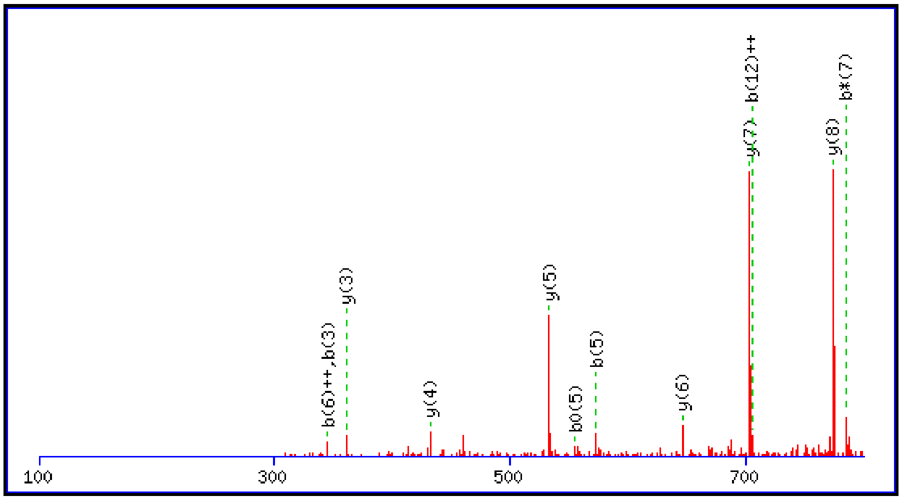

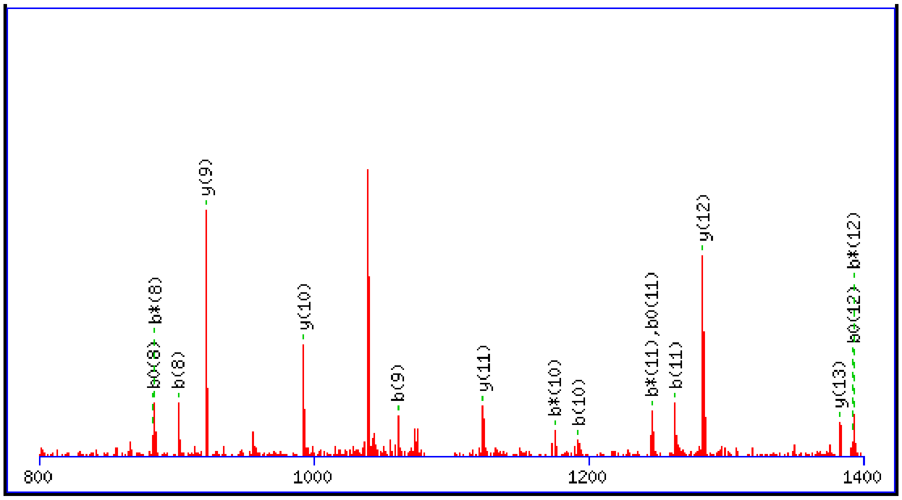

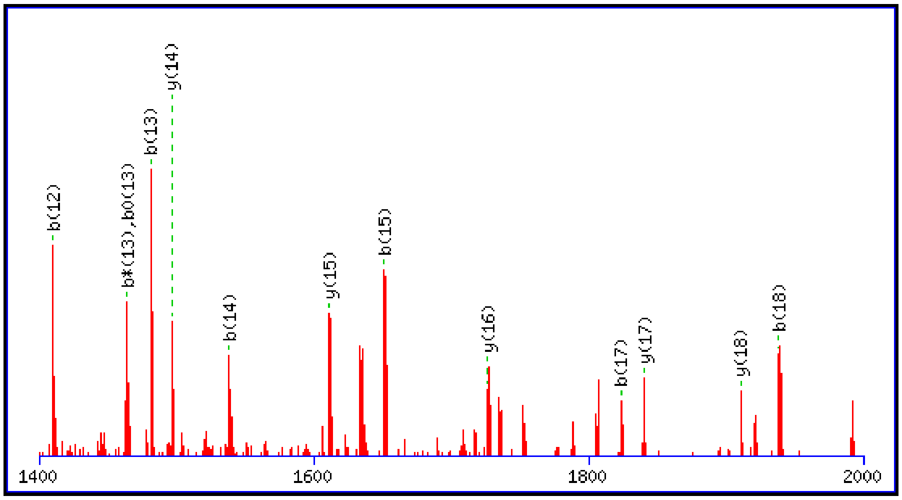

Monoisotopic mass of neutral peptide Mr(calc): 2182.9809
Fixed modifications: Carbamidomethyl (C)
Ions Score: 103 **Expect:** 1.5e-008
Matches (Bold Red): 41/204 fragment ions using 74 most intense peaks

#	b	b^{++}	b*	b^{*++}	b^0	b^{0++}	Seq.	y	y^{++}	y*	y^{*++}	y^0	y^{0++}	#
1	114.0913	57.5493					I							20
2	274.1220	137.5646					C	2070.9042	1035.9557	2053.8776	1027.4424	2052.8936	1026.9504	19
3	**345.1591**	173.0832					A	**1910.8735**	955.9404	1893.8470	947.4271	1892.8629	946.9351	18
4	458.2432	229.6252					L	**1839.8364**	920.4218	1822.8098	911.9086	1821.8258	911.4166	17
5	**573.2701**	287.1387			**555.2595**	278.1334	D	**1726.7523**	863.8798	1709.7258	855.3665	1708.7418	854.8745	16
6	688.2971	**344.6522**			670.2865	335.6469	D	**1611.7254**	806.3663	1594.6988	797.8531	1593.7148	797.3611	15
7	802.3400	401.6736	**785.3134**	393.1604	784.3294	392.6683	N	**1496.6984**	748.8529	1479.6719	740.3396	1478.6879	739.8476	14
8	**901.4084**	451.2078	**884.3818**	442.6946	**883.3978**	442.2026	V	**1382.6555**	691.8314	1365.6290	683.3181	1364.6450	682.8261	13
9	**1061.4390**	531.2232	1044.4125	522.7099	1043.4285	522.2179	C	**1283.5871**	642.2972	1266.5606	633.7839	1265.5765	633.2919	12
10	**1192.4795**	596.7434	**1175.4530**	588.2301	1174.4690	587.7381	M	**1123.5565**	562.2819	1106.5299	553.7686	1105.5459	553.2766	11
11	**1263.5166**	632.2620	**1246.4901**	623.7487	**1245.5061**	623.2567	A	**992.5160**	496.7616	975.4894	488.2483	974.5054	487.7563	10
12	**1410.5851**	**705.7962**	**1393.5585**	697.2829	**1392.5745**	696.7909	F	**921.4789**	461.2431	904.4523	452.7298	903.4683	452.2378	9
13	**1481.6222**	741.3147	**1464.5956**	732.8014	**1463.6116**	732.3094	A	**774.4104**	387.7089	757.3839	379.1956	756.3999	378.7036	8
14	**1538.6436**	769.8255	1521.6171	761.3122	1520.6331	760.8202	G	**703.3733**	352.1903	686.3468	343.6770	685.3628	343.1850	7
15	**1651.7277**	826.3675	1634.7011	817.8542	1633.7171	817.3622	L	**646.3519**	323.6796	629.3253	315.1663	628.3413	314.6743	6
16	1752.7754	876.8913	1735.7488	868.3781	1734.7648	867.8860	T	**533.2678**	267.1375	516.2413	258.6243	515.2572	258.1323	5
17	**1823.8125**	912.4099	1806.7859	903.8966	1805.8019	903.4046	A	**432.2201**	216.6137	415.1936	208.1004	414.2096	207.6084	4
18	**1938.8394**	969.9234	1921.8129	961.4101	1920.8289	960.9181	D	**361.1830**	181.0951	344.1565	172.5819	343.1724	172.0899	3
19	2009.8765	1005.4419	1992.8500	996.9286	1991.8660	996.4366	A	246.1561	123.5817	229.1295	115.0684			2

**20**							**R**	175.1190	88.0631	158.0924	79.5498			**1**

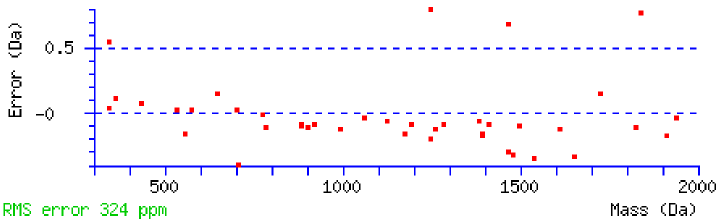

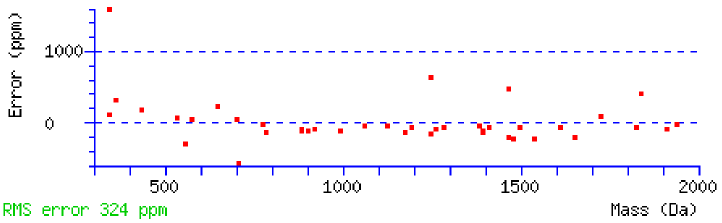

All matches to this query

Score	Mr(calc):	Delta	Sequence
102.8	2182.9809	0.0164	ICALDDNVCMAFAGLTADAR
9.8	2180.9830	2.0144	YGMVAQVTQTLKLEDTPK
7.8	2181.9742	1.0232	ITSEMGSASQANIRLTSLK
7.8	2181.9742	1.0232	ITSEMGSASQANIRLTSLK
7.8	2181.9742	1.0232	ITSEMGSASQANIRLTSLK
7.8	2181.9742	1.0232	ITSEMGSASQANIRLTSLK
7.2	2183.0154	-0.0181	GVNTERSSKLGSNAGNKPCK
6.5	2180.9830	2.0144	YGMVAQVTQTLKLEDTPK
5.4	2181.9782	1.0191	EIKSVMNTAGNSAPSLFLK
4.9	2183.0023	-0.0050	SRFKGMYYLHPGTLDYR

Spectrum No: 6; Query: 4381; Rank: 1

Peptide View

MS/MS Fragmentation of **ILNPEEIEK**
Found in **IPI00024175**, Tax_Id=9606 Gene_Symbol=PSMA7 Isoform 1 of Proteasome subunit alpha type-7

Match to Query 4381: 1083.585948 from(542.800250,2+)
Title: 091224LimSK_Exosome3_06.1889.1889.2.dta
Data file C:\Temp\Mascoti\LSK_2D\091224LimSK_Exosome3_01_2D.mgf

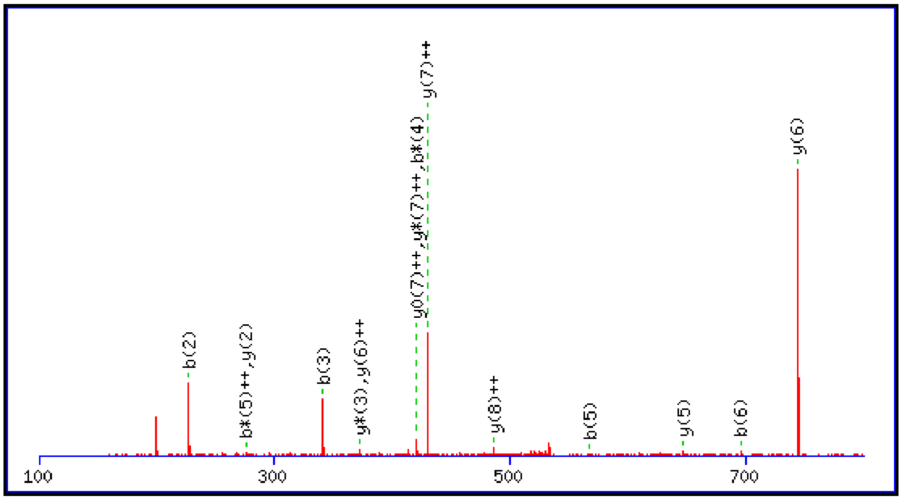

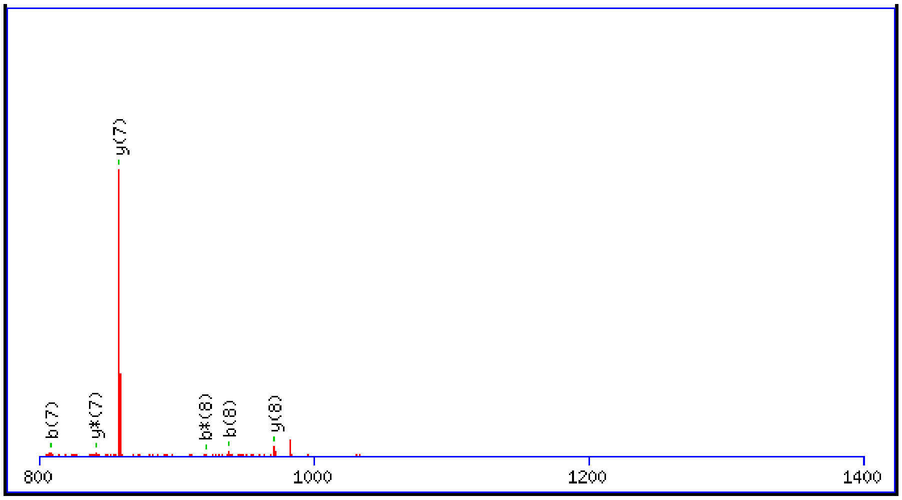

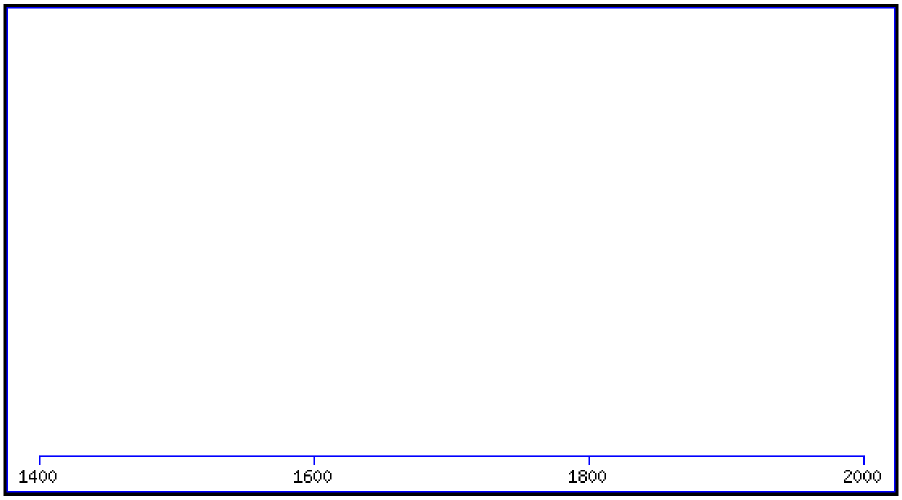

Monoisotopic mass of neutral peptide Mr(calc): 1083.5811
Fixed modifications: Carbamidomethyl (C)
Ions Score: 40 **Expect:** 0.0095
Matches (Bold Red): 21/82 fragment ions using 36 most intense peaks

#	b	b^{++}	b*	b^{*++}	b^0	b^{0++}	Seq.	y	y^{++}	y*	y^{*++}	y^0	y^{0++}	#
1	114.0913	57.5493					I							9
2	**227.1754**	114.0913					L	**971.5044**	**486.2558**	954.4779	477.7426	953.4938	477.2506	8
3	**341.2183**	171.1128	324.1918	162.5995			N	**858.4203**	**429.7138**	**841.3938**	**421.2005**	840.4098	**420.7085**	7
4	438.2711	219.6392	**421.2445**	211.1259			P	**744.3774**	**372.6923**	727.3509	364.1791	726.3668	363.6871	6
5	**567.3137**	284.1605	550.2871	**275.6472**	549.3031	275.1552	E	**647.3246**	324.1660	630.2981	315.6527	629.3141	315.1607	5
6	**696.3563**	348.6818	679.3297	340.1685	678.3457	339.6765	E	518.2821	259.6447	501.2555	251.1314	500.2715	250.6394	4
7	**809.4403**	405.2238	792.4138	396.7105	791.4298	396.2185	I	389.2395	195.1234	**372.2129**	186.6101	371.2289	186.1181	3
8	**938.4829**	469.7451	**921.4564**	461.2318	920.4724	460.7398	E	**276.1554**	138.5813	259.1288	130.0681	258.1448	129.5761	2
9							K	147.1128	74.0600	130.0863	65.5468			1

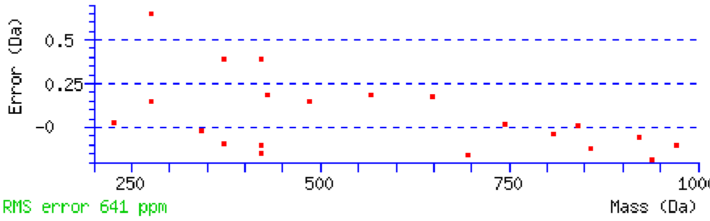

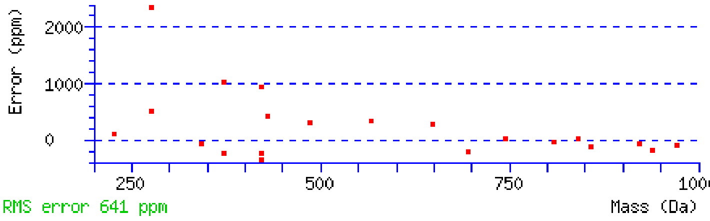

All matches to this query

Score	Mr(calc):	Delta	Sequence

39.8	1083.5811	0.0048	ILNPEEIEK
7.7	1083.5763	0.0097	LLATKAMLK
6.9	1083.5954	-0.0094	ILHKKHTK
6.2	1083.5841	0.0018	ILQLYARK
4.0	1082.5923	0.9937	KILGKMVSK
2.6	1083.5859	0.0001	LLHSSQMLR
2.2	1083.5924	-0.0064	NIQPKVEEK
1.8	1082.5760	1.0099	LLIFGSYDR
0.5	1083.5812	0.0048	EPAVLELEGK
0.4	1083.5924	-0.0064	EPKLNENIK

Spectrum No: 7; Query: 51343; Rank: 1

Peptide View

MS/MS Fragmentation of **KICALDDNVCMAFAGLTADAR**
Found in **IPI00024175**, Tax_Id=9606 Gene_Symbol=PSMA7 Isoform 1 of Proteasome subunit alpha type-7

Match to Query 51343: 2311.094622 from(771.372150,3+)
Title: 091224LimSK_Exosome3_06.6940.6940.3.dta
Data file C:\Temp\Mascoti\LSK_2D\091224LimSK_Exosome3_01_2D.mgf

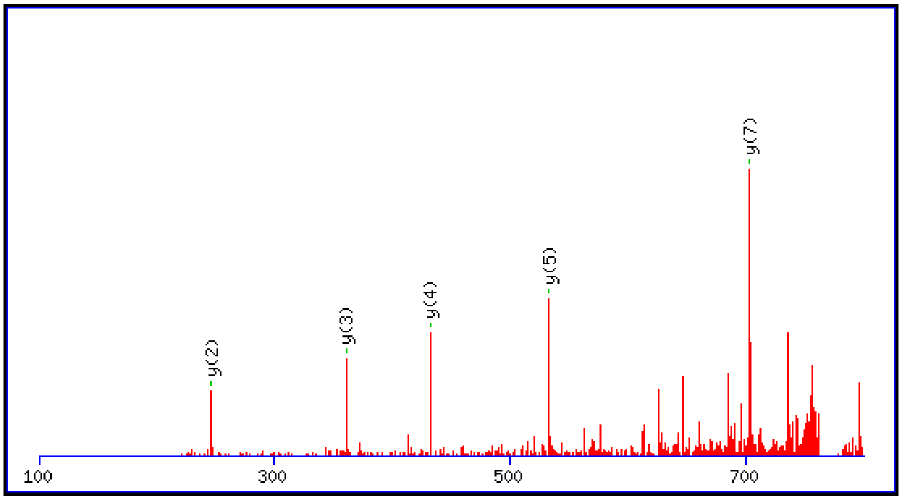

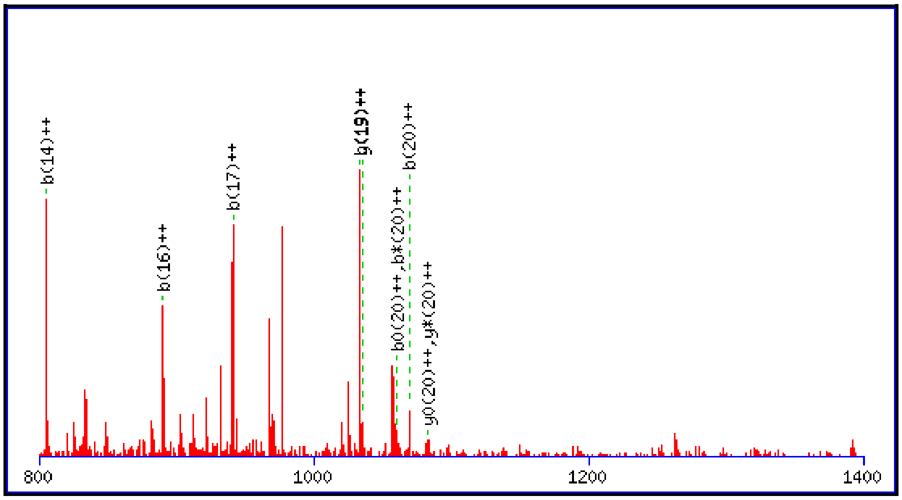

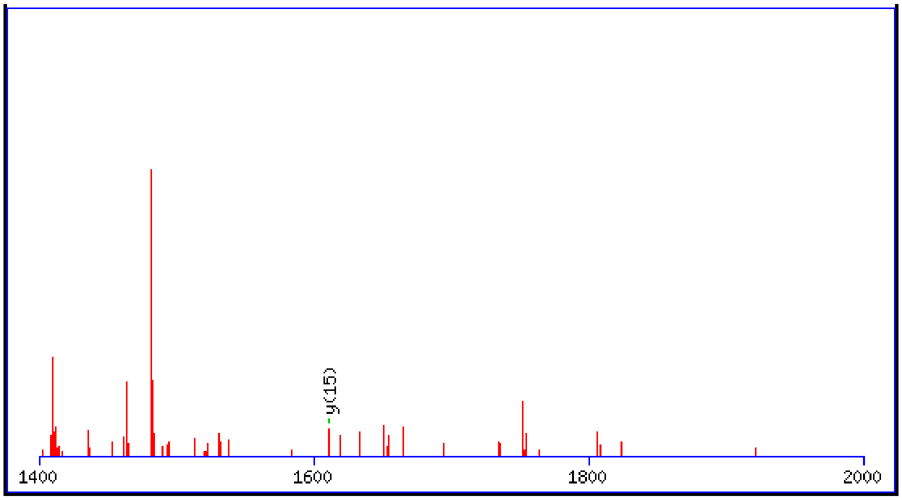

Monoisotopic mass of neutral peptide Mr(calc): 2311.0759
Fixed modifications: Carbamidomethyl (C)
Ions Score: 29 **Expect:** 0.33
Matches (Bold Red): 16/226 fragment ions using 36 most intense peaks

#	b	b^{++}	b*	b^{*++}	b^{0}	b^{0++}	Seq.	y	y^{++}	y*	y^{*++}	y^{0}	y^{0++}	#
1	129.1022	65.0548	112.0757	56.5415			K							21
2	242.1863	121.5968	225.1598	113.0835			I	2183.9882	1092.4977	2166.9617	**1083.9845**	2165.9777	**1083.4925**	20
3	402.2170	201.6121	385.1904	193.0988			C	2070.9042	**1035.9557**	2053.8776	1027.4424	2052.8936	1026.9504	19
4	473.2541	237.1307	456.2275	228.6174			A	1910.8735	955.9404	1893.8470	947.4271	1892.8629	946.9351	18
5	586.3381	293.6727	569.3116	285.1594			L	1839.8364	920.4218	1822.8098	911.9086	1821.8258	911.4166	17
6	701.3651	351.1862	684.3385	342.6729	683.3545	342.1809	D	1726.7523	863.8798	1709.7258	855.3665	1708.7418	854.8745	16
7	816.3920	408.6996	799.3655	400.1864	798.3815	399.6944	D	**1611.7254**	806.3663	1594.6988	797.8531	1593.7148	797.3611	15
8	930.4349	465.7211	913.4084	457.2078	912.4244	456.7158	N	1496.6984	748.8529	1479.6719	740.3396	1478.6879	739.8476	14
9	1029.5034	515.2553	1012.4768	506.7420	1011.4928	506.2500	V	1382.6555	691.8314	1365.6290	683.3181	1364.6450	682.8261	13
10	1189.5340	595.2706	1172.5075	586.7574	1171.5234	586.2654	C	1283.5871	642.2972	1266.5606	633.7839	1265.5765	633.2919	12
11	1320.5745	660.7909	1303.5479	652.2776	1302.5639	651.7856	M	1123.5565	562.2819	1106.5299	553.7686	1105.5459	553.2766	11
12	1391.6116	696.3094	1374.5851	687.7962	1373.6010	687.3042	A	992.5160	496.7616	975.4894	488.2483	974.5054	487.7563	10
13	1538.6800	769.8436	1521.6535	761.3304	1520.6695	760.8384	F	921.4789	461.2431	904.4523	452.7298	903.4683	452.2378	9
14	1609.7171	**805.3622**	1592.6906	796.8489	1591.7066	796.3569	A	774.4104	387.7089	757.3839	379.1956	756.3999	378.7036	8
15	1666.7386	833.8729	1649.7120	825.3597	1648.7280	824.8677	G	**703.3733**	352.1903	686.3468	343.6770	685.3628	343.1850	7
16	1779.8227	**890.4150**	1762.7961	881.9017	1761.8121	881.4097	L	646.3519	323.6796	629.3253	315.1663	628.3413	314.6743	6
17	1880.8703	**940.9388**	1863.8438	932.4255	1862.8598	931.9335	T	**533.2678**	267.1375	516.2413	258.6243	515.2572	258.1323	5
18	1951.9075	976.4574	1934.8809	967.9441	1933.8969	967.4521	A	**432.2201**	216.6137	415.1936	208.1004	414.2096	207.6084	4
19	2066.9344	**1033.9708**	2049.9078	1025.4576	2048.9238	1024.9656	D	**361.1830**	181.0951	344.1565	172.5819	343.1724	172.0899	3
20	2137.9715	**1069.4894**	2120.9450	**1060.9761**	2119.9609	**1060.4841**	A	**246.1561**	123.5817	229.1295	115.0684			2
21							R	175.1190	88.0631	158.0924	79.5498			1

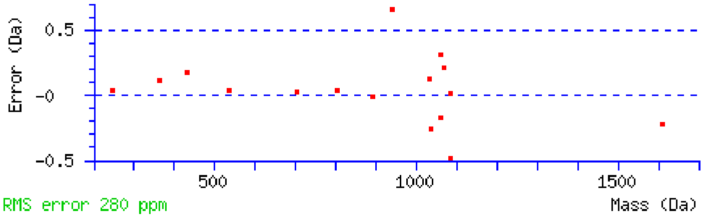

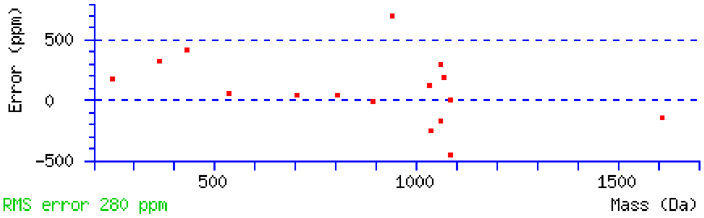

All matches to this query

Score	Mr(calc):	Delta	Sequence
29.1	2311.0759	0.0187	KICALDDNVCMAFAGLTADAR

17.7	2311.0959	-0.0013	KRFMLKPYESFEELTGEK
9.2	2311.1032	-0.0086	MGWTGGGLGKSGEGIREPISVK
8.3	2309.0876	2.0071	SGGCFPGSATVHLEQGGTKLVK
7.5	2311.1147	-0.0201	MAAKMEITLSSNTEASSKQER
5.0	2309.0859	2.0087	GNAGDCLANPAVPWGQGVEIER
4.9	2310.1079	0.9867	SRLFVGNLPPDITEEEMRK
4.5	2310.0947	1.0000	RQHGQLWFPEGFKVSEASK
4.2	2310.1039	0.9907	EELARDKCVVIGLQSTGEAR
4.0	2311.1038	-0.0092	QKQSGFFLENKSTYQLWK

Spectrum No: 8; Query: 27223; Rank: 1

Peptide View

MS/MS Fragmentation of **LTVEDPVTVEYITR**
Found in **IPI00024175**, Tax_Id=9606 Gene_Symbol=PSMA7 Isoform 1 of Proteasome subunit alpha type-7

Match to Query 27223: 1633.866328 from(817.940440,2+)
Title: 091224LimSK_Exosome3_07.5092.5092.2.dta
Data file C:\Temp\Mascoti\LSK_2D\091224LimSK_Exosome3_01_2D.mgf

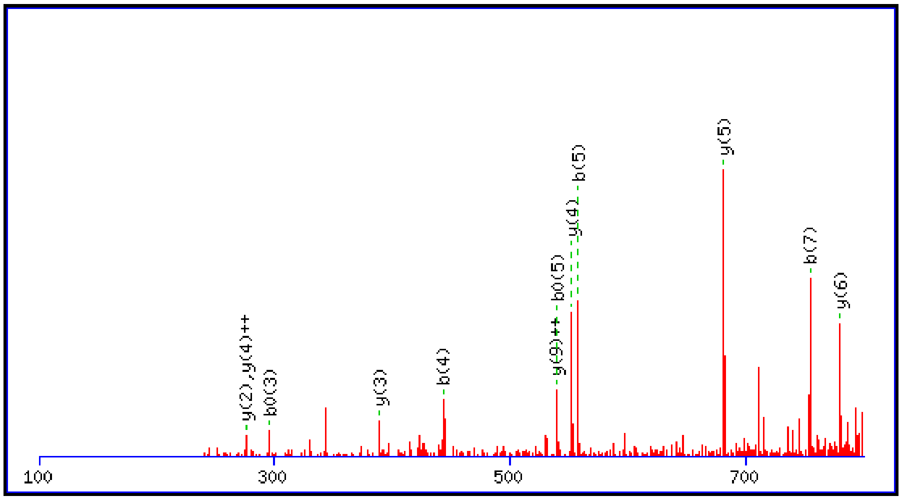

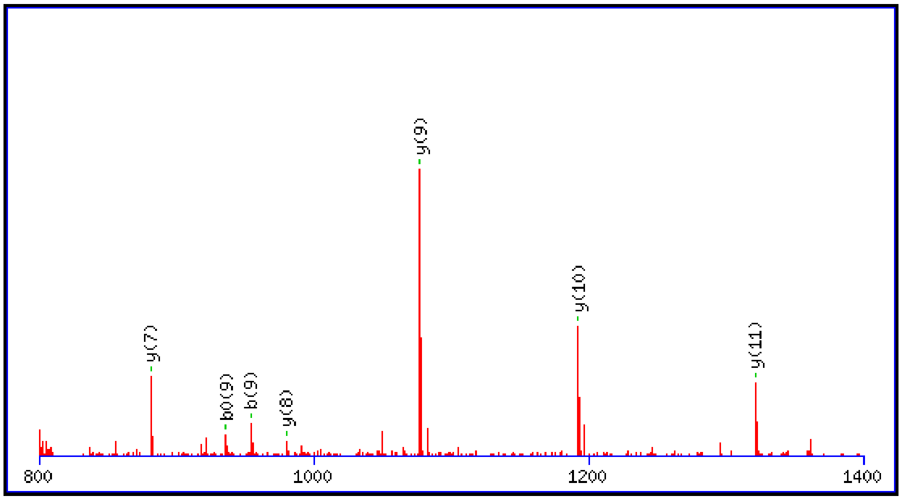

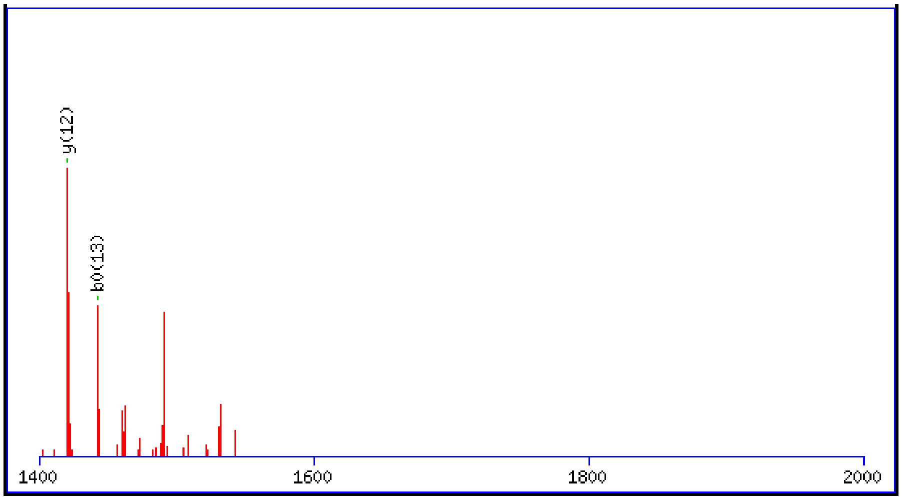

Monoisotopic mass of neutral peptide Mr(calc): 1633.8563
Fixed modifications: Carbamidomethyl (C)
Ions Score: 91 **Expect:** 9.7e-008
Matches (Bold Red): 21/126 fragment ions using 32 most intense peaks

#	b	b^{++}	b^{0}	b^{0++}	Seq.	y	y^{++}	y*	y^{*++}	y^{0}	y^{0++}	#
1	114.0913	57.5493			L							14
2	215.1390	108.0731	197.1285	99.0679	T	1521.7795	761.3934	1504.7530	752.8801	1503.7690	752.3881	13
3	314.2074	157.6074	**296.1969**	148.6021	V	**1420.7318**	710.8696	1403.7053	702.3563	1402.7213	701.8643	12
4	**443.2500**	222.1287	425.2395	213.1234	E	**1321.6634**	661.3353	1304.6369	652.8221	1303.6529	652.3301	11
5	**558.2770**	279.6421	**540.2664**	270.6368	D	**1192.6208**	596.8141	1175.5943	588.3008	1174.6103	587.8088	10
6	655.3297	328.1685	637.3192	319.1632	P	**1077.5939**	**539.3006**	1060.5673	530.7873	1059.5833	530.2953	9
7	**754.3981**	377.7027	736.3876	368.6974	V	**980.5411**	490.7742	963.5146	482.2609	962.5306	481.7689	8
8	855.4458	428.2266	837.4353	419.2213	T	**881.4727**	441.2400	864.4462	432.7267	863.4621	432.2347	7
9	**954.5142**	477.7608	**936.5037**	468.7555	V	**780.4250**	390.7162	763.3985	382.2029	762.4145	381.7109	6
10	1083.5568	542.2821	1065.5463	533.2768	E	**681.3566**	341.1819	664.3301	332.6687	663.3461	332.1767	5
11	1246.6202	623.8137	1228.6096	614.8084	Y	**552.3140**	**276.6607**	535.2875	268.1474	534.3035	267.6554	4
12	1359.7042	680.3558	1341.6937	671.3505	I	**389.2507**	195.1290	372.2241	186.6157	371.2401	186.1237	3
13	1460.7519	730.8796	**1442.7413**	721.8743	T	**276.1666**	138.5870	259.1401	130.0737	258.1561	129.5817	2
14					R	175.1190	88.0631	158.0924	79.5498			1

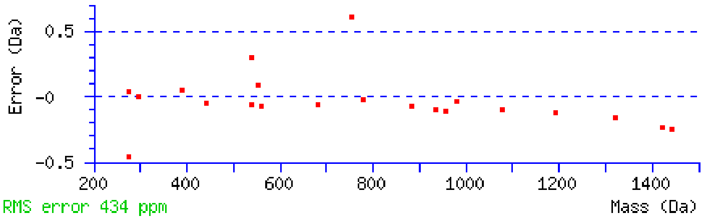

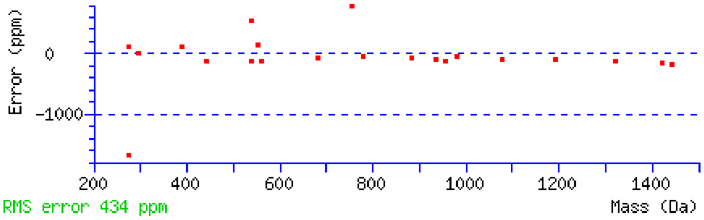

All matches to this query

Score	Mr(calc):	Delta	Sequence
91.3	1633.8563	0.0100	LTVEDPVTVEYITR
8.8	1632.8501	1.0162	KFLPSTANRGLPPR
8.8	1632.8501	1.0162	KFLPSTANRGLPPR
8.6	1632.8528	1.0135	TLNINTKYYVLVI
8.6	1632.8528	1.0135	TLNINTKYYVLVI
5.2	1632.8528	1.0135	TLNINTKYYVLVI
2.7	1631.8743	1.9920	RGGAEGSPKLAVYATR
2.3	1633.8665	-0.0002	LQIVNNTRKVDVR

2.3	1631.8661	2.0002	QVRFRLTPPSPVR
1.8	1631.8631	2.0032	VLETLVTVAEEHHR

Spectrum No: 9; Query: 28501; Rank: 1

Peptide View

MS/MS Fragmentation of **LYQTDPSGTYHAWK**
Found in **IPI00024175**, Tax_Id=9606 Gene_Symbol=PSMA7 Isoform 1 of Proteasome subunit alpha type-7

Match to Query 28501: 1665.789888 from(833.902220,2+)
Title: 091224LimSK_Exosome3_05.1898.1898.2.dta
Data file C:\Temp\Mascoti\LSK_2D\091224LimSK_Exosome3_01_2D.mgf

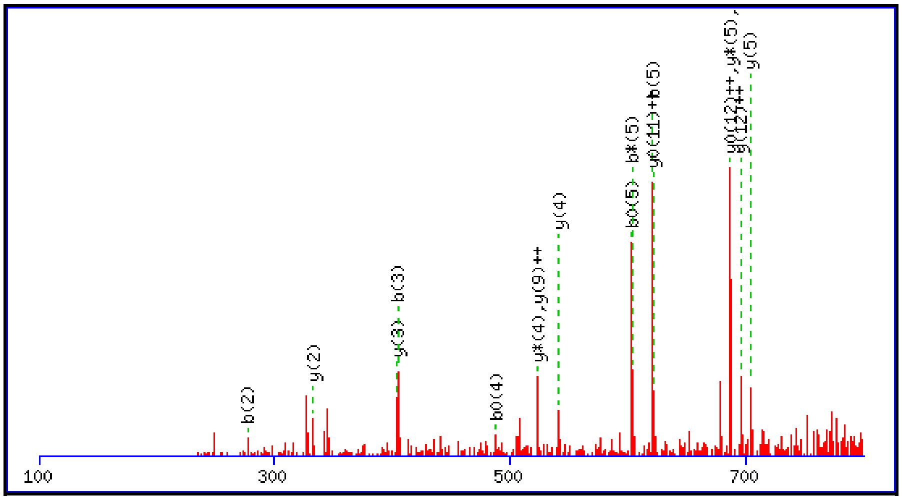

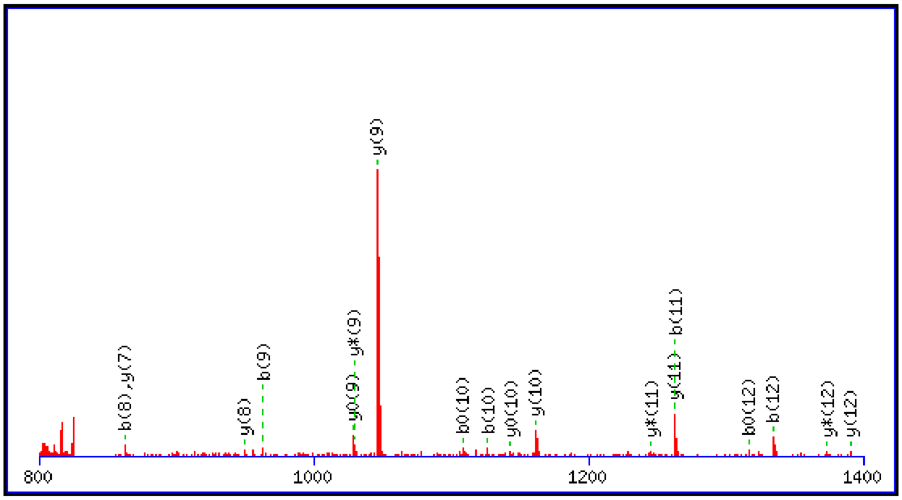

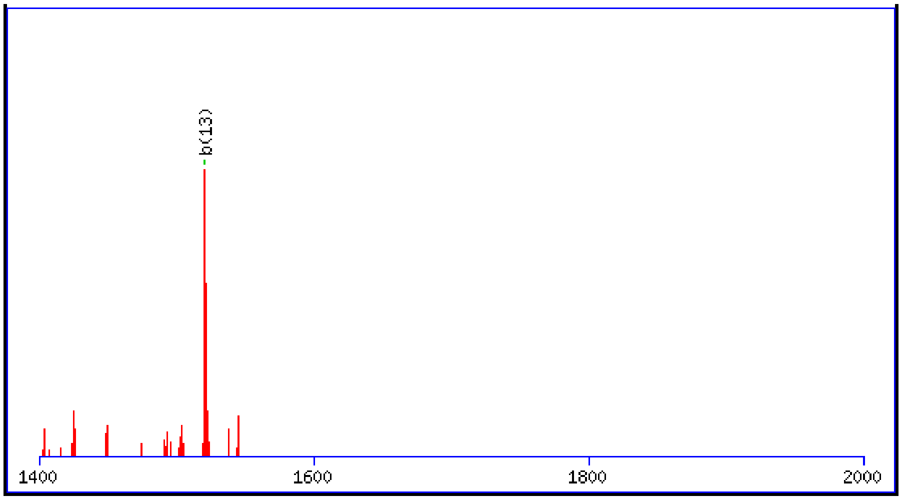

Monoisotopic mass of neutral peptide Mr(calc): 1665.7787
Fixed modifications: Carbamidomethyl (C)
Ions Score: 54 **Expect:** 0.00087
Matches (Bold Red): 37/136 fragment ions using 71 most intense peaks

#	b	b^{++}	b*	b^{*++}	b^0	b^{0++}	Seq.	y	y^{++}	y*	y^{*++}	y^0	y^{0++}	#
1	114.0913	57.5493					L							14
2	**277.1547**	139.0810					Y	1553.7019	777.3546	1536.6754	768.8413	1535.6914	768.3493	13
3	**405.2132**	203.1103	388.1867	194.5970			Q	**1390.6386**	**695.8229**	**1373.6121**	**687.3097**	1372.6280	**686.8177**	12
4	506.2609	253.6341	489.2344	245.1208	**488.2504**	244.6288	T	**1262.5800**	631.7937	**1245.5535**	623.2804	1244.5695	**622.7884**	11
5	**621.2879**	311.1476	**604.2613**	302.6343	**603.2773**	302.1423	D	**1161.5324**	581.2698	1144.5058	572.7565	**1143.5218**	572.2645	10
6	718.3406	359.6740	701.3141	351.1607	700.3301	350.6687	P	**1046.5054**	**523.7563**	**1029.4789**	515.2431	**1028.4948**	514.7511	9
7	805.3727	403.1900	788.3461	394.6767	787.3621	394.1847	S	**949.4526**	475.2300	932.4261	466.7167	931.4421	466.2247	8
8	**862.3941**	431.7007	845.3676	423.1874	844.3836	422.6954	G	**862.4206**	431.7139	845.3941	423.2007	844.4100	422.7087	7
9	**963.4418**	482.2245	946.4153	473.7113	945.4312	473.2193	T	805.3992	403.2032	788.3726	394.6899	787.3886	394.1979	6
10	**1126.5051**	563.7562	1109.4786	555.2429	**1108.4946**	554.7509	Y	**704.3515**	352.6794	**687.3249**	344.1661			5
11	**1263.5640**	632.2857	1246.5375	623.7724	**1245.5535**	623.2804	H	**541.2881**	271.1477	**524.2616**	262.6344			4
12	**1334.6012**	667.8042	1317.5746	659.2909	**1316.5906**	658.7989	A	**404.2292**	202.6183	387.2027	194.1050			3
13	**1520.6805**	760.8439	1503.6539	752.3306	1502.6699	751.8386	W	**333.1921**	167.0997	316.1656	158.5864			2
14							K	147.1128	74.0600	130.0863	65.5468			1

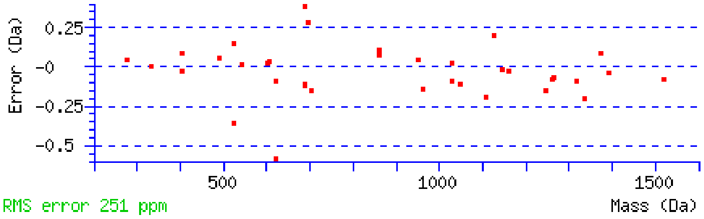

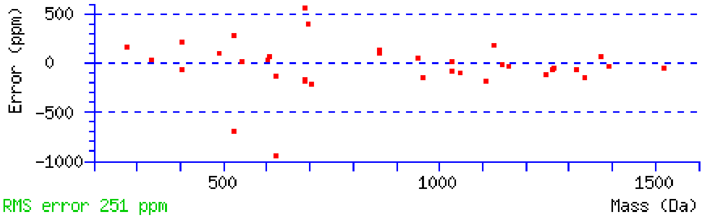

All matches to this query

Score	Mr(calc):	Delta	Sequence
53.9	1665.7787	0.0112	LYQTDPSGTYHAWK
10.0	1665.7954	-0.0055	GSSVEMLQDVIDVMK
9.8	1665.7780	0.0118	QQMEERFVASNPSK
8.5	1665.7854	0.0045	NMVYILTITTPLK
8.5	1665.7854	0.0045	NMVYILTITTPLK
6.2	1665.7767	0.0132	MEIDDLASNVETVSK
5.2	1665.7892	0.0007	GASVIIRFVTNTTK
4.5	1664.7716	1.0183	MQNNIADPEELFTK

4.3	1664.7885	1.0014	VDIPPPFDLACITK
3.9	1665.7780	0.0119	GLIQQFTTITEKK

Spectrum No: 10; Query: 1875; Rank: 1

Peptide View

MS/MS Fragmentation of **NIELAVMR**
Found in **IPI00024175**, Tax_Id=9606 Gene_Symbol=PSMA7 Isoform 1 of Proteasome subunit alpha type-7

Match to Query 1875: 944.515768 from(473.265160,2+)
Title: 091224LimSK_Exosome3_06.2979.2979.2.dta
Data file C:\Temp\Mascoti\LSK_2D\091224LimSK_Exosome3_01_2D.mgf

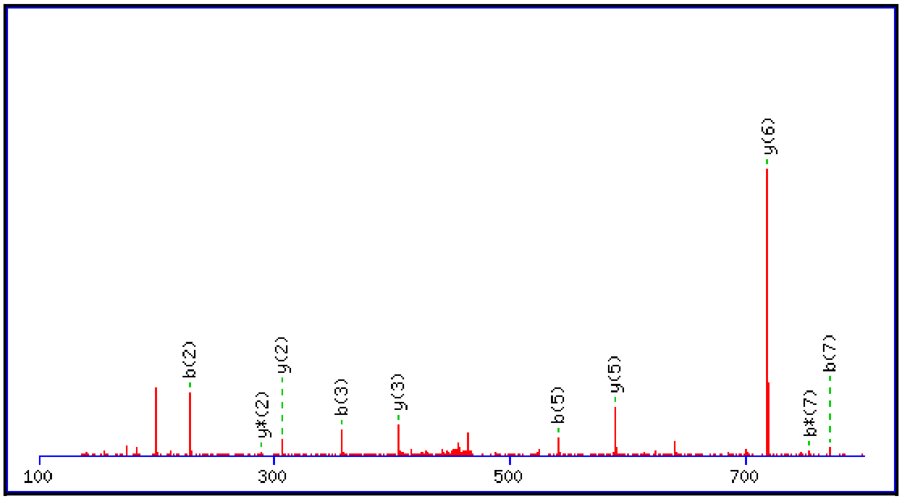

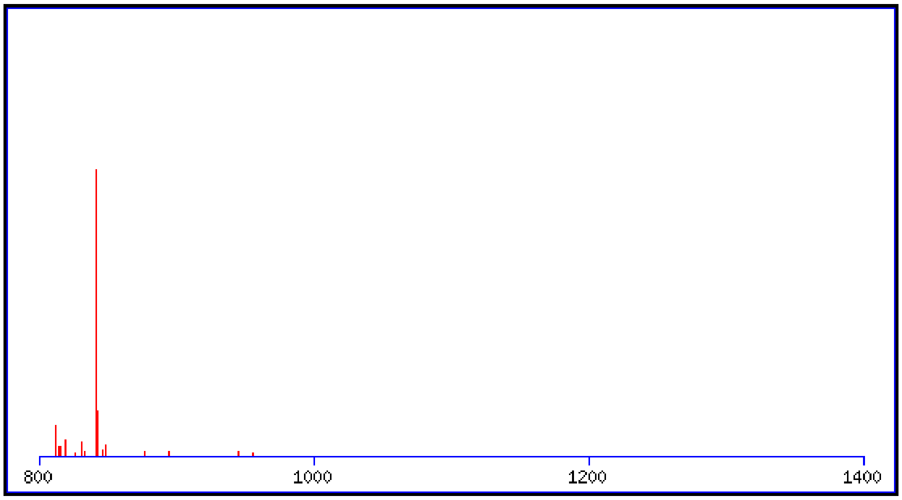

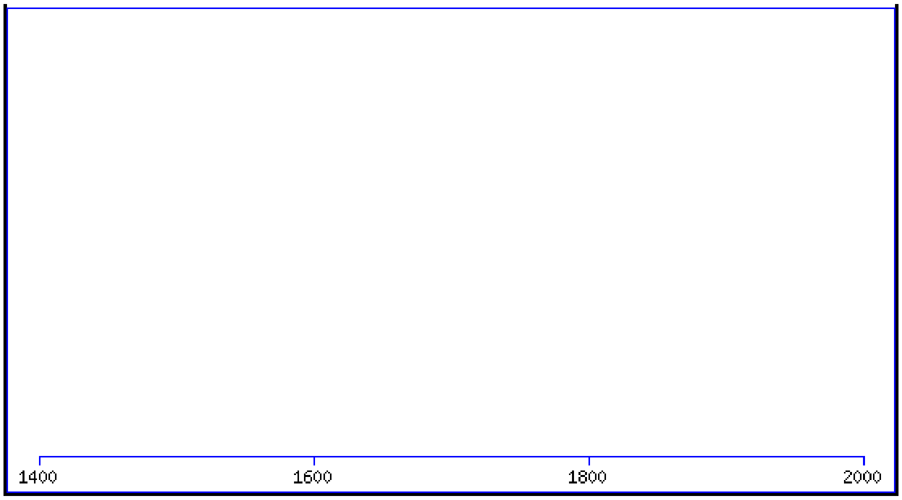

Monoisotopic mass of neutral peptide Mr(calc): 944.5113
Fixed modifications: Carbamidomethyl (C)
Ions Score: 33 **Expect:** 0.063
Matches (Bold Red): 10/70 fragment ions using 17 most intense peaks

#	b	b^{++}	b*	b^{*++}	b^{0}	b^{0++}	Seq.	y	y^{++}	y*	y^{*++}	y^{0}	y^{0++}	#
1	115.0502	58.0287	98.0237	49.5155			N							8
2	**228.1343**	114.5708	211.1077	106.0575			I	831.4757	416.2415	814.4491	407.7282	813.4651	407.2362	7
3	**357.1769**	179.0921	340.1503	170.5788	339.1663	170.0868	E	**718.3916**	359.6994	701.3651	351.1862	700.3811	350.6942	6
4	470.2609	235.6341	453.2344	227.1208	452.2504	226.6288	L	**589.3490**	295.1782	572.3225	286.6649			5
5	**541.2980**	271.1527	524.2715	262.6394	523.2875	262.1474	A	476.2650	238.6361	459.2384	230.1228			4
6	640.3665	320.6869	623.3399	312.1736	622.3559	311.6816	V	**405.2279**	203.1176	388.2013	194.6043			3
7	**771.4069**	386.2071	**754.3804**	377.6938	753.3964	377.2018	M	**306.1594**	153.5834	**289.1329**	145.0701			2
8							R	175.1190	88.0631	158.0924	79.5498			1

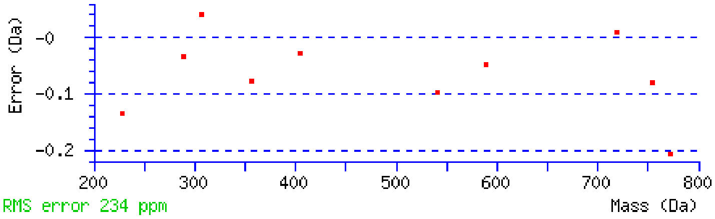

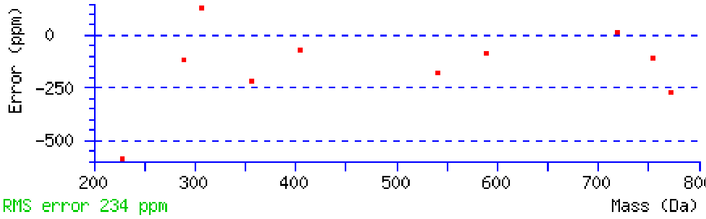

All matches to this query

Score	Mr(calc):	Delta	Sequence
33.2	944.5113	0.0045	NIELAVMR
12.0	944.5151	0.0006	LNARAASSR
8.2	943.5087	1.0071	LLQTGQER
7.1	943.5087	1.0071	EPTKIGSGR
7.1	943.5087	1.0071	EPTKVASGR
5.3	944.5152	0.0006	SSPTRKNR
4.5	944.5179	-0.0021	ITIXPDTSK
4.2	944.5152	0.0006	SSSPGAKRR
4.1	942.5134	2.0023	DGLLGAELR
3.8	943.5100	1.0057	GRQGGRWK

Spectrum No: 11; Query: 31171; Rank: 1

Peptide View

MS/MS Fragmentation of **NYTDEAIETDDLTIK**
Found in **IPI00024175**, Tax_Id=9606 Gene_Symbol=PSMA7 Isoform 1 of Proteasome subunit alpha type-7

Match to Query 31171: 1739.820948 from(870.917750,2+)
Title: 091224LimSK_Exosome3_06.4135.4135.2.dta
Data file C:\Temp\Mascoti\LSK_2D\091224LimSK_Exosome3_01_2D.mgf

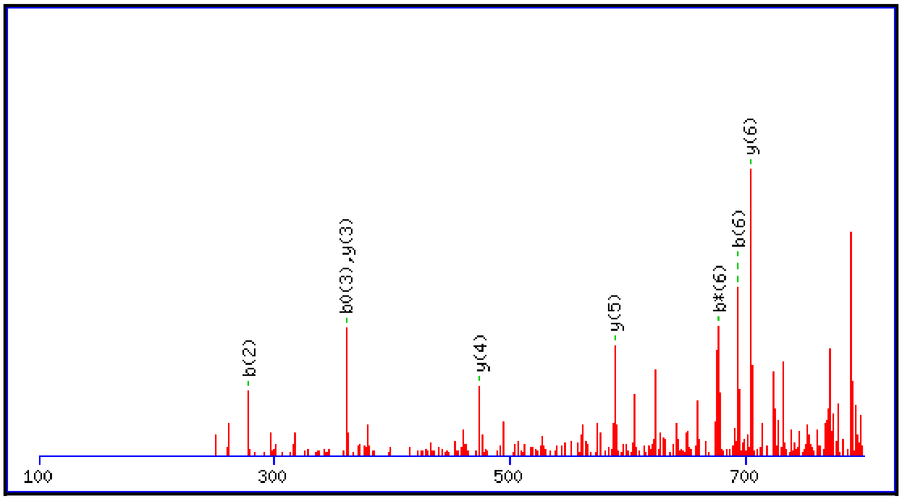

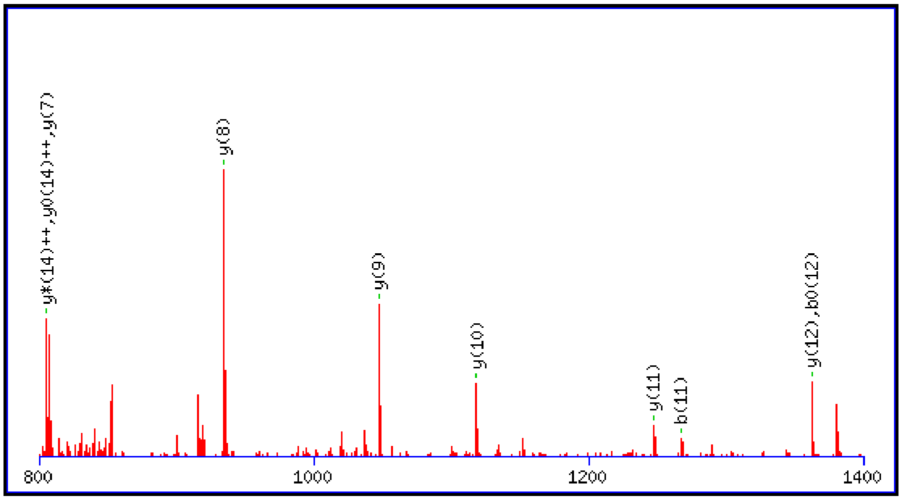

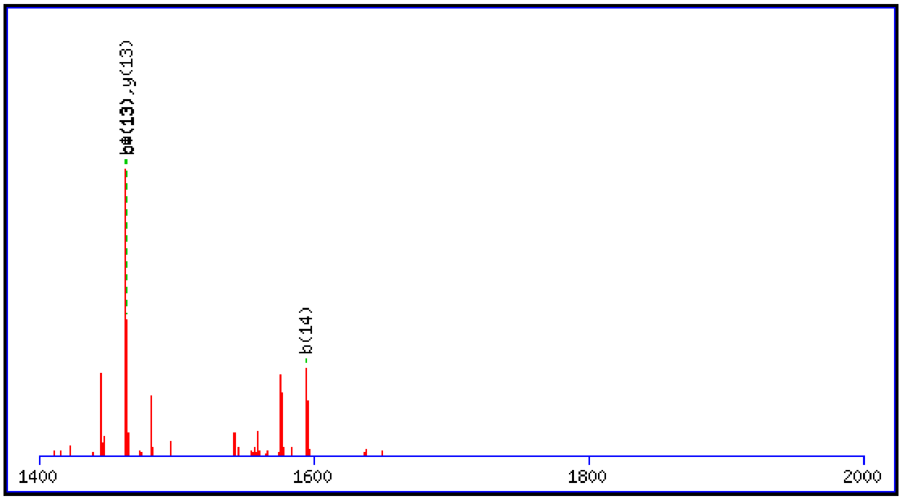

Monoisotopic mass of neutral peptide Mr(calc): 1739.8101
Fixed modifications: Carbamidomethyl (C)
Ions Score: 119 **Expect:** 2.7e-010
Matches (Bold Red): 22/160 fragment ions using 18 most intense peaks

		++		++	0	0++			++		++	0	0++	

#	b	b	b*	b*	b	b	Seq.	y	y	y*	y*	y	y	#
1	115.0502	58.0287	98.0237	49.5155			N							15
2	**278.1135**	139.5604	261.0870	131.0471			Y	1626.7745	813.8909	1609.7479	**805.3776**	1608.7639	**804.8856**	14
3	379.1612	190.0842	362.1347	181.5710	**361.1506**	181.0790	T	**1463.7112**	732.3592	1446.6846	723.8459	1445.7006	723.3539	13
4	494.1882	247.5977	477.1616	239.0844	476.1776	238.5924	D	**1362.6635**	681.8354	1345.6369	673.3221	1344.6529	672.8301	12
5	623.2307	312.1190	606.2042	303.6057	605.2202	303.1137	E	**1247.6365**	624.3219	1230.6100	615.8086	1229.6260	615.3166	11
6	**694.2679**	347.6376	**677.2413**	339.1243	676.2573	338.6323	A	**1118.5939**	559.8006	1101.5674	551.2873	1100.5834	550.7953	10
7	807.3519	404.1796	790.3254	395.6663	789.3414	395.1743	I	**1047.5568**	524.2821	1030.5303	515.7688	1029.5463	515.2768	9
8	936.3945	468.7009	919.3680	460.1876	918.3840	459.6956	E	**934.4728**	467.7400	917.4462	459.2267	916.4622	458.7347	8
9	1037.4422	519.2247	1020.4156	510.7115	1019.4316	510.2195	T	**805.4302**	403.2187	788.4036	394.7055	787.4196	394.2134	7
10	1152.4691	576.7382	1135.4426	568.2249	1134.4586	567.7329	D	**704.3825**	352.6949	687.3559	344.1816	686.3719	343.6896	6
11	**1267.4961**	634.2517	1250.4695	625.7384	1249.4855	625.2464	D	**589.3556**	295.1814	572.3290	286.6681	571.3450	286.1761	5
12	1380.5801	690.7937	1363.5536	682.2804	**1362.5696**	681.7884	L	**474.3286**	237.6679	457.3021	229.1547	456.3180	228.6627	4
13	1481.6278	741.3176	**1464.6013**	732.8043	**1463.6173**	732.3123	T	**361.2445**	181.1259	344.2180	172.6126	343.2340	172.1206	3
14	**1594.7119**	797.8596	1577.6853	789.3463	1576.7013	788.8543	I	260.1969	130.6021	243.1703	122.0888			2
15							K	147.1128	74.0600	130.0863	65.5468			1

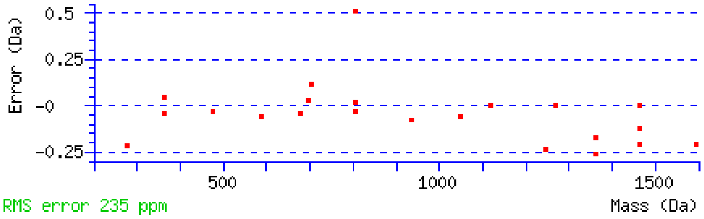

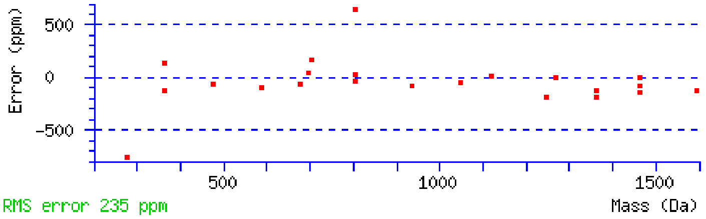

All matches to this query

Score	Mr(calc):	Delta	Sequence
119.1	1739.8101	0.0108	NYTDEAIETDDLTIK
9.7	1739.8222	-0.0013	MKQYASPMPTQTDVK
7.9	1739.8066	0.0144	DEGHRVLIFSQMTK
7.2	1739.8335	-0.0125	CNSVKDGAICYKPTK
7.0	1739.8379	-0.0170	FERNFIAQGPYENR
6.3	1739.8284	-0.0074	FKNSTYSKPNSFLK
6.3	1739.8284	-0.0074	FKNSTYSKPNSFLK
6.3	1738.8010	1.0200	KQSTQFSGGPTEDTEK
6.0	1739.8288	-0.0078	TLFEVMATKPETDDK
6.0	1739.8317	-0.0108	YQMPTGIKGPLPNTK

Spectrum No: 12; Query: 31027; Rank: 1

Peptide View

MS/MS Fragmentation of **YVAEIEKEKEEENEK**
Found in **IPI00024175**, Tax_Id=9606 Gene_Symbol=PSMA7 Isoform 1 of Proteasome subunit alpha type-7

Match to Query 31027: 1736.852568 from(869.433560,2+)
Title: 091224LimSK_Exosome3_06.245.245.2.dta
Data file C:\Temp\Mascoti\LSK_2D\091224LimSK_Exosome3_01_2D.mgf

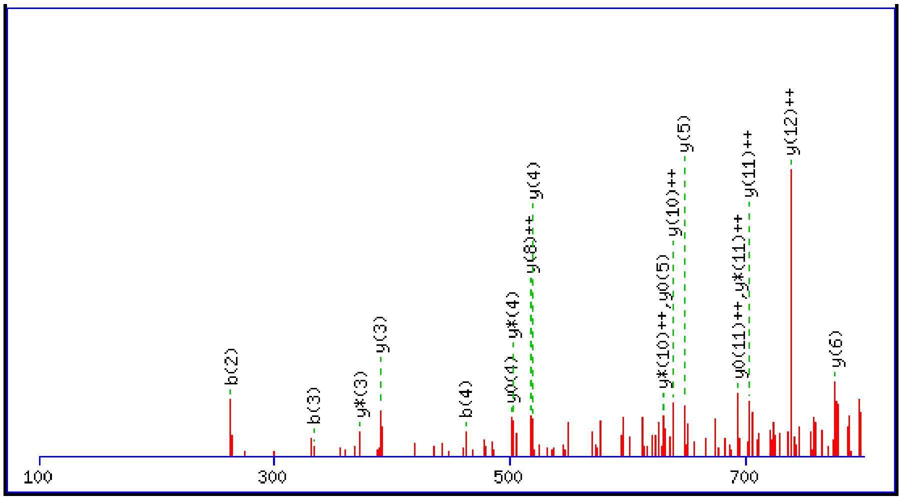

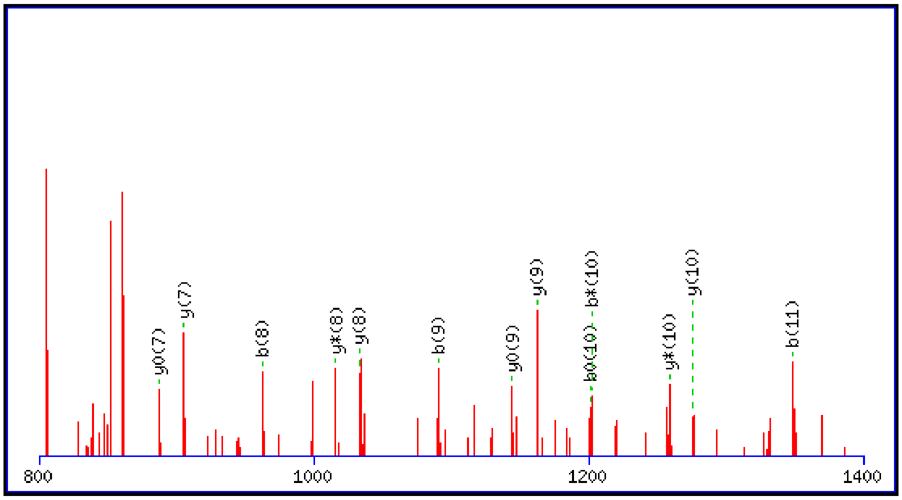

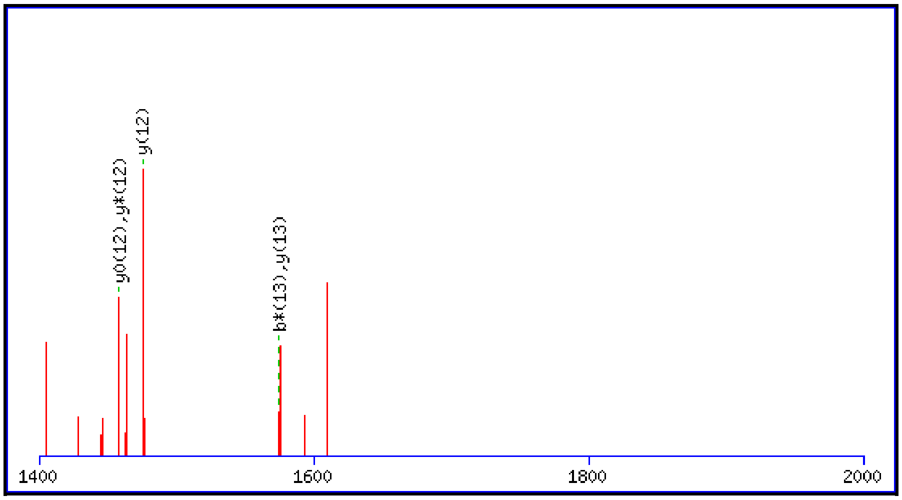

Monoisotopic mass of neutral peptide Mr(calc): 1736.8468
Fixed modifications: Carbamidomethyl (C)
Ions Score: 50 **Expect:** 0.0023
Matches (Bold Red): 36/136 fragment ions using 59 most intense peaks

#	b	b^{++}	b*	b^{*++}	b^0	b^{0++}	Seq.	y	y^{++}	y*	y^{*++}	y^0	y^{0++}	#
1	164.0706	82.5389					Y							14
2	**263.1390**	132.0731					V	**1574.7908**	787.8990	1557.7643	779.3858	1556.7802	778.8938	13
3	**334.1761**	167.5917					A	**1475.7224**	**738.3648**	**1458.6958**	729.8516	**1457.7118**	729.3596	12
4	**463.2187**	232.1130			445.2082	223.1077	E	1404.6853	**702.8463**	1387.6587	**694.3330**	1386.6747	**693.8410**	11
5	576.3028	288.6550			558.2922	279.6498	I	**1275.6427**	**638.3250**	**1258.6161**	**629.8117**	1257.6321	629.3197	10
6	705.3454	353.1763			687.3348	344.1710	E	**1162.5586**	581.7829	1145.5321	573.2697	**1144.5481**	572.7777	9

**7**	833.4403	417.2238	816.4138	408.7105	815.4298	408.2185	**K**	**1033.5160**	**517.2617**	**1016.4895**	508.7484	1015.5055	508.2564	**8**
**8**	**962.4829**	481.7451	945.4564	473.2318	944.4724	472.7398	**E**	**905.4211**	453.2142	888.3945	444.7009	**887.4105**	444.2089	**7**
**9**	**1090.5779**	545.7926	1073.5514	537.2793	1072.5673	536.7873	**K**	**776.3785**	388.6929	759.3519	380.1796	758.3679	379.6876	**6**
**10**	1219.6205	610.3139	**1202.5939**	601.8006	**1201.6099**	601.3086	**E**	**648.2835**	324.6454	631.2570	316.1321	**630.2729**	315.6401	**5**
**11**	**1348.6631**	674.8352	1331.6365	666.3219	1330.6525	665.8299	**E**	**519.2409**	260.1241	**502.2144**	251.6108	**501.2304**	251.1188	**4**
**12**	1462.7060	731.8566	1445.6795	723.3434	1444.6955	722.8514	**N**	**390.1983**	195.6028	**373.1718**	187.0895	372.1878	186.5975	**3**
**13**	1591.7486	796.3779	**1574.7221**	787.8647	1573.7380	787.3727	**E**	276.1554	138.5813	259.1288	130.0681	258.1448	129.5761	**2**
**14**							**K**	147.1128	74.0600	130.0863	65.5468			**1**

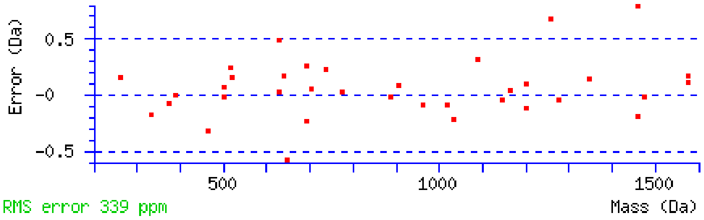

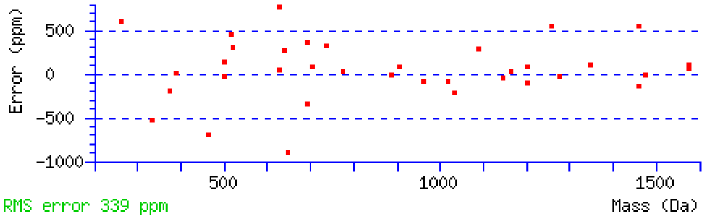

All matches to this query

Score	Mr(calc):	Delta	Sequence
49.6	1736.8468	0.0058	YVAEIEKEKEENEK
8.2	1736.8359	0.0167	VYQAVRHNKATENK
7.3	1736.8498	0.0027	YSLIVAQHVEKEGGK
5.0	1735.8553	0.9973	GPSLRLAGVTMGGRER
4.9	1735.8593	0.9933	YLSQLLMRRSAYR
4.2	1736.8498	0.0027	YSLIVAQHVEKEGGK
3.9	1734.8342	2.0184	FLEYGKDTLQRTGK
3.6	1735.8662	0.9864	ESMAVLEKQDISDKK
3.3	1734.8537	1.9989	SSSSKYFLTSESTRR
3.0	1736.8386	0.0139	LGSGSFGTVYLVSDKK

Spectrum No: 13; Query: 10725; Rank: 1

Peptide View

MS/MS Fragmentation of **AINQGGLTSVAVR**
Found in **IPI00029623**, Tax_Id=9606 Gene_Symbol=PSMA6 Proteasome subunit alpha type-6

Match to Query 10725: 1284.722048 from(643.368300,2+)
Title: 091224LimSK_Exosome3_07.1898.1898.2.dta
Data file C:\Temp\Mascoti\LSK_2D\091224LimSK_Exosome3_01_2D.mgf

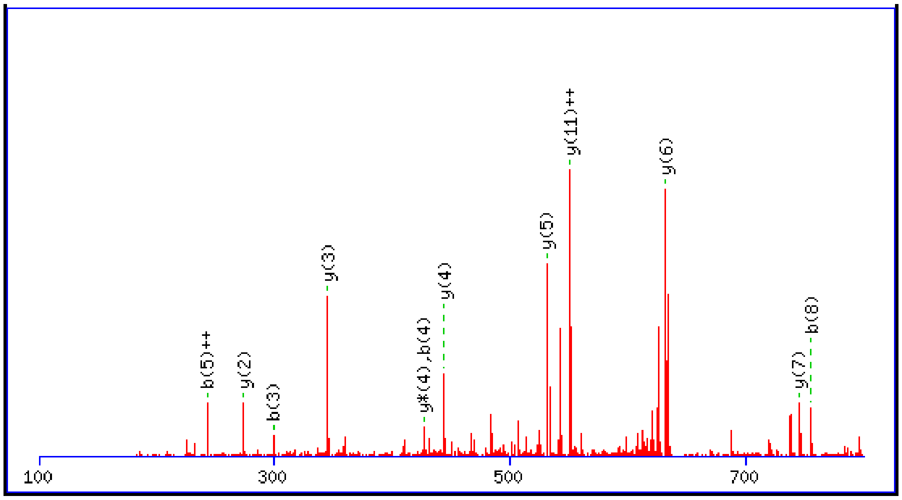

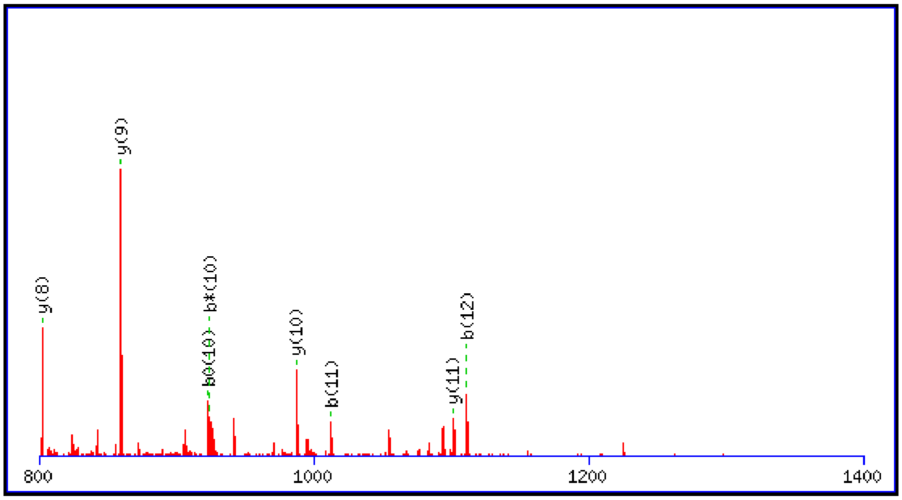

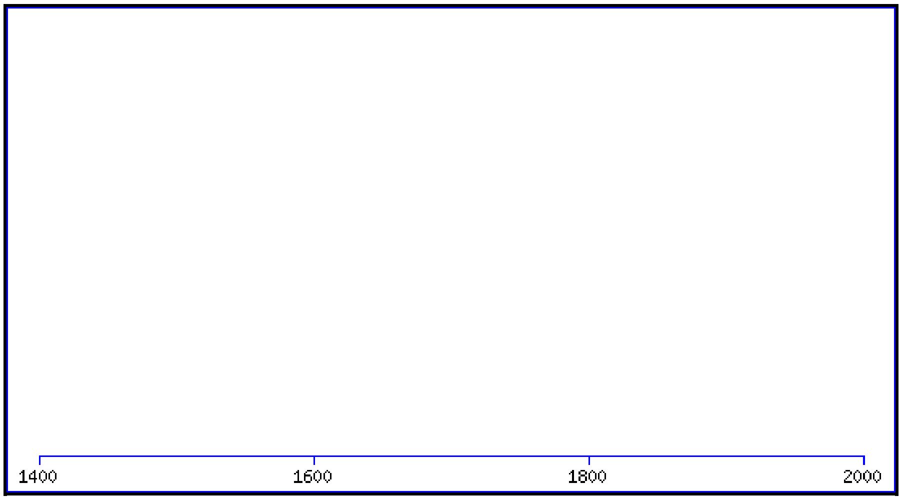

Monoisotopic mass of neutral peptide Mr(calc): 1284.7150
Fixed modifications: Carbamidomethyl (C)
Ions Score: 95 **Expect:** 2.7e-008
Matches (Bold Red): 20/118 fragment ions using 24 most intense peaks

#	b	b^{++}	b*	b^{*++}	b^0	b^{0++}	Seq.	y	y^{++}	y*	y^{*++}	y^0	y^{0++}	#
1	72.0444	36.5258					A							13
2	185.1285	93.0679					I	1214.6852	607.8462	1197.6586	599.3329	1196.6746	598.8409	12
3	**299.1714**	150.0893	282.1448	141.5761			N	**1101.6011**	**551.3042**	1084.5745	542.7909	1083.5905	542.2989	11
4	**427.2300**	214.1186	410.2034	205.6053			Q	**987.5582**	494.2827	970.5316	485.7694	969.5476	485.2774	10
5	484.2514	**242.6293**	467.2249	234.1161			G	**859.4996**	430.2534	842.4730	421.7402	841.4890	421.2482	9
6	541.2729	271.1401	524.2463	262.6268			G	**802.4781**	401.7427	785.4516	393.2294	784.4676	392.7374	8

**7**	654.3570	327.6821	637.3304	319.1688			**L**	**745.4567**	373.2320	728.4301	364.7187	727.4461	364.2267	**7**
**8**	**755.4046**	378.2060	738.3781	369.6927	737.3941	369.2007	**T**	**632.3726**	316.6899	615.3461	308.1767	614.3620	307.6847	**6**
**9**	842.4367	421.7220	825.4101	413.2087	824.4261	412.7167	**S**	**531.3249**	266.1661	514.2984	257.6528	513.3144	257.1608	**5**
**10**	941.5051	471.2562	**924.4785**	462.7429	**923.4945**	462.2509	**V**	**444.2929**	222.6501	**427.2663**	214.1368			**4**
**11**	**1012.5422**	506.7747	995.5156	498.2615	994.5316	497.7694	**A**	**345.2245**	173.1159	328.1979	164.6026			**3**
**12**	**1111.6106**	556.3089	1094.5841	547.7957	1093.6000	547.3037	**V**	**274.1874**	137.5973	257.1608	129.0840			**2**
**13**							**R**	175.1190	88.0631	158.0924	79.5498			**1**

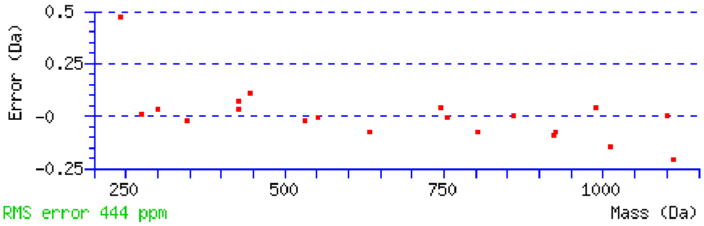

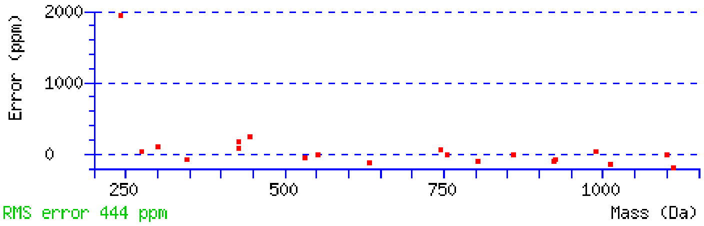

All matches to this query

Score	Mr(calc):	Delta	Sequence
94.6	1284.7150	0.0071	AINQGGLTSVAVR
3.7	1284.7224	-0.0003	MKEEVKGIPVR
3.1	1284.7262	-0.0041	LQEVRESIRR
2.5	1284.7150	0.0071	LRELVAGDSAVR
2.2	1284.7262	-0.0042	ERRVLTATPSR
2.0	1284.7262	-0.0041	ELEQRVSLRR
1.4	1284.7224	-0.0003	NVVLCLLELGR

Spectrum No: 14; Query: 12579; Rank: 1

Peptide View

MS/MS Fragmentation of **CDPAGYYCGFK**
Found in **IPI00029623**, Tax_Id=9606 Gene_Symbol=PSMA6 Proteasome subunit alpha type-6

Match to Query 12579: 1336.526908 from(669.270730,2+)
Title: 091224LimSK_Exosome3_06.2429.2429.2.dta
Data file C:\Temp\Mascoti\LSK_2D\091224LimSK_Exosome3_01_2D.mgf

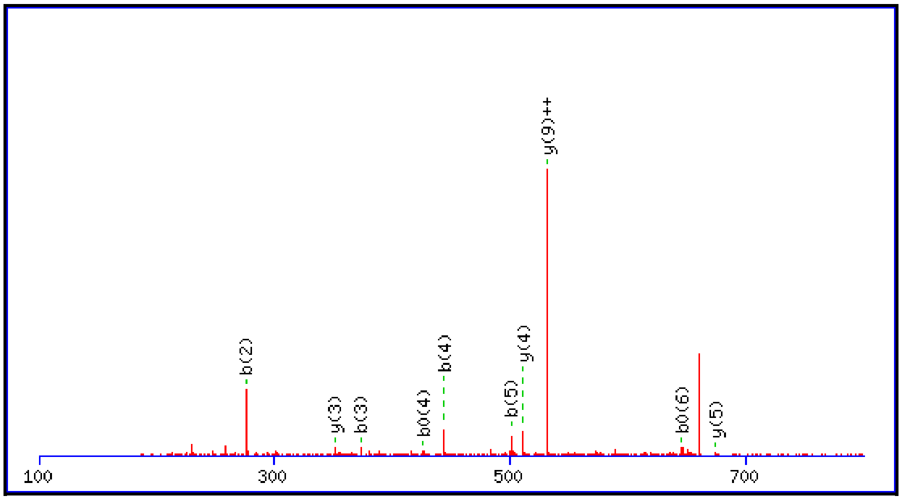

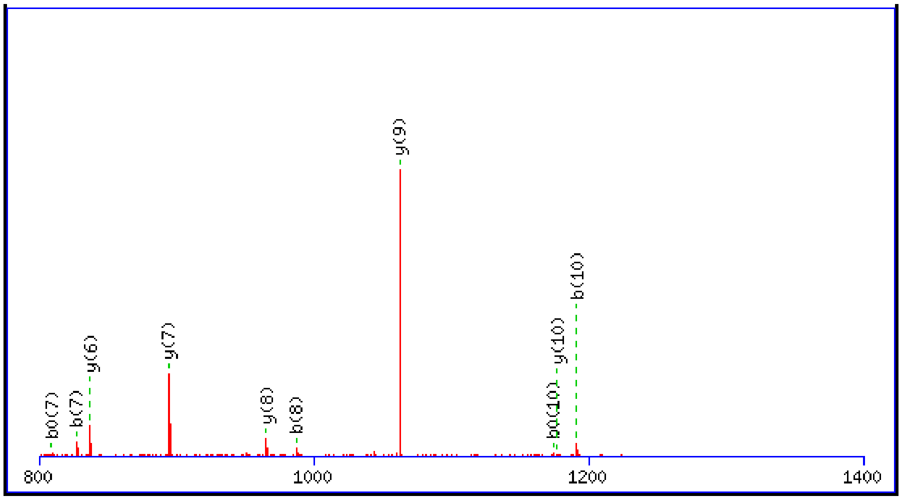

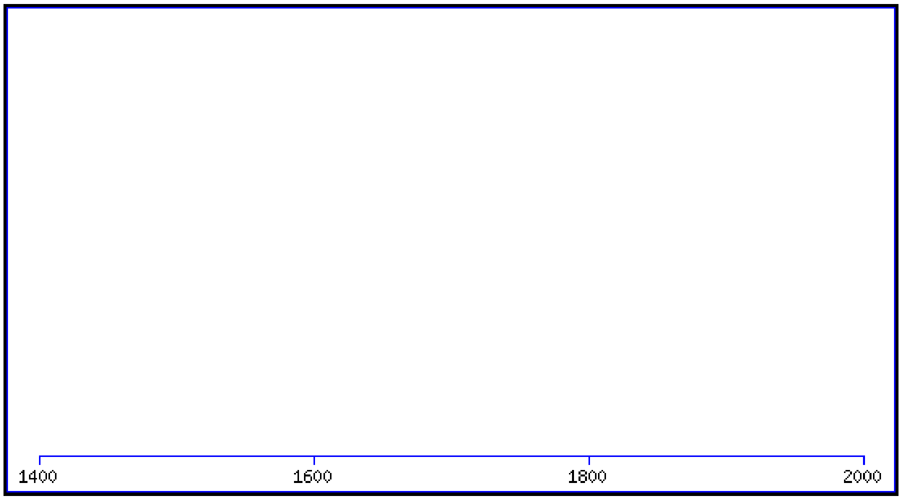

Monoisotopic mass of neutral peptide Mr(calc): 1336.5217
Fixed modifications: Carbamidomethyl (C)
Ions Score: 45 **Expect:** 0.0034
Matches (Bold Red): 20/80 fragment ions using 45 most intense peaks

#	b	b^{++}	b^{0}	b^{0++}	Seq.	y	y^{++}	y^{*}	y^{*++}	y^{0}	y^{0++}	#
1	161.0379	81.0226			C							11
2	**276.0649**	138.5361	258.0543	129.5308	D	**1177.4983**	589.2528	1160.4717	580.7395	1159.4877	580.2475	10
3	**373.1176**	187.0625	355.1071	178.0572	P	**1062.4713**	**531.7393**	1045.4448	523.2260			9
4	**444.1547**	222.5810	**426.1442**	213.5757	A	**965.4186**	483.2129	948.3920	474.6996			8
5	**501.1762**	251.0917	483.1656	242.0865	G	**894.3815**	447.6944	877.3549	439.1811			7
6	664.2395	332.6234	**646.2290**	323.6181	Y	**837.3600**	419.1836	820.3334	410.6704			6
7	**827.3029**	414.1551	**809.2923**	405.1498	Y	**674.2967**	337.6520	657.2701	329.1387			5
8	**987.3335**	494.1704	969.3230	485.1651	C	**511.2333**	256.1203	494.2068	247.6070			4
9	1044.3550	522.6811	1026.3444	513.6758	G	**351.2027**	176.1050	334.1761	167.5917			3
10	**1191.4234**	596.2153	**1173.4128**	587.2101	F	294.1812	147.5942	277.1547	139.0810			2
11					K	147.1128	74.0600	130.0863	65.5468			1

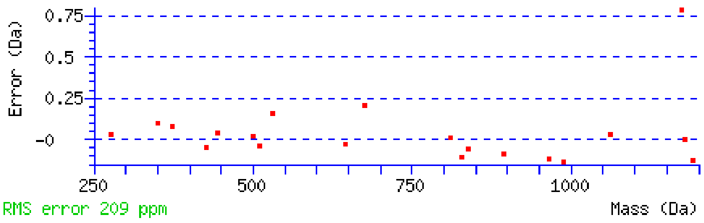

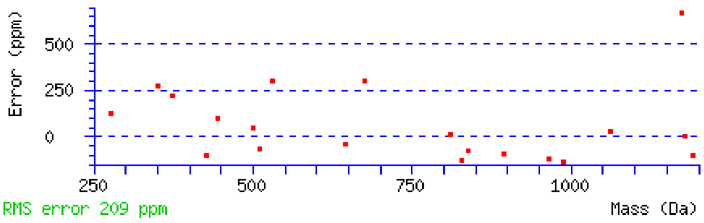

All matches to this query

Score	Mr(calc):	Delta	Sequence
45.1	1336.5217	0.0053	CDPAGYYCGFK
26.3	1336.5257	0.0012	YAESLSEAMEK
23.5	1336.5387	-0.0117	MKSTEVLPEK
17.4	1336.5193	0.0076	XCSFGVQLMGK
14.7	1336.5257	0.0012	YAESLSEAMEK
12.8	1335.5353	0.9916	XCSFGVQLMGK
12.1	1336.5313	-0.0044	TGGTNTEKLEK
12.1	1336.5313	-0.0044	TGGTNTEKLEK
11.3	1336.5257	0.0012	YAESLSEAMEK
11.1	1336.5313	-0.0044	LGSEIIDSSTR

Spectrum No: 15; Query: 8543; Rank: 1

Peptide View

MS/MS Fragmentation of **GKDCAVIVTQK**
Found in **IPI00029623**, Tax_Id=9606 Gene_Symbol=PSMA6 Proteasome subunit alpha type-6

Match to Query 8543: 1217.648952 from(406.890260,3+)
Title: 091224LimSK_Exosome3_06.125.125.3.dta
Data file C:\Temp\Mascoti\LSK_2D\091224LimSK_Exosome3_01_2D.mgf

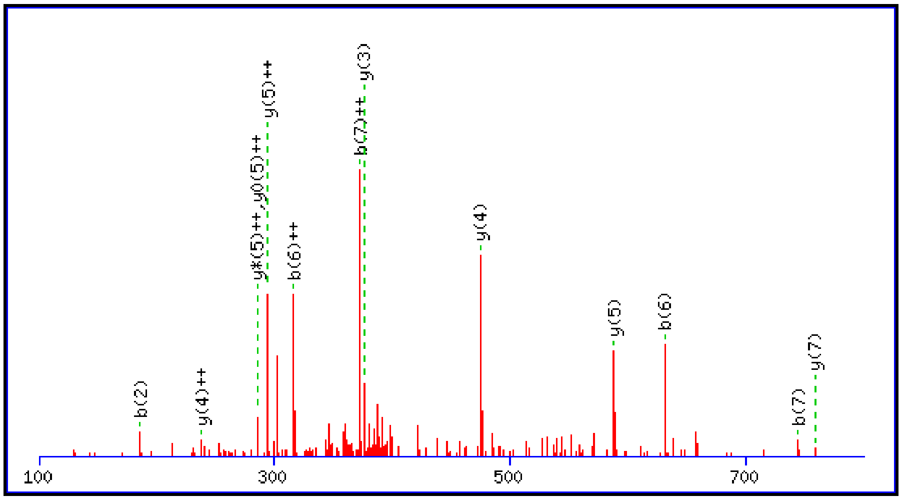

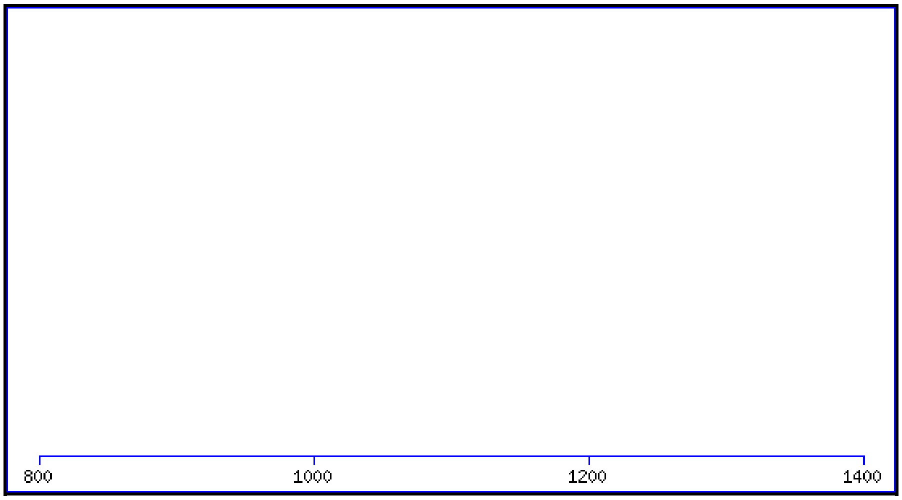

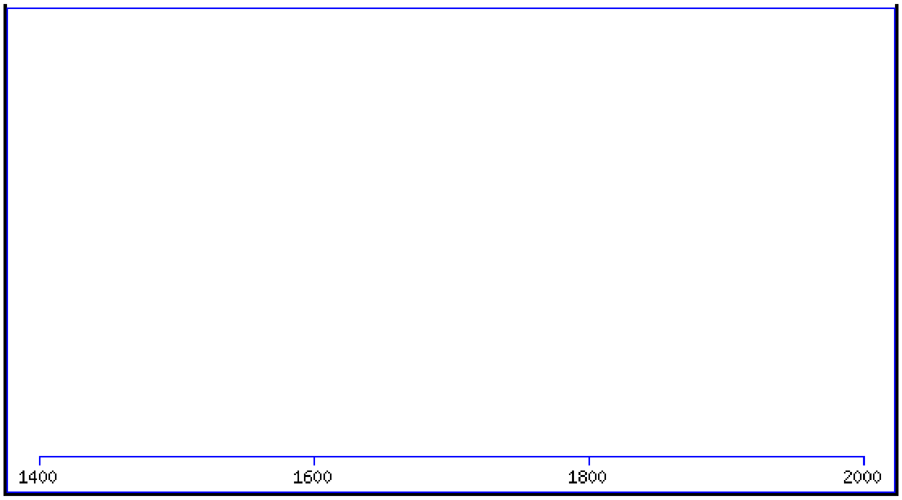

Monoisotopic mass of neutral peptide Mr(calc): 1217.6438
Fixed modifications: Carbamidomethyl (C)
Ions Score: 25 **Expect:** 0.39
Matches (Bold Red): 13/110 fragment ions using 17 most intense peaks

#	b	b^{++}	b*	b^{*++}	b^{0}	b^{0++}	Seq.	y	y^{++}	y*	y^{*++}	y^{0}	y^{0++}	#
1	58.0287	29.5180					G							11
2	**186.1237**	93.5655	169.0972	85.0522			K	1161.6296	581.3184	1144.6031	572.8052	1143.6191	572.3132	10
3	301.1506	151.0790	284.1241	142.5657	283.1401	142.0737	D	1033.5347	517.2710	1016.5081	508.7577	1015.5241	508.2657	9
4	461.1813	231.0943	444.1547	222.5810	443.1707	222.0890	C	918.5077	459.7575	901.4812	451.2442	900.4972	450.7522	8
5	532.2184	266.6128	515.1919	258.0996	514.2078	257.6076	A	**758.4771**	379.7422	741.4505	371.2289	740.4665	370.7369	7
6	**631.2868**	**316.1470**	614.2603	307.6338	613.2763	307.1418	V	687.4400	344.2236	670.4134	335.7103	669.4294	335.2183	6
7	**744.3709**	**372.6891**	727.3443	364.1758	726.3603	363.6838	I	**588.3715**	**294.6894**	571.3450	**286.1761**	570.3610	**285.6841**	5
8	843.4393	422.2233	826.4128	413.7100	825.4287	413.2180	V	**475.2875**	**238.1474**	458.2609	229.6341	457.2769	229.1421	4
9	944.4870	472.7471	927.4604	464.2339	926.4764	463.7418	T	**376.2191**	188.6132	359.1925	180.0999	358.2085	179.6079	3
10	1072.5456	536.7764	1055.5190	528.2631	1054.5350	527.7711	Q	275.1714	138.0893	258.1448	129.5761			2
11							K	147.1128	74.0600	130.0863	65.5468			1

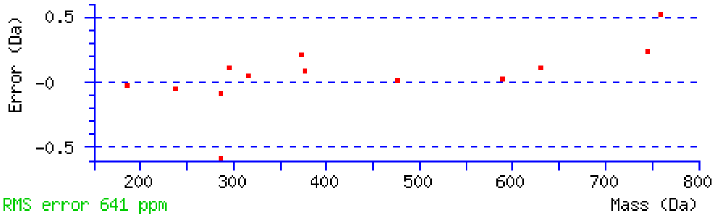

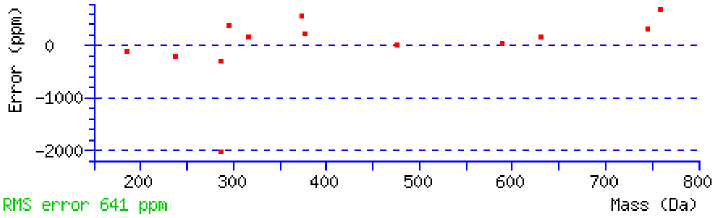

All matches to this query

Score	Mr(calc):	Delta	Sequence
25.3	1217.6438	0.0051	GKDCAVIVTQK
17.4	1215.6360	2.0129	FVPEIRDGQR
17.4	1216.6468	1.0021	YVLLSSLSKK
17.4	1216.6468	1.0021	YVLLSSLSKK
15.2	1217.6503	-0.0013	DAVESIEKISK
14.8	1216.6524	0.9965	LAGSSGRGGQTVK
14.4	1217.6438	0.0052	LMDDLLNKTR
14.1	1217.6478	0.0011	NALADFLPVMK
11.1	1216.6524	0.9966	DRTQELRTAK
10.6	1216.6403	1.0087	TKRIMITFK

Spectrum No: 16; Query: 6446; Rank: 1

Peptide View

MS/MS Fragmentation of **HITIFSPEGR**
Found in **IPI00029623**, Tax_Id=9606 Gene_Symbol=PSMA6 Proteasome subunit alpha type-6

Match to Query 6446: 1155.608408 from(578.811480,2+)
Title: 091224LimSK_Exosome3_06.1790.1790.2.dta
Data file C:\Temp\Mascoti\LSK_2D\091224LimSK_Exosome3_01_2D.mgf

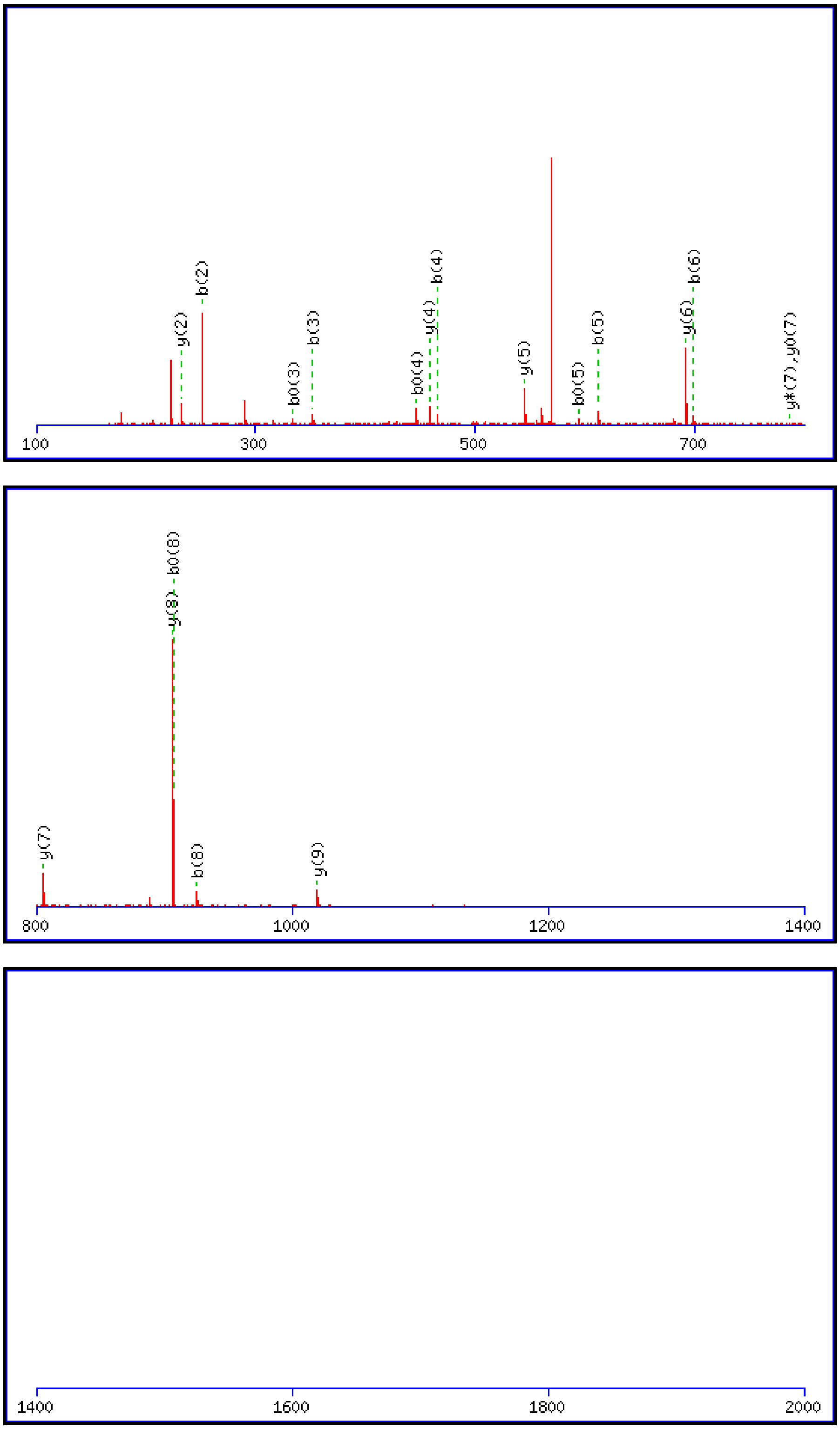

Monoisotopic mass of neutral peptide Mr(calc): 1155.6037

Fixed modifications: Carbamidomethyl (C)
Ions Score: 52 **Expect:** 0.00067
Matches (Bold Red): 19/82 fragment ions using 31 most intense peaks

#	b	b^{++}	b^0	b^{0++}	Seq.	y	y^{++}	y*	y^{*++}	y^0	y^{0++}	#
1	138.0662	69.5367			H							10
2	**251.1503**	126.0788			I	**1019.5520**	510.2796	1002.5255	501.7664	1001.5415	501.2744	9
3	**352.1979**	176.6026	**334.1874**	167.5973	T	**906.4680**	453.7376	889.4414	445.2243	888.4574	444.7323	8
4	**465.2820**	233.1446	**447.2714**	224.1394	I	**805.4203**	403.2138	**788.3937**	394.7005	**787.4097**	394.2085	7
5	**612.3504**	306.6788	**594.3398**	297.6736	F	**692.3362**	346.6717	675.3097	338.1585	674.3257	337.6665	6
6	**699.3824**	350.1949	681.3719	341.1896	S	**545.2678**	273.1375	528.2413	264.6243	527.2572	264.1323	5
7	796.4352	398.7212	778.4246	389.7160	P	**458.2358**	229.6215	441.2092	221.1083	440.2252	220.6162	4
8	**925.4778**	463.2425	**907.4672**	454.2373	E	361.1830	181.0951	344.1565	172.5819	343.1724	172.0899	3
9	982.4993	491.7533	964.4887	482.7480	G	**232.1404**	116.5738	215.1139	108.0606			2
10					R	175.1190	88.0631	158.0924	79.5498			1

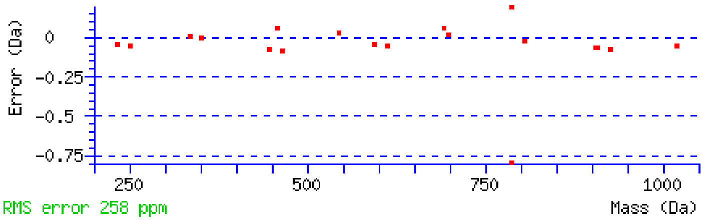

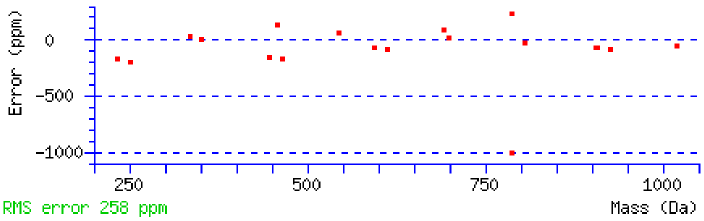

All matches to this query

Score	Mr(calc):	Delta	Sequence
52.1	1155.6037	0.0048	HITIFSPEGR
12.3	1155.6070	0.0014	HLTQAVCTVK
7.6	1155.6183	-0.0098	CVPGREILGR
3.1	1155.6182	-0.0098	HLNTIQKMR
3.0	1155.5996	0.0088	HLSDSKSQVR
2.6	1153.6131	1.9953	HITIEDLWK
2.3	1153.6132	1.9952	TFSPTYGLLR

Spectrum No: 17; Query: 39057; Rank: 1

Peptide View

MS/MS Fragmentation of **ILTEAEIDAHLVALAERD**
Found in **IPI00029623**, Tax_Id=9606 Gene_Symbol=PSMA6 Proteasome subunit alpha type-6

Match to Query 39057: 1978.050432 from(660.357420,3+)
Title: 091224LimSK_Exosome3_06.9882.9882.3.dta
Data file C:\Temp\Mascoti\LSK_2D\091224LimSK_Exosome3_01_2D.mgf

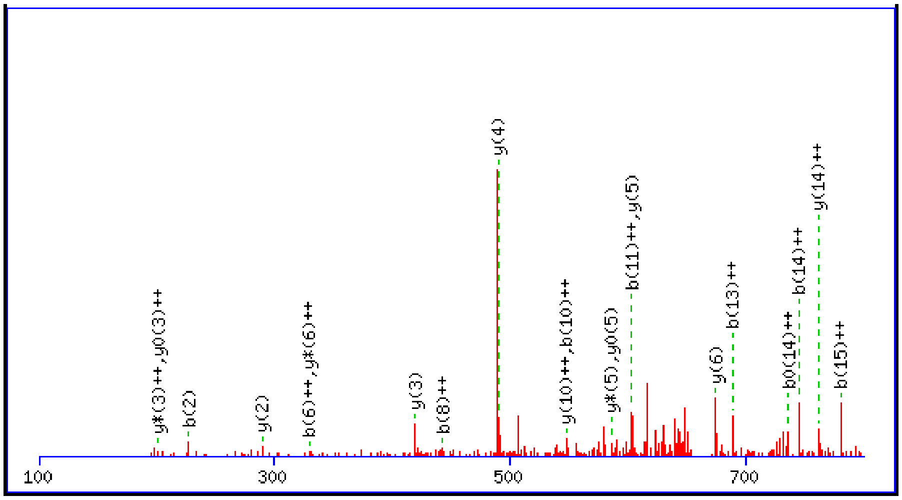

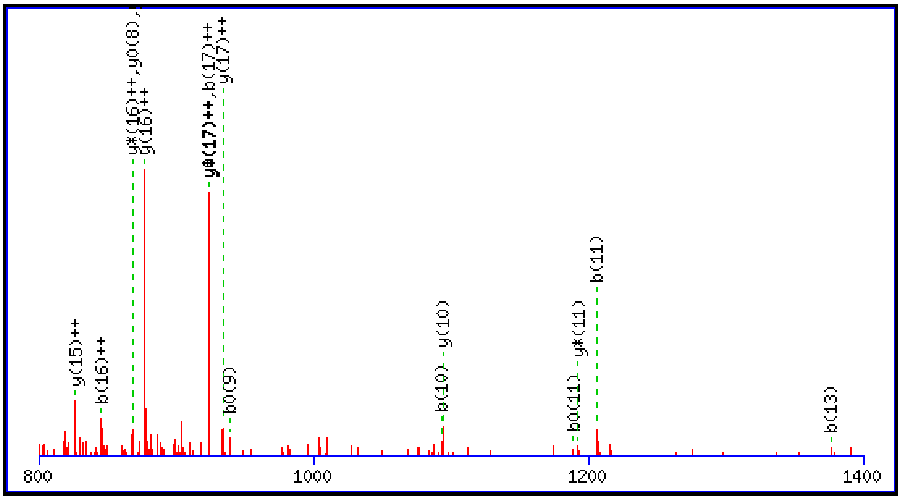

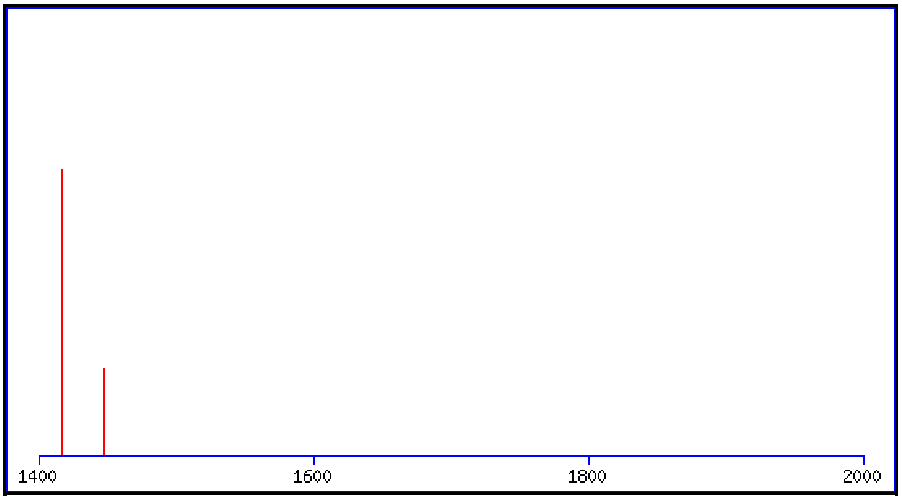

Monoisotopic mass of neutral peptide Mr(calc): 1978.0371
Fixed modifications: Carbamidomethyl (C)
Ions Score: 39 **Expect:** 0.013
Matches (Bold Red): 38/166 fragment ions using 74 most intense peaks

#	b	b^{++}	b*	b^{*++}	b^0	b^{0++}	Seq.	y	y^{++}	y*	y^{*++}	y^0	y^{0++}	#
1	114.0913	57.5493					I							18
2	**227.1754**	114.0913					L	1865.9603	**933.4838**	1848.9338	**924.9705**	1847.9498	**924.4785**	17
3	328.2231	164.6152			310.2125	155.6099	T	1752.8763	**876.9418**	1735.8497	**868.4285**	1734.8657	**867.9365**	16
4	457.2657	229.1365			439.2551	220.1312	E	1651.8286	**826.4179**	1634.8020	817.9047	1633.8180	817.4127	15
5	528.3028	264.6550			510.2922	255.6498	A	1522.7860	**761.8966**	1505.7594	753.3834	1504.7754	752.8914	14
6	657.3454	**329.1763**			639.3348	320.1710	E	1451.7489	726.3781	1434.7223	717.8648	1433.7383	717.3728	13

**7**	770.4294	385.7184			752.4189	376.7131	**I**	1322.7063	661.8568	1305.6797	653.3435	1304.6957	652.8515	**12**
**8**	885.4564	**443.2318**			867.4458	434.2266	**D**	1209.6222	605.3148	**1192.5957**	596.8015	1191.6117	596.3095	**11**
**9**	956.4935	478.7504			**938.4829**	469.7451	**A**	**1094.5953**	**547.8013**	1077.5687	539.2880	1076.5847	538.7960	**10**
**10**	**1093.5524**	**547.2798**			1075.5419	538.2746	**H**	1023.5582	512.2827	1006.5316	503.7694	1005.5476	503.2774	**9**
**11**	**1206.6365**	**603.8219**			**1188.6259**	594.8166	**L**	886.4993	443.7533	869.4727	435.2400	**868.4887**	434.7480	**8**
**12**	1305.7049	653.3561			1287.6943	644.3508	**V**	773.4152	387.2112	756.3886	378.6980	755.4046	378.2060	**7**
**13**	**1376.7420**	**688.8746**			1358.7314	679.8694	**A**	**674.3468**	337.6770	657.3202	**329.1638**	656.3362	328.6717	**6**
**14**	1489.8261	**745.4167**			1471.8155	**736.4114**	**L**	**603.3097**	302.1585	**586.2831**	293.6452	**585.2991**	293.1532	**5**
**15**	1560.8632	**780.9352**			1542.8526	771.9299	**A**	**490.2256**	245.6164	473.1991	237.1032	472.2150	236.6112	**4**
**16**	1689.9058	**845.4565**			1671.8952	836.4512	**E**	**419.1885**	210.0979	402.1619	**201.5846**	401.1779	**201.0926**	**3**
**17**	1846.0069	**923.5071**	1828.9803	914.9938	1827.9963	914.5018	**R**	**290.1459**	145.5766	273.1193	137.0633	272.1353	136.5713	**2**
**18**							**D**	134.0448	67.5260			116.0342	58.5207	**1**

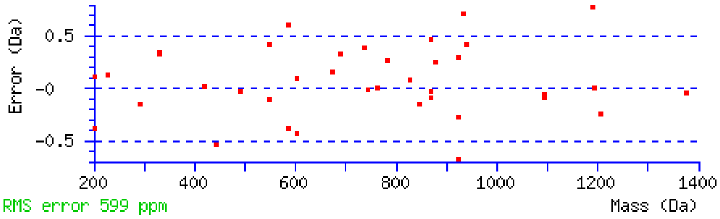

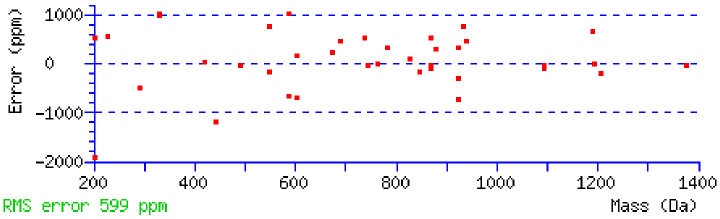

All matches to this query

Score	Mr(calc):	Delta	Sequence
39.2	1978.0371	0.0134	ILTEAEIDAHLVALAERD
11.1	1976.0401	2.0103	CTVKPLAFIEGLSDTGLR
10.6	1977.0482	1.0023	ILIEENRKVIAPMLSR
1.7	1978.0553	-0.0049	FTLGTFLRLAALRTYR
1.6	1977.0304	1.0200	SIVRIIGKMLPLEPCR
0.8	1978.0418	0.0087	MAAEALRNYRNTQAILK
0.8	1978.0645	-0.0140	MFPISVVRPFRKMNEK
0.3	1976.0466	2.0038	EDELTFIKSAIIQNVEK
0.3	1976.0327	2.0178	LAGSSAASSPNGQGLQKLYK
0.3	1976.0327	2.0177	FLIDSNGQVITTERLDR

Spectrum No: 18; Query: 35132; Rank: 1

Peptide View

MS/MS Fragmentation of **ITENIGCVMTGMTADSR**
Found in **IPI00029623**, Tax_Id=9606 Gene_Symbol=PSMA6 Proteasome subunit alpha type-6

Match to Query 35132: 1854.842608 from(928.428580,2+)
Title: 091224LimSK_Exosome3_06.4869.4869.2.dta
Data file C:\Temp\Mascoti\LSK_2D\091224LimSK_Exosome3_01_2D.mgf

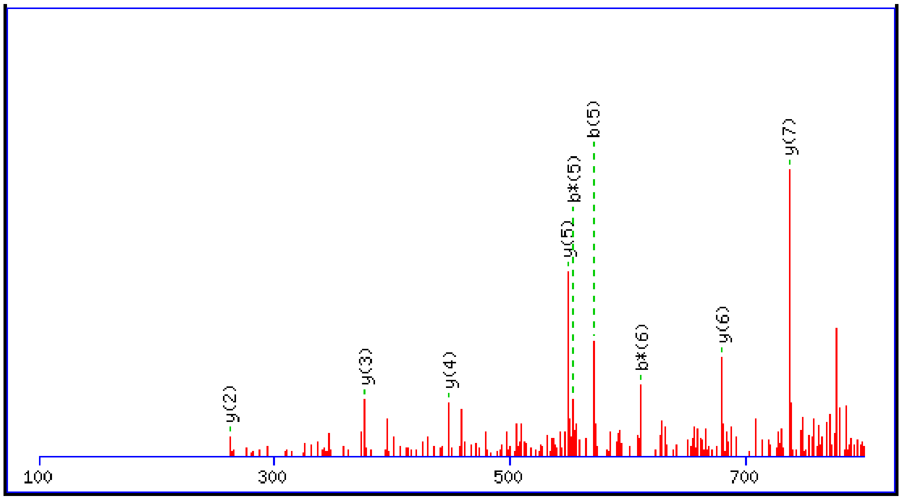

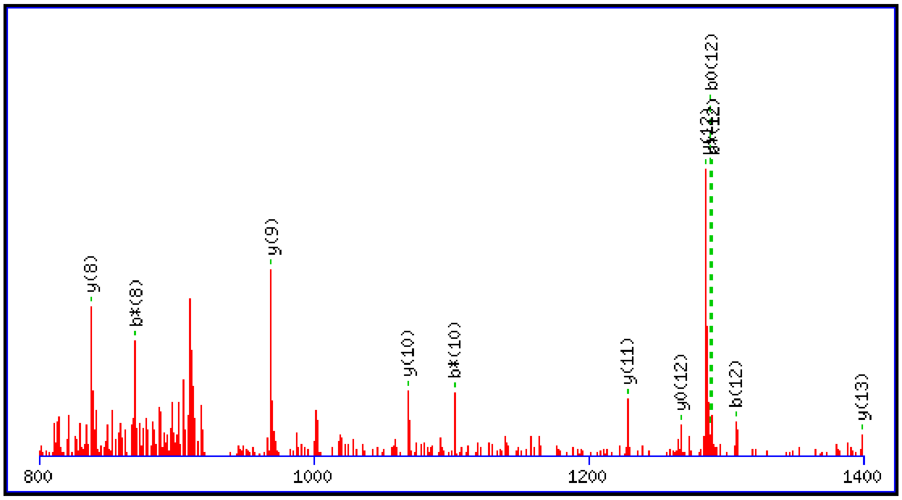

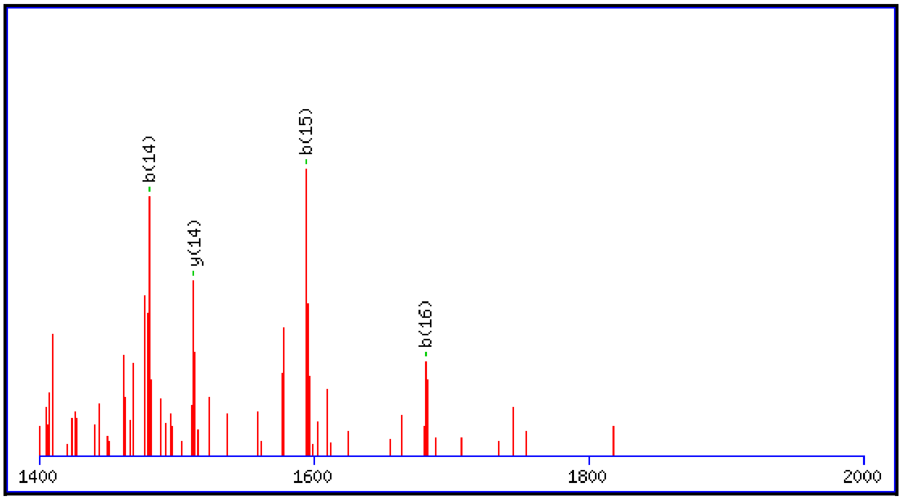

Monoisotopic mass of neutral peptide Mr(calc): 1854.8274
Fixed modifications: Carbamidomethyl (C)
Ions Score: 113 **Expect:** 1.3e-009
Matches (Bold Red): 25/182 fragment ions using 41 most intense peaks

#	b	b++	b*	b*++	b0	b0++	Seq.	y	y++	y*	y*++	y0	y0++	#
1	114.0913	57.5493					I							17
2	215.1390	108.0731			197.1285	99.0679	T	1742.7506	871.8789	1725.7241	863.3657	1724.7401	862.8737	16
3	344.1816	172.5944			326.1710	163.5892	E	1641.7029	821.3551	1624.6764	812.8418	1623.6924	812.3498	15
4	458.2245	229.6159	441.1980	221.1026	440.2140	220.6106	N	**1512.6603**	756.8338	1495.6338	748.3205	1494.6498	747.8285	14
5	**571.3086**	286.1579	**554.2821**	277.6447	553.2980	277.1527	I	**1398.6174**	699.8123	1381.5909	691.2991	1380.6069	690.8071	13
6	628.3301	314.6687	**611.3035**	306.1554	610.3195	305.6634	G	**1285.5334**	643.2703	1268.5068	634.7570	**1267.5228**	634.2650	12

**7**	788.3607	394.6840	771.3342	386.1707	770.3502	385.6787	**C**	**1228.5119**	614.7596	1211.4853	606.2463	1210.5013	605.7543	**11**
**8**	887.4291	444.2182	**870.4026**	435.7049	869.4186	435.2129	**V**	**1068.4812**	534.7443	1051.4547	526.2310	1050.4707	525.7390	**10**
**9**	1018.4696	509.7384	1001.4431	501.2252	1000.4591	500.7332	**M**	**969.4128**	485.2101	952.3863	476.6968	951.4023	476.2048	**9**
**10**	1119.5173	560.2623	**1102.4907**	551.7490	1101.5067	551.2570	**T**	**838.3723**	419.6898	821.3458	411.1765	820.3618	410.6845	**8**
**11**	1176.5388	588.7730	1159.5122	580.2597	1158.5282	579.7677	**G**	**737.3247**	369.1660	720.2981	360.6527	719.3141	360.1607	**7**
**12**	**1307.5792**	654.2933	**1290.5527**	645.7800	**1289.5687**	645.2880	**M**	**680.3032**	340.6552	663.2767	332.1420	662.2926	331.6500	**6**
**13**	1408.6269	704.8171	1391.6004	696.3038	1390.6164	695.8118	**T**	**549.2627**	275.1350	532.2362	266.6217	531.2522	266.1297	**5**
**14**	**1479.6640**	740.3357	1462.6375	731.8224	1461.6535	731.3304	**A**	**448.2150**	224.6112	431.1885	216.0979	430.2045	215.6059	**4**
**15**	**1594.6910**	797.8491	1577.6644	789.3359	1576.6804	788.8438	**D**	**377.1779**	189.0926	360.1514	180.5793	359.1674	180.0873	**3**
**16**	**1681.7230**	841.3651	1664.6965	832.8519	1663.7124	832.3599	**S**	**262.1510**	131.5791	245.1244	123.0659	244.1404	122.5738	**2**
**17**							**R**	175.1190	88.0631	158.0924	79.5498			**1**

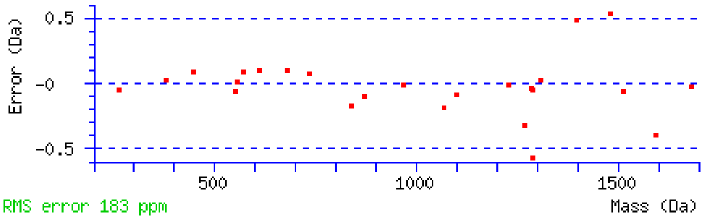

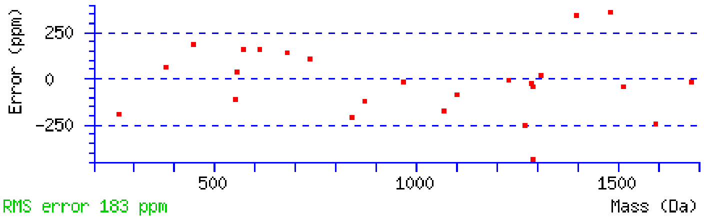

All matches to this query

Score	Mr(calc):	Delta	Sequence
112.5	1854.8274	0.0152	ITENIGCVMTGMTADSR
5.2	1854.8587	-0.0161	TSLGKITGNYLGYTCK
1.2	1854.8587	-0.0161	TSLGKITGNYLGYTCK
0.6	1853.8520	0.9906	DGEKSGTVSPQKQSAQK
0.5	1854.8587	-0.0161	TSLGKITGNYLGYTCK

Spectrum No: 19; Query: 13505; Rank: 1

Peptide View

MS/MS Fragmentation of **LLDSSTVTHLFK**
Found in **IPI00029623**, Tax_Id=9606 Gene_Symbol=PSMA6 Proteasome subunit alpha type-6

Match to Query 13505: 1359.749188 from(680.881870,2+)
Title: 091224LimSK_Exosome3_06.5181.5181.2.dta
Data file C:\Temp\Mascoti\LSK_2D\091224LimSK_Exosome3_01_2D.mgf

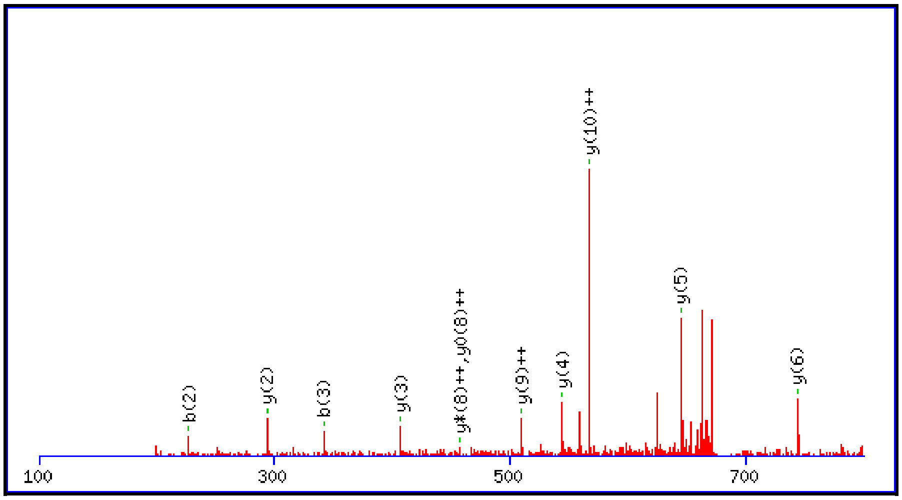

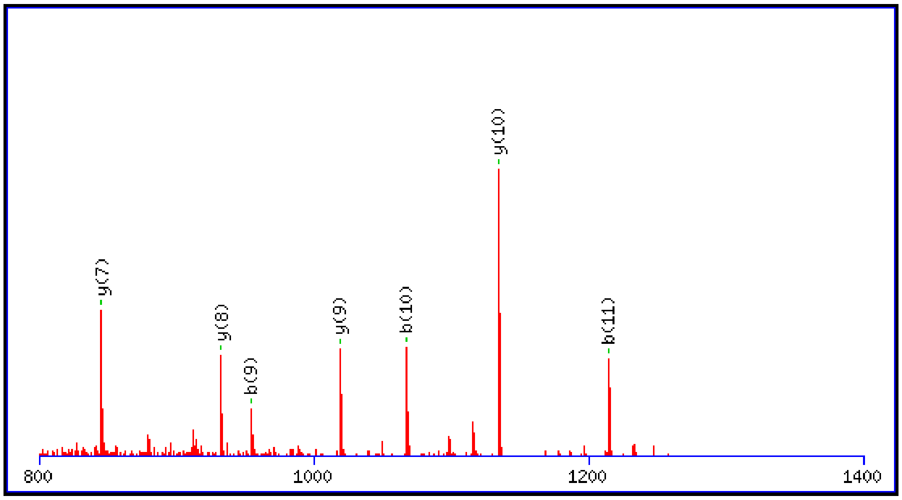

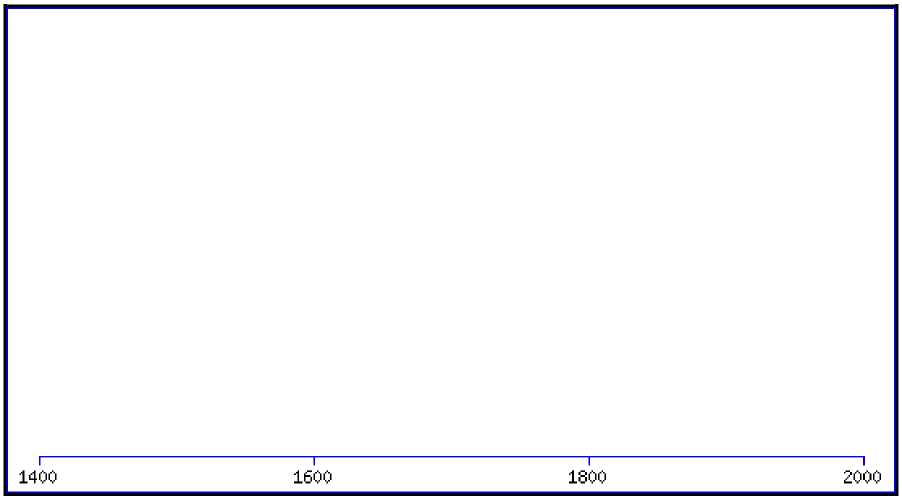

Monoisotopic mass of neutral peptide Mr(calc): 1359.7398
Fixed modifications: Carbamidomethyl (C)
Ions Score: 75 **Expect:** 3.3e-006
Matches (Bold Red): 18/98 fragment ions using 26 most intense peaks

#	b	b^{++}	b^{0}	b^{0++}	Seq.	y	y^{++}	y*	y^{*++}	y^{0}	y^{0++}	#
1	114.0913	57.5493			L							12
2	**227.1754**	114.0913			L	1247.6630	624.3352	1230.6365	615.8219	1229.6525	615.3299	11
3	**342.2023**	171.6048	324.1918	162.5995	D	**1134.5790**	**567.7931**	1117.5524	559.2798	1116.5684	558.7878	10
4	429.2344	215.1208	411.2238	206.1155	S	**1019.5520**	**510.2796**	1002.5255	501.7664	1001.5415	501.2744	9
5	516.2664	258.6368	498.2558	249.6316	S	**932.5200**	466.7636	915.4934	**458.2504**	914.5094	**457.7584**	8
6	617.3141	309.1607	599.3035	300.1554	T	**845.4880**	423.2476	828.4614	414.7343	827.4774	414.2423	7
7	716.3825	358.6949	698.3719	349.6896	V	**744.4403**	372.7238	727.4137	364.2105	726.4297	363.7185	6
8	817.4302	409.2187	799.4196	400.2134	T	**645.3719**	323.1896	628.3453	314.6763	627.3613	314.1843	5
9	**954.4891**	477.7482	936.4785	468.7429	H	**544.3242**	272.6657	527.2976	264.1525			4
10	**1067.5732**	534.2902	1049.5626	525.2849	L	**407.2653**	204.1363	390.2387	195.6230			3
11	**1214.6416**	607.8244	1196.6310	598.8191	F	**294.1812**	147.5942	277.1547	139.0810			2
12					K	147.1128	74.0600	130.0863	65.5468			1

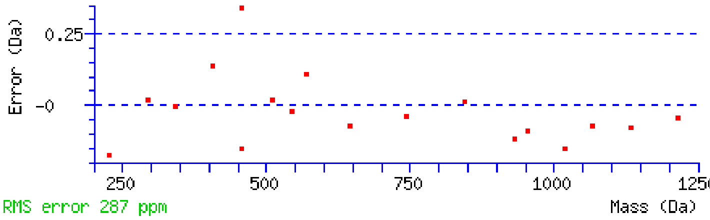

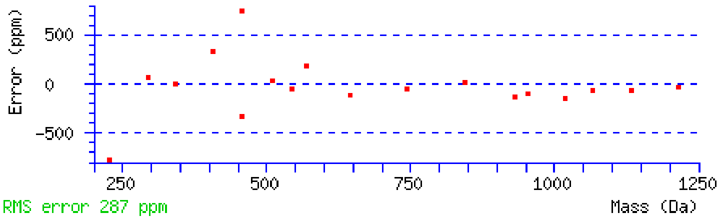

All matches to this query

Score	Mr(calc):	Delta	Sequence
75.0	1359.7398	0.0094	LLDSSTVTHLFK
6.9	1359.7544	-0.0052	LAAKSPIRETMK
5.2	1359.7373	0.0119	MIGVTGWFKPPK
4.5	1359.7623	-0.0131	LLINDFGRERK
4.5	1359.7527	-0.0035	ILDLLIPLTNR
2.5	1359.7544	-0.0052	LSERLIQNIMK
2.5	1359.7366	0.0126	TPMRKINPLMK
1.1	1359.7510	-0.0019	HDSITGSLKKFK
0.6	1359.7609	-0.0117	EIVSGLLAAVSSSK
0.6	1359.7358	0.0134	ARDTVTSELAAVK

Spectrum No: 20; Query: 6609; Rank: 1

Peptide View

MS/MS Fragmentation of **LYQVEYAFK**
Found in **IPI00029623**, Tax_Id=9606 Gene_Symbol=PSMA6 Proteasome subunit alpha type-6

Match to Query 6609: 1159.597848 from(580.806200,2+)
Title: 091224LimSK_Exosome3_06.3990.3990.2.dta
Data file C:\Temp\Mascoti\LSK_2D\091224LimSK_Exosome3_01_2D.mgf

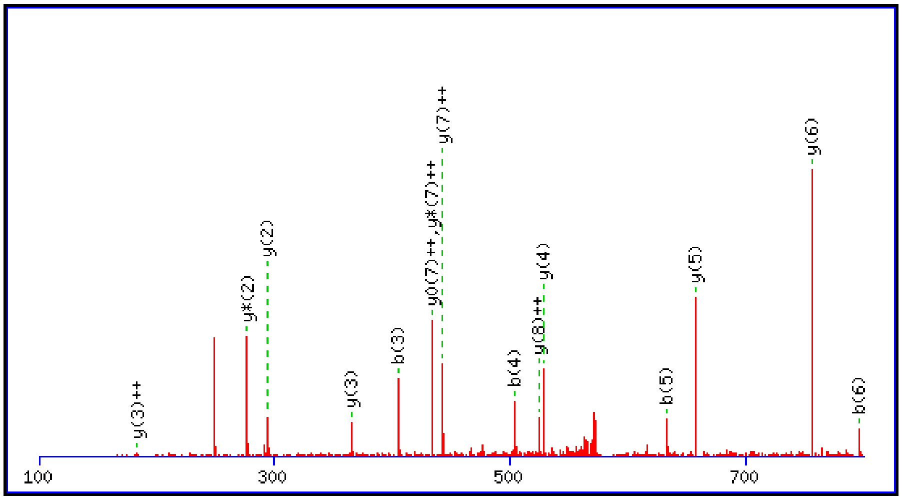

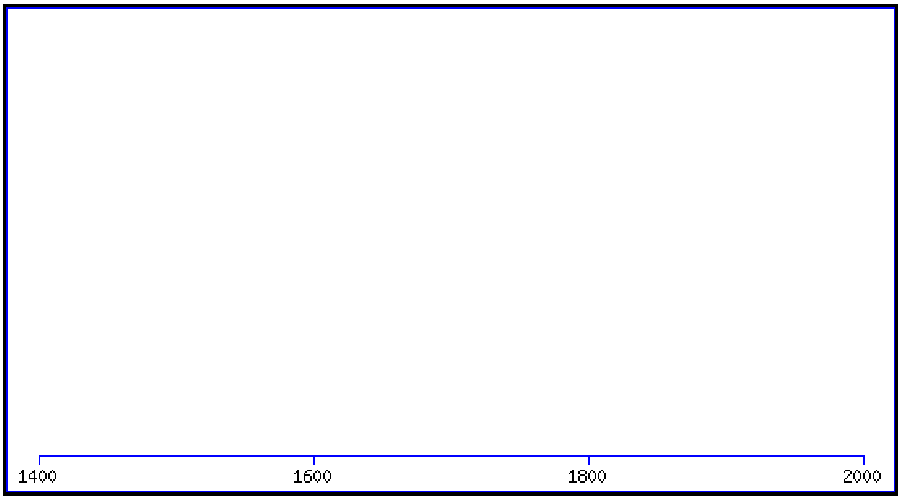

Monoisotopic mass of neutral peptide Mr(calc): 1159.5913
Fixed modifications: Carbamidomethyl (C)
Ions Score: 57 **Expect:** 0.00025
Matches (Bold Red): 25/76 fragment ions using 33 most intense peaks

#	b	b^{++}	b*	b^{*++}	b^0	b^{0++}	Seq.	y	y^{++}	y*	y^{*++}	y^0	y^{0++}	#
1	114.0913	57.5493					L							9
2	**277.1547**	139.0810					Y	**1047.5146**	**524.2609**	1030.4880	515.7477	1029.5040	515.2556	8
3	**405.2132**	203.1103	388.1867	194.5970			Q	**884.4512**	**442.7293**	**867.4247**	**434.2160**	**866.4407**	**433.7240**	7
4	**504.2817**	252.6445	487.2551	244.1312			V	**756.3927**	378.7000	739.3661	370.1867	738.3821	369.6947	6
5	**633.3243**	317.1658	616.2977	308.6525	615.3137	308.1605	E	**657.3243**	329.1658	640.2977	320.6525	639.3137	320.1605	5
6	**796.3876**	398.6974	779.3610	390.1842	778.3770	389.6921	Y	**528.2817**	264.6445	511.2551	256.1312			4
7	**867.4247**	**434.2160**	**850.3981**	425.7027	**849.4141**	425.2107	A	**365.2183**	**183.1128**	348.1918	174.5995			3
8	**1014.4931**	507.7502	997.4666	499.2369	996.4825	498.7449	F	**294.1812**	147.5942	**277.1547**	139.0810			2
9							K	147.1128	74.0600	130.0863	65.5468			1

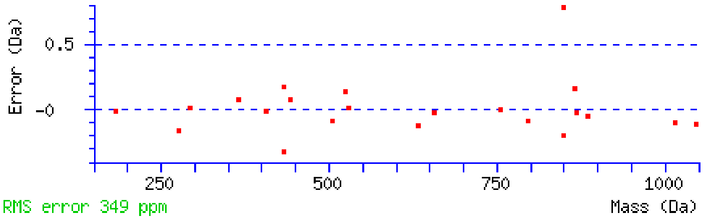

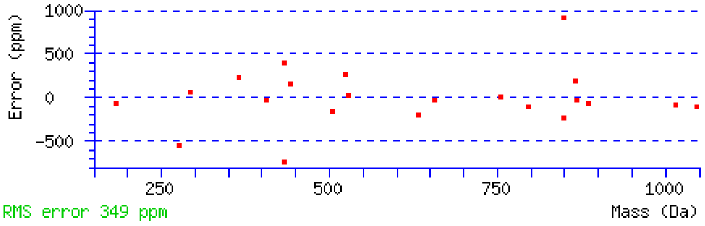

All matches to this query

Score	Mr(calc):	Delta	Sequence

57.4	1159.5913	0.0065	LYQVEYAFK
14.7	1159.5890	0.0089	LYKTLSEVK
14.0	1159.5890	0.0089	LYKTLSEVK
13.1	1159.5986	-0.0007	EFKAGGEVPAR
12.1	1159.5985	-0.0007	ETLRAWQEK
10.7	1158.6049	0.9929	KTFSLVKEK
10.7	1158.6049	0.9929	KTFSLVKEK
8.6	1159.5873	0.0105	YIQAEPPTNK
8.1	1159.6026	-0.0047	EFINHYLPK
6.3	1159.6084	-0.0106	EKQLKEEEK

Spectrum No: 21; Query: 6877; Rank: 1

Peptide View

MS/MS Fragmentation of **QTESTSFLEK**
Found in **IPI00029623**, Tax_Id=9606 Gene_Symbol=PSMA6 Proteasome subunit alpha type-6

Match to Query 6877: 1168.565848 from(585.290200,2+)
Title: 091224LimSK_Exosome3_07.1131.1131.2.dta
Data file C:\Temp\Mascoti\LSK_2D\091224LimSK_Exosome3_01_2D.mgf

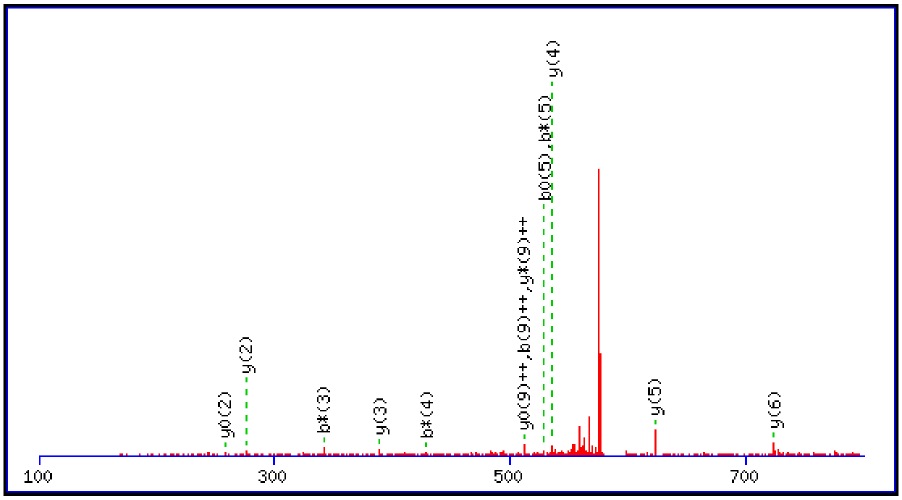

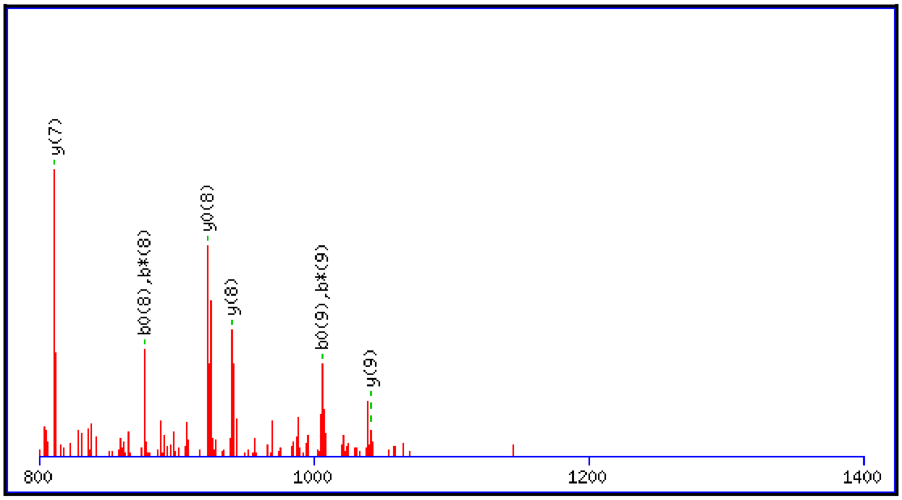

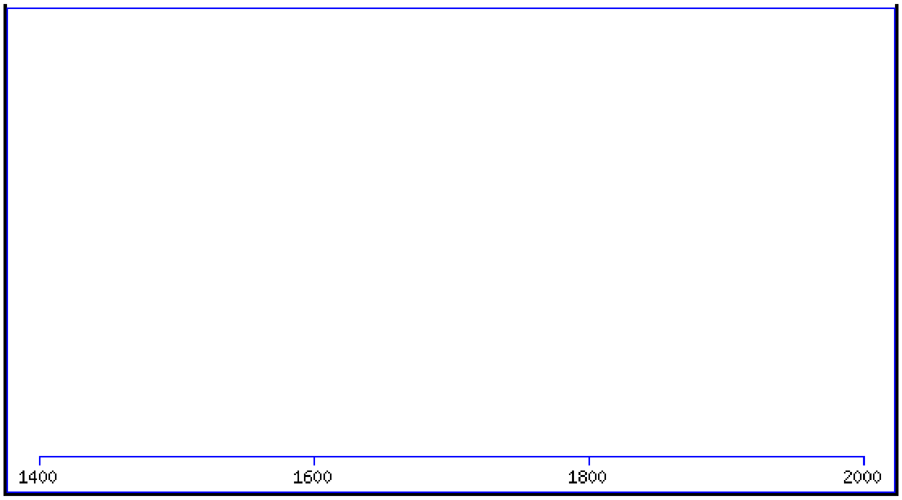

Monoisotopic mass of neutral peptide Mr(calc): 1168.5612
Fixed modifications: Carbamidomethyl (C)
Ions Score: 25 **Expect:** 0.58
Matches (Bold Red): 21/104 fragment ions using 52 most intense peaks

#	b	b++	b*	b*++	b0	b0++	Seq.	y	y++	y*	y*++	y0	y0++	#
1	129.0659	65.0366	112.0393	56.5233			Q							10
2	230.1135	115.5604	213.0870	107.0471	212.1030	106.5551	T	**1041.5099**	521.2586	1024.4833	**512.7453**	1023.4993	**512.2533**	9
3	359.1561	180.0817	**342.1296**	171.5684	341.1456	171.0764	E	**940.4622**	470.7347	923.4357	462.2215	**922.4516**	461.7295	8
4	446.1882	223.5977	**429.1616**	215.0844	428.1776	214.5924	S	**811.4196**	406.2134	794.3931	397.7002	793.4090	397.2082	7
5	547.2358	274.1216	**530.2093**	265.6083	**529.2253**	265.1163	T	**724.3876**	362.6974	707.3610	354.1842	706.3770	353.6921	6
6	634.2679	317.6376	617.2413	309.1243	616.2573	308.6323	S	**623.3399**	312.1736	606.3134	303.6603	605.3293	303.1683	5
7	781.3363	391.1718	764.3097	382.6585	763.3257	382.1665	F	**536.3079**	268.6576	519.2813	260.1443	518.2973	259.6523	4
8	894.4203	447.7138	**877.3938**	439.2005	**876.4098**	438.7085	L	**389.2395**	195.1234	372.2129	186.6101	371.2289	186.1181	3
9	1023.4629	**512.2351**	**1006.4364**	503.7218	**1005.4524**	503.2298	E	**276.1554**	138.5813	259.1288	130.0681	**258.1448**	129.5761	2
10							K	147.1128	74.0600	130.0863	65.5468			1

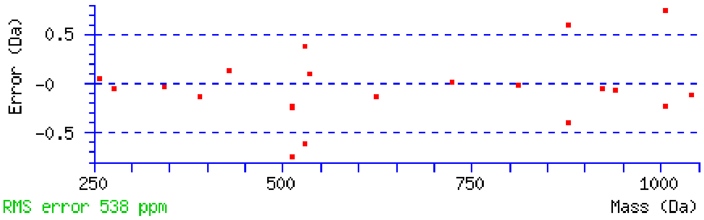

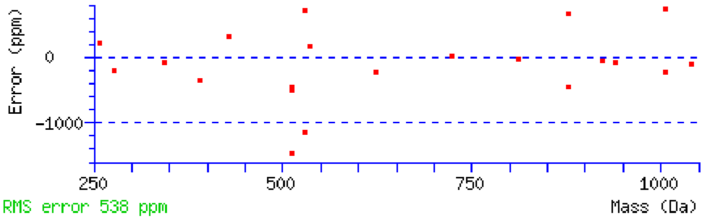

All matches to this query

Score	Mr(calc):	Delta	Sequence
25.1	1168.5612	0.0047	QTESTSFLEK
11.1	1168.5741	-0.0082	EKTEGVVSIK
10.0	1168.5601	0.0057	AARSAAGASSLK
9.5	1167.5509	1.0149	XQSRTGARAK
9.2	1168.5740	-0.0082	ESVLGEKSLK
8.9	1167.5594	1.0065	DKGKFEDMAK
8.3	1168.5641	0.0017	RLASSWLEK
8.0	1167.5723	0.9936	MAAVKTLNPK
7.5	1167.5509	1.0149	DLNTRARSR
7.4	1168.5641	0.0017	RLASSWLEK

Spectrum No: 22; Query: 19659; Rank: 1

Peptide View

MS/MS Fragmentation of **YGYEIPVDMLCK**
Found in **IPI00029623**, Tax_Id=9606 Gene_Symbol=PSMA6 Proteasome subunit alpha type-6

Match to Query 19659: 1486.690048 from(744.352300,2+)
Title: 091224LimSK_Exosome3_06.5785.5785.2.dta
Data file C:\Temp\Mascoti\LSK_2D\091224LimSK_Exosome3_01_2D.mgf

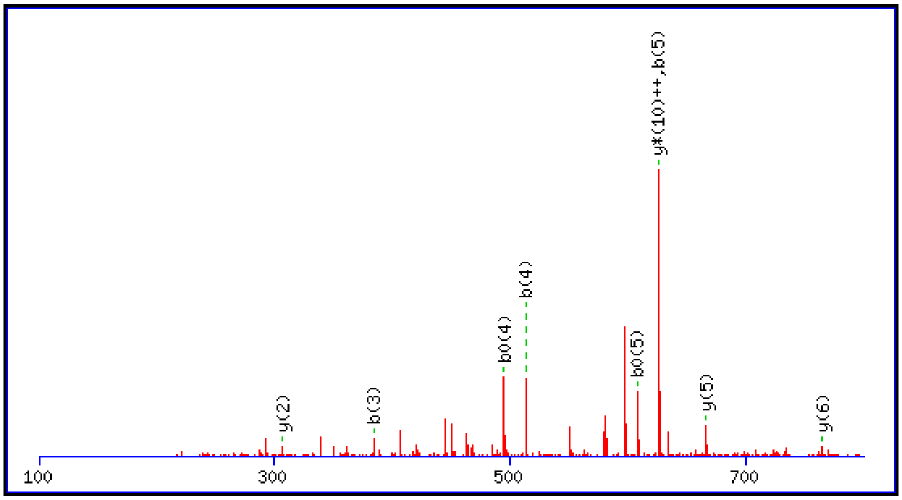

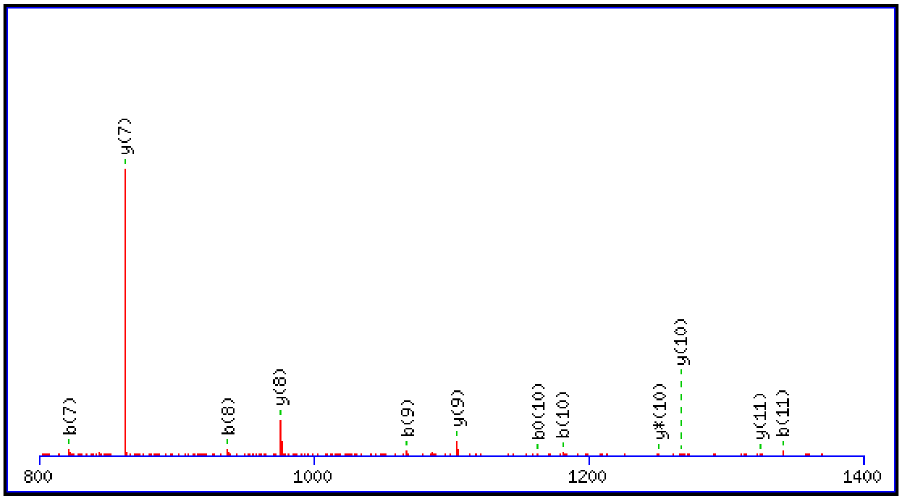

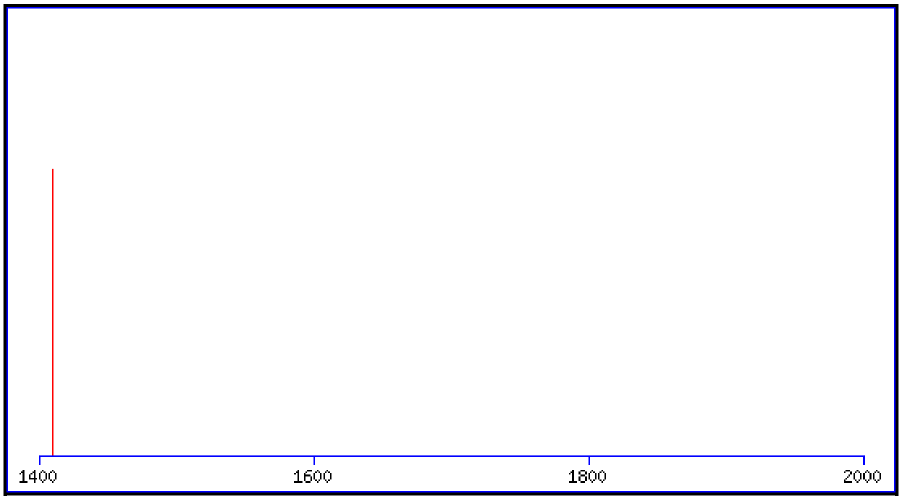

Monoisotopic mass of neutral peptide Mr(calc): 1486.6836
Fixed modifications: Carbamidomethyl (C)
Ions Score: 67 **Expect:** 4.4e-005

Matches (Bold Red): 21/96 fragment ions using 37 most intense peaks

#	b	b^{++}	b^{0}	b^{0++}	Seq.	y	y^{++}	y*	y^{*++}	y^{0}	y^{0++}	#
1	164.0706	82.5389			Y							12
2	221.0921	111.0497			G	**1324.6276**	662.8174	1307.6010	654.3042	1306.6170	653.8121	11
3	**384.1554**	192.5813			Y	**1267.6061**	634.3067	**1250.5796**	**625.7934**	1249.5955	625.3014	10
4	**513.1980**	257.1026	**495.1874**	248.0974	E	**1104.5428**	552.7750	1087.5162	544.2618	1086.5322	543.7697	9
5	**626.2821**	313.6447	**608.2715**	304.6394	I	**975.5002**	488.2537	958.4736	479.7405	957.4896	479.2484	8
6	723.3348	362.1710	705.3243	353.1658	P	**862.4161**	431.7117	845.3896	423.1984	844.4056	422.7064	7
7	**822.4032**	411.7053	804.3927	402.7000	V	**765.3634**	383.1853	748.3368	374.6720	747.3528	374.1800	6
8	**937.4302**	469.2187	919.4196	460.2134	D	**666.2949**	333.6511	649.2684	325.1378	648.2844	324.6458	5
9	**1068.4707**	534.7390	1050.4601	525.7337	M	551.2680	276.1376	534.2415	267.6244			4
10	**1181.5547**	591.2810	**1163.5442**	582.2757	L	420.2275	210.6174	403.2010	202.1041			3
11	**1341.5854**	671.2963	1323.5748	662.2910	C	**307.1435**	154.0754	290.1169	145.5621			2
12					K	147.1128	74.0600	130.0863	65.5468			1

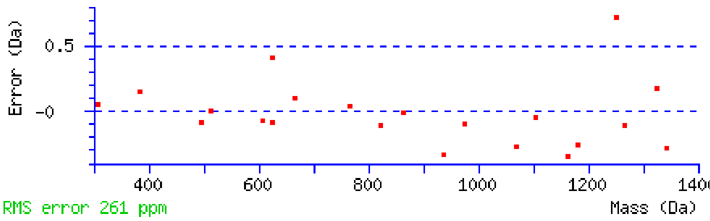

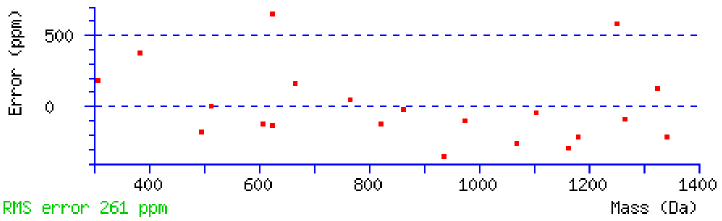

All matches to this query

Score	Mr(calc):	Delta	Sequence
66.6	1486.6836	0.0064	YGYEIPVDMLCK
14.2	1485.6993	0.9907	AVKDLASPLIGRS
6.9	1486.6929	-0.0029	ARGPQPSPPGSSAAK
6.9	1486.6929	-0.0029	ARGPQPSPPGSSAAK
6.6	1486.6833	0.0067	AITSPLAAGSKPSK
5.7	1486.6907	-0.0007	LPATSMAIPLIGK
5.6	1486.6833	0.0067	AITSPLAAGSKPSK
4.0	1484.6813	2.0087	AFNWSSSLTKHK
4.0	1484.6813	2.0087	AFNWSSSLTKHK
3.0	1486.6830	0.0070	NSHRIGPHQSFK

Spectrum No: 23; Query: 25974; Rank: 1

Peptide View

MS/MS Fragmentation of **ATCIGNNSAAAVSMLK**
Found in **IPI00299155**, Tax_Id=9606 Gene_Symbol=PSMA4 Proteasome subunit alpha type-4

Match to Query 25974: 1606.790368 from(804.402460,2+)
Title: 091224LimSK_Exosome3_06.3300.3300.2.dta
Data file C:\Temp\Mascoti\LSK_2D\091224LimSK_Exosome3_01_2D.mgf

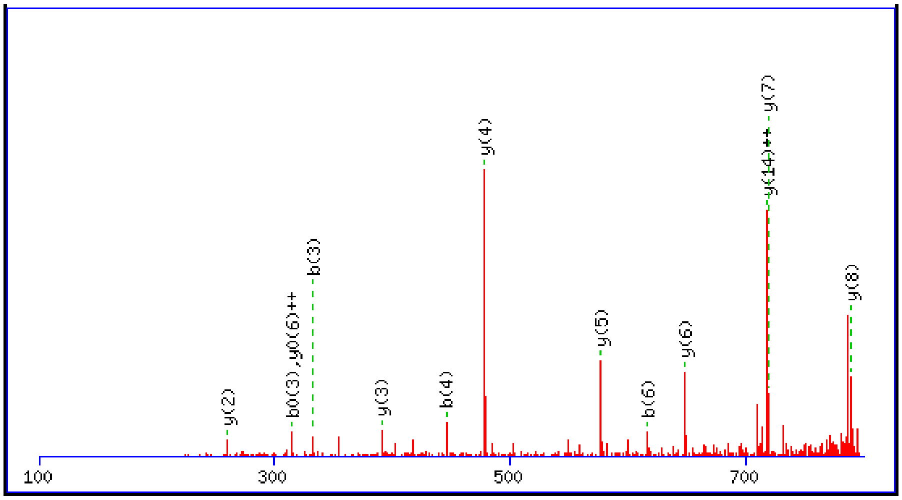

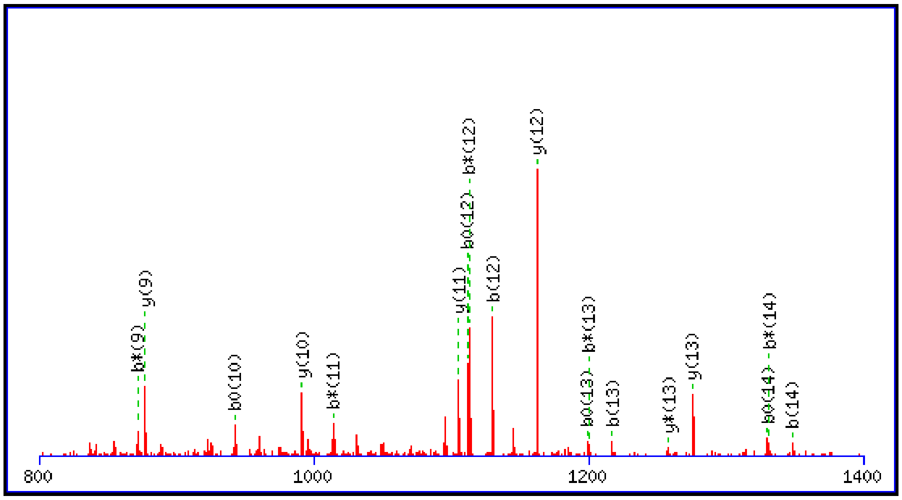

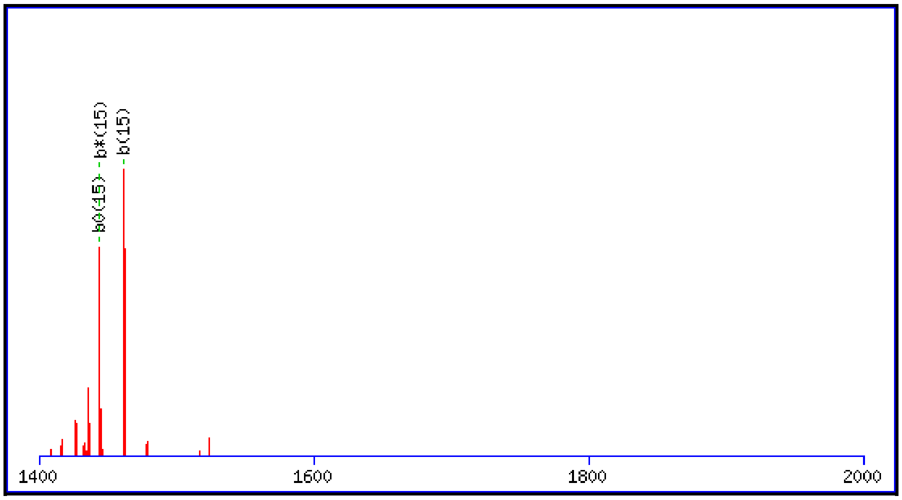

Monoisotopic mass of neutral peptide Mr(calc): 1606.7807
Fixed modifications: Carbamidomethyl (C)
Ions Score: 101 **Expect:** 1.6e-008
Matches (Bold Red): 34/162 fragment ions using 48 most intense peaks

#	b	b^{++}	b*	b^{*++}	b^{0}	b^{0++}	Seq.	y	y^{++}	y*	y^{*++}	y^{0}	y^{0++}	#
1	72.0444	36.5258					A							16
2	173.0921	87.0497			155.0815	78.0444	T	1536.7509	768.8791	1519.7243	760.3658	1518.7403	759.8738	15
3	**333.1227**	167.0650			**315.1122**	158.0597	C	1435.7032	**718.3552**	1418.6766	709.8420	1417.6926	709.3500	14
4	**446.2068**	223.6070			428.1962	214.6017	I	**1275.6725**	638.3399	**1258.6460**	629.8266	1257.6620	629.3346	13
5	503.2282	252.1178			485.2177	243.1125	G	**1162.5885**	581.7979	1145.5619	573.2846	1144.5779	572.7926	12
6	**617.2712**	309.1392	600.2446	300.6260	599.2606	300.1339	N	**1105.5670**	553.2871	1088.5405	544.7739	1087.5565	544.2819	11

7	731.3141	366.1607	714.2876	357.6474	713.3035	357.1554	N	991.5241	496.2657	974.4975	487.7524	973.5135	487.2604	10
8	818.3461	409.6767	801.3196	401.1634	800.3356	400.6714	S	877.4812	439.2442	860.4546	430.7309	859.4706	430.2389	9
9	889.3832	445.1953	872.3567	436.6820	871.3727	436.1900	A	790.4491	395.7282	773.4226	387.2149	772.4386	386.7229	8
10	960.4204	480.7138	943.3938	472.2005	942.4098	471.7085	A	719.4120	360.2096	702.3855	351.6964	701.4015	351.2044	7
11	1031.4575	516.2324	1014.4309	507.7191	1013.4469	507.2271	A	648.3749	324.6911	631.3484	316.1778	630.3643	315.6858	6
12	1130.5259	565.7666	1113.4993	557.2533	1112.5153	556.7613	V	577.3378	289.1725	560.3112	280.6593	559.3272	280.1673	5
13	1217.5579	609.2826	1200.5314	600.7693	1199.5473	600.2773	S	478.2694	239.6383	461.2428	231.1251	460.2588	230.6330	4
14	1348.5984	674.8028	1331.5718	666.2896	1330.5878	665.7976	M	391.2374	196.1223	374.2108	187.6090			3
15	1461.6825	731.3449	1444.6559	722.8316	1443.6719	722.3396	L	260.1969	130.6021	243.1703	122.0888			2
16							K	147.1128	74.0600	130.0863	65.5468			1

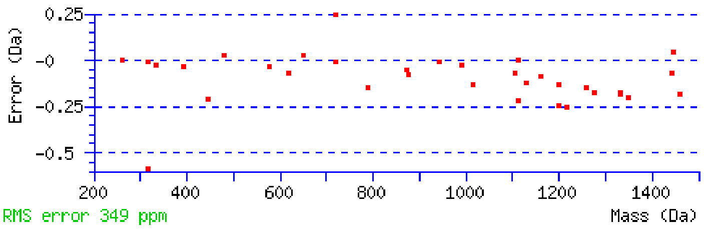

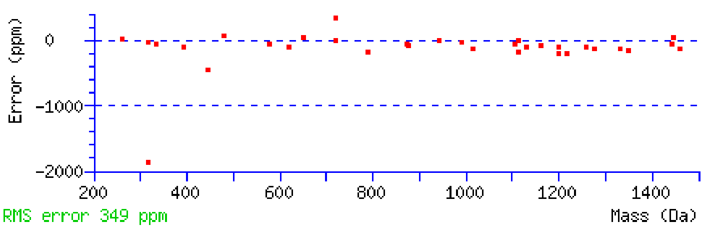

All matches to this query

Score	Mr(calc):	Delta	Sequence
100.9	1606.7807	0.0097	ATCIGNNSAAAVSMLK
15.4	1606.7838	0.0065	YINSTLPNDSENIK
10.3	1606.7913	-0.0009	FVGTDPASDEVVLMK
9.4	1606.8058	-0.0155	NSLQKMLEVCELK
9.3	1606.7991	-0.0088	FQDSSQWLQIDLK
8.1	1605.8007	0.9896	MYQGHMQVVGVTLK
7.9	1606.7774	0.0130	VCTSVTHSFISAGNK
6.0	1605.8007	0.9896	MYQGHMQVVGVTLK
3.6	1606.8025	-0.0121	SLTTSQYLMHEVAK
2.7	1606.7908	-0.0005	LGFSKITYREWK

Spectrum No: 24; Query: 8739; Rank: 1

Peptide View

MS/MS Fragmentation of **LLDEVFFSEK**
Found in **IPI00299155**, Tax_Id=9606 Gene_Symbol=PSMA4 Proteasome subunit alpha type-4

Match to Query 8739: 1225.629288 from(613.821920,2+)
Title: 091224LimSK_Exosome3_06.5499.5499.2.dta
Data file C:\Temp\Mascoti\LSK_2D\091224LimSK_Exosome3_01_2D.mgf

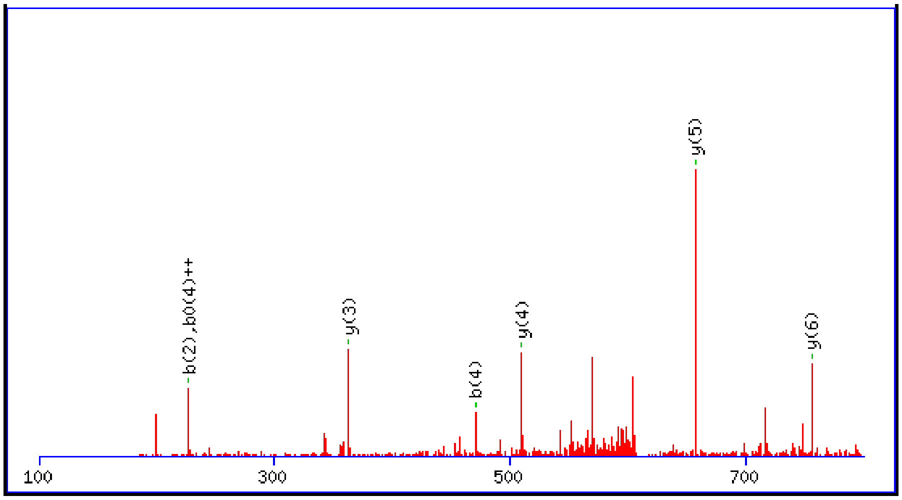

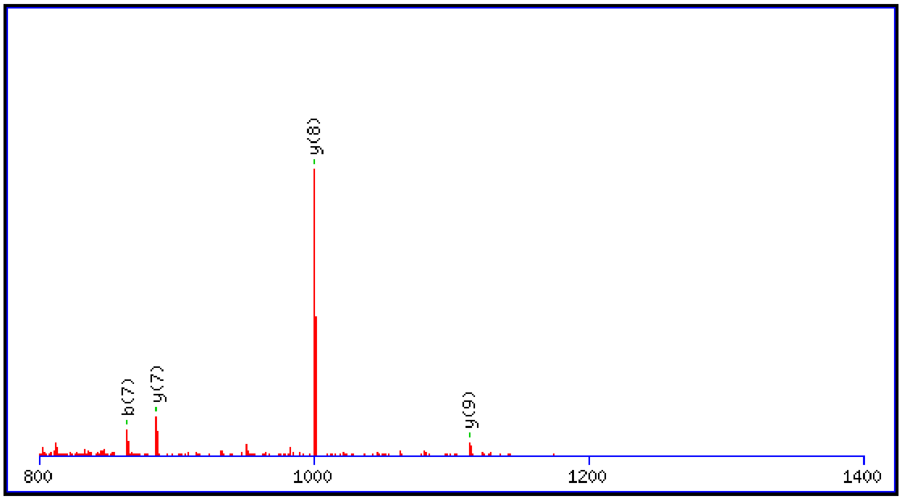

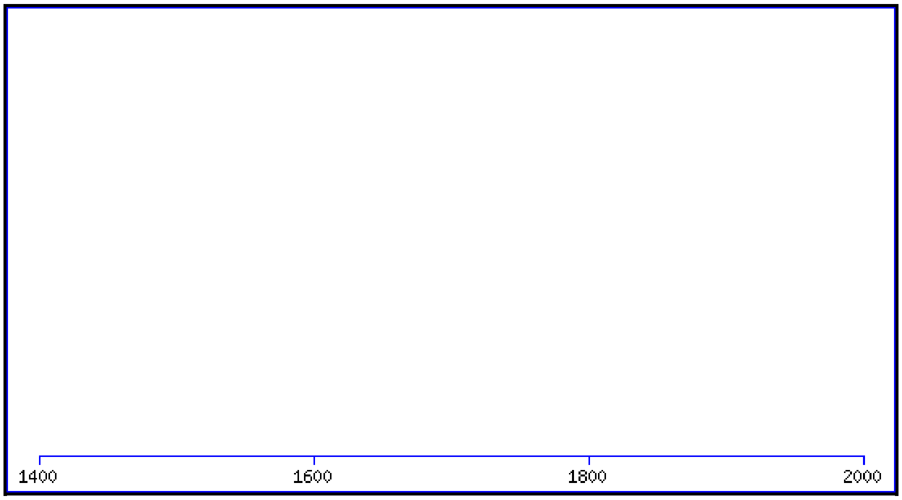

Monoisotopic mass of neutral peptide Mr(calc): 1225.6230
Fixed modifications: Carbamidomethyl (C)
Ions Score: 73 **Expect:** 8.2e-006
Matches (Bold Red): 11/84 fragment ions using 11 most intense peaks

#	b	b^{++}	b^{0}	b^{0++}	Seq.	y	y^{++}	y*	y^{*++}	y^{0}	y^{0++}	#
1	114.0913	57.5493			L							10
2	**227.1754**	114.0913			L	**1113.5463**	557.2768	1096.5197	548.7635	1095.5357	548.2715	9
3	342.2023	171.6048	324.1918	162.5995	D	**1000.4622**	500.7347	983.4357	492.2215	982.4516	491.7295	8
4	**471.2449**	236.1261	453.2344	**227.1208**	E	**885.4353**	443.2213	868.4087	434.7080	867.4247	434.2160	7
5	570.3134	285.6603	552.3028	276.6550	V	**756.3927**	378.7000	739.3661	370.1867	738.3821	369.6947	6
6	717.3818	359.1945	699.3712	350.1892	F	**657.3243**	329.1658	640.2977	320.6525	639.3137	320.1605	5

**7**	**864.4502**	432.7287	846.4396	423.7234	**F**	**510.2558**	255.6316	493.2293	247.1183	492.2453	246.6263	**4**
**8**	951.4822	476.2447	933.4716	467.2395	**S**	**363.1874**	182.0974	346.1609	173.5841	345.1769	173.0921	**3**
**9**	1080.5248	540.7660	1062.5142	531.7608	**E**	276.1554	138.5813	259.1288	130.0681	258.1448	129.5761	**2**
**10**					**K**	147.1128	74.0600	130.0863	65.5468			**1**

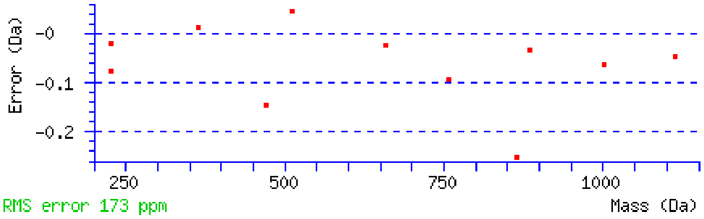

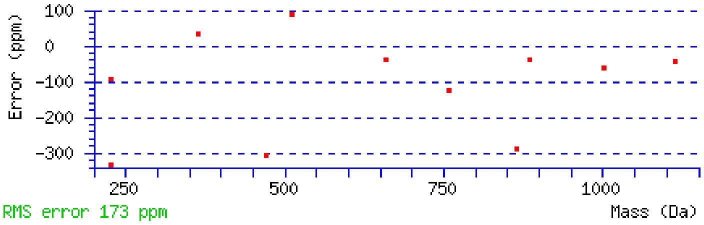

All matches to this query

Score	Mr(calc):	Delta	Sequence
72.6	1225.6230	0.0062	LLDEVFFSEK
6.8	1223.6275	2.0018	NKKVDAAASIK
5.1	1225.6302	-0.0010	VHSEVASLQEK
3.6	1225.6343	-0.0050	EPFPPSSPLQK
3.1	1225.6349	-0.0057	LIHDTNMRAR
2.5	1224.6172	1.0121	MPDPAKSAPAPK
2.1	1224.6340	0.9953	KLTGAVRSSAR
2.1	1224.6340	0.9953	KLTGAVRSSAR
1.6	1225.6292	0.0001	SLRIASRSTR
1.5	1225.6254	0.0039	ILSACVLSKR

Spectrum No: 25; Query: 58319; Rank: 1

Peptide View

MS/MS Fragmentation of **LNEDMACSVAGITSDANVLTNELR**
Found in **IPI00299155**, Tax_Id=9606 Gene_Symbol=PSMA4 Proteasome subunit alpha type-4

Match to Query 58319: 2592.237888 from(1297.126220,2+)
Title: 091224LimSK_Exosome3_05.7610.7610.2.dta
Data file C:\Temp\Mascoti\LSK_2D\091224LimSK_Exosome3_01_2D.mgf

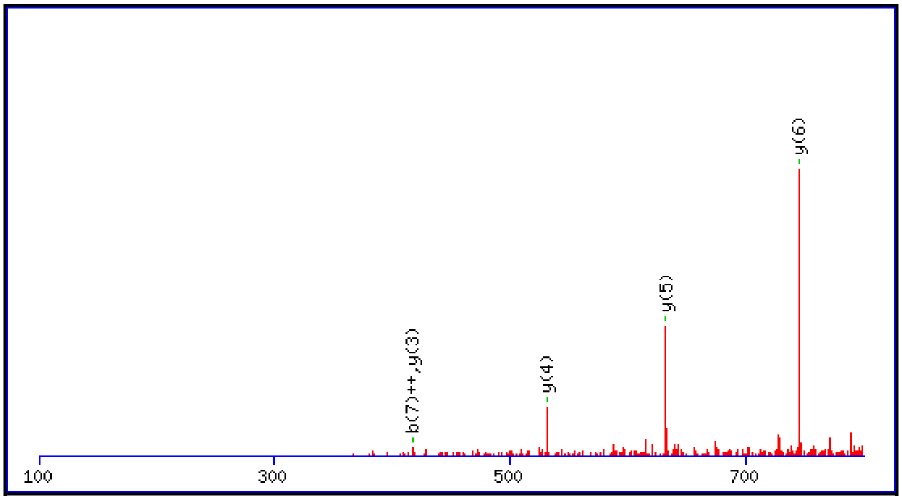

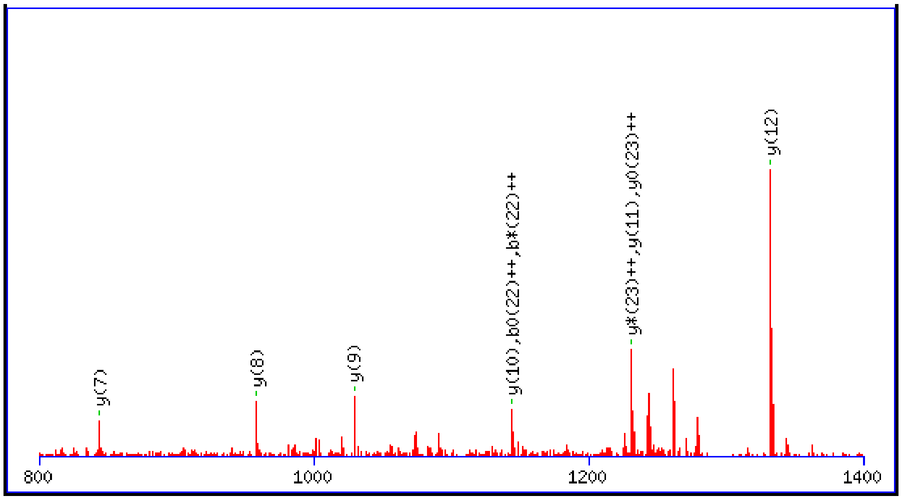

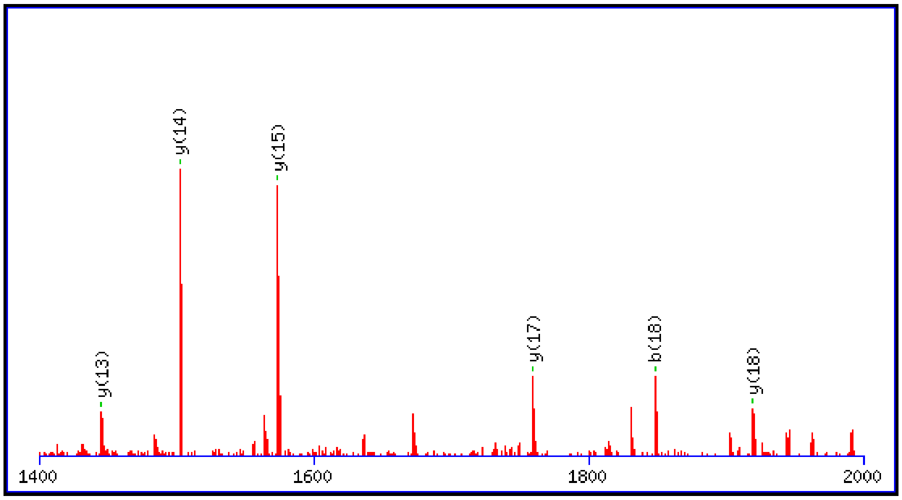

Monoisotopic mass of neutral peptide Mr(calc): 2592.2159
Fixed modifications: Carbamidomethyl (C)
Ions Score: 144 **Expect:** 9.4e-013
Matches (Bold Red): 21/266 fragment ions using 24 most intense peaks

#	b	b^{++}	b^{*}	b^{*++}	b^{0}	b^{0++}	Seq.	y	y^{++}	y^{*}	y^{*++}	y^{0}	y^{0++}	#
1	114.0913	57.5493					L							24
2	228.1343	114.5708	211.1077	106.0575			N	2480.1392	1240.5732	2463.1126	**1232.0600**	2462.1286	**1231.5679**	23
3	357.1769	179.0921	340.1503	170.5788	339.1663	170.0868	E	2366.0963	1183.5518	2349.0697	1175.0385	2348.0857	1174.5465	22
4	472.2038	236.6055	455.1773	228.0923	454.1932	227.6003	D	2237.0537	1119.0305	2220.0271	1110.5172	2219.0431	1110.0252	21
5	603.2443	302.1258	586.2177	293.6125	585.2337	293.1205	M	2122.0267	1061.5170	2105.0002	1053.0037	2104.0162	1052.5117	20
6	674.2814	337.6443	657.2549	329.1311	656.2708	328.6391	A	1990.9862	995.9968	1973.9597	987.4835	1972.9757	986.9915	19
7	834.3121	**417.6597**	817.2855	409.1464	816.3015	408.6544	C	**1919.9491**	960.4782	1902.9226	951.9649	1901.9386	951.4729	18
8	921.3441	461.1757	904.3175	452.6624	903.3335	452.1704	S	**1759.9185**	880.4629	1742.8919	871.9496	1741.9079	871.4576	17
9	1020.4125	510.7099	1003.3859	502.1966	1002.4019	501.7046	V	1672.8864	836.9469	1655.8599	828.4336	1654.8759	827.9416	16
10	1091.4496	546.2284	1074.4231	537.7152	1073.4390	537.2232	A	**1573.8180**	787.4127	1556.7915	778.8994	1555.8075	778.4074	15
11	1148.4711	574.7392	1131.4445	566.2259	1130.4605	565.7339	G	**1502.7809**	751.8941	1485.7544	743.3808	1484.7703	742.8888	14
12	1261.5551	631.2812	1244.5286	622.7679	1243.5446	622.2759	I	**1445.7594**	723.3834	1428.7329	714.8701	1427.7489	714.3781	13
13	1362.6028	681.8050	1345.5763	673.2918	1344.5922	672.7998	T	**1332.6754**	666.8413	1315.6488	658.3281	1314.6648	657.8360	12
14	1449.6348	725.3211	1432.6083	716.8078	1431.6243	716.3158	S	**1231.6277**	616.3175	1214.6012	607.8042	1213.6171	607.3122	11
15	1564.6618	782.8345	1547.6352	774.3213	1546.6512	773.8292	D	**1144.5957**	572.8015	1127.5691	564.2882	1126.5851	563.7962	10
16	1635.6989	818.3531	1618.6724	809.8398	1617.6883	809.3478	A	**1029.5687**	515.2880	1012.5422	506.7747	1011.5582	506.2827	9
17	1749.7418	875.3746	1732.7153	866.8613	1731.7313	866.3693	N	**958.5316**	479.7694	941.5051	471.2562	940.5211	470.7642	8
18	**1848.8102**	924.9088	1831.7837	916.3955	1830.7997	915.9035	V	**844.4887**	422.7480	827.4621	414.2347	826.4781	413.7427	7
19	1961.8943	981.4508	1944.8678	972.9375	1943.8837	972.4455	L	**745.4203**	373.2138	728.3937	364.7005	727.4097	364.2085	6

**20**	2062.9420	1031.9746	2045.9154	1023.4614	2044.9314	1022.9693	**T**	**632.3362**	316.6717	615.3097	308.1585	614.3257	307.6665	**5**
**21**	2176.9849	1088.9961	2159.9584	1080.4828	2158.9743	1079.9908	**N**	**531.2885**	266.1479	514.2620	257.6346	513.2780	257.1426	**4**
**22**	2306.0275	1153.5174	2289.0010	**1145.0041**	2288.0169	**1144.5121**	**E**	**417.2456**	209.1264	400.2191	200.6132	399.2350	200.1212	**3**
**23**	2419.1116	1210.0594	2402.0850	1201.5461	2401.1010	1201.0541	**L**	288.2030	144.6051	271.1765	136.0919			**2**
**24**							**R**	175.1190	88.0631	158.0924	79.5498			**1**

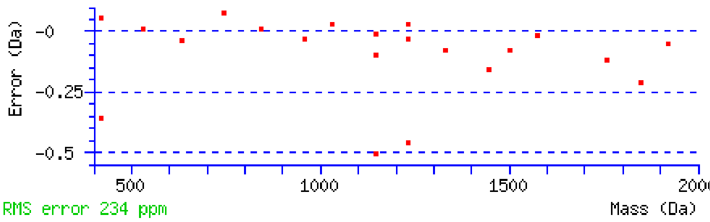

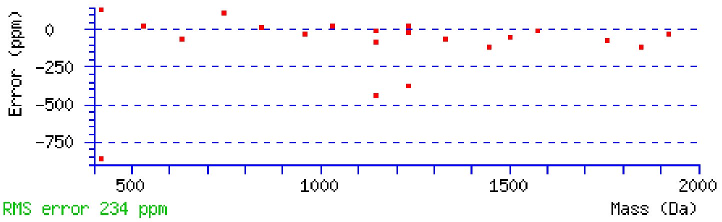

All matches to this query

Score	Mr(calc):	Delta	Sequence
144.4	2592.2159	0.0219	LNEDMACSVAGITSDANVLTNELR
5.3	2590.2388	1.9991	NADLTVHRLGALSLGDGGTTATNEK
4.5	2592.2389	-0.0010	YIWICWFAALAAGGAYIAGVSPR
4.1	2590.2332	2.0046	EPAPTTPKGTAPTTLKEPAPTTPK
3.9	2592.2287	0.0092	VSKVILPLTLSISCPCFSHFK
2.9	2591.2219	1.0160	QSARKPLLGLPSYMLQSEELR
1.9	2592.2226	0.0153	HANRANKCIFIFVSILTWSR
1.9	2592.2226	0.0153	HANRANKCIFIFVSILTWSR
1.7	2592.2185	0.0194	VMSAQLTMAKTSGAMPTGSMKAVAK
1.7	2592.2185	0.0194	VMSAQLTMAKTSGAMPTGSMKAVAK

Spectrum No: 26; Query: 215; Rank: 1

Peptide View

MS/MS Fragmentation of **SALALAIK**
Found in **IPI00299155**, Tax_Id=9606 Gene_Symbol=PSMA4 Proteasome subunit alpha type-4

Match to Query 215: 785.504788 from(393.759670,2+)
Title: 091224LimSK_Exosome3_06.2155.2155.2.dta
Data file C:\Temp\Mascoti\LSK_2D\091224LimSK_Exosome3_01_2D.mgf

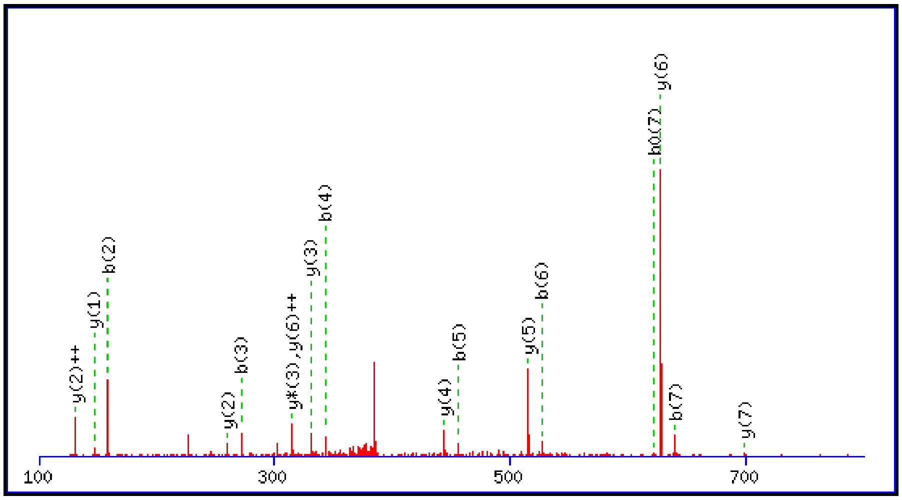

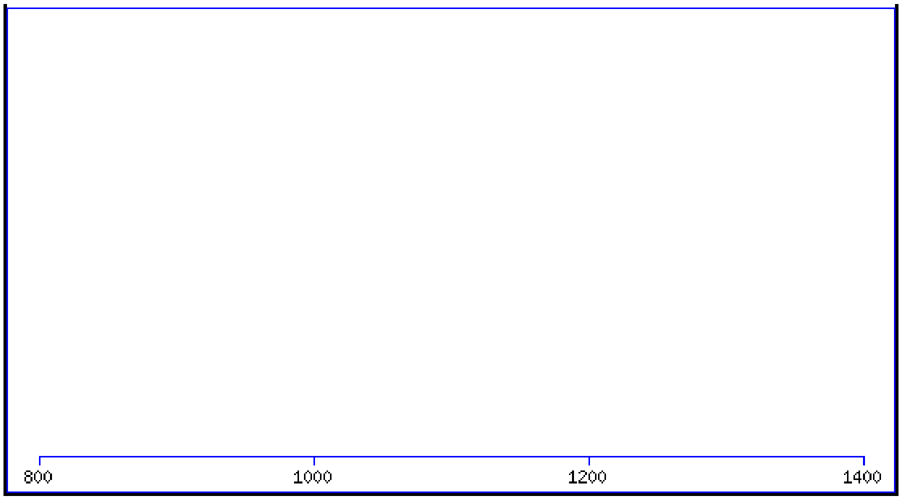

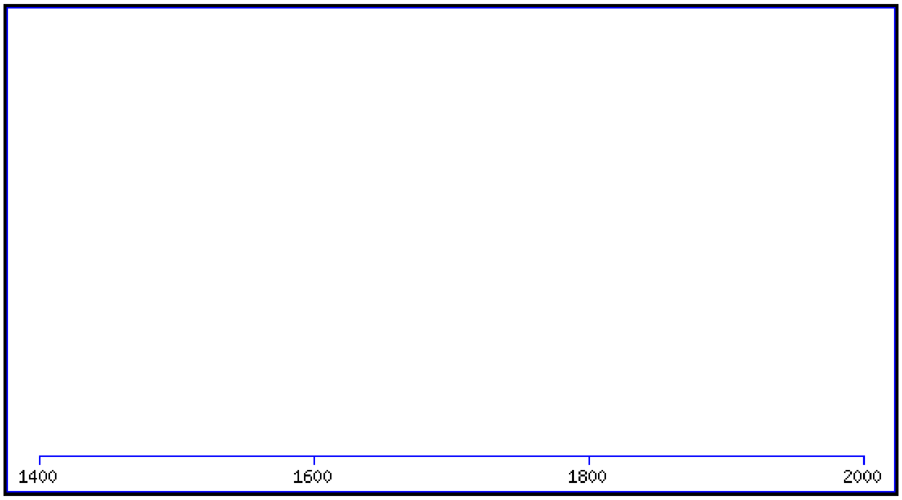

Monoisotopic mass of neutral peptide Mr(calc): 785.5011
Fixed modifications: Carbamidomethyl (C)
Ions Score: 52 **Expect:** 0.00029
Matches (Bold Red): 17/56 fragment ions using 34 most intense peaks

#	b	b^{++}	b^{0}	b^{0++}	Seq.	y	y^{++}	y*	y^{*++}	#
1	88.0393	44.5233	70.0287	35.5180	S					8
2	**159.0764**	80.0418	141.0659	71.0366	A	**699.4763**	350.2418	682.4498	341.7285	7
3	**272.1605**	136.5839	254.1499	127.5786	L	**628.4392**	**314.7233**	611.4127	306.2100	6
4	**343.1976**	172.1024	325.1870	163.0972	A	**515.3552**	258.1812	498.3286	249.6679	5
5	**456.2817**	228.6445	438.2711	219.6392	L	**444.3180**	222.6627	427.2915	214.1494	4
6	**527.3188**	264.1630	509.3082	255.1577	A	**331.2340**	166.1206	**314.2074**	157.6074	3
7	**640.4028**	320.7051	**622.3923**	311.6998	I	**260.1969**	**130.6021**	243.1703	122.0888	2
8					K	**147.1128**	74.0600	130.0863	65.5468	1

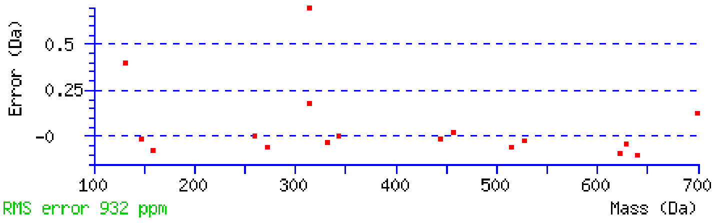

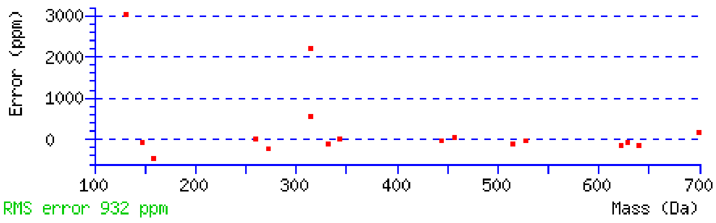

All matches to this query

Score	Mr(calc):	Delta	Sequence
51.8	785.5011	0.0037	SALALAIK

24.6	785.5011	0.0037	GLTLAALK
21.2	785.5123	-0.0075	GTLVRIK
20.2	783.4966	2.0081	RLSPALK
17.5	785.5123	-0.0075	TGLIRVK
17.0	785.5123	-0.0075	GTRVLLK
16.6	785.5123	-0.0075	XSRLALK
13.9	785.5123	-0.0075	KKQAALK
11.5	785.5123	-0.0075	SRVAILK
8.6	785.5011	0.0037	KVDALLK

Spectrum No: 27; Query: 2884; Rank: 1

Peptide View

MS/MS Fragmentation of **TTIFSPEGR**
Found in **IPI00299155**, Tax_Id=9606 Gene_Symbol=PSMA4 Proteasome subunit alpha type-4

Match to Query 2884: 1006.513488 from(504.264020,2+)
Title: 091224LimSK_Exosome3_06.1415.1415.2.dta
Data file C:\Temp\Mascoti\LSK_2D\091224LimSK_Exosome3_01_2D.mgf

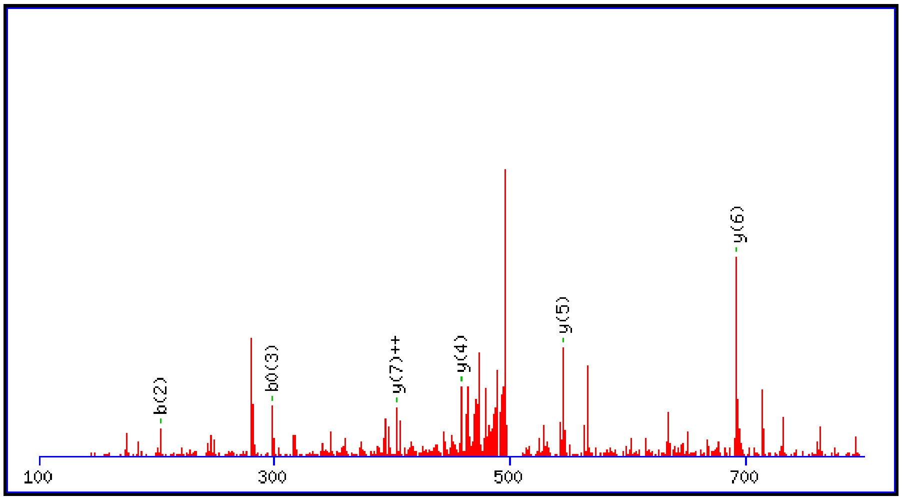

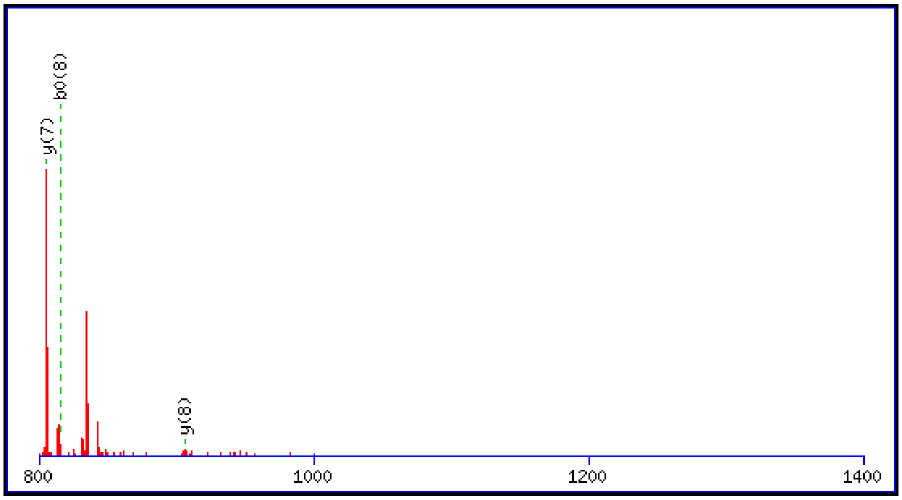

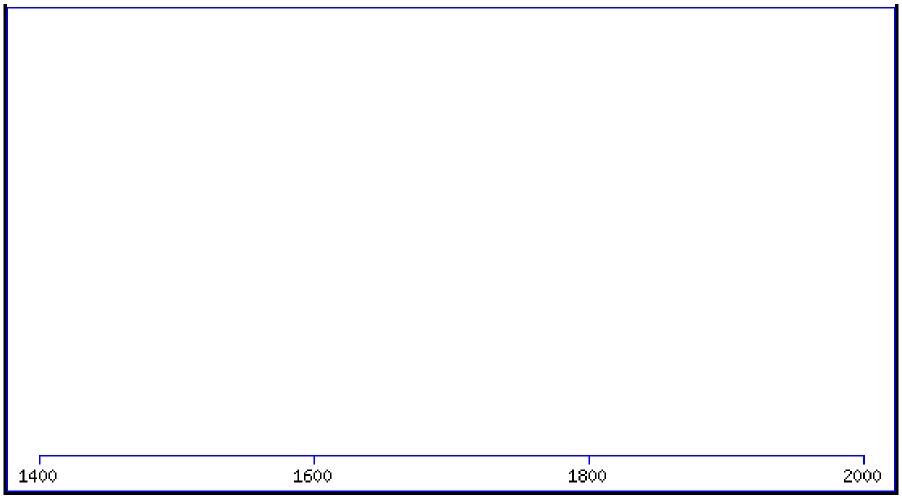

Monoisotopic mass of neutral peptide Mr(calc): 1006.5084
Fixed modifications: Carbamidomethyl (C)
Ions Score: 22 **Expect:** 0.91
Matches (Bold Red): 9/76 fragment ions using 33 most intense peaks

#	b	b^{++}	b^0	b^{0++}	Seq.	y	y^{++}	y*	y^{*++}	y^0	y^{0++}	#
1	102.0550	51.5311	84.0444	42.5258	T							9
2	**203.1026**	102.0550	185.0921	93.0497	T	**906.4680**	453.7376	889.4414	445.2243	888.4574	444.7323	8
3	316.1867	158.5970	**298.1761**	149.5917	I	**805.4203**	**403.2138**	788.3937	394.7005	787.4097	394.2085	7
4	463.2551	232.1312	445.2445	223.1259	F	**692.3362**	346.6717	675.3097	338.1585	674.3257	337.6665	6
5	550.2871	275.6472	532.2766	266.6419	S	**545.2678**	273.1375	528.2413	264.6243	527.2572	264.1323	5
6	647.3399	324.1736	629.3293	315.1683	P	**458.2358**	229.6215	441.2092	221.1083	440.2252	220.6162	4
7	776.3825	388.6949	758.3719	379.6896	E	361.1830	181.0951	344.1565	172.5819	343.1724	172.0899	3
8	833.4040	417.2056	**815.3934**	408.2003	G	232.1404	116.5738	215.1139	108.0606			2
9					R	175.1190	88.0631	158.0924	79.5498			1

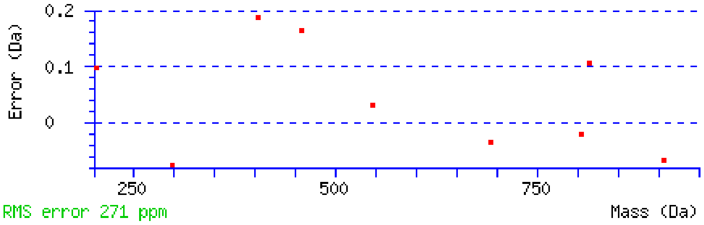

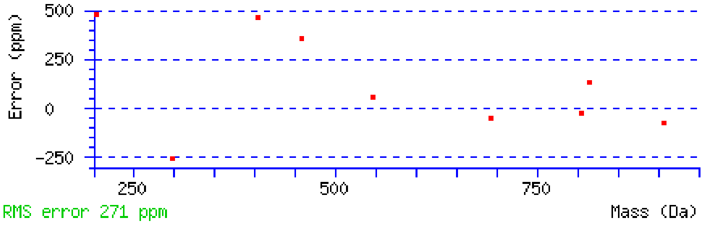

All matches to this query

Score	Mr(calc):	Delta	Sequence
21.6	1006.5084	0.0051	TTIFSPEGR
5.3	1005.5178	0.9957	TGRLPFCR
5.2	1004.5147	1.9988	SLLCMPIR
4.6	1004.5152	1.9983	LSIGHHGER
4.0	1004.5080	2.0055	EAPSFWIR
3.9	1005.5026	1.0109	GGSMGQGLRK
2.2	1005.5025	1.0109	MGGKQSTAAR
1.4	1006.5229	-0.0095	KCSSSVAIR
1.1	1006.5052	0.0083	MMALVRDR
1.1	1006.5052	0.0083	MMALVRDR

Spectrum No: 28; Query: 1296; Rank: 1

Peptide View

MS/MS Fragmentation of **VEIATLTR**
Found in **IPI00299155**, Tax_Id=9606 Gene_Symbol=PSMA4 Proteasome subunit alpha type-4

Match to Query 1296: 901.526888 from(451.770720,2+)
Title: 091224LimSK_Exosome3_07.1534.1534.2.dta
Data file C:\Temp\Mascoti\LSK_2D\091224LimSK_Exosome3_01_2D.mgf

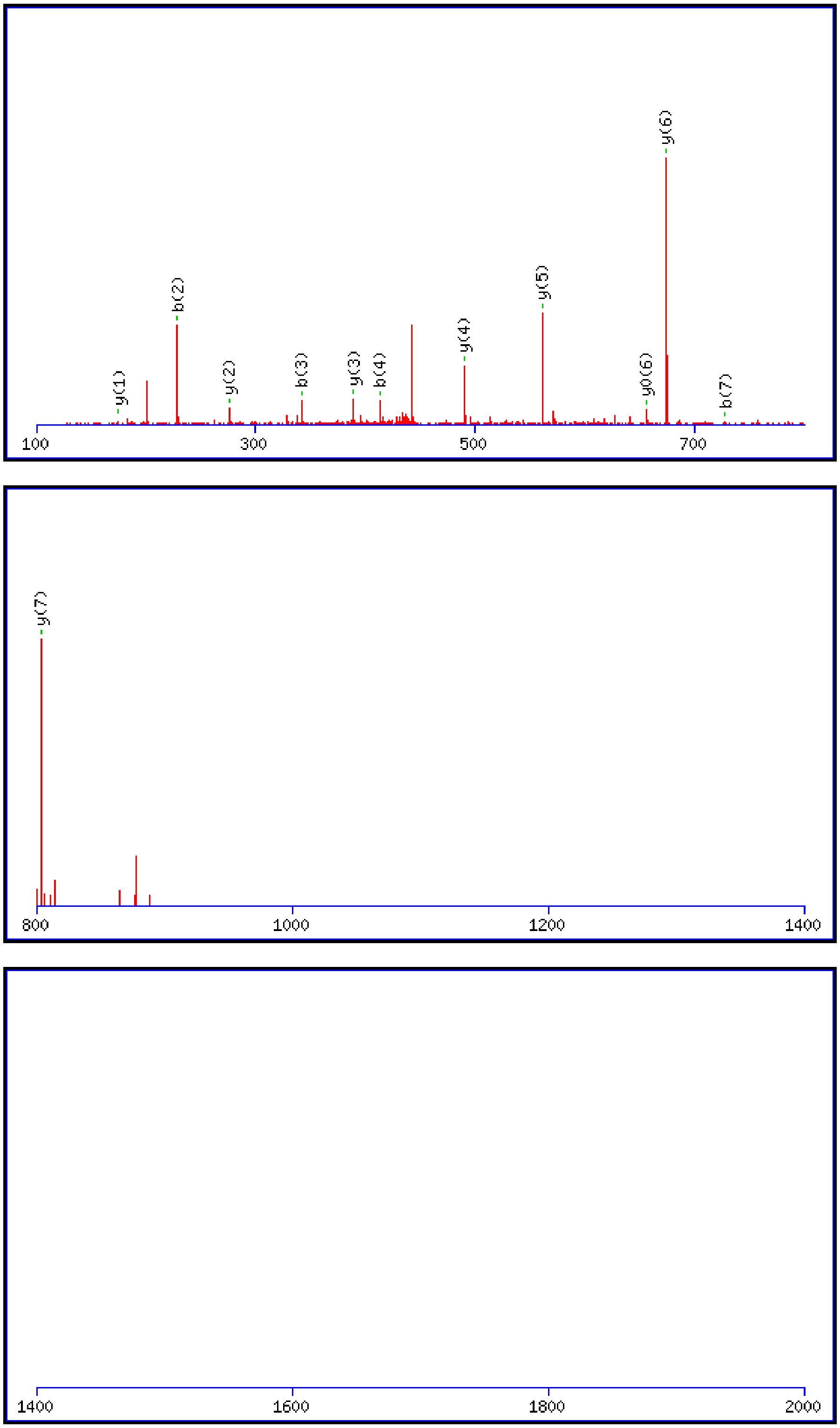

Monoisotopic mass of neutral peptide Mr(calc): 901.5233
Fixed modifications: Carbamidomethyl (C)
Ions Score: 52 **Expect:** 0.00054
Matches (Bold Red): 12/66 fragment ions using 28 most intense peaks

#	b	b^{++}	b^{0}	b^{0++}	Seq.	y	y^{++}	y*	y^{*++}	y^{0}	y^{0++}	#
1	100.0757	50.5415			V							8
2	**229.1183**	115.0628	211.1077	106.0575	E	**803.4621**	402.2347	786.4356	393.7214	785.4516	393.2294	7
3	**342.2023**	171.6048	324.1918	162.5995	I	**674.4196**	337.7134	657.3930	329.2001	**656.4090**	328.7081	6
4	**413.2395**	207.1234	395.2289	198.1181	A	**561.3355**	281.1714	544.3089	272.6581	543.3249	272.1661	5
5	514.2871	257.6472	496.2766	248.6419	T	**490.2984**	245.6528	473.2718	237.1396	472.2878	236.6475	4
6	627.3712	314.1892	609.3606	305.1840	L	**389.2507**	195.1290	372.2241	186.6157	371.2401	186.1237	3
7	**728.4189**	364.7131	710.4083	355.7078	T	**276.1666**	138.5870	259.1401	130.0737	258.1561	129.5817	2
8					R	**175.1190**	88.0631	158.0924	79.5498			1

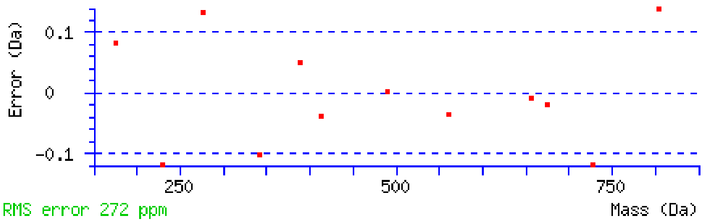

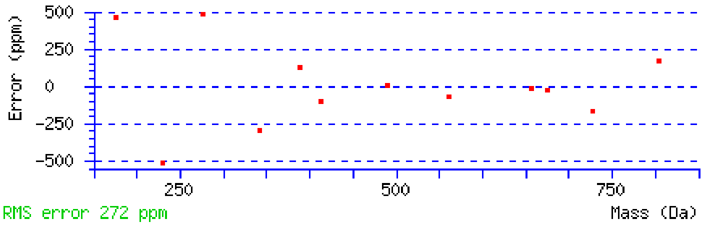

All matches to this query

Score	Mr(calc):	Delta	Sequence
52.4	901.5233	0.0036	VEIATLTR
20.4	901.5233	0.0036	VTLVDVTR
15.2	900.5280	0.9989	GAVIATELK
12.2	901.5233	0.0036	DLVISISR
9.4	900.5181	1.0088	LNLWISR
8.2	901.5345	-0.0076	LVQKTASR
8.1	900.5181	1.0088	KVLWEAR
8.1	901.5233	0.0036	LDLSAKQK
6.2	900.5280	0.9989	AEKLEIAK
5.4	899.5188	2.0080	ILREVDR

Spectrum No: 29; Query: 60146; Rank: 1

Peptide View

MS/MS Fragmentation of **YLLQYQEPIPCEQLVTALCDIK**
Found in **IPI00299155**, Tax_Id=9606 Gene_Symbol=PSMA4 Proteasome subunit alpha type-4

Match to Query 60146: 2693.362302 from(898.794710,3+)
Title: 091224LimSK_Exosome3_05.8322.8322.3.dta
Data file C:\Temp\Mascoti\LSK_2D\091224LimSK_Exosome3_01_2D.mgf

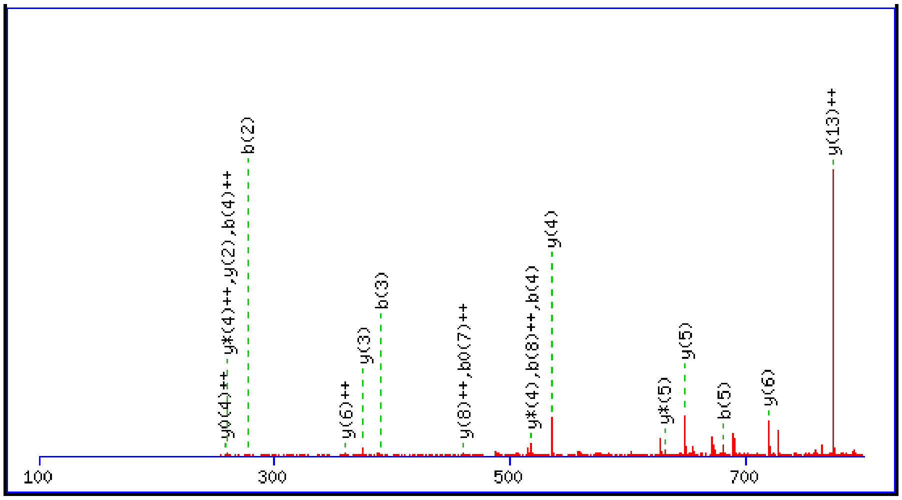

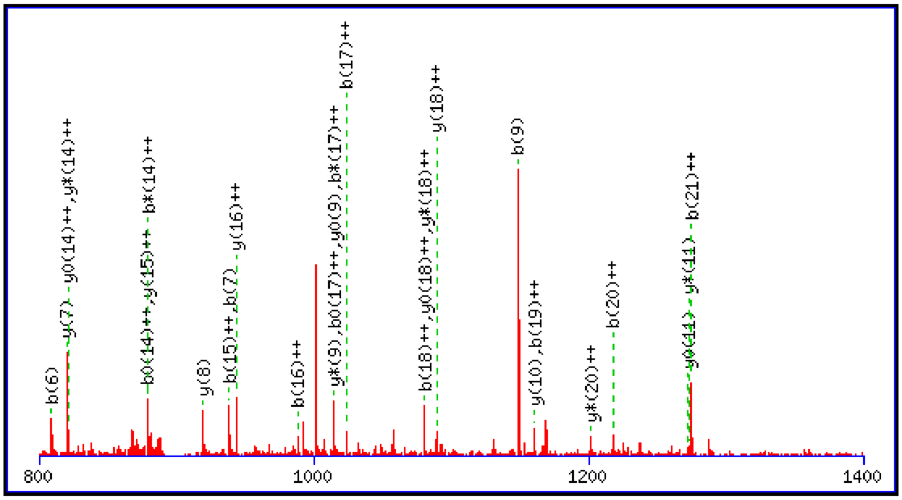

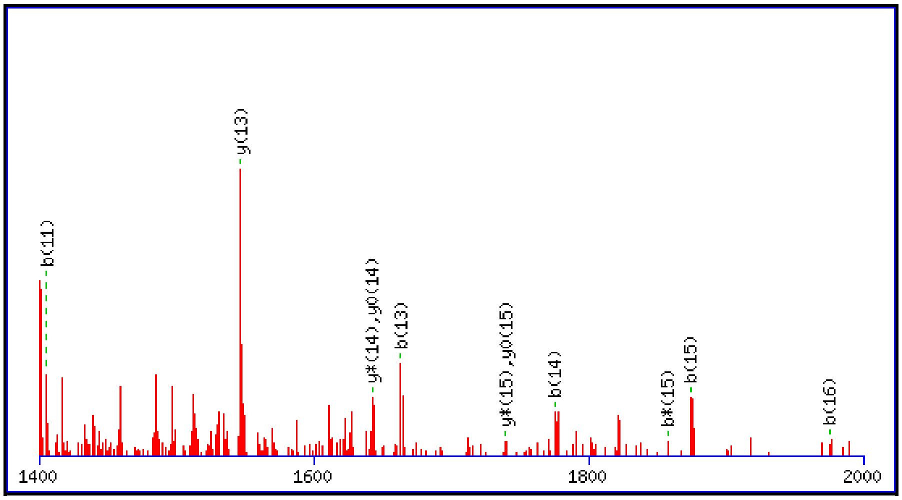

Monoisotopic mass of neutral peptide Mr(calc): 2693.3444
Fixed modifications: Carbamidomethyl (C)
Ions Score: 55 **Expect:** 0.00058
Matches (Bold Red): 59/230 fragment ions using 115 most intense peaks

#	b	b^{++}	b*	b^{*++}	b^0	b^{0++}	Seq.	y	y^{++}	y*	y^{*++}	y^0	y^{0++}	#
1	164.0706	82.5389					Y							22
2	**277.1547**	139.0810					L	2531.2884	1266.1478	2514.2619	1257.6346	2513.2778	1257.1426	21
3	**390.2387**	195.6230					L	2418.2043	1209.6058	2401.1778	**1201.0925**	2400.1938	1200.6005	20
4	**518.2973**	**259.6523**	501.2708	251.1390			Q	2305.1203	1153.0638	2288.0937	1144.5505	2287.1097	1144.0585	19
5	**681.3606**	341.1840	664.3341	332.6707			Y	2177.0617	**1089.0345**	2160.0352	**1080.5212**	2159.0511	**1080.0292**	18
6	**809.4192**	405.2132	792.3927	396.7000			Q	2013.9984	1007.5028	1996.9718	998.9896	1995.9878	998.4975	17

7	938.4618	469.7345	921.4353	461.2213	920.4512	460.7293	E	1885.9398	943.4735	1868.9133	934.9603	1867.9292	934.4683	16
8	1035.5146	518.2609	1018.4880	509.7477	1017.5040	509.2556	P	1756.8972	878.9522	1739.8707	870.4390	1738.8866	869.9470	15
9	1148.5986	574.8030	1131.5721	566.2897	1130.5881	565.7977	I	1659.8444	830.4259	1642.8179	821.9126	1641.8339	821.4206	14
10	1245.6514	623.3293	1228.6249	614.8161	1227.6408	614.3241	P	1546.7604	773.8838	1529.7338	765.3706	1528.7498	764.8785	13
11	1405.6821	703.3447	1388.6555	694.8314	1387.6715	694.3394	C	1449.7076	725.3574	1432.6811	716.8442	1431.6971	716.3522	12
12	1534.7246	767.8660	1517.6981	759.3527	1516.7141	758.8607	E	1289.6770	645.3421	1272.6504	636.8288	1271.6664	636.3368	11
13	1662.7832	831.8952	1645.7567	823.3820	1644.7727	822.8900	Q	1160.6344	580.8208	1143.6078	572.3076	1142.6238	571.8155	10
14	1775.8673	888.4373	1758.8407	879.9240	1757.8567	879.4320	L	1032.5758	516.7915	1015.5492	508.2783	1014.5652	507.7863	9
15	1874.9357	937.9715	1857.9092	929.4582	1856.9251	928.9662	V	919.4917	460.2495	902.4652	451.7362	901.4812	451.2442	8
16	1975.9834	988.4953	1958.9568	979.9821	1957.9728	979.4900	T	820.4233	410.7153	803.3968	402.2020	802.4128	401.7100	7
17	2047.0205	1024.0139	2029.9939	1015.5006	2029.0099	1015.0086	A	719.3756	360.1915	702.3491	351.6782	701.3651	351.1862	6
18	2160.1046	1080.5559	2143.0780	1072.0426	2142.0940	1071.5506	L	648.3385	324.6729	631.3120	316.1596	630.3280	315.6676	5
19	2320.1352	1160.5712	2303.1087	1152.0580	2302.1246	1151.5660	C	535.2545	268.1309	518.2279	259.6176	517.2439	259.1256	4
20	2435.1621	1218.0847	2418.1356	1209.5714	2417.1516	1209.0794	D	375.2238	188.1155	358.1973	179.6023	357.2132	179.1103	3
21	2548.2462	1274.6267	2531.2197	1266.1135	2530.2356	1265.6215	I	260.1969	130.6021	243.1703	122.0888			2
22							K	147.1128	74.0600	130.0863	65.5468			1

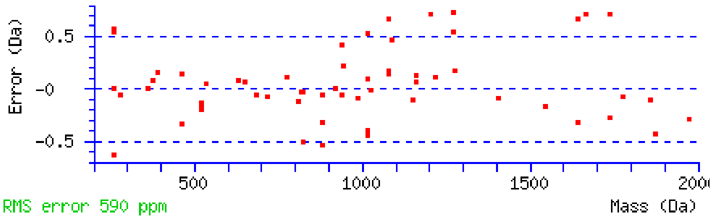

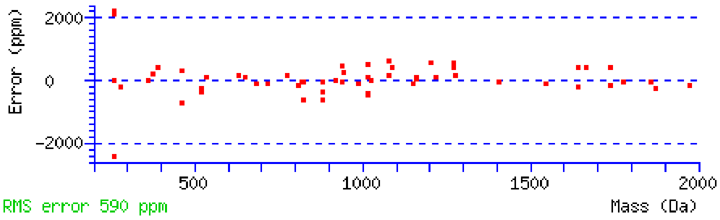

All matches to this query

Score	Mr(calc):	Delta	Sequence
55.0	2693.3444	0.0179	YLLQYQEPIPCEQLVTALCDIK
14.4	2692.3765	0.9858	TGVFLFTASSGGLPTDEITFAKLLK
10.8	2692.3765	0.9858	TGVFLFTASSGGLPTDEITFAKLLK
10.8	2692.3765	0.9858	TGVFLFTASSGGLPTDEITFAKLLK
9.7	2692.3765	0.9858	TGVFLFTASSGGLPTDEITFAKLLK
4.8	2691.3737	1.9886	TMAFHGLLNDGTVGIFRGNQMRLK
4.1	2693.3538	0.0085	NHYVVGAERKQLAGSLSLSETQVK
3.9	2692.3765	0.9858	TGVFLFTASSGGLPTDEITFAKLLK
3.5	2693.3370	0.0253	VPAEEPANELPMNEIEAWKAAEKK
3.0	2692.3853	0.9770	TLTLIEKGSQSVQKFVTLSNVLK

Spectrum No: 30; Query: 2493; Rank: 1

Peptide View

MS/MS Fragmentation of **AIYQATYR**
Found in **IPI00479306**, Tax_Id=9606 Gene_Symbol=PSMB5 Proteasome subunit beta type-5

Match to Query 2493: 984.507028 from(493.260790,2+)
Title: 091224LimSK_Exosome3_07.804.804.2.dta
Data file C:\Temp\Mascoti\LSK_2D\091224LimSK_Exosome3_01_2D.mgf

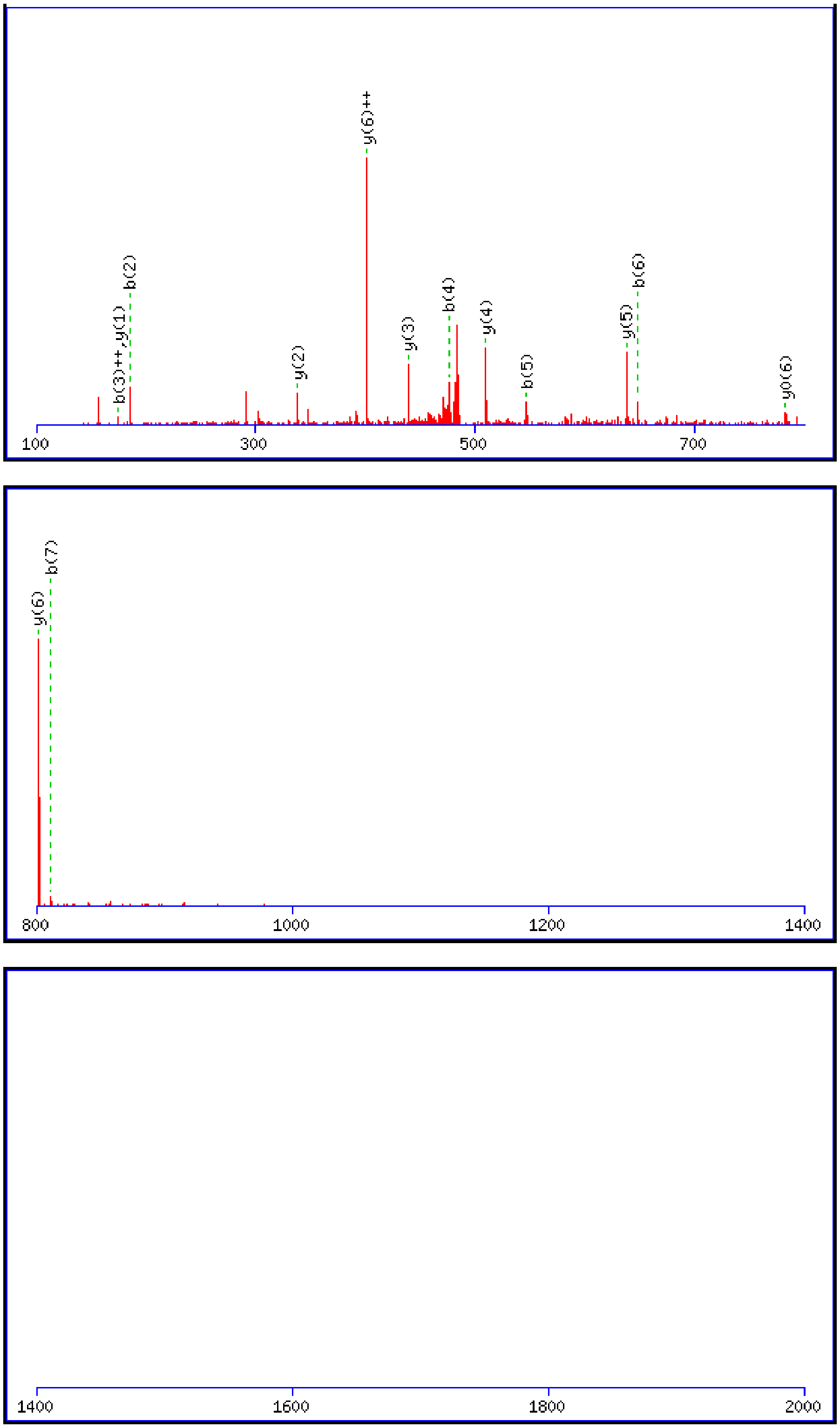

Monoisotopic mass of neutral peptide Mr(calc): 984.5029
Fixed modifications: Carbamidomethyl (C)
Ions Score: 36 **Expect:** 0.028
Matches (Bold Red): 14/64 fragment ions using 38 most intense peaks

#	b	b^{++}	b*	b^{*++}	b^0	b^{0++}	Seq.	y	y^{++}	y*	y^{*++}	y^0	y^{0++}	#
1	72.0444	36.5258					A							8
2	**185.1285**	93.0679					I	914.4730	457.7402	897.4465	449.2269	896.4625	448.7349	7
3	348.1918	**174.5995**					Y	**801.3890**	**401.1981**	784.3624	392.6849	**783.3784**	392.1928	6
4	**476.2504**	238.6288	459.2238	230.1155			Q	**638.3257**	319.6665	621.2991	311.1532	620.3151	310.6612	5
5	**547.2875**	274.1474	530.2609	265.6341			A	**510.2671**	255.6372	493.2405	247.1239	492.2565	246.6319	4
6	**648.3352**	324.6712	631.3086	316.1579	630.3246	315.6659	T	**439.2300**	220.1186	422.2034	211.6053	421.2194	211.1133	3

**7**	**811.3985**	406.2029	794.3719	397.6896	793.3879	397.1976	**Y**	**338.1823**	169.5948	321.1557	161.0815			**2**
**8**							**R**	**175.1190**	88.0631	158.0924	79.5498			**1**

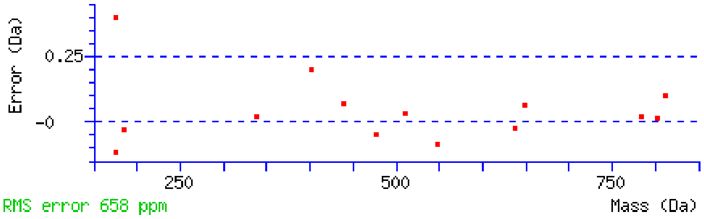

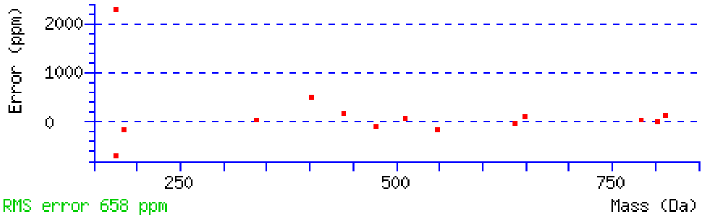

All matches to this query

Score	Mr(calc):	Delta	Sequence
35.8	984.5029	0.0042	AIYQATYR
6.2	984.5101	-0.0030	QRPQATER
5.9	982.5097	1.9974	YKHDHKR
5.2	982.5018	2.0052	KCGKAYTR
4.4	983.4971	1.0100	SNLMVHQR
4.3	984.5101	-0.0031	SPRQQVDR
4.1	984.5101	-0.0031	KSAGGTTAHR
4.0	982.5100	1.9970	TKLLVSDK
3.5	984.5141	-0.0071	QYTVRYR
3.2	984.5005	0.0065	TVKTVTTR

Spectrum No: 31; Query: 10547; Rank: 1

Peptide View

MS/MS Fragmentation of **ATAGAYIASQTVK**
Found in **IPI00383971**, Tax_Id=9606 Gene_Symbol=PSMB5 proteasome beta 5 subunit isoform 3

Match to Query 10547: 1279.682928 from(640.848740,2+)
Title: 091224LimSK_Exosome3_07.1335.1335.2.dta
Data file C:\Temp\Mascoti\LSK_2D\091224LimSK_Exosome3_01_2D.mgf

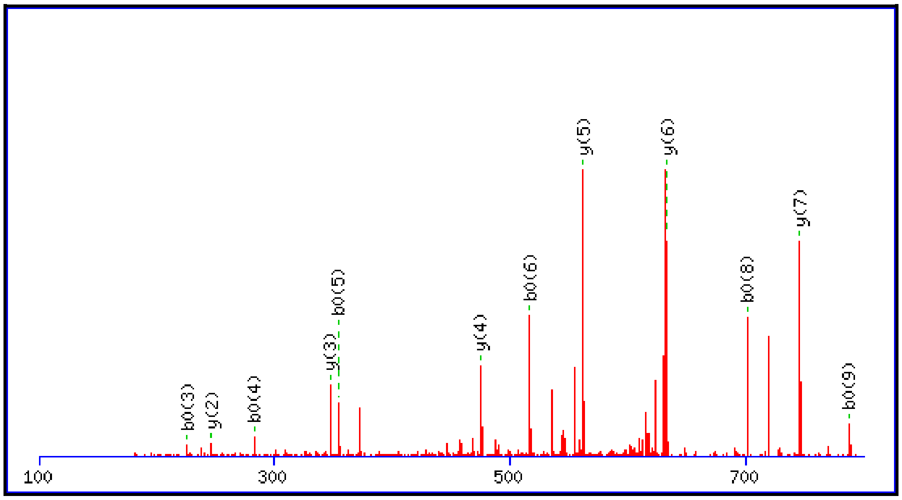

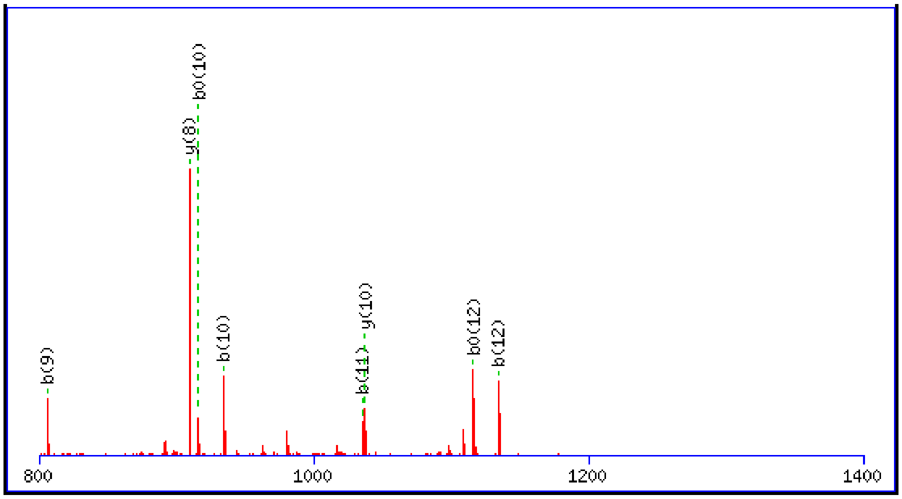

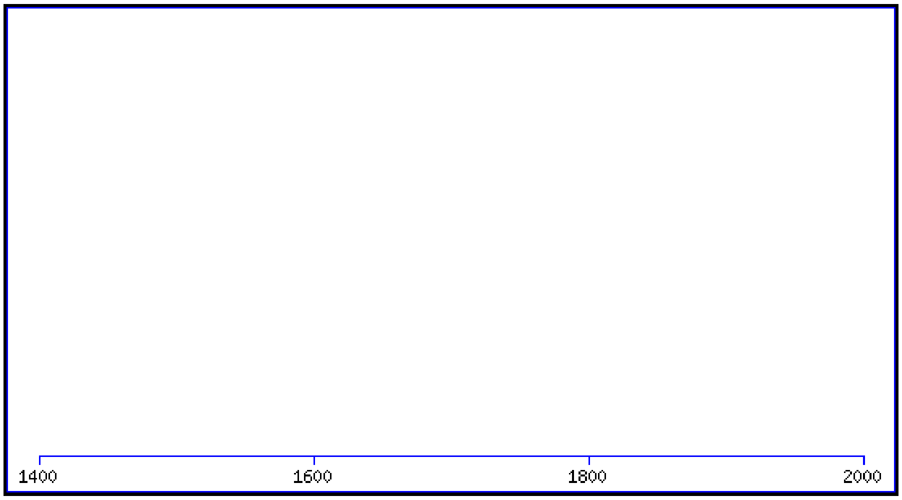

Monoisotopic mass of neutral peptide Mr(calc): 1279.6772
Fixed modifications: Carbamidomethyl (C)
Ions Score: 75 **Expect:** 4e-006
Matches (Bold Red): 20/120 fragment ions using 24 most intense peaks

#	b	b^{++}	b*	b^{*++}	b^0	b^{0++}	Seq.	y	y^{++}	y*	y^{*++}	y^0	y^{0++}	#
1	72.0444	36.5258					A							13
2	173.0921	87.0497			155.0815	78.0444	T	1209.6474	605.3273	1192.6208	596.8141	1191.6368	596.3220	12
3	244.1292	122.5682			**226.1186**	113.5629	A	1108.5997	554.8035	1091.5732	546.2902	1090.5891	545.7982	11
4	301.1506	151.0790			**283.1401**	142.0737	G	**1037.5626**	519.2849	1020.5360	510.7717	1019.5520	510.2796	10
5	372.1878	186.5975			**354.1772**	177.5922	A	980.5411	490.7742	963.5146	482.2609	962.5306	481.7689	9
6	535.2511	268.1292			**517.2405**	259.1239	Y	**909.5040**	455.2556	892.4775	446.7424	891.4934	446.2504	8
7	648.3352	324.6712			630.3246	315.6659	I	**746.4407**	373.7240	729.4141	365.2107	728.4301	364.7187	7
8	719.3723	360.1898			**701.3617**	351.1845	A	**633.3566**	317.1819	616.3301	308.6687	615.3461	308.1767	6
9	**806.4043**	403.7058			**788.3937**	394.7005	S	**562.3195**	281.6634	545.2930	273.1501	544.3089	272.6581	5
10	**934.4629**	467.7351	917.4363	459.2218	**916.4523**	458.7298	Q	**475.2875**	238.1474	458.2609	229.6341	457.2769	229.1421	4
11	**1035.5106**	518.2589	1018.4840	509.7456	1017.5000	509.2536	T	**347.2289**	174.1181	330.2023	165.6048	329.2183	165.1128	3
12	**1134.5790**	567.7931	1117.5524	559.2798	**1116.5684**	558.7878	V	**246.1812**	123.5942	229.1547	115.0810			2
13							K	147.1128	74.0600	130.0863	65.5468			1

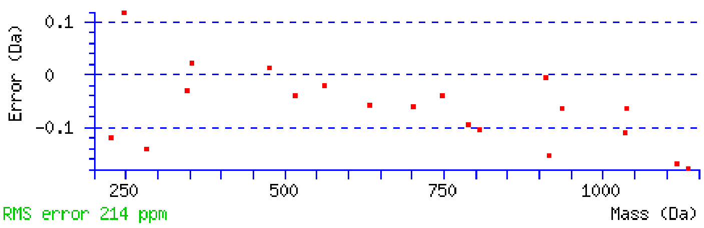

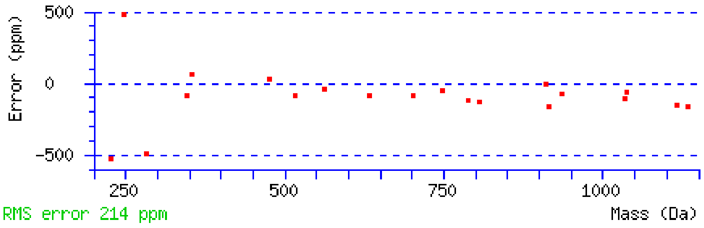

All matches to this query

Score	Mr(calc):	Delta	Sequence
75.3	1279.6772	0.0057	ATAGAYIASQTVK
19.9	1279.6901	-0.0072	KPTQASITKVK
14.3	1279.6901	-0.0072	KPTQASITKVK
8.8	1279.6772	0.0057	NVFTVSASSIQK
7.6	1277.6723	2.0106	VMMAELEKTVK
6.7	1277.6857	1.9973	ARLQSPATKVK
6.6	1277.6857	1.9973	ARLQSPATKVK
6.1	1279.6880	-0.0050	LIKSMESVMVK
3.8	1279.6880	-0.0050	LIKSMESVMVK
1.6	1279.6788	0.0041	ELSLLKGDVVK

Spectrum No: 32; Query: 21296; Rank: 1

Peptide View

MS/MS Fragmentation of **DAYSGGAVNLYHVR**
Found in **IPI00479306**, Tax_Id=9606 Gene_Symbol=PSMB5 Proteasome subunit beta type-5

Match to Query 21296: 1520.747688 from(761.381120,2+)
Title: 091224LimSK_Exosome3_07.2739.2739.2.dta
Data file C:\Temp\Mascoti\LSK_2D\091224LimSK_Exosome3_01_2D.mgf

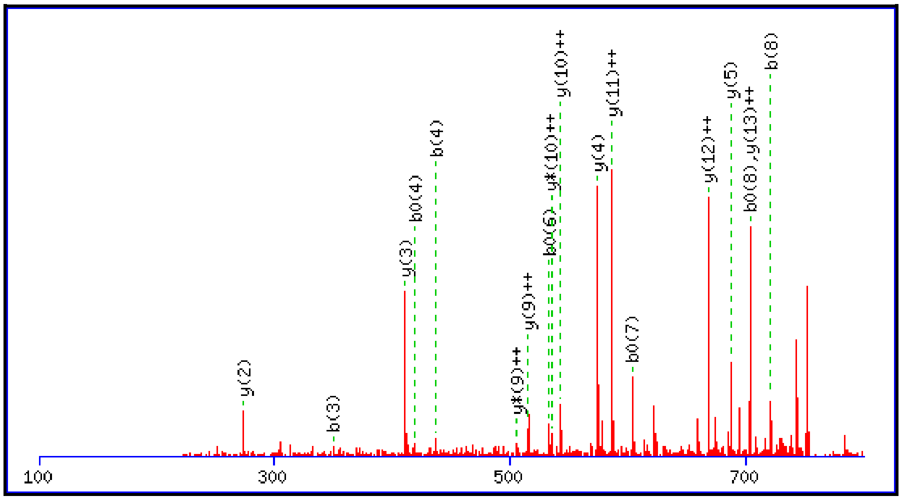

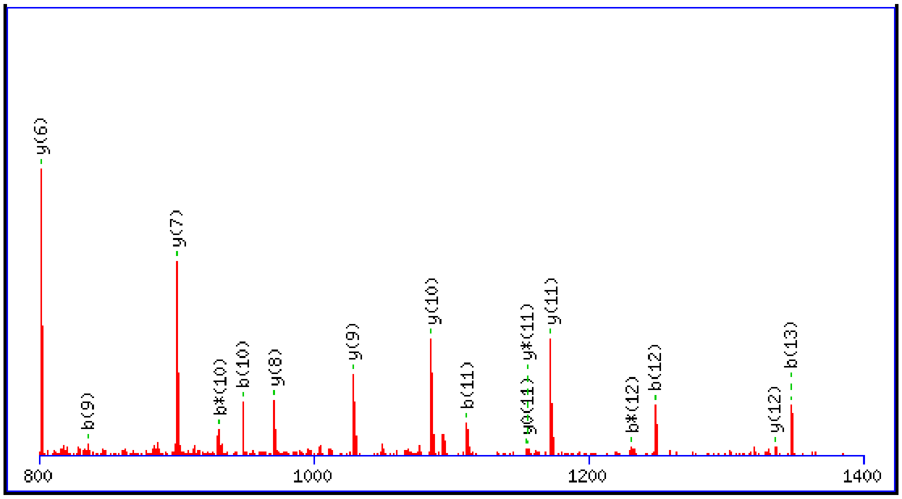

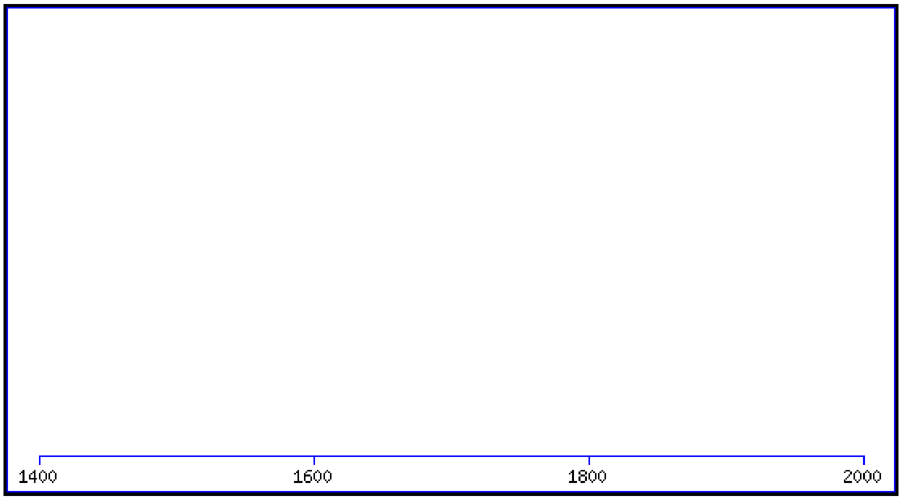

Monoisotopic mass of neutral peptide Mr(calc): 1520.7372
Fixed modifications: Carbamidomethyl (C)
Ions Score: 73 **Expect:** 9.6e-006
Matches (Bold Red): 34/120 fragment ions using 67 most intense peaks

#	b	b^{++}	b*	b^{*++}	b^{0}	b^{0++}	Seq.	y	y^{++}	y*	y^{*++}	y^{0}	y^{0++}	#
1	116.0342	58.5207			98.0237	49.5155	D							14
2	187.0713	94.0393			169.0608	85.0340	A	1406.7175	**703.8624**	1389.6910	695.3491	1388.7070	694.8571	13
3	**350.1347**	175.5710			332.1241	166.5657	Y	**1335.6804**	**668.3438**	1318.6539	659.8306	1317.6698	659.3386	12
4	**437.1667**	219.0870			**419.1561**	210.0817	S	**1172.6171**	**586.8122**	**1155.5905**	578.2989	**1154.6065**	577.8069	11
5	494.1882	247.5977			476.1776	238.5924	G	**1085.5851**	**543.2962**	1068.5585	**534.7829**			10
6	551.2096	276.1084			**533.1991**	267.1032	G	**1028.5636**	**514.7854**	1011.5370	**506.2722**			9
7	622.2467	311.6270			**604.2362**	302.6217	A	**971.5421**	486.2747	954.5156	477.7614			8
8	**721.3151**	361.1612			**703.3046**	352.1559	V	**900.5050**	450.7561	883.4785	442.2429			7
9	**835.3581**	418.1827	818.3315	409.6694	817.3475	409.1774	N	**801.4366**	401.2219	784.4100	392.7087			6
10	**948.4421**	474.7247	**931.4156**	466.2114	930.4316	465.7194	L	**687.3937**	344.2005	670.3671	335.6872			5
11	**1111.5055**	556.2564	1094.4789	547.7431	1093.4949	547.2511	Y	**574.3096**	287.6584	557.2831	279.1452			4
12	**1248.5644**	624.7858	**1231.5378**	616.2726	1230.5538	615.7805	H	**411.2463**	206.1268	394.2197	197.6135			3
13	**1347.6328**	674.3200	1330.6062	665.8068	1329.6222	665.3148	V	**274.1874**	137.5973	257.1608	129.0840			2
14							R	175.1190	88.0631	158.0924	79.5498			1

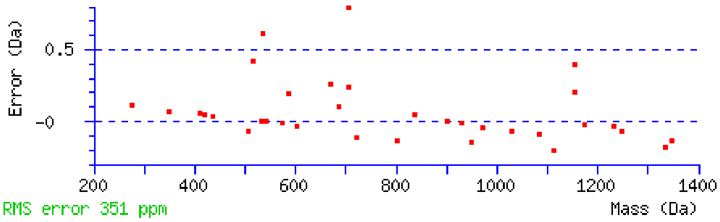

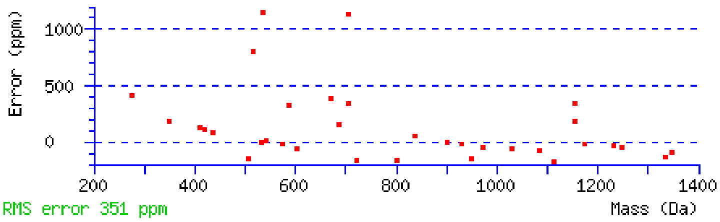

All matches to this query

Score	Mr(calc):	Delta	Sequence
73.2	1520.7372	0.0105	DAYSGGAVNLYHVR
6.8	1520.7501	-0.0024	IHSPSPHKQVPSK
2.9	1520.7471	0.0006	VNEKYSTLPAEDR
2.5	1519.7396	1.0081	GSGKPPGVPASSKSGK
2.4	1519.7552	0.9925	IAEENIMKSNIDK
1.9	1519.7396	1.0081	GSGKPPGVPASSKSGK
1.9	1520.7479	-0.0002	LAKNMEEMFHKK
0.5	1519.7297	1.0180	AFSAHSSLVTHKR
0.2	1520.7599	-0.0123	TPPKSSQELLSVR
0.2	1520.7599	-0.0123	TPPKSSQELLSVR

Spectrum No: 33; Query: 16441; Rank: 1

Peptide View

MS/MS Fragmentation of **GPGLYYVDSEGNR**
Found in **IPI00479306**, Tax_Id=9606 Gene_Symbol=PSMB5 Proteasome subunit beta type-5

Match to Query 16441: 1425.659468 from(713.837010,2+)
Title: 091224LimSK_Exosome3_07.2142.2142.2.dta
Data file C:\Temp\Mascoti\LSK_2D\091224LimSK_Exosome3_01_2D.mgf

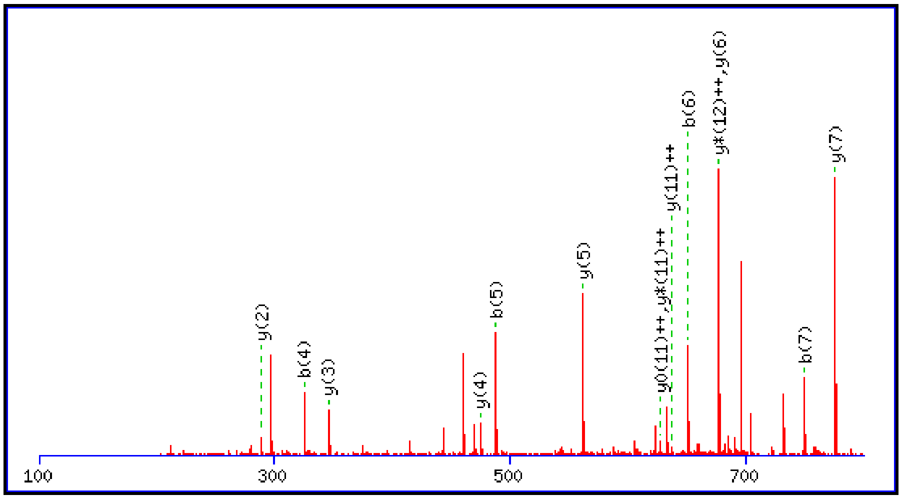

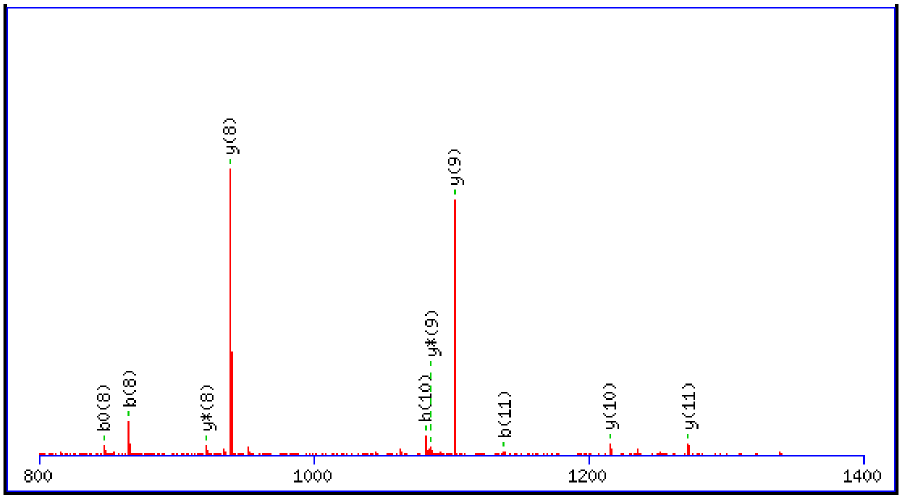

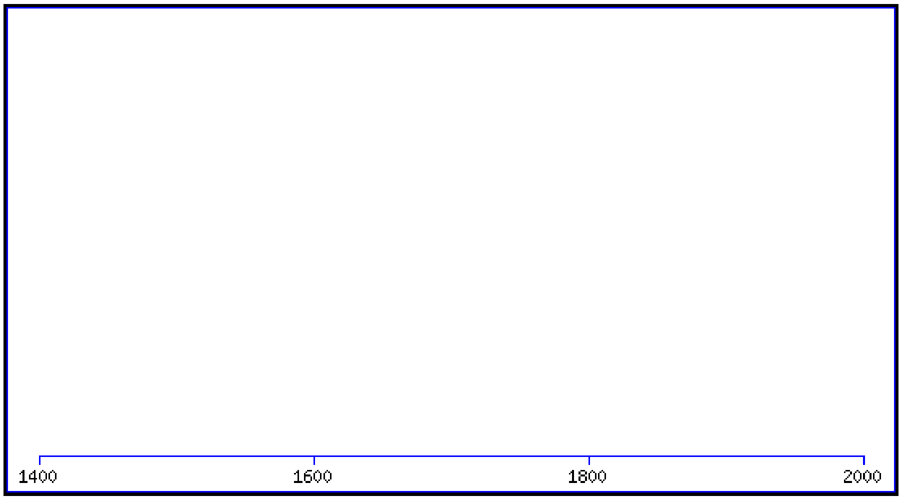

Monoisotopic mass of neutral peptide Mr(calc): 1425.6525
Fixed modifications: Carbamidomethyl (C)
Ions Score: 72 **Expect:** 1.3e-005
Matches (Bold Red): 24/102 fragment ions using 46 most intense peaks

#	b	b^{++}	b*	$b*^{++}$	b^0	b^{0++}	Seq.	y	y^{++}	y*	$y*^{++}$	y^0	y^{0++}	#
1	58.0287	29.5180					G							13
2	155.0815	78.0444					P	1369.6383	685.3228	1352.6117	**676.8095**	1351.6277	676.3175	12
3	212.1030	106.5551					G	**1272.5855**	**636.7964**	1255.5590	**628.2831**	1254.5749	**627.7911**	11
4	**325.1870**	163.0972					L	**1215.5640**	608.2857	1198.5375	599.7724	1197.5535	599.2804	10
5	**488.2504**	244.6288					Y	**1102.4800**	551.7436	**1085.4534**	543.2304	1084.4694	542.7383	9
6	**651.3137**	326.1605					Y	**939.4166**	470.2120	**922.3901**	461.6987	921.4061	461.2067	8
7	**750.3821**	375.6947					V	**776.3533**	388.6803	759.3268	380.1670	758.3428	379.6750	7
8	**865.4090**	433.2082			**847.3985**	424.2029	D	**677.2849**	339.1461	660.2584	330.6328	659.2743	330.1408	6
9	952.4411	476.7242			934.4305	467.7189	S	**562.2580**	281.6326	545.2314	273.1193	544.2474	272.6273	5
10	**1081.4837**	541.2455			1063.4731	532.2402	E	**475.2259**	238.1166	458.1994	229.6033	457.2154	229.1113	4
11	**1138.5051**	569.7562			1120.4946	560.7509	G	**346.1833**	173.5953	329.1568	165.0820			3
12	1252.5481	626.7777	1235.5215	618.2644	1234.5375	617.7724	N	**289.1619**	145.0846	272.1353	136.5713			2
13							R	175.1190	88.0631	158.0924	79.5498			1

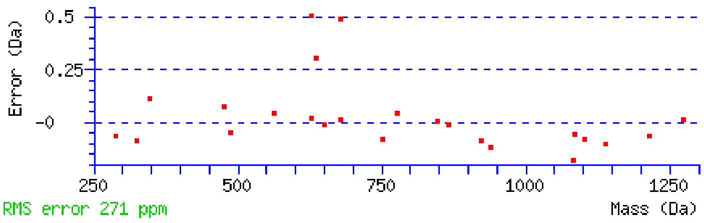

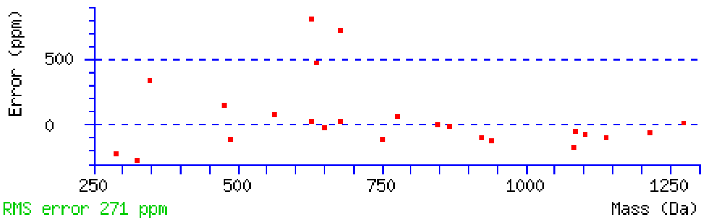

All matches to this query

Score	Mr(calc):	Delta	Sequence
72.0	1425.6525	0.0070	GPGLYYVDSEGNR
8.1	1423.6578	2.0017	MKHVTVGRNMR
4.4	1425.6736	-0.0141	XTDPVDVVPQDGR
2.4	1425.6493	0.0102	CQGPAPSPSPCLR
0.7	1423.6531	2.0064	SVTHANALTVMGK
0.7	1423.6531	2.0064	SVTHANALTVMGK
0.5	1424.6602	0.9993	AAARDYTFFKR
0.4	1425.6687	-0.0092	LLTSQELQMQR
0.4	1425.6687	-0.0092	LLTSQELQMQR
0.2	1425.6574	0.0020	LLSVMEGELEAR

Spectrum No: 34; Query: 36207; Rank: 1

Peptide View

MS/MS Fragmentation of **GYSYDLEVEQAYDLAR**
Found in **IPI00479306**, Tax_Id=9606 Gene_Symbol=PSMB5 Proteasome subunit beta type-5

Match to Query 36207: 1890.875988 from(946.445270,2+)
Title: 091224LimSK_Exosome3_07.6197.6197.2.dta
Data file C:\Temp\Mascoti\LSK_2D\091224LimSK_Exosome3_01_2D.mgf

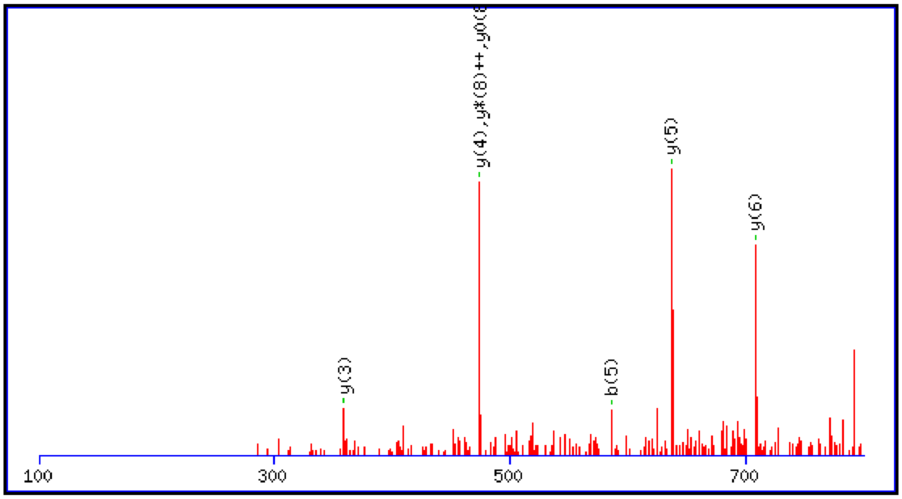

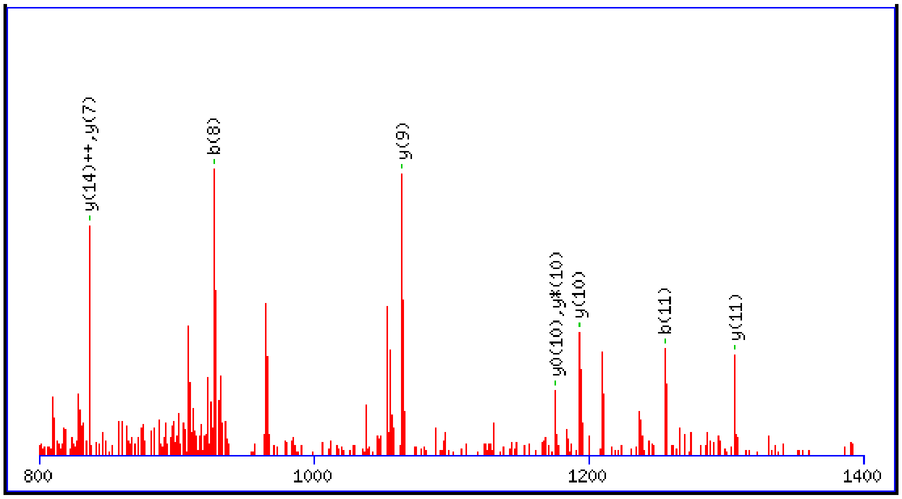

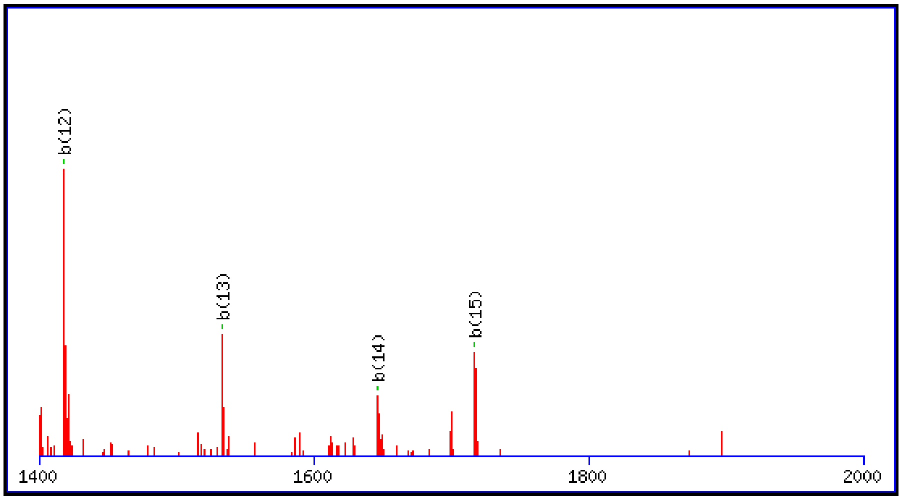

Monoisotopic mass of neutral peptide Mr(calc): 1890.8635
Fixed modifications: Carbamidomethyl (C)
Ions Score: 83 **Expect:** 1.2e-006
Matches (Bold Red): 20/152 fragment ions using 24 most intense peaks

#	b	b++	b*	b*++	b0	b0++	Seq.	y	y++	y*	y*++	y0	y0++	#
1	58.0287	29.5180					G							16
2	221.0921	111.0497					Y	1834.8494	917.9283	1817.8228	909.4151	1816.8388	908.9230	15
3	308.1241	154.5657			290.1135	145.5604	S	1671.7861	**836.3967**	1654.7595	827.8834	1653.7755	827.3914	14
4	471.1874	236.0974			453.1769	227.0921	Y	1584.7540	792.8807	1567.7275	784.3674	1566.7435	783.8754	13
5	**586.2144**	293.6108			568.2038	284.6055	D	1421.6907	711.3490	1404.6642	702.8357	1403.6801	702.3437	12
6	699.2984	350.1529			681.2879	341.1476	L	**1306.6638**	653.8355	1289.6372	645.3222	1288.6532	644.8302	11
7	828.3410	414.6742			810.3305	405.6689	E	**1193.5797**	597.2935	**1176.5531**	588.7802	**1175.5691**	588.2882	10
8	**927.4094**	464.2084			909.3989	455.2031	V	**1064.5371**	532.7722	1047.5106	524.2589	1046.5265	523.7669	9
9	1056.4520	528.7297			1038.4415	519.7244	E	965.4687	483.2380	948.4421	**474.7247**	947.4581	**474.2327**	8
10	1184.5106	592.7589	1167.4841	584.2457	1166.5000	583.7537	Q	**836.4261**	418.7167	819.3995	410.2034	818.4155	409.7114	7
11	**1255.5477**	628.2775	1238.5212	619.7642	1237.5372	619.2722	A	**708.3675**	354.6874	691.3410	346.1741	690.3570	345.6821	6
12	**1418.6111**	709.8092	1401.5845	701.2959	1400.6005	700.8039	Y	**637.3304**	319.1688	620.3039	310.6556	619.3198	310.1636	5
13	**1533.6380**	767.3226	1516.6114	758.8094	1515.6274	758.3174	D	**474.2671**	237.6372	457.2405	229.1239	456.2565	228.6319	4
14	**1646.7221**	823.8647	1629.6955	815.3514	1628.7115	814.8594	L	**359.2401**	180.1237	342.2136	171.6104			3
15	**1717.7592**	859.3832	1700.7326	850.8700	1699.7486	850.3779	A	246.1561	123.5817	229.1295	115.0684			2
16							R	175.1190	88.0631	158.0924	79.5498			1

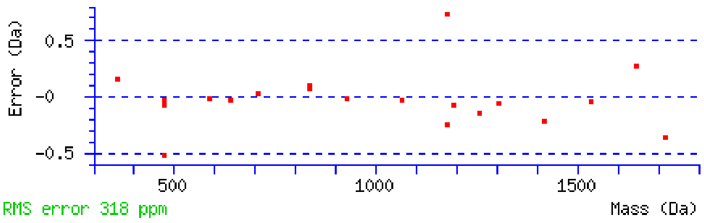

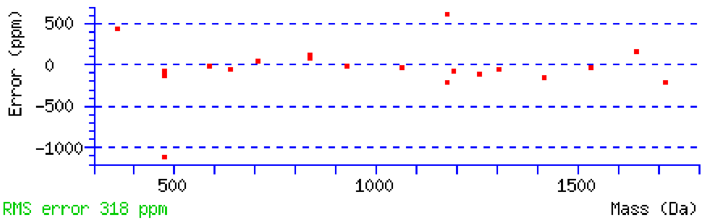

All matches to this query

Score	Mr(calc):	Delta	Sequence
82.9	1890.8635	0.0124	GYSYDLEVEQAYDLAR
16.8	1888.8833	1.9927	PQTFSLGTLHEPAGAQR
16.8	1888.8833	1.9927	PQTFSLGTLHEPAGAQR
15.2	1890.8893	-0.0133	LLIYDASKLEAGVPSR
12.6	1890.8688	0.0072	MRPYRESPLLARAR
10.2	1889.8745	1.0015	LSPAGASPGPQSASSRGQR
9.5	1889.8668	1.0092	MFETMAIEIEQLLAR
8.6	1888.8833	1.9927	PQTFSLGTLHEPAGAQR
7.8	1890.8949	-0.0189	QLIRETSTHQLNSER
6.8	1890.8599	0.0161	THTHTGIHTQTHRER

Spectrum No: 35; Query: 3147; Rank: 1

Peptide View

MS/MS Fragmentation of **HGVIVAADSR**
Found in **IPI00383971**, Tax_Id=9606 Gene_Symbol=PSMB5 proteasome beta 5 subunit isoform 3

Match to Query 3147: 1023.549968 from(512.782260,2+)
Title: 091224LimSK_Exosome3_07.231.231.2.dta
Data file C:\Temp\Mascoti\LSK_2D\091224LimSK_Exosome3_01_2D.mgf

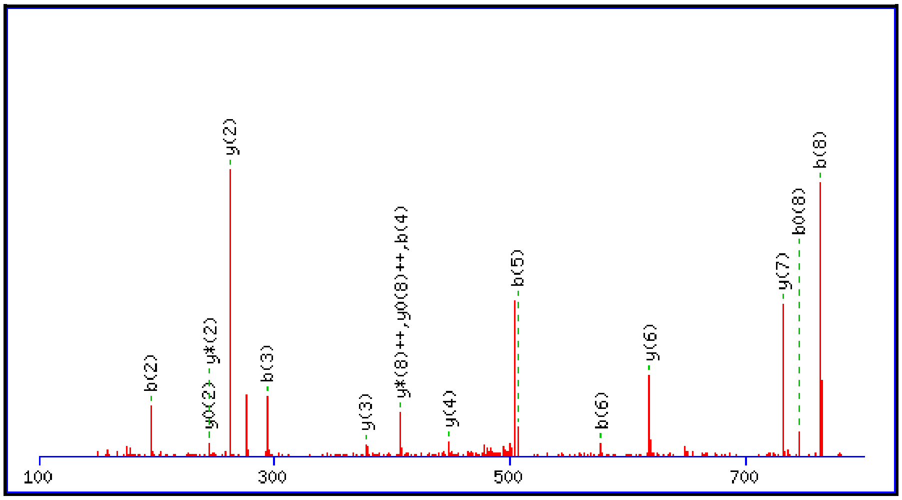

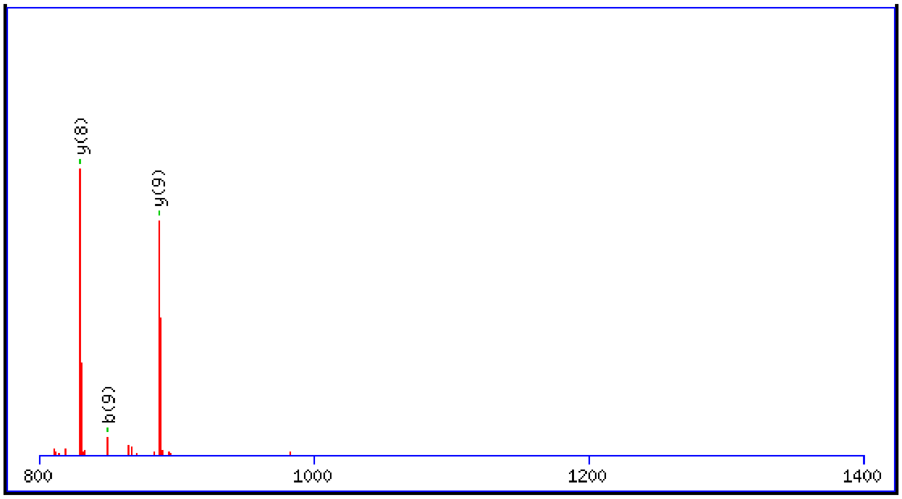

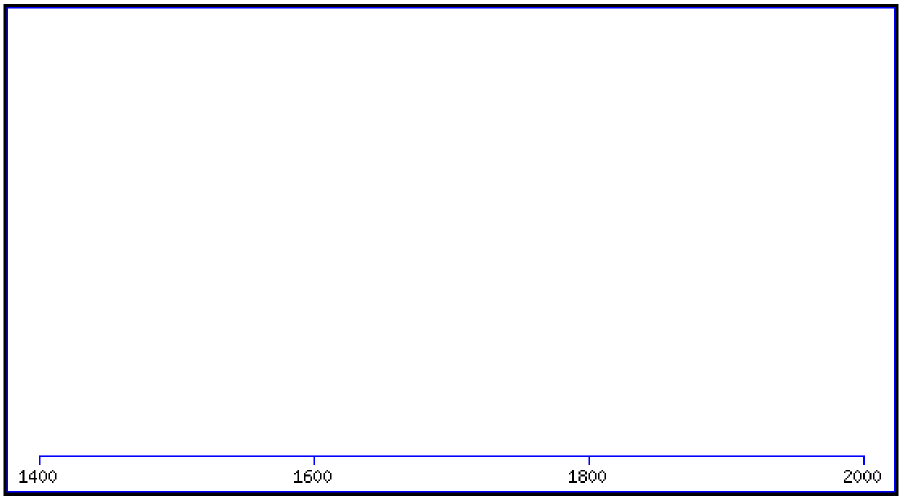

Monoisotopic mass of neutral peptide Mr(calc): 1023.5461
Fixed modifications: Carbamidomethyl (C)
Ions Score: 67 **Expect:** 2.1e-005
Matches (Bold Red): 19/74 fragment ions using 27 most intense peaks

#	b	b^{++}	b^{0}	b^{0++}	Seq.	y	y^{++}	y*	y^{*++}	y^{0}	y^{0++}	#
1	138.0662	69.5367			H							10
2	**195.0877**	98.0475			G	**887.4945**	444.2509	870.4680	435.7376	869.4839	435.2456	9
3	**294.1561**	147.5817			V	**830.4730**	415.7402	813.4465	**407.2269**	812.4625	**406.7349**	8
4	**407.2401**	204.1237			I	**731.4046**	366.2060	714.3781	357.6927	713.3941	357.2007	7
5	**506.3085**	253.6579			V	**618.3206**	309.6639	601.2940	301.1506	600.3100	300.6586	6
6	**577.3457**	289.1765			A	519.2522	260.1297	502.2256	251.6164	501.2416	251.1244	5
7	648.3828	324.6950			A	**448.2150**	224.6112	431.1885	216.0979	430.2045	215.6059	4
8	**763.4097**	382.2085	**745.3991**	373.2032	D	**377.1779**	189.0926	360.1514	180.5793	359.1674	180.0873	3
9	**850.4417**	425.7245	832.4312	416.7192	S	**262.1510**	131.5791	**245.1244**	123.0659	**244.1404**	122.5738	2
10					R	175.1190	88.0631	158.0924	79.5498			1

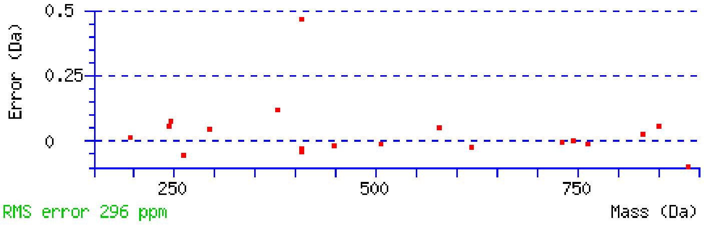

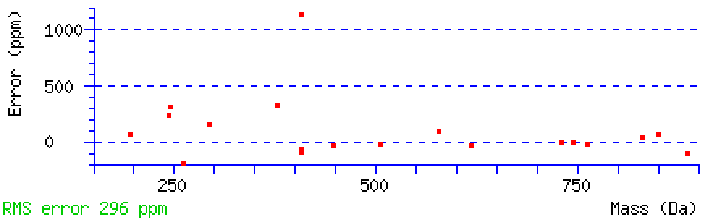

All matches to this query

Score	Mr(calc):	Delta	Sequence
67.0	1023.5461	0.0038	HGVIVAADSR
10.4	1023.5461	0.0038	YSRGVLSSR
8.1	1023.5569	-0.0069	MKVKMLSR
7.6	1023.5423	0.0077	KDDKMLFK
6.1	1021.5444	2.0056	YQLTLEQK
6.0	1021.5444	2.0056	FKTELEQK
5.5	1021.5444	2.0056	LYKQLDDK
4.3	1022.5430	1.0070	KNSLSSIMK
3.7	1021.5457	2.0042	AGIRYWTR
3.6	1021.5444	2.0055	DKTVGDFLK

Spectrum No: 36; Query: 44178; Rank: 1

Peptide View

MS/MS Fragmentation of **ISGATFSVGSGSVYAYGVMDR**
Found in **IPI00479306**, Tax_Id=9606 Gene_Symbol=PSMB5 Proteasome subunit beta type-5

Match to Query 44178: 2123.007708 from(1062.511130,2+)
Title: 091224LimSK_Exosome3_07.6355.6355.2.dta
Data file C:\Temp\Mascoti\LSK_2D\091224LimSK_Exosome3_01_2D.mgf

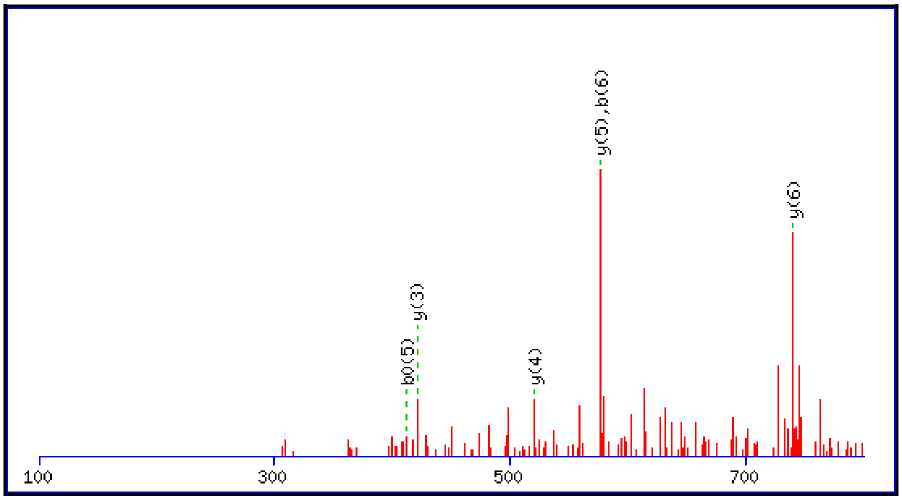

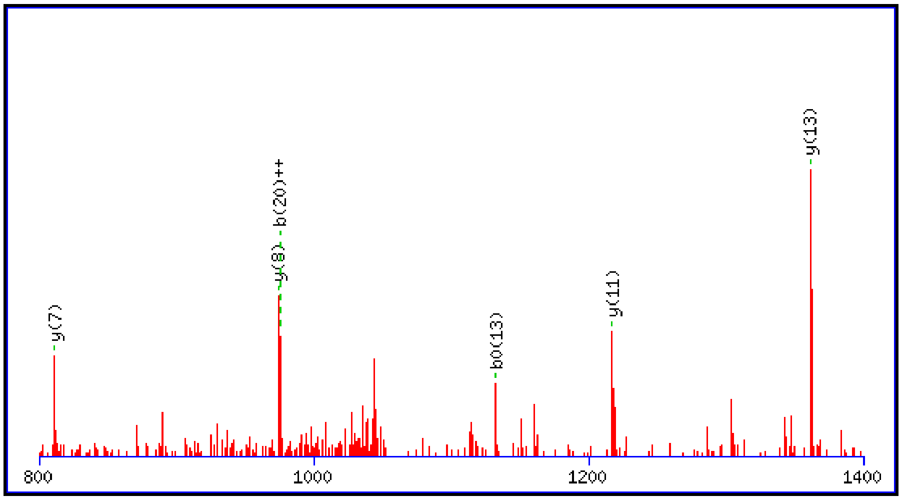

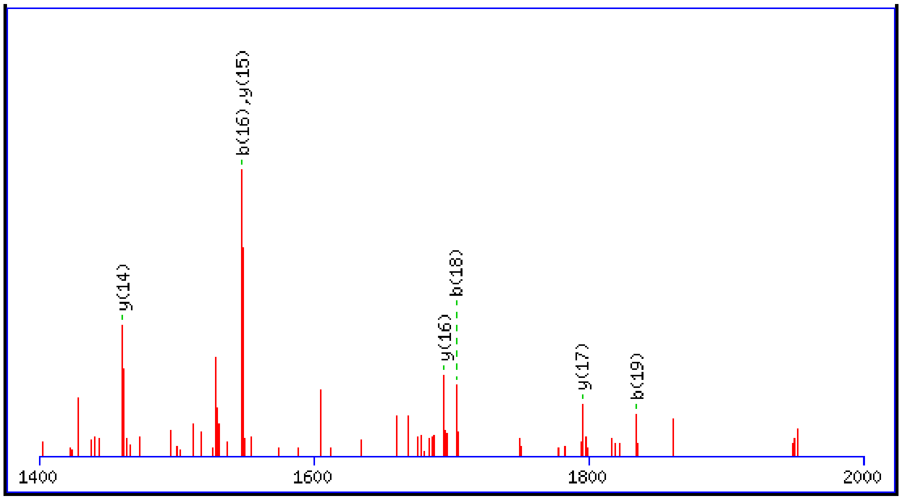

Monoisotopic mass of neutral peptide Mr(calc): 2122.9994
Fixed modifications: Carbamidomethyl (C)
Ions Score: 93 **Expect:** 1.4e-007
Matches (Bold Red): 19/196 fragment ions using 25 most intense peaks

#	b	b^{++}	b^{0}	b^{0++}	Seq.	y	y^{++}	y^{*}	y^{*++}	y^{0}	y^{0++}	#
1	114.0913	57.5493			I							21
2	201.1234	101.0653	183.1128	92.0600	S	2010.9226	1005.9649	1993.8960	997.4516	1992.9120	996.9596	20
3	258.1448	129.5761	240.1343	120.5708	G	1923.8905	962.4489	1906.8640	953.9356	1905.8800	953.4436	19
4	329.1819	165.0946	311.1714	156.0893	A	1866.8691	933.9382	1849.8425	925.4249	1848.8585	924.9329	18
5	430.2296	215.6185	**412.2191**	206.6132	T	**1795.8320**	898.4196	1778.8054	889.9063	1777.8214	889.4143	17
6	**577.2980**	289.1527	559.2875	280.1474	F	**1694.7843**	847.8958	1677.7577	839.3825	1676.7737	838.8905	16
7	664.3301	332.6687	646.3195	323.6634	S	**1547.7159**	774.3616	1530.6893	765.8483	1529.7053	765.3563	15
8	763.3985	382.2029	745.3879	373.1976	V	**1460.6838**	730.8456	1443.6573	722.3323	1442.6733	721.8403	14
9	820.4199	410.7136	802.4094	401.7083	G	**1361.6154**	681.3114	1344.5889	672.7981	1343.6049	672.3061	13
10	907.4520	454.2296	889.4414	445.2243	S	1304.5940	652.8006	1287.5674	644.2873	1286.5834	643.7953	12
11	964.4734	482.7404	946.4629	473.7351	G	**1217.5619**	609.2846	1200.5354	600.7713	1199.5514	600.2793	11
12	1051.5055	526.2564	1033.4949	517.2511	S	1160.5405	580.7739	1143.5139	572.2606	1142.5299	571.7686	10
13	1150.5739	575.7906	**1132.5633**	566.7853	V	1073.5084	537.2579	1056.4819	528.7446	1055.4979	528.2526	9
14	1313.6372	657.3222	1295.6266	648.3170	Y	**974.4400**	487.7237	957.4135	479.2104	956.4295	478.7184	8
15	1384.6743	692.8408	1366.6638	683.8355	A	**811.3767**	406.1920	794.3502	397.6787	793.3661	397.1867	7
16	**1547.7377**	774.3725	1529.7271	765.3672	Y	**740.3396**	370.6734	723.3130	362.1602	722.3290	361.6681	6
17	1604.7591	802.8832	1586.7486	793.8779	G	**577.2763**	289.1418	560.2497	280.6285	559.2657	280.1365	5
18	**1703.8275**	852.4174	1685.8170	843.4121	V	**520.2548**	260.6310	503.2282	252.1178	502.2442	251.6258	4
19	**1834.8680**	917.9376	1816.8574	908.9324	M	**421.1864**	211.0968	404.1598	202.5836	403.1758	202.0915	3
20	1949.8950	**975.4511**	1931.8844	966.4458	D	290.1459	145.5766	273.1193	137.0633	272.1353	136.5713	2
21					R	175.1190	88.0631	158.0924	79.5498			1

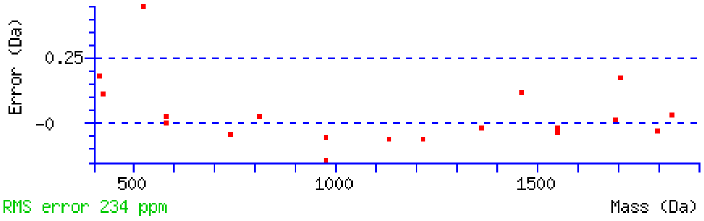

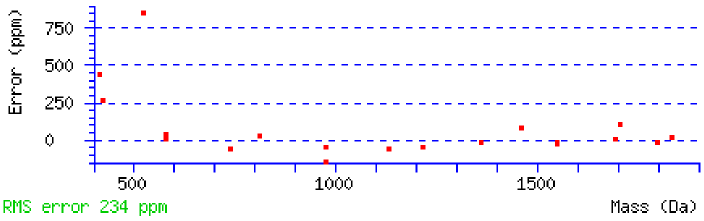

All matches to this query

Score	Mr(calc):	Delta	Sequence
92.7	2122.9994	0.0083	ISGATFSVGSGSVYAYGVMDR

8.3	2122.9942	0.0135	AKPSLLSRVGALTNTLSR
6.7	2120.9923	2.0154	MLSFWWPLALILATQR
6.3	2123.0049	0.0029	XTSAPPTLGQPKGVSASQDR
5.5	2123.0010	0.0067	IYAMNSEGLGEPALVPGTPK
5.4	2123.0252	-0.0175	CFIVGADNVNSKQMQQIR
5.3	2123.0049	0.0029	XTSAPPTLGQPKGVSASQDR
4.7	2123.0049	0.0029	XTSAPPTLGQPKGVSASQDR
4.1	2120.9921	2.0156	RFNYVVRALPLASEGNR
4.1	2123.0038	0.0039	QTPHLGEQRRTTASLLR

Spectrum No: 37; Query: 61596; Rank: 1

Peptide View

MS/MS Fragmentation of **KVIEINPYLLGTMAGGAADCSFWER**
Found in **IPI00383971**, Tax_Id=9606 Gene_Symbol=PSMB5 proteasome beta 5 subunit isoform 3

Match to Query 61596: 2798.372742 from(933.798190,3+)
Title: 091224LimSK_Exosome3_08.7700.7700.3.dta
Data file C:\Temp\Mascoti\LSK_2D\091224LimSK_Exosome3_01_2D.mgf

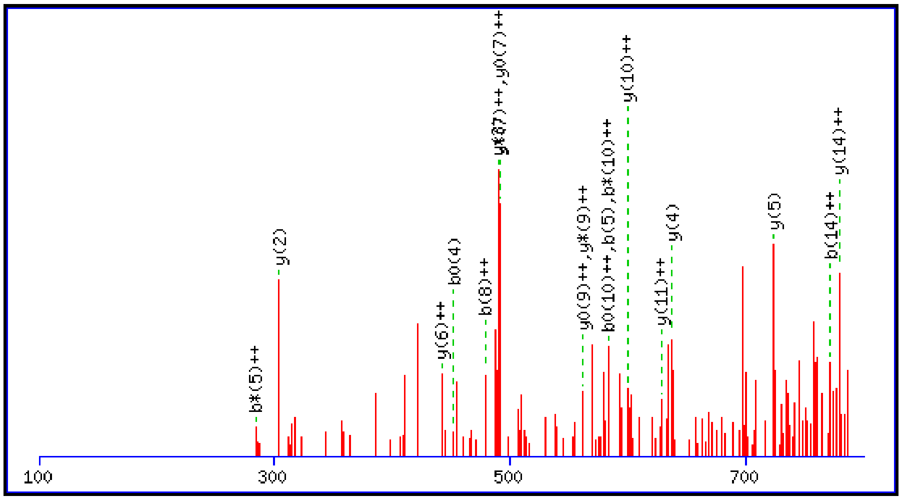

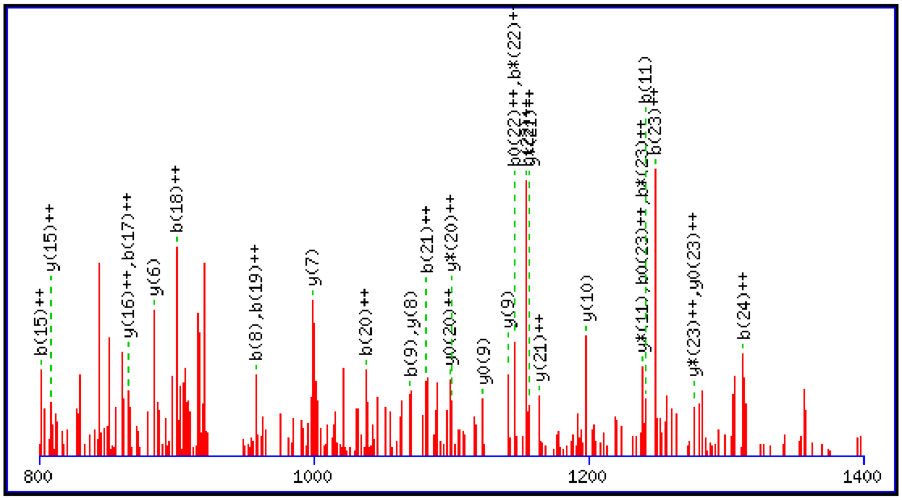

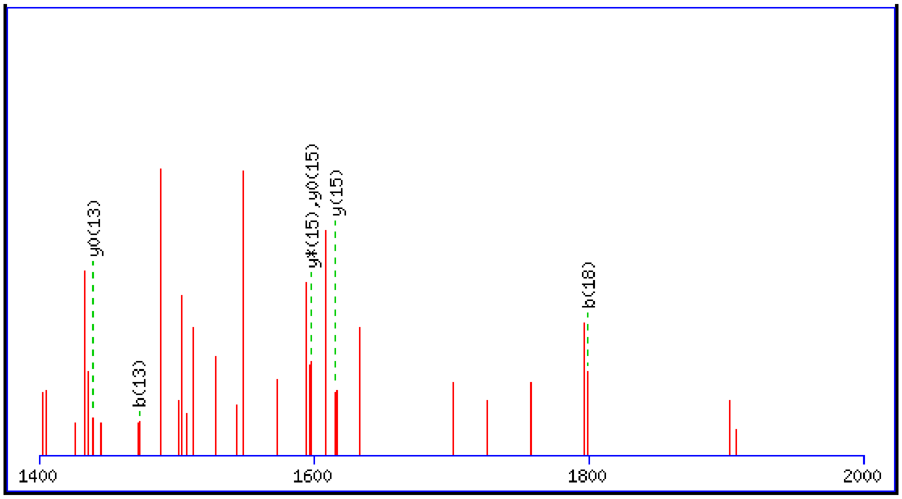

Monoisotopic mass of neutral peptide Mr(calc): 2797.3567
Fixed modifications: Carbamidomethyl (C)
Ions Score: 27 **Expect:** 0.52
Matches (Bold Red): 56/280 fragment ions using 131 most intense peaks

#	b	b^{++}	b*	b^{*++}	b^{0}	b^{0++}	Seq.	y	y^{++}	y*	y^{*++}	y^{0}	y^{0++}	#
1	129.1022	65.0548	112.0757	56.5415			K							25
2	228.1707	114.5890	211.1441	106.0757			V	2670.2691	1335.6382	2653.2425	1327.1249	2652.2585	1326.6329	24
3	341.2547	171.1310	324.2282	162.6177			I	2571.2007	1286.1040	2554.1741	**1277.5907**	2553.1901	**1277.0987**	23
4	470.2973	235.6523	453.2708	227.1390	**452.2867**	226.6470	E	2458.1166	1229.5619	2441.0900	1221.0487	2440.1060	1220.5567	22
5	**583.3814**	292.1943	566.3548	**283.6811**	565.3708	283.1890	I	2329.0740	**1165.0406**	2312.0475	**1156.5274**	2311.0634	1156.0354	21
6	697.4243	349.2158	680.3978	340.7025	679.4137	340.2105	N	2215.9899	1108.4986	2198.9634	**1099.9853**	2197.9794	**1099.4933**	20
7	794.4771	397.7422	777.4505	389.2289	776.4665	388.7369	P	2101.9470	1051.4771	2084.9205	1042.9639	2083.9364	1042.4719	19
8	**957.5404**	**479.2738**	940.5138	470.7606	939.5298	470.2686	Y	2004.8942	1002.9508	1987.8677	994.4375	1986.8837	993.9455	18
9	**1070.6245**	535.8159	1053.5979	527.3026	1052.6139	526.8106	L	1841.8309	921.4191	1824.8044	912.9058	1823.8204	912.4138	17
10	1183.7085	592.3579	1166.6820	**583.8446**	1165.6980	**583.3526**	L	1728.7469	**864.8771**	1711.7203	856.3638	1710.7363	855.8718	16
11	**1240.7300**	620.8686	1223.7034	612.3554	1222.7194	611.8633	G	**1615.6628**	**808.3350**	**1598.6362**	799.8218	**1597.6522**	799.3298	15
12	1341.7777	671.3925	1324.7511	662.8792	1323.7671	662.3872	T	1558.6413	**779.8243**	1541.6148	771.3110	1540.6308	770.8190	14
13	**1472.8181**	736.9127	1455.7916	728.3994	1454.8076	727.9074	M	1457.5936	729.3005	1440.5671	720.7872	**1439.5831**	720.2952	13
14	1543.8553	**772.4313**	1526.8287	763.9180	1525.8447	763.4260	A	1326.5532	663.7802	1309.5266	655.2669	1308.5426	654.7749	12
15	1600.8767	**800.9420**	1583.8502	792.4287	1582.8662	791.9367	G	1255.5160	**628.2617**	**1238.4895**	619.7484	1237.5055	619.2564	11
16	1657.8982	829.4527	1640.8716	820.9395	1639.8876	820.4475	G	**1198.4946**	**599.7509**	1181.4680	591.2377	1180.4840	590.7456	10
17	1728.9353	**864.9713**	1711.9088	856.4580	1710.9247	855.9660	A	**1141.4731**	571.2402	1124.4466	**562.7269**	**1123.4626**	**562.2349**	9
18	**1799.9724**	**900.4898**	1782.9459	891.9766	1781.9619	891.4846	A	**1070.4360**	535.7216	1053.4095	527.2084	1052.4254	526.7164	8
19	1914.9994	**958.0033**	1897.9728	949.4900	1896.9888	948.9980	D	**999.3989**	500.2031	982.3723	**491.6898**	981.3883	**491.1978**	7
20	2075.0300	**1038.0186**	2058.0035	1029.5054	2057.0194	1029.0134	C	**884.3719**	**442.6896**	867.3454	434.1763	866.3614	433.6843	6
21	2162.0620	**1081.5347**	2145.0355	1073.0214	2144.0515	1072.5294	S	**724.3413**	362.6743	707.3148	354.1610	706.3307	353.6690	5
22	2309.1305	**1155.0689**	2292.1039	**1146.5556**	2291.1199	**1146.0636**	F	**637.3093**	319.1583	620.2827	310.6450	619.2987	310.1530	4
23	2495.2098	**1248.1085**	2478.1832	**1239.5952**	2477.1992	**1239.1032**	W	**490.2409**	245.6241	473.2143	237.1108	472.2303	236.6188	3
24	2624.2524	**1312.6298**	2607.2258	1304.1165	2606.2418	1303.6245	E	**304.1615**	152.5844	287.1350	144.0711	286.1510	143.5791	2
25							R	175.1190	88.0631	158.0924	79.5498			1

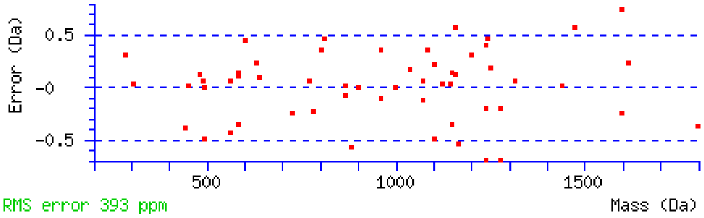

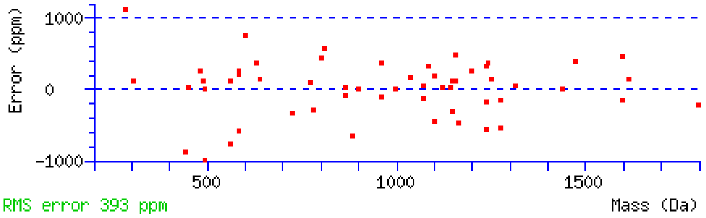

All matches to this query

Score	Mr(calc):	Delta	Sequence
26.5	2797.3567	1.0160	KVIEINPYLLGTMAGGAADCSFWER
18.1	2798.3962	-0.0235	LPEEHARFYAAEICIALNFLHER
15.3	2797.3800	0.9927	FQDVGPQAPVGSVYQKTNAVSEIKR
11.5	2797.3565	1.0162	GEGGQMARIASESAGSDPLERLPWQR
10.6	2796.3881	1.9846	LGDSSDLLSPGGPGPPLPMSSPGLGKKR
9.1	2797.3800	0.9927	FQDVGPQAPVGSVYQKTNAVSEIKR
8.6	2797.3800	0.9927	FQDVGPQAPVGSVYQKTNAVSEIKR
8.6	2797.3800	0.9927	FQDVGPQAPVGSVYQKTNAVSEIKR
7.8	2797.3452	1.0275	YDELYAALTALLAAGSQLDTVRRK
7.6	2796.3881	1.9846	LGDSSDLLSPGGPGPPLPMSSPGLGKKR

Spectrum No: 38; Query: 9350; Rank: 1

Peptide View

MS/MS Fragmentation of **LLANMVYQYK**
Found in **IPI00383971**, Tax_Id=9606 Gene_Symbol=PSMB5 proteasome beta 5 subunit isoform 3

Match to Query 9350: 1241.653808 from(621.834180,2+)
Title: 091224LimSK_Exosome3_07.3815.3815.2.dta
Data file C:\Temp\Mascoti\LSK_2D\091224LimSK_Exosome3_01_2D.mgf

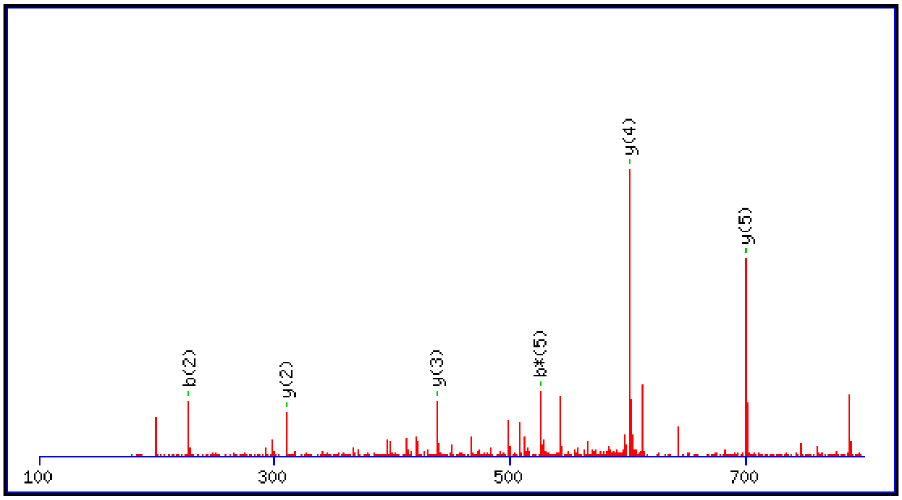

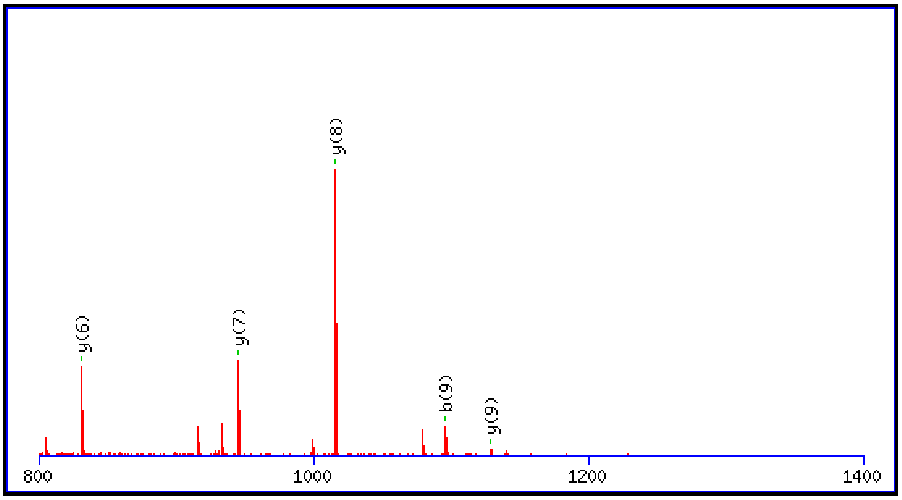

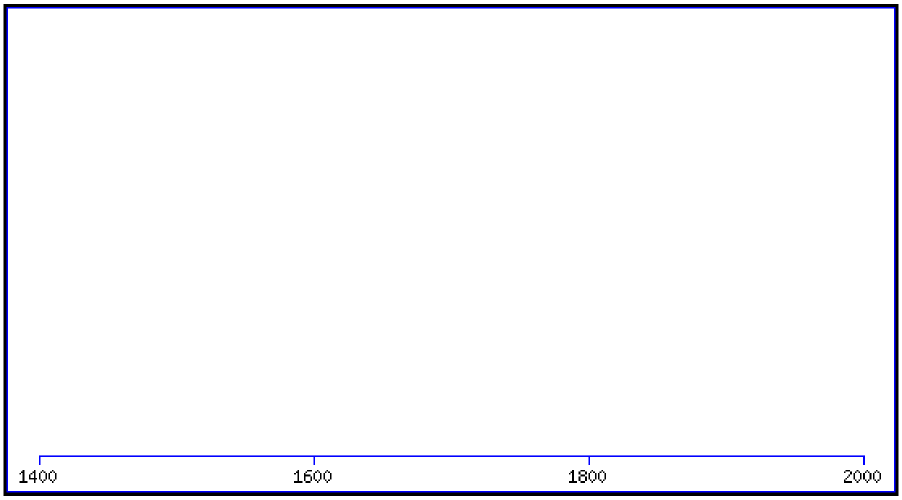

Monoisotopic mass of neutral peptide Mr(calc): 1241.6478
Fixed modifications: Carbamidomethyl (C)
Ions Score: 67 **Expect:** 2.3e-005
Matches (Bold Red): 11/66 fragment ions using 20 most intense peaks

#	b	b++	b*	b*++	Seq.	y	y++	y*	y*++	#
1	114.0913	57.5493			L					10
2	**227.1754**	114.0913			L	**1129.5710**	565.2892	1112.5445	556.7759	9
3	298.2125	149.6099			A	**1016.4870**	508.7471	999.4604	500.2339	8
4	412.2554	206.6314	395.2289	198.1181	N	**945.4499**	473.2286	928.4233	464.7153	7
5	543.2959	272.1516	**526.2694**	263.6383	M	**831.4069**	416.2071	814.3804	407.6938	6
6	642.3643	321.6858	625.3378	313.1725	V	**700.3665**	350.6869	683.3399	342.1736	5
7	805.4277	403.2175	788.4011	394.7042	Y	**601.2980**	301.1527	584.2715	292.6394	4
8	933.4863	467.2468	916.4597	458.7335	Q	**438.2347**	219.6210	421.2082	211.1077	3
9	**1096.5496**	548.7784	1079.5230	540.2652	Y	**310.1761**	155.5917	293.1496	147.0784	2
10					K	147.1128	74.0600	130.0863	65.5468	1

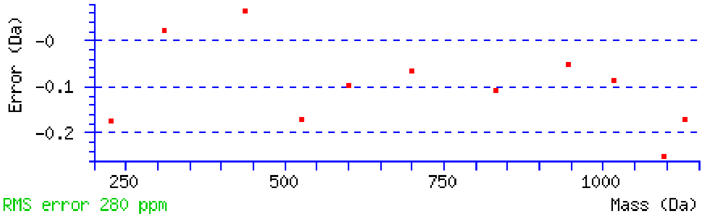

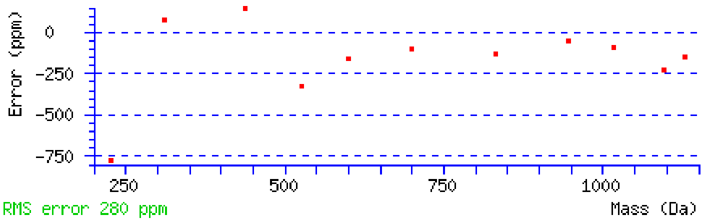

All matches to this query

Score	Mr(calc):	Delta	Sequence
67.0	1241.6478	0.0060	LLANMVYQYK
0.7	1239.6476	2.0062	TLLSGSASVVVK
0.2	1239.6360	2.0178	GRLANQYYQK

Spectrum No: 39; Query: 5936; Rank: 1

Peptide View

MS/MS Fragmentation of **RAIYQATYR**
Found in **IPI00479306**, Tax_Id=9606 Gene_Symbol=PSMB5 Proteasome subunit beta type-5

Match to Query 5936: 1140.607388 from(571.310970,2+)

Title: 091224LimSK_Exosome3_07.561.561.2.dta
Data file C:\Temp\Mascoti\LSK_2D\091224LimSK_Exosome3_01_2D.mgf

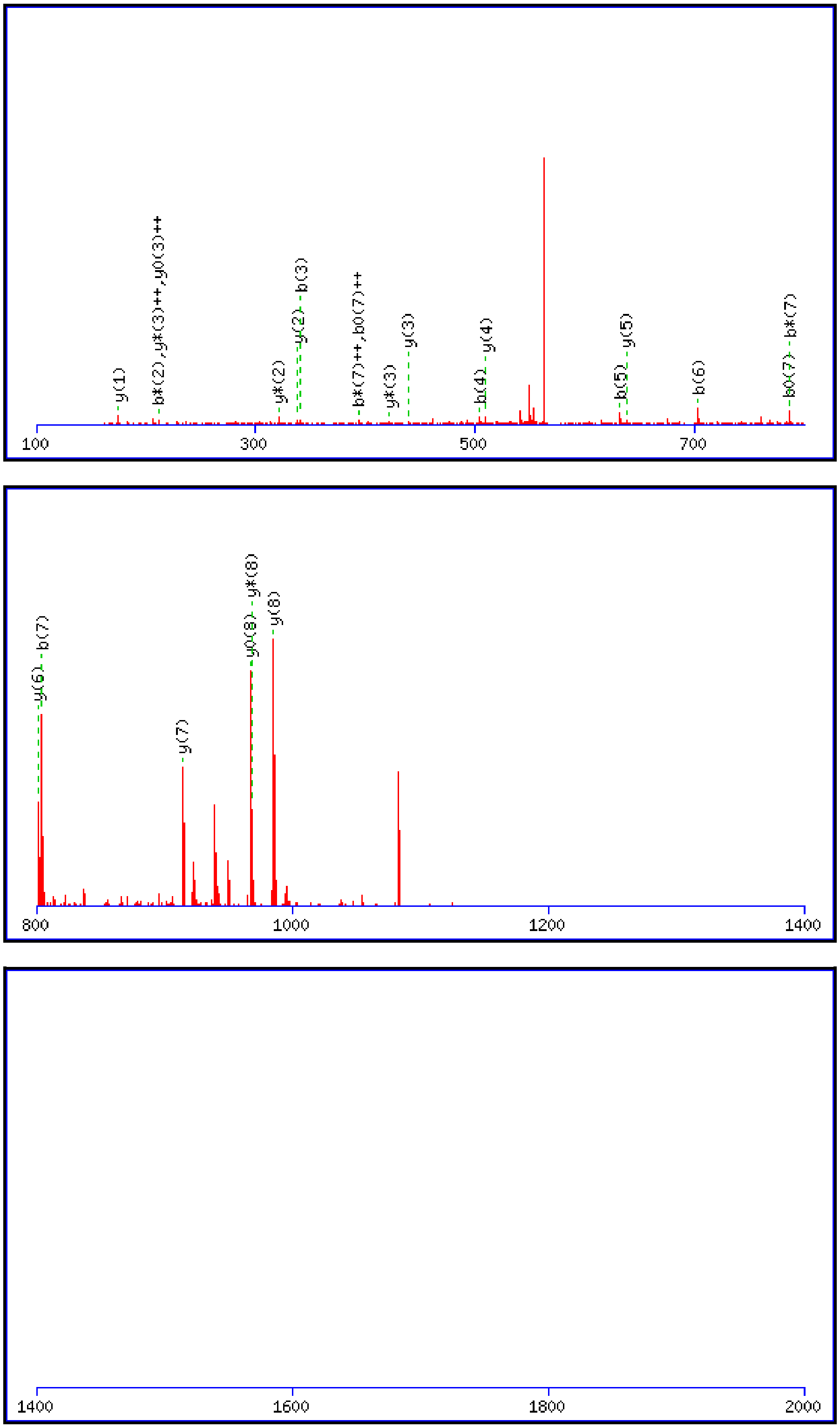

Monoisotopic mass of neutral peptide Mr(calc): 1140.6040
Fixed modifications: Carbamidomethyl (C)
Ions Score: 39 **Expect:** 0.013
Matches (Bold Red): 25/80 fragment ions using 50 most intense peaks

#	b	b++	b*	b*++	b0	b0++	Seq.	y	y++	y*	y*++	y0	y0++	#
1	157.1084	79.0578	140.0818	70.5446			R							9
2	228.1455	114.5764	**211.1190**	106.0631			A	**985.5102**	493.2587	**968.4836**	484.7454	**967.4996**	484.2534	8
3	**341.2296**	171.1184	324.2030	162.6051			I	**914.4730**	457.7402	897.4465	449.2269	896.4625	448.7349	7
4	**504.2929**	252.6501	487.2663	244.1368			Y	**801.3890**	401.1981	784.3624	392.6849	783.3784	392.1928	6

5	**632.3515**	316.6794	615.3249	308.1661			Q	**638.3257**	319.6665	621.2991	311.1532	620.3151	310.6612	5
6	**703.3886**	352.1979	686.3620	343.6847			A	**510.2671**	255.6372	493.2405	247.1239	492.2565	246.6319	4
7	**804.4363**	402.7218	**787.4097**	**394.2085**	**786.4257**	**393.7165**	T	**439.2300**	220.1186	**422.2034**	**211.6053**	421.2194	**211.1133**	3
8	**967.4996**	484.2534	950.4730	475.7402	949.4890	475.2482	Y	**338.1823**	169.5948	**321.1557**	161.0815			2
9							R	**175.1190**	88.0631	158.0924	79.5498			1

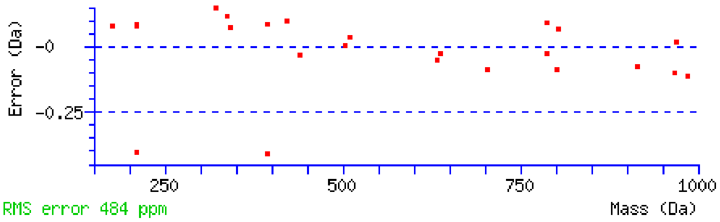

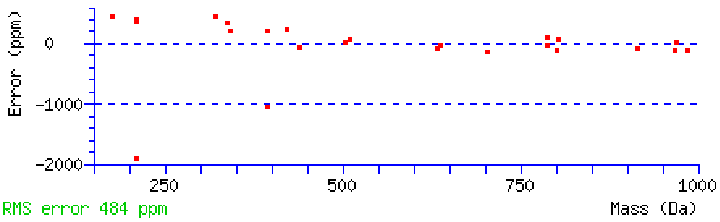

All matches to this query

Score	Mr(calc):	Delta	Sequence
39.4	1140.6040	0.0034	RAIYQATYR
23.1	1138.6095	1.9979	GPGKPLQEGTR
12.8	1139.6087	0.9987	LLXPPPGNYR
9.0	1140.5961	0.0113	YSSQIMLKR
8.9	1139.5975	1.0099	GDYLKELFR
7.8	1140.6179	-0.0105	LIIEYAYTR
6.0	1139.6087	0.9986	GPQGPPPAGLLH
5.2	1139.6104	0.9970	KILVFTPSR
4.8	1139.6047	1.0027	GKEGSALSHVR
4.7	1140.5961	0.0113	IAPGLPGCQTK

Spectrum No: 40; Query: 24678; Rank: 1

Peptide View

MS/MS Fragmentation of **RGPGLYYVDSEGNR**
Found in **IPI00479306**, Tax_Id=9606 Gene_Symbol=PSMB5 Proteasome subunit beta type-5

Match to Query 24678: 1581.765588 from(791.890070,2+)
Title: 091224LimSK_Exosome3_07.1767.1767.2.dta
Data file C:\Temp\Mascoti\LSK_2D\091224LimSK_Exosome3_01_2D.mgf

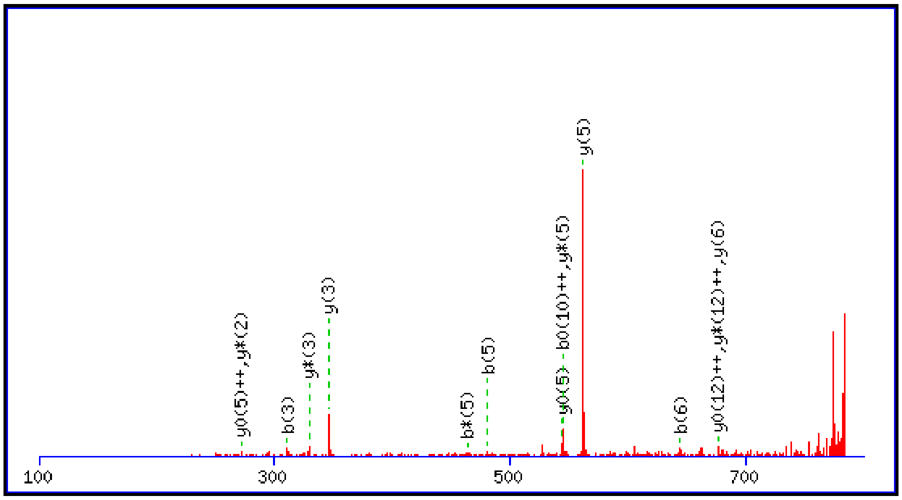

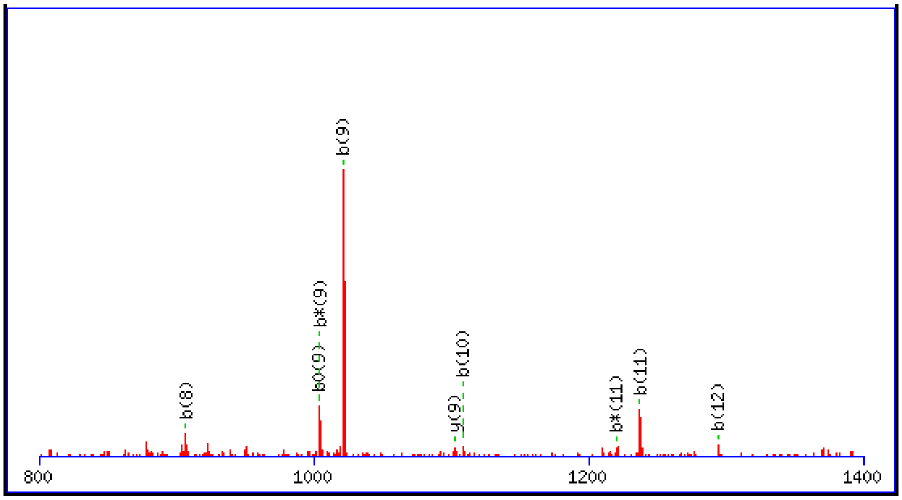

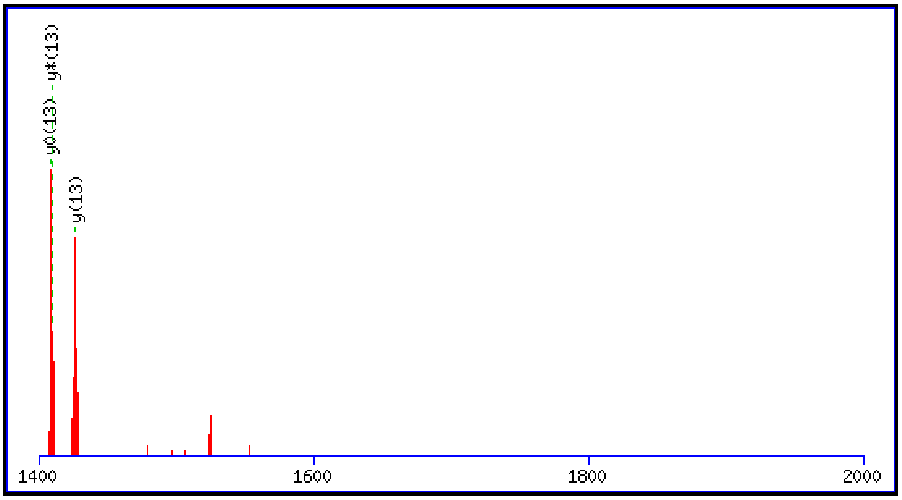

Monoisotopic mass of neutral peptide Mr(calc): 1581.7536
Fixed modifications: Carbamidomethyl (C)
Ions Score: 58 **Expect:** 0.00031
Matches (Bold Red): 28/134 fragment ions using 57 most intense peaks

#	b	b^{++}	b*	b^{*++}	b^0	b^{0++}	Seq.	y	y^{++}	y*	y^{*++}	y^0	y^{0++}	#
1	157.1084	79.0578	140.0818	70.5446			R							14
2	214.1299	107.5686	197.1033	99.0553			G	**1426.6597**	713.8335	**1409.6332**	705.3202	**1408.6492**	704.8282	13
3	**311.1826**	156.0949	294.1561	147.5817			P	1369.6383	685.3228	1352.6117	**676.8095**	1351.6277	**676.3175**	12
4	368.2041	184.6057	351.1775	176.0924			G	1272.5855	636.7964	1255.5590	628.2831	1254.5749	627.7911	11
5	**481.2881**	241.1477	**464.2616**	232.6344			L	1215.5640	608.2857	1198.5375	599.7724	1197.5535	599.2804	10
6	**644.3515**	322.6794	627.3249	314.1661			Y	**1102.4800**	551.7436	1085.4534	543.2304	1084.4694	542.7383	9
7	807.4148	404.2110	790.3883	395.6978			Y	939.4166	470.2120	922.3901	461.6987	921.4061	461.2067	8
8	**906.4832**	453.7452	889.4567	445.2320			V	776.3533	388.6803	759.3268	380.1670	758.3428	379.6750	7
9	**1021.5102**	511.2587	**1004.4836**	502.7454	**1003.4996**	502.2534	D	**677.2849**	339.1461	660.2584	330.6328	659.2743	330.1408	6
10	**1108.5422**	554.7747	1091.5156	546.2615	1090.5316	**545.7694**	S	**562.2580**	281.6326	**545.2314**	273.1193	**544.2474**	**272.6273**	5
11	**1237.5848**	619.2960	**1220.5582**	610.7828	1219.5742	610.2907	E	475.2259	238.1166	458.1994	229.6033	457.2154	229.1113	4
12	**1294.6062**	647.8068	1277.5797	639.2935	1276.5957	638.8015	G	**346.1833**	173.5953	**329.1568**	165.0820			3
13	**1408.6492**	704.8282	1391.6226	696.3149	1390.6386	695.8229	N	289.1619	145.0846	**272.1353**	136.5713			2
14							R	175.1190	88.0631	158.0924	79.5498			1

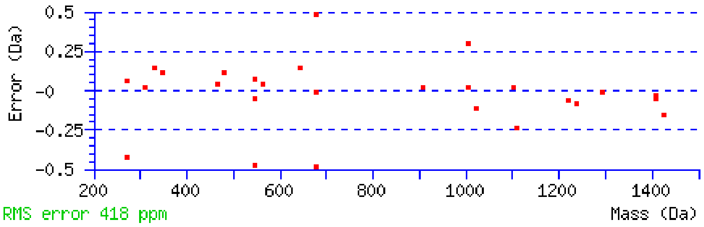

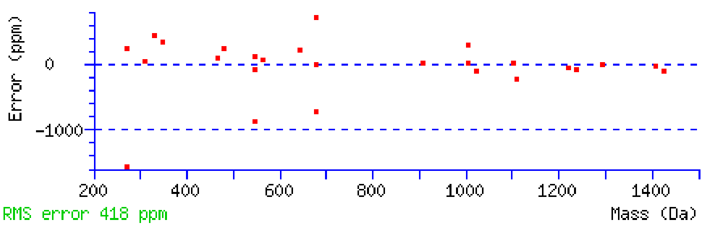

All matches to this query

Score	Mr(calc):	Delta	Sequence
57.8	1581.7536	0.0120	RGPGLYYVDSEGNR
10.4	1581.7651	0.0005	SLPPSDSKKTVESK
10.4	1581.7651	0.0005	XLPPSDSKKTVESK
9.8	1581.7651	0.0005	SLPPSDSKKTVESK
9.8	1581.7651	0.0005	XLPPSDSKKTVESK
7.2	1580.7534	1.0121	GGMIVDPLRSGFPR
6.6	1581.7569	0.0087	ECGKSFSSLGNLQR
3.2	1579.7743	1.9913	GNSLWGFKAGSSNQK
2.7	1581.7810	-0.0154	MVAAAAATEARLRR
2.4	1581.7722	-0.0066	HPWLQDEDMKRK

Spectrum No: 41; Query: 59664; Rank: 1

Peptide View

MS/MS Fragmentation of **VIEINPYLLGTMAGGAADCSFWER**
Found in **IPI00383971**, Tax_Id=9606 Gene_Symbol=PSMB5 proteasome beta 5 subunit isoform 3

Match to Query 59664: 2669.281422 from(890.767750,3+)
Title: 091224LimSK_Exosome3_07.8862.8862.3.dta
Data file C:\Temp\Mascoti\LSK_2D\091224LimSK_Exosome3_01_2D.mgf

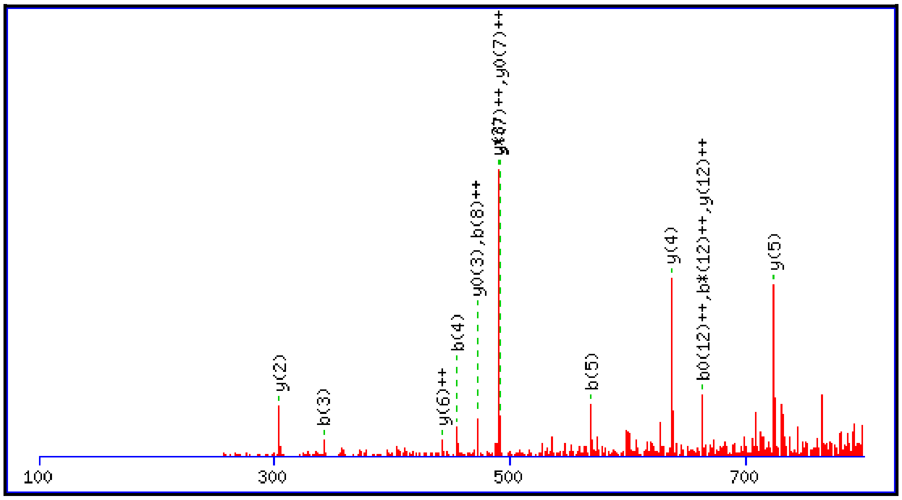

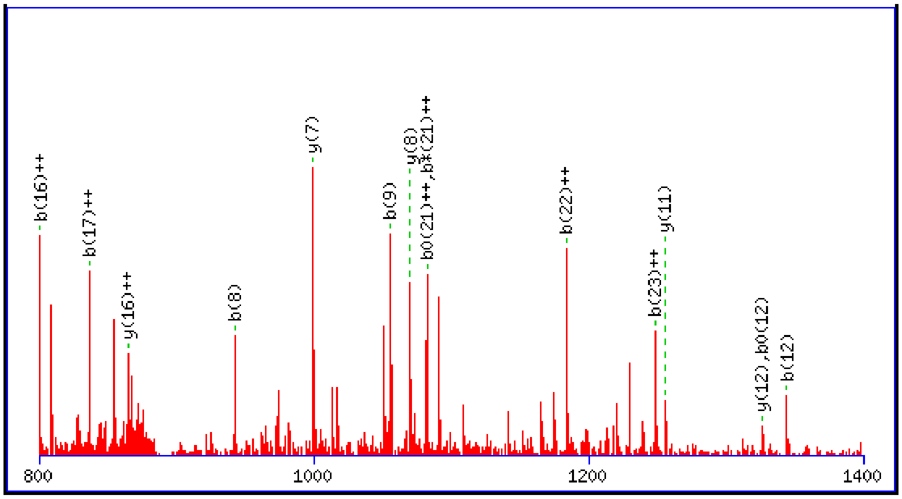

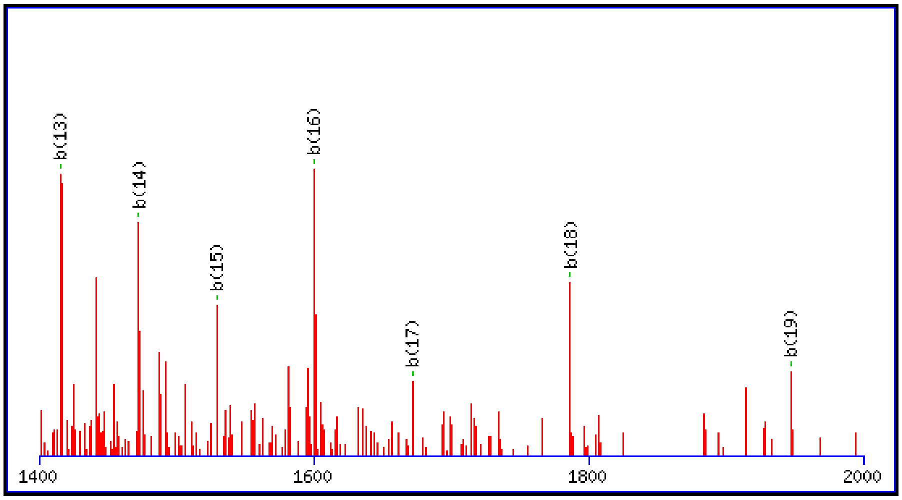

Monoisotopic mass of neutral peptide Mr(calc): 2669.2618
Fixed modifications: Carbamidomethyl (C)
Ions Score: 105 **Expect:** 8.9e-009
Matches (Bold Red): 37/262 fragment ions using 42 most intense peaks

#	b	b^{++}	b*	b^{*++}	b^{0}	b^{0++}	Seq.	y	y^{++}	y*	y^{*++}	y^{0}	y^{0++}	#
1	100.0757	50.5415					V							24
2	213.1598	107.0835					I	2571.2007	1286.1040	2554.1741	1277.5907	2553.1901	1277.0987	23
3	**342.2023**	171.6048			324.1918	162.5995	E	2458.1166	1229.5619	2441.0900	1221.0487	2440.1060	1220.5567	22
4	**455.2864**	228.1468			437.2758	219.1416	I	2329.0740	1165.0406	2312.0475	1156.5274	2311.0634	1156.0354	21
5	**569.3293**	285.1683	552.3028	276.6550	551.3188	276.1630	N	2215.9899	1108.4986	2198.9634	1099.9853	2197.9794	1099.4933	20
6	666.3821	333.6947	649.3556	325.1814	648.3715	324.6894	P	2101.9470	1051.4771	2084.9205	1042.9639	2083.9364	1042.4719	19
7	829.4454	415.2264	812.4189	406.7131	811.4349	406.2211	Y	2004.8942	1002.9508	1987.8677	994.4375	1986.8837	993.9455	18
8	**942.5295**	**471.7684**	925.5029	463.2551	924.5189	462.7631	L	1841.8309	921.4191	1824.8044	912.9058	1823.8204	912.4138	17
9	**1055.6136**	528.3104	1038.5870	519.7971	1037.6030	519.3051	L	1728.7469	**864.8771**	1711.7203	856.3638	1710.7363	855.8718	16
10	1112.6350	556.8211	1095.6085	548.3079	1094.6245	547.8159	G	1615.6628	808.3350	1598.6362	799.8218	1597.6522	799.3298	15
11	1213.6827	607.3450	1196.6562	598.8317	1195.6721	598.3397	T	1558.6413	779.8243	1541.6148	771.3110	1540.6308	770.8190	14
12	**1344.7232**	672.8652	1327.6966	**664.3520**	**1326.7126**	**663.8599**	M	1457.5936	729.3005	1440.5671	720.7872	1439.5831	720.2952	13
13	**1415.7603**	708.3838	1398.7338	699.8705	1397.7497	699.3785	A	**1326.5532**	**663.7802**	1309.5266	655.2669	1308.5426	654.7749	12
14	**1472.7818**	736.8945	1455.7552	728.3812	1454.7712	727.8892	G	**1255.5160**	628.2617	1238.4895	619.7484	1237.5055	619.2564	11
15	**1529.8032**	765.4053	1512.7767	756.8920	1511.7927	756.4000	G	1198.4946	599.7509	1181.4680	591.2377	1180.4840	590.7456	10
16	**1600.8403**	**800.9238**	1583.8138	792.4105	1582.8298	791.9185	A	1141.4731	571.2402	1124.4466	562.7269	1123.4626	562.2349	9
17	**1671.8775**	**836.4424**	1654.8509	827.9291	1653.8669	827.4371	A	**1070.4360**	535.7216	1053.4095	527.2084	1052.4254	526.7164	8
18	**1786.9044**	893.9558	1769.8779	885.4426	1768.8938	884.9506	D	**999.3989**	500.2031	982.3723	**491.6898**	981.3883	**491.1978**	7
19	**1946.9350**	973.9712	1929.9085	965.4579	1928.9245	964.9659	C	884.3719	**442.6896**	867.3454	434.1763	866.3614	433.6843	6

20	2033.9671	1017.4872	2016.9405	1008.9739	2015.9565	1008.4819	S	724.3413	362.6743	707.3148	354.1610	706.3307	353.6690	5
21	2181.0355	1091.0214	2164.0089	1082.5081	2163.0249	1082.0161	F	637.3093	319.1583	620.2827	310.6450	619.2987	310.1530	4
22	2367.1148	1184.0610	2350.0883	1175.5478	2349.1042	1175.0558	W	490.2409	245.6241	473.2143	237.1108	472.2303	236.6188	3
23	2496.1574	1248.5823	2479.1308	1240.0691	2478.1468	1239.5771	E	304.1615	152.5844	287.1350	144.0711	286.1510	143.5791	2
24							R	175.1190	88.0631	158.0924	79.5498			1

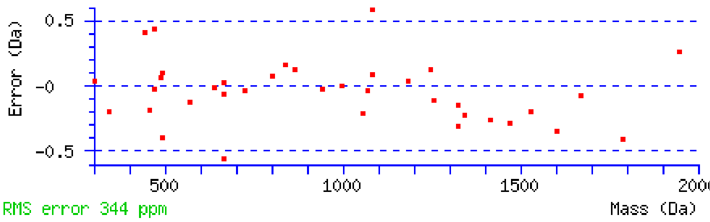

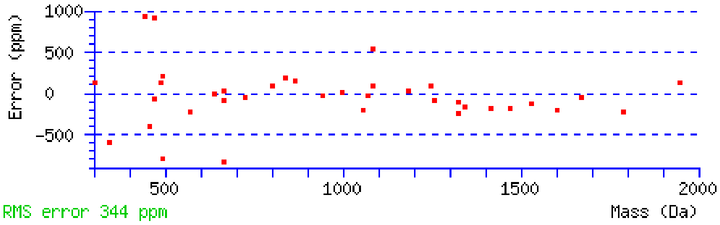

All matches to this query

Score	Mr(calc):	Delta	Sequence
104.9	2669.2618	0.0196	VIEINPYLLGTMAGGAADCSFWER
8.1	2667.2727	2.0087	HYTSQQELKDECALVPRTLATK
8.1	2667.2727	2.0087	HYTSQQELKDECALVPRTLATK
8.1	2669.2840	-0.0026	SPAVPSGLSGGRTWSLRALISGGGAR
8.1	2669.2840	-0.0026	SPAVPSGLSGGRTWSLRALISGGGAR
8.1	2669.2840	-0.0026	SPAVPSGLSGGRTWSLRALISGGGAR
7.6	2667.2808	2.0006	DFILTVSIVIGVGGCWFAYTQNK
6.8	2667.2727	2.0087	HYTSQQELKDECALVPRTLATK
6.7	2669.3067	-0.0253	VNLQNNPGAMEHFHMKLFRAQR
5.6	2667.2515	2.0299	SQYLQKFSPPLLGKGLAALDGPK

Spectrum No: 42; Query: 11753; Rank: 1

Peptide View

MS/MS Fragmentation of **VSSDNVADLHEK**
Found in **IPI00479306**, Tax_Id=9606 Gene_Symbol=PSMB5 Proteasome subunit beta type-5

Match to Query 11753: 1312.631088 from(657.322820,2+)
Title: 091224LimSK_Exosome3_07.320.320.2.dta
Data file C:\Temp\Mascoti\LSK_2D\091224LimSK_Exosome3_01_2D.mgf

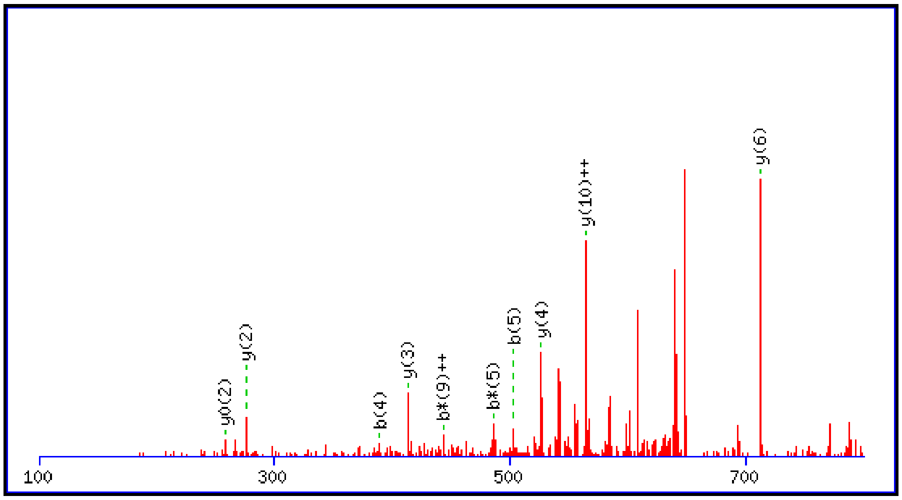

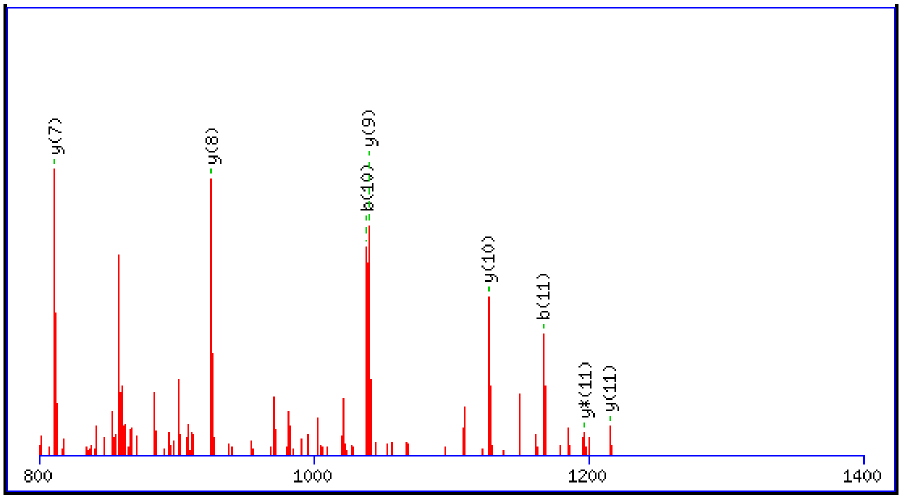

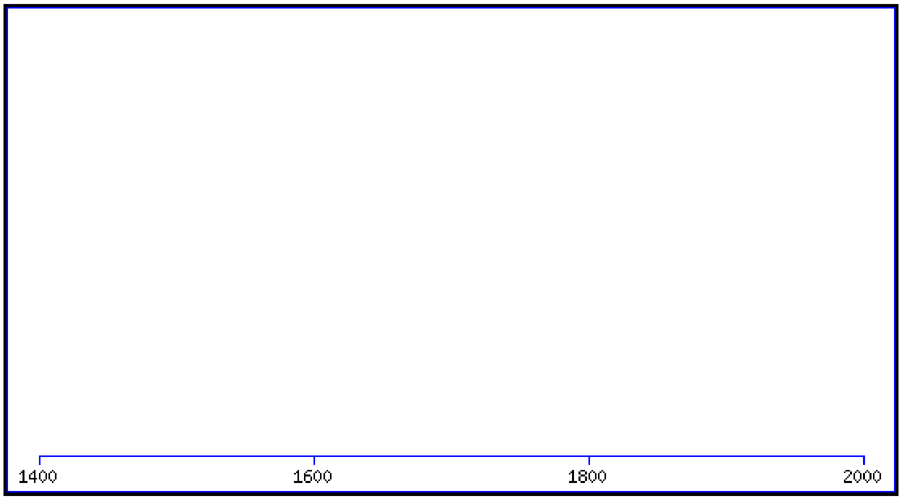

Monoisotopic mass of neutral peptide Mr(calc): 1312.6259
Fixed modifications: Carbamidomethyl (C)
Ions Score: 77 **Expect:** 3.5e-006
Matches (Bold Red): 18/120 fragment ions using 27 most intense peaks

#	b	b^{++}	b*	b^{*++}	b^0	b^{0++}	Seq.	y	y^{++}	y*	y^{*++}	y^0	y^{0++}	#
1	100.0757	50.5415					V							12
2	187.1077	94.0575			169.0972	85.0522	S	**1214.5648**	607.7860	**1197.5382**	599.2727	1196.5542	598.7807	11
3	274.1397	137.5735			256.1292	128.5682	S	**1127.5327**	**564.2700**	1110.5062	555.7567	1109.5222	555.2647	10
4	**389.1667**	195.0870			371.1561	186.0817	D	**1040.5007**	520.7540	1023.4742	512.2407	1022.4901	511.7487	9
5	**503.2096**	252.1084	**486.1831**	243.5952	485.1991	243.1032	N	**925.4738**	463.2405	908.4472	454.7272	907.4632	454.2352	8
6	602.2780	301.6427	585.2515	293.1294	584.2675	292.6374	V	**811.4308**	406.2191	794.4043	397.7058	793.4203	397.2138	7
7	673.3151	337.1612	656.2886	328.6479	655.3046	328.1559	A	**712.3624**	356.6849	695.3359	348.1716	694.3519	347.6796	6
8	788.3421	394.6747	771.3155	386.1614	770.3315	385.6694	D	641.3253	321.1663	624.2988	312.6530	623.3148	312.1610	5
9	901.4262	451.2167	884.3996	**442.7034**	883.4156	442.2114	L	**526.2984**	263.6528	509.2718	255.1396	508.2878	254.6475	4
10	**1038.4851**	519.7462	1021.4585	511.2329	1020.4745	510.7409	H	**413.2143**	207.1108	396.1878	198.5975	395.2037	198.1055	3
11	**1167.5277**	584.2675	1150.5011	575.7542	1149.5171	575.2622	E	**276.1554**	138.5813	259.1288	130.0681	**258.1448**	129.5761	2
12							K	147.1128	74.0600	130.0863	65.5468			1

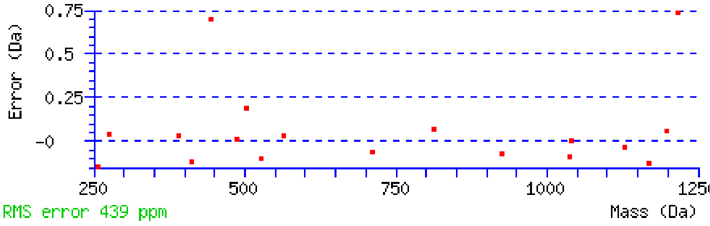

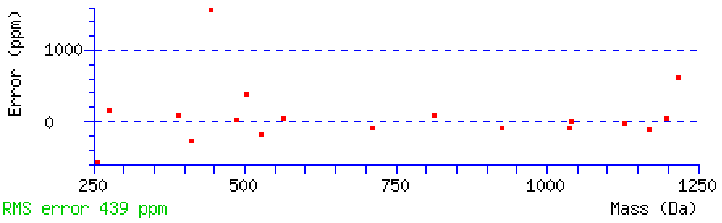

All matches to this query

Score	Mr(calc):	Delta	Sequence
76.7	1312.6259	0.0052	VSSDNVADLHEK
17.2	1312.6372	-0.0061	VTGSAGTTEPHTR
13.4	1312.6388	-0.0077	SGTSATLGITGLR
13.1	1310.6272	2.0039	NSTAWLLLGGIS
9.8	1312.6388	-0.0077	SVKNSITQQTK
9.2	1312.6340	-0.0029	SVRMASSRMTR
8.7	1312.6306	0.0005	EGRLQQGNMHK
8.4	1312.6411	-0.0100	EIEQSWKHEK
7.5	1312.6349	-0.0038	VSSMPIELLTK
7.4	1312.6428	-0.0117	GAFLDKPKTEK

Spectrum No: 43; Query: 4059; Rank: 1

Peptide View

MS/MS Fragmentation of **AANGVVLATEK**
Found in **IPI00219622**, Tax_Id=9606 Gene_Symbol=PSMA2 Proteasome subunit alpha type-2

Match to Query 4059: 1071.597168 from(536.805860,2+)
Title: 091224LimSK_Exosome3_06.531.531.2.dta
Data file C:\Temp\Mascoti\LSK_2D\091224LimSK_Exosome3_01_2D.mgf

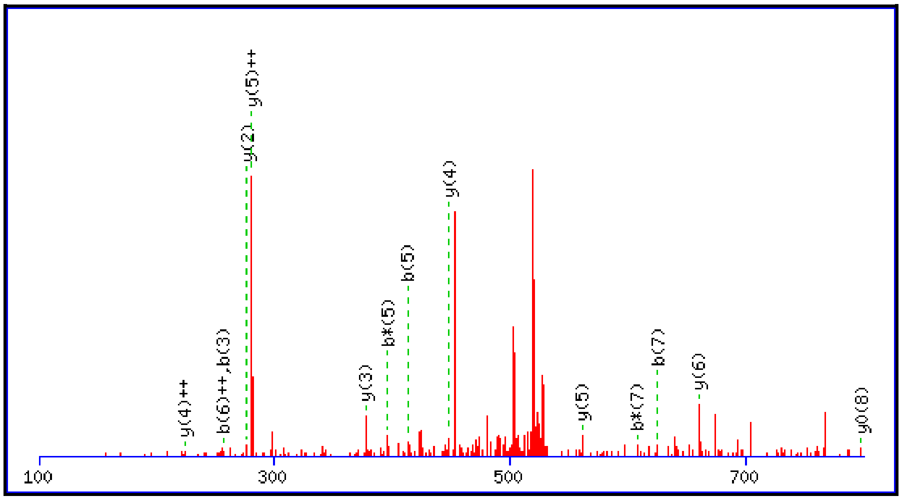

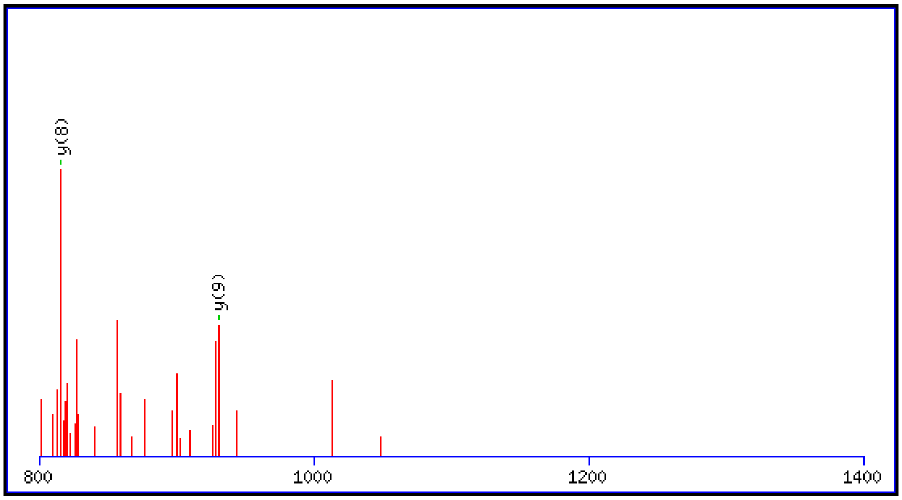

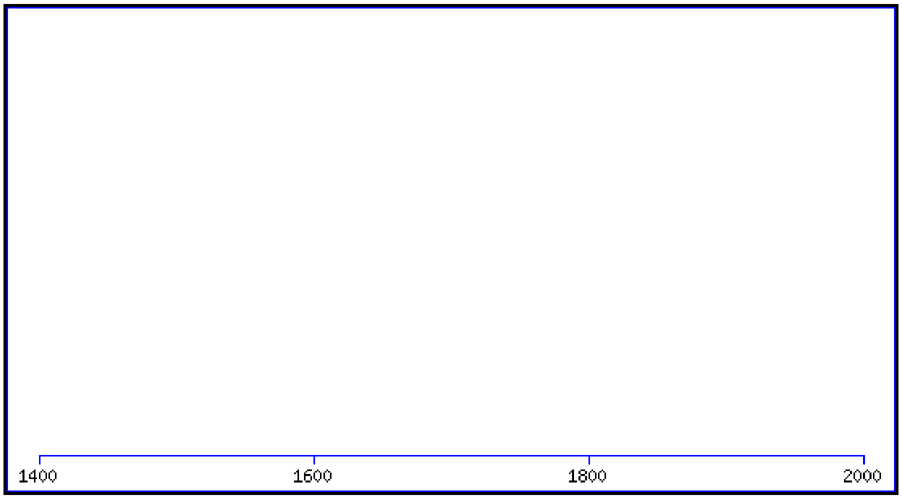

Monoisotopic mass of neutral peptide Mr(calc): 1071.5924
Fixed modifications: Carbamidomethyl (C)
Ions Score: 21 **Expect:** 0.88
Matches (Bold Red): 16/98 fragment ions using 58 most intense peaks

#	b	b++	b*	b*++	b0	b0++	Seq.	y	y++	y*	y*++	y0	y0++	#
1	72.0444	36.5258					A							11
2	143.0815	72.0444					A	1001.5626	501.2849	984.5360	492.7717	983.5520	492.2796	10
3	**257.1244**	129.0659	240.0979	120.5526			N	**930.5255**	465.7664	913.4989	457.2531	912.5149	456.7611	9
4	314.1459	157.5766	297.1193	149.0633			G	**816.4825**	408.7449	799.4560	400.2316	**798.4720**	399.7396	8
5	**413.2143**	207.1108	**396.1878**	198.5975			V	759.4611	380.2342	742.4345	371.7209	741.4505	371.2289	7
6	512.2827	**256.6450**	495.2562	248.1317			V	**660.3927**	330.7000	643.3661	322.1867	642.3821	321.6947	6
7	**625.3668**	313.1870	**608.3402**	304.6738			L	**561.3243**	**281.1658**	544.2977	272.6525	543.3137	272.1605	5
8	696.4039	348.7056	679.3774	340.1923			A	**448.2402**	**224.6237**	431.2136	216.1105	430.2296	215.6185	4
9	797.4516	399.2294	780.4250	390.7162	779.4410	390.2241	T	**377.2031**	189.1052	360.1765	180.5919	359.1925	180.0999	3
10	926.4942	463.7507	909.4676	455.2375	908.4836	454.7454	E	**276.1554**	138.5813	259.1288	130.0681	258.1448	129.5761	2
11							K	147.1128	74.0600	130.0863	65.5468			1

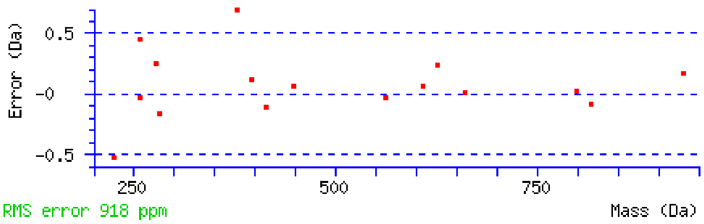

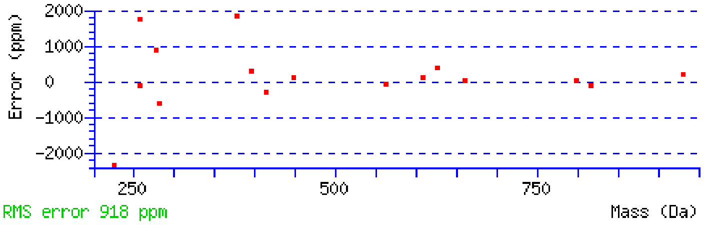

All matches to this query

Score	Mr(calc):	Delta	Sequence
20.8	1071.5924	0.0048	AANGVVLATEK

Spectrum No: 44; Query: 19275; Rank: 1

Peptide View

MS/MS Fragmentation of **GYSFSLTTFSPSGK**
Found in **IPI00219622**, Tax_Id=9606 Gene_Symbol=PSMA2 Proteasome subunit alpha type-2

Match to Query 19275: 1477.716268 from(739.865410,2+)
Title: 091224LimSK_Exosome3_06.5477.5477.2.dta
Data file C:\Temp\Mascoti\LSK_2D\091224LimSK_Exosome3_01_2D.mgf

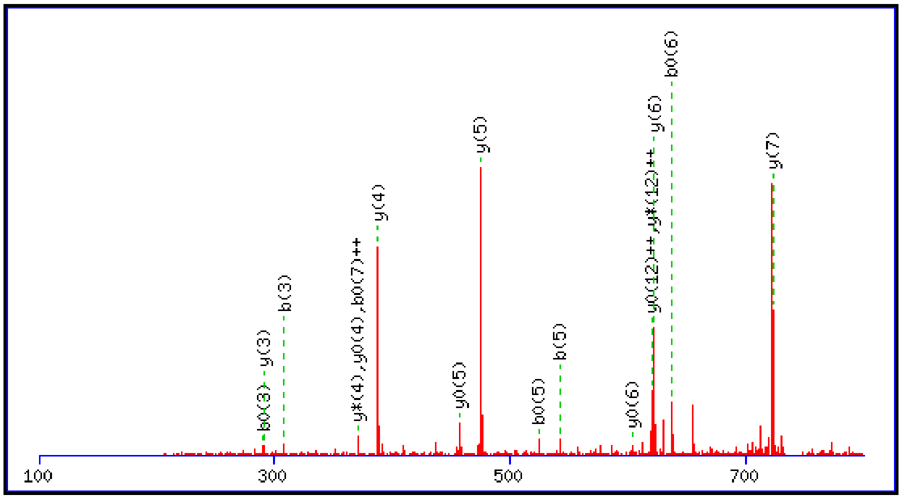

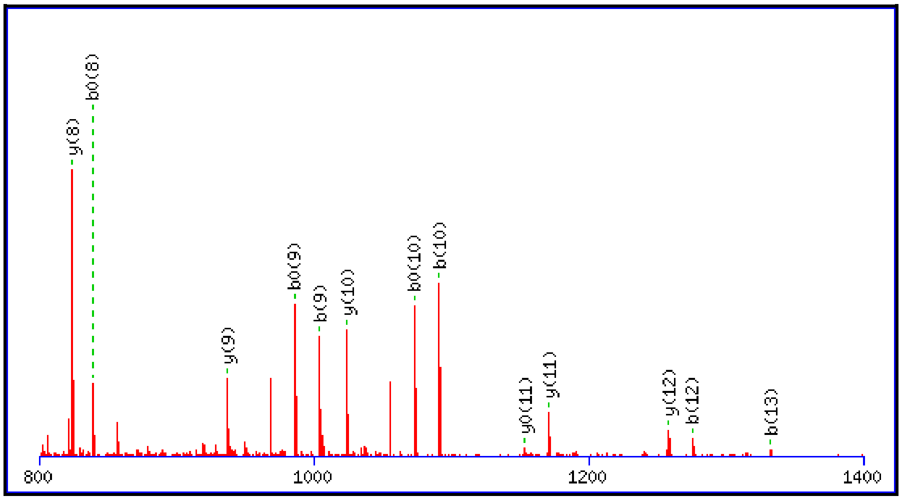

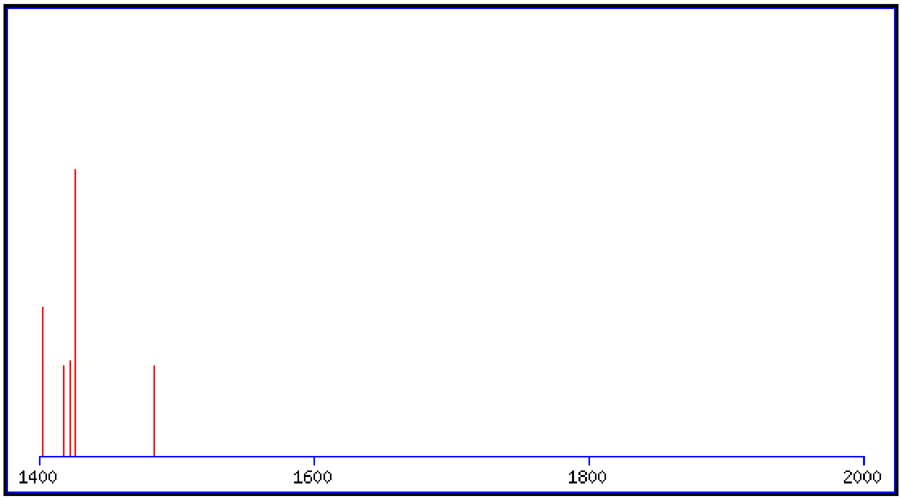

Monoisotopic mass of neutral peptide Mr(calc): 1477.7089
Fixed modifications: Carbamidomethyl (C)
Ions Score: 76 **Expect:** 4.4e-006
Matches (Bold Red): 30/122 fragment ions using 47 most intense peaks

#	b	b^{++}	b^0	b^{0++}	Seq.	y	y^{++}	y*	y^{*++}	y^0	y^{0++}	#
1	58.0287	29.5180			G							14
2	221.0921	111.0497			Y	1421.6947	711.3510	1404.6682	702.8377	1403.6842	702.3457	13
3	**308.1241**	154.5657	**290.1135**	145.5604	S	**1258.6314**	629.8193	1241.6048	**621.3061**	1240.6208	**620.8141**	12
4	455.1925	228.0999	437.1819	219.0946	F	**1171.5994**	586.3033	1154.5728	577.7900	**1153.5888**	577.2980	11
5	**542.2245**	271.6159	**524.2140**	262.6106	S	**1024.5310**	512.7691	1007.5044	504.2558	1006.5204	503.7638	10

**6**	655.3086	328.1579	**637.2980**	319.1527	**L**	**937.4989**	469.2531	920.4724	460.7398	919.4884	460.2478	**9**
**7**	756.3563	378.6818	738.3457	**369.6765**	**T**	**824.4149**	412.7111	807.3883	404.1978	806.4043	403.7058	**8**
**8**	857.4040	429.2056	**839.3934**	420.2003	**T**	**723.3672**	362.1872	706.3406	353.6740	705.3566	353.1819	**7**
**9**	**1004.4724**	502.7398	**986.4618**	493.7345	**F**	**622.3195**	311.6634	605.2930	303.1501	**604.3089**	302.6581	**6**
**10**	**1091.5044**	546.2558	**1073.4938**	537.2506	**S**	**475.2511**	238.1292	458.2245	229.6159	**457.2405**	229.1239	**5**
**11**	1188.5572	594.7822	1170.5466	585.7769	**P**	**388.2191**	194.6132	**371.1925**	186.0999	**370.2085**	185.6079	**4**
**12**	**1275.5892**	638.2982	1257.5786	629.2930	**S**	**291.1663**	146.0868	274.1397	137.5735	273.1557	137.0815	**3**
**13**	**1332.6107**	666.8090	1314.6001	657.8037	**G**	204.1343	102.5708	187.1077	94.0575			**2**
**14**					**K**	147.1128	74.0600	130.0863	65.5468			**1**

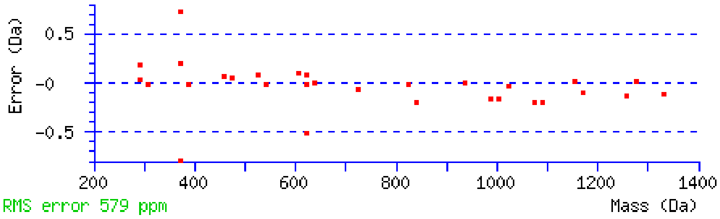

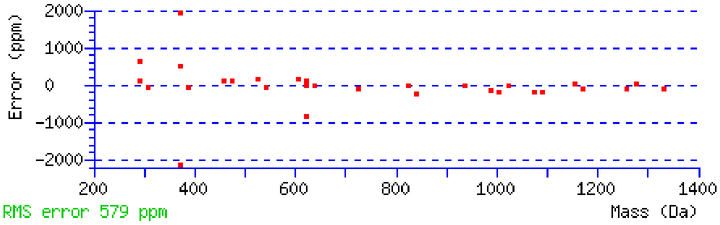

All matches to this query

Score	Mr(calc):	Delta	Sequence
76.4	1477.7089	0.0073	GYSFSLTTFSPSGK
12.0	1475.7061	2.0101	VKFASLEFSPGSK
7.1	1477.7040	0.0122	VLLSFGKAFECK
6.1	1477.7040	0.0123	GYLQMSPLFKAK
5.7	1477.7290	-0.0127	RSSLPNGEGLQLK
5.7	1477.7290	-0.0127	RSSLPNGEGLQLK
5.4	1477.7112	0.0050	MLLAPPSTPSRGR
5.4	1477.7112	0.0050	MLLAPPSTPSRGR
4.6	1477.7040	0.0122	YGAICGFGLSLIK
3.8	1476.7143	1.0019	MAAPSDGFKPRER

Spectrum No: 45; Query: 23954; Rank: 1

Peptide View

MS/MS Fragmentation of **HIGLVYSGMGPDYR**
Found in **IPI00219622**, Tax_Id=9606 Gene_Symbol=PSMA2 Proteasome subunit alpha type-2

Match to Query 23954: 1563.760388 from(782.887470,2+)
Title: 091224LimSK_Exosome3_06.3980.3980.2.dta
Data file C:\Temp\Mascoti\LSK_2D\091224LimSK_Exosome3_01_2D.mgf

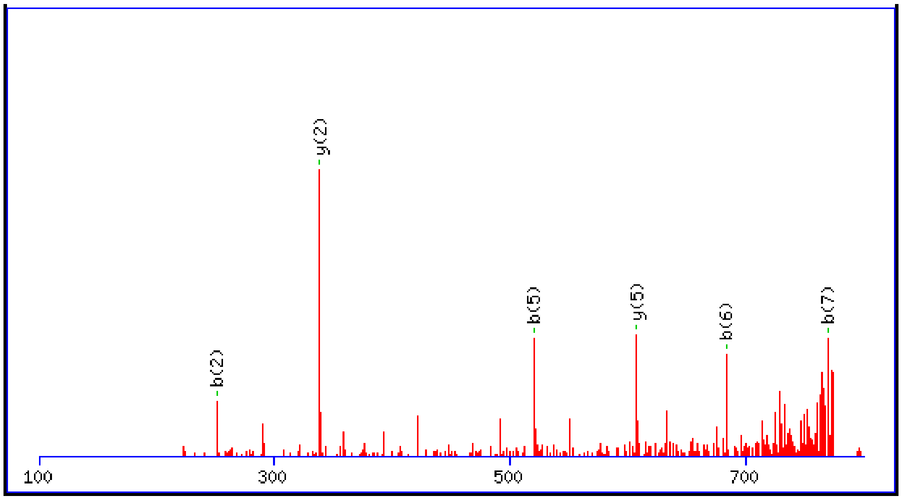

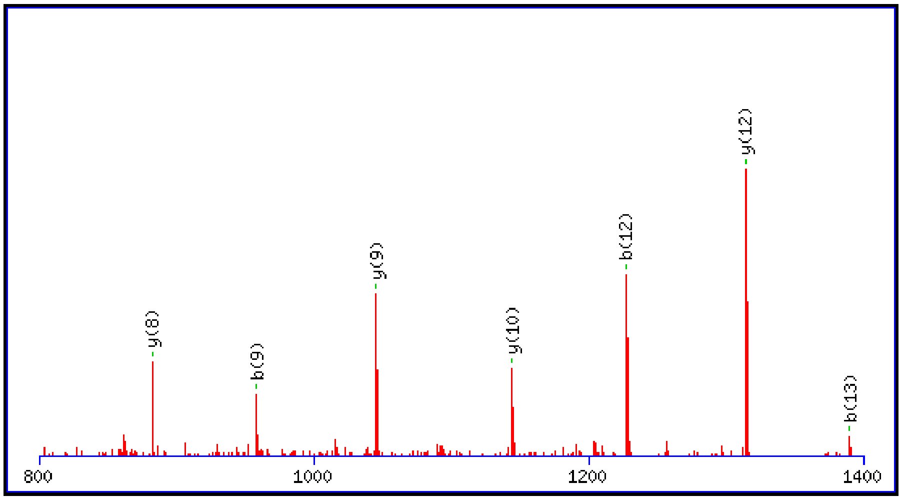

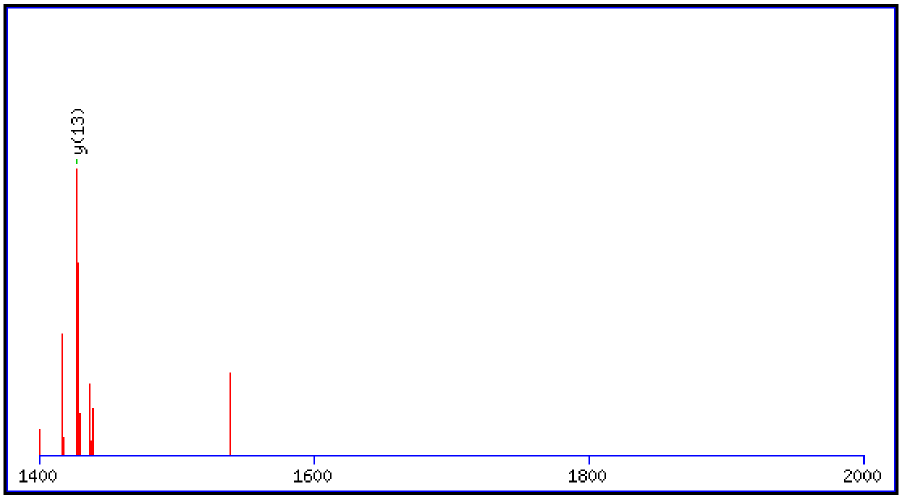

Monoisotopic mass of neutral peptide Mr(calc): 1563.7504
Fixed modifications: Carbamidomethyl (C)
Ions Score: 72 **Expect:** 1.3e-005
Matches (Bold Red): 14/114 fragment ions using 19 most intense peaks

#	b	b^{++}	b^0	b^{0++}	Seq.	y	y^{++}	y*	y^{*++}	y^0	y^{0++}	#
1	138.0662	69.5367			H							14
2	**251.1503**	126.0788			I	**1427.6988**	714.3530	1410.6722	705.8397	1409.6882	705.3477	13
3	308.1717	154.5895			G	**1314.6147**	657.8110	1297.5882	649.2977	1296.6041	648.8057	12
4	421.2558	211.1315			L	1257.5932	629.3003	1240.5667	620.7870	1239.5827	620.2950	11
5	**520.3242**	260.6657			V	**1144.5092**	572.7582	1127.4826	564.2449	1126.4986	563.7529	10
6	**683.3875**	342.1974			Y	**1045.4408**	523.2240	1028.4142	514.7107	1027.4302	514.2187	9

**7**	**770.4196**	385.7134	752.4090	376.7081	**S**	**882.3774**	441.6924	865.3509	433.1791	864.3669	432.6871	**8**
**8**	827.4410	414.2241	809.4305	405.2189	**G**	795.3454	398.1763	778.3189	389.6631	777.3348	389.1711	**7**
**9**	**958.4815**	479.7444	940.4709	470.7391	**M**	738.3239	369.6656	721.2974	361.1523	720.3134	360.6603	**6**
**10**	1015.5030	508.2551	997.4924	499.2498	**G**	**607.2835**	304.1454	590.2569	295.6321	589.2729	295.1401	**5**
**11**	1112.5557	556.7815	1094.5452	547.7762	**P**	550.2620	275.6346	533.2354	267.1214	532.2514	266.6293	**4**
**12**	**1227.5827**	614.2950	1209.5721	605.2897	**D**	453.2092	227.1083	436.1827	218.5950	435.1987	218.1030	**3**
**13**	**1390.6460**	695.8266	1372.6354	686.8214	**Y**	**338.1823**	169.5948	321.1557	161.0815			**2**
**14**					**R**	175.1190	88.0631	158.0924	79.5498			**1**

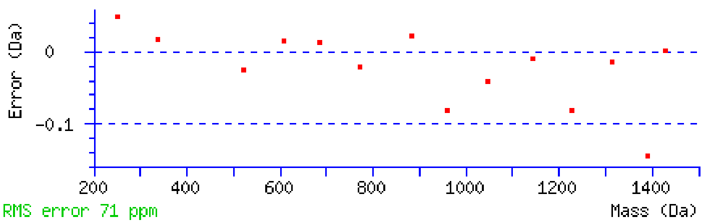

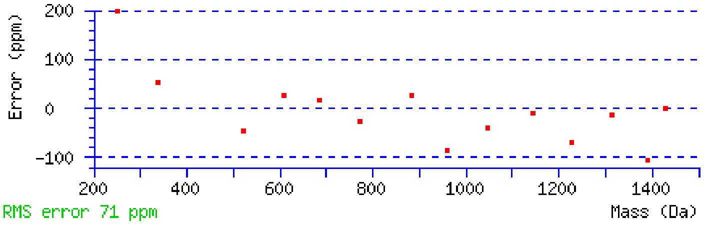

All matches to this query

Score	Mr(calc):	Delta	Sequence
71.6	1563.7504	0.0100	HIGLVYSGMGPDYR
9.4	1563.7715	-0.0111	EDFKQMSPGIIQR
4.3	1563.7631	-0.0027	RTITHSRLGNSSR
0.3	1563.7586	0.0018	VVEKAKYSLDSVF
0.3	1563.7586	0.0018	VVEKAKYSLDSVF
0.1	1561.7649	1.9955	VILTCTVIYCIK
0.1	1563.7517	0.0087	YIYMHIHSHAHR

Spectrum No: 46; Query: 59196; Rank: 1

Peptide View

MS/MS Fragmentation of **KLAQQYYLVYQEPIPTAQLVQR**
Found in **IPI00219622**, Tax_Id=9606 Gene_Symbol=PSMA2 Proteasome subunit alpha type-2

Match to Query 59196: 2648.452752 from(883.824860,3+)
Title: 091224LimSK_Exosome3_07.6329.6329.3.dta
Data file C:\Temp\Mascoti\LSK_2D\091224LimSK_Exosome3_01_2D.mgf

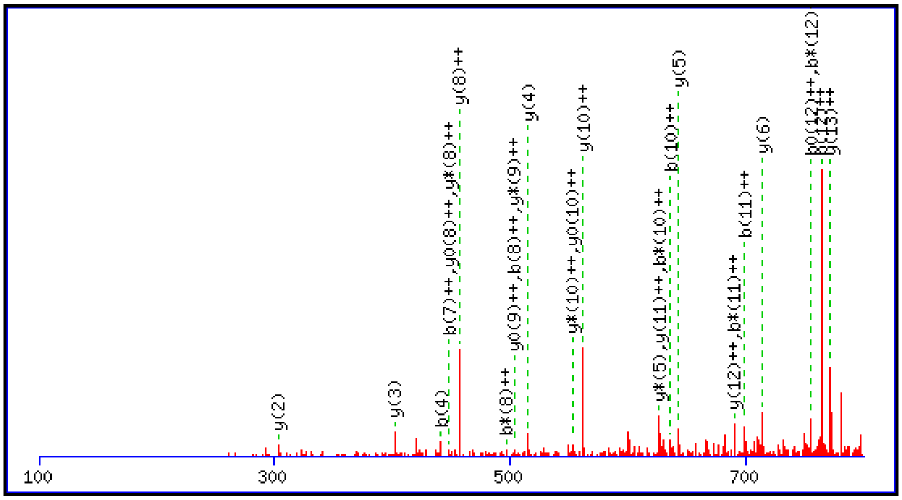

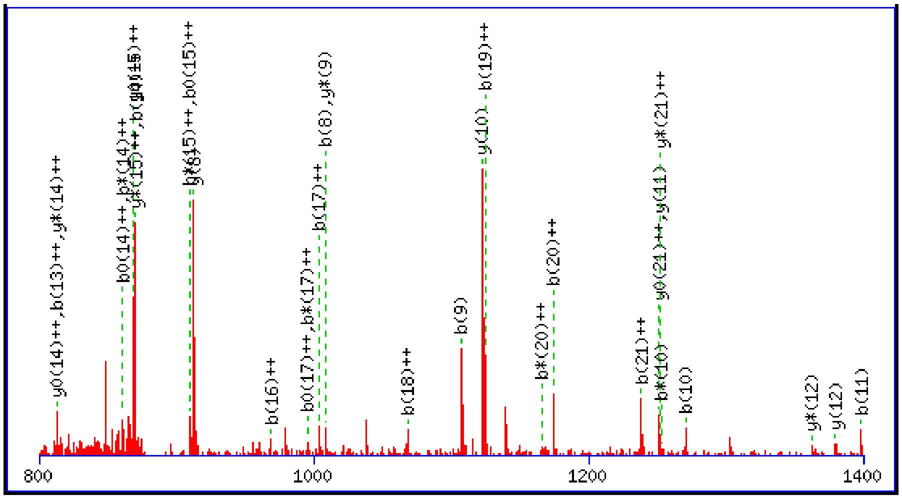

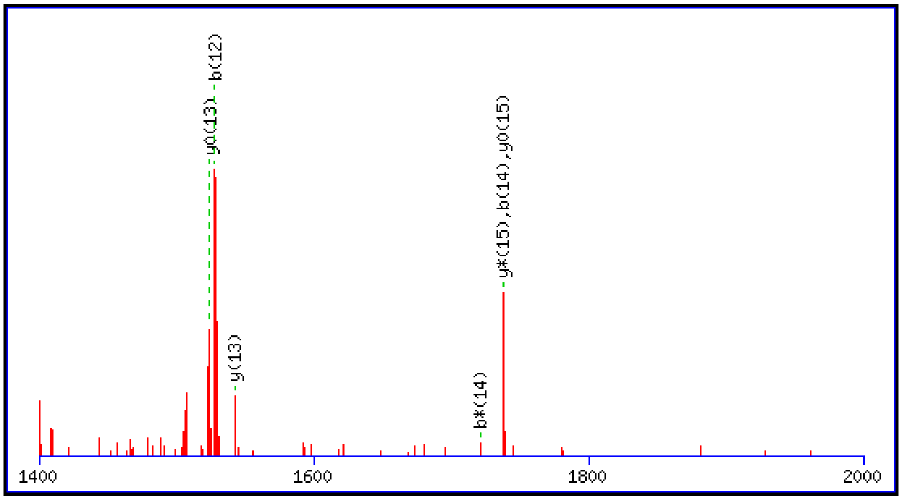

Monoisotopic mass of neutral peptide Mr(calc): 2648.4326
Fixed modifications: Carbamidomethyl (C)
Ions Score: 44 **Expect:** 0.0018
Matches (Bold Red): 67/218 fragment ions using 128 most intense peaks

#	b	b++	b*	b*++	b0	b0++	Seq.	y	y++	y*	y*++	y0	y0++	#
1	129.1022	65.0548	112.0757	56.5415			K							22
2	242.1863	121.5968	225.1598	113.0835			L	2521.3449	1261.1761	2504.3184	**1252.6628**	2503.3344	**1252.1708**	21
3	313.2234	157.1153	296.1969	148.6021			A	2408.2609	1204.6341	2391.2343	1196.1208	2390.2503	1195.6288	20
4	**441.2820**	221.1446	424.2554	212.6314			Q	2337.2238	1169.1155	2320.1972	1160.6022	2319.2132	1160.1102	19
5	569.3406	285.1739	552.3140	276.6607			Q	2209.1652	1105.0862	2192.1386	1096.5730	2191.1546	1096.0809	18
6	732.4039	366.7056	715.3774	358.1923			Y	2081.1066	1041.0569	2064.0801	1032.5437	2063.0960	1032.0517	17
7	895.4672	**448.2373**	878.4407	439.7240			Y	1918.0433	959.5253	1901.0167	951.0120	1900.0327	950.5200	16
8	**1008.5513**	**504.7793**	991.5247	**496.2660**			L	1754.9799	877.9936	**1737.9534**	**869.4803**	**1736.9694**	**868.9883**	15
9	**1107.6197**	554.3135	1090.5932	545.8002			V	1641.8959	821.4516	1624.8693	**812.9383**	1623.8853	**812.4463**	14
10	**1270.6830**	**635.8452**	**1253.6565**	**627.3319**			Y	**1542.8275**	**771.9174**	1525.8009	763.4041	**1524.8169**	762.9121	13
11	**1398.7416**	**699.8744**	1381.7151	**691.3612**			Q	**1379.7641**	**690.3857**	**1362.7376**	681.8724	1361.7536	681.3804	12
12	**1527.7842**	**764.3957**	1510.7577	**755.8825**	1509.7736	**755.3905**	E	**1251.7056**	**626.3564**	1234.6790	617.8431	1233.6950	617.3511	11
13	1624.8370	**812.9221**	1607.8104	804.4089	1606.8264	803.9168	P	**1122.6630**	**561.8351**	1105.6364	**553.3218**	1104.6524	**552.8298**	10
14	**1737.9210**	**869.4642**	**1720.8945**	**860.9509**	1719.9105	**860.4589**	I	1025.6102	513.3087	**1008.5837**	**504.7955**	1007.5996	**504.3035**	9
15	1834.9738	917.9905	1817.9473	**909.4773**	1816.9632	**908.9853**	P	**912.5261**	**456.7667**	895.4996	**448.2534**	894.5156	**447.7614**	8
16	1936.0215	**968.5144**	1918.9949	960.0011	1918.0109	959.5091	T	815.4734	408.2403	798.4468	399.7271	797.4628	399.2350	7
17	2007.0586	**1004.0329**	1990.0320	**995.5197**	1989.0480	**995.0277**	A	**714.4257**	357.7165	697.3992	349.2032			6
18	2135.1172	**1068.0622**	2118.0906	1059.5489	2117.1066	1059.0569	Q	**643.3886**	322.1979	**626.3620**	313.6847			5
19	2248.2012	**1124.6043**	2231.1747	1116.0910	2230.1907	1115.5990	L	**515.3300**	258.1686	498.3035	249.6554			4

**20**	2347.2696	**1174.1385**	2330.2431	**1165.6252**	2329.2591	1165.1332	**V**	**402.2459**	201.6266	385.2194	193.1133			**3**
**21**	2475.3282	**1238.1678**	2458.3017	1229.6545	2457.3177	1229.1625	**Q**	**303.1775**	152.0924	286.1510	143.5791			**2**
**22**							**R**	175.1190	88.0631	158.0924	79.5498			**1**

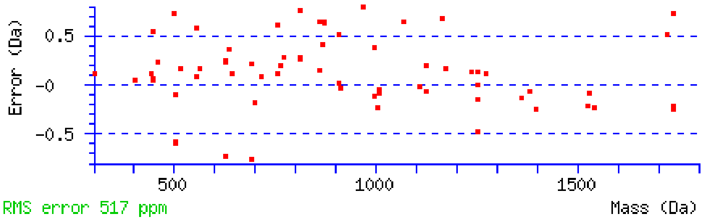

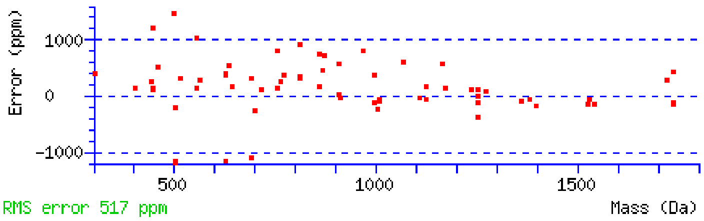

All matches to this query

Score	Mr(calc):	Delta	Sequence
43.7	2648.4326	0.0202	KLAQQYYLVYQEPIPTAQLVQR
7.6	2647.4495	1.0033	ISYLMIALTVVGCIFHWLLRAR
4.9	2647.4293	1.0235	GSSDVQELQRIGQLLIHLQEKEK
2.5	2648.4320	0.0208	VTIAELGVQLVAGMSLSLQPHRADK
1.3	2648.4277	0.0250	AHLTGWLMTLKKTFVLAPSSVLR
1.1	2648.4286	0.0242	NALTGILTQFEQIVAVFNASTRQK
0.3	2646.4277	2.0250	SSVFLGNSIMSQKFFSLILLMLR

Spectrum No: 47; Query: 56429; Rank: 1

Peptide View

MS/MS Fragmentation of **LAQQYYLVYQEPIPTAQLVQR**
Found in **IPI00219622**, Tax_Id=9606 Gene_Symbol=PSMA2 Proteasome subunit alpha type-2

Match to Query 56429: 2520.356868 from(1261.185710,2+)
Title: 091224LimSK_Exosome3_06.6454.6454.2.dta
Data file C:\Temp\Mascoti\LSK_2D\091224LimSK_Exosome3_01_2D.mgf

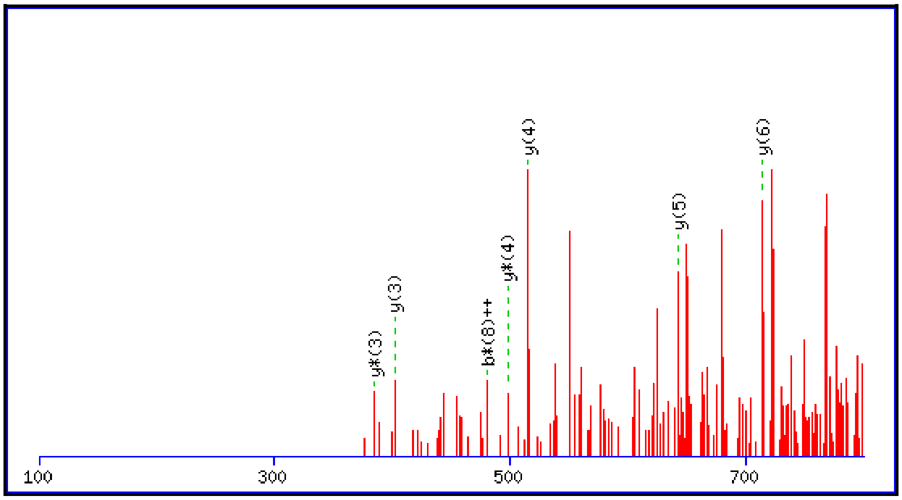

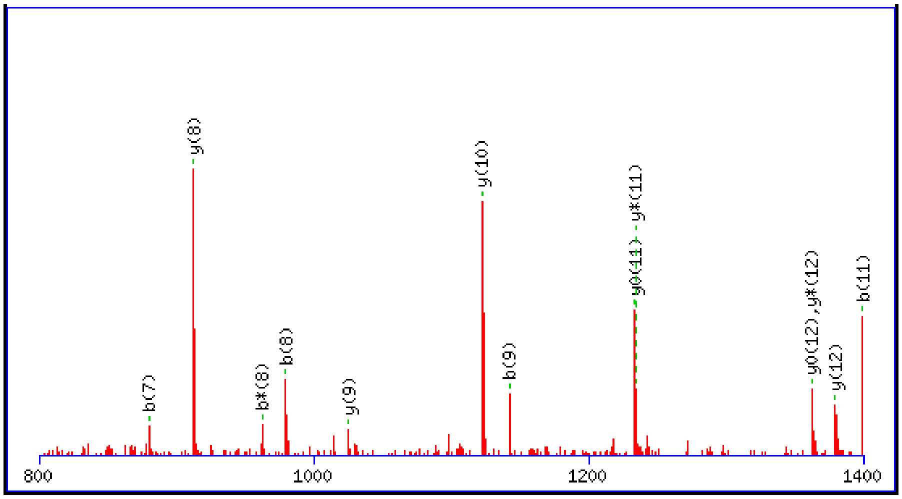

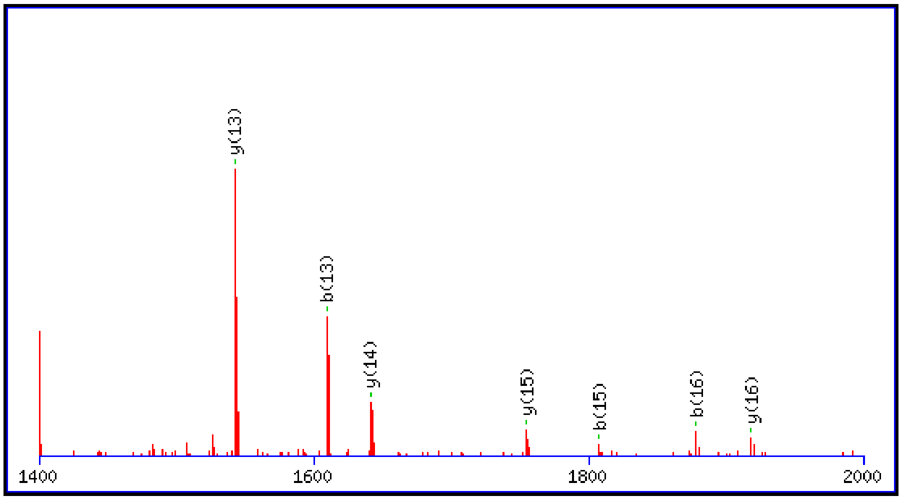

Monoisotopic mass of neutral peptide Mr(calc): 2520.3376
Fixed modifications: Carbamidomethyl (C)
Ions Score: 65 **Expect:** 2.3e-005
Matches (Bold Red): 27/204 fragment ions using 59 most intense peaks

#	b	b^{++}	b*	b*$^{++}$	b^{0}	b^{0++}	Seq.	y	y^{++}	y*	y*$^{++}$	y^{0}	y^{0++}	#
1	114.0913	57.5493					L							21
2	185.1285	93.0679					A	2408.2609	1204.6341	2391.2343	1196.1208	2390.2503	1195.6288	20
3	313.1870	157.0972	296.1605	148.5839			Q	2337.2238	1169.1155	2320.1972	1160.6022	2319.2132	1160.1102	19
4	441.2456	221.1264	424.2191	212.6132			Q	2209.1652	1105.0862	2192.1386	1096.5730	2191.1546	1096.0809	18
5	604.3089	302.6581	587.2824	294.1448			Y	2081.1066	1041.0569	2064.0801	1032.5437	2063.0960	1032.0517	17
6	767.3723	384.1898	750.3457	375.6765			Y	**1918.0433**	959.5253	1901.0167	951.0120	1900.0327	950.5200	16
7	**880.4563**	440.7318	863.4298	432.2185			L	**1754.9799**	877.9936	1737.9534	869.4803	1736.9694	868.9883	15
8	**979.5247**	490.2660	**962.4982**	**481.7527**			V	**1641.8959**	821.4516	1624.8693	812.9383	1623.8853	812.4463	14
9	**1142.5881**	571.7977	1125.5615	563.2844			Y	**1542.8275**	771.9174	1525.8009	763.4041	1524.8169	762.9121	13
10	1270.6467	635.8270	1253.6201	627.3137			Q	**1379.7641**	690.3857	**1362.7376**	681.8724	**1361.7536**	681.3804	12
11	**1399.6892**	700.3483	1382.6627	691.8350	1381.6787	691.3430	E	1251.7056	626.3564	**1234.6790**	617.8431	**1233.6950**	617.3511	11
12	1496.7420	748.8746	1479.7155	740.3614	1478.7314	739.8694	P	**1122.6630**	561.8351	1105.6364	553.3218	1104.6524	552.8298	10
13	**1609.8261**	805.4167	1592.7995	796.9034	1591.8155	796.4114	I	**1025.6102**	513.3087	1008.5837	504.7955	1007.5996	504.3035	9
14	1706.8788	853.9431	1689.8523	845.4298	1688.8683	844.9378	P	**912.5261**	456.7667	895.4996	448.2534	894.5156	447.7614	8
15	**1807.9265**	904.4669	1790.9000	895.9536	1789.9160	895.4616	T	815.4734	408.2403	798.4468	399.7271	797.4628	399.2350	7
16	**1878.9636**	939.9855	1861.9371	931.4722	1860.9531	930.9802	A	**714.4257**	357.7165	697.3992	349.2032			6
17	2007.0222	1004.0147	1989.9957	995.5015	1989.0116	995.0095	Q	**643.3886**	322.1979	626.3620	313.6847			5
18	2120.1063	1060.5568	2103.0797	1052.0435	2102.0957	1051.5515	L	**515.3300**	258.1686	**498.3035**	249.6554			4
19	2219.1747	1110.0910	2202.1481	1101.5777	2201.1641	1101.0857	V	**402.2459**	201.6266	**385.2194**	193.1133			3

20	2347.2333	1174.1203	2330.2067	1165.6070	2329.2227	1165.1150	Q	303.1775	152.0924	286.1510	143.5791			2
21							R	175.1190	88.0631	158.0924	79.5498			1

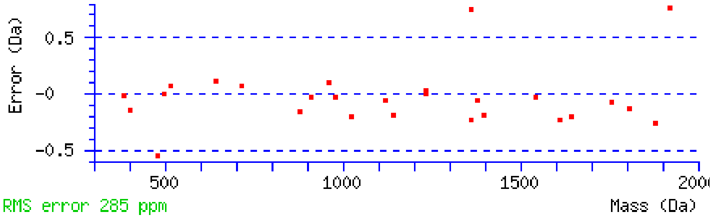

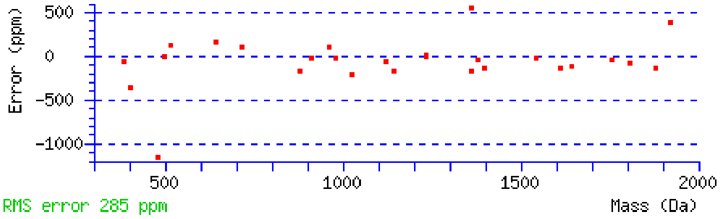

All matches to this query

Score	Mr(calc):	Delta	Sequence
65.1	2520.3376	0.0192	LAQQYYLVYQEPIPTAQLVQR
3.2	2518.3501	2.0068	WQLALAVGAPLLLGAGAIYLWSR
0.4	2519.3400	1.0168	DLAVVLETAPILTALDIFVDRR

Spectrum No: 48; Query: 20380; Rank: 1

Peptide View

MS/MS Fragmentation of **LTPTEVKDYLAAIA**
Found in **IPI00219622**, Tax_Id=9606 Gene_Symbol=PSMA2 Proteasome subunit alpha type-2

Match to Query 20380: 1503.826388 from(752.920470,2+)
Title: 091224LimSK_Exosome3_06.7667.7667.2.dta
Data file C:\Temp\Mascoti\LSK_2D\091224LimSK_Exosome3_01_2D.mgf

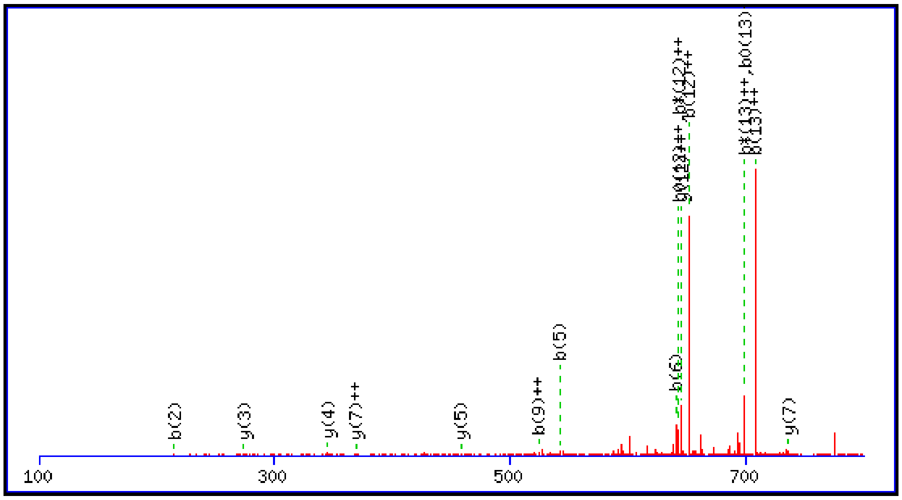

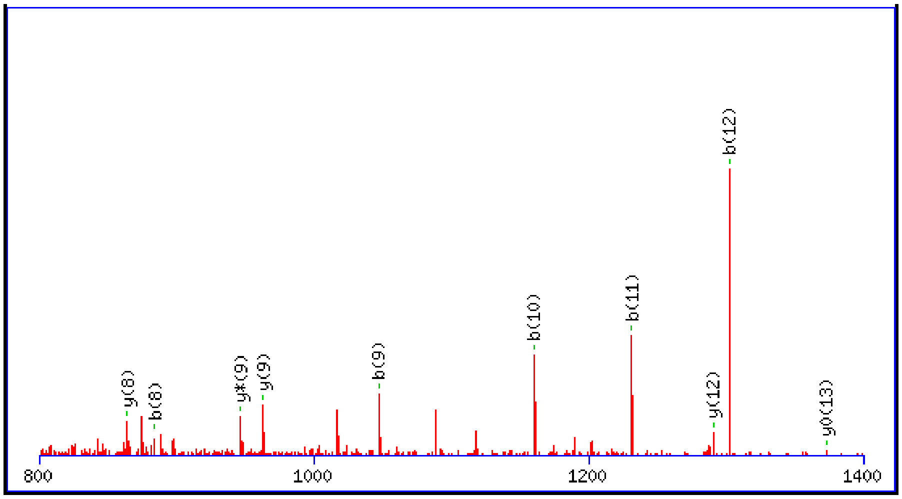

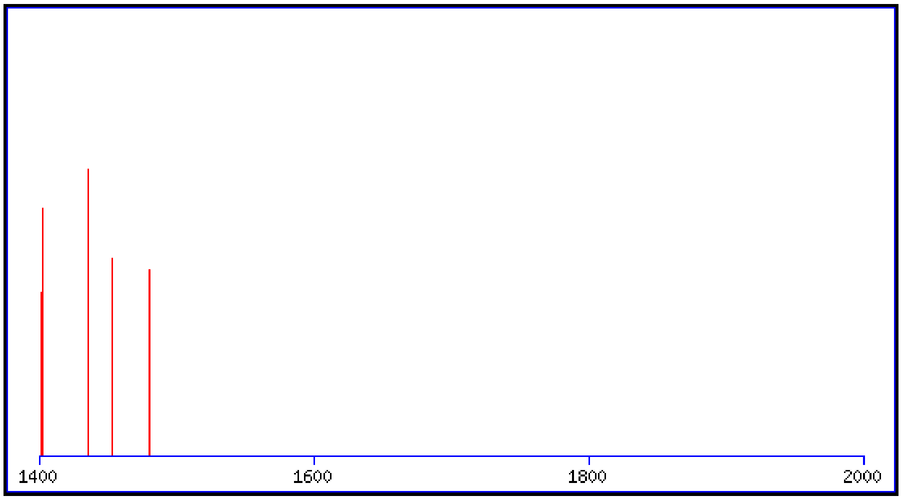

Monoisotopic mass of neutral peptide Mr(calc): 1503.8184
Fixed modifications: Carbamidomethyl (C)
Ions Score: 23 **Expect:** 0.45
Matches (Bold Red): 27/116 fragment ions using 81 most intense peaks

#	b	b^{++}	b*	b^{*++}	b^{0}	b^{0++}	Seq.	y	y^{++}	y*	y^{*++}	y^{0}	y^{0++}	#
1	114.0913	57.5493					L							14
2	**215.1390**	108.0731			197.1285	99.0679	T	1391.7417	696.3745	1374.7151	687.8612	**1373.7311**	687.3692	13
3	312.1918	156.5995			294.1812	147.5942	P	**1290.6940**	**645.8506**	1273.6674	637.3374	1272.6834	636.8454	12
4	413.2395	207.1234			395.2289	198.1181	T	1193.6412	597.3243	1176.6147	588.8110	1175.6307	588.3190	11
5	**542.2821**	271.6447			**524.2715**	262.6394	E	1092.5936	546.8004	1075.5670	538.2871	1074.5830	537.7951	10
6	**641.3505**	321.1789			623.3399	312.1736	V	**963.5510**	482.2791	**946.5244**	473.7658	945.5404	473.2738	9
7	769.4454	385.2264	752.4189	376.7131	751.4349	376.2211	K	**864.4825**	432.7449	847.4560	424.2316	846.4720	423.7396	8
8	**884.4724**	442.7398	867.4458	434.2266	866.4618	433.7345	D	**736.3876**	**368.6974**			718.3770	359.6921	7
9	**1047.5357**	**524.2715**	1030.5092	515.7582	1029.5251	515.2662	Y	621.3606	311.1840					6
10	**1160.6198**	580.8135	1143.5932	572.3002	1142.6092	571.8082	L	**458.2973**	229.6523					5
11	**1231.6569**	616.3321	1214.6303	607.8188	1213.6463	607.3268	A	**345.2132**	173.1103					4
12	**1302.6940**	**651.8506**	1285.6674	**643.3374**	1284.6834	**642.8454**	A	**274.1761**	137.5917					3
13	1415.7781	**708.3927**	1398.7515	**699.8794**	1397.7675	**699.3874**	I	203.1390	102.0731					2
14							A	90.0550	45.5311					1

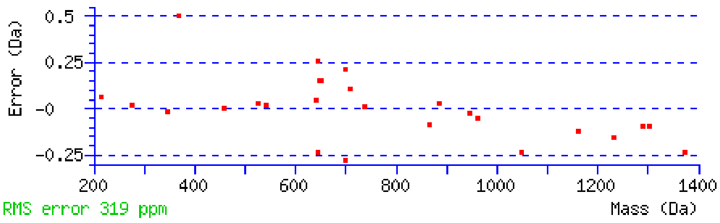

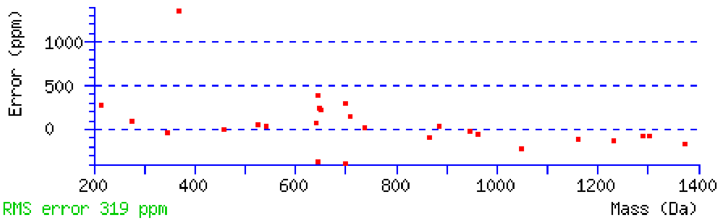

All matches to this query

Score	Mr(calc):	Delta	Sequence
23.3	1503.8184	0.0080	LTPTEVKDYLAAIA
2.3	1502.8239	1.0025	SNLREVAQMLKSK
1.9	1501.8286	1.9978	SNIREIEKLCLK
1.2	1501.8075	2.0189	ASLALCAFVAVPQR

Spectrum No: 49; Query: 40917; Rank: 1

Peptide View

MS/MS Fragmentation of **LVQIEYALAAVAGGAPSVGIK**
Found in **IPI00219622**, Tax_Id=9606 Gene_Symbol=PSMA2 Proteasome subunit alpha type-2

Match to Query 40917: 2026.158968 from(1014.086760,2+)
Title: 091224LimSK_Exosome3_06.8096.8096.2.dta
Data file C:\Temp\Mascoti\LSK_2D\091224LimSK_Exosome3_01_2D.mgf

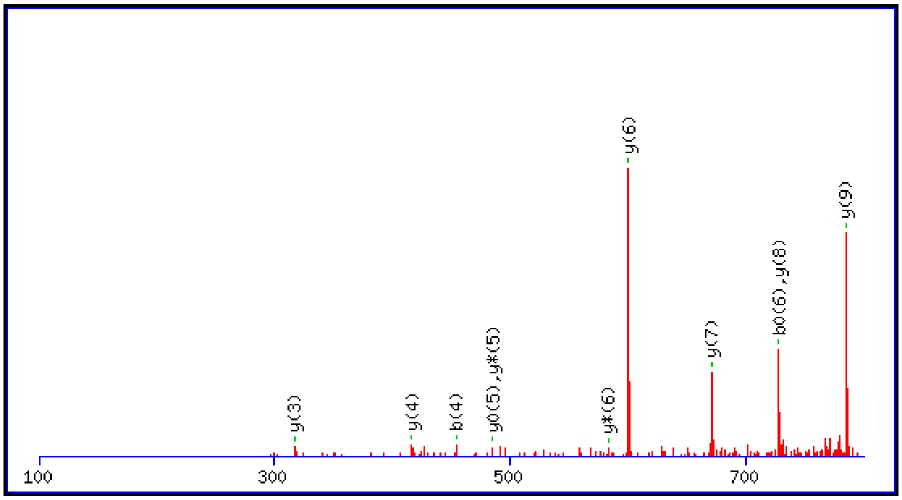

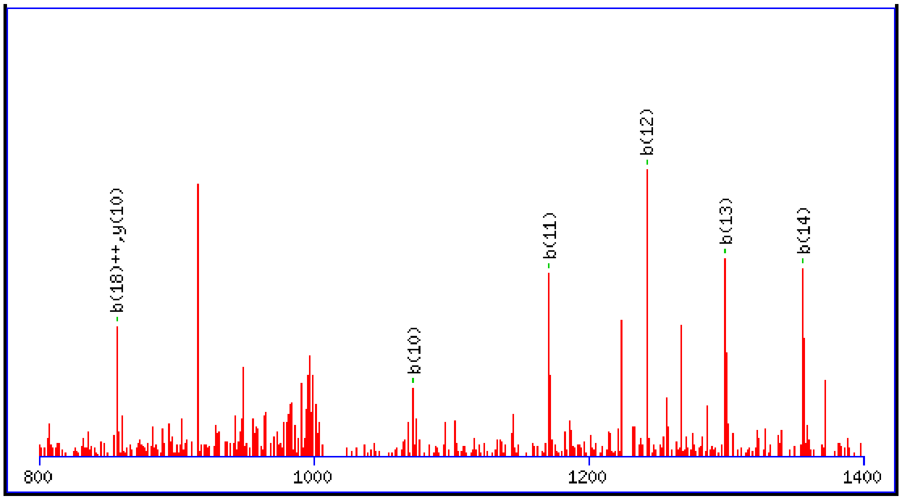

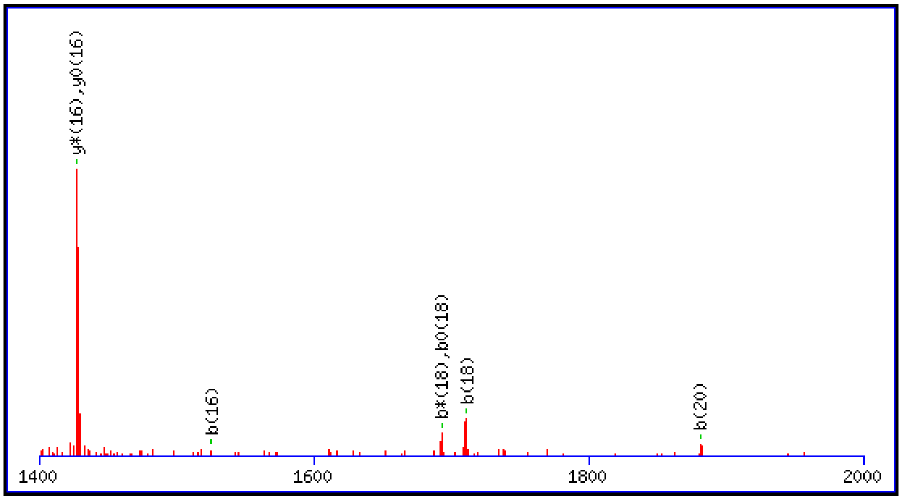

Monoisotopic mass of neutral peptide Mr(calc): 2026.1463
Fixed modifications: Carbamidomethyl (C)
Ions Score: 77 **Expect:** 5.8e-007
Matches (Bold Red): 27/220 fragment ions using 44 most intense peaks

#	b	b++	b*	b*++	b0	b0++	Seq.	y	y++	y*	y*++	y0	y0++	#
1	114.0913	57.5493					L							21
2	213.1598	107.0835					V	1914.0695	957.5384	1897.0429	949.0251	1896.0589	948.5331	20
3	341.2183	171.1128	324.1918	162.5995			Q	1815.0011	908.0042	1797.9745	899.4909	1796.9905	898.9989	19
4	**454.3024**	227.6548	437.2758	219.1416			I	1686.9425	843.9749	1669.9160	835.4616	1668.9319	834.9696	18
5	**583.3450**	292.1761	566.3184	283.6629	565.3344	283.1709	E	1573.8584	787.4329	1556.8319	778.9196	1555.8479	778.4276	17
6	746.4083	373.7078	729.3818	365.1945	**728.3978**	364.7025	Y	1444.8158	722.9116	**1427.7893**	714.3983	**1426.8053**	713.9063	16
7	817.4454	409.2264	800.4189	400.7131	799.4349	400.2211	A	1281.7525	641.3799	1264.7260	632.8666	1263.7419	632.3746	15
8	930.5295	465.7684	913.5029	457.2551	912.5189	456.7631	L	1210.7154	605.8613	1193.6889	597.3481	1192.7048	596.8561	14
9	1001.5666	501.2869	984.5401	492.7737	983.5560	492.2817	A	1097.6313	549.3193	1080.6048	540.8060	1079.6208	540.3140	13
10	**1072.6037**	536.8055	1055.5772	528.2922	1054.5932	527.8002	A	1026.5942	513.8007	1009.5677	505.2875	1008.5837	504.7955	12
11	**1171.6721**	586.3397	1154.6456	577.8264	1153.6616	577.3344	V	955.5571	478.2822	938.5306	469.7689	937.5465	469.2769	11
12	**1242.7093**	621.8583	1225.6827	613.3450	1224.6987	612.8530	A	**856.4887**	428.7480	839.4621	420.2347	838.4781	419.7427	10
13	**1299.7307**	650.3690	1282.7042	641.8557	1281.7202	641.3637	G	**785.4516**	393.2294	768.4250	384.7162	767.4410	384.2241	9
14	**1356.7522**	678.8797	1339.7256	670.3665	1338.7416	669.8744	G	**728.4301**	364.7187	711.4036	356.2054	710.4196	355.7134	8
15	**1427.7893**	714.3983	1410.7627	705.8850	1409.7787	705.3930	A	**671.4087**	336.2080	654.3821	327.6947	653.3981	327.2027	7
16	**1524.8421**	762.9247	1507.8155	754.4114	1506.8315	753.9194	P	**600.3715**	300.6894	**583.3450**	292.1761	582.3610	291.6841	6
17	1611.8741	806.4407	1594.8475	797.9274	1593.8635	797.4354	S	503.3188	252.1630	**486.2922**	243.6498	**485.3082**	243.1577	5
18	**1710.9425**	**855.9749**	**1693.9160**	847.4616	**1692.9319**	846.9696	V	**416.2867**	208.6470	399.2602	200.1337			4
19	1767.9640	884.4856	1750.9374	875.9723	1749.9534	875.4803	G	**317.2183**	159.1128	300.1918	150.5995			3

20	1881.0480	941.0277	1864.0215	932.5144	1863.0375	932.0224	I	260.1969	130.6021	243.1703	122.0888			2
21							K	147.1128	74.0600	130.0863	65.5468			1

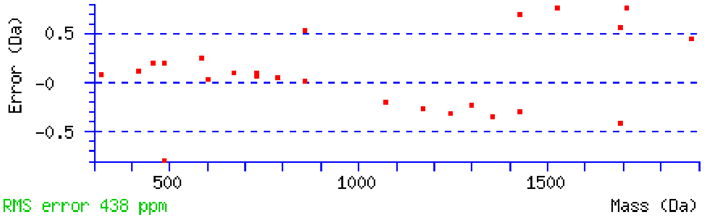

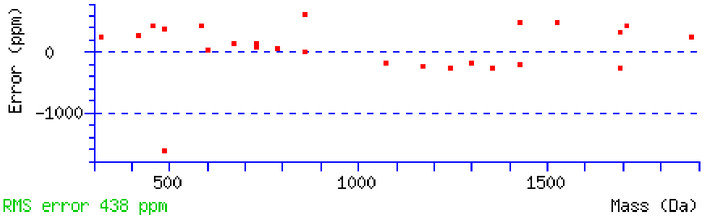

All matches to this query

Score	Mr(calc):	Delta	Sequence
77.2	2026.1463	0.0127	LVQIEYALAAVAGGAPSVGIK
11.5	2026.1649	-0.0059	ELIMIAGGNQSILLWIKK
9.5	2026.1575	0.0015	NLVPKKSSFANVAAATPAIK
4.8	2025.1443	1.0147	GSTAVGIRGLTADARVVINR
2.4	2026.1786	-0.0197	LQTSSVLVSGLRGLGVEIAK
0.3	2025.1735	0.9855	LSPVTYKLQPGNKPSRLK

Spectrum No: 50; Query: 28329; Rank: 1

Peptide View

MS/MS Fragmentation of **RLTPTEVKDYLAAIA**
Found in **IPI00219622**, Tax_Id=9606 Gene_Symbol=PSMA2 Proteasome subunit alpha type-2

Match to Query 28329: 1659.931528 from(830.973040,2+)
Title: 091224LimSK_Exosome3_06.6854.6854.2.dta
Data file C:\Temp\Mascoti\LSK_2D\091224LimSK_Exosome3_01_2D.mgf

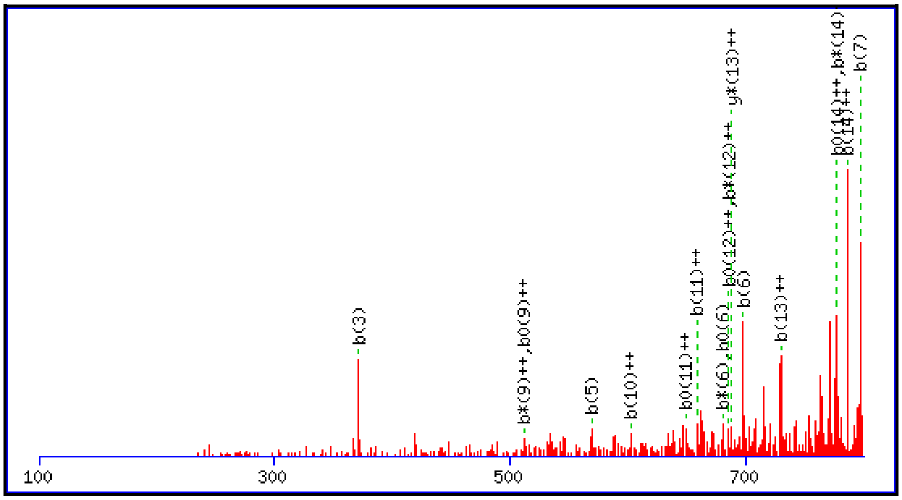

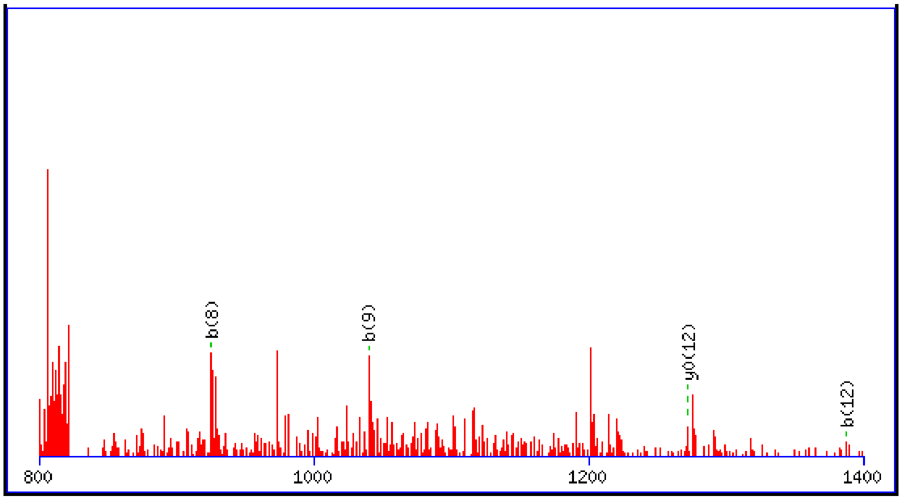

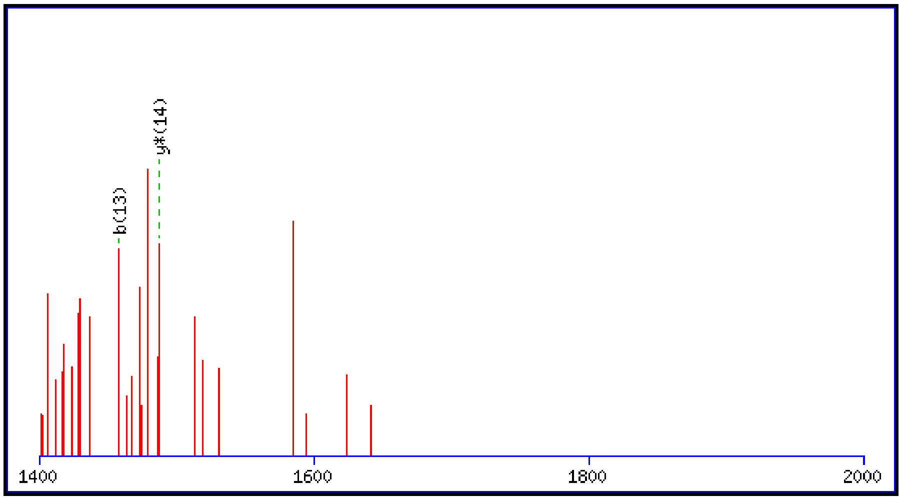

Monoisotopic mass of neutral peptide Mr(calc): 1659.9195
Fixed modifications: Carbamidomethyl (C)
Ions Score: 29 **Expect:** 0.079
Matches (Bold Red): 24/138 fragment ions using 66 most intense peaks

#	b	b^{++}	b*	b^{*++}	b^{0}	b^{0++}	Seq.	y	y^{++}	y*	y^{*++}	y^{0}	y^{0++}	#
1	157.1084	79.0578	140.0818	70.5446			R							15
2	270.1925	135.5999	253.1659	127.0866			L	1504.8257	752.9165	**1487.7992**	744.4032	1486.8152	743.9112	14
3	**371.2401**	186.1237	354.2136	177.6104	353.2296	177.1184	T	1391.7417	696.3745	1374.7151	**687.8612**	1373.7311	687.3692	13
4	468.2929	234.6501	451.2663	226.1368	450.2823	225.6448	P	1290.6940	645.8506	1273.6674	637.3374	**1272.6834**	636.8454	12
5	**569.3406**	285.1739	552.3140	276.6607	551.3300	276.1686	T	1193.6412	597.3243	1176.6147	588.8110	1175.6307	588.3190	11
6	**698.3832**	349.6952	**681.3566**	341.1819	**680.3726**	340.6899	E	1092.5936	546.8004	1075.5670	538.2871	1074.5830	537.7951	10
7	**797.4516**	399.2294	780.4250	390.7162	779.4410	390.2241	V	963.5510	482.2791	946.5244	473.7658	945.5404	473.2738	9
8	**925.5465**	463.2769	908.5200	454.7636	907.5360	454.2716	K	864.4825	432.7449	847.4560	424.2316	846.4720	423.7396	8
9	**1040.5735**	520.7904	1023.5469	**512.2771**	1022.5629	**511.7851**	D	736.3876	368.6974			718.3770	359.6921	7
10	1203.6368	**602.3220**	1186.6103	593.8088	1185.6263	593.3168	Y	621.3606	311.1840					6
11	1316.7209	**658.8641**	1299.6943	650.3508	1298.7103	**649.8588**	L	458.2973	229.6523					5
12	**1387.7580**	694.3826	1370.7314	**685.8694**	1369.7474	**685.3774**	A	345.2132	173.1103					4
13	**1458.7951**	**729.9012**	1441.7686	721.3879	1440.7845	720.8959	A	274.1761	137.5917					3
14	1571.8792	**786.4432**	1554.8526	**777.9299**	1553.8686	**777.4379**	I	203.1390	102.0731					2
15							A	90.0550	45.5311					1

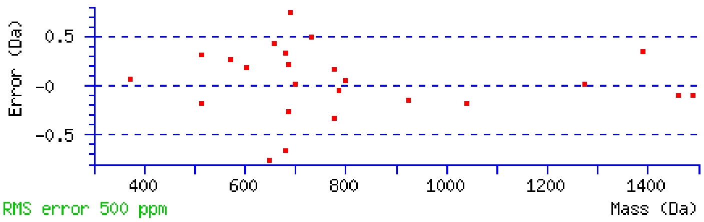

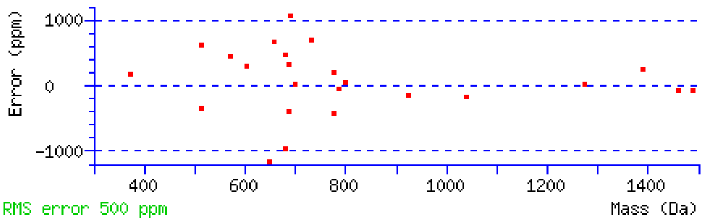

All matches to this query

Score	Mr(calc):	Delta	Sequence
28.8	1659.9195	0.0120	RLTPTEVKDYLAAIA
1.1	1659.9437	-0.0121	IKKISKPPVVQTSR
0.9	1659.9437	-0.0121	IKKISKPPVVQTSR
0.2	1658.9233	1.0083	SQPLLIPTTGRKLR

Spectrum No: 51; Query: 52630; Rank: 1

Peptide View

MS/MS Fragmentation of **RYNEDLELEDAIHTAILTLK**
Found in **IPI00219622**, Tax_Id=9606 Gene_Symbol=PSMA2 Proteasome subunit alpha type-2

Match to Query 52630: 2356.247982 from(786.423270,3+)
Title: 091224LimSK_Exosome3_06.8753.8753.3.dta
Data file C:\Temp\Mascoti\LSK_2D\091224LimSK_Exosome3_01_2D.mgf

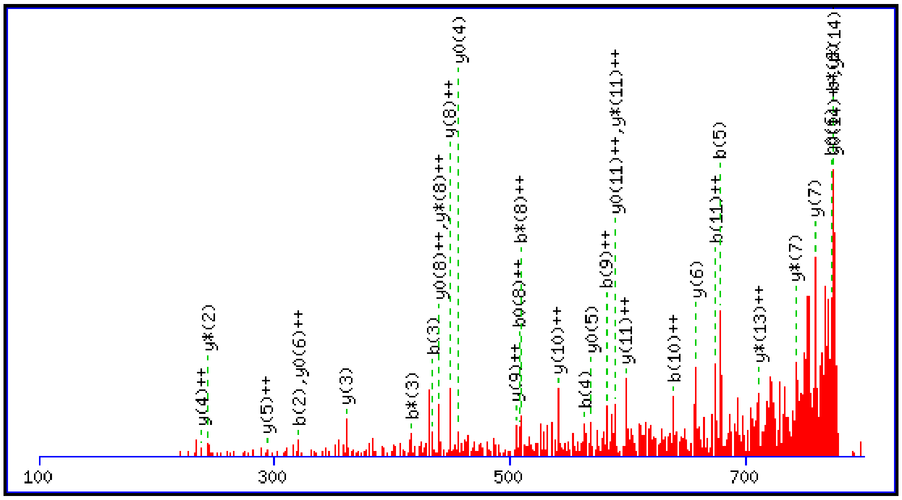

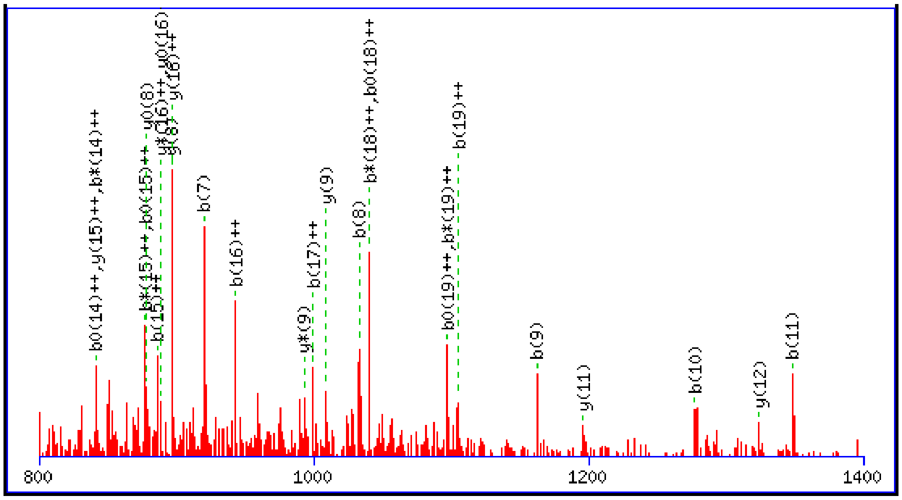

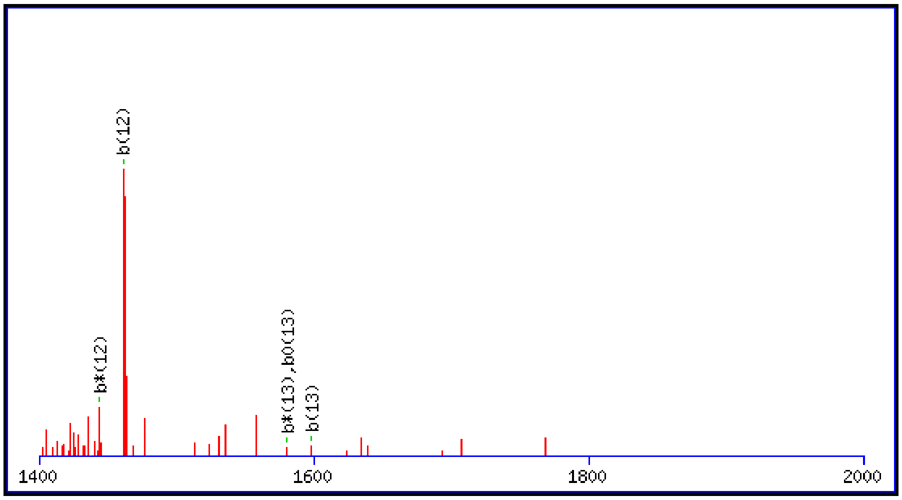

Monoisotopic mass of neutral peptide Mr(calc): 2356.2274
Fixed modifications: Carbamidomethyl (C)
Ions Score: 40 **Expect:** 0.011
Matches (Bold Red): 65/218 fragment ions using 136 most intense peaks

#	b	b^{++}	b*	b^{*++}	b^0	b^{0++}	Seq.	y	y^{++}	y*	y^{*++}	y^0	y^{0++}	#
1	157.1084	79.0578	140.0818	70.5446			R							20
2	**320.1717**	160.5895	303.1452	152.0762			Y	2201.1336	1101.0704	2184.1071	1092.5572	2183.1230	1092.0652	19
3	**434.2146**	217.6110	**417.1881**	209.0977			N	2038.0703	1019.5388	2021.0437	1011.0255	2020.0597	1010.5335	18
4	**563.2572**	282.1323	546.2307	273.6190	545.2467	273.1270	E	1924.0274	962.5173	1907.0008	954.0040	1906.0168	953.5120	17
5	**678.2842**	339.6457	661.2576	331.1325	660.2736	330.6404	D	1794.9848	**897.9960**	1777.9582	**889.4827**	1776.9742	**888.9907**	16
6	791.3682	396.1878	**774.3417**	387.6745	**773.3577**	387.1825	L	1679.9578	**840.4825**	1662.9313	831.9693	1661.9472	831.4773	15
7	**920.4108**	460.7091	903.3843	452.1958	902.4003	451.7038	E	1566.8738	783.9405	1549.8472	**775.4272**	1548.8632	**774.9352**	14
8	**1033.4949**	517.2511	1016.4684	**508.7378**	1015.4843	**508.2458**	L	1437.8312	719.4192	1420.8046	**710.9059**	1419.8206	710.4139	13
9	**1162.5375**	**581.7724**	1145.5109	573.2591	1144.5269	572.7671	E	**1324.7471**	662.8772	1307.7205	654.3639	1306.7365	653.8719	12
10	**1277.5644**	**639.2859**	1260.5379	630.7726	1259.5539	630.2806	D	**1195.7045**	**598.3559**	1178.6780	**589.8426**	1177.6939	**589.3506**	11
11	**1348.6016**	**674.8044**	1331.5750	666.2911	1330.5910	665.7991	A	1080.6776	**540.8424**	1063.6510	532.3291	1062.6670	531.8371	10
12	**1461.6856**	731.3464	**1444.6591**	722.8332	1443.6750	722.3412	I	**1009.6404**	**505.3239**	**992.6139**	496.8106	991.6299	496.3186	9
13	**1598.7445**	799.8759	**1581.7180**	791.3626	**1580.7340**	790.8706	H	**896.5564**	**448.7818**	879.5298	**440.2686**	**878.5458**	**439.7765**	8
14	1699.7922	850.3997	1682.7657	**841.8865**	1681.7816	**841.3945**	T	**759.4975**	380.2524	**742.4709**	371.7391	741.4869	371.2471	7
15	1770.8293	**885.9183**	1753.8028	**877.4050**	1752.8188	**876.9130**	A	**658.4498**	329.7285	641.4232	321.2153	640.4392	**320.7233**	6
16	1883.9134	**942.4603**	1866.8868	933.9471	1865.9028	933.4550	I	587.4127	**294.2100**	570.3861	285.6967	**569.4021**	285.2047	5
17	1996.9974	**999.0024**	1979.9709	990.4891	1978.9869	989.9971	L	474.3286	**237.6679**	457.3021	229.1547	**456.3180**	228.6627	4
18	2098.0451	1049.5262	2081.0186	**1041.0129**	2080.0346	**1040.5209**	T	**361.2445**	181.1259	344.2180	172.6126	343.2340	172.1206	3
19	2211.1292	**1106.0682**	2194.1026	**1097.5550**	2193.1186	**1097.0630**	L	260.1969	130.6021	**243.1703**	122.0888			2

20							K	147.1128	74.0600	130.0863	65.5468			1

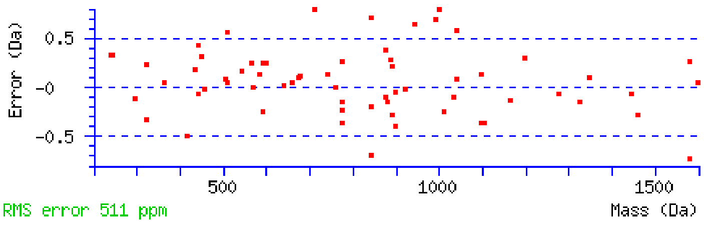

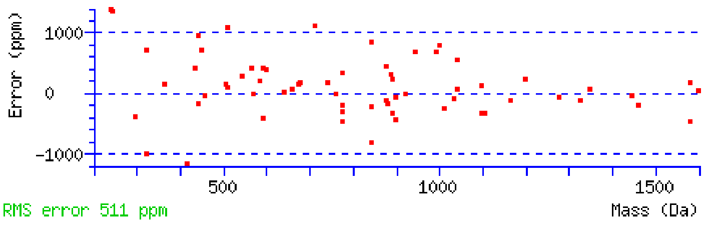

All matches to this query

Score	Mr(calc):	Delta	Sequence
40.1	2356.2274	0.0206	RYNEDLELEDAIHTAILTLK
5.3	2356.2589	-0.0109	MLLPLPPQSSKPVPKKSQSSK
4.6	2354.2376	2.0104	TVARGPVAPSKVATTSVITIVK
1.6	2354.2627	1.9852	IQNTLQKAKIPLVTNEECQK
0.5	2355.2506	0.9974	QQLPGQSRLSKQSGSLGLSQEK

Spectrum No: 52; Query: 5850; Rank: 1

Peptide View

MS/MS Fragmentation of **SVHKVEPITK**
Found in **IPI00219622**, Tax_Id=9606 Gene_Symbol=PSMA2 Proteasome subunit alpha type-2

Match to Query 5850: 1136.658762 from(379.893530,3+)
Title: 091224LimSK_Exosome3_06.60.60.3.dta
Data file C:\Temp\Mascoti\LSK_2D\091224LimSK_Exosome3_01_2D.mgf

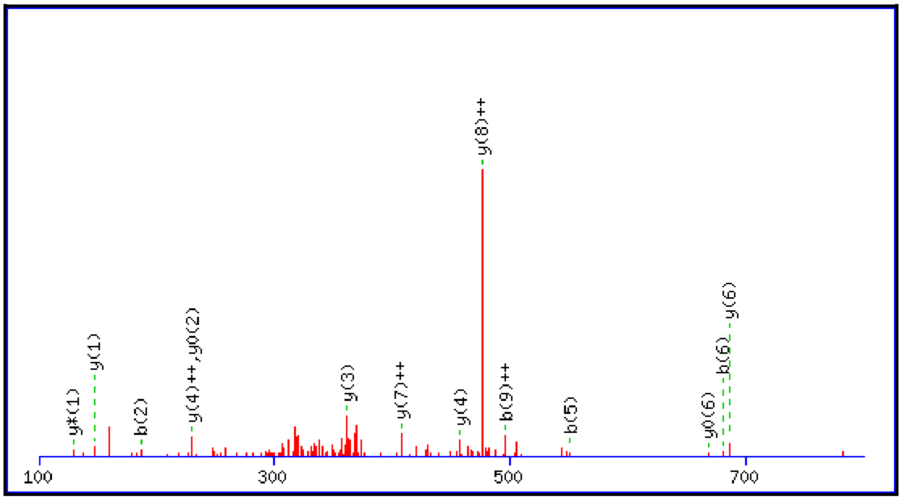

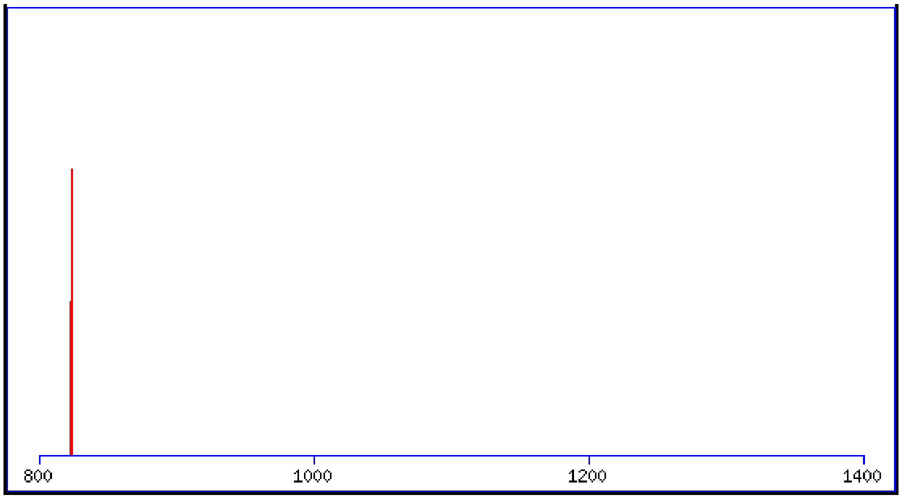

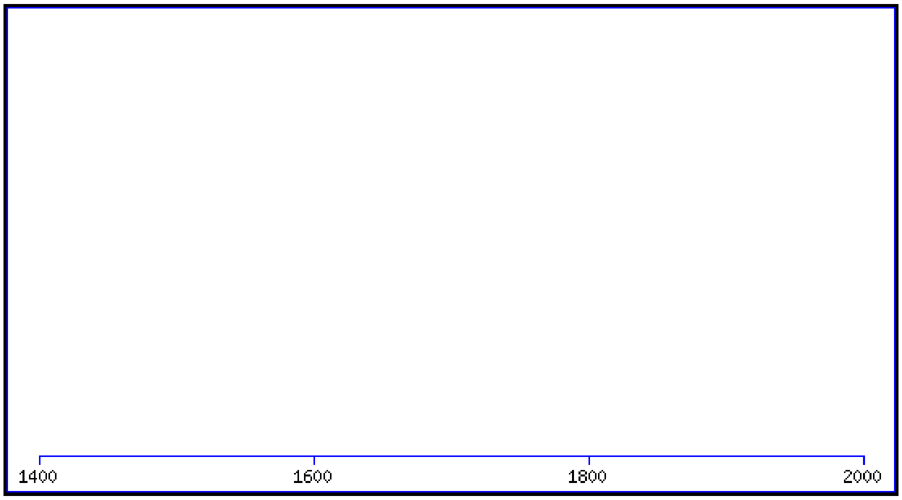

Monoisotopic mass of neutral peptide Mr(calc): 1136.6554
Fixed modifications: Carbamidomethyl (C)
Ions Score: 20 **Expect:** 0.38
Matches (Bold Red): 14/100 fragment ions using 26 most intense peaks

#	b	b++	b*	b*++	b0	b0++	Seq.	y	y++	y*	y*++	y0	y0++	#
1	88.0393	44.5233			70.0287	35.5180	S							10
2	**187.1077**	94.0575			169.0972	85.0522	V	1050.6306	525.8189	1033.6041	517.3057	1032.6200	516.8137	9
3	324.1666	162.5870			306.1561	153.5817	H	951.5622	**476.2847**	934.5356	467.7715	933.5516	467.2795	8
4	452.2616	226.6344	435.2350	218.1212	434.2510	217.6292	K	814.5033	**407.7553**	797.4767	399.2420	796.4927	398.7500	7
5	**551.3300**	276.1686	534.3035	267.6554	533.3194	267.1634	V	**686.4083**	343.7078	669.3818	335.1945	**668.3978**	334.7025	6
6	**680.3726**	340.6899	663.3461	332.1767	662.3620	331.6847	E	587.3399	294.1736	570.3134	285.6603	569.3293	285.1683	5
7	777.4254	389.2163	760.3988	380.7030	759.4148	380.2110	P	**458.2973**	**229.6523**	441.2708	221.1390	440.2867	220.6470	4
8	890.5094	445.7584	873.4829	437.2451	872.4989	436.7531	I	**361.2445**	181.1259	344.2180	172.6126	343.2340	172.1206	3
9	991.5571	**496.2822**	974.5306	487.7689	973.5465	487.2769	T	248.1605	124.5839	231.1339	116.0706	**230.1499**	115.5786	2
10							K	**147.1128**	74.0600	**130.0863**	65.5468			1

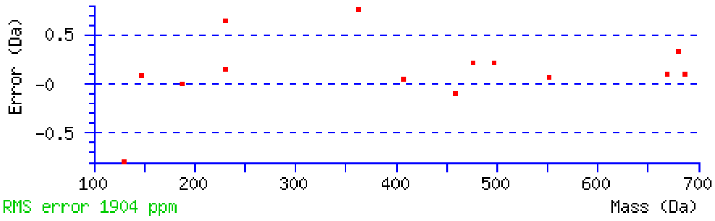

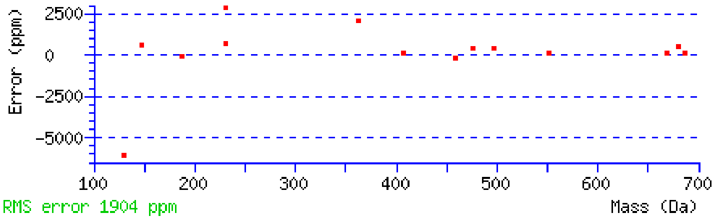

All matches to this query

Score	Mr(calc):	Delta	Sequence
20.4	1136.6554	0.0034	SVHKVEPITK
12.5	1135.6574	1.0014	EGARAHLKVR
5.0	1134.6471	2.0117	AIPMYKIATK
4.7	1134.6413	2.0174	SVVALKTPIK
2.9	1136.6627	-0.0040	KKFTMIEIK
2.6	1135.6574	1.0014	SVLIQHQRR
2.3	1136.6666	-0.0078	VHREIVSGLK
1.4	1136.6666	-0.0078	GKAHPGTKTIK
1.4	1136.6554	0.0034	LALEVTVHQK
1.3	1136.6526	0.0061	XARKLAGATGR

Spectrum No: 53; Query: 46805; Rank: 1

Peptide View

MS/MS Fragmentation of **YNEDLELEDAIHTAILTLK**
Found in **IPI00219622**, Tax_Id=9606 Gene_Symbol=PSMA2 Proteasome subunit alpha type-2

Match to Query 46805: 2200.145168 from(1101.079860,2+)
Title: 091224LimSK_Exosome3_06.8735.8735.2.dta
Data file C:\Temp\Mascoti\LSK_2D\091224LimSK_Exosome3_01_2D.mgf

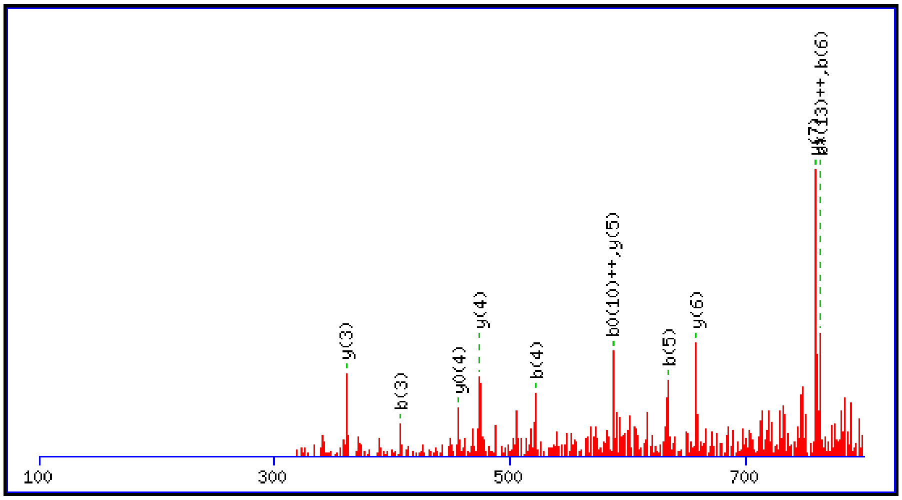

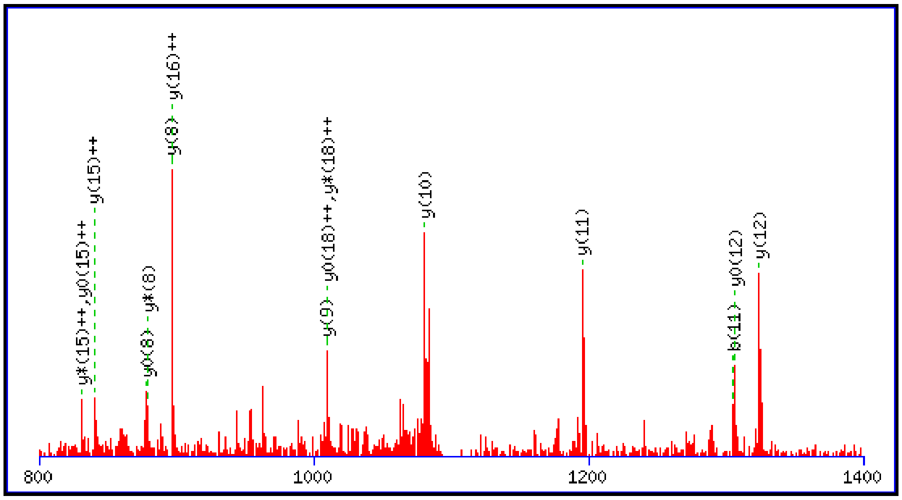

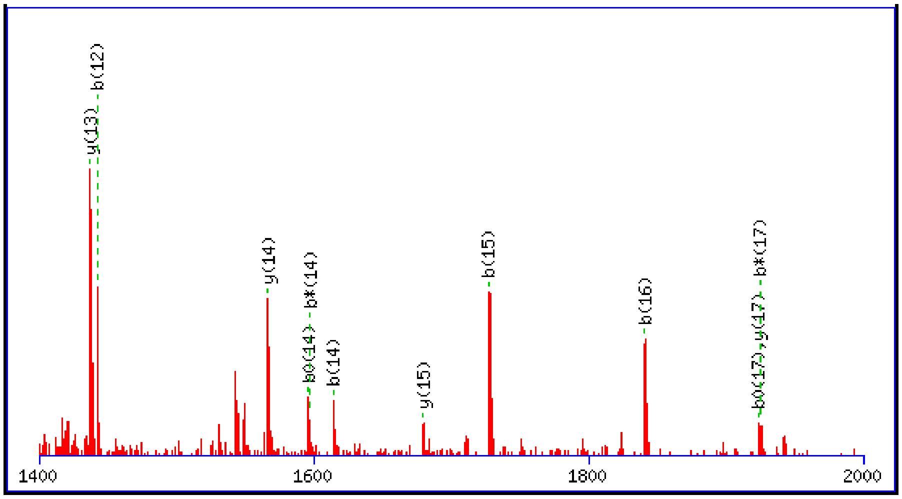

Monoisotopic mass of neutral peptide Mr(calc): 2200.1263
Fixed modifications: Carbamidomethyl (C)
Ions Score: 126 **Expect:** 3.7e-011
Matches (Bold Red): 39/206 fragment ions using 45 most intense peaks

#	b	b^{++}	b*	b^{*++}	b^0	b^{0++}	Seq.	y	y^{++}	y*	y^{*++}	y^0	y^{0++}	#
1	164.0706	82.5389					Y							19
2	278.1135	139.5604	261.0870	131.0471			N	2038.0703	1019.5388	2021.0437	**1011.0255**	2020.0597	**1010.5335**	18
3	**407.1561**	204.0817	390.1296	195.5684	389.1456	195.0764	E	**1924.0274**	962.5173	1907.0008	954.0040	1906.0168	953.5120	17
4	**522.1831**	261.5952	505.1565	253.0819	504.1725	252.5899	D	1794.9848	**897.9960**	1777.9582	889.4827	1776.9742	888.9907	16
5	**635.2671**	318.1372	618.2406	309.6239	617.2566	309.1319	L	**1679.9578**	**840.4825**	1662.9313	**831.9693**	1661.9472	**831.4773**	15
6	**764.3097**	382.6585	747.2832	374.1452	746.2992	373.6532	E	**1566.8738**	783.9405	1549.8472	775.4272	1548.8632	774.9352	14
7	877.3938	439.2005	860.3672	430.6873	859.3832	430.1953	L	**1437.8312**	719.4192	1420.8046	710.9059	1419.8206	710.4139	13
8	1006.4364	503.7218	989.4098	495.2086	988.4258	494.7165	E	**1324.7471**	662.8772	1307.7205	654.3639	**1306.7365**	653.8719	12
9	1121.4633	561.2353	1104.4368	552.7220	1103.4528	552.2300	D	**1195.7045**	598.3559	1178.6780	589.8426	1177.6939	589.3506	11
10	1192.5004	596.7539	1175.4739	588.2406	1174.4899	**587.7486**	A	**1080.6776**	540.8424	1063.6510	532.3291	1062.6670	531.8371	10
11	**1305.5845**	653.2959	1288.5580	644.7826	1287.5739	644.2906	I	**1009.6404**	505.3239	992.6139	496.8106	991.6299	496.3186	9
12	**1442.6434**	721.8253	1425.6169	713.3121	1424.6329	712.8201	H	**896.5564**	448.7818	**879.5298**	440.2686	**878.5458**	439.7765	8
13	1543.6911	772.3492	1526.6645	**763.8359**	1525.6805	763.3439	T	**759.4975**	380.2524	742.4709	371.7391	741.4869	371.2471	7
14	**1614.7282**	807.8677	**1597.7017**	799.3545	**1596.7176**	798.8625	A	**658.4498**	329.7285	641.4232	321.2153	640.4392	320.7233	6
15	**1727.8123**	864.4098	1710.7857	855.8965	1709.8017	855.4045	I	**587.4127**	294.2100	570.3861	285.6967	569.4021	285.2047	5
16	**1840.8963**	920.9518	1823.8698	912.4385	1822.8858	911.9465	L	**474.3286**	237.6679	457.3021	229.1547	**456.3180**	228.6627	4
17	1941.9440	971.4756	**1924.9175**	962.9624	**1923.9334**	962.4704	T	**361.2445**	181.1259	344.2180	172.6126	343.2340	172.1206	3
18	2055.0281	1028.0177	2038.0015	1019.5044	2037.0175	1019.0124	L	260.1969	130.6021	243.1703	122.0888			2
19							K	147.1128	74.0600	130.0863	65.5468			1

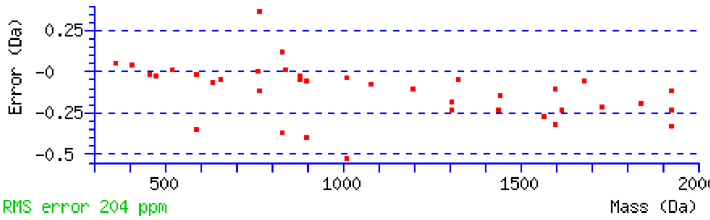

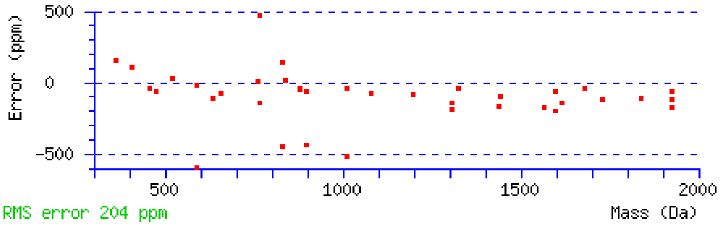

All matches to this query

Score	Mr(calc):	Delta	Sequence
125.8	2200.1263	0.0189	YNEDLELEDAIHTAILTLK
3.1	2200.1528	-0.0076	YPIINYTFTSGLSGVLGAATR
2.3	2200.1575	-0.0124	LAWLHPGVPKPCSVEGLGQR

2.2	2199.1300	1.0151	TIHSNQTVVIPENVNITLK
2.2	2199.1300	1.0151	TIHSNQTVVIPENVNITLK
1.8	2200.1261	0.0190	RMSVLVRKPEGAICLYTK
1.8	2200.1261	0.0190	RMSVLVRKPEGAICLYTK
1.2	2199.1349	1.0103	MKVLWAALLVTFLAGCQAK
0.8	2199.1487	0.9965	TSAMLTVRALPIKFTEGLR

Spectrum No: 54; Query: 45395; Rank: 1

Peptide View

MS/MS Fragmentation of **AQPAQPADEPAEKADEPMEH**
Found in **IPI00016832**, Tax_Id=9606 Gene_Symbol=PSMA1 Isoform Short of Proteasome subunit alpha type-1

Match to Query 45395: 2159.953032 from(720.991620,3+)
Title: 091224LimSK_Exosome3_05.874.874.3.dta
Data file C:\Temp\Mascoti\LSK_2D\091224LimSK_Exosome3_01_2D.mgf

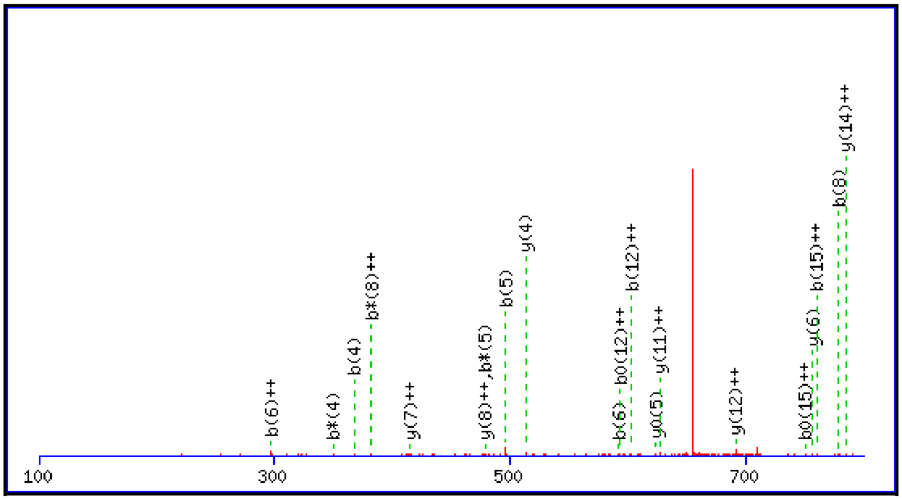

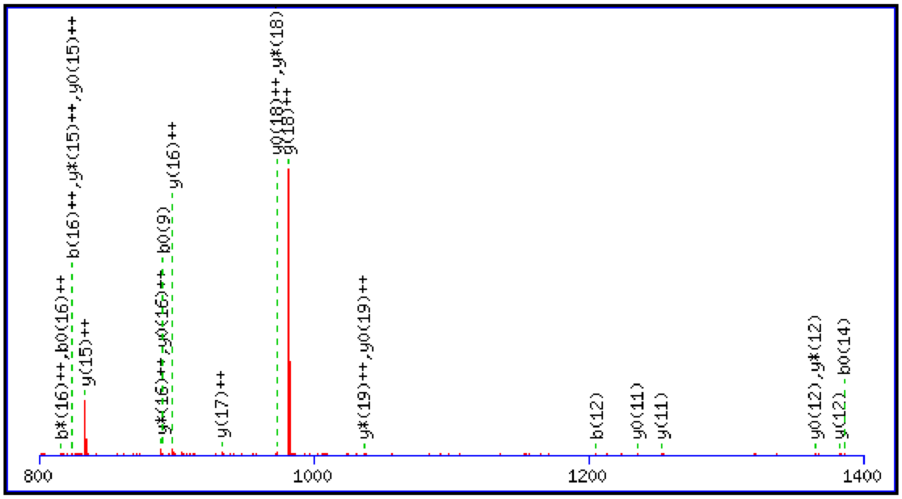

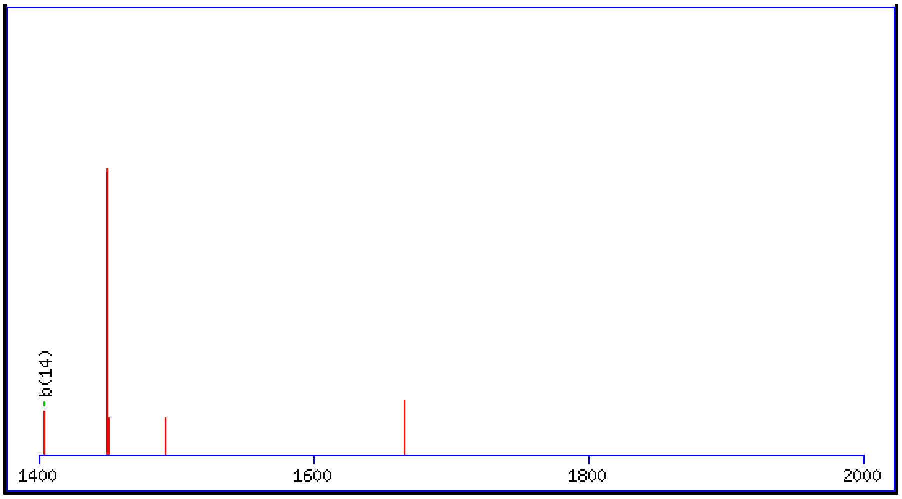

Monoisotopic mass of neutral peptide Mr(calc): 2159.9429
Fixed modifications: Carbamidomethyl (C)
Ions Score: 24 **Expect:** 1
Matches (Bold Red): 45/196 fragment ions using 103 most intense peaks

#	b	b^{++}	b*	b*$^{++}$	b^{0}	b^{0++}	Seq.	y	y^{++}	y*	y*$^{++}$	y^{0}	y^{0++}	#
1	72.0444	36.5258					A							20
2	200.1030	100.5551	183.0764	92.0418			Q	2089.9131	1045.4602	2072.8866	**1036.9469**	2071.9026	**1036.4549**	19
3	**297.1557**	149.0815	280.1292	140.5682			P	1961.8545	**981.4309**	1944.8280	**972.9176**	1943.8440	**972.4256**	18
4	**368.1928**	184.6001	**351.1663**	176.0868			A	1864.8018	**932.9045**	1847.7752	924.3913	1846.7912	923.8992	17
5	**496.2514**	248.6293	**479.2249**	240.1161			Q	1793.7647	**897.3860**	1776.7381	**888.8727**	1775.7541	**888.3807**	16
6	**593.3042**	**297.1557**	576.2776	288.6425			P	1665.7061	**833.3567**	1648.6795	**824.8434**	1647.6955	**824.3514**	15
7	664.3413	332.6743	647.3148	324.1610			A	1568.6533	**784.8303**	1551.6268	776.3170	1550.6428	775.8250	14
8	**779.3682**	390.1878	762.3417	**381.6745**	761.3577	381.1825	D	1497.6162	749.3117	1480.5897	740.7985	1479.6057	740.3065	13
9	908.4108	454.7091	891.3843	446.1958	**890.4003**	445.7038	E	**1382.5893**	**691.7983**	**1365.5627**	683.2850	**1364.5787**	682.7930	12
10	1005.4636	503.2354	988.4371	494.7222	987.4530	494.2302	P	**1253.5467**	**627.2770**	1236.5201	618.7637	**1235.5361**	618.2717	11
11	1076.5007	538.7540	1059.4742	530.2407	1058.4902	529.7487	A	1156.4939	578.7506	1139.4674	570.2373	1138.4834	569.7453	10
12	**1205.5433**	**603.2753**	1188.5168	594.7620	1187.5327	**594.2700**	E	1085.4568	543.2320	1068.4303	534.7188	1067.4462	534.2268	9
13	1333.6383	667.3228	1316.6117	658.8095	1315.6277	658.3175	K	956.4142	**478.7107**	939.3877	470.1975	938.4036	469.7055	8
14	**1404.6754**	702.8413	1387.6488	694.3281	**1386.6648**	693.8360	A	828.3192	**414.6633**			810.3087	405.6580	7
15	1519.7023	**760.3548**	1502.6758	751.8415	1501.6918	**751.3495**	D	**757.2821**	379.1447			739.2716	370.1394	6
16	1648.7449	**824.8761**	1631.7184	**816.3628**	1630.7344	**815.8708**	E	642.2552	321.6312			**624.2446**	312.6259	5
17	1745.7977	873.4025	1728.7711	864.8892	1727.7871	864.3972	P	**513.2126**	257.1099			495.2020	248.1047	4
18	1876.8382	938.9227	1859.8116	930.4094	1858.8276	929.9174	M	416.1598	208.5836			398.1493	199.5783	3
19	2005.8808	1003.4440	1988.8542	994.9307	1987.8702	994.4387	E	285.1193	143.0633			267.1088	134.0580	2
20							H	156.0768	78.5420					1

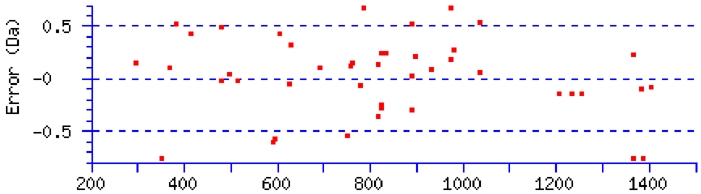

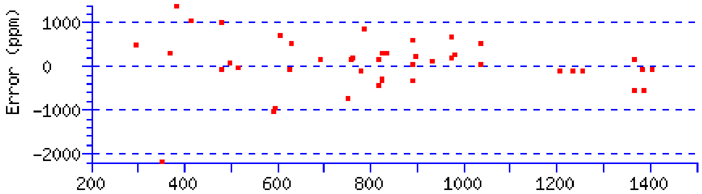

All matches to this query

Score	Mr(calc):	Delta	Sequence
24.0	2159.9429	0.0101	AQPAQPADEPAEKADEPMEH
9.6	2157.9432	2.0099	LLASWVSGSSGRSGGFMRK

9.1	2159.9574	-0.0043	YSFFVAMGYLTICHISR
8.3	2157.9432	2.0099	LLASWVSGSSGRSGGFMRK
8.0	2157.9476	2.0055	XSHGTKMMTPEVLAEAYGK
7.9	2157.9402	2.0129	SAQELPQVEAYSPSACSVR
7.2	2157.9670	1.9860	VASSDLVNMGISVVSYTLK
7.2	2157.9670	1.9860	VASSDLVNMGISVVSYTLK
6.8	2157.9418	2.0112	LGGTSLSLSVMEVNSGIYR
6.8	2158.9337	1.0193	TAENVRALSTEEKGFGYK

Spectrum No: 55; Query: 65422; Rank: 1

Peptide View

MS/MS Fragmentation of **DLEFTIYDDDDVSPFLEGLEERPQR**
Found in **IPI00016832**, Tax_Id=9606 Gene_Symbol=PSMA1 Isoform Short of Proteasome subunit alpha type-1

Match to Query 65422: 2997.408372 from(1000.143400,3+)
Title: 091224LimSK_Exosome3_06.8382.8382.3.dta
Data file C:\Temp\Mascoti\LSK_2D\091224LimSK_Exosome3_01_2D.mgf

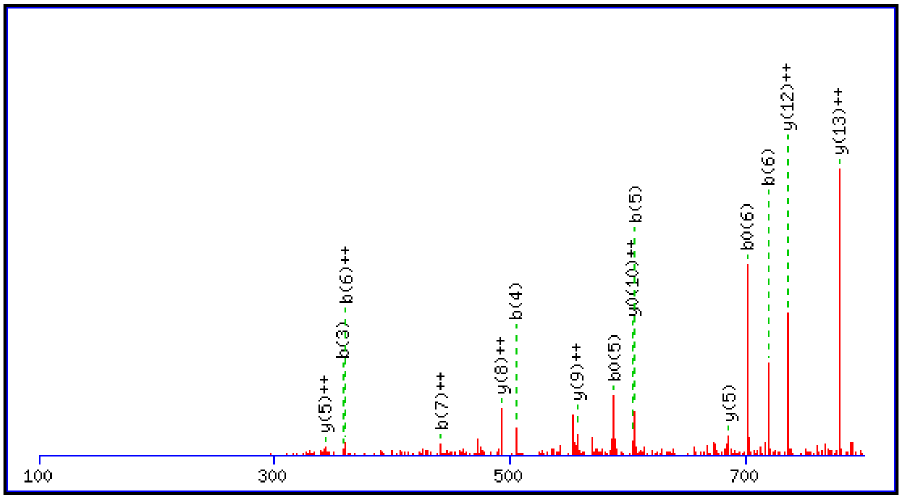

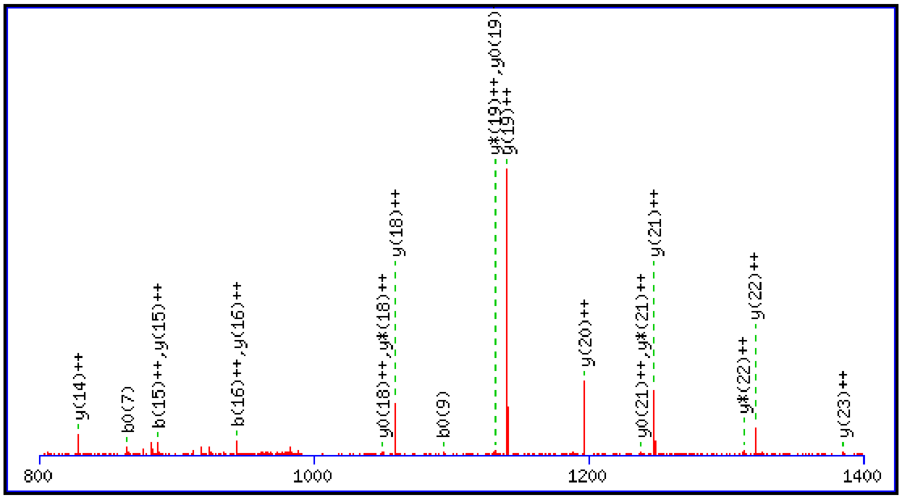

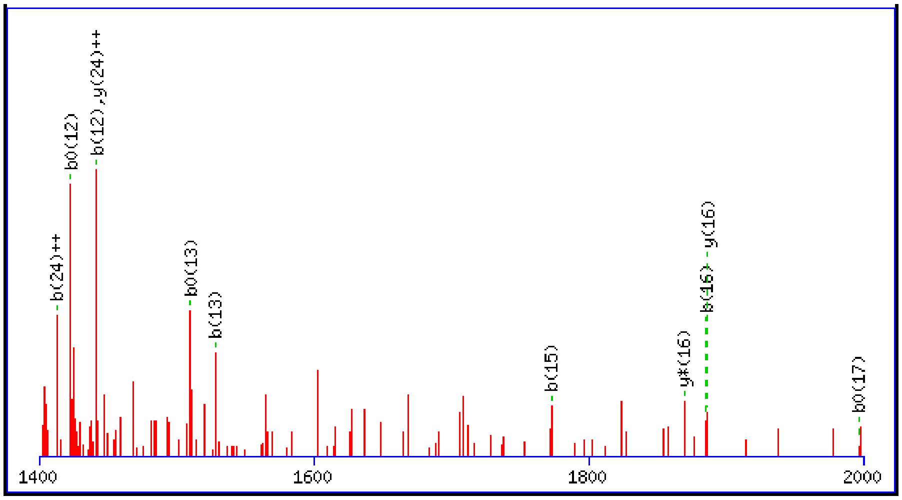

Monoisotopic mass of neutral peptide Mr(calc): 2997.3880
Fixed modifications: Carbamidomethyl (C)
Ions Score: 77 **Expect:** 5.9e-006
Matches (Bold Red): 46/238 fragment ions using 73 most intense peaks

#	b	b^{++}	b*	b^{*++}	b^0	b^{0++}	Seq.	y	y^{++}	y*	y^{*++}	y^0	y^{0++}	#
1	116.0342	58.5207			98.0237	49.5155	D							25
2	229.1183	115.0628			211.1077	106.0575	L	2883.3683	**1442.1878**	2866.3418	1433.6745	2865.3577	1433.1825	24
3	**358.1609**	179.5841			340.1503	170.5788	E	2770.2842	**1385.6458**	2753.2577	1377.1325	2752.2737	1376.6405	23
4	**505.2293**	253.1183			487.2187	244.1130	F	2641.2417	**1321.1245**	2624.2151	**1312.6112**	2623.2311	1312.1192	22
5	**606.2770**	303.6421			**588.2664**	294.6368	T	2494.1732	**1247.5903**	2477.1467	**1239.0770**	2476.1627	**1238.5850**	21
6	**719.3610**	**360.1842**			**701.3505**	351.1789	I	2393.1256	**1197.0664**	2376.0990	1188.5531	2375.1150	1188.0611	20
7	882.4244	**441.7158**			**864.4138**	432.7105	Y	2280.0415	**1140.5244**	2263.0149	**1132.0111**	2262.0309	**1131.5191**	19
8	997.4513	499.2293			979.4407	490.2240	D	2116.9782	**1058.9927**	2099.9516	**1050.4794**	2098.9676	**1049.9874**	18
9	1112.4782	556.7428			**1094.4677**	547.7375	D	2001.9512	1001.4793	1984.9247	992.9660	1983.9407	992.4740	17
10	1227.5052	614.2562			1209.4946	605.2510	D	**1886.9243**	**943.9658**	**1869.8977**	935.4525	1868.9137	934.9605	16
11	1342.5321	671.7697			1324.5216	662.7644	D	1771.8973	**886.4523**	1754.8708	877.9390	1753.8868	877.4470	15
12	**1441.6005**	721.3039			**1423.5900**	712.2986	V	1656.8704	**828.9388**	1639.8438	820.4256	1638.8598	819.9336	14
13	**1528.6326**	764.8199			**1510.6220**	755.8146	S	1557.8020	**779.4046**	1540.7754	770.8914	1539.7914	770.3993	13
14	1625.6853	813.3463			1607.6748	804.3410	P	1470.7700	**735.8886**	1453.7434	727.3753	1452.7594	726.8833	12
15	**1772.7538**	**886.8805**			1754.7432	877.8752	F	1373.7172	687.3622	1356.6906	678.8490	1355.7066	678.3570	11
16	**1885.8378**	**943.4225**			1867.8273	934.4173	L	1226.6488	613.8280	1209.6222	605.3148	1208.6382	**604.8227**	10
17	2014.8804	1007.9438			**1996.8698**	998.9386	E	1113.5647	**557.2860**	1096.5382	548.7727	1095.5541	548.2807	9
18	2071.9019	1036.4546			2053.8913	1027.4493	G	984.5221	**492.7647**	967.4956	484.2514	966.5116	483.7594	8
19	2184.9859	1092.9966			2166.9754	1083.9913	L	927.5007	464.2540	910.4741	455.7407	909.4901	455.2487	7
20	2314.0285	1157.5179			2296.0180	1148.5126	E	814.4166	407.7119	797.3900	399.1987	796.4060	398.7067	6
21	2443.0711	1222.0392			2425.0606	1213.0339	E	**685.3740**	**343.1906**	668.3474	334.6774	667.3634	334.1854	5
22	2599.1722	1300.0898	2582.1457	1291.5765	2581.1617	1291.0845	R	556.3314	278.6693	539.3049	270.1561			4
23	2696.2250	1348.6161	2679.1985	1340.1029	2678.2144	1339.6109	P	400.2303	200.6188	383.2037	192.1055			3
24	2824.2836	**1412.6454**	2807.2570	1404.1322	2806.2730	1403.6401	Q	303.1775	152.0924	286.1510	143.5791			2
25							R	175.1190	88.0631	158.0924	79.5498			1

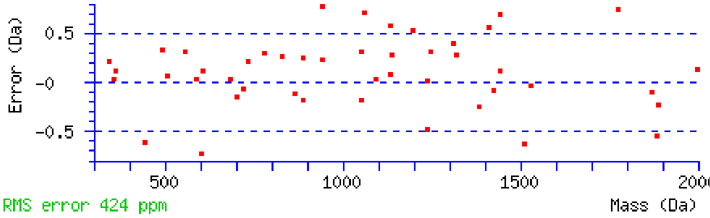

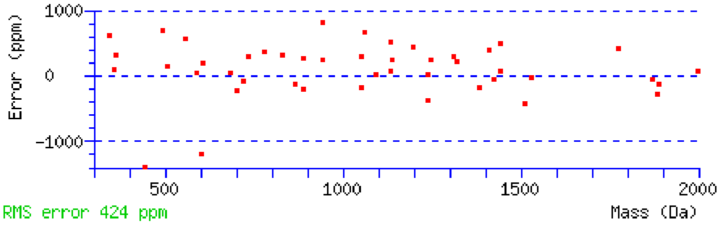

All matches to this query

Score	Mr(calc):	Delta	Sequence
77.2	2997.3880	0.0204	DLEFTIYDDDDVSPFLEGLEERPQR
8.8	2997.4356	-0.0272	ICGSPCPAVWPDVIKLPYFNTMKPK
8.8	2997.4356	-0.0272	LCGSPCPAVWPDVIKLPYFNTMKPK
7.7	2997.3919	0.0164	ETFMKTSLQTVDLRLAQLEELSNR
7.7	2997.3919	0.0164	ETFMKTSLQTVDLRLAQLEELSNR
7.6	2996.3908	1.0176	FGDEMPARYGGGGSGAAAGVVVGSGGGRGAGGSR
6.7	2995.3959	2.0124	YLVMDEADRILNMDFETEVDKILK
6.0	2995.4222	1.9861	LGNPEEGQRMRLSSAPSQAQDFSILGK
5.6	2996.3795	1.0289	NYKVMAAENIPENPLKYLYPNIDK
5.6	2997.3860	0.0224	MLIRHKMTHNPNRPLAECQFCHK

Spectrum No: 56; Query: 12922; Rank: 1

Peptide View

MS/MS Fragmentation of **ETLPAEQDLTTK**
Found in **IPI00016832**, Tax_Id=9606 Gene_Symbol=PSMA1 Isoform Short of Proteasome subunit alpha type-1

Match to Query 12922: 1344.683928 from(673.349240,2+)
Title: 091224LimSK_Exosome3_05.1782.1782.2.dta
Data file C:\Temp\Mascoti\LSK_2D\091224LimSK_Exosome3_01_2D.mgf

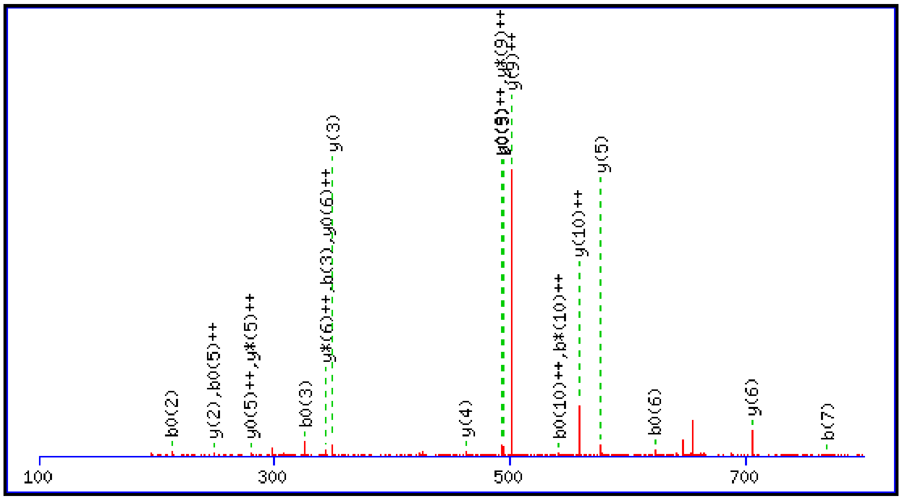

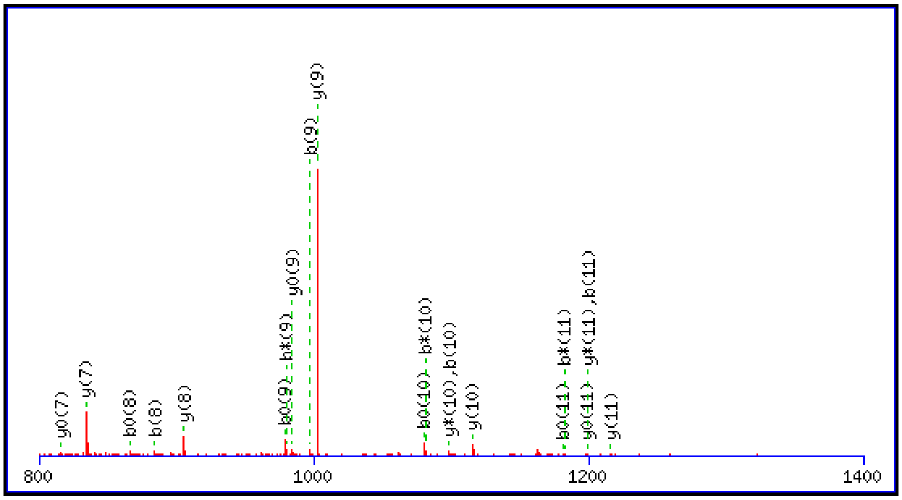

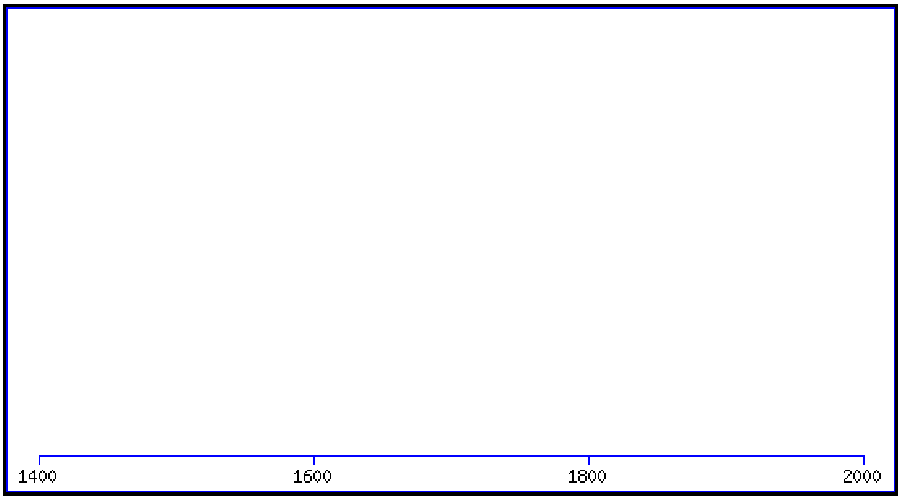

Monoisotopic mass of neutral peptide Mr(calc): 1344.6773
Fixed modifications: Carbamidomethyl (C)
Ions Score: 78 **Expect:** 3e-006
Matches (Bold Red): 43/118 fragment ions using 53 most intense peaks

#	b	b^{++}	b*	b^{*++}	b^{0}	b^{0++}	Seq.	y	y^{++}	y*	y^{*++}	y^{0}	y^{0++}	#
1	130.0499	65.5286			112.0393	56.5233	E							12
2	231.0975	116.0524			**213.0870**	107.0471	T	**1216.6420**	608.8246	**1199.6154**	600.3113	**1198.6314**	599.8193	11
3	**344.1816**	172.5944			**326.1710**	163.5892	L	**1115.5943**	**558.3008**	**1098.5677**	549.7875	1097.5837	549.2955	10
4	441.2344	221.1208			423.2238	212.1155	P	**1002.5102**	**501.7587**	985.4837	**493.2455**	**984.4997**	**492.7535**	9
5	512.2715	256.6394			**494.2609**	**247.6341**	A	**905.4575**	453.2324	888.4309	444.7191	887.4469	444.2271	8
6	641.3141	321.1607			**623.3035**	312.1554	E	**834.4203**	417.7138	817.3938	409.2005	**816.4098**	408.7085	7
7	**769.3727**	385.1900	752.3461	376.6767	751.3621	376.1847	Q	**705.3777**	353.1925	688.3512	**344.6792**	687.3672	**344.1872**	6
8	**884.3996**	442.7034	867.3731	434.1902	**866.3890**	433.6982	D	**577.3192**	289.1632	560.2926	**280.6499**	559.3086	**280.1579**	5
9	**997.4837**	499.2455	**980.4571**	490.7322	**979.4731**	490.2402	L	**462.2922**	231.6498	445.2657	223.1365	444.2817	222.6445	4
10	**1098.5313**	549.7693	**1081.5048**	**541.2560**	**1080.5208**	**540.7640**	T	**349.2082**	175.1077	332.1816	166.5944	331.1976	166.1024	3
11	**1199.5790**	600.2932	**1182.5525**	591.7799	**1181.5685**	591.2879	T	**248.1605**	124.5839	231.1339	116.0706	230.1499	115.5786	2
12							K	147.1128	74.0600	130.0863	65.5468			1

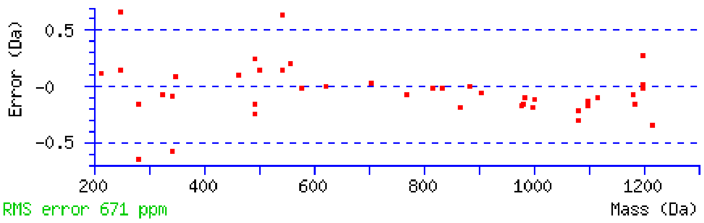

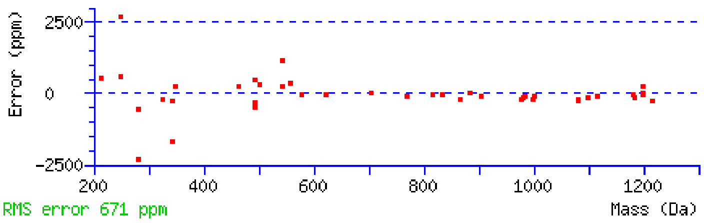

All matches to this query

Score	Mr(calc):	Delta	Sequence
77.8	1344.6773	0.0067	ETLPAEQDLTTK
11.2	1342.6646	2.0193	DLLAPPSPALGGR
10.2	1344.6915	-0.0075	DPLSAPALRLGR
8.3	1344.6737	0.0102	MNVSPVPPLRR
7.9	1342.6881	1.9958	EVLEGLDYLHR
7.5	1343.6711	1.0128	LLGPGGRPSSPAR
7.0	1344.6819	0.0021	KTSKIASLIPK
6.8	1342.6662	2.0177	KVPVSVNLLSK
6.6	1344.6819	0.0021	KTSKIASLIPK
6.5	1344.6826	0.0013	DVERFFKGYGK

Spectrum No: 57; Query: 12473; Rank: 1

Peptide View

MS/MS Fragmentation of **FVFDRPLPVSR**
Found in **IPI00016832**, Tax_Id=9606 Gene_Symbol=PSMA1 Isoform Short of Proteasome subunit alpha type-1

Match to Query 12473: 1331.742972 from(444.921600,3+)
Title: 091224LimSK_Exosome3_06.4388.4388.3.dta
Data file C:\Temp\Mascoti\LSK_2D\091224LimSK_Exosome3_01_2D.mgf

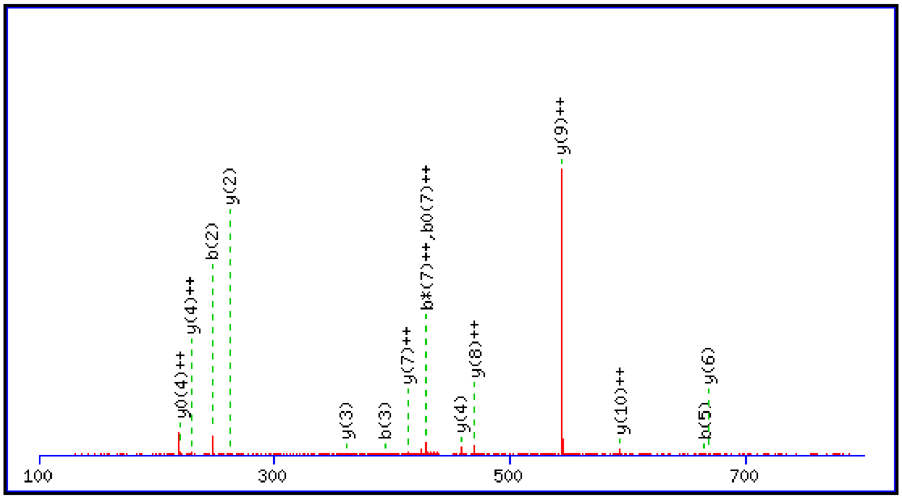

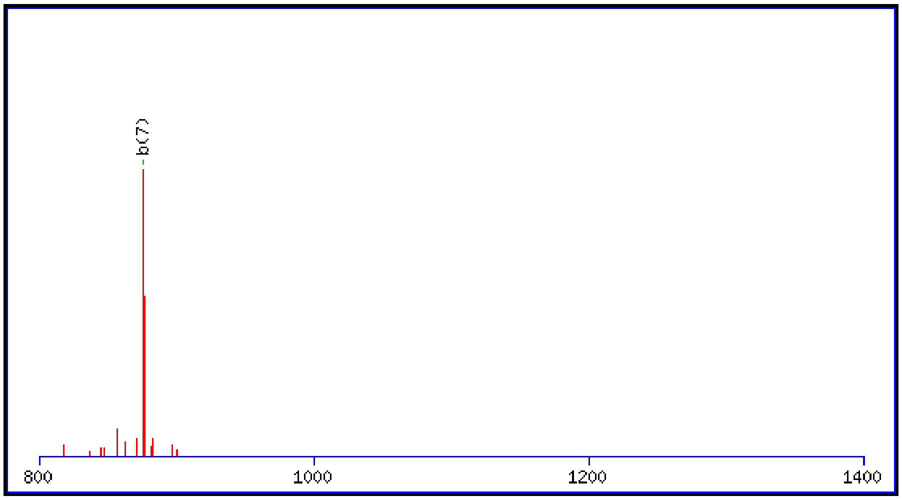

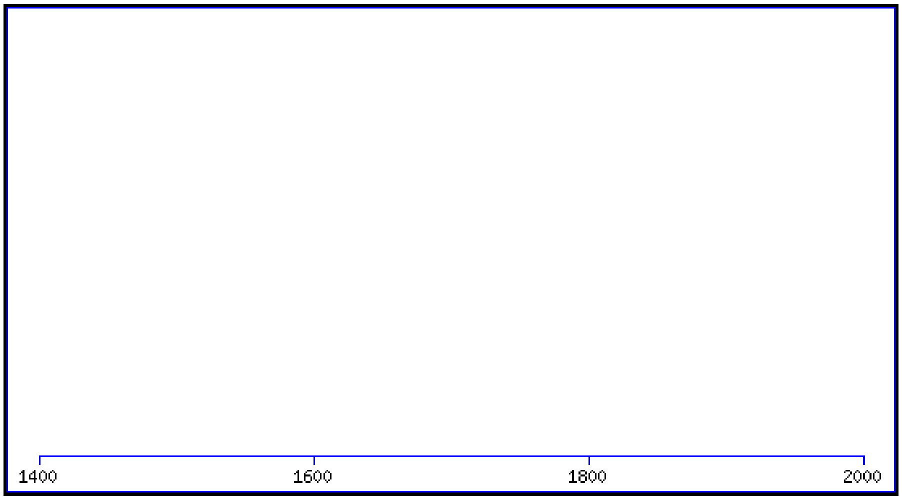

Monoisotopic mass of neutral peptide Mr(calc): 1331.7350
Fixed modifications: Carbamidomethyl (C)
Ions Score: 29 **Expect:** 0.11
Matches (Bold Red): 16/104 fragment ions using 33 most intense peaks

#	b	b^{++}	b*	b^{*++}	b^{0}	b^{0++}	Seq.	y	y^{++}	y*	y^{*++}	y^{0}	y^{0++}	#
1	148.0757	74.5415					F							11
2	**247.1441**	124.0757					V	1185.6739	**593.3406**	1168.6473	584.8273	1167.6633	584.3353	10
3	**394.2125**	197.6099					F	1086.6055	**543.8064**	1069.5789	535.2931	1068.5949	534.8011	9
4	509.2395	255.1234			491.2289	246.1181	D	939.5370	**470.2722**	922.5105	461.7589	921.5265	461.2669	8
5	**665.3406**	333.1739	648.3140	324.6606	647.3300	324.1686	R	824.5101	**412.7587**	807.4835	404.2454	806.4995	403.7534	7
6	762.3933	381.7003	745.3668	373.1870	744.3828	372.6950	P	**668.4090**	334.7081	651.3824	326.1949	650.3984	325.7028	6
7	**875.4774**	438.2423	858.4509	**429.7291**	857.4668	**429.2371**	L	571.3562	286.1817	554.3297	277.6685	553.3457	277.1765	5
8	972.5302	486.7687	955.5036	478.2554	954.5196	477.7634	P	**458.2722**	**229.6397**	441.2456	221.1264	440.2616	**220.6344**	4
9	1071.5986	536.3029	1054.5720	527.7897	1053.5880	527.2976	V	**361.2194**	181.1133	344.1928	172.6001	343.2088	172.1081	3
10	1158.6306	579.8189	1141.6041	571.3057	1140.6200	570.8137	S	**262.1510**	131.5791	245.1244	123.0659	244.1404	122.5738	2
11							R	175.1190	88.0631	158.0924	79.5498			1

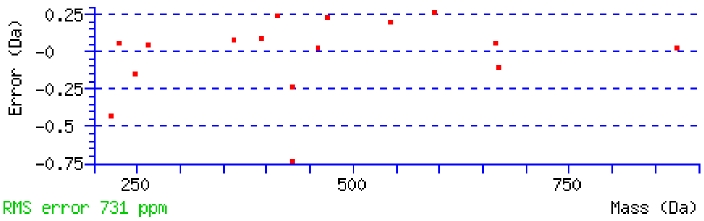

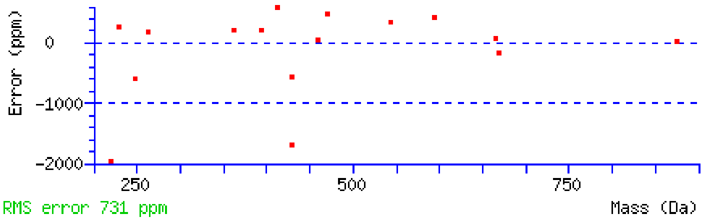

All matches to this query

Score	Mr(calc):	Delta	Sequence
29.1	1331.7350	0.0080	FVFDRPLPVSR
8.4	1329.7252	2.0178	QTVSQVVNAKEK
6.6	1331.7561	-0.0131	QIPPRPPEGLTK
6.5	1330.7503	0.9927	MVAVNLATGKRR
5.9	1330.7486	0.9944	KLSNAPRPLKK
5.1	1329.7405	2.0025	RSFLGQKDPALV
5.1	1331.7534	-0.0104	LELAGAPHGRRR
5.0	1329.7252	2.0178	VSEEVEKGRAVK
4.8	1331.7449	-0.0019	AFLVIADSLQQK
4.8	1330.7486	0.9944	XHVITALRVKK

Spectrum No: 58; Query: 35891; Rank: 1

Peptide View

MS/MS Fragmentation of **HMSEFMECNLNELVK**
Found in **IPI00016832**, Tax_Id=9606 Gene_Symbol=PSMA1 Isoform Short of Proteasome subunit alpha type-1

Match to Query 35891: 1879.841868 from(940.928210,2+)
Title: 091224LimSK_Exosome3_06.5841.5841.2.dta
Data file C:\Temp\Mascoti\LSK_2D\091224LimSK_Exosome3_01_2D.mgf

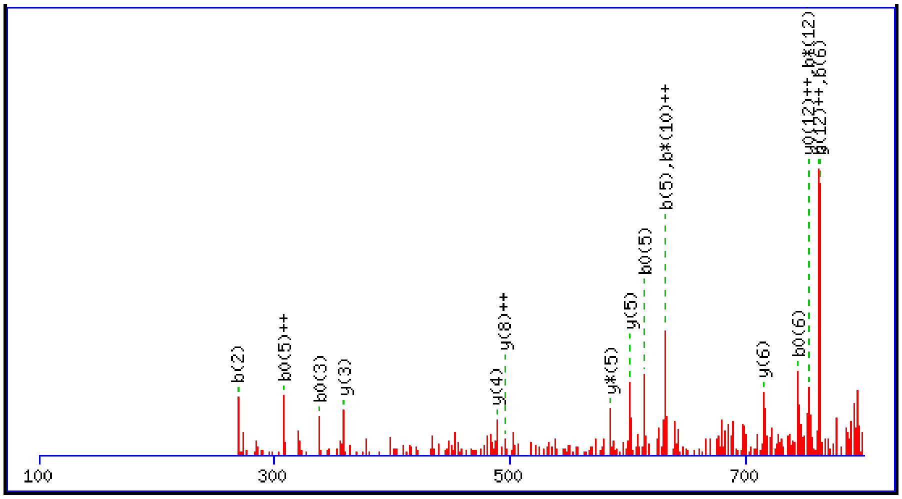

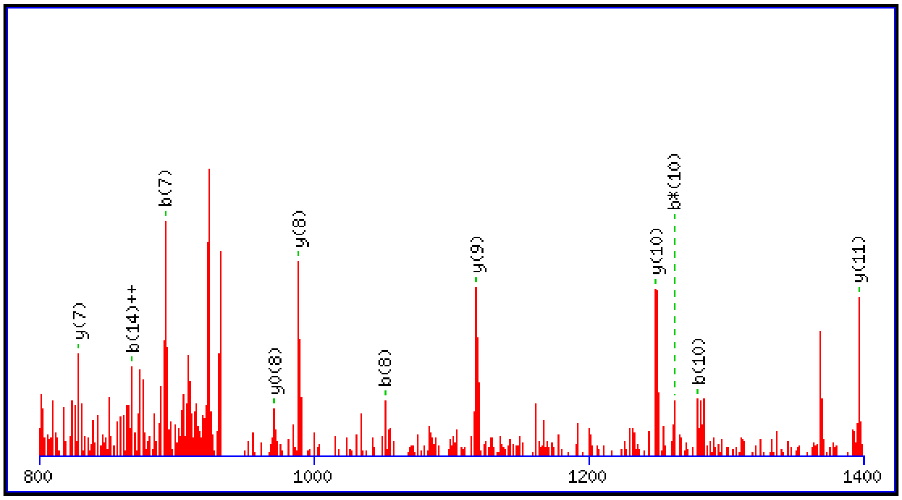

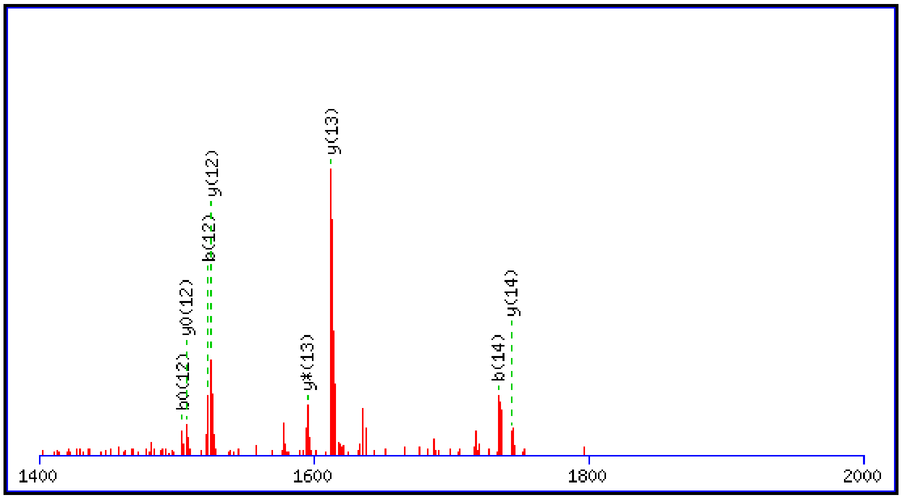

Monoisotopic mass of neutral peptide Mr(calc): 1879.8266
Fixed modifications: Carbamidomethyl (C)
Ions Score: 62 **Expect:** 0.00017
Matches (Bold Red): 37/142 fragment ions using 80 most intense peaks

#	b	b^{++}	b*	b^{*++}	b^0	b^{0++}	Seq.	y	y^{++}	y*	y^{*++}	y^0	y^{0++}	#
1	138.0662	69.5367					H							15
2	**269.1067**	135.0570					M	**1743.7750**	872.3912	1726.7485	863.8779	1725.7645	863.3859	14
3	356.1387	178.5730			**338.1281**	169.5677	S	**1612.7346**	806.8709	**1595.7080**	798.3576	1594.7240	797.8656	13
4	485.1813	243.0943			467.1707	234.0890	E	**1525.7025**	**763.3549**	1508.6760	754.8416	**1507.6920**	**754.3496**	12
5	**632.2497**	316.6285			**614.2391**	**307.6232**	F	**1396.6599**	698.8336	1379.6334	690.3203	1378.6494	689.8283	11
6	**763.2902**	382.1487			**745.2796**	373.1435	M	**1249.5915**	625.2994	1232.5650	616.7861	1231.5810	616.2941	10

7	**892.3328**	446.6700			874.3222	437.6647	E	**1118.5510**	559.7792	1101.5245	551.2659	1100.5405	550.7739	9
8	**1052.3634**	526.6854			1034.3529	517.6801	C	**989.5084**	**495.2579**	972.4819	486.7446	**971.4979**	486.2526	8
9	1166.4064	583.7068	1149.3798	575.1935	1148.3958	574.7015	N	**829.4778**	415.2425	812.4512	406.7293	811.4672	406.2373	7
10	**1279.4904**	640.2489	**1262.4639**	**631.7356**	1261.4799	631.2436	L	**715.4349**	358.2211	698.4083	349.7078	697.4243	349.2158	6
11	1393.5334	697.2703	1376.5068	688.7570	1375.5228	688.2650	N	**602.3508**	301.6790	**585.3243**	293.1658	584.3402	292.6738	5
12	**1522.5759**	**761.7916**	1505.5494	**753.2783**	**1504.5654**	752.7863	E	**488.3079**	244.6576	471.2813	236.1443	470.2973	235.6523	4
13	1635.6600	818.3336	1618.6335	809.8204	1617.6494	809.3284	L	**359.2653**	180.1363	342.2387	171.6230			3
14	**1734.7284**	**867.8678**	1717.7019	859.3546	1716.7179	858.8626	V	246.1812	123.5942	229.1547	115.0810			2
15							K	147.1128	74.0600	130.0863	65.5468			1

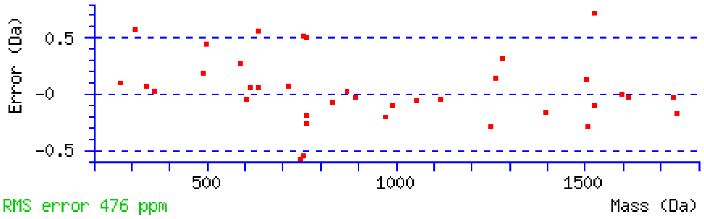

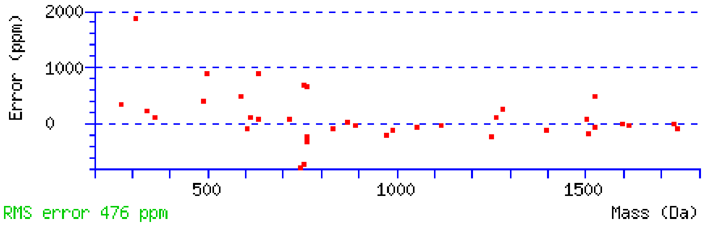

All matches to this query

Score	Mr(calc):	Delta	Sequence
61.6	1879.8266	0.0152	HMSEFMECNLNELVK
5.1	1879.8270	0.0148	KFRAHVEDSDLIYK
4.7	1879.8499	-0.0080	ISDQNASGAPPMTVREK
4.7	1879.8499	-0.0080	ISDQNASGAPPMTVREK
2.8	1879.8481	-0.0063	KSVPRNSLESAEYLK
2.1	1879.8369	0.0049	GENVFGSAEIIASLSVK
1.0	1878.8334	1.0085	GSRGGNTSSSHGSAGPKQK
0.5	1878.8336	1.0083	QTMPRPYTFQVSSGGK
0.2	1878.8223	1.0196	CFEISDADNKVVFAGK

Spectrum No: 59; Query: 16758; Rank: 1

Peptide View

MS/MS Fragmentation of **IHQIEYAMEAVK**
Found in **IPI00016832**, Tax_Id=9606 Gene_Symbol=PSMA1 Isoform Short of Proteasome subunit alpha type-1

Match to Query 16758: 1430.732288 from(716.373420,2+)
Title: 091224LimSK_Exosome3_05.2499.2499.2.dta
Data file C:\Temp\Mascoti\LSK_2D\091224LimSK_Exosome3_01_2D.mgf

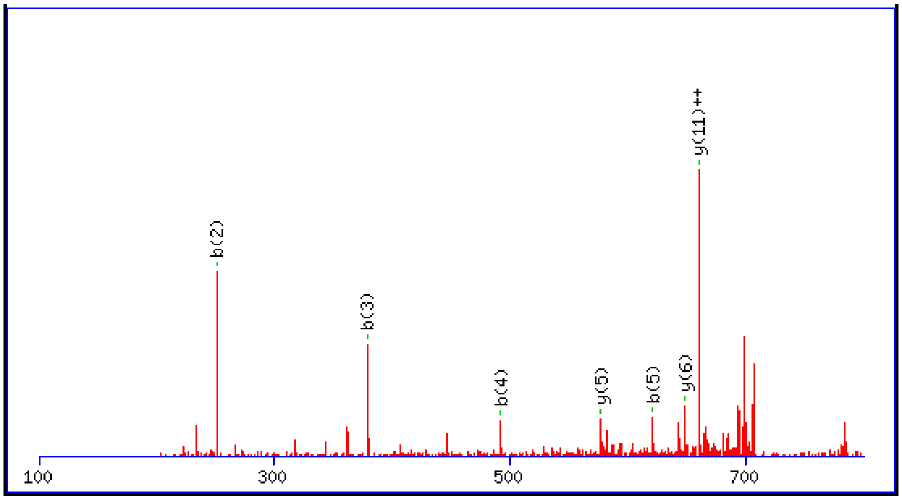

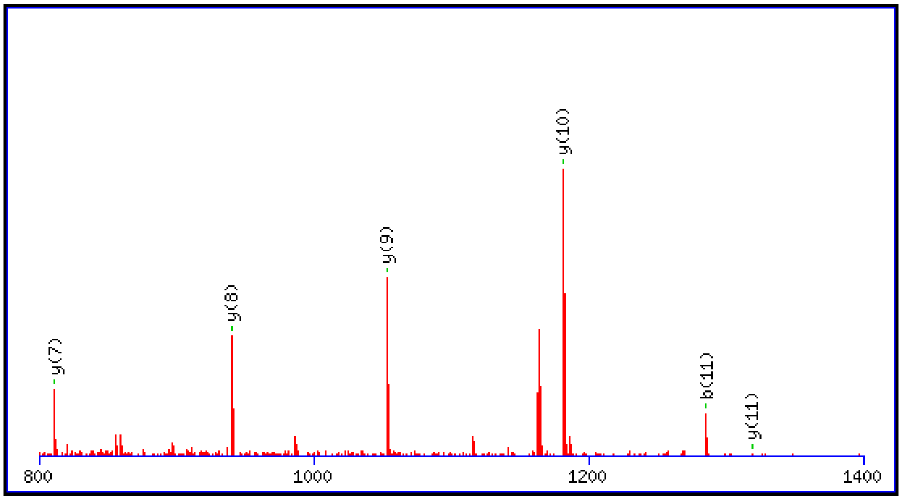

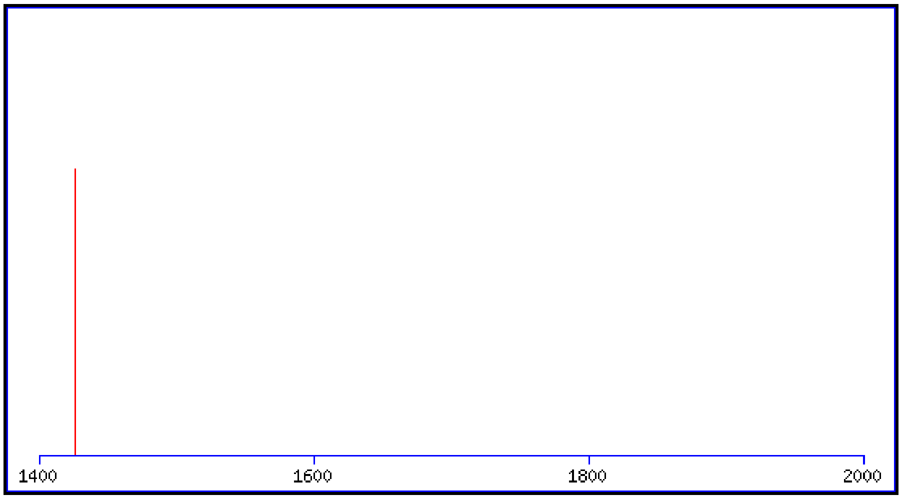

Monoisotopic mass of neutral peptide Mr(calc): 1430.7227
Fixed modifications: Carbamidomethyl (C)
Ions Score: 49 **Expect:** 0.0022
Matches (Bold Red): 13/114 fragment ions using 20 most intense peaks

#	b	b++	b*	b*++	b0	b0++	Seq.	y	y++	y*	y*++	y0	y0++	#
1	114.0913	57.5493					I							12
2	**251.1503**	126.0788					H	**1318.6460**	**659.8266**	1301.6195	651.3134	1300.6354	650.8214	11
3	**379.2088**	190.1081	362.1823	181.5948			Q	**1181.5871**	591.2972	1164.5605	582.7839	1163.5765	582.2919	10
4	**492.2929**	246.6501	475.2663	238.1368			I	**1053.5285**	527.2679	1036.5020	518.7546	1035.5179	518.2626	9
5	**621.3355**	311.1714	604.3089	302.6581	603.3249	302.1661	E	**940.4444**	470.7259	923.4179	462.2126	922.4339	461.7206	8
6	784.3988	392.7030	767.3723	384.1898	766.3883	383.6978	Y	**811.4019**	406.2046	794.3753	397.6913	793.3913	397.1993	7

**7**	855.4359	428.2216	838.4094	419.7083	837.4254	419.2163	**A**	**648.3385**	324.6729	631.3120	316.1596	630.3280	315.6676	**6**
**8**	986.4764	493.7418	969.4499	485.2286	968.4659	484.7366	**M**	**577.3014**	289.1543	560.2749	280.6411	559.2908	280.1491	**5**
**9**	1115.5190	558.2631	1098.4925	549.7499	1097.5084	549.2579	**E**	446.2609	223.6341	429.2344	215.1208	428.2504	214.6288	**4**
**10**	1186.5561	593.7817	1169.5296	585.2684	1168.5456	584.7764	**A**	317.2183	159.1128	300.1918	150.5995			**3**
**11**	**1285.6245**	643.3159	1268.5980	634.8026	1267.6140	634.3106	**V**	246.1812	123.5942	229.1547	115.0810			**2**
**12**							**K**	147.1128	74.0600	130.0863	65.5468			**1**

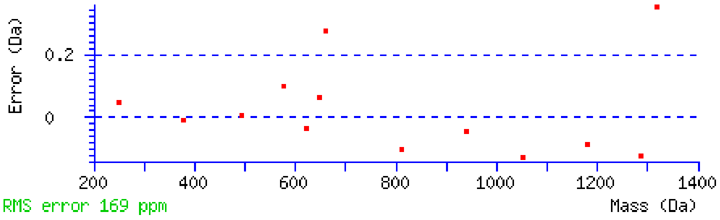

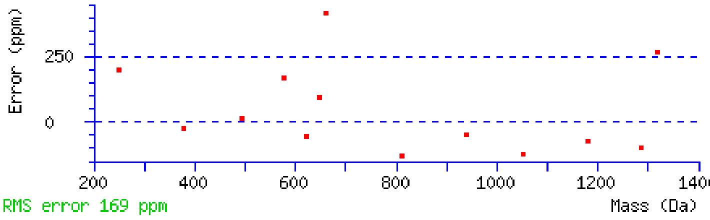

All matches to this query

Score	Mr(calc):	Delta	Sequence
49.0	1430.7227	0.0095	IHQIEYAMEAVK
13.1	1430.7395	-0.0072	QHLLSLQQRTK
6.8	1430.7184	0.0139	IHGVLTHYQKR
6.6	1430.7395	-0.0072	QHLLSLQQRTK
6.5	1430.7184	0.0139	HLQSLHKFVSR
6.4	1430.7340	-0.0017	IHTGEKPYKCAK
6.0	1430.7184	0.0139	IHGVLTHYQKR
5.2	1430.7307	0.0016	LHTFGNPFKQDK
4.1	1430.7340	-0.0017	IHTKEQPYKCK
3.4	1430.7252	0.0071	EQAEIQIKEESK

Spectrum No: 60; Query: 50562; Rank: 1

Peptide View

MS/MS Fragmentation of **KAQPAQPADEPAEKADEPMEH**
Found in **IPI00016832**, Tax_Id=9606 Gene_Symbol=PSMA1 Isoform Short of Proteasome subunit alpha type-1

Match to Query 50562: 2288.047242 from(763.689690,3+)
Title: 091224LimSK_Exosome3_05.523.523.3.dta
Data file C:\Temp\Mascoti\LSK_2D\091224LimSK_Exosome3_01_2D.mgf

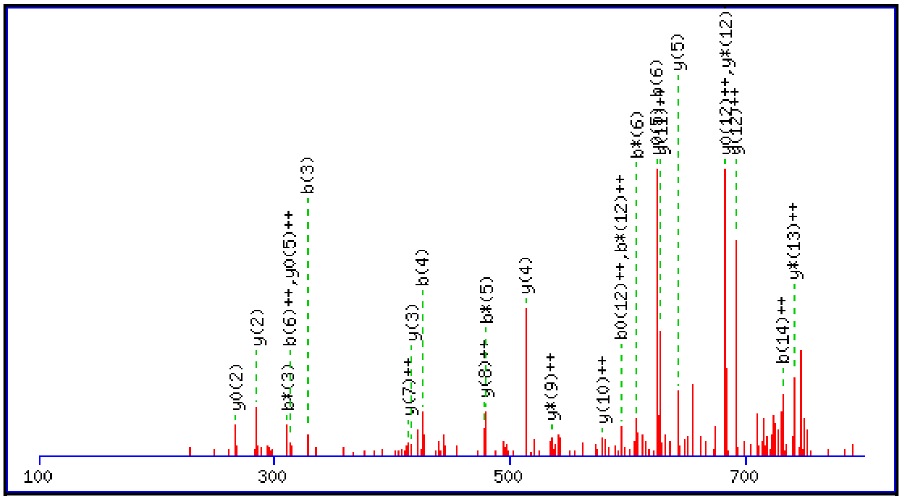

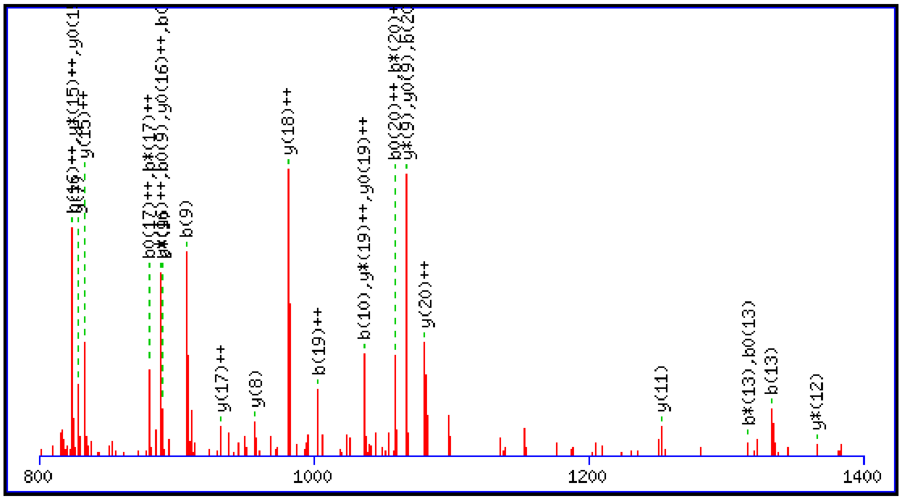

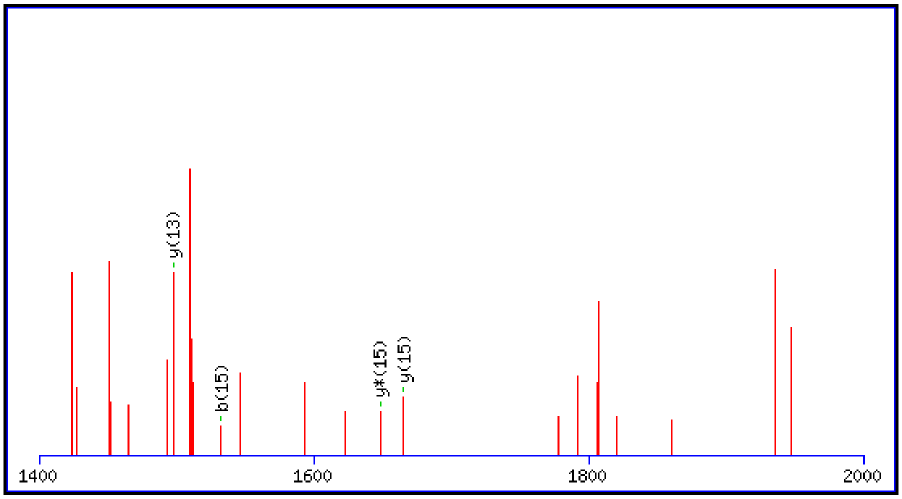

Monoisotopic mass of neutral peptide Mr(calc): 2288.0379
Fixed modifications: Carbamidomethyl (C)
Ions Score: 45 **Expect:** 0.01
Matches (Bold Red): 61/208 fragment ions using 102 most intense peaks

#	b	b^{++}	b*	b^{*++}	b^0	b^{0++}	Seq.	y	y^{++}	y*	y^{*++}	y^0	y^{0++}	#
1	129.1022	65.0548	112.0757	56.5415			K							21
2	200.1394	100.5733	183.1128	92.0600			A	2160.9502	**1080.9788**	2143.9237	1072.4655	2142.9397	1071.9735	20
3	**328.1979**	164.6026	**311.1714**	156.0893			Q	2089.9131	1045.4602	2072.8866	**1036.9469**	2071.9026	**1036.4549**	19
4	**425.2507**	213.1290	408.2241	204.6157			P	1961.8545	**981.4309**	1944.8280	972.9176	1943.8440	972.4256	18
5	496.2878	248.6475	**479.2613**	240.1343			A	1864.8018	**932.9045**	1847.7752	924.3913	1846.7912	923.8992	17
6	**624.3464**	**312.6768**	**607.3198**	304.1636			Q	1793.7647	897.3860	1776.7381	**888.8727**	1775.7541	**888.3807**	16
7	721.3992	361.2032	704.3726	352.6899			P	**1665.7061**	**833.3567**	**1648.6795**	**824.8434**	1647.6955	**824.3514**	15
8	792.4363	396.7218	775.4097	388.2085			A	1568.6533	784.8303	1551.6268	776.3170	1550.6428	775.8250	14
9	**907.4632**	454.2352	**890.4367**	445.7220	**889.4526**	445.2300	D	**1497.6162**	749.3117	1480.5897	**740.7985**	1479.6057	740.3065	13
10	**1036.5058**	518.7565	1019.4793	510.2433	1018.4952	509.7513	E	1382.5893	**691.7983**	**1365.5627**	**683.2850**	1364.5787	**682.7930**	12
11	1133.5586	567.2829	1116.5320	558.7696	1115.5480	558.2776	P	**1253.5467**	**627.2770**	1236.5201	618.7637	1235.5361	618.2717	11
12	1204.5957	602.8015	1187.5691	**594.2882**	1186.5851	**593.7962**	A	1156.4939	**578.7506**	1139.4674	570.2373	1138.4834	569.7453	10
13	**1333.6383**	667.3228	**1316.6117**	658.8095	**1315.6277**	658.3175	E	1085.4568	543.2320	**1068.4303**	**534.7188**	**1067.4462**	534.2268	9
14	1461.7332	**731.3703**	1444.7067	722.8570	1443.7227	722.3650	K	**956.4142**	**478.7107**	939.3877	470.1975	938.4036	469.7055	8
15	**1532.7703**	766.8888	1515.7438	758.3755	1514.7598	757.8835	A	**828.3192**	**414.6633**			810.3087	405.6580	7
16	1647.7973	**824.4023**	1630.7707	815.8890	1629.7867	815.3970	D	757.2821	379.1447			739.2716	370.1394	6
17	1776.8399	**888.9236**	1759.8133	**880.4103**	1758.8293	**879.9183**	E	**642.2552**	321.6312			**624.2446**	**312.6259**	5
18	1873.8926	937.4500	1856.8661	928.9367	1855.8821	928.4447	P	**513.2126**	257.1099			495.2020	248.1047	4
19	2004.9331	**1002.9702**	1987.9066	994.4569	1986.9226	993.9649	M	**416.1598**	208.5836			398.1493	199.5783	3

**20**	2133.9757	**1067.4915**	2116.9492	**1058.9782**	2115.9652	**1058.4862**	**E**	**285.1193**	143.0633			**267.1088**	134.0580	**2**
**21**							**H**	156.0768	78.5420					**1**

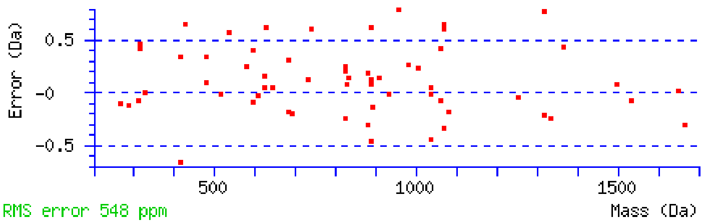

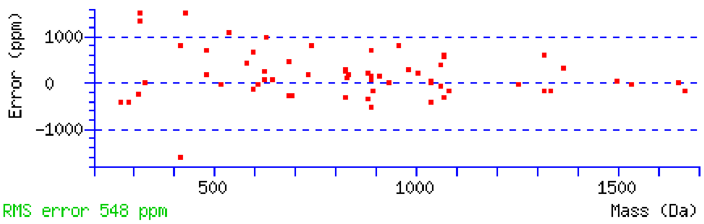

All matches to this query

Score	Mr(calc):	Delta	Sequence
44.5	2288.0379	0.0094	KAQPAQPADEPAEKADEPMEH
9.0	2288.0686	-0.0213	GGADVSGGVSAPDISLGEGHLSVK
7.3	2287.0572	0.9900	KISPKGVDIVMDPLGGSDTAK
4.8	2287.0498	0.9975	QGLKSPQESLSDLGAIESLR
4.2	2286.0318	2.0155	NLPTKEQNFSHSISENFSK
4.2	2286.0318	2.0155	NLPTKEQNFSHSISENFSK
3.9	2287.0368	1.0105	WVNAQFSKEYQKHMETFD
3.3	2288.0459	0.0013	MIGPTHGQMTVTRLVNTLK
3.3	2286.0384	2.0088	DVQSLSLRIMLAEILTTK
2.9	2287.0572	0.9900	KISPKGVDIVMDPLGGSDTAK

Spectrum No: 61; Query: 1985; Rank: 1

Peptide View

MS/MS Fragmentation of **LLCNFMR**
Found in **IPI00016832**, Tax_Id=9606 Gene_Symbol=PSMA1 Isoform Short of Proteasome subunit alpha type-1

Match to Query 1985: 952.466588 from(477.240570,2+)
Title: 091224LimSK_Exosome3_06.3045.3045.2.dta
Data file C:\Temp\Mascoti\LSK_2D\091224LimSK_Exosome3_01_2D.mgf

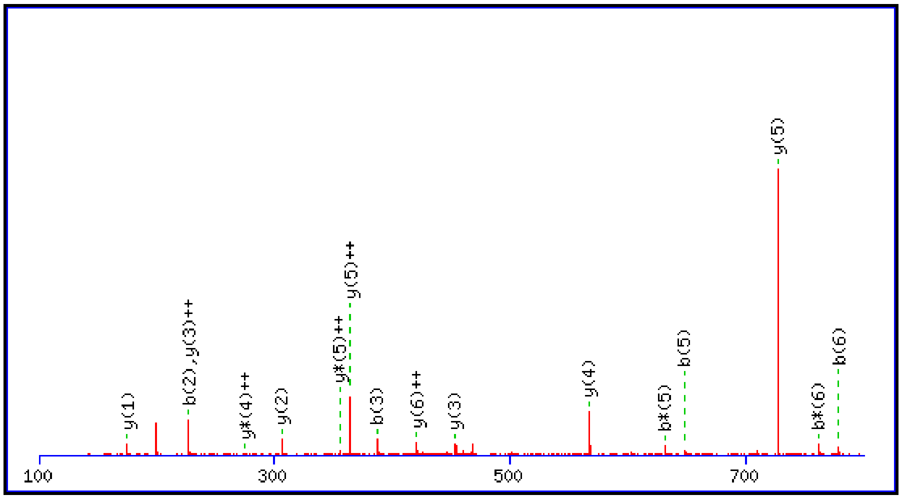

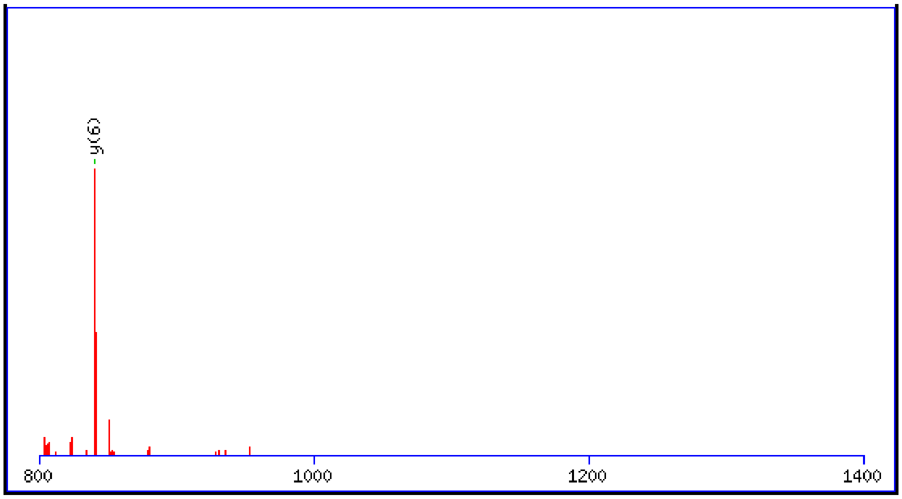

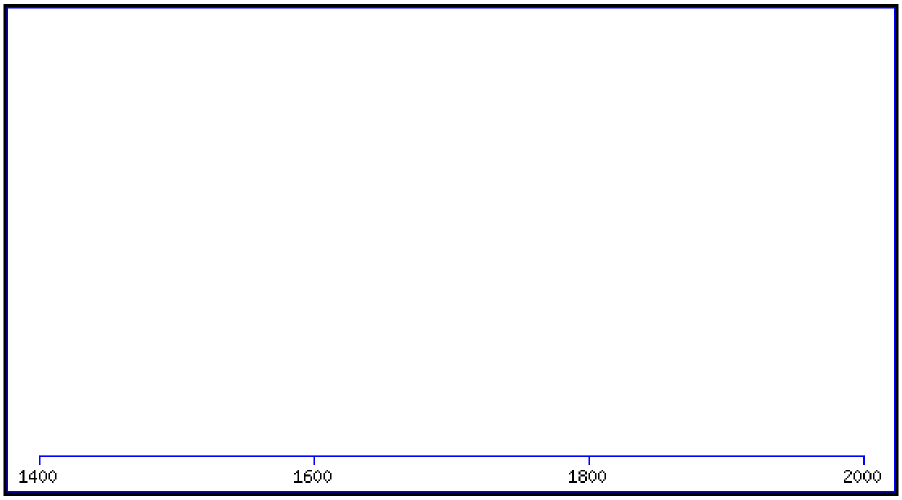

Monoisotopic mass of neutral peptide Mr(calc): 952.4623
Fixed modifications: Carbamidomethyl (C)
Ions Score: 45 **Expect:** 0.0048
Matches (Bold Red): 17/42 fragment ions using 26 most intense peaks

#	b	b++	b*	b*++	Seq.	y	y++	y*	y*++	#
1	114.0913	57.5493			L					7
2	**227.1754**	114.0913			L	**840.3855**	**420.6964**	823.3589	412.1831	6
3	**387.2061**	194.1067			C	**727.3014**	**364.1544**	710.2749	**355.6411**	5
4	501.2490	251.1281	484.2224	242.6149	N	**567.2708**	284.1390	550.2442	**275.6258**	4
5	**648.3174**	324.6623	**631.2908**	316.1491	F	**453.2279**	**227.1176**	436.2013	218.6043	3
6	**779.3579**	390.1826	**762.3313**	381.6693	M	**306.1594**	153.5834	289.1329	145.0701	2
7					R	**175.1190**	88.0631	158.0924	79.5498	1

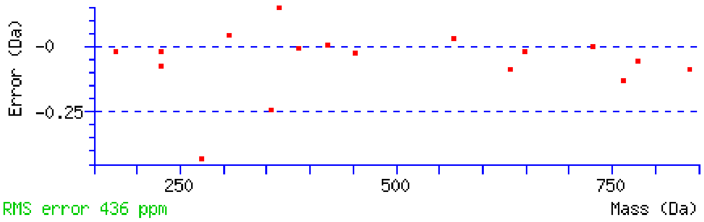

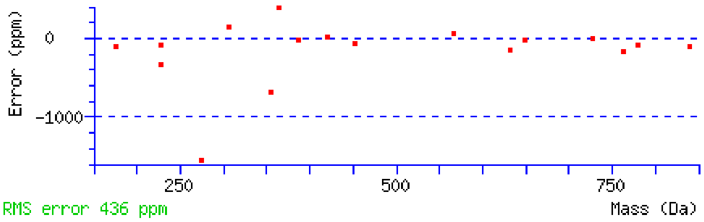

All matches to this query

Score	Mr(calc):	Delta	Sequence
44.8	952.4623	0.0043	LLCNFMR
19.1	952.4743	-0.0077	IIQISSGR

16.9	952.4743	-0.0077	LLSNAISR
15.6	952.4743	-0.0077	LLVQTGSR
14.4	952.4630	0.0036	LIIEETR
14.3	952.4743	-0.0077	IIQISSGR
13.8	952.4743	-0.0077	LLVQTGSR
13.1	951.4539	1.0127	GSGGRGLLVG
12.2	952.4630	0.0036	LLELETR
10.2	952.4743	-0.0077	LLEKSQR

Spectrum No: 62; Query: 398; Rank: 1

Peptide View

MS/MS Fragmentation of **LVSLIGSK**
Found in **IPI00016832**, Tax_Id=9606 Gene_Symbol=PSMA1 Isoform Short of Proteasome subunit alpha type-1

Match to Query 398: 815.515368 from(408.764960,2+)
Title: 091224LimSK_Exosome3_06.1657.1657.2.dta
Data file C:\Temp\Mascoti\LSK_2D\091224LimSK_Exosome3_01_2D.mgf

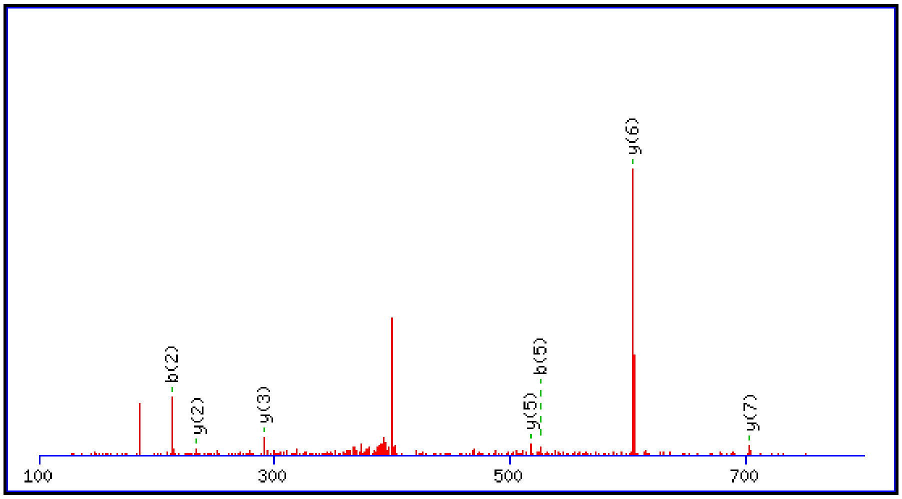

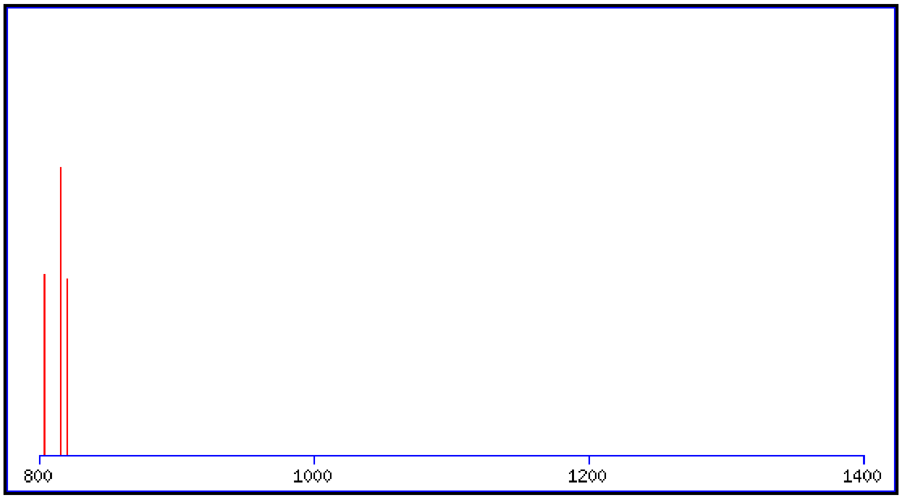

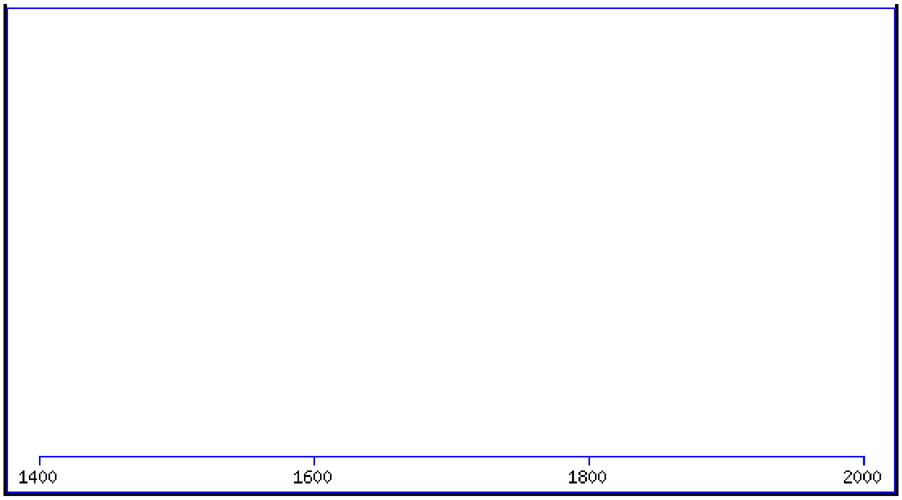

Monoisotopic mass of neutral peptide Mr(calc): 815.5116
Fixed modifications: Carbamidomethyl (C)
Ions Score: 38 **Expect:** 0.0067
Matches (Bold Red): 7/64 fragment ions using 15 most intense peaks

#	b	b^{++}	b^{0}	b^{0++}	Seq.	y	y^{++}	y*	y^{*++}	y^{0}	y^{0++}	#
1	114.0913	57.5493			L							8
2	**213.1598**	107.0835			V	**703.4349**	352.2211	686.4083	343.7078	685.4243	343.2158	7
3	300.1918	150.5995	282.1812	141.5942	S	**604.3665**	302.6869	587.3399	294.1736	586.3559	293.6816	6
4	413.2758	207.1416	395.2653	198.1363	L	**517.3344**	259.1709	500.3079	250.6576	499.3239	250.1656	5
5	**526.3599**	263.6836	508.3493	254.6783	I	404.2504	202.6288	387.2238	194.1155	386.2398	193.6235	4
6	583.3814	292.1943	565.3708	283.1890	G	**291.1663**	146.0868	274.1397	137.5735	273.1557	137.0815	3
7	670.4134	335.7103	652.4028	326.7051	S	**234.1448**	117.5761	217.1183	109.0628	216.1343	108.5708	2
8					K	147.1128	74.0600	130.0863	65.5468			1

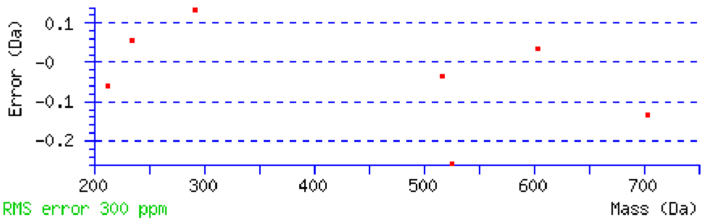

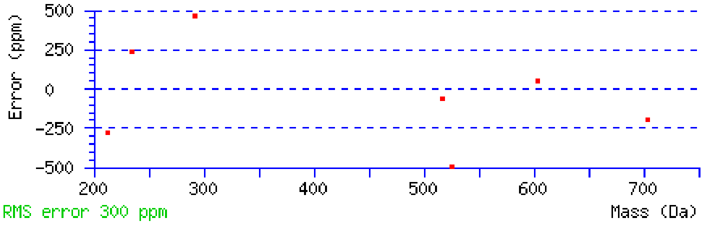

All matches to this query

Score	Mr(calc):	Delta	Sequence
37.7	815.5116	0.0037	LVSLIGSK
18.3	815.5116	0.0037	IVSISLGK
10.4	815.5116	0.0037	VISKLEK
8.2	815.5116	0.0037	IVELSKK
8.2	815.5116	0.0037	IVTVEKK
8.2	815.5116	0.0037	LVVTKEK
7.7	813.5072	2.0082	LPSTLRK
6.4	813.5072	2.0082	AILQKNK
5.3	815.5229	-0.0075	ISLLRSK
3.2	815.5229	-0.0075	LSVLRTK

Spectrum No: 63; Query: 32590; Rank: 1

Peptide View

MS/MS Fragmentation of **NQYDNDVTVWSPQGR**
Found in **IPI00016832**, Tax_Id=9606 Gene_Symbol=PSMA1 Isoform Short of Proteasome subunit alpha type-1

Match to Query 32590: 1777.813028 from(889.913790,2+)
Title: 091224LimSK_Exosome3_07.3191.3191.2.dta
Data file C:\Temp\Mascoti\LSK_2D\091224LimSK_Exosome3_01_2D.mgf

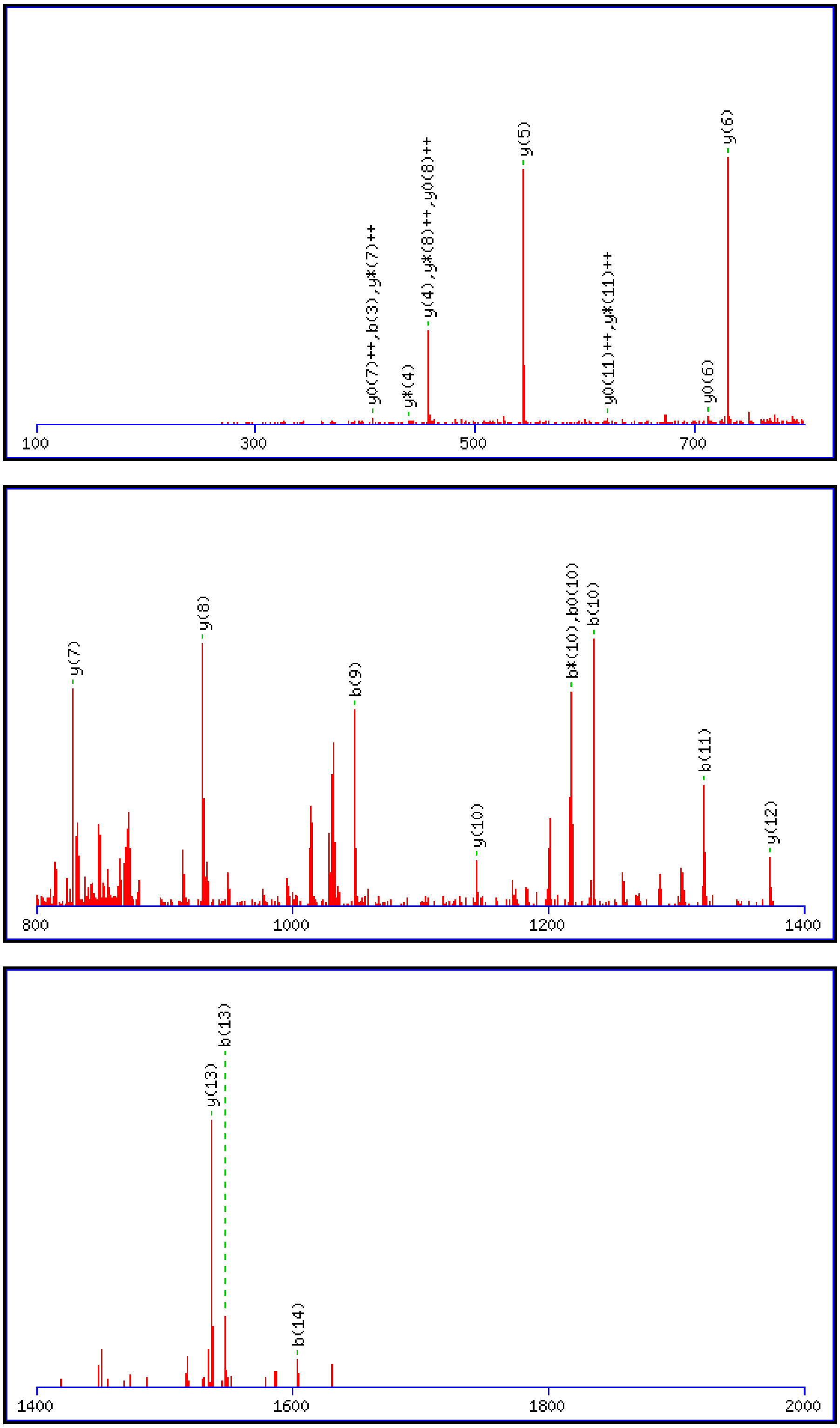

Monoisotopic mass of neutral peptide Mr(calc): 1777.8020
Fixed modifications: Carbamidomethyl (C)
Ions Score: 72 **Expect:** 1.4e-005
Matches (Bold Red): 24/154 fragment ions using 37 most intense peaks

		++		++	0	0++			++		++	0	0++	

#	b	b	b*	b*	b	b	Seq.	y	y	y*	y*	y	y	#
1	115.0502	58.0287	98.0237	49.5155			N							15
2	243.1088	122.0580	226.0822	113.5448			Q	1664.7663	832.8868	1647.7398	824.3735	1646.7558	823.8815	14
3	**406.1721**	203.5897	389.1456	195.0764			Y	**1536.7077**	768.8575	1519.6812	760.3442	1518.6972	759.8522	13
4	521.1991	261.1032	504.1725	252.5899	503.1885	252.0979	D	**1373.6444**	687.3258	1356.6179	678.8126	1355.6339	678.3206	12
5	635.2420	318.1246	618.2154	309.6114	617.2314	309.1193	N	1258.6175	629.8124	1241.5909	**621.2991**	1240.6069	**620.8071**	11
6	750.2689	375.6381	733.2424	367.1248	732.2584	366.6328	D	**1144.5745**	572.7909	1127.5480	564.2776	1126.5640	563.7856	10
7	849.3373	425.1723	832.3108	416.6590	831.3268	416.1670	V	1029.5476	515.2774	1012.5211	506.7642	1011.5370	506.2722	9
8	950.3850	475.6961	933.3585	467.1829	932.3745	466.6909	T	**930.4792**	465.7432	913.4526	**457.2300**	912.4686	**456.7380**	8
9	**1049.4534**	525.2304	1032.4269	516.7171	1031.4429	516.2251	V	**829.4315**	415.2194	812.4050	**406.7061**	811.4209	**406.2141**	7
10	**1235.5327**	618.2700	**1218.5062**	609.7567	**1217.5222**	609.2647	W	**730.3631**	365.6852	713.3366	357.1719	**712.3525**	356.6799	6
11	**1322.5648**	661.7860	1305.5382	653.2727	1304.5542	652.7807	S	**544.2838**	272.6455	527.2572	264.1323	526.2732	263.6402	5
12	1419.6175	710.3124	1402.5910	701.7991	1401.6070	701.3071	P	**457.2518**	229.1295	**440.2252**	220.6162			4
13	**1547.6761**	774.3417	1530.6496	765.8284	1529.6655	765.3364	Q	360.1990	180.6031	343.1724	172.0899			3
14	**1604.6976**	802.8524	1587.6710	794.3392	1586.6870	793.8471	G	232.1404	116.5738	215.1139	108.0606			2
15							R	175.1190	88.0631	158.0924	79.5498			1

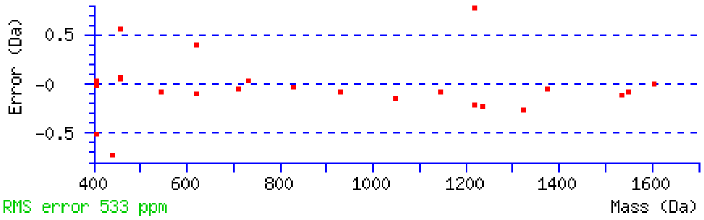

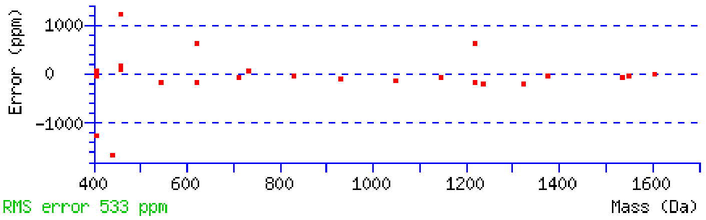

All matches to this query

Score	Mr(calc):	Delta	Sequence
72.5	1777.8020	0.0110	NQYDNDVTVWSPQGR
9.4	1775.7909	2.0222	XLKMETPGMTTPSLK
6.6	1777.8221	-0.0091	VPERGVGNSGDSVRGGR
5.0	1775.8239	1.9891	XLKMETPGMTTPSLK
5.0	1775.7909	2.0222	XLKMETPGMTTPSLK
4.5	1776.8068	1.0062	MKSLPCLLLSAQTR
3.9	1775.7930	2.0200	SGSSPLGVVTKAASMPK
3.7	1777.8294	-0.0164	MDLAGARPRPGLSEGR
3.6	1776.8043	1.0087	SIHEQDTNVNNSVLK
3.5	1775.7930	2.0200	SGSSPLGVVTKAASMPK

Spectrum No: 64; Query: 1100; Rank: 1

Peptide View

MS/MS Fragmentation of **NVSIGIVGK**
Found in **IPI00016832**, Tax_Id=9606 Gene_Symbol=PSMA1 Isoform Short of Proteasome subunit alpha type-1

Match to Query 1100: 885.532028 from(443.773290,2+)
Title: 091224LimSK_Exosome3_06.1634.1634.2.dta
Data file C:\Temp\Mascoti\LSK_2D\091224LimSK_Exosome3_01_2D.mgf

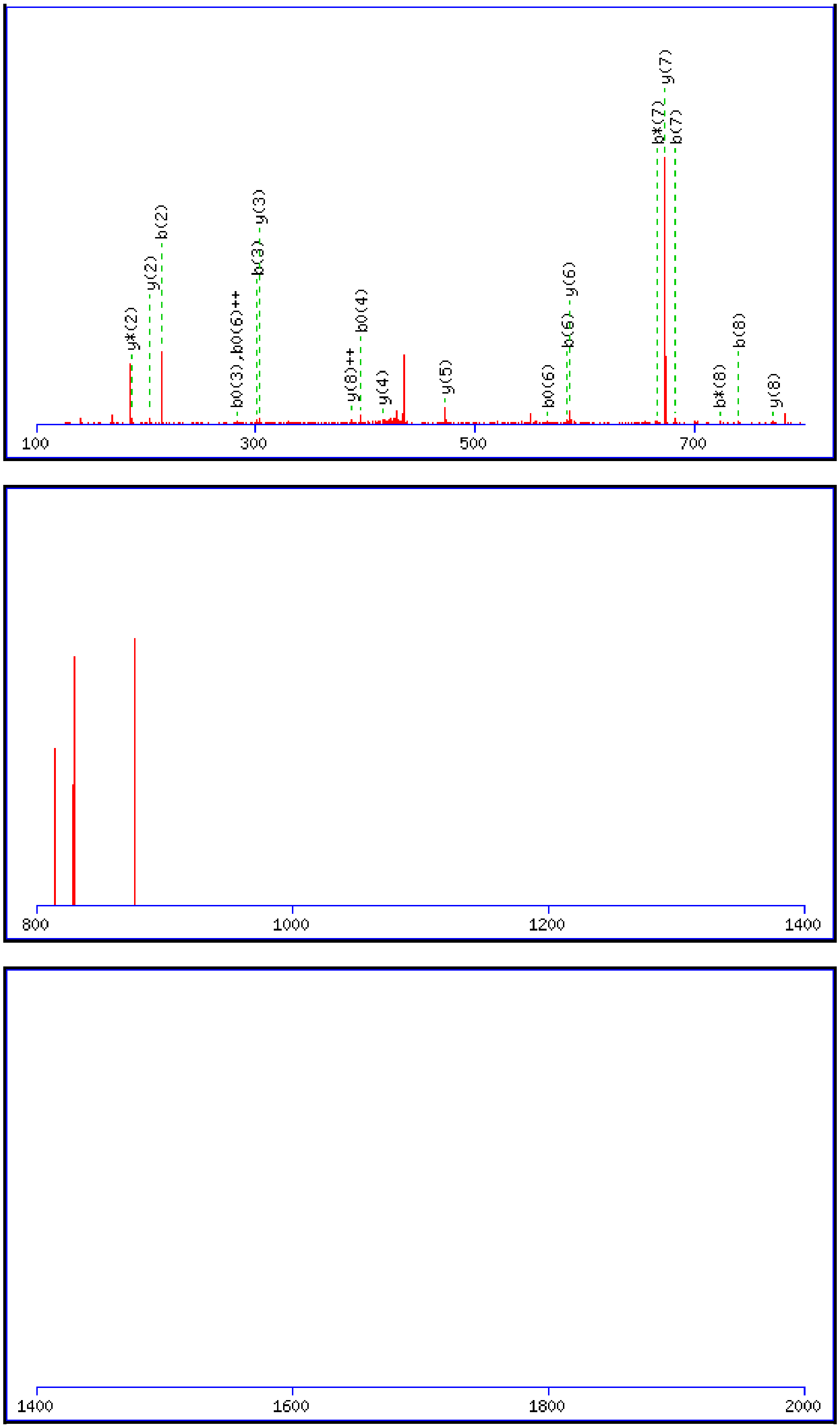

Monoisotopic mass of neutral peptide Mr(calc): 885.5284
Fixed modifications: Carbamidomethyl (C)
Ions Score: 45 **Expect:** 0.0027
Matches (Bold Red): 20/80 fragment ions using 45 most intense peaks

#	b	b^{++}	b*	b^{*++}	b^0	b^{0++}	Seq.	y	y^{++}	y*	y^{*++}	y^0	y^{0++}	#
1	115.0502	58.0287	98.0237	49.5155			N							9
2	**214.1186**	107.5629	197.0921	99.0497			V	**772.4927**	**386.7500**	755.4662	378.2367	754.4822	377.7447	8
3	**301.1506**	151.0790	284.1241	142.5657	**283.1401**	142.0737	S	**673.4243**	337.2158	656.3978	328.7025	655.4137	328.2105	7
4	414.2347	207.6210	397.2082	199.1077	**396.2241**	198.6157	I	**586.3923**	293.6998	569.3657	285.1865			6
5	471.2562	236.1317	454.2296	227.6185	453.2456	227.1264	G	**473.3082**	237.1577	456.2817	228.6445			5
6	**584.3402**	292.6738	567.3137	284.1605	**566.3297**	**283.6685**	I	**416.2867**	208.6470	399.2602	200.1337			4

**7**	**683.4087**	342.2080	**666.3821**	333.6947	665.3981	333.2027	**V**	**303.2027**	152.1050	286.1761	143.5917			**3**
**8**	**740.4301**	370.7187	**723.4036**	362.2054	722.4195	361.7134	**G**	**204.1343**	102.5708	**187.1077**	94.0575			**2**
**9**							**K**	147.1128	74.0600	130.0863	65.5468			**1**

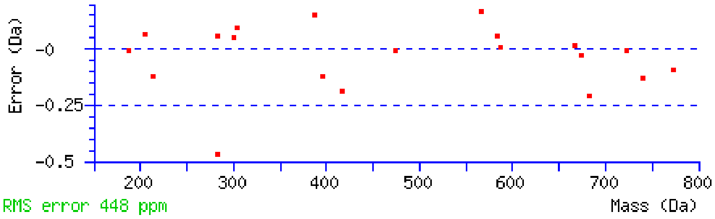

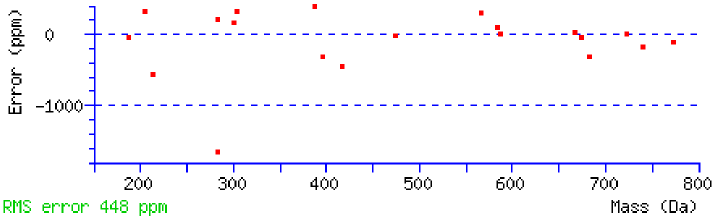

All matches to this query

Score	Mr(calc):	Delta	Sequence
44.6	885.5284	0.0037	NVSIGIVGK
10.7	884.5232	1.0088	WLLGIAGR
7.3	885.5283	0.0037	DALQKALK
6.5	885.5284	0.0037	LVIQTQGK
6.0	885.5284	0.0037	GELTVVLR
4.9	884.5232	1.0088	WLLGQLR
3.3	884.5232	1.0088	VLWLAQR
2.9	885.5297	0.0024	WLRGARK
2.9	885.5297	0.0024	WRQLRK
2.3	885.5396	-0.0076	GVGKSAIVR

Spectrum No: 65; Query: 1975; Rank: 1

Peptide View

MS/MS Fragmentation of **THAVLVALK**
Found in **IPI00016832**, Tax_Id=9606 Gene_Symbol=PSMA1 Isoform Short of Proteasome subunit alpha type-1

Match to Query 1975: 950.595808 from(476.305180,2+)
Title: 091224LimSK_Exosome3_06.1003.1003.2.dta
Data file C:\Temp\Mascoti\LSK_2D\091224LimSK_Exosome3_01_2D.mgf

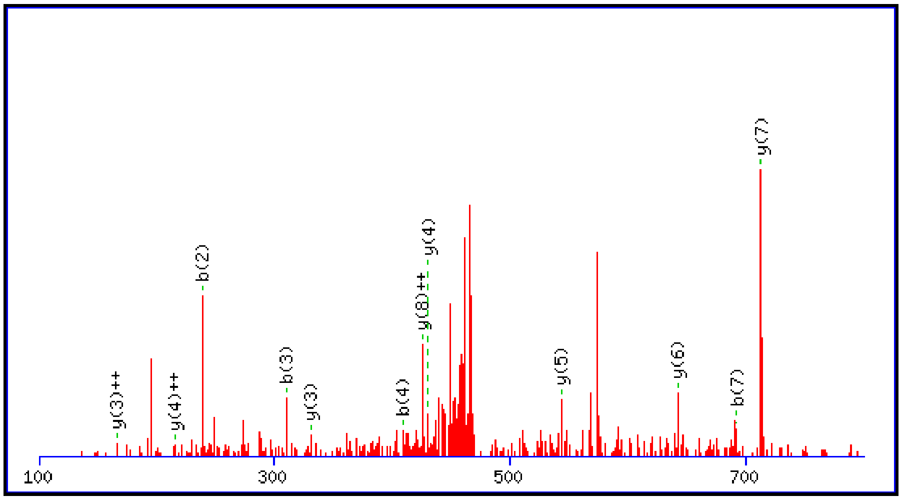

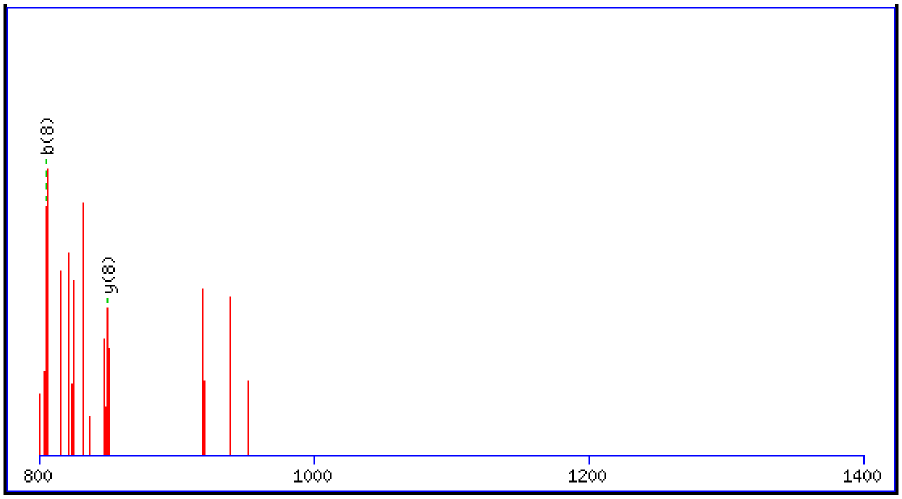

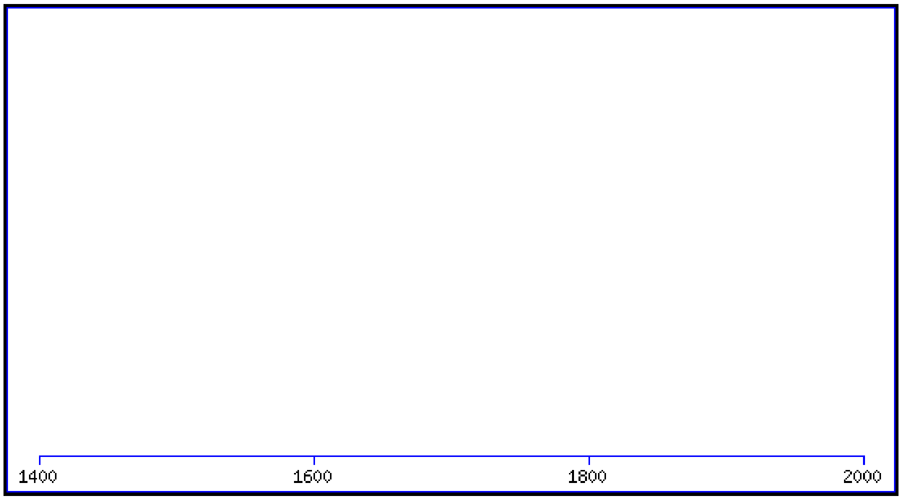

Monoisotopic mass of neutral peptide Mr(calc): 950.5913
Fixed modifications: Carbamidomethyl (C)
Ions Score: 23 **Expect:** 0.062
Matches (Bold Red): 14/64 fragment ions using 63 most intense peaks

#	b	b++	b0	b0++	Seq.	y	y++	y*	y*++	#
1	102.0550	51.5311	84.0444	42.5258	T					9
2	**239.1139**	120.0606	221.1033	111.0553	H	**850.5509**	**425.7791**	833.5244	417.2658	8
3	**310.1510**	155.5791	292.1404	146.5738	A	**713.4920**	357.2496	696.4654	348.7364	7
4	**409.2194**	205.1133	391.2088	196.1081	V	**642.4549**	321.7311	625.4283	313.2178	6
5	522.3035	261.6554	504.2929	252.6501	L	**543.3865**	272.1969	526.3599	263.6836	5
6	621.3719	311.1896	603.3613	302.1843	V	**430.3024**	**215.6548**	413.2758	207.1416	4
7	**692.4090**	346.7081	674.3984	337.7028	A	**331.2340**	**166.1206**	314.2074	157.6074	3
8	**805.4931**	403.2502	787.4825	394.2449	L	260.1969	130.6021	243.1703	122.0888	2
9					K	147.1128	74.0600	130.0863	65.5468	1

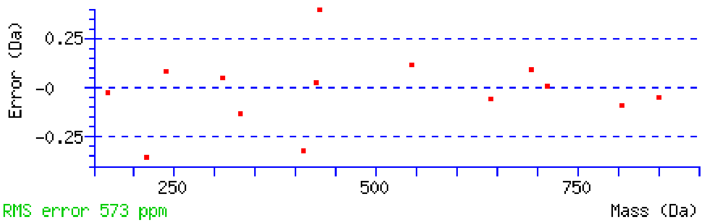

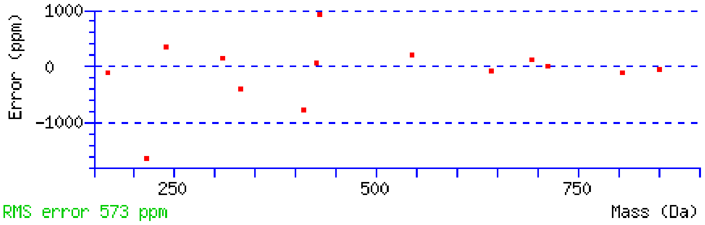

All matches to this query

Score	Mr(calc):	Delta	Sequence

22.6	950.5913	0.0045	THAVLVALK

Spectrum No: 66; Query: 4930; Rank: 1

Peptide View

MS/MS Fragmentation of **THAVLVALKR**
Found in **IPI00016832**, Tax_Id=9606 Gene_Symbol=PSMA1 Isoform Short of Proteasome subunit alpha type-1

Match to Query 4930: 1106.697102 from(369.906310,3+)
Title: 091224LimSK_Exosome3_05.720.720.3.dta
Data file C:\Temp\Mascoti\LSK_2D\091224LimSK_Exosome3_01_2D.mgf

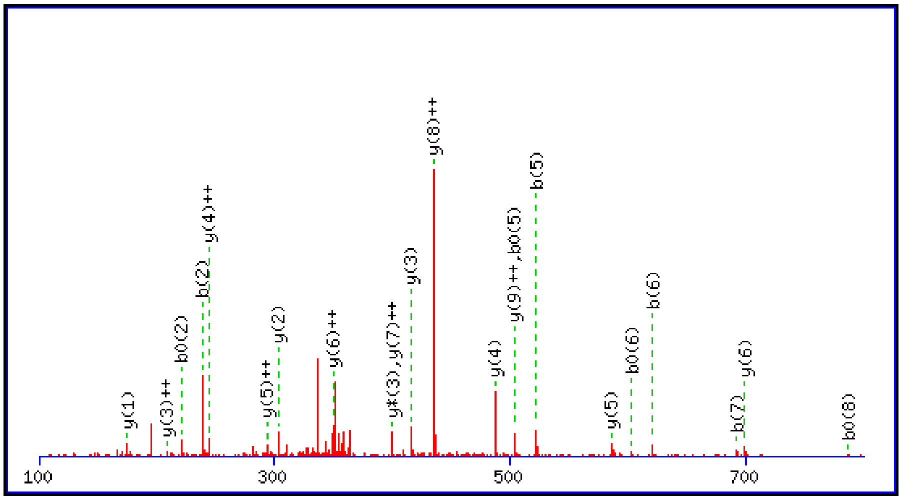

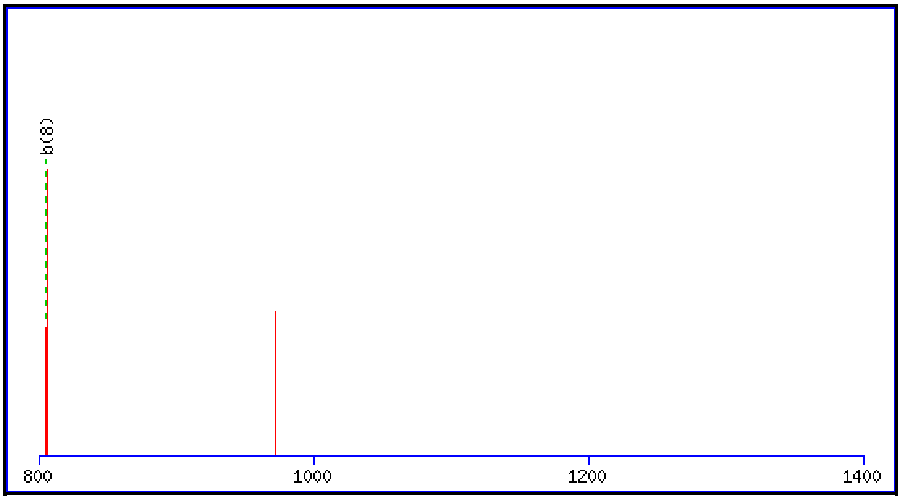

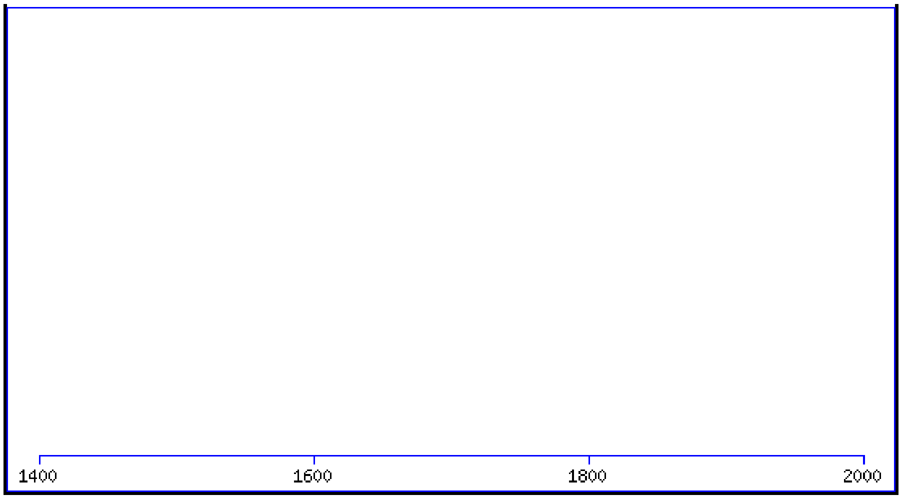

Monoisotopic mass of neutral peptide Mr(calc): 1106.6924
Fixed modifications: Carbamidomethyl (C)
Ions Score: 43 **Expect:** 0.0004
Matches (Bold Red): 23/74 fragment ions using 44 most intense peaks

#	b	b^{++}	b*	b^{*++}	b^{0}	b^{0++}	Seq.	y	y^{++}	y*	y^{*++}	#
1	102.0550	51.5311			84.0444	42.5258	T					10
2	**239.1139**	120.0606			**221.1033**	111.0553	H	1006.6520	**503.8296**	989.6255	495.3164	9
3	310.1510	155.5791			292.1404	146.5738	A	869.5931	**435.3002**	852.5665	426.7869	8
4	409.2194	205.1133			391.2088	196.1081	V	798.5560	**399.7816**	781.5294	391.2684	7
5	**522.3035**	261.6554			**504.2929**	252.6501	L	**699.4876**	**350.2474**	682.4610	341.7341	6
6	**621.3719**	311.1896			**603.3613**	302.1843	V	**586.4035**	**293.7054**	569.3770	285.1921	5
7	**692.4090**	346.7081			674.3984	337.7028	A	**487.3351**	**244.1712**	470.3085	235.6579	4
8	**805.4931**	403.2502			**787.4825**	394.2449	L	**416.2980**	**208.6526**	**399.2714**	200.1394	3
9	933.5880	467.2976	916.5615	458.7844	915.5774	458.2924	K	**303.2139**	152.1106	286.1874	143.5973	2
10							R	**175.1190**	88.0631	158.0924	79.5498	1

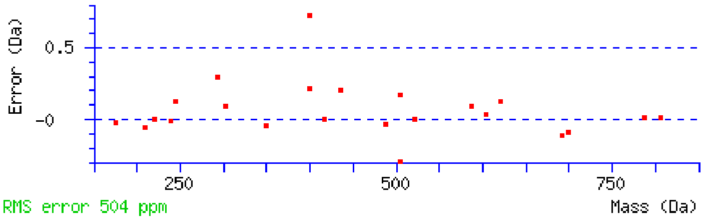

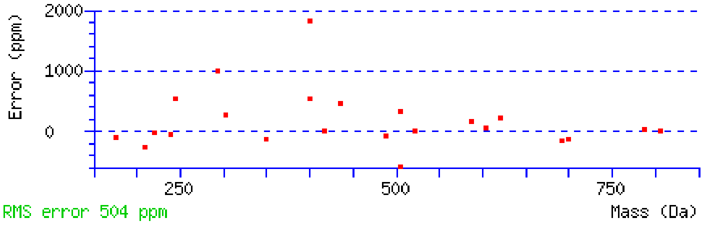

All matches to this query

Score	Mr(calc):	Delta	Sequence
43.5	1106.6924	0.0047	THAVLVALKR
4.8	1105.6859	1.0112	IYLSNLKKK
1.9	1105.6859	1.0112	ALHLLAVLEK

Spectrum No: 67; Query: 33737; Rank: 1

Peptide View

MS/MS Fragmentation of **DAVSGMGVIVHIIEKDK**
Found in **IPI00028004**, Tax_Id=9606 Gene_Symbol=PSMB3 Proteasome subunit beta type-3

Match to Query 33737: 1810.975902 from(604.665910,3+)
Title: 091224LimSK_Exosome3_06.7253.7253.3.dta

Data file C:\Temp\Mascoti\LSK_2D\091224LimSK_Exosome3_01_2D.mgf

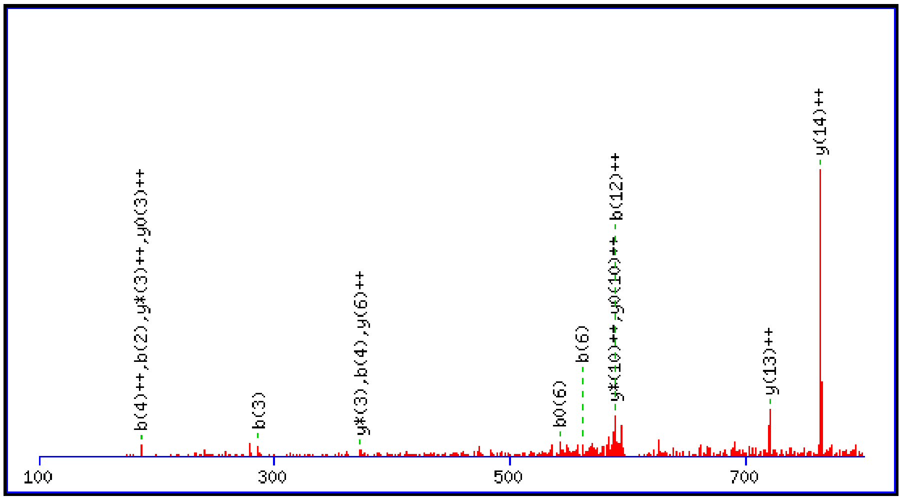

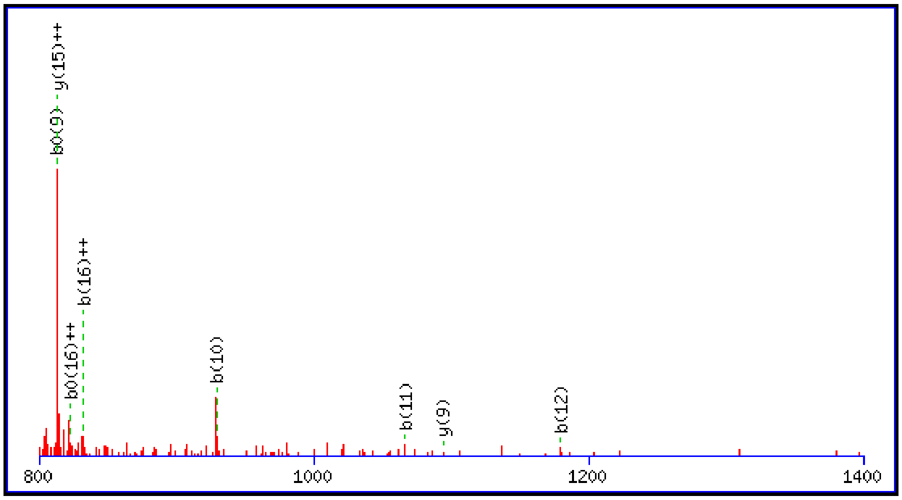

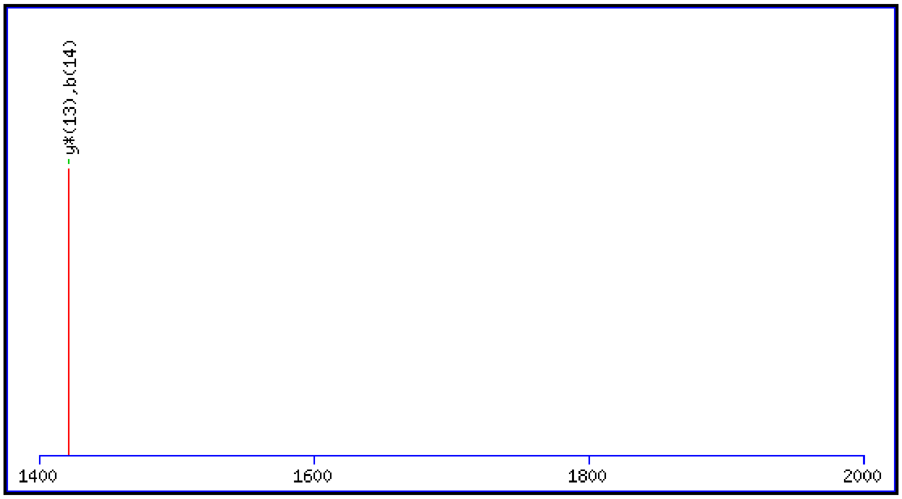

Monoisotopic mass of neutral peptide Mr(calc): 1809.9659
Fixed modifications: Carbamidomethyl (C)
Ions Score: 25 **Expect:** 0.29
Matches (Bold Red): 25/162 fragment ions using 58 most intense peaks

#	b	b^{++}	b*	b^{*++}	b^0	b^{0++}	Seq.	y	y^{++}	y*	y^{*++}	y^0	y^{0++}	#
1	116.0342	58.5207			98.0237	49.5155	D							17
2	**187.0713**	94.0393			169.0608	85.0340	A	1695.9462	848.4767	1678.9197	839.9635	1677.9356	839.4715	16
3	**286.1397**	143.5735			268.1292	134.5682	V	1624.9091	**812.9582**	1607.8825	804.4449	1606.8985	803.9529	15
4	**373.1718**	**187.0895**			355.1612	178.0842	S	1525.8407	**763.4240**	1508.8141	754.9107	1507.8301	754.4187	14
5	430.1932	215.6003			412.1827	206.5950	G	1438.8086	**719.9080**	**1421.7821**	711.3947	1420.7981	710.9027	13

6	**561.2337**	281.1205			**543.2232**	272.1152	M	1381.7872	691.3972	1364.7606	682.8840	1363.7766	682.3919	12
7	618.2552	309.6312			600.2446	300.6259	G	1250.7467	625.8770	1233.7201	617.3637	1232.7361	616.8717	11
8	717.3236	359.1654			699.3130	350.1602	V	1193.7252	597.3663	1176.6987	**588.8530**	1175.7147	**588.3610**	10
9	830.4077	415.7075			**812.3971**	406.7022	I	**1094.6568**	547.8320	1077.6303	539.3188	1076.6463	538.8268	9
10	**929.4761**	465.2417			911.4655	456.2364	V	981.5728	491.2900	964.5462	482.7767	963.5622	482.2847	8
11	**1066.5350**	533.7711			1048.5244	524.7659	H	882.5043	441.7558	865.4778	433.2425	864.4938	432.7505	7
12	**1179.6191**	**590.3132**			1161.6085	581.3079	I	745.4454	**373.2264**	728.4189	364.7131	727.4349	364.2211	6
13	1292.7031	646.8552			1274.6926	637.8499	I	632.3614	316.6843	615.3348	308.1710	614.3508	307.6790	5
14	**1421.7457**	711.3765			1403.7351	702.3712	E	519.2773	260.1423	502.2508	251.6290	501.2667	251.1370	4
15	1549.8407	775.4240	1532.8141	766.9107	1531.8301	766.4187	K	390.2347	195.6210	**373.2082**	**187.1077**	372.2241	**186.6157**	3
16	1664.8676	**832.9374**	1647.8411	824.4242	1646.8571	**823.9322**	D	262.1397	131.5735	245.1132	123.0602	244.1292	122.5682	2
17							K	147.1128	74.0600	130.0863	65.5468			1

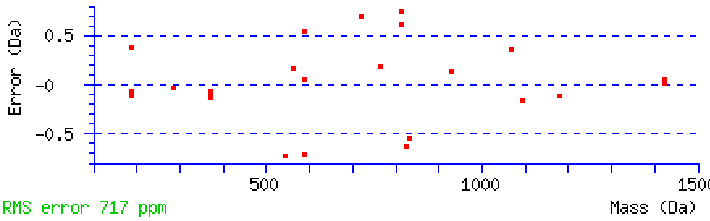

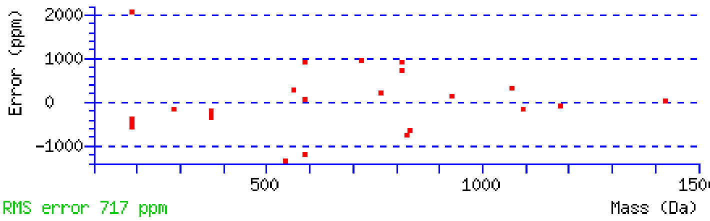

All matches to this query

Score	Mr(calc):	Delta	Sequence
25.1	1809.9659	1.0100	DAVSGMGVIVHIIEKDK
9.2	1810.9690	0.0069	WVVDLDRGLVLNAEGR
7.2	1810.9649	0.0110	EALGKAKDANNGNLQLR
5.0	1810.9703	0.0056	XVFSNHALRRLQER
2.6	1810.9876	-0.0117	LGFNVTLSLFMRNKR
2.1	1810.9764	-0.0005	HSFSVSSCVIPLPIIR
1.7	1808.9858	1.9901	ALQQFLYVQMAKELK
0.6	1809.9697	1.0062	QGVSPKPLQSSRPSQSK
0.2	1810.9901	-0.0142	EGGLKLIQIQDNGTGIR

Spectrum No: 68; Query: 26203; Rank: 1

Peptide View

MS/MS Fragmentation of **FGIQAQMVTTDFQK**
Found in **IPI00028004**, Tax_Id=9606 Gene_Symbol=PSMB3 Proteasome subunit beta type-3

Match to Query 26203: 1612.803968 from(807.409260,2+)
Title: 091224LimSK_Exosome3_06.4983.4983.2.dta
Data file C:\Temp\Mascoti\LSK_2D\091224LimSK_Exosome3_01_2D.mgf

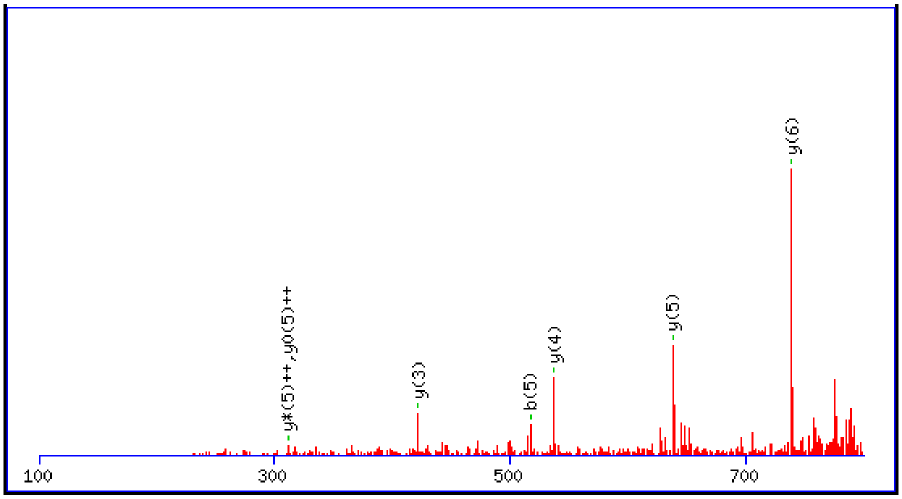

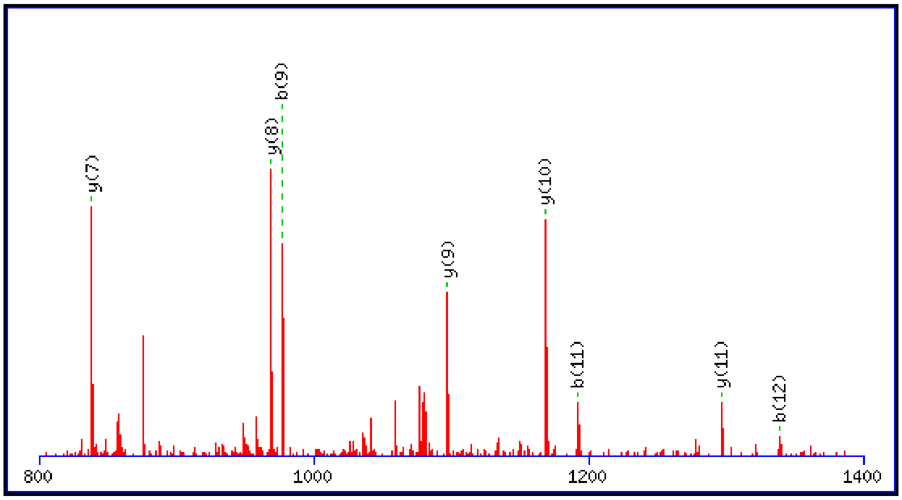

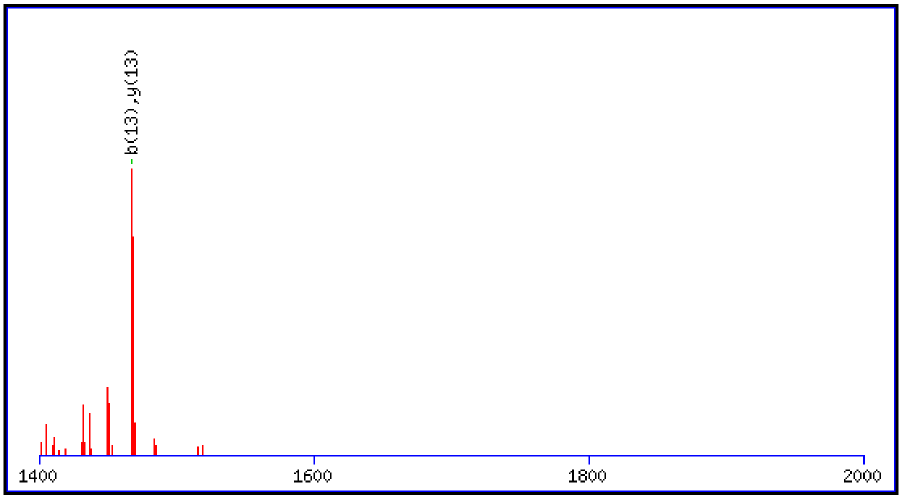

Monoisotopic mass of neutral peptide Mr(calc): 1612.7919
Fixed modifications: Carbamidomethyl (C)
Ions Score: 107 **Expect:** 3.6e-009
Matches (Bold Red): 17/128 fragment ions using 17 most intense peaks

#	b	b^{++}	b*	b^{*++}	b^0	b^{0++}	Seq.	y	y^{++}	y*	y^{*++}	y^0	y^{0++}	#
1	148.0757	74.5415					F							14
2	205.0972	103.0522					G	**1466.7308**	733.8690	1449.7042	725.3558	1448.7202	724.8638	13
3	318.1812	159.5942					I	1409.7093	705.3583	1392.6828	696.8450	1391.6988	696.3530	12
4	446.2398	223.6235	429.2132	215.1103			Q	**1296.6253**	648.8163	1279.5987	640.3030	1278.6147	639.8110	11
5	**517.2769**	259.1421	500.2504	250.6288			A	**1168.5667**	584.7870	1151.5401	576.2737	1150.5561	575.7817	10
6	645.3355	323.1714	628.3089	314.6581			Q	**1097.5296**	549.2684	1080.5030	540.7552	1079.5190	540.2631	9

**7**	776.3760	388.6916	759.3494	380.1784			**M**	**969.4710**	485.2391	952.4444	476.7259	951.4604	476.2339	**8**
**8**	875.4444	438.2258	858.4178	429.7126			**V**	**838.4305**	419.7189	821.4040	411.2056	820.4199	410.7136	**7**
**9**	**976.4921**	488.7497	959.4655	480.2364	958.4815	479.7444	**T**	**739.3621**	370.1847	722.3355	361.6714	721.3515	361.1794	**6**
**10**	1077.5397	539.2735	1060.5132	530.7602	1059.5292	530.2682	**T**	**638.3144**	319.6608	621.2879	**311.1476**	620.3039	**310.6556**	**5**
**11**	**1192.5667**	596.7870	1175.5401	588.2737	1174.5561	587.7817	**D**	**537.2667**	269.1370	520.2402	260.6237	519.2562	260.1317	**4**
**12**	**1339.6351**	670.3212	1322.6086	661.8079	1321.6245	661.3159	**F**	**422.2398**	211.6235	405.2132	203.1103			**3**
**13**	**1467.6937**	734.3505	1450.6671	725.8372	1449.6831	725.3452	**Q**	275.1714	138.0893	258.1448	129.5761			**2**
**14**							**K**	147.1128	74.0600	130.0863	65.5468			**1**

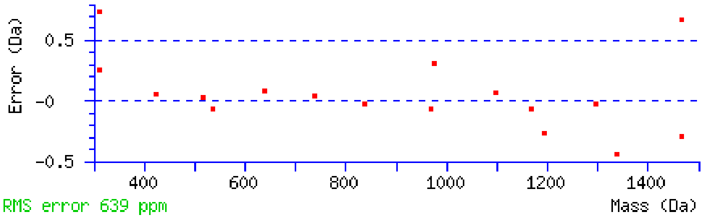

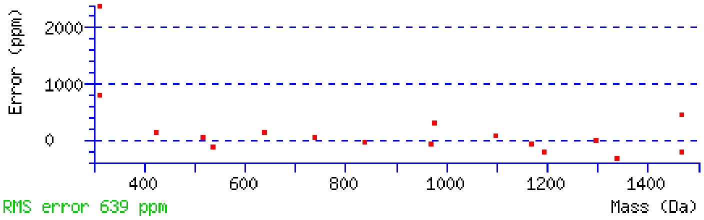

All matches to this query

Score	Mr(calc):	Delta	Sequence
107.2	1612.7919	0.0120	FGIQAQMVTTDFQK
8.2	1610.7851	2.0189	CNTNTAIELKLVR
6.4	1612.8178	-0.0138	MATGCTLPQLPRPR
6.1	1612.7902	0.0138	IYYTGKYQSLGIK
5.9	1610.7851	2.0189	CNTNTAIELKLVR
5.9	1612.7902	0.0138	IYYTGKYQSLGIK
5.8	1612.8056	-0.0017	THSPSSKDEQSIGLK
4.9	1612.7902	0.0138	IYYTGKYQSLGIK
4.2	1612.7902	0.0138	IYYTGKYQSLGIK
3.8	1612.8130	-0.0091	MSPALQDLSQPEGLK

Spectrum No: 69; Query: 32090; Rank: 1

Peptide View

MS/MS Fragmentation of **FGPYYTEPVIAGLDPK**
Found in **IPI00028004**, Tax_Id=9606 Gene_Symbol=PSMB3 Proteasome subunit beta type-3

Match to Query 32090: 1765.904368 from(883.959460,2+)
Title: 091224LimSK_Exosome3_07.6121.6121.2.dta
Data file C:\Temp\Mascoti\LSK_2D\091224LimSK_Exosome3_01_2D.mgf

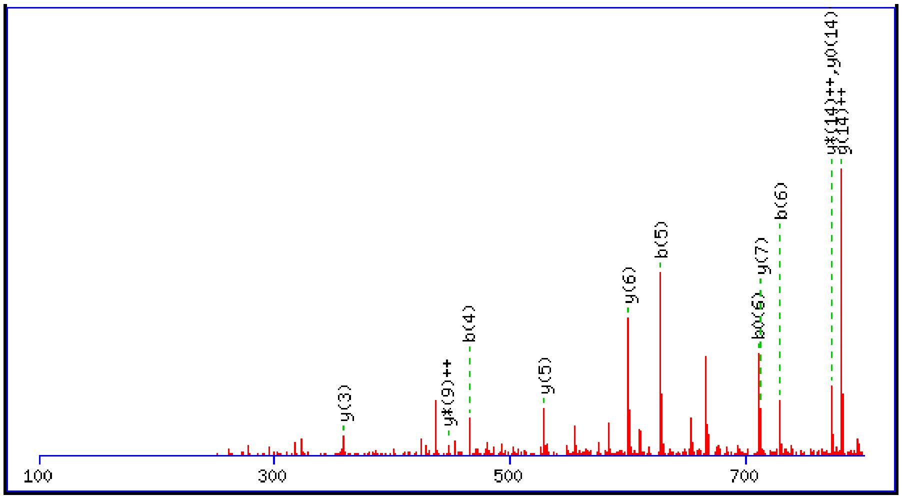

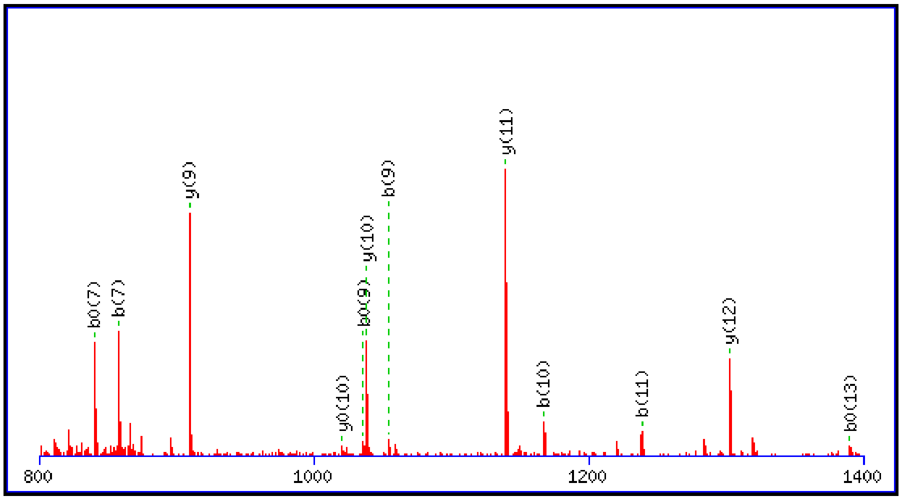

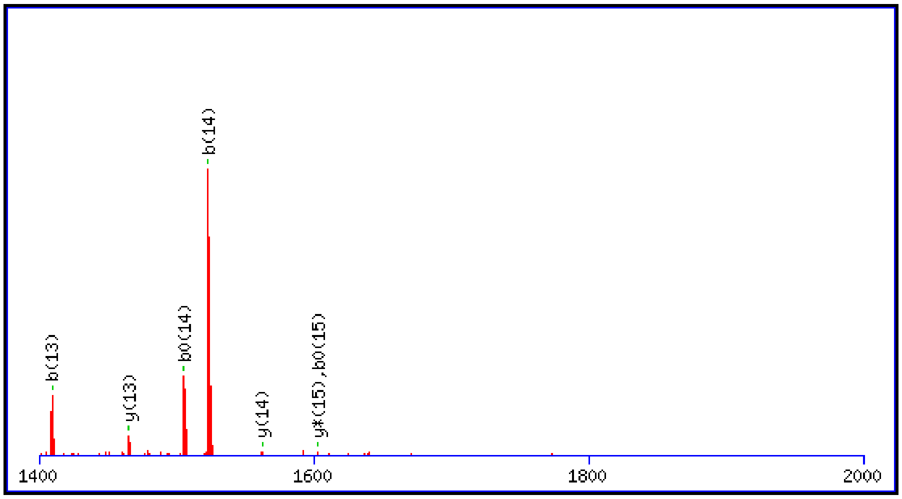

Monoisotopic mass of neutral peptide Mr(calc): 1765.8927
Fixed modifications: Carbamidomethyl (C)
Ions Score: 47 **Expect:** 0.0035
Matches (Bold Red): 31/136 fragment ions using 67 most intense peaks

#	b	b^{++}	b^{0}	b^{0++}	Seq.	y	y^{++}	y*	y*$^{++}$	y^{0}	y^{0++}	#
**1**	148.0757	74.5415			**F**							**16**
**2**	205.0972	103.0522			**G**	1619.8316	810.4194	**1602.8050**	801.9061	1601.8210	801.4141	**15**
**3**	302.1499	151.5786			**P**	**1562.8101**	**781.9087**	1545.7835	**773.3954**	1544.7995	**772.9034**	**14**
**4**	**465.2132**	233.1103			**Y**	**1465.7573**	733.3823	1448.7308	724.8690	1447.7468	724.3770	**13**
**5**	**628.2766**	314.6419			**Y**	**1302.6940**	651.8506	1285.6674	643.3374	1284.6834	642.8454	**12**
**6**	**729.3243**	365.1658	**711.3137**	356.1605	**T**	**1139.6307**	570.3190	1122.6041	561.8057	1121.6201	561.3137	**11**

**7**	**858.3668**	429.6871	**840.3563**	420.6818	**E**	**1038.5830**	519.7951	1021.5564	511.2819	**1020.5724**	510.7898	**10**
**8**	955.4196	478.2134	937.4090	469.2082	**P**	**909.5404**	455.2738	892.5138	**446.7606**	891.5298	446.2686	**9**
**9**	**1054.4880**	527.7477	**1036.4775**	518.7424	**V**	812.4876	406.7475	795.4611	398.2342	794.4771	397.7422	**8**
**10**	**1167.5721**	584.2897	1149.5615	575.2844	**I**	**713.4192**	357.2132	696.3927	348.7000	695.4087	348.2080	**7**
**11**	**1238.6092**	619.8082	1220.5986	610.8030	**A**	**600.3352**	300.6712	583.3086	292.1579	582.3246	291.6659	**6**
**12**	1295.6307	648.3190	1277.6201	639.3137	**G**	**529.2980**	265.1527	512.2715	256.6394	511.2875	256.1474	**5**
**13**	**1408.7147**	704.8610	**1390.7042**	695.8557	**L**	472.2766	236.6419	455.2500	228.1287	454.2660	227.6366	**4**
**14**	**1523.7417**	762.3745	**1505.7311**	753.3692	**D**	**359.1925**	180.0999	342.1660	171.5866	341.1819	171.0946	**3**
**15**	1620.7944	810.9009	**1602.7839**	801.8956	**P**	244.1656	122.5864	227.1390	114.0731			**2**
**16**					**K**	147.1128	74.0600	130.0863	65.5468			**1**

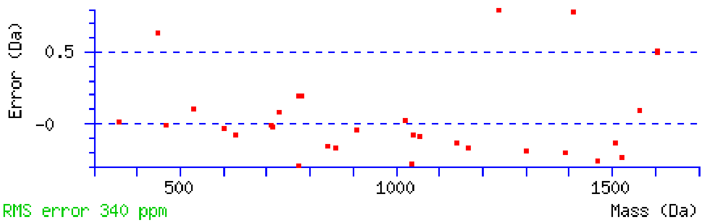

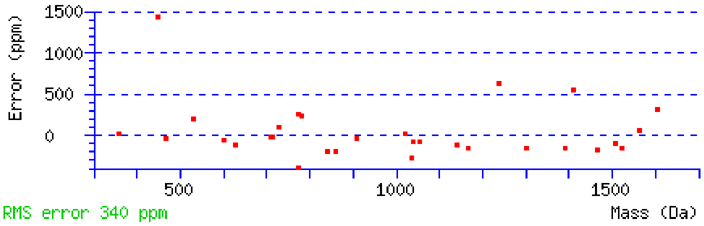

All matches to this query

Score	Mr(calc):	Delta	Sequence
47.0	1765.8927	0.0117	FGPYYTEPVIAGLDPK
16.5	1763.9070	1.9973	SGGGEQLDILSVGILVK
5.6	1765.9161	-0.0117	EILQIMNKTISKPR
5.4	1765.8975	0.0069	IEATKKAESEIQAIR
5.3	1763.9110	1.9934	LQLYKLLEIPDPDK
3.2	1765.8910	0.0134	RAGLLTGLGLTCDALR
2.8	1765.8975	0.0069	IEATKKAESEIQAIR
1.9	1764.9135	0.9909	VSNGLKAIRVEAVSDK
0.3	1764.9135	0.9909	ELRKAIEGSVTAQGVK
0.1	1764.8867	1.0177	DRNGTHLDAGTVEPKR

Spectrum No: 70; Query: 1153; Rank: 1

Peptide View

MS/MS Fragmentation of **LNLYELK**
Found in **IPI00028004**, Tax_Id=9606 Gene_Symbol=PSMB3 Proteasome subunit beta type-3

Match to Query 1153: 891.511088 from(446.762820,2+)
Title: 091224LimSK_Exosome3_07.3190.3190.2.dta
Data file C:\Temp\Mascoti\LSK_2D\091224LimSK_Exosome3_01_2D.mgf

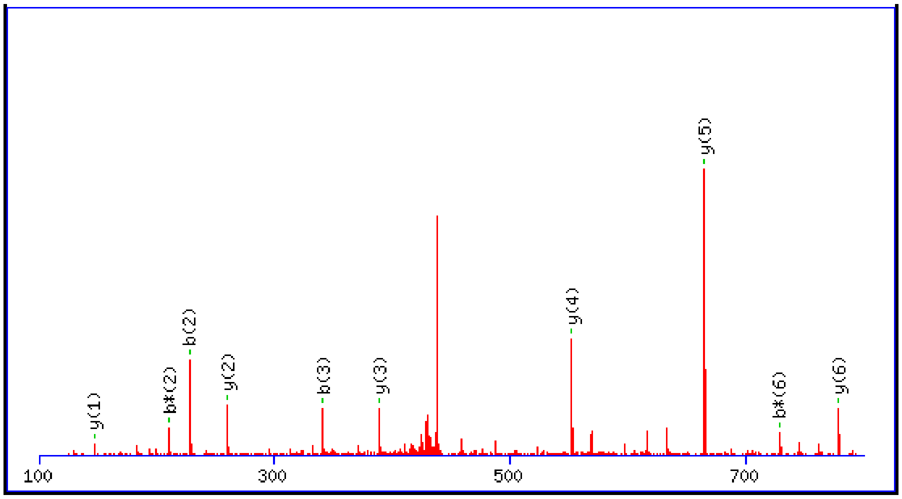

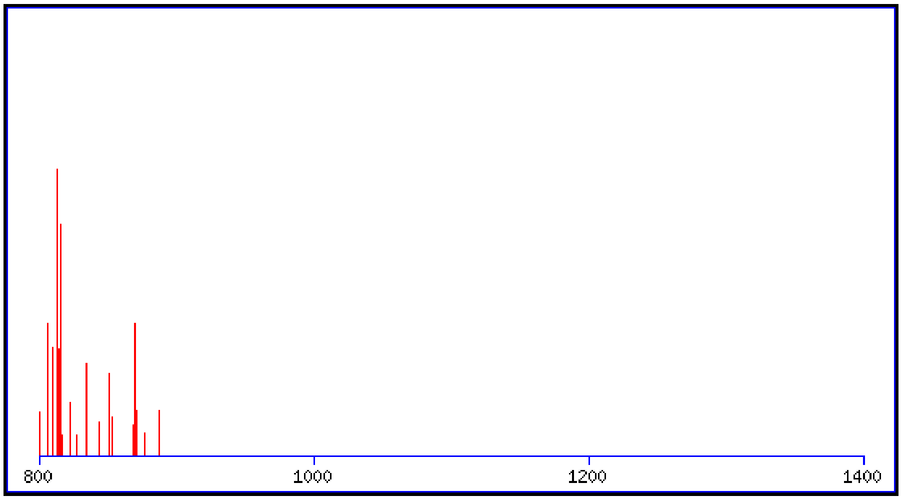

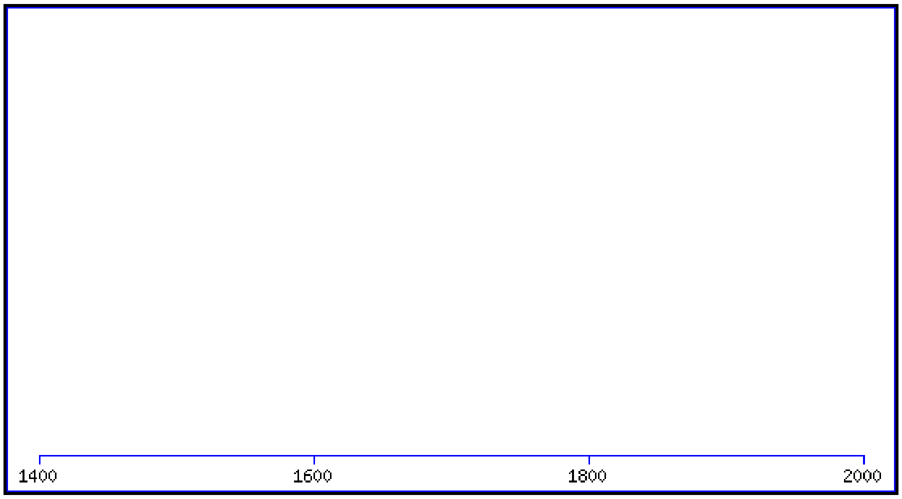

Monoisotopic mass of neutral peptide Mr(calc): 891.5065
Fixed modifications: Carbamidomethyl (C)
Ions Score: 51 **Expect:** 0.00062
Matches (Bold Red): 10/58 fragment ions using 25 most intense peaks

#	b	b^{++}	b*	b^{*++}	b^{0}	b^{0++}	Seq.	y	y^{++}	y*	y^{*++}	y^{0}	y^{0++}	#
1	114.0913	57.5493					L							7
2	**228.1343**	114.5708	**211.1077**	106.0575			N	**779.4298**	390.2185	762.4032	381.7053	761.4192	381.2132	6
3	**341.2183**	171.1128	324.1918	162.5995			L	**665.3869**	333.1971	648.3603	324.6838	647.3763	324.1918	5
4	504.2817	252.6445	487.2551	244.1312			Y	**552.3028**	276.6550	535.2762	268.1418	534.2922	267.6498	4
5	633.3243	317.1658	616.2977	308.6525	615.3137	308.1605	E	**389.2395**	195.1234	372.2129	186.6101	371.2289	186.1181	3
6	746.4083	373.7078	**729.3818**	365.1945	728.3978	364.7025	L	**260.1969**	130.6021	243.1703	122.0888			2

7							K	147.1128	74.0600	130.0863	65.5468			1

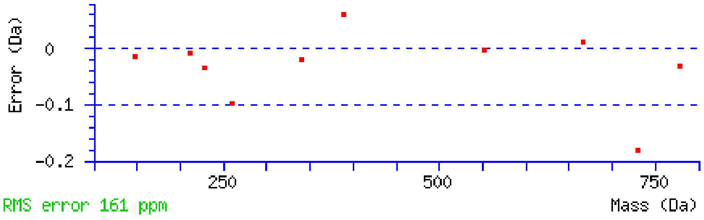

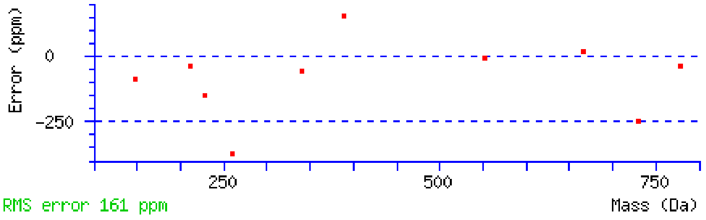

All matches to this query

Score	Mr(calc):	Delta	Sequence
50.9	891.5065	0.0046	LNLYELK
36.6	891.5065	0.0045	VQLYELK
18.5	891.5065	0.0046	NLYLEIK
17.0	891.5065	0.0046	INYLIEK
16.7	891.5178	-0.0067	INVQKYK
16.4	891.5065	0.0046	YNLLELK
15.4	891.5065	0.0045	LVAGEYLK
15.4	891.5065	0.0045	IIDLGYAK
14.8	891.5065	0.0045	IIGDAYLK
10.0	891.5178	-0.0067	QVPNPLPK

Spectrum No: 71; Query: 36128; Rank: 1

Peptide View

MS/MS Fragmentation of **LYIGLAGLATDVQTVAQR**
Found in **IPI00028004**, Tax_Id=9606 Gene_Symbol=PSMB3 Proteasome subunit beta type-3

Match to Query 36128: 1888.053788 from(945.034170,2+)
Title: 091224LimSK_Exosome3_06.7721.7721.2.dta
Data file C:\Temp\Mascoti\LSK_2D\091224LimSK_Exosome3_01_2D.mgf

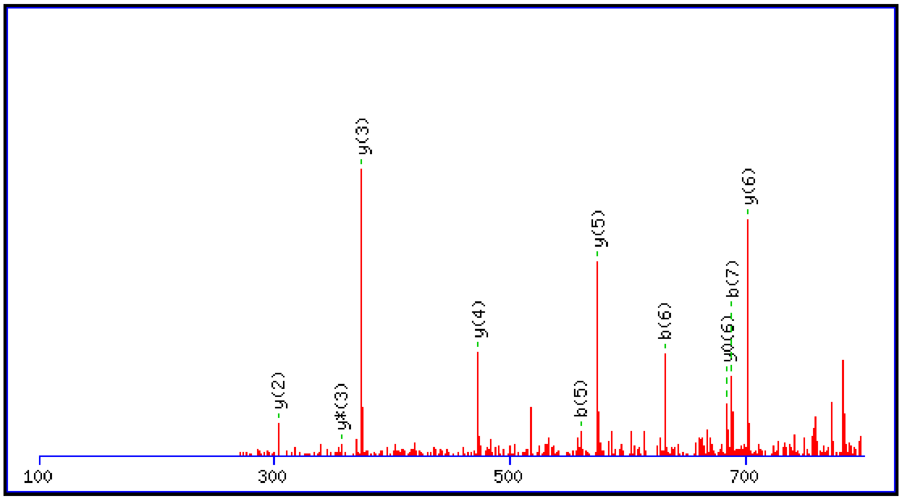

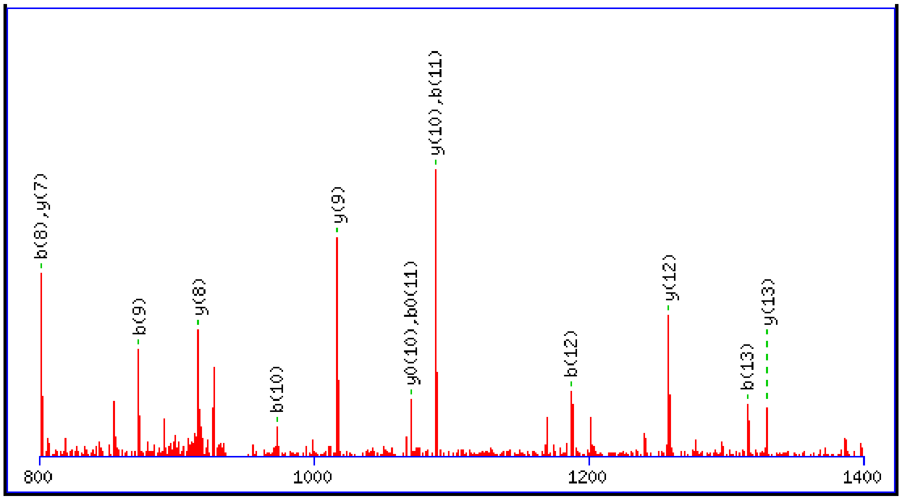

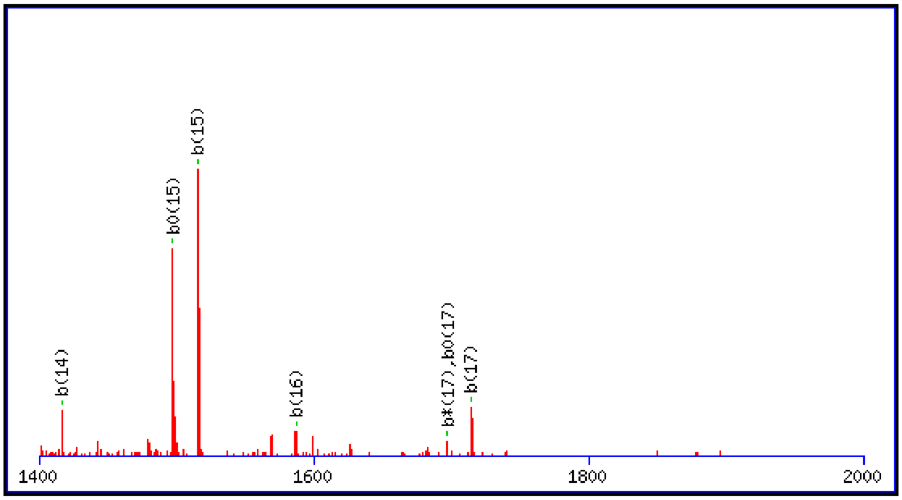

Monoisotopic mass of neutral peptide Mr(calc): 1888.0418
Fixed modifications: Carbamidomethyl (C)
Ions Score: 97 **Expect:** 1.1e-008
Matches (Bold Red): 31/154 fragment ions using 50 most intense peaks

#	b	b^{++}	b*	b^{*++}	b^{0}	b^{0++}	Seq.	y	y^{++}	y*	y^{*++}	y^{0}	y^{0++}	#
1	114.0913	57.5493					L							18
2	277.1547	139.0810					Y	1775.9650	888.4862	1758.9385	879.9729	1757.9545	879.4809	17
3	390.2387	195.6230					I	1612.9017	806.9545	1595.8752	798.4412	1594.8911	797.9492	16
4	447.2602	224.1337					G	1499.8176	750.4125	1482.7911	741.8992	1481.8071	741.4072	15
5	**560.3443**	280.6758					L	1442.7962	721.9017	1425.7696	713.3884	1424.7856	712.8964	14
6	**631.3814**	316.1943					A	**1329.7121**	665.3597	1312.6856	656.8464	1311.7015	656.3544	13
7	**688.4028**	344.7051					G	**1258.6750**	629.8411	1241.6484	621.3279	1240.6644	620.8359	12
8	**801.4869**	401.2471					L	1201.6535	601.3304	1184.6270	592.8171	1183.6430	592.3251	11
9	**872.5240**	436.7656					A	**1088.5695**	544.7884	1071.5429	536.2751	**1070.5589**	535.7831	10
10	**973.5717**	487.2895			955.5611	478.2842	T	**1017.5324**	509.2698	1000.5058	500.7565	999.5218	500.2645	9
11	**1088.5986**	544.8030			**1070.5881**	535.7977	D	**916.4847**	458.7460	899.4581	450.2327	898.4741	449.7407	8
12	**1187.6671**	594.3372			1169.6565	585.3319	V	**801.4577**	401.2325	784.4312	392.7192	783.4472	392.2272	7
13	**1315.7256**	658.3665	1298.6991	649.8532	1297.7151	649.3612	Q	**702.3893**	351.6983	685.3628	343.1850	**684.3787**	342.6930	6
14	**1416.7733**	708.8903	1399.7468	700.3770	1398.7627	699.8850	T	**574.3307**	287.6690	557.3042	279.1557	556.3202	278.6637	5
15	**1515.8417**	758.4245	1498.8152	749.9112	**1497.8312**	749.4192	V	**473.2831**	237.1452	456.2565	228.6319			4
16	**1586.8788**	793.9431	1569.8523	785.4298	1568.8683	784.9378	A	**374.2146**	187.6110	**357.1881**	179.0977			3
17	**1714.9374**	857.9723	**1697.9109**	849.4591	**1696.9269**	848.9671	Q	**303.1775**	152.0924	286.1510	143.5791			2
18							R	175.1190	88.0631	158.0924	79.5498			1

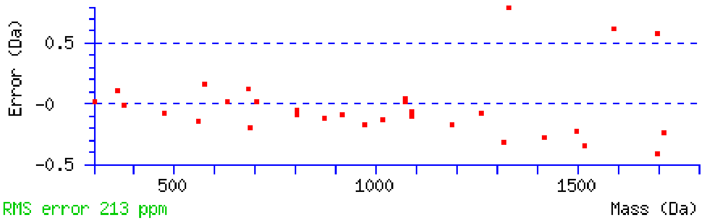

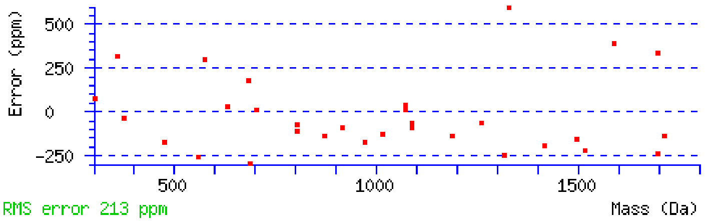

All matches to this query

Score	Mr(calc):	Delta	Sequence
97.0	1888.0418	0.0120	LYIGLAGLATDVQTVAQR
13.1	1886.0560	1.9978	TAPSLVMVLSWKSGVRR
4.8	1886.0414	2.0124	GALLDFLWDLNAAIAKR
4.0	1888.0683	-0.0145	FNFVGKILGPQGNTIKR
3.8	1888.0643	-0.0105	GHGPTVGLLLSRGASPTLR
2.1	1887.0578	0.9960	AKNGTGKSGAYLIPLLER
2.0	1888.0492	0.0046	MLLSLQPFLQDAIQKK
1.8	1888.0431	0.0107	RLWGFSESLLIRGAAGR
1.2	1887.0578	0.9960	NALEVDLGYLIITAARR

Spectrum No: 72; Query: 6084; Rank: 1

Peptide View

MS/MS Fragmentation of **NCVAIAADRR**
Found in **IPI00028004**, Tax_Id=9606 Gene_Symbol=PSMB3 Proteasome subunit beta type-3

Match to Query 6084: 1144.581252 from(382.534360,3+)
Title: 091224LimSK_Exosome3_06.134.134.3.dta
Data file C:\Temp\Mascoti\LSK_2D\091224LimSK_Exosome3_01_2D.mgf

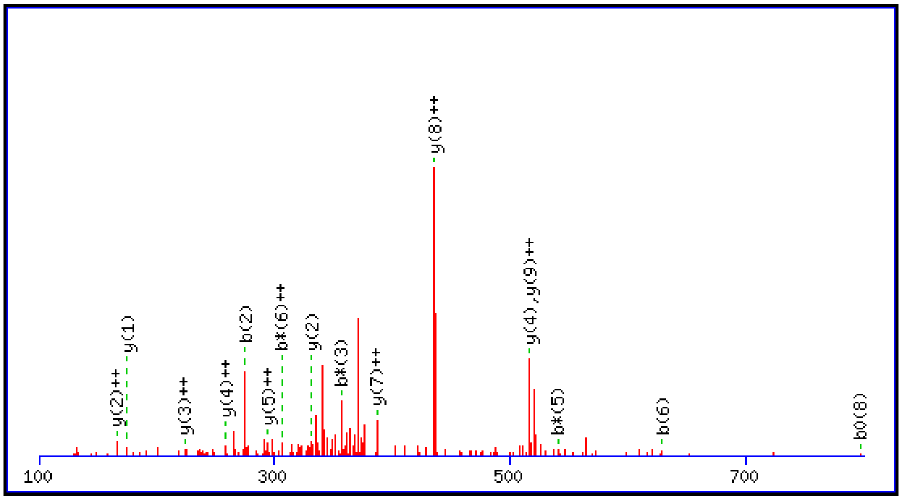

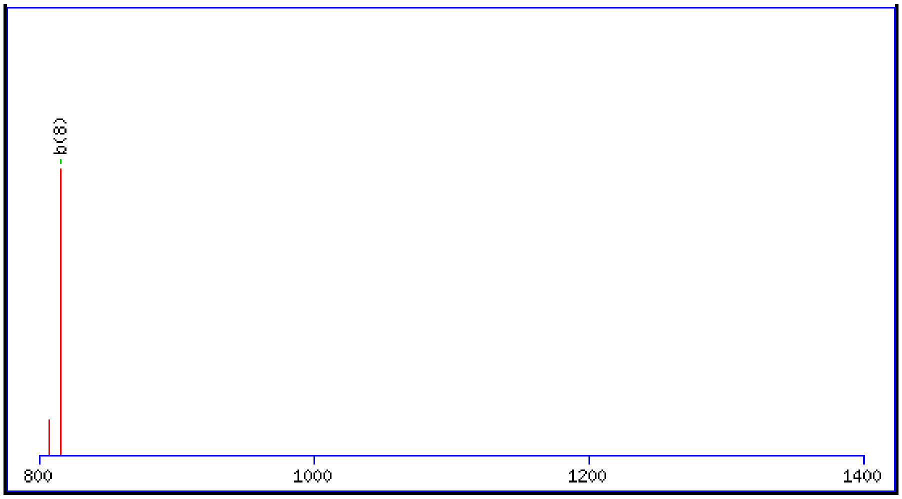

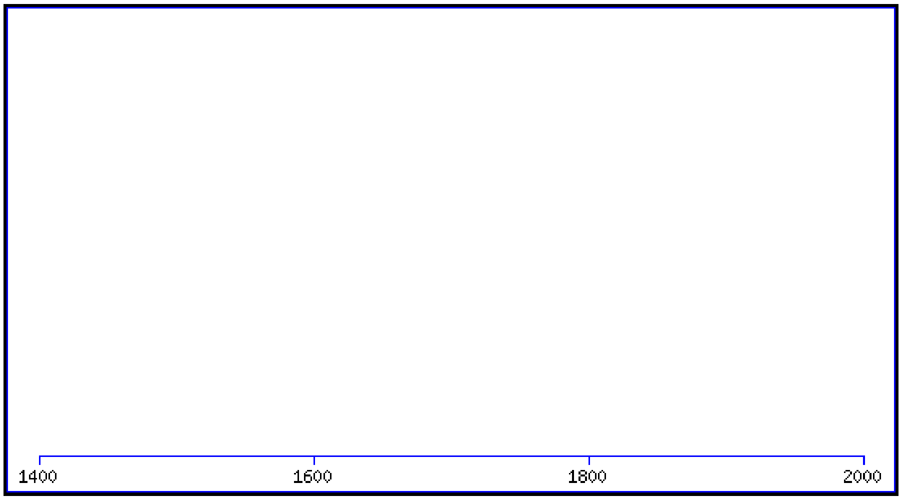

Monoisotopic mass of neutral peptide Mr(calc): 1144.5771
Fixed modifications: Carbamidomethyl (C)
Ions Score: 25 **Expect:** 0.49
Matches (Bold Red): 17/90 fragment ions using 39 most intense peaks

#	b	b^{++}	b^{*}	b^{*++}	b^{0}	b^{0++}	Seq.	y	y^{++}	y^{*}	y^{*++}	y^{0}	y^{0++}	#
1	115.0502	58.0287	98.0237	49.5155			N							10
2	**275.0809**	138.0441	258.0543	129.5308			C	1031.5415	**516.2744**	1014.5149	507.7611	1013.5309	507.2691	9
3	374.1493	187.5783	**357.1227**	179.0650			V	871.5108	**436.2591**	854.4843	427.7458	853.5003	427.2538	8
4	445.1864	223.0968	428.1598	214.5836			A	772.4424	**386.7248**	755.4159	378.2116	754.4318	377.7196	7
5	558.2704	279.6389	**541.2439**	271.1256			I	701.4053	351.2063	684.3787	342.6930	683.3947	342.2010	6
6	**629.3076**	315.1574	612.2810	**306.6441**			A	588.3212	**294.6643**	571.2947	286.1510	570.3107	285.6590	5
7	700.3447	350.6760	683.3181	342.1627			A	**517.2841**	**259.1457**	500.2576	250.6324	499.2736	250.1404	4
8	**815.3716**	408.1894	798.3451	399.6762	**797.3610**	399.1842	D	446.2470	**223.6271**	429.2205	215.1139	428.2364	214.6219	3
9	971.4727	486.2400	954.4462	477.7267	953.4622	477.2347	R	**331.2201**	**166.1137**	314.1935	157.6004			2
10							R	**175.1190**	88.0631	158.0924	79.5498			1

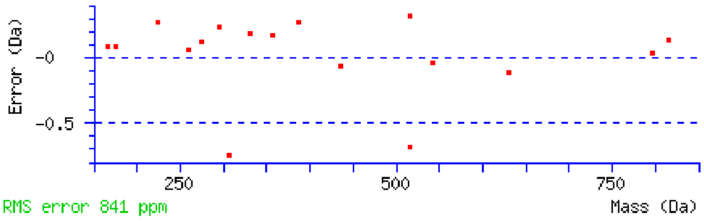

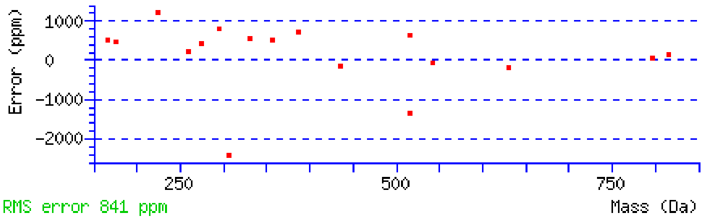

All matches to this query

Score	Mr(calc):	Delta	Sequence
24.9	1144.5771	0.0041	NCVAIAADRR
11.7	1144.5771	0.0042	MAAAAAAGEARR
11.4	1142.5696	2.0116	SSVKSLSISR
9.8	1144.5910	-0.0098	SLMSSTAPPVR
9.4	1144.5699	0.0114	MLSAHWEKK
9.1	1143.5801	1.0011	RPLTEPPVR
8.1	1143.5884	0.9929	AEEAVNEVKR
7.8	1144.5836	-0.0024	ELEEKADRR
7.7	1142.5849	1.9964	VVLKSTFNR
7.0	1144.5924	-0.0111	LCGALGWRGR

Spectrum No: 73; Query: 37082; Rank: 1

Peptide View

MS/MS Fragmentation of **RFGPYYTEPVIAGLDPK**
Found in **IPI00028004**, Tax_Id=9606 Gene_Symbol=PSMB3 Proteasome subunit beta type-3

Match to Query 37082: 1923.006462 from(642.009430,3+)
Title: 091224LimSK_Exosome3_06.5711.5711.3.dta
Data file C:\Temp\Mascoti\LSK_2D\091224LimSK_Exosome3_01_2D.mgf

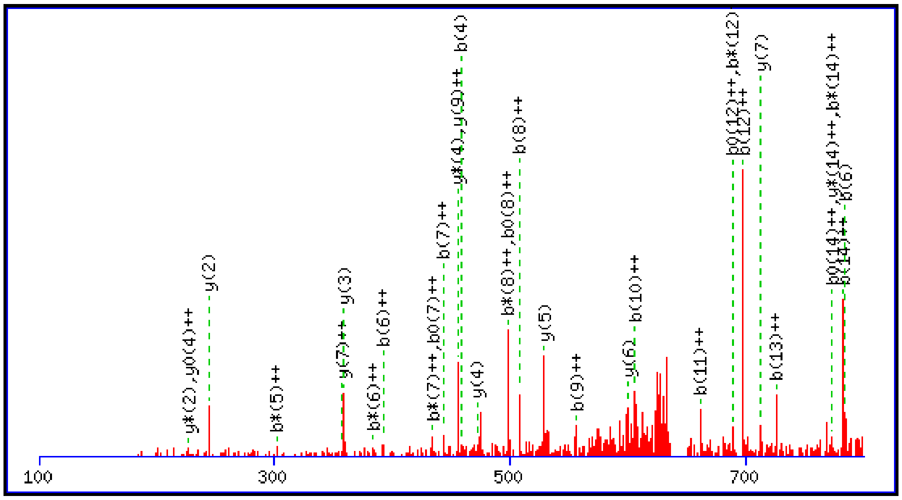

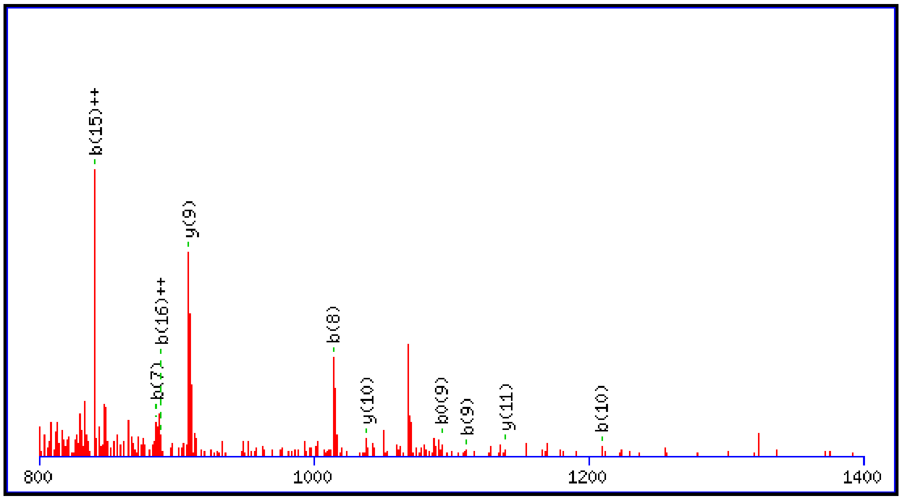

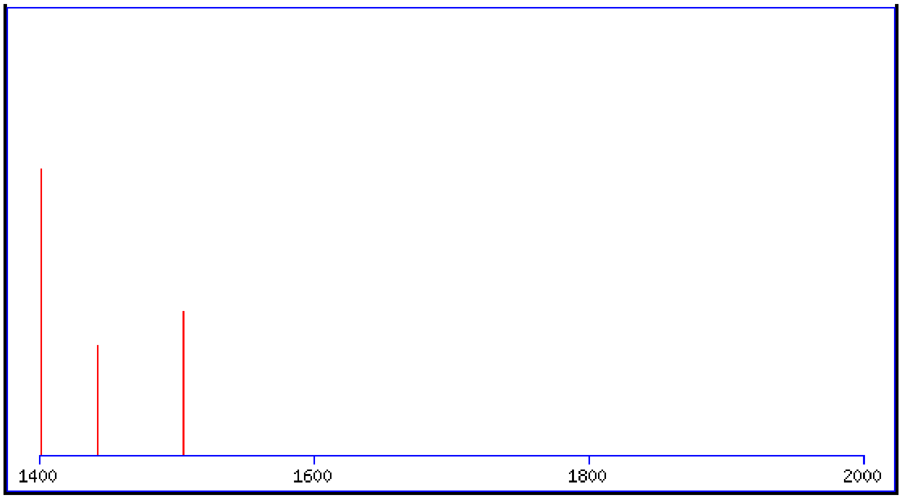

Monoisotopic mass of neutral peptide Mr(calc): 1921.9938
Fixed modifications: Carbamidomethyl (C)
Ions Score: 34 **Expect:** 0.062
Matches (Bold Red): 43/176 fragment ions using 129 most intense peaks

#	b	b^{++}	b*	b^{*++}	b^{0}	b^{0++}	Seq.	y	y^{++}	y*	y^{*++}	y^{0}	y^{0++}	#
1	157.1084	79.0578	140.0818	70.5446			R							17
2	304.1768	152.5920	287.1503	144.0788			F	1766.9000	883.9536	1749.8734	875.4403	1748.8894	874.9483	16
3	361.1983	181.1028	344.1717	172.5895			G	1619.8316	810.4194	1602.8050	801.9061	1601.8210	801.4141	15
4	**458.2510**	229.6292	441.2245	221.1159			P	1562.8101	781.9087	1545.7835	**773.3954**	1544.7995	772.9034	14
5	621.3144	311.1608	604.2878	**302.6475**			Y	1465.7573	733.3823	1448.7308	724.8690	1447.7468	724.3770	13
6	**784.3777**	**392.6925**	767.3511	**384.1792**			Y	1302.6940	651.8506	1285.6674	643.3374	1284.6834	642.8454	12
7	**885.4254**	**443.2163**	868.3988	**434.7030**	867.4148	**434.2110**	T	**1139.6307**	570.3190	1122.6041	561.8057	1121.6201	561.3137	11
8	**1014.4680**	**507.7376**	997.4414	**499.2243**	996.4574	**498.7323**	E	**1038.5830**	519.7951	1021.5564	511.2819	1020.5724	510.7898	10
9	**1111.5207**	**556.2640**	1094.4942	547.7507	**1093.5102**	547.2587	P	**909.5404**	**455.2738**	892.5138	446.7606	891.5298	446.2686	9
10	**1210.5891**	**605.7982**	1193.5626	597.2849	1192.5786	596.7929	V	812.4876	406.7475	795.4611	398.2342	794.4771	397.7422	8
11	1323.6732	**662.3402**	1306.6467	653.8270	1305.6626	653.3350	I	**713.4192**	**357.2132**	696.3927	348.7000	695.4087	348.2080	7
12	1394.7103	**697.8588**	1377.6838	**689.3455**	1376.6998	**688.8535**	A	**600.3352**	300.6712	583.3086	292.1579	582.3246	291.6659	6
13	1451.7318	**726.3695**	1434.7052	717.8563	1433.7212	717.3642	G	**529.2980**	265.1527	512.2715	256.6394	511.2875	256.1474	5
14	1564.8158	**782.9116**	1547.7893	**774.3983**	1546.8053	**773.9063**	L	**472.2766**	236.6419	**455.2500**	228.1287	454.2660	**227.6366**	4
15	1679.8428	**840.4250**	1662.8162	831.9118	1661.8322	831.4197	D	**359.1925**	180.0999	342.1660	171.5866	341.1819	171.0946	3
16	1776.8956	**888.9514**	1759.8690	880.4381	1758.8850	879.9461	P	**244.1656**	122.5864	**227.1390**	114.0731			2
17							K	147.1128	74.0600	130.0863	65.5468			1

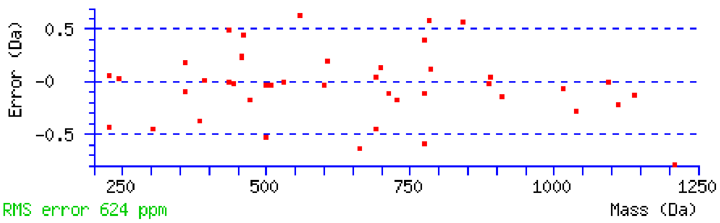

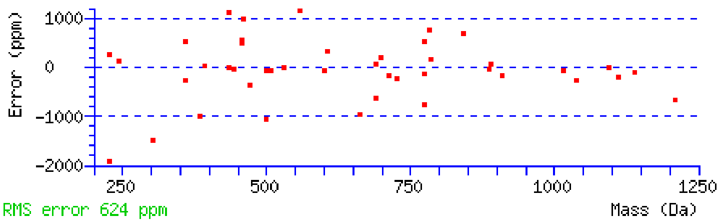

All matches to this query

Score	Mr(calc):	Delta	Sequence
33.6	1921.9938	1.0127	RFGPYYTEPVIAGLDPK
8.9	1922.0125	0.9939	DKTEKPDTVQLLTVKK
6.7	1920.9839	2.0226	LDKLSVLRLSVSYLR
6.2	1922.9900	0.0165	KARDMLGLEETQVILK
4.0	1920.9839	2.0226	LDKLSVLRLSVSYLR

2.1	1921.9874	1.0191	TQGSEKAALQLQLNTIK
2.0	1921.9874	1.0191	KSEVENGPSSVVTRILK
1.8	1923.0248	-0.0183	TXQDNPIMALSPGPVSLR
1.8	1922.9884	0.0181	TXQDNPIMALSPGPVSLR
1.0	1921.9874	1.0191	KSEVENGPSSVVTRILK

Spectrum No: 74; Query: 2474; Rank: 1

Peptide View

MS/MS Fragmentation of **AIHSWLTR**
Found in **IPI00555956**, Tax_Id=9606 Gene_Symbol=PSMB4 Proteasome subunit beta type-4

Match to Query 2474: 982.538708 from(492.276630,2+)
Title: 091224LimSK_Exosome3_06.1343.1343.2.dta
Data file C:\Temp\Mascoti\LSK_2D\091224LimSK_Exosome3_01_2D.mgf

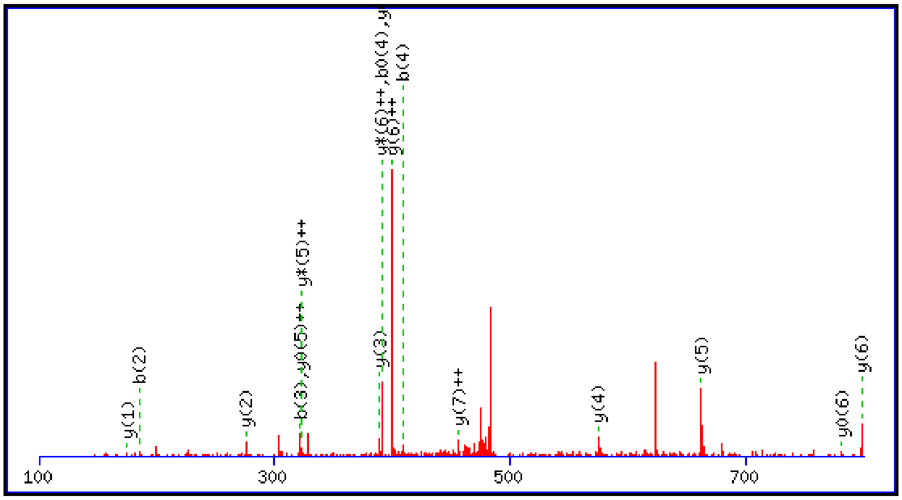

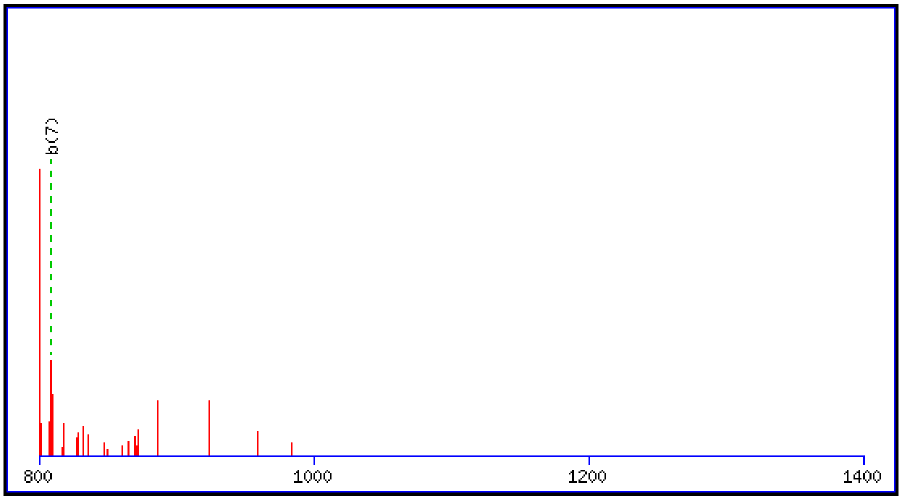

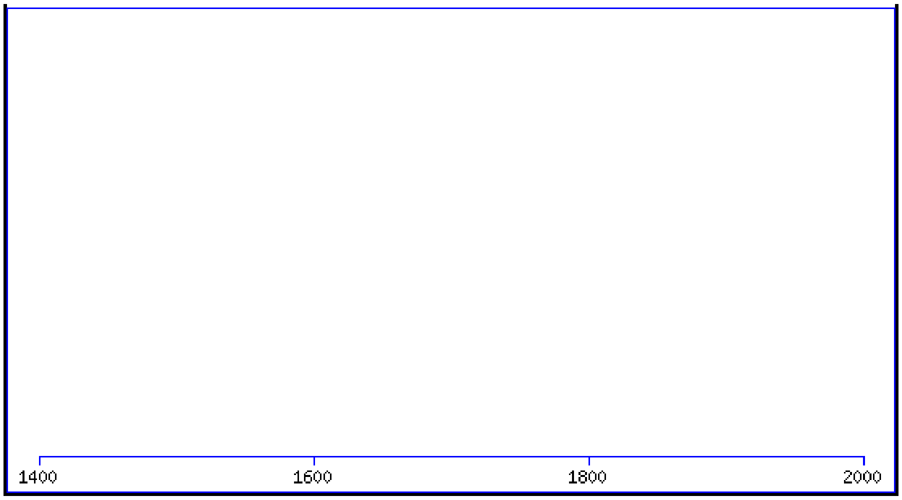

Monoisotopic mass of neutral peptide Mr(calc): 982.5348
Fixed modifications: Carbamidomethyl (C)
Ions Score: 25 **Expect:** 0.26
Matches (Bold Red): 18/62 fragment ions using 53 most intense peaks

#	b	b++	b0	b0++	Seq.	y	y++	y*	y*++	y0	y0++	#
1	72.0444	36.5258			A							8
2	**185.1285**	93.0679			I	912.5050	**456.7561**	895.4785	448.2429	894.4944	447.7509	7
3	**322.1874**	161.5973			H	**799.4209**	**400.2141**	782.3944	**391.7008**	**781.4104**	**391.2088**	6
4	**409.2194**	205.1133	**391.2088**	196.1081	S	**662.3620**	331.6847	645.3355	**323.1714**	644.3515	**322.6794**	5
5	595.2987	298.1530	577.2881	289.1477	W	**575.3300**	288.1686	558.3035	279.6554	557.3194	279.1634	4
6	708.3828	354.6950	690.3722	345.6897	L	**389.2507**	195.1290	372.2241	186.6157	371.2401	186.1237	3
7	**809.4304**	405.2189	791.4199	396.2136	T	**276.1666**	138.5870	259.1401	130.0737	258.1561	129.5817	2
8					R	**175.1190**	88.0631	158.0924	79.5498			1

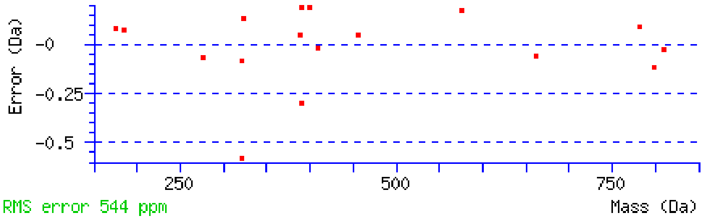

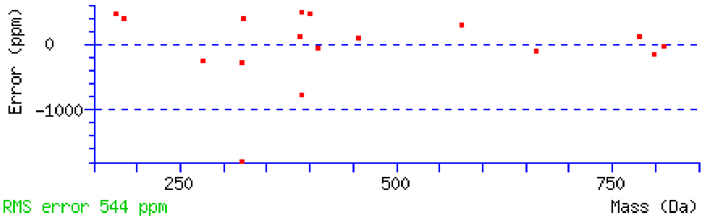

All matches to this query

Score	Mr(calc):	Delta	Sequence
24.9	982.5348	0.0039	AIHSWLTR
8.4	982.5420	-0.0033	RATHSAGKR
6.7	981.5260	1.0127	LLISSLTR
6.3	982.5348	0.0039	RFSISFAR
5.8	981.5260	1.0127	ILTITVSR
5.4	982.5348	0.0039	FKKNGSFR
5.3	982.5447	-0.0060	LAPLAEDVR
4.8	982.5447	-0.0060	ILPQDLER
1.5	981.5260	1.0127	LLISSLTR
1.5	982.5388	-0.0001	ALRFWYK

Spectrum No: 75; Query: 40349; Rank: 1

Peptide View

MS/MS Fragmentation of **FEGGVVIAADMLGSYGSLAR**
Found in **IPI00555956**, Tax_Id=9606 Gene_Symbol=PSMB4 Proteasome subunit beta type-4

Match to Query 40349: 2012.019688 from(1007.017120,2+)
Title: 091224LimSK_Exosome3_06.9282.9282.2.dta
Data file C:\Temp\Mascoti\LSK_2D\091224LimSK_Exosome3_01_2D.mgf

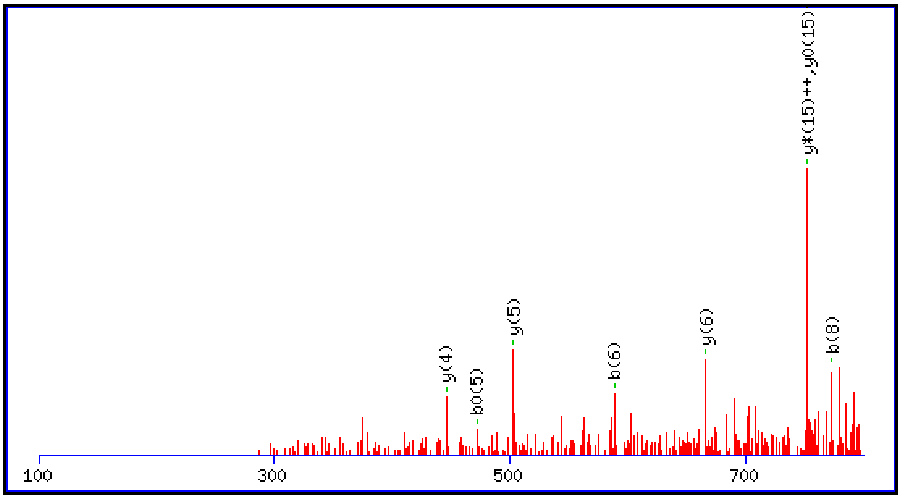

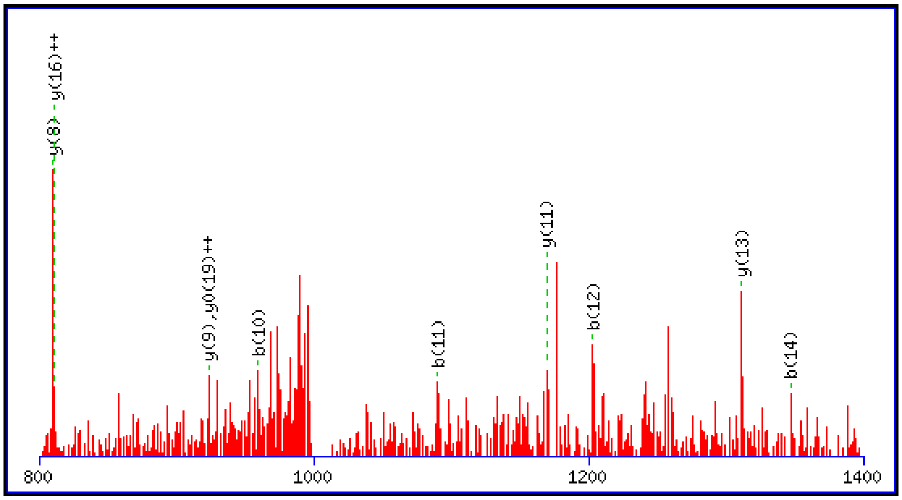

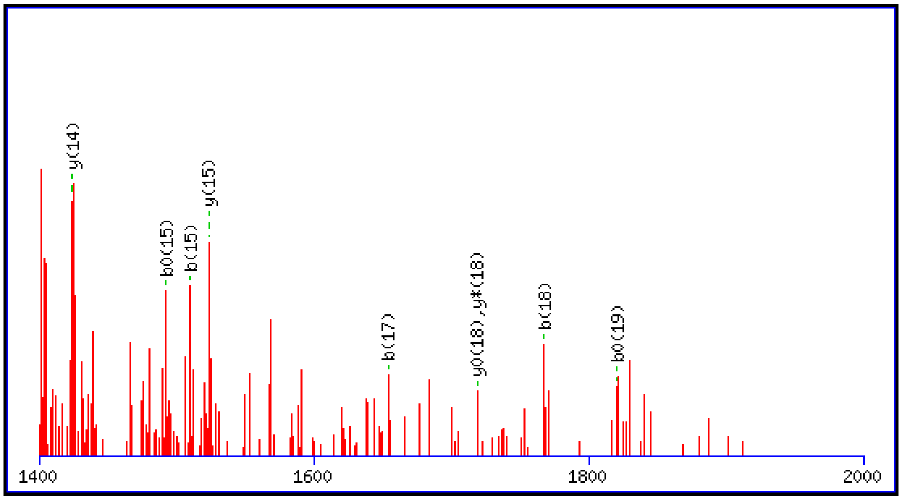

Monoisotopic mass of neutral peptide Mr(calc): 2012.0037
Fixed modifications: Carbamidomethyl (C)
Ions Score: 43 **Expect:** 0.0085
Matches (Bold Red): 28/182 fragment ions using 60 most intense peaks

		++	0	0++			++		++	0	0++	

#	b	b	b	b	Seq.	y	y	y*	y*	y	y	#
1	148.0757	74.5415			F							20
2	277.1183	139.0628	259.1077	130.0575	E	1865.9426	933.4749	1848.9160	924.9617	1847.9320	**924.4696**	19
3	334.1397	167.5735	316.1292	158.5682	G	1736.9000	868.9536	**1719.8734**	860.4404	**1718.8894**	859.9483	18
4	391.1612	196.0842	373.1506	187.0790	G	1679.8785	840.4429	1662.8520	831.9296	1661.8680	831.4376	17
5	490.2296	245.6185	**472.2191**	236.6132	V	1622.8571	**811.9322**	1605.8305	803.4189	1604.8465	802.9269	16
6	**589.2980**	295.1527	571.2875	286.1474	V	**1523.7886**	762.3980	1506.7621	**753.8847**	1505.7781	**753.3927**	15
7	702.3821	351.6947	684.3715	342.6894	I	**1424.7202**	712.8638	1407.6937	704.3505	1406.7097	703.8585	14
8	**773.4192**	387.2132	755.4087	378.2080	A	**1311.6362**	656.3217	1294.6096	647.8084	1293.6256	647.3164	13
9	844.4563	422.7318	826.4458	413.7265	A	1240.5990	620.8032	1223.5725	612.2899	1222.5885	611.7979	12
10	**959.4833**	480.2453	941.4727	471.2400	D	**1169.5619**	585.2846	1152.5354	576.7713	1151.5514	576.2793	11
11	**1090.5238**	545.7655	1072.5132	536.7602	M	1054.5350	527.7711	1037.5084	519.2579	1036.5244	518.7659	10
12	**1203.6078**	602.3075	1185.5973	593.3023	L	**923.4945**	462.2509	906.4680	453.7376	905.4839	453.2456	9
13	1260.6293	630.8183	1242.6187	621.8130	G	**810.4104**	405.7089	793.3839	397.1956	792.3999	396.7036	8
14	**1347.6613**	674.3343	1329.6507	665.3290	S	**753.3890**	377.1981	736.3624	368.6849	735.3784	368.1928	7
15	**1510.7246**	755.8660	**1492.7141**	746.8607	Y	**666.3570**	333.6821	649.3304	325.1688	648.3464	324.6768	6
16	1567.7461	784.3767	1549.7355	775.3714	G	**503.2936**	252.1504	486.2671	243.6372	485.2831	243.1452	5
17	**1654.7781**	827.8927	1636.7676	818.8874	S	**446.2722**	223.6397	429.2456	215.1264	428.2616	214.6344	4
18	**1767.8622**	884.4347	1749.8516	875.4295	L	359.2401	180.1237	342.2136	171.6104			3
19	1838.8993	919.9533	**1820.8887**	910.9480	A	246.1561	123.5817	229.1295	115.0684			2
20					R	175.1190	88.0631	158.0924	79.5498			1

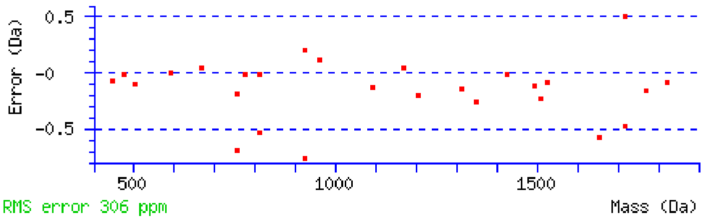

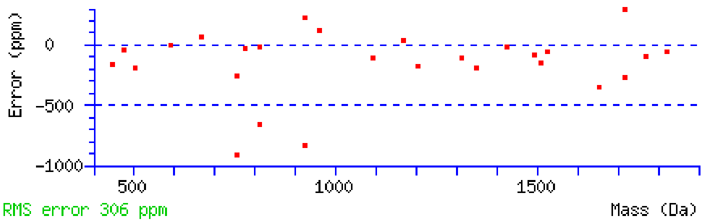

All matches to this query

Score	Mr(calc):	Delta	Sequence
43.2	2012.0037	0.0160	FEGGVVIAADMLGSYGSLAR

Spectrum No: 76; Query: 3424; Rank: 1

Peptide View

MS/MS Fragmentation of **FQIATVTEK**
Found in **IPI00555956**, Tax_Id=9606 Gene_Symbol=PSMB4 Proteasome subunit beta type-4

Match to Query 3424: 1035.565248 from(518.789900,2+)
Title: 091224LimSK_Exosome3_06.1901.1901.2.dta
Data file C:\Temp\Mascoti\LSK_2D\091224LimSK_Exosome3_01_2D.mgf

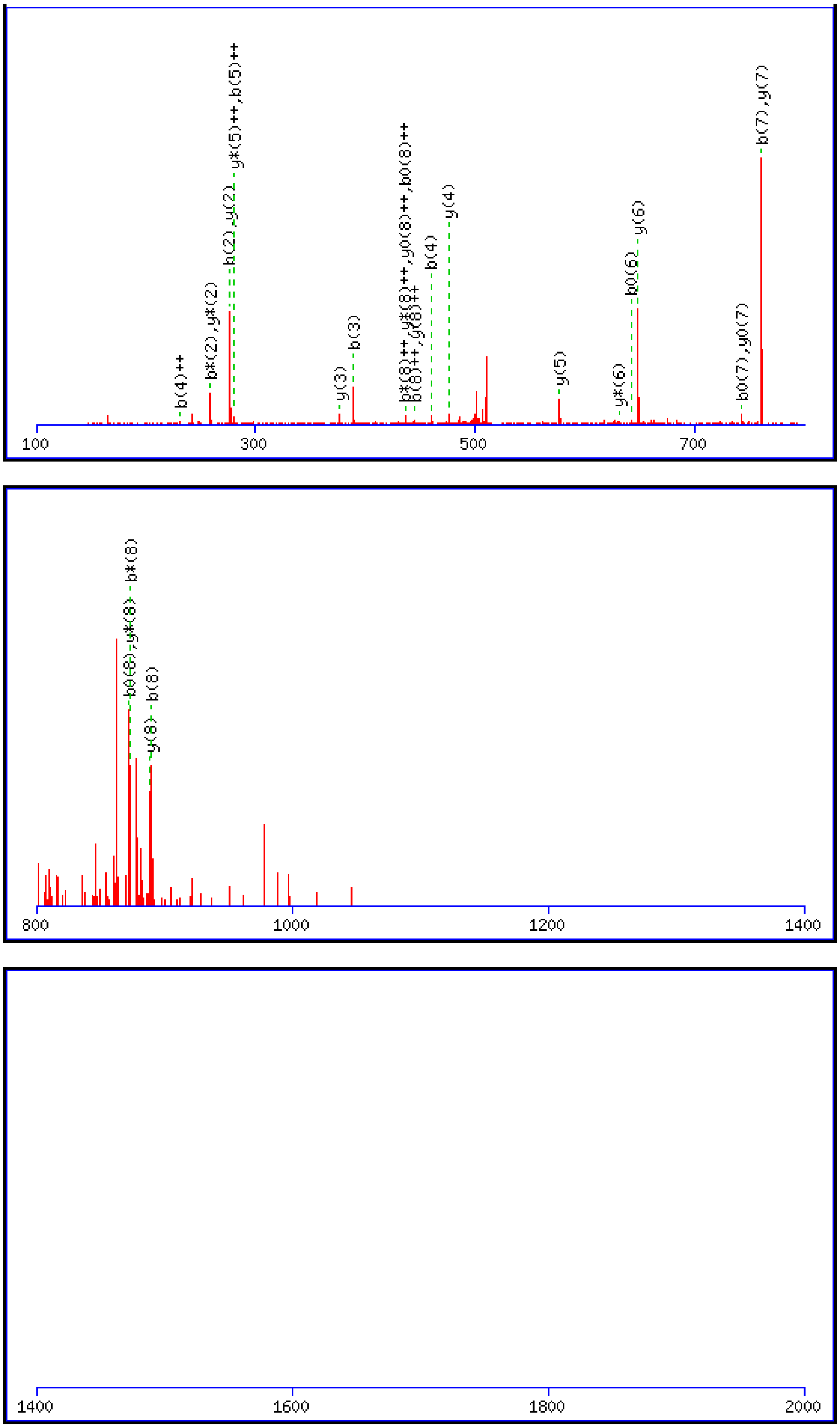

Monoisotopic mass of neutral peptide Mr(calc): 1035.5601
Fixed modifications: Carbamidomethyl (C)
Ions Score: 35 **Expect:** 0.035
Matches (Bold Red): 30/84 fragment ions using 59 most intense peaks

#	b	b^{++}	b*	b^{*++}	b^{0}	b^{0++}	Seq.	y	y^{++}	y*	y^{*++}	y^{0}	y^{0++}	#
1	148.0757	74.5415					F							9
2	**276.1343**	138.5708	**259.1077**	130.0575			Q	**889.4989**	**445.2531**	**872.4724**	**436.7398**	871.4884	**436.2478**	8
3	**389.2183**	195.1128	372.1918	186.5995			I	**761.4403**	381.2238	744.4138	372.7105	**743.4298**	372.2185	7
4	**460.2554**	**230.6314**	443.2289	222.1181			A	**648.3563**	324.6818	**631.3297**	316.1685	630.3457	315.6765	6
5	561.3031	**281.1552**	544.2766	272.6419	543.2926	272.1499	T	**577.3192**	289.1632	560.2926	**280.6499**	559.3086	280.1579	5
6	660.3715	330.6894	643.3450	322.1761	**642.3610**	321.6841	V	**476.2715**	238.6394	459.2449	230.1261	458.2609	229.6341	4

7	761.4192	381.2132	744.3927	372.7000	743.4087	372.2080	T	377.2031	189.1052	360.1765	180.5919	359.1925	180.0999	3
8	890.4618	445.7345	873.4353	437.2213	872.4512	436.7293	E	276.1554	138.5813	259.1288	130.0681	258.1448	129.5761	2
9							K	147.1128	74.0600	130.0863	65.5468			1

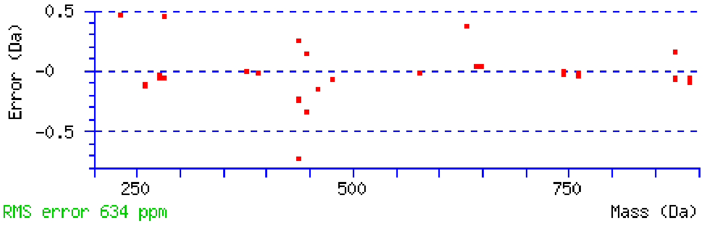

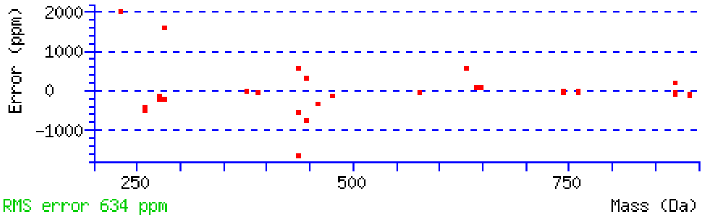

All matches to this query

Score	Mr(calc):	Delta	Sequence
35.0	1035.5601	0.0052	FQIATVTEK
18.1	1035.5600	0.0052	VAAYDKLEK
17.8	1035.5600	0.0052	FKIDASVEK
13.3	1034.5525	1.0127	GAITQPLKK
13.1	1035.5729	-0.0077	LKETPKIK
11.8	1035.5713	-0.0060	GFASITITAR
11.7	1035.5600	0.0052	EEKDFLKK
11.1	1035.5753	-0.0101	EFQPFLKK
9.9	1035.5712	-0.0060	AYREKIEK
9.9	1035.5713	-0.0060	EKVQYLTR

Spectrum No: 77; Query: 59024; Rank: 1

Peptide View

MS/MS Fragmentation of **GVEIEGPLSTETNWDIAHMISGFE**
Found in **IPI00555956**, Tax_Id=9606 Gene_Symbol=PSMB4 Proteasome subunit beta type-4

Match to Query 59024: 2631.238388 from(1316.626470,2+)
Title: 091224LimSK_Exosome3_06.8778.8778.2.dta
Data file C:\Temp\Mascoti\LSK_2D\091224LimSK_Exosome3_01_2D.mgf

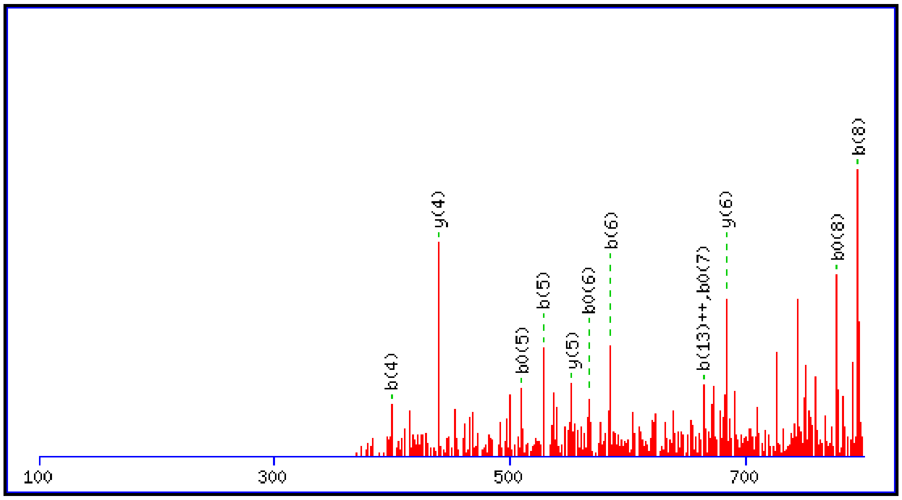

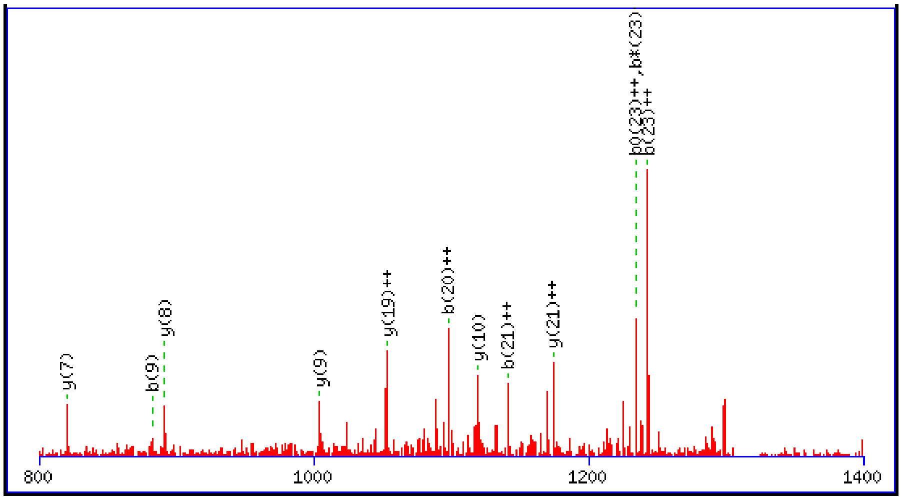

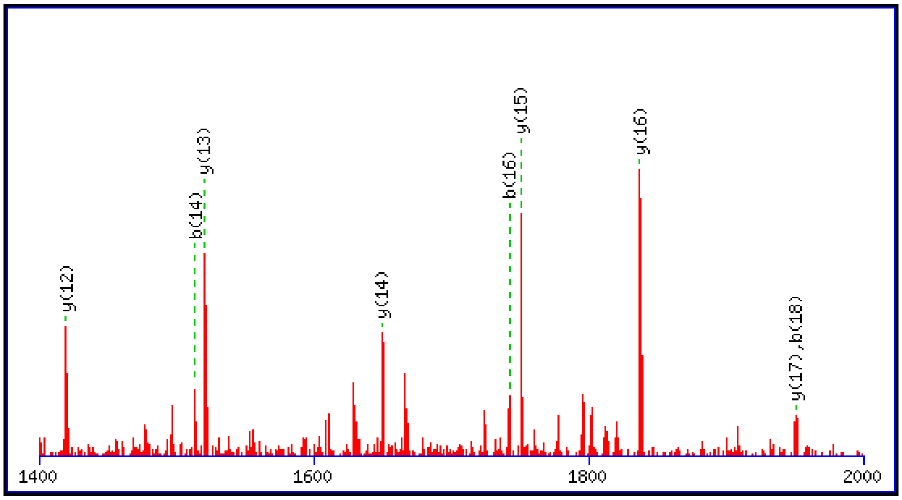

Monoisotopic mass of neutral peptide Mr(calc): 2631.2163
Fixed modifications: Carbamidomethyl (C)
Ions Score: 78 **Expect:** 4.6e-006
Matches (Bold Red): 33/226 fragment ions using 60 most intense peaks

#	b	b^{++}	b^{*}	b^{*++}	b^{0}	b^{0++}	Seq.	y	y^{++}	y^{*}	y^{*++}	y^{0}	y^{0++}	#
1	58.0287	29.5180					G							24
2	157.0972	79.0522					V	2575.2021	1288.1047	2558.1755	1279.5914	2557.1915	1279.0994	23
3	286.1397	143.5735			268.1292	134.5682	E	2476.1337	1238.5705	2459.1071	1230.0572	2458.1231	1229.5652	22
4	**399.2238**	200.1155			381.2132	191.1103	I	2347.0911	**1174.0492**	2330.0645	1165.5359	2329.0805	1165.0439	21
5	**528.2664**	264.6368			**510.2558**	255.6316	E	2234.0070	1117.5072	2216.9805	1108.9939	2215.9965	1108.5019	20
6	**585.2879**	293.1476			**567.2773**	284.1423	G	2104.9644	**1052.9859**	2087.9379	1044.4726	2086.9539	1043.9806	19
7	682.3406	341.6740			**664.3301**	332.6687	P	2047.9430	1024.4751	2030.9164	1015.9618	2029.9324	1015.4698	18
8	**795.4247**	398.2160			**777.4141**	389.2107	L	**1950.8902**	975.9487	1933.8637	967.4355	1932.8796	966.9435	17
9	**882.4567**	441.7320			864.4462	432.7267	S	**1837.8061**	919.4067	1820.7796	910.8934	1819.7956	910.4014	16
10	983.5044	492.2558			965.4938	483.2506	T	**1750.7741**	875.8907	1733.7476	867.3774	1732.7635	866.8854	15
11	1112.5470	556.7771			1094.5364	547.7719	E	**1649.7264**	825.3669	1632.6999	816.8536	1631.7159	816.3616	14
12	1213.5947	607.3010			1195.5841	598.2957	T	**1520.6838**	760.8456	1503.6573	752.3323	1502.6733	751.8403	13
13	1327.6376	**664.3224**	1310.6111	655.8092	1309.6270	655.3172	N	**1419.6362**	710.3217	1402.6096	701.8084	1401.6256	701.3164	12
14	**1513.7169**	757.3621	1496.6904	748.8488	1495.7064	748.3568	W	1305.5932	653.3003			1287.5827	644.2950	11
15	1628.7439	814.8756	1611.7173	806.3623	1610.7333	805.8703	D	**1119.5139**	560.2606			1101.5034	551.2553	10
16	**1741.8279**	871.4176	1724.8014	862.9043	1723.8174	862.4123	I	**1004.4870**	502.7471			986.4764	493.7418	9
17	1812.8650	906.9362	1795.8385	898.4229	1794.8545	897.9309	A	**891.4029**	446.2051			873.3923	437.1998	8
18	**1949.9239**	975.4656	1932.8974	966.9523	1931.9134	966.4603	H	**820.3658**	410.6865			802.3552	401.6813	7
19	2080.9644	1040.9859	2063.9379	1032.4726	2062.9539	1031.9806	M	**683.3069**	342.1571			665.2963	333.1518	6

20	2194.0485	**1097.5279**	2177.0219	1089.0146	2176.0379	1088.5226	I	**552.2664**	276.6368			534.2558	267.6316	5
21	2281.0805	**1141.0439**	2264.0540	1132.5306	2263.0700	1132.0386	S	**439.1823**	220.0948			421.1718	211.0895	4
22	2338.1020	1169.5546	2321.0754	1161.0414	2320.0914	1160.5493	G	352.1503	176.5788			334.1397	167.5735	3
23	2485.1704	**1243.0888**	2468.1439	**1234.5756**	2467.1598	**1234.0836**	F	295.1288	148.0681			277.1183	139.0628	2
24							E	148.0604	74.5339			130.0499	65.5286	1

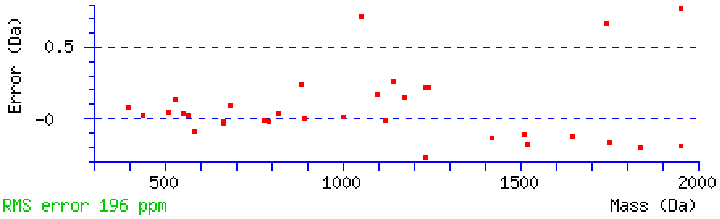

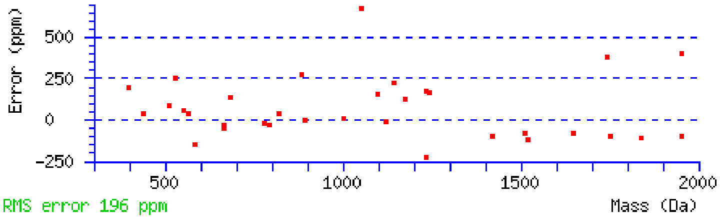

All matches to this query

Score	Mr(calc):	Delta	Sequence
78.2	2631.2163	0.0221	GVEIEGPLSTETNWDIAHMISGFE
11.9	2630.2506	0.9878	SVPPSPPERPSLATASQNGAPALVK
9.6	2630.2506	0.9878	SVPPSPPERPSLATASQNGAPALVK
5.6	2630.2506	0.9878	SVPPSPPERPSLATASQNGAPALVK
5.3	2630.2418	0.9966	QVEPLDPPAGSAPGEHVFVKGYEK
3.5	2630.2159	1.0224	SSKRPASPYGEADGEVAMVTSRQK
3.5	2630.2159	1.0224	SSKRPASPYGEADGEVAMVTSRQK
3.2	2631.2169	0.0215	SLEVGTVMTLFYSKKSQRPER
3.2	2631.2169	0.0215	SLEVGTVMTLFYSKKSQRPER
2.6	2630.2159	1.0224	SSKRPASPYGEADGEVAMVTSRQK

Spectrum No: 78; Query: 54324; Rank: 1

Peptide View

MS/MS Fragmentation of **QVLGQMVIDEELLGDGHSYSPR**
Found in **IPI00555956**, Tax_Id=9606 Gene_Symbol=PSMB4 Proteasome subunit beta type-4

Match to Query 54324: 2442.199722 from(815.073850,3+)
Title: 091224LimSK_Exosome3_06.6600.6600.3.dta
Data file C:\Temp\Mascoti\LSK_2D\091224LimSK_Exosome3_01_2D.mgf

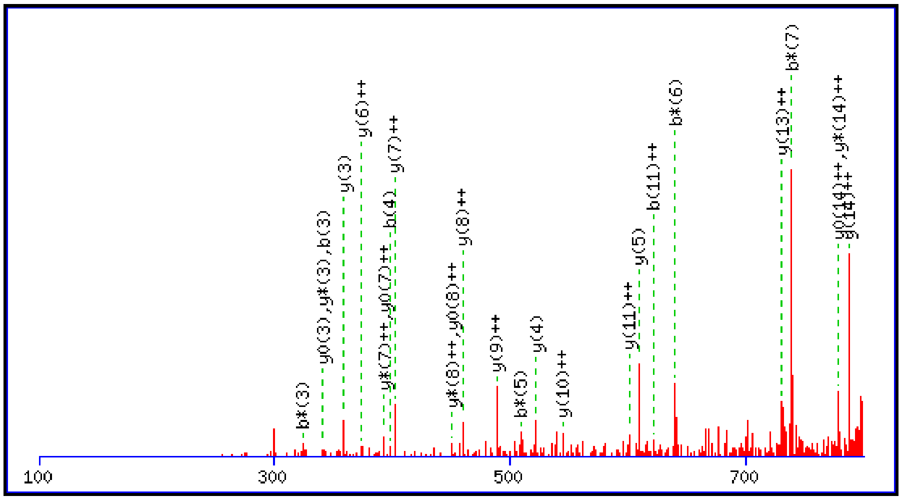

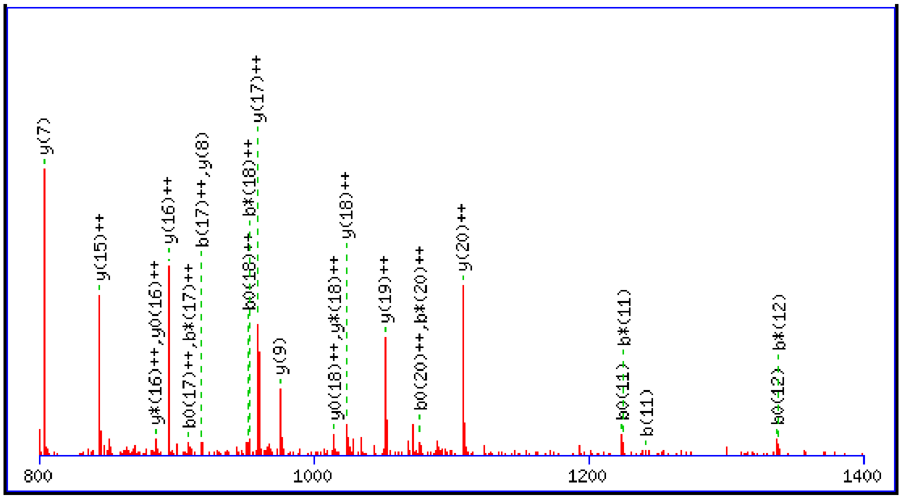

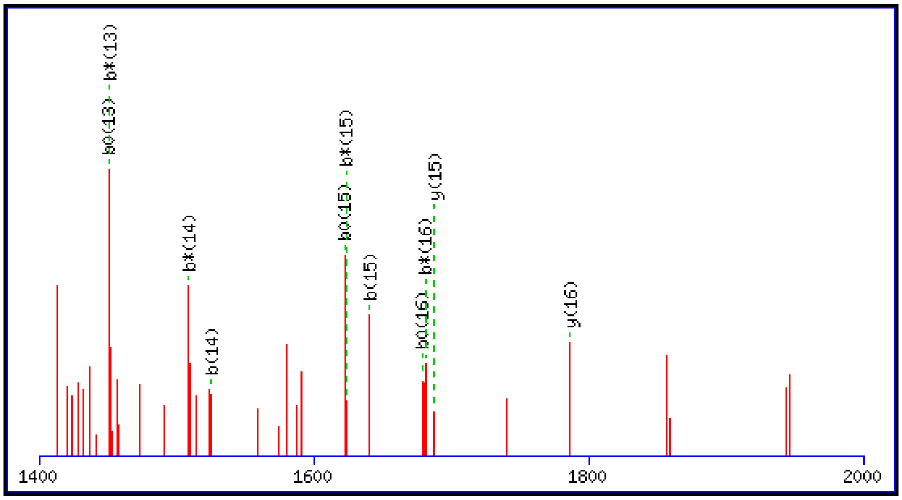

Monoisotopic mass of neutral peptide Mr(calc): 2442.1849
Fixed modifications: Carbamidomethyl (C)
Ions Score: 66 **Expect:** 5.3e-005
Matches (Bold Red): 62/232 fragment ions using 110 most intense peaks

#	b	b^{++}	b*	b^{*++}	b^0	b^{0++}	Seq.	y	y^{++}	y*	y^{*++}	y^0	y^{0++}	#
1	129.0659	65.0366	112.0393	56.5233			Q							22
2	228.1343	114.5708	211.1077	106.0575			V	2315.1336	1158.0704	2298.1071	1149.5572	2297.1231	1149.0652	21
3	**341.2183**	171.1128	**324.1918**	162.5995			L	2216.0652	**1108.5362**	2199.0387	1100.0230	2198.0546	1099.5310	20
4	**398.2398**	199.6235	381.2132	191.1103			G	2102.9811	**1051.9942**	2085.9546	1043.4809	2084.9706	1042.9889	19
5	526.2984	263.6528	**509.2718**	255.1396			Q	2045.9597	**1023.4835**	2028.9331	**1014.9702**	2027.9491	**1014.4782**	18
6	657.3389	329.1731	**640.3123**	320.6598			M	1917.9011	**959.4542**	1900.8746	950.9409	1899.8905	950.4489	17
7	756.4073	378.7073	**739.3807**	370.1940			V	**1786.8606**	**893.9339**	1769.8341	**885.4207**	1768.8501	**884.9287**	16
8	869.4913	435.2493	852.4648	426.7360			I	**1687.7922**	**844.3997**	1670.7657	835.8865	1669.7816	835.3945	15
9	984.5183	492.7628	967.4917	484.2495	966.5077	483.7575	D	1574.7081	**787.8577**	1557.6816	**779.3444**	1556.6976	**778.8524**	14
10	1113.5609	557.2841	1096.5343	548.7708	1095.5503	548.2788	E	1459.6812	**730.3442**	1442.6546	721.8310	1441.6706	721.3390	13
11	**1242.6035**	**621.8054**	**1225.5769**	613.2921	**1224.5929**	612.8001	E	1330.6386	665.8229	1313.6121	657.3097	1312.6280	656.8177	12
12	1355.6875	678.3474	**1338.6610**	669.8341	**1337.6770**	669.3421	L	1201.5960	**601.3016**	1184.5695	592.7884	1183.5854	592.2964	11
13	1468.7716	734.8894	**1451.7450**	726.3762	**1450.7610**	725.8842	L	1088.5119	**544.7596**	1071.4854	536.2463	1070.5014	535.7543	10
14	**1525.7931**	763.4002	**1508.7665**	754.8869	1507.7825	754.3949	G	**975.4279**	**488.2176**	958.4013	479.7043	957.4173	479.2123	9
15	**1640.8200**	820.9136	**1623.7935**	812.4004	**1622.8094**	811.9084	D	**918.4064**	**459.7068**	901.3799	**451.1936**	900.3959	**450.7016**	8
16	1697.8415	849.4244	**1680.8149**	840.9111	**1679.8309**	840.4191	G	**803.3795**	**402.1934**	786.3529	**393.6801**	785.3689	**393.1881**	7
17	1834.9004	**917.9538**	1817.8738	**909.4406**	1816.8898	**908.9485**	H	746.3580	**373.6826**	729.3315	365.1694	728.3474	364.6774	6
18	1921.9324	961.4698	1904.9059	**952.9566**	1903.9218	**952.4646**	S	**609.2991**	305.1532	592.2726	296.6399	591.2885	296.1479	5
19	2084.9957	1043.0015	2067.9692	1034.4882	2066.9852	1033.9962	Y	**522.2671**	261.6372	505.2405	253.1239	504.2565	252.6319	4

**20**	2172.0278	1086.5175	2155.0012	**1078.0042**	2154.0172	**1077.5122**	**S**	**359.2037**	180.1055	**342.1772**	171.5922	**341.1932**	171.1002	**3**
**21**	2269.0805	1135.0439	2252.0540	1126.5306	2251.0700	1126.0386	**P**	272.1717	136.5895	255.1452	128.0762			**2**
**22**							**R**	175.1190	88.0631	158.0924	79.5498			**1**

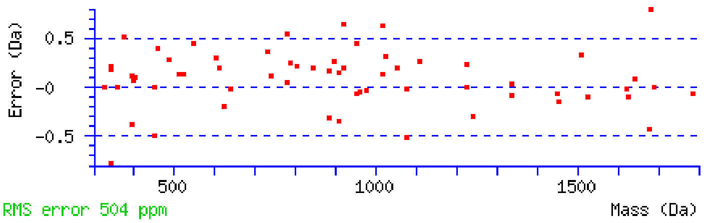

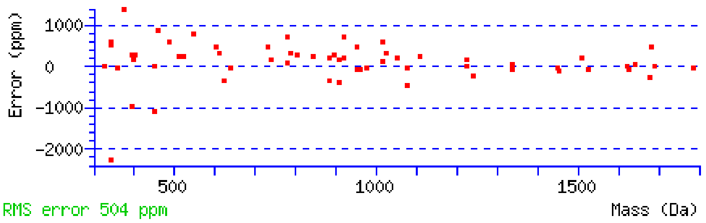

All matches to this query

Score	Mr(calc):	Delta	Sequence
66.5	2442.1849	0.0148	QVLGQMVIDEELLGDGHSYSPR
5.1	2441.2193	0.9805	VLRATGRAQALVTDLGTAELAR
4.4	2441.1910	1.0087	FEKINEGRMPGTVHSSWHSVK
3.8	2441.2064	0.9933	QDSAAHLGKLSKSTAPGHTSQLK
3.8	2441.2064	0.9933	QDSAAHLGKLSKSTAPGHTSQLK
2.8	2441.1839	1.0158	PPSLSPQLLVADSSPSSTAPSPAR
2.4	2442.2040	-0.0043	RGWNTTSQRYSNVIQPSSFSK
2.1	2441.2193	0.9805	VLRATGRAQALVTDLGTAELAR
1.7	2442.1879	0.0118	EYRPHLVDQGAMLLNVSGHVK
1.7	2440.2064	1.9933	KAILTMTQLYKVALQWMVK

Spectrum No: 79; Query: 22048; Rank: 1

Peptide View

MS/MS Fragmentation of **TQNPMVTGTSVLGVK**
Found in **IPI00555956**, Tax_Id=9606 Gene_Symbol=PSMB4 Proteasome subunit beta type-4

Match to Query 22048: 1530.816668 from(766.415610,2+)
Title: 091224LimSK_Exosome3_06.3921.3921.2.dta
Data file C:\Temp\Mascoti\LSK_2D\091224LimSK_Exosome3_01_2D.mgf

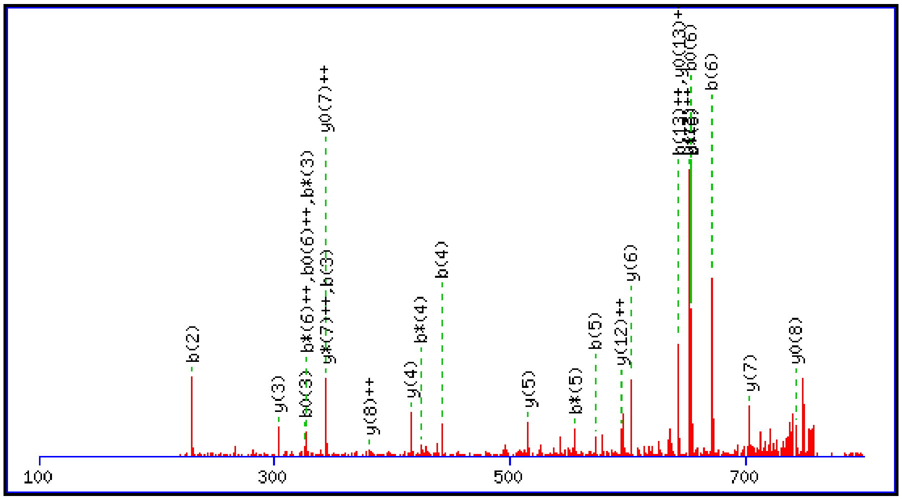

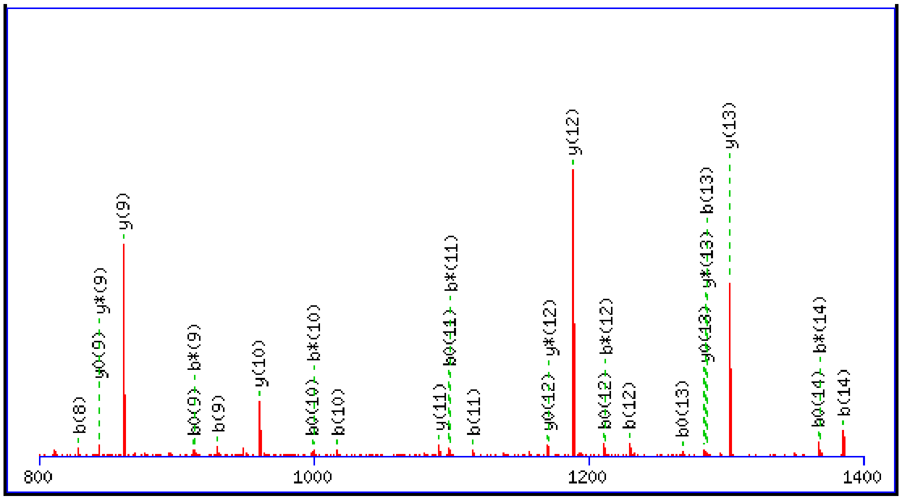

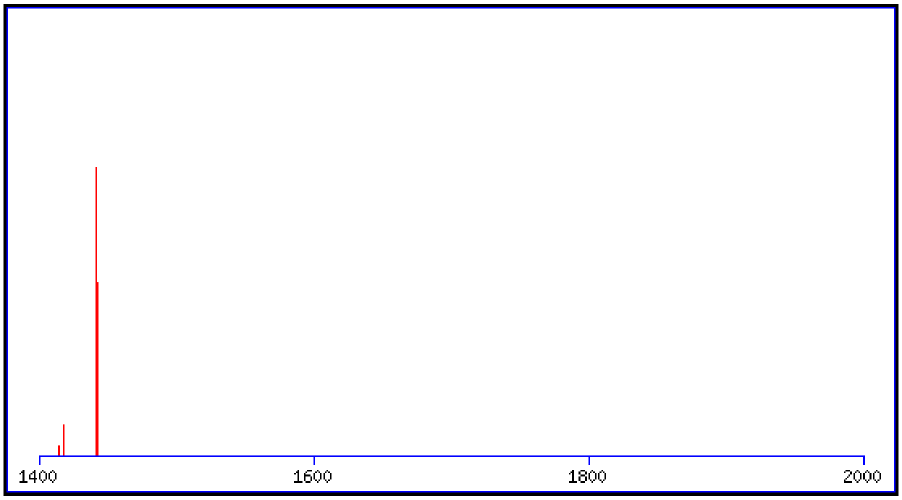

Monoisotopic mass of neutral peptide Mr(calc): 1530.8076
Fixed modifications: Carbamidomethyl (C)
Ions Score: 69 **Expect:** 1.6e-005
Matches (Bold Red): 56/156 fragment ions using 100 most intense peaks

#	b	b^{++}	b*	b^{*++}	b^0	b^{0++}	Seq.	y	y^{++}	y*	y^{*++}	y^0	y^{0++}	#
1	102.0550	51.5311			84.0444	42.5258	T							15
2	**230.1135**	115.5604	213.0870	107.0471	212.1030	106.5551	Q	1430.7672	715.8872	1413.7406	707.3740	1412.7566	706.8819	14
3	**344.1565**	172.5819	**327.1299**	164.0686	**326.1459**	163.5766	N	**1302.7086**	**651.8579**	**1285.6821**	**643.3447**	**1284.6980**	**642.8527**	13
4	**441.2092**	221.1083	**424.1827**	212.5950	423.1987	212.1030	P	**1188.6657**	**594.8365**	**1171.6391**	586.3232	**1170.6551**	585.8312	12
5	**572.2497**	286.6285	**555.2232**	278.1152	554.2391	277.6232	M	**1091.6129**	546.3101	1074.5864	537.7968	1073.6023	537.3048	11
6	**671.3181**	336.1627	**654.2916**	**327.6494**	**653.3076**	**327.1574**	V	**960.5724**	480.7899	943.5459	472.2766	942.5619	471.7846	10
7	772.3658	386.6865	755.3393	378.1733	754.3552	377.6813	T	**861.5040**	431.2556	**844.4775**	422.7424	**843.4934**	422.2504	9
8	**829.3873**	415.1973	812.3607	406.6840	811.3767	406.1920	G	760.4563	**380.7318**	743.4298	372.2185	**742.4458**	371.7265	8
9	**930.4349**	465.7211	**913.4084**	457.2078	**912.4244**	456.7158	T	**703.4349**	352.2211	686.4083	**343.7078**	685.4243	**343.2158**	7
10	**1017.4670**	509.2371	**1000.4404**	500.7239	**999.4564**	500.2318	S	**602.3872**	301.6972	585.3606	293.1840	584.3766	292.6919	6
11	**1116.5354**	558.7713	**1099.5088**	550.2581	**1098.5248**	549.7660	V	**515.3552**	258.1812	498.3286	249.6679			5
12	**1229.6195**	615.3134	**1212.5929**	606.8001	**1211.6089**	606.3081	L	**416.2867**	208.6470	399.2602	200.1337			4
13	**1286.6409**	**643.8241**	1269.6144	635.3108	**1268.6303**	634.8188	G	**303.2027**	152.1050	286.1761	143.5917			3
14	**1385.7093**	693.3583	**1368.6828**	684.8450	**1367.6988**	684.3530	V	246.1812	123.5942	229.1547	115.0810			2
15							K	147.1128	74.0600	130.0863	65.5468			1

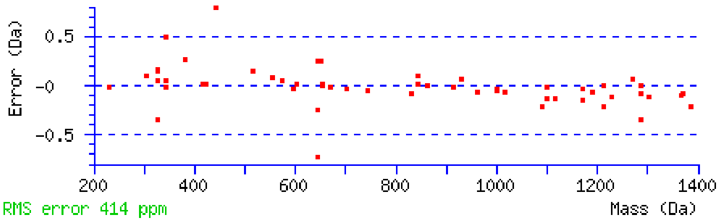

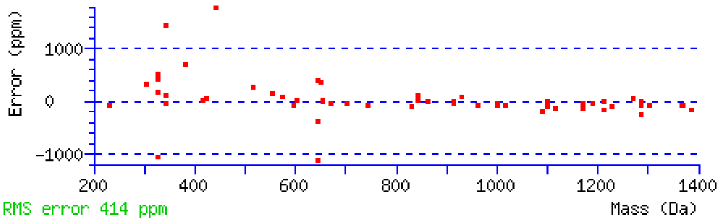

All matches to this query

Score	Mr(calc):	Delta	Sequence
69.1	1530.8076	0.0091	TQNPMVTGTSVLGVK
12.8	1528.8097	2.0070	LNNDLVGSTENLLK
6.2	1528.8167	2.0000	KVIPYIASQFRK
5.3	1530.8076	0.0091	DLIVMLRDTDVNK
4.6	1530.8188	-0.0021	MVVQTASQASPLKR
3.9	1529.8161	1.0005	LQTLQSENSNLRK
3.5	1530.8194	-0.0028	PIAFNEPLSFLQR
3.5	1530.8187	-0.0020	QTLKSLIIKLSK
2.7	1530.8266	-0.0100	SSWSSLGRAPSLKR
0.9	1530.8208	-0.0041	FFLYVLGHSRHR

Spectrum No: 80; Query: 46479; Rank: 1

Peptide View

MS/MS Fragmentation of **VNNSTMLGASGDYADFQYLK**
Found in **IPI00555956**, Tax_Id=9606 Gene_Symbol=PSMB4 Proteasome subunit beta type-4

Match to Query 46479: 2193.019848 from(1097.517200,2+)
Title: 091224LimSK_Exosome3_06.5930.5930.2.dta
Data file C:\Temp\Mascoti\LSK_2D\091224LimSK_Exosome3_01_2D.mgf

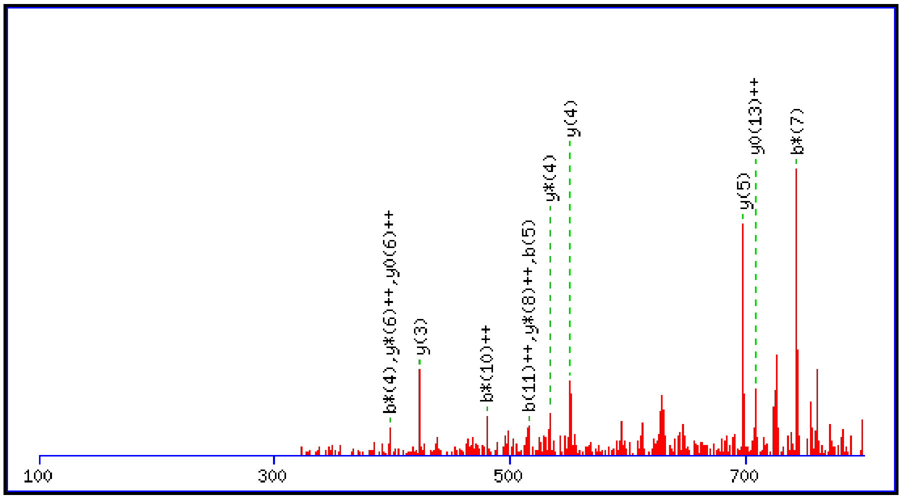

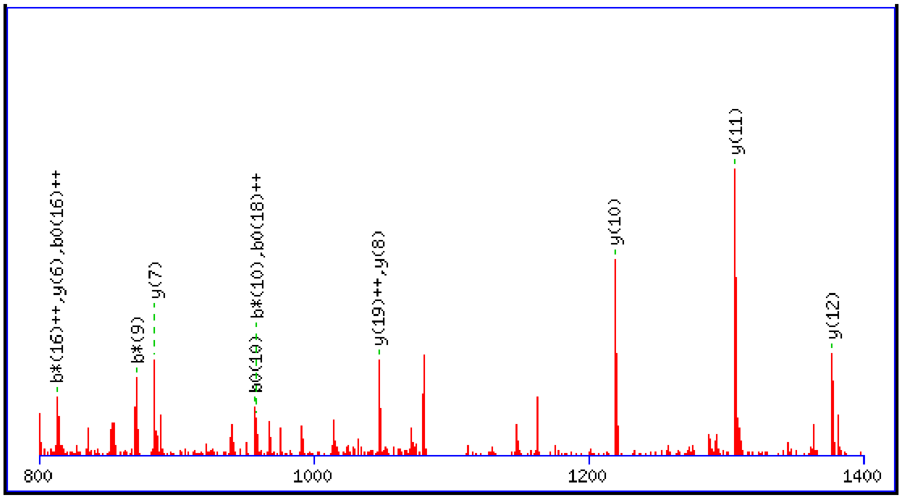

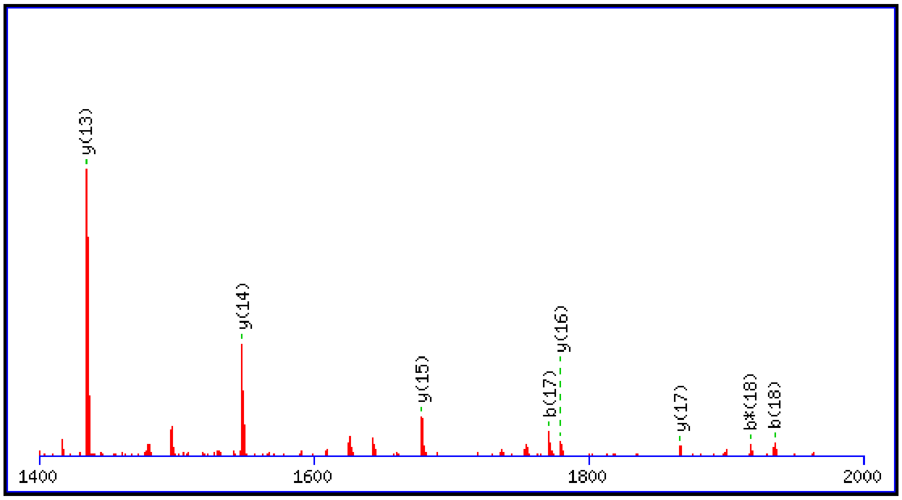

Monoisotopic mass of neutral peptide Mr(calc): 2193.0048
Fixed modifications: Carbamidomethyl (C)
Ions Score: 122 **Expect:** 1.7e-010
Matches (Bold Red): 34/210 fragment ions using 37 most intense peaks

#	b	b^{++}	b*	b^{*++}	b^0	b^{0++}	Seq.	y	y^{++}	y*	y^{*++}	y^0	y^{0++}	#
1	100.0757	50.5415					V							20
2	214.1186	107.5629	197.0921	99.0497			N	2094.9437	**1047.9755**	2077.9171	1039.4622	2076.9331	1038.9702	19
3	328.1615	164.5844	311.1350	156.0711			N	1980.9008	990.9540	1963.8742	982.4407	1962.8902	981.9487	18
4	415.1936	208.1004	**398.1670**	199.5871	397.1830	199.0951	S	**1866.8578**	933.9326	1849.8313	925.4193	1848.8473	924.9273	17
5	**516.2413**	258.6243	499.2147	250.1110	498.2307	249.6190	T	**1779.8258**	890.4165	1762.7993	881.9033	1761.8153	881.4113	16
6	647.2817	324.1445	630.2552	315.6312	629.2712	315.1392	M	**1678.7781**	839.8927	1661.7516	831.3794	1660.7676	830.8874	15
7	760.3658	380.6865	**743.3393**	372.1733	742.3552	371.6813	L	**1547.7377**	774.3725	1530.7111	765.8592	1529.7271	765.3672	14
8	817.3873	409.1973	800.3607	400.6840	799.3767	400.1920	G	**1434.6536**	717.8304	1417.6270	709.3172	1416.6430	**708.8251**	13
9	888.4244	444.7158	**871.3978**	436.2026	870.4138	435.7105	A	**1377.6321**	689.3197	1360.6056	680.8064	1359.6216	680.3144	12
10	975.4564	488.2318	**958.4299**	**479.7186**	**957.4458**	479.2266	S	**1306.5950**	653.8011	1289.5685	645.2879	1288.5844	644.7959	11
11	1032.4779	**516.7426**	1015.4513	508.2293	1014.4673	507.7373	G	**1219.5630**	610.2851	1202.5364	601.7719	1201.5524	601.2798	10
12	1147.5048	574.2560	1130.4783	565.7428	1129.4942	565.2508	D	1162.5415	581.7744	1145.5150	573.2611	1144.5310	572.7691	9
13	1310.5681	655.7877	1293.5416	647.2744	1292.5576	646.7824	Y	**1047.5146**	524.2609	1030.4880	**515.7477**	1029.5040	515.2556	8
14	1381.6053	691.3063	1364.5787	682.7930	1363.5947	682.3010	A	**884.4512**	442.7293	867.4247	434.2160	866.4407	433.7240	7
15	1496.6322	748.8197	1479.6057	740.3065	1478.6216	739.8145	D	**813.4141**	407.2107	796.3876	**398.6974**	795.4036	**398.2054**	6
16	1643.7006	822.3539	1626.6741	**813.8407**	1625.6900	**813.3487**	F	**698.3872**	349.6972	681.3606	341.1840			5
17	**1771.7592**	886.3832	1754.7326	877.8700	1753.7486	877.3780	Q	**551.3188**	276.1630	**534.2922**	267.6498			4
18	**1934.8225**	967.9149	**1917.7960**	959.4016	1916.8120	**958.9096**	Y	**423.2602**	212.1337	406.2336	203.6205			3
19	2047.9066	1024.4569	2030.8800	1015.9437	2029.8960	1015.4516	L	260.1969	130.6021	243.1703	122.0888			2

20							K	147.1128	74.0600	130.0863	65.5468			1

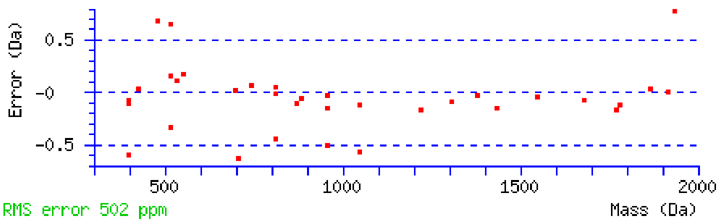

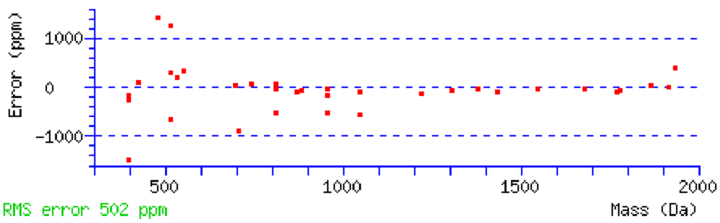

All matches to this query

Score	Mr(calc):	Delta	Sequence
122.3	2193.0048	0.0150	VNNSTMLGASGDYADFQYLK
7.7	2191.0211	1.9987	EKKWWQSENGLDHVSIR
5.2	2193.0098	0.0100	EKVADMTSVLDPNKMYLK
2.9	2191.0324	1.9875	GGQRATPSWREAGLGAWPSK
2.9	2191.0324	1.9875	GGQRATPSWREAGLGAWPSK
2.6	2191.0216	1.9983	DQSEPVWDMTTQKVQQQK
2.0	2192.0310	0.9888	DTLLTRHRAPGWGTQMTR
1.7	2193.0098	0.0100	EKVADMTSVLDPNKMYLK
1.6	2191.0054	2.0144	EEILKTTCFICGLERDK
1.6	2191.0054	2.0144	EEILKTTCFICGLERDK

Spectrum No: 81; Query: 40090; Rank: 1

Peptide View

MS/MS Fragmentation of **AGGSASAMLQPLLDNQVGFK**
Found in **IPI00025019**, Tax_Id=9606 Gene_Symbol=PSMB1 Proteasome subunit beta type-1

Match to Query 40090: 2003.027728 from(1002.521140,2+)
Title: 091224LimSK_Exosome3_07.6973.6973.2.dta
Data file C:\Temp\Mascoti\LSK_2D\091224LimSK_Exosome3_01_2D.mgf

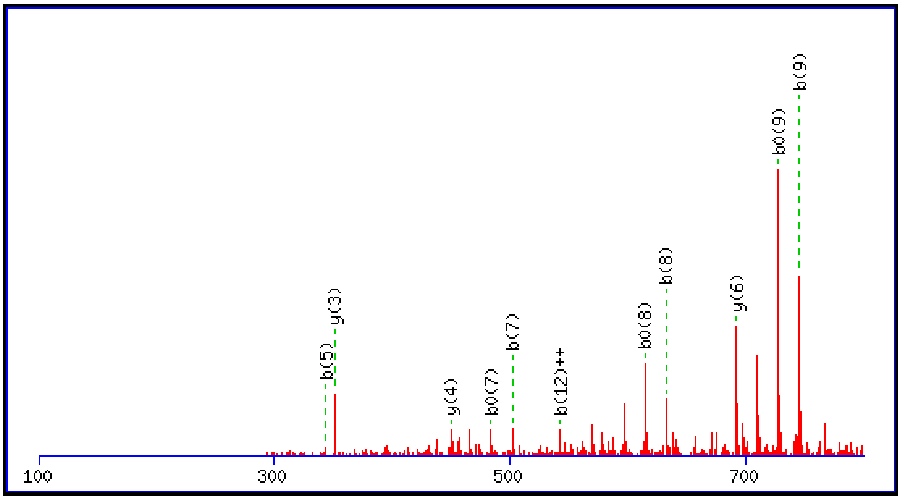

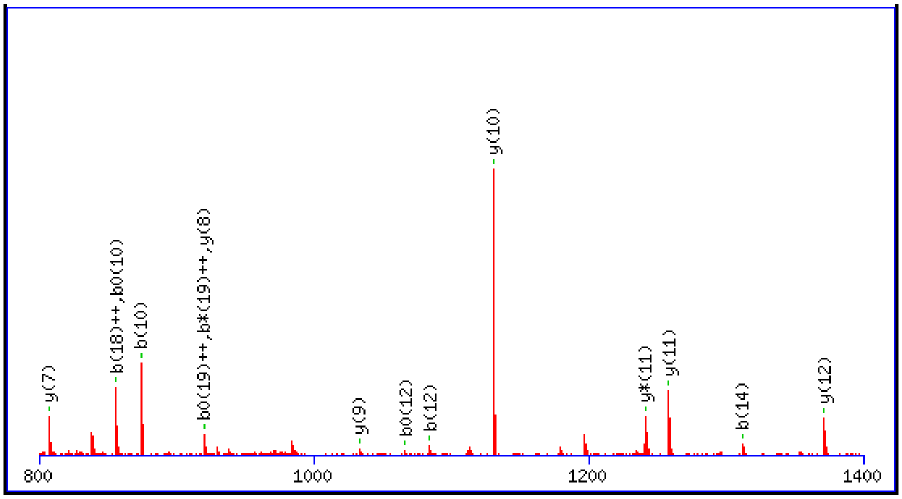

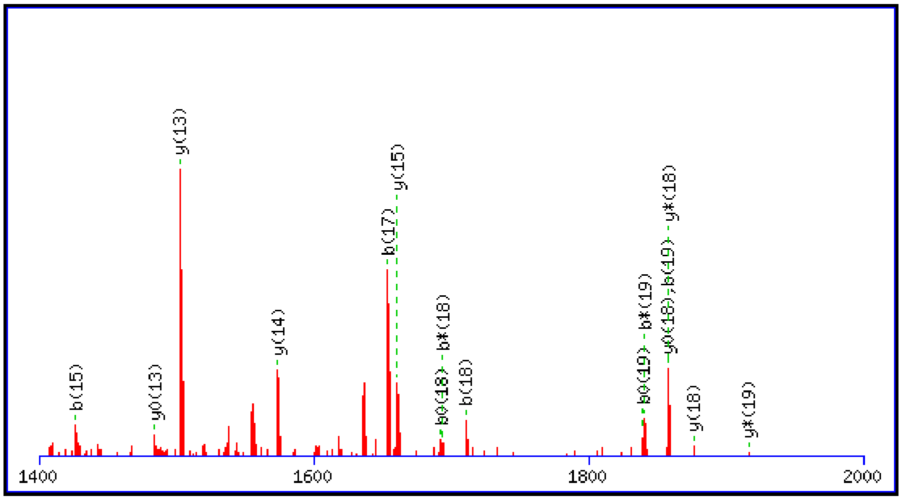

Monoisotopic mass of neutral peptide Mr(calc): 2003.0146
Fixed modifications: Carbamidomethyl (C)
Ions Score: 93 **Expect:** 8.9e-008
Matches (Bold Red): 42/192 fragment ions using 62 most intense peaks

#	b	b^{++}	b*	b^{*++}	b^0	b^{0++}	Seq.	y	y^{++}	y*	y^{*++}	y^0	y^{0++}	#
1	72.0444	36.5258					A							20
2	129.0659	65.0366					G	1932.9848	966.9960	**1915.9582**	958.4828	1914.9742	957.9907	19
3	186.0873	93.5473					G	**1875.9633**	938.4853	**1858.9368**	929.9720	**1857.9527**	929.4800	18
4	273.1193	137.0633			255.1088	128.0580	S	1818.9418	909.9746	1801.9153	901.4613	1800.9313	900.9693	17
5	**344.1565**	172.5819			326.1459	163.5766	A	1731.9098	866.4585	1714.8833	857.9453	1713.8993	857.4533	16
6	431.1885	216.0979			413.1779	207.0926	S	**1660.8727**	830.9400	1643.8462	822.4267	1642.8621	821.9347	15
7	**502.2256**	251.6164			**484.2150**	242.6112	A	**1573.8407**	787.4240	1556.8141	778.9107	1555.8301	778.4187	14
8	**633.2661**	317.1367			**615.2555**	308.1314	M	**1502.8036**	751.9054	1485.7770	743.3921	**1484.7930**	742.9001	13
9	**746.3502**	373.6787			**728.3396**	364.6734	L	**1371.7631**	686.3852	1354.7365	677.8719	1353.7525	677.3799	12
10	**874.4087**	437.7080	857.3822	429.1947	**856.3982**	428.7027	Q	**1258.6790**	629.8431	**1241.6525**	621.3299	1240.6684	620.8379	11
11	971.4615	486.2344	954.4349	477.7211	953.4509	477.2291	P	**1130.6204**	565.8139	1113.5939	557.3006	1112.6099	556.8086	10
12	**1084.5456**	**542.7764**	1067.5190	534.2631	**1066.5350**	533.7711	L	**1033.5677**	517.2875	1016.5411	508.7742	1015.5571	508.2822	9
13	1197.6296	599.3184	1180.6031	590.8052	1179.6191	590.3132	L	**920.4836**	460.7454	903.4571	452.2322	902.4730	451.7402	8
14	**1312.6566**	656.8319	1295.6300	648.3186	1294.6460	647.8266	D	**807.3995**	404.2034	790.3730	395.6901	789.3890	395.1981	7
15	**1426.6995**	713.8534	1409.6729	705.3401	1408.6889	704.8481	N	**692.3726**	346.6899	675.3461	338.1767			6
16	1554.7581	777.8827	1537.7315	769.3694	1536.7475	768.8774	Q	578.3297	289.6685	561.3031	281.1552			5
17	**1653.8265**	827.4169	1636.7999	818.9036	1635.8159	818.4116	V	**450.2711**	225.6392	433.2445	217.1259			4
18	**1710.8479**	**855.9276**	**1693.8214**	847.4143	**1692.8374**	846.9223	G	**351.2027**	176.1050	334.1761	167.5917			3
19	**1857.9164**	929.4618	**1840.8898**	**920.9485**	**1839.9058**	**920.4565**	F	294.1812	147.5942	277.1547	139.0810			2

20							K	147.1128	74.0600	130.0863	65.5468			1

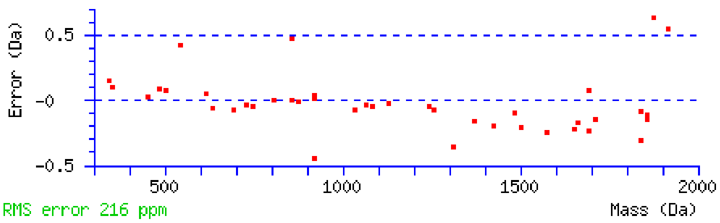

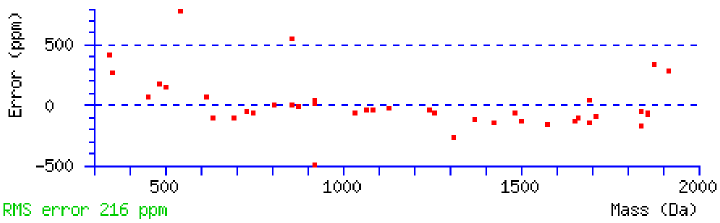

All matches to this query

Score	Mr(calc):	Delta	Sequence
92.7	2003.0146	0.0131	AGGSASAMLQPLLDNQVGFK
0.8	2003.0364	-0.0086	FNAHEFATLVIDILSDAK
0.7	2002.0361	0.9917	HTLTRNLDVQLLDTKR
0.7	2002.0361	0.9917	HTLTRNLDVQLLDTKR
0.7	2001.0379	1.9899	QTASQVEQPIITQGSSVTK

Spectrum No: 82; Query: 35592; Rank: 1

Peptide View

MS/MS Fragmentation of **AMTTGAIAAMLSTILYSR**
Found in **IPI00025019**, Tax_Id=9606 Gene_Symbol=PSMB1 Proteasome subunit beta type-1

Match to Query 35592: 1869.985348 from(935.999950,2+)
Title: 091224LimSK_Exosome3_07.9275.9275.2.dta
Data file C:\Temp\Mascoti\LSK_2D\091224LimSK_Exosome3_01_2D.mgf

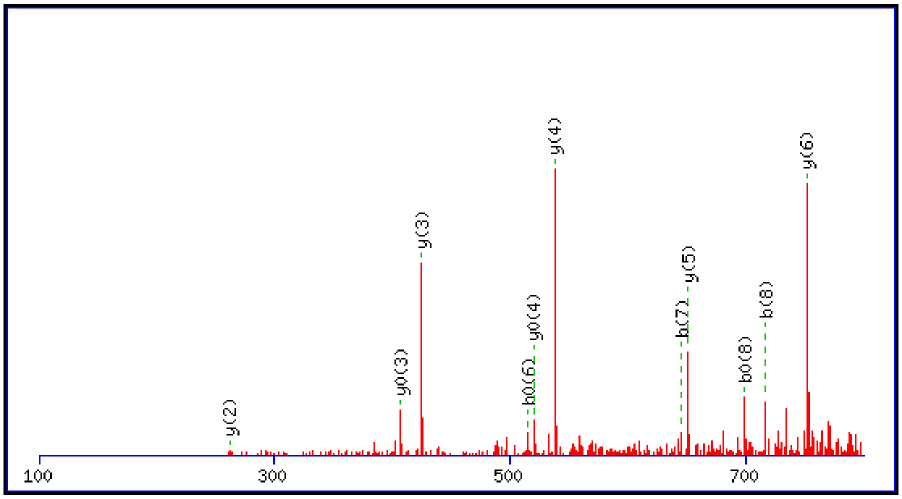

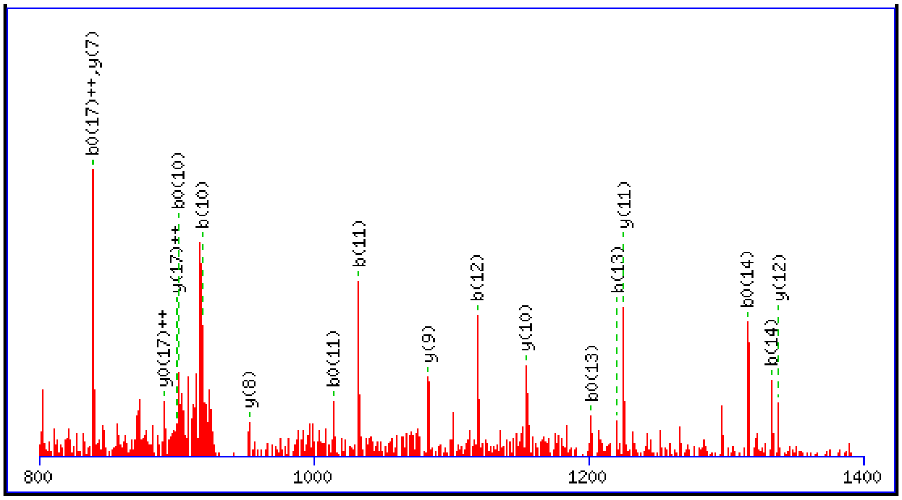

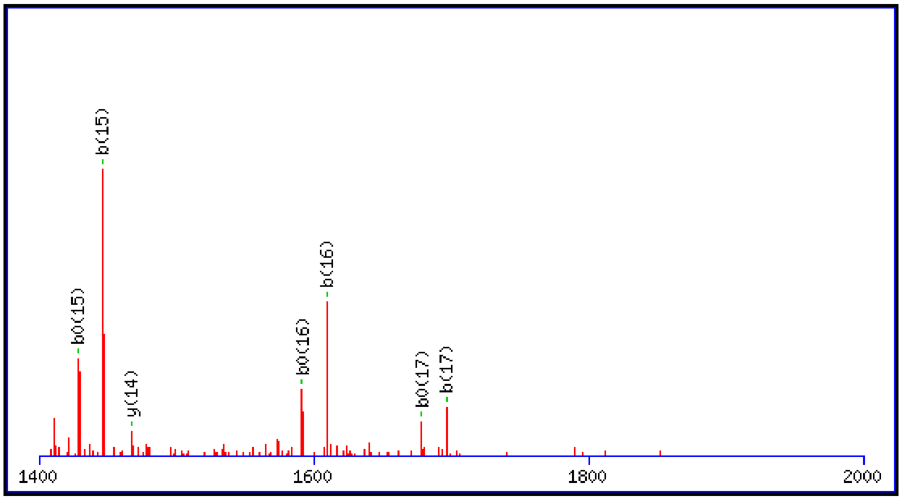

Monoisotopic mass of neutral peptide Mr(calc): 1869.9692
Fixed modifications: Carbamidomethyl (C)
Ions Score: 70 **Expect:** 1.3e-005
Matches (Bold Red): 36/164 fragment ions using 84 most intense peaks

#	b	b^{++}	b^{0}	b^{0++}	Seq.	y	y^{++}	y^{*}	y^{*++}	y^{0}	y^{0++}	#
1	72.0444	36.5258			A							18
2	203.0849	102.0461			M	1799.9394	**900.4733**	1782.9129	891.9601	1781.9288	**891.4681**	17
3	304.1326	152.5699	286.1220	143.5646	T	1668.8989	834.9531	1651.8724	826.4398	1650.8884	825.9478	16
4	405.1802	203.0938	387.1697	194.0885	T	1567.8512	784.4293	1550.8247	775.9160	1549.8407	775.4240	15
5	462.2017	231.6045	444.1911	222.5992	G	**1466.8036**	733.9054	1449.7770	725.3921	1448.7930	724.9001	14
6	533.2388	267.1230	**515.2282**	258.1178	A	1409.7821	705.3947	1392.7556	696.8814	1391.7715	696.3894	13
7	**646.3229**	323.6651	628.3123	314.6598	I	**1338.7450**	669.8761	1321.7184	661.3629	1320.7344	660.8708	12
8	**717.3600**	359.1836	**699.3494**	350.1784	A	**1225.6609**	613.3341	1208.6344	604.8208	1207.6504	604.3288	11
9	788.3971	394.7022	770.3865	385.6969	A	**1154.6238**	577.8155	1137.5973	569.3023	1136.6132	568.8103	10
10	**919.4376**	460.2224	**901.4270**	451.2171	M	**1083.5867**	542.2970	1066.5601	533.7837	1065.5761	533.2917	9
11	**1032.5217**	516.7645	**1014.5111**	507.7592	L	**952.5462**	476.7767	935.5197	468.2635	934.5356	467.7715	8
12	**1119.5537**	560.2805	1101.5431	551.2752	S	**839.4621**	420.2347	822.4356	411.7214	821.4516	411.2294	7
13	**1220.6014**	610.8043	**1202.5908**	601.7990	T	**752.4301**	376.7187	735.4036	368.2054	734.4196	367.7134	6
14	**1333.6854**	667.3463	**1315.6749**	658.3411	I	**651.3824**	326.1949	634.3559	317.6816	633.3719	317.1896	5
15	**1446.7695**	723.8884	**1428.7589**	714.8831	L	**538.2984**	269.6528	521.2718	261.1396	**520.2878**	260.6475	4
16	**1609.8328**	805.4200	**1591.8223**	796.4148	Y	**425.2143**	213.1108	408.1878	204.5975	**407.2037**	204.1055	3
17	**1696.8648**	848.9361	**1678.8543**	**839.9308**	S	**262.1510**	131.5791	245.1244	123.0659	244.1404	122.5738	2
18					R	175.1190	88.0631	158.0924	79.5498			1

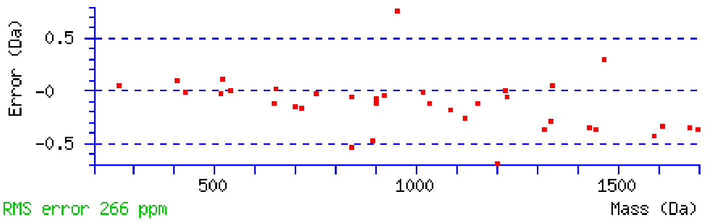

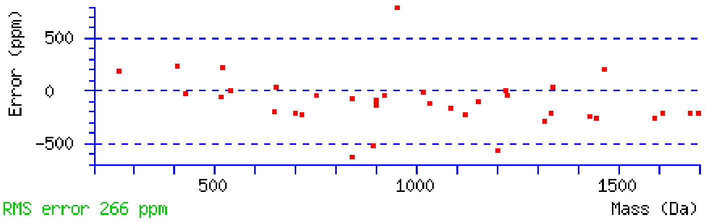

All matches to this query

Score	Mr(calc):	Delta	Sequence
69.8	1869.9692	0.0161	AMTTGAIAAMLSTILYSR
5.7	1868.9761	1.0093	NLVDPITHDLIISLAR
4.2	1869.9737	0.0116	LWIEQYGTVEILNHR
3.5	1869.9727	0.0126	VHVKTHHGVPLPQVSR
3.2	1868.9734	1.0120	EPLTRIRNNSAIHIR
2.3	1869.9809	0.0044	LRHVYTPSQTLEDRR
1.4	1869.9870	-0.0017	MPTVTVPQLELDVGLSR
1.3	1868.9857	0.9996	GQERPGIQGVPQLSFTR
0.9	1869.9697	0.0156	QPQAGLSQANFTLGPVSR
0.9	1869.9826	0.0028	SIKAPATPASATLGRPPR

Spectrum No: 83; Query: 2883; Rank: 1

Peptide View

MS/MS Fragmentation of **DVFISAAER**
Found in **IPI00025019**, Tax_Id=9606 Gene_Symbol=PSMB1 Proteasome subunit beta type-1

Match to Query 2883: 1006.513348 from(504.263950,2+)
Title: 091224LimSK_Exosome3_07.1952.1952.2.dta
Data file C:\Temp\Mascoti\LSK_2D\091224LimSK_Exosome3_01_2D.mgf

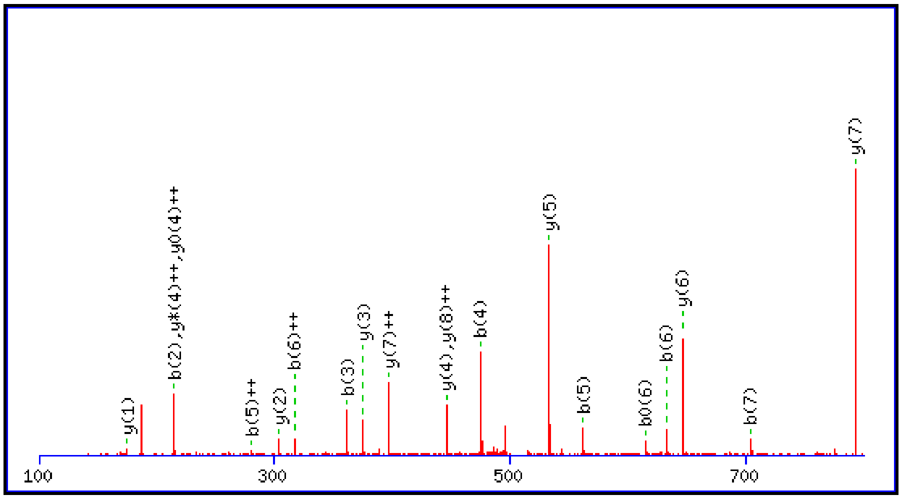

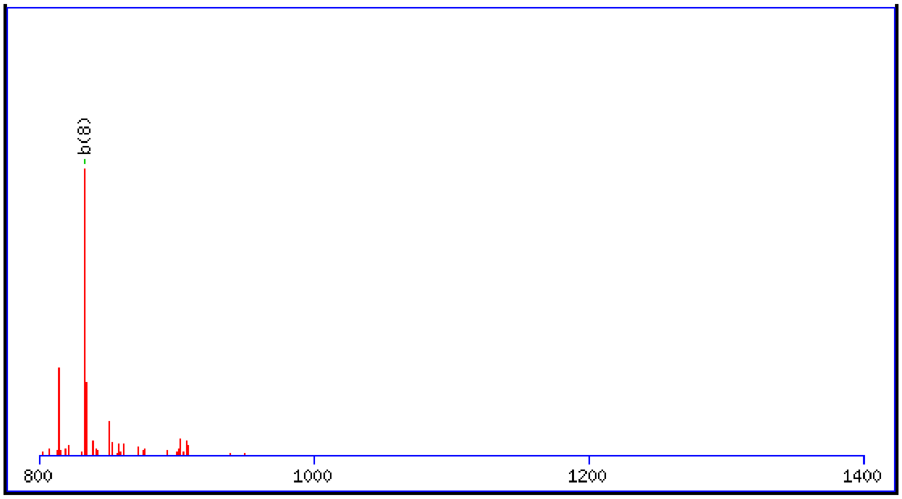

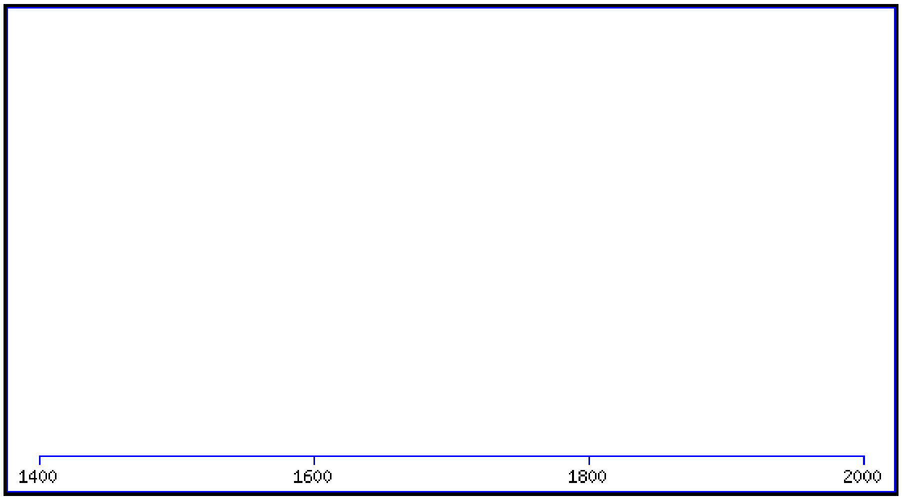

Monoisotopic mass of neutral peptide Mr(calc): 1006.5083
Fixed modifications: Carbamidomethyl (C)
Ions Score: 57 **Expect:** 0.00026
Matches (Bold Red): 21/78 fragment ions using 29 most intense peaks

#	b	b^{++}	b^0	b^{0++}	Seq.	y	y^{++}	y^*	y^{*++}	y^0	y^{0++}	#
1	116.0342	58.5207	98.0237	49.5155	D							9
2	**215.1026**	108.0550	197.0921	99.0497	V	892.4887	**446.7480**	875.4621	438.2347	874.4781	437.7427	8
3	**362.1710**	181.5892	344.1605	172.5839	F	**793.4203**	**397.2138**	776.3937	388.7005	775.4097	388.2085	7
4	**475.2551**	238.1312	457.2445	229.1259	I	**646.3519**	323.6796	629.3253	315.1663	628.3413	314.6743	6
5	**562.2871**	**281.6472**	544.2766	272.6419	S	**533.2678**	267.1375	516.2413	258.6243	515.2572	258.1323	5
6	**633.3243**	**317.1658**	**615.3137**	308.1605	A	**446.2358**	223.6215	429.2092	**215.1083**	428.2252	**214.6162**	4
7	**704.3614**	352.6843	686.3508	343.6790	A	**375.1987**	188.1030	358.1721	179.5897	357.1881	179.0977	3
8	**833.4040**	417.2056	815.3934	408.2003	E	**304.1615**	152.5844	287.1350	144.0711	286.1510	143.5791	2
9					R	**175.1190**	88.0631	158.0924	79.5498			1

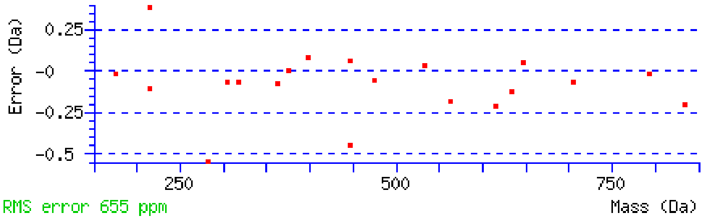

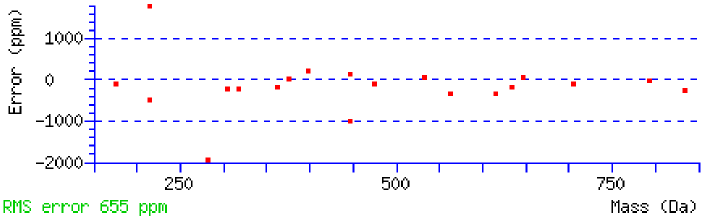

All matches to this query

Score	Mr(calc):	Delta	Sequence

56.9	1006.5083	0.0050	DVFISAAER
11.2	1006.5084	0.0050	DVAEVTAFR
11.2	1006.5083	0.0050	FLNVTEER
11.0	1004.5073	2.0060	VLCKGSGER
10.3	1005.5145	0.9989	GRFGEVWR
9.1	1006.5229	-0.0096	VNMSEKKR
7.7	1005.5165	0.9969	VIMITGDNK
5.7	1005.5121	1.0013	VPLTRGQR
5.1	1004.5096	2.0038	IFLTYIR
5.1	1006.5117	0.0016	MIDAVVTSR

Spectrum No: 84; Query: 2914; Rank: 1

Peptide View

MS/MS Fragmentation of **DVYTGDALR**
Found in **IPI00025019**, Tax_Id=9606 Gene_Symbol=PSMB1 Proteasome subunit beta type-1

Match to Query 2914: 1008.492288 from(505.253420,2+)
Title: 091224LimSK_Exosome3_07.1243.1243.2.dta
Data file C:\Temp\Mascoti\LSK_2D\091224LimSK_Exosome3_01_2D.mgf

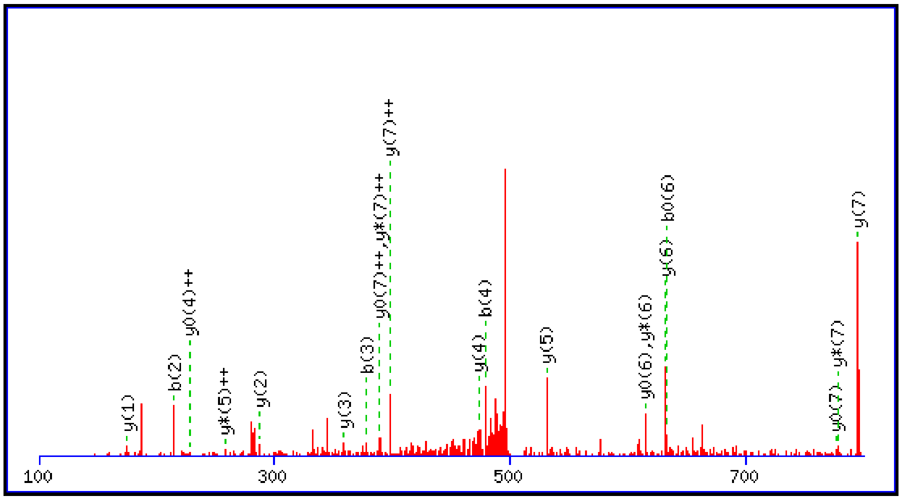

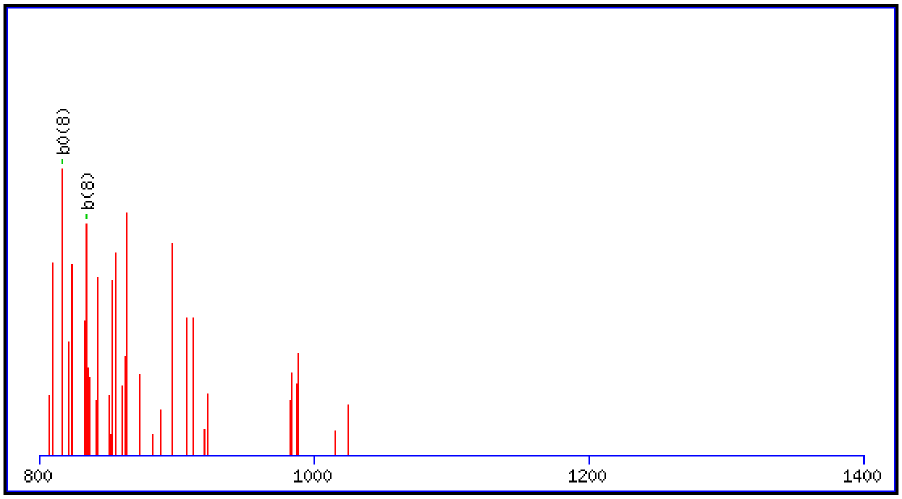

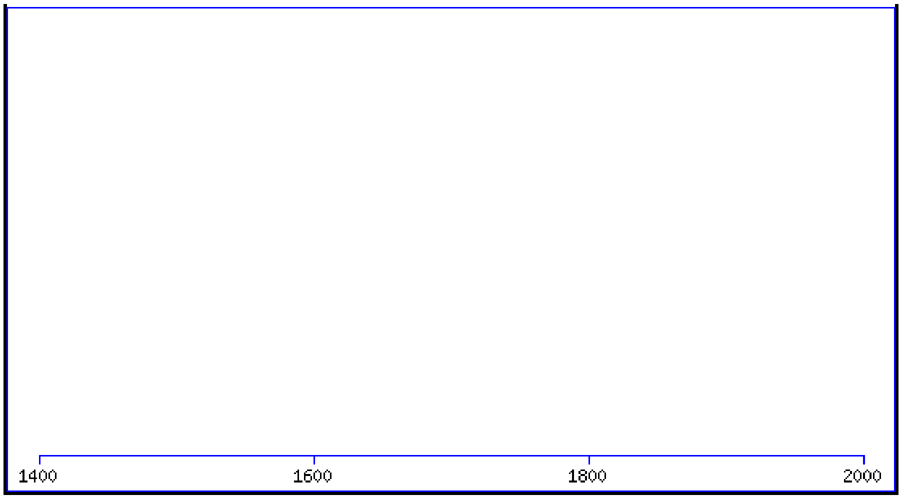

Monoisotopic mass of neutral peptide Mr(calc): 1008.4876
Fixed modifications: Carbamidomethyl (C)
Ions Score: 21 **Expect:** 0.95
Matches (Bold Red): 22/74 fragment ions using 81 most intense peaks

#	b	b^{++}	b^{0}	b^{0++}	Seq.	y	y^{++}	y*	y^{*++}	y^{0}	y^{0++}	#
1	116.0342	58.5207	98.0237	49.5155	D							9
2	**215.1026**	108.0550	197.0921	99.0497	V	894.4680	447.7376	877.4414	439.2243	876.4574	438.7323	8
3	**378.1660**	189.5866	360.1554	180.5813	Y	**795.3995**	**398.2034**	**778.3730**	**389.6901**	**777.3890**	**389.1981**	7
4	**479.2136**	240.1105	461.2031	231.1052	T	**632.3362**	316.6717	**615.3097**	308.1585	**614.3257**	307.6665	6
5	536.2351	268.6212	518.2245	259.6159	G	**531.2885**	266.1479	514.2620	**257.6346**	513.2780	257.1426	5
6	651.2620	326.1347	**633.2515**	317.1294	D	**474.2671**	237.6372	457.2405	229.1239	456.2565	**228.6319**	4
7	722.2992	361.6532	704.2886	352.6479	A	**359.2401**	180.1237	342.2136	171.6104			3
8	**835.3832**	418.1953	**817.3727**	409.1900	L	**288.2030**	144.6051	271.1765	136.0919			2
9					R	**175.1190**	88.0631	158.0924	79.5498			1

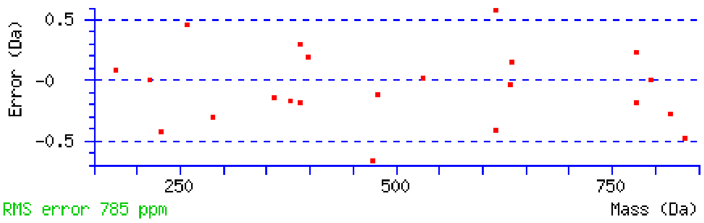

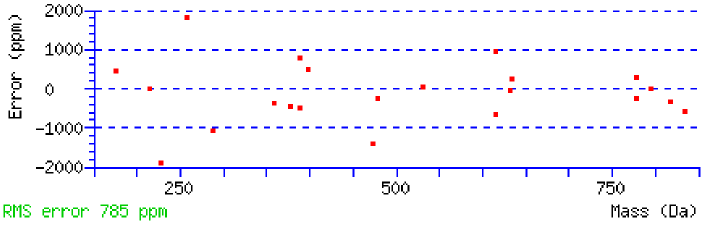

All matches to this query

Score	Mr(calc):	Delta	Sequence
20.8	1008.4876	0.0047	DVYTGDALR
8.4	1008.4866	0.0057	GRGVNTLGR
8.2	1007.4841	1.0082	KTFTLYR
7.1	1006.4822	2.0101	GPGTRGARR
6.7	1007.4801	1.0122	GKPPLSSSR
6.5	1008.4876	0.0046	VDYGDVSVR
6.3	1008.5005	-0.0082	ALLTLADGR
6.0	1008.4906	0.0017	TLTPWRR
6.0	1008.4876	0.0047	ITVDDYGAR
5.0	1007.4801	1.0122	VLGAPATSGR

Spectrum No: 85; Query: 10820; Rank: 1

Peptide View

MS/MS Fragmentation of **EGIREETVSLR**
Found in **IPI00025019**, Tax_Id=9606 Gene_Symbol=PSMB1 Proteasome subunit beta type-1

Match to Query 10820: 1287.686288 from(644.850420,2+)
Title: 091224LimSK_Exosome3_07.1247.1247.2.dta
Data file C:\Temp\Mascoti\LSK_2D\091224LimSK_Exosome3_01_2D.mgf

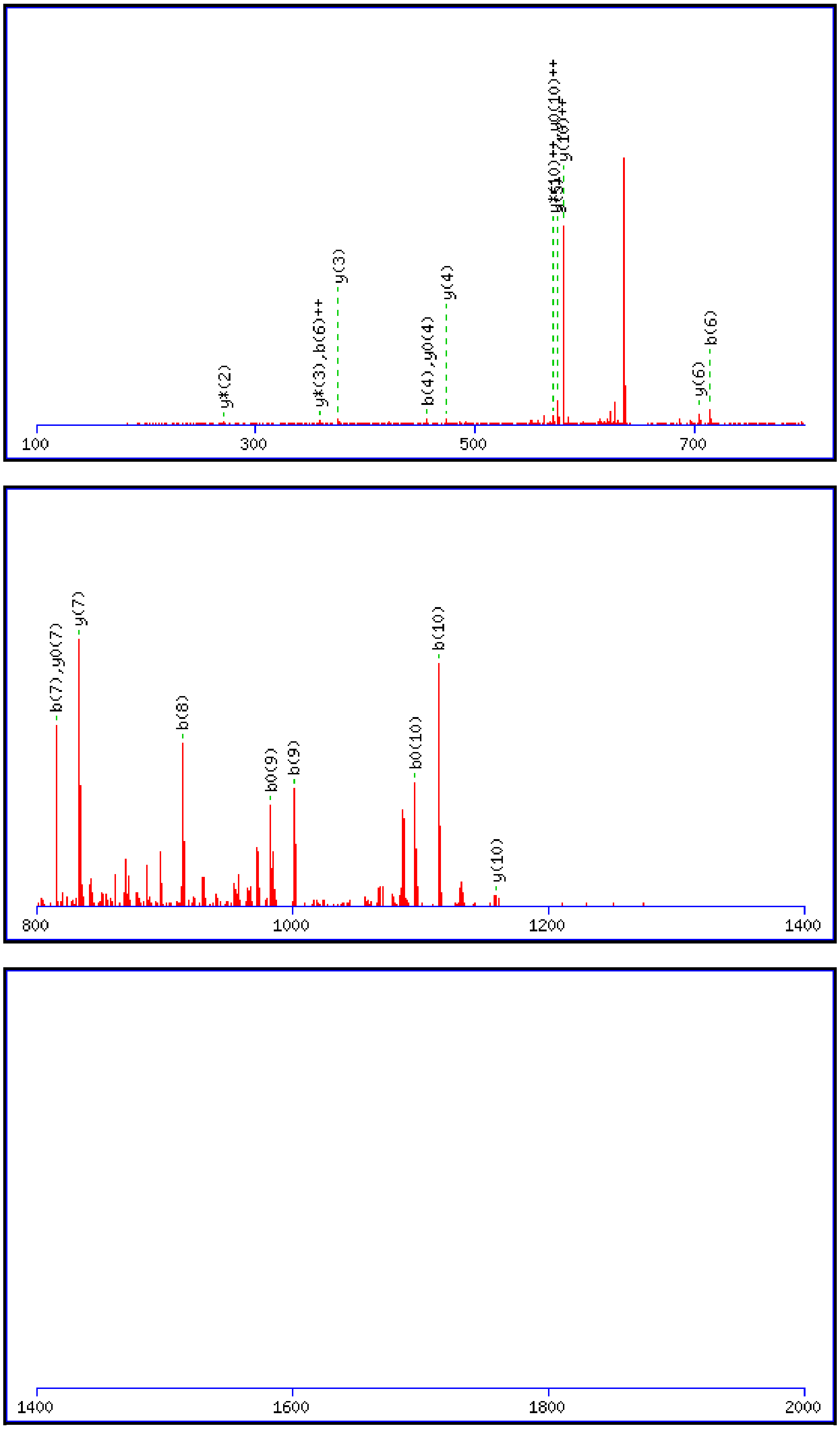

Monoisotopic mass of neutral peptide Mr(calc): 1287.6783
Fixed modifications: Carbamidomethyl (C)
Ions Score: 48 **Expect:** 0.0023
Matches (Bold Red): 22/110 fragment ions using 34 most intense peaks

#	b	b^{++}	b*	b^{*++}	b^0	b^{0++}	Seq.	y	y^{++}	y*	y^{*++}	y^0	y^{0++}	#
1	130.0499	65.5286			112.0393	56.5233	E							11
2	187.0713	94.0393			169.0608	85.0340	G	**1159.6430**	**580.3251**	1142.6164	**571.8118**	1141.6324	**571.3198**	10
3	300.1554	150.5813			282.1448	141.5761	I	1102.6215	551.8144	1085.5949	543.3011	1084.6109	542.8091	9
4	**456.2565**	228.6319	439.2300	220.1186	438.2459	219.6266	R	989.5374	495.2724	972.5109	486.7591	971.5269	486.2671	8
5	585.2991	293.1532	568.2726	284.6399	567.2885	284.1479	E	**833.4363**	417.2218	816.4098	408.7085	**815.4258**	408.2165	7
6	**714.3417**	**357.6745**	697.3151	349.1612	696.3311	348.6692	E	**704.3937**	352.7005	687.3672	344.1872	686.3832	343.6952	6
7	**815.3894**	408.1983	798.3628	399.6851	797.3788	399.1930	T	**575.3511**	288.1792	558.3246	279.6659	557.3406	279.1739	5
8	**914.4578**	457.7325	897.4312	449.2193	896.4472	448.7272	V	**474.3035**	237.6554	457.2769	229.1421	**456.2929**	228.6501	4
9	**1001.4898**	501.2485	984.4633	492.7353	**983.4792**	492.2433	S	**375.2350**	188.1212	**358.2085**	179.6079	357.2245	179.1159	3
10	**1114.5739**	557.7906	1097.5473	549.2773	**1096.5633**	548.7853	L	288.2030	144.6051	**271.1765**	136.0919			2
11							R	175.1190	88.0631	158.0924	79.5498			1

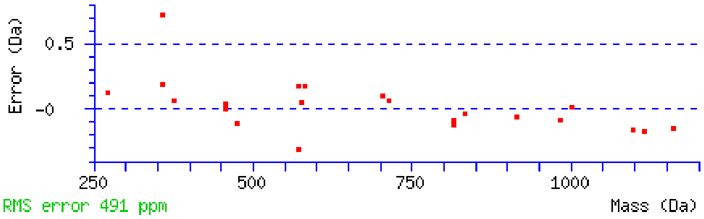

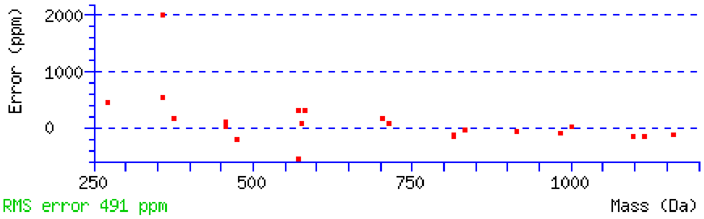

All matches to this query

Score	Mr(calc):	Delta	Sequence
47.7	1287.6783	0.0080	EGIREETVSLR
16.8	1287.6823	0.0040	SSEAVIWEVLR
14.3	1287.6823	0.0040	TDKELIDWLR
12.3	1287.6952	-0.0089	GPALSLLTPAIR
12.2	1286.6718	1.0145	EIPSLDQEKTK
10.6	1287.6952	-0.0089	GPALSLLTPAIR
9.3	1287.6758	0.0105	QHLLFMSQIR
9.2	1287.6782	0.0080	EASPAAKTKETR
6.9	1287.6857	0.0006	NQMNLLTLDVK
6.1	1287.6783	0.0080	EGTEASLQIRGK

Spectrum No: 86; Query: 44191; Rank: 1

Peptide View

MS/MS Fragmentation of **FFPYYVYNIIGGLDEEGK**
Found in **IPI00025019**, Tax_Id=9606 Gene_Symbol=PSMB1 Proteasome subunit beta type-1

Match to Query 44191: 2123.042028 from(1062.528290,2+)
Title: 091224LimSK_Exosome3_07.8986.8986.2.dta
Data file C:\Temp\Mascoti\LSK_2D\091224LimSK_Exosome3_01_2D.mgf

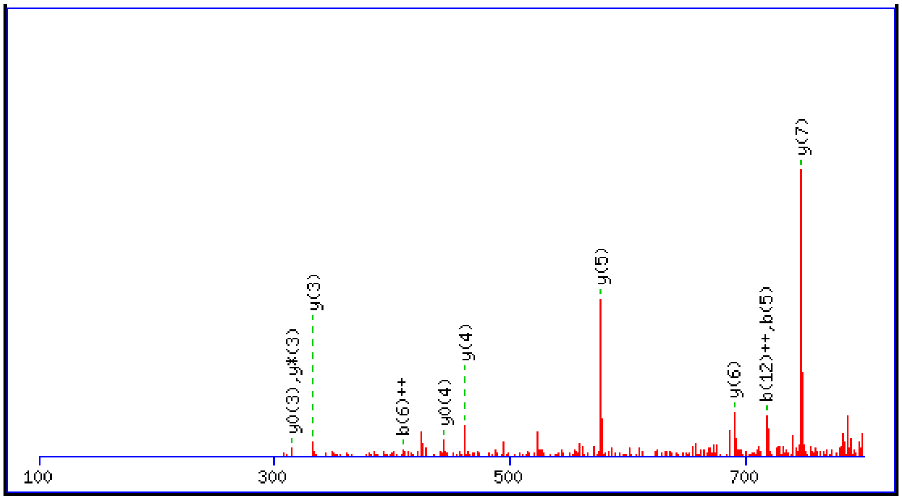

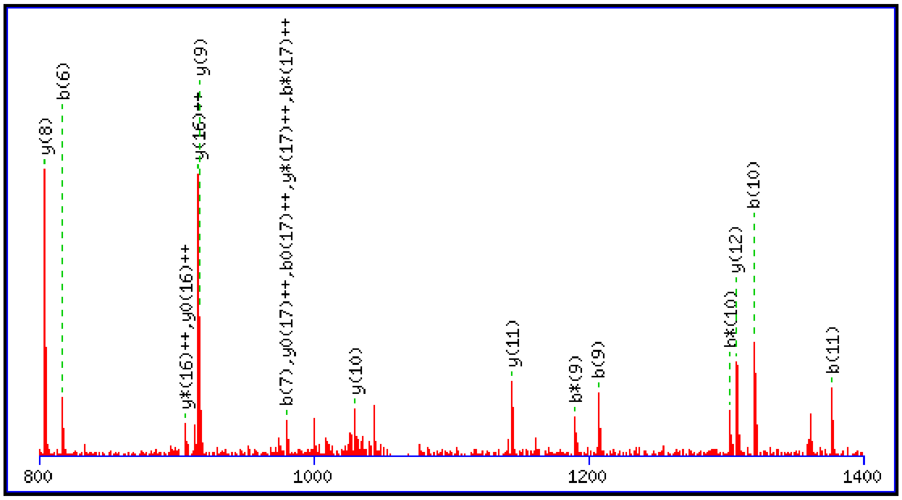

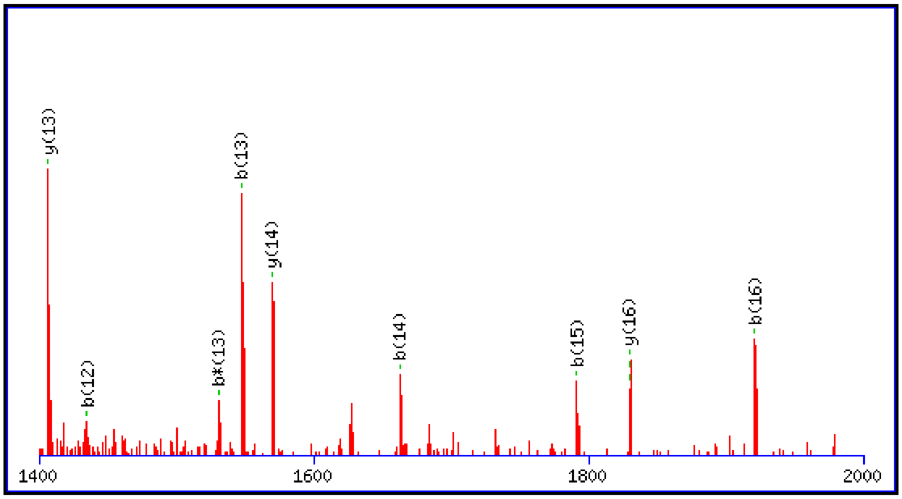

Monoisotopic mass of neutral peptide Mr(calc): 2123.0251
Fixed modifications: Carbamidomethyl (C)
Ions Score: 84 **Expect:** 9.5e-007
Matches (Bold Red): 39/160 fragment ions using 67 most intense peaks

#	b	b^{++}	b*	b^{*++}	b^0	b^{0++}	Seq.	y	y^{++}	y*	y^{*++}	y^0	y^{0++}	#
1	148.0757	74.5415					F							18
2	295.1441	148.0757					F	1976.9640	988.9856	1959.9375	**980.4724**	1958.9535	**979.9804**	17
3	392.1969	196.6021					P	**1829.8956**	**915.4514**	1812.8691	**906.9382**	1811.8850	**906.4462**	16
4	555.2602	278.1337					Y	1732.8428	866.9251	1715.8163	858.4118	1714.8323	857.9198	15
5	**718.3235**	359.6654					Y	**1569.7795**	785.3934	1552.7530	776.8801	1551.7690	776.3881	14
6	**817.3919**	**409.1996**					V	**1406.7162**	703.8617	1389.6896	695.3485	1388.7056	694.8564	13

7	**980.4553**	490.7313					Y	**1307.6478**	654.3275	1290.6212	645.8142	1289.6372	645.3222	12
8	1094.4982	547.7527	1077.4716	539.2395			N	**1144.5844**	572.7959	1127.5579	564.2826	1126.5739	563.7906	11
9	**1207.5823**	604.2948	**1190.5557**	595.7815			I	**1030.5415**	515.7744	1013.5150	507.2611	1012.5310	506.7691	10
10	**1320.6663**	660.8368	**1303.6398**	652.3235			I	**917.4575**	459.2324	900.4309	450.7191	899.4469	450.2271	9
11	**1377.6878**	689.3475	1360.6612	680.8343			G	**804.3734**	402.6903	787.3468	394.1771	786.3628	393.6850	8
12	**1434.7093**	**717.8583**	1417.6827	709.3450			G	**747.3519**	374.1796	730.3254	365.6663	729.3414	365.1743	7
13	**1547.7933**	774.4003	**1530.7668**	765.8870			L	**690.3305**	345.6689	673.3039	337.1556	672.3199	336.6636	6
14	**1662.8203**	831.9138	1645.7937	823.4005	1644.8097	822.9085	D	**577.2464**	289.1268	560.2198	280.6136	559.2358	280.1216	5
15	**1791.8629**	896.4351	1774.8363	887.9218	1773.8523	887.4298	E	**462.2195**	231.6134	445.1929	223.1001	**444.2089**	222.6081	4
16	**1920.9054**	960.9564	1903.8789	952.4431	1902.8949	951.9511	E	**333.1769**	167.0921	**316.1503**	158.5788	**315.1663**	158.0868	3
17	1977.9269	989.4671	1960.9004	**980.9538**	1959.9163	**980.4618**	G	204.1343	102.5708	187.1077	94.0575			2
18							K	147.1128	74.0600	130.0863	65.5468			1

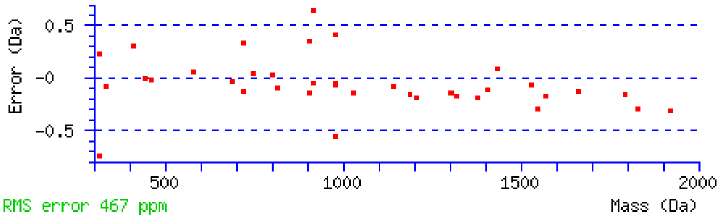

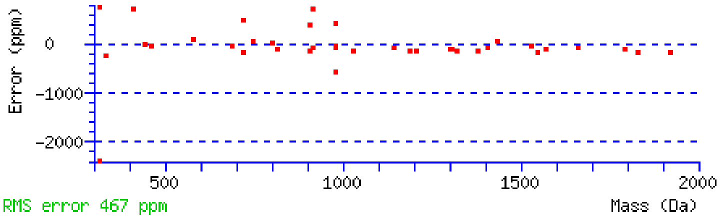

All matches to this query

Score	Mr(calc):	Delta	Sequence
83.8	2123.0251	0.0169	FFPYYVYNIIGGLDEEGK
10.8	2123.0364	0.0056	GSMGPMGPSPDLSHIKQGRR
5.8	2123.0535	-0.0115	IAIQQTYSWTNQIDISDK
4.6	2123.0470	-0.0049	QFSDVLDQISRNEKMWK
3.7	2123.0446	-0.0025	XSLMGLGDIISQQLVERR
3.6	2123.0364	0.0056	GSMGPMGPSPDLSHIKQGRR
3.5	2122.0267	1.0154	MASRSMRLLLLLSCLAK
3.3	2121.0412	2.0008	KSLMDIPESQSEQDFVLR
2.4	2122.0306	1.0115	AFLLMASQYQQPHEANKF
0.9	2121.0272	2.0148	LLSSLGIDTRNILFTGNK

Spectrum No: 87; Query: 22776; Rank: 1

Peptide View

MS/MS Fragmentation of **GAVYSFDPVGSYQR**
Found in **IPI00025019**, Tax_Id=9606 Gene_Symbol=PSMB1 Proteasome subunit beta type-1

Match to Query 22776: 1544.732648 from(773.373600,2+)
Title: 091224LimSK_Exosome3_07.3814.3814.2.dta
Data file C:\Temp\Mascoti\LSK_2D\091224LimSK_Exosome3_01_2D.mgf

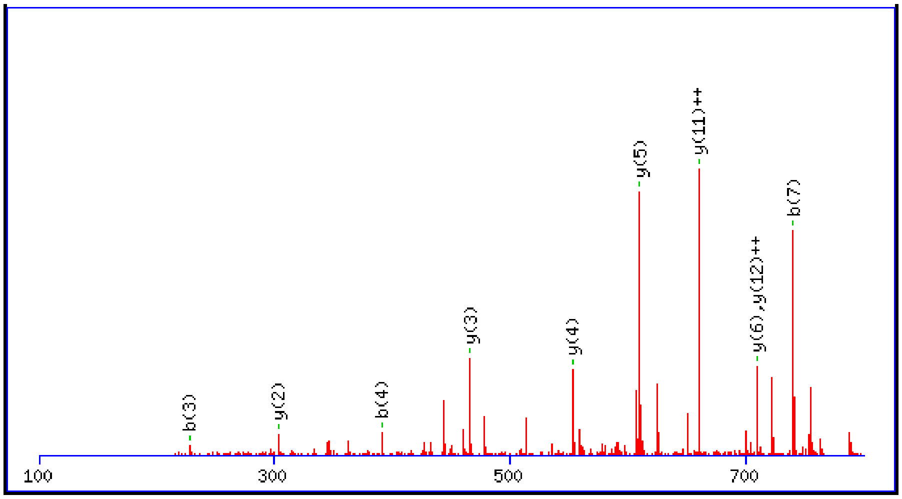

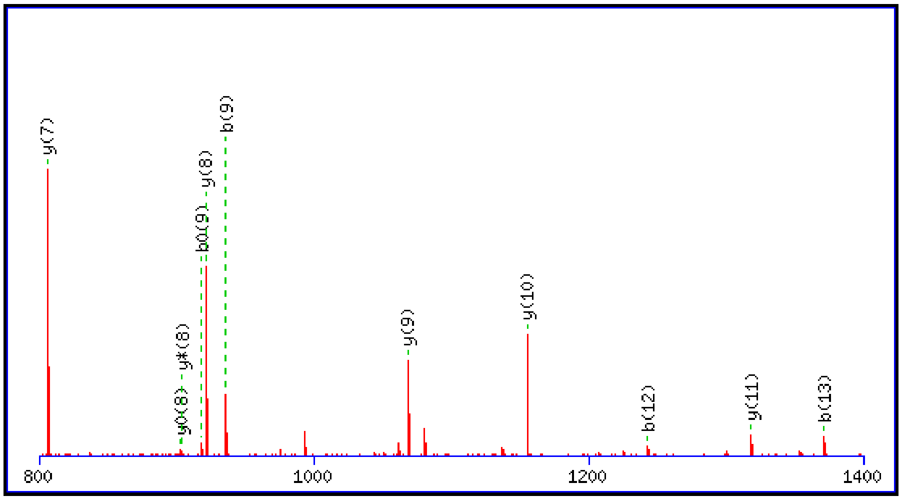

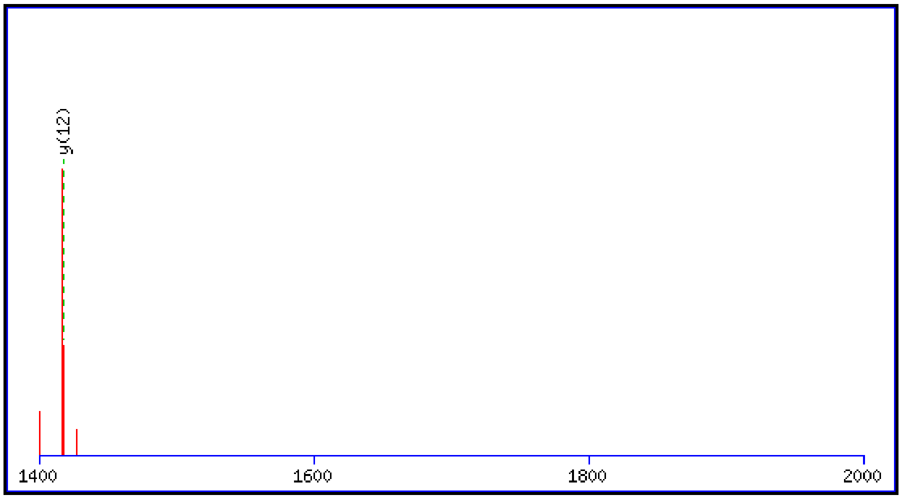

Monoisotopic mass of neutral peptide Mr(calc): 1544.7260
Fixed modifications: Carbamidomethyl (C)
Ions Score: 101 **Expect:** 1.8e-008
Matches (Bold Red): 22/118 fragment ions using 30 most intense peaks

#	b	b^{++}	b*	b^{*++}	b^{0}	b^{0++}	Seq.	y	y^{++}	y*	y^{*++}	y^{0}	y^{0++}	#
1	58.0287	29.5180					G							14
2	129.0659	65.0366					A	1488.7118	744.8595	1471.6852	736.3462	1470.7012	735.8542	13
3	**228.1343**	114.5708					V	**1417.6747**	**709.3410**	1400.6481	700.8277	1399.6641	700.3357	12
4	**391.1976**	196.1024					Y	**1318.6062**	**659.8068**	1301.5797	651.2935	1300.5957	650.8015	11
5	478.2296	239.6185			460.2191	230.6132	S	**1155.5429**	578.2751	1138.5164	569.7618	1137.5323	569.2698	10
6	625.2980	313.1527			607.2875	304.1474	F	**1068.5109**	534.7591	1051.4843	526.2458	1050.5003	525.7538	9

**7**	**740.3250**	370.6661			722.3144	361.6608	**D**	**921.4425**	461.2249	**904.4159**	452.7116	**903.4319**	452.2196	**8**
**8**	837.3777	419.1925			819.3672	410.1872	**P**	**806.4155**	403.7114	789.3890	395.1981	788.4050	394.7061	**7**
**9**	**936.4462**	468.7267			**918.4356**	459.7214	**V**	**709.3628**	355.1850	692.3362	346.6717	691.3522	346.1797	**6**
**10**	993.4676	497.2375			975.4571	488.2322	**G**	**610.2944**	305.6508	593.2678	297.1375	592.2838	296.6455	**5**
**11**	1080.4997	540.7535			1062.4891	531.7482	**S**	**553.2729**	277.1401	536.2463	268.6268	535.2623	268.1348	**4**
**12**	**1243.5630**	622.2851			1225.5524	613.2798	**Y**	**466.2409**	233.6241	449.2143	225.1108			**3**
**13**	**1371.6216**	686.3144	1354.5950	677.8011	1353.6110	677.3091	**Q**	**303.1775**	152.0924	286.1510	143.5791			**2**
**14**							**R**	175.1190	88.0631	158.0924	79.5498			**1**

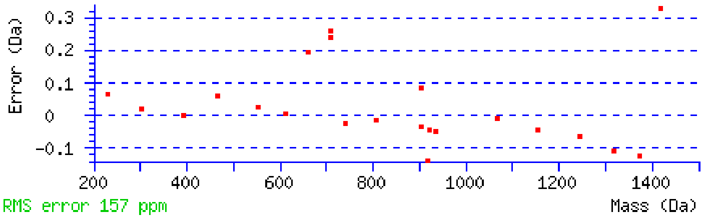

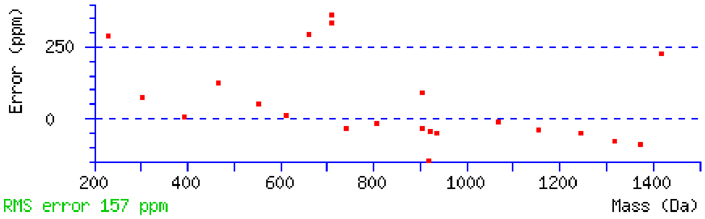

All matches to this query

Score	Mr(calc):	Delta	Sequence
100.5	1544.7260	0.0067	GAVYSFDPVGSYQR
8.0	1542.7249	2.0077	FCDRVQDAYTLR
6.5	1544.7357	-0.0030	KVVAVSMHPGPGMR
4.8	1543.7395	0.9931	ETAASLLQAGYKGR
4.5	1544.7236	0.0091	SFQDSLEDIKKR
4.4	1544.7364	-0.0038	KPATLSQIGTNKK
4.2	1544.7388	-0.0062	YKTHVDTVLAYR
4.1	1543.7283	1.0043	TLRYVPEESKDK
4.1	1543.7283	1.0043	TLRYVPEESKDK
3.8	1544.7388	-0.0062	YKTHVDTVLAYR

Spectrum No: 88; Query: 6117; Rank: 1

Peptide View

MS/MS Fragmentation of **LSEGFSIHTR**
Found in **IPI00025019**, Tax_Id=9606 Gene_Symbol=PSMB1 Proteasome subunit beta type-1

Match to Query 6117: 1145.589882 from(382.870570,3+)
Title: 091224LimSK_Exosome3_07.3308.3308.3.dta
Data file C:\Temp\Mascoti\LSK_2D\091224LimSK_Exosome3_01_2D.mgf

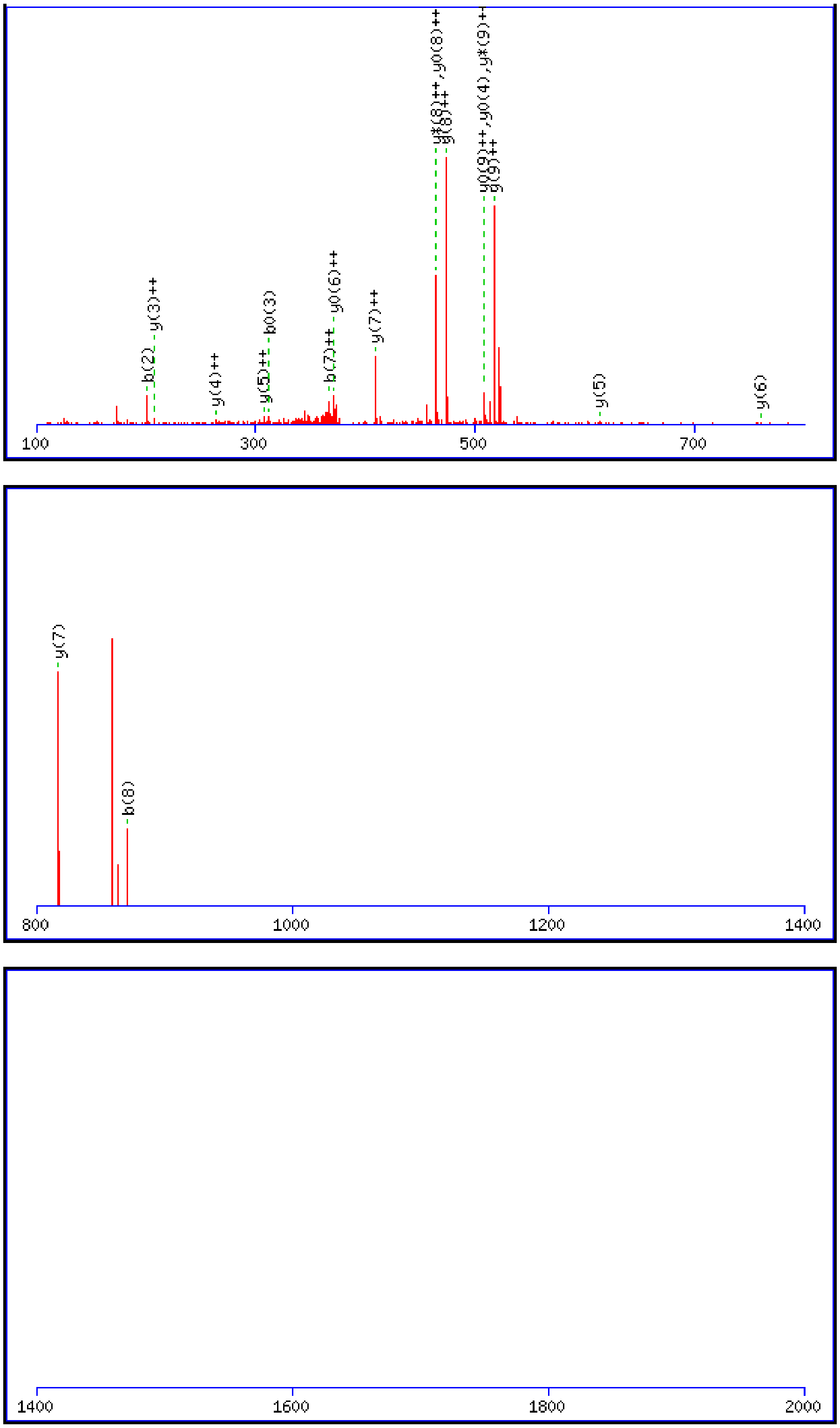

Monoisotopic mass of neutral peptide Mr(calc): 1145.5829
Fixed modifications: Carbamidomethyl (C)
Ions Score: 38 **Expect:** 0.023
Matches (Bold Red): 19/86 fragment ions using 45 most intense peaks

#	b	b^{++}	b^0	b^{0++}	Seq.	y	y^{++}	y^*	y^{*++}	y^0	y^{0++}	#
1	114.0913	57.5493			L							10
2	**201.1234**	101.0653	183.1128	92.0600	S	1033.5061	**517.2567**	1016.4796	**508.7434**	1015.4956	**508.2514**	9
3	330.1660	165.5866	**312.1554**	156.5813	E	946.4741	**473.7407**	929.4476	**465.2274**	928.4635	**464.7354**	8
4	387.1874	194.0974	369.1769	185.0921	G	**817.4315**	**409.2194**	800.4050	400.7061	799.4209	400.2141	7
5	534.2558	267.6316	516.2453	258.6263	F	**760.4100**	380.7087	743.3835	372.1954	742.3995	**371.7034**	6
6	621.2879	311.1476	603.2773	302.1423	S	**613.3416**	**307.1745**	596.3151	298.6612	595.3311	298.1692	5

7	734.3719	**367.6896**	716.3614	358.6843	I	526.3096	**263.6584**	509.2831	255.1452	**508.2990**	254.6532	4
8	**871.4308**	436.2191	853.4203	427.2138	H	413.2255	**207.1164**	396.1990	198.6031	395.2150	198.1111	3
9	972.4785	486.7429	954.4680	477.7376	T	276.1666	138.5870	259.1401	130.0737	258.1561	129.5817	2
10					R	175.1190	88.0631	158.0924	79.5498			1

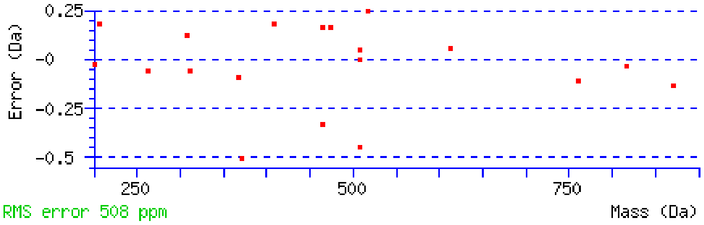

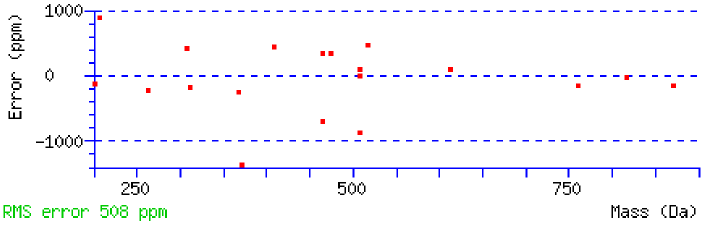

All matches to this query

Score	Mr(calc):	Delta	Sequence
37.6	1145.5829	0.0070	LSEGFSIHTR
12.2	1144.5976	0.9923	ISQLVDVDEK
8.0	1143.5745	2.0154	ERVGEGGRER
7.2	1144.5754	1.0145	TPRPPALGTR
6.7	1144.5754	1.0145	ISPGIRGTHK
5.8	1144.5794	1.0105	TKFWKTVR
5.4	1144.5754	1.0145	TPSPARPALR
5.1	1144.5893	1.0006	TYGITGVKVK
4.9	1145.5928	-0.0029	DGDLKTQIEK
4.8	1143.5884	2.0015	GVQLDSAGLER

Spectrum No: 89; Query: 27970; Rank: 1

Peptide View

MS/MS Fragmentation of **NMQNVEHVPLSLDR**
Found in **IPI00025019**, Tax_Id=9606 Gene_Symbol=PSMB1 Proteasome subunit beta type-1

Match to Query 27970: 1650.826928 from(826.420740,2+)
Title: 091224LimSK_Exosome3_07.3635.3635.2.dta
Data file C:\Temp\Mascoti\LSK_2D\091224LimSK_Exosome3_01_2D.mgf

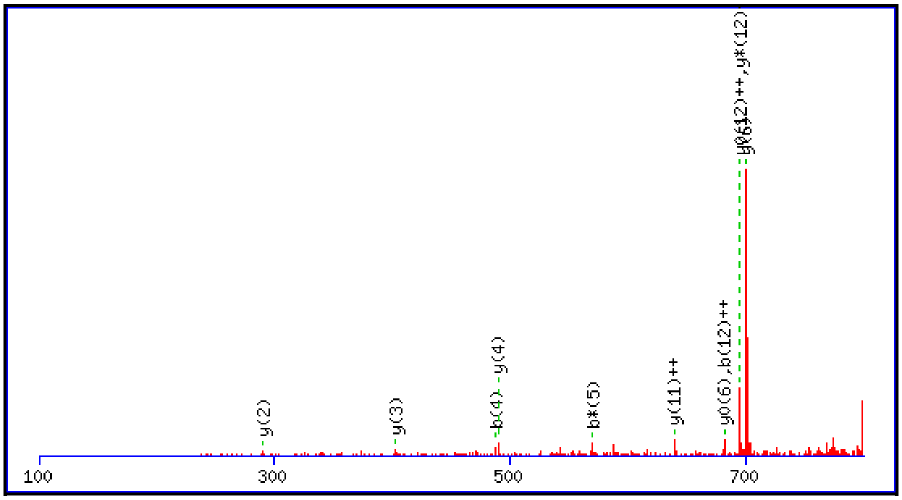

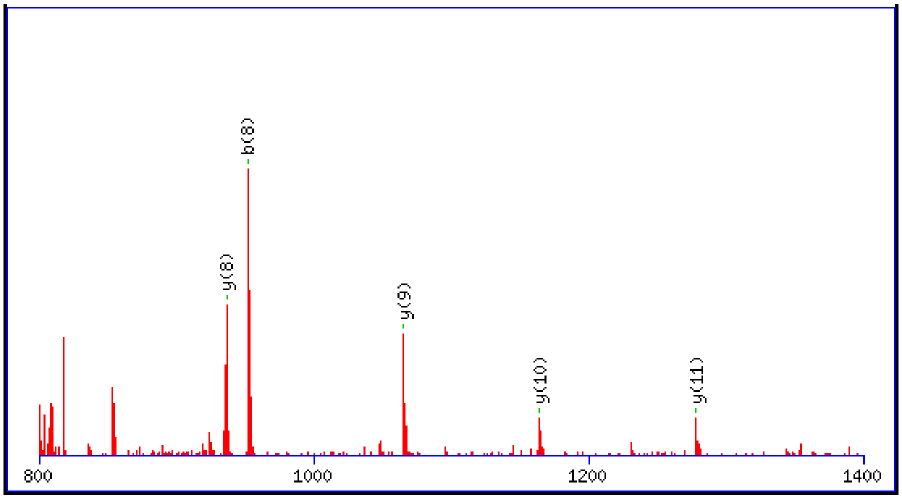

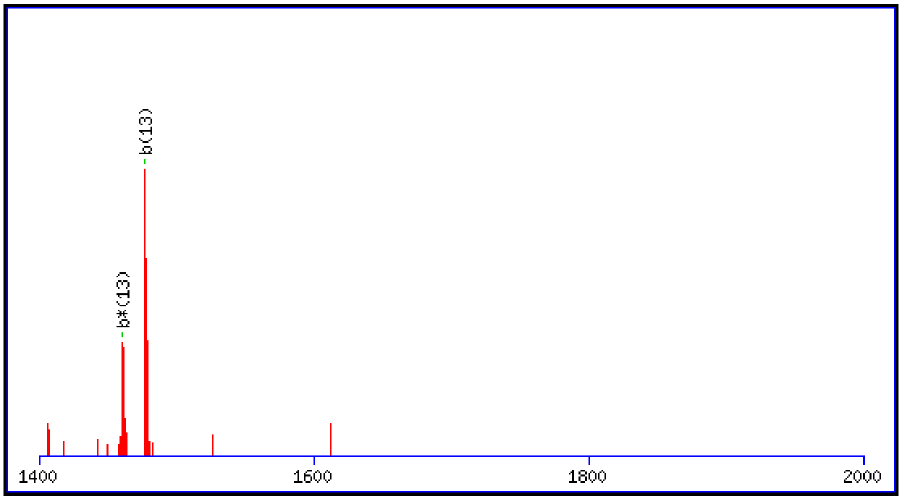

Monoisotopic mass of neutral peptide Mr(calc): 1650.8148
Fixed modifications: Carbamidomethyl (C)
Ions Score: 70 **Expect:** 2.1e-005
Matches (Bold Red): 18/144 fragment ions using 27 most intense peaks

#	b	b^{++}	b*	b^{*++}	b^0	b^{0++}	Seq.	y	y^{++}	y*	y^{*++}	y^0	y^{0++}	#
1	115.0502	58.0287	98.0237	49.5155			N							14
2	246.0907	123.5490	229.0641	115.0357			M	1537.7791	769.3932	1520.7526	760.8799	1519.7686	760.3879	13
3	374.1493	187.5783	357.1227	179.0650			Q	1406.7387	703.8730	1389.7121	**695.3597**	1388.7281	**694.8677**	12
4	**488.1922**	244.5997	471.1656	236.0865			N	**1278.6801**	**639.8437**	1261.6535	631.3304	1260.6695	630.8384	11
5	587.2606	294.1339	**570.2341**	285.6207			V	**1164.6371**	582.8222	1147.6106	574.3089	1146.6266	573.8169	10
6	716.3032	358.6552	699.2767	350.1420	698.2926	349.6500	E	**1065.5687**	533.2880	1048.5422	524.7747	1047.5582	524.2827	9
7	853.3621	427.1847	836.3356	418.6714	835.3515	418.1794	H	**936.5261**	468.7667	919.4996	460.2534	918.5156	459.7614	8
8	**952.4305**	476.7189	935.4040	468.2056	934.4200	467.7136	V	799.4672	400.2373	782.4407	391.7240	781.4567	391.2320	7
9	1049.4833	525.2453	1032.4567	516.7320	1031.4727	516.2400	P	**700.3988**	350.7030	683.3723	342.1898	**682.3882**	341.6978	6
10	1162.5674	581.7873	1145.5408	573.2740	1144.5568	572.7820	L	603.3461	302.1767	586.3195	293.6634	585.3355	293.1714	5
11	1249.5994	625.3033	1232.5728	616.7901	1231.5888	616.2980	S	**490.2620**	245.6346	473.2354	237.1214	472.2514	236.6293	4
12	1362.6834	**681.8454**	1345.6569	673.3321	1344.6729	672.8401	L	**403.2300**	202.1186	386.2034	193.6053	385.2194	193.1133	3
13	**1477.7104**	739.3588	**1460.6838**	730.8456	1459.6998	730.3535	D	**290.1459**	145.5766	273.1193	137.0633	272.1353	136.5713	2
14							R	175.1190	88.0631	158.0924	79.5498			1

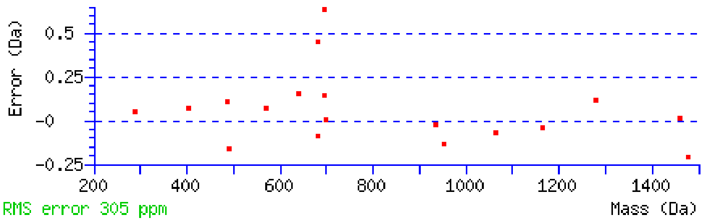

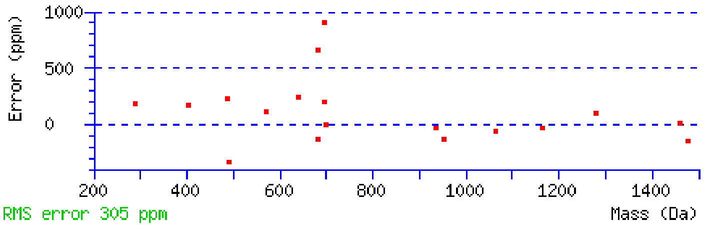

All matches to this query

Score	Mr(calc):	Delta	Sequence
69.8	1650.8148	0.0122	NMQNVEHVPLSLDR
7.8	1650.8416	-0.0146	VLEKQLPGSMTIQK
7.3	1648.8355	1.9914	GLHMKLDHDLSLDR
7.3	1650.8416	-0.0146	VLEKQLPGSMTIQK
6.6	1650.8131	0.0139	RFVAVPEGIPTETR
5.8	1650.8131	0.0139	RFVAVPEGIPTETR
3.8	1650.8416	-0.0146	SPLVLQSILSLMDR
3.2	1650.8160	0.0109	RHSIISVLPWKR
3.2	1650.8260	0.0009	GLPGADGRAGVMGPPGSR
3.1	1648.8321	1.9948	GNDNWLVRYEKQK

Spectrum No: 90; Query: 50223; Rank: 1

Peptide View

MS/MS Fragmentation of **RFFPYYVYNIIGGLDEEGK**
Found in **IPI00025019**, Tax_Id=9606 Gene_Symbol=PSMB1 Proteasome subunit beta type-1

Match to Query 50223: 2279.141292 from(760.721040,3+)
Title: 091224LimSK_Exosome3_08.8210.8210.3.dta
Data file C:\Temp\Mascoti\LSK_2D\091224LimSK_Exosome3_01_2D.mgf

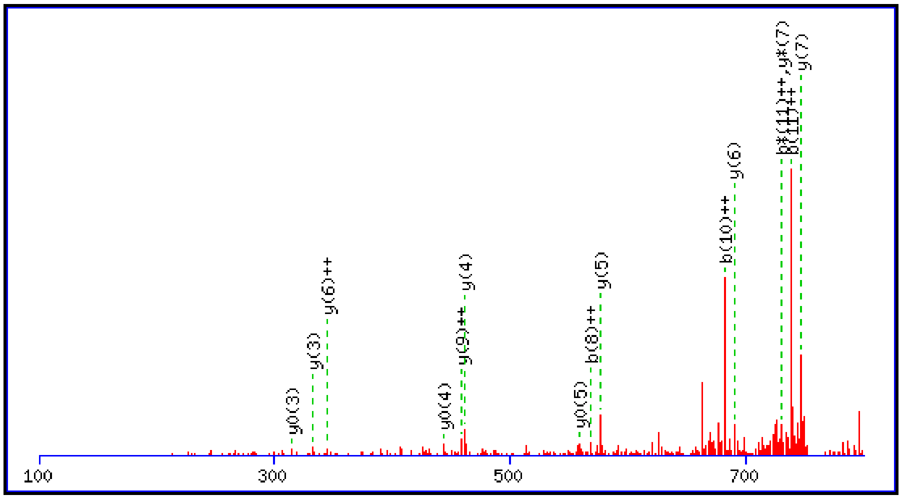

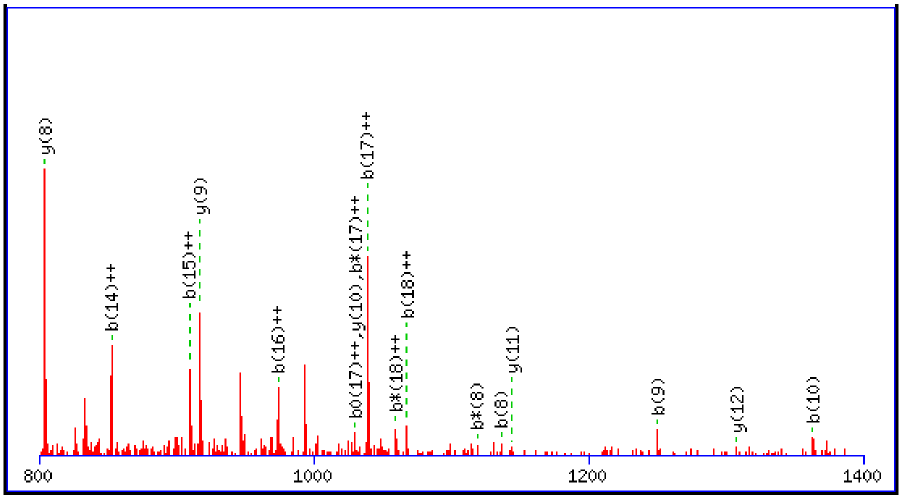

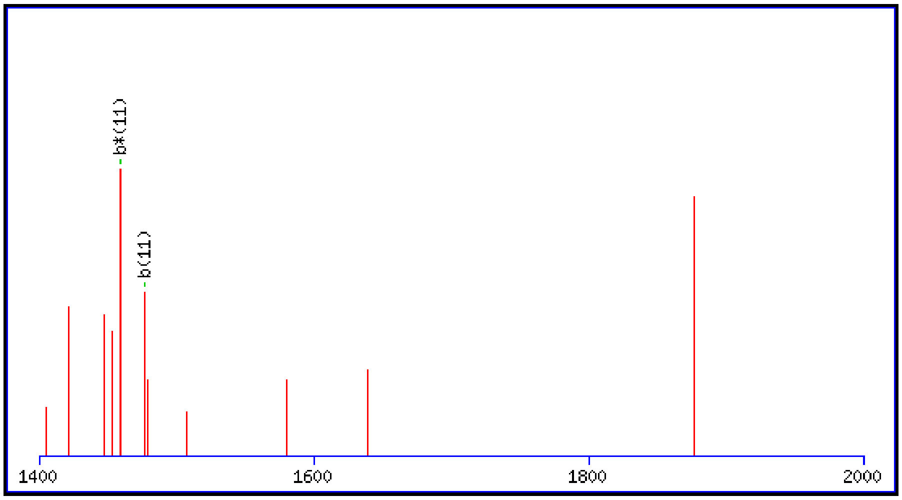

Monoisotopic mass of neutral peptide Mr(calc): 2279.1263
Fixed modifications: Carbamidomethyl (C)
Ions Score: 36 **Expect:** 0.046
Matches (Bold Red): 34/184 fragment ions using 77 most intense peaks

#	b	b^{++}	b*	b^{*++}	b^{0}	b^{0++}	Seq.	y	y^{++}	y*	y^{*++}	y^{0}	y^{0++}	#
1	157.1084	79.0578	140.0818	70.5446			R							19
2	304.1768	152.5920	287.1503	144.0788			F	2124.0324	1062.5199	2107.0059	1054.0066	2106.0219	1053.5146	18
3	451.2452	226.1262	434.2187	217.6130			F	1976.9640	988.9856	1959.9375	980.4724	1958.9535	979.9804	17
4	548.2980	274.6526	531.2714	266.1394			P	1829.8956	915.4514	1812.8691	906.9382	1811.8850	906.4462	16
5	711.3613	356.1843	694.3348	347.6710			Y	1732.8428	866.9251	1715.8163	858.4118	1714.8323	857.9198	15
6	874.4246	437.7160	857.3981	429.2027			Y	1569.7795	785.3934	1552.7530	776.8801	1551.7690	776.3881	14
7	973.4931	487.2502	956.4665	478.7369			V	1406.7162	703.8617	1389.6896	695.3485	1388.7056	694.8564	13
8	**1136.5564**	**568.7818**	**1119.5298**	560.2686			Y	**1307.6478**	654.3275	1290.6212	645.8142	1289.6372	645.3222	12
9	**1250.5993**	625.8033	1233.5728	617.2900			N	**1144.5844**	572.7959	1127.5579	564.2826	1126.5739	563.7906	11
10	**1363.6834**	**682.3453**	1346.6568	673.8320			I	**1030.5415**	515.7744	1013.5150	507.2611	1012.5310	506.7691	10
11	**1476.7674**	**738.8874**	**1459.7409**	**730.3741**			I	**917.4575**	**459.2324**	900.4309	450.7191	899.4469	450.2271	9
12	1533.7889	767.3981	1516.7623	758.8848			G	**804.3734**	402.6903	787.3468	394.1771	786.3628	393.6850	8
13	1590.8104	795.9088	1573.7838	787.3955			G	**747.3519**	374.1796	**730.3254**	365.6663	729.3414	365.1743	7
14	1703.8944	**852.4509**	1686.8679	843.9376			L	**690.3305**	**345.6689**	673.3039	337.1556	672.3199	336.6636	6
15	1818.9214	**909.9643**	1801.8948	901.4510	1800.9108	900.9590	D	**577.2464**	289.1268	560.2198	280.6136	**559.2358**	280.1216	5
16	1947.9640	**974.4856**	1930.9374	965.9723	1929.9534	965.4803	E	**462.2195**	231.6134	445.1929	223.1001	**444.2089**	222.6081	4
17	2077.0066	**1039.0069**	2059.9800	**1030.4936**	2058.9960	**1030.0016**	E	**333.1769**	167.0921	316.1503	158.5788	**315.1663**	158.0868	3
18	2134.0280	**1067.5176**	2117.0015	**1059.0044**	2116.0175	1058.5124	G	204.1343	102.5708	187.1077	94.0575			2
19							K	147.1128	74.0600	130.0863	65.5468			1

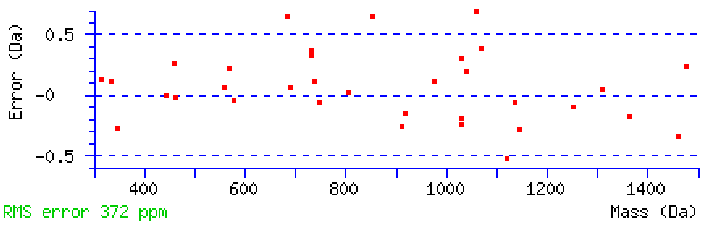

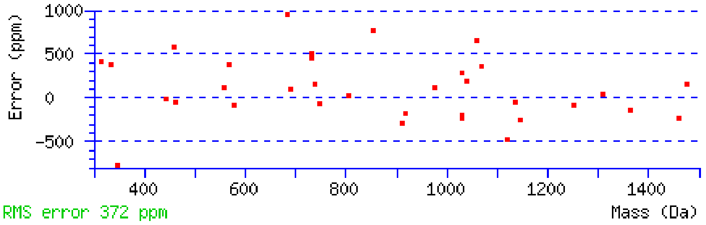

All matches to this query

Score	Mr(calc):	Delta	Sequence
36.3	2279.1263	0.0150	RFFPYYVYNIIGGLDEEGK
8.6	2279.1440	-0.0027	LSINTHPSQKPLSITVRTK
8.3	2277.1165	2.0248	XLEKNVKEVLEDFAEDGEK
7.2	2277.1365	2.0048	NSDQDVALKLAQERAEIVAK
7.0	2279.1596	-0.0183	IINDLLARSIGMVEVNTVDK
6.6	2277.1478	1.9935	ITLQTRLNQEALEDRLER
6.6	2277.1478	1.9935	ITLQTRLNQEALEDRLER
5.9	2278.1286	1.0127	FYFVSSADLLDILSKGAQPK
5.5	2278.1409	1.0004	SMSLDMGQPSQANTKKLLGTR
5.3	2277.1328	2.0085	TVTSSTEKGLIVYCVTSPKK

Spectrum No: 91; Query: 35461; Rank: 1

Peptide View

MS/MS Fragmentation of **TVIGCSGFHGDCLTLTK**
Found in **IPI00025019**, Tax_Id=9606 Gene_Symbol=PSMB1 Proteasome subunit beta type-1

Match to Query 35461: 1864.894248 from(933.454400,2+)
Title: 091224LimSK_Exosome3_07.3982.3982.2.dta
Data file C:\Temp\Mascoti\LSK_2D\091224LimSK_Exosome3_01_2D.mgf

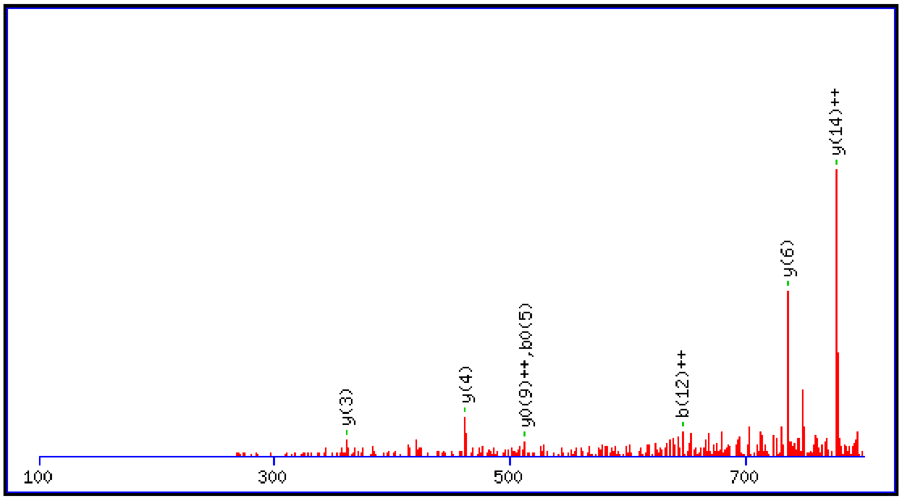

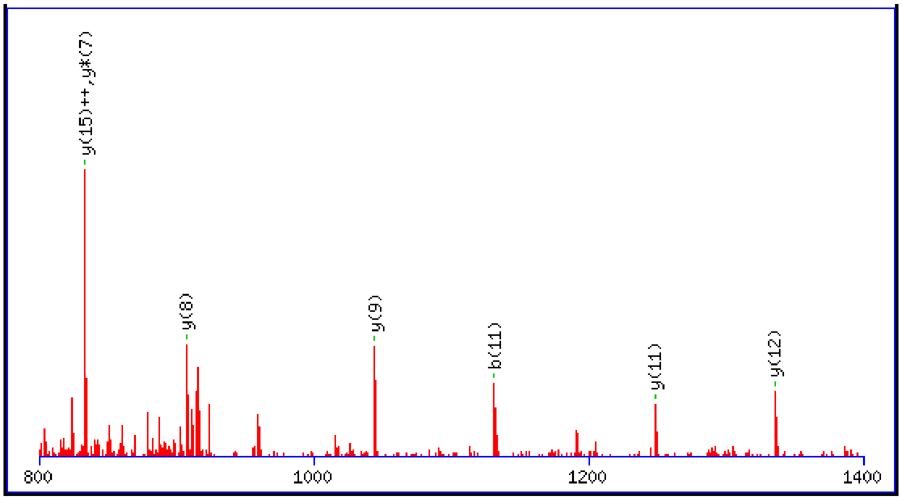

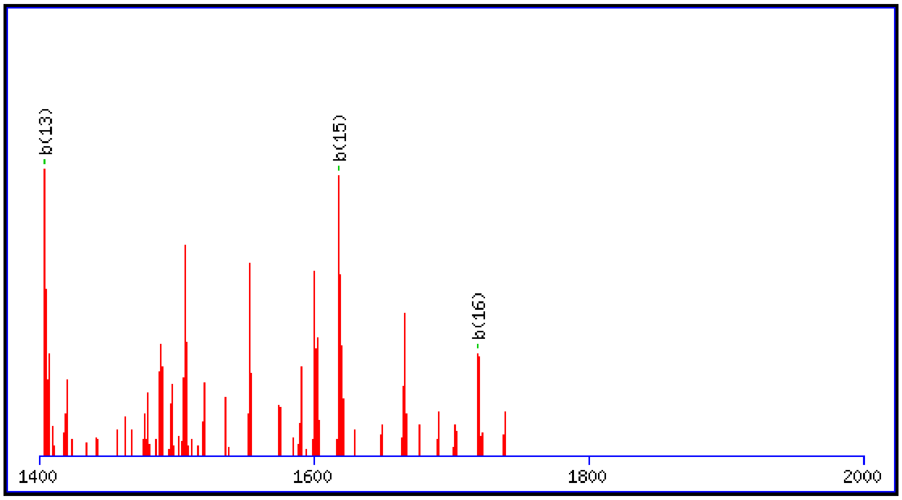

Monoisotopic mass of neutral peptide Mr(calc): 1864.8812
Fixed modifications: Carbamidomethyl (C)
Ions Score: 56 **Expect:** 0.00064
Matches (Bold Red): 17/158 fragment ions using 28 most intense peaks

#	b	b^{++}	b^0	b^{0++}	Seq.	y	y^{++}	y*	y^{*++}	y^0	y^{0++}	#
1	102.0550	51.5311	84.0444	42.5258	T							17
2	201.1234	101.0653	183.1128	92.0600	V	1764.8408	882.9240	1747.8142	874.4107	1746.8302	873.9187	16
3	314.2074	157.6074	296.1969	148.6021	I	1665.7723	**833.3898**	1648.7458	824.8765	1647.7618	824.3845	15
4	371.2289	186.1181	353.2183	177.1128	G	1552.6883	**776.8478**	1535.6617	768.3345	1534.6777	767.8425	14
5	531.2595	266.1334	**513.2490**	257.1281	C	1495.6668	748.3370	1478.6403	739.8238	1477.6562	739.3318	13
6	618.2916	309.6494	600.2810	300.6441	S	**1335.6362**	668.3217	1318.6096	659.8084	1317.6256	659.3164	12
7	675.3130	338.1602	657.3025	329.1549	G	**1248.6041**	624.8057	1231.5776	616.2924	1230.5936	615.8004	11
8	822.3815	411.6944	804.3709	402.6891	F	1191.5827	596.2950	1174.5561	587.7817	1173.5721	587.2897	10
9	959.4404	480.2238	941.4298	471.2185	H	**1044.5143**	522.7608	1027.4877	514.2475	1026.5037	**513.7555**	9
10	1016.4618	508.7346	998.4513	499.7293	G	**907.4553**	454.2313	890.4288	445.7180	889.4448	445.2260	8
11	**1131.4888**	566.2480	1113.4782	557.2427	D	850.4339	425.7206	**833.4073**	417.2073	832.4233	416.7153	7
12	1291.5194	**646.2633**	1273.5089	637.2581	C	**735.4069**	368.2071	718.3804	359.6938	717.3964	359.2018	6
13	**1404.6035**	702.8054	1386.5929	693.8001	L	575.3763	288.1918	558.3497	279.6785	557.3657	279.1865	5
14	1505.6512	753.3292	1487.6406	744.3239	T	**462.2922**	231.6498	445.2657	223.1365	444.2817	222.6445	4
15	**1618.7352**	809.8713	1600.7247	800.8660	L	**361.2445**	181.1259	344.2180	172.6126	343.2340	172.1206	3
16	**1719.7829**	860.3951	1701.7723	851.3898	T	248.1605	124.5839	231.1339	116.0706	230.1499	115.5786	2
17					K	147.1128	74.0600	130.0863	65.5468			1

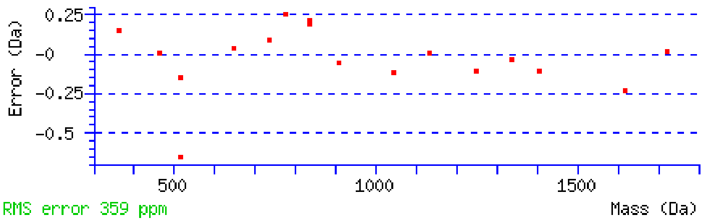

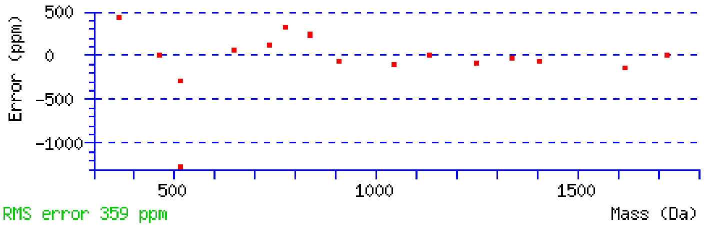

All matches to this query

Score	Mr(calc):	Delta	Sequence
55.7	1864.8812	0.0131	TVIGCSGFHGDCLTLTK
5.8	1864.9100	-0.0158	AKILIDSIYKVTEGR
3.5	1864.9118	-0.0175	LSLSLSPSPQTGKPSMR
3.3	1862.9000	1.9943	RSLPTGSPTRPTASEAR
2.0	1864.8932	0.0011	SRDSQVPVTSSVVPEAK
1.6	1864.8989	-0.0047	GLDKMFLSSVGSGHGETK
1.1	1864.8866	0.0077	TVTLSIALGKVASPLR
1.1	1862.8849	2.0094	SFQVTKEIISECKSK
0.7	1864.8961	-0.0019	VSSFLRNVRANLTTK
0.5	1862.8849	2.0094	SFQVTKEIISECKSK

Spectrum No: 92; Query: 38393; Rank: 1

Peptide View

MS/MS Fragmentation of **AIGSASEGAQSSLQEVYHK**
Found in **IPI00291922**, Tax_Id=9606 Gene_Symbol=PSMA5 Proteasome subunit alpha type-5

Match to Query 38393: 1960.962328 from(981.488440,2+)
Title: 091224LimSK_Exosome3_05.2179.2179.2.dta
Data file C:\Temp\Mascoti\LSK_2D\091224LimSK_Exosome3_01_2D.mgf

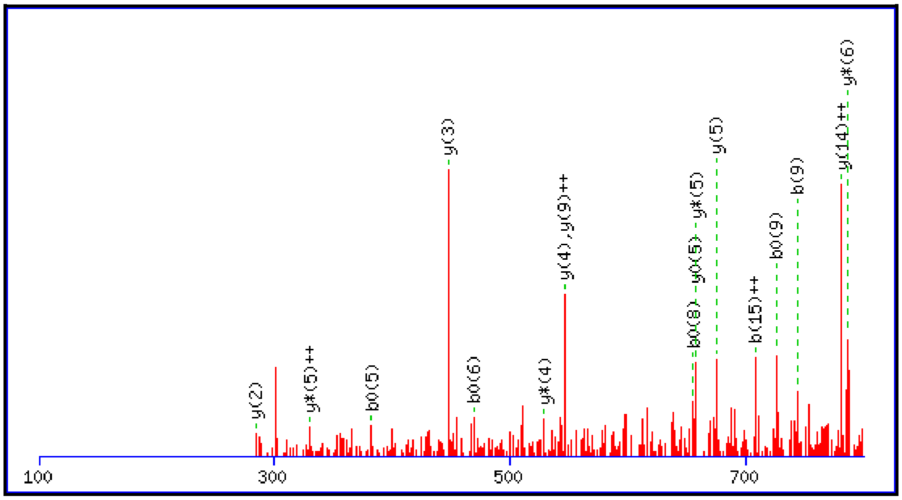

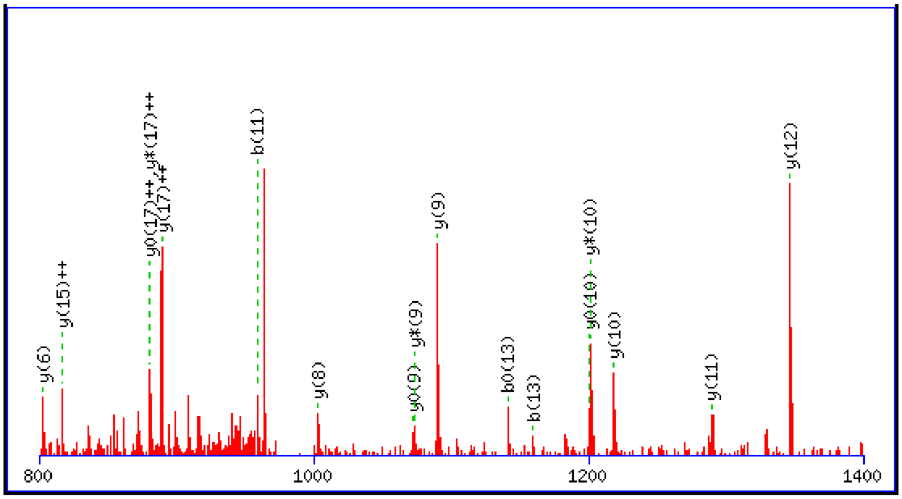

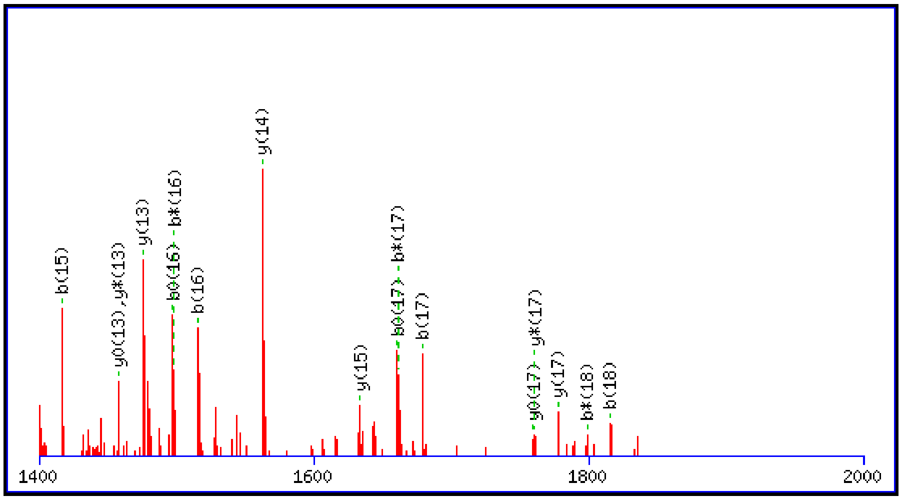

Monoisotopic mass of neutral peptide Mr(calc): 1960.9490
Fixed modifications: Carbamidomethyl (C)
Ions Score: 93 **Expect:** 1.1e-007
Matches (Bold Red): 51/184 fragment ions using 83 most intense peaks

#	b	b^{++}	b*	b^{*++}	b^0	b^{0++}	Seq.	y	y^{++}	y*	y^{*++}	y^0	y^{0++}	#
1	72.0444	36.5258					A							19
2	185.1285	93.0679					I	1890.9192	945.9632	1873.8926	937.4500	1872.9086	936.9580	18
3	242.1499	121.5786					G	**1777.8351**	**889.4212**	**1760.8086**	**880.9079**	**1759.8246**	**880.4159**	17
4	329.1819	165.0946			311.1714	156.0893	S	1720.8137	860.9105	1703.7871	852.3972	1702.8031	851.9052	16
5	400.2191	200.6132			**382.2085**	191.6079	A	**1633.7816**	**817.3945**	1616.7551	808.8812	1615.7711	808.3892	15
6	487.2511	244.1292			**469.2405**	235.1239	S	**1562.7445**	**781.8759**	1545.7180	773.3626	1544.7340	772.8706	14
7	616.2937	308.6505			598.2831	299.6452	E	**1475.7125**	738.3599	**1458.6860**	729.8466	**1457.7019**	729.3546	13
8	673.3151	337.1612			**655.3046**	328.1559	G	**1346.6699**	673.8386	1329.6434	665.3253	1328.6593	664.8333	12
9	**744.3523**	372.6798			**726.3417**	363.6745	A	**1289.6484**	645.3279	1272.6219	636.8146	1271.6379	636.3226	11
10	872.4108	436.7091	855.3843	428.1958	854.4003	427.7038	Q	**1218.6113**	609.8093	**1201.5848**	601.2960	**1200.6008**	600.8040	10
11	**959.4429**	480.2251	942.4163	471.7118	941.4323	471.2198	S	**1090.5528**	**545.7800**	**1073.5262**	537.2667	**1072.5422**	536.7747	9
12	1046.4749	523.7411	1029.4483	515.2278	1028.4643	514.7358	S	**1003.5207**	502.2640	986.4942	493.7507	985.5102	493.2587	8
13	**1159.5590**	580.2831	1142.5324	571.7698	**1141.5484**	571.2778	L	916.4887	458.7480	899.4621	450.2347	898.4781	449.7427	7
14	1287.6175	644.3124	1270.5910	635.7991	1269.6070	635.3071	Q	**803.4046**	402.2060	**786.3781**	393.6927	785.3941	393.2007	6
15	**1416.6601**	**708.8337**	1399.6336	700.3204	1398.6496	699.8284	E	**675.3461**	338.1767	**658.3195**	**329.6634**	**657.3355**	329.1714	5
16	**1515.7285**	758.3679	**1498.7020**	749.8546	**1497.7180**	749.3626	V	**546.3035**	273.6554	**529.2769**	265.1421			4
17	**1678.7919**	839.8996	**1661.7653**	831.3863	**1660.7813**	830.8943	Y	**447.2350**	224.1212	430.2085	215.6079			3
18	**1815.8508**	908.4290	**1798.8242**	899.9158	1797.8402	899.4237	H	**284.1717**	142.5895	267.1452	134.0762			2
19							K	147.1128	74.0600	130.0863	65.5468			1

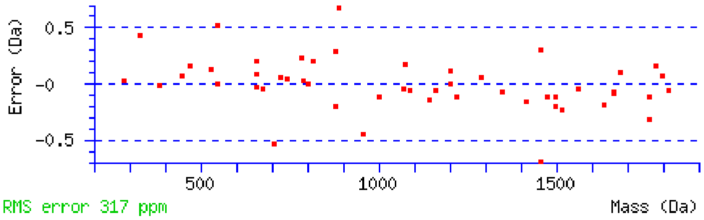

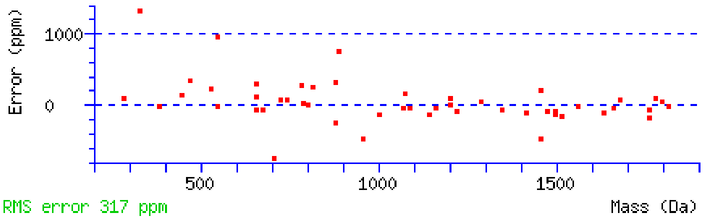

All matches to this query

Score	Mr(calc):	Delta	Sequence
93.4	1960.9490	0.0133	AIGSASEGAQSSLQEVYHK
4.0	1960.9710	-0.0087	QLSAQGQMTQLCELINK
4.0	1960.9636	-0.0013	SMNDISLTPNTDQRKNK
2.9	1960.9547	0.0077	VSEAKPSQYYELQVLK
2.9	1960.9547	0.0077	VSEAKPSQYYELQVLK
1.8	1960.9520	0.0103	RPHGDIYGINQALGATAK
1.8	1960.9809	-0.0186	SFHHLWPQGQWASKPR

Spectrum No: 93; Query: 8487; Rank: 1

Peptide View

MS/MS Fragmentation of **EELEEVIKDI**
Found in **IPI00291922**, Tax_Id=9606 Gene_Symbol=PSMA5 Proteasome subunit alpha type-5

Match to Query 8487: 1215.629328 from(608.821940,2+)
Title: 091224LimSK_Exosome3_06.6218.6218.2.dta
Data file C:\Temp\Mascoti\LSK_2D\091224LimSK_Exosome3_01_2D.mgf

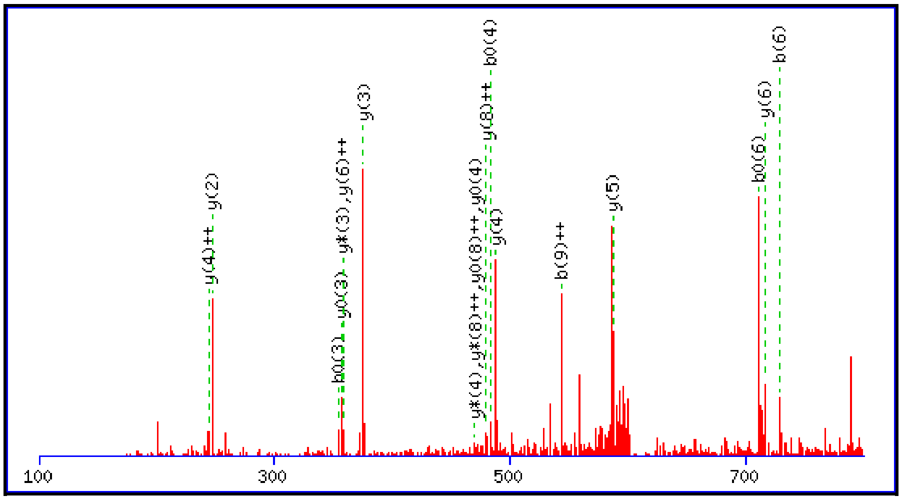

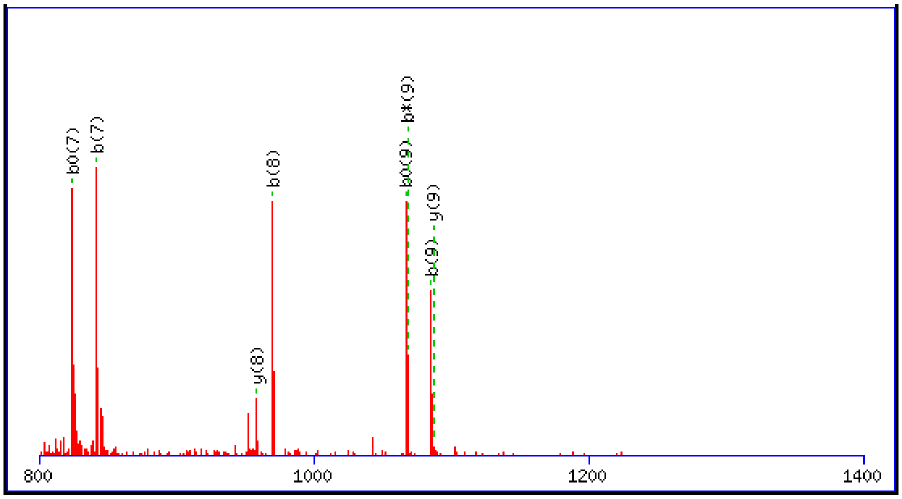

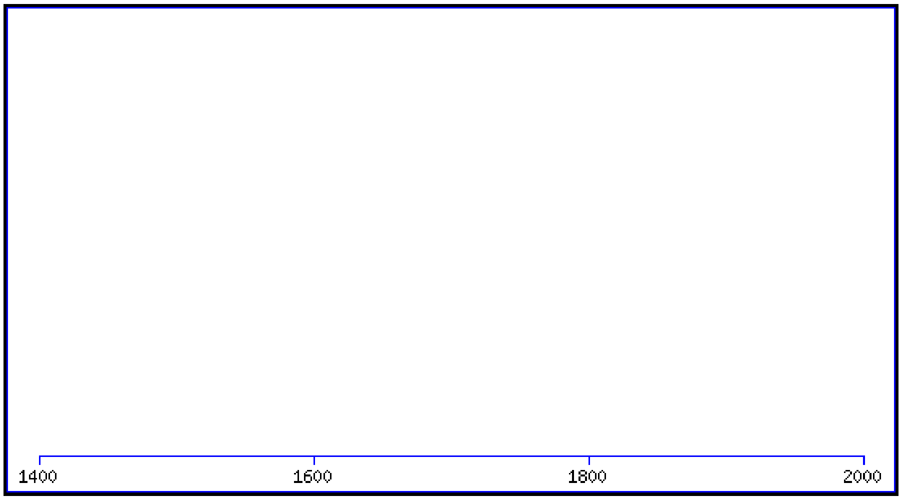

Monoisotopic mass of neutral peptide Mr(calc): 1215.6234
Fixed modifications: Carbamidomethyl (C)
Ions Score: 43 **Expect:** 0.0068
Matches (Bold Red): 27/88 fragment ions using 48 most intense peaks

#	b	b^{++}	b*	b^{*++}	b^0	b^{0++}	Seq.	y	y^{++}	y*	y^{*++}	y^0	y^{0++}	#
1	130.0499	65.5286			112.0393	56.5233	E							10
2	259.0925	130.0499			241.0819	121.0446	E	**1087.5881**	544.2977	1070.5616	535.7844	1069.5776	535.2924	9
3	372.1765	186.5919			**354.1660**	177.5866	L	**958.5455**	**479.7764**	941.5190	**471.2631**	940.5350	**470.7711**	8
4	501.2191	251.1132			**483.2086**	242.1079	E	845.4615	423.2344	828.4349	414.7211	827.4509	414.2291	7
5	630.2617	315.6345			612.2511	306.6292	E	**716.4189**	**358.7131**	699.3923	350.1998	698.4083	349.7078	6
6	**729.3301**	365.1687			**711.3196**	356.1634	V	**587.3763**	294.1918	570.3497	285.6785	569.3657	285.1865	5
7	**842.4142**	421.7107			**824.4036**	412.7055	I	**488.3079**	**244.6576**	**471.2813**	236.1443	**470.2973**	235.6523	4
8	**970.5092**	485.7582	953.4826	477.2449	952.4986	476.7529	K	**375.2238**	188.1155	**358.1973**	179.6023	**357.2132**	179.1103	3
9	**1085.5361**	**543.2717**	**1068.5095**	534.7584	**1067.5255**	534.2664	D	**247.1288**	124.0681			229.1183	115.0628	2
10							I	132.1019	66.5546					1

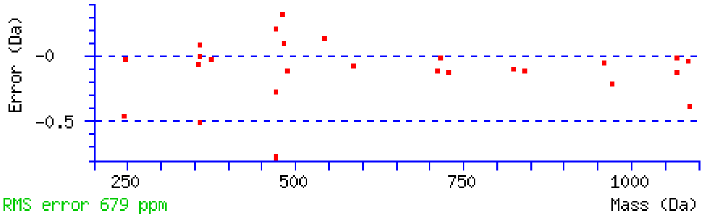

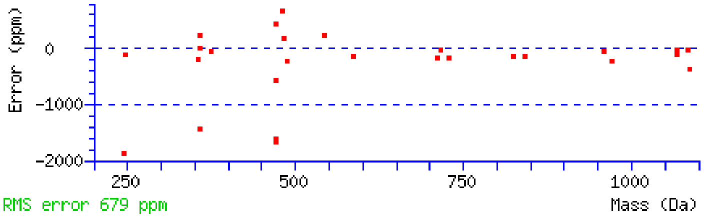

All matches to this query

Score	Mr(calc):	Delta	Sequence
43.5	1215.6234	0.0059	EELEEVIKDI
16.8	1213.6294	1.9999	MVKLVWKSK
14.5	1214.6329	0.9964	IQMINGSDLPK
13.5	1214.6367	0.9926	DKERAELAAGR
13.3	1213.6107	2.0186	YTPKEQLKK
11.8	1215.6281	0.0012	MIQDSGKVNPK
9.9	1215.6281	0.0012	MALTPTNLNNK
9.4	1213.6190	2.0103	EVEEKQPEVK
8.0	1215.6394	-0.0100	MAPERVASLSR
7.3	1213.6199	2.0095	GAVAMAPEMLPK

Spectrum No: 94; Query: 45554; Rank: 1

Peptide View

MS/MS Fragmentation of **GPQLFHMDPSGTFVQCDAR**
Found in **IPI00291922**, Tax_Id=9606 Gene_Symbol=PSMA5 Proteasome subunit alpha type-5

Match to Query 45554: 2161.979982 from(721.667270,3+)
Title: 091224LimSK_Exosome3_07.5269.5269.3.dta
Data file C:\Temp\Mascoti\LSK_2D\091224LimSK_Exosome3_01_2D.mgf

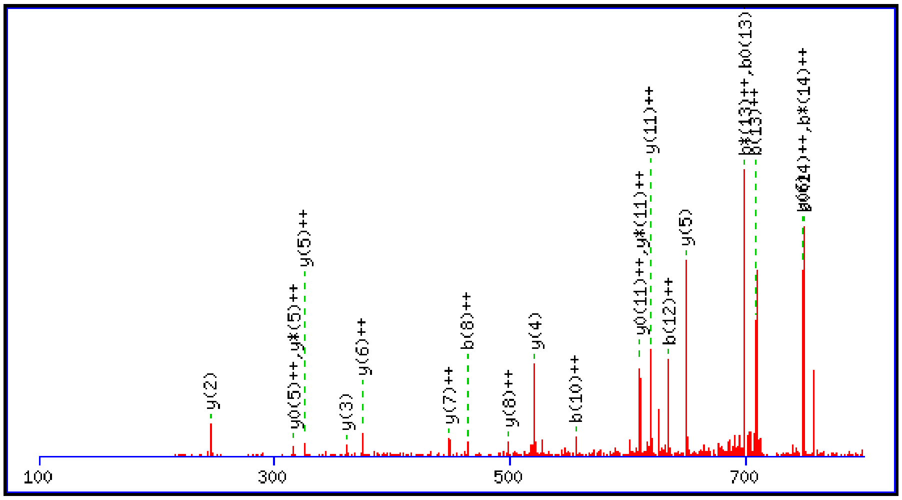

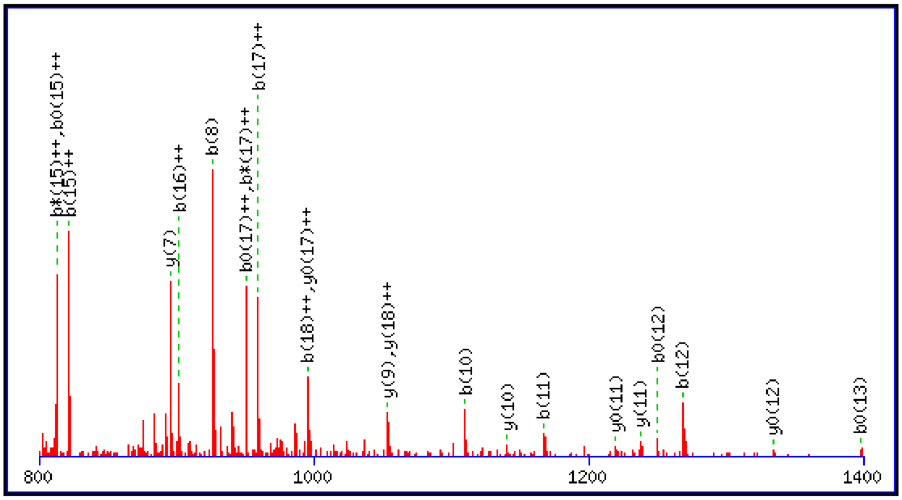

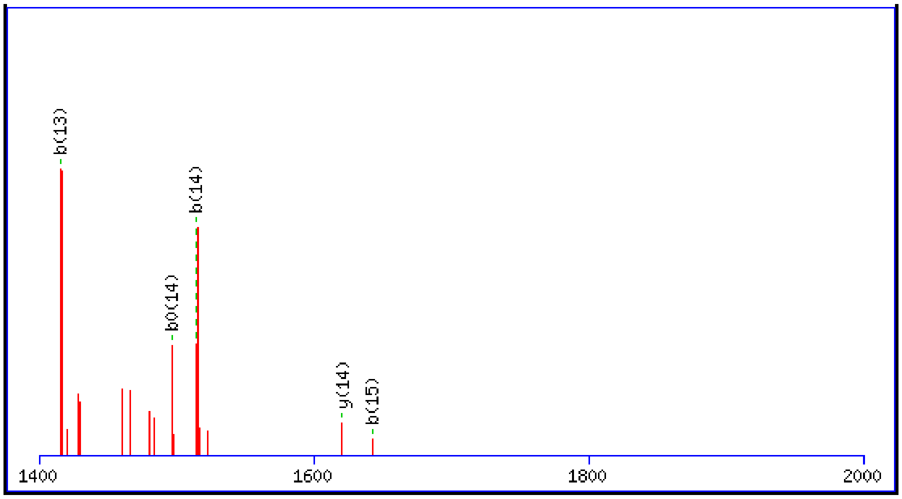

Monoisotopic mass of neutral peptide Mr(calc): 2161.9674
Fixed modifications: Carbamidomethyl (C)
Ions Score: 61 **Expect:** 0.00023
Matches (Bold Red): 49/194 fragment ions using 61 most intense peaks

#	b	b^{++}	b*	b^{*++}	b^0	b^{0++}	Seq.	y	y^{++}	y*	y^{*++}	y^0	y^{0++}	#
1	58.0287	29.5180					G							19
2	155.0815	78.0444					P	2105.9532	**1053.4802**	2088.9266	1044.9669	2087.9426	1044.4749	18
3	283.1401	142.0737	266.1135	133.5604			Q	2008.9004	1004.9538	1991.8738	996.4406	1990.8898	**995.9486**	17
4	396.2241	198.6157	379.1976	190.1024			L	1880.8418	940.9245	1863.8153	932.4113	1862.8313	931.9193	16
5	543.2926	272.1499	526.2660	263.6366			F	1767.7578	884.3825	1750.7312	875.8692	1749.7472	875.3772	15
6	680.3515	340.6794	663.3249	332.1661			H	**1620.6893**	810.8483	1603.6628	802.3350	1602.6788	801.8430	14
7	811.3920	406.1996	794.3654	397.6863			M	1483.6304	742.3189	1466.6039	733.8056	1465.6199	733.3136	13
8	**926.4189**	**463.7131**	909.3924	455.1998	908.4083	454.7078	D	1352.5899	676.7986	1335.5634	668.2853	**1334.5794**	667.7933	12
9	1023.4717	512.2395	1006.4451	503.7262	1005.4611	503.2342	P	**1237.5630**	**619.2851**	1220.5365	**610.7719**	**1219.5524**	**610.2799**	11
10	**1110.5037**	**555.7555**	1093.4771	547.2422	1092.4931	546.7502	S	**1140.5102**	570.7588	1123.4837	562.2455	1122.4997	561.7535	10
11	**1167.5252**	584.2662	1150.4986	575.7529	1149.5146	575.2609	G	**1053.4782**	527.2427	1036.4517	518.7295	1035.4676	518.2375	9
12	**1268.5728**	**634.7901**	1251.5463	626.2768	**1250.5623**	625.7848	T	996.4567	**498.7320**	979.4302	490.2187	978.4462	489.7267	8
13	**1415.6412**	**708.3243**	1398.6147	**699.8110**	**1397.6307**	**699.3190**	F	**895.4091**	**448.2082**	878.3825	439.6949	877.3985	439.2029	7
14	**1514.7097**	757.8585	1497.6831	**749.3452**	**1496.6991**	**748.8532**	V	**748.3407**	**374.6740**	731.3141	366.1607	730.3301	365.6687	6
15	**1642.7682**	**821.8878**	1625.7417	**813.3745**	1624.7577	**812.8825**	Q	**649.2722**	**325.1398**	632.2457	**316.6265**	631.2617	**316.1345**	5
16	1802.7989	**901.9031**	1785.7723	893.3898	1784.7883	892.8978	C	**521.2137**	261.1105	504.1871	252.5972	503.2031	252.1052	4
17	1917.8258	**959.4166**	1900.7993	**950.9033**	1899.8153	**950.4113**	D	**361.1830**	181.0951	344.1565	172.5819	343.1724	172.0899	3
18	1988.8629	**994.9351**	1971.8364	986.4218	1970.8524	985.9298	A	**246.1561**	123.5817	229.1295	115.0684			2
19							R	175.1190	88.0631	158.0924	79.5498			1

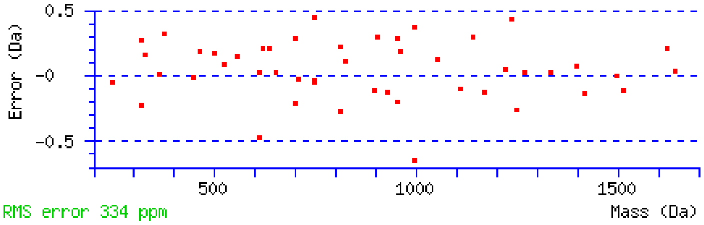

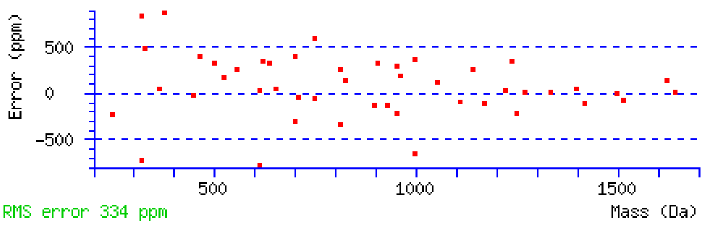

All matches to this query

Score	Mr(calc):	Delta	Sequence
61.0	2161.9674	0.0126	GPQLFHMDPSGTFVQCDAR
12.6	2160.9633	1.0167	SAKYSEAWQSKEEMENTK
10.0	2159.9840	1.9960	FPRPMSPLQDVSTIIGSR

9.7	2161.9843	-0.0044	NSGEVMTVGINAIKEITAR
9.4	2162.0015	-0.0215	IYAAMMIMDYYKQSKVK
8.8	2161.9633	0.0166	MRRLFQTCDGDGDGYISR
8.6	2159.9759	2.0040	ENYLEATVWIHGDNEENK
5.9	2161.9861	-0.0061	LVASESQTVGSMTVGSMRAR
5.8	2161.9861	-0.0061	LVASESQTVGSMTVGSMRAR
5.7	2161.9861	-0.0061	LVASESQTVGSMTVGSMRAR

Spectrum No: 95; Query: 16765; Rank: 1

Peptide View

MS/MS Fragmentation of **ITSPLMEPSSIEK**
Found in **IPI00291922**, Tax_Id=9606 Gene_Symbol=PSMA5 Proteasome subunit alpha type-5

Match to Query 16765: 1430.739968 from(716.377260,2+)
Title: 091224LimSK_Exosome3_06.3321.3321.2.dta
Data file C:\Temp\Mascoti\LSK_2D\091224LimSK_Exosome3_01_2D.mgf

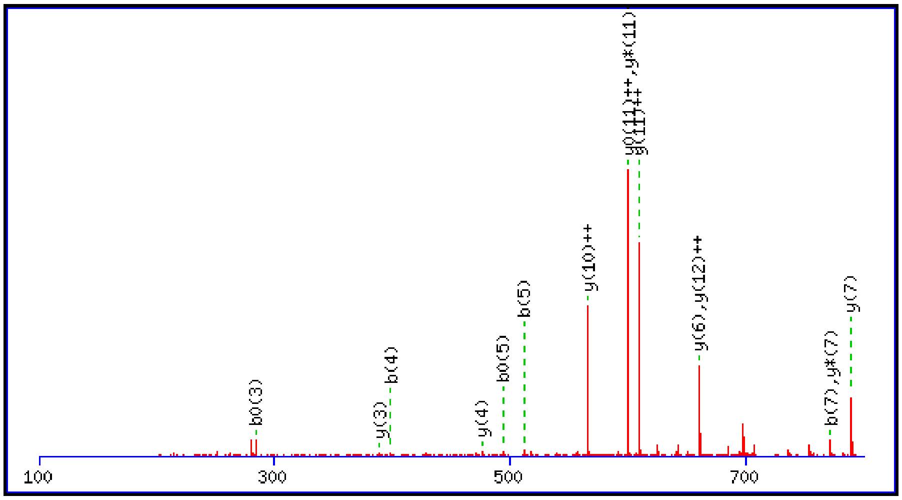

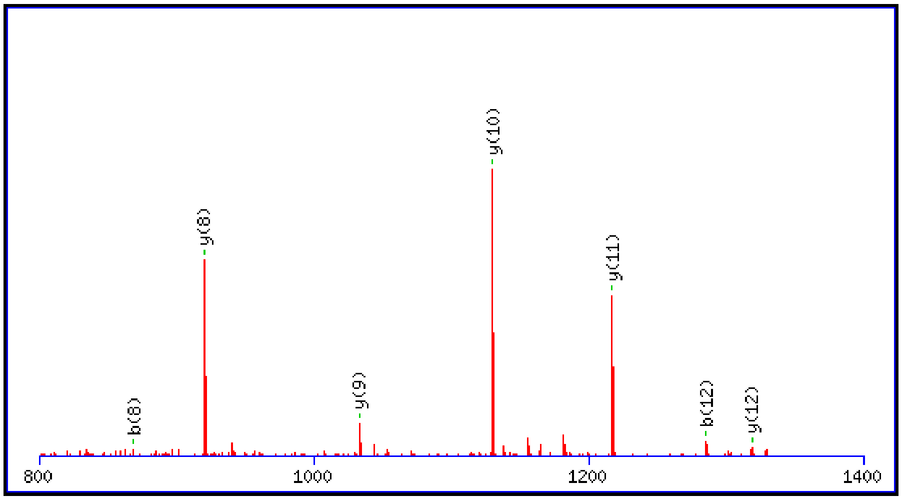

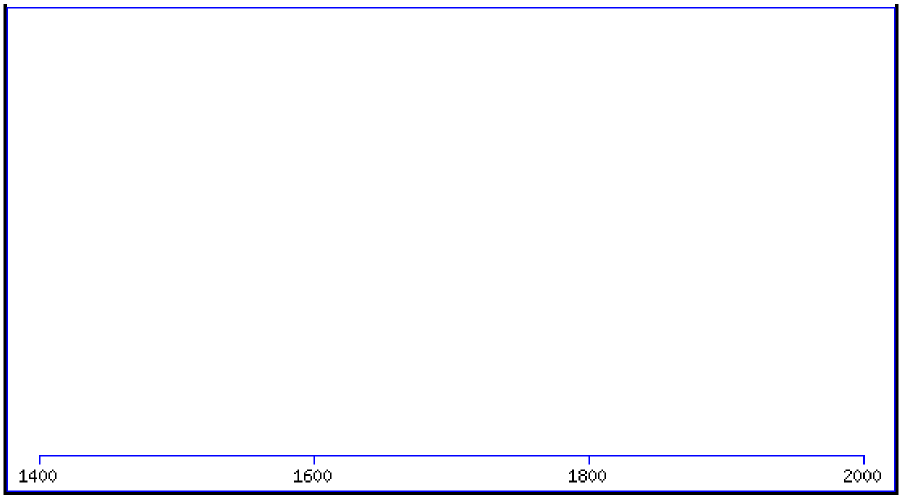

Monoisotopic mass of neutral peptide Mr(calc): 1430.7327
Fixed modifications: Carbamidomethyl (C)
Ions Score: 77 **Expect:** 3e-006
Matches (Bold Red): 22/116 fragment ions using 33 most intense peaks

#	b	b^{++}	b^{0}	b^{0++}	Seq.	y	y^{++}	y*	y^{*++}	y^{0}	y^{0++}	#
1	114.0913	57.5493			I							13
2	215.1390	108.0731	197.1285	99.0679	T	**1318.6559**	**659.8316**	1301.6293	651.3183	1300.6453	650.8263	12
3	302.1710	151.5892	**284.1605**	142.5839	S	**1217.6082**	**609.3077**	1200.5817	**600.7945**	1199.5976	**600.3025**	11
4	**399.2238**	200.1155	381.2132	191.1103	P	**1130.5762**	**565.7917**	1113.5496	557.2785	1112.5656	556.7864	10
5	**512.3079**	256.6576	**494.2973**	247.6523	L	**1033.5234**	517.2653	1016.4969	508.7521	1015.5129	508.2601	9
6	643.3484	322.1778	625.3378	313.1725	M	**920.4394**	460.7233	903.4128	452.2100	902.4288	451.7180	8
7	**772.3910**	386.6991	754.3804	377.6938	E	**789.3989**	395.2031	**772.3723**	386.6898	771.3883	386.1978	7
8	**869.4437**	435.2255	851.4332	426.2202	P	**660.3563**	330.6818	643.3297	322.1685	642.3457	321.6765	6
9	956.4757	478.7415	938.4652	469.7362	S	563.3035	282.1554	546.2770	273.6421	545.2930	273.1501	5
10	1043.5078	522.2575	1025.4972	513.2522	S	**476.2715**	238.6394	459.2449	230.1261	458.2609	229.6341	4
11	1156.5918	578.7996	1138.5813	569.7943	I	**389.2395**	195.1234	372.2129	186.6101	371.2289	186.1181	3
12	**1285.6344**	643.3209	1267.6239	634.3156	E	276.1554	138.5813	259.1288	130.0681	258.1448	129.5761	2
13					K	147.1128	74.0600	130.0863	65.5468			1

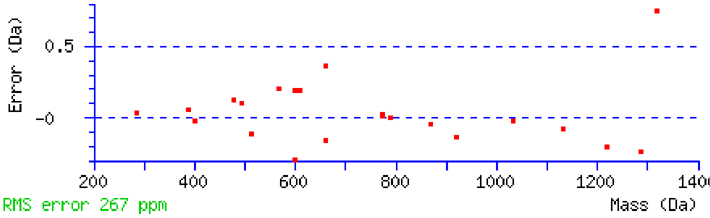

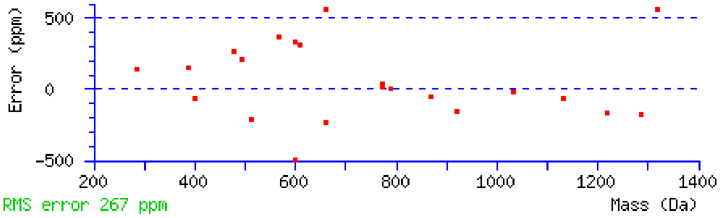

All matches to this query

Score	Mr(calc):	Delta	Sequence
77.0	1430.7327	0.0073	ITSPLMEPSSIEK
17.1	1429.7460	0.9940	VNSPIRMKNGNGK
6.0	1429.7330	1.0070	ALVLNTPNNPLGK
4.2	1430.7283	0.0117	ELQIVRTLDHK
2.8	1429.7330	1.0070	NIANSLPNVALPK
2.5	1430.7518	-0.0118	NADVNVLPIPDHK
1.7	1430.7307	0.0093	WPDVQYQILGGR
1.6	1429.7500	0.9900	NVSQLPATWRMK
1.3	1430.7518	-0.0118	ITTTEDFKHLAR

0.9	1428.7249	2.0151	DPYATSVGHLIEK

Spectrum No: 96; Query: 16335; Rank: 1

Peptide View

MS/MS Fragmentation of **LFQVEYAIEAIK**
Found in **IPI00291922**, Tax_Id=9606 Gene_Symbol=PSMA5 Proteasome subunit alpha type-5

Match to Query 16335: 1422.784928 from(712.399740,2+)
Title: 091224LimSK_Exosome3_06.6995.6995.2.dta
Data file C:\Temp\Mascoti\LSK_2D\091224LimSK_Exosome3_01_2D.mgf

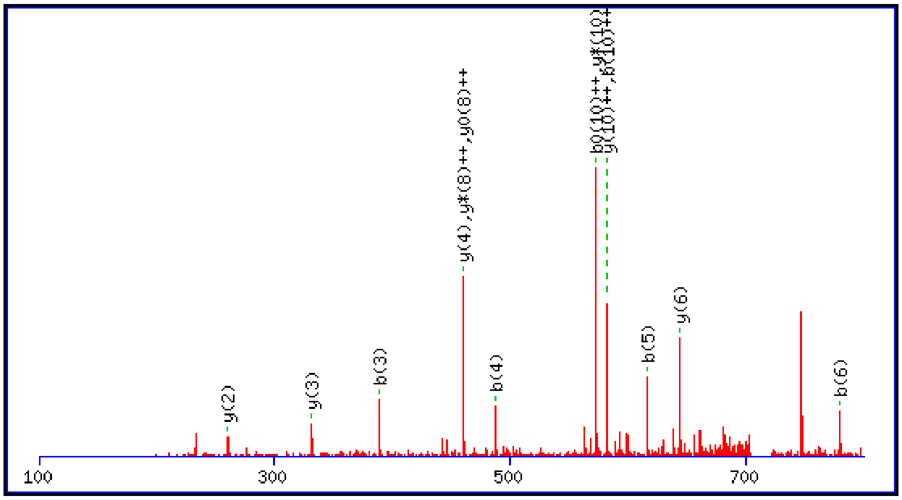

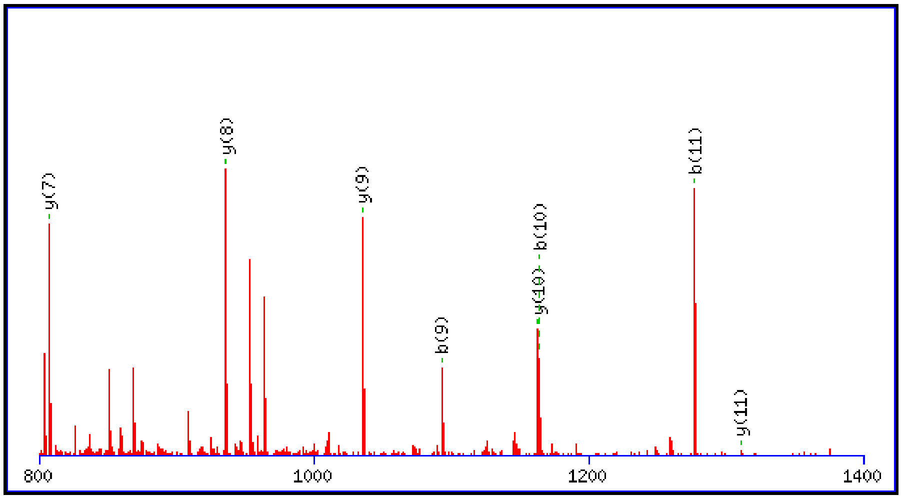

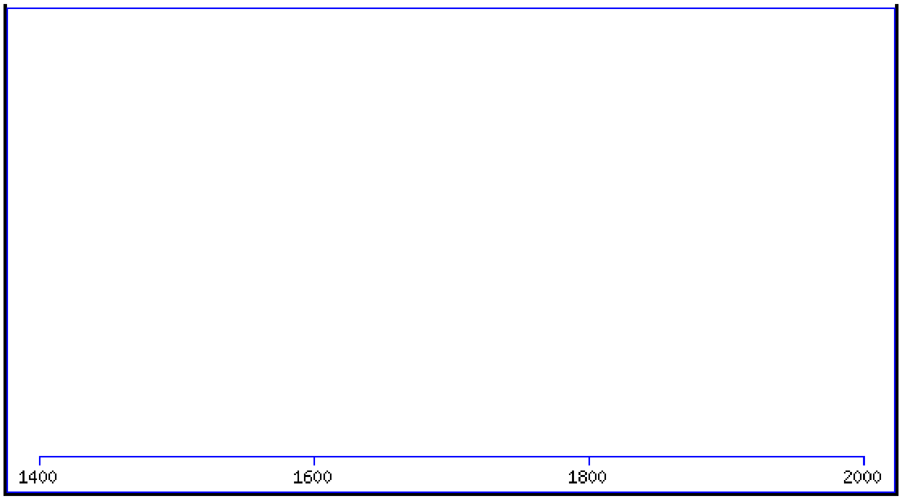

Monoisotopic mass of neutral peptide Mr(calc): 1422.7758
Fixed modifications: Carbamidomethyl (C)
Ions Score: 85 **Expect:** 2.4e-007
Matches (Bold Red): 25/114 fragment ions using 24 most intense peaks

#	b	b^{++}	b*	b^{*++}	b^0	b^{0++}	Seq.	y	y^{++}	y*	y^{*++}	y^0	y^{0++}	#
1	114.0913	57.5493					L							12
2	261.1598	131.0835					F	**1310.6991**	655.8532	1293.6725	647.3399	1292.6885	646.8479	11
3	**389.2183**	195.1128	372.1918	186.5995			Q	**1163.6307**	**582.3190**	1146.6041	**573.8057**	1145.6201	**573.3137**	10
4	**488.2867**	244.6470	471.2602	236.1337			V	**1035.5721**	518.2897	1018.5455	509.7764	1017.5615	509.2844	9
5	**617.3293**	309.1683	600.3028	300.6550	599.3188	300.1630	E	**936.5037**	468.7555	919.4771	**460.2422**	918.4931	**459.7502**	8
6	**780.3927**	390.7000	763.3661	382.1867	762.3821	381.6947	Y	**807.4611**	404.2342	790.4345	395.7209	789.4505	395.2289	7
7	851.4298	426.2185	834.4032	417.7053	833.4192	417.2132	A	**644.3978**	322.7025	627.3712	314.1892	626.3872	313.6972	6
8	964.5138	482.7606	947.4873	474.2473	946.5033	473.7553	I	**573.3606**	287.1840	556.3341	278.6707	555.3501	278.1787	5
9	**1093.5564**	547.2819	1076.5299	538.7686	1075.5459	538.2766	E	**460.2766**	230.6419	443.2500	222.1287	442.2660	221.6366	4
10	**1164.5936**	**582.8004**	1147.5670	**574.2871**	1146.5830	**573.7951**	A	**331.2340**	166.1206	314.2074	157.6074			3
11	**1277.6776**	639.3424	1260.6511	630.8292	1259.6671	630.3372	I	**260.1969**	130.6021	243.1703	122.0888			2
12							K	147.1128	74.0600	130.0863	65.5468			1

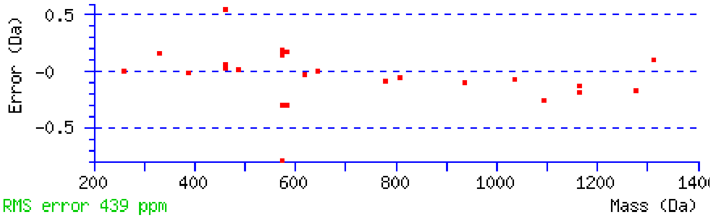

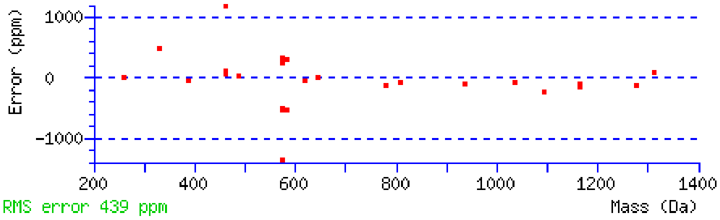

All matches to this query

Score	Mr(calc):	Delta	Sequence
85.4	1422.7758	0.0091	LFQVEYAIEAIK
8.6	1422.7904	-0.0055	IMKGLNAYLEKK
7.5	1422.7732	0.0118	QFGHPQIEARIK
7.3	1420.7899	1.9951	EQARLKAHVVDR
6.9	1422.7752	0.0098	MLKGISSSSLKEK
5.3	1422.7878	-0.0028	VGHRMNIERALK
5.0	1422.7710	0.0139	VVWMQTLLILK
4.9	1422.7718	0.0131	AALDSPPGALAELAK
4.7	1422.7718	0.0131	AHLEEISALVVDK
4.5	1422.7983	-0.0134	FLSRTFALAELR

Spectrum No: 97; Query: 55322; Rank: 1

Peptide View

MS/MS Fragmentation of **LNATNIELATVQPGQNFHMFTK**
Found in **IPI00291922**, Tax_Id=9606 Gene_Symbol=PSMA5 Proteasome subunit alpha type-5

Match to Query 55322: 2473.259292 from(825.427040,3+)
Title: 091224LimSK_Exosome3_06.6229.6229.3.dta
Data file C:\Temp\Mascoti\LSK_2D\091224LimSK_Exosome3_01_2D.mgf

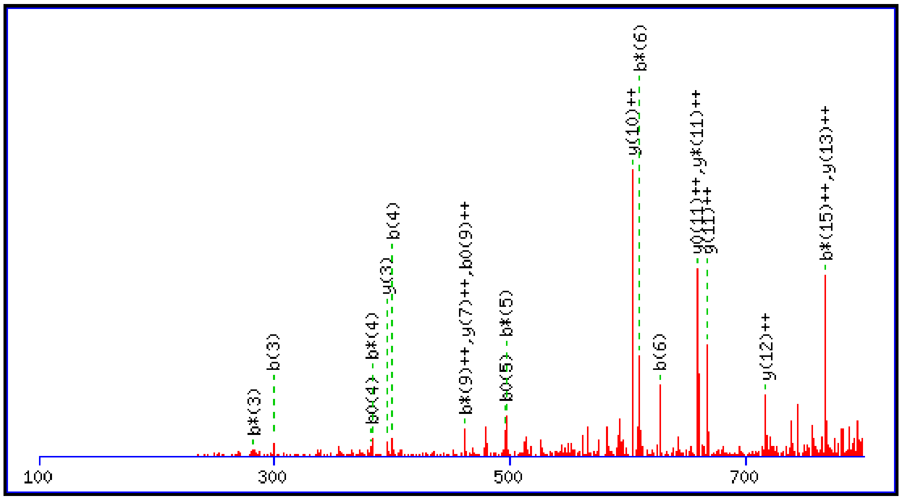

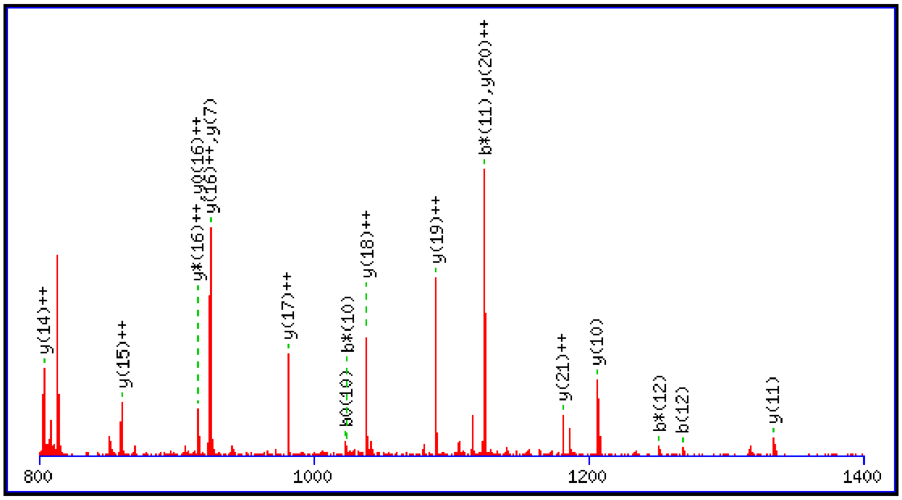

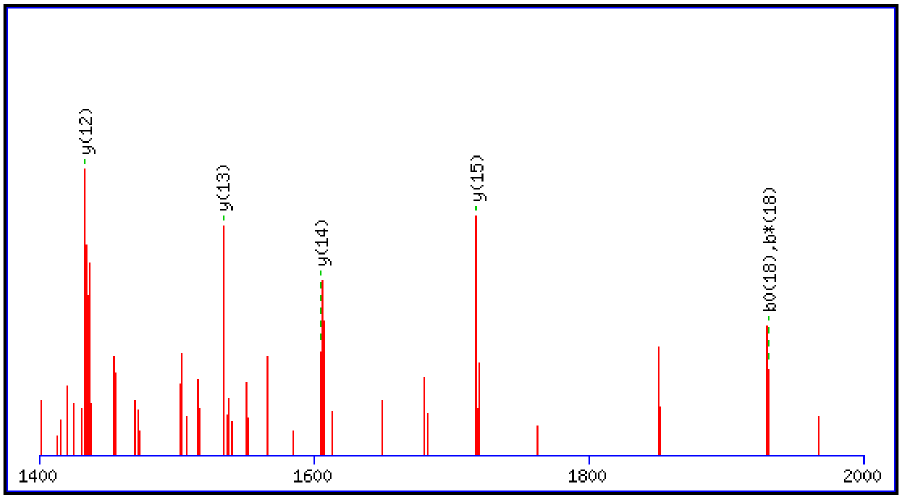

Monoisotopic mass of neutral peptide Mr(calc): 2473.2424
Fixed modifications: Carbamidomethyl (C)
Ions Score: 74 **Expect:** 7.1e-006
Matches (Bold Red): 44/242 fragment ions using 69 most intense peaks

#	b	b^{++}	b*	b^{*++}	b^0	b^{0++}	Seq.	y	y^{++}	y*	y^{*++}	y^0	y^{0++}	#
1	114.0913	57.5493					L							22
2	228.1343	114.5708	211.1077	106.0575			N	2361.1656	**1181.0864**	2344.1390	1172.5732	2343.1550	1172.0812	21
3	**299.1714**	150.0893	**282.1448**	141.5761			A	2247.1227	**1124.0650**	2230.0961	1115.5517	2229.1121	1115.0597	20
4	**400.2191**	200.6132	**383.1925**	192.0999	**382.2085**	191.6079	T	2176.0856	**1088.5464**	2159.0590	1080.0331	2158.0750	1079.5411	19
5	514.2620	257.6346	**497.2354**	249.1214	**496.2514**	248.6293	N	2075.0379	**1038.0226**	2058.0113	1029.5093	2057.0273	1029.0173	18
6	**627.3461**	314.1767	**610.3195**	305.6634	609.3355	305.1714	I	1960.9949	**981.0011**	1943.9684	972.4878	1942.9844	971.9958	17
7	756.3886	378.6980	739.3621	370.1847	738.3781	369.6927	E	1847.9109	**924.4591**	1830.8843	**915.9458**	1829.9003	**915.4538**	16
8	869.4727	435.2400	852.4462	426.7267	851.4621	426.2347	L	**1718.8683**	**859.9378**	1701.8417	851.4245	1700.8577	850.9325	15
9	940.5098	470.7585	923.4833	**462.2453**	922.4993	**461.7533**	A	**1605.7842**	**803.3958**	1588.7577	794.8825	1587.7737	794.3905	14
10	1041.5575	521.2824	**1024.5310**	512.7691	**1023.5469**	512.2771	T	**1534.7471**	**767.8772**	1517.7206	759.3639	1516.7365	758.8719	13
11	1140.6259	570.8166	**1123.5994**	562.3033	1122.6154	561.8113	V	**1433.6994**	**717.3534**	1416.6729	708.8401	1415.6889	708.3481	12
12	**1268.6845**	634.8459	**1251.6579**	626.3326	1250.6739	625.8406	Q	**1334.6310**	**667.8191**	1317.6045	**659.3059**	1316.6205	**658.8139**	11
13	1365.7373	683.3723	1348.7107	674.8590	1347.7267	674.3670	P	**1206.5724**	**603.7899**	1189.5459	595.2766	1188.5619	594.7846	10
14	1422.7587	711.8830	1405.7322	703.3697	1404.7482	702.8777	G	1109.5197	555.2635	1092.4931	546.7502	1091.5091	546.2582	9
15	1550.8173	775.9123	1533.7908	**767.3990**	1532.8067	766.9070	Q	1052.4982	526.7527	1035.4717	518.2395	1034.4876	517.7475	8
16	1664.8602	832.9338	1647.8337	824.4205	1646.8497	823.9285	N	**924.4396**	**462.7235**	907.4131	454.2102	906.4291	453.7182	7
17	1811.9286	906.4680	1794.9021	897.9547	1793.9181	897.4627	F	810.3967	405.7020	793.3702	397.1887	792.3861	396.6967	6
18	1948.9876	974.9974	**1931.9610**	966.4841	**1930.9770**	965.9921	H	663.3283	332.1678	646.3017	323.6545	645.3177	323.1625	5
19	2080.0280	1040.5177	2063.0015	1032.0044	2062.0175	1031.5124	M	526.2694	263.6383	509.2428	255.1251	508.2588	254.6330	4
20	2227.0965	1114.0519	2210.0699	1105.5386	2209.0859	1105.0466	F	**395.2289**	198.1181	378.2023	189.6048	377.2183	189.1128	3
21	2328.1441	1164.5757	2311.1176	1156.0624	2310.1336	1155.5704	T	248.1605	124.5839	231.1339	116.0706	230.1499	115.5786	2
22							K	147.1128	74.0600	130.0863	65.5468			1

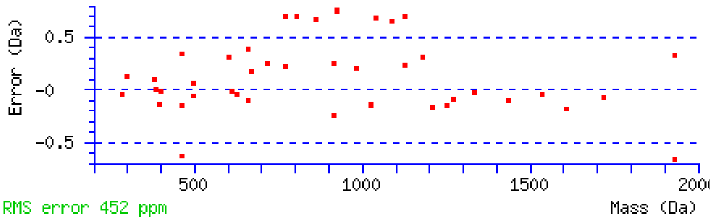

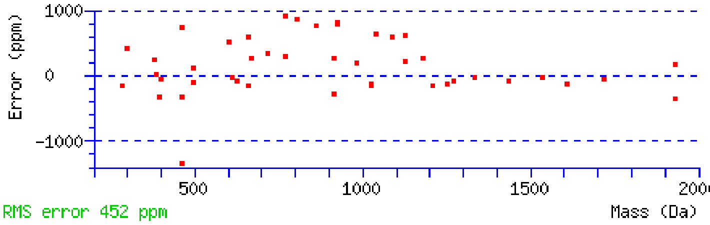

All matches to this query

Score	Mr(calc):	Delta	Sequence
74.1	2473.2424	0.0169	LNATNIELATVQPGQNFHMFTK
12.0	2471.2322	2.0271	RGGLEVALQVQVLDTPGYPHSR
9.4	2472.2373	1.0220	LKTGPEGALGSEKNGVPARPGTEK
1.1	2471.2515	2.0078	HVGPSFVRKEFQNNFVFIVK
0.4	2473.2512	0.0081	KVLGRGGEAASGGLAPPAVAMVTER
0.3	2471.2557	2.0036	QPGASPSQERKPTGVSVIYWER
0.3	2471.2560	2.0033	GELIPISPSTEVGGSGIGTPPSVLK
0.3	2473.2465	0.0128	GSTGGVAPESLTLVLSAASPLVDPR
0.2	2473.2730	-0.0137	IGPIATPDYIQNAPGLPKTRSGK
0.1	2471.2445	2.0148	GDIGIGIAGENGLPGPPGPQGPPGYGK

Spectrum No: 98; Query: 24989; Rank: 1

Peptide View

MS/MS Fragmentation of **RITSPLMEPSSIEK**
Found in **IPI00291922**, Tax_Id=9606 Gene_Symbol=PSMA5 Proteasome subunit alpha type-5

Match to Query 24989: 1586.843688 from(794.429120,2+)
Title: 091224LimSK_Exosome3_05.2426.2426.2.dta
Data file C:\Temp\Mascoti\LSK_2D\091224LimSK_Exosome3_01_2D.mgf

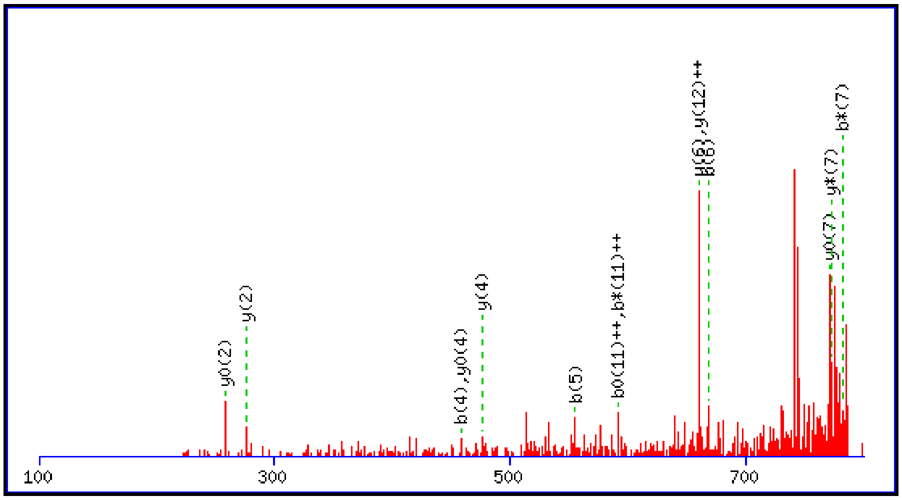

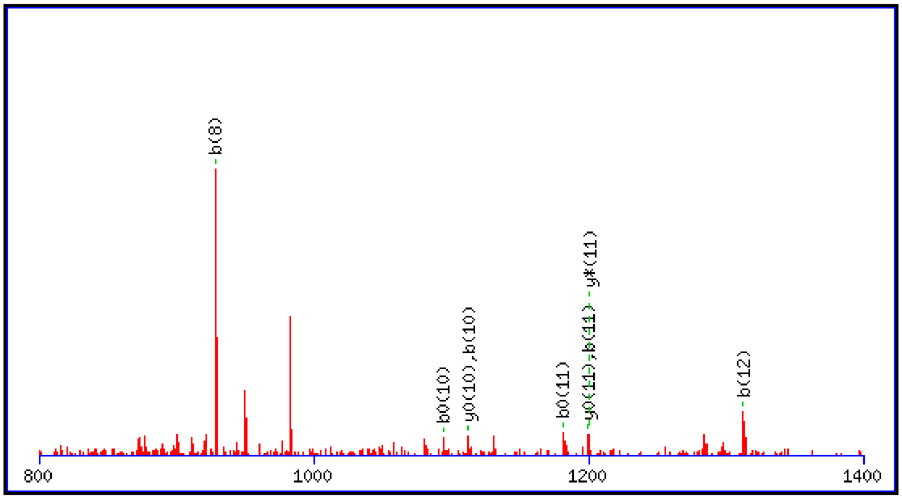

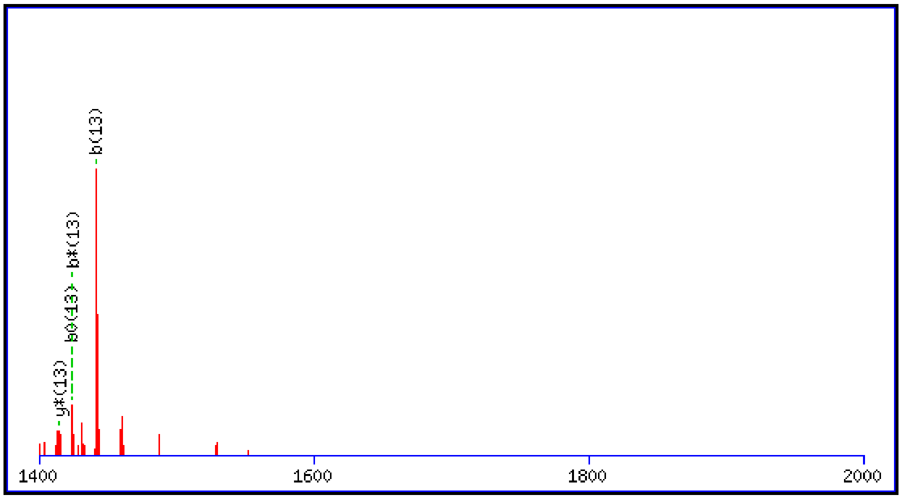

Monoisotopic mass of neutral peptide Mr(calc): 1586.8338
Fixed modifications: Carbamidomethyl (C)
Ions Score: 36 **Expect:** 0.033
Matches (Bold Red): 27/150 fragment ions using 61 most intense peaks

#	b	b++	b*	b*++	b0	b0++	Seq.	y	y++	y*	y*++	y0	y0++	#
1	157.1084	79.0578	140.0818	70.5446			R							14
2	270.1925	135.5999	253.1659	127.0866			I	1431.7400	716.3736	**1414.7134**	707.8603	1413.7294	707.3683	13
3	371.2401	186.1237	354.2136	177.6104	353.2296	177.1184	T	1318.6559	**659.8316**	1301.6293	651.3183	1300.6453	650.8263	12
4	**458.2722**	229.6397	441.2456	221.1264	440.2616	220.6344	S	1217.6082	609.3077	**1200.5817**	600.7945	**1199.5977**	600.3025	11
5	**555.3249**	278.1661	538.2984	269.6528	537.3144	269.1608	P	1130.5762	565.7917	1113.5496	557.2785	**1112.5656**	556.7864	10
6	**668.4090**	334.7081	651.3824	326.1949	650.3984	325.7028	L	1033.5234	517.2653	1016.4969	508.7521	1015.5129	508.2601	9
7	799.4495	400.2284	**782.4229**	391.7151	781.4389	391.2231	M	920.4394	460.7233	903.4128	452.2100	902.4288	451.7180	8
8	**928.4921**	464.7497	911.4655	456.2364	910.4815	455.7444	E	789.3989	395.2031	**772.3723**	386.6898	**771.3883**	386.1978	7
9	1025.5448	513.2761	1008.5183	504.7628	1007.5343	504.2708	P	**660.3563**	330.6818	643.3297	322.1685	642.3457	321.6765	6
10	**1112.5769**	556.7921	1095.5503	548.2788	**1094.5663**	547.7868	S	563.3035	282.1554	546.2770	273.6421	545.2930	273.1501	5
11	**1199.6089**	600.3081	1182.5823	**591.7948**	**1181.5983**	**591.3028**	S	**476.2715**	238.6394	459.2449	230.1261	**458.2609**	229.6341	4
12	**1312.6929**	656.8501	1295.6664	648.3368	1294.6824	647.8448	I	389.2395	195.1234	372.2129	186.6101	371.2289	186.1181	3
13	**1441.7355**	721.3714	**1424.7090**	712.8581	**1423.7250**	712.3661	E	**276.1554**	138.5813	259.1288	130.0681	**258.1448**	129.5761	2
14							K	147.1128	74.0600	130.0863	65.5468			1

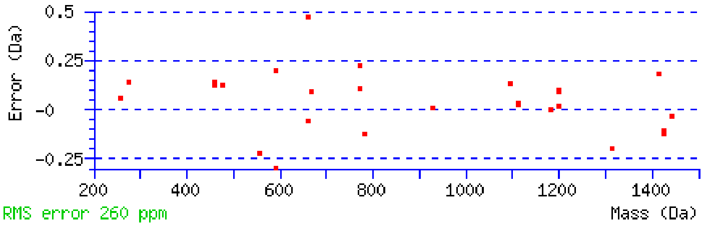

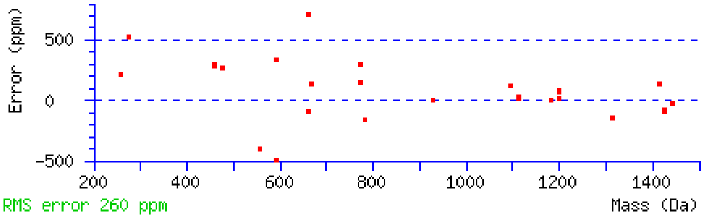

All matches to this query

Score	Mr(calc):	Delta	Sequence
36.0	1586.8338	0.0099	RITSPLMEPSSIEK
9.9	1585.8415	1.0022	QFKKMLTSAILAR
8.1	1586.8338	0.0099	GMPETLGQVAKALEK
5.9	1586.8545	-0.0108	DHSIQIKIGKLQK
5.7	1586.8507	-0.0070	KIAVISTKLFMEK
5.2	1586.8562	-0.0126	GSLAMSIQRGSLVPR
5.1	1586.8507	-0.0070	KIAVISTKLFMEK
5.1	1585.8464	0.9973	DGAYLDNPPKGALKK
4.6	1585.8246	1.0191	EHLTASMSRVALQK
4.4	1586.8515	-0.0079	KEVAETSAGPSVVSVK

Spectrum No: 99; Query: 181; Rank: 1

Peptide View

MS/MS Fragmentation of **SSLIILK**
Found in **IPI00291922**, Tax_Id=9606 Gene_Symbol=PSMA5 Proteasome subunit alpha type-5

Match to Query 181: 772.509268 from(387.261910,2+)
Title: 091224LimSK_Exosome3_06.2488.2488.2.dta
Data file C:\Temp\Mascoti\LSK_2D\091224LimSK_Exosome3_01_2D.mgf

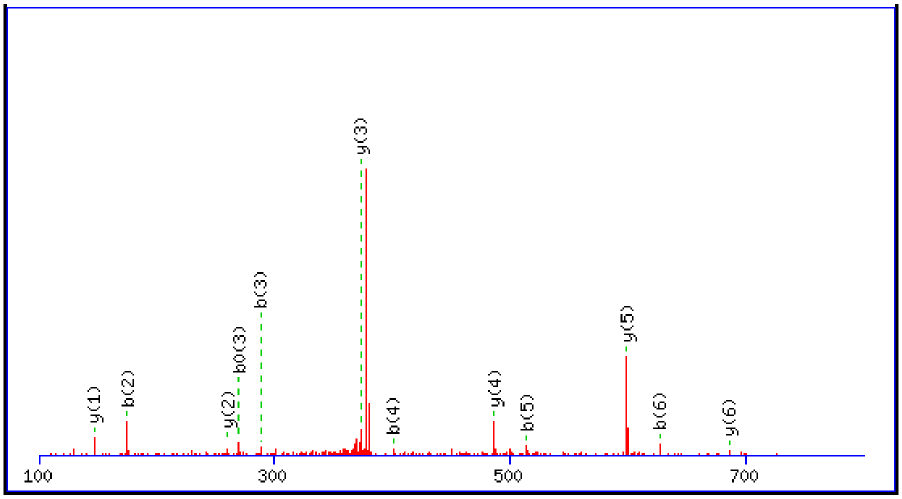

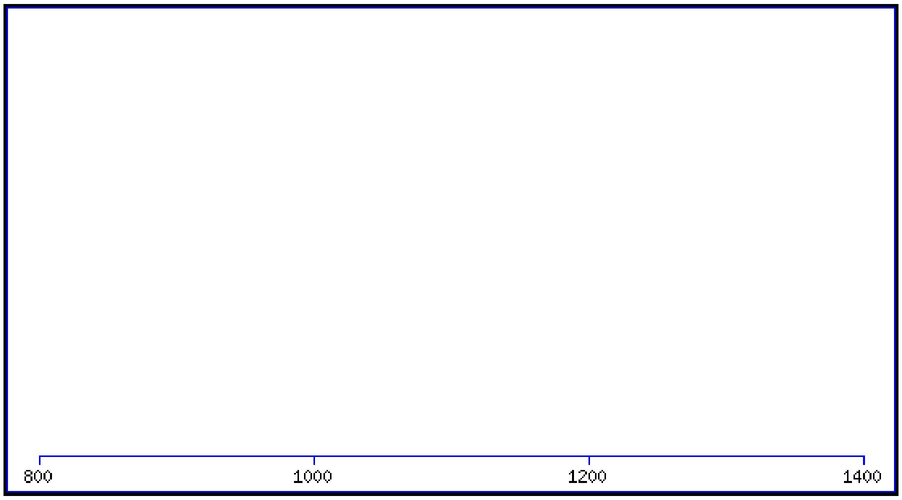

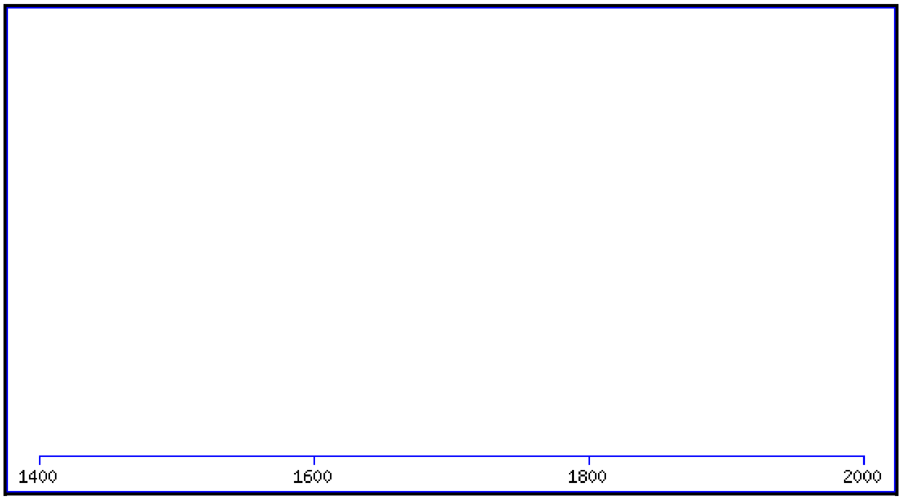

Monoisotopic mass of neutral peptide Mr(calc): 772.5058
Fixed modifications: Carbamidomethyl (C)
Ions Score: 50 **Expect:** 0.00026
Matches (Bold Red): 12/50 fragment ions using 24 most intense peaks

#	b	b^{++}	b^{0}	b^{0++}	Seq.	y	y^{++}	y*	y^{*++}	y^{0}	y^{0++}	#
1	88.0393	44.5233	70.0287	35.5180	S							7
2	**175.0713**	88.0393	157.0608	79.0340	S	**686.4811**	343.7442	669.4545	335.2309	668.4705	334.7389	6
3	**288.1554**	144.5813	**270.1448**	135.5761	L	**599.4491**	300.2282	582.4225	291.7149			5
4	**401.2395**	201.1234	383.2289	192.1181	I	**486.3650**	243.6861	469.3384	235.1729			4
5	**514.3235**	257.6654	496.3130	248.6601	I	**373.2809**	187.1441	356.2544	178.6308			3
6	**627.4076**	314.2074	609.3970	305.2021	L	**260.1969**	130.6021	243.1703	122.0888			2

7					K	147.1128	74.0600	130.0863	65.5468			1

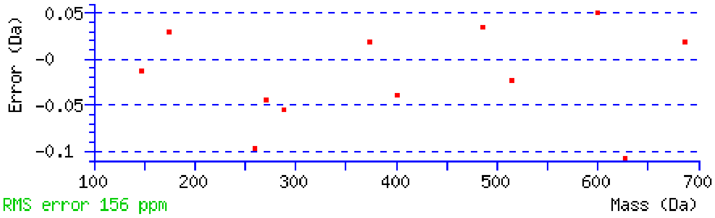

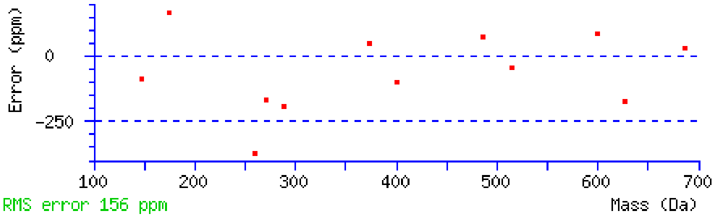

All matches to this query

Score	Mr(calc):	Delta	Sequence
50.0	772.5058	0.0035	SSLIILK
32.8	772.5058	0.0035	SLSLLLK
32.8	772.5058	0.0034	SVTLLIK
14.4	770.5014	2.0079	ERLLLK
11.3	770.5014	2.0079	EKIILR
11.3	770.5014	2.0079	KELLLR
10.3	770.5014	2.0079	NGLVKIK
9.2	772.5058	0.0035	ISISLLK
8.9	771.5079	1.0014	RSRLLK
8.3	770.5014	2.0079	ASVLLIR

Spectrum No: 100; Query: 8522; Rank: 1

Peptide View

MS/MS Fragmentation of **AVENSSTAIGIR**
Found in **IPI00171199**, Tax_Id=9606 Gene_Symbol=PSMA3 Isoform 2 of Proteasome subunit alpha type-3

Match to Query 8522: 1216.646368 from(609.330460,2+)
Title: 091224LimSK_Exosome3_06.994.994.2.dta
Data file C:\Temp\Mascoti\LSK_2D\091224LimSK_Exosome3_01_2D.mgf

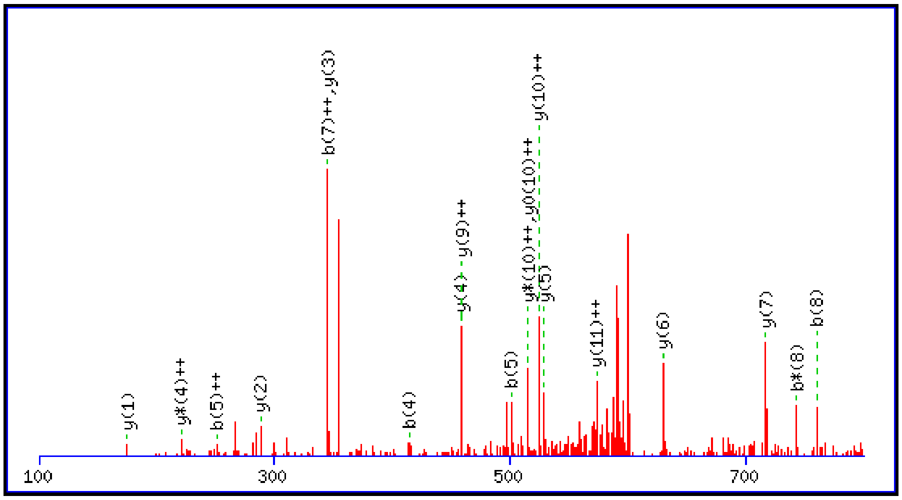

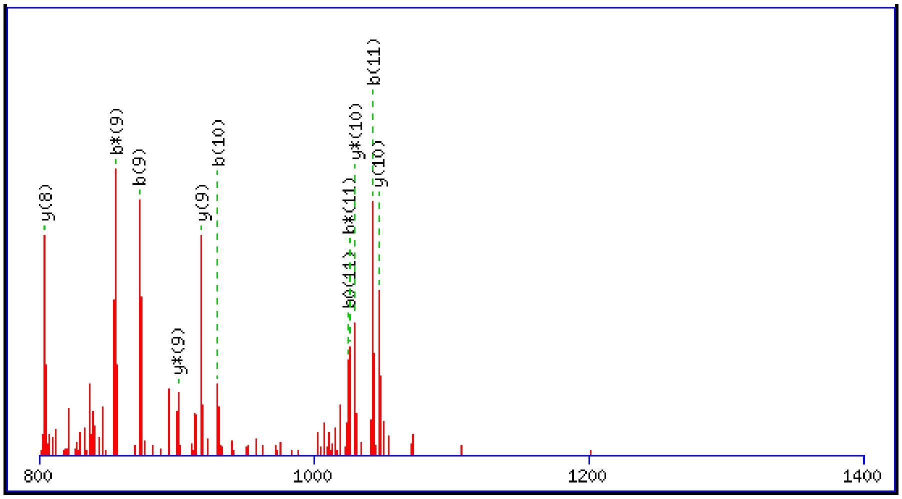

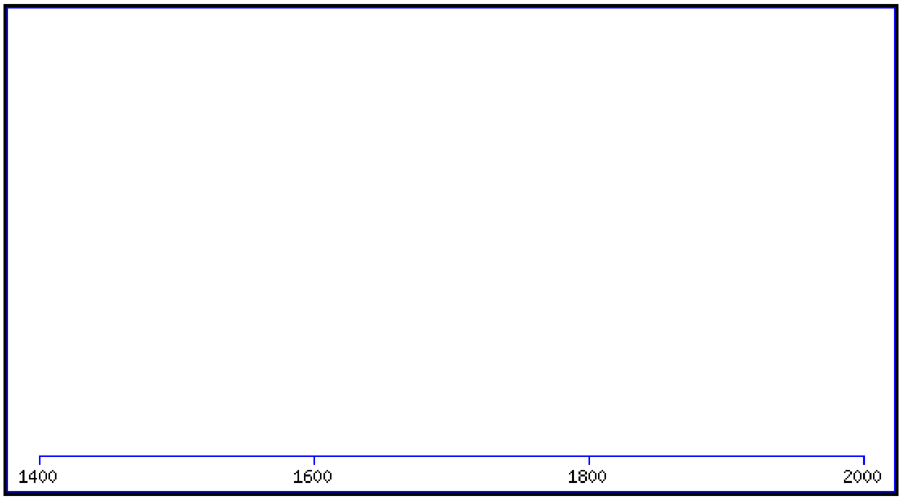

Monoisotopic mass of neutral peptide Mr(calc): 1216.6411
Fixed modifications: Carbamidomethyl (C)
Ions Score: 90 **Expect:** 1.4e-007
Matches (Bold Red): 30/112 fragment ions using 48 most intense peaks

#	b	b^{++}	b*	b^{*++}	b^0	b^{0++}	Seq.	y	y^{++}	y*	y^{*++}	y^0	y^{0++}	#
1	72.0444	36.5258					A							12
2	171.1128	86.0600					V	1146.6113	**573.8093**	1129.5848	565.2960	1128.6008	564.8040	11
3	300.1554	150.5813			282.1448	141.5761	E	**1047.5429**	**524.2751**	**1030.5164**	**515.7618**	1029.5323	**515.2698**	10
4	**414.1983**	207.6028	397.1718	199.0895	396.1878	198.5975	N	**918.5003**	**459.7538**	**901.4738**	451.2405	900.4898	450.7485	9
5	**501.2304**	**251.1188**	484.2038	242.6055	483.2198	242.1135	S	**804.4574**	402.7323	787.4308	394.2191	786.4468	393.7271	8
6	588.2624	294.6348	571.2358	286.1216	570.2518	285.6295	S	**717.4254**	359.2163	700.3988	350.7030	699.4148	350.2110	7
7	689.3101	**345.1587**	672.2835	336.6454	671.2995	336.1534	T	**630.3933**	315.7003	613.3668	307.1870	612.3828	306.6950	6
8	**760.3472**	380.6772	**743.3206**	372.1640	742.3366	371.6719	A	**529.3457**	265.1765	512.3191	256.6632			5
9	**873.4312**	437.2193	**856.4047**	428.7060	855.4207	428.2140	I	**458.3085**	229.6579	441.2820	**221.1446**			4
10	**930.4527**	465.7300	913.4262	457.2167	912.4421	456.7247	G	**345.2245**	173.1159	328.1979	164.6026			3
11	**1043.5368**	522.2720	**1026.5102**	513.7587	**1025.5262**	513.2667	I	**288.2030**	144.6051	271.1765	136.0919			2
12							R	**175.1190**	88.0631	158.0924	79.5498			1

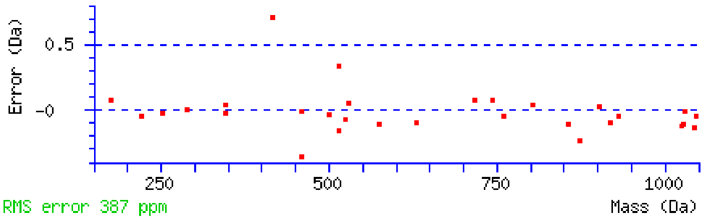

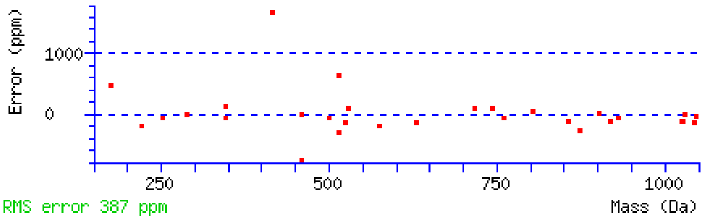

All matches to this query

Score	Mr(calc):	Delta	Sequence
89.9	1216.6411	0.0052	AVENSSTAIGIR
15.3	1215.6489	0.9975	ARLPLPGSGLR
13.8	1216.6346	0.0118	VAEATRMLNGR
12.0	1215.6360	1.0104	RDWTAELGIR
11.7	1216.6425	0.0039	WTGRDLASRR
6.5	1216.6411	0.0052	ADTLNGSLSALR
6.5	1216.6387	0.0077	TLNQWVMGIR
6.4	1215.6376	1.0087	ATPLVAGPAALR
6.2	1216.6524	-0.0060	SSLVNNRTQAK
5.5	1216.6524	-0.0060	TRSTGQLELGR

Spectrum No: 101; Query: 9178; Rank: 1

Peptide View

MS/MS Fragmentation of **CKDGVVFGVEK**
Found in **IPI00171199**, Tax_Id=9606 Gene_Symbol=PSMA3 Isoform 2 of Proteasome subunit alpha type-3

Match to Query 9178: 1236.623628 from(619.319090,2+)
Title: 091224LimSK_Exosome3_05.1172.1172.2.dta
Data file C:\Temp\Mascoti\LSK_2D\091224LimSK_Exosome3_01_2D.mgf

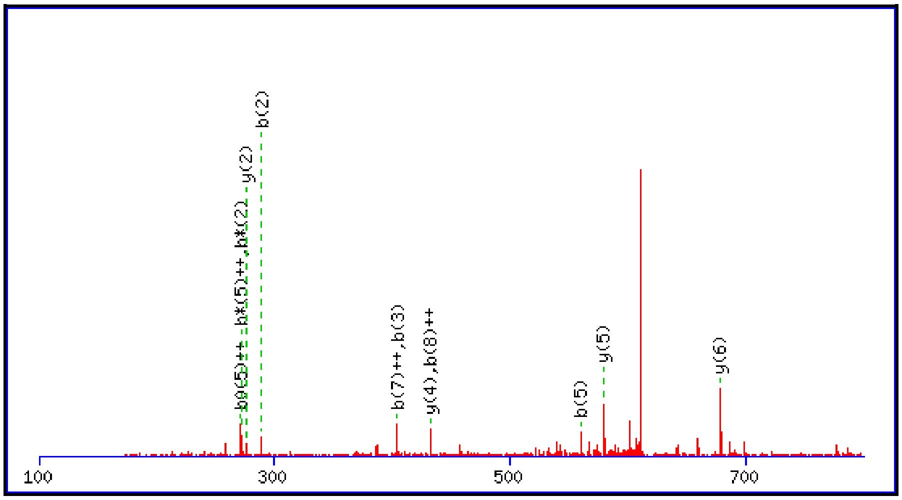

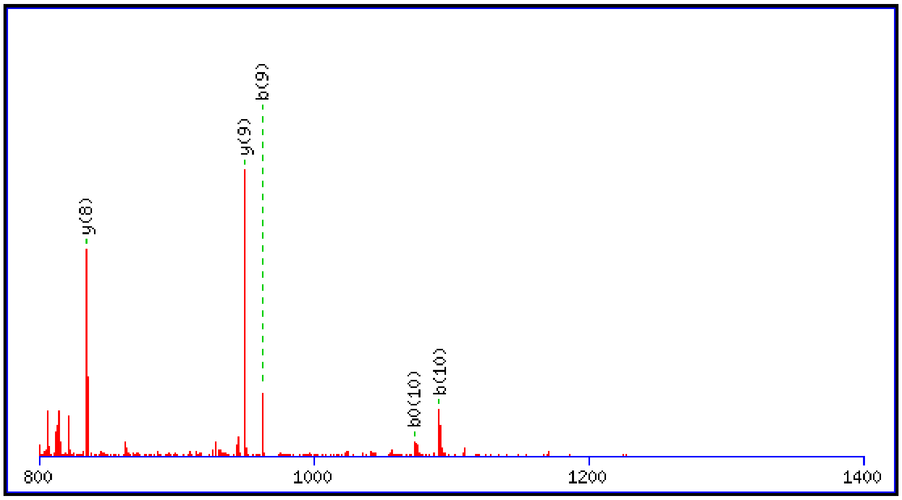

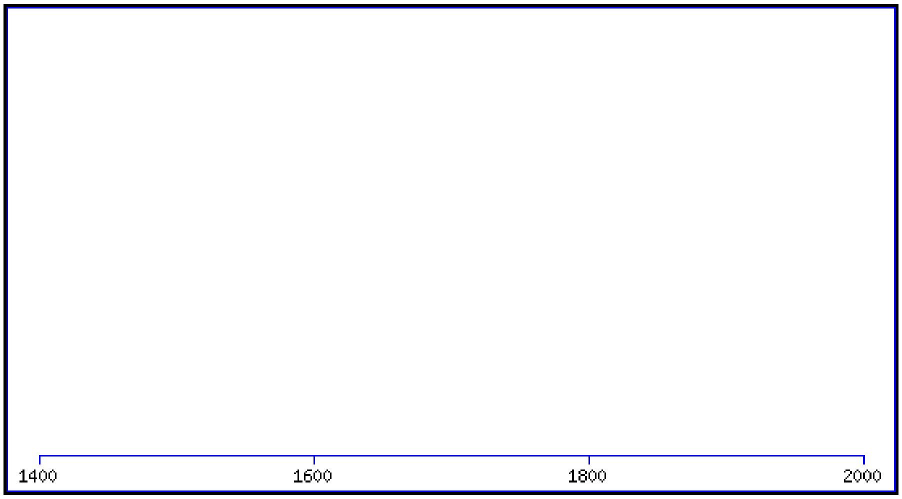

Monoisotopic mass of neutral peptide Mr(calc): 1236.6173
Fixed modifications: Carbamidomethyl (C)
Ions Score: 38 **Expect:** 0.023
Matches (Bold Red): 17/112 fragment ions using 36 most intense peaks

#	b	b^{++}	b*	b^{*++}	b^0	b^{0++}	Seq.	y	y^{++}	y*	y^{*++}	y^0	y^{0++}	#
1	161.0379	81.0226					C							11
2	**289.1329**	145.0701	**272.1063**	136.5568			K	1077.5939	539.3006	1060.5673	530.7873	1059.5833	530.2953	10
3	**404.1598**	202.5836	387.1333	194.0703	386.1493	193.5783	D	**949.4989**	475.2531	932.4724	466.7398	931.4884	466.2478	9
4	461.1813	231.0943	444.1547	222.5810	443.1707	222.0890	G	**834.4720**	417.7396	817.4454	409.2264	816.4614	408.7343	8
5	**560.2497**	280.6285	543.2232	**272.1152**	542.2391	**271.6232**	V	777.4505	389.2289	760.4240	380.7156	759.4400	380.2236	7
6	659.3181	330.1627	642.2916	321.6494	641.3076	321.1574	V	**678.3821**	339.6947	661.3556	331.1814	660.3715	330.6894	6
7	806.3865	**403.6969**	789.3600	395.1836	788.3760	394.6916	F	**579.3137**	290.1605	562.2871	281.6472	561.3031	281.1552	5
8	863.4080	**432.2076**	846.3815	423.6944	845.3974	423.2024	G	**432.2453**	216.6263	415.2187	208.1130	414.2347	207.6210	4
9	**962.4764**	481.7418	945.4499	473.2286	944.4658	472.7366	V	375.2238	188.1155	358.1973	179.6023	357.2132	179.1103	3
10	**1091.5190**	546.2631	1074.4925	537.7499	**1073.5084**	537.2579	E	**276.1554**	138.5813	259.1288	130.0681	258.1448	129.5761	2
11							K	147.1128	74.0600	130.0863	65.5468			1

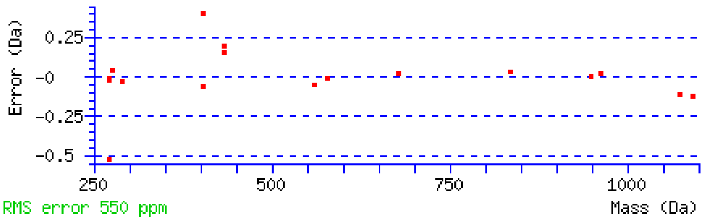

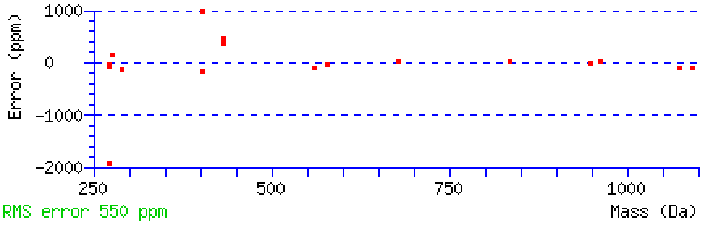

All matches to this query

Score	Mr(calc):	Delta	Sequence
38.1	1236.6173	0.0063	CKDGVVFGVEK
16.2	1236.6285	-0.0048	CKDKYGKPNK
9.0	1235.6153	1.0083	SGGNSRVTRMR
6.9	1236.6227	0.0009	DTQPRKATLK
5.7	1236.6227	0.0009	RGTLGLQGDIK
3.9	1236.6251	-0.0015	WSLYKQQER
3.6	1236.6350	-0.0114	VISQISADNYK
3.2	1236.6340	-0.0103	GRSKGILNGQK
2.9	1236.6285	-0.0049	MGLAGFTKQER
2.5	1236.6350	-0.0114	TNYVENLKEK

Spectrum No: 102; Query: 1933; Rank: 1

Peptide View

MS/MS Fragmentation of **DGVVFGVEK**
Found in **IPI00171199**, Tax_Id=9606 Gene_Symbol=PSMA3 Isoform 2 of Proteasome subunit alpha type-3

Match to Query 1933: 948.496728 from(475.255640,2+)
Title: 091224LimSK_Exosome3_07.1949.1949.2.dta
Data file C:\Temp\Mascoti\LSK_2D\091224LimSK_Exosome3_01_2D.mgf

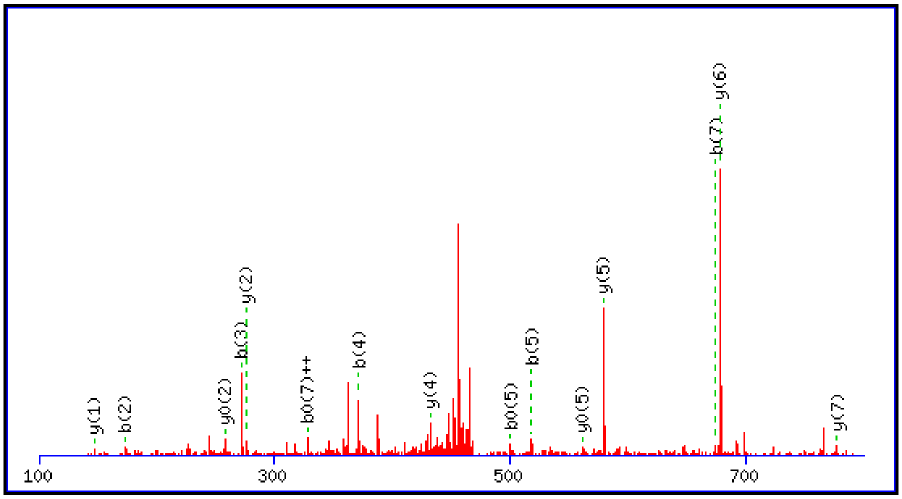

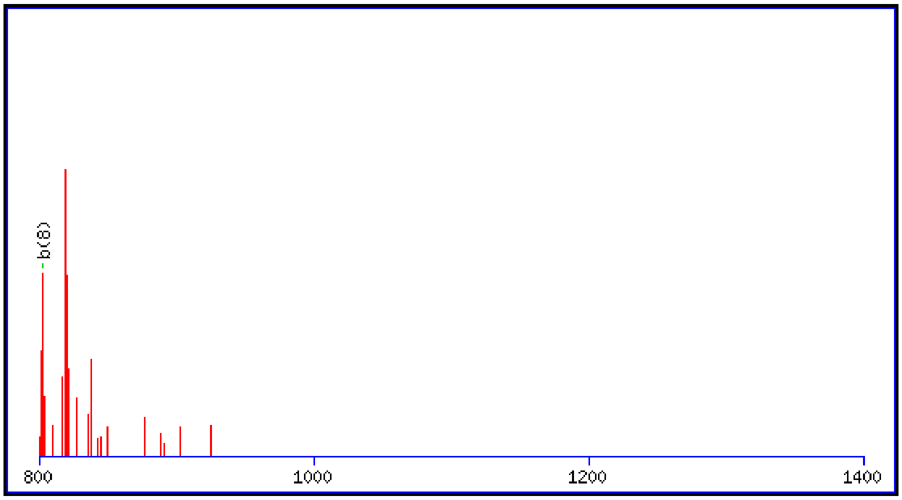

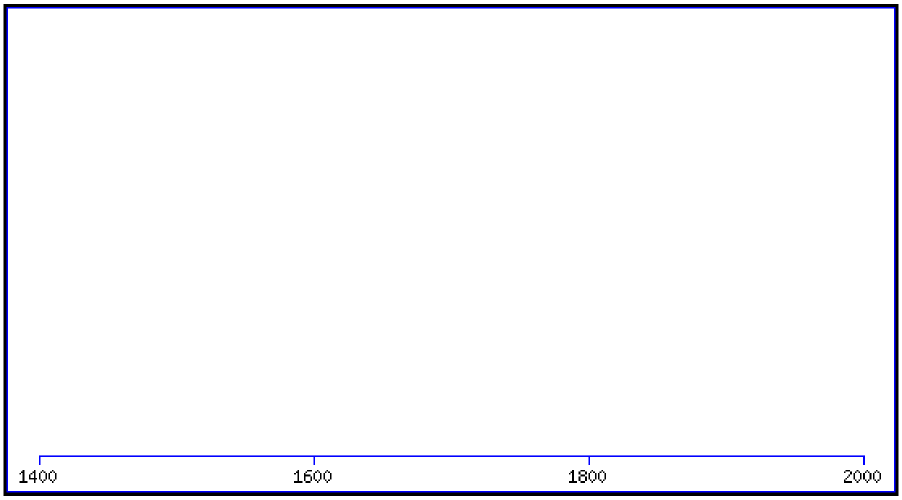

Monoisotopic mass of neutral peptide Mr(calc): 948.4917

Fixed modifications: Carbamidomethyl (C)
Ions Score: 27 **Expect:** 0.28
Matches (Bold Red): 16/78 fragment ions using 55 most intense peaks

#	b	b^{++}	b^{0}	b^{0++}	Seq.	y	y^{++}	y*	y^{*++}	y^{0}	y^{0++}	#
1	116.0342	58.5207	98.0237	49.5155	D							9
2	**173.0557**	87.0315	155.0451	78.0262	G	834.4720	417.7396	817.4454	409.2264	816.4614	408.7343	8
3	**272.1241**	136.5657	254.1135	127.5604	V	**777.4505**	389.2289	760.4240	380.7156	759.4400	380.2236	7
4	**371.1925**	186.0999	353.1819	177.0946	V	**678.3821**	339.6947	661.3556	331.1814	660.3715	330.6894	6
5	**518.2609**	259.6341	**500.2504**	250.6288	F	**579.3137**	290.1605	562.2871	281.6472	**561.3031**	281.1552	5
6	575.2824	288.1448	557.2718	279.1396	G	**432.2453**	216.6263	415.2187	208.1130	414.2347	207.6210	4
7	**674.3508**	337.6790	656.3402	**328.6738**	V	375.2238	188.1155	358.1973	179.6023	357.2132	179.1103	3
8	**803.3934**	402.2003	785.3828	393.1951	E	**276.1554**	138.5813	259.1288	130.0681	**258.1448**	129.5761	2
9					K	**147.1128**	74.0600	130.0863	65.5468			1

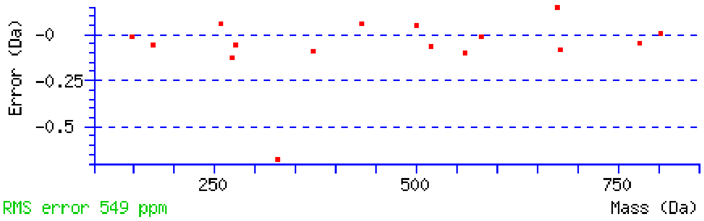

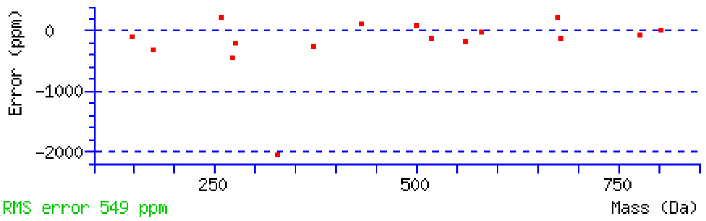

All matches to this query

Score	Mr(calc):	Delta	Sequence
27.0	948.4917	0.0051	DGVVFGVEK
8.2	948.4950	0.0017	KMTSPLEK
5.8	948.4916	0.0051	ENILFGEK
4.9	946.4946	2.0021	AVIAWMEK
4.8	948.4906	0.0061	GRGLLTPR
4.4	947.4924	1.0043	AVTDQTVSK
4.2	946.4906	2.0062	ACQKAIEK
4.1	947.4953	1.0014	KVKQHTK
3.9	947.4841	1.0126	ASLLTVHK
3.7	946.4832	2.0136	TEREREK

Spectrum No: 103; Query: 28119; Rank: 1

Peptide View

MS/MS Fragmentation of **ESLKEEDESDDDNM**
Found in **IPI00171199**, Tax_Id=9606 Gene_Symbol=PSMA3 Isoform 2 of Proteasome subunit alpha type-3

Match to Query 28119: 1654.620288 from(828.317420,2+)
Title: 091224LimSK_Exosome3_07.595.595.2.dta
Data file C:\Temp\Mascoti\LSK_2D\091224LimSK_Exosome3_01_2D.mgf

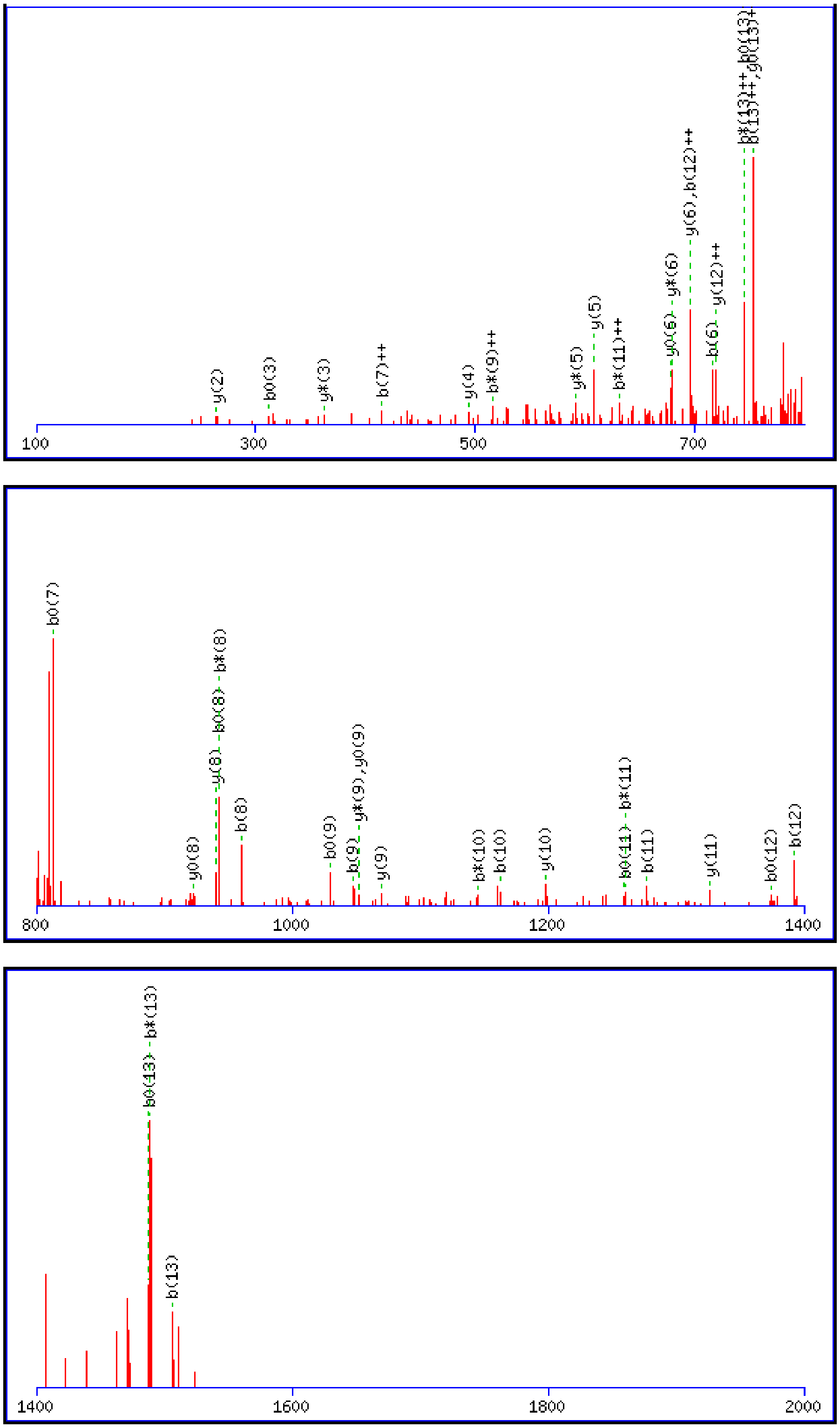

Monoisotopic mass of neutral peptide Mr(calc): 1654.6152
Fixed modifications: Carbamidomethyl (C)
Ions Score: 43 **Expect:** 0.0056
Matches (Bold Red): 42/144 fragment ions using 68 most intense peaks

#	b	b^{++}	b*	b^{*++}	b^{0}	b^{0++}	Seq.	y	y^{++}	y*	y^{*++}	y^{0}	y^{0++}	#
1	130.0499	65.5286			112.0393	56.5233	E							14
2	217.0819	109.0446			199.0713	100.0393	S	1526.5799	763.7936	1509.5533	755.2803	1508.5693	**754.7883**	13
3	330.1660	165.5866			**312.1554**	156.5813	L	1439.5479	**720.2776**	1422.5213	711.7643	1421.5373	711.2723	12
4	458.2609	229.6341	441.2344	221.1208	440.2504	220.6288	K	**1326.4638**	663.7355	1309.4372	655.2223	1308.4532	654.7303	11
5	587.3035	294.1554	570.2770	285.6421	569.2930	285.1501	E	**1198.3688**	599.6881	1181.3423	591.1748	1180.3583	590.6828	10
6	**716.3461**	358.6767	699.3196	350.1634	698.3355	349.6714	E	**1069.3262**	535.1668	**1052.2997**	526.6535	**1051.3157**	526.1615	9

7	831.3731	416.1902	814.3465	407.6769	813.3625	407.1849	D	940.2836	470.6455	923.2571	462.1322	922.2731	461.6402	8
8	960.4156	480.7115	943.3891	472.1982	942.4051	471.7062	E	825.2567	413.1320	808.2302	404.6187	807.2461	404.1267	7
9	1047.4477	524.2275	1030.4211	515.7142	1029.4371	515.2222	S	696.2141	348.6107	679.1876	340.0974	678.2035	339.6054	6
10	1162.4746	581.7409	1145.4481	573.2277	1144.4641	572.7357	D	609.1821	305.0947	592.1555	296.5814	591.1715	296.0894	5
11	1277.5016	639.2544	1260.4750	630.7411	1259.4910	630.2491	D	494.1551	247.5812	477.1286	239.0679	476.1446	238.5759	4
12	1392.5285	696.7679	1375.5020	688.2546	1374.5179	687.7626	D	379.1282	190.0677	362.1016	181.5545	361.1176	181.0625	3
13	1506.5714	753.7894	1489.5449	745.2761	1488.5609	744.7841	N	264.1013	132.5543	247.0747	124.0410			2
14							M	150.0583	75.5328					1

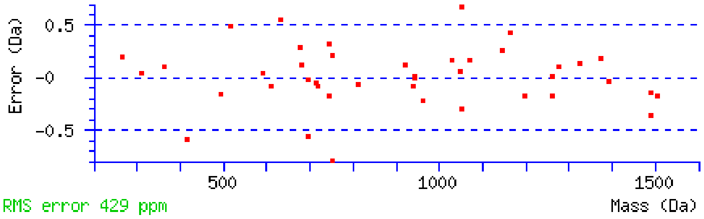

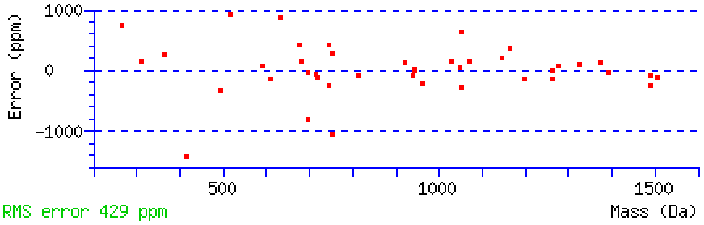

All matches to this query

Score	Mr(calc):	Delta	Sequence
42.6	1654.6152	0.0051	ESLKEEDESDDDNM
5.1	1654.6117	0.0086	SNHVLAQMEQGDCS
4.1	1652.6155	2.0048	VTVGDTSCTGQGPSK
4.0	1652.5977	2.0226	MSGTKLEDSPPCR
4.0	1652.5977	2.0226	MSGTKLEDSPPCR
4.0	1652.6042	2.0161	LAQESTMDIENDK
3.6	1652.6155	2.0048	VTVGDTSCTGQGPSK
2.9	1653.6284	0.9919	TEANAASISNNSTSK
2.2	1652.6155	2.0048	VTVGDTSCTGQGPSK

Spectrum No: 104; Query: 14250; Rank: 1

Peptide View

MS/MS Fragmentation of **HVGMAVAGLLADAR**
Found in **IPI00171199**, Tax_Id=9606 Gene_Symbol=PSMA3 Isoform 2 of Proteasome subunit alpha type-3

Match to Query 14250: 1379.745048 from(690.879800,2+)
Title: 091224LimSK_Exosome3_06.5159.5159.2.dta
Data file C:\Temp\Mascoti\LSK_2D\091224LimSK_Exosome3_01_2D.mgf

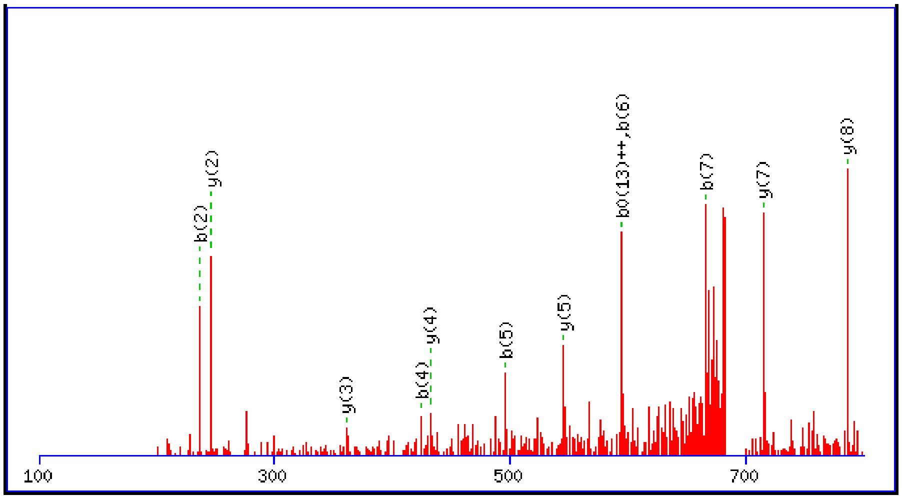

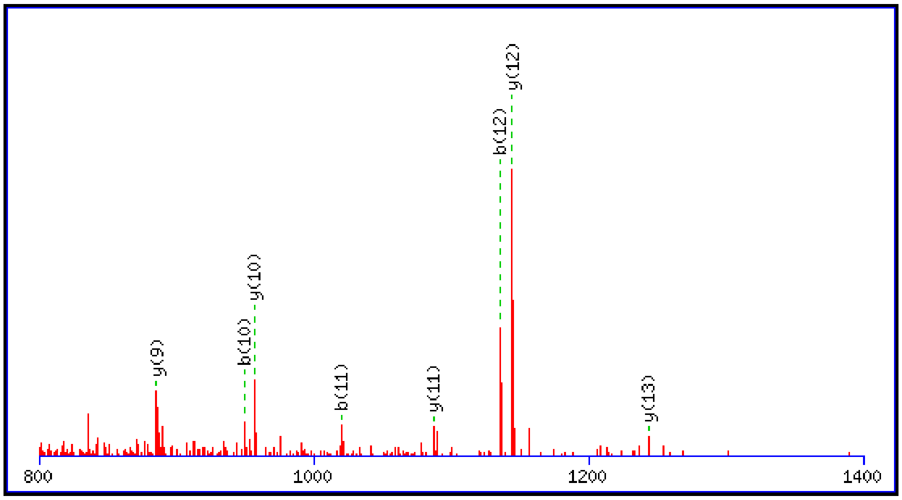

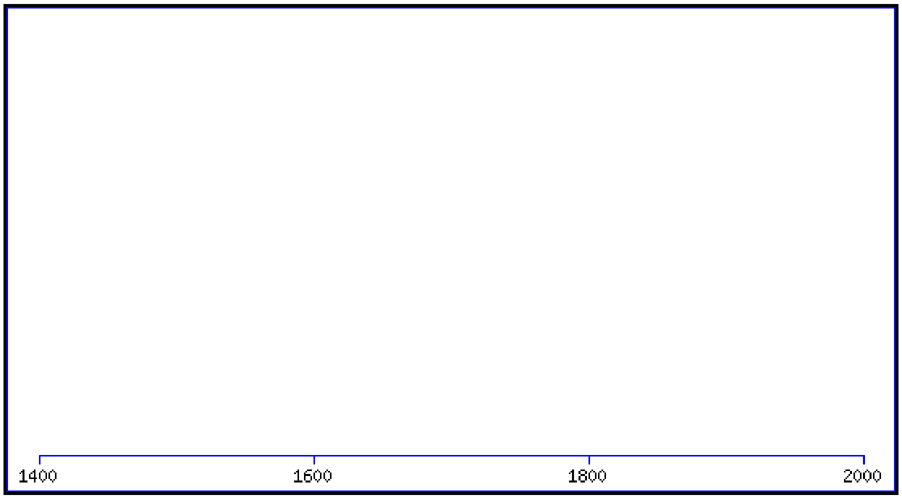

Monoisotopic mass of neutral peptide Mr(calc): 1379.7343
Fixed modifications: Carbamidomethyl (C)
Ions Score: 102 **Expect:** 7.8e-009
Matches (Bold Red): 20/104 fragment ions using 32 most intense peaks

#	b	b^{++}	b^{0}	b^{0++}	Seq.	y	y^{++}	y^{*}	y^{*++}	y^{0}	y^{0++}	#
1	138.0662	69.5367			H							14
2	**237.1346**	119.0709			V	**1243.6827**	622.3450	1226.6562	613.8317	1225.6722	613.3397	13
3	294.1561	147.5817			G	**1144.6143**	572.8108	1127.5878	564.2975	1126.6037	563.8055	12
4	**425.1966**	213.1019			M	**1087.5928**	544.3001	1070.5663	535.7868	1069.5823	535.2948	11
5	**496.2337**	248.6205			A	**956.5524**	478.7798	939.5258	470.2665	938.5418	469.7745	10
6	**595.3021**	298.1547			V	**885.5152**	443.2613	868.4887	434.7480	867.5047	434.2560	9

**7**	**666.3392**	333.6732			**A**	**786.4468**	393.7271	769.4203	385.2138	768.4363	384.7218	**8**
**8**	723.3607	362.1840			**G**	**715.4097**	358.2085	698.3832	349.6952	697.3992	349.2032	**7**
**9**	836.4447	418.7260			**L**	658.3883	329.6978	641.3617	321.1845	640.3777	320.6925	**6**
**10**	**949.5288**	475.2680			**L**	**545.3042**	273.1557	528.2776	264.6425	527.2936	264.1504	**5**
**11**	**1020.5659**	510.7866			**A**	**432.2201**	216.6137	415.1936	208.1004	414.2096	207.6084	**4**
**12**	**1135.5928**	568.3001	1117.5823	559.2948	**D**	**361.1830**	181.0951	344.1565	172.5819	343.1724	172.0899	**3**
**13**	1206.6300	603.8186	1188.6194	**594.8133**	**A**	**246.1561**	123.5817	229.1295	115.0684			**2**
**14**					**R**	175.1190	88.0631	158.0924	79.5498			**1**

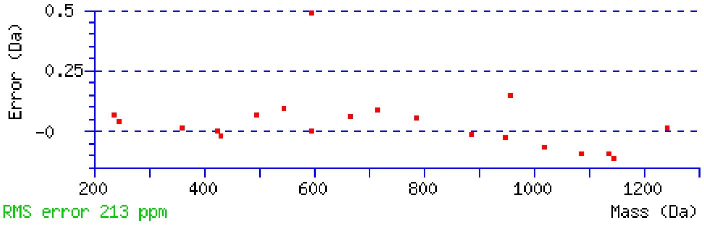

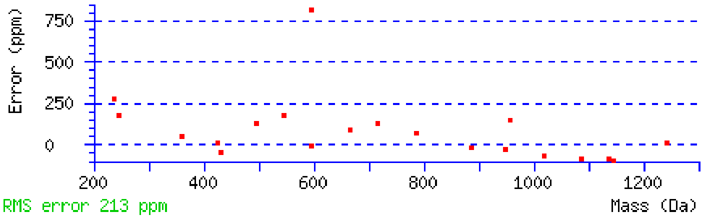

All matches to this query

Score	Mr(calc):	Delta	Sequence
101.6	1379.7343	0.0107	HVGMAVAGLLADAR

Spectrum No: 105; Query: 8867; Rank: 1

Peptide View

MS/MS Fragmentation of **IIYIVHDEVK**
Found in **IPI00171199**, Tax_Id=9606 Gene_Symbol=PSMA3 Isoform 2 of Proteasome subunit alpha type-3

Match to Query 8867: 1227.692468 from(614.853510,2+)
Title: 091224LimSK_Exosome3_06.1885.1885.2.dta
Data file C:\Temp\Mascoti\LSK_2D\091224LimSK_Exosome3_01_2D.mgf

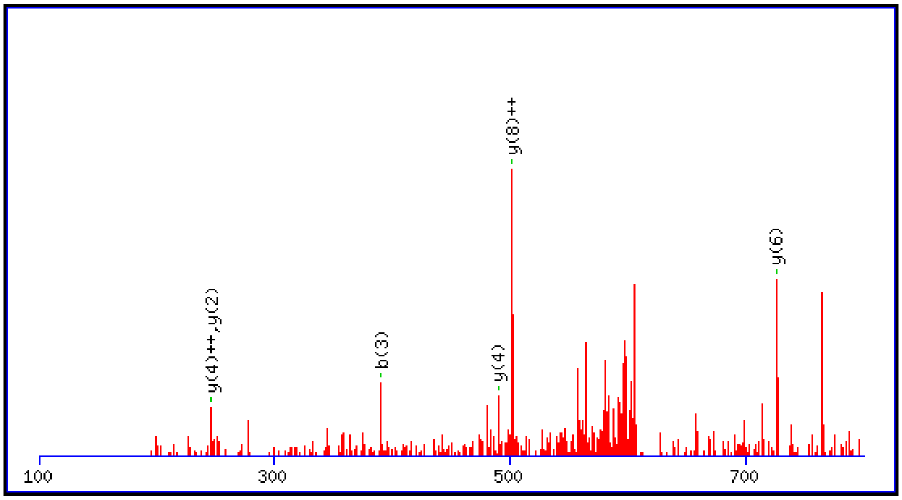

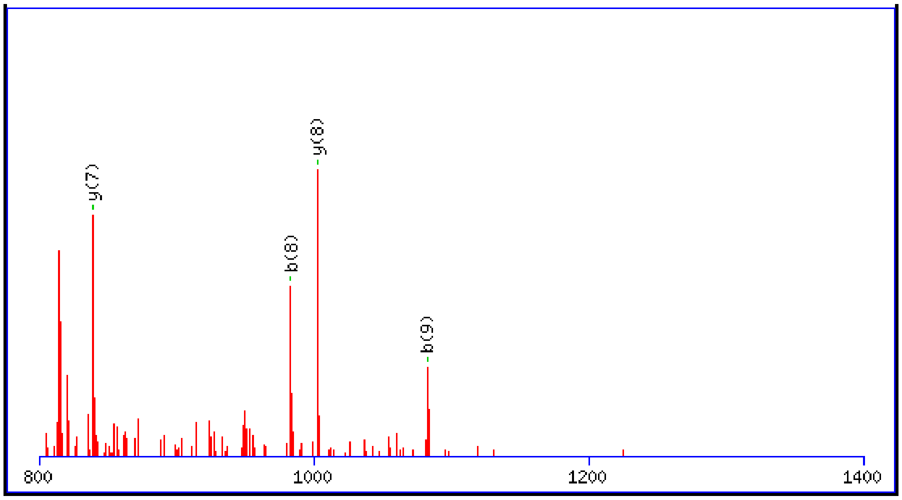

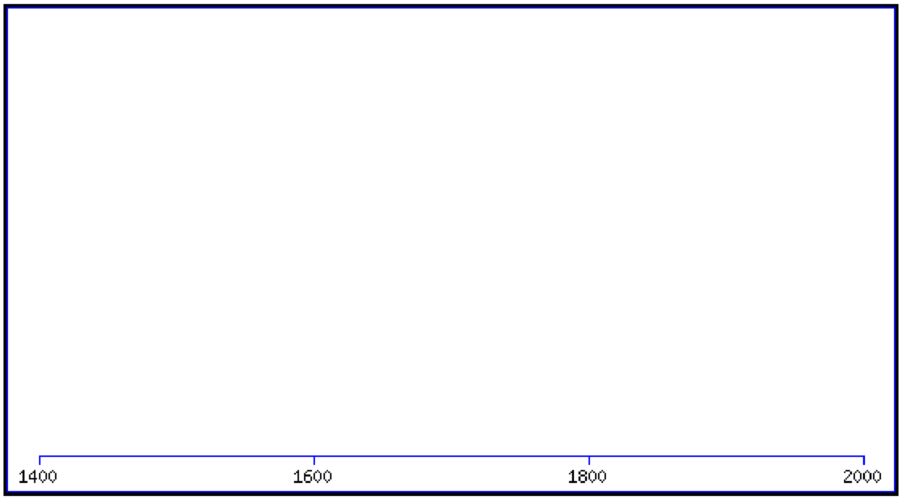

Monoisotopic mass of neutral peptide Mr(calc): 1227.6863
Fixed modifications: Carbamidomethyl (C)
Ions Score: 37 **Expect:** 0.019
Matches (Bold Red): 10/74 fragment ions using 23 most intense peaks

#	b	b^{++}	b^0	b^{0++}	Seq.	y	y^{++}	y*	y^{*++}	y^0	y^{0++}	#
1	114.0913	57.5493			I							10
2	227.1754	114.0913			I	1115.6095	558.3084	1098.5830	549.7951	1097.5990	549.3031	9
3	**390.2387**	195.6230			Y	**1002.5255**	**501.7664**	985.4989	493.2531	984.5149	492.7611	8
4	503.3228	252.1650			I	**839.4621**	420.2347	822.4356	411.7214	821.4516	411.2294	7
5	602.3912	301.6992			V	**726.3781**	363.6927	709.3515	355.1794	708.3675	354.6874	6
6	739.4501	370.2287			H	627.3097	314.1585	610.2831	305.6452	609.2991	305.1532	5
7	854.4771	427.7422	836.4665	418.7369	D	**490.2508**	**245.6290**	473.2242	237.1157	472.2402	236.6237	4
8	**983.5197**	492.2635	965.5091	483.2582	E	375.2238	188.1155	358.1973	179.6023	357.2132	179.1103	3
9	**1082.5881**	541.7977	1064.5775	532.7924	V	**246.1812**	123.5942	229.1547	115.0810			2
10					K	147.1128	74.0600	130.0863	65.5468			1

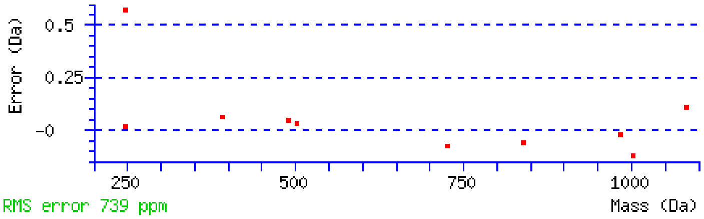

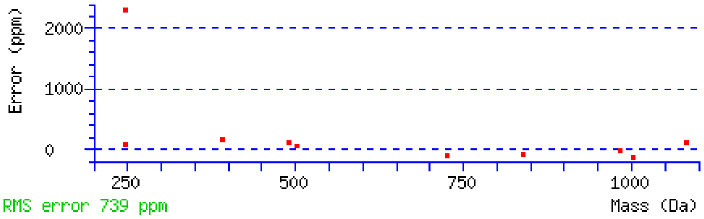

All matches to this query

Score	Mr(calc):	Delta	Sequence
36.6	1227.6863	0.0062	IIYIVHDEVK
5.3	1227.6976	-0.0051	FIGPSPEVVRK
4.8	1227.6823	0.0102	IVAEAIEKAER
3.5	1227.6836	0.0088	EFVHGRLKSR
2.2	1227.6992	-0.0067	LLYKASVVKK
1.4	1227.6935	-0.0011	VRDDLQKQVK
0.1	1226.6996	0.9929	FRPAGPRATVR

Spectrum No: 106; Query: 19026; Rank: 1

Peptide View

MS/MS Fragmentation of **IIYIVHDEVKDK**
Found in **IPI00171199**, Tax_Id=9606 Gene_Symbol=PSMA3 Isoform 2 of Proteasome subunit alpha type-3

Match to Query 19026: 1470.816688 from(736.415620,2+)
Title: 091224LimSK_Exosome3_05.1438.1438.2.dta
Data file C:\Temp\Mascoti\LSK_2D\091224LimSK_Exosome3_01_2D.mgf

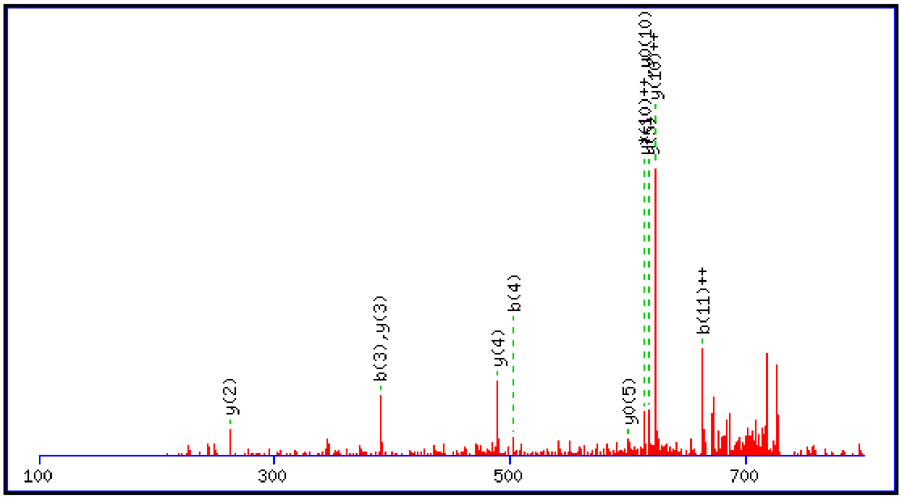

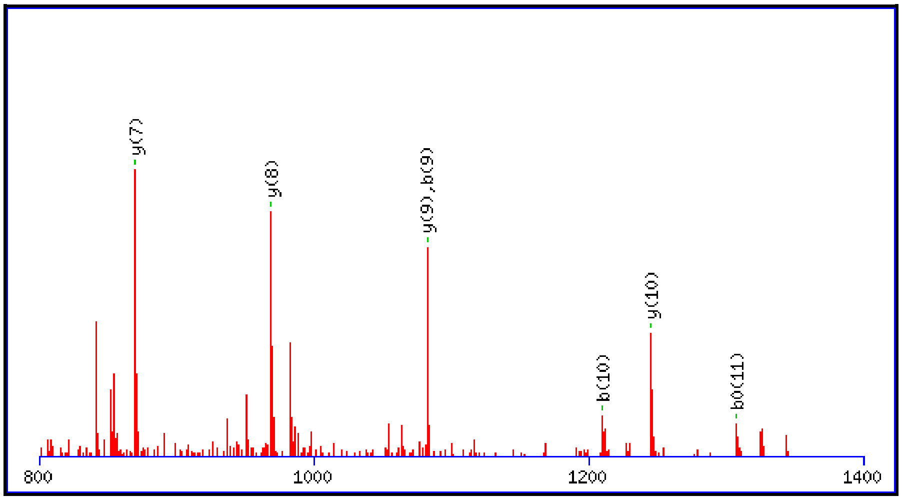

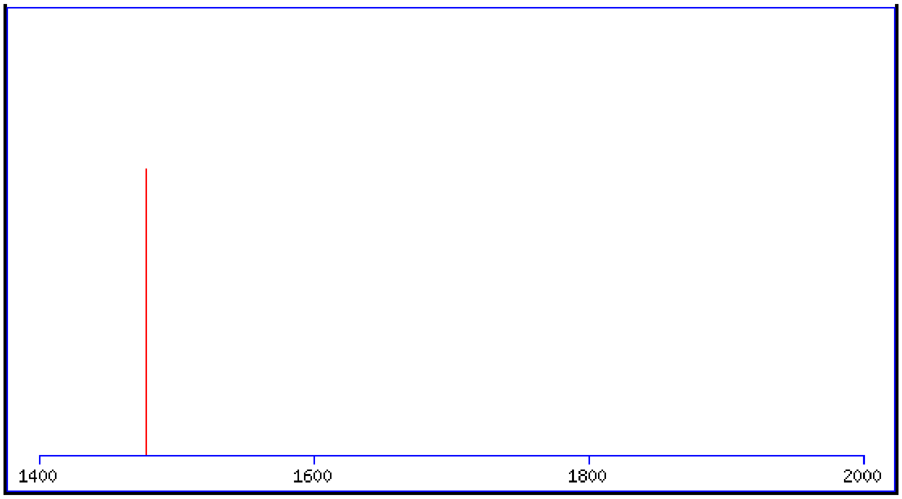

Monoisotopic mass of neutral peptide Mr(calc): 1470.8082
Fixed modifications: Carbamidomethyl (C)
Ions Score: 54 **Expect:** 0.00032
Matches (Bold Red): 18/100 fragment ions using 24 most intense peaks

#	b	b^{++}	b*	b^{*++}	b^{0}	b^{0++}	Seq.	y	y^{++}	y*	y^{*++}	y^{0}	y^{0++}	#
1	114.0913	57.5493					I							12
2	227.1754	114.0913					I	1358.7314	679.8694	1341.7049	671.3561	1340.7209	670.8641	11
3	**390.2387**	195.6230					Y	**1245.6474**	**623.3273**	1228.6208	**614.8141**	1227.6368	**614.3220**	10
4	**503.3228**	252.1650					I	**1082.5841**	541.7957	1065.5575	533.2824	1064.5735	532.7904	9
5	602.3912	301.6992					V	**969.5000**	485.2536	952.4734	476.7404	951.4894	476.2483	8
6	739.4501	370.2287					H	**870.4316**	435.7194	853.4050	427.2061	852.4210	426.7141	7
7	854.4771	427.7422			836.4665	418.7369	D	733.3727	367.1900	716.3461	358.6767	715.3621	358.1847	6
8	983.5197	492.2635			965.5091	483.2582	E	**618.3457**	309.6765	601.3192	301.1632	**600.3352**	300.6712	5
9	**1082.5881**	541.7977			1064.5775	532.7924	V	**489.3031**	245.1552	472.2766	236.6419	471.2926	236.1499	4
10	**1210.6830**	605.8452	1193.6565	597.3319	1192.6725	596.8399	K	**390.2347**	195.6210	373.2082	187.1077	372.2241	186.6157	3
11	1325.7100	**663.3586**	1308.6834	654.8454	**1307.6994**	654.3533	D	**262.1397**	131.5735	245.1132	123.0602	244.1292	122.5682	2
12							K	147.1128	74.0600	130.0863	65.5468			1

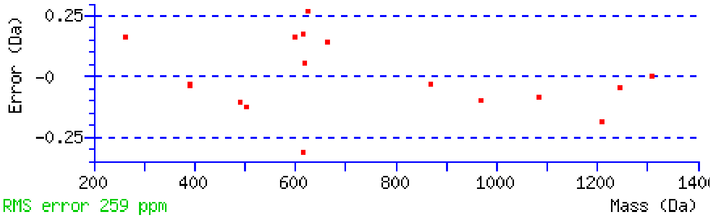

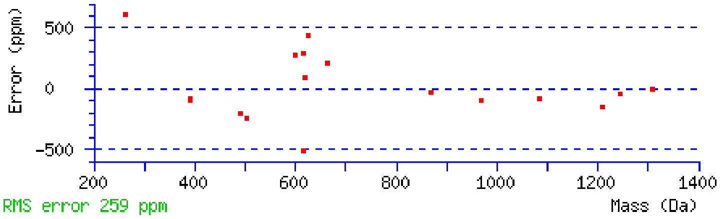

All matches to this query

Score	Mr(calc):	Delta	Sequence
54.2	1470.8082	0.0085	IIYIVHDEVKDK
16.4	1470.8042	0.0125	LLDKKQQENIDK
11.3	1470.8042	0.0125	KDQDQIIAGVEKK
10.7	1470.8099	0.0068	YTTILTPKTIIK
7.1	1470.8099	0.0068	YTTILTPKTIIK
5.8	1469.8143	1.0024	QPYSRFLLRYK
5.6	1469.8201	0.9966	AKERQLENLIEK
5.2	1470.8154	0.0013	NITRGQSLDNLIK
4.9	1469.8089	1.0078	KEEKKPIETGSPK
4.7	1470.8129	0.0038	XSCRVAGWVAKLK

Spectrum No: 107; Query: 6296; Rank: 1

Peptide View

MS/MS Fragmentation of **SNFGYNIPLK**
Found in **IPI00171199**, Tax_Id=9606 Gene_Symbol=PSMA3 Isoform 2 of Proteasome subunit alpha type-3

Match to Query 6296: 1151.602728 from(576.808640,2+)
Title: 091224LimSK_Exosome3_06.3765.3765.2.dta
Data file C:\Temp\Mascoti\LSK_2D\091224LimSK_Exosome3_01_2D.mgf

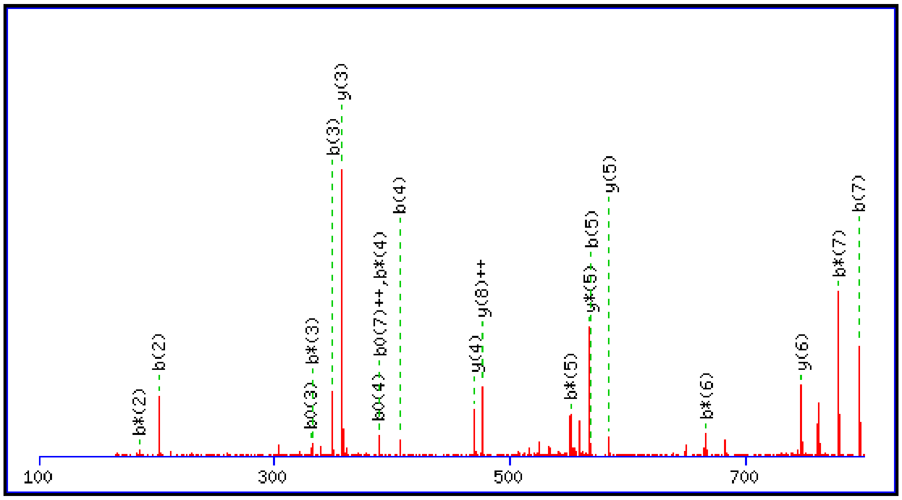

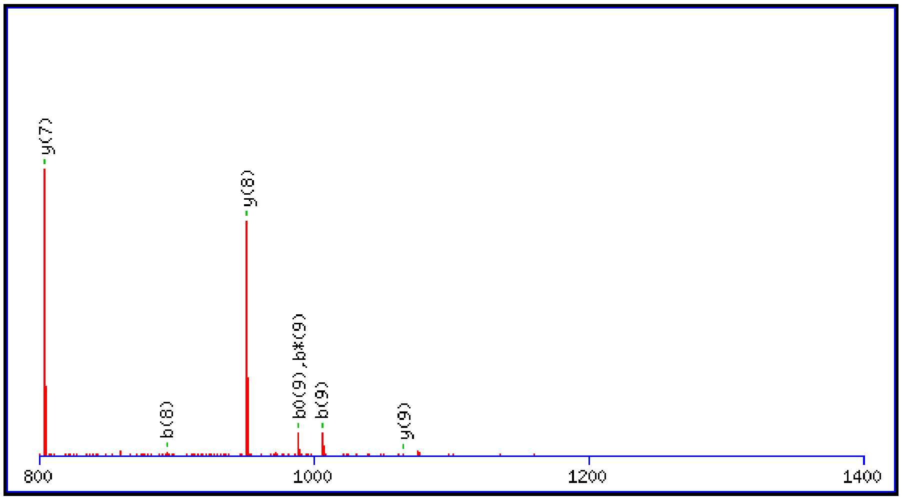

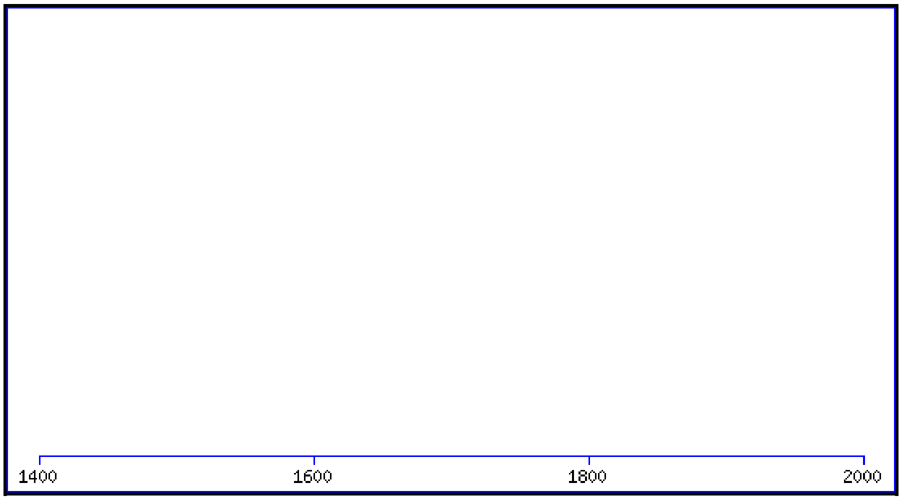

Monoisotopic mass of neutral peptide Mr(calc): 1151.5975
Fixed modifications: Carbamidomethyl (C)
Ions Score: 61 **Expect:** 0.00012
Matches (Bold Red): 27/88 fragment ions using 38 most intense peaks

#	b	b^{++}	b*	b^{*++}	b^0	b^{0++}	Seq.	y	y^{++}	y*	y^{*++}	#
1	88.0393	44.5233			70.0287	35.5180	S					10
2	**202.0822**	101.5448	**185.0557**	93.0315	184.0717	92.5395	N	**1065.5728**	533.2900	1048.5462	524.7767	9
3	**349.1506**	175.0790	**332.1241**	166.5657	**331.1401**	166.0737	F	**951.5298**	**476.2686**	934.5033	467.7553	8
4	**406.1721**	203.5897	**389.1456**	195.0764	**388.1615**	194.5844	G	**804.4614**	402.7343	787.4349	394.2211	7
5	**569.2354**	285.1214	**552.2089**	276.6081	551.2249	276.1161	Y	**747.4400**	374.2236	730.4134	365.7103	6
6	683.2784	342.1428	**666.2518**	333.6295	665.2678	333.1375	N	**584.3766**	292.6919	**567.3501**	284.1787	5
7	**796.3624**	398.6849	**779.3359**	390.1716	778.3519	**389.6796**	I	**470.3337**	235.6705	453.3071	227.1572	4
8	**893.4152**	447.2112	876.3886	438.6980	875.4046	438.2060	P	**357.2496**	179.1285	340.2231	170.6152	3
9	**1006.4993**	503.7533	**989.4727**	495.2400	**988.4887**	494.7480	L	260.1969	130.6021	243.1703	122.0888	2
10							K	147.1128	74.0600	130.0863	65.5468	1

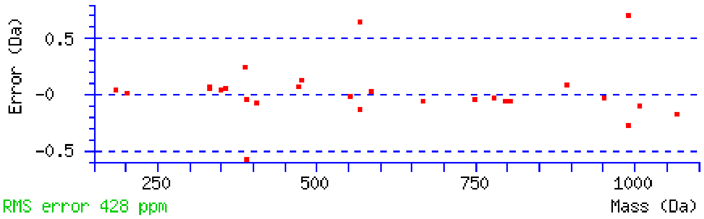

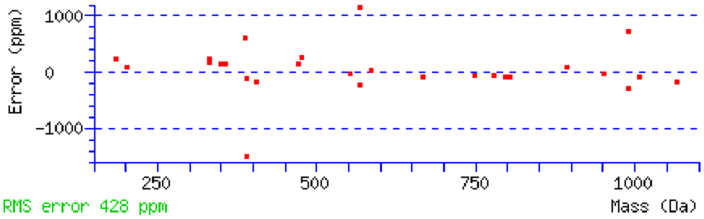

All matches to this query

Score	Mr(calc):	Delta	Sequence
60.9	1151.5975	0.0052	SNFGYNIPLK
13.4	1151.5951	0.0076	SVLAVGTANIK
12.9	1151.6025	0.0002	SLTLGIPMLK
8.2	1151.6063	-0.0036	RGELQTKLK
7.9	1151.6063	-0.0036	SRLVLLSER
7.2	1151.6047	-0.0020	SLRDPASPPGR
6.9	1151.5951	0.0076	VLKEGGSLAAK
6.1	1151.6063	-0.0036	KLDQTIARK
6.0	1149.6046	1.9981	TSAELVLPIK
4.8	1150.5999	1.0029	TSGKVELLPK

Spectrum No: 108; Query: 5157; Rank: 1

Peptide View

MS/MS Fragmentation of **VFQVEYAMK**
Found in **IPI00171199**, Tax_Id=9606 Gene_Symbol=PSMA3 Isoform 2 of Proteasome subunit alpha type-3

Match to Query 5157: 1113.558048 from(557.786300,2+)
Title: 091224LimSK_Exosome3_05.3026.3026.2.dta
Data file C:\Temp\Mascoti\LSK_2D\091224LimSK_Exosome3_01_2D.mgf

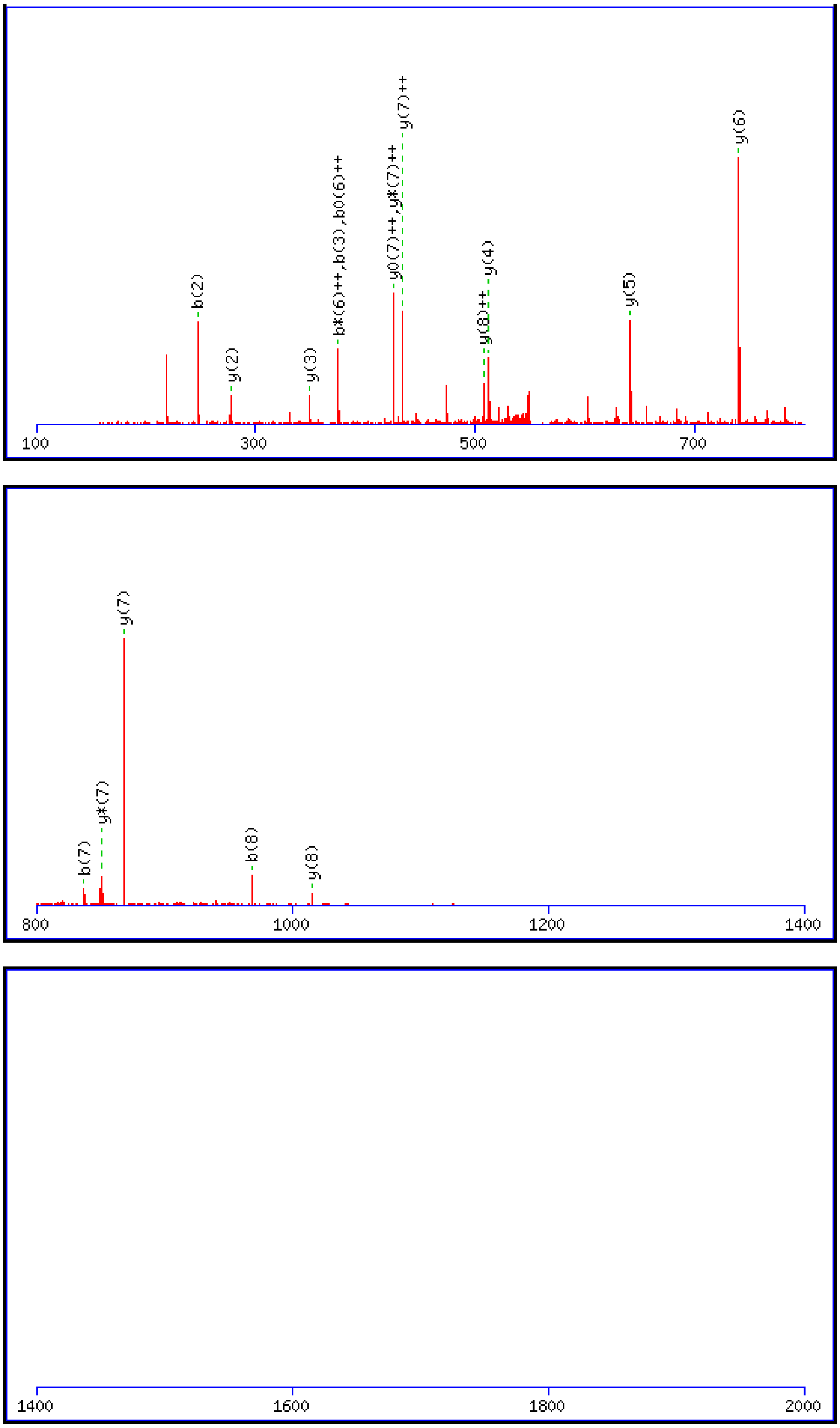

Monoisotopic mass of neutral peptide Mr(calc): 1113.5529
Fixed modifications: Carbamidomethyl (C)
Ions Score: 57 **Expect:** 0.00026
Matches (Bold Red): 18/76 fragment ions using 32 most intense peaks

#	b	b++	b*	b*++	b0	b0++	Seq.	y	y++	y*	y*++	y0	y0++	#
1	100.0757	50.5415					V							9
2	**247.1441**	124.0757					F	**1015.4917**	**508.2495**	998.4652	499.7362	997.4812	499.2442	8
3	**375.2027**	188.1050	358.1761	179.5917			Q	**868.4233**	**434.7153**	**851.3968**	**426.2020**	850.4128	**425.7100**	7
4	474.2711	237.6392	457.2445	229.1259			V	**740.3647**	370.6860	723.3382	362.1727	722.3542	361.6807	6
5	603.3137	302.1605	586.2871	293.6472	585.3031	293.1552	E	**641.2963**	321.1518	624.2698	312.6385	623.2858	312.1465	5
6	766.3770	383.6921	749.3505	**375.1789**	748.3665	**374.6869**	Y	**512.2537**	256.6305	495.2272	248.1172			4

7	837.4141	419.2107	820.3876	410.6974	819.4036	410.2054	A	349.1904	175.0988	332.1639	166.5856			3
8	968.4546	484.7309	951.4281	476.2177	950.4441	475.7257	M	278.1533	139.5803	261.1267	131.0670			2
9							K	147.1128	74.0600	130.0863	65.5468			1

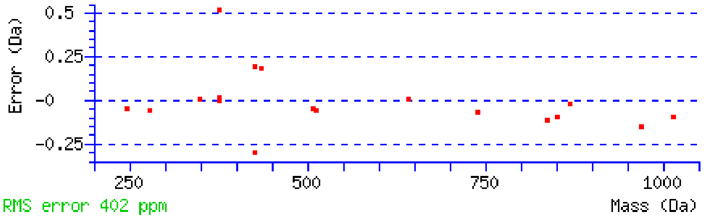

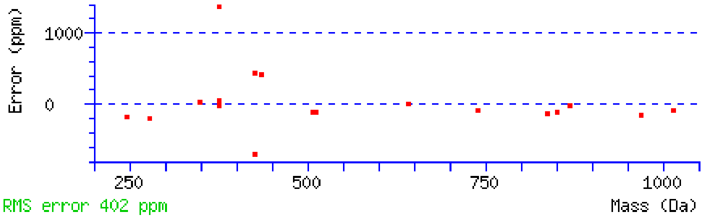

All matches to this query

Score	Mr(calc):	Delta	Sequence
57.0	1113.5529	0.0052	VFQVEYAMK
23.1	1111.5526	2.0055	LLTDSDLKK
14.6	1111.5526	2.0055	LLTDSDLKK
13.9	1111.5526	2.0055	LITTTEQVK
13.9	1111.5526	2.0055	LITTTEQVK
13.9	1111.5526	2.0055	LITTTEQVK
10.9	1112.5478	1.0102	VETLSQTKK
10.5	1111.5427	2.0154	SLNKEWKK
10.4	1111.5526	2.0055	LELSDLSKK
10.4	1112.5631	0.9950	LIQAAYQVK

Spectrum No: 109; Query: 11548; Rank: 1

Peptide View

MS/MS Fragmentation of **FILNLPTFSVR**
Found in **IPI00028006**, Tax_Id=9606 Gene_Symbol=PSMB2 Proteasome subunit beta type-2

Match to Query 11548: 1305.751788 from(653.883170,2+)
Title: 091224LimSK_Exosome3_06.7754.7754.2.dta
Data file C:\Temp\Mascoti\LSK_2D\091224LimSK_Exosome3_01_2D.mgf

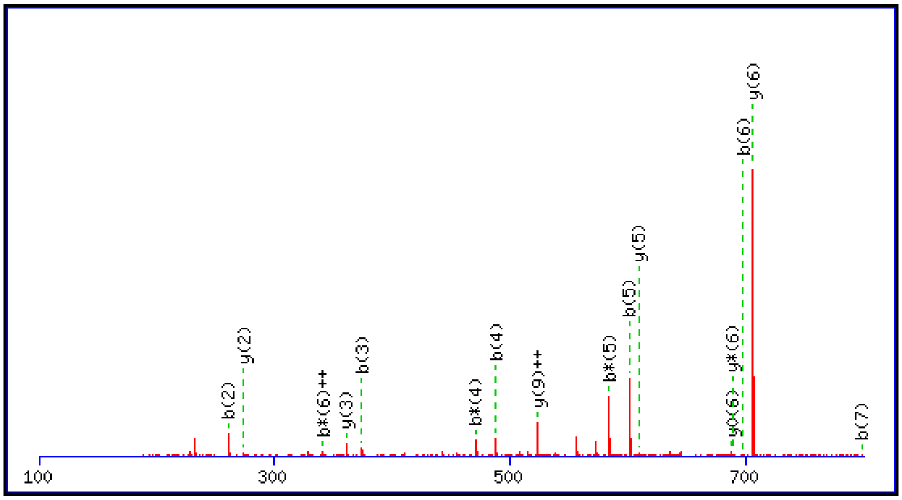

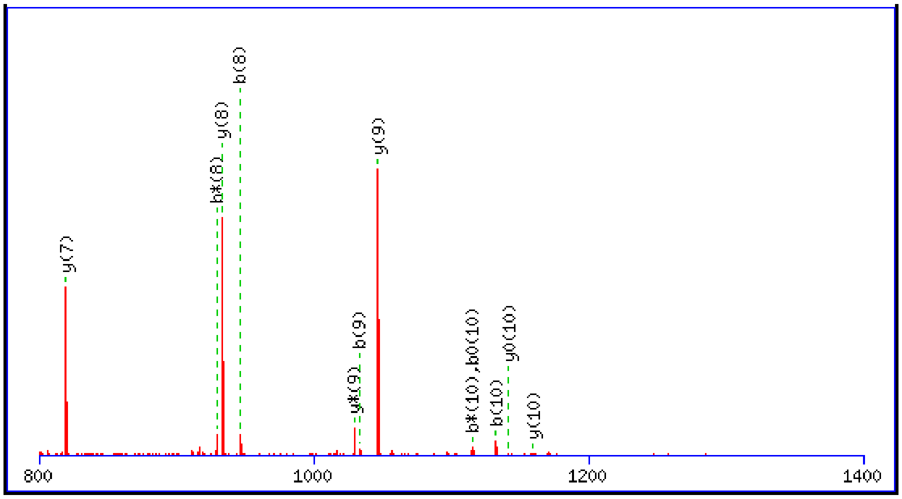

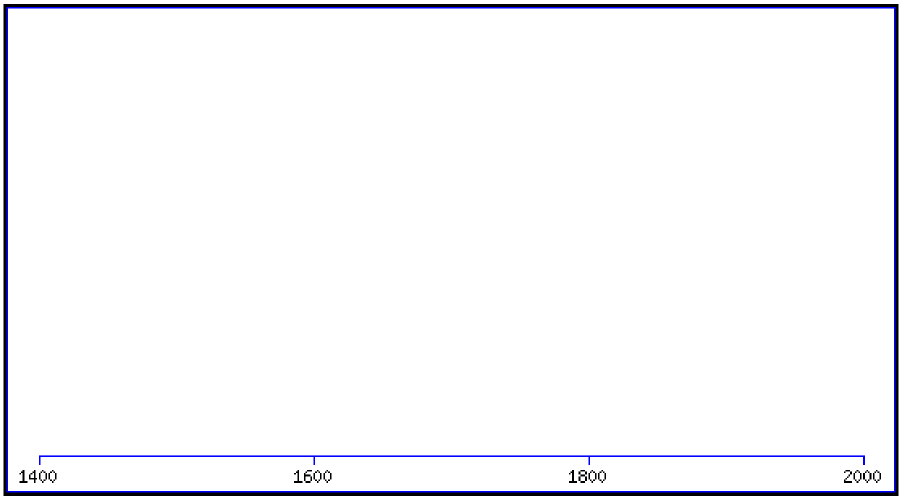

Monoisotopic mass of neutral peptide Mr(calc): 1305.7445
Fixed modifications: Carbamidomethyl (C)
Ions Score: 55 **Expect:** 0.00019
Matches (Bold Red): 28/98 fragment ions using 62 most intense peaks

#	b	b^{++}	b^*	b^{*++}	b^0	b^{0++}	Seq.	y	y^{++}	y^*	y^{*++}	y^0	y^{0++}	#
1	148.0757	74.5415					F							11
2	**261.1598**	131.0835					I	**1159.6834**	580.3453	1142.6568	571.8320	**1141.6728**	571.3400	10
3	**374.2438**	187.6255					L	**1046.5993**	**523.8033**	**1029.5728**	515.2900	1028.5887	514.7980	9
4	**488.2867**	244.6470	**471.2602**	236.1337			N	**933.5152**	467.2613	916.4887	458.7480	915.5047	458.2560	8
5	**601.3708**	301.1890	**584.3443**	292.6758			L	**819.4723**	410.2398	802.4458	401.7265	801.4617	401.2345	7
6	**698.4236**	349.7154	681.3970	**341.2022**			P	**706.3883**	353.6978	**689.3617**	345.1845	**688.3777**	344.6925	6
7	**799.4713**	400.2393	782.4447	391.7260	781.4607	391.2340	T	**609.3355**	305.1714	592.3089	296.6581	591.3249	296.1661	5
8	**946.5397**	473.7735	**929.5131**	465.2602	928.5291	464.7682	F	508.2878	254.6475	491.2613	246.1343	490.2772	245.6423	4
9	**1033.5717**	517.2895	1016.5451	508.7762	1015.5611	508.2842	S	**361.2194**	181.1133	344.1928	172.6001	343.2088	172.1081	3
10	**1132.6401**	566.8237	**1115.6136**	558.3104	**1114.6295**	557.8184	V	**274.1874**	137.5973	257.1608	129.0840			2
11							R	175.1190	88.0631	158.0924	79.5498			1

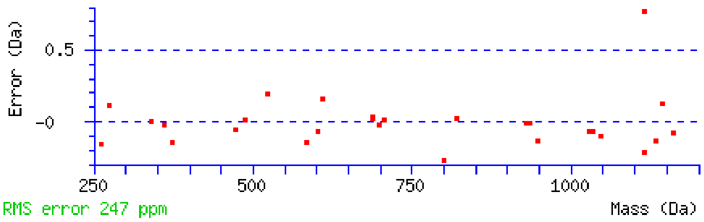

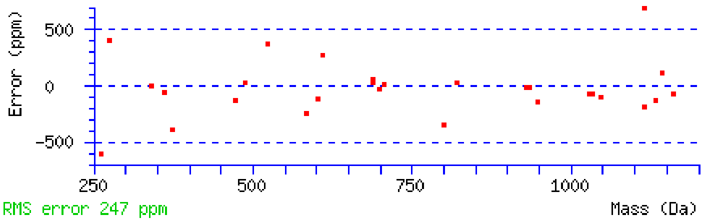

All matches to this query

Score	Mr(calc):	Delta	Sequence
54.8	1305.7445	0.0073	FILNLPTFSVR
9.7	1305.7517	0.0001	NAGQVVHLTLVR
7.0	1305.7478	0.0039	MLEFVSLLARK
5.9	1305.7517	0.0001	HRAAEAAINILK
5.1	1305.7478	0.0039	RIYLCLDLLK
4.8	1304.7452	1.0066	IHETQPLLNLK
2.1	1305.7517	0.0001	QAPIPAELRGVR
0.8	1305.7405	0.0113	RFEVSSSLILR
0.5	1305.7421	0.0097	GLLSRVVLELK

Spectrum No: 110; Query: 37606; Rank: 1

Peptide View

MS/MS Fragmentation of **IIDKNGIHDLDNISFPK**
Found in **IPI00028006**, Tax_Id=9606 Gene_Symbol=PSMB2 Proteasome subunit beta type-2

Match to Query 37606: 1939.015002 from(647.345610,3+)
Title: 091224LimSK_Exosome3_06.5301.5301.3.dta
Data file C:\Temp\Mascoti\LSK_2D\091224LimSK_Exosome3_01_2D.mgf

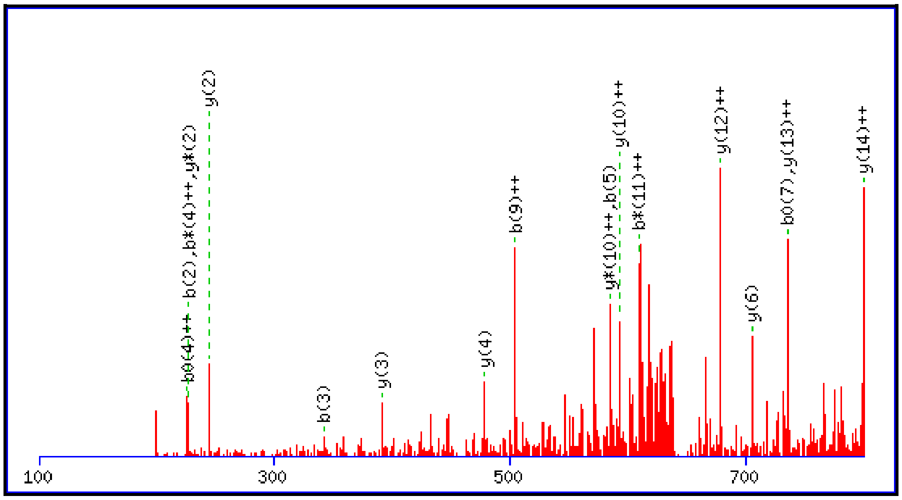

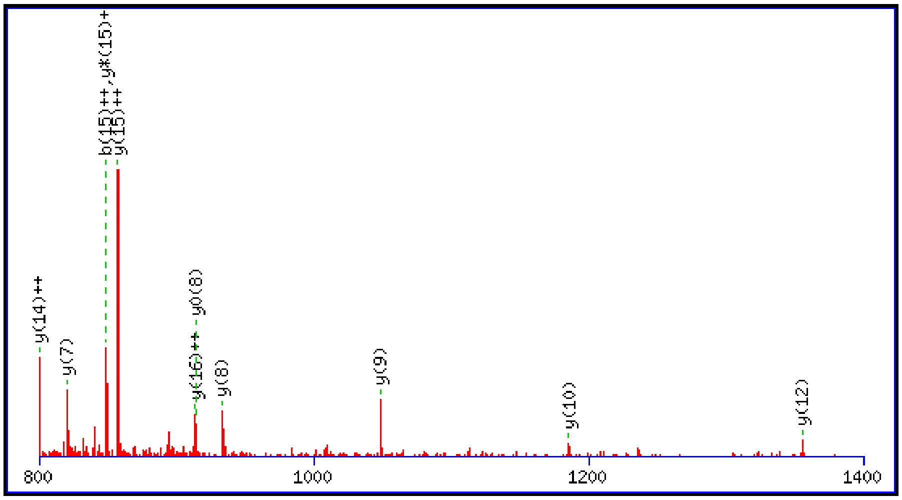

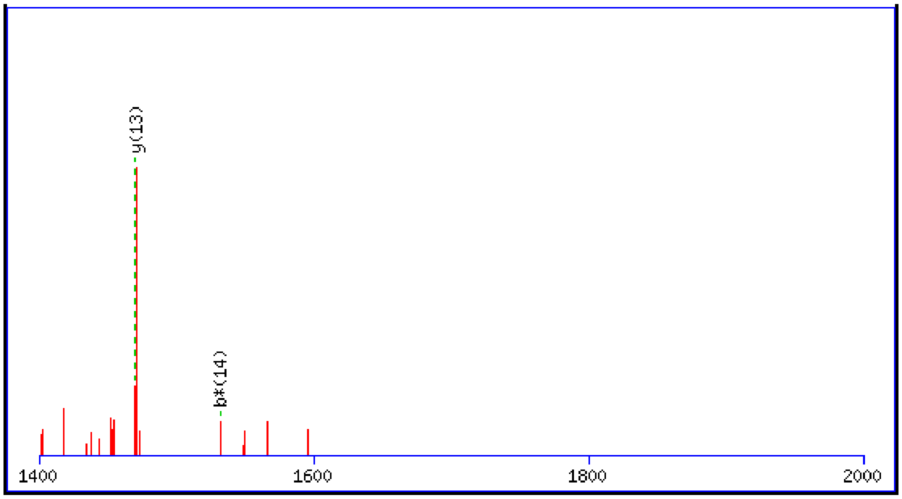

Monoisotopic mass of neutral peptide Mr(calc): 1938.0211
Fixed modifications: Carbamidomethyl (C)
Ions Score: 46 **Expect:** 0.0031
Matches (Bold Red): 31/176 fragment ions using 46 most intense peaks

#	b	b^{++}	b*	b^{*++}	b^{0}	b^{0++}	Seq.	y	y^{++}	y*	y^{*++}	y^{0}	y^{0++}	#
1	114.0913	57.5493					I							17
2	**227.1754**	114.0913					I	1825.9443	**913.4758**	1808.9177	904.9625	1807.9337	904.4705	16
3	**342.2023**	171.6048			324.1918	162.5995	D	1712.8602	**856.9338**	1695.8337	**848.4205**	1694.8497	**847.9285**	15
4	470.2973	235.6523	453.2708	**227.1390**	452.2867	**226.6470**	K	1597.8333	**799.4203**	1580.8067	790.9070	1579.8227	790.4150	14
5	**584.3402**	292.6738	567.3137	284.1605	566.3297	283.6685	N	**1469.7383**	**735.3728**	1452.7118	726.8595	1451.7278	726.3675	13
6	641.3617	321.1845	624.3352	312.6712	623.3511	312.1792	G	**1355.6954**	**678.3513**	1338.6688	669.8381	1337.6848	669.3461	12
7	754.4458	377.7265	737.4192	369.2132	**736.4352**	368.7212	I	1298.6739	649.8406	1281.6474	641.3273	1280.6634	640.8353	11
8	891.5047	446.2560	874.4781	437.7427	873.4941	437.2507	H	**1185.5899**	**593.2986**	1168.5633	**584.7853**	1167.5793	584.2933	10
9	1006.5316	**503.7694**	989.5051	495.2562	988.5211	494.7642	D	**1048.5310**	524.7691	1031.5044	516.2558	1030.5204	515.7638	9
10	1119.6157	560.3115	1102.5891	551.7982	1101.6051	551.3062	L	**933.5040**	467.2556	916.4775	458.7424	**915.4934**	458.2504	8
11	1234.6426	617.8250	1217.6161	**609.3117**	1216.6321	608.8197	D	**820.4199**	410.7136	803.3934	402.2003	802.4094	401.7083	7
12	1348.6856	674.8464	1331.6590	666.3331	1330.6750	665.8411	N	**705.3930**	353.2001	688.3665	344.6869	687.3824	344.1949	6
13	1461.7696	731.3884	1444.7431	722.8752	1443.7591	722.3832	I	591.3501	296.1787	574.3235	287.6654	573.3395	287.1734	5
14	1548.8016	774.9045	**1531.7751**	766.3912	1530.7911	765.8992	S	**478.2660**	239.6366	461.2395	231.1234	460.2554	230.6314	4
15	1695.8701	**848.4387**	1678.8435	839.9254	1677.8595	839.4334	F	**391.2340**	196.1206	374.2074	187.6074			3
16	1792.9228	896.9651	1775.8963	888.4518	1774.9123	887.9598	P	**244.1656**	122.5864	**227.1390**	114.0731			2
17							K	147.1128	74.0600	130.0863	65.5468			1

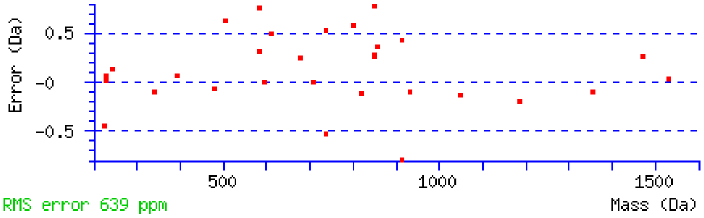

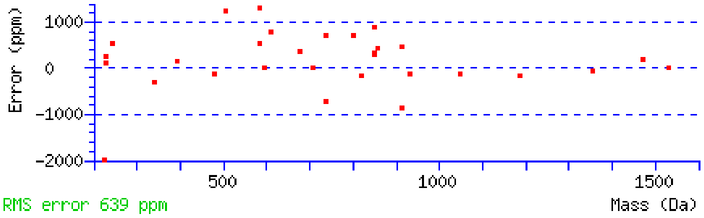

All matches to this query

Score	Mr(calc):	Delta	Sequence
46.5	1938.0211	0.9939	IIDKNGIHDLDNISFPK
4.0	1938.0210	0.9940	LLNLEDAQSPPSAFIAPR
3.6	1939.0309	-0.0159	AVGLEDQIVSQGIPMRAR
3.6	1938.9986	0.0164	NKTTCVPVQLHPSSFPK
0.4	1937.9937	1.0213	MGSSPVIKLPLLYPSEK

0.0	1939.0040	0.0110	KDSLVLLGRVPAHPDSR

Spectrum No: 111; Query: 52854; Rank: 1

Peptide View

MS/MS Fragmentation of **ILLLCVGEAGDTVQFAEYIQK**
Found in **IPI00028006**, Tax_Id=9606 Gene_Symbol=PSMB2 Proteasome subunit beta type-2

Match to Query 52854: 2367.239082 from(790.086970,3+)
Title: 091224LimSK_Exosome3_06.9656.9656.3.dta
Data file C:\Temp\Mascoti\LSK_2D\091224LimSK_Exosome3_01_2D.mgf

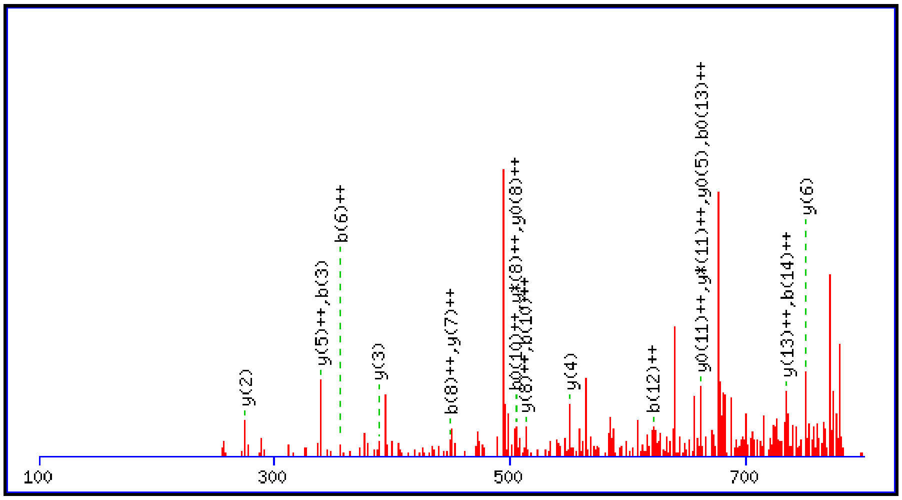

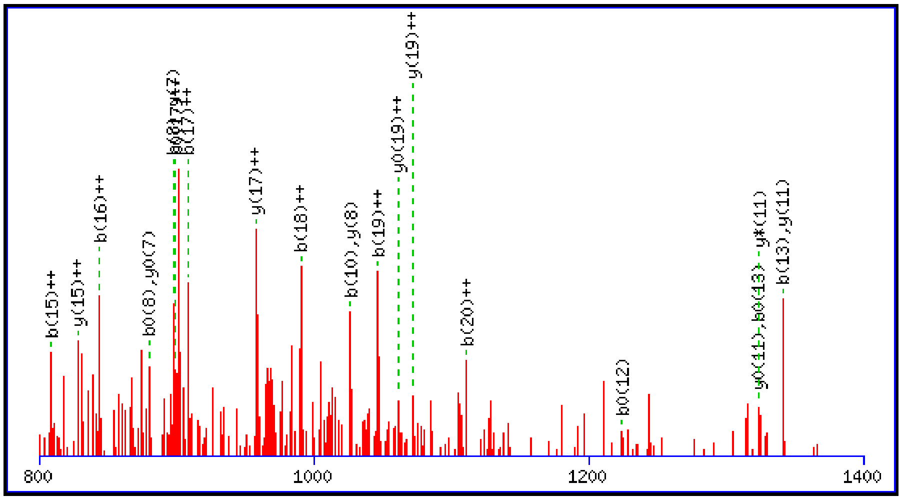

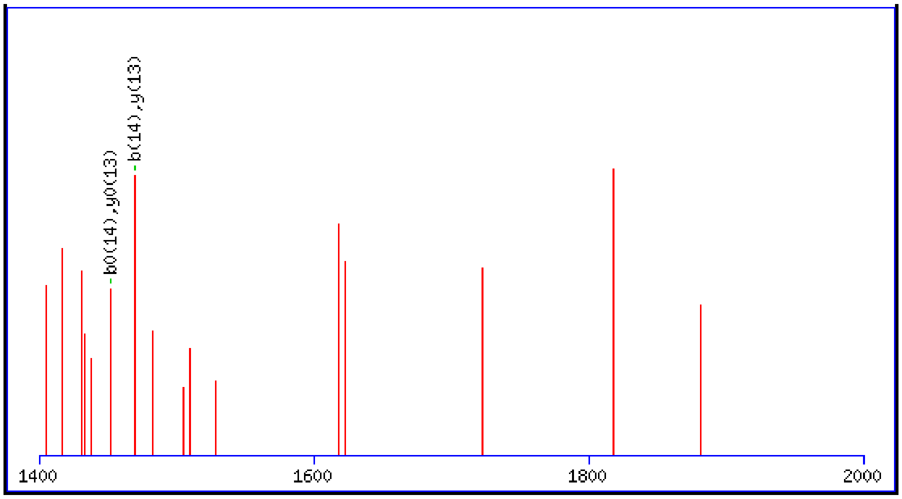

Monoisotopic mass of neutral peptide Mr(calc): 2366.2192
Fixed modifications: Carbamidomethyl (C)
Ions Score: 33 **Expect:** 0.071
Matches (Bold Red): 48/192 fragment ions using 98 most intense peaks

#	b	b^{++}	b*	b^{*++}	b^0	b^{0++}	Seq.	y	y^{++}	y*	y^{*++}	y^0	y^{0++}	#
1	114.0913	57.5493					I							21
2	227.1754	114.0913					L	2254.1424	1127.5748	2237.1159	1119.0616	2236.1318	1118.5696	20
3	**340.2595**	170.6334					L	2141.0583	**1071.0328**	2124.0318	1062.5195	2123.0478	**1062.0275**	19
4	453.3435	227.1754					L	2027.9743	1014.4908	2010.9477	1005.9775	2009.9637	1005.4855	18
5	613.3742	307.1907					C	1914.8902	**957.9487**	1897.8637	949.4355	1896.8796	948.9435	17
6	712.4426	**356.7249**					V	1754.8596	877.9334	1737.8330	869.4201	1736.8490	868.9281	16
7	769.4641	385.2357					G	1655.7911	**828.3992**	1638.7646	819.8859	1637.7806	819.3939	15
8	**898.5067**	**449.7570**			**880.4961**	440.7517	E	1598.7697	799.8885	1581.7431	791.3752	1580.7591	790.8832	14
9	969.5438	485.2755			951.5332	476.2702	A	**1469.7271**	**735.3672**	1452.7005	726.8539	**1451.7165**	726.3619	13
10	**1026.5652**	**513.7863**			1008.5547	**504.7810**	G	1398.6900	699.8486	1381.6634	691.3354	1380.6794	690.8433	12
11	1141.5922	571.2997			1123.5816	562.2944	D	**1341.6685**	671.3379	**1324.6420**	**662.8246**	**1323.6579**	**662.3326**	11
12	1242.6399	**621.8236**			**1224.6293**	612.8183	T	1226.6416	613.8244	1209.6150	605.3111	1208.6310	604.8191	10
13	**1341.7083**	671.3578			**1323.6977**	**662.3525**	V	1125.5939	563.3006	1108.5673	554.7873	1107.5833	554.2953	9
14	**1469.7668**	**735.3871**	1452.7403	726.8738	**1451.7563**	726.3818	Q	**1026.5255**	**513.7664**	1009.4989	**505.2531**	1008.5149	**504.7611**	8
15	1616.8353	**808.9213**	1599.8087	800.4080	1598.8247	799.9160	F	**898.4669**	**449.7371**	881.4403	441.2238	**880.4563**	440.7318	7
16	1687.8724	**844.4398**	1670.8458	835.9265	1669.8618	835.4345	A	**751.3985**	376.2029	734.3719	367.6896	733.3879	367.1976	6
17	1816.9150	**908.9611**	1799.8884	900.4478	1798.9044	**899.9558**	E	680.3614	**340.6843**	663.3348	332.1710	**662.3508**	331.6790	5
18	1979.9783	**990.4928**	1962.9517	981.9795	1961.9677	981.4875	Y	**551.3188**	276.1630	534.2922	267.6498			4
19	2093.0624	**1047.0348**	2076.0358	1038.5215	2075.0518	1038.0295	I	**388.2554**	194.6314	371.2289	186.1181			3
20	2221.1209	**1111.0641**	2204.0944	1102.5508	2203.1104	1102.0588	Q	**275.1714**	138.0893	258.1448	129.5761			2
21							K	147.1128	74.0600	130.0863	65.5468			1

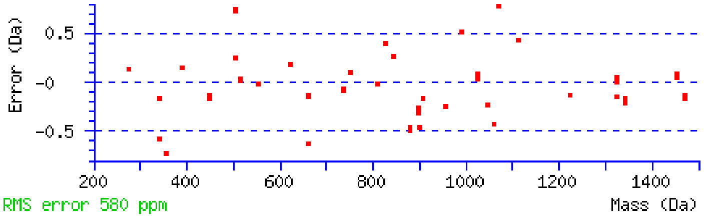

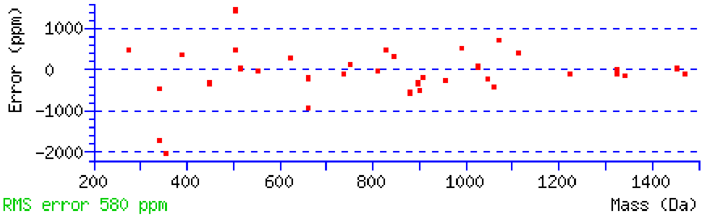

All matches to this query

Score	Mr(calc):	Delta	Sequence
32.5	2366.2192	1.0199	ILLLCVGEAGDTVQFAEYIQK

8.7	2367.2199	0.0192	KRDLQTSTHVETLLTFSALK
8.1	2367.2199	0.0192	KRDLQTSTHVETLLTFSALK
7.3	2366.2233	1.0158	RPAQPHRPSPASSLQRARFK
6.8	2365.2134	2.0257	SMLDTIKKGHITGLTGVMEFR
5.8	2366.2220	1.0171	RLSVEESGLGLGLGPGRSPHLR
5.0	2366.2233	1.0158	RPAQPHRPSPASSLQRARFK
4.7	2365.2112	2.0279	ETLITIMKTLIDFVKMMVK
4.7	2365.2112	2.0279	ETLITIMKTLIDFVKMMVK
4.3	2365.2112	2.0279	ETLITIMKTLIDFVKMMVK

Spectrum No: 112; Query: 47990; Rank: 1

Peptide View

MS/MS Fragmentation of **MEYLIGIQGPDYVLVASDR**
Found in **IPI00028006**, Tax_Id=9606 Gene_Symbol=PSMB2 Proteasome subunit beta type-2

Match to Query 47990: 2218.044942 from(740.355590,3+)
Title: 091224LimSK_Exosome3_06.8914.8914.3.dta
Data file C:\Temp\Mascoti\LSK_2D\091224LimSK_Exosome3_01_2D.mgf

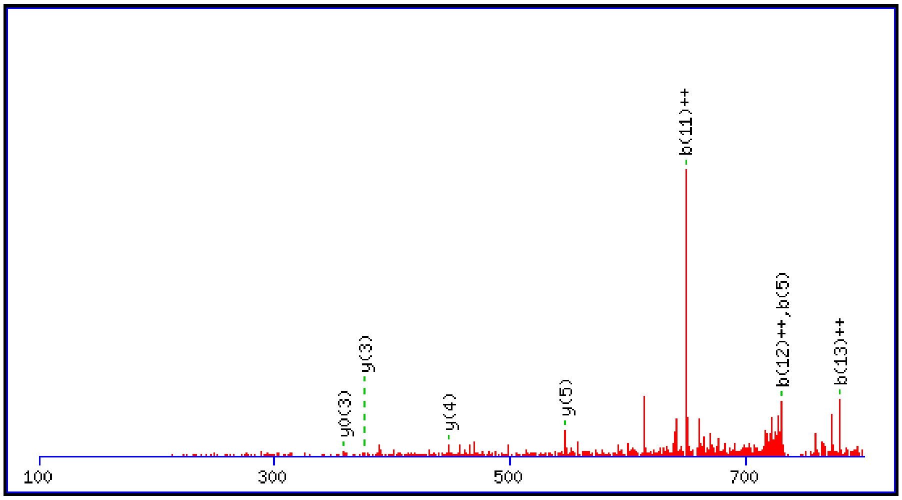

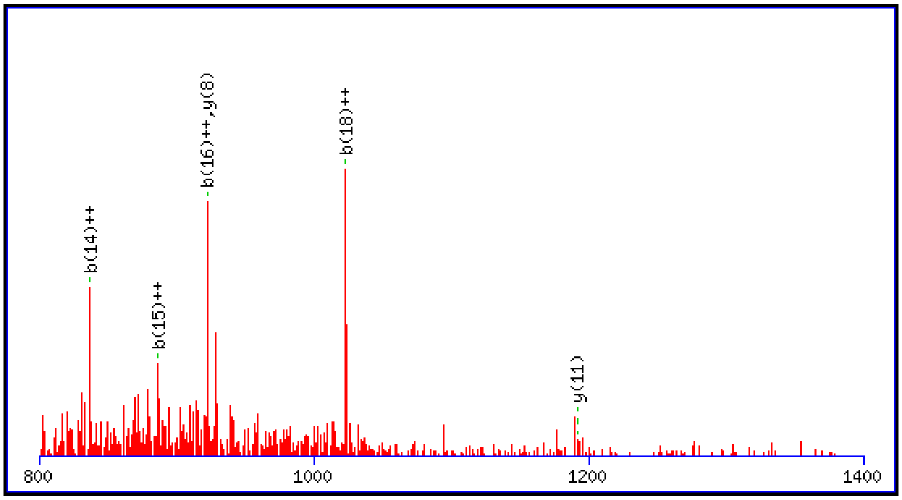

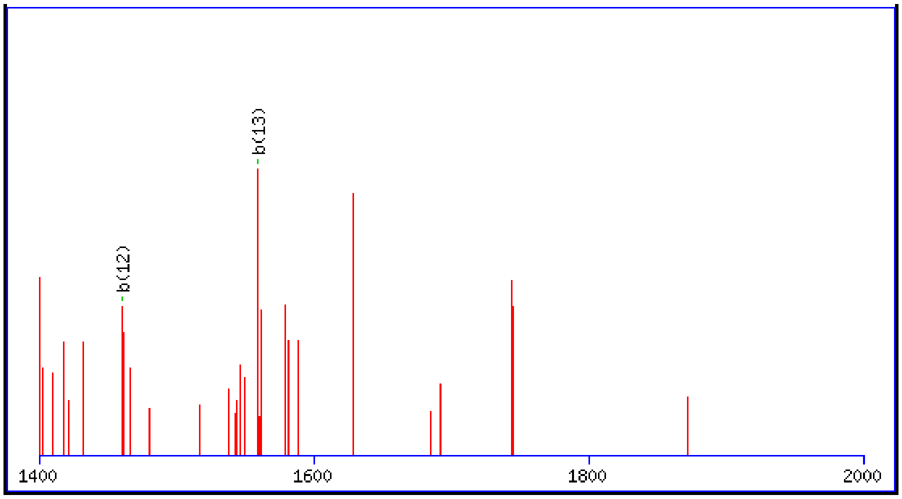

Monoisotopic mass of neutral peptide Mr(calc): 2218.0381
Fixed modifications: Carbamidomethyl (C)
Variable modifications:
Y3 : Phospho (Y)
Ions Score: 22 **Expect:** 1.8
Matches (Bold Red): 16/198 fragment ions using 43 most intense peaks

#	b	b^{++}	b*	b^{*++}	b^{0}	b^{0++}	Seq.	y	y^{++}	y*	y^{*++}	y^{0}	y^{0++}	#
1	132.0478	66.5275					M							19
2	261.0904	131.0488			243.0798	122.0435	E	2088.0049	1044.5061	2070.9784	1035.9928	2069.9943	1035.5008	18
3	504.1200	252.5636			486.1094	243.5584	Y	1958.9623	979.9848	1941.9358	971.4715	1940.9518	970.9795	17
4	617.2041	309.1057			599.1935	300.1004	L	1715.9327	858.4700	1698.9061	849.9567	1697.9221	849.4647	16
5	**730.2881**	365.6477			712.2776	356.6424	I	1602.8486	801.9279	1585.8221	793.4147	1584.8380	792.9227	15
6	787.3096	394.1584			769.2990	385.1532	G	1489.7645	745.3859	1472.7380	736.8726	1471.7540	736.3806	14
7	900.3937	450.7005			882.3831	441.6952	I	1432.7431	716.8752	1415.7165	708.3619	1414.7325	707.8699	13
8	1028.4522	514.7298	1011.4257	506.2165	1010.4417	505.7245	Q	1319.6590	660.3331	1302.6325	651.8199	1301.6484	651.3279	12
9	1085.4737	543.2405	1068.4472	534.7272	1067.4631	534.2352	G	**1191.6004**	596.3039	1174.5739	587.7906	1173.5899	587.2986	11
10	1182.5265	591.7669	1165.4999	583.2536	1164.5159	582.7616	P	1134.5790	567.7931	1117.5524	559.2798	1116.5684	558.7878	10
11	1297.5534	**649.2803**	1280.5269	640.7671	1279.5428	640.2751	D	1037.5262	519.2667	1020.4997	510.7535	1019.5156	510.2615	9
12	**1460.6167**	**730.8120**	1443.5902	722.2987	1442.6062	721.8067	Y	**922.4993**	461.7533	905.4727	453.2400	904.4887	452.7480	8
13	**1559.6852**	**780.3462**	1542.6586	771.8329	1541.6746	771.3409	V	759.4359	380.2216	742.4094	371.7083	741.4254	371.2163	7
14	1672.7692	**836.8882**	1655.7427	828.3750	1654.7587	827.8830	L	660.3675	330.6874	643.3410	322.1741	642.3570	321.6821	6
15	1771.8376	**886.4225**	1754.8111	877.9092	1753.8271	877.4172	V	**547.2835**	274.1454	530.2569	265.6321	529.2729	265.1401	5
16	1842.8747	**921.9410**	1825.8482	913.4277	1824.8642	912.9357	A	**448.2150**	224.6112	431.1885	216.0979	430.2045	215.6059	4
17	1929.9068	965.4570	1912.8802	956.9438	1911.8962	956.4517	S	**377.1779**	189.0926	360.1514	180.5793	**359.1674**	180.0873	3
18	2044.9337	**1022.9705**	2027.9072	1014.4572	2026.9232	1013.9652	D	290.1459	145.5766	273.1193	137.0633	272.1353	136.5713	2
19							R	175.1190	88.0631	158.0924	79.5498			1

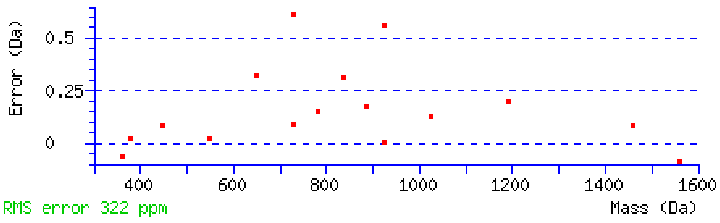

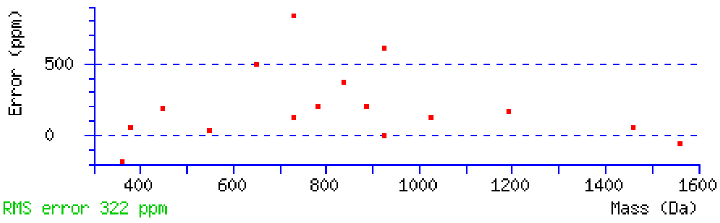

All matches to this query

Score	Mr(calc):	Delta	Sequence
22.0	2218.0381	0.0068	MEYLIGIQGPDYVLVASDR
14.1	2218.0381	0.0068	MEYLIGIQGPDYVLVASDR

8.6	2217.0596	0.9854	ISSGDPKLPVLYQVERTR
8.6	2217.0596	0.9854	ISSGDPKLPVLYQVERTR
6.0	2218.0589	-0.0140	NTDMSPYPQRPAQGLVWSR
5.7	2216.0335	2.0115	EEHRLSATQQNSRGPAVEK
5.3	2216.0409	2.0040	DHCKVSKSAHSLQPSISQK
4.7	2218.0341	0.0109	DIMESSPGSSPPNLTPKPLR
4.5	2216.0409	2.0040	DHCKVSKSAHSLQPSISQK
4.4	2218.0314	0.0135	MGRLAGRGADPPTSLPDGAAGR

Spectrum No: 113; Query: 18907; Rank: 1

Peptide View

MS/MS Fragmentation of **NGIHDLDNISFPK**
Found in **IPI00028006**, Tax_Id=9606 Gene_Symbol=PSMB2 Proteasome subunit beta type-2

Match to Query 18907: 1468.741988 from(735.378270,2+)
Title: 091224LimSK_Exosome3_06.4581.4581.2.dta
Data file C:\Temp\Mascoti\LSK_2D\091224LimSK_Exosome3_01_2D.mgf

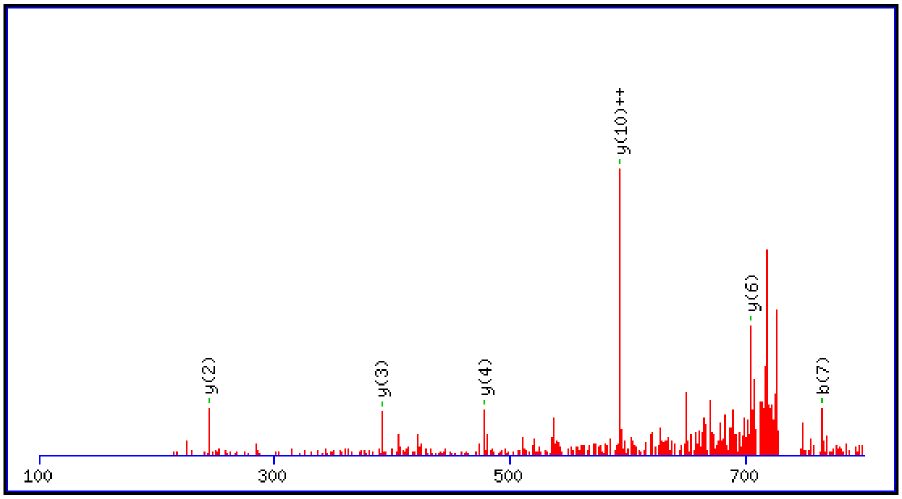

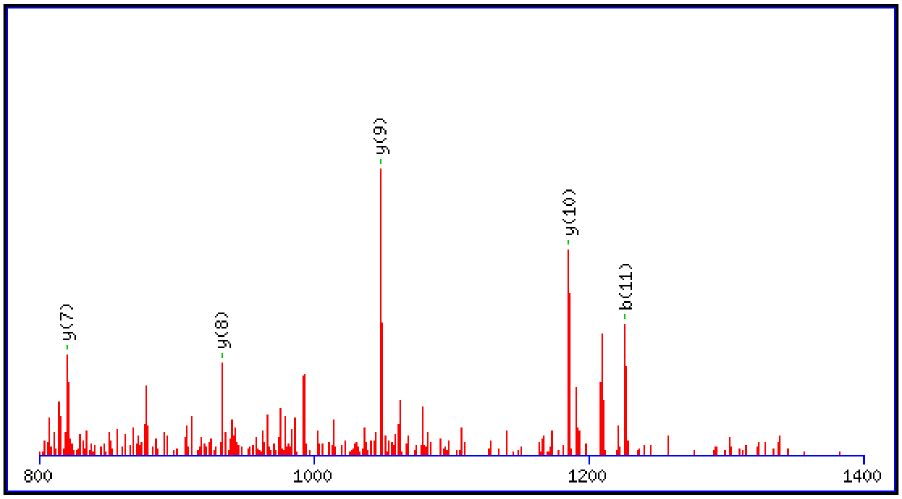

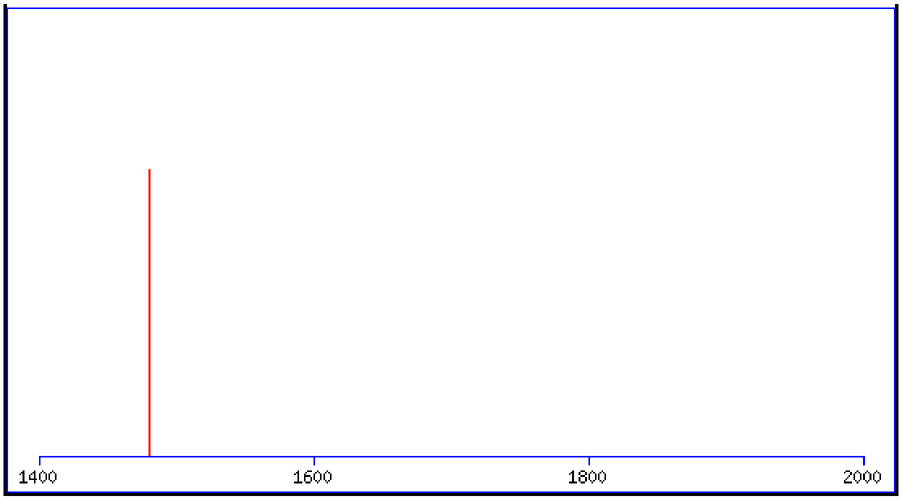

Monoisotopic mass of neutral peptide Mr(calc): 1468.7310
Fixed modifications: Carbamidomethyl (C)
Ions Score: 61 **Expect:** 0.00013
Matches (Bold Red): 11/130 fragment ions using 21 most intense peaks

#	b	b^{++}	b*	b^{*++}	b^{0}	b^{0++}	Seq.	y	y^{++}	y*	y^{*++}	y^{0}	y^{0++}	#
1	115.0502	58.0287	98.0237	49.5155			N							13
2	172.0717	86.5395	155.0451	78.0262			G	1355.6954	678.3513	1338.6688	669.8381	1337.6848	669.3461	12
3	285.1557	143.0815	268.1292	134.5682			I	1298.6739	649.8406	1281.6474	641.3273	1280.6634	640.8353	11
4	422.2146	211.6110	405.1881	203.0977			H	**1185.5899**	**593.2986**	1168.5633	584.7853	1167.5793	584.2933	10
5	537.2416	269.1244	520.2150	260.6112	519.2310	260.1191	D	**1048.5310**	524.7691	1031.5044	516.2558	1030.5204	515.7638	9
6	650.3257	325.6665	633.2991	317.1532	632.3151	316.6612	L	**933.5040**	467.2556	916.4775	458.7424	915.4934	458.2504	8
7	**765.3526**	383.1799	748.3260	374.6667	747.3420	374.1747	D	**820.4199**	410.7136	803.3934	402.2003	802.4094	401.7083	7
8	879.3955	440.2014	862.3690	431.6881	861.3850	431.1961	N	**705.3930**	353.2001	688.3665	344.6869	687.3824	344.1949	6
9	992.4796	496.7434	975.4530	488.2302	974.4690	487.7381	I	591.3501	296.1787	574.3235	287.6654	573.3395	287.1734	5
10	1079.5116	540.2594	1062.4851	531.7462	1061.5010	531.2542	S	**478.2660**	239.6366	461.2395	231.1234	460.2554	230.6314	4
11	**1226.5800**	613.7937	1209.5535	605.2804	1208.5695	604.7884	F	**391.2340**	196.1206	374.2074	187.6074			3
12	1323.6328	662.3200	1306.6062	653.8068	1305.6222	653.3148	P	**244.1656**	122.5864	227.1390	114.0731			2
13							K	147.1128	74.0600	130.0863	65.5468			1

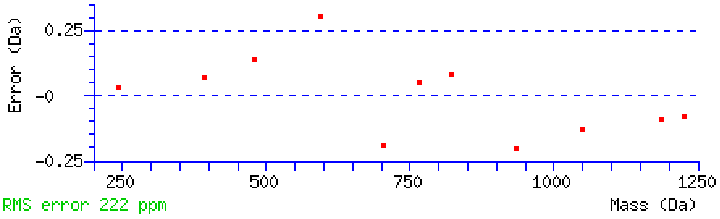

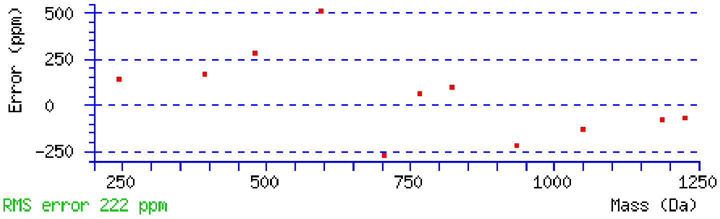

All matches to this query

Score	Mr(calc):	Delta	Sequence
61.4	1468.7310	0.0110	NGIHDLDNISFPK
10.0	1466.7365	2.0055	KLNHQEVVEEDK
9.9	1467.7470	0.9950	RSFSIIGDFQNGK
7.6	1468.7286	0.0134	QDSVKSILERSK
7.1	1466.7287	2.0133	KIMGGSGTETTLEK
7.1	1468.7439	-0.0019	LLGKNGTGGLFQGK
5.3	1468.7300	0.0120	RQHLAVSHEKGK
4.4	1468.7455	-0.0035	KKSLLLPAPSQK
3.7	1468.7286	0.0134	QDSVKSILERSK

3.1	1467.7392	1.0028	VPSTPVHLTNEMK

Spectrum No: 114; Query: 26112; Rank: 1

Peptide View

MS/MS Fragmentation of **NGYELSPTAAANFTR**
Found in **IPI00028006**, Tax_Id=9606 Gene_Symbol=PSMB2 Proteasome subunit beta type-2

Match to Query 26112: 1610.779388 from(806.396970,2+)
Title: 091224LimSK_Exosome3_06.3957.3957.2.dta
Data file C:\Temp\Mascoti\LSK_2D\091224LimSK_Exosome3_01_2D.mgf

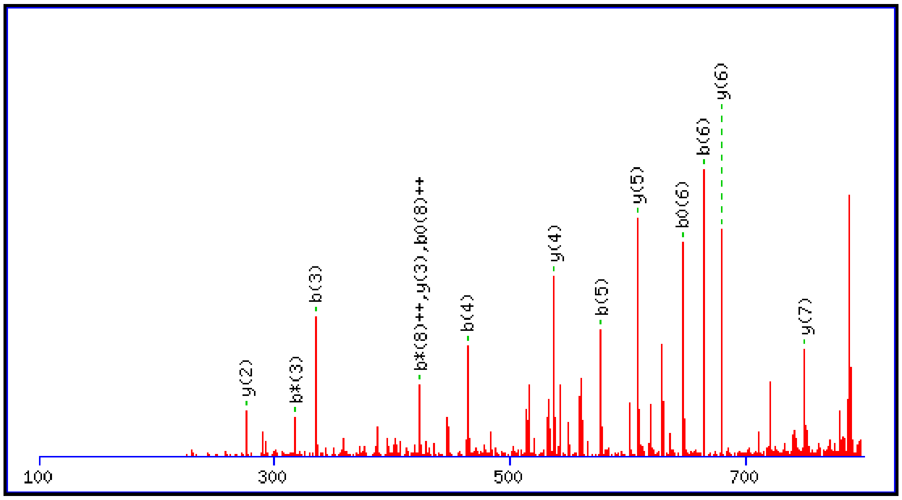

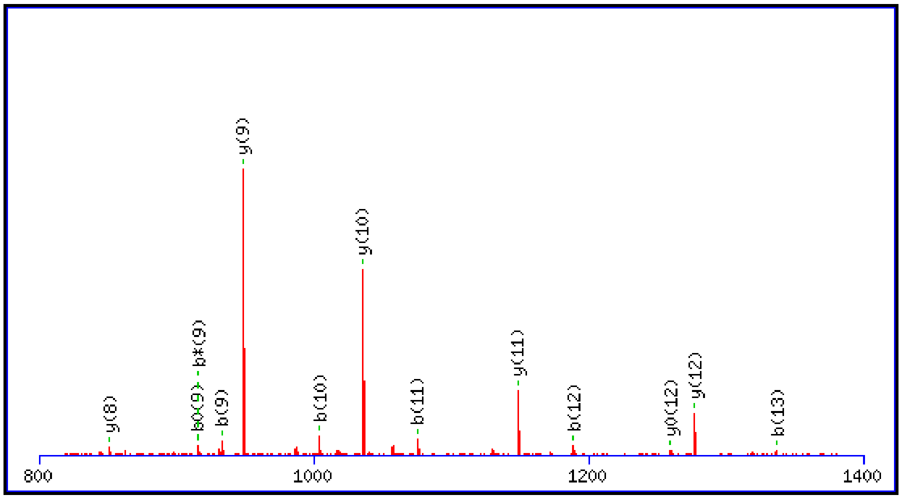

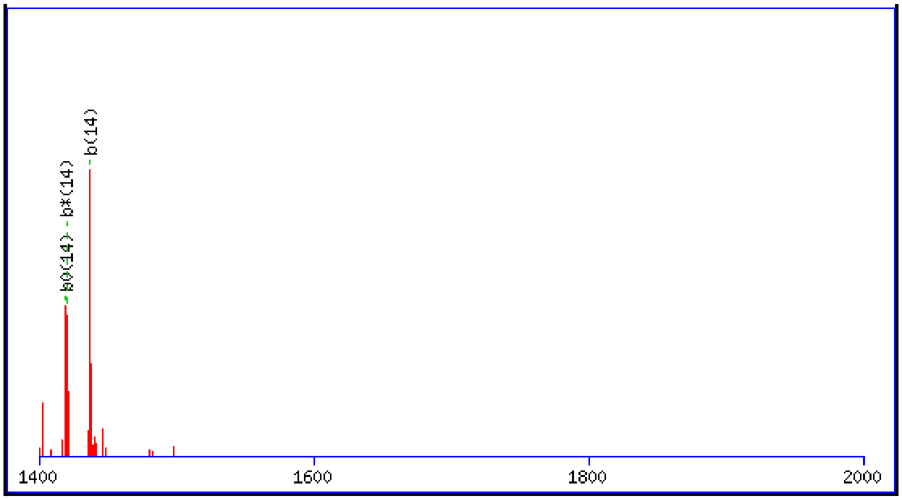

Monoisotopic mass of neutral peptide Mr(calc): 1610.7689
Fixed modifications: Carbamidomethyl (C)
Ions Score: 90 **Expect:** 1.9e-007
Matches (Bold Red): 30/160 fragment ions using 41 most intense peaks

#	b	b^{++}	b*	b^{*++}	b^0	b^{0++}	Seq.	y	y^{++}	y*	y^{*++}	y^0	y^{0++}	#
1	115.0502	58.0287	98.0237	49.5155			N							15
2	172.0717	86.5395	155.0451	78.0262			G	1497.7332	749.3703	1480.7067	740.8570	1479.7227	740.3650	14
3	**335.1350**	168.0711	**318.1084**	159.5579			Y	1440.7118	720.8595	1423.6852	712.3462	1422.7012	711.8542	13
4	**464.1776**	232.5924	447.1510	224.0792	446.1670	223.5872	E	**1277.6484**	639.3279	1260.6219	630.8146	**1259.6379**	630.3226	12
5	**577.2617**	289.1345	560.2351	280.6212	559.2511	280.1292	L	**1148.6058**	574.8066	1131.5793	566.2933	1130.5953	565.8013	11
6	**664.2937**	332.6505	647.2671	324.1372	**646.2831**	323.6452	S	**1035.5218**	518.2645	1018.4952	509.7513	1017.5112	509.2592	10
7	761.3464	381.1769	744.3199	372.6636	743.3359	372.1716	P	**948.4898**	474.7485	931.4632	466.2352	930.4792	465.7432	9
8	862.3941	431.7007	845.3676	**423.1874**	844.3836	**422.6954**	T	**851.4370**	426.2221	834.4104	417.7089	833.4264	417.2169	8
9	**933.4312**	467.2193	**916.4047**	458.7060	**915.4207**	458.2140	A	**750.3893**	375.6983	733.3628	367.1850	732.3787	366.6930	7
10	**1004.4684**	502.7378	987.4418	494.2245	986.4578	493.7325	A	**679.3522**	340.1797	662.3257	331.6665	661.3416	331.1745	6
11	**1075.5055**	538.2564	1058.4789	529.7431	1057.4949	529.2511	A	**608.3151**	304.6612	591.2885	296.1479	590.3045	295.6559	5
12	**1189.5484**	595.2778	1172.5218	586.7646	1171.5378	586.2726	N	**537.2780**	269.1426	520.2514	260.6293	519.2674	260.1373	4
13	**1336.6168**	668.8120	1319.5903	660.2988	1318.6062	659.8068	F	**423.2350**	212.1212	406.2085	203.6079	405.2245	203.1159	3
14	**1437.6645**	719.3359	**1420.6379**	710.8226	**1419.6539**	710.3306	T	**276.1666**	138.5870	259.1401	130.0737	258.1561	129.5817	2
15							R	175.1190	88.0631	158.0924	79.5498			1

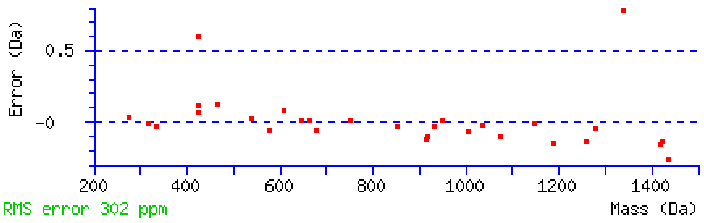

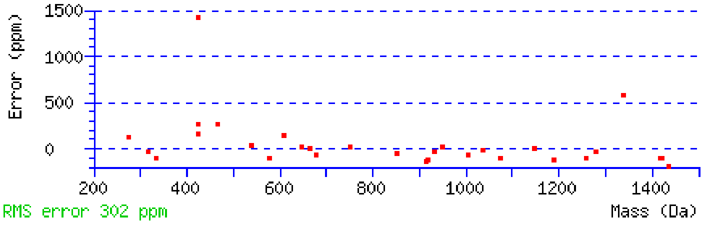

All matches to this query

Score	Mr(calc):	Delta	Sequence
90.3	1610.7689	0.0105	NGYELSPTAAANFTR
5.6	1610.7943	-0.0149	RAWHGARLPGSPAR
5.0	1610.7900	-0.0106	SSGSEESILDLRYR
4.5	1610.7818	-0.0024	TFSEKATLTIHQR
3.6	1609.7709	1.0085	ANQNVNAVHSHYTR
3.3	1609.7882	0.9911	GFKIMSAGDGAGDRTK
1.9	1610.7851	-0.0057	LRKGGATALMDAAEK

1.3	1608.7872	1.9922	EKRVTVLEQNGEK
0.9	1610.7657	0.0137	EFGKNREVMGLCR
0.7	1609.7899	0.9895	DPAPVKTMTISSKR

Spectrum No: 115; Query: 3033; Rank: 1

Peptide View

MS/MS Fragmentation of **RNLADCLR**
Found in **IPI00028006**, Tax_Id=9606 Gene_Symbol=PSMB2 Proteasome subunit beta type-2

Match to Query 3033: 1016.522928 from(509.268740,2+)
Title: 091224LimSK_Exosome3_06.650.650.2.dta
Data file C:\Temp\Mascoti\LSK_2D\091224LimSK_Exosome3_01_2D.mgf

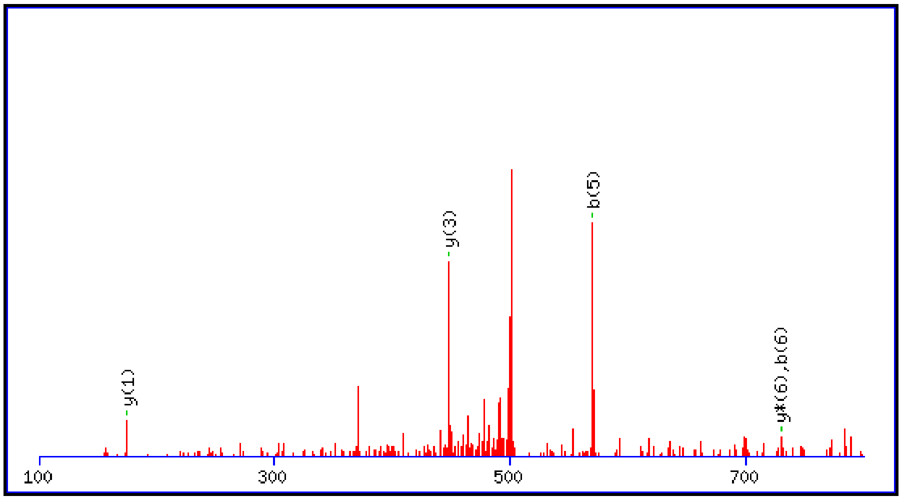

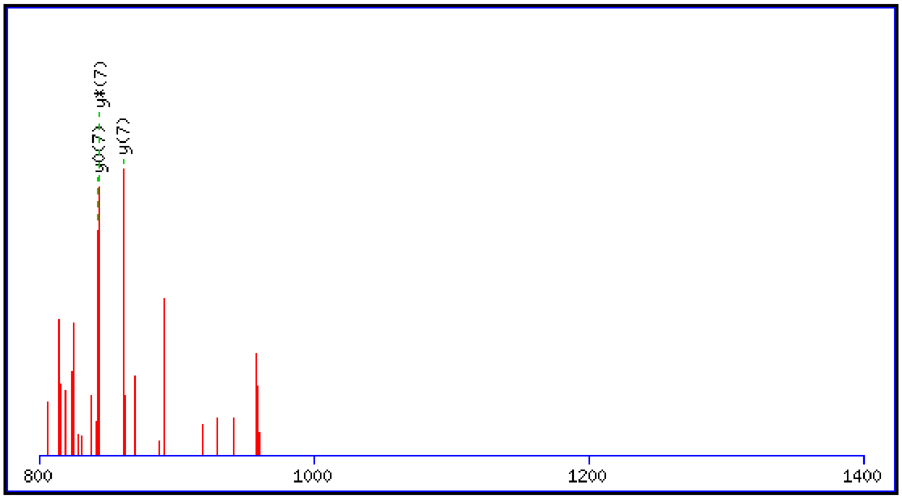

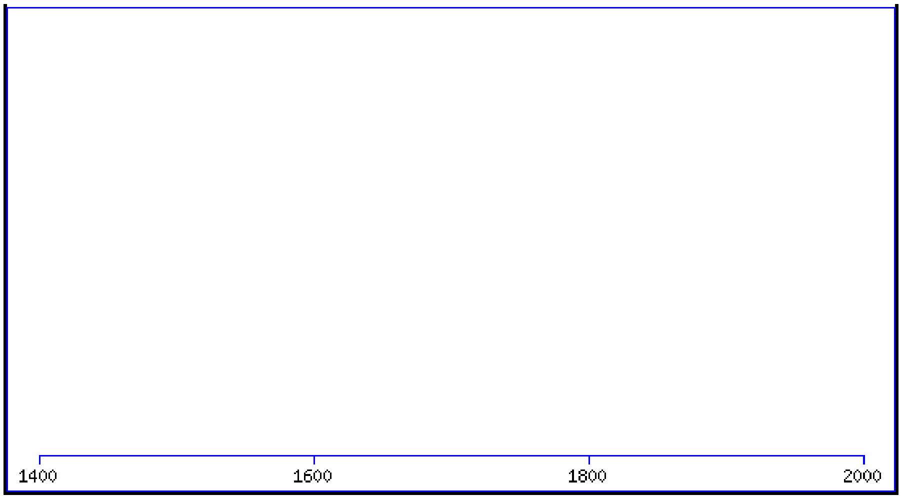

Monoisotopic mass of neutral peptide Mr(calc): 1016.5185
Fixed modifications: Carbamidomethyl (C)
Ions Score: 20 **Expect:** 1.2
Matches (Bold Red): 9/70 fragment ions using 18 most intense peaks

#	b	b^{++}	b*	b^{*++}	b^0	b^{0++}	Seq.	y	y^{++}	y*	y^{*++}	y^0	y^{0++}	#
1	157.1084	79.0578	140.0818	70.5446			R							8
2	271.1513	136.0793	254.1248	127.5660			N	**861.4247**	431.2160	**844.3982**	422.7027	**843.4141**	422.2107	7
3	384.2354	192.6213	367.2088	184.1081			L	747.3818	374.1945	**730.3552**	365.6813	729.3712	365.1892	6
4	455.2725	228.1399	438.2459	219.6266			A	634.2977	317.6525	617.2712	309.1392	616.2872	308.6472	5
5	**570.2994**	285.6534	553.2729	277.1401	552.2889	276.6481	D	563.2606	282.1339	546.2341	273.6207	545.2500	273.1287	4
6	**730.3301**	365.6687	713.3035	357.1554	712.3195	356.6634	C	**448.2337**	224.6205	431.2071	216.1072			3
7	**843.4141**	422.2107	826.3876	413.6974	825.4036	413.2054	L	288.2030	144.6051	271.1765	136.0919			2
8							R	**175.1190**	88.0631	158.0924	79.5498			1

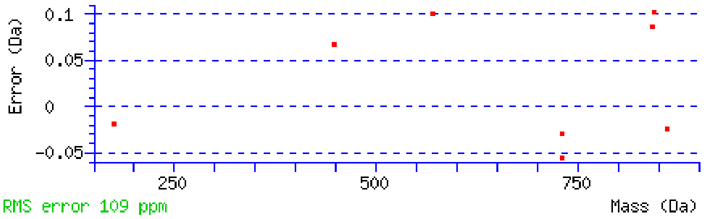

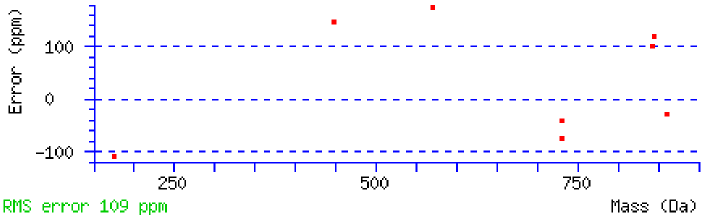

All matches to this query

Score	Mr(calc):	Delta	Sequence
20.1	1016.5185	0.0044	RNLADCLR
12.7	1016.5147	0.0082	MMPGAPLLR
11.2	1016.5297	-0.0068	RQMAARER
8.7	1015.5120	1.0109	GMKESISHK
8.2	1015.5271	0.9958	GRQSQRER
7.9	1016.5325	-0.0095	GIMESPIVR
7.2	1016.5168	0.0061	HVAAASLIR
6.5	1016.5138	0.0091	STSLEPVER
6.3	1016.5251	-0.0021	EDQAGKTLR
5.9	1016.5251	-0.0021	RDLDLETR

Spectrum No: 116; Query: 3868; Rank: 1

Peptide View

MS/MS Fragmentation of **VAASNIVQMK**
Found in **IPI00028006**, Tax_Id=9606 Gene_Symbol=PSMB2 Proteasome subunit beta type-2

Match to Query 3868: 1059.579548 from(530.797050,2+)
Title: 091224LimSK_Exosome3_07.1303.1303.2.dta
Data file C:\Temp\Mascoti\LSK_2D\091224LimSK_Exosome3_01_2D.mgf

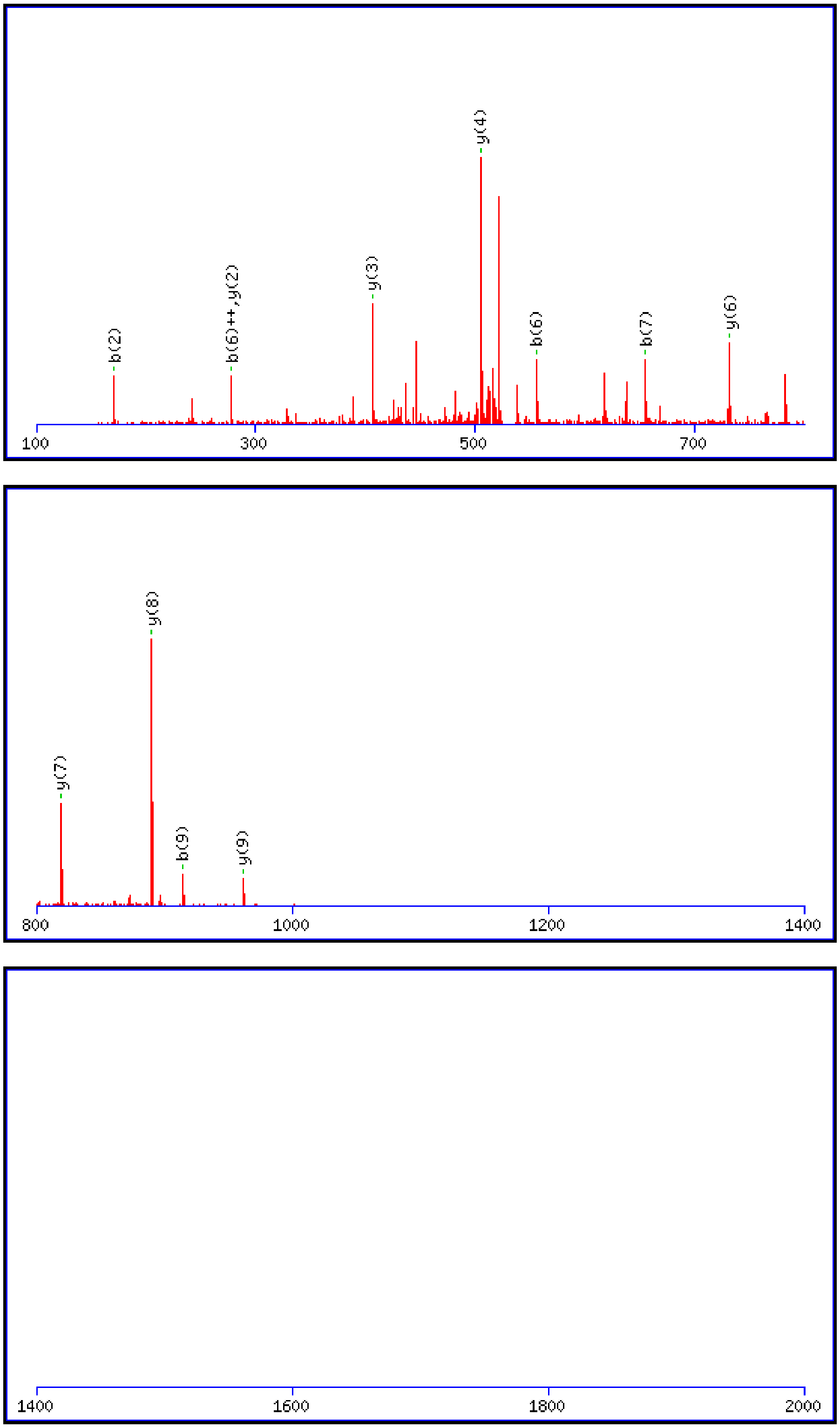

Monoisotopic mass of neutral peptide Mr(calc): 1059.5746
Fixed modifications: Carbamidomethyl (C)
Ions Score: 76 **Expect:** 3.4e-006
Matches (Bold Red): 12/82 fragment ions using 13 most intense peaks

		++		++	0	0++			++		++	0	0++	

#	b	b	b*	b*	b	b	Seq.	y	y	y*	y*	y	y	#
**1**	100.0757	50.5415					**V**							**10**
**2**	**171.1128**	86.0600					**A**	**961.5135**	481.2604	944.4870	472.7471	943.5030	472.2551	**9**
**3**	242.1499	121.5786					**A**	**890.4764**	445.7418	873.4499	437.2286	872.4658	436.7366	**8**
**4**	329.1819	165.0946			311.1714	156.0893	**S**	**819.4393**	410.2233	802.4128	401.7100	801.4287	401.2180	**7**
**5**	443.2249	222.1161	426.1983	213.6028	425.2143	213.1108	**N**	**732.4073**	366.7073	715.3807	358.1940			**6**
**6**	**556.3089**	**278.6581**	539.2824	270.1448	538.2984	269.6528	**I**	618.3643	309.6858	601.3378	301.1725			**5**
**7**	**655.3774**	328.1923	638.3508	319.6790	637.3668	319.1870	**V**	**505.2803**	253.1438	488.2537	244.6305			**4**
**8**	783.4359	392.2216	766.4094	383.7083	765.4254	383.2163	**Q**	**406.2119**	203.6096	389.1853	195.0963			**3**
**9**	**914.4764**	457.7418	897.4499	449.2286	896.4658	448.7366	**M**	**278.1533**	139.5803	261.1267	131.0670			**2**
**10**							**K**	147.1128	74.0600	130.0863	65.5468			**1**

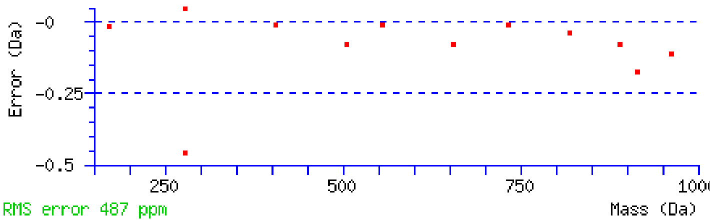

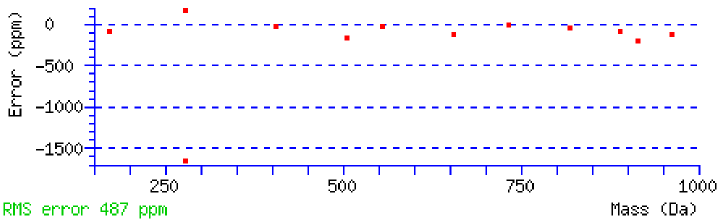

All matches to this query

Score	Mr(calc):	Delta	Sequence
76.0	1059.5746	0.0049	VAASNIVQMK
2.4	1058.5720	1.0076	DAAEREKLK
1.5	1057.5628	2.0167	XSLSGARSAPR
1.3	1059.5785	0.0011	IQSSEGRRK
0.8	1059.5825	-0.0030	VTWRETIR
0.7	1057.5768	2.0028	QQIIATQEK

Spectrum No: 117; Query: 28688; Rank: 1

Peptide View

MS/MS Fragmentation of **VAASNIVQMKDDHDK**
Found in **IPI00028006**, Tax_Id=9606 Gene_Symbol=PSMB2 Proteasome subunit beta type-2

Match to Query 28688: 1669.815816 from(418.461230,4+)
Title: 091224LimSK_Exosome3_06.615.615.4.dta
Data file C:\Temp\Mascoti\LSK_2D\091224LimSK_Exosome3_01_2D.mgf

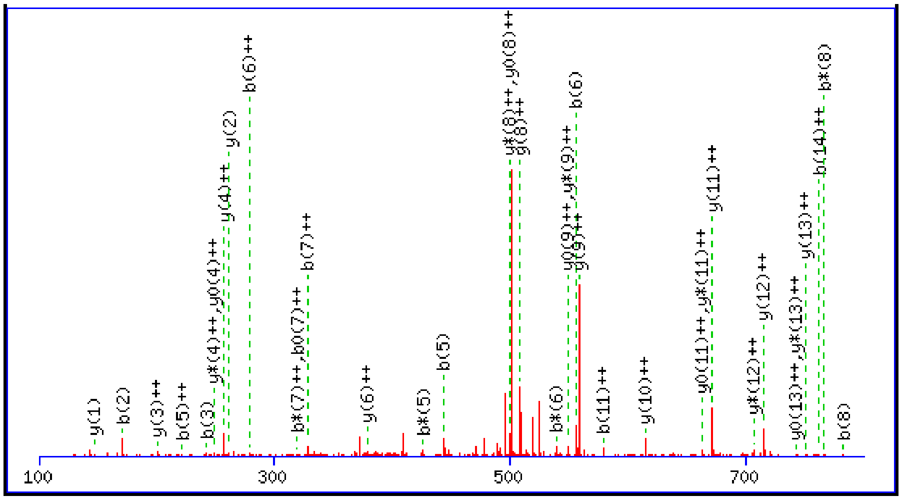

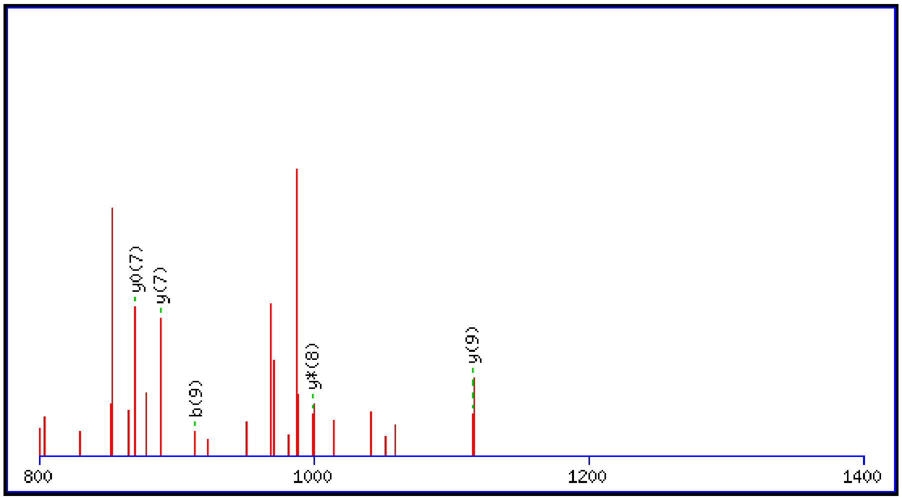

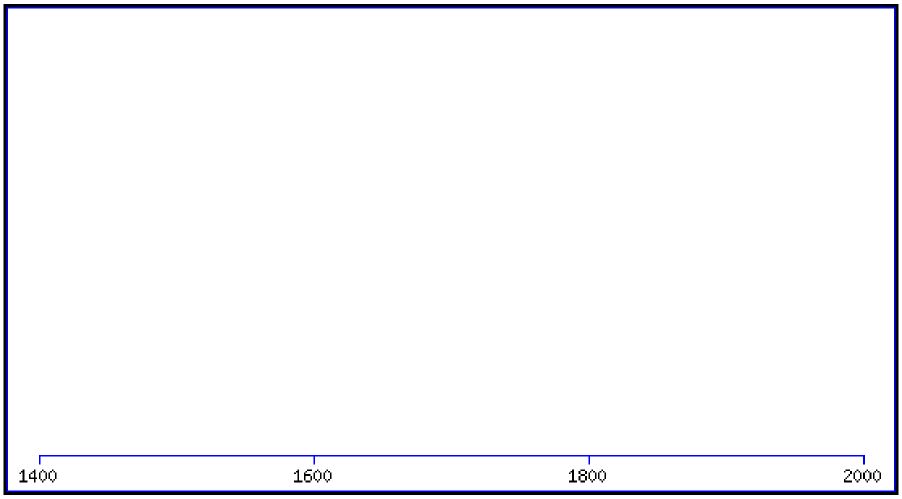

Monoisotopic mass of neutral peptide Mr(calc): 1669.8094
Fixed modifications: Carbamidomethyl (C)
Ions Score: 36 **Expect:** 0.057
Matches (Bold Red): 42/152 fragment ions using 88 most intense peaks

#	b	b^{++}	b*	b^{*++}	b^0	b^{0++}	Seq.	y	y^{++}	y*	y^{*++}	y^0	y^{0++}	#
1	100.0757	50.5415					V							15
2	**171.1128**	86.0600					A	1571.7482	786.3778	1554.7217	777.8645	1553.7377	777.3725	14
3	**242.1499**	121.5786					A	1500.7111	**750.8592**	1483.6846	**742.3459**	1482.7006	**741.8539**	13
4	329.1819	165.0946			311.1714	156.0893	S	1429.6740	**715.3406**	1412.6475	**706.8274**	1411.6634	706.3354	12
5	**443.2249**	**222.1161**	**426.1983**	213.6028	425.2143	213.1108	N	1342.6420	**671.8246**	1325.6154	**663.3114**	1324.6314	**662.8193**	11
6	**556.3089**	**278.6581**	**539.2824**	270.1448	538.2984	269.6528	I	1228.5990	**614.8032**	1211.5725	606.2899	1210.5885	605.7979	10

7	655.3774	328.1923	638.3508	319.6790	637.3668	319.1870	V	1115.5150	558.2611	1098.4884	549.7479	1097.5044	549.2558	9
8	783.4359	392.2216	766.4094	383.7083	765.4254	383.2163	Q	1016.4466	508.7269	999.4200	500.2136	998.4360	499.7216	8
9	914.4764	457.7418	897.4499	449.2286	896.4658	448.7366	M	888.3880	444.6976	871.3614	436.1844	870.3774	435.6924	7
10	1042.5714	521.7893	1025.5448	513.2761	1024.5608	512.7840	K	757.3475	379.1774	740.3210	370.6641	739.3369	370.1721	6
11	1157.5983	579.3028	1140.5718	570.7895	1139.5878	570.2975	D	629.2525	315.1299	612.2260	306.6166	611.2420	306.1246	5
12	1272.6253	636.8163	1255.5987	628.3030	1254.6147	627.8110	D	514.2256	257.6164	497.1991	249.1032	496.2150	248.6112	4
13	1409.6842	705.3457	1392.6576	696.8325	1391.6736	696.3404	H	399.1987	200.1030	382.1721	191.5897	381.1881	191.0977	3
14	1524.7111	762.8592	1507.6846	754.3459	1506.7006	753.8539	D	262.1397	131.5735	245.1132	123.0602	244.1292	122.5682	2
15							K	147.1128	74.0600	130.0863	65.5468			1

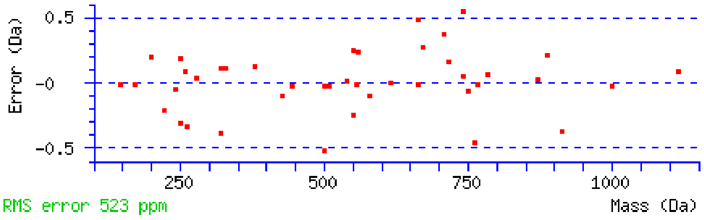

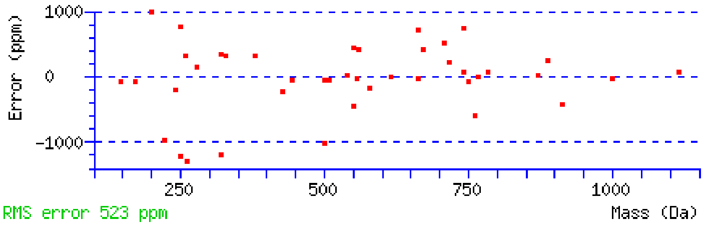

All matches to this query

Score	Mr(calc):	Delta	Sequence
35.8	1669.8094	0.0065	VAASNIVQMKDDHDK
16.6	1668.8018	1.0140	LKMQNGSKGSGLQNK
14.3	1669.8157	0.0001	AARLCLSICSVKGR
14.1	1667.8079	2.0079	ASRCPPPPRGAAVPR
12.2	1668.8084	1.0075	DTLSKGSAAAATAVAAGK
11.9	1669.8172	-0.0014	QYDPPAESQVPRAGR
11.5	1667.8172	1.9987	GLGSLFSGSLLPTDPK
11.4	1667.8162	1.9996	GRAGADGARGMPGDPGVK
11.1	1667.8079	2.0079	ARARGSPCPPLPPGR
9.3	1667.8049	2.0109	VRSVILQGSGVSPLP

Spectrum No: 118; Query: 2751; Rank: 1

Peptide View

MS/MS Fragmentation of **YYTPTISR**
Found in **IPI00028006**, Tax_Id=9606 Gene_Symbol=PSMB2 Proteasome subunit beta type-2

Match to Query 2751: 999.507288 from(500.760920,2+)
Title: 091224LimSK_Exosome3_06.1370.1370.2.dta
Data file C:\Temp\Mascoti\LSK_2D\091224LimSK_Exosome3_01_2D.mgf

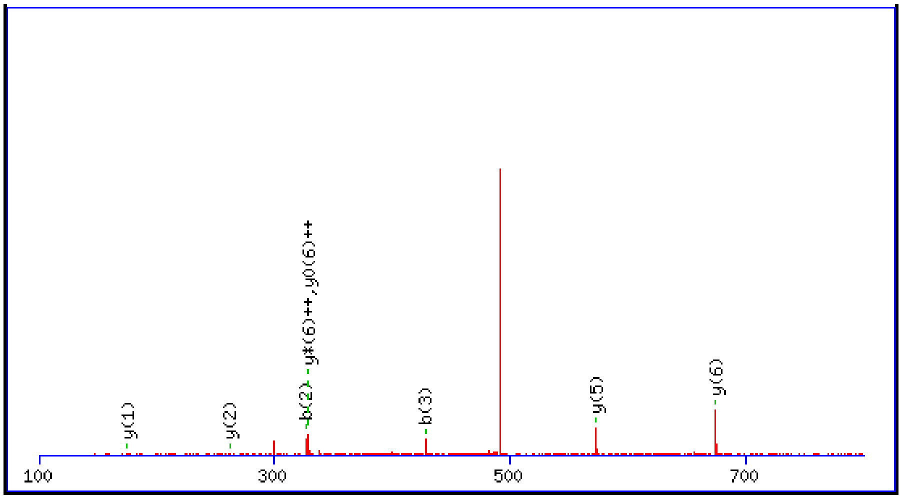

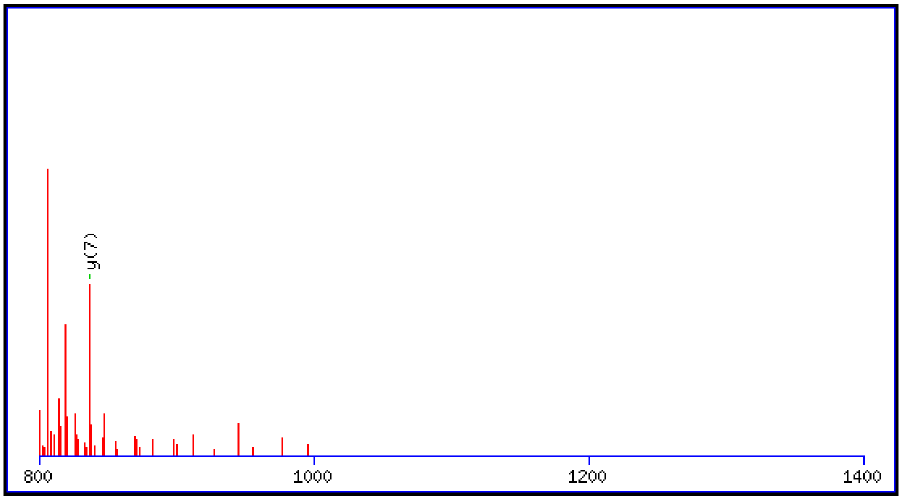

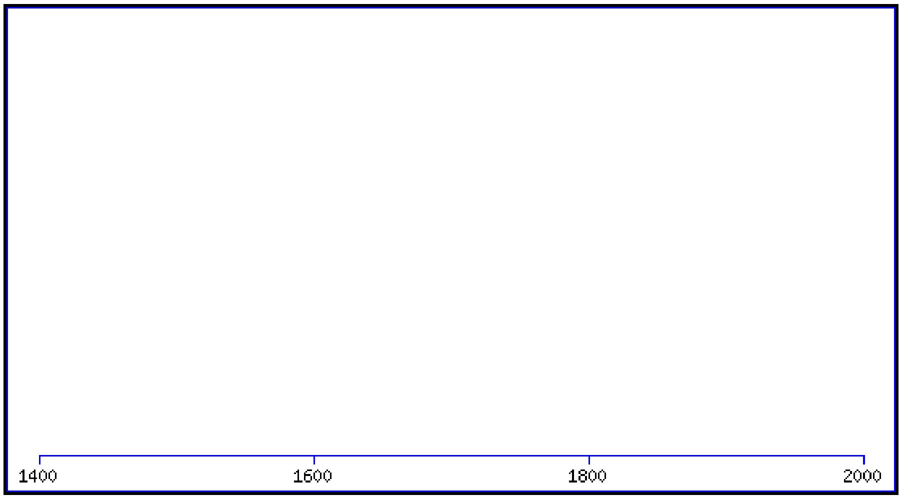

Monoisotopic mass of neutral peptide Mr(calc): 999.5025
Fixed modifications: Carbamidomethyl (C)
Ions Score: 21 **Expect:** 0.84
Matches (Bold Red): 9/64 fragment ions using 23 most intense peaks

#	b	b^{++}	b^{0}	b^{0++}	Seq.	y	y^{++}	y*	y^{*++}	y^{0}	y^{0++}	#
1	164.0706	82.5389			Y							8
2	**327.1339**	164.0706			Y	**837.4465**	419.2269	820.4199	410.7136	819.4359	410.2216	7
3	**428.1816**	214.5944	410.1710	205.5892	T	**674.3832**	337.6952	657.3566	**329.1819**	656.3726	**328.6899**	6
4	525.2344	263.1208	507.2238	254.1155	P	**573.3355**	287.1714	556.3089	278.6581	555.3249	278.1661	5
5	626.2821	313.6447	608.2715	304.6394	T	476.2827	238.6450	459.2562	230.1317	458.2722	229.6397	4
6	739.3661	370.1867	721.3556	361.1814	I	375.2350	188.1212	358.2085	179.6079	357.2245	179.1159	3

**7**	826.3981	413.7027	808.3876	404.6974	**S**	**262.1510**	131.5791	245.1244	123.0659	244.1404	122.5738	**2**
**8**					**R**	**175.1190**	88.0631	158.0924	79.5498			**1**

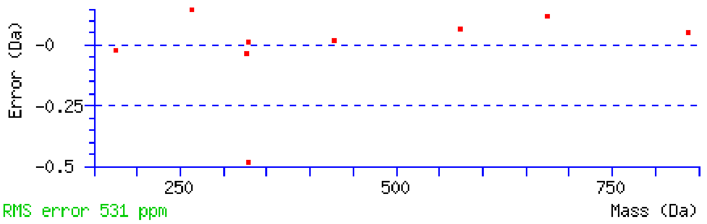

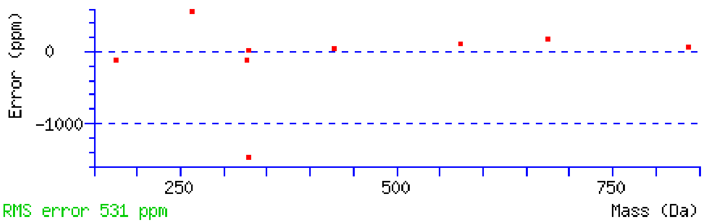

All matches to this query

Score	Mr(calc):	Delta	Sequence
21.1	999.5025	0.0048	YYTPTISR
15.1	998.4950	1.0122	GAVVTGFLR
12.3	997.4957	2.0116	SLQKLSSR
11.5	997.4957	2.0116	KALSGLSSR
9.9	998.4950	1.0123	KPVLGSYR
9.9	998.4950	1.0123	KPVLGSYR
9.9	998.4968	1.0105	KPVQDCPR
9.4	999.5025	0.0048	YYSLGEIR
8.4	997.4941	2.0132	SPREEAGPR
7.1	997.4957	2.0116	KALSGLSSR

Spectrum No: 119; Query: 3022; Rank: 1

Peptide View

MS/MS Fragmentation of **DGIVLGADTR**
Found in **IPI00003217**, Tax_Id=9606 Gene_Symbol=PSMB7 Proteasome subunit beta type-7

Match to Query 3022: 1015.535168 from(508.774860,2+)
Title: 091224LimSK_Exosome3_06.1359.1359.2.dta
Data file C:\Temp\Mascoti\LSK_2D\091224LimSK_Exosome3_01_2D.mgf

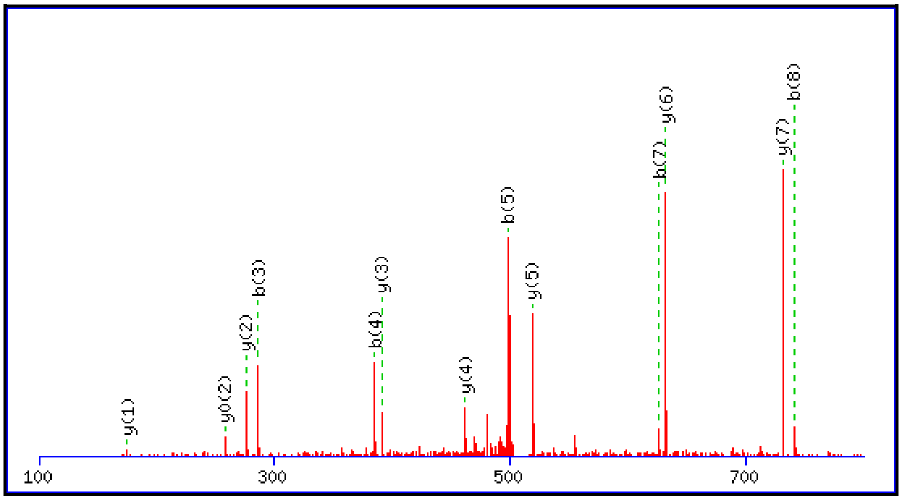

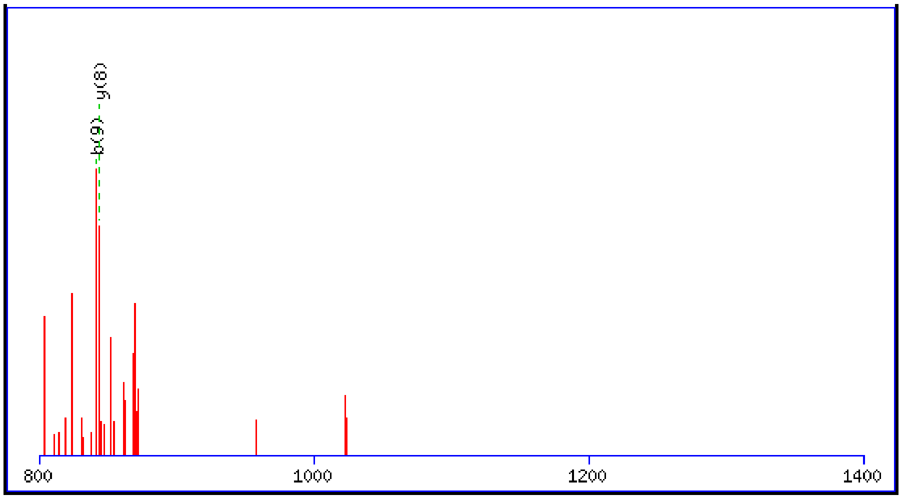

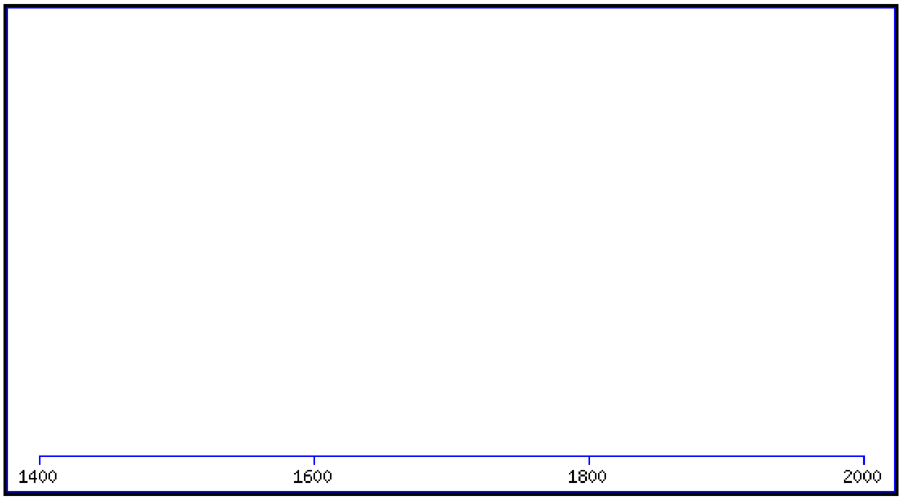

Monoisotopic mass of neutral peptide Mr(calc): 1015.5298
Fixed modifications: Carbamidomethyl (C)
Ions Score: 69 **Expect:** 1.4e-005
Matches (Bold Red): 15/88 fragment ions using 26 most intense peaks

#	b	b^{++}	b^0	b^{0++}	Seq.	y	y^{++}	y*	y^{*++}	y^0	y^{0++}	#
1	116.0342	58.5207	98.0237	49.5155	D							10
2	173.0557	87.0315	155.0451	78.0262	G	901.5102	451.2587	884.4836	442.7454	883.4996	442.2534	9
3	**286.1397**	143.5735	268.1292	134.5682	I	**844.4887**	422.7480	827.4621	414.2347	826.4781	413.7427	8
4	**385.2082**	193.1077	367.1976	184.1024	V	**731.4046**	366.2060	714.3781	357.6927	713.3941	357.2007	7
5	**498.2922**	249.6498	480.2817	240.6445	L	**632.3362**	316.6717	615.3097	308.1585	614.3257	307.6665	6
6	555.3137	278.1605	537.3031	269.1552	G	**519.2522**	260.1297	502.2256	251.6164	501.2416	251.1244	5
7	**626.3508**	313.6790	608.3402	304.6738	A	**462.2307**	231.6190	445.2041	223.1057	444.2201	222.6137	4
8	**741.3777**	371.1925	723.3672	362.1872	D	**391.1936**	196.1004	374.1670	187.5872	373.1830	187.0951	3
9	**842.4254**	421.7164	824.4149	412.7111	T	**276.1666**	138.5870	259.1401	130.0737	**258.1561**	129.5817	2
10					R	**175.1190**	88.0631	158.0924	79.5498			1

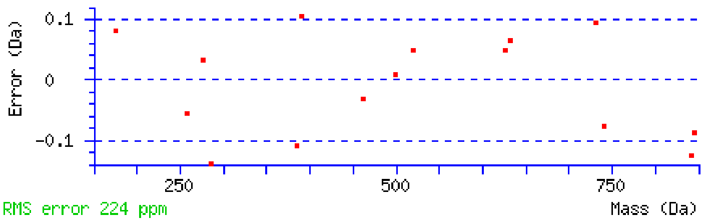

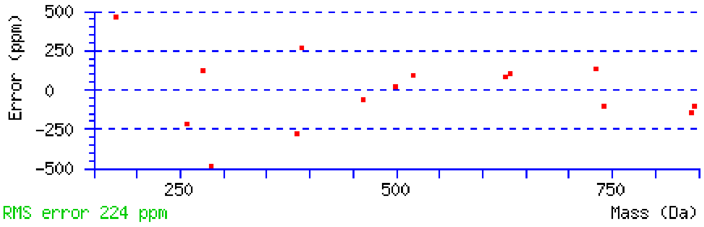

All matches to this query

Score	Mr(calc):	Delta	Sequence
69.2	1015.5298	0.0053	DGIVLGADTR
20.8	1014.5319	1.0033	AGRVGGQSGAR
18.3	1015.5298	0.0054	LATVVNDER
16.8	1015.5410	-0.0059	GQQALGSSLR
12.2	1014.5263	1.0088	GIIFTKTR
11.8	1015.5410	-0.0058	RLNLSEER
10.7	1015.5298	0.0054	LRDEINEK
9.3	1014.5263	1.0088	GIIFTKTR
8.9	1013.5310	2.0041	ILRLYEK
7.2	1013.5310	2.0041	IRLLYEK

Spectrum No: 120; Query: 14207; Rank: 1

Peptide View

MS/MS Fragmentation of **FRPDMEEEEAK**
Found in **IPI00003217**, Tax_Id=9606 Gene_Symbol=PSMB7 Proteasome subunit beta type-7

Match to Query 14207: 1379.609568 from(690.812060,2+)
Title: 091224LimSK_Exosome3_06.573.573.2.dta
Data file C:\Temp\Mascoti\LSK_2D\091224LimSK_Exosome3_01_2D.mgf

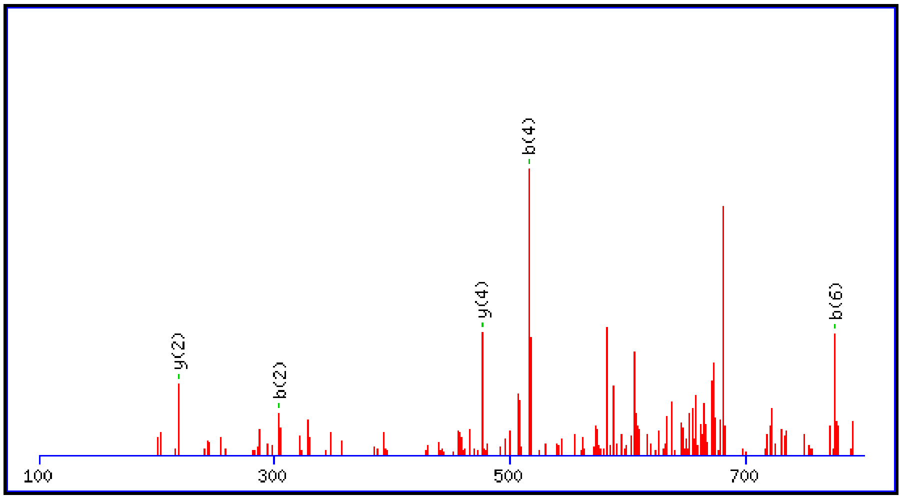

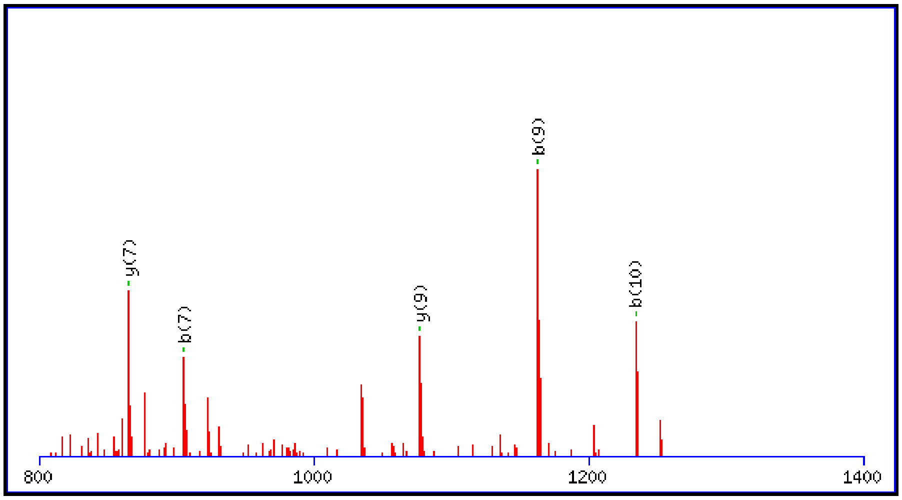

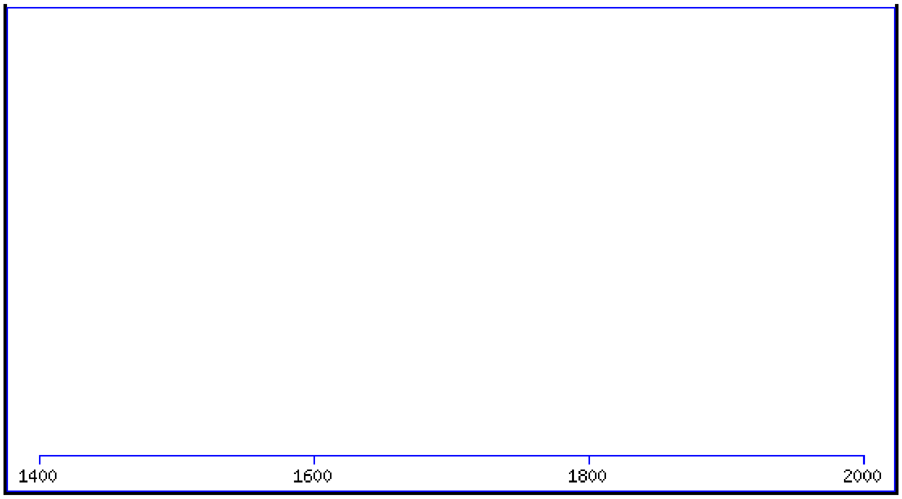

Monoisotopic mass of neutral peptide Mr(calc): 1379.6027
Fixed modifications: Carbamidomethyl (C)
Ions Score: 57 **Expect:** 0.00033
Matches (Bold Red): 10/108 fragment ions using 12 most intense peaks

#	b	b^{++}	b*	b*$^{++}$	b^{0}	b^{0++}	Seq.	y	y^{++}	y*	y*$^{++}$	y^{0}	y^{0++}	#
1	148.0757	74.5415					F							11
2	**304.1768**	152.5920	287.1503	144.0788			R	1233.5416	617.2744	1216.5150	608.7612	1215.5310	608.2692	10
3	401.2296	201.1184	384.2030	192.6051			P	**1077.4405**	539.2239	1060.4139	530.7106	1059.4299	530.2186	9
4	**516.2565**	258.6319	499.2300	250.1186	498.2459	249.6266	D	980.3877	490.6975	963.3612	482.1842	962.3772	481.6922	8
5	647.2970	324.1521	630.2704	315.6389	629.2864	315.1469	M	**865.3608**	433.1840	848.3342	424.6708	847.3502	424.1787	7
6	**776.3396**	388.6734	759.3130	380.1602	758.3290	379.6681	E	734.3203	367.6638	717.2937	359.1505	716.3097	358.6585	6
7	**905.3822**	453.1947	888.3556	444.6815	887.3716	444.1894	E	605.2777	303.1425	588.2511	294.6292	587.2671	294.1372	5
8	1034.4248	517.7160	1017.3982	509.2027	1016.4142	508.7107	E	**476.2351**	238.6212	459.2086	230.1079	458.2245	229.6159	4
9	**1163.4674**	582.2373	1146.4408	573.7240	1145.4568	573.2320	E	347.1925	174.0999	330.1660	165.5866	329.1819	165.0946	3
10	**1234.5045**	617.7559	1217.4779	609.2426	1216.4939	608.7506	A	**218.1499**	109.5786	201.1234	101.0653			2
11							K	147.1128	74.0600	130.0863	65.5468			1

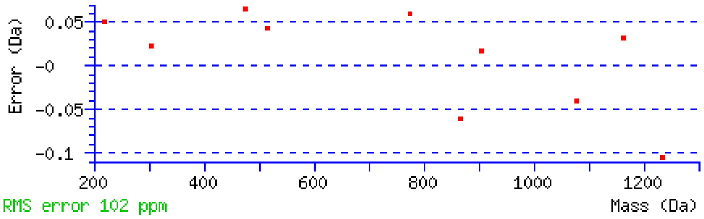

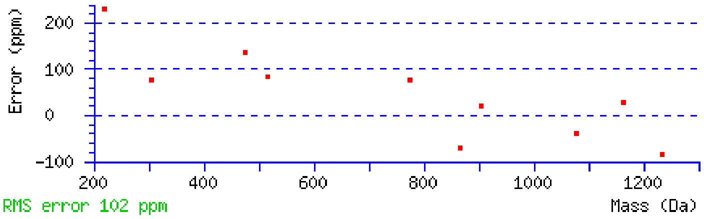

All matches to this query

Score	Mr(calc):	Delta	Sequence
57.2	1379.6027	0.0069	FRPDMEEEEAK
19.9	1379.6213	-0.0118	CWEKIMPDSAK
9.6	1379.6061	0.0035	DGVMGMNSIEPAK
9.6	1379.5985	0.0111	MYTWEKPFAK
9.6	1379.5985	0.0111	MYTWEKPFAK
7.1	1379.6156	-0.0060	MTPGLSPSLPER
3.5	1379.5970	0.0126	EQEEAVPTIER
2.9	1379.6172	-0.0077	ITTEKMLLQK
2.8	1379.6194	-0.0098	RPSASSPNNTAAK
2.5	1379.6082	0.0014	AKSSSSRFTDSK

Mascot Search Results: Peptide View

Spectrum No: 121; Query: 74371; Rank: 1

Peptide View

MS/MS Fragmentation of **IHFISPNIYCCGAGTAADTDMTTQLISSNLELHSLSTGR**
Found in **IPI00003217**, Tax_Id=9606 Gene_Symbol=PSMB7 Proteasome subunit beta type-7

Match to Query 74371: 4252.036296 from(1064.016350,4+)
Title: 091224LimSK_Exosome3_06.7965.7965.4.dta
Data file C:\Temp\Mascoti\LSK_2D\091224LimSK_Exosome3_01_2D.mgf

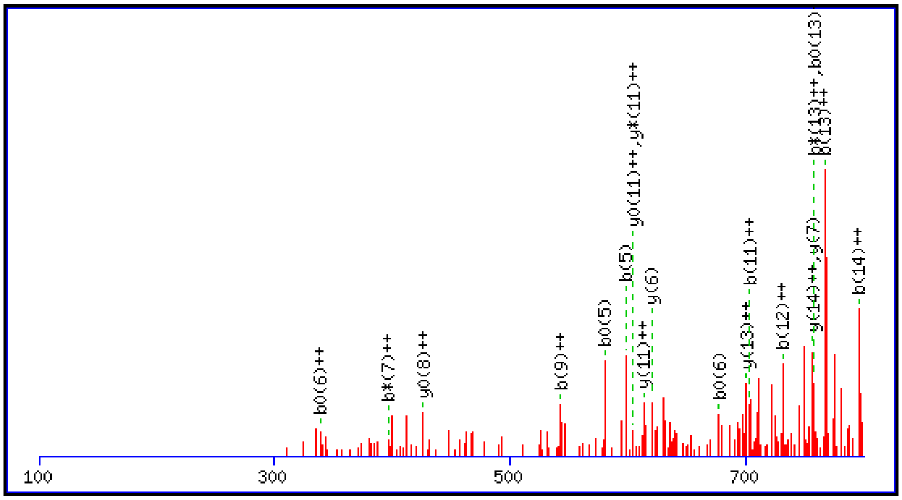

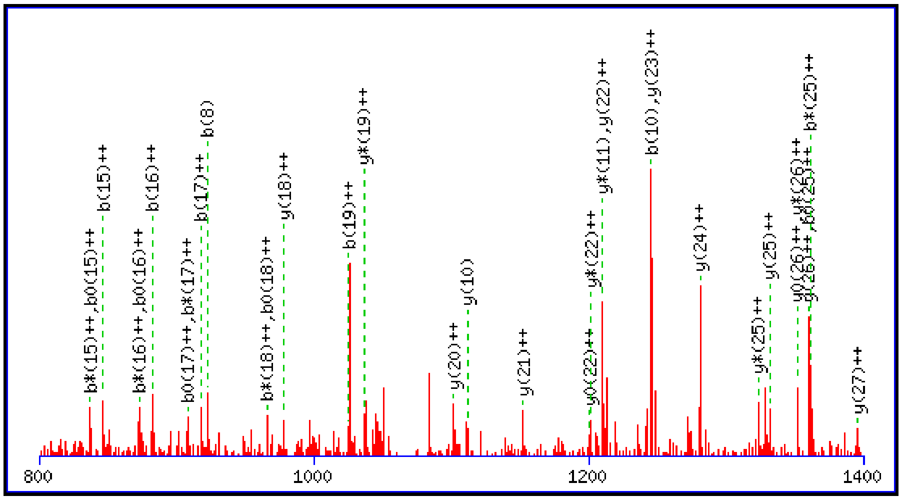

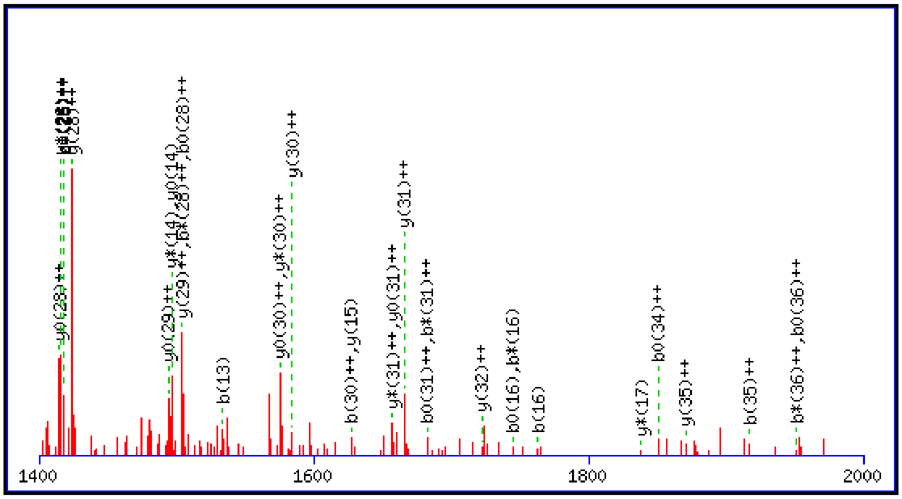

Monoisotopic mass of neutral peptide Mr(calc): 4251.0035

Fixed modifications: Carbamidomethyl (C)
Ions Score: 48 **Expect:** 0.0038
Matches (Bold Red): 85/432 fragment ions using 151 most intense peaks

#	b	b^{++}	b*	b^{*++}	b^{0}	b^{0++}	Seq.	y	y^{++}	y*	y^{*++}	y^{0}	y^{0++}	#
1	114.0913	57.5493					I							39
2	251.1503	126.0788					H	4138.9267	2069.9670	4121.9001	2061.4537	4120.9161	2060.9617	38
3	398.2187	199.6130					F	4001.8678	2001.4375	3984.8412	1992.9243	3983.8572	1992.4322	37
4	511.3027	256.1550					I	3854.7994	1927.9033	3837.7728	1919.3900	3836.7888	1918.8980	36
5	**598.3348**	299.6710			**580.3242**	290.6657	S	3741.7153	**1871.3613**	3724.6888	1862.8480	3723.7047	1862.3560	35
6	695.3875	348.1974			**677.3770**	**339.1921**	P	3654.6833	1827.8453	3637.6567	1819.3320	3636.6727	1818.8400	34
7	809.4304	405.2189	792.4039	**396.7056**	791.4199	396.2136	N	3557.6305	1779.3189	3540.6040	1770.8056	3539.6199	1770.3136	33
8	**922.5145**	461.7609	905.4880	453.2476	904.5039	452.7556	I	3443.5876	**1722.2974**	3426.5610	1713.7842	3425.5770	1713.2921	32
9	1085.5778	**543.2926**	1068.5513	534.7793	1067.5673	534.2873	Y	3330.5035	**1665.7554**	3313.4770	**1657.2421**	3312.4930	**1656.7501**	31
10	**1245.6085**	623.3079	1228.5819	614.7946	1227.5979	614.3026	C	3167.4402	**1584.2237**	3150.4136	**1575.7105**	3149.4296	**1575.2185**	30
11	1405.6391	**703.3232**	1388.6126	694.8099	1387.6286	694.3179	C	3007.4095	**1504.2084**	2990.3830	1495.6951	2989.3990	**1495.2031**	29
12	1462.6606	**731.8339**	1445.6341	723.3207	1444.6500	722.8287	G	2847.3789	**1424.1931**	2830.3523	**1415.6798**	2829.3683	**1415.1878**	28
13	**1533.6977**	**767.3525**	1516.6712	**758.8392**	1515.6872	**758.3472**	A	2790.3574	**1395.6824**	2773.3309	1387.1691	2772.3469	1386.6771	27
14	1590.7192	**795.8632**	1573.6926	787.3500	1572.7086	786.8579	G	2719.3203	**1360.1638**	2702.2938	**1351.6505**	2701.3098	**1351.1585**	26
15	1691.7669	**846.3871**	1674.7403	**837.8738**	1673.7563	**837.3818**	T	2662.2989	**1331.6531**	2645.2723	**1323.1398**	2644.2883	1322.6478	25
16	**1762.8040**	**881.9056**	**1745.7774**	**873.3924**	**1744.7934**	**872.9003**	A	2561.2512	**1281.1292**	2544.2246	1272.6159	2543.2406	1272.1239	24
17	1833.8411	**917.4242**	1816.8145	**908.9109**	1815.8305	**908.4189**	A	2490.2141	**1245.6107**	2473.1875	1237.0974	2472.2035	1236.6054	23
18	1948.8680	974.9377	1931.8415	**966.4244**	1930.8575	**965.9324**	D	2419.1769	**1210.0921**	2402.1504	**1201.5788**	2401.1664	**1201.0868**	22
19	2049.9157	**1025.4615**	2032.8892	1016.9482	2031.9051	1016.4562	T	2304.1500	**1152.5786**	2287.1235	1144.0654	2286.1394	1143.5734	21
20	2164.9427	1082.9750	2147.9161	1074.4617	2146.9321	1073.9697	D	2203.1023	**1102.0548**	2186.0758	1093.5415	2185.0918	1093.0495	20
21	2295.9831	1148.4952	2278.9566	1139.9819	2277.9726	1139.4899	M	2088.0754	1044.5413	2071.0488	**1036.0281**	2070.0648	1035.5360	19
22	2397.0308	1199.0190	2380.0043	1190.5058	2379.0203	1190.0138	T	1957.0349	**979.0211**	1940.0083	970.5078	1939.0243	970.0158	18
23	2498.0785	1249.5429	2481.0519	1241.0296	2480.0679	1240.5376	T	1855.9872	928.4972	**1838.9607**	919.9840	1837.9767	919.4920	17
24	2626.1371	1313.5722	2609.1105	1305.0589	2608.1265	1304.5669	Q	1754.9395	877.9734	1737.9130	869.4601	1736.9290	868.9681	16
25	2739.2211	1370.1142	2722.1946	**1361.6009**	2721.2106	**1361.1089**	L	**1626.8810**	813.9441	1609.8544	805.4308	1608.8704	804.9388	15
26	2852.3052	1426.6562	2835.2787	**1418.1430**	2834.2946	**1417.6510**	I	1513.7969	**757.4021**	**1496.7703**	748.8888	**1495.7863**	748.3968	14
27	2939.3372	1470.1723	2922.3107	1461.6590	2921.3267	1461.1670	S	1400.7128	**700.8601**	1383.6863	692.3468	1382.7023	691.8548	13
28	3026.3693	1513.6883	3009.3427	**1505.1750**	3008.3587	**1504.6830**	S	1313.6808	657.3440	1296.6543	648.8308	1295.6702	648.3388	12
29	3140.4122	1570.7097	3123.3856	1562.1965	3122.4016	1561.7044	N	1226.6488	**613.8280**	**1209.6222**	**605.3148**	1208.6382	**604.8227**	11
30	3253.4962	**1627.2518**	3236.4697	1618.7385	3235.4857	1618.2465	L	**1112.6058**	556.8066	1095.5793	548.2933	1094.5953	547.8013	10
31	3382.5388	1691.7731	3365.5123	**1683.2598**	3364.5283	**1682.7678**	E	999.5218	500.2645	982.4952	491.7513	981.5112	491.2592	9
32	3495.6229	1748.3151	3478.5964	1739.8018	3477.6123	1739.3098	L	870.4792	435.7432	853.4526	427.2300	852.4686	**426.7380**	8
33	3632.6818	1816.8445	3615.6553	1808.3313	3614.6713	1807.8393	H	**757.3951**	379.2012	740.3686	370.6879	739.3846	370.1959	7
34	3719.7138	1860.3606	3702.6873	1851.8473	3701.7033	**1851.3553**	S	**620.3362**	310.6717	603.3097	302.1585	602.3257	301.6665	6
35	3832.7979	**1916.9026**	3815.7714	1908.3893	3814.7873	1907.8973	L	533.3042	267.1557	516.2776	258.6425	515.2936	258.1504	5
36	3919.8299	1960.4186	3902.8034	**1951.9053**	3901.8194	**1951.4133**	S	420.2201	210.6137	403.1936	202.1004	402.2096	201.6084	4
37	4020.8776	2010.9424	4003.8511	2002.4292	4002.8671	2001.9372	T	333.1881	167.0977	316.1615	158.5844	315.1775	158.0924	3
38	4077.8991	2039.4532	4060.8725	2030.9399	4059.8885	2030.4479	G	232.1404	116.5738	215.1139	108.0606			2
39							R	175.1190	88.0631	158.0924	79.5498			1

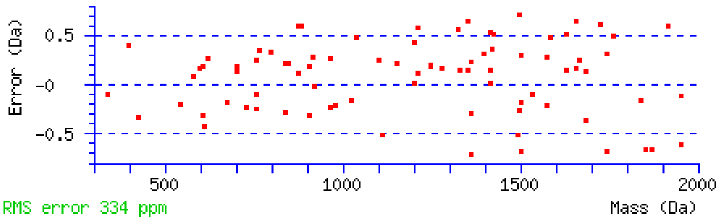

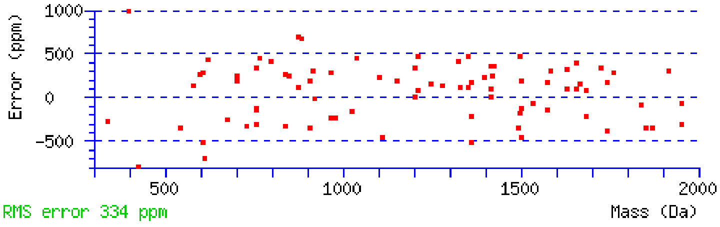

All matches to this query

Score	Mr(calc):	Delta	Sequence
48.5	4251.0035	1.0328	IHFISPNIYCCGAGTAADTDMTTQLISSNLELHSLSTGR
10.7	4252.0055	0.0308	GTIAVGLVPQYYSLDHQPGWLPDSVAYHADDGKLYNGR
9.4	4250.0357	2.0006	GWLSAIGFFQYSPGAAVVMLLPAIMFSVSAAMMAIAIMK
9.4	4250.0357	2.0006	GWLSAIGFFQYSPGAAVVMLLPAIMFSVSAAMMAIAIMK
8.1	4252.0055	0.0308	GTIAVGLVPQYYSLDHQPGWLPDSVAYHADDGKLYNGR
7.5	4250.9975	1.0388	GPSHDGVAEFHMSLTVSCPDPTPSTDPQGRHNREPILGR
7.2	4252.0518	-0.0155	LRELSTLAHCSRELQTQVPDASAHSLAVTLSELHSCR
7.2	4252.0518	-0.0155	LRELSTLAHCSRELQTQVPDASAHSLAVTLSELHSCR
7.2	4252.0007	0.0356	QITNFQLSIENLDQSLAHYGRPKIDGELKITSVER
7.1	4251.0128	1.0235	MPHPQLFSGESVVSWSDLNIIISVPWYLGLMFRTR

Spectrum No: 122; Query: 45016; Rank: 1

Peptide View

MS/MS Fragmentation of **ITPLEIEVLEETVQTMDTS**
Found in **IPI00003217**, Tax_Id=9606 Gene_Symbol=PSMB7 Proteasome subunit beta type-7

Match to Query 45016: 2147.069128 from(1074.541840,2+)
Title: 091224LimSK_Exosome3_07.9078.9078.2.dta
Data file C:\Temp\Mascoti\LSK_2D\091224LimSK_Exosome3_01_2D.mgf

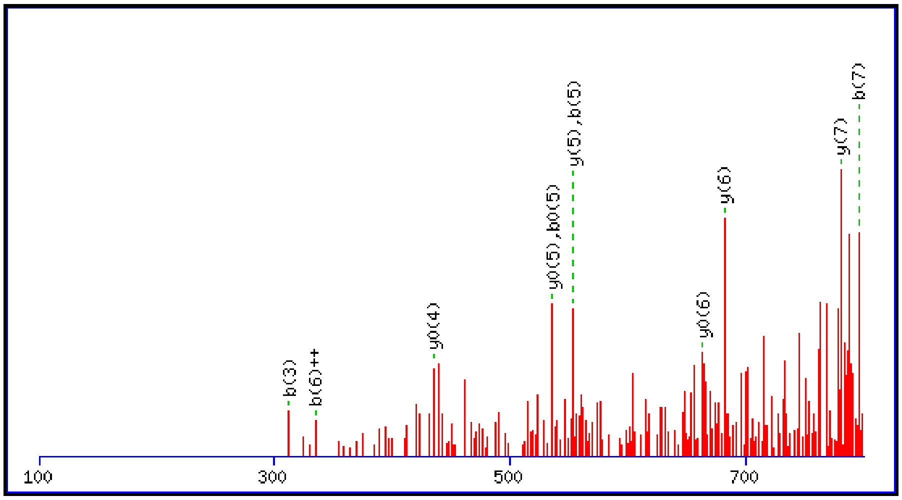

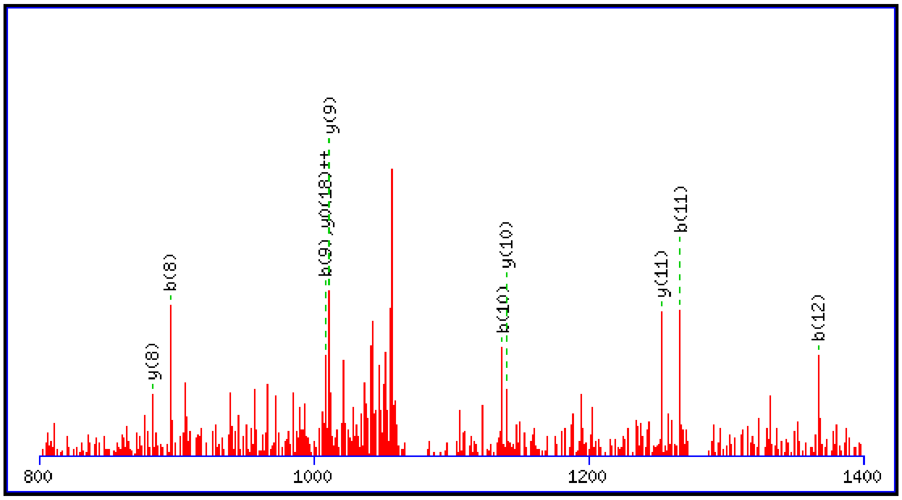

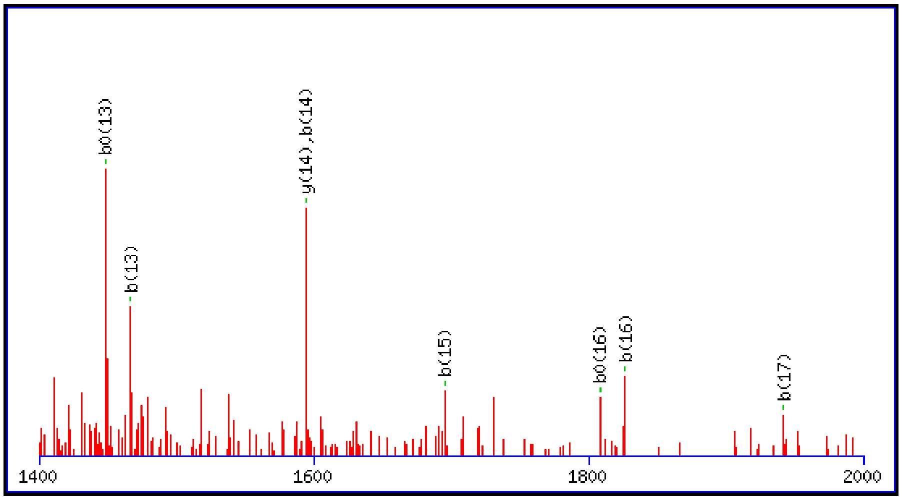

Monoisotopic mass of neutral peptide Mr(calc): 2147.0555
Fixed modifications: Carbamidomethyl (C)
Ions Score: 100 **Expect:** 2.4e-008
Matches (Bold Red): 29/178 fragment ions using 38 most intense peaks

#	b	b^{++}	b*	b^{*++}	b^0	b^{0++}	Seq.	y	y^{++}	y*	y^{*++}	y^0	y^{0++}	#
1	114.0913	57.5493					I							19
2	215.1390	108.0731			197.1285	99.0679	T	2034.9787	1017.9930	2017.9522	1009.4797	2016.9682	**1008.9877**	18
3	**312.1918**	156.5995			294.1812	147.5942	P	1933.9311	967.4692	1916.9045	958.9559	1915.9205	958.4639	17
4	425.2758	213.1416			407.2653	204.1363	L	1836.8783	918.9428	1819.8518	910.4295	1818.8677	909.9375	16
5	**554.3184**	277.6629			**536.3079**	268.6576	E	1723.7942	862.4008	1706.7677	853.8875	1705.7837	853.3955	15
6	667.4025	**334.2049**			649.3919	325.1996	I	**1594.7516**	797.8795	1577.7251	789.3662	1576.7411	788.8742	14
7	**796.4451**	398.7262			778.4345	389.7209	E	1481.6676	741.3374	1464.6410	732.8242	1463.6570	732.3321	13
8	**895.5135**	448.2604			877.5029	439.2551	V	1352.6250	676.8161	1335.5984	668.3029	1334.6144	667.8109	12
9	**1008.5976**	504.8024			990.5870	495.7971	L	**1253.5566**	627.2819	1236.5300	618.7687	1235.5460	618.2766	11
10	**1137.6402**	569.3237			1119.6296	560.3184	E	**1140.4725**	570.7399	1123.4460	562.2266	1122.4619	561.7346	10
11	**1266.6828**	633.8450			1248.6722	624.8397	E	**1011.4299**	506.2186	994.4034	497.7053	993.4194	497.2133	9
12	**1367.7304**	684.3689			1349.7199	675.3636	T	**882.3873**	441.6973	865.3608	433.1840	864.3768	432.6920	8
13	**1466.7989**	733.9031			**1448.7883**	724.8978	V	**781.3396**	391.1735	764.3131	382.6602	763.3291	382.1682	7
14	**1594.8574**	797.9324	1577.8309	789.4191	1576.8469	788.9271	Q	**682.2712**	341.6393	665.2447	333.1260	**664.2607**	332.6340	6
15	**1695.9051**	848.4562	1678.8786	839.9429	1677.8945	839.4509	T	**554.2127**	277.6100			**536.2021**	268.6047	5
16	**1826.9456**	913.9764	1809.9190	905.4632	**1808.9350**	904.9712	M	453.1650	227.0861			**435.1544**	218.0808	4
17	**1941.9725**	971.4899	1924.9460	962.9766	1923.9620	962.4846	D	322.1245	161.5659			304.1139	152.5606	3
18	2043.0202	1022.0137	2025.9937	1013.5005	2025.0097	1013.0085	T	207.0975	104.0524			189.0870	95.0471	2
19							S	106.0499	53.5286			88.0393	44.5233	1

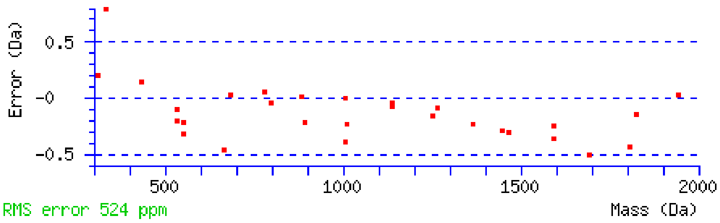

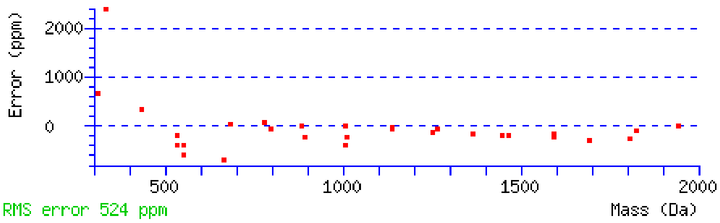

All matches to this query

Score	Mr(calc):	Delta	Sequence
99.9	2147.0555	0.0136	ITPLEIEVLEETVQTMDTS
9.7	2147.0872	-0.0181	DSHLTQHTRIHTGEKPYK
6.5	2146.0558	1.0133	LKLTEDTSEKAGFASTLQK

5.6	2146.0558	1.0133	LKLTEDTSEKAGFASTLQK
4.0	2146.0534	1.0158	MPTSGLIPLQAFLDADIHK
3.7	2146.0534	1.0158	MPTSGLIPLQAFLDADIHK
3.5	2147.0881	-0.0189	LAGPPSCIVPLMRQYRHH
2.8	2146.0493	1.0198	LEAKGTGGTDLMNFLKTVR
2.7	2145.0483	2.0208	LEIDINKITVKTNESIR
0.2	2145.0507	2.0184	QALKLQIEGSDPSFNYKK

Spectrum No: 123; Query: 18591; Rank: 1

Peptide View

MS/MS Fragmentation of **LDFLRPYTVPNK**
Found in **IPI00003217**, Tax_Id=9606 Gene_Symbol=PSMB7 Proteasome subunit beta type-7

Match to Query 18591: 1461.807388 from(731.910970,2+)
Title: 091224LimSK_Exosome3_06.4674.4674.2.dta
Data file C:\Temp\Mascoti\LSK_2D\091224LimSK_Exosome3_01_2D.mgf

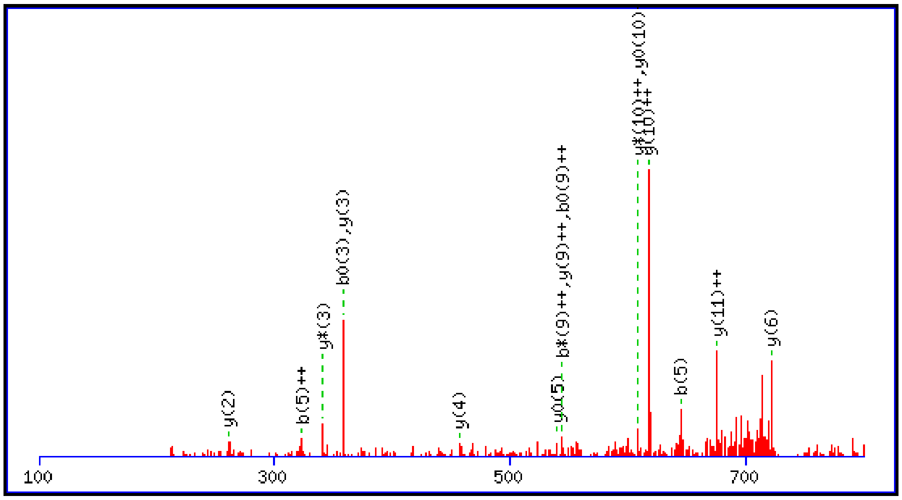

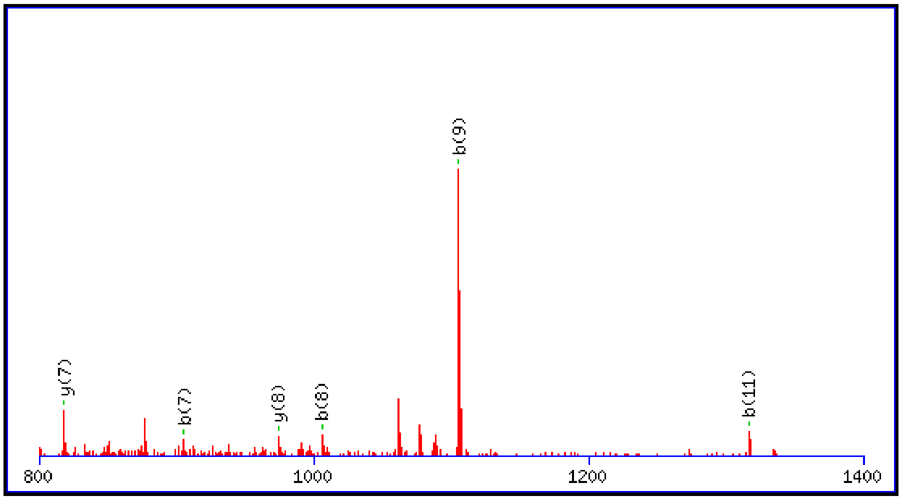

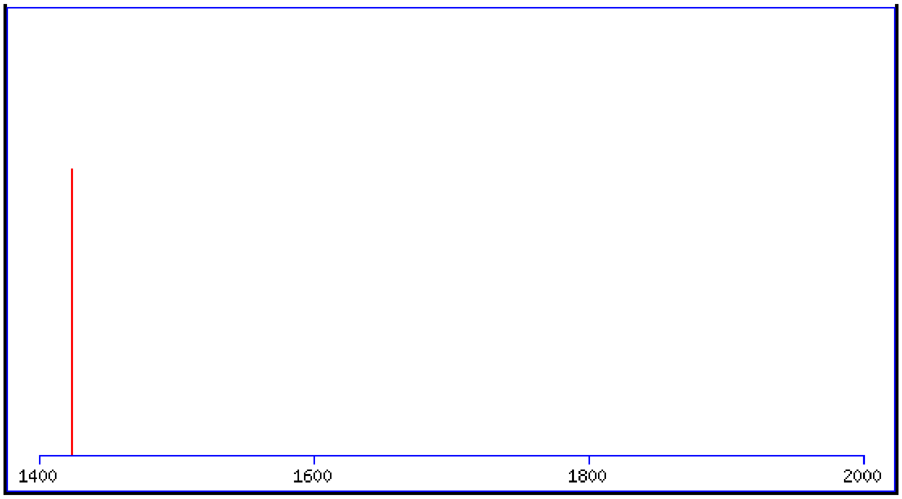

Monoisotopic mass of neutral peptide Mr(calc): 1461.7980
Fixed modifications: Carbamidomethyl (C)
Ions Score: 33 **Expect:** 0.045
Matches (Bold Red): 22/114 fragment ions using 44 most intense peaks

#	b	b^{++}	b*	b^{*++}	b^{0}	b^{0++}	Seq.	y	y^{++}	y*	y^{*++}	y^{0}	y^{0++}	#
1	114.0913	57.5493					L							12
2	229.1183	115.0628			211.1077	106.0575	D	1349.7212	**675.3642**	1332.6947	666.8510	1331.7106	666.3590	11
3	376.1867	188.5970			**358.1761**	179.5917	F	1234.6943	**617.8508**	1217.6677	**609.3375**	1216.6837	**608.8455**	10
4	489.2708	245.1390			471.2602	236.1337	L	1087.6259	**544.3166**	1070.5993	535.8033	1069.6153	535.3113	9
5	**645.3719**	**323.1896**	628.3453	314.6763	627.3613	314.1843	R	**974.5418**	487.7745	957.5152	479.2613	956.5312	478.7693	8
6	742.4246	371.7160	725.3981	363.2027	724.4141	362.7107	P	**818.4407**	409.7240	801.4141	401.2107	800.4301	400.7187	7
7	**905.4880**	453.2476	888.4614	444.7343	887.4774	444.2423	Y	**721.3879**	361.1976	704.3614	352.6843	703.3774	352.1923	6
8	**1006.5356**	503.7715	989.5091	495.2582	988.5251	494.7662	T	558.3246	279.6659	541.2980	271.1527	**540.3140**	270.6606	5
9	**1105.6041**	553.3057	1088.5775	**544.7924**	1087.5935	**544.3004**	V	**457.2769**	229.1421	440.2504	220.6288			4
10	1202.6568	601.8320	1185.6303	593.3188	1184.6463	592.8268	P	**358.2085**	179.6079	**341.1819**	171.0946			3
11	**1316.6997**	658.8535	1299.6732	650.3402	1298.6892	649.8482	N	**261.1557**	131.0815	244.1292	122.5682			2
12							K	147.1128	74.0600	130.0863	65.5468			1

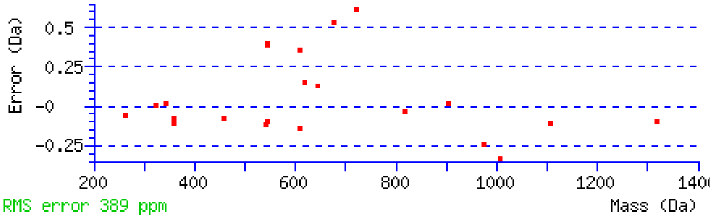

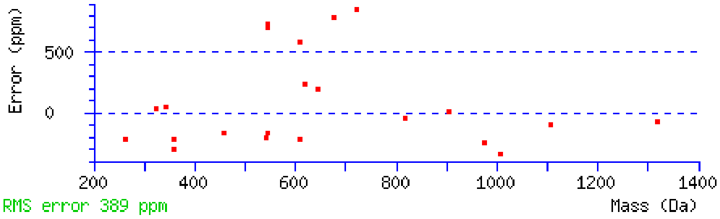

All matches to this query

Score	Mr(calc):	Delta	Sequence
33.0	1461.7980	0.0094	LDFLRPYTVPNK
13.9	1460.8004	1.0070	ELDVVLRTVIPK
8.9	1461.7948	0.0126	MLLCYRHLKTK
7.3	1459.8109	1.9965	FLPVMTEILLER
5.8	1461.7969	0.0105	RAALLVPPRYAR
5.3	1461.7956	0.0118	INGALNITLNLVK
4.4	1461.7939	0.0135	ARIGQYEKQLEK
1.2	1461.7940	0.0134	QGTGLQGQAVFKTK

Spectrum No: 124; Query: 1096; Rank: 1

Peptide View

MS/MS Fragmentation of **FAVATLPPA**
Found in **IPI00000811**, Tax_Id=9606 Gene_Symbol=PSMB6 Proteasome subunit beta type-6

Match to Query 1096: 885.501608 from(443.758080,2+)
Title: 091224LimSK_Exosome3_06.3912.3912.2.dta
Data file C:\Temp\Mascoti\LSK_2D\091224LimSK_Exosome3_01_2D.mgf

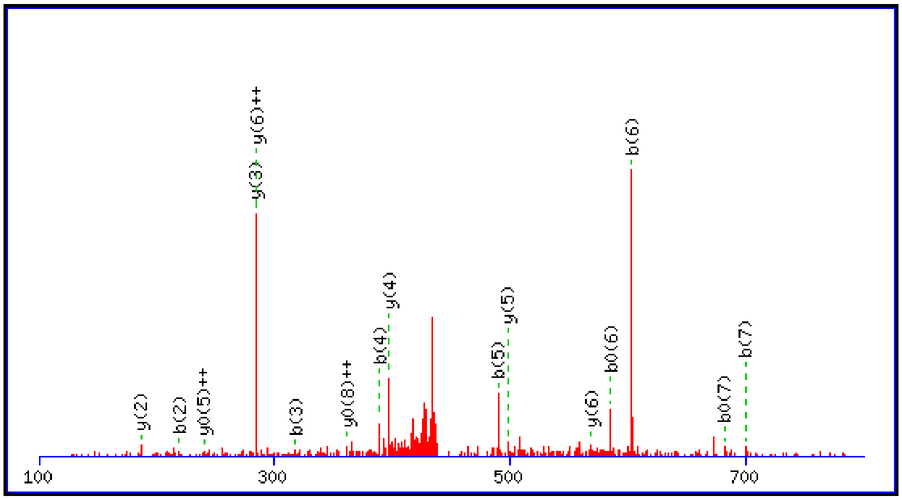

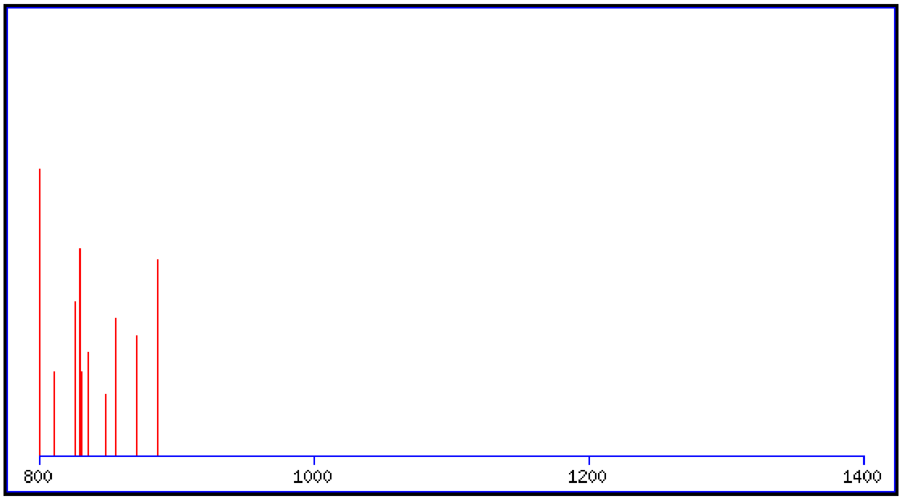

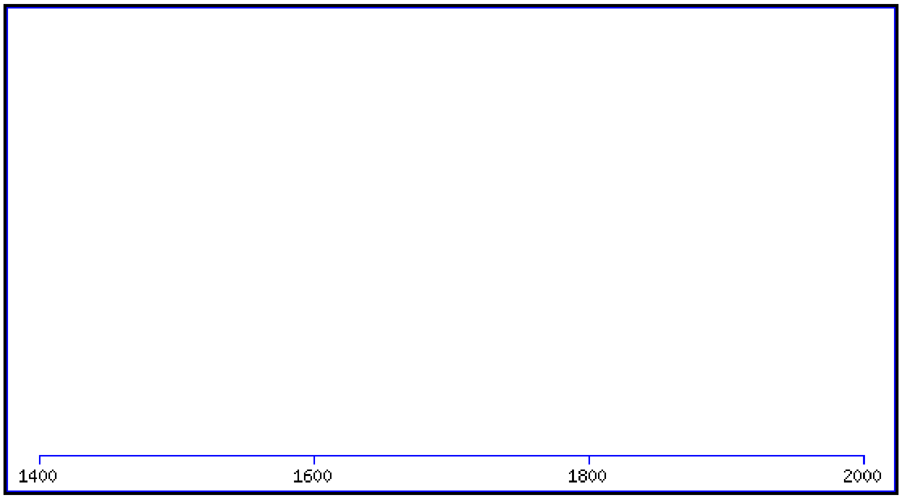

Monoisotopic mass of neutral peptide Mr(calc): 885.4960
Fixed modifications: Carbamidomethyl (C)
Ions Score: 20 **Expect:** 0.66

Matches (Bold Red): 16/48 fragment ions using 70 most intense peaks

#	b	b^{++}	b^{0}	b^{0++}	Seq.	y	y^{++}	y^{0}	y^{0++}	#
1	148.0757	74.5415			F					9
2	**219.1128**	110.0600			A	739.4349	370.2211	721.4243	**361.2158**	8
3	**318.1812**	159.5942			V	668.3978	334.7025	650.3872	325.6972	7
4	**389.2183**	195.1128			A	**569.3293**	**285.1683**	551.3188	276.1630	6
5	**490.2660**	245.6366	472.2554	236.6314	T	**498.2922**	249.6498	480.2817	**240.6445**	5
6	**603.3501**	302.1787	**585.3395**	293.1734	L	**397.2445**	199.1259			4
7	**700.4028**	350.7051	**682.3923**	341.6998	P	**284.1605**	142.5839			3
8	797.4556	399.2314	779.4450	390.2262	P	**187.1077**	94.0575			2
9					A	90.0550	45.5311			1

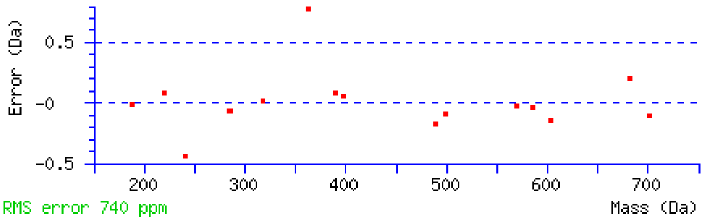

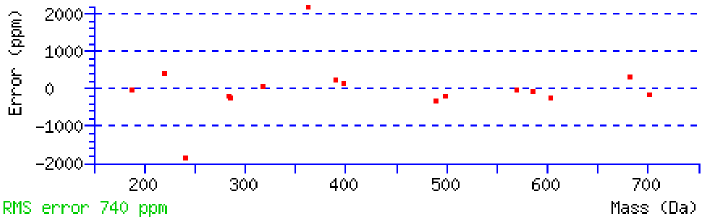

All matches to this query

Score	Mr(calc):	Delta	Sequence
20.3	885.4960	0.0056	FAVATLPPA
10.0	883.5015	2.0001	LADPEVLK
4.5	885.5032	-0.0016	AAAAAAAKNK
4.3	883.5015	2.0001	ILEDGLPK
4.2	885.5032	-0.0016	GRALGAVDK
4.2	883.4949	2.0067	MLLNLHK
2.6	885.5032	-0.0016	GLIKNGER
2.4	883.5015	2.0001	LLGPDLEK
2.3	884.4967	1.0049	XTPIGEIK
2.0	883.5015	2.0001	LVIDEPAK

Spectrum No: 125; Query: 5201; Rank: 1

Peptide View

MS/MS Fragmentation of **LAAIAESGVER**
Found in **IPI00000811**, Tax_Id=9606 Gene_Symbol=PSMB6 Proteasome subunit beta type-6

Match to Query 5201: 1114.601488 from(558.308020,2+)
Title: 091224LimSK_Exosome3_07.1136.1136.2.dta
Data file C:\Temp\Mascoti\LSK_2D\091224LimSK_Exosome3_01_2D.mgf

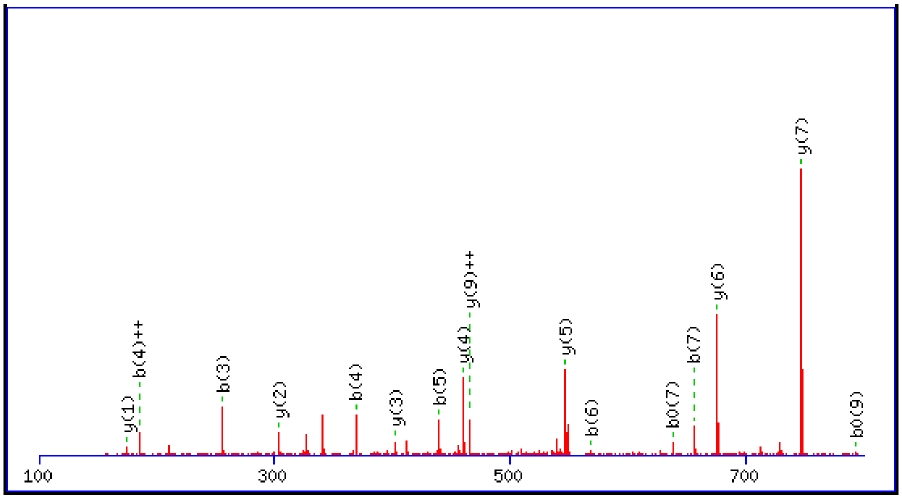

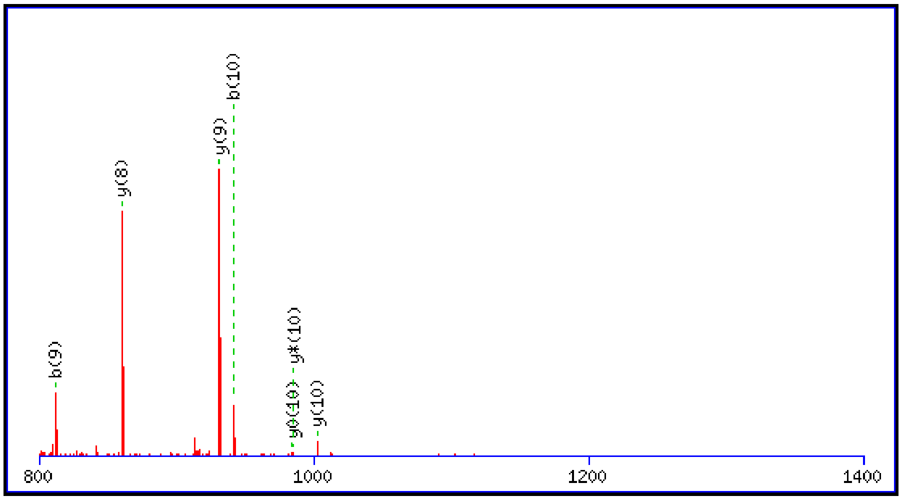

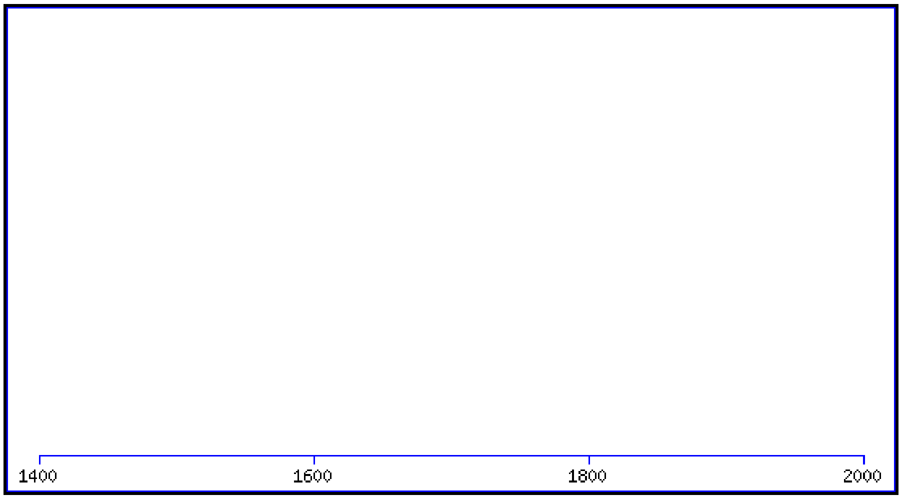

Monoisotopic mass of neutral peptide Mr(calc): 1114.5982
Fixed modifications: Carbamidomethyl (C)
Ions Score: 80 **Expect:** 1.2e-006
Matches (Bold Red): 24/88 fragment ions using 42 most intense peaks

#	b	b^{++}	b^0	b^{0++}	Seq.	y	y^{++}	y*	y^{*++}	y^0	y^{0++}	#
1	114.0913	57.5493			L							11
2	**185.1285**	93.0679			A	**1002.5215**	501.7644	**985.4949**	493.2511	**984.5109**	492.7591	10
3	**256.1656**	128.5864			A	**931.4843**	**466.2458**	914.4578	457.7325	913.4738	457.2405	9
4	**369.2496**	**185.1285**			I	**860.4472**	430.7272	843.4207	422.2140	842.4367	421.7220	8
5	**440.2867**	220.6470			A	**747.3632**	374.1852	730.3366	365.6719	729.3526	365.1799	7
6	**569.3293**	285.1683	551.3188	276.1630	E	**676.3260**	338.6667	659.2995	330.1534	658.3155	329.6614	6

**7**	**656.3614**	328.6843	**638.3508**	319.6790	**S**	**547.2835**	274.1454	530.2569	265.6321	529.2729	265.1401	**5**
**8**	713.3828	357.1951	695.3723	348.1898	**G**	**460.2514**	230.6293	443.2249	222.1161	442.2409	221.6241	**4**
**9**	**812.4512**	406.7293	**794.4407**	397.7240	**V**	**403.2300**	202.1186	386.2034	193.6053	385.2194	193.1133	**3**
**10**	**941.4938**	471.2506	923.4833	462.2453	**E**	**304.1615**	152.5844	287.1350	144.0711	286.1510	143.5791	**2**
**11**					**R**	**175.1190**	88.0631	158.0924	79.5498			**1**

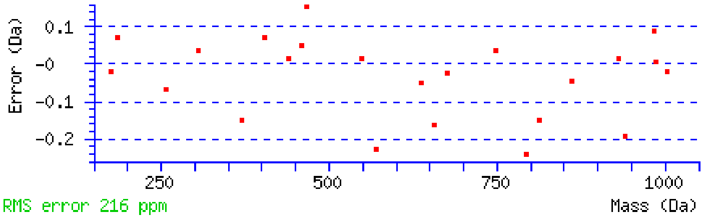

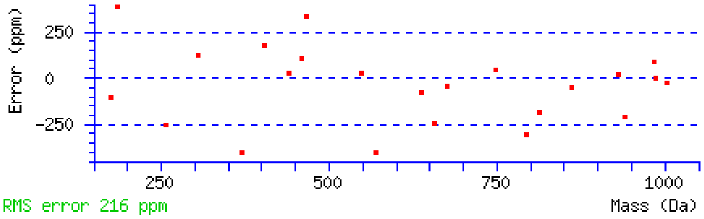

All matches to this query

Score	Mr(calc):	Delta	Sequence
80.0	1114.5982	0.0033	LAAIAESGVER
7.7	1114.6095	-0.0080	LAPSSDLRTR
5.1	1114.6094	-0.0079	ALDEKVRER
4.3	1114.5982	0.0033	LAKQEELER
2.0	1112.5979	2.0036	TKAHVSSVVW
1.9	1114.5982	0.0033	TQEPSLGKQK
1.8	1114.6094	-0.0079	LDINLERSR
1.4	1114.5982	0.0033	ISTGPLGDLSR
0.6	1112.5938	2.0077	EARISPLGDR
0.6	1114.5957	0.0058	KMSIWTPPR

Spectrum No: 126; Query: 5045; Rank: 1

Peptide View

MS/MS Fragmentation of **QVLLGDQIPK**
Found in **IPI00000811**, Tax_Id=9606 Gene_Symbol=PSMB6 Proteasome subunit beta type-6

Match to Query 5045: 1109.649448 from(555.832000,2+)
Title: 091224LimSK_Exosome3_06.2638.2638.2.dta
Data file C:\Temp\Mascoti\LSK_2D\091224LimSK_Exosome3_01_2D.mgf

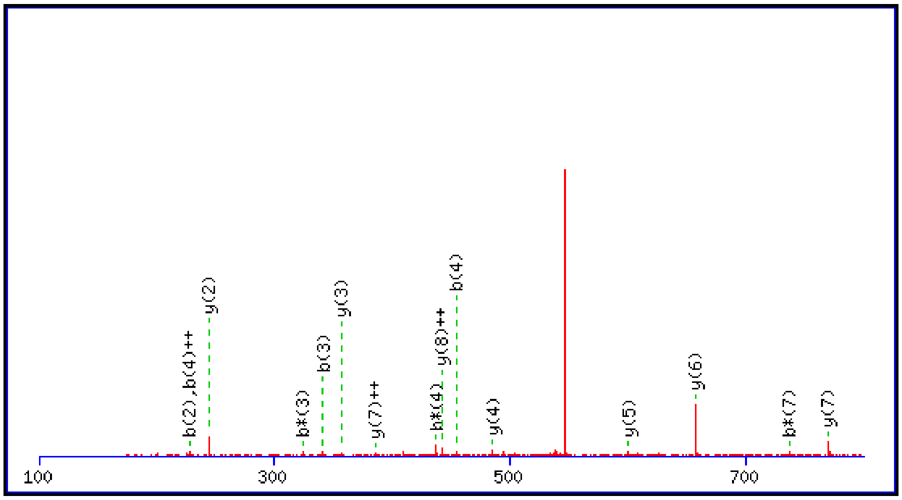

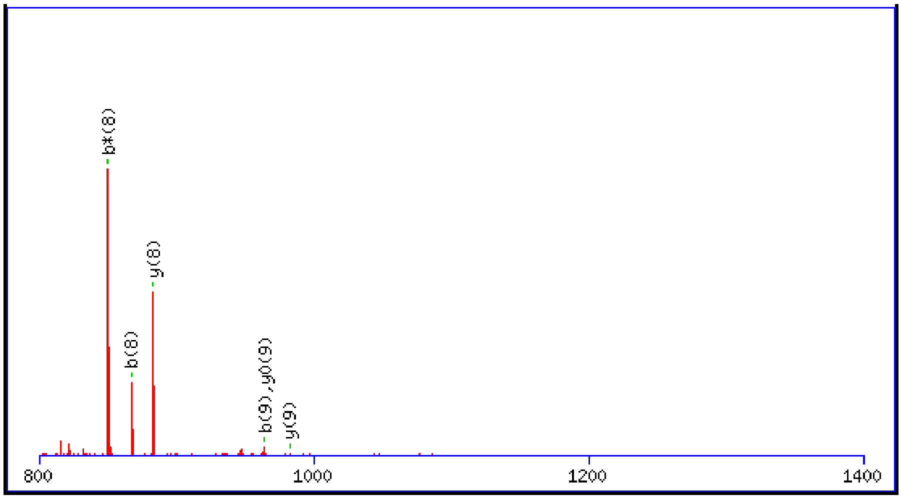

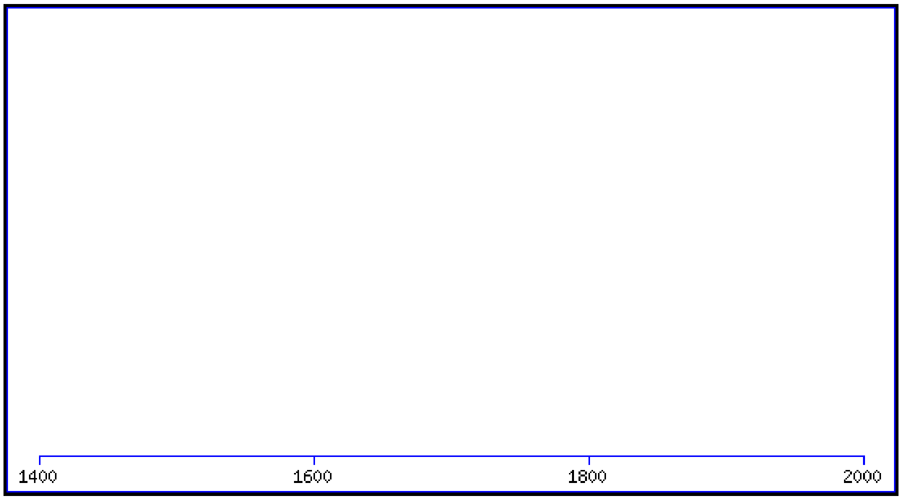

Monoisotopic mass of neutral peptide Mr(calc): 1109.6445
Fixed modifications: Carbamidomethyl (C)
Ions Score: 65 **Expect:** 1.5e-005
Matches (Bold Red): 21/90 fragment ions using 32 most intense peaks

#	b	b^{++}	b*	b^{*++}	b^0	b^{0++}	Seq.	y	y^{++}	y*	y^{*++}	y^0	y^{0++}	#
1	129.0659	65.0366	112.0393	56.5233			Q							10
2	**228.1343**	114.5708	211.1077	106.0575			V	**982.5932**	491.8002	965.5666	483.2869	**964.5826**	482.7949	9
3	**341.2183**	171.1128	**324.1918**	162.5995			L	**883.5247**	**442.2660**	866.4982	433.7527	865.5142	433.2607	8
4	**454.3024**	**227.6548**	**437.2758**	219.1416			L	**770.4407**	**385.7240**	753.4141	377.2107	752.4301	376.7187	7
5	511.3239	256.1656	494.2973	247.6523			G	**657.3566**	329.1819	640.3301	320.6687	639.3461	320.1767	6
6	626.3508	313.6790	609.3243	305.1658	608.3402	304.6738	D	**600.3352**	300.6712	583.3086	292.1579	582.3246	291.6659	5
7	754.4094	377.7083	**737.3828**	369.1951	736.3988	368.7030	Q	**485.3082**	243.1577	468.2817	234.6445			4
8	**867.4934**	434.2504	**850.4669**	425.7371	849.4829	425.2451	I	**357.2496**	179.1285	340.2231	170.6152			3
9	**964.5462**	482.7767	947.5197	474.2635	946.5356	473.7715	P	**244.1656**	122.5864	227.1390	114.0731			2
10							K	147.1128	74.0600	130.0863	65.5468			1

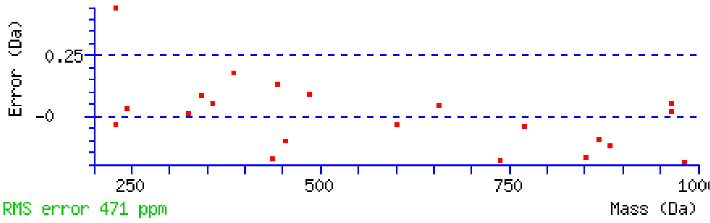

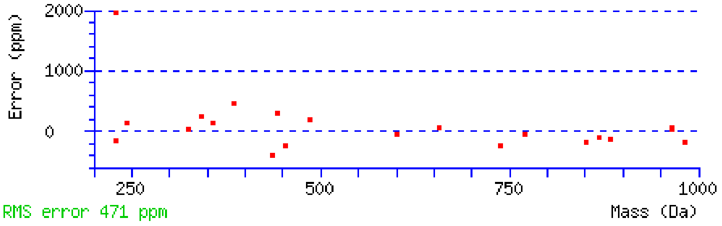

All matches to this query

Score	Mr(calc):	Delta	Sequence
64.5	1109.6445	0.0050	QVLLGDQIPK
12.0	1109.6557	-0.0062	RAIPANVEIK
11.3	1109.6444	0.0050	DKDGKPLLPK
10.3	1109.6557	-0.0062	LNREQLLPK
10.1	1109.6557	-0.0062	RALEQVPGLK
8.8	1109.6444	0.0050	LDKIGLNLPAG
6.3	1108.6492	1.0003	LLEKTSKYK
6.2	1109.6557	-0.0062	LLIRENQPK
6.2	1109.6597	-0.0102	LLLPWLEAR
5.5	1109.6386	0.0109	KVWAGKFFK

Spectrum No: 127; Query: 74020; Rank: 1

Peptide View

MS/MS Fragmentation of **SGSAADTQAVADAVTYQLGFHSIELNEPPLVHTAASLFK**
Found in **IPI00000811**, Tax_Id=9606 Gene_Symbol=PSMB6 Proteasome subunit beta type-6

Match to Query 74020: 4055.063336 from(1014.773110,4+)
Title: 091224LimSK_Exosome3_06.9066.9066.4.dta
Data file C:\Temp\Mascoti\LSK_2D\091224LimSK_Exosome3_01_2D.mgf

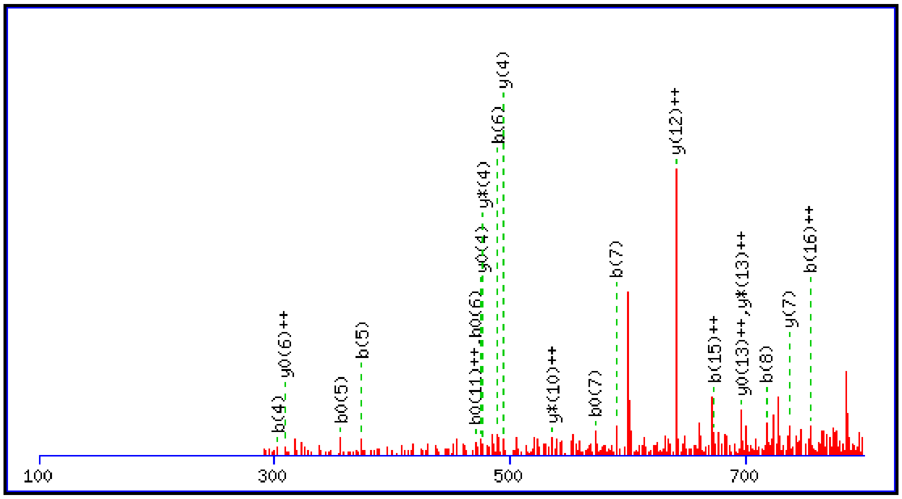

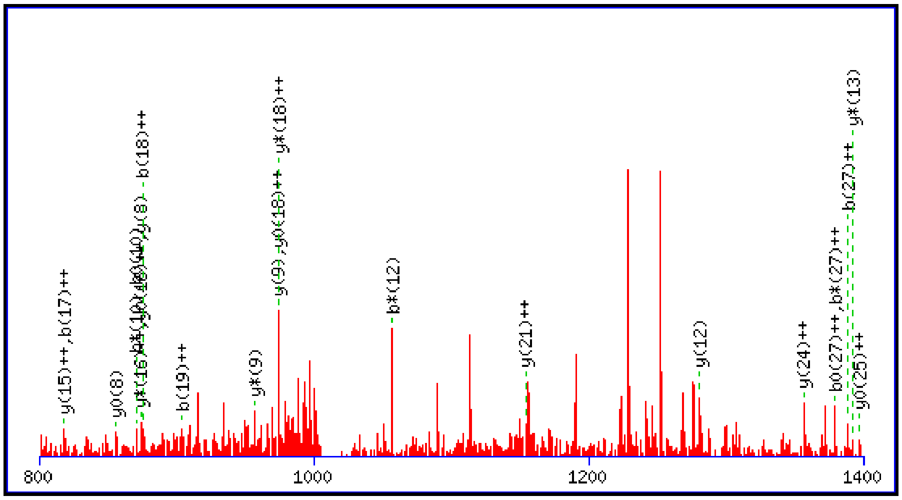

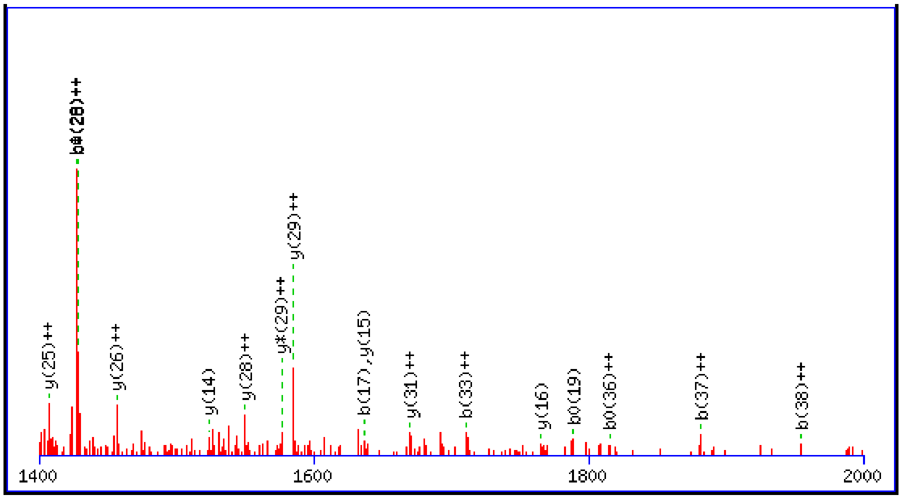

Monoisotopic mass of neutral peptide Mr(calc): 4055.0276
Fixed modifications: Carbamidomethyl (C)
Ions Score: 23 **Expect:** 0.62
Matches (Bold Red): 60/436 fragment ions using 145 most intense peaks

#	b	b^{++}	b*	b^{*++}	b^{0}	b^{0++}	Seq.	y	y^{++}	y*	y^{*++}	y^{0}	y^{0++}	#
1	88.0393	44.5233			70.0287	35.5180	S							39
2	145.0608	73.0340			127.0502	64.0287	G	3969.0029	1985.0051	3951.9763	1976.4918	3950.9923	1975.9998	38
3	232.0928	116.5500			214.0822	107.5448	S	3911.9814	1956.4943	3894.9549	1947.9811	3893.9708	1947.4891	37
4	**303.1299**	152.0686			285.1193	143.0633	A	3824.9494	1912.9783	3807.9228	1904.4651	3806.9388	1903.9730	36
5	**374.1670**	187.5872			**356.1565**	178.5819	A	3753.9123	1877.4598	3736.8857	1868.9465	3735.9017	1868.4545	35
6	**489.1940**	245.1006			**471.1834**	236.0953	D	3682.8752	1841.9412	3665.8486	1833.4279	3664.8646	1832.9359	34
7	**590.2416**	295.6245			**572.2311**	286.6192	T	3567.8482	1784.4277	3550.8217	1775.9145	3549.8376	1775.4225	33
8	**718.3002**	359.6538	701.2737	351.1405	700.2897	350.6485	Q	3466.8005	1733.9039	3449.7740	1725.3906	3448.7900	1724.8986	32
9	789.3373	395.1723	772.3108	386.6590	771.3268	386.1670	A	3338.7420	**1669.8746**	3321.7154	1661.3613	3320.7314	1660.8693	31
10	888.4058	444.7065	**871.3792**	436.1932	**870.3952**	435.7012	V	3267.7048	1634.3561	3250.6783	1625.8428	3249.6943	1625.3508	30
11	959.4429	480.2251	942.4163	471.7118	941.4323	**471.2198**	A	3168.6364	**1584.8219**	3151.6099	**1576.3086**	3150.6259	1575.8166	29
12	1074.4698	537.7385	**1057.4433**	529.2253	1056.4592	528.7333	D	3097.5993	**1549.3033**	3080.5728	1540.7900	3079.5887	1540.2980	28
13	1145.5069	573.2571	1128.4804	564.7438	1127.4964	564.2518	A	2982.5724	1491.7898	2965.5458	1483.2765	2964.5618	1482.7845	27
14	1244.5753	622.7913	1227.5488	614.2780	1226.5648	613.7860	V	2911.5353	**1456.2713**	2894.5087	1447.7580	2893.5247	1447.2660	26
15	1345.6230	**673.3151**	1328.5965	664.8019	1327.6125	664.3099	T	2812.4668	**1406.7371**	2795.4403	1398.2238	2794.4563	**1397.7318**	25
16	1508.6863	**754.8468**	1491.6598	746.3335	1490.6758	745.8415	Y	2711.4192	**1356.2132**	2694.3926	1347.6999	2693.4086	1347.2079	24
17	**1636.7449**	**818.8761**	1619.7184	810.3628	1618.7344	809.8708	Q	2548.3558	1274.6816	2531.3293	1266.1683	2530.3453	1265.6763	23
18	1749.8290	**875.4181**	1732.8024	866.9049	1731.8184	866.4128	L	2420.2973	1210.6523	2403.2707	1202.1390	2402.2867	1201.6470	22
19	1806.8505	**903.9289**	1789.8239	895.4156	**1788.8399**	894.9236	G	2307.2132	**1154.1102**	2290.1866	1145.5970	2289.2026	1145.1050	21
20	1953.9189	977.4631	1936.8923	968.9498	1935.9083	968.4578	F	2250.1917	1125.5995	2233.1652	1117.0862	2232.1812	1116.5942	20
21	2090.9778	1045.9925	2073.9512	1037.4793	2072.9672	1036.9872	H	2103.1233	1052.0653	2086.0968	1043.5520	2085.1128	1043.0600	19
22	2178.0098	1089.5085	2160.9833	1080.9953	2159.9992	1080.5033	S	1966.0644	983.5358	1949.0379	**975.0226**	1948.0538	**974.5306**	18
23	2291.0939	1146.0506	2274.0673	1137.5373	2273.0833	1137.0453	I	1879.0324	940.0198	1862.0058	931.5066	1861.0218	931.0145	17
24	2420.1365	1210.5719	2403.1099	1202.0586	2402.1259	1201.5666	E	**1765.9483**	883.4778	1748.9218	**874.9645**	1747.9377	**874.4725**	16
25	2533.2205	1267.1139	2516.1940	1258.6006	2515.2100	1258.1086	L	**1636.9057**	**818.9565**	1619.8792	810.4432	1618.8952	809.9512	15
26	2647.2635	1324.1354	2630.2369	1315.6221	2629.2529	1315.1301	N	**1523.8217**	762.4145	1506.7951	753.9012	1505.8111	753.4092	14
27	2776.3060	**1388.6567**	2759.2795	**1380.1434**	2758.2955	**1379.6514**	E	1409.7787	705.3930	**1392.7522**	**696.8797**	1391.7682	**696.3877**	13
28	2873.3588	1437.1830	2856.3323	**1428.6698**	2855.3482	**1428.1778**	P	**1280.7361**	**640.8717**	1263.7096	632.3584	1262.7256	631.8664	12
29	2970.4116	1485.7094	2953.3850	1477.1962	2952.4010	1476.7041	P	1183.6834	592.3453	1166.6568	583.8320	1165.6728	583.3400	11
30	3083.4956	1542.2515	3066.4691	1533.7382	3065.4851	1533.2462	L	1086.6306	543.8189	1069.6041	**535.3057**	1068.6200	534.8137	10
31	3182.5641	1591.7857	3165.5375	1583.2724	3164.5535	1582.7804	V	**973.5465**	487.2769	**956.5200**	478.7636	955.5360	478.2716	9
32	3319.6230	1660.3151	3302.5964	1651.8018	3301.6124	1651.3098	H	**874.4781**	437.7427	857.4516	429.2294	**856.4676**	428.7374	8
33	3420.6706	**1710.8390**	3403.6441	1702.3257	3402.6601	1701.8337	T	**737.4192**	369.2132	720.3927	360.7000	719.4087	360.2080	7

**34**	3491.7078	1746.3575	3474.6812	1737.8442	3473.6972	1737.3522	**A**	636.3715	318.6894	619.3450	310.1761	618.3610	**309.6841**	**6**
**35**	3562.7449	1781.8761	3545.7183	1773.3628	3544.7343	1772.8708	**A**	565.3344	283.1709	548.3079	274.6576	547.3239	274.1656	**5**
**36**	3649.7769	1825.3921	3632.7504	1816.8788	3631.7663	**1816.3868**	**S**	**494.2973**	247.6523	**477.2708**	239.1390	**476.2867**	238.6470	**4**
**37**	3762.8610	**1881.9341**	3745.8344	1873.4208	3744.8504	1872.9288	**L**	407.2653	204.1363	390.2387	195.6230			**3**
**38**	3909.9294	**1955.4683**	3892.9028	1946.9551	3891.9188	1946.4630	**F**	294.1812	147.5942	277.1547	139.0810			**2**
**39**							**K**	147.1128	74.0600	130.0863	65.5468			**1**

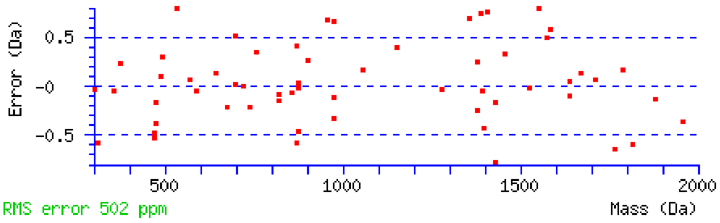

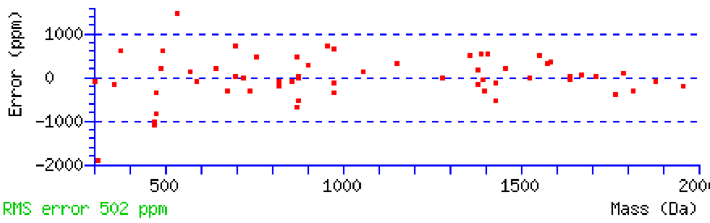

All matches to this query

Score	Mr(calc):	Delta	Sequence
22.9	4055.0276	0.0357	SGSAADTQAVADAVTYQLGFHSIELNEPPLVHTAASLFK
11.9	4053.0322	2.0311	MAERGYSFSLTTFSPSGKLVQIEYALAAVAGGAPSVGIK
7.9	4055.0379	0.0254	LSIFIYEVVTLSLSLQIAQSGVLWFLLSHSPARK
6.3	4053.0322	2.0311	MAERGYSFSLTTFSPSGKLVQIEYALAAVAGGAPSVGIK
5.5	4054.0947	0.9687	APMFLLIGSLALADLLAGIGLITNFVFAYLLQSEATK
5.5	4053.0728	1.9905	GFITIVDVQRVLESINVQMDENTLHEILNEVDLNK
5.3	4054.0947	0.9687	APMFLLIGSLALADLLAGIGLITNFVFAYLLQSEATK
5.2	4053.0322	2.0311	MAERGYSFSLTTFSPSGKLVQIEYALAAVAGGAPSVGIK
5.2	4053.0322	2.0311	MAERGYSFSLTTFSPSGKLVQIEYALAAVAGGAPSVGIK
5.2	4053.0322	2.0311	MAERGYSFSLTTFSPSGKLVQIEYALAAVAGGAPSVGIK

Spectrum No: 128; Query: 4307; Rank: 1

Peptide View

MS/MS Fragmentation of **TTTGSYIANR**
Found in **IPI00000811**, Tax_Id=9606 Gene_Symbol=PSMB6 Proteasome subunit beta type-6

Match to Query 4307: 1082.539168 from(542.276860,2+)
Title: 091224LimSK_Exosome3_07.361.361.2.dta
Data file C:\Temp\Mascoti\LSK_2D\091224LimSK_Exosome3_01_2D.mgf

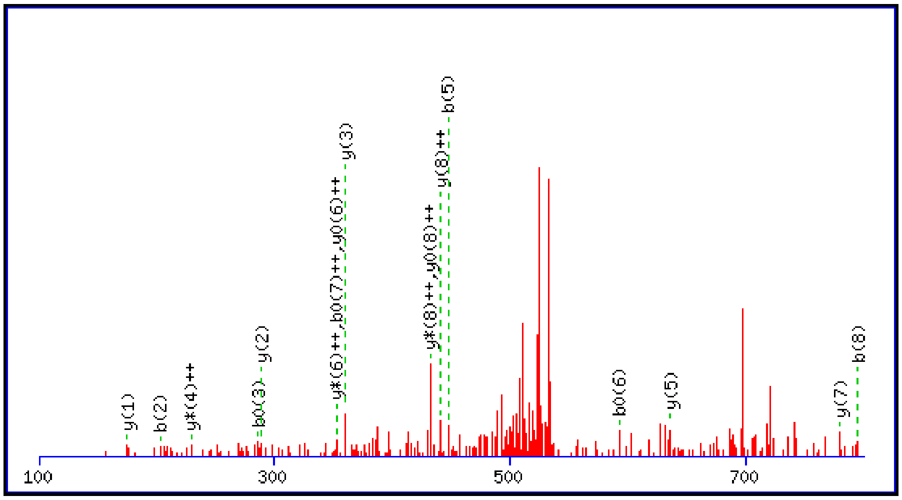

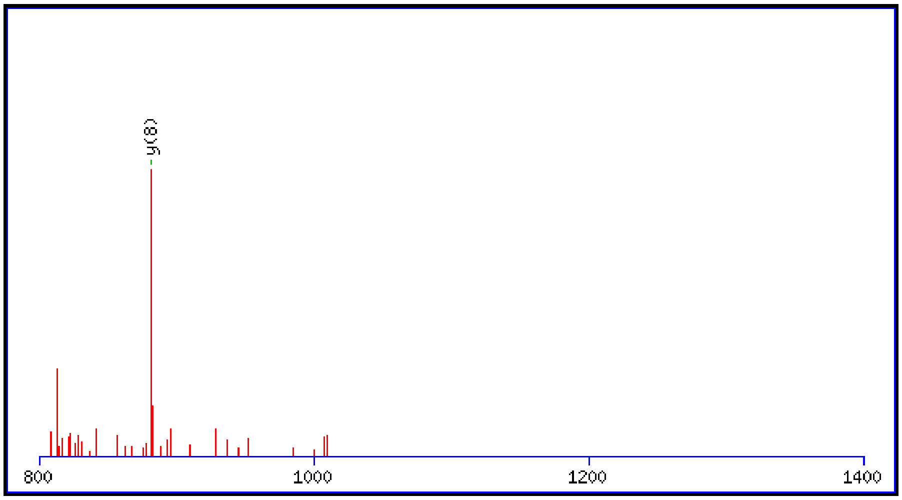

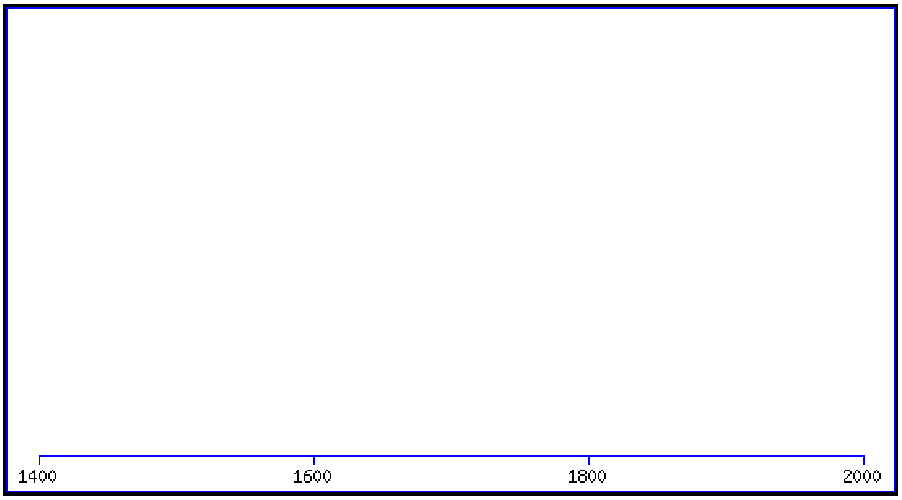

Monoisotopic mass of neutral peptide Mr(calc): 1082.5356
Fixed modifications: Carbamidomethyl (C)
Ions Score: 25 **Expect:** 0.45
Matches (Bold Red): 18/82 fragment ions using 55 most intense peaks

#	b	b^{++}	b*	b^{*++}	b^0	b^{0++}	Seq.	y	y^{++}	y*	y^{*++}	y^0	y^{0++}	#
1	102.0550	51.5311			84.0444	42.5258	T							10
2	**203.1026**	102.0550			185.0921	93.0497	T	982.4952	491.7513	965.4687	483.2380	964.4847	482.7460	9
3	304.1503	152.5788			**286.1397**	143.5735	T	**881.4476**	**441.2274**	864.4210	**432.7141**	863.4370	**432.2221**	8
4	361.1718	181.0895			343.1612	172.0842	G	**780.3999**	390.7036	763.3733	382.1903	762.3893	381.6983	7
5	**448.2038**	224.6055			430.1932	215.6003	S	723.3784	362.1928	706.3519	**353.6796**	705.3678	**353.1876**	6
6	611.2671	306.1372			**593.2566**	297.1319	Y	**636.3464**	318.6768	619.3198	310.1636			5
7	724.3512	362.6792			706.3406	**353.6740**	I	473.2831	237.1452	456.2565	**228.6319**			4
8	**795.3883**	398.1978			777.3777	389.1925	A	**360.1990**	180.6031	343.1724	172.0899			3
9	909.4312	455.2193	892.4047	446.7060	891.4207	446.2140	N	**289.1619**	145.0846	272.1353	136.5713			2
10							R	**175.1190**	88.0631	158.0924	79.5498			1

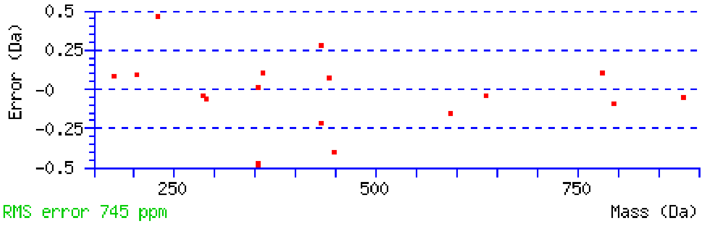

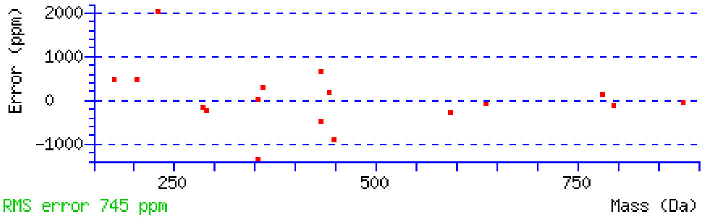

All matches to this query

Score	Mr(calc):	Delta	Sequence
25.1	1082.5356	0.0035	TTTGSYIANR
13.0	1081.5451	0.9941	RLDAAPHMR
5.7	1080.5312	2.0080	EASGQPSLHR
5.1	1082.5468	-0.0077	NSSLIQHER
4.6	1080.5386	2.0006	EGLQMYGKR
4.3	1082.5403	-0.0011	AMYSGRRSR
4.1	1081.5281	1.0111	SISRPSSLR
4.1	1081.5281	1.0111	SISRPSSLR
3.9	1082.5485	-0.0093	TTIKKQER
3.9	1082.5485	-0.0093	TTIKKQER

Spectrum No: 129; Query: 10994; Rank: 1

Peptide View

MS/MS Fragmentation of **VTDKLTPIHDR**
Found in **IPI00000811**, Tax_Id=9606 Gene_Symbol=PSMB6 Proteasome subunit beta type-6

Match to Query 10994: 1293.709422 from(432.243750,3+)
Title: 091224LimSK_Exosome3_07.408.408.3.dta
Data file C:\Temp\Mascoti\LSK_2D\091224LimSK_Exosome3_01_2D.mgf

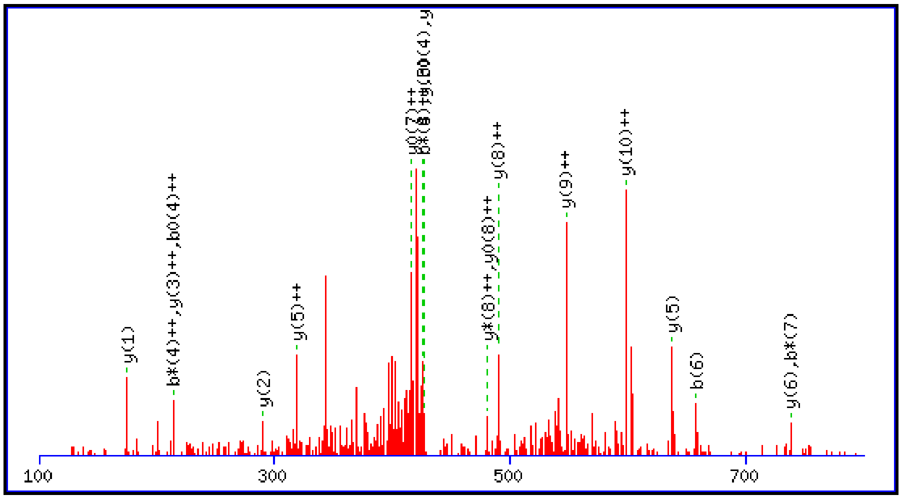

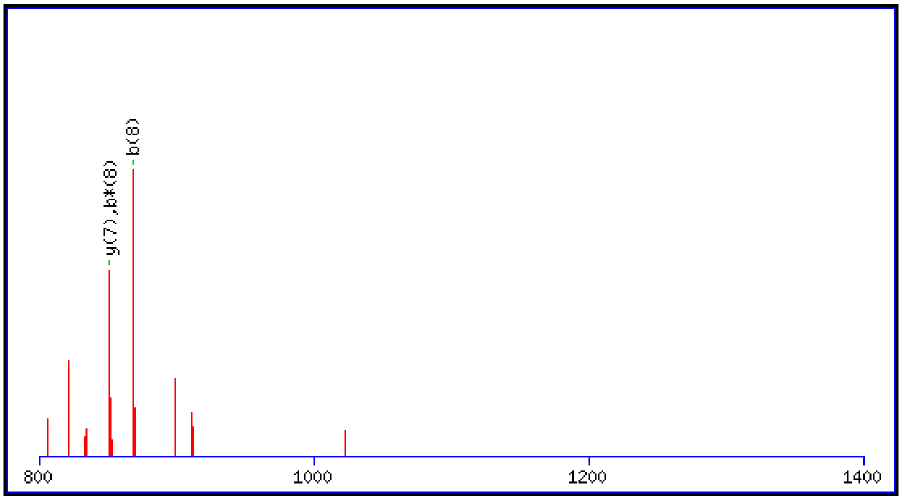

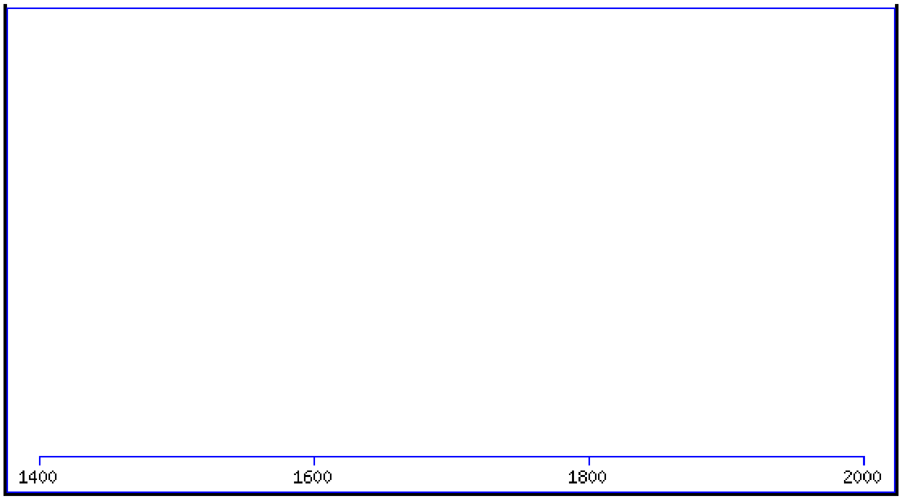

Monoisotopic mass of neutral peptide Mr(calc): 1293.7041
Fixed modifications: Carbamidomethyl (C)
Ions Score: 52 **Expect:** 0.00074
Matches (Bold Red): 25/110 fragment ions using 29 most intense peaks

#	b	b^{++}	b*	b^{*++}	b^0	b^{0++}	Seq.	y	y^{++}	y*	y^{*++}	y^0	y^{0++}	#
1	100.0757	50.5415					V							11
2	201.1234	101.0653			183.1128	92.0600	T	1195.6430	**598.3251**	1178.6164	589.8118	1177.6324	589.3198	10
3	316.1503	158.5788			298.1397	149.5735	D	1094.5953	**547.8013**	1077.5687	539.2880	1076.5847	538.7960	9
4	444.2453	222.6263	**427.2187**	**214.1130**	**426.2347**	**213.6210**	K	979.5683	**490.2878**	962.5418	**481.7745**	961.5578	**481.2825**	8
5	557.3293	279.1683	540.3028	270.6550	539.3188	270.1630	L	**851.4734**	**426.2403**	834.4468	417.7271	833.4628	**417.2350**	7
6	**658.3770**	329.6921	641.3505	321.1789	640.3665	320.6869	T	**738.3893**	369.6983	721.3628	361.1850	720.3787	360.6930	6
7	755.4298	378.2185	**738.4032**	369.7053	737.4192	369.2132	P	**637.3416**	**319.1745**	620.3151	310.6612	619.3311	310.1692	5
8	**868.5138**	434.7606	**851.4873**	**426.2473**	850.5033	**425.7553**	I	540.2889	270.6481	523.2623	262.1348	522.2783	261.6428	4
9	1005.5728	503.2900	988.5462	494.7767	987.5622	494.2847	H	**427.2048**	**214.1060**	410.1783	205.5928	409.1942	205.1008	3
10	1120.5997	560.8035	1103.5732	552.2902	1102.5891	551.7982	D	**290.1459**	145.5766	273.1193	137.0633	272.1353	136.5713	2
11							R	**175.1190**	88.0631	158.0924	79.5498			1

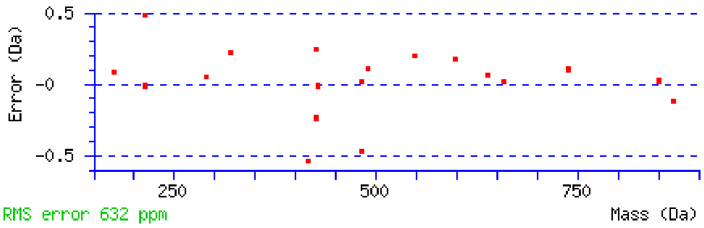

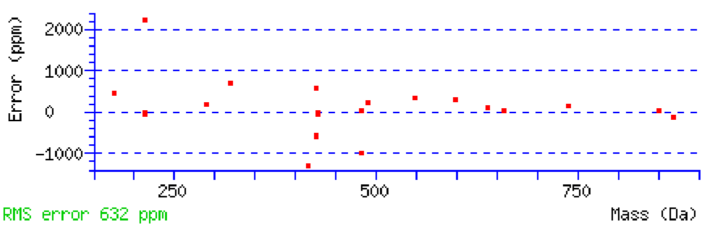

All matches to this query

Score	Mr(calc):	Delta	Sequence
51.8	1293.7041	0.0053	VTDKLTPIHDR
17.3	1293.7170	-0.0075	VIVISRSLTAR
14.1	1292.7078	1.0016	LRGVRLLSGSR
13.0	1293.7170	-0.0075	VIVISRSLTAR
8.6	1293.7153	-0.0059	SHALQLPKTSGR
8.4	1291.7013	2.0081	IRALTLQLER
7.3	1293.7071	0.0024	XWTLTLLRR
6.7	1293.7041	0.0053	VLSTVHTHSSVK
6.4	1293.7170	-0.0075	VIVISRSLTAR
6.1	1291.7071	2.0024	KAIACLLFGGSR

Spectrum No: 130; Query: 4664; Rank: 1

Peptide View

MS/MS Fragmentation of **ASAGSYISALR**
Found in **IPI00000783**, Tax_Id=9606 Gene_Symbol=PSMB8 Isoform 1 of Proteasome subunit beta type-8

Match to Query 4664: 1094.577288 from(548.295920,2+)
Title: 091224LimSK_Exosome3_06.2748.2748.2.dta
Data file C:\Temp\Mascoti\LSK_2D\091224LimSK_Exosome3_01_2D.mgf

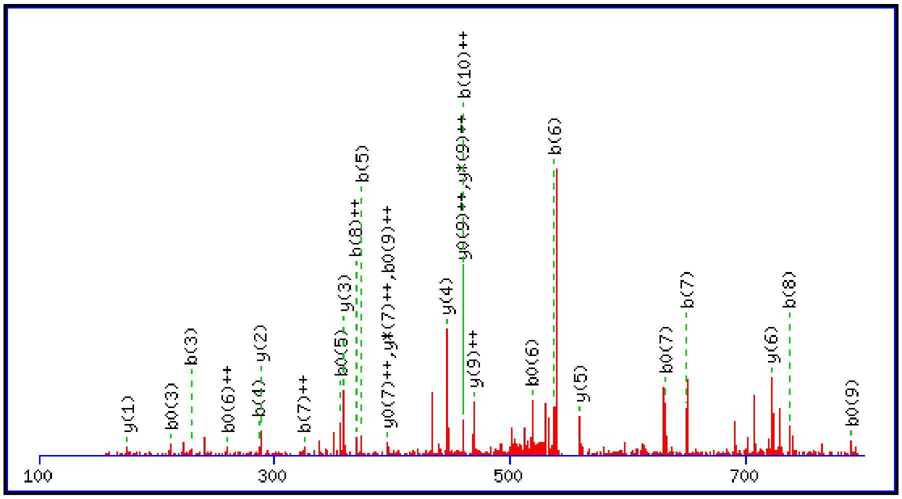

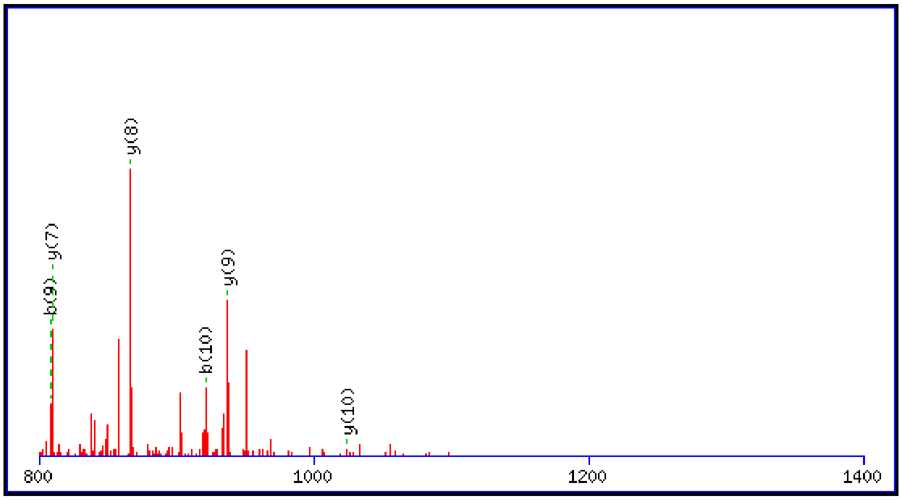

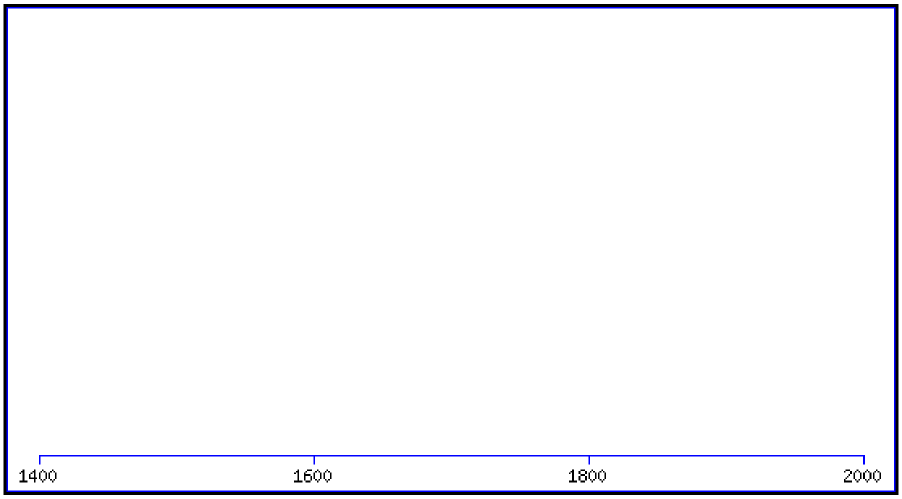

Monoisotopic mass of neutral peptide Mr(calc): 1094.5720

Fixed modifications: Carbamidomethyl (C)
Ions Score: 59 **Expect:** 0.00015
Matches (Bold Red): 33/92 fragment ions using 70 most intense peaks

#	b	b^{++}	b^{0}	b^{0++}	Seq.	y	y^{++}	y*	y^{*++}	y^{0}	y^{0++}	#
1	72.0444	36.5258			A							11
2	159.0764	80.0418	141.0659	71.0366	S	**1024.5422**	512.7747	1007.5156	504.2615	1006.5316	503.7694	10
3	**230.1135**	115.5604	**212.1030**	106.5551	A	**937.5102**	**469.2587**	920.4836	**460.7454**	919.4996	**460.2534**	9
4	**287.1350**	144.0711	269.1244	135.0659	G	**866.4730**	433.7402	849.4465	425.2269	848.4625	424.7349	8
5	**374.1670**	187.5872	**356.1565**	178.5819	S	**809.4516**	405.2294	792.4250	**396.7162**	791.4410	**396.2241**	7
6	**537.2304**	269.1188	**519.2198**	**260.1135**	Y	**722.4196**	361.7134	705.3930	353.2001	704.4090	352.7081	6
7	**650.3144**	**325.6608**	**632.3039**	316.6556	I	**559.3562**	280.1817	542.3297	271.6685	541.3457	271.1765	5
8	**737.3464**	**369.1769**	719.3359	360.1716	S	**446.2722**	223.6397	429.2456	215.1264	428.2616	214.6344	4
9	**808.3836**	404.6954	**790.3730**	**395.6901**	A	**359.2401**	180.1237	342.2136	171.6104			3
10	**921.4676**	**461.2374**	903.4571	452.2322	L	**288.2030**	144.6051	271.1765	136.0919			2
11					R	**175.1190**	88.0631	158.0924	79.5498			1

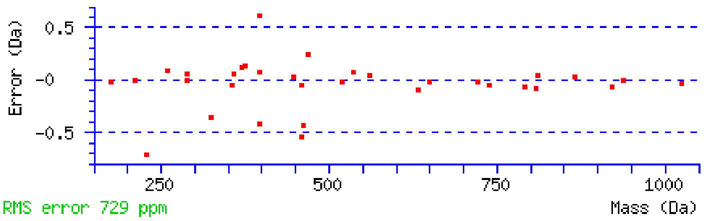

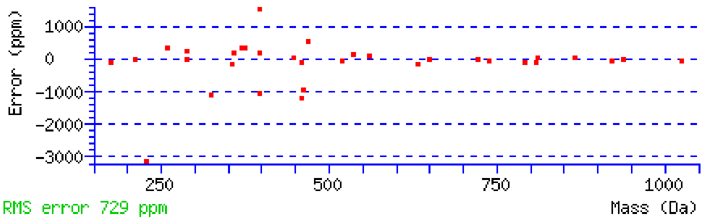

All matches to this query

Score	Mr(calc):	Delta	Sequence
59.3	1094.5720	0.0053	ASAGSYISALR
13.3	1094.5720	0.0053	VFSQTSSLAR
9.9	1094.5832	-0.0059	LNQISHDIR
7.1	1094.5849	-0.0076	ASKLTQVLR
6.8	1094.5849	-0.0076	TGKLNLLTR
5.8	1094.5832	-0.0059	AESAALHAGLR
5.3	1094.5794	-0.0021	QMVSFEKVK
5.0	1093.5698	1.0075	XIMMLSVLK
4.9	1093.5757	1.0016	RAVASGVKAR
4.8	1094.5832	-0.0059	AFSASTSLRR

Spectrum No: 131; Query: 11154; Rank: 1

Peptide View

MS/MS Fragmentation of **FQHGVIAAVDSR**
Found in **IPI00000783**, Tax_Id=9606 Gene_Symbol=PSMB8 Isoform 1 of Proteasome subunit beta type-8

Match to Query 11154: 1298.680212 from(433.900680,3+)
Title: 091224LimSK_Exosome3_06.1172.1172.3.dta
Data file C:\Temp\Mascoti\LSK_2D\091224LimSK_Exosome3_01_2D.mgf

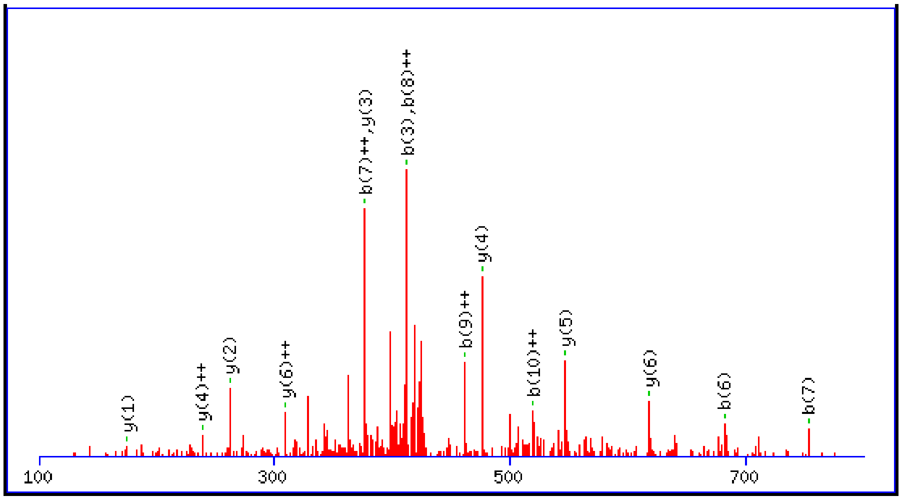

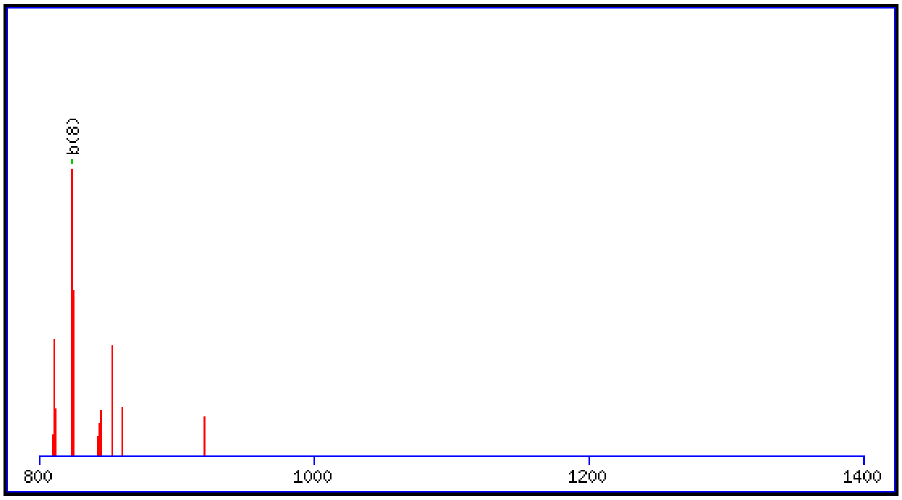

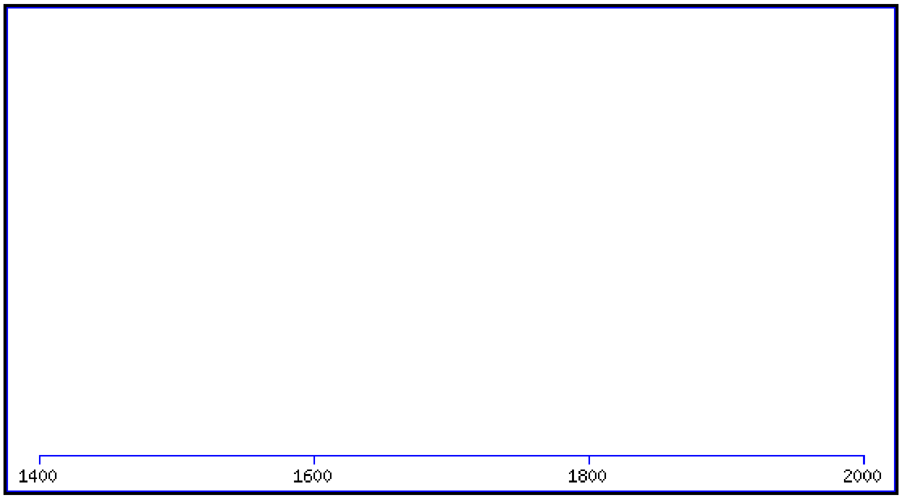

Monoisotopic mass of neutral peptide Mr(calc): 1298.6731
Fixed modifications: Carbamidomethyl (C)
Ions Score: 31 **Expect:** 0.092
Matches (Bold Red): 16/110 fragment ions using 28 most intense peaks

#	b	b^{++}	b*	b^{*++}	b^0	b^{0++}	Seq.	y	y^{++}	y*	y^{*++}	y^0	y^{0++}	#
1	148.0757	74.5415					F							12
2	276.1343	138.5708	259.1077	130.0575			Q	1152.6120	576.8096	1135.5854	568.2964	1134.6014	567.8044	11
3	**413.1932**	207.1002	396.1666	198.5870			H	1024.5534	512.7803	1007.5269	504.2671	1006.5429	503.7751	10
4	470.2146	235.6110	453.1881	227.0977			G	887.4945	444.2509	870.4680	435.7376	869.4839	435.2456	9
5	569.2831	285.1452	552.2565	276.6319			V	830.4730	415.7402	813.4465	407.2269	812.4625	406.7349	8
6	**682.3671**	341.6872	665.3406	333.1739			I	731.4046	366.2060	714.3781	357.6927	713.3941	357.2007	7

**7**	**753.4042**	**377.2058**	736.3777	368.6925			**A**	**618.3206**	**309.6639**	601.2940	301.1506	600.3100	300.6586	**6**
**8**	**824.4413**	**412.7243**	807.4148	404.2110			**A**	**547.2835**	274.1454	530.2569	265.6321	529.2729	265.1401	**5**
**9**	923.5098	**462.2585**	906.4832	453.7452			**V**	**476.2463**	**238.6268**	459.2198	230.1135	458.2358	229.6215	**4**
**10**	1038.5367	**519.7720**	1021.5102	511.2587	1020.5261	510.7667	**D**	**377.1779**	189.0926	360.1514	180.5793	359.1674	180.0873	**3**
**11**	1125.5687	563.2880	1108.5422	554.7747	1107.5582	554.2827	**S**	**262.1510**	131.5791	245.1244	123.0659	244.1404	122.5738	**2**
**12**							**R**	**175.1190**	88.0631	158.0924	79.5498			**1**

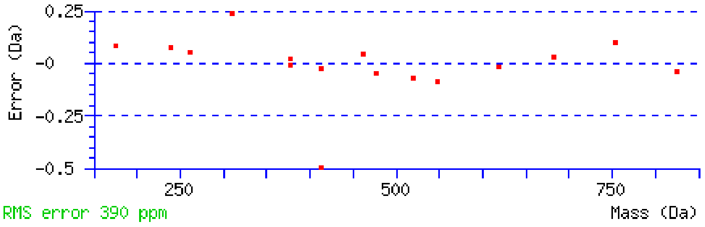

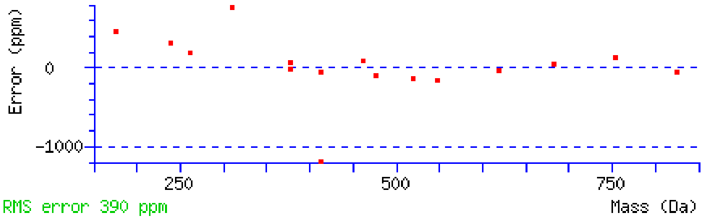

All matches to this query

Score	Mr(calc):	Delta	Sequence
31.2	1298.6731	0.0071	FQHGVIAAVDSR
11.5	1298.6731	0.0071	RPWEPSALSTR
11.2	1297.6779	1.0023	NFSKFQATSLR
8.2	1296.6690	2.0112	KPISDTISKTK
7.0	1298.6803	-0.0001	RALAQAGRGEDR
6.6	1297.6740	1.0062	LCFAVYAVLDK
6.2	1296.6690	2.0112	TIKTVKADVDK
6.2	1296.6690	2.0112	TIKTVKADVDK
5.8	1296.6690	2.0112	SSVLPKASTLSK
5.0	1296.6663	2.0139	RAVSTATLRSR

Spectrum No: 132; Query: 18625; Rank: 1

Peptide View

MS/MS Fragmentation of **GPGLYYVDEHGTR**
Found in **IPI00000783**, Tax_Id=9606 Gene_Symbol=PSMB8 Isoform 1 of Proteasome subunit beta type-8

Match to Query 18625: 1462.690868 from(732.352710,2+)
Title: 091224LimSK_Exosome3_06.1477.1477.2.dta
Data file C:\Temp\Mascoti\LSK_2D\091224LimSK_Exosome3_01_2D.mgf

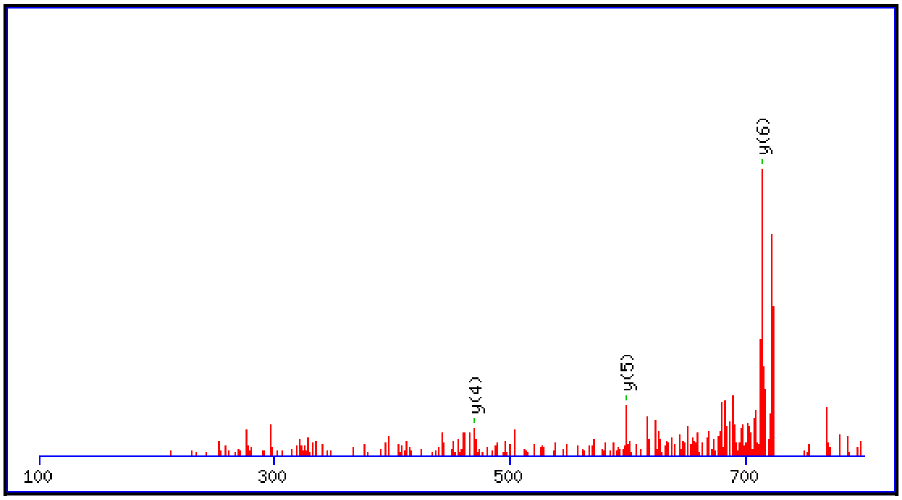

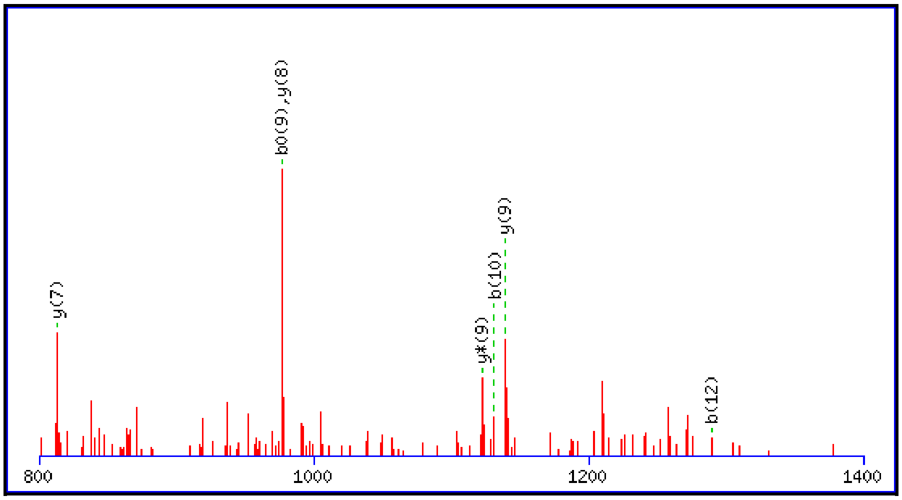

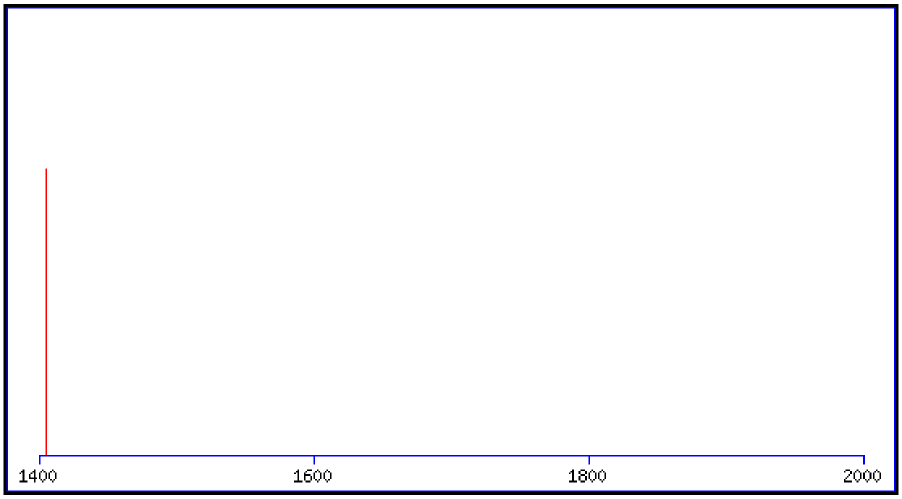

Monoisotopic mass of neutral peptide Mr(calc): 1462.6841
Fixed modifications: Carbamidomethyl (C)
Ions Score: 31 **Expect:** 0.15
Matches (Bold Red): 10/104 fragment ions using 37 most intense peaks

#	b	b^{++}	b^{0}	b^{0++}	Seq.	y	y^{++}	y*	y*$^{++}$	y^{0}	y^{0++}	#
1	58.0287	29.5180			G							13
2	155.0815	78.0444			P	1406.6699	703.8386	1389.6434	695.3253	1388.6593	694.8333	12
3	212.1030	106.5551			G	1309.6171	655.3122	1292.5906	646.7989	1291.6066	646.3069	11
4	325.1870	163.0972			L	1252.5957	626.8015	1235.5691	618.2882	1234.5851	617.7962	10
5	488.2504	244.6288			Y	**1139.5116**	570.2594	**1122.4851**	561.7462	1121.5010	561.2542	9
6	651.3137	326.1605			Y	**976.4483**	488.7278	959.4217	480.2145	958.4377	479.7225	8
7	750.3821	375.6947			V	**813.3850**	407.1961	796.3584	398.6828	795.3744	398.1908	7
8	865.4090	433.2082	847.3985	424.2029	D	**714.3165**	357.6619	697.2900	349.1486	696.3060	348.6566	6
9	994.4516	497.7295	**976.4411**	488.7242	E	**599.2896**	300.1484	582.2631	291.6352	581.2790	291.1432	5
10	**1131.5106**	566.2589	1113.5000	557.2536	H	**470.2470**	235.6271	453.2205	227.1139	452.2364	226.6219	4
11	1188.5320	594.7696	1170.5215	585.7644	G	333.1881	167.0977	316.1615	158.5844	315.1775	158.0924	3
12	**1289.5797**	645.2935	1271.5691	636.2882	T	276.1666	138.5870	259.1401	130.0737	258.1561	129.5817	2
13					R	175.1190	88.0631	158.0924	79.5498			1

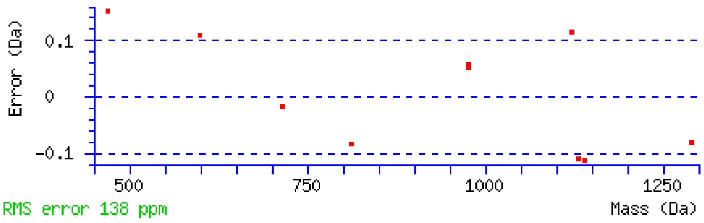

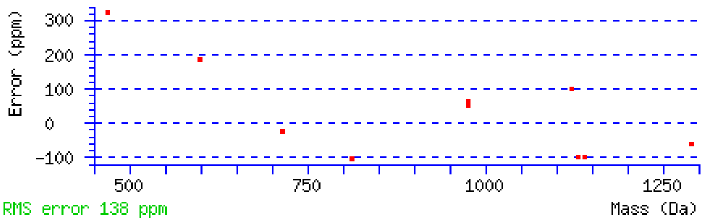

All matches to this query

Score	Mr(calc):	Delta	Sequence
31.4	1462.6841	0.0068	GPGLYYVDEHGTR
1.3	1461.6881	1.0028	VSGIKSKVDELK
0.3	1462.6986	-0.0077	LLTPAQRTLYK

Spectrum No: 133; Query: 11817; Rank: 1

Peptide View

MS/MS Fragmentation of **LLSNMMCQYR**
Found in **IPI00000783**, Tax_Id=9606 Gene_Symbol=PSMB8 Isoform 1 of Proteasome subunit beta type-8

Match to Query 11817: 1314.596188 from(658.305370,2+)
Title: 091224LimSK_Exosome3_06.3302.3302.2.dta
Data file C:\Temp\Mascoti\LSK_2D\091224LimSK_Exosome3_01_2D.mgf

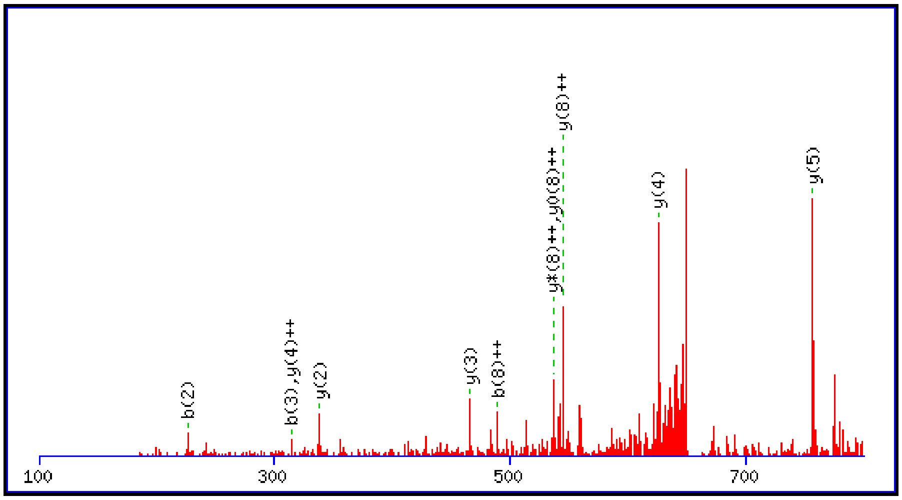

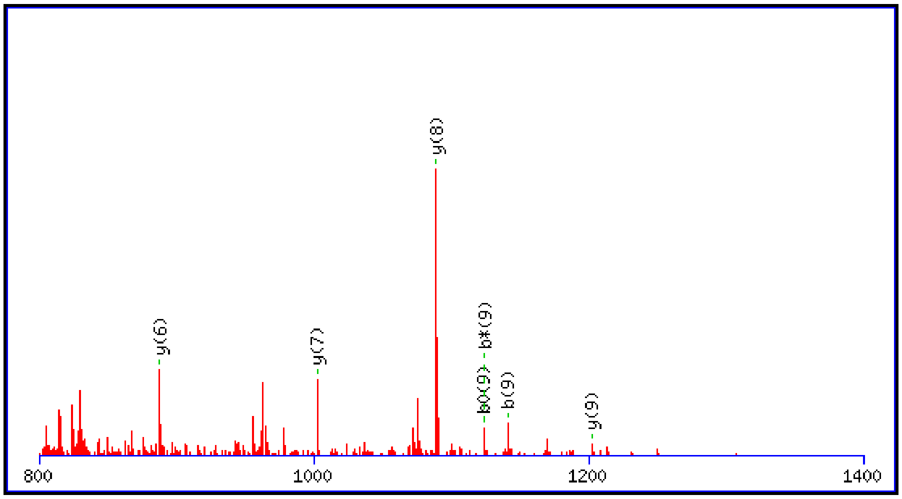

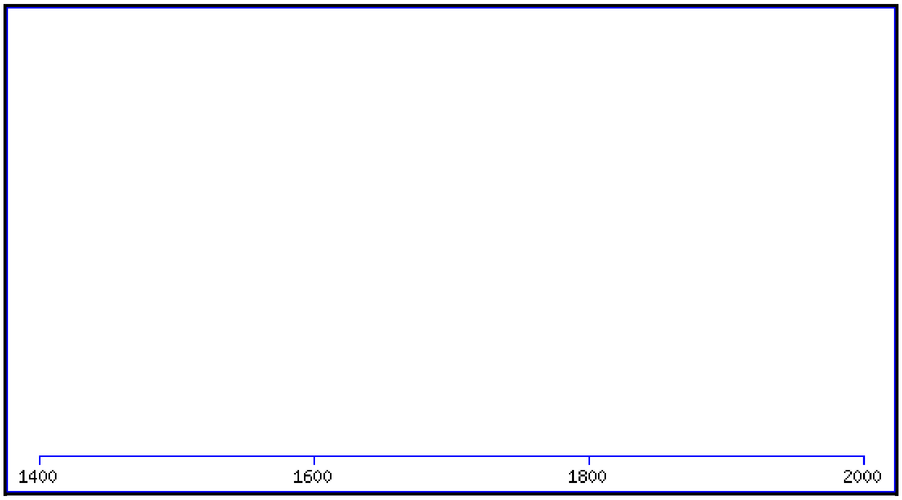

Monoisotopic mass of neutral peptide Mr(calc): 1314.5883
Fixed modifications: Carbamidomethyl (C)
Ions Score: 54 **Expect:** 0.00073
Matches (Bold Red): 18/84 fragment ions using 37 most intense peaks

#	b	b^{++}	b*	b^{*++}	b^0	b^{0++}	Seq.	y	y^{++}	y*	y^{*++}	y^0	y^{0++}	#
1	114.0913	57.5493					L							10
2	**227.1754**	114.0913					L	**1202.5115**	601.7594	1185.4849	593.2461	1184.5009	592.7541	9
3	**314.2074**	157.6074			296.1969	148.6021	S	**1089.4274**	**545.2174**	1072.4009	**536.7041**	1071.4169	**536.2121**	8
4	428.2504	214.6288	411.2238	206.1155	410.2398	205.6235	N	**1002.3954**	501.7013	985.3689	493.1881			7
5	559.2908	280.1491	542.2643	271.6358	541.2803	271.1438	M	**888.3525**	444.6799	871.3259	436.1666			6
6	690.3313	345.6693	673.3048	337.1560	672.3208	336.6640	M	**757.3120**	379.1596	740.2854	370.6464			5
7	850.3620	425.6846	833.3354	417.1714	832.3514	416.6793	C	**626.2715**	**313.6394**	609.2450	305.1261			4
8	978.4206	**489.7139**	961.3940	481.2006	960.4100	480.7086	Q	**466.2409**	233.6241	449.2143	225.1108			3
9	**1141.4839**	571.2456	**1124.4573**	562.7323	**1123.4733**	562.2403	Y	**338.1823**	169.5948	321.1557	161.0815			2
10							R	175.1190	88.0631	158.0924	79.5498			1

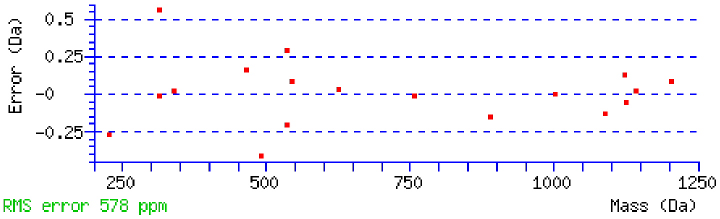

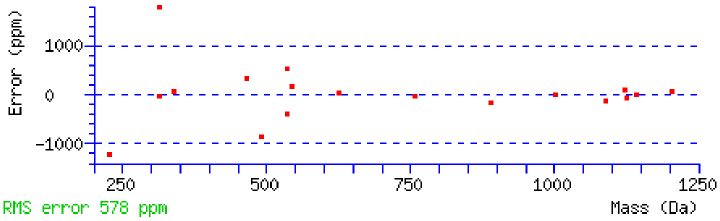

All matches to this query

Score	Mr(calc):	Delta	Sequence
53.9	1314.5883	0.0079	LLSNMMCQYR
7.2	1314.6003	-0.0041	LLSLSCSVNSR
7.2	1314.6003	-0.0041	LLSLSCSVNSR
6.9	1314.6003	-0.0041	LLSLSCSVNSR
6.5	1314.5846	0.0115	LLSGPGATQRR
6.0	1314.6026	-0.0064	KLSYVIAFSK
5.8	1312.5942	2.0020	XSSNDKHVMSR
5.8	1314.5914	0.0048	EPLETTCFYR
4.8	1314.5891	0.0071	LLASCSADGTLK
4.8	1314.5891	0.0071	LLASCSADGTLK

Spectrum No: 134; Query: 73733; Rank: 1

Peptide View

MS/MS Fragmentation of **LSGNMFSTGSGNTYAYGVMDSGYRPNLSPEEAYDLGR**
Found in **IPI00000783**, Tax_Id=9606 Gene_Symbol=PSMB8 Isoform 1 of Proteasome subunit beta type-8

Match to Query 73733: 3976.801662 from(1326.607830,3+)
Title: 091224LimSK_Exosome3_06.7068.7068.3.dta
Data file C:\Temp\Mascoti\LSK_2D\091224LimSK_Exosome3_01_2D.mgf

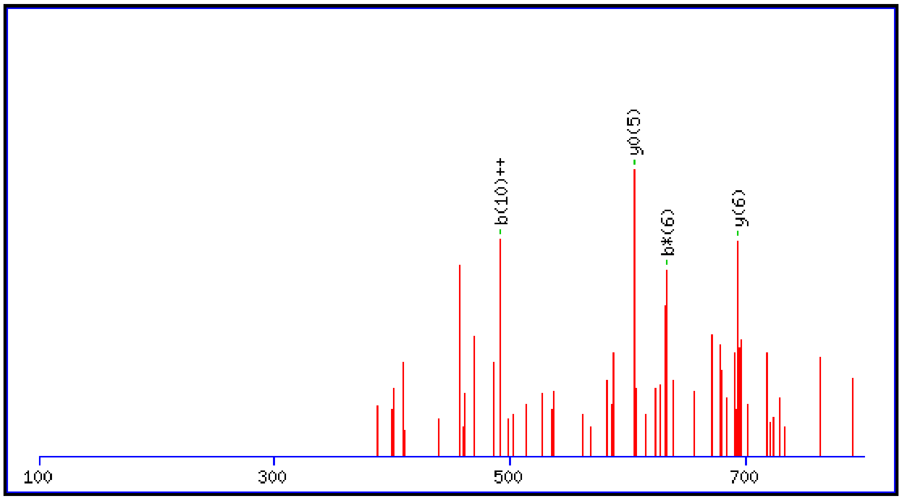

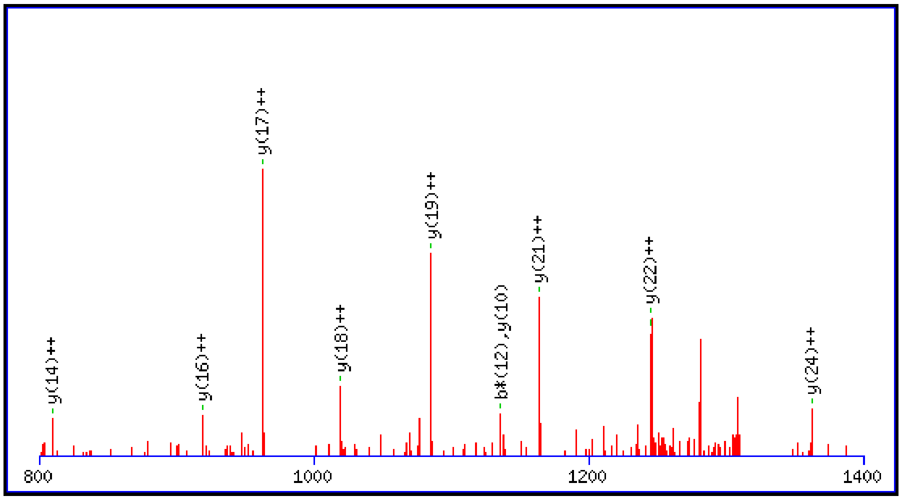

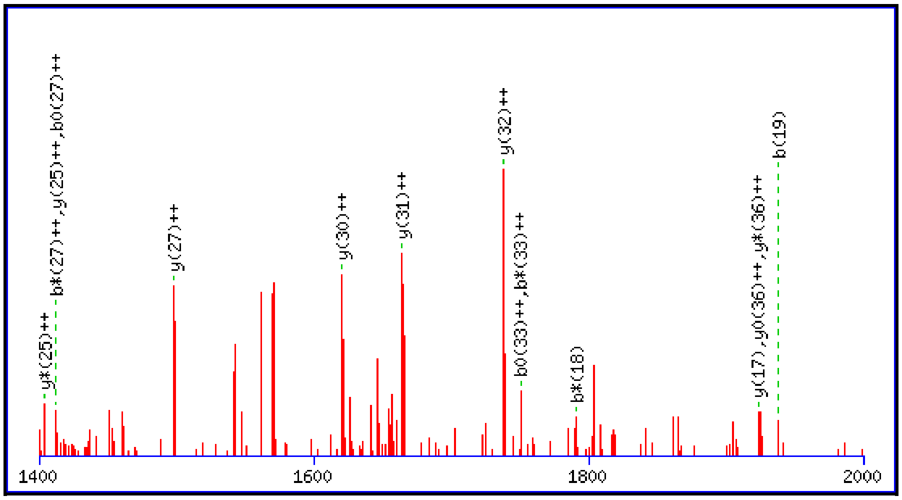

Monoisotopic mass of neutral peptide Mr(calc): 3975.7680
Fixed modifications: Carbamidomethyl (C)
Ions Score: 67 **Expect:** 8.5e-005

Matches (Bold Red): 29/418 fragment ions using 43 most intense peaks

#	b	b^{++}	b*	b^{*++}	b^0	b^{0++}	Seq.	y	y^{++}	y*	y^{*++}	y^0	y^{0++}	#
1	114.0913	57.5493					L							37
2	201.1234	101.0653			183.1128	92.0600	S	3863.6912	1932.3492	3846.6646	**1923.8360**	3845.6806	**1923.3440**	36
3	258.1448	129.5761			240.1343	120.5708	G	3776.6592	1888.8332	3759.6326	1880.3199	3758.6486	1879.8279	35
4	372.1878	186.5975	355.1612	178.0842	354.1772	177.5922	N	3719.6377	1860.3225	3702.6112	1851.8092	3701.6271	1851.3172	34
5	503.2282	252.1178	486.2017	243.6045	485.2177	243.1125	M	3605.5948	1803.3010	3588.5682	1794.7878	3587.5842	1794.2957	33
6	650.2967	325.6520	**633.2701**	317.1387	632.2861	316.6467	F	3474.5543	**1737.7808**	3457.5277	1729.2675	3456.5437	1728.7755	32
7	737.3287	369.1680	720.3021	360.6547	719.3181	360.1627	S	3327.4859	**1664.2466**	3310.4593	1655.7333	3309.4753	1655.2413	31
8	838.3764	419.6918	821.3498	411.1785	820.3658	410.6865	T	3240.4538	**1620.7306**	3223.4273	1612.2173	3222.4433	1611.7253	30
9	895.3978	448.2026	878.3713	439.6893	877.3873	439.1973	G	3139.4062	1570.2067	3122.3796	1561.6934	3121.3956	1561.2014	29
10	982.4299	**491.7186**	965.4033	483.2053	964.4193	482.7133	S	3082.3847	1541.6960	3065.3582	1533.1827	3064.3741	1532.6907	28
11	1039.4513	520.2293	1022.4248	511.7160	1021.4408	511.2240	G	2995.3527	**1498.1800**	2978.3261	1489.6667	2977.3421	1489.1747	27
12	1153.4942	577.2508	**1136.4677**	568.7375	1135.4837	568.2455	N	2938.3312	1469.6692	2921.3047	1461.1560	2920.3206	1460.6640	26
13	1254.5419	627.7746	1237.5154	619.2613	1236.5314	618.7693	T	2824.2883	**1412.6478**	2807.2617	**1404.1345**	2806.2777	1403.6425	25
14	1417.6053	709.3063	1400.5787	700.7930	1399.5947	700.3010	Y	2723.2406	**1362.1239**	2706.2141	1353.6107	2705.2300	1353.1187	24
15	1488.6424	744.8248	1471.6158	736.3115	1470.6318	735.8195	A	2560.1773	1280.5923	2543.1507	1272.0790	2542.1667	1271.5870	23
16	1651.7057	826.3565	1634.6792	817.8432	1633.6951	817.3512	Y	2489.1402	**1245.0737**	2472.1136	1236.5604	2471.1296	1236.0684	22
17	1708.7272	854.8672	1691.7006	846.3539	1690.7166	845.8619	G	2326.0768	**1163.5421**	2309.0503	1155.0288	2308.0663	1154.5368	21
18	1807.7956	904.4014	**1790.7690**	895.8882	1789.7850	895.3961	V	2269.0554	1135.0313	2252.0288	1126.5180	2251.0448	1126.0260	20
19	**1938.8361**	969.9217	1921.8095	961.4084	1920.8255	960.9164	M	2169.9870	**1085.4971**	2152.9604	1076.9838	2151.9764	1076.4918	19
20	2053.8630	1027.4351	2036.8365	1018.9219	2035.8524	1018.4299	D	2038.9465	**1019.9769**	2021.9199	1011.4636	2020.9359	1010.9716	18
21	2140.8950	1070.9512	2123.8685	1062.4379	2122.8845	1061.9459	S	**1923.9195**	**962.4634**	1906.8930	953.9501	1905.9090	953.4581	17
22	2197.9165	1099.4619	2180.8899	1090.9486	2179.9059	1090.4566	G	1836.8875	**918.9474**	1819.8610	910.4341	1818.8769	909.9421	16
23	2360.9798	1180.9936	2343.9533	1172.4803	2342.9693	1171.9883	Y	1779.8660	890.4367	1762.8395	881.9234	1761.8555	881.4314	15
24	2517.0809	1259.0441	2500.0544	1250.5308	2499.0704	1250.0388	R	1616.8027	**808.9050**	1599.7762	800.3917	1598.7921	799.8997	14
25	2614.1337	1307.5705	2597.1072	1299.0572	2596.1231	1298.5652	P	1460.7016	730.8544	1443.6751	722.3412	1442.6910	721.8492	13
26	2728.1766	1364.5920	2711.1501	1356.0787	2710.1661	1355.5867	N	1363.6488	682.3281	1346.6223	673.8148	1345.6383	673.3228	12
27	2841.2607	1421.1340	2824.2341	**1412.6207**	2823.2501	**1412.1287**	L	1249.6059	625.3066	1232.5794	616.7933	1231.5953	616.3013	11
28	2928.2927	1464.6500	2911.2662	1456.1367	2910.2822	1455.6447	S	**1136.5218**	568.7646	1119.4953	560.2513	1118.5113	559.7593	10
29	3025.3455	1513.1764	3008.3189	1504.6631	3007.3349	1504.1711	P	1049.4898	525.2485	1032.4633	516.7353	1031.4793	516.2433	9
30	3154.3881	1577.6977	3137.3615	1569.1844	3136.3775	1568.6924	E	952.4371	476.7222	935.4105	468.2089	934.4265	467.7169	8
31	3283.4307	1642.2190	3266.4041	1633.7057	3265.4201	1633.2137	E	823.3945	412.2009	806.3679	403.6876	805.3839	403.1956	7
32	3354.4678	1677.7375	3337.4412	1669.2243	3336.4572	1668.7322	A	**694.3519**	347.6796	677.3253	339.1663	676.3413	338.6743	6
33	3517.5311	1759.2692	3500.5046	**1750.7559**	3499.5205	**1750.2639**	Y	623.3148	312.1610	606.2882	303.6477	**605.3042**	303.1557	5
34	3632.5581	1816.7827	3615.5315	1808.2694	3614.5475	1807.7774	D	460.2514	230.6293	443.2249	222.1161	442.2409	221.6241	4
35	3745.6421	1873.3247	3728.6156	1864.8114	3727.6316	1864.3194	L	345.2245	173.1159	328.1979	164.6026			3
36	3802.6636	1901.8354	3785.6370	1893.3222	3784.6530	1892.8301	G	232.1404	116.5738	215.1139	108.0606			2
37							R	175.1190	88.0631	158.0924	79.5498			1

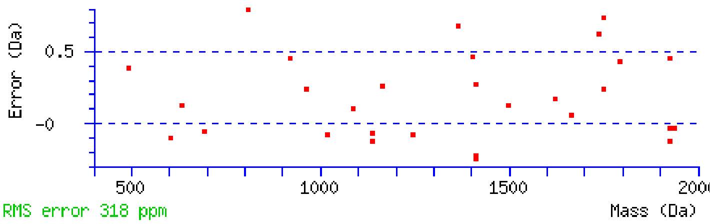

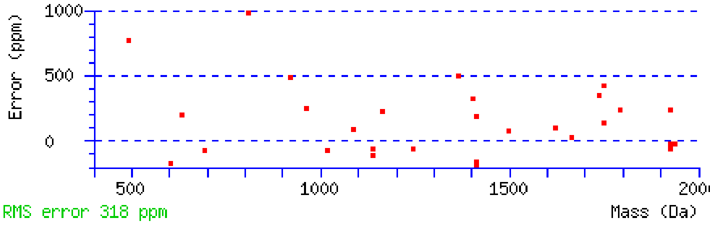

All matches to this query

Score	Mr(calc):	Delta	Sequence

66.5	3975.7680	1.0337	LSGNMFSTGSGNTYAYGVMDSGYRPNLSPEEAYDLGR
12.7	3975.7673	1.0343	STPYECGFDPMSPARVPFSMKFFLVAMPSYYLI
12.7	3975.7673	1.0343	STPYECGFDPMSPARVPFSMKFFLVAMPSYYLI
9.6	3975.7603	1.0413	MVAMQEAAQHLLGTHDFSAFQSAGSPVPSPVRTLR
8.5	3976.8238	-0.0221	MSACHTQPSLGGGGSAGSGLGEAAWAGLPPDTGSFLLQVR
8.5	3976.8238	-0.0221	MSACHTQPSLGGGGSAGSGLGEAAWAGLPPDTGSFLLQVR
8.5	3976.8238	-0.0221	MSACHTQPSLGGGGSAGSGLGEAAWAGLPPDTGSFLLQVR
8.3	3975.7673	1.0343	STPYECGFDPMSPARVPFSMKFFLVAMPSYYLI
8.3	3974.7676	2.0341	GNVAGDSKNDPPMEAAGFTAQVIILNHPGQISQKSK
7.9	3974.7679	2.0337	XPTIPAVANSSANSSPSELADELPDMTLDKISGRER

Spectrum No: 135; Query: 28355; Rank: 1

Peptide View

MS/MS Fragmentation of **VESTDVSDLLHQYR**
Found in **IPI00000783**, Tax_Id=9606 Gene_Symbol=PSMB8 Isoform 1 of Proteasome subunit beta type-8

Match to Query 28355: 1660.813872 from(554.611900,3+)
Title: 091224LimSK_Exosome3_06.4600.4600.3.dta
Data file C:\Temp\Mascoti\LSK_2D\091224LimSK_Exosome3_01_2D.mgf

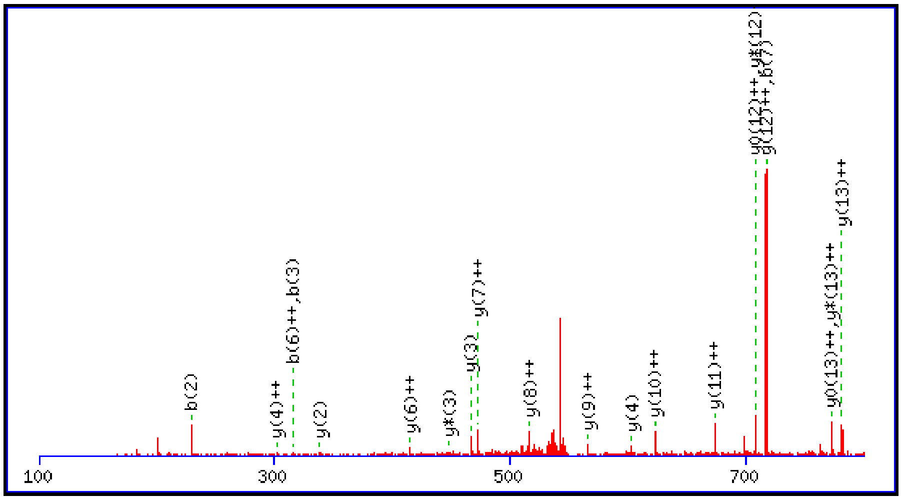

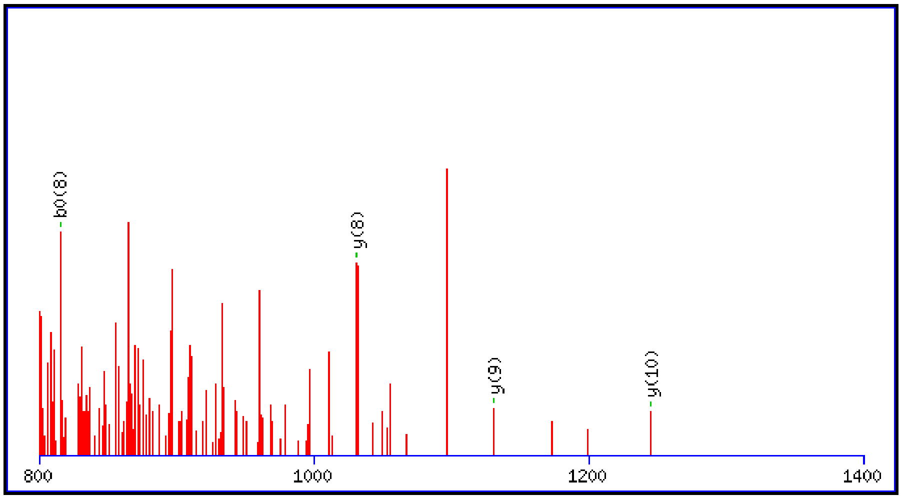

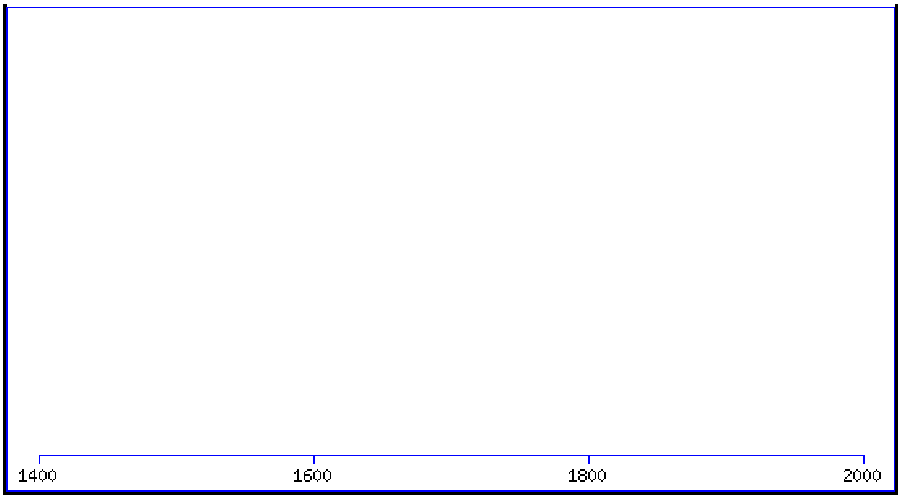

Monoisotopic mass of neutral peptide Mr(calc): 1660.8057
Fixed modifications: Carbamidomethyl (C)
Ions Score: 50 **Expect:** 0.0024
Matches (Bold Red): 25/120 fragment ions using 57 most intense peaks

#	b	b^{++}	b*	b^{*++}	b^0	b^{0++}	Seq.	y	y^{++}	y*	y^{*++}	y^0	y^{0++}	#
1	100.0757	50.5415					V							14
2	**229.1183**	115.0628			211.1077	106.0575	E	1562.7445	**781.8759**	1545.7180	**773.3626**	1544.7340	**772.8706**	13
3	**316.1503**	158.5788			298.1397	149.5735	S	1433.7019	**717.3546**	1416.6754	**708.8413**	1415.6914	**708.3493**	12
4	417.1980	209.1026			399.1874	200.0974	T	1346.6699	**673.8386**	1329.6434	665.3253	1328.6593	664.8333	11
5	532.2249	266.6161			514.2144	257.6108	D	**1245.6222**	**623.3148**	1228.5957	614.8015	1227.6117	614.3095	10
6	631.2933	**316.1503**			613.2828	307.1450	V	**1130.5953**	**565.8013**	1113.5687	557.2880	1112.5847	556.7960	9
7	**718.3254**	359.6663			700.3148	350.6610	S	**1031.5269**	**516.2671**	1014.5003	507.7538	1013.5163	507.2618	8
8	833.3523	417.1798			**815.3418**	408.1745	D	944.4948	**472.7511**	927.4683	464.2378	926.4843	463.7458	7
9	946.4364	473.7218			928.4258	464.7165	L	829.4679	**415.2376**	812.4413	406.7243			6
10	1059.5204	530.2639			1041.5099	521.2586	L	716.3838	358.6956	699.3573	350.1823			5
11	1196.5794	598.7933			1178.5688	589.7880	H	**603.2998**	**302.1535**	586.2732	293.6402			4
12	1324.6379	662.8226	1307.6114	654.3093	1306.6274	653.8173	Q	**466.2409**	233.6241	**449.2143**	225.1108			3
13	1487.7013	744.3543	1470.6747	735.8410	1469.6907	735.3490	Y	**338.1823**	169.5948	321.1557	161.0815			2
14							R	175.1190	88.0631	158.0924	79.5498			1

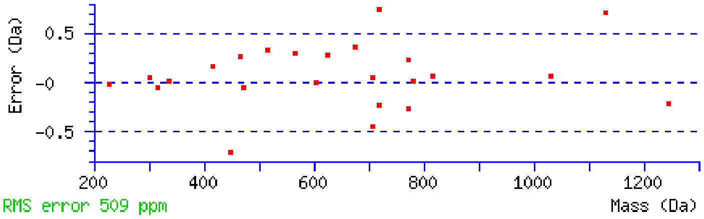

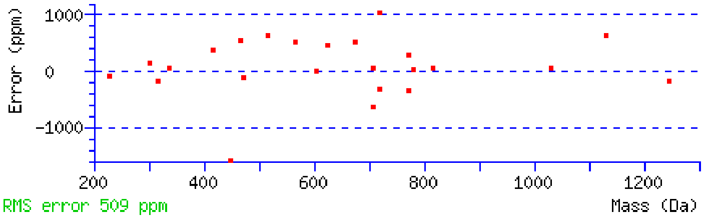

All matches to this query

Score	Mr(calc):	Delta	Sequence
49.6	1660.8057	0.0082	VESTDVSDLLHQYR
11.1	1658.8215	1.9924	QLFERLKMSLGNK
10.6	1659.8241	0.9897	KFTMLALRDLGMGK
10.3	1659.7981	1.0158	ENNTVSNIHPKLSK
10.3	1660.8298	-0.0159	EVSVRHSQTILNAK
8.9	1659.7981	1.0157	TRADSVANKLGSFSK
8.9	1659.7981	1.0157	TRADSVANKLGSFSK
8.6	1658.8103	2.0036	TGGMKLSATVIFNPK

7.0	1660.8298	-0.0159	EVSVRHSQTILNAK
6.8	1658.7916	2.0222	KGSEQESVKEFLAK

Spectrum No: 136; Query: 41347; Rank: 1

Peptide View

MS/MS Fragmentation of **LPFTALGSGQDAALAVLEDR**
Found in **IPI00027933**, Tax_Id=9606 Gene_Symbol=PSMB10 Proteasome subunit beta type-10

Match to Query 41347: 2043.080748 from(1022.547650,2+)
Title: 091224LimSK_Exosome3_06.7779.7779.2.dta
Data file C:\Temp\Mascoti\LSK_2D\091224LimSK_Exosome3_01_2D.mgf

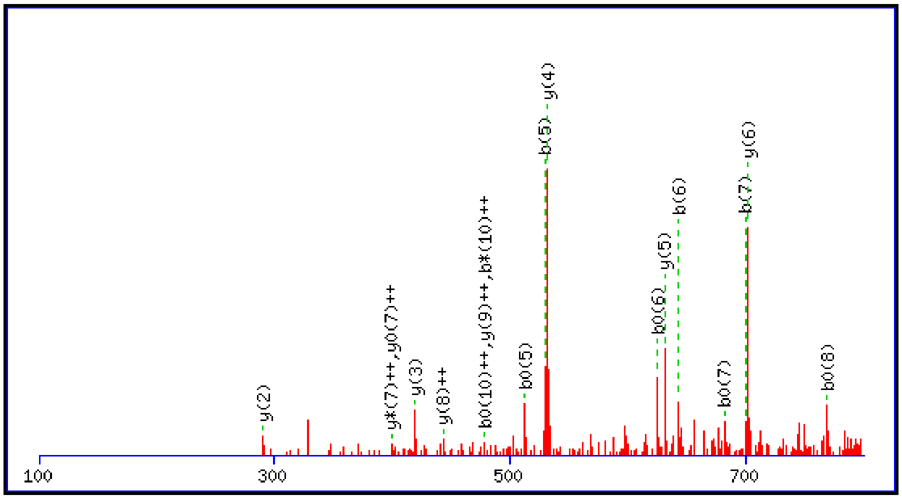

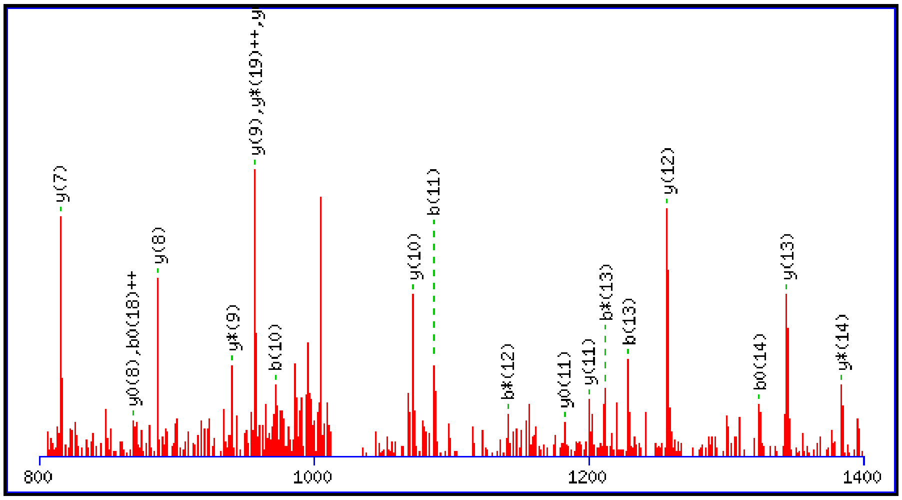

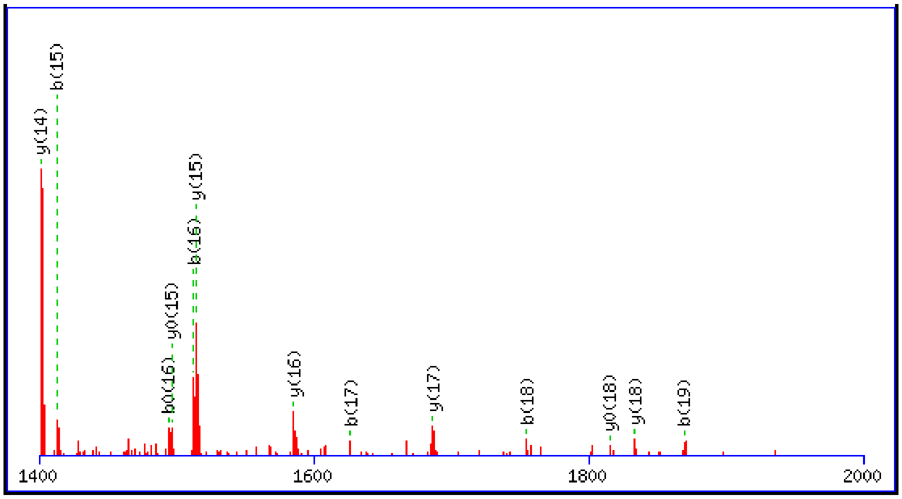

Monoisotopic mass of neutral peptide Mr(calc): 2043.0637
Fixed modifications: Carbamidomethyl (C)
Ions Score: 113 **Expect:** 6e-010
Matches (Bold Red): 51/202 fragment ions using 92 most intense peaks

#	b	b^{++}	b*	b^{*++}	b^{0}	b^{0++}	Seq.	y	y^{++}	y*	y^{*++}	y^{0}	y^{0++}	#
1	114.0913	57.5493					L							20
2	211.1441	106.0757					P	1930.9869	965.9971	1913.9603	**957.4838**	1912.9763	**956.9918**	19
3	358.2125	179.6099					F	**1833.9341**	917.4707	1816.9076	908.9574	**1815.9236**	908.4654	18
4	459.2602	230.1337			441.2496	221.1285	T	**1686.8657**	843.9365	1669.8392	835.4232	1668.8551	834.9312	17
5	**530.2973**	265.6523			**512.2867**	256.6470	A	**1585.8180**	793.4127	1568.7915	784.8994	1567.8075	784.4074	16
6	**643.3814**	322.1943			**625.3708**	313.1890	L	**1514.7809**	757.8941	1497.7544	749.3808	**1496.7703**	748.8888	15
7	**700.4028**	350.7051			**682.3923**	341.6998	G	**1401.6968**	701.3521	**1384.6703**	692.8388	1383.6863	692.3468	14
8	787.4349	394.2211			**769.4243**	385.2158	S	**1344.6754**	672.8413	1327.6488	664.3281	1326.6648	663.8360	13
9	844.4563	422.7318			826.4458	413.7265	G	**1257.6434**	629.3253	1240.6168	620.8120	1239.6328	620.3200	12
10	**972.5149**	486.7611	955.4884	**478.2478**	954.5043	**477.7558**	Q	**1200.6219**	600.8146	1183.5953	592.3013	**1182.6113**	591.8093	11
11	**1087.5419**	544.2746	1070.5153	535.7613	1069.5313	535.2693	D	**1072.5633**	536.7853	1055.5368	528.2720	1054.5528	527.7800	10
12	1158.5790	579.7931	**1141.5524**	571.2798	1140.5684	570.7878	A	**957.5364**	**479.2718**	**940.5098**	470.7585	939.5258	470.2665	9
13	**1229.6161**	615.3117	**1212.5895**	606.7984	1211.6055	606.3064	A	**886.4993**	**443.7533**	869.4727	435.2400	**868.4887**	434.7480	8
14	1342.7001	671.8537	1325.6736	663.3404	**1324.6896**	662.8484	L	**815.4621**	408.2347	798.4356	**399.7214**	797.4516	**399.2294**	7
15	**1413.7373**	707.3723	1396.7107	698.8590	1395.7267	698.3670	A	**702.3781**	351.6927	685.3515	343.1794	684.3675	342.6874	6
16	**1512.8057**	756.9065	1495.7791	748.3932	**1494.7951**	747.9012	V	**631.3410**	316.1741	614.3144	307.6608	613.3304	307.1688	5
17	**1625.8897**	813.4485	1608.8632	804.9352	1607.8792	804.4432	L	**532.2726**	266.6399	515.2460	258.1266	514.2620	257.6346	4
18	**1754.9323**	877.9698	1737.9058	869.4565	1736.9218	**868.9645**	E	**419.1885**	210.0979	402.1619	201.5846	401.1779	201.0926	3
19	**1869.9593**	935.4833	1852.9327	926.9700	1851.9487	926.4780	D	**290.1459**	145.5766	273.1193	137.0633	272.1353	136.5713	2
20							R	175.1190	88.0631	158.0924	79.5498			1

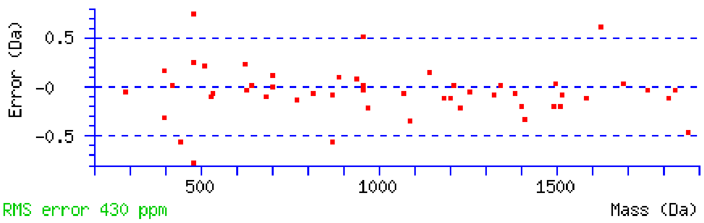

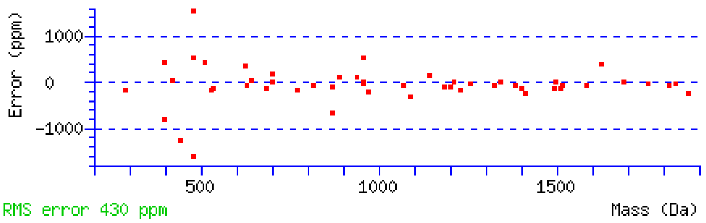

All matches to this query

Score	Mr(calc):	Delta	Sequence
113.0	2043.0637	0.0171	LPFTALGSGQDAALAVLEDR
5.3	2043.0666	0.0141	LNAVYLNSSSKRVIFPR

0.8	2042.0579	1.0229	LEGVCERLDTVAGGLQGLR

Spectrum No: 137; Query: 25774; Rank: 1

Peptide View

MS/MS Fragmentation of **GAEILEVLHSLPAVR**
Found in **IPI00014151**, Tax_Id=9606 Gene_Symbol=PSMD6 26S proteasome non-ATPase regulatory subunit 6

Match to Query 25774: 1602.918702 from(535.313510,3+)
Title: 091224LimSK_Exosome3_04.7281.7281.3.dta
Data file C:\Temp\Mascoti\LSK_2D\091224LimSK_Exosome3_01_2D.mgf

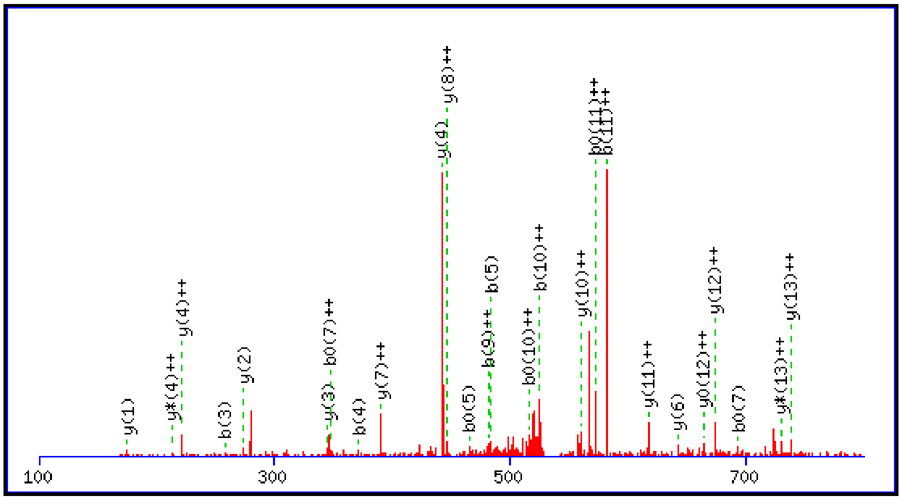

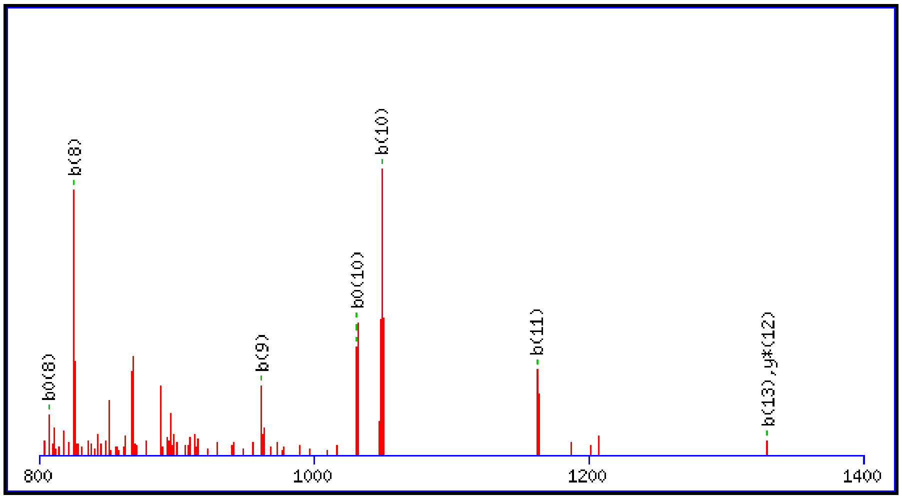

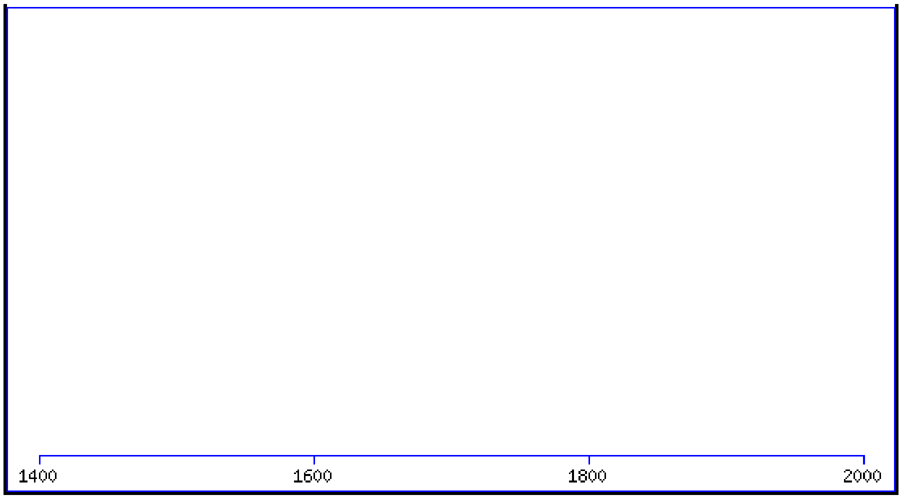

Monoisotopic mass of neutral peptide Mr(calc): 1602.9093
Fixed modifications: Carbamidomethyl (C)
Ions Score: 32 **Expect:** 0.026
Matches (Bold Red): 34/126 fragment ions using 81 most intense peaks

#	b	b^{++}	b^{0}	b^{0++}	Seq.	y	y^{++}	y*	y^{*++}	y^{0}	y^{0++}	#
1	58.0287	29.5180			G							15
2	129.0659	65.0366			A	1546.8952	773.9512	1529.8686	765.4379	1528.8846	764.9459	14
3	**258.1084**	129.5579	240.0979	120.5526	E	1475.8580	**738.4327**	1458.8315	**729.9194**	1457.8475	729.4274	13
4	**371.1925**	186.0999	353.1819	177.0946	I	1346.8154	**673.9114**	**1329.7889**	665.3981	1328.8049	**664.9061**	12
5	**484.2766**	242.6419	**466.2660**	233.6366	L	1233.7314	**617.3693**	1216.7048	608.8561	1215.7208	608.3640	11
6	613.3192	307.1632	595.3086	298.1579	E	1120.6473	**560.8273**	1103.6208	552.3140	1102.6368	551.8220	10
7	712.3876	356.6974	**694.3770**	**347.6921**	V	991.6047	496.3060	974.5782	487.7927	973.5942	487.3007	9
8	**825.4716**	413.2395	**807.4611**	404.2342	L	892.5363	**446.7718**	875.5098	438.2585	874.5257	437.7665	8
9	**962.5306**	**481.7689**	944.5200	472.7636	H	779.4522	**390.2298**	762.4257	381.7165	761.4417	381.2245	7
10	**1049.5626**	**525.2849**	**1031.5520**	**516.2796**	S	**642.3933**	321.7003	625.3668	313.1870	624.3828	312.6950	6
11	**1162.6466**	**581.8270**	1144.6361	**572.8217**	L	555.3613	278.1843	538.3348	269.6710			5
12	1259.6994	630.3533	1241.6888	621.3481	P	**442.2772**	**221.6423**	425.2507	**213.1290**			4
13	**1330.7365**	665.8719	1312.7260	656.8666	A	**345.2245**	173.1159	328.1979	164.6026			3
14	1429.8049	715.4061	1411.7944	706.4008	V	**274.1874**	137.5973	257.1608	129.0840			2
15					R	**175.1190**	88.0631	158.0924	79.5498			1

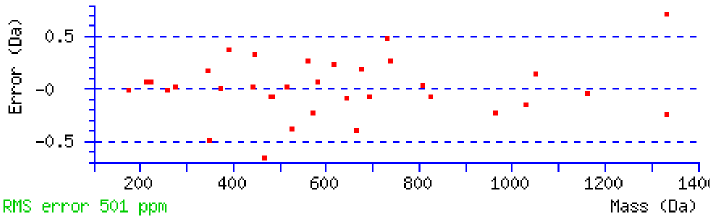

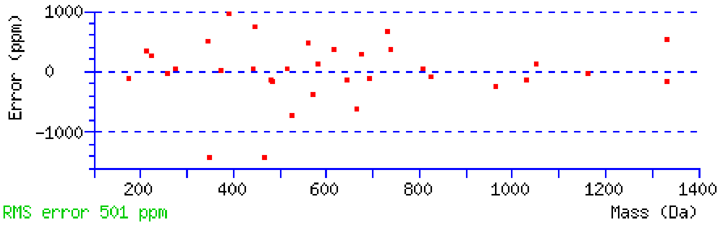

All matches to this query

Score	Mr(calc):	Delta	Sequence
32.2	1602.9093	0.0094	GAEILEVLHSLPAVR
5.8	1601.9242	0.9945	TLRALRPLRALSR
0.7	1601.9253	0.9934	RVVSVYQAALDLLR
0.5	1602.9205	-0.0018	GALLSYRRNDLVVK

Spectrum No: 138; Query: 13733; Rank: 1

Peptide View

MS/MS Fragmentation of **PLENLEEEGLPK**
Found in **IPI00014151**, Tax_Id=9606 Gene_Symbol=PSMD6 26S proteasome non-ATPase regulatory subunit 6

Match to Query 13733: 1366.707948 from(684.361250,2+)
Title: 091224LimSK_Exosome3_04.3023.3023.2.dta
Data file C:\Temp\Mascoti\LSK_2D\091224LimSK_Exosome3_01_2D.mgf

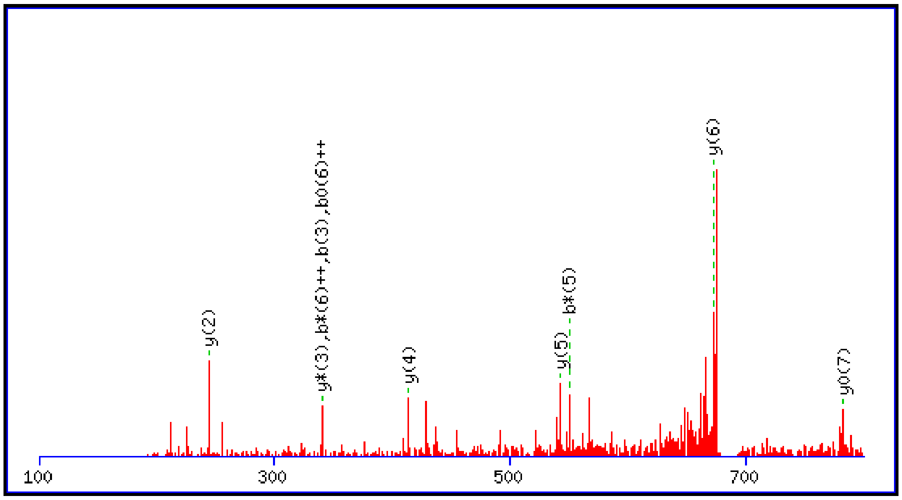

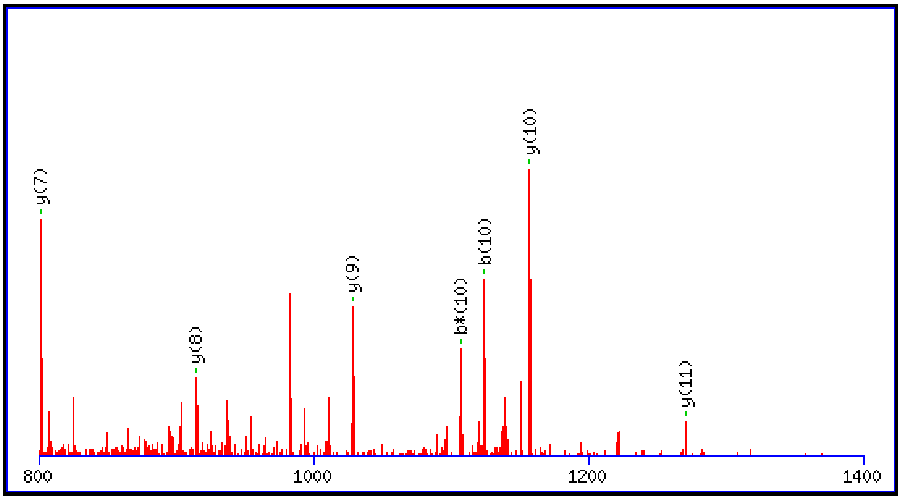

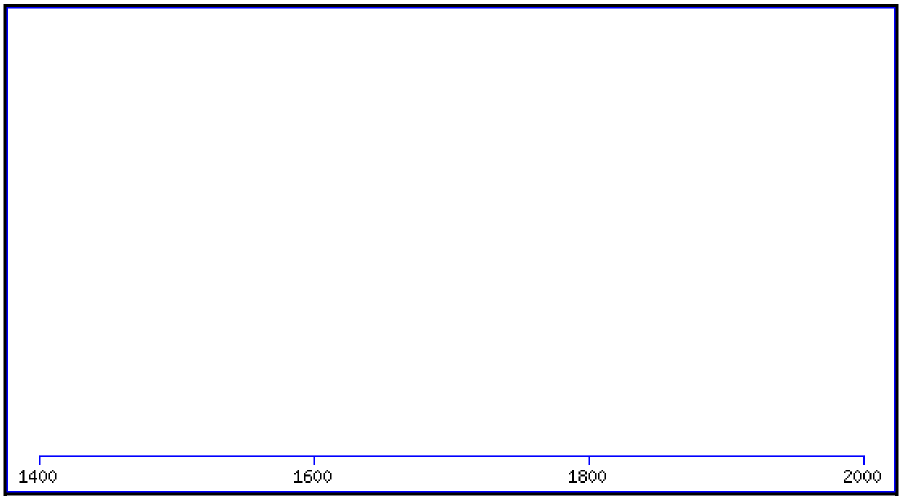

Monoisotopic mass of neutral peptide Mr(calc): 1366.6980
Fixed modifications: Carbamidomethyl (C)
Ions Score: 63 **Expect:** 6.5e-005
Matches (Bold Red): 17/114 fragment ions using 32 most intense peaks

#	b	b^{++}	b*	b^{*++}	b^0	b^{0++}	Seq.	y	y^{++}	y*	y^{*++}	y^0	y^{0++}	#
1	98.0600	49.5337					P							12
2	211.1441	106.0757					L	**1270.6525**	635.8299	1253.6260	627.3166	1252.6420	626.8246	11
3	**340.1867**	170.5970			322.1761	161.5917	E	**1157.5685**	579.2879	1140.5419	570.7746	1139.5579	570.2826	10
4	454.2296	227.6185	437.2031	219.1052	436.2191	218.6132	N	**1028.5259**	514.7666	1011.4993	506.2533	1010.5153	505.7613	9
5	567.3137	284.1605	**550.2871**	275.6472	549.3031	275.1552	L	**914.4829**	457.7451	897.4564	449.2318	896.4724	448.7398	8
6	696.3563	348.6818	679.3297	**340.1685**	678.3457	**339.6765**	E	**801.3989**	401.2031	784.3723	392.6898	**783.3883**	392.1978	7
7	825.3989	413.2031	808.3723	404.6898	807.3883	404.1978	E	**672.3563**	336.6818	655.3297	328.1685	654.3457	327.6765	6
8	954.4415	477.7244	937.4149	469.2111	936.4309	468.7191	E	**543.3137**	272.1605	526.2871	263.6472	525.3031	263.1552	5
9	1011.4629	506.2351	994.4364	497.7218	993.4524	497.2298	G	**414.2711**	207.6392	397.2445	199.1259			4
10	**1124.5470**	562.7771	**1107.5204**	554.2639	1106.5364	553.7719	L	357.2496	179.1285	**340.2231**	170.6152			3
11	1221.5998	611.3035	1204.5732	602.7902	1203.5892	602.2982	P	**244.1656**	122.5864	227.1390	114.0731			2
12							K	147.1128	74.0600	130.0863	65.5468			1

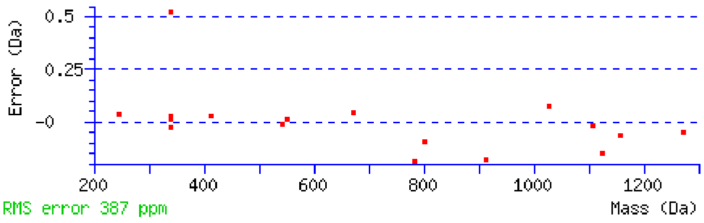

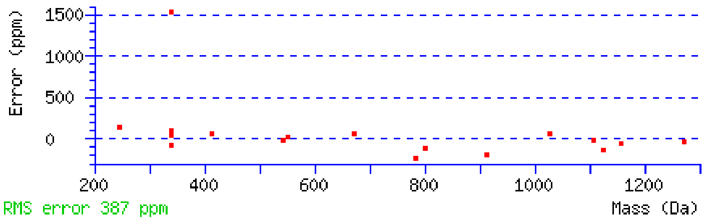

All matches to this query

Score	Mr(calc):	Delta	Sequence
63.5	1366.6980	0.0100	PLENLEEEGLPK
4.5	1365.6929	1.0151	GSSYLGIPFNPSK
4.2	1365.7170	0.9910	RSPLLKSGPFGK
2.5	1366.7156	-0.0076	RSPVLIAKTMR
1.9	1365.7113	0.9966	GLADHVLQDRSR
1.4	1366.6969	0.0110	RASVTERLDLK
1.2	1366.7043	0.0036	ENILVMRLATK

Spectrum No: 139; Query: 12962; Rank: 1

Peptide View

MS/MS Fragmentation of **RLDEELEDAEK**
Found in **IPI00014151**, Tax_Id=9606 Gene_Symbol=PSMD6 26S proteasome non-ATPase regulatory subunit 6

Match to Query 12962: 1345.643328 from(673.828940,2+)
Title: 091224LimSK_Exosome3_04.1124.1124.2.dta
Data file C:\Temp\Mascoti\LSK_2D\091224LimSK_Exosome3_01_2D.mgf

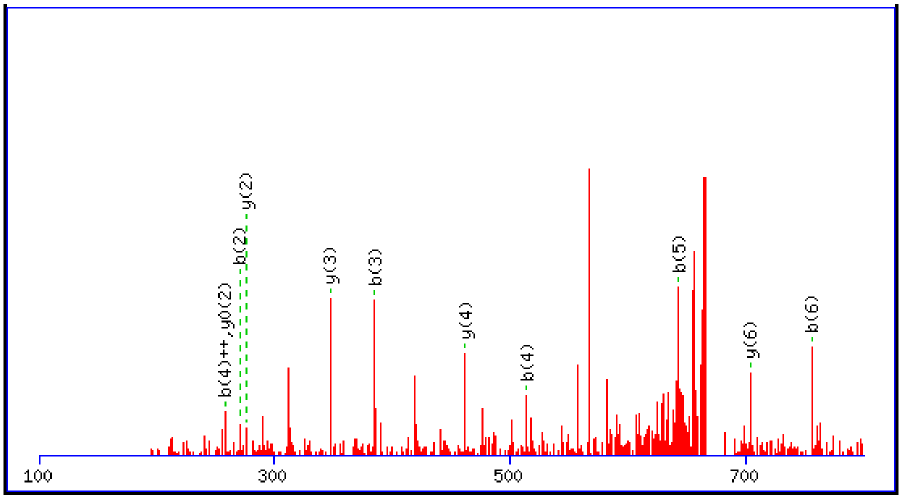

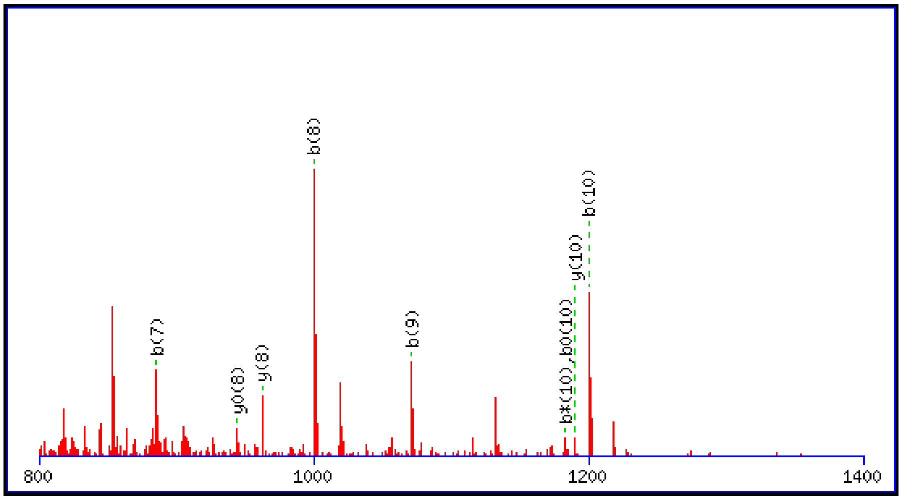

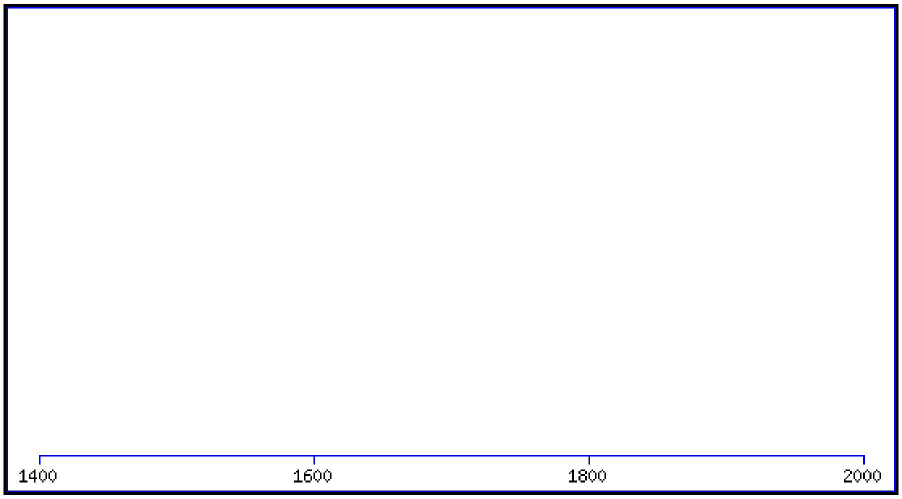

Monoisotopic mass of neutral peptide Mr(calc): 1345.6361
Fixed modifications: Carbamidomethyl (C)
Ions Score: 41 **Expect:** 0.017
Matches (Bold Red): 20/114 fragment ions using 59 most intense peaks

#	b	b^{++}	b*	b^{*++}	b^0	b^{0++}	Seq.	y	y^{++}	y*	y^{*++}	y^0	y^{0++}	#
1	157.1084	79.0578	140.0818	70.5446			R							11
2	**270.1925**	135.5999	253.1659	127.0866			L	**1190.5423**	595.7748	1173.5158	587.2615	1172.5317	586.7695	10
3	**385.2194**	193.1133	368.1928	184.6001	367.2088	184.1081	D	1077.4582	539.2328	1060.4317	530.7195	1059.4477	530.2275	9
4	**514.2620**	**257.6346**	497.2354	249.1214	496.2514	248.6293	E	**962.4313**	481.7193	945.4047	473.2060	**944.4207**	472.7140	8
5	**643.3046**	322.1559	626.2780	313.6427	625.2940	313.1506	E	833.3887	417.1980	816.3622	408.6847	815.3781	408.1927	7
6	**756.3886**	378.6980	739.3621	370.1847	738.3781	369.6927	L	**704.3461**	352.6767	687.3196	344.1634	686.3355	343.6714	6

**7**	**885.4312**	443.2193	868.4047	434.7060	867.4207	434.2140	**E**	591.2620	296.1347	574.2355	287.6214	573.2515	287.1294	**5**
**8**	**1000.4582**	500.7327	983.4316	492.2195	982.4476	491.7274	**D**	**462.2195**	231.6134	445.1929	223.1001	444.2089	222.6081	**4**
**9**	**1071.4953**	536.2513	1054.4687	527.7380	1053.4847	527.2460	**A**	**347.1925**	174.0999	330.1660	165.5866	329.1819	165.0946	**3**
**10**	**1200.5379**	600.7726	**1183.5113**	592.2593	**1182.5273**	591.7673	**E**	**276.1554**	138.5813	259.1288	130.0681	**258.1448**	129.5761	**2**
**11**							**K**	147.1128	74.0600	130.0863	65.5468			**1**

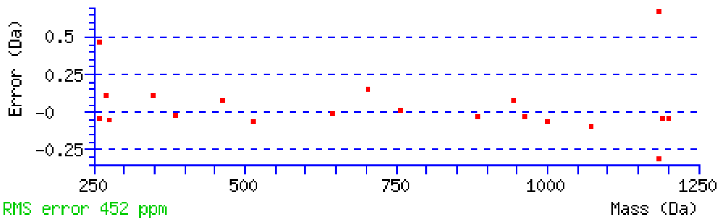

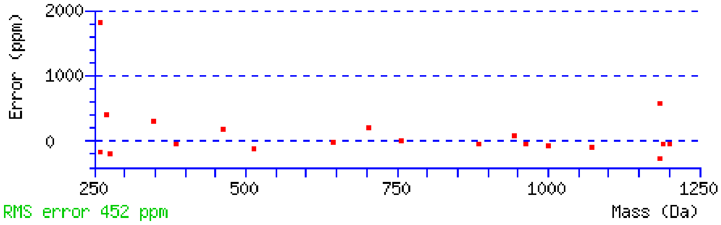

All matches to this query

Score	Mr(calc):	Delta	Sequence
40.7	1345.6361	0.0072	RLDEELEDAEK
8.2	1345.6520	-0.0087	IRSVIKQGGTK
8.1	1345.6337	0.0097	XIGLMYTFGDGR
5.4	1343.6470	1.9964	ATGDWAKVPEDR
4.2	1345.6449	-0.0016	AAGFPTASVCHTK
3.6	1345.6407	0.0026	LRLSIQDITK
2.5	1343.6486	1.9947	DLLRLSYGEAK
2.2	1343.6486	1.9947	SSVEEALFVKR
2.2	1343.6486	1.9947	SSVEEALFVKR
1.7	1345.6530	-0.0097	LKLSYEGEVTK

Spectrum No: 140; Query: 16923; Rank: 1

Peptide View

MS/MS Fragmentation of **DIKDTTVGTLSQR**
Found in **IPI00019927**, Tax_Id=9606 Gene_Symbol=PSMD7 26S proteasome non-ATPase regulatory subunit 7

Match to Query 16923: 1432.761048 from(717.387800,2+)
Title: 091224LimSK_Exosome3_05.1608.1608.2.dta
Data file C:\Temp\Mascoti\LSK_2D\091224LimSK_Exosome3_01_2D.mgf

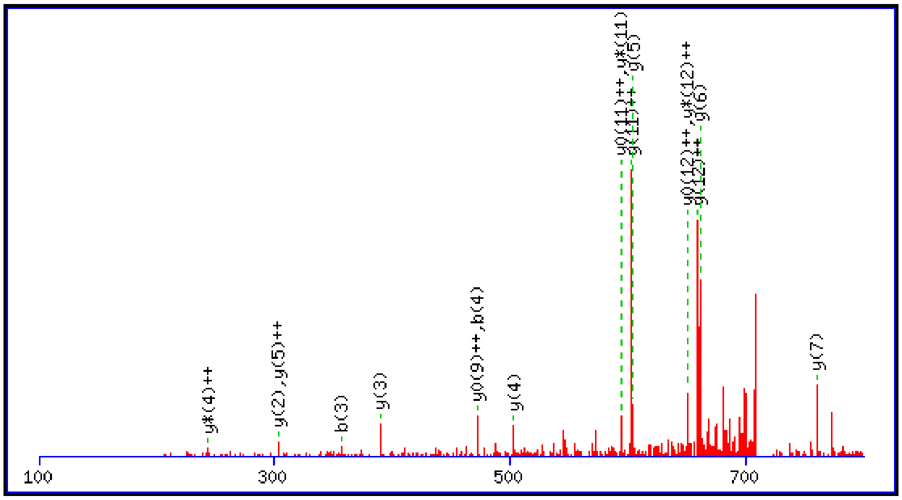

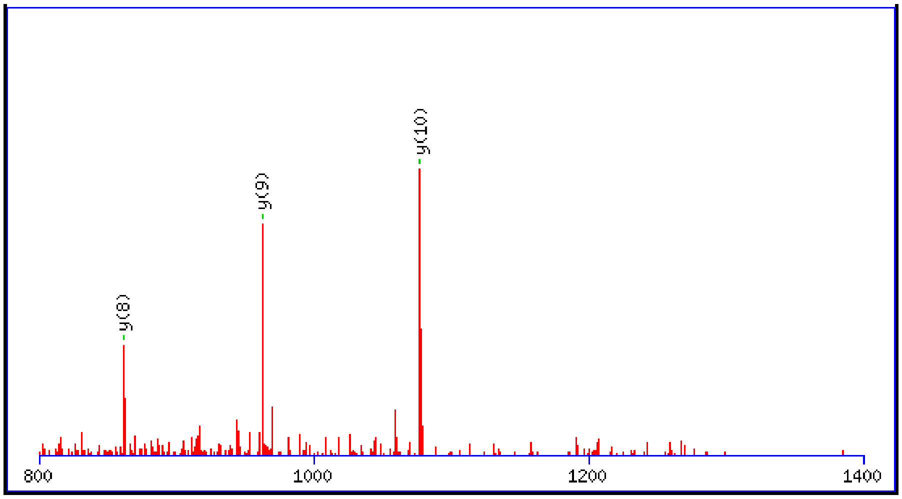

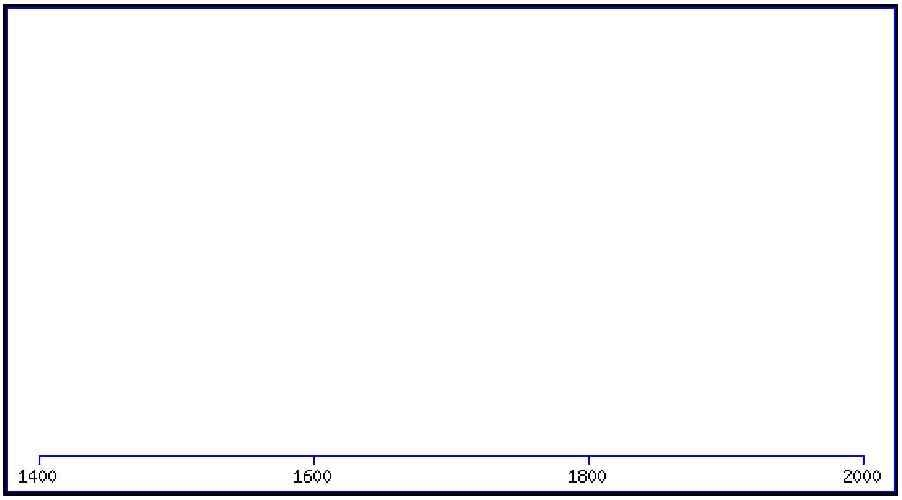

Monoisotopic mass of neutral peptide Mr(calc): 1432.7522
Fixed modifications: Carbamidomethyl (C)
Ions Score: 70 **Expect:** 1.5e-005
Matches (Bold Red): 20/136 fragment ions using 28 most intense peaks

#	b	b^{++}	b*	b^{*++}	b^{0}	b^{0++}	Seq.	y	y^{++}	y*	y^{*++}	y^{0}	y^{0++}	#
1	116.0342	58.5207			98.0237	49.5155	D							13
2	229.1183	115.0628			211.1077	106.0575	I	1318.7325	**659.8699**	1301.7060	**651.3566**	1300.7219	**650.8646**	12
3	**357.2132**	179.1103	340.1867	170.5970	339.2027	170.1050	K	1205.6484	**603.3279**	1188.6219	**594.8146**	1187.6379	**594.3226**	11
4	**472.2402**	236.6237	455.2136	228.1105	454.2296	227.6185	D	**1077.5535**	539.2804	1060.5269	530.7671	1059.5429	530.2751	10
5	573.2879	287.1476	556.2613	278.6343	555.2773	278.1423	T	**962.5265**	481.7669	945.5000	473.2536	944.5160	**472.7616**	9
6	674.3355	337.6714	657.3090	329.1581	656.3250	328.6661	T	**861.4789**	431.2431	844.4523	422.7298	843.4683	422.2378	8
7	773.4040	387.2056	756.3774	378.6923	755.3934	378.2003	V	**760.4312**	380.7192	743.4046	372.2060	742.4206	371.7139	7
8	830.4254	415.7164	813.3989	407.2031	812.4149	406.7111	G	**661.3628**	331.1850	644.3362	322.6717	643.3522	322.1797	6
9	931.4731	466.2402	914.4466	457.7269	913.4625	457.2349	T	**604.3413**	**302.6743**	587.3148	294.1610	586.3307	293.6690	5
10	1044.5572	522.7822	1027.5306	514.2689	1026.5466	513.7769	L	**503.2936**	252.1504	486.2671	**243.6372**	485.2831	243.1452	4
11	1131.5892	566.2982	1114.5626	557.7850	1113.5786	557.2930	S	**390.2096**	195.6084	373.1830	187.0951	372.1990	186.6031	3
12	1259.6478	630.3275	1242.6212	621.8143	1241.6372	621.3222	Q	**303.1775**	152.0924	286.1510	143.5791			2
13							R	175.1190	88.0631	158.0924	79.5498			1

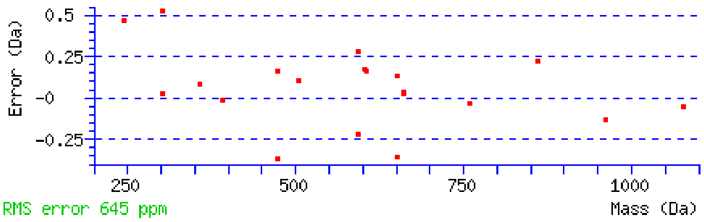

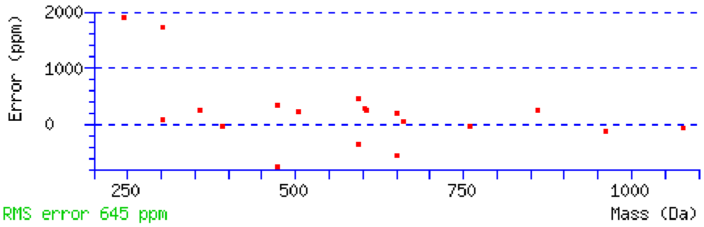

All matches to this query

Score	Mr(calc):	Delta	Sequence
69.5	1432.7522	0.0089	DIKDTTVGTLSQR
12.1	1431.7504	1.0107	MNQIVGLGISTQR
9.0	1432.7596	0.0015	NLTLMATTSQLPK
6.7	1430.7477	2.0133	LLAGEDGTSERRK
6.1	1432.7674	-0.0063	EYRLTPALDSLR
6.1	1430.7551	2.0059	LLGMLPSENSSKR
4.8	1431.7527	1.0084	LKLVNASVAFYK
4.5	1432.7634	-0.0023	LELRQLSTGSSSR
4.3	1432.7748	-0.0137	LMKDISQNFPIK
3.6	1430.7405	2.0205	DKIIFPNDENVK

Mascot: http://www.matrixscience.com/
